# Targeted RNA sequencing enhances gene expression profiling of ultra-low input samples

**DOI:** 10.1080/15476286.2020.1777768

**Published:** 2020-06-28

**Authors:** Fabiola Curion, Adam E Handel, Moustafa Attar, Giuseppe Gallone, Rory Bowden, M. Zameel Cader, Michael B Clark

**Affiliations:** a Wellcome Centre for Human Genetics, University of Oxford, Oxford, UK; b Nuffield Department of Clinical Neurosciences, University of Oxford, Oxford, UK; c Translational Molecular Neuroscience Group, Weatherall Institute of Molecular Medicine, University of Oxford, Oxford, UK; d Kennedy Institute of Rheumatology, University of Oxford, Oxford, UK; e Department of Physiology, Anatomy, and Genetics, University of Oxford, Oxford, UK; f Department of Psychiatry, University of Oxford, Oxford, UK; g Centre for Stem Cell Systems, Department of Anatomy and Neuroscience, The University of Melbourne, Parkville, Australia

**Keywords:** CaptureSeq, gene expression, method, RNA-seq, stem-cell-derived neurons, low-input sequencing, targeted RNA sequencing

## Abstract

RNA-seq is the standard method for profiling gene expression in many biological systems. Due to the wide dynamic range and complex nature of the transcriptome, RNA-seq provides an incomplete characterization, especially of lowly expressed genes and transcripts. Targeted RNA sequencing (RNA CaptureSeq) focuses sequencing on genes of interest, providing exquisite sensitivity for transcript detection and quantification. However, uses of CaptureSeq have focused on bulk samples and its performance on very small populations of cells is unknown. Here we show CaptureSeq greatly enhances transcriptomic profiling of target genes in ultra-low-input samples and provides equivalent performance to that on bulk samples. We validate the performance of CaptureSeq using multiple probe sets on samples of iPSC-derived cortical neurons. We demonstrate up to 275-fold enrichment for target genes, the detection of 10% additional genes and a greater than 5-fold increase in identified gene isoforms. Analysis of spike-in controls demonstrated CaptureSeq improved both detection sensitivity and expression quantification. Comparison to the CORTECON database of cerebral cortex development revealed CaptureSeq enhanced the identification of sample differentiation stage. CaptureSeq provides sensitive, reliable and quantitative expression measurements on hundreds-to-thousands of target genes from ultra-low-input samples and has the potential to greatly enhance transcriptomic profiling when samples are limiting.

## Introduction

Conventional bulk RNA-seq has become a staple method of interrogating biological systems but full representation of the transcriptome in many samples is difficult even with extremely deep sequencing [1]. The use of targeted RNA-seq (CaptureSeq) can help resolve this issue by focusing sequencing on genes of interest and allowing the interrogation of low-expressed genes and transcripts that would be missed by conventional methods [2–5]. Targeted RNA-seq is a widely applicable method and has been successfully utilized to enrich genes and transcripts from mammals [2,3], plants [6], fungi [7], bacteria [8] and viruses [9]. CaptureSeq performs well on different sample types, including formalin-fixed paraffin-embedded (FFPE) samples with highly degraded RNA and has been widely applied to identify molecular alterations in cancer in both research and clinical settings [10–15]. An additional benefit of targeted RNA-seq is to allow more samples to be sequenced at greater effective depth without increasing sequencing costs [16].

However, in many situations, such as in some clinical samples, the scarcity of biological material precludes the generation of sequencing libraries for bulk CaptureSeq. In addition, many experimental questions are best answered by investigating gene expression in small populations of relatively homogenous cells as opposed to heterogenous bulk samples. Developing the CaptureSeq methodology to function on such ultra-low-input samples would allow its use on many more sample types.

Other methods for highly sensitive quantitation of target genes from small initial samples include multiplex qPCR and the nCounter^Ⓡ^ system [17] from NanoString Technologies, however, these are limited to quantifying short-stretches of known sequence. Alternative methodologies for targeted cDNA sequencing, including multiplexed primer extension [18] and molecular inversion probes (cDNA-smMIP) [19] have recently been developed but have not been validated to work on ultra-low input samples. cDNA-smMIP is a method to sequence multiplex PCR products and so has the same core limitation as multiplex PCR, while multiplexed primer extension sequences only portions of transcripts. The advantage of CaptureSeq over these technologies for bulk expression profiling is that CaptureSeq is a ‘discovery and quantification’ method that can provide much more information about gene expression than gene (or transcript) level expression counts. CaptureSeq sequences entire transcripts and is well suited to identifying the expression of known and novel transcripts, as well as refining transcript annotations by identifying novel exons, novel splice sites and even new genes from intergenic spaces (by profiling non-exonic genome regions) [2–5,20].

One application in which low-input targeted RNA-seq would be particularly useful is in induced pluripotent stem cell (iPSC)-based models of corticogenesis. Many protocols to generate cortical neurons are time-consuming and labour-intensive, often compounded by the requirement to study experimental conditions in biological and technical replicates [21]. Drug screening is one common application of iPSC-based systems; generating sufficient cell numbers to permit conventional RNA-seq analysis to screen even a modest library of compounds is challenging and moreover, the amount of sequencing required to process these large numbers of RNA-seq libraries is prohibitive [22]. A protocol that allowed targeted RNA-seq of hundreds or thousands of genes from low-input samples would be highly beneficial to these applications.

**Figure 1.**
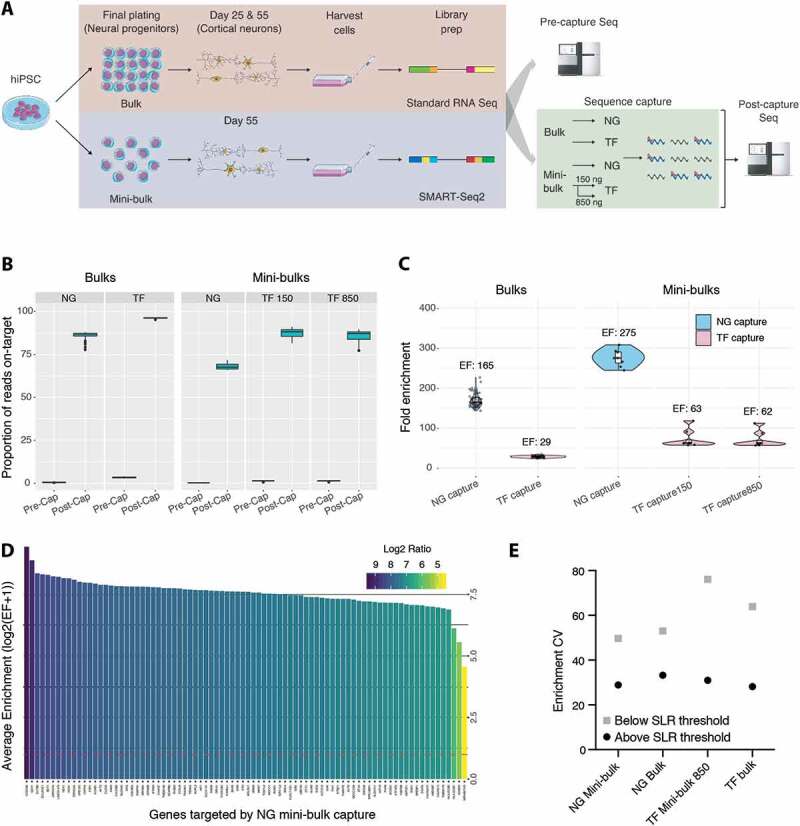
Mini-bulk CaptureSeq targets sequencing to genes of interest. (A) Schematic overview of project. Human-induced pluripotent stem cells (hiPSCs) were differentiated into cortical neurons as described in Volpato et al. [23]. Neural progenitor cells were separated into bulk and mini-bulk plates at the final plating stage. Sequencing libraries were utilized for standard (pre-capture) sequencing and sequence capture. Sequence capture with the NG and TF probe sets was performed on both bulk and mini-bulk samples, with an additional low cDNA (150 ng) hybridization capture performed on a mini-bulk pool with the TF probe set. Post-capture samples were sequenced to evaluate capture performance between bulk and mini-bulk samples. (B) Proportion of on-target reads pre- and post-capture. On-target reads are those overlapped by capture probes. Proportions shown as box plots, error bars span minimum to maximum values. (C) Enrichment of each library by capture. Enrichment factor (EF) is ratio of reads overlapping probe positions pre- and post-capture. Median EF reported. Box plot overlaid with violin plot displaying the EF ratio for each sample. Error bars span minimum to maximum values. (D) Genewise enrichment of coding genes targeted by NG capture in mini-bulk sample pool. Eighty-two genes detected pre-capture, all were enriched post-capture. Dashed red line represents no enrichment. (E) Between gene enrichment variability in bulk and mini-bulk captures. Enrichment CV shown for genes above and below the SLR expression cut-off. CV, coefficient of variation; SLR, segmental linear regression

We aimed to develop and validate CaptureSeq for ultra-low-input RNA samples generated from several thousand cells, which we term ‘mini-bulks’. We utilized forebrain cortical neurons differentiated *in vitro* from iPSCs [23]. We compared CaptureSeq performance on mini-bulk samples to standard Illumina sequencing on the same low-input samples and to gene expression results generated from CaptureSeq and standard sequencing on matched conventional bulk samples (Fig. 1A). Our results demonstrate that CaptureSeq provides excellent performance on mini-bulk samples, greatly enriching for targeted genes and improving the sensitivity of gene expression profiling, while maintaining expression level quantification. Comparison to CaptureSeq on bulk samples suggests similar capture performance for both sample types. Capture on ultra-low-input RNA may have greater utility in helping overcome some of the issues with poor quality and/or lower complexity common to libraries from small numbers of cells, making ultra-low-input RNA CaptureSeq a valuable addition to methods for gene expression profiling.

## Materials and methods

### Summary

We evaluated CaptureSeq for low-input samples using experimental materials and data initially generated for a recently published study [23], where full details may be found. The following is a summary of experimental methods relevant to this study.

### Generation of cortical neurons from iPSC

Dermal fibroblasts from a healthy control individual (line SB-AD3-1) and an individual with Alzheimer’s disease caused by a *PSEN1* intron four mutation (line SB808-03-04) were used to generate iPSCs as detailed in Volpato et al. [23]. As described previously, confluent monolayer iPSCs were induced by dual-SMAD inhibition for 12 days after which progenitors were expanded and differentiated for 3 weeks [21]. Thereafter, independent inductions were seeded into 12-well culture dishes at a final plating density of 8.5 × 10^4^ cells/cm^2^. Cells were then cultured for 25 or 55 days (D25 and D55) post-final plating before being harvested for bulk, and 55 days (D55) for low-input, RNA-seq. Three replicate differentiations of each iPSC line (Control and PS1) were carried out in five independent laboratories, with the exception that one laboratory only produced two successful differentiations of the *PS1* line [23]. Bulk libraries generated from each differentiation were included in this study. Low-input libraries generated in parallel in two independent laboratories (duplicate samples from two controls and one PS1 differentiation) were included in this study. Final plating was undertaken in separate culture plates for bulk and low-input samples from the same pooled progenitors.

### RNA processing

For conventional bulk RNA samples, three wells of a 12-well plate were pooled and RNA was isolated using RNeasy Mini Kits (QIAGEN). For ultra-low-input bulks (hereafter referred to as mini-bulks), total RNA was extracted from 4000 cells using RNeasy Micro Kits (QIAGEN) and 1 ng of the extracted RNA solution utilized for the library prep. Mini-bulk RNA-seq was undertaken using the SMART-seq2 protocol [24].

### Design of oligonucleotide capture pools

Two sets of oligonucleotide capture probes were designed for synthesis as Roche NimbleGen SeqCap Libraries. The first targeted human neurological disease risk loci and candidate genes (NeuroGWAS) **Tables S1 and 2**, while the second targeted human transcription factor genes **Tables S3 and 4** (hereafter referred to as the NG and TF captures, respectively).

### NeuroGWAS (NG) Capture

SeqCap EZ Choice XL Library oligonucleotide probes were designed to target all regions of the genome that were associated with neurological diseases and traits, but which did not contain any protein-coding genes. This was determined by identifying linkage-disequilibrium (LD) blocks containing genome-wide significant GWAS SNPs, which had no overlapping protein-coding genes. Also targeted were a set of candidate protein-coding genes suspected by biology, GWAS or both of being involved in schizophrenia or Parkinson’s disease.

To identify all intergenic genomic regions identified as playing a role in neurological diseases and traits by GWAS, disease-linked SNPs from the NHGRI GWAS catalogue were downloaded from the UCSC genome browser hg19 build (accessed 2015.02.11, version last updated 2015.02.10 [25]); and filtered to retain only those with p values <5x10^–8^ [26]. This consisted of 8145 entries (6757 unique SNP – phenotype associations). These were supplemented with 207 additional unique SNP – phenotype associations from more recent studies not present in the catalogue at the time, with the majority from [27,28]. LD blocks were calculated by plink [29] based on *D*′ with 1000 Genomes SNPs [30]. Blocks were determined for European panel with LD block settings as follows: lowci 0.7005; hici 0.98; recombci 0.9; informfrac 0.85. GWAS SNPs overlapped 3390 LD blocks. Three hundred and twenty-two SNPs had no associated LD block, the majority of these were rescued by allowing different parameters for LD block estimation, in order of preference 1. Lowci 0.6005; hici 0.83; recombci 0.7. 2. Lowci 0.7005; hici 0.98; recombci 0.9; informfrac 0.95. 3. Overlap with LD blocks from [27]. LD blocks were then filtered firstly for those with GWAS SNPs associated with neurological diseases and traits and secondly to remove those with any protein-coding transcripts using GencodeV19comprehensive [31] and Refseq [32] (downloaded from UCSC genome browser 2015.03.23). This left 142 unique LD blocks covering 6.2Mb, which were tiled with probes.

Some of the 142 LD blocks partially overlapped long noncoding RNA (lncRNA) transcripts or had lncRNA transcripts nearby. To allow these transcripts to be characterized, all exons from lncRNA genes that overlapped an LD block or had an exon within 50kb of an LD block were targeted for capture. LncRNA were those characterized by previous capture experiments [2,4], as well as spliced transcripts targeted for capture which had no overlap with coding transcripts [2]. This targeted the exons of 1240 lncRNA transcripts. To ensure no synthesized probes could inadvertently pulldown coding transcripts, any target regions within 50nt of a coding transcript (GencodeV19 comprehensive) were trimmed.

Probes to candidate genes for schizophrenia and Parkinson’s disease were also included for capture, including 33 coding genes and 3 lncRNA loci for schizophrenia and 58 coding genes as well as nearby and antisense lncRNAs for Parkinson’s. A 500 nt intronic region for each schizophrenia gene and for ten Parkinson’s genes were targeted as an expression and transcript assembly control; these were chosen to be repeat-free with high uniqueness and no evidence for transcription (GencodeV19).

Additional control sequences were targeted, including a set of 28 coding genes, chosen to help identify cell types being captured including *GAD2* and *CALB1* for neurons relevant to Parkinson’s; a set of genes for following iPSC differentiation into cortical neurons; controls for various types of neurons; and controls for non-neuronal cell types. Target regions were trimmed to remove overlap with other genes and for the most highly expressed control genes 3ʹUTR regions were also trimmed. Other control regions included a gene desert region; a region of the *E.coli* K12 genome as a contamination control and a partial set of the ERCC control spike-in set, comprising 56 of the 92 ERCC controls, with the most highly abundant controls omitted and others not targeted to create a within-experiment control for capture enrichment (see [16]).

All targeted human sequences were merged and filtered to remove any regions within 50nt of highly expressed rRNA repeats (rRNA, tRNA, 7SK and srpRNA/7SL). The final design targeted 8.0Mb of human sequence and 378 annotated genes (GencodeV19 comprehensive). The design of probes from target regions and probe synthesis was performed by Roche NimbleGen. Probes were allowed up to five matches to the human genome unless no probe could be designed for a specific target region, in which case up to ten matches were allowed. Synthesized probes covered 83.8% of target regions directly, with 92.3% of target regions estimated to be within range of the synthesized probes. Capture design coordinates (hg19) are provided in **Table S1** and a list of the ERCC controls targeted is provided in **Table S2**. This study focused on 101 mainly coding genes targeted in full by the synthesized probes.

### Transcription Factor (TF) capture

SeqCap RNA Choice oligonucleotide probes were designed to target all high-confidence human DNA-binding transcription factor (DbTF) genes from http://www.tfcheckpoint.org [33](downloaded 2017.02.14). All genes placed on the hg38 human reference assembly with HGNC annotations were selected (1014 DbTFs). To minimize the impact of highly expressed genes on overall capture enrichment, the expression levels of all 1014 DbTFs were examined in scRNA-seq neuronal cells and ENCODE cell lines [34,35]. Forty-two DbTF that were predicted to account for 0.5% or more of captured reads in neuronal cells or over 5% of targeted reads in an ENCODE sample were identified and only probed in part (see below). For the remaining 972 DbTFs, oligonucleotide probes were designed against all GencodeV26 transcripts except those from retained introns & TEC problem transcripts.

For the 42 DbTFs probed in part, only GencodeV26 basic protein-coding transcripts were selected. For mono-exonic transcripts, probes were designed against the last 200nt of the CDS, while for multi-exonic transcripts the last 100 nt of the penultimate exon (or the entire exon if it was shorter than 100 nt) and the first 150 nt of the final exon (or the entire final exon if shorter than 150nt) were targeted.

The TF capture also targeted control genes which are brain cell-type markers; housekeeping genes; cell and developmental stage identity genes for brain; iPSC differentiation markers; and marker genes of various human cell and tissue types. Two hundred and twenty-one control genes were identified and probes designed as per the 42 highly expressed DbTFs.

To prevent capture of non-targeted genes or highly expressed RNA repeats, all probes were filtered to trim any that were within 150nt of non-targeted loci from the GencodeV26 annotation file. Non-targeted loci included coding genes; most RFAM type ncRNAs; transcribed and untranscribed pseudogenes; IG; and TcR genes. Also filtered were any probes within 120nt of Repeat Masker RNA (repClass) repeats.

A 200nt repeat-free intronic control probe was designed against all possible target genes containing an intron. All intronic regions to targeted genes were identified and trimmed to remove any regions within 500nt of GencodeV26 annotations, within 100 nt of any Repeat Masker repeats or within 50nt of any human ESTs (downloaded from UCSC genome browser 2017.06.07). All remaining intron pieces were filtered to retain those over 200nt and the longest intron piece remaining per gene was selected. The targeted region was the middle 200nt of the selected intronic pieces. Intronic probes were designed against 890 (of 1141) intron-containing target genes. An additional human gene desert region (no GencodeV26 annotations, pseudogenes, or RNA repeats) was also included. The design targeted 5.10 Mb of human hg38 sequence.

Additional non-human controls included a region of the *E.coli* K12 genome as a contamination control; a cut-down set of ERCC spike-in (56 of 92 transcripts); Spike-In RNA Variants (SIRVs) (Lexogen) and Sequins [36]. SIRV and Sequin transcripts were made compatible with the Roche probe design procedure by removing all target regions under 30nt and padding all target regions between 30 and 49nt to 50/51nt.

The design of probes from target regions and probe synthesis was performed by Roche NimbleGen. Probes were allowed up to five matches to the human genome (hg38) unless no probe could be designed for a specific target region, in which case up to ten matches were allowed. Synthesized probes covered 92.7% of target regions directly, with 97.9% of target regions estimated to be within range of the synthesized probes. Probes to *E. coli* and RNA spike-ins were allowed zero close matches to hg38. Capture design coordinates (hg38) are provided in **Table S3** and a list of spike-in controls targeted is provided in **Table S4**. This study focused on 967 coding genes targeted in full by the synthesized probes.

### Sequence capture of bulk and low-input mini-bulk samples

Bulk and Mini-bulk (low-input) sample capture hybridizations were performed in parallel. Premade sequencing libraries were quantitated by Qubit (ThermoFisher); any samples not in nuclease-free water (NFW) (i.e. in EB buffer) were cleaned up using 1.8x AMPure XP beads (Beckman Coulter), with an 80% ethanol wash, and eluted in 100 ul of NFW.

Capture was performed similarly to previously described [16], with slight modifications: Briefly, low-input mini-bulk and bulk-sample library pools were captured separately with both the NeuroGWAS (NG) and Transcription Factor (TF) probe sets. Bulk libraries were combined into one pooled sample and both captures were performed on cDNA from this pool. NG and TF captures were performed on pools of separate mini-bulk libraries. Standard capture hybridizations utilized 800–850 ng of pooled library, while a low-cDNA hybridization capture was performed with 150 ng of library using the TF probes. Capture included Cot1 and blocking oligos (xGen® Universal Blockers – TS Mix or NXT Mix, IDT) appropriate to the library type at manufacturer’s recommended amount. Capture hybridization was performed for 3 days. Capture hybridization, bead binding and washing were performed as previously described. Post-capture LMPCR was performed as per SeqCap RNA Enrichment System User’s Guide V1.0 (Roche) with KAPA Taq and Roche post-cap LMPCR primers, except that PCR input was 17ul of resuspended capture beads. Standard hybridization: 12 PCR cycles, low-cDNA hybridization: 14 cycles (though subsequent QC confirmed the standard 12 cycles would have been sufficient). PCR products were cleaned up using 1.8x AMPure XP beads and 80% ethanol washes and eluted in 50ul of NFW.

Captured libraries were QC’d by Qubit and Tapestation (Agilent) to confirm the concentration and overall library yield and size distribution, respectively. Successful capture enrichment was confirmed by qPCR (QuantStudio 6, ThermoFisher). Libraries were sequenced at the Wellcome Centre for Human Genetics (WHG) (Oxford, UK).

### Library sequencing pre-capture

The bulk pre-capture samples were sequenced as a single pool of 58 samples on 8 lanes of HS4000, resulting in an average of 23 million raw reads per sample. The mini-bulk samples were sequenced on a HS4000 as part of 2 bigger experiments each containing 376 single-cell and 8 mini-bulk libraries [23]. The 16 pre-capture libraries had an average of 0.9 million raw reads (**Figure S1A, B, Table S5**).

### Library sequencing post-capture

The bulk post-capture samples were sequenced as 2 pools of 58 samples on 2 independent lanes of HS4000, resulting in an average of 6 million reads per sample. The 24 mini-bulk post-capture libraries were sequenced along with the single-cell libraries on 3 independent lanes of HS4000, resulting in an average of 1.2 million raw reads per sample (**Figure S1A, B, Table S5**).

### Bioinformatic methods

We used custom R and Shell scripts to perform multiple analyses including evaluation of the enrichment at library level and the gene-level, isoform quantification, data analysis, integration and visualization, plotting. Statistical analyses were performed in R and PRISM (GraphPad software).

### Read mapping and gene and transcript identification and quantification

All the samples were processed together. FASTQ files were trimmed using skewer [37] supplying adapter sequences. The data were mapped using Hisat2 [38] supplying a custom hg38 GTF annotation, with default parameters and specifying the option – –dta. We evaluated the mapping quality metrics, performed quantification and removal of duplicated reads, and gene body coverage analysis with the Picard toolkit (Broad Institute, accessed 15 March 2019, http://broadinstitute.github.io/picard/), Samtools (1.4.1) [39] and Bedtools (2.27.0) [40]. Although we have previously recommended against removal of apparently duplicated sequences following capture [2], the high level of duplication in lower-complexity mini-bulk samples made it appropriate in this case (**Figure S2**). These pass-qc, de-duplicated, uniquely mapped reads (hereafter referred to as reads) were used for subsequent analyses.

We summarized gene-level read counts using featureCounts [41], removing multimapping reads and duplicated reads. We performed transcriptomic assembly using StringTie [42] and evaluated the novel assembly and splicing isoform detection using GffCompare (The Centre for Computational Biology at Johns Hopkins University, accessed 15 March 2019, https://ccb.jhu.edu/software/stringtie/gffcompare.shtml) and the same GTF used for mapping. Labels given to transcript class codes were as follows: known (=), partial (c), possible pre-mRNA (e), intronic (i), novel isoform (j), other (o,p,x,k,y). Except for Fig. 3A where: known (=,c).

Two TF mini-bulk samples (255,208 and 256,208) were found to be of poor quality pre-capture, leading to low complexity pre- and post-capture and few post-capture reads (**Figure S1A, S2, S7B**). While they showed equivalent capture enrichment performance, the poor quality of these libraries meant they were removed for ERCC quantifications.

ERCCs were quantified as CPKMs. Expression was compared pre- and post-capture on the subset of ERCCs spike-ins targeted by the capture probes. Relationship between detected expression and known original ERCC concentration was examined using Spearman correlations. To compare linear slopes between pre- and post-capture samples, data were log10 transformed and the slope calculated for all datapoints using non-linear regression with a straight line fit.

### Sample and gene-level enrichment analyses

The proportion of reads mapping to the captured probes was estimated by counting the number of pass-qc, deduplicated, uniquely mapped reads assigned to the probed coordinates, over the total amount of pass-qc deduplicated, uniquely mapped reads.

Sample-wise capture enrichment was calculated as the proportion of reads mapping to probes in the pre-capture library divided by the proportion of reads mapping to probes in the post-capture library. The median enrichment for each capture was then calculated.

Gene-wise enrichment was calculated using CPM normalized data. For each gene, for each capture, we calculated the ratio between the average CPMs in the pre-capture libraries and the average CPMs in the post-capture libraries. We plotted the enrichment factor (EF) in log2 scale by adding a pseudocount of 1, so that not-enriched genes have 0< EF<1. Enrichment success rates were the percentage of genes detected pre-capture that were enriched post-capture.

The relationship between gene enrichment and pre-capture gene expression was examined using non-linear regression. Akaike’s Information Criterion (AICc) [43] identified segmental linear regression (SLR) as the best fit in all cases. AICc was further used to identify the threshold/best expression breakpoint position.

In most cases the original pre-capture libraries had a higher count of QC pass mapped reads than the equivalent libraries post-capture (**Table S6)**. For all the calculations aimed at evaluating the performance of the post-capture data (gene-level enrichment scores, on target read counts, isoform quantification) we ensured that for each pre-capture library the number of usable reads (that is pass-qc, de-duplicated, uniquely mapped reads) was higher or equal to the number of usable reads falling in the probed genes coordinates for the equivalent library post-capture (**Table S6, Figure S2**). While this strategy could potentially have penalized the performance of the captured libraries, we reasoned that any downsampling of the pre-capture libraries would have introduced a bias in the selection of the reads.

### Differential expression (DE) analysis

We analysed the changes in gene expression of a subset of the pre- and post-capture libraries (12 libraries), using the same rationale as the original study [23], which aimed to describe key genes involved in the neuronal differentiation from an iPS cell line.

After evaluating the capture efficiency, 956 transcription factors (TFs) were classified as enriched in the conventional bulk TFcapture experiment. To remove genes with counts too low to allow a reliable determination of differential expression, we applied a mildly stringent CPM threshold to the pre-capture data, retaining genes with a CPM higher than five in at least three libraries. Of the 12,761 genes passing the filter, 596 of 956 were on the TF panel and enriched post-capture. In the post-capture data, we limited our list of genes to be tested to the 956 enriched TFs.

We performed the DE analysis using edgeR [44] between D25 and D55 of differentiation post-final plating in 2 scenarios: 1. Post-capture: DE between D25 and D55 calculating the dispersion only on 956 TF in the panel; 2. Pre-capture: DE between D25 and D55 calculating the dispersion from all the genes whose expression was detectable. Different strategies to obtain DE genes in the pre-capture data included limiting the list of genes to the same 956 TF as in the post-capture test or estimating the dispersion on all genes and only performing multiple test correction (FDR) on the subset of 596 TF detectable in the data, which both led to similar results upon comparison to the post-capture data (data not shown).

### Cortecon dataset comparison

We downloaded the CORTECON dataset [45] and averaged the reported gene expression profiles across replicates for each of the 9 timepoints profiled in the study. We used the averaged profiles to test the correlation with the mini-bulk pre- and post-capture expression data. We relied on CORTECON’s five different categories: Pluripotency (PP); Neural Differentiation (ND); Cortical Specification (CS); Deep Layer neuron generation (DL); and Upper Layer neuron generation (UL) to classify our TFs as relevant to one or more developmental stages.

## Results

### Comparable performance between standard and ultra-low-input (mini-bulk) CaptureSeq

We applied CaptureSeq to ultra-low-input mini-bulk RNA-seq libraries made from 1 ng of total RNA extracted from 4000 cells. Capture probes targeted either a panel of protein-coding genes involved in neural development and brain diseases (NG Capture) or known transcription factors (TF Capture). To allow comparison of mini-bulk CaptureSeq with standard CaptureSeq, we also captured multiplexed bulk RNA libraries derived from the same pool of neural progenitors. (Fig. 1A**, Tables S1–5**). In addition, we also tested a modified low-cDNA hybridization version of CaptureSeq using the TF panel (TF 150). This utilized only 150 ng of library in the capture hybridization to evaluate capture performance when library (as well as sample) input is limiting.

CaptureSeq using either conventional bulk RNA-seq or ultra-low-input SMART-seq2 libraries (mini-bulks) yielded a high proportion (0.68–0.95) of reads assigned to targeted genes (on-target reads) after capture and high levels of enrichment (28–275 fold) (Fig. 1B-C**, S2A, Table S7**). All mini-bulk captures demonstrated excellent performance characteristics. Differences between sample types and capture panels in enrichment results and on-target reads post-capture largely reflected the proportion of the original library targeted for capture, with a clear relationship (Spearman r = 0.975, **Figure S3**) between targeting smaller proportions and higher enrichments. This suggests that rather than inter-experiment variation, the higher nominal enrichments observed with the NG capture panel and the mini-bulk captures were because in each case they targeted less of the transcriptome than the TF panel and bulk captures **(Table S7**). Together these results demonstrate similar capture performance, at a global level, between mini-bulk and conventional bulk libraries.

We next investigated capture enrichment at the gene level. Virtually all genes were successfully enriched (100% and >99.5% for the NG and TF panels, respectively) with almost identical proportions for bulk and mini-bulk captures. The level of enrichment seen across multiple genes was highly consistent within a capture (Fig. 1D**, S4-5**). An effect of expression level was observed, with extremely lowly expressed genes showing higher levels of enrichment and between-gene variability in both mini-bulk and bulk captures, likely due to inconsistent or inaccurate pre-capture detection (Fig. 1E**, S6, Table S8**). Above this threshold (identified by segmental linear regression (SLR) as 1 CPM in mini-bulk samples and 0.1 CPM in bulks), between-gene enrichment variability was equivalent in mini-bulk and bulk captures (Fig. 1E). Taken together the gene-level enrichment results again demonstrate very similar performance for capture on both ultra-low-input and bulk samples.

Read coverage across the whole gene body of targeted genes was maintained after CaptureSeq (**Figure S6F**). Read coverage was similar pre- and post-capture for conventional bulks, in mini-bulks samples capture gave more consistent coverage and better coverage of the 5ʹ and 3ʹ end of targeted genes.

### Expression quantitation and improved gene detection with CaptureSeq on mini-bulk samples

Previous studies have shown CaptureSeq improves sequencing sensitivity, increasing the number of expressed genes of interest detected in a sample, while maintaining expression level quantitation over a large dynamic range [2].

**Figure 2.**
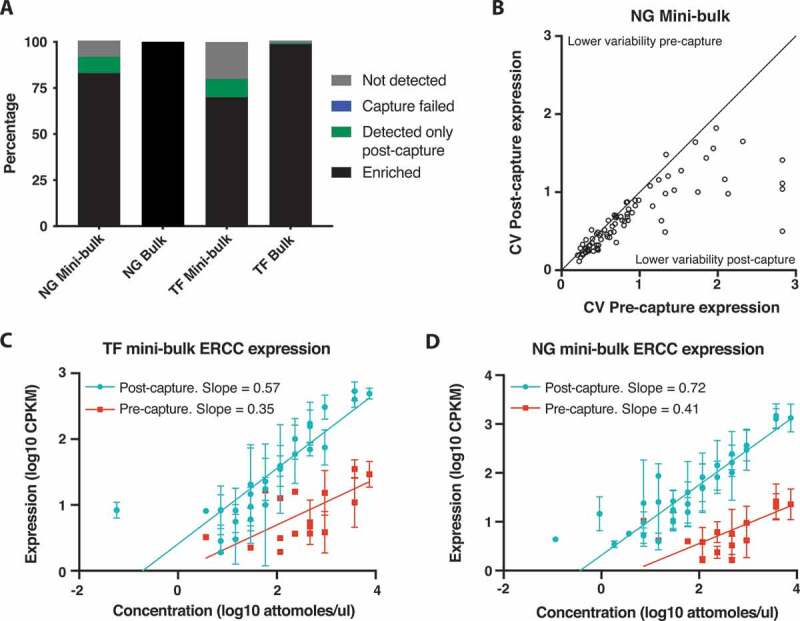
Improved gene detection and expression quantification with mini-bulk CaptureSeq. (A) Percentage of targeted genes enriched after capture. Black: genes detected pre-capture and enriched post-capture. Green: genes only detected post-capture and hence enriched above detection threshold by capture. Blue: genes detected pre-capture but not enriched by post-capture. Grey: gene not detected pre- or post-capture. TF mini-bulk capture is TF850. (B) Expression variability of targeted genes between replicate pre- and post-capture NG mini-bulk samples. CV, coefficient of variation. (C and D) Quantification of ERCCs targeted for capture in pre- and post-capture mini-bulk libraries. TF capture 850 (C), NG capture (D). Pre-capture quantification of ERCC shown in red, post-capture in teal. Compares known ERCC abundance (original concentration in attomoles/ul) to measured expression in CPKM. Mean and standard deviation plotted, n = 8 NG capture, n = 6 TF capture. Trend line, non-linear regression with a straight line fit

Compared with bulk sequencing samples, in which 99% (TF) and 100% (NG) of those genes subsequently targeted for capture were detected with at least one read in one replicate pre-capture, mini-bulks had lower detection rates. CaptureSeq improved detection sensitivity for mini-bulks, recovering reads from ~10% of targeted genes where no reads were detected before enrichment (Fig. 2A), to detect a total of 92% (NG capture) and 80% (TF capture) of targeted genes, respectively. Expression of targeted genes was also measured more reproducibly after CaptureSeq with decreased expression variability between replicates (p < 0.0001, Wilcoxon matched-pairs signed rank two-tailed test, Fig. 2B, **S8D**).

These results suggest that mini-bulk samples have decreased library complexity compared to bulk samples, some of which is technical and can be overcome through the use of CaptureSeq. This is further supported by the lower proportion of the transcriptome coming from targeted genes in the mini-bulk pre-capture samples (Fig. 1C**, Table S7**), in comparison to matched bulk samples, suggesting lower mRNA complexity and less representation of lowly expressed genes. Taken together these results show CaptureSeq on ultra-low-input samples improves the sensitivity and reproducibility of target gene detection, as in bulk samples.

To investigate if ultra-low-input CaptureSeq maintained gene quantification we focused on the detection of ERCC spike-ins, where the abundance of each spike-in pre-capture is known (**Table S9**). CaptureSeq improved the ERCC detection threshold dramatically by 64-fold for both TF and NG captures. This was associated with the detection of a much larger number of the targeted spike-ins, increasing from 16 to 29 for the TF captures and from 15 to 30 for the NG capture. Conversely, ERCCs not targeted for capture were significantly depleted from the CaptureSeq libraries by >20-fold (TF captures) and >7-fold (NG capture), respectively.

The level of ERCC spike-in gene expression post-capture was directly correlated with the known abundance in the sequencing library (Fig. 2C,D). ERCCs detected by CaptureSeq showed higher correlations (Spearman correlation: 0.914 NG capture and 0.894 TF captures, all p < 0.0001, two-tailed) with ERCC abundance than those obtained from pre-capture sequencing (Spearman correlation: 0.777 NG capture and 0.735 TF captures, all p < 0.002, two-tailed). To further investigate gene expression quantitation in mini-bulk samples we utilized non-linear regression to ask if CaptureSeq maintained quantitation compared to pre-capture samples. Regression slopes were higher in CaptureSeq samples (NG capture – pre-capture: 0.411, post-capture: 0.720; TF capture – pre-capture 0.350, post-capture: 0.573) likely reflecting the increased number of ERCCs detected and suggesting greater accuracy in quantifying expression levels with CaptureSeq.

In summary, these results demonstrate that CaptureSeq on mini-bulk samples preserves the quantitative measurement of the targeted transcriptome while expanding the dynamic range to allow the detection of previously undetectable low-level transcripts.

### CaptureSeq provides a more comprehensive profile of the mini-bulk transcriptome

**Figure 3.**
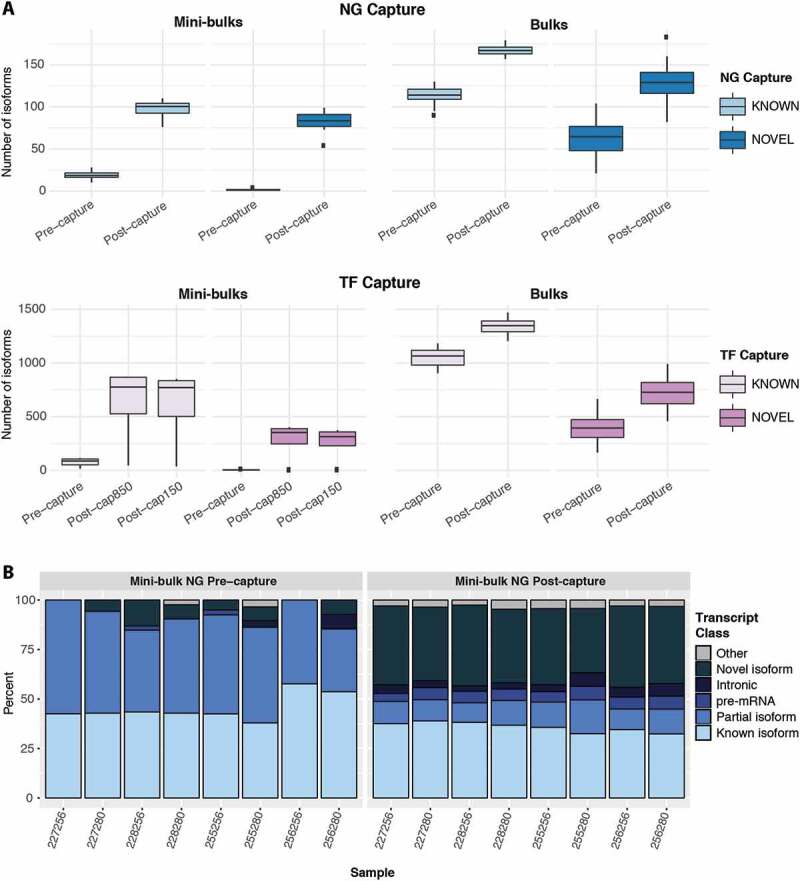
Mini-bulk CaptureSeq allows comprehensive profiling of expressed gene isoforms. (A) Number of known and novel isoforms detected pre- and post-capture. (B) Percentage of detected isoforms in different classifications classes pre- and post-capture. Isoform classes described in methods

The targeted sequencing of genes of interest with CaptureSeq led to the identification of more known and novel splicing isoforms than were identified in pre-capture libraries (Fig. 3A**, S7A**). This result was consistent for bulk and mini-bulk libraries and matches previous results in bulk samples [2]. Focusing on complete and partial known isoforms, which should be less susceptible to false positives than novel isoforms, mini-bulk CaptureSeq produced a much greater fold increase in isoforms detected (5-fold NG, 9-fold TF), over standard RNA-seq than in bulk samples (<2-fold) (**Table S10)**. Mini-bulk capture also decreased the proportion of partial or incomplete isoforms (Fig. 3B**, S7B**) from ~55% to ~14% (NG capture) and ~37% to 22% (TF capture). Together these results demonstrate that, just as for low-expressed genes, capture improves the sensitivity of transcript identification in mini-bulk libraries and may also have a greater effect on detection sensitivity in lower complexity, low-input samples than in bulks.

### Equivalent CaptureSeq performance from low-cDNA hybridizations

Regardless of the amount of original cellular input, standard capture hybridization protocols call for the addition of ~1 µg of prepared cDNA library. Generating this amount of pre-capture library from very small amounts of original material may not be feasible or may require a level of PCR amplification that could reduce library complexity and bias the representation of transcripts. We investigated capture performance using a reduced hybridization input of 150 ng cDNA library with the TF panel. Results showed almost identical performance compared to the use of standard hybridization inputs (Figs. 1B-C, 3A**, S6-8**), demonstrating that cDNA input into the capture can be reduced at least 5-fold without compromising capture enrichment and quantification.

### CaptureSeq enables cell-state determination and identifies transcription factors enriched during neural differentiation

**Figure 4.**
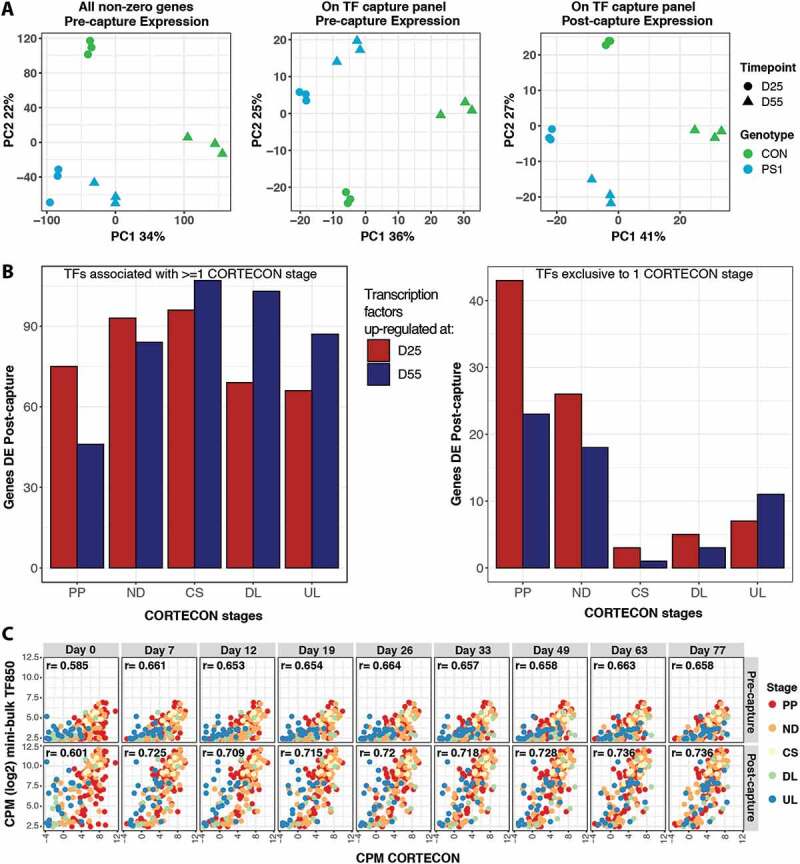
CaptureSeq enhances identification of sample differentiation stage. (A) Principal component analysis of bulk samples from different timepoints (D25, D55) and genotype status (CON and PS1). Panels show PC1 and PC2 of all genes expressed pre-capture (left, replicating [23]); pre-capture expression of genes targeted by TF capture (middle); and post-capture expression of TF capture targeted genes (right). (B) Overlap between genes associated with different developmental stages in CORTECON dataset and DE genes with higher expression at D25 or D55 in bulk TF post-capture samples. From earlier: Pluripotency (PP), Neural Differentiation (ND), Cortical Specification (CS); to later: Deep Layer Generation (DL), Upper Layer Generation (UL) stages. Left: All 597 TF DE genes, including genes which associate with multiple CORTECON stages. Right: TF DE genes (212) exclusive to one CORTECON stage. (C) Pearson correlations (all significant at p ≤ 0.001), between mini-bulk pre- (top) and post- (bottom) TF850 capture gene expression profiles and matched gene expression levels from each CORTECON timepoint. Colours represent CORTECON stages genes are associated with

The pre-capture libraries used in this work were part of a previous study which characterized neuronal differentiation from iPSCs, identifying a clear separation between samples of different timepoints (Day 25 and Day 55) and genotypes (control (CON) and patient (PS1)) by principal component analysis [23]. We replicated this pre-capture result in bulk samples using all genes, as well as just those transcription factors (TFs) on the capture panel (Fig. 4A). Furthermore, the same analysis on the 956 TFs enriched post-capture achieved a comparable degree of separation in the first 2 components and explained a higher proportion of variance (68%) than in the pre-capture data (61%) (Fig. 4A**, S9A**). This demonstrated the information content of TF expression is relevant to assessing neuronal differentiation and is further increased by targeted sequencing of the TF panel.

To further examine the extra resolution on TF expression obtained by sequence capture we performed a differential expression (DE) analysis contrasting the two differentiation timepoints, while accounting for differences in the genotype, in the pre- and post-capture bulk samples. Fifty per cent of genes detected pre-capture and 61% detected post-capture were significantly DE, suggesting a more stable estimation of the expression of these genes and a lower dispersion post-capture (**Figure S9B-C**). The >100 additional TFs identified as DE during neuronal differentiation with CaptureSeq demonstrates its ability to identify additional genes involved in biological processes and to resolve gene expression changes pivotal to cell stage classification during differentiation.

To support a biological role of these genes in neuronal differentiation, we classified them using the CORTECON [45] dataset, which characterized the progressive changes in gene expression during the *in vitro* differentiation of cerebral cortex from human embryonic stem cells. TFs associated with early developmental stages in the CORTECON data (i.e. pluripotency (PP), neural differentiation (ND)) were enriched at the earlier (D25) timepoint post-capture, while those associated with later developmental (i.e. upper layer generation (UL)) were enriched for upregulation at D55 (Fig. 4B).

Mini-bulk samples were collected at the second of the two timepoints (D55). We asked whether mini-bulk post-capture samples also detected the neuronal differentiation signal observed in bulks and whether capture improved this detection compared to pre-capture samples. We correlated the expression profiles of TF genes associated with developmental stages in the CORTECON data with expression of the same TFs in D55 mini-bulk pre- and post-capture samples. Post-capture D55 samples showed the highest correlations with the later CORTECON stages (Pearson correlation ≥0.736), compared to pre-capture samples (Pearson correlation ≤0.66), which had lower correlations overall and showed little discrimination between early and later CORTECON stages (Fig. 4C**, S9D**). These results confirm mini-bulk captures are detecting biologically relevant gene expression important for cell stage classification and improving classification compared to pre-capture samples.

## Discussion

We have demonstrated CaptureSeq is able to provide sensitive, reliable and quantitative expression measurements on hundreds to thousands of target genes on samples derived from small numbers of cells. Splicing information within these target genes is maintained and the detection of both known and novel isoforms is enhanced over untargeted sequencing. Comparison of capture on mini-bulk samples with matched standard bulk samples demonstrated very similar performance across multiple capture panels and that both can identify biological relevant information regarding cell state, indicating CaptureSeq is well suited to being performed on ultra-low-input samples. The very similar performance of CaptureSeq on bulk and mini-bulk samples suggests that ultra-low input CaptureSeq will also be widely applicable to samples from many sources and species, including those containing degraded RNA. Our demonstration that 1 ng of RNA input derived from 4000 cells is sufficient for CaptureSeq widens the range of sample types that can be confidently profiled with targeted RNA sequencing. Any sample containing a greater number of cells or more input RNA would be expected to demonstrate good performance, while our input is unlikely to be a lower bound.

The key benefit of CaptureSeq is the large increase in sensitivity and therefore sequencing coverage of target genes of interest [16]. Mini-bulk CaptureSeq produced a 62-fold (TF captures) and 275-fold (NG capture) overall enrichment for target sequences, leading to a 64-fold increase in sensitivity to detect gene expression. CaptureSeq thereby enabled the detection of an extra 10% of targeted genes, which had no reads in any replicate pre-capture. The combination of detecting more genes and more reads per gene led to a ~ 9-fold (NG capture) and ~11-fold (TF captures) increase in the number of known isoforms identified in the mini-bulk samples, demonstrating that CaptureSeq greatly increased the proportion of the transcriptome identifiable in mini-bulk samples.

CaptureSeq in bulk samples has previously been shown to improve the identification of differential expression [2] and we show here it identifies additional DE genes during neuronal differentiation with the potential to improve cell-state identification. Comparison of mini-bulk D55 CaptureSeq to the CORTECON dataset demonstrated it correlated best with later stages of neuronal differentiation, as would be expected if mini-bulk capture retained the cell state and differentiation signal. Furthermore, mini-bulk post-capture samples outperformed pre-capture samples for cell-state identification, suggesting the additional gene expression information obtained through sequence capture can improve our ability to interrogate the underlying biology of ultra-low-input samples.

The enrichment for reads within target genes also has the advantage of greatly reducing the required sequencing depth to obtain similar target gene coverage compared to standard RNA-seq. This can save on sequencing costs and/or allow more samples to be sequenced for the same cost. For example, we conservatively estimate our NG panel (~100 genes, 0.25% of the pre-capture transcriptome) could sequence ten times as many, and TF panel (~1000 genes, 1.4% of the pre-capture transcriptome) could sequence five times as many, mini-bulk samples, respectively, while still providing 10-fold greater read depth than the same number of reads from standard RNA-seq.

Another important feature of the CaptureSeq method is the large number of genes that can be interrogated with no extra experimental effort. Similar to bulk CaptureSeq the number of genes that can be targeted appears limited only by the percentage of the mRNA transcriptome they encompass (as this determines the possible enrichment factor) and that mini-bulk captures of >1000 protein-coding genes are feasible.

A key practical benefit of CaptureSeq is that it can be applied to existing sequencing libraries without the need to generate new material. This means that previous experiments can be re-interrogated at higher resolution using CaptureSeq or re-analysed in a more targeted fashion if the required sequencing depth to detect genes of interest is found to be impractical via standard methods. We demonstrate in this study that this benefit of CaptureSeq can be practically applied to libraries from very low numbers of cells.

The similar performance of CaptureSeq on bulk libraries and mini-bulk libraries made using 1 ng of the RNA extracted from 4000 cells suggests capture could be applicable to even lower numbers of cells. However, while the overall performance of CaptureSeq was similar, mini-bulk samples showed some consistent differences to matched bulk samples, having slightly lower on-target proportions, higher enrichments and lower numbers of detected genes and isoforms (while also producing a greater relative increase in the number of genes and isoforms detected). Although differences in capture performance or sequencing depth could play a role, a likely important factor was the composition and complexity of the mini-bulk and bulk pre-capture libraries. This was supported by the observation that the proportion of on-target reads in mini-bulk pre-capture libraries was less than half that in bulk pre-capture libraries. In libraries from single cells, many genes cannot be detected because they are not incorporated into the library and our results suggest a similar phenomenon may occur in mini-bulk libraries made with similar methods. The recently described RAGE-seq method [46] demonstrated the applicability of capture on single cells for antigen receptor profiling in lymphocytes; however, the ~13-fold enrichment observed and the small number of highly expressed genes targeted means the overall performance of CaptureSeq on single cells remains to be determined. Together these results show capture on mini-bulks samples can produce a larger relative improvement in gene and isoform identification than on bulk samples, but also that enrichment techniques cannot negate limitations imposed by the composition of the libraries themselves.

While several targeted cDNA sequencing methods are now available, CaptureSeq is the first to be validated for ultra-low-input mini-bulk samples. Multiplexed primer extension enables sensitive detection of splice junctions and splicing intermediates but to-date has only been demonstrated in yeast with RNA inputs of 10 ug or more [18]. In contrast, cDNA-smMIP has been performed using mammalian samples but was reported to be less sensitive than CaptureSeq for transcript detection and has not been shown to work with very low sample inputs [19]. Mini-bulk CaptureSeq showed excellent sensitivity for gene and isoform detection, identifying the vast majority of targeted expressed genes and greatly increasing the number of identified mRNA isoforms (which depends on the detection of splice junctions). Even if these alternative methods are validated in ultra-low-input samples, the ‘discovery and quantification’ nature of CaptureSeq; its applicability to many sample types; and its ability to profile previously unannotated mRNAs such as intergenic transcripts [4] or novel oncogenic genes fusions [14], will provide it with many advantages.

In addition to investigating low-input samples, we also investigated the feasibility of performing capture hybridization with less than one-fifth of the recommended input. Lowering hybridization inputs could save money by using less reagents and allow a reduction in the large number of PCR cycles required to amplify libraries from small samples. We found almost identical performance between our standard and low-cDNA hybridizations, though further testing is required to identify the practical lower input limit and ensure consistent performance across different probe pools.

In conclusion, we have validated the effective use of CaptureSeq on ultra-low-input ‘mini-bulk’ samples. In addition, we find capture hybridization can be performed on greatly decreased amounts of cDNA, further facilitating the use of CaptureSeq on low input-samples. Our results suggest that gene expression profiling of mini-bulk samples can greatly benefit from the application of targeted RNA-sequencing.

## Supplementary Material

Supplemental Material

## Data Availability

RNA-seq datasets utilised in this study are available from GEO, accession number: GSE140442.
