# Supplementary material for: Targeted RNA sequencing enhances gene expression profiling of ultra-low input samples
Source: RNA Biol. 2020 Jun 28;17(12):1741–53. doi: 10.1080/15476286.2020.1777768 (PMC7746246; doi:10.1080/15476286.2020.1777768)
Supplement: Supplemental Material [file KRNB_A_1777768_SM6200.zip › Supplemementary_Figures_combined.pdf]

Figure S1

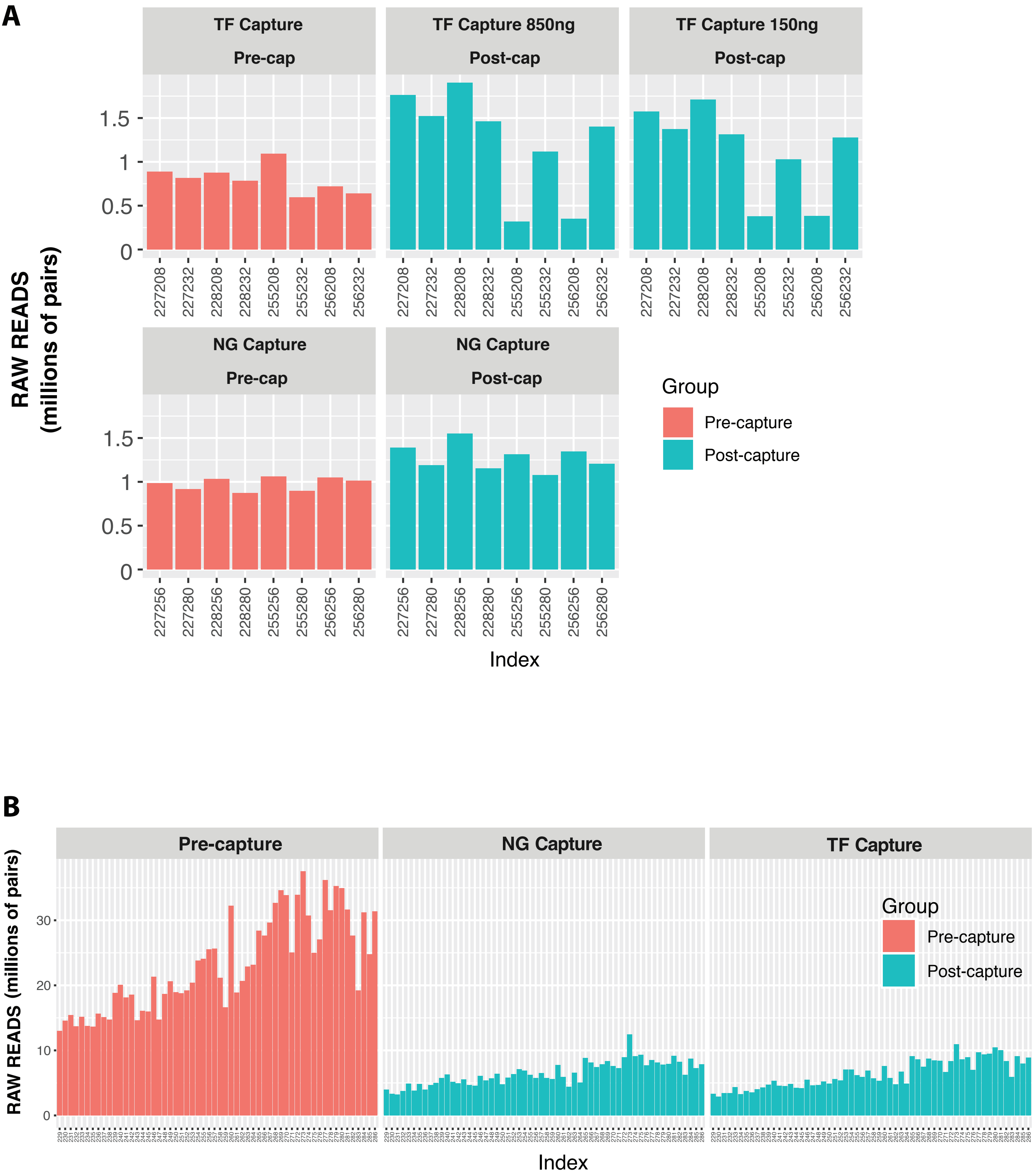
A
TF Capture
Pre-cap
TF Capture 850ng
Post-cap
TF Capture 150ng
Post-cap
NG Capture
Pre-cap
NG Capture
Post-cap
RAW READS
(millions of pairs)
0
0.5
1
1.5
227208
227232
228208
228232
255208
255232
256208
256232
227256
227280
228256
228280
255256
255280
256256
256280
Index
Group
Pre-capture
Post-capture
B
Pre-capture
NG Capture
TF Capture
RAW READS (millions of pairs)
0
10
20
30
Index
Group
Pre-capture
Post-capture

Figure S2

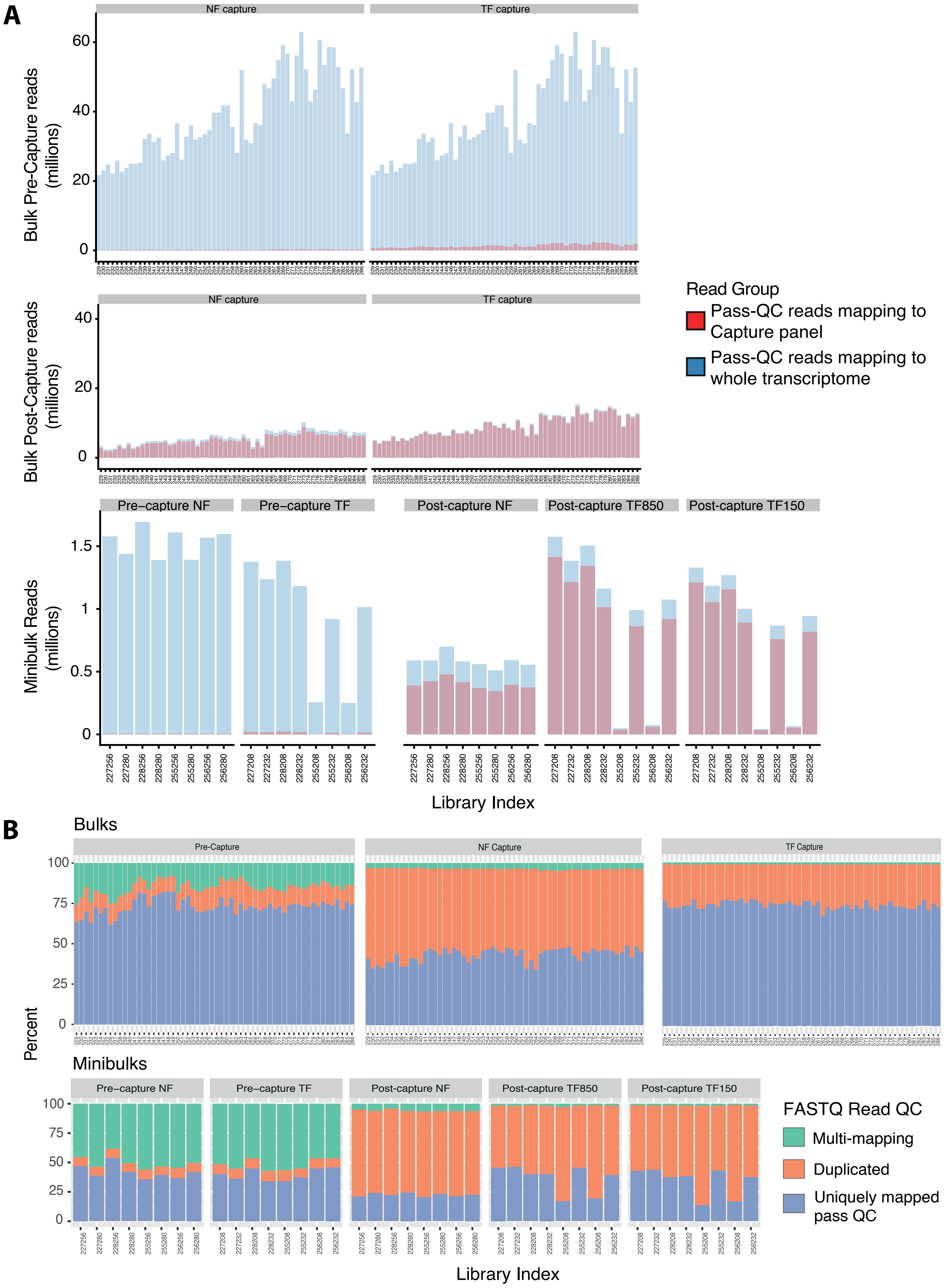

A
NF capture
TF capture
Bulk Pre-Capture reads (millions)
60
40
20
0
Bulk Post-Capture reads (millions)
Read Group
Pass-QC reads mapping to Capture panel
Pass-QC reads mapping to whole transcriptome
Minibulk Reads (millions)
1.5
1
0.5
Pre–capture NF
Pre–capture TF
Post-capture NF
Post-capture TF850
Post-capture TF150
227256
227280
228256
228280
255256
255280
256256
256280
227208
227232
228208
228232
255208
255232
256208
256232
Library Index
B
Bulks
Pre-Capture
NF Capture
TF Capture
100
75
50
25
0
Percent
Minibulks
FASTQ Read QC
Multi-mapping
Duplicated
Uniquely mapped pass QC
Library Index

Figure S3

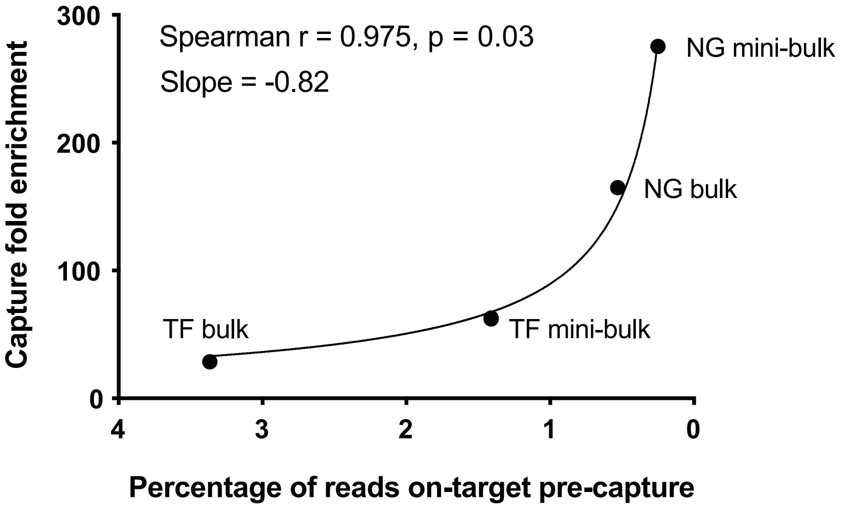
Spearman r = 0.975, p = 0.03
Slope = -0.82
NG mini-bulk
NG bulk
TF mini-bulk
TF bulk
Capture fold enrichment
300
200
100
0
4
3
2
1
0
Percentage of reads on-target pre-capture

Figure S4

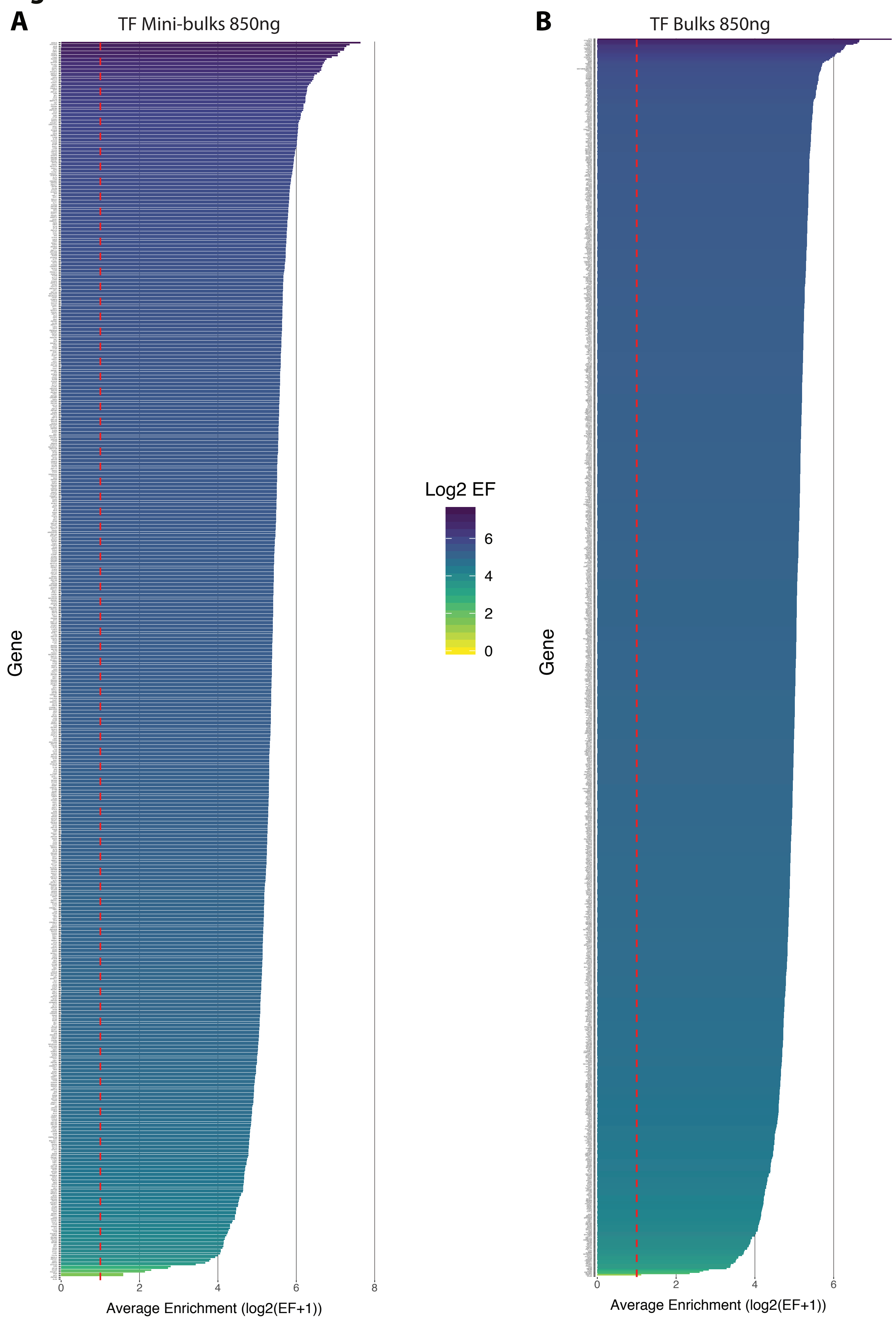
A
TF Mini-bulks 850ng
Gene
Average Enrichment (log2(EF+1))
0
2
4
6
8
Log2 EF
6
4
2
0
B
TF Bulks 850ng
Gene
Average Enrichment (log2(EF+1))
0
2
4
6

Figure S5

A

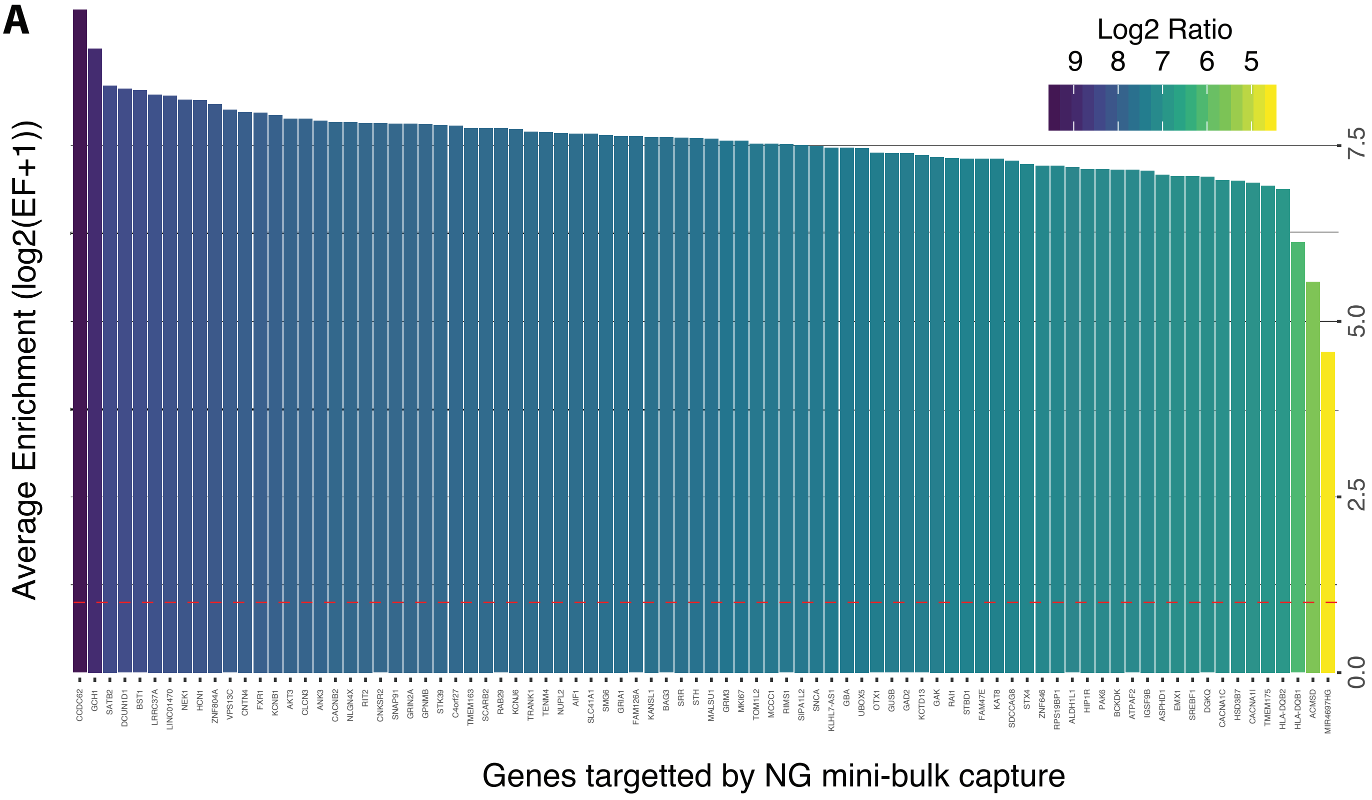
Log2 Ratio
9 8 7 6 5
Average Enrichment (log2(EF+1))
7.5
5.0
2.5
0.0
Genes targetted by NG mini-bulk capture

B

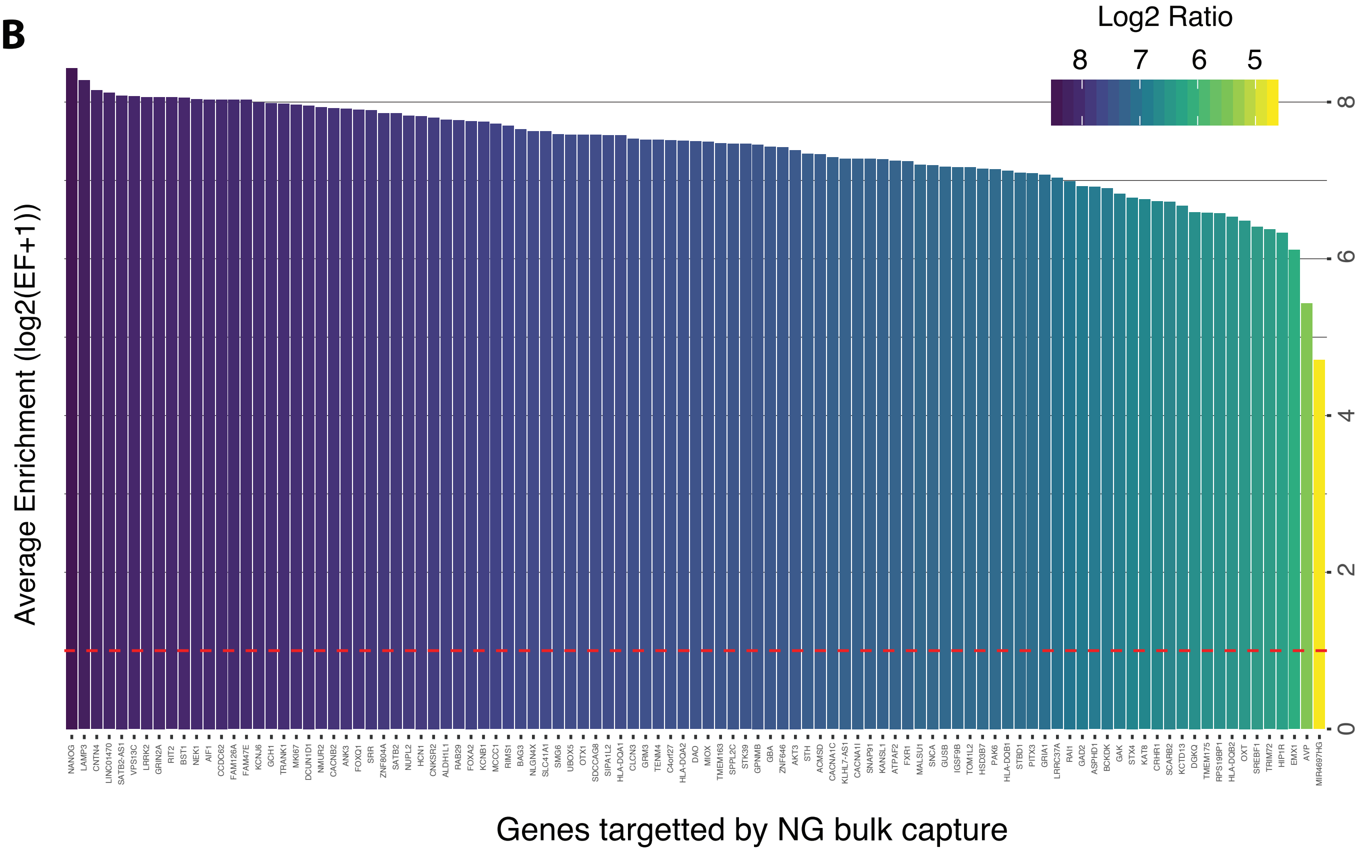
Log2 Ratio
8 7 6 5
Average Enrichment (log2(EF+1))
8
6
4
2
0
Genes targetted by NG bulk capture

Figure S6

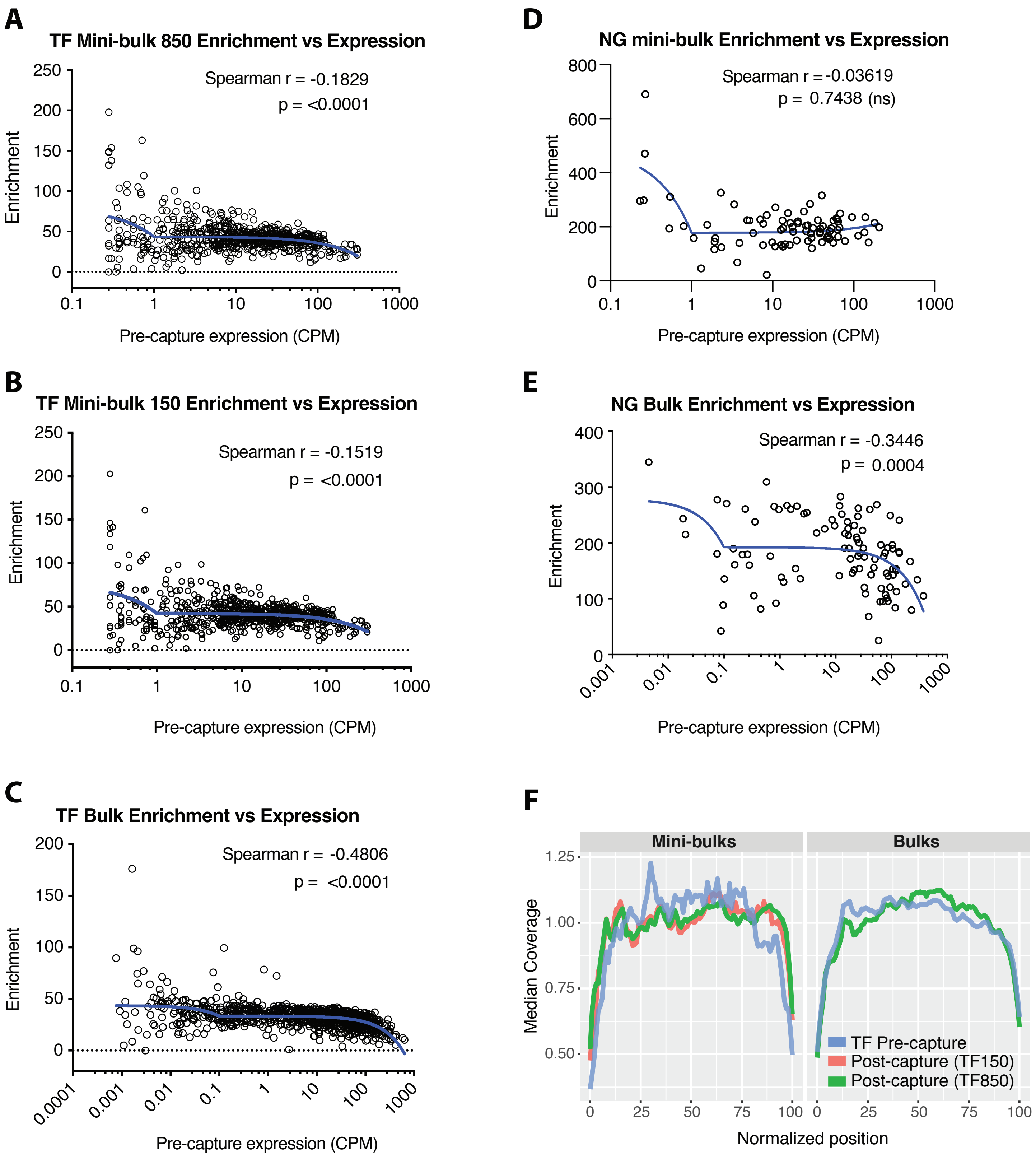

A
TF Mini-bulk 850 Enrichment vs Expression
Spearman r = -0.1829
p = <0.0001
Enrichment
Pre-capture expression (CPM)
0.1
1
10
100
1000
0
50
100
150
200
250
D
NG mini-bulk Enrichment vs Expression
Spearman r = -0.03619
p = 0.7438 (ns)
Enrichment
Pre-capture expression (CPM)
0
200
400
600
800
B
TF Mini-bulk 150 Enrichment vs Expression
Spearman r = -0.1519
p = <0.0001
E
NG Bulk Enrichment vs Expression
Spearman r = -0.3446
p = 0.0004
0.001
0.01
0.1
1
10
100
1000
0
100
200
300
400
C
TF Bulk Enrichment vs Expression
Spearman r = -0.4806
p = <0.0001
0.0001
F
Mini-bulks
Bulks
Median Coverage
Normalized position
0.50
0.75
1.00
1.25
0
25
50
75
100
TF Pre-capture
Post-capture (TF150)
Post-capture (TF850)

Figure S7

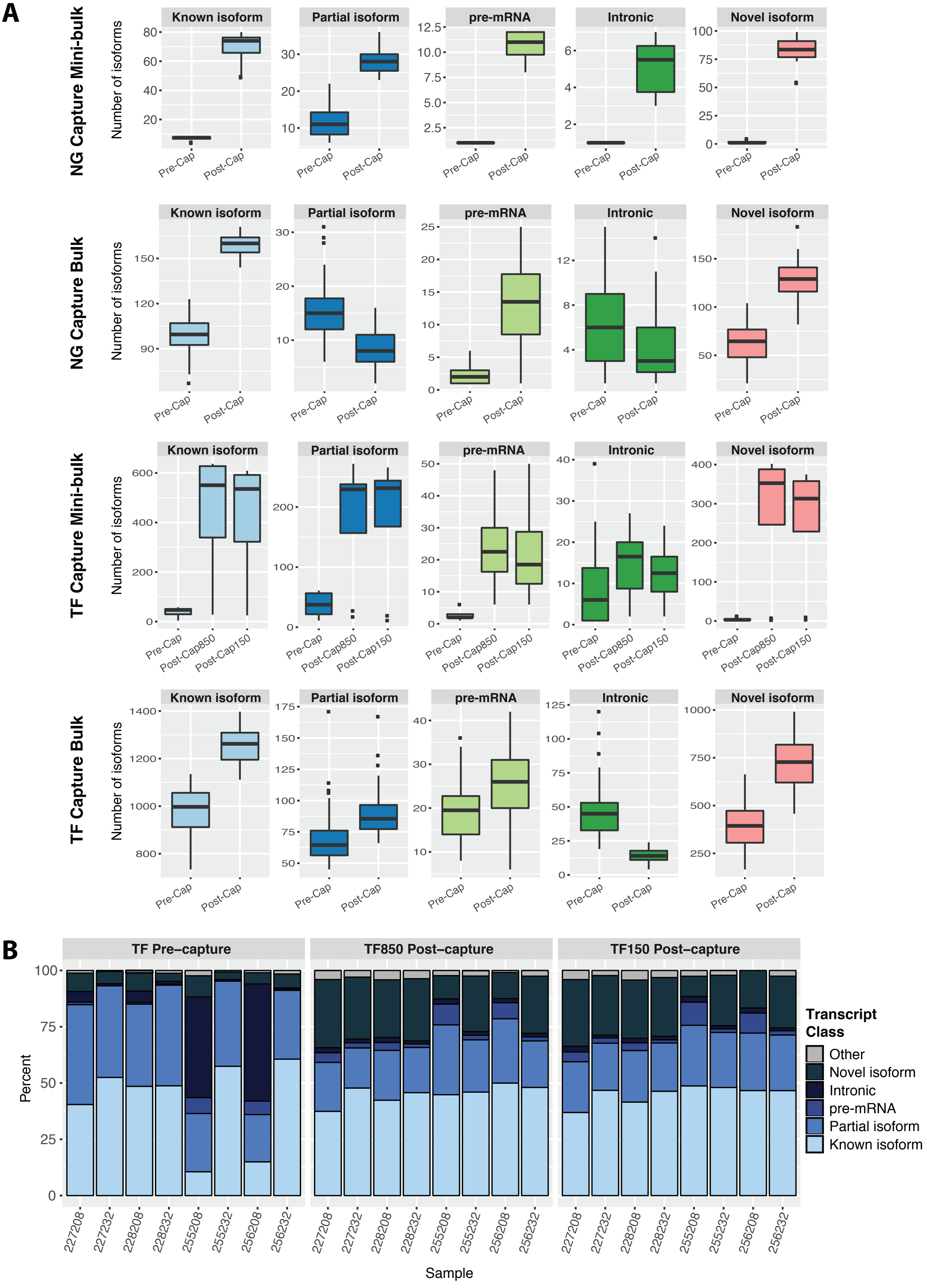
A
NG Capture Mini-bulk
NG Capture Bulk
TF Capture Mini-bulk
TF Capture Bulk
Number of isoforms
Known isoform
Partial isoform
pre-mRNA
Intronic
Novel isoform
Pre-Cap
Post-Cap
Post-Cap850
Post-Cap150
B
TF Pre-capture
TF850 Post-capture
TF150 Post-capture
Percent
Sample
227208
227232
228208
228232
255208
255232
256208
256232
Transcript Class
Other
Novel isoform
Intronic
pre-mRNA
Partial isoform
Known isoform

Figure S8

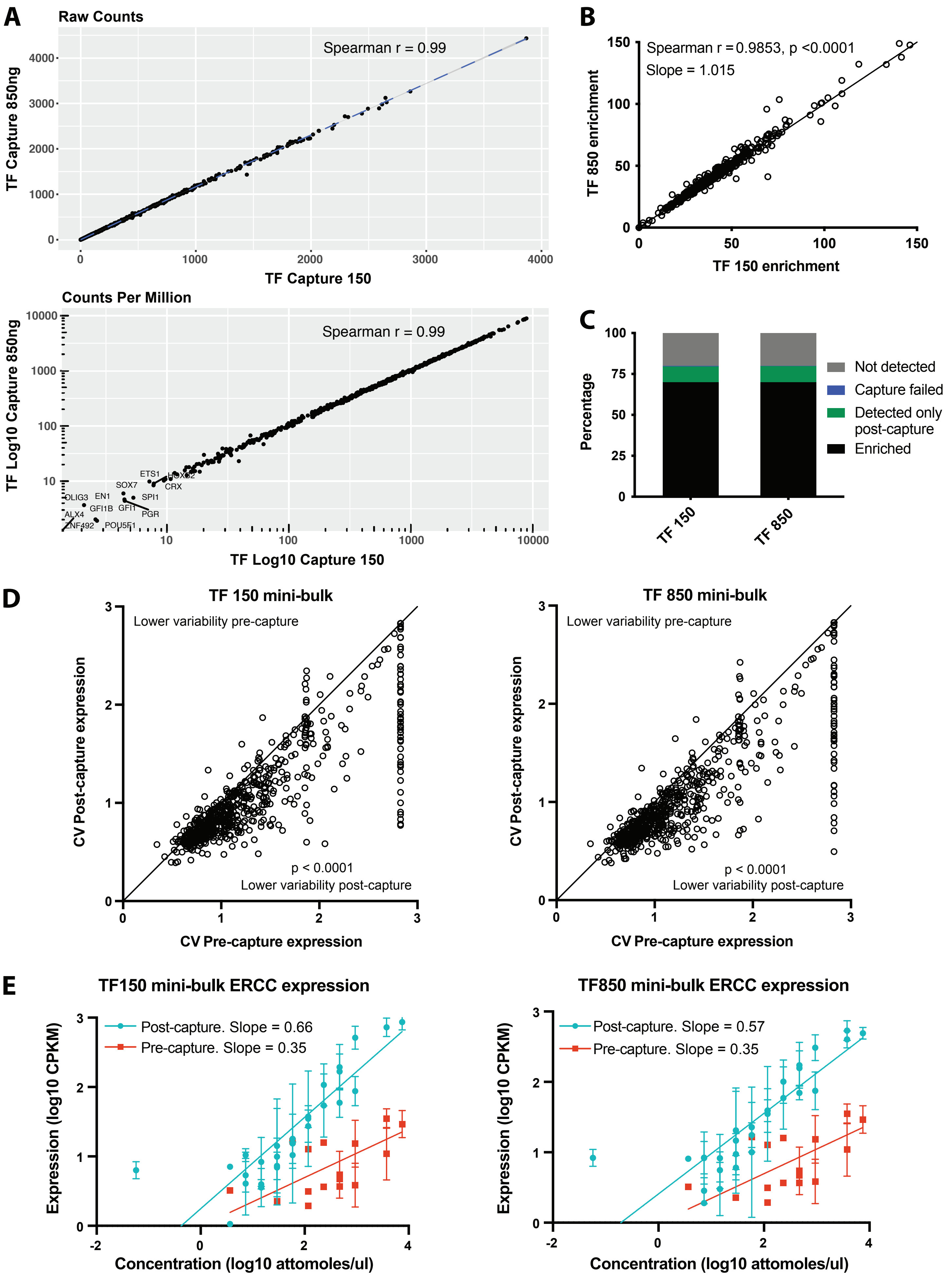

A
Raw Counts
Spearman r = 0.99
TF Capture 850ng
TF Capture 150
Counts Per Million
Spearman r = 0.99
TF Log10 Capture 850ng
TF Log10 Capture 150
ETS1
HOXB2
CRX
SOX7
SPI1
OLIG3
EN1
GFI1B
GFI1
ALX4
PGR
ZNF492
POU5F1
B
Spearman r = 0.9853, p <0.0001
Slope = 1.015
TF 850 enrichment
TF 150 enrichment
C
Percentage
Not detected
Capture failed
Detected only post-capture
Enriched
TF 150
TF 850
D
TF 150 mini-bulk
Lower variability pre-capture
p < 0.0001
Lower variability post-capture
CV Post-capture expression
CV Pre-capture expression
TF 850 mini-bulk
Lower variability pre-capture
p < 0.0001
Lower variability post-capture
CV Post-capture expression
CV Pre-capture expression
E
TF150 mini-bulk ERCC expression
Post-capture. Slope = 0.66
Pre-capture. Slope = 0.35
Expression (log10 CPKM)
Concentration (log10 attomoles/ul)
TF850 mini-bulk ERCC expression
Post-capture. Slope = 0.57
Pre-capture. Slope = 0.35
Expression (log10 CPKM)
Concentration (log10 attomoles/ul)

Figure S9

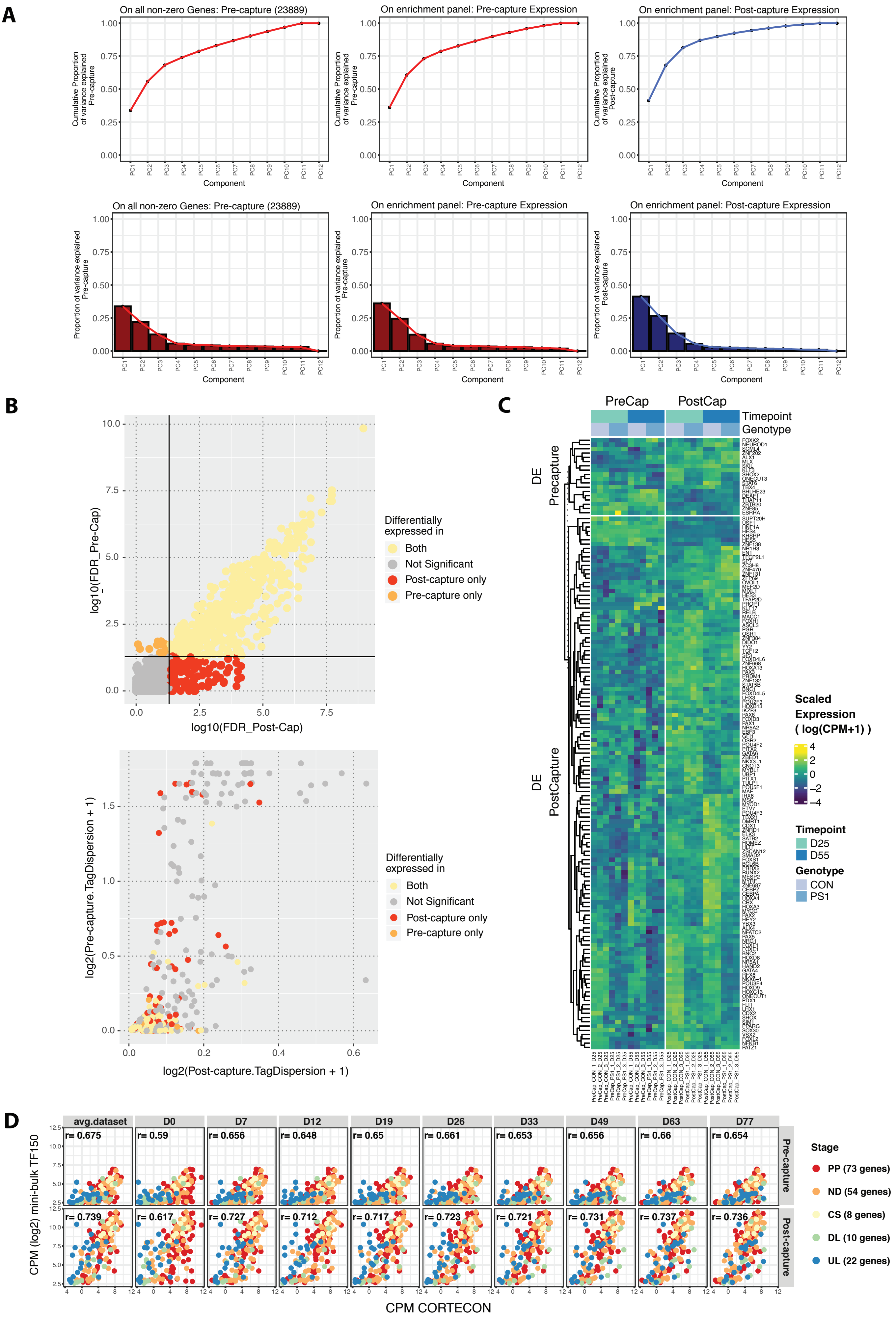
A
On all non-zero Genes: Pre-capture (23889)
On enrichment panel: Pre-capture Expression
On enrichment panel: Post-capture Expression
Cumulative Proportion of variance explained Pre-capture
Cumulative Proportion of variance explained Post-capture
Proportion of variance explained Pre-capture
Proportion of variance explained Post-capture
Component
PC1 PC2 PC3 PC4 PC5 PC6 PC7 PC8 PC9 PC10 PC11 PC12
0.00 0.25 0.50 0.75 1.00
B
log10(FDR_Pre-Cap)
log10(FDR_Post-Cap)
Differentially expressed in
Both
Not Significant
Post-capture only
Pre-capture only
log2(Pre-capture.TagDispersion + 1)
log2(Post-capture.TagDispersion + 1)
C
PreCap
PostCap
Timepoint
Genotype
DE Precapture
DE PostCapture
Scaled Expression (log(CPM+1))
Timepoint
D25
D55
Genotype
CON
PS1
D
avg.dataset
D0
D7
D12
D19
D26
D33
D49
D63
D77
r= 0.675
r= 0.59
r= 0.656
r= 0.648
r= 0.65
r= 0.661
r= 0.653
r= 0.656
r= 0.66
r= 0.654
r= 0.739
r= 0.617
r= 0.727
r= 0.712
r= 0.717
r= 0.723
r= 0.721
r= 0.731
r= 0.737
r= 0.736
Pre-capture
Post-capture
CPM (log2) mini-bulk TF150
CPM CORTECON
Stage
PP (73 genes)
ND (54 genes)
CS (8 genes)
DL (10 genes)
UL (22 genes)
