# Supplementary material for: Targeted RNA sequencing enhances gene expression profiling of ultra-low input samples
Source: RNA Biol. 2020 Jun 28;17(12):1741–53. doi: 10.1080/15476286.2020.1777768 (PMC7746246; doi:10.1080/15476286.2020.1777768)
Supplement: Supplemental Material [file KRNB_A_1777768_SM6200.zip › TableS1_NeuroGWAS_capture_targets_hg19.pdf]

Chromosome	Start_coordinate	End_coordinate	Original_target_region
chr1	4180563	4183988	chr1:4180589-4187821
chr1	4184263	4184483	chr1:4180589-4187821
chr1	4184788	4185540	chr1:4180589-4187821
chr1	4185823	4186807	chr1:4180589-4187821
chr1	4186818	4187170	chr1:4180589-4187821
chr1	4187313	4187397	chr1:4180589-4187821
chr1	4187428	4187861	chr1:4180589-4187821
chr1	4188153	4188268	chr1:4188200-4188226
chr1	4192583	4192974	chr1:4192616-4192942
chr1	4193853	4194246	chr1:4193887-4194222
chr1	30409587	30410354	chr1:30409608-30434937
chr1	30410392	30411038	chr1:30409608-30434937
chr1	30411327	30411454	chr1:30409608-30434937
chr1	30411462	30411717	chr1:30409608-30434937
chr1	30411722	30412455	chr1:30409608-30434937
chr1	30412467	30416089	chr1:30409608-30434937
chr1	30416092	30416515	chr1:30409608-30434937
chr1	30416802	30417938	chr1:30409608-30434937
chr1	30418207	30418751	chr1:30409608-30434937
chr1	30418752	30418843	chr1:30409608-30434937
chr1	30418852	30419877	chr1:30409608-30434937
chr1	30420167	30421458	chr1:30409608-30434937
chr1	30421582	30428849	chr1:30409608-30434937
chr1	30429182	30430511	chr1:30409608-30434937
chr1	30430512	30430963	chr1:30409608-30434937
chr1	30430987	30432765	chr1:30409608-30434937
chr1	30432852	30434964	chr1:30409608-30434937
chr1	30464052	30464397	chr1:30464077-30464372
chr1	30464642	30465284	chr1:30464674-30465246
chr1	30482532	30482610	chr1:30482562-30482575
chr1	30488057	30488168	chr1:30488090-30488142
chr1	30489537	30489777	chr1:30489566-30489768
chr1	30497842	30498049	chr1:30497871-30498030
chr1	30502107	30502349	chr1:30502136-30502337
chr1	30510312	30510495	chr1:30510344-30510459
chr1	44098885	44098961	chr1:44098904-44099804
chr1	44099065	44099317	chr1:44098904-44099804
chr1	44099350	44099539	chr1:44098904-44099804
chr1	44099915	44100833	chr1:44099942-44110656
chr1	44101465	44101544	chr1:44099942-44110656
chr1	44101570	44102104	chr1:44099942-44110656
chr1	44102110	44102186	chr1:44099942-44110656
chr1	44102465	44102539	chr1:44099942-44110656
chr1	44102540	44102959	chr1:44099942-44110656
chr1	44103155	44103480	chr1:44099942-44110656
chr1	44103755	44103912	chr1:44099942-44110656
chr1	44104630	44104849	chr1:44099942-44110656
chr1	44105110	44105280	chr1:44099942-44110656
chr1	44105560	44105992	chr1:44099942-44110656
chr1	44106265	44106579	chr1:44099942-44110656
chr1	44106580	44106788	chr1:44099942-44110656
chr1	44107190	44107312	chr1:44099942-44110656
chr1	44108160	44108471	chr1:44099942-44110656
chr1	44108475	44108558	chr1:44099942-44110656
chr1	44108665	44108805	chr1:44099942-44110656
chr1	44108845	44108962	chr1:44099942-44110656
chr1	44108980	44109104	chr1:44099942-44110656
chr1	44109160	44109484	chr1:44099942-44110656
chr1	44110075	44110360	chr1:44099942-44110656
chr1	44110460	44110680	chr1:44099942-44110656
chr1	73651171	73651265	chr1:73651204-73657323
chr1	73651316	73653308	chr1:73651204-73657323

chr1	73653316	73654741	chr1:73651204-73657323
chr1	73654746	73655316	chr1:73651204-73657323
chr1	73655321	73655944	chr1:73651204-73657323
chr1	73655956	73656056	chr1:73651204-73657323
chr1	73656116	73656484	chr1:73651204-73657323
chr1	73656536	73656646	chr1:73651204-73657323
chr1	73656761	73657351	chr1:73651204-73657323
chr1	73657546	73657811	chr1:73657580-74032392
chr1	73657816	73658174	chr1:73657580-74032392
chr1	73658181	73658900	chr1:73657580-74032392
chr1	73658901	73659292	chr1:73657580-74032392
chr1	73660686	73661169	chr1:73657580-74032392
chr1	73661471	73661646	chr1:73657580-74032392
chr1	73661911	73662198	chr1:73657580-74032392
chr1	73662531	73662639	chr1:73657580-74032392
chr1	73662646	73663613	chr1:73657580-74032392
chr1	73663621	73665609	chr1:73657580-74032392
chr1	73665611	73666181	chr1:73657580-74032392
chr1	73666201	73666662	chr1:73657580-74032392
chr1	73667376	73667450	chr1:73657580-74032392
chr1	73667471	73668294	chr1:73657580-74032392
chr1	73668306	73668382	chr1:73657580-74032392
chr1	73668386	73668817	chr1:73657580-74032392
chr1	73668891	73668964	chr1:73657580-74032392
chr1	73669091	73669264	chr1:73657580-74032392
chr1	73669356	73669484	chr1:73657580-74032392
chr1	73669536	73672096	chr1:73657580-74032392
chr1	73672136	73672503	chr1:73657580-74032392
chr1	73672561	73672784	chr1:73657580-74032392
chr1	73672786	73672974	chr1:73657580-74032392
chr1	73672986	73673269	chr1:73657580-74032392
chr1	73673301	73673379	chr1:73657580-74032392
chr1	73673386	73673734	chr1:73657580-74032392
chr1	73673736	73673813	chr1:73657580-74032392
chr1	73673896	73674071	chr1:73657580-74032392
chr1	73674126	73674282	chr1:73657580-74032392
chr1	73674296	73674495	chr1:73657580-74032392
chr1	73674756	73674905	chr1:73657580-74032392
chr1	73675286	73675436	chr1:73657580-74032392
chr1	73675631	73675707	chr1:73657580-74032392
chr1	73675951	73676045	chr1:73657580-74032392
chr1	73676081	73676198	chr1:73657580-74032392
chr1	73676411	73676506	chr1:73657580-74032392
chr1	73676526	73676607	chr1:73657580-74032392
chr1	73676726	73676811	chr1:73657580-74032392
chr1	73676856	73677028	chr1:73657580-74032392
chr1	73677031	73678545	chr1:73657580-74032392
chr1	73679016	73679255	chr1:73657580-74032392
chr1	73679256	73679677	chr1:73657580-74032392
chr1	73679736	73679958	chr1:73657580-74032392
chr1	73679971	73681478	chr1:73657580-74032392
chr1	73681506	73683867	chr1:73657580-74032392
chr1	73684166	73686263	chr1:73657580-74032392
chr1	73686321	73686396	chr1:73657580-74032392
chr1	73686506	73686607	chr1:73657580-74032392
chr1	73686826	73686901	chr1:73657580-74032392
chr1	73687241	73687329	chr1:73657580-74032392
chr1	73687626	73687702	chr1:73657580-74032392
chr1	73687736	73687814	chr1:73657580-74032392
chr1	73687981	73688059	chr1:73657580-74032392
chr1	73688331	73688413	chr1:73657580-74032392
chr1	73688631	73689042	chr1:73657580-74032392
chr1	73689046	73689613	chr1:73657580-74032392

chr1	73689891	73692501	chr1:73657580-74032392
chr1	73692536	73692624	chr1:73657580-74032392
chr1	73692741	73692823	chr1:73657580-74032392
chr1	73692841	73692917	chr1:73657580-74032392
chr1	73693061	73695450	chr1:73657580-74032392
chr1	73695486	73695652	chr1:73657580-74032392
chr1	73695821	73695917	chr1:73657580-74032392
chr1	73696221	73696390	chr1:73657580-74032392
chr1	73696436	73697299	chr1:73657580-74032392
chr1	73697321	73698657	chr1:73657580-74032392
chr1	73698971	73699796	chr1:73657580-74032392
chr1	73699801	73700070	chr1:73657580-74032392
chr1	73700136	73700623	chr1:73657580-74032392
chr1	73700896	73701512	chr1:73657580-74032392
chr1	73701516	73701696	chr1:73657580-74032392
chr1	73701701	73704120	chr1:73657580-74032392
chr1	73706376	73706800	chr1:73657580-74032392
chr1	73706901	73707451	chr1:73657580-74032392
chr1	73707456	73707637	chr1:73657580-74032392
chr1	73707661	73708175	chr1:73657580-74032392
chr1	73708211	73708504	chr1:73657580-74032392
chr1	73708556	73708625	chr1:73657580-74032392
chr1	73708646	73708802	chr1:73657580-74032392
chr1	73708816	73709217	chr1:73657580-74032392
chr1	73709231	73709372	chr1:73657580-74032392
chr1	73709431	73709519	chr1:73657580-74032392
chr1	73709561	73709796	chr1:73657580-74032392
chr1	73709921	73709989	chr1:73657580-74032392
chr1	73710021	73710097	chr1:73657580-74032392
chr1	73710101	73710214	chr1:73657580-74032392
chr1	73710561	73710633	chr1:73657580-74032392
chr1	73710706	73710850	chr1:73657580-74032392
chr1	73711191	73711373	chr1:73657580-74032392
chr1	73711676	73711982	chr1:73657580-74032392
chr1	73711986	73713467	chr1:73657580-74032392
chr1	73713471	73713799	chr1:73657580-74032392
chr1	73713811	73714136	chr1:73657580-74032392
chr1	73714246	73715550	chr1:73657580-74032392
chr1	73715566	73715685	chr1:73657580-74032392
chr1	73715691	73715874	chr1:73657580-74032392
chr1	73716156	73716760	chr1:73657580-74032392
chr1	73716916	73719275	chr1:73657580-74032392
chr1	73719276	73719344	chr1:73657580-74032392
chr1	73719351	73719491	chr1:73657580-74032392
chr1	73719591	73719724	chr1:73657580-74032392
chr1	73719731	73719829	chr1:73657580-74032392
chr1	73720371	73720647	chr1:73657580-74032392
chr1	73720901	73721570	chr1:73657580-74032392
chr1	73721576	73722327	chr1:73657580-74032392
chr1	73722616	73723017	chr1:73657580-74032392
chr1	73723071	73723285	chr1:73657580-74032392
chr1	73723776	73725062	chr1:73657580-74032392
chr1	73725066	73725701	chr1:73657580-74032392
chr1	73725771	73725858	chr1:73657580-74032392
chr1	73725901	73726228	chr1:73657580-74032392
chr1	73726291	73727416	chr1:73657580-74032392
chr1	73727456	73728330	chr1:73657580-74032392
chr1	73728331	73729451	chr1:73657580-74032392
chr1	73729536	73729769	chr1:73657580-74032392
chr1	73729791	73730670	chr1:73657580-74032392
chr1	73730681	73730844	chr1:73657580-74032392
chr1	73730851	73731339	chr1:73657580-74032392
chr1	73731406	73731484	chr1:73657580-74032392

chr1	73731521	73731613	chr1:73657580-74032392
chr1	73731621	73731877	chr1:73657580-74032392
chr1	73731951	73732107	chr1:73657580-74032392
chr1	73732121	73732345	chr1:73657580-74032392
chr1	73732371	73732442	chr1:73657580-74032392
chr1	73732466	73732683	chr1:73657580-74032392
chr1	73732721	73733016	chr1:73657580-74032392
chr1	73733176	73733619	chr1:73657580-74032392
chr1	73733636	73733704	chr1:73657580-74032392
chr1	73733811	73734241	chr1:73657580-74032392
chr1	73734606	73734718	chr1:73657580-74032392
chr1	73734966	73735147	chr1:73657580-74032392
chr1	73735186	73735286	chr1:73657580-74032392
chr1	73735316	73735502	chr1:73657580-74032392
chr1	73735521	73735615	chr1:73657580-74032392
chr1	73735631	73735714	chr1:73657580-74032392
chr1	73735726	73735851	chr1:73657580-74032392
chr1	73735856	73735941	chr1:73657580-74032392
chr1	73736046	73736118	chr1:73657580-74032392
chr1	73736131	73736236	chr1:73657580-74032392
chr1	73736361	73736436	chr1:73657580-74032392
chr1	73736576	73736799	chr1:73657580-74032392
chr1	73737061	73737367	chr1:73657580-74032392
chr1	73737376	73738224	chr1:73657580-74032392
chr1	73738231	73738375	chr1:73657580-74032392
chr1	73738386	73741018	chr1:73657580-74032392
chr1	73741021	73743229	chr1:73657580-74032392
chr1	73743466	73744557	chr1:73657580-74032392
chr1	73744566	73745863	chr1:73657580-74032392
chr1	73745871	73746348	chr1:73657580-74032392
chr1	73746386	73747903	chr1:73657580-74032392
chr1	73747931	73748165	chr1:73657580-74032392
chr1	73748171	73748400	chr1:73657580-74032392
chr1	73748466	73748900	chr1:73657580-74032392
chr1	73748976	73749047	chr1:73657580-74032392
chr1	73749056	73749229	chr1:73657580-74032392
chr1	73749231	73749832	chr1:73657580-74032392
chr1	73749841	73749951	chr1:73657580-74032392
chr1	73750561	73750639	chr1:73657580-74032392
chr1	73751081	73751161	chr1:73657580-74032392
chr1	73751296	73751834	chr1:73657580-74032392
chr1	73751836	73752183	chr1:73657580-74032392
chr1	73752186	73752261	chr1:73657580-74032392
chr1	73752271	73753063	chr1:73657580-74032392
chr1	73753071	73753604	chr1:73657580-74032392
chr1	73753906	73754273	chr1:73657580-74032392
chr1	73754276	73755219	chr1:73657580-74032392
chr1	73755226	73755728	chr1:73657580-74032392
chr1	73756041	73756305	chr1:73657580-74032392
chr1	73756371	73756791	chr1:73657580-74032392
chr1	73756801	73756916	chr1:73657580-74032392
chr1	73758056	73760417	chr1:73657580-74032392
chr1	73760426	73761237	chr1:73657580-74032392
chr1	73761246	73762950	chr1:73657580-74032392
chr1	73763001	73763113	chr1:73657580-74032392
chr1	73763121	73763658	chr1:73657580-74032392
chr1	73763946	73764093	chr1:73657580-74032392
chr1	73764776	73765342	chr1:73657580-74032392
chr1	73765351	73765987	chr1:73657580-74032392
chr1	73766056	73768602	chr1:73657580-74032392
chr1	73768606	73768807	chr1:73657580-74032392
chr1	73769126	73770536	chr1:73657580-74032392
chr1	73770541	73772295	chr1:73657580-74032392

chr1	73772306	73772468	chr1:73657580-74032392
chr1	73772476	73773508	chr1:73657580-74032392
chr1	73773531	73773804	chr1:73657580-74032392
chr1	73773806	73773889	chr1:73657580-74032392
chr1	73773891	73774215	chr1:73657580-74032392
chr1	73774236	73774667	chr1:73657580-74032392
chr1	73774671	73777977	chr1:73657580-74032392
chr1	73778316	73778587	chr1:73657580-74032392
chr1	73778606	73779169	chr1:73657580-74032392
chr1	73779526	73780704	chr1:73657580-74032392
chr1	73780961	73781254	chr1:73657580-74032392
chr1	73781261	73781472	chr1:73657580-74032392
chr1	73781476	73783549	chr1:73657580-74032392
chr1	73783556	73784896	chr1:73657580-74032392
chr1	73784931	73785362	chr1:73657580-74032392
chr1	73785391	73786846	chr1:73657580-74032392
chr1	73786911	73787075	chr1:73657580-74032392
chr1	73787216	73787329	chr1:73657580-74032392
chr1	73787526	73787622	chr1:73657580-74032392
chr1	73787746	73787813	chr1:73657580-74032392
chr1	73787921	73788056	chr1:73657580-74032392
chr1	73788116	73788250	chr1:73657580-74032392
chr1	73788306	73788404	chr1:73657580-74032392
chr1	73788586	73788731	chr1:73657580-74032392
chr1	73788856	73789062	chr1:73657580-74032392
chr1	73789081	73789193	chr1:73657580-74032392
chr1	73789201	73789285	chr1:73657580-74032392
chr1	73789391	73789471	chr1:73657580-74032392
chr1	73789536	73790121	chr1:73657580-74032392
chr1	73790126	73790239	chr1:73657580-74032392
chr1	73790291	73790734	chr1:73657580-74032392
chr1	73790816	73795239	chr1:73657580-74032392
chr1	73796821	73797072	chr1:73657580-74032392
chr1	73797351	73797457	chr1:73657580-74032392
chr1	73797471	73797928	chr1:73657580-74032392
chr1	73797956	73798626	chr1:73657580-74032392
chr1	73798636	73798791	chr1:73657580-74032392
chr1	73798896	73800667	chr1:73657580-74032392
chr1	73800706	73800890	chr1:73657580-74032392
chr1	73800896	73801709	chr1:73657580-74032392
chr1	73801726	73802427	chr1:73657580-74032392
chr1	73802676	73803677	chr1:73657580-74032392
chr1	73803716	73804406	chr1:73657580-74032392
chr1	73804411	73805368	chr1:73657580-74032392
chr1	73805586	73806872	chr1:73657580-74032392
chr1	73807146	73807957	chr1:73657580-74032392
chr1	73807976	73808610	chr1:73657580-74032392
chr1	73808621	73808804	chr1:73657580-74032392
chr1	73808896	73809077	chr1:73657580-74032392
chr1	73809126	73809204	chr1:73657580-74032392
chr1	73809836	73809909	chr1:73657580-74032392
chr1	73809921	73809998	chr1:73657580-74032392
chr1	73810156	73810264	chr1:73657580-74032392
chr1	73810841	73810970	chr1:73657580-74032392
chr1	73811096	73812279	chr1:73657580-74032392
chr1	73812286	73812879	chr1:73657580-74032392
chr1	73812881	73813609	chr1:73657580-74032392
chr1	73813611	73814882	chr1:73657580-74032392
chr1	73814886	73815216	chr1:73657580-74032392
chr1	73815286	73816121	chr1:73657580-74032392
chr1	73816126	73816866	chr1:73657580-74032392
chr1	73816906	73817961	chr1:73657580-74032392
chr1	73817966	73818034	chr1:73657580-74032392

chr1	73818036	73818635	chr1:73657580-74032392
chr1	73818646	73819805	chr1:73657580-74032392
chr1	73819811	73822006	chr1:73657580-74032392
chr1	73822081	73822187	chr1:73657580-74032392
chr1	73822346	73823191	chr1:73657580-74032392
chr1	73823261	73823464	chr1:73657580-74032392
chr1	73823481	73823726	chr1:73657580-74032392
chr1	73823736	73824509	chr1:73657580-74032392
chr1	73824516	73824803	chr1:73657580-74032392
chr1	73824826	73825114	chr1:73657580-74032392
chr1	73825161	73826069	chr1:73657580-74032392
chr1	73826131	73826472	chr1:73657580-74032392
chr1	73826486	73827309	chr1:73657580-74032392
chr1	73827606	73828490	chr1:73657580-74032392
chr1	73828536	73828724	chr1:73657580-74032392
chr1	73828746	73829038	chr1:73657580-74032392
chr1	73829311	73829388	chr1:73657580-74032392
chr1	73829426	73830047	chr1:73657580-74032392
chr1	73830186	73830511	chr1:73657580-74032392
chr1	73830516	73830593	chr1:73657580-74032392
chr1	73830601	73831293	chr1:73657580-74032392
chr1	73831296	73833060	chr1:73657580-74032392
chr1	73833066	73834070	chr1:73657580-74032392
chr1	73834081	73836716	chr1:73657580-74032392
chr1	73837021	73837851	chr1:73657580-74032392
chr1	73838081	73838340	chr1:73657580-74032392
chr1	73838341	73839514	chr1:73657580-74032392
chr1	73839526	73840479	chr1:73657580-74032392
chr1	73840486	73840667	chr1:73657580-74032392
chr1	73840721	73841489	chr1:73657580-74032392
chr1	73841501	73842006	chr1:73657580-74032392
chr1	73842331	73843815	chr1:73657580-74032392
chr1	73843816	73844671	chr1:73657580-74032392
chr1	73845376	73846363	chr1:73657580-74032392
chr1	73846636	73846818	chr1:73657580-74032392
chr1	73846871	73847050	chr1:73657580-74032392
chr1	73847056	73847690	chr1:73657580-74032392
chr1	73847696	73848773	chr1:73657580-74032392
chr1	73848781	73849444	chr1:73657580-74032392
chr1	73849456	73849828	chr1:73657580-74032392
chr1	73849836	73849915	chr1:73657580-74032392
chr1	73849936	73850154	chr1:73657580-74032392
chr1	73850306	73850423	chr1:73657580-74032392
chr1	73850456	73851090	chr1:73657580-74032392
chr1	73851096	73852224	chr1:73657580-74032392
chr1	73852286	73855266	chr1:73657580-74032392
chr1	73855281	73855711	chr1:73657580-74032392
chr1	73855876	73856164	chr1:73657580-74032392
chr1	73856416	73856550	chr1:73657580-74032392
chr1	73856551	73856910	chr1:73657580-74032392
chr1	73856931	73857971	chr1:73657580-74032392
chr1	73857991	73858416	chr1:73657580-74032392
chr1	73858721	73859227	chr1:73657580-74032392
chr1	73859481	73860045	chr1:73657580-74032392
chr1	73860051	73862108	chr1:73657580-74032392
chr1	73862116	73862520	chr1:73657580-74032392
chr1	73862526	73862766	chr1:73657580-74032392
chr1	73862771	73863290	chr1:73657580-74032392
chr1	73863621	73864914	chr1:73657580-74032392
chr1	73864926	73865234	chr1:73657580-74032392
chr1	73865236	73866330	chr1:73657580-74032392
chr1	73866331	73866990	chr1:73657580-74032392
chr1	73867276	73868241	chr1:73657580-74032392

chr1	73868391	73868815	chr1:73657580-74032392
chr1	73868821	73870292	chr1:73657580-74032392
chr1	73870301	73871140	chr1:73657580-74032392
chr1	73871141	73871459	chr1:73657580-74032392
chr1	73871711	73873044	chr1:73657580-74032392
chr1	73873231	73873318	chr1:73657580-74032392
chr1	73874016	73874408	chr1:73657580-74032392
chr1	73874416	73874668	chr1:73657580-74032392
chr1	73874676	73876083	chr1:73657580-74032392
chr1	73876391	73876715	chr1:73657580-74032392
chr1	73876721	73878003	chr1:73657580-74032392
chr1	73878056	73879025	chr1:73657580-74032392
chr1	73879181	73879754	chr1:73657580-74032392
chr1	73879996	73881109	chr1:73657580-74032392
chr1	73881111	73882233	chr1:73657580-74032392
chr1	73882241	73884309	chr1:73657580-74032392
chr1	73884331	73884688	chr1:73657580-74032392
chr1	73884736	73885968	chr1:73657580-74032392
chr1	73886141	73886241	chr1:73657580-74032392
chr1	73886316	73888148	chr1:73657580-74032392
chr1	73888176	73888556	chr1:73657580-74032392
chr1	73888836	73889198	chr1:73657580-74032392
chr1	73889206	73890822	chr1:73657580-74032392
chr1	73891191	73891543	chr1:73657580-74032392
chr1	73891871	73892850	chr1:73657580-74032392
chr1	73892856	73893074	chr1:73657580-74032392
chr1	73893086	73893679	chr1:73657580-74032392
chr1	73893681	73894135	chr1:73657580-74032392
chr1	73894136	73894419	chr1:73657580-74032392
chr1	73894471	73894610	chr1:73657580-74032392
chr1	73897191	73897285	chr1:73657580-74032392
chr1	73897286	73899098	chr1:73657580-74032392
chr1	73899106	73900289	chr1:73657580-74032392
chr1	73900291	73901497	chr1:73657580-74032392
chr1	73901826	73901952	chr1:73657580-74032392
chr1	73901961	73902303	chr1:73657580-74032392
chr1	73902306	73902540	chr1:73657580-74032392
chr1	73902541	73902787	chr1:73657580-74032392
chr1	73902846	73902927	chr1:73657580-74032392
chr1	73903526	73903928	chr1:73657580-74032392
chr1	73903931	73904064	chr1:73657580-74032392
chr1	73904171	73904270	chr1:73657580-74032392
chr1	73904406	73904503	chr1:73657580-74032392
chr1	73904611	73904722	chr1:73657580-74032392
chr1	73904731	73904850	chr1:73657580-74032392
chr1	73905166	73905242	chr1:73657580-74032392
chr1	73905446	73905523	chr1:73657580-74032392
chr1	73905931	73906505	chr1:73657580-74032392
chr1	73906506	73906939	chr1:73657580-74032392
chr1	73906951	73907491	chr1:73657580-74032392
chr1	73907566	73910196	chr1:73657580-74032392
chr1	73910276	73910352	chr1:73657580-74032392
chr1	73910421	73911381	chr1:73657580-74032392
chr1	73911391	73912551	chr1:73657580-74032392
chr1	73912556	73912778	chr1:73657580-74032392
chr1	73913291	73913407	chr1:73657580-74032392
chr1	73913511	73913672	chr1:73657580-74032392
chr1	73913726	73913794	chr1:73657580-74032392
chr1	73914036	73914123	chr1:73657580-74032392
chr1	73914186	73914257	chr1:73657580-74032392
chr1	73914331	73914455	chr1:73657580-74032392
chr1	73914561	73914815	chr1:73657580-74032392
chr1	73914831	73914911	chr1:73657580-74032392

chr1	73914916	73915002	chr1:73657580-74032392
chr1	73915151	73915466	chr1:73657580-74032392
chr1	73916556	73917163	chr1:73657580-74032392
chr1	73917166	73917863	chr1:73657580-74032392
chr1	73917866	73918200	chr1:73657580-74032392
chr1	73918206	73921386	chr1:73657580-74032392
chr1	73921401	73921709	chr1:73657580-74032392
chr1	73921756	73922198	chr1:73657580-74032392
chr1	73922206	73924092	chr1:73657580-74032392
chr1	73924106	73924353	chr1:73657580-74032392
chr1	73924366	73925987	chr1:73657580-74032392
chr1	73925991	73926890	chr1:73657580-74032392
chr1	73926896	73928825	chr1:73657580-74032392
chr1	73928891	73930281	chr1:73657580-74032392
chr1	73930331	73931399	chr1:73657580-74032392
chr1	73931401	73932635	chr1:73657580-74032392
chr1	73932641	73934282	chr1:73657580-74032392
chr1	73934371	73934587	chr1:73657580-74032392
chr1	73934596	73934723	chr1:73657580-74032392
chr1	73934726	73936033	chr1:73657580-74032392
chr1	73936041	73936118	chr1:73657580-74032392
chr1	73937581	73938043	chr1:73657580-74032392
chr1	73938046	73938215	chr1:73657580-74032392
chr1	73938216	73938463	chr1:73657580-74032392
chr1	73938576	73938663	chr1:73657580-74032392
chr1	73938796	73940235	chr1:73657580-74032392
chr1	73940276	73940872	chr1:73657580-74032392
chr1	73940891	73942657	chr1:73657580-74032392
chr1	73942661	73943124	chr1:73657580-74032392
chr1	73943131	73943637	chr1:73657580-74032392
chr1	73943681	73943815	chr1:73657580-74032392
chr1	73943831	73944021	chr1:73657580-74032392
chr1	73944051	73944898	chr1:73657580-74032392
chr1	73944906	73945075	chr1:73657580-74032392
chr1	73945091	73945521	chr1:73657580-74032392
chr1	73945526	73946438	chr1:73657580-74032392
chr1	73946736	73950850	chr1:73657580-74032392
chr1	73951056	73951290	chr1:73657580-74032392
chr1	73951326	73951690	chr1:73657580-74032392
chr1	73951691	73951803	chr1:73657580-74032392
chr1	73957226	73957306	chr1:73657580-74032392
chr1	73957596	73957745	chr1:73657580-74032392
chr1	73957961	73958455	chr1:73657580-74032392
chr1	73958511	73960154	chr1:73657580-74032392
chr1	73960156	73960670	chr1:73657580-74032392
chr1	73960681	73961062	chr1:73657580-74032392
chr1	73961376	73961696	chr1:73657580-74032392
chr1	73961706	73963325	chr1:73657580-74032392
chr1	73963331	73964338	chr1:73657580-74032392
chr1	73964341	73968700	chr1:73657580-74032392
chr1	73968726	73971658	chr1:73657580-74032392
chr1	73971686	73972735	chr1:73657580-74032392
chr1	73972736	73972822	chr1:73657580-74032392
chr1	73972906	73973500	chr1:73657580-74032392
chr1	73973501	73974592	chr1:73657580-74032392
chr1	73974611	73974814	chr1:73657580-74032392
chr1	73974816	73975002	chr1:73657580-74032392
chr1	73975006	73975407	chr1:73657580-74032392
chr1	73975461	73975583	chr1:73657580-74032392
chr1	73975856	73976531	chr1:73657580-74032392
chr1	73976536	73976823	chr1:73657580-74032392
chr1	73976831	73977051	chr1:73657580-74032392
chr1	73977056	73979519	chr1:73657580-74032392

chr1	73979536	73980556	chr1:73657580-74032392
chr1	73980566	73982495	chr1:73657580-74032392
chr1	73982501	73986030	chr1:73657580-74032392
chr1	73986031	73986262	chr1:73657580-74032392
chr1	73986316	73987237	chr1:73657580-74032392
chr1	73987256	73987719	chr1:73657580-74032392
chr1	73987726	73989274	chr1:73657580-74032392
chr1	73989276	73990981	chr1:73657580-74032392
chr1	73990996	73991257	chr1:73657580-74032392
chr1	73991271	73991369	chr1:73657580-74032392
chr1	73991371	73991781	chr1:73657580-74032392
chr1	73991871	73992175	chr1:73657580-74032392
chr1	73992176	73992352	chr1:73657580-74032392
chr1	73992396	73992959	chr1:73657580-74032392
chr1	73992966	73993407	chr1:73657580-74032392
chr1	73993416	73993587	chr1:73657580-74032392
chr1	73993591	73997419	chr1:73657580-74032392
chr1	73997526	73997762	chr1:73657580-74032392
chr1	73997871	73997975	chr1:73657580-74032392
chr1	73998511	73998628	chr1:73657580-74032392
chr1	73998661	73998747	chr1:73657580-74032392
chr1	73998771	73998845	chr1:73657580-74032392
chr1	73998866	73998948	chr1:73657580-74032392
chr1	73999151	73999248	chr1:73657580-74032392
chr1	73999341	73999454	chr1:73657580-74032392
chr1	73999561	73999643	chr1:73657580-74032392
chr1	73999816	73999910	chr1:73657580-74032392
chr1	73999971	74000136	chr1:73657580-74032392
chr1	74000191	74000395	chr1:73657580-74032392
chr1	74000421	74000534	chr1:73657580-74032392
chr1	74000541	74000793	chr1:73657580-74032392
chr1	74000851	74001093	chr1:73657580-74032392
chr1	74001226	74001301	chr1:73657580-74032392
chr1	74001341	74001421	chr1:73657580-74032392
chr1	74001501	74001784	chr1:73657580-74032392
chr1	74002121	74002333	chr1:73657580-74032392
chr1	74002341	74002516	chr1:73657580-74032392
chr1	74002561	74003546	chr1:73657580-74032392
chr1	74003831	74004495	chr1:73657580-74032392
chr1	74004531	74004926	chr1:73657580-74032392
chr1	74004931	74005897	chr1:73657580-74032392
chr1	74006176	74006601	chr1:73657580-74032392
chr1	74006626	74007611	chr1:73657580-74032392
chr1	74007901	74009572	chr1:73657580-74032392
chr1	74009576	74009660	chr1:73657580-74032392
chr1	74009661	74011602	chr1:73657580-74032392
chr1	74011611	74012294	chr1:73657580-74032392
chr1	74012336	74012610	chr1:73657580-74032392
chr1	74012626	74013889	chr1:73657580-74032392
chr1	74013916	74014575	chr1:73657580-74032392
chr1	74014576	74014671	chr1:73657580-74032392
chr1	74014676	74014754	chr1:73657580-74032392
chr1	74015046	74015128	chr1:73657580-74032392
chr1	74015281	74015358	chr1:73657580-74032392
chr1	74015526	74015610	chr1:73657580-74032392
chr1	74015866	74016033	chr1:73657580-74032392
chr1	74016036	74016117	chr1:73657580-74032392
chr1	74016121	74016203	chr1:73657580-74032392
chr1	74016551	74016697	chr1:73657580-74032392
chr1	74016761	74016877	chr1:73657580-74032392
chr1	74016886	74016958	chr1:73657580-74032392
chr1	74016966	74017530	chr1:73657580-74032392
chr1	74017571	74017982	chr1:73657580-74032392

chr1	74018001	74018399	chr1:73657580-74032392
chr1	74018401	74018623	chr1:73657580-74032392
chr1	74018626	74018807	chr1:73657580-74032392
chr1	74018876	74018979	chr1:73657580-74032392
chr1	74019151	74022812	chr1:73657580-74032392
chr1	74022816	74023132	chr1:73657580-74032392
chr1	74023626	74023701	chr1:73657580-74032392
chr1	74023746	74023820	chr1:73657580-74032392
chr1	74024536	74024644	chr1:73657580-74032392
chr1	74024711	74024791	chr1:73657580-74032392
chr1	74024926	74024995	chr1:73657580-74032392
chr1	74025186	74025271	chr1:73657580-74032392
chr1	74025396	74026997	chr1:73657580-74032392
chr1	74027121	74027226	chr1:73657580-74032392
chr1	74027251	74027416	chr1:73657580-74032392
chr1	74027421	74027673	chr1:73657580-74032392
chr1	74027726	74027807	chr1:73657580-74032392
chr1	74027826	74027928	chr1:73657580-74032392
chr1	74027941	74028056	chr1:73657580-74032392
chr1	74028136	74028275	chr1:73657580-74032392
chr1	74029246	74030453	chr1:73657580-74032392
chr1	74030471	74030546	chr1:73657580-74032392
chr1	74030556	74030734	chr1:73657580-74032392
chr1	74030746	74031378	chr1:73657580-74032392
chr1	74031401	74031764	chr1:73657580-74032392
chr1	74031841	74032369	chr1:73657580-74032392
chr1	79176653	79176787	chr1:79176528-79253016
chr1	79176788	79176934	chr1:79176528-79253016
chr1	79176943	79177197	chr1:79176528-79253016
chr1	79177233	79178925	chr1:79176528-79253016
chr1	79178928	79180027	chr1:79176528-79253016
chr1	79180038	79182669	chr1:79176528-79253016
chr1	79182928	79183328	chr1:79176528-79253016
chr1	79183418	79183709	chr1:79176528-79253016
chr1	79183738	79183856	chr1:79176528-79253016
chr1	79183858	79185437	chr1:79176528-79253016
chr1	79185448	79186360	chr1:79176528-79253016
chr1	79186368	79186822	chr1:79176528-79253016
chr1	79186828	79187577	chr1:79176528-79253016
chr1	79187578	79190092	chr1:79176528-79253016
chr1	79190128	79190321	chr1:79176528-79253016
chr1	79190403	79190566	chr1:79176528-79253016
chr1	79190593	79191107	chr1:79176528-79253016
chr1	79191373	79192844	chr1:79176528-79253016
chr1	79192848	79195003	chr1:79176528-79253016
chr1	79195013	79195752	chr1:79176528-79253016
chr1	79195753	79195912	chr1:79176528-79253016
chr1	79195913	79196694	chr1:79176528-79253016
chr1	79196863	79197122	chr1:79176528-79253016
chr1	79197138	79199950	chr1:79176528-79253016
chr1	79199968	79200233	chr1:79176528-79253016
chr1	79200513	79200731	chr1:79176528-79253016
chr1	79200748	79200905	chr1:79176528-79253016
chr1	79200933	79201307	chr1:79176528-79253016
chr1	79201313	79201589	chr1:79176528-79253016
chr1	79201648	79201769	chr1:79176528-79253016
chr1	79201783	79201949	chr1:79176528-79253016
chr1	79201978	79204000	chr1:79176528-79253016
chr1	79204493	79204636	chr1:79176528-79253016
chr1	79205318	79205401	chr1:79176528-79253016
chr1	79205738	79205875	chr1:79176528-79253016
chr1	79206593	79207027	chr1:79176528-79253016
chr1	79207268	79207413	chr1:79176528-79253016

chr1	79207568	79207870	chr1:79176528-79253016
chr1	79207878	79208674	chr1:79176528-79253016
chr1	79208678	79210895	chr1:79176528-79253016
chr1	79210898	79211727	chr1:79176528-79253016
chr1	79211793	79211942	chr1:79176528-79253016
chr1	79211948	79213183	chr1:79176528-79253016
chr1	79213263	79217394	chr1:79176528-79253016
chr1	79217398	79217941	chr1:79176528-79253016
chr1	79217948	79218327	chr1:79176528-79253016
chr1	79218373	79218616	chr1:79176528-79253016
chr1	79218623	79219162	chr1:79176528-79253016
chr1	79219343	79219439	chr1:79176528-79253016
chr1	79219958	79220036	chr1:79176528-79253016
chr1	79220133	79220208	chr1:79176528-79253016
chr1	79220288	79220363	chr1:79176528-79253016
chr1	79220623	79220772	chr1:79176528-79253016
chr1	79220833	79220907	chr1:79176528-79253016
chr1	79221178	79221253	chr1:79176528-79253016
chr1	79221478	79222236	chr1:79176528-79253016
chr1	79222243	79222772	chr1:79176528-79253016
chr1	79222823	79223622	chr1:79176528-79253016
chr1	79223668	79224059	chr1:79176528-79253016
chr1	79224068	79228429	chr1:79176528-79253016
chr1	79228718	79230543	chr1:79176528-79253016
chr1	79230713	79231133	chr1:79176528-79253016
chr1	79231198	79231270	chr1:79176528-79253016
chr1	79231308	79231391	chr1:79176528-79253016
chr1	79231393	79231540	chr1:79176528-79253016
chr1	79231758	79231836	chr1:79176528-79253016
chr1	79231888	79232084	chr1:79176528-79253016
chr1	79232228	79232340	chr1:79176528-79253016
chr1	79232413	79232607	chr1:79176528-79253016
chr1	79232648	79234833	chr1:79176528-79253016
chr1	79234868	79234979	chr1:79176528-79253016
chr1	79235028	79235233	chr1:79176528-79253016
chr1	79235503	79235948	chr1:79176528-79253016
chr1	79235953	79236449	chr1:79176528-79253016
chr1	79236458	79236574	chr1:79176528-79253016
chr1	79236578	79236722	chr1:79176528-79253016
chr1	79237468	79239034	chr1:79176528-79253016
chr1	79239038	79239458	chr1:79176528-79253016
chr1	79239473	79239947	chr1:79176528-79253016
chr1	79239948	79240356	chr1:79176528-79253016
chr1	79240418	79240625	chr1:79176528-79253016
chr1	79240658	79241127	chr1:79176528-79253016
chr1	79241138	79242011	chr1:79176528-79253016
chr1	79242013	79243437	chr1:79176528-79253016
chr1	79243473	79246176	chr1:79176528-79253016
chr1	79246183	79246334	chr1:79176528-79253016
chr1	79246338	79246752	chr1:79176528-79253016
chr1	79247008	79250403	chr1:79176528-79253016
chr1	79250408	79251288	chr1:79176528-79253016
chr1	79251368	79251480	chr1:79176528-79253016
chr1	79251593	79252369	chr1:79176528-79253016
chr1	79252378	79252750	chr1:79176528-79253016
chr1	80176383	80177517	chr1:80176414-80390413
chr1	80177808	80178055	chr1:80176414-80390413
chr1	80178063	80178590	chr1:80176414-80390413
chr1	80178888	80179592	chr1:80176414-80390413
chr1	80179598	80181792	chr1:80176414-80390413
chr1	80181798	80183217	chr1:80176414-80390413
chr1	80183223	80183828	chr1:80176414-80390413
chr1	80183983	80184061	chr1:80176414-80390413

chr1	80184293	80184469	chr1:80176414-80390413
chr1	80184543	80184761	chr1:80176414-80390413
chr1	80184843	80184920	chr1:80176414-80390413
chr1	80185018	80185195	chr1:80176414-80390413
chr1	80185518	80185593	chr1:80176414-80390413
chr1	80185763	80185842	chr1:80176414-80390413
chr1	80185968	80186047	chr1:80176414-80390413
chr1	80186083	80186160	chr1:80176414-80390413
chr1	80186168	80186284	chr1:80176414-80390413
chr1	80186288	80186833	chr1:80176414-80390413
chr1	80186858	80187000	chr1:80176414-80390413
chr1	80187023	80188036	chr1:80176414-80390413
chr1	80188048	80188417	chr1:80176414-80390413
chr1	80188533	80188632	chr1:80176414-80390413
chr1	80188633	80188750	chr1:80176414-80390413
chr1	80188978	80189110	chr1:80176414-80390413
chr1	80189113	80189469	chr1:80176414-80390413
chr1	80189523	80189617	chr1:80176414-80390413
chr1	80189683	80190654	chr1:80176414-80390413
chr1	80190658	80191856	chr1:80176414-80390413
chr1	80191878	80192697	chr1:80176414-80390413
chr1	80192723	80194901	chr1:80176414-80390413
chr1	80194908	80196867	chr1:80176414-80390413
chr1	80196868	80197857	chr1:80176414-80390413
chr1	80197893	80197979	chr1:80176414-80390413
chr1	80198083	80198229	chr1:80176414-80390413
chr1	80198238	80200988	chr1:80176414-80390413
chr1	80201413	80201695	chr1:80176414-80390413
chr1	80201928	80203186	chr1:80176414-80390413
chr1	80203198	80205687	chr1:80176414-80390413
chr1	80205693	80206267	chr1:80176414-80390413
chr1	80206268	80208190	chr1:80176414-80390413
chr1	80208193	80209654	chr1:80176414-80390413
chr1	80209658	80210508	chr1:80176414-80390413
chr1	80210513	80210889	chr1:80176414-80390413
chr1	80210893	80210988	chr1:80176414-80390413
chr1	80210993	80211103	chr1:80176414-80390413
chr1	80211123	80211639	chr1:80176414-80390413
chr1	80211713	80211930	chr1:80176414-80390413
chr1	80211943	80212167	chr1:80176414-80390413
chr1	80212178	80212572	chr1:80176414-80390413
chr1	80212578	80213436	chr1:80176414-80390413
chr1	80213438	80213520	chr1:80176414-80390413
chr1	80213543	80213722	chr1:80176414-80390413
chr1	80213793	80214048	chr1:80176414-80390413
chr1	80214053	80214198	chr1:80176414-80390413
chr1	80214458	80214654	chr1:80176414-80390413
chr1	80214658	80215241	chr1:80176414-80390413
chr1	80215268	80215376	chr1:80176414-80390413
chr1	80215443	80216748	chr1:80176414-80390413
chr1	80217038	80217439	chr1:80176414-80390413
chr1	80217723	80218781	chr1:80176414-80390413
chr1	80218833	80219622	chr1:80176414-80390413
chr1	80219633	80220032	chr1:80176414-80390413
chr1	80220253	80220409	chr1:80176414-80390413
chr1	80220428	80221238	chr1:80176414-80390413
chr1	80221248	80221364	chr1:80176414-80390413
chr1	80221653	80222170	chr1:80176414-80390413
chr1	80222173	80222461	chr1:80176414-80390413
chr1	80222468	80222777	chr1:80176414-80390413
chr1	80222778	80223031	chr1:80176414-80390413
chr1	80223353	80225043	chr1:80176414-80390413
chr1	80225113	80225212	chr1:80176414-80390413

chr1	80225213	80225410	chr1:80176414-80390413
chr1	80225418	80225571	chr1:80176414-80390413
chr1	80225648	80225852	chr1:80176414-80390413
chr1	80225853	80227784	chr1:80176414-80390413
chr1	80227788	80228059	chr1:80176414-80390413
chr1	80228078	80228207	chr1:80176414-80390413
chr1	80228228	80228938	chr1:80176414-80390413
chr1	80228958	80230247	chr1:80176414-80390413
chr1	80230508	80231220	chr1:80176414-80390413
chr1	80231228	80231463	chr1:80176414-80390413
chr1	80231468	80232283	chr1:80176414-80390413
chr1	80232348	80233996	chr1:80176414-80390413
chr1	80234003	80235365	chr1:80176414-80390413
chr1	80235468	80236695	chr1:80176414-80390413
chr1	80236738	80236903	chr1:80176414-80390413
chr1	80236938	80237330	chr1:80176414-80390413
chr1	80237623	80238331	chr1:80176414-80390413
chr1	80238333	80238577	chr1:80176414-80390413
chr1	80238578	80239564	chr1:80176414-80390413
chr1	80239583	80240193	chr1:80176414-80390413
chr1	80240313	80240392	chr1:80176414-80390413
chr1	80240403	80240497	chr1:80176414-80390413
chr1	80240543	80240627	chr1:80176414-80390413
chr1	80240643	80242169	chr1:80176414-80390413
chr1	80242203	80245352	chr1:80176414-80390413
chr1	80245633	80246057	chr1:80176414-80390413
chr1	80246408	80246609	chr1:80176414-80390413
chr1	80246913	80247320	chr1:80176414-80390413
chr1	80247358	80247933	chr1:80176414-80390413
chr1	80247948	80250290	chr1:80176414-80390413
chr1	80250303	80252044	chr1:80176414-80390413
chr1	80252048	80252896	chr1:80176414-80390413
chr1	80252898	80252976	chr1:80176414-80390413
chr1	80253258	80253394	chr1:80176414-80390413
chr1	80253413	80253910	chr1:80176414-80390413
chr1	80253913	80254092	chr1:80176414-80390413
chr1	80254208	80254290	chr1:80176414-80390413
chr1	80254383	80259402	chr1:80176414-80390413
chr1	80259413	80259558	chr1:80176414-80390413
chr1	80259663	80259871	chr1:80176414-80390413
chr1	80259878	80259956	chr1:80176414-80390413
chr1	80260098	80260193	chr1:80176414-80390413
chr1	80260198	80260307	chr1:80176414-80390413
chr1	80260318	80260578	chr1:80176414-80390413
chr1	80260628	80260741	chr1:80176414-80390413
chr1	80260763	80261132	chr1:80176414-80390413
chr1	80261153	80261531	chr1:80176414-80390413
chr1	80261688	80261785	chr1:80176414-80390413
chr1	80261843	80261926	chr1:80176414-80390413
chr1	80262153	80263787	chr1:80176414-80390413
chr1	80263793	80263977	chr1:80176414-80390413
chr1	80263983	80268048	chr1:80176414-80390413
chr1	80268328	80268850	chr1:80176414-80390413
chr1	80268853	80272425	chr1:80176414-80390413
chr1	80272463	80272579	chr1:80176414-80390413
chr1	80272583	80272884	chr1:80176414-80390413
chr1	80272953	80273039	chr1:80176414-80390413
chr1	80273368	80273445	chr1:80176414-80390413
chr1	80273558	80273657	chr1:80176414-80390413
chr1	80274178	80274345	chr1:80176414-80390413
chr1	80274363	80274494	chr1:80176414-80390413
chr1	80274528	80279373	chr1:80176414-80390413
chr1	80279383	80280273	chr1:80176414-80390413

chr1	80280288	80280417	chr1:80176414-80390413
chr1	80280418	80282364	chr1:80176414-80390413
chr1	80282368	80283949	chr1:80176414-80390413
chr1	80283953	80286343	chr1:80176414-80390413
chr1	80286348	80286973	chr1:80176414-80390413
chr1	80287243	80287357	chr1:80176414-80390413
chr1	80287393	80289976	chr1:80176414-80390413
chr1	80289978	80290950	chr1:80176414-80390413
chr1	80290958	80291659	chr1:80176414-80390413
chr1	80291663	80291766	chr1:80176414-80390413
chr1	80291803	80294509	chr1:80176414-80390413
chr1	80294528	80294858	chr1:80176414-80390413
chr1	80294868	80294948	chr1:80176414-80390413
chr1	80294953	80295104	chr1:80176414-80390413
chr1	80295118	80295262	chr1:80176414-80390413
chr1	80295263	80295604	chr1:80176414-80390413
chr1	80295608	80296761	chr1:80176414-80390413
chr1	80296763	80298543	chr1:80176414-80390413
chr1	80298588	80299429	chr1:80176414-80390413
chr1	80299443	80300702	chr1:80176414-80390413
chr1	80300703	80300855	chr1:80176414-80390413
chr1	80300893	80301541	chr1:80176414-80390413
chr1	80301668	80304846	chr1:80176414-80390413
chr1	80304848	80307396	chr1:80176414-80390413
chr1	80307398	80307630	chr1:80176414-80390413
chr1	80307638	80308912	chr1:80176414-80390413
chr1	80308928	80311685	chr1:80176414-80390413
chr1	80311688	80311923	chr1:80176414-80390413
chr1	80311973	80312821	chr1:80176414-80390413
chr1	80313038	80314024	chr1:80176414-80390413
chr1	80314148	80314598	chr1:80176414-80390413
chr1	80314618	80315968	chr1:80176414-80390413
chr1	80316023	80317139	chr1:80176414-80390413
chr1	80317423	80317749	chr1:80176414-80390413
chr1	80318083	80319649	chr1:80176414-80390413
chr1	80319653	80320366	chr1:80176414-80390413
chr1	80320368	80322001	chr1:80176414-80390413
chr1	80322023	80322408	chr1:80176414-80390413
chr1	80322413	80322878	chr1:80176414-80390413
chr1	80322918	80323074	chr1:80176414-80390413
chr1	80323388	80323713	chr1:80176414-80390413
chr1	80323733	80323811	chr1:80176414-80390413
chr1	80323818	80324108	chr1:80176414-80390413
chr1	80324113	80325923	chr1:80176414-80390413
chr1	80325928	80326273	chr1:80176414-80390413
chr1	80326328	80327057	chr1:80176414-80390413
chr1	80327063	80327649	chr1:80176414-80390413
chr1	80327683	80328386	chr1:80176414-80390413
chr1	80328688	80329734	chr1:80176414-80390413
chr1	80329748	80329962	chr1:80176414-80390413
chr1	80330243	80330933	chr1:80176414-80390413
chr1	80331223	80332025	chr1:80176414-80390413
chr1	80332043	80332121	chr1:80176414-80390413
chr1	80332133	80332793	chr1:80176414-80390413
chr1	80334763	80334838	chr1:80176414-80390413
chr1	80336393	80338316	chr1:80176414-80390413
chr1	80338438	80338519	chr1:80176414-80390413
chr1	80338658	80339297	chr1:80176414-80390413
chr1	80339333	80339549	chr1:80176414-80390413
chr1	80339553	80340116	chr1:80176414-80390413
chr1	80340123	80340476	chr1:80176414-80390413
chr1	80340483	80340609	chr1:80176414-80390413
chr1	80340663	80343184	chr1:80176414-80390413

chr1	80343198	80343566	chr1:80176414-80390413
chr1	80343573	80343647	chr1:80176414-80390413
chr1	80343788	80343868	chr1:80176414-80390413
chr1	80343918	80344128	chr1:80176414-80390413
chr1	80344453	80344526	chr1:80176414-80390413
chr1	80344603	80344758	chr1:80176414-80390413
chr1	80344813	80344932	chr1:80176414-80390413
chr1	80344963	80345111	chr1:80176414-80390413
chr1	80345163	80345295	chr1:80176414-80390413
chr1	80345358	80345440	chr1:80176414-80390413
chr1	80345443	80345520	chr1:80176414-80390413
chr1	80345603	80345720	chr1:80176414-80390413
chr1	80345723	80345843	chr1:80176414-80390413
chr1	80345888	80346006	chr1:80176414-80390413
chr1	80346068	80346154	chr1:80176414-80390413
chr1	80346253	80346337	chr1:80176414-80390413
chr1	80346493	80346565	chr1:80176414-80390413
chr1	80346568	80346641	chr1:80176414-80390413
chr1	80346848	80346947	chr1:80176414-80390413
chr1	80347103	80347167	chr1:80176414-80390413
chr1	80347213	80347288	chr1:80176414-80390413
chr1	80347638	80347743	chr1:80176414-80390413
chr1	80347958	80348037	chr1:80176414-80390413
chr1	80348088	80348194	chr1:80176414-80390413
chr1	80348323	80348960	chr1:80176414-80390413
chr1	80349473	80349556	chr1:80176414-80390413
chr1	80349868	80349945	chr1:80176414-80390413
chr1	80350028	80350110	chr1:80176414-80390413
chr1	80350338	80350426	chr1:80176414-80390413
chr1	80350948	80351038	chr1:80176414-80390413
chr1	80351183	80352723	chr1:80176414-80390413
chr1	80352728	80353314	chr1:80176414-80390413
chr1	80353323	80353545	chr1:80176414-80390413
chr1	80353548	80354294	chr1:80176414-80390413
chr1	80359848	80359938	chr1:80176414-80390413
chr1	80359998	80360079	chr1:80176414-80390413
chr1	80360403	80362557	chr1:80176414-80390413
chr1	80362568	80363764	chr1:80176414-80390413
chr1	80363783	80364499	chr1:80176414-80390413
chr1	80364503	80364896	chr1:80176414-80390413
chr1	80364903	80365975	chr1:80176414-80390413
chr1	80365983	80370327	chr1:80176414-80390413
chr1	80370363	80370457	chr1:80176414-80390413
chr1	80370928	80371002	chr1:80176414-80390413
chr1	80371413	80371484	chr1:80176414-80390413
chr1	80371518	80371706	chr1:80176414-80390413
chr1	80372203	80372290	chr1:80176414-80390413
chr1	80372928	80375297	chr1:80176414-80390413
chr1	80375313	80376081	chr1:80176414-80390413
chr1	80376083	80376733	chr1:80176414-80390413
chr1	80376803	80377367	chr1:80176414-80390413
chr1	80377378	80378055	chr1:80176414-80390413
chr1	80378353	80379994	chr1:80176414-80390413
chr1	80379998	80381446	chr1:80176414-80390413
chr1	80381448	80381729	chr1:80176414-80390413
chr1	80381798	80383317	chr1:80176414-80390413
chr1	80383598	80384064	chr1:80176414-80390413
chr1	80384068	80384536	chr1:80176414-80390413
chr1	80384538	80384797	chr1:80176414-80390413
chr1	80384798	80385030	chr1:80176414-80390413
chr1	80385323	80388132	chr1:80176414-80390413
chr1	80388138	80388505	chr1:80176414-80390413
chr1	80388518	80388700	chr1:80176414-80390413

chr1	80388873	80388954	chr1:80176414-80390413
chr1	80388983	80389057	chr1:80176414-80390413
chr1	80389218	80389373	chr1:80176414-80390413
chr1	80389433	80389511	chr1:80176414-80390413
chr1	80389668	80389837	chr1:80176414-80390413
chr1	80389968	80390042	chr1:80176414-80390413
chr1	98453422	98453532	chr1:98453454-98455125
chr1	98453577	98453813	chr1:98453454-98455125
chr1	98453817	98454670	chr1:98453454-98455125
chr1	98454672	98454991	chr1:98453454-98455125
chr1	98460447	98460589	chr1:98460471-98460556
chr1	98509822	98510692	chr1:98509851-98510810
chr1	98510697	98510830	chr1:98509851-98510810
chr1	98511532	98511991	chr1:98511563-98511952
chr1	98512042	98512223	chr1:98512074-98512183
chr1	98513822	98514601	chr1:98513845-98564736
chr1	98514667	98517139	chr1:98513845-98564736
chr1	98517147	98518664	chr1:98513845-98564736
chr1	98518742	98520204	chr1:98513845-98564736
chr1	98520252	98520510	chr1:98513845-98564736
chr1	98520517	98521560	chr1:98513845-98564736
chr1	98521577	98527895	chr1:98513845-98564736
chr1	98528172	98528603	chr1:98513845-98564736
chr1	98528622	98529600	chr1:98513845-98564736
chr1	98529912	98530028	chr1:98513845-98564736
chr1	98530032	98530462	chr1:98513845-98564736
chr1	98530477	98530579	chr1:98513845-98564736
chr1	98530597	98531188	chr1:98513845-98564736
chr1	98531207	98531418	chr1:98513845-98564736
chr1	98531422	98532689	chr1:98513845-98564736
chr1	98532692	98532832	chr1:98513845-98564736
chr1	98533097	98534301	chr1:98513845-98564736
chr1	98534302	98534804	chr1:98513845-98564736
chr1	98534832	98535513	chr1:98513845-98564736
chr1	98535517	98537048	chr1:98513845-98564736
chr1	98537052	98542048	chr1:98513845-98564736
chr1	98542077	98543961	chr1:98513845-98564736
chr1	98544072	98546158	chr1:98513845-98564736
chr1	98546162	98546415	chr1:98513845-98564736
chr1	98546477	98546549	chr1:98513845-98564736
chr1	98546557	98546641	chr1:98513845-98564736
chr1	98546782	98548047	chr1:98513845-98564736
chr1	98548082	98548206	chr1:98513845-98564736
chr1	98548232	98548895	chr1:98513845-98564736
chr1	98548907	98549687	chr1:98513845-98564736
chr1	98549707	98551732	chr1:98513845-98564736
chr1	98552257	98552371	chr1:98513845-98564736
chr1	98552392	98555441	chr1:98513845-98564736
chr1	98555762	98557052	chr1:98513845-98564736
chr1	98557077	98557209	chr1:98513845-98564736
chr1	98557212	98557479	chr1:98513845-98564736
chr1	98557752	98558889	chr1:98513845-98564736
chr1	98559282	98560098	chr1:98513845-98564736
chr1	98560102	98561782	chr1:98513845-98564736
chr1	98562337	98562987	chr1:98513845-98564736
chr1	98562997	98563490	chr1:98513845-98564736
chr1	98563502	98563619	chr1:98513845-98564736
chr1	98563622	98564767	chr1:98513845-98564736
chr1	98736127	98736946	chr1:98736150-98738286
chr1	98737322	98737391	chr1:98736150-98738286
chr1	98737447	98737528	chr1:98736150-98738286
chr1	98737562	98737630	chr1:98736150-98738286
chr1	98737742	98737812	chr1:98736150-98738286

chr1	98738047	98738125	chr1:98736150-98738286
chr1	101777397	101777504	chr1:101777418-101777483
chr1	101788862	101789044	chr1:101788886-101789022
chr1	101789357	101789473	chr1:101789392-101789431
chr1	101803797	101803871	chr1:101803793-101803854
chr1	101809327	101809589	chr1:101809348-101809551
chr1	101811347	101811429	chr1:101811318-101811401
chr1	101815577	101815765	chr1:101815600-101815741
chr1	101842792	101842901	chr1:101842813-101842869
chr1	101855307	101856005	chr1:101855225-101855979
chr1	101877402	101877511	chr1:101877290-101898311
chr1	101878102	101878308	chr1:101877290-101898311
chr1	101878462	101878594	chr1:101877290-101898311
chr1	101879017	101879135	chr1:101877290-101898311
chr1	101879662	101879774	chr1:101877290-101898311
chr1	101880042	101880133	chr1:101877290-101898311
chr1	101880637	101880735	chr1:101877290-101898311
chr1	101880947	101881031	chr1:101877290-101898311
chr1	101881072	101881145	chr1:101877290-101898311
chr1	101881437	101881520	chr1:101877290-101898311
chr1	101882117	101882223	chr1:101877290-101898311
chr1	101882267	101882369	chr1:101877290-101898311
chr1	101882532	101882638	chr1:101877290-101898311
chr1	101882782	101882865	chr1:101877290-101898311
chr1	101882867	101883257	chr1:101877290-101898311
chr1	101883292	101884344	chr1:101877290-101898311
chr1	101884427	101884773	chr1:101877290-101898311
chr1	101885042	101886400	chr1:101877290-101898311
chr1	101886402	101886500	chr1:101877290-101898311
chr1	101886502	101887485	chr1:101877290-101898311
chr1	101887497	101888469	chr1:101877290-101898311
chr1	101889087	101889209	chr1:101877290-101898311
chr1	101889267	101889400	chr1:101877290-101898311
chr1	101889492	101889585	chr1:101877290-101898311
chr1	101889682	101889764	chr1:101877290-101898311
chr1	101891402	101891473	chr1:101877290-101898311
chr1	101891767	101891838	chr1:101877290-101898311
chr1	101892692	101892825	chr1:101877290-101898311
chr1	101892902	101893049	chr1:101877290-101898311
chr1	101893152	101893249	chr1:101877290-101898311
chr1	101893382	101893463	chr1:101877290-101898311
chr1	101893482	101893559	chr1:101877290-101898311
chr1	101893957	101894034	chr1:101877290-101898311
chr1	101894127	101895521	chr1:101877290-101898311
chr1	101895762	101895859	chr1:101877290-101898311
chr1	101895872	101896262	chr1:101877290-101898311
chr1	101896452	101896946	chr1:101877290-101898311
chr1	101896977	101898348	chr1:101877290-101898311
chr1	115642104	115642385	chr1:115642139-115642372
chr1	115644839	115645051	chr1:115644868-115645279
chr1	115645064	115645302	chr1:115644868-115645279
chr1	115675314	115675425	chr1:115675354-115675383
chr1	115676964	115678210	chr1:115676999-115680366
chr1	115678214	115678942	chr1:115676999-115680366
chr1	115678954	115679747	chr1:115676999-115680366
chr1	115679774	115680399	chr1:115676999-115680366
chr1	115722706	115722885	chr1:115722735-115722847
chr1	115723951	115724334	chr1:115723972-115724294
chr1	115764386	115764616	chr1:115764418-115765741
chr1	115764916	115765751	chr1:115764418-115765741
chr1	155204215	155204933	chr1:155204242-155204891
chr1	155204960	155205134	chr1:155204985-155205102
chr1	155205450	155205656	chr1:155205471-155205635

chr1	155206000	155206295	chr1:155206035-155206260
chr1	155207100	155207394	chr1:155207131-155207369
chr1	155207890	155208113	chr1:155207924-155208097
chr1	155208285	155208470	chr1:155208307-155208441
chr1	155209375	155209593	chr1:155209406-155209553
chr1	155209655	155209907	chr1:155209676-155209868
chr1	155210385	155210541	chr1:155210420-155210508
chr1	155210855	155211101	chr1:155210876-155211066
chr1	155213850	155213920	chr1:155213885-155214024
chr1	155214265	155214512	chr1:155214296-155214490
chr1	177264981	177265081	chr1:177264922-177316726
chr1	177265106	177265216	chr1:177264922-177316726
chr1	177265591	177265663	chr1:177264922-177316726
chr1	177265781	177265857	chr1:177264922-177316726
chr1	177266251	177266326	chr1:177264922-177316726
chr1	177266336	177266465	chr1:177264922-177316726
chr1	177266466	177266568	chr1:177264922-177316726
chr1	177267361	177267441	chr1:177264922-177316726
chr1	177267596	177268045	chr1:177264922-177316726
chr1	177268061	177270672	chr1:177264922-177316726
chr1	177270681	177272305	chr1:177264922-177316726
chr1	177272306	177272524	chr1:177264922-177316726
chr1	177272531	177274590	chr1:177264922-177316726
chr1	177274591	177275846	chr1:177264922-177316726
chr1	177275861	177276546	chr1:177264922-177316726
chr1	177276556	177277926	chr1:177264922-177316726
chr1	177277936	177278890	chr1:177264922-177316726
chr1	177278941	177283324	chr1:177264922-177316726
chr1	177283326	177283929	chr1:177264922-177316726
chr1	177283936	177285139	chr1:177264922-177316726
chr1	177285241	177286686	chr1:177264922-177316726
chr1	177286696	177286775	chr1:177264922-177316726
chr1	177286801	177287085	chr1:177264922-177316726
chr1	177287336	177288346	chr1:177264922-177316726
chr1	177288361	177288544	chr1:177264922-177316726
chr1	177289181	177289408	chr1:177264922-177316726
chr1	177289441	177289585	chr1:177264922-177316726
chr1	177289661	177289770	chr1:177264922-177316726
chr1	177289826	177289903	chr1:177264922-177316726
chr1	177290131	177290222	chr1:177264922-177316726
chr1	177290256	177290332	chr1:177264922-177316726
chr1	177290631	177290711	chr1:177264922-177316726
chr1	177290726	177290801	chr1:177264922-177316726
chr1	177290991	177291074	chr1:177264922-177316726
chr1	177291646	177291777	chr1:177264922-177316726
chr1	177291811	177291945	chr1:177264922-177316726
chr1	177291956	177292035	chr1:177264922-177316726
chr1	177292281	177292484	chr1:177264922-177316726
chr1	177292551	177292678	chr1:177264922-177316726
chr1	177292696	177292880	chr1:177264922-177316726
chr1	177292931	177293109	chr1:177264922-177316726
chr1	177293476	177293616	chr1:177264922-177316726
chr1	177293626	177293773	chr1:177264922-177316726
chr1	177294021	177294094	chr1:177264922-177316726
chr1	177294266	177294359	chr1:177264922-177316726
chr1	177294381	177294469	chr1:177264922-177316726
chr1	177294546	177294617	chr1:177264922-177316726
chr1	177295356	177295739	chr1:177264922-177316726
chr1	177295826	177297314	chr1:177264922-177316726
chr1	177297326	177298598	chr1:177264922-177316726
chr1	177298631	177299238	chr1:177264922-177316726
chr1	177299241	177299564	chr1:177264922-177316726
chr1	177299586	177299725	chr1:177264922-177316726

chr1	177299736	177299904	chr1:177264922-177316726
chr1	177299906	177301562	chr1:177264922-177316726
chr1	177301566	177303265	chr1:177264922-177316726
chr1	177303276	177305053	chr1:177264922-177316726
chr1	177305071	177307155	chr1:177264922-177316726
chr1	177307496	177308373	chr1:177264922-177316726
chr1	177308671	177309357	chr1:177264922-177316726
chr1	177309366	177310714	chr1:177264922-177316726
chr1	177310721	177311570	chr1:177264922-177316726
chr1	177311571	177311883	chr1:177264922-177316726
chr1	177311886	177312817	chr1:177264922-177316726
chr1	177312821	177313740	chr1:177264922-177316726
chr1	177313741	177316764	chr1:177264922-177316726
chr1	177320586	177320809	chr1:177320621-177320786
chr1	177325951	177326231	chr1:177325982-177326201
chr1	177332536	177332713	chr1:177332569-177332677
chr1	177335271	177335553	chr1:177335304-177335549
chr1	177362246	177362948	chr1:177362281-177362924
chr1	177406486	177406622	chr1:177406510-177406587
chr1	177418301	177418419	chr1:177418338-177418368
chr1	177442166	177442247	chr1:177442173-177442191
chr1	177448706	177448877	chr1:177448727-177448861
chr1	177525611	177525715	chr1:177525620-177525691
chr1	177542831	177542940	chr1:177542862-177542919
chr1	177545096	177545209	chr1:177545120-177545163
chr1	177545811	177546098	chr1:177545845-177546085
chr1	177547616	177547763	chr1:177547647-177547723
chr1	177566681	177566856	chr1:177566705-177566844
chr1	177570481	177570586	chr1:177570521-177570559
chr1	177589466	177589572	chr1:177589499-177589551
chr1	177599546	177599644	chr1:177599567-177599627
chr1	177618326	177618424	chr1:177618347-177618413
chr1	177713821	177714057	chr1:177713843-177714036
chr1	205737083	205737653	chr1:205737113-205739581
chr1	205737993	205738561	chr1:205737113-205739581
chr1	205738848	205739616	chr1:205737113-205739581
chr1	205739833	205740017	chr1:205739860-205739982
chr1	205740573	205740817	chr1:205740599-205740781
chr1	205741598	205741733	chr1:205741623-205741695
chr1	205742043	205742455	chr1:205742064-205742563
chr1	205742458	205742586	chr1:205742064-205742563
chr1	205743938	205744804	chr1:205743960-205744588;chr1:205744629-205744782
chr1	205751838	205752708	chr1:205751860-205752673
chr1	205758193	205759470	chr1:205758220-205760846
chr1	205759473	205760246	chr1:205758220-205760846
chr1	205760273	205760880	chr1:205758220-205760846
chr1	205763963	205764173	chr1:205763997-205764146
chr1	205764448	205764635	chr1:205764471-205764606
chr1	205766023	205766172	chr1:205766051-205766131
chr1	205767003	205767218	chr1:205767031-205767179
chr1	205767768	205767988	chr1:205767796-205767943
chr1	205768053	205768269	chr1:205768084-205768229
chr1	205768863	205768998	chr1:205768886-205768958
chr1	205770053	205770225	chr1:205770080-205770188
chr1	205770413	205770629	chr1:205770448-205770616
chr1	205773983	205774527	chr1:205774004-205774503
chr1	205779163	205780230	chr1:205779197-205780215
chr1	205781913	205782327	chr1:205781935-205782304
chr1	205782793	205782897	chr1:205782822-205782876
chr1	209690599	209690719	chr1:209690632-209690698
chr1	209697419	209697503	chr1:209697447-209717109
chr1	209697839	209698058	chr1:209697447-209717109
chr1	209698329	209698442	chr1:209697447-209717109

chr1	209698459	209700486	chr1:209697447-209717109
chr1	209700489	209701620	chr1:209697447-209717109
chr1	209701624	209702652	chr1:209697447-209717109
chr1	209702814	209702960	chr1:209697447-209717109
chr1	209702964	209703764	chr1:209697447-209717109
chr1	209704039	209707547	chr1:209697447-209717109
chr1	209707584	209711686	chr1:209697447-209717109
chr1	209711699	209716750	chr1:209697447-209717109
chr1	209716764	209717152	chr1:209697447-209717109
chr1	209719334	209719500	chr1:209719357-209719484
chr1	209720804	209720951	chr1:209720836-209720925
chr1	209740289	209740362	chr1:209740254-209740334
chr1	209740854	209741178	chr1:209740879-209741144
chr1	209742864	209742966	chr1:209742892-209742935
chr1	232533675	232534203	chr1:232533710-232535019
chr1	232534205	232535053	chr1:232533710-232535019
chr1	232538115	232538251	chr1:232538137-232538219
chr1	232539165	232539349	chr1:232539193-232539317
chr1	232539835	232539947	chr1:232539870-232539924
chr1	232551215	232551386	chr1:232551239-232551371
chr1	232561310	232561576	chr1:232561334-232561554
chr1	232564125	232564350	chr1:232564156-232564310
chr1	232567970	232568258	chr1:232567992-232568217
chr1	232574820	232575285	chr1:232574853-232575242
chr1	232577005	232577153	chr1:232577036-232577140
chr1	232577480	232577630	chr1:232577513-232577614
chr1	232579315	232579473	chr1:232579347-232579431
chr1	232581250	232581560	chr1:232581274-232581532
chr1	232586130	232586681	chr1:232586159-232586658
chr1	232596600	232596929	chr1:232596632-232596907
chr1	232597915	232598196	chr1:232597938-232598163
chr1	232600550	232601182	chr1:232600585-232601162
chr1	232607090	232607302	chr1:232607116-232607274
chr1	232615345	232615487	chr1:232615372-232615476
chr1	232619515	232619735	chr1:232619537-232619712
chr1	232626410	232626831	chr1:232626431-232626808
chr1	232629245	232629421	chr1:232629272-232629406
chr1	232649580	232651388	chr1:232649602-232651354
chr1	232697190	232697333	chr1:232697214-232697304
chr1	243419275	243419563	chr1:243419306-243419542
chr1	243432405	243432522	chr1:243432437-243432502
chr1	243433360	243433595	chr1:243433393-243433559
chr1	243434245	243434387	chr1:243434279-243434365
chr1	243437835	243437973	chr1:243437844-243437958
chr1	243449550	243449722	chr1:243449573-243449699
chr1	243456345	243456557	chr1:243456370-243456521
chr1	243456665	243456812	chr1:243456700-243456796
chr1	243467990	243468334	chr1:243468014-243469850
chr1	243468620	243469015	chr1:243468014-243469850
chr1	243469020	243469141	chr1:243468014-243469850
chr1	243469455	243469545	chr1:243468014-243469850
chr1	243469600	243469833	chr1:243468014-243469850
chr1	243471050	243471227	chr1:243471085-243471223
chr1	243471265	243471511	chr1:243471290-243471479
chr1	243479880	243480227	chr1:243479902-243480197
chr1	243481320	243481398	chr1:243481338-243481353
chr1	243493815	243494027	chr1:243493841-243493994
chr1	243502020	243502271	chr1:243502054-243502242
chr1	243504315	243504486	chr1:243504340-243504475
chr1	243507485	243507675	chr1:243507516-243507633
chr1	243541825	243542210	chr1:243541847-243542165
chr1	243551310	243551628	chr1:243551295-243551617
chr1	243578975	243579154	chr1:243579003-243579131

chr1	243581235	243581413	chr1:243581269-243581378
chr1	243589705	243589885	chr1:243589728-243589860
chr1	243626750	243627311	chr1:243626773-243627272
chr1	243635260	243635383	chr1:243635291-243635353
chr1	243639200	243639707	chr1:243639232-243639666
chr1	243646080	243646474	chr1:243646113-243646229;chr1:243646257-243646447
chr1	243651060	243651208	chr1:243651093-243651180
chr1	243651510	243651793	chr1:243651534-243651752
chr1	243652280	243652820	chr1:243652315-243652782
chr1	243662885	243663042	chr1:243662918-243663394
chr1	243663050	243663425	chr1:243662918-243663394
chr1	243664050	243667025	chr1:243664074-243668636
chr1	243667030	243667934	chr1:243664074-243668636
chr1	243667950	243668662	chr1:243664074-243668636
chr1	243675600	243675739	chr1:243675625-243675728
chr1	243680155	243680467	chr1:243679873-243680406;chr1:243680407-243680420
chr1	243688025	243688225	chr1:243688049-243688197
chr1	243706696	243706824	chr1:243706740-243706768;chr1:243706769-243706796
chr1	243708786	243708953	chr1:243708811-243708923
chr1	243709911	243710294	chr1:243709920-243710269
chr1	243711286	243711639	chr1:243711319-243711631
chr1	243716006	243716279	chr1:243716030-243716245
chr1	243726986	243727172	chr1:243727021-243727150
chr1	243736221	243736386	chr1:243736227-243736350
chr1	243738046	243738192	chr1:243738073-243738156
chr1	243775806	243776079	chr1:243775784-243776336
chr1	243776951	243777051	chr1:243776972-243777041
chr1	243778376	243778474	chr1:243778397-243778463
chr1	243800891	243801056	chr1:243800912-243801044
chr1	243809161	243809377	chr1:243809194-243809339
chr1	243828051	243828200	chr1:243828073-243828185
chr1	243850706	243850920	chr1:243850738-243851174
chr1	243850921	243851090	chr1:243850738-243851174
chr1	243851091	243851198	chr1:243850738-243851174
chr1	243858871	243859041	chr1:243858892-243859018
chr1	243866126	243866371	chr1:243866158-243866347
chr1	243878796	243879357	chr1:243878817-243879316
chr1	243898276	243898410	chr1:243898259-243898418
chr1	243898886	243899085	chr1:243898908-243899049
chr1	243902791	243902947	chr1:243902823-243902915
chr1	243903886	243903968	chr1:243903910-243903934
chr1	243903981	243904245	chr1:243904014-243904281
chr1	243905031	243905134	chr1:243905069-243905109
chr1	243905236	243905356	chr1:243905259-243905345
chr1	243952031	243952127	chr1:243952003-243952128
chr1	243956471	243956554	chr1:243956506-243956882
chr1	243956666	243956806	chr1:243956506-243956882
chr1	243957626	243957727	chr1:243957561-243957702
chr1	243977776	243978067	chr1:243977800-243978032
chr1	243981326	243981512	chr1:243981350-243981476
chr1	244006401	244006614	chr1:244006426-244006584
chr1	244006821	244006962	chr1:244006845-244006975
chr1	244007431	244007541	chr1:244007466-244007512
chr1	244013316	244013441	chr1:244013341-244013430
chr1	244014316	244014424	chr1:244014350-244014381
chr2	43054847	43055613	chr2:43054870-43056048
chr2	43055657	43056074	chr2:43054870-43056048
chr2	43160807	43160921	chr2:43160840-43160892
chr2	43180197	43180382	chr2:43180229-43180342
chr2	43254902	43255361	chr2:43254932-43255324
chr2	43256022	43256875	chr2:43256052-43257116
chr2	43258252	43258458	chr2:43258273-43258440
chr2	43259547	43259801	chr2:43259580-43259775

chr2	43259842	43260062	chr2:43259872-43260041
chr2	43260332	43260666	chr2:43260366-43260665
chr2	43263802	43263911	chr2:43263834-43263904
chr2	43265637	43265775	chr2:43265661-43265738
chr2	43266227	43266869	chr2:43266258-43266847
chr2	43268307	43268792	chr2:43268331-43268782
chr2	43270772	43271018	chr2:43270803-43271012
chr2	43314377	43315882	chr2:43314412-43325570
chr2	43315887	43316467	chr2:43314412-43325570
chr2	43316747	43317258	chr2:43314412-43325570
chr2	43317547	43317834	chr2:43314412-43325570
chr2	43318157	43318498	chr2:43314412-43325570
chr2	43318592	43319036	chr2:43314412-43325570
chr2	43319332	43320657	chr2:43314412-43325570
chr2	43320952	43322341	chr2:43314412-43325570
chr2	43322632	43322719	chr2:43314412-43325570
chr2	43323062	43323136	chr2:43314412-43325570
chr2	43323147	43323254	chr2:43314412-43325570
chr2	43323307	43323819	chr2:43314412-43325570
chr2	43323827	43323947	chr2:43314412-43325570
chr2	43324232	43324549	chr2:43314412-43325570
chr2	43324552	43325606	chr2:43314412-43325570
chr2	43327422	43327571	chr2:43327457-43327536
chr2	43329137	43330506	chr2:43329172-43330480
chr2	43355292	43356148	chr2:43355324-43361560
chr2	43356382	43357157	chr2:43355324-43361560
chr2	43357167	43360447	chr2:43355324-43361560
chr2	43360467	43361587	chr2:43355324-43361560
chr2	43364722	43364837	chr2:43364751-43364819
chr2	43369972	43370217	chr2:43370000-43370236
chr2	43384582	43384732	chr2:43384611-43384706
chr2	43401112	43401287	chr2:43401091-43401259
chr2	43402697	43402842	chr2:43402720-43402811
chr2	43411592	43411740	chr2:43411627-43411709
chr2	55502863	55502940	chr2:55502898-55507333
chr2	55503178	55503370	chr2:55502898-55507333
chr2	55503663	55504176	chr2:55502898-55507333
chr2	55504473	55504928	chr2:55502898-55507333
chr2	55505198	55505610	chr2:55502898-55507333
chr2	55505613	55505867	chr2:55502898-55507333
chr2	55505868	55505944	chr2:55502898-55507333
chr2	55506178	55506414	chr2:55502898-55507333
chr2	55535603	55535907	chr2:55535627-55535894
chr2	55540933	55541546	chr2:55540964-55541797
chr2	55556533	55556887	chr2:55556557-55556849
chr2	55566813	55566954	chr2:55566834-55567446
chr2	55567238	55567474	chr2:55566834-55567446
chr2	55572823	55573216	chr2:55572492-55573185
chr2	57541265	57541582	chr2:57541244-57541655
chr2	57542575	57542710	chr2:57542598-57542674
chr2	57607155	57607300	chr2:57607187-57607265
chr2	57892860	57892983	chr2:57892888-57892986
chr2	57896470	57896574	chr2:57896503-57896556
chr2	57923005	57923314	chr2:57923027-57923277
chr2	57926225	57926375	chr2:57926256-57926333
chr2	57928080	57928958	chr2:57928102-57928926
chr2	57929760	57930134	chr2:57929795-57939866
chr2	57930180	57931116	chr2:57929795-57939866
chr2	57931120	57931517	chr2:57929795-57939866
chr2	57931785	57934649	chr2:57929795-57939866
chr2	57934670	57934919	chr2:57929795-57939866
chr2	57935200	57936112	chr2:57929795-57939866
chr2	57936145	57939752	chr2:57929795-57939866

chr2	57939755	57939903	chr2:57929795-57939866
chr2	57940075	57940356	chr2:57940100-57940316
chr2	57943315	57943560	chr2:57943342-57943520
chr2	57947825	57948146	chr2:57947848-57948111
chr2	57949945	57950131	chr2:57949990-57950009;chr2:57950062-57950087
chr2	57952805	57953359	chr2:57952829-57953337
chr2	63277157	63277297	chr2:63277191-63277287
chr2	63277912	63278050	chr2:63277936-63278032
chr2	63278352	63278463	chr2:63278383-63278452
chr2	63279982	63280232	chr2:63280014-63280222
chr2	63281037	63281355	chr2:63281066-63281333
chr2	63282602	63283271	chr2:63282635-63284966
chr2	63283282	63284270	chr2:63282635-63284966
chr2	63284307	63284623	chr2:63282635-63284966
chr2	63284667	63284983	chr2:63282635-63284966
chr2	67792493	67792802	chr2:67792515-67792776
chr2	67884098	67884181	chr2:67884129-67884168
chr2	67911053	67911215	chr2:67911083-67911209
chr2	68023153	68023762	chr2:68023185-68023730
chr2	68036698	68037125	chr2:68036723-68070896
chr2	68037168	68037247	chr2:68036723-68070896
chr2	68037688	68037761	chr2:68036723-68070896
chr2	68038078	68038163	chr2:68036723-68070896
chr2	68038893	68039022	chr2:68036723-68070896
chr2	68039923	68039998	chr2:68036723-68070896
chr2	68041163	68041328	chr2:68036723-68070896
chr2	68041333	68041498	chr2:68036723-68070896
chr2	68041503	68042386	chr2:68036723-68070896
chr2	68042808	68042882	chr2:68036723-68070896
chr2	68042893	68044004	chr2:68036723-68070896
chr2	68044008	68044139	chr2:68036723-68070896
chr2	68044143	68045285	chr2:68036723-68070896
chr2	68045293	68045404	chr2:68036723-68070896
chr2	68045413	68046123	chr2:68036723-68070896
chr2	68046128	68046907	chr2:68036723-68070896
chr2	68046958	68047303	chr2:68036723-68070896
chr2	68047333	68047429	chr2:68036723-68070896
chr2	68047433	68048545	chr2:68036723-68070896
chr2	68048548	68050274	chr2:68036723-68070896
chr2	68050593	68053008	chr2:68036723-68070896
chr2	68053033	68053216	chr2:68036723-68070896
chr2	68053223	68054443	chr2:68036723-68070896
chr2	68054483	68054599	chr2:68036723-68070896
chr2	68054938	68055561	chr2:68036723-68070896
chr2	68055563	68055751	chr2:68036723-68070896
chr2	68055753	68055867	chr2:68036723-68070896
chr2	68055888	68055962	chr2:68036723-68070896
chr2	68055968	68056501	chr2:68036723-68070896
chr2	68056508	68057605	chr2:68036723-68070896
chr2	68057953	68058533	chr2:68036723-68070896
chr2	68058968	68059049	chr2:68036723-68070896
chr2	68059328	68059671	chr2:68036723-68070896
chr2	68059673	68061525	chr2:68036723-68070896
chr2	68061753	68061901	chr2:68036723-68070896
chr2	68062178	68062274	chr2:68036723-68070896
chr2	68062278	68064670	chr2:68036723-68070896
chr2	68064728	68064954	chr2:68036723-68070896
chr2	68064958	68065073	chr2:68036723-68070896
chr2	68065078	68065598	chr2:68036723-68070896
chr2	68065878	68066220	chr2:68036723-68070896
chr2	68066223	68066338	chr2:68036723-68070896
chr2	68066403	68068364	chr2:68036723-68070896
chr2	68068463	68068561	chr2:68036723-68070896

chr2	68068863	68068965	chr2:68036723-68070896
chr2	68068978	68069143	chr2:68036723-68070896
chr2	68069148	68070054	chr2:68036723-68070896
chr2	68070063	68070383	chr2:68036723-68070896
chr2	68070393	68070921	chr2:68036723-68070896
chr2	68085118	68085294	chr2:68085151-68085263
chr2	68192538	68192660	chr2:68192568-68192613
chr2	68645843	68647254	chr2:68645870-68662388
chr2	68647258	68649621	chr2:68645870-68662388
chr2	68649913	68650369	chr2:68645870-68662388
chr2	68650493	68650568	chr2:68645870-68662388
chr2	68650743	68653265	chr2:68645870-68662388
chr2	68653513	68654474	chr2:68645870-68662388
chr2	68654483	68654986	chr2:68645870-68662388
chr2	68654988	68655928	chr2:68645870-68662388
chr2	68655943	68656754	chr2:68645870-68662388
chr2	68656898	68656982	chr2:68645870-68662388
chr2	68657013	68658300	chr2:68645870-68662388
chr2	68658533	68658919	chr2:68645870-68662388
chr2	68658923	68659284	chr2:68645870-68662388
chr2	68659363	68661409	chr2:68645870-68662388
chr2	68661518	68662434	chr2:68645870-68662388
chr2	73144578	73145520	chr2:73144603-73145501
chr2	73151408	73151659	chr2:73151437-73151622
chr2	73160883	73162055	chr2:73160915-73162020
chr2	109635196	109636792	chr2:109635221-109636789
chr2	109683931	109684040	chr2:109683955-109684013
chr2	109684856	109685294	chr2:109684887-109685262
chr2	109685981	109686187	chr2:109686011-109686625
chr2	109686191	109686644	chr2:109686011-109686625
chr2	109688721	109689825	chr2:109688749-109690800
chr2	109690156	109690563	chr2:109688749-109690800
chr2	127884572	127885102	chr2:127884594-127894758
chr2	127885127	127885444	chr2:127884594-127894758
chr2	127885452	127886952	chr2:127884594-127894758
chr2	127887237	127888050	chr2:127884594-127894758
chr2	127888072	127888292	chr2:127884594-127894758
chr2	127888347	127888976	chr2:127884594-127894758
chr2	127888997	127889788	chr2:127884594-127894758
chr2	127889812	127889995	chr2:127884594-127894758
chr2	127890047	127890182	chr2:127884594-127894758
chr2	127890452	127891262	chr2:127884594-127894758
chr2	127891572	127892212	chr2:127884594-127894758
chr2	127892247	127893346	chr2:127884594-127894758
chr2	127893347	127894798	chr2:127884594-127894758
chr2	129982148	129982838	chr2:129982173-130005694
chr2	129983143	129984519	chr2:129982173-130005694
chr2	129984538	129988167	chr2:129982173-130005694
chr2	129988453	129989128	chr2:129982173-130005694
chr2	129989388	129989789	chr2:129982173-130005694
chr2	129990183	129990256	chr2:129982173-130005694
chr2	129990513	129991222	chr2:129982173-130005694
chr2	129991268	129994683	chr2:129982173-130005694
chr2	129994693	129996396	chr2:129982173-130005694
chr2	129996703	130004391	chr2:129982173-130005694
chr2	130004443	130004660	chr2:129982173-130005694
chr2	130004663	130005095	chr2:129982173-130005694
chr2	130005103	130005732	chr2:129982173-130005694
chr2	130007413	130007669	chr2:130007438-130007647
chr2	130008418	130008556	chr2:130008442-130008539
chr2	130031148	130031451	chr2:130031179-130031421
chr2	130046498	130046770	chr2:130046522-130046751
chr2	130068483	130068725	chr2:130068516-130068692

chr2	130076118	130076217	chr2:130076145-130076188
chr2	130111968	130112067	chr2:130111997-130112031
chr2	135213300	135214394	chr2:135213329-135214347
chr2	135215580	135215773	chr2:135215602-135215744
chr2	135223660	135223833	chr2:135223684-135223796
chr2	135260440	135260594	chr2:135260471-135260568
chr2	135308115	135308256	chr2:135308140-135308232
chr2	135309590	135309667	chr2:135309618-135309662
chr2	135470735	135470898	chr2:135470769-135470889
chr2	135476280	135476591	chr2:135476303-135476571
chr2	135493010	135493184	chr2:135493033-135493151
chr2	135529795	135530242	chr2:135529817-135574435
chr2	135530275	135530478	chr2:135529817-135574435
chr2	135530480	135530664	chr2:135529817-135574435
chr2	135530670	135535012	chr2:135529817-135574435
chr2	135535295	135536472	chr2:135529817-135574435
chr2	135536475	135537111	chr2:135529817-135574435
chr2	135537130	135537734	chr2:135529817-135574435
chr2	135537745	135537980	chr2:135529817-135574435
chr2	135538265	135541111	chr2:135529817-135574435
chr2	135541145	135541222	chr2:135529817-135574435
chr2	135541630	135541797	chr2:135529817-135574435
chr2	135541805	135543159	chr2:135529817-135574435
chr2	135543165	135543941	chr2:135529817-135574435
chr2	135543965	135544123	chr2:135529817-135574435
chr2	135544150	135544953	chr2:135529817-135574435
chr2	135545230	135545980	chr2:135529817-135574435
chr2	135545985	135548105	chr2:135529817-135574435
chr2	135548115	135548457	chr2:135529817-135574435
chr2	135548460	135548536	chr2:135529817-135574435
chr2	135548680	135549111	chr2:135529817-135574435
chr2	135549135	135549240	chr2:135529817-135574435
chr2	135549285	135550431	chr2:135529817-135574435
chr2	135550450	135551264	chr2:135529817-135574435
chr2	135551355	135551435	chr2:135529817-135574435
chr2	135551575	135551768	chr2:135529817-135574435
chr2	135551780	135552313	chr2:135529817-135574435
chr2	135552470	135553077	chr2:135529817-135574435
chr2	135553340	135556427	chr2:135529817-135574435
chr2	135556485	135556744	chr2:135529817-135574435
chr2	135556745	135558056	chr2:135529817-135574435
chr2	135558065	135558787	chr2:135529817-135574435
chr2	135559370	135559596	chr2:135529817-135574435
chr2	135559865	135559967	chr2:135529817-135574435
chr2	135560065	135560549	chr2:135529817-135574435
chr2	135560570	135560756	chr2:135529817-135574435
chr2	135560760	135560949	chr2:135529817-135574435
chr2	135561095	135561169	chr2:135529817-135574435
chr2	135562110	135562188	chr2:135529817-135574435
chr2	135562520	135562597	chr2:135529817-135574435
chr2	135563000	135563154	chr2:135529817-135574435
chr2	135563190	135563265	chr2:135529817-135574435
chr2	135563275	135563404	chr2:135529817-135574435
chr2	135563640	135563728	chr2:135529817-135574435
chr2	135563870	135563943	chr2:135529817-135574435
chr2	135564105	135564182	chr2:135529817-135574435
chr2	135564255	135564344	chr2:135529817-135574435
chr2	135564535	135564615	chr2:135529817-135574435
chr2	135564710	135564826	chr2:135529817-135574435
chr2	135565040	135565160	chr2:135529817-135574435
chr2	135565185	135565287	chr2:135529817-135574435
chr2	135565330	135565401	chr2:135529817-135574435
chr2	135565460	135565538	chr2:135529817-135574435

chr2	135565560	135565633	chr2:135529817-135574435
chr2	135566080	135566177	chr2:135529817-135574435
chr2	135566455	135566529	chr2:135529817-135574435
chr2	135566770	135566863	chr2:135529817-135574435
chr2	135566865	135566946	chr2:135529817-135574435
chr2	135566950	135567026	chr2:135529817-135574435
chr2	135567105	135567194	chr2:135529817-135574435
chr2	135567210	135567291	chr2:135529817-135574435
chr2	135567490	135567681	chr2:135529817-135574435
chr2	135567740	135567818	chr2:135529817-135574435
chr2	135567915	135567990	chr2:135529817-135574435
chr2	135568060	135568177	chr2:135529817-135574435
chr2	135568340	135568425	chr2:135529817-135574435
chr2	135568460	135568573	chr2:135529817-135574435
chr2	135569405	135570007	chr2:135529817-135574435
chr2	135570690	135573019	chr2:135529817-135574435
chr2	135573280	135574466	chr2:135529817-135574435
chr2	135574550	135574833	chr2:135574573-135592245
chr2	135575145	135575253	chr2:135574573-135592245
chr2	135575590	135576288	chr2:135574573-135592245
chr2	135576620	135577600	chr2:135574573-135592245
chr2	135578555	135580244	chr2:135574573-135592245
chr2	135580500	135580855	chr2:135574573-135592245
chr2	135580930	135582434	chr2:135574573-135592245
chr2	135582725	135583031	chr2:135574573-135592245
chr2	135589020	135589460	chr2:135574573-135592245
chr2	135589490	135589982	chr2:135574573-135592245
chr2	135589985	135590070	chr2:135574573-135592245
chr2	135590250	135590365	chr2:135574573-135592245
chr2	135590380	135590666	chr2:135574573-135592245
chr2	135590970	135591147	chr2:135574573-135592245
chr2	135591175	135591344	chr2:135574573-135592245
chr2	135591345	135592021	chr2:135574573-135592245
chr2	135596085	135596335	chr2:135596116-135596309
chr2	135601910	135602083	chr2:135601934-135602041
chr2	135602765	135602885	chr2:135602802-135602847
chr2	135616805	135616946	chr2:135616830-135616927
chr2	135619510	135619616	chr2:135619538-135619588
chr2	135620800	135620912	chr2:135620837-135620859
chr2	135620940	135621215	chr2:135620964-135621201
chr2	135624605	135625368	chr2:135624631-135625098;chr2:135625148-135625242;chr2:135625292-135625309
chr2	135626480	135626627	chr2:135626513-135626611
chr2	135628510	135628644	chr2:135628534-135628630
chr2	135630015	135630223	chr2:135630038-135630211
chr2	135634965	135635257	chr2:135634986-135635213
chr2	135655885	135656017	chr2:135655910-135656009
chr2	135659360	135659634	chr2:135659367-135659604
chr2	135676105	135676310	chr2:135676132-135676283
chr2	136742710	136742952	chr2:136742745-136742922
chr2	136751380	136751584	chr2:136751401-136751555
chr2	136753740	136754015	chr2:136753761-136753978
chr2	136756235	136756857	chr2:136756256-136756843
chr2	136757225	136757963	chr2:136757217-136758049
chr2	136757975	136758077	chr2:136757217-136758049
chr2	136759545	136759723	chr2:136759570-136759696
chr2	136761290	136761425	chr2:136761312-136761392
chr2	136764740	136764993	chr2:136764765-136765327
chr2	136766425	136766498	chr2:136766436-136766643
chr2	136770005	136770232	chr2:136770034-136770552
chr2	136770235	136770580	chr2:136770034-136770552
chr2	136771585	136771736	chr2:136771618-136771704
chr2	136772190	136772367	chr2:136772225-136772332
chr2	136773815	136773960	chr2:136773836-136773916

chr2	136774005	136774919	chr2:136774027-136774882
chr2	136802430	136804246	chr2:136802456-136822267
chr2	136804400	136806061	chr2:136802456-136822267
chr2	136806355	136811089	chr2:136802456-136822267
chr2	136811385	136812751	chr2:136802456-136822267
chr2	136813180	136813827	chr2:136802456-136822267
chr2	136813885	136814435	chr2:136802456-136822267
chr2	136814440	136815467	chr2:136802456-136822267
chr2	136815470	136815634	chr2:136802456-136822267
chr2	136815635	136817027	chr2:136802456-136822267
chr2	136817030	136817264	chr2:136802456-136822267
chr2	136817540	136819288	chr2:136802456-136822267
chr2	136819315	136819474	chr2:136802456-136822267
chr2	136819625	136820577	chr2:136802456-136822267
chr2	136820860	136820944	chr2:136802456-136822267
chr2	136821035	136821431	chr2:136802456-136822267
chr2	136821465	136822305	chr2:136802456-136822267
chr2	136835440	136835587	chr2:136835461-136835564
chr2	136835695	136836117	chr2:136835717-136836083
chr2	136843295	136843598	chr2:136843288-136843869
chr2	136843600	136843882	chr2:136843288-136843869
chr2	136844440	136844831	chr2:136844471-136844794
chr2	136844940	136845043	chr2:136844876-136845078
chr2	136846105	136846275	chr2:136846038-136846255
chr2	136868380	136868733	chr2:136868409-136869424
chr2	136868775	136868963	chr2:136868409-136869424
chr2	136868965	136869475	chr2:136868409-136869424
chr2	145134566	145135469	chr2:145134588-145141541
chr2	145135471	145136385	chr2:145134588-145141541
chr2	145136386	145137221	chr2:145134588-145141541
chr2	145137226	145137695	chr2:145134588-145141541
chr2	145137696	145139975	chr2:145134588-145141541
chr2	145139976	145141559	chr2:145134588-145141541
chr2	145279376	145279515	chr2:145279405-145279493
chr2	145281641	145281751	chr2:145281682-145281712
chr2	145299896	145300005	chr2:145299930-145299960
chr2	145323976	145324149	chr2:145323997-145324137
chr2	145336751	145337028	chr2:145336775-145337001
chr2	145341236	145341548	chr2:145341259-145412271
chr2	145341561	145343408	chr2:145341259-145412271
chr2	145343436	145343834	chr2:145341259-145412271
chr2	145343841	145344847	chr2:145341259-145412271
chr2	145344861	145347252	chr2:145341259-145412271
chr2	145347561	145347942	chr2:145341259-145412271
chr2	145348241	145350135	chr2:145341259-145412271
chr2	145350141	145350690	chr2:145341259-145412271
chr2	145350711	145350839	chr2:145341259-145412271
chr2	145350846	145352918	chr2:145341259-145412271
chr2	145352921	145353138	chr2:145341259-145412271
chr2	145353141	145354303	chr2:145341259-145412271
chr2	145354311	145358095	chr2:145341259-145412271
chr2	145358346	145359353	chr2:145341259-145412271
chr2	145359641	145360034	chr2:145341259-145412271
chr2	145360066	145363515	chr2:145341259-145412271
chr2	145363521	145363610	chr2:145341259-145412271
chr2	145363611	145368340	chr2:145341259-145412271
chr2	145368346	145369366	chr2:145341259-145412271
chr2	145369651	145371108	chr2:145341259-145412271
chr2	145371126	145371934	chr2:145341259-145412271
chr2	145371956	145372070	chr2:145341259-145412271
chr2	145372091	145372178	chr2:145341259-145412271
chr2	145372491	145374303	chr2:145341259-145412271
chr2	145374321	145374625	chr2:145341259-145412271

chr2	145374701	145374777	chr2:145341259-145412271
chr2	145375071	145375169	chr2:145341259-145412271
chr2	145375181	145375267	chr2:145341259-145412271
chr2	145375691	145375799	chr2:145341259-145412271
chr2	145375866	145376610	chr2:145341259-145412271
chr2	145376616	145376816	chr2:145341259-145412271
chr2	145376821	145378639	chr2:145341259-145412271
chr2	145378651	145379304	chr2:145341259-145412271
chr2	145379951	145380020	chr2:145341259-145412271
chr2	145380186	145380256	chr2:145341259-145412271
chr2	145380866	145382370	chr2:145341259-145412271
chr2	145382371	145383068	chr2:145341259-145412271
chr2	145383076	145384348	chr2:145341259-145412271
chr2	145384351	145385850	chr2:145341259-145412271
chr2	145385861	145388355	chr2:145341259-145412271
chr2	145388366	145388764	chr2:145341259-145412271
chr2	145388786	145389089	chr2:145341259-145412271
chr2	145389096	145389330	chr2:145341259-145412271
chr2	145389511	145389634	chr2:145341259-145412271
chr2	145389651	145389750	chr2:145341259-145412271
chr2	145389761	145389841	chr2:145341259-145412271
chr2	145390021	145390134	chr2:145341259-145412271
chr2	145390176	145390410	chr2:145341259-145412271
chr2	145390411	145390563	chr2:145341259-145412271
chr2	145390671	145390817	chr2:145341259-145412271
chr2	145395306	145395384	chr2:145341259-145412271
chr2	145395726	145395844	chr2:145341259-145412271
chr2	145395941	145396032	chr2:145341259-145412271
chr2	145396046	145396121	chr2:145341259-145412271
chr2	145396431	145396584	chr2:145341259-145412271
chr2	145396691	145397257	chr2:145341259-145412271
chr2	145397311	145397478	chr2:145341259-145412271
chr2	145397751	145398585	chr2:145341259-145412271
chr2	145398586	145400787	chr2:145341259-145412271
chr2	145401056	145402119	chr2:145341259-145412271
chr2	145402121	145404601	chr2:145341259-145412271
chr2	145404956	145406542	chr2:145341259-145412271
chr2	145406581	145407187	chr2:145341259-145412271
chr2	145407191	145407304	chr2:145341259-145412271
chr2	145407311	145407418	chr2:145341259-145412271
chr2	145407491	145408353	chr2:145341259-145412271
chr2	145408451	145409480	chr2:145341259-145412271
chr2	145409481	145410274	chr2:145341259-145412271
chr2	145410281	145411202	chr2:145341259-145412271
chr2	145411206	145412263	chr2:145341259-145412271
chr2	145425501	145425700	chr2:145425533-145425675
chr2	145429141	145429225	chr2:145429168-145429231
chr2	145431021	145431206	chr2:145431053-145431172
chr2	145445946	145446653	chr2:145445979-145446642
chr2	145447591	145447774	chr2:145447616-145447752
chr2	145448571	145448719	chr2:145448605-145448677
chr2	145520936	145521114	chr2:145520963-145521095
chr2	145523911	145524120	chr2:145523936-145524097
chr2	145525631	145525977	chr2:145525664-145525950
chr2	145568656	145568874	chr2:145568691-145568842
chr2	145621501	145621606	chr2:145621511-145621565
chr2	145622936	145623041	chr2:145622961-145623027
chr2	145647481	145647810	chr2:145647513-145647781
chr2	145661206	145661396	chr2:145661232-145661370
chr2	145692901	145693081	chr2:145692923-145693050
chr2	145698401	145698536	chr2:145698424-145698512
chr2	145801911	145802043	chr2:145801932-145802010
chr2	145815236	145815317	chr2:145815217-145815295

chr2	145828361	145828531	chr2:145828386-145828508
chr2	145830061	145831039	chr2:145830090-145834340
chr2	145831326	145832472	chr2:145830090-145834340
chr2	145832476	145833566	chr2:145830090-145834340
chr2	145833576	145834080	chr2:145830090-145834340
chr2	145834091	145834338	chr2:145830090-145834340
chr2	145870766	145871040	chr2:145870787-145871010
chr2	145875101	145875246	chr2:145875122-145875220
chr2	145921706	145921897	chr2:145921739-145921879
chr2	145929541	145929707	chr2:145929564-145929698
chr2	145938626	145938694	chr2:145938576-145938679
chr2	145939586	145939693	chr2:145939612-145939660
chr2	145939756	145940084	chr2:145939770-145940216
chr2	145940091	145940225	chr2:145939770-145940216
chr2	146330751	146331003	chr2:146330776-146330965
chr2	146370286	146371059	chr2:146370319-146371028
chr2	146373981	146374120	chr2:146373973-146374101
chr2	146378406	146378550	chr2:146378438-146378530
chr2	146389226	146389442	chr2:146389257-146389421
chr2	146392021	146392238	chr2:146392054-146392198
chr2	146419276	146419678	chr2:146419303-146441828
chr2	146419681	146420520	chr2:146419303-146441828
chr2	146420801	146421226	chr2:146419303-146441828
chr2	146421266	146422040	chr2:146419303-146441828
chr2	146422041	146422332	chr2:146419303-146441828
chr2	146422341	146422505	chr2:146419303-146441828
chr2	146422516	146422585	chr2:146419303-146441828
chr2	146422586	146423169	chr2:146419303-146441828
chr2	146423176	146423702	chr2:146419303-146441828
chr2	146423741	146425150	chr2:146419303-146441828
chr2	146425156	146426080	chr2:146419303-146441828
chr2	146426096	146426766	chr2:146419303-146441828
chr2	146426776	146428402	chr2:146419303-146441828
chr2	146428406	146428548	chr2:146419303-146441828
chr2	146428561	146430667	chr2:146419303-146441828
chr2	146430671	146430933	chr2:146419303-146441828
chr2	146430946	146431053	chr2:146419303-146441828
chr2	146431061	146431176	chr2:146419303-146441828
chr2	146431181	146431268	chr2:146419303-146441828
chr2	146431291	146431389	chr2:146419303-146441828
chr2	146431396	146431495	chr2:146419303-146441828
chr2	146431506	146431736	chr2:146419303-146441828
chr2	146431751	146432163	chr2:146419303-146441828
chr2	146432166	146432429	chr2:146419303-146441828
chr2	146432431	146432554	chr2:146419303-146441828
chr2	146432586	146433098	chr2:146419303-146441828
chr2	146433101	146433614	chr2:146419303-146441828
chr2	146433621	146434340	chr2:146419303-146441828
chr2	146434636	146434851	chr2:146419303-146441828
chr2	146434886	146435172	chr2:146419303-146441828
chr2	146435176	146435889	chr2:146419303-146441828
chr2	146436136	146436215	chr2:146419303-146441828
chr2	146436246	146436346	chr2:146419303-146441828
chr2	146436616	146437017	chr2:146419303-146441828
chr2	146437031	146438223	chr2:146419303-146441828
chr2	146438231	146438679	chr2:146419303-146441828
chr2	146439041	146439397	chr2:146419303-146441828
chr2	146439406	146441484	chr2:146419303-146441828
chr2	146441531	146441656	chr2:146419303-146441828
chr2	146441696	146441867	chr2:146419303-146441828
chr2	146522236	146522456	chr2:146522270-146522414
chr2	168810505	168812117	chr2:168810529-168812080
chr2	168821145	168821258	chr2:168821171-168821236

chr2	168869115	168869231	chr2:168869143-168869212
chr2	168873530	168873629	chr2:168873552-168873605
chr2	168919980	168920116	chr2:168920009-168920080
chr2	168921795	168921921	chr2:168921828-168921891
chr2	168931470	168931543	chr2:168931491-168931521
chr2	168931590	168931758	chr2:168931618-168931741
chr2	168969405	168969653	chr2:168969427-168969616
chr2	168986015	168986127	chr2:168986050-168986116
chr2	168986195	168986294	chr2:168986219-168986268
chr2	168994565	168994775	chr2:168994597-168994731
chr2	168996770	168996912	chr2:168996798-168996900
chr2	168997150	168997276	chr2:168997158-168997268
chr2	169018275	169018370	chr2:169018296-169018352
chr2	169020225	169020428	chr2:169020248-169020390
chr2	169023785	169023959	chr2:169023808-169023917
chr2	169038460	169038647	chr2:169038487-169038600
chr2	169103715	169104685	chr2:169103737-169104651
chr2	169105000	169105206	chr2:169105025-169105172
chr2	169121580	169121664	chr2:169121605-169121809
chr2	169121665	169121842	chr2:169121605-169121809
chr2	169122840	169122984	chr2:169122874-169122946
chr2	169197600	169197911	chr2:169197622-169197886
chr2	169197940	169198681	chr2:169197973-169198663
chr2	169204800	169204919	chr2:169204831-169204893
chr2	185463068	185463815	chr2:185463092-185463797
chr2	185603503	185604068	chr2:185603534-185604033
chr2	185731073	185731283	chr2:185731095-185731239
chr2	185771348	185772083	chr2:185771370-185771869;chr2:185771896-185771940;chr2:185771951-185771970;chr2:185771971-185771987;chr2:185772010-185772024;chr2:185772030-185772042
chr2	185798308	185798485	chr2:185798329-185798460
chr2	185800483	185801912	chr2:185800509-185804219
chr2	185801918	185804246	chr2:185800509-185804219
chr2	188577509	188577619	chr2:188577542-188577606
chr2	188801884	188802716	chr2:188801907-188853104
chr2	188802744	188804953	chr2:188801907-188853104
chr2	188805084	188806144	chr2:188801907-188853104
chr2	188806149	188806315	chr2:188801907-188853104
chr2	188806319	188807276	chr2:188801907-188853104
chr2	188807324	188809169	chr2:188801907-188853104
chr2	188809459	188810096	chr2:188801907-188853104
chr2	188810139	188810859	chr2:188801907-188853104
chr2	188810904	188810994	chr2:188801907-188853104
chr2	188811054	188812614	chr2:188801907-188853104
chr2	188812624	188812745	chr2:188801907-188853104
chr2	188813049	188813199	chr2:188801907-188853104
chr2	188813204	188813592	chr2:188801907-188853104
chr2	188813594	188815058	chr2:188801907-188853104
chr2	188815654	188815730	chr2:188801907-188853104
chr2	188816439	188816573	chr2:188801907-188853104
chr2	188816589	188816670	chr2:188801907-188853104
chr2	188816704	188816818	chr2:188801907-188853104
chr2	188817949	188818020	chr2:188801907-188853104
chr2	188820864	188820980	chr2:188801907-188853104
chr2	188820984	188821238	chr2:188801907-188853104
chr2	188821244	188821401	chr2:188801907-188853104
chr2	188821519	188821614	chr2:188801907-188853104
chr2	188821739	188822780	chr2:188801907-188853104
chr2	188822814	188824528	chr2:188801907-188853104
chr2	188824569	188825330	chr2:188801907-188853104
chr2	188825344	188825955	chr2:188801907-188853104
chr2	188825959	188826509	chr2:188801907-188853104
chr2	188826544	188829734	chr2:188801907-188853104

chr2	188829994	188830212	chr2:188801907-188853104
chr2	188830339	188833569	chr2:188801907-188853104
chr2	188833574	188833677	chr2:188801907-188853104
chr2	188833884	188834891	chr2:188801907-188853104
chr2	188834894	188835139	chr2:188801907-188853104
chr2	188835274	188835348	chr2:188801907-188853104
chr2	188835964	188836042	chr2:188801907-188853104
chr2	188836344	188836422	chr2:188801907-188853104
chr2	188836759	188836859	chr2:188801907-188853104
chr2	188836934	188838185	chr2:188801907-188853104
chr2	188838489	188839900	chr2:188801907-188853104
chr2	188839914	188840939	chr2:188801907-188853104
chr2	188840944	188843509	chr2:188801907-188853104
chr2	188843524	188844820	chr2:188801907-188853104
chr2	188845089	188846311	chr2:188801907-188853104
chr2	188846399	188849222	chr2:188801907-188853104
chr2	188849224	188852800	chr2:188801907-188853104
chr2	189030519	189030654	chr2:189030480-189030635
chr2	193718784	193719009	chr2:193718808-193719099
chr2	193719054	193719133	chr2:193718808-193719099
chr2	193742494	193743945	chr2:193742529-193989223
chr2	193743994	193744138	chr2:193742529-193989223
chr2	193744149	193745758	chr2:193742529-193989223
chr2	193745774	193745911	chr2:193742529-193989223
chr2	193745949	193747495	chr2:193742529-193989223
chr2	193747499	193748238	chr2:193742529-193989223
chr2	193748244	193748704	chr2:193742529-193989223
chr2	193748714	193748884	chr2:193742529-193989223
chr2	193749169	193749596	chr2:193742529-193989223
chr2	193749604	193753291	chr2:193742529-193989223
chr2	193753304	193753704	chr2:193742529-193989223
chr2	193753734	193753913	chr2:193742529-193989223
chr2	193753949	193754031	chr2:193742529-193989223
chr2	193754049	193754182	chr2:193742529-193989223
chr2	193754184	193754587	chr2:193742529-193989223
chr2	193754869	193756561	chr2:193742529-193989223
chr2	193756564	193757594	chr2:193742529-193989223
chr2	193757604	193758195	chr2:193742529-193989223
chr2	193759694	193760853	chr2:193742529-193989223
chr2	193760909	193761441	chr2:193742529-193989223
chr2	193761444	193761620	chr2:193742529-193989223
chr2	193761664	193761994	chr2:193742529-193989223
chr2	193762309	193762601	chr2:193742529-193989223
chr2	193762629	193765463	chr2:193742529-193989223
chr2	193765704	193767497	chr2:193742529-193989223
chr2	193767534	193767683	chr2:193742529-193989223
chr2	193768009	193768993	chr2:193742529-193989223
chr2	193769049	193769123	chr2:193742529-193989223
chr2	193769129	193769201	chr2:193742529-193989223
chr2	193769734	193769807	chr2:193742529-193989223
chr2	193769864	193769953	chr2:193742529-193989223
chr2	193769959	193770142	chr2:193742529-193989223
chr2	193770169	193770662	chr2:193742529-193989223
chr2	193771214	193772948	chr2:193742529-193989223
chr2	193773259	193773944	chr2:193742529-193989223
chr2	193773949	193774147	chr2:193742529-193989223
chr2	193774364	193776085	chr2:193742529-193989223
chr2	193776094	193777103	chr2:193742529-193989223
chr2	193777134	193777326	chr2:193742529-193989223
chr2	193777339	193777896	chr2:193742529-193989223
chr2	193778029	193778106	chr2:193742529-193989223
chr2	193778109	193778186	chr2:193742529-193989223
chr2	193778284	193778514	chr2:193742529-193989223

chr2	193779034	193779349	chr2:193742529-193989223
chr2	193779399	193779776	chr2:193742529-193989223
chr2	193779869	193780104	chr2:193742529-193989223
chr2	193780184	193780392	chr2:193742529-193989223
chr2	193781389	193781481	chr2:193742529-193989223
chr2	193781524	193782353	chr2:193742529-193989223
chr2	193782629	193783042	chr2:193742529-193989223
chr2	193783229	193783295	chr2:193742529-193989223
chr2	193783814	193783991	chr2:193742529-193989223
chr2	193784524	193784670	chr2:193742529-193989223
chr2	193784864	193784947	chr2:193742529-193989223
chr2	193787214	193787299	chr2:193742529-193989223
chr2	193787374	193787496	chr2:193742529-193989223
chr2	193787589	193787679	chr2:193742529-193989223
chr2	193787744	193787842	chr2:193742529-193989223
chr2	193787844	193787985	chr2:193742529-193989223
chr2	193788019	193788102	chr2:193742529-193989223
chr2	193788154	193788254	chr2:193742529-193989223
chr2	193788324	193788397	chr2:193742529-193989223
chr2	193788449	193788607	chr2:193742529-193989223
chr2	193788859	193788933	chr2:193742529-193989223
chr2	193789549	193789866	chr2:193742529-193989223
chr2	193792829	193792905	chr2:193742529-193989223
chr2	193795179	193795257	chr2:193742529-193989223
chr2	193795809	193795919	chr2:193742529-193989223
chr2	193796004	193797254	chr2:193742529-193989223
chr2	193797259	193797462	chr2:193742529-193989223
chr2	193797524	193797710	chr2:193742529-193989223
chr2	193797774	193797937	chr2:193742529-193989223
chr2	193797974	193798108	chr2:193742529-193989223
chr2	193798149	193798382	chr2:193742529-193989223
chr2	193798634	193798786	chr2:193742529-193989223
chr2	193798909	193799026	chr2:193742529-193989223
chr2	193799074	193799170	chr2:193742529-193989223
chr2	193799174	193799340	chr2:193742529-193989223
chr2	193799389	193799569	chr2:193742529-193989223
chr2	193799669	193799748	chr2:193742529-193989223
chr2	193799829	193799996	chr2:193742529-193989223
chr2	193800044	193800194	chr2:193742529-193989223
chr2	193800199	193800433	chr2:193742529-193989223
chr2	193800434	193800992	chr2:193742529-193989223
chr2	193801009	193801448	chr2:193742529-193989223
chr2	193801574	193801687	chr2:193742529-193989223
chr2	193801799	193802320	chr2:193742529-193989223
chr2	193802374	193802968	chr2:193742529-193989223
chr2	193802969	193803755	chr2:193742529-193989223
chr2	193803764	193804046	chr2:193742529-193989223
chr2	193804054	193806318	chr2:193742529-193989223
chr2	193806339	193806836	chr2:193742529-193989223
chr2	193806844	193807032	chr2:193742529-193989223
chr2	193807034	193807141	chr2:193742529-193989223
chr2	193807154	193807297	chr2:193742529-193989223
chr2	193807309	193807911	chr2:193742529-193989223
chr2	193807914	193808799	chr2:193742529-193989223
chr2	193808814	193811910	chr2:193742529-193989223
chr2	193811929	193812615	chr2:193742529-193989223
chr2	193812624	193812703	chr2:193742529-193989223
chr2	193812709	193814053	chr2:193742529-193989223
chr2	193814054	193814205	chr2:193742529-193989223
chr2	193814239	193814757	chr2:193742529-193989223
chr2	193814759	193815366	chr2:193742529-193989223
chr2	193815369	193816200	chr2:193742529-193989223
chr2	193816369	193817467	chr2:193742529-193989223

chr2	193817534	193819686	chr2:193742529-193989223
chr2	193819984	193820241	chr2:193742529-193989223
chr2	193820259	193820683	chr2:193742529-193989223
chr2	193820939	193822087	chr2:193742529-193989223
chr2	193822109	193822474	chr2:193742529-193989223
chr2	193822759	193823162	chr2:193742529-193989223
chr2	193823214	193823308	chr2:193742529-193989223
chr2	193823719	193824586	chr2:193742529-193989223
chr2	193824619	193827164	chr2:193742529-193989223
chr2	193827169	193828380	chr2:193742529-193989223
chr2	193828389	193829207	chr2:193742529-193989223
chr2	193829209	193829363	chr2:193742529-193989223
chr2	193829659	193831830	chr2:193742529-193989223
chr2	193831859	193833103	chr2:193742529-193989223
chr2	193833114	193833350	chr2:193742529-193989223
chr2	193833354	193833692	chr2:193742529-193989223
chr2	193833699	193834457	chr2:193742529-193989223
chr2	193834464	193836445	chr2:193742529-193989223
chr2	193836454	193837514	chr2:193742529-193989223
chr2	193837529	193838538	chr2:193742529-193989223
chr2	193838569	193838653	chr2:193742529-193989223
chr2	193838739	193838826	chr2:193742529-193989223
chr2	193838854	193838958	chr2:193742529-193989223
chr2	193839299	193839498	chr2:193742529-193989223
chr2	193839789	193840951	chr2:193742529-193989223
chr2	193840954	193842149	chr2:193742529-193989223
chr2	193842174	193842514	chr2:193742529-193989223
chr2	193842804	193843057	chr2:193742529-193989223
chr2	193843064	193845645	chr2:193742529-193989223
chr2	193845934	193846155	chr2:193742529-193989223
chr2	193846159	193846312	chr2:193742529-193989223
chr2	193846579	193848606	chr2:193742529-193989223
chr2	193848849	193850923	chr2:193742529-193989223
chr2	193850924	193852554	chr2:193742529-193989223
chr2	193852584	193853140	chr2:193742529-193989223
chr2	193853149	193854120	chr2:193742529-193989223
chr2	193855534	193856219	chr2:193742529-193989223
chr2	193856244	193856398	chr2:193742529-193989223
chr2	193856439	193857017	chr2:193742529-193989223
chr2	193857029	193859331	chr2:193742529-193989223
chr2	193859334	193859746	chr2:193742529-193989223
chr2	193859749	193859866	chr2:193742529-193989223
chr2	193859869	193859983	chr2:193742529-193989223
chr2	193860259	193862420	chr2:193742529-193989223
chr2	193862424	193864490	chr2:193742529-193989223
chr2	193864494	193864870	chr2:193742529-193989223
chr2	193864889	193865963	chr2:193742529-193989223
chr2	193866374	193866506	chr2:193742529-193989223
chr2	193866509	193866988	chr2:193742529-193989223
chr2	193866994	193867181	chr2:193742529-193989223
chr2	193867184	193867910	chr2:193742529-193989223
chr2	193867989	193868486	chr2:193742529-193989223
chr2	193868754	193868881	chr2:193742529-193989223
chr2	193868884	193869141	chr2:193742529-193989223
chr2	193869144	193869223	chr2:193742529-193989223
chr2	193869229	193869829	chr2:193742529-193989223
chr2	193871644	193871977	chr2:193742529-193989223
chr2	193872059	193872139	chr2:193742529-193989223
chr2	193873414	193873481	chr2:193742529-193989223
chr2	193874219	193874434	chr2:193742529-193989223
chr2	193874444	193875399	chr2:193742529-193989223
chr2	193875444	193875774	chr2:193742529-193989223
chr2	193875819	193878040	chr2:193742529-193989223

chr2	193878054	193878201	chr2:193742529-193989223
chr2	193878204	193878622	chr2:193742529-193989223
chr2	193878634	193878846	chr2:193742529-193989223
chr2	193878854	193879539	chr2:193742529-193989223
chr2	193879599	193879812	chr2:193742529-193989223
chr2	193879824	193880251	chr2:193742529-193989223
chr2	193880389	193880509	chr2:193742529-193989223
chr2	193880604	193880708	chr2:193742529-193989223
chr2	193880754	193881120	chr2:193742529-193989223
chr2	193881124	193881214	chr2:193742529-193989223
chr2	193881254	193881442	chr2:193742529-193989223
chr2	193881489	193881570	chr2:193742529-193989223
chr2	193881574	193881733	chr2:193742529-193989223
chr2	193881874	193882139	chr2:193742529-193989223
chr2	193882324	193882548	chr2:193742529-193989223
chr2	193882569	193883001	chr2:193742529-193989223
chr2	193883014	193883467	chr2:193742529-193989223
chr2	193883484	193883557	chr2:193742529-193989223
chr2	193883579	193883808	chr2:193742529-193989223
chr2	193883839	193883997	chr2:193742529-193989223
chr2	193883999	193884070	chr2:193742529-193989223
chr2	193884104	193884204	chr2:193742529-193989223
chr2	193884209	193884290	chr2:193742529-193989223
chr2	193884339	193884493	chr2:193742529-193989223
chr2	193884494	193885538	chr2:193742529-193989223
chr2	193885834	193885910	chr2:193742529-193989223
chr2	193885919	193889212	chr2:193742529-193989223
chr2	193889239	193889402	chr2:193742529-193989223
chr2	193889464	193889535	chr2:193742529-193989223
chr2	193889569	193889668	chr2:193742529-193989223
chr2	193889744	193889839	chr2:193742529-193989223
chr2	193889844	193889940	chr2:193742529-193989223
chr2	193890409	193890515	chr2:193742529-193989223
chr2	193890629	193890706	chr2:193742529-193989223
chr2	193890764	193890830	chr2:193742529-193989223
chr2	193890984	193891062	chr2:193742529-193989223
chr2	193891129	193891528	chr2:193742529-193989223
chr2	193891544	193891619	chr2:193742529-193989223
chr2	193891629	193891796	chr2:193742529-193989223
chr2	193891859	193891982	chr2:193742529-193989223
chr2	193892144	193892245	chr2:193742529-193989223
chr2	193892274	193892387	chr2:193742529-193989223
chr2	193892389	193892671	chr2:193742529-193989223
chr2	193892679	193892755	chr2:193742529-193989223
chr2	193892809	193893039	chr2:193742529-193989223
chr2	193893049	193893180	chr2:193742529-193989223
chr2	193893214	193893399	chr2:193742529-193989223
chr2	193893419	193893531	chr2:193742529-193989223
chr2	193893704	193893901	chr2:193742529-193989223
chr2	193893909	193894009	chr2:193742529-193989223
chr2	193894114	193894251	chr2:193742529-193989223
chr2	193894279	193894446	chr2:193742529-193989223
chr2	193894629	193894801	chr2:193742529-193989223
chr2	193894869	193894974	chr2:193742529-193989223
chr2	193895004	193895090	chr2:193742529-193989223
chr2	193895189	193895479	chr2:193742529-193989223
chr2	193895654	193895797	chr2:193742529-193989223
chr2	193895854	193896002	chr2:193742529-193989223
chr2	193896019	193896169	chr2:193742529-193989223
chr2	193896194	193897252	chr2:193742529-193989223
chr2	193902714	193904367	chr2:193742529-193989223
chr2	193904809	193905928	chr2:193742529-193989223
chr2	193906014	193906299	chr2:193742529-193989223

chr2	193906324	193907010	chr2:193742529-193989223
chr2	193907019	193907795	chr2:193742529-193989223
chr2	193907809	193907999	chr2:193742529-193989223
chr2	193908034	193908113	chr2:193742529-193989223
chr2	193908224	193908968	chr2:193742529-193989223
chr2	193908999	193909224	chr2:193742529-193989223
chr2	193909494	193910656	chr2:193742529-193989223
chr2	193910659	193910858	chr2:193742529-193989223
chr2	193910864	193912515	chr2:193742529-193989223
chr2	193912519	193913071	chr2:193742529-193989223
chr2	193913074	193913294	chr2:193742529-193989223
chr2	193913299	193913968	chr2:193742529-193989223
chr2	193913974	193914049	chr2:193742529-193989223
chr2	193914059	193914561	chr2:193742529-193989223
chr2	193914579	193915626	chr2:193742529-193989223
chr2	193915634	193915882	chr2:193742529-193989223
chr2	193916169	193916445	chr2:193742529-193989223
chr2	193916474	193916780	chr2:193742529-193989223
chr2	193916804	193917055	chr2:193742529-193989223
chr2	193917064	193917688	chr2:193742529-193989223
chr2	193917689	193918010	chr2:193742529-193989223
chr2	193918019	193921299	chr2:193742529-193989223
chr2	193921364	193922490	chr2:193742529-193989223
chr2	193922909	193923011	chr2:193742529-193989223
chr2	193923014	193923121	chr2:193742529-193989223
chr2	193923164	193923302	chr2:193742529-193989223
chr2	193923414	193923611	chr2:193742529-193989223
chr2	193923629	193923731	chr2:193742529-193989223
chr2	193923779	193925328	chr2:193742529-193989223
chr2	193925494	193925652	chr2:193742529-193989223
chr2	193925714	193926532	chr2:193742529-193989223
chr2	193926534	193926951	chr2:193742529-193989223
chr2	193927144	193927946	chr2:193742529-193989223
chr2	193928199	193928965	chr2:193742529-193989223
chr2	193929004	193929169	chr2:193742529-193989223
chr2	193929299	193929539	chr2:193742529-193989223
chr2	193929594	193929785	chr2:193742529-193989223
chr2	193929804	193930004	chr2:193742529-193989223
chr2	193932364	193932707	chr2:193742529-193989223
chr2	193932754	193932868	chr2:193742529-193989223
chr2	193932914	193933143	chr2:193742529-193989223
chr2	193933159	193933278	chr2:193742529-193989223
chr2	193933309	193933722	chr2:193742529-193989223
chr2	193933739	193933882	chr2:193742529-193989223
chr2	193933894	193933968	chr2:193742529-193989223
chr2	193933989	193934088	chr2:193742529-193989223
chr2	193934094	193934222	chr2:193742529-193989223
chr2	193934304	193934706	chr2:193742529-193989223
chr2	193934709	193934846	chr2:193742529-193989223
chr2	193934849	193935257	chr2:193742529-193989223
chr2	193935294	193935746	chr2:193742529-193989223
chr2	193935754	193935869	chr2:193742529-193989223
chr2	193935889	193936156	chr2:193742529-193989223
chr2	193936164	193936304	chr2:193742529-193989223
chr2	193936309	193936715	chr2:193742529-193989223
chr2	193936759	193939873	chr2:193742529-193989223
chr2	193939874	193941471	chr2:193742529-193989223
chr2	193941479	193944042	chr2:193742529-193989223
chr2	193944049	193945092	chr2:193742529-193989223
chr2	193945094	193948583	chr2:193742529-193989223
chr2	193948584	193949271	chr2:193742529-193989223
chr2	193949274	193950076	chr2:193742529-193989223
chr2	193950109	193950496	chr2:193742529-193989223

chr2	193950509	193950951	chr2:193742529-193989223
chr2	193950959	193951683	chr2:193742529-193989223
chr2	193951689	193952219	chr2:193742529-193989223
chr2	193952224	193953895	chr2:193742529-193989223
chr2	193953909	193954997	chr2:193742529-193989223
chr2	193954999	193955382	chr2:193742529-193989223
chr2	193955389	193955631	chr2:193742529-193989223
chr2	193955659	193956456	chr2:193742529-193989223
chr2	193956459	193956646	chr2:193742529-193989223
chr2	193956724	193957015	chr2:193742529-193989223
chr2	193957019	193958169	chr2:193742529-193989223
chr2	193958434	193959076	chr2:193742529-193989223
chr2	193959084	193961074	chr2:193742529-193989223
chr2	193961309	193962899	chr2:193742529-193989223
chr2	193962989	193963088	chr2:193742529-193989223
chr2	193963119	193963571	chr2:193742529-193989223
chr2	193963714	193963819	chr2:193742529-193989223
chr2	193963829	193964069	chr2:193742529-193989223
chr2	193964094	193964318	chr2:193742529-193989223
chr2	193964344	193964581	chr2:193742529-193989223
chr2	193964634	193964920	chr2:193742529-193989223
chr2	193964939	193965319	chr2:193742529-193989223
chr2	193965364	193966158	chr2:193742529-193989223
chr2	193966434	193969887	chr2:193742529-193989223
chr2	193969894	193970043	chr2:193742529-193989223
chr2	193970049	193971114	chr2:193742529-193989223
chr2	193971169	193971482	chr2:193742529-193989223
chr2	193971499	193971651	chr2:193742529-193989223
chr2	193971654	193972560	chr2:193742529-193989223
chr2	193972574	193973646	chr2:193742529-193989223
chr2	193973659	193973738	chr2:193742529-193989223
chr2	193973864	193974435	chr2:193742529-193989223
chr2	193974439	193975989	chr2:193742529-193989223
chr2	193975994	193978875	chr2:193742529-193989223
chr2	193978884	193978956	chr2:193742529-193989223
chr2	193978969	193979135	chr2:193742529-193989223
chr2	193979184	193979382	chr2:193742529-193989223
chr2	193979389	193979647	chr2:193742529-193989223
chr2	193979649	193979783	chr2:193742529-193989223
chr2	193979799	193980961	chr2:193742529-193989223
chr2	193981214	193983024	chr2:193742529-193989223
chr2	193983034	193983863	chr2:193742529-193989223
chr2	193983954	193984422	chr2:193742529-193989223
chr2	193984424	193985293	chr2:193742529-193989223
chr2	193985294	193986144	chr2:193742529-193989223
chr2	193986149	193986245	chr2:193742529-193989223
chr2	193986249	193987652	chr2:193742529-193989223
chr2	193987664	193988057	chr2:193742529-193989223
chr2	193988064	193988426	chr2:193742529-193989223
chr2	193988429	193989251	chr2:193742529-193989223
chr2	200134189	200134415	chr2:200134222-200137395
chr2	200134424	200135234	chr2:200134222-200137395
chr2	200135239	200136194	chr2:200134222-200137395
chr2	200136199	200136293	chr2:200134222-200137395
chr2	200136294	200136501	chr2:200134222-200137395
chr2	200136529	200136813	chr2:200134222-200137395
chr2	200136854	200137416	chr2:200134222-200137395
chr2	200173459	200173706	chr2:200173482-200173680
chr2	200188504	200188703	chr2:200188525-200188681
chr2	200190049	200190223	chr2:200190032-200190186
chr2	200190694	200190842	chr2:200190717-200190820
chr2	200193394	200194089	chr2:200193420-200194085
chr2	200211894	200212713	chr2:200211588-200213896

chr2	200212729	200212961	chr2:200211588-200213896
chr2	200212984	200213920	chr2:200211588-200213896
chr2	200233304	200233687	chr2:200233327-200233666
chr2	200245064	200245245	chr2:200245086-200245210
chr2	200246394	200246566	chr2:200246416-200246543
chr2	200260574	200261598	chr2:200260553-200261729
chr2	200261604	200261742	chr2:200260553-200261729
chr2	200262229	200262343	chr2:200262252-200262563
chr2	200279804	200280363	chr2:200279828-200280327
chr2	200288384	200288676	chr2:200288408-200288638
chr2	200298039	200298261	chr2:200298060-200298237
chr2	200320569	200320835	chr2:200320591-200320819
chr2	200322039	200322292	chr2:200322061-200323468
chr2	200322299	200323494	chr2:200322061-200323468
chr2	200324334	200324655	chr2:200324365-200324615
chr2	200325149	200325362	chr2:200325174-200325324
chr2	200329534	200329821	chr2:200329558-200329890
chr2	200329829	200329931	chr2:200329558-200329890
chr2	200332114	200332252	chr2:200332135-200332228
chr2	200332804	200333016	chr2:200332810-200332984
chr2	200333569	200333683	chr2:200333608-200333642
chr2	200334299	200336689	chr2:200334329-200337479
chr2	200336694	200337503	chr2:200334329-200337479
chr2	200338914	200339464	chr2:200338936-200339435
chr2	200341174	200341687	chr2:200341199-200341658
chr3	1856715	1856896	chr3:1856749-1909453
chr3	1857170	1858495	chr3:1856749-1909453
chr3	1858515	1859261	chr3:1856749-1909453
chr3	1859910	1861508	chr3:1856749-1909453
chr3	1861515	1861947	chr3:1856749-1909453
chr3	1861965	1862452	chr3:1856749-1909453
chr3	1862460	1864709	chr3:1856749-1909453
chr3	1864740	1864843	chr3:1856749-1909453
chr3	1864900	1865963	chr3:1856749-1909453
chr3	1865965	1866710	chr3:1856749-1909453
chr3	1866715	1866868	chr3:1856749-1909453
chr3	1866875	1866949	chr3:1856749-1909453
chr3	1866980	1871785	chr3:1856749-1909453
chr3	1871790	1872185	chr3:1856749-1909453
chr3	1872190	1872422	chr3:1856749-1909453
chr3	1872425	1873645	chr3:1856749-1909453
chr3	1873695	1873793	chr3:1856749-1909453
chr3	1873810	1873890	chr3:1856749-1909453
chr3	1873910	1877019	chr3:1856749-1909453
chr3	1877020	1877419	chr3:1856749-1909453
chr3	1877430	1877657	chr3:1856749-1909453
chr3	1877945	1879370	chr3:1856749-1909453
chr3	1879490	1879589	chr3:1856749-1909453
chr3	1879695	1883289	chr3:1856749-1909453
chr3	1883290	1883702	chr3:1856749-1909453
chr3	1883705	1884548	chr3:1856749-1909453
chr3	1884555	1885895	chr3:1856749-1909453
chr3	1885915	1886321	chr3:1856749-1909453
chr3	1886330	1886601	chr3:1856749-1909453
chr3	1886605	1887196	chr3:1856749-1909453
chr3	1887240	1887455	chr3:1856749-1909453
chr3	1887510	1887807	chr3:1856749-1909453
chr3	1887820	1888641	chr3:1856749-1909453
chr3	1888665	1889666	chr3:1856749-1909453
chr3	1889675	1891649	chr3:1856749-1909453
chr3	1894360	1897098	chr3:1856749-1909453
chr3	1897105	1897431	chr3:1856749-1909453
chr3	1897435	1898911	chr3:1856749-1909453

chr3	1898950	1899276	chr3:1856749-1909453
chr3	1899280	1899477	chr3:1856749-1909453
chr3	1899480	1900055	chr3:1856749-1909453
chr3	1900350	1901188	chr3:1856749-1909453
chr3	1901200	1901276	chr3:1856749-1909453
chr3	1901335	1901407	chr3:1856749-1909453
chr3	1901505	1902101	chr3:1856749-1909453
chr3	1902110	1905406	chr3:1856749-1909453
chr3	1905860	1905931	chr3:1856749-1909453
chr3	1905960	1906525	chr3:1856749-1909453
chr3	1906530	1906677	chr3:1856749-1909453
chr3	1906900	1907098	chr3:1856749-1909453
chr3	1907535	1908358	chr3:1856749-1909453
chr3	1908365	1909407	chr3:1856749-1909453
chr3	2140455	2140695	chr3:2140486-2140662
chr3	2140930	2141483	chr3:2140957-2141457
chr3	2142220	2142350	chr3:2142241-2142323
chr3	2152060	2152341	chr3:2152092-2152316
chr3	2174020	2174166	chr3:2174044-2174134
chr3	2185780	2185961	chr3:2185813-2185925
chr3	2280700	2280769	chr3:2280512-2280752
chr3	2304005	2304138	chr3:2304029-2304114
chr3	2380840	2380940	chr3:2380861-2380917
chr3	2411065	2411463	chr3:2411087-2411586
chr3	2411475	2411622	chr3:2411087-2411586
chr3	2428140	2428702	chr3:2428172-2428682
chr3	2434685	2434796	chr3:2434699-2434774
chr3	2552895	2553749	chr3:2552928-2553710
chr3	2612995	2613389	chr3:2613024-2613344
chr3	2671175	2671287	chr3:2671209-2671263
chr3	2777535	2778054	chr3:2777556-2778025
chr3	2787180	2787425	chr3:2787205-2787381
chr3	2861145	2861281	chr3:2861169-2861265
chr3	2908075	2908218	chr3:2908101-2908185
chr3	2908410	2908646	chr3:2908435-2908633
chr3	2924400	2924961	chr3:2924422-2924931
chr3	2928700	2928943	chr3:2928723-2928908
chr3	2933875	2933975	chr3:2933898-2933944
chr3	2942340	2942515	chr3:2942365-2942505
chr3	2944535	2944712	chr3:2944559-2944689
chr3	2967290	2967488	chr3:2967312-2967463
chr3	2968950	2969337	chr3:2968981-2969315
chr3	3030005	3030728	chr3:3030028-3030697
chr3	3040710	3040858	chr3:3040731-3040828
chr3	3067775	3067978	chr3:3067785-3067961
chr3	3072505	3072672	chr3:3072538-3072659
chr3	3076290	3076504	chr3:3076315-3076474
chr3	3078840	3079402	chr3:3078862-3079360
chr3	3080595	3080710	chr3:3080616-3080687
chr3	3080950	3082109	chr3:3080956-3082066
chr3	3082700	3082882	chr3:3082728-3082838
chr3	3083970	3084147	chr3:3083993-3084106
chr3	3084565	3084888	chr3:3084596-3084847
chr3	3085245	3085417	chr3:3085275-3085388
chr3	3095460	3095674	chr3:3095490-3095659
chr3	3097775	3099486	chr3:3097803-3099645
chr3	3099505	3099684	chr3:3097803-3099645
chr3	3102715	3102866	chr3:3102750-3102829
chr3	17784399	17784650	chr3:17784427-17784610
chr3	17786824	17787138	chr3:17786859-17787157
chr3	17793319	17793554	chr3:17793295-17888256
chr3	17793829	17796097	chr3:17793295-17888256
chr3	17796384	17799524	chr3:17793295-17888256

chr3	17799799	17800762	chr3:17793295-17888256
chr3	17801054	17801622	chr3:17793295-17888256
chr3	17801629	17802073	chr3:17793295-17888256
chr3	17802079	17802388	chr3:17793295-17888256
chr3	17802679	17803380	chr3:17793295-17888256
chr3	17803669	17803911	chr3:17793295-17888256
chr3	17804189	17806910	chr3:17793295-17888256
chr3	17807189	17807482	chr3:17793295-17888256
chr3	17807484	17809039	chr3:17793295-17888256
chr3	17809714	17809851	chr3:17793295-17888256
chr3	17810014	17810082	chr3:17793295-17888256
chr3	17810304	17813169	chr3:17793295-17888256
chr3	17813179	17813482	chr3:17793295-17888256
chr3	17813489	17813716	chr3:17793295-17888256
chr3	17813729	17815066	chr3:17793295-17888256
chr3	17815074	17815342	chr3:17793295-17888256
chr3	17815664	17815778	chr3:17793295-17888256
chr3	17816764	17816857	chr3:17793295-17888256
chr3	17817029	17817241	chr3:17793295-17888256
chr3	17817249	17817319	chr3:17793295-17888256
chr3	17817749	17817835	chr3:17793295-17888256
chr3	17818409	17818485	chr3:17793295-17888256
chr3	17818494	17818712	chr3:17793295-17888256
chr3	17818739	17818811	chr3:17793295-17888256
chr3	17818869	17818945	chr3:17793295-17888256
chr3	17818969	17819085	chr3:17793295-17888256
chr3	17819629	17819713	chr3:17793295-17888256
chr3	17819924	17820000	chr3:17793295-17888256
chr3	17820119	17820193	chr3:17793295-17888256
chr3	17820224	17820331	chr3:17793295-17888256
chr3	17820369	17820570	chr3:17793295-17888256
chr3	17820574	17820652	chr3:17793295-17888256
chr3	17820759	17820837	chr3:17793295-17888256
chr3	17821164	17821289	chr3:17793295-17888256
chr3	17821514	17821589	chr3:17793295-17888256
chr3	17822139	17822218	chr3:17793295-17888256
chr3	17822824	17822903	chr3:17793295-17888256
chr3	17823014	17823086	chr3:17793295-17888256
chr3	17823599	17823676	chr3:17793295-17888256
chr3	17823854	17824599	chr3:17793295-17888256
chr3	17824904	17825406	chr3:17793295-17888256
chr3	17825444	17825708	chr3:17793295-17888256
chr3	17825719	17825821	chr3:17793295-17888256
chr3	17825914	17826055	chr3:17793295-17888256
chr3	17826344	17826430	chr3:17793295-17888256
chr3	17826589	17826658	chr3:17793295-17888256
chr3	17826719	17826819	chr3:17793295-17888256
chr3	17826854	17827028	chr3:17793295-17888256
chr3	17827149	17827221	chr3:17793295-17888256
chr3	17827389	17827462	chr3:17793295-17888256
chr3	17827469	17827543	chr3:17793295-17888256
chr3	17827964	17828081	chr3:17793295-17888256
chr3	17828084	17828278	chr3:17793295-17888256
chr3	17828304	17828503	chr3:17793295-17888256
chr3	17828504	17828647	chr3:17793295-17888256
chr3	17828679	17828851	chr3:17793295-17888256
chr3	17829694	17829806	chr3:17793295-17888256
chr3	17829934	17830021	chr3:17793295-17888256
chr3	17830024	17830105	chr3:17793295-17888256
chr3	17830304	17830400	chr3:17793295-17888256
chr3	17831334	17832018	chr3:17793295-17888256
chr3	17832414	17832929	chr3:17793295-17888256
chr3	17833399	17834325	chr3:17793295-17888256

chr3	17834609	17835677	chr3:17793295-17888256
chr3	17835684	17836195	chr3:17793295-17888256
chr3	17836204	17837259	chr3:17793295-17888256
chr3	17837294	17837483	chr3:17793295-17888256
chr3	17837589	17837726	chr3:17793295-17888256
chr3	17837879	17837950	chr3:17793295-17888256
chr3	17838009	17838826	chr3:17793295-17888256
chr3	17838849	17839185	chr3:17793295-17888256
chr3	17839479	17841309	chr3:17793295-17888256
chr3	17841594	17842713	chr3:17793295-17888256
chr3	17842714	17845214	chr3:17793295-17888256
chr3	17845499	17845870	chr3:17793295-17888256
chr3	17845874	17846135	chr3:17793295-17888256
chr3	17846139	17846226	chr3:17793295-17888256
chr3	17846254	17846631	chr3:17793295-17888256
chr3	17846929	17847522	chr3:17793295-17888256
chr3	17847804	17849093	chr3:17793295-17888256
chr3	17849139	17849596	chr3:17793295-17888256
chr3	17849609	17852656	chr3:17793295-17888256
chr3	17852664	17853667	chr3:17793295-17888256
chr3	17853944	17854657	chr3:17793295-17888256
chr3	17854959	17855573	chr3:17793295-17888256
chr3	17855869	17856466	chr3:17793295-17888256
chr3	17856474	17857305	chr3:17793295-17888256
chr3	17857594	17858081	chr3:17793295-17888256
chr3	17858409	17858480	chr3:17793295-17888256
chr3	17859169	17859407	chr3:17793295-17888256
chr3	17859419	17859498	chr3:17793295-17888256
chr3	17860029	17861174	chr3:17793295-17888256
chr3	17861224	17862810	chr3:17793295-17888256
chr3	17862854	17863327	chr3:17793295-17888256
chr3	17863759	17864991	chr3:17793295-17888256
chr3	17865039	17866231	chr3:17793295-17888256
chr3	17866499	17870547	chr3:17793295-17888256
chr3	17870554	17871120	chr3:17793295-17888256
chr3	17871429	17871525	chr3:17793295-17888256
chr3	17871664	17871884	chr3:17793295-17888256
chr3	17871889	17872253	chr3:17793295-17888256
chr3	17872294	17875659	chr3:17793295-17888256
chr3	17875869	17876296	chr3:17793295-17888256
chr3	17876599	17876678	chr3:17793295-17888256
chr3	17876809	17876890	chr3:17793295-17888256
chr3	17876959	17879791	chr3:17793295-17888256
chr3	17879799	17880020	chr3:17793295-17888256
chr3	17880024	17880316	chr3:17793295-17888256
chr3	17880379	17883209	chr3:17793295-17888256
chr3	17883219	17884525	chr3:17793295-17888256
chr3	17884834	17885157	chr3:17793295-17888256
chr3	17885244	17885320	chr3:17793295-17888256
chr3	17885844	17888268	chr3:17793295-17888256
chr3	20370499	20370570	chr3:20370546-20380546
chr3	20370604	20371108	chr3:20370546-20380546
chr3	20371109	20371936	chr3:20370546-20380546
chr3	20372229	20372906	chr3:20370546-20380546
chr3	20372909	20373473	chr3:20370546-20380546
chr3	20373769	20374025	chr3:20370546-20380546
chr3	20374029	20377323	chr3:20370546-20380546
chr3	20377324	20377456	chr3:20370546-20380546
chr3	20377574	20378461	chr3:20370546-20380546
chr3	20378499	20379462	chr3:20370546-20380546
chr3	20379474	20380571	chr3:20370546-20380546
chr3	20383759	20385552	chr3:20383781-20385528
chr3	20387414	20387586	chr3:20387151-20387558

chr3	20392179	20392275	chr3:20392204-20392514
chr3	20392279	20392522	chr3:20392204-20392514
chr3	20392724	20392870	chr3:20392746-20392838
chr3	20401004	20401112	chr3:20401013-20401081
chr3	20422634	20422854	chr3:20422666-20423969
chr3	20422859	20422983	chr3:20422666-20423969
chr3	20422984	20423058	chr3:20422666-20423969
chr3	20423069	20423145	chr3:20422666-20423969
chr3	20423154	20423370	chr3:20422666-20423969
chr3	20423524	20423659	chr3:20422666-20423969
chr3	20423719	20423945	chr3:20422666-20423969
chr3	20429709	20429884	chr3:20429740-20432054
chr3	20429979	20430151	chr3:20429740-20432054
chr3	20430209	20430316	chr3:20429740-20432054
chr3	20430344	20432088	chr3:20429740-20432054
chr3	27758412	27759321	chr3:27758439-27759299
chr3	27760047	27760153	chr3:27760077-27760139
chr3	27760192	27760405	chr3:27760225-27760384
chr3	27760817	27760993	chr3:27760850-27760972
chr3	27761627	27761842	chr3:27761661-27761816
chr3	27762882	27763404	chr3:27762904-27764206
chr3	27763417	27764227	chr3:27762904-27764206
chr3	28039152	28039397	chr3:28038700-28039359
chr3	28047217	28047310	chr3:28047238-28047291
chr3	28051937	28052069	chr3:28051960-28052055
chr3	28054957	28055106	chr3:28054990-28055089
chr3	28056382	28056507	chr3:28056434-28056447
chr3	28061657	28061820	chr3:28061679-28078936
chr3	28061827	28062067	chr3:28061679-28078936
chr3	28062082	28063440	chr3:28061679-28078936
chr3	28063442	28063627	chr3:28061679-28078936
chr3	28063632	28064169	chr3:28061679-28078936
chr3	28064172	28064769	chr3:28061679-28078936
chr3	28064872	28065053	chr3:28061679-28078936
chr3	28067177	28067263	chr3:28061679-28078936
chr3	28067397	28067473	chr3:28061679-28078936
chr3	28067572	28067709	chr3:28061679-28078936
chr3	28067972	28068110	chr3:28061679-28078936
chr3	28068172	28068274	chr3:28061679-28078936
chr3	28068422	28068506	chr3:28061679-28078936
chr3	28068622	28068694	chr3:28061679-28078936
chr3	28068722	28069309	chr3:28061679-28078936
chr3	28069687	28071581	chr3:28061679-28078936
chr3	28071587	28072391	chr3:28061679-28078936
chr3	28072672	28073021	chr3:28061679-28078936
chr3	28073102	28073522	chr3:28061679-28078936
chr3	28073532	28073621	chr3:28061679-28078936
chr3	28073692	28073766	chr3:28061679-28078936
chr3	28073892	28074066	chr3:28061679-28078936
chr3	28074167	28074242	chr3:28061679-28078936
chr3	28074292	28074371	chr3:28061679-28078936
chr3	28074392	28074471	chr3:28061679-28078936
chr3	28074542	28074943	chr3:28061679-28078936
chr3	28075037	28075187	chr3:28061679-28078936
chr3	28075222	28075370	chr3:28061679-28078936
chr3	28075587	28075701	chr3:28061679-28078936
chr3	28075927	28077487	chr3:28061679-28078936
chr3	28077542	28078339	chr3:28061679-28078936
chr3	28078347	28078921	chr3:28061679-28078936
chr3	28079017	28079121	chr3:28078979-28079085
chr3	30391727	30392465	chr3:30391759-30392431
chr3	30401847	30401954	chr3:30401884-30401903
chr3	30402642	30402841	chr3:30402663-30402807

chr3	30414307	30414667	chr3:30414334-30414649
chr3	30416177	30416703	chr3:30416201-30416679
chr3	30431192	30431325	chr3:30431199-30431315
chr3	30431467	30431576	chr3:30431491-30431539
chr3	30433317	30437174	chr3:30433345-30437550
chr3	30437297	30437382	chr3:30433345-30437550
chr3	30450467	30452884	chr3:30450499-30470027
chr3	30455762	30457251	chr3:30450499-30470027
chr3	30457262	30457418	chr3:30450499-30470027
chr3	30457462	30460523	chr3:30450499-30470027
chr3	30460532	30460742	chr3:30450499-30470027
chr3	30460802	30460916	chr3:30450499-30470027
chr3	30460917	30461076	chr3:30450499-30470027
chr3	30461077	30461187	chr3:30450499-30470027
chr3	30461227	30461352	chr3:30450499-30470027
chr3	30461362	30461484	chr3:30450499-30470027
chr3	30461497	30461768	chr3:30450499-30470027
chr3	30461772	30462923	chr3:30450499-30470027
chr3	30462962	30464330	chr3:30450499-30470027
chr3	30464627	30465575	chr3:30450499-30470027
chr3	30465582	30466175	chr3:30450499-30470027
chr3	30466177	30466293	chr3:30450499-30470027
chr3	30466312	30467023	chr3:30450499-30470027
chr3	30467042	30467326	chr3:30450499-30470027
chr3	30467382	30467549	chr3:30450499-30470027
chr3	30467552	30467663	chr3:30450499-30470027
chr3	30467667	30468972	chr3:30450499-30470027
chr3	30469142	30469376	chr3:30450499-30470027
chr3	30469397	30470139	chr3:30450499-30470027;chr3:30470063-30470107
chr3	30475547	30478434	chr3:30475572-30480946
chr3	30478447	30478793	chr3:30475572-30480946
chr3	30478807	30480791	chr3:30475572-30480946
chr3	30480797	30480974	chr3:30475572-30480946
chr3	30482477	30482682	chr3:30482498-30482646
chr3	30485137	30485236	chr3:30485163-30485202
chr3	30510502	30510615	chr3:30510524-30510576
chr3	30528612	30528681	chr3:30528660-30528678
chr3	36808577	36808661	chr3:36808607-36810751
chr3	36808692	36809037	chr3:36808607-36810751
chr3	36809057	36809160	chr3:36808607-36810751
chr3	36809197	36809570	chr3:36808607-36810751
chr3	36809607	36809801	chr3:36808607-36810751
chr3	36809827	36810070	chr3:36808607-36810751
chr3	36810137	36810390	chr3:36808607-36810751
chr3	36810517	36810760	chr3:36808607-36810751
chr3	36813397	36813510	chr3:36813422-36813489
chr3	36825602	36825810	chr3:36825623-36825778
chr3	36838537	36838969	chr3:36838564-36858583
chr3	36838977	36839434	chr3:36838564-36858583
chr3	36841907	36842793	chr3:36838564-36858583
chr3	36842797	36844098	chr3:36838564-36858583
chr3	36844162	36844233	chr3:36838564-36858583
chr3	36844572	36849158	chr3:36838564-36858583
chr3	36849372	36849504	chr3:36838564-36858583
chr3	36849507	36850104	chr3:36838564-36858583
chr3	36850187	36850579	chr3:36838564-36858583
chr3	36850587	36850727	chr3:36838564-36858583
chr3	36851007	36851280	chr3:36838564-36858583
chr3	36851387	36852077	chr3:36838564-36858583
chr3	36852082	36852688	chr3:36838564-36858583
chr3	36852977	36853085	chr3:36838564-36858583
chr3	36853202	36853611	chr3:36838564-36858583
chr3	36853617	36854218	chr3:36838564-36858583

chr3	36854227	36858007	chr3:36838564-36858583
chr3	36858072	36858599	chr3:36838564-36858583
chr3	36858982	36859557	chr3:36859013-36859512
chr3	36860742	36860847	chr3:36860766-36861123
chr3	36860872	36861154	chr3:36860766-36861123
chr3	36862197	36862802	chr3:36862219-36862908
chr3	36862807	36862944	chr3:36862219-36862908
chr3	36863862	36864281	chr3:36863886-36863991;chr3:36864019-36864244
chr3	36868277	36869871	chr3:36868310-36869866
chr3	36871022	36871174	chr3:36871054-36871153
chr3	36872342	36874619	chr3:36872363-36875410
chr3	36874632	36875437	chr3:36872363-36875410
chr3	36876227	36876433	chr3:36876252-36876398
chr3	36879837	36880003	chr3:36879862-36879995
chr3	36880072	36880224	chr3:36880103-36880207
chr3	36884082	36884224	chr3:36884112-36884201
chr3	36887707	36887940	chr3:36887738-36887895
chr3	36888657	36888853	chr3:36888690-36888837
chr3	36890357	36890912	chr3:36890382-36890882
chr3	36891622	36892007	chr3:36891644-36892142
chr3	36893182	36893354	chr3:36893209-36893347
chr3	36893612	36893853	chr3:36893636-36893836
chr3	36896642	36897313	chr3:36896663-36899540
chr3	36897322	36897810	chr3:36896663-36899540
chr3	36897822	36899567	chr3:36896663-36899540
chr3	36900177	36900417	chr3:36900208-36900385
chr3	36902362	36902682	chr3:36902396-36902651
chr3	36905787	36905998	chr3:36905809-36905971
chr3	36915587	36915811	chr3:36915616-36915787
chr3	36931292	36931465	chr3:36931318-36931451
chr3	36933667	36933836	chr3:36933692-36933831
chr3	36934367	36934506	chr3:36934391-36934475
chr3	36937102	36937277	chr3:36937130-36937249
chr3	36940567	36940788	chr3:36940597-36940750
chr3	36944612	36944783	chr3:36944635-36944766
chr3	36949787	36950101	chr3:36949813-36950062
chr3	36986252	36986569	chr3:36986277-36986548
chr3	51907587	51907728	chr3:51907611-51907686
chr3	51908207	51908351	chr3:51908241-51908314
chr3	51908372	51909531	chr3:51908403-51909493
chr3	51909627	51909800	chr3:51909650-51909783
chr3	51912357	51912890	chr3:51912388-51926817
chr3	51913022	51913142	chr3:51912388-51926817
chr3	51913502	51913578	chr3:51912388-51926817
chr3	51913597	51913913	chr3:51912388-51926817
chr3	51913952	51914315	chr3:51912388-51926817
chr3	51914652	51914781	chr3:51912388-51926817
chr3	51914787	51916270	chr3:51912388-51926817
chr3	51916562	51917772	chr3:51912388-51926817
chr3	51918012	51918090	chr3:51912388-51926817
chr3	51918157	51918590	chr3:51912388-51926817
chr3	51918592	51918707	chr3:51912388-51926817
chr3	51918772	51920951	chr3:51912388-51926817
chr3	51920962	51921103	chr3:51912388-51926817
chr3	51921372	51922021	chr3:51912388-51926817
chr3	51922317	51923277	chr3:51912388-51926817
chr3	51923562	51924768	chr3:51912388-51926817
chr3	51925047	51925931	chr3:51912388-51926817
chr3	51925937	51926564	chr3:51912388-51926817
chr3	77714436	77714511	chr3:77714427-77720924
chr3	77714776	77714845	chr3:77714427-77720924
chr3	77715536	77715613	chr3:77714427-77720924
chr3	77715756	77715953	chr3:77714427-77720924

chr3	77716221	77718819	chr3:77714427-77720924
chr3	77719116	77720961	chr3:77714427-77720924
chr3	87471197	87471471	chr3:87471224-87550580
chr3	87471507	87474545	chr3:87471224-87550580
chr3	87474847	87475498	chr3:87471224-87550580
chr3	87475617	87475698	chr3:87471224-87550580
chr3	87475717	87475843	chr3:87471224-87550580
chr3	87475852	87476860	chr3:87471224-87550580
chr3	87476867	87477480	chr3:87471224-87550580
chr3	87477492	87478289	chr3:87471224-87550580
chr3	87478307	87479349	chr3:87471224-87550580
chr3	87479367	87479781	chr3:87471224-87550580
chr3	87479797	87481614	chr3:87471224-87550580
chr3	87481907	87482623	chr3:87471224-87550580
chr3	87482777	87482848	chr3:87471224-87550580
chr3	87482902	87483013	chr3:87471224-87550580
chr3	87483042	87483401	chr3:87471224-87550580
chr3	87483577	87483841	chr3:87471224-87550580
chr3	87483852	87485888	chr3:87471224-87550580
chr3	87485892	87486120	chr3:87471224-87550580
chr3	87486147	87486515	chr3:87471224-87550580
chr3	87486527	87489588	chr3:87471224-87550580
chr3	87489602	87490871	chr3:87471224-87550580
chr3	87490877	87491307	chr3:87471224-87550580
chr3	87491312	87492946	chr3:87471224-87550580
chr3	87492962	87493203	chr3:87471224-87550580
chr3	87493492	87493917	chr3:87471224-87550580
chr3	87494347	87494914	chr3:87471224-87550580
chr3	87494917	87495030	chr3:87471224-87550580
chr3	87495037	87495259	chr3:87471224-87550580
chr3	87495287	87495586	chr3:87471224-87550580
chr3	87495587	87495665	chr3:87471224-87550580
chr3	87495667	87495740	chr3:87471224-87550580
chr3	87495857	87498312	chr3:87471224-87550580
chr3	87498317	87499606	chr3:87471224-87550580
chr3	87499607	87500123	chr3:87471224-87550580
chr3	87500172	87501914	chr3:87471224-87550580
chr3	87502052	87502156	chr3:87471224-87550580
chr3	87502212	87502288	chr3:87471224-87550580
chr3	87502822	87502905	chr3:87471224-87550580
chr3	87503657	87503785	chr3:87471224-87550580
chr3	87503877	87503955	chr3:87471224-87550580
chr3	87503992	87504115	chr3:87471224-87550580
chr3	87504167	87504270	chr3:87471224-87550580
chr3	87504302	87504462	chr3:87471224-87550580
chr3	87504472	87504684	chr3:87471224-87550580
chr3	87504737	87504974	chr3:87471224-87550580
chr3	87504977	87505387	chr3:87471224-87550580
chr3	87505392	87505511	chr3:87471224-87550580
chr3	87505512	87506148	chr3:87471224-87550580
chr3	87506442	87507584	chr3:87471224-87550580
chr3	87507872	87511063	chr3:87471224-87550580
chr3	87511122	87511202	chr3:87471224-87550580
chr3	87511207	87511496	chr3:87471224-87550580
chr3	87511497	87512078	chr3:87471224-87550580
chr3	87512087	87512304	chr3:87471224-87550580
chr3	87512307	87512602	chr3:87471224-87550580
chr3	87512617	87512805	chr3:87471224-87550580
chr3	87512807	87512972	chr3:87471224-87550580
chr3	87512977	87513095	chr3:87471224-87550580
chr3	87513117	87513338	chr3:87471224-87550580
chr3	87513382	87513454	chr3:87471224-87550580
chr3	87513467	87513858	chr3:87471224-87550580

chr3	87513942	87514138	chr3:87471224-87550580
chr3	87514147	87514293	chr3:87471224-87550580
chr3	87514377	87514453	chr3:87471224-87550580
chr3	87514737	87514811	chr3:87471224-87550580
chr3	87514812	87514962	chr3:87471224-87550580
chr3	87515042	87515130	chr3:87471224-87550580
chr3	87515437	87515530	chr3:87471224-87550580
chr3	87515932	87516049	chr3:87471224-87550580
chr3	87516052	87516140	chr3:87471224-87550580
chr3	87516272	87516599	chr3:87471224-87550580
chr3	87516692	87516771	chr3:87471224-87550580
chr3	87516837	87516986	chr3:87471224-87550580
chr3	87517112	87518479	chr3:87471224-87550580
chr3	87518492	87519530	chr3:87471224-87550580
chr3	87519547	87520392	chr3:87471224-87550580
chr3	87520402	87520784	chr3:87471224-87550580
chr3	87520797	87521297	chr3:87471224-87550580
chr3	87521497	87522409	chr3:87471224-87550580
chr3	87523267	87523393	chr3:87471224-87550580
chr3	87523442	87523527	chr3:87471224-87550580
chr3	87523727	87523812	chr3:87471224-87550580
chr3	87523902	87524005	chr3:87471224-87550580
chr3	87524257	87524355	chr3:87471224-87550580
chr3	87524582	87526504	chr3:87471224-87550580
chr3	87526547	87526934	chr3:87471224-87550580
chr3	87526977	87527697	chr3:87471224-87550580
chr3	87527732	87527955	chr3:87471224-87550580
chr3	87528232	87528545	chr3:87471224-87550580
chr3	87528617	87528759	chr3:87471224-87550580
chr3	87528872	87529023	chr3:87471224-87550580
chr3	87529157	87530005	chr3:87471224-87550580
chr3	87530012	87530598	chr3:87471224-87550580
chr3	87531967	87532905	chr3:87471224-87550580
chr3	87532957	87533029	chr3:87471224-87550580
chr3	87533102	87534098	chr3:87471224-87550580
chr3	87534137	87534480	chr3:87471224-87550580
chr3	87534482	87534653	chr3:87471224-87550580
chr3	87534827	87535676	chr3:87471224-87550580
chr3	87535702	87535824	chr3:87471224-87550580
chr3	87536177	87537569	chr3:87471224-87550580
chr3	87537572	87537878	chr3:87471224-87550580
chr3	87537927	87539073	chr3:87471224-87550580
chr3	87539077	87539510	chr3:87471224-87550580
chr3	87539522	87539601	chr3:87471224-87550580
chr3	87539612	87540125	chr3:87471224-87550580
chr3	87540457	87540961	chr3:87471224-87550580
chr3	87540967	87541035	chr3:87471224-87550580
chr3	87541062	87541517	chr3:87471224-87550580
chr3	87541842	87542719	chr3:87471224-87550580
chr3	87542757	87543314	chr3:87471224-87550580
chr3	87543967	87544139	chr3:87471224-87550580
chr3	87544142	87544627	chr3:87471224-87550580
chr3	87544667	87544782	chr3:87471224-87550580
chr3	87544827	87544911	chr3:87471224-87550580
chr3	87544917	87545305	chr3:87471224-87550580
chr3	87545312	87546644	chr3:87471224-87550580
chr3	87546937	87549290	chr3:87471224-87550580
chr3	87549307	87549476	chr3:87471224-87550580
chr3	87549477	87550593	chr3:87471224-87550580
chr3	101587485	101587663	chr3:101587520-101587637
chr3	101605520	101605699	chr3:101605553-101605676
chr3	101645800	101645910	chr3:101645845-101645870
chr3	101659460	101659894	chr3:101659492-101659869

chr3	101677555	101677657	chr3:101677593-101677611
chr3	101678215	101678659	chr3:101678237-101678648
chr3	101679175	101679339	chr3:101679201-101679575
chr3	101679340	101679564	chr3:101679201-101679575
chr3	101684875	101685268	chr3:101684480-101755683
chr3	101685275	101685388	chr3:101684480-101755683
chr3	101685425	101685629	chr3:101684480-101755683
chr3	101688500	101688606	chr3:101684480-101755683
chr3	101688970	101689134	chr3:101684480-101755683
chr3	101689140	101689218	chr3:101684480-101755683
chr3	101689900	101690028	chr3:101684480-101755683
chr3	101690060	101690161	chr3:101684480-101755683
chr3	101690180	101690258	chr3:101684480-101755683
chr3	101690790	101692390	chr3:101684480-101755683
chr3	101692680	101693513	chr3:101684480-101755683
chr3	101694145	101694216	chr3:101684480-101755683
chr3	101694330	101694403	chr3:101684480-101755683
chr3	101694485	101694568	chr3:101684480-101755683
chr3	101694930	101695059	chr3:101684480-101755683
chr3	101695065	101695151	chr3:101684480-101755683
chr3	101695195	101695987	chr3:101684480-101755683
chr3	101696055	101697111	chr3:101684480-101755683
chr3	101697115	101698092	chr3:101684480-101755683
chr3	101698095	101698399	chr3:101684480-101755683
chr3	101698420	101699932	chr3:101684480-101755683
chr3	101700395	101700565	chr3:101684480-101755683
chr3	101700595	101700663	chr3:101684480-101755683
chr3	101700975	101701718	chr3:101684480-101755683
chr3	101701890	101704225	chr3:101684480-101755683
chr3	101704230	101704589	chr3:101684480-101755683
chr3	101704625	101705287	chr3:101684480-101755683
chr3	101705465	101706452	chr3:101684480-101755683
chr3	101706760	101707048	chr3:101684480-101755683
chr3	101707070	101707309	chr3:101684480-101755683
chr3	101707335	101707767	chr3:101684480-101755683
chr3	101707895	101709392	chr3:101684480-101755683
chr3	101709410	101710010	chr3:101684480-101755683
chr3	101710035	101710974	chr3:101684480-101755683
chr3	101711280	101711631	chr3:101684480-101755683
chr3	101711650	101715708	chr3:101684480-101755683
chr3	101715730	101716443	chr3:101684480-101755683
chr3	101716565	101717049	chr3:101684480-101755683
chr3	101717230	101720612	chr3:101684480-101755683
chr3	101720890	101723048	chr3:101684480-101755683
chr3	101723160	101724119	chr3:101684480-101755683
chr3	101724180	101726596	chr3:101684480-101755683
chr3	101726865	101727059	chr3:101684480-101755683
chr3	101733215	101733513	chr3:101684480-101755683
chr3	101733520	101735102	chr3:101684480-101755683
chr3	101735105	101735294	chr3:101684480-101755683
chr3	101735460	101736687	chr3:101684480-101755683
chr3	101736695	101737419	chr3:101684480-101755683
chr3	101737485	101737714	chr3:101684480-101755683
chr3	101737765	101742662	chr3:101684480-101755683
chr3	101742665	101744116	chr3:101684480-101755683
chr3	101744120	101744286	chr3:101684480-101755683
chr3	101744410	101745191	chr3:101684480-101755683
chr3	101745210	101746877	chr3:101684480-101755683
chr3	101746885	101747769	chr3:101684480-101755683
chr3	101747775	101748273	chr3:101684480-101755683
chr3	101748310	101751653	chr3:101684480-101755683
chr3	101751660	101754764	chr3:101684480-101755683
chr3	101754770	101755318	chr3:101684480-101755683

chr3	101755320	101755713	chr3:101684480-101755683
chr3	104384695	104384776	chr3:104384693-104513265
chr3	104384785	104384894	chr3:104384693-104513265
chr3	104384955	104385024	chr3:104384693-104513265
chr3	104385030	104385128	chr3:104384693-104513265
chr3	104385250	104385318	chr3:104384693-104513265
chr3	104385825	104385973	chr3:104384693-104513265
chr3	104385980	104389313	chr3:104384693-104513265
chr3	104389540	104389618	chr3:104384693-104513265
chr3	104389640	104389955	chr3:104384693-104513265
chr3	104390250	104391149	chr3:104384693-104513265
chr3	104391155	104392158	chr3:104384693-104513265
chr3	104392165	104393659	chr3:104384693-104513265
chr3	104393665	104393741	chr3:104384693-104513265
chr3	104393780	104394273	chr3:104384693-104513265
chr3	104394370	104394443	chr3:104384693-104513265
chr3	104394575	104394674	chr3:104384693-104513265
chr3	104394705	104394780	chr3:104384693-104513265
chr3	104394940	104395088	chr3:104384693-104513265
chr3	104395140	104395349	chr3:104384693-104513265
chr3	104395370	104395491	chr3:104384693-104513265
chr3	104395530	104395642	chr3:104384693-104513265
chr3	104395655	104395739	chr3:104384693-104513265
chr3	104396055	104396134	chr3:104384693-104513265
chr3	104396255	104396522	chr3:104384693-104513265
chr3	104396530	104396895	chr3:104384693-104513265
chr3	104397035	104397127	chr3:104384693-104513265
chr3	104397190	104397309	chr3:104384693-104513265
chr3	104397355	104397735	chr3:104384693-104513265
chr3	104397765	104397858	chr3:104384693-104513265
chr3	104397925	104398249	chr3:104384693-104513265
chr3	104398250	104398415	chr3:104384693-104513265
chr3	104398465	104398616	chr3:104384693-104513265
chr3	104398660	104398807	chr3:104384693-104513265
chr3	104398850	104398927	chr3:104384693-104513265
chr3	104399330	104399447	chr3:104384693-104513265
chr3	104399540	104399614	chr3:104384693-104513265
chr3	104399690	104399796	chr3:104384693-104513265
chr3	104399905	104400003	chr3:104384693-104513265
chr3	104400060	104400354	chr3:104384693-104513265
chr3	104400640	104400795	chr3:104384693-104513265
chr3	104400805	104400918	chr3:104384693-104513265
chr3	104400965	104401039	chr3:104384693-104513265
chr3	104401040	104401113	chr3:104384693-104513265
chr3	104401115	104401299	chr3:104384693-104513265
chr3	104401470	104402316	chr3:104384693-104513265
chr3	104402630	104402983	chr3:104384693-104513265
chr3	104403275	104403503	chr3:104384693-104513265
chr3	104403510	104403615	chr3:104384693-104513265
chr3	104403670	104404383	chr3:104384693-104513265
chr3	104404390	104404534	chr3:104384693-104513265
chr3	104404560	104405042	chr3:104384693-104513265
chr3	104405045	104406475	chr3:104384693-104513265
chr3	104406755	104406906	chr3:104384693-104513265
chr3	104407190	104408454	chr3:104384693-104513265
chr3	104408465	104408819	chr3:104384693-104513265
chr3	104408830	104409798	chr3:104384693-104513265
chr3	104414370	104414444	chr3:104384693-104513265
chr3	104414690	104414761	chr3:104384693-104513265
chr3	104414810	104414916	chr3:104384693-104513265
chr3	104415060	104415140	chr3:104384693-104513265
chr3	104415400	104415473	chr3:104384693-104513265
chr3	104415550	104415633	chr3:104384693-104513265

chr3	104415690	104415775	chr3:104384693-104513265
chr3	104415780	104415864	chr3:104384693-104513265
chr3	104415920	104416344	chr3:104384693-104513265
chr3	104416415	104417345	chr3:104384693-104513265
chr3	104417415	104419631	chr3:104384693-104513265
chr3	104419635	104419811	chr3:104384693-104513265
chr3	104419850	104420017	chr3:104384693-104513265
chr3	104420125	104421232	chr3:104384693-104513265
chr3	104421240	104421357	chr3:104384693-104513265
chr3	104421405	104422988	chr3:104384693-104513265
chr3	104423350	104423809	chr3:104384693-104513265
chr3	104423820	104424880	chr3:104384693-104513265
chr3	104424885	104425096	chr3:104384693-104513265
chr3	104425110	104432583	chr3:104384693-104513265
chr3	104432585	104432732	chr3:104384693-104513265
chr3	104432740	104433978	chr3:104384693-104513265
chr3	104434070	104434143	chr3:104384693-104513265
chr3	104434225	104435389	chr3:104384693-104513265
chr3	104435390	104436236	chr3:104384693-104513265
chr3	104436495	104436868	chr3:104384693-104513265
chr3	104436875	104437114	chr3:104384693-104513265
chr3	104437135	104437234	chr3:104384693-104513265
chr3	104437235	104437986	chr3:104384693-104513265
chr3	104438010	104438856	chr3:104384693-104513265
chr3	104438880	104439069	chr3:104384693-104513265
chr3	104439070	104440925	chr3:104384693-104513265
chr3	104440940	104441180	chr3:104384693-104513265
chr3	104441195	104441275	chr3:104384693-104513265
chr3	104441770	104441872	chr3:104384693-104513265
chr3	104441915	104442000	chr3:104384693-104513265
chr3	104442015	104444714	chr3:104384693-104513265
chr3	104444725	104444801	chr3:104384693-104513265
chr3	104444910	104445029	chr3:104384693-104513265
chr3	104445045	104446136	chr3:104384693-104513265
chr3	104446165	104448029	chr3:104384693-104513265
chr3	104448045	104450745	chr3:104384693-104513265
chr3	104450750	104450915	chr3:104384693-104513265
chr3	104450920	104451279	chr3:104384693-104513265
chr3	104451285	104452573	chr3:104384693-104513265
chr3	104452580	104453151	chr3:104384693-104513265
chr3	104453475	104453717	chr3:104384693-104513265
chr3	104453940	104454142	chr3:104384693-104513265
chr3	104454145	104455645	chr3:104384693-104513265
chr3	104455655	104456688	chr3:104384693-104513265
chr3	104456700	104457544	chr3:104384693-104513265
chr3	104457555	104458301	chr3:104384693-104513265
chr3	104458365	104458633	chr3:104384693-104513265
chr3	104458640	104459033	chr3:104384693-104513265
chr3	104459085	104459302	chr3:104384693-104513265
chr3	104459365	104461008	chr3:104384693-104513265
chr3	104461130	104461332	chr3:104384693-104513265
chr3	104461335	104462161	chr3:104384693-104513265
chr3	104462420	104462811	chr3:104384693-104513265
chr3	104462970	104463101	chr3:104384693-104513265
chr3	104463105	104463690	chr3:104384693-104513265
chr3	104463695	104463929	chr3:104384693-104513265
chr3	104463935	104464291	chr3:104384693-104513265
chr3	104464295	104464554	chr3:104384693-104513265
chr3	104464555	104467105	chr3:104384693-104513265
chr3	104467180	104468444	chr3:104384693-104513265
chr3	104468480	104473548	chr3:104384693-104513265
chr3	104473835	104474390	chr3:104384693-104513265
chr3	104474405	104474973	chr3:104384693-104513265

chr3	104474980	104476330	chr3:104384693-104513265
chr3	104476340	104476933	chr3:104384693-104513265
chr3	104476945	104477947	chr3:104384693-104513265
chr3	104477955	104478560	chr3:104384693-104513265
chr3	104478565	104478794	chr3:104384693-104513265
chr3	104478815	104479164	chr3:104384693-104513265
chr3	104479165	104479374	chr3:104384693-104513265
chr3	104479385	104479915	chr3:104384693-104513265
chr3	104479985	104480936	chr3:104384693-104513265
chr3	104480945	104481217	chr3:104384693-104513265
chr3	104481230	104481436	chr3:104384693-104513265
chr3	104481500	104482203	chr3:104384693-104513265
chr3	104482215	104483312	chr3:104384693-104513265
chr3	104483315	104483569	chr3:104384693-104513265
chr3	104483575	104484933	chr3:104384693-104513265
chr3	104484935	104485018	chr3:104384693-104513265
chr3	104485020	104485658	chr3:104384693-104513265
chr3	104485665	104485780	chr3:104384693-104513265
chr3	104485785	104486391	chr3:104384693-104513265
chr3	104486395	104486690	chr3:104384693-104513265
chr3	104486765	104487367	chr3:104384693-104513265
chr3	104487370	104488384	chr3:104384693-104513265
chr3	104488640	104489954	chr3:104384693-104513265
chr3	104489965	104490064	chr3:104384693-104513265
chr3	104490065	104492370	chr3:104384693-104513265
chr3	104492715	104493465	chr3:104384693-104513265
chr3	104493730	104494008	chr3:104384693-104513265
chr3	104494065	104494261	chr3:104384693-104513265
chr3	104494405	104494916	chr3:104384693-104513265
chr3	104495225	104496553	chr3:104384693-104513265
chr3	104496895	104497830	chr3:104384693-104513265
chr3	104497890	104498185	chr3:104384693-104513265
chr3	104498410	104498632	chr3:104384693-104513265
chr3	104498670	104499739	chr3:104384693-104513265
chr3	104499740	104501239	chr3:104384693-104513265
chr3	104501240	104501960	chr3:104384693-104513265
chr3	104502210	104503090	chr3:104384693-104513265
chr3	104503100	104503777	chr3:104384693-104513265
chr3	104503825	104504718	chr3:104384693-104513265
chr3	104504730	104504981	chr3:104384693-104513265
chr3	104505045	104505605	chr3:104384693-104513265
chr3	104505610	104505726	chr3:104384693-104513265
chr3	104506030	104507176	chr3:104384693-104513265
chr3	104507180	104507683	chr3:104384693-104513265
chr3	104507985	104508870	chr3:104384693-104513265
chr3	104508875	104509375	chr3:104384693-104513265
chr3	104509455	104510905	chr3:104384693-104513265
chr3	104510920	104511196	chr3:104384693-104513265
chr3	104511210	104511840	chr3:104384693-104513265
chr3	104512135	104512605	chr3:104384693-104513265
chr3	104512610	104513304	chr3:104384693-104513265
chr3	104536120	104536465	chr3:104536127-104536457
chr3	104546365	104546448	chr3:104546407-104546424
chr3	104548470	104548585	chr3:104548502-104548557
chr3	104574175	104574290	chr3:104574202-104574257
chr3	125822370	125822728	chr3:125822403-125822689
chr3	125824535	125824790	chr3:125824568-125824768
chr3	125825960	125826112	chr3:125825983-125826089
chr3	125828755	125828974	chr3:125828786-125828952
chr3	125831590	125831743	chr3:125831624-125831723
chr3	125833365	125833519	chr3:125833399-125833499
chr3	125836825	125836959	chr3:125836847-125836941
chr3	125843185	125843324	chr3:125843206-125843294

chr3	125844435	125844598	chr3:125844458-125844564
chr3	125849040	125849172	chr3:125849065-125849136
chr3	125850200	125850421	chr3:125850226-125850377
chr3	125854355	125854533	chr3:125854377-125854505
chr3	125855585	125855768	chr3:125855606-125855726
chr3	125856630	125856836	chr3:125856655-125856803
chr3	125865650	125865793	chr3:125865677-125865769
chr3	125869215	125869398	chr3:125869248-125869374
chr3	125872260	125872434	chr3:125872286-125872424
chr3	125873375	125873515	chr3:125873396-125873486
chr3	125874215	125874370	chr3:125874244-125874346
chr3	125876160	125876363	chr3:125876185-125876351
chr3	125877225	125877507	chr3:125877247-125877482
chr3	125879670	125879891	chr3:125879695-125879845
chr3	125899290	125899678	chr3:125899318-125899645
chr3	125899780	125900059	chr3:125899804-125900029
chr3	164168848	164169350	chr3:164168871-164169323
chr3	164326748	164326898	chr3:164326779-164326856
chr3	164337088	164337219	chr3:164337112-164337228
chr3	164378233	164378345	chr3:164378273-164378298
chr3	164388658	164389035	chr3:164388665-164389019
chr3	164401583	164401713	chr3:164401604-164401689
chr3	164402918	164403094	chr3:164402940-164403072
chr3	164403788	164403896	chr3:164403811-164403862
chr3	164431853	164432285	chr3:164431888-164432270
chr3	164434078	164434181	chr3:164434114-164434152
chr3	164435333	164435608	chr3:164435358-164435580
chr3	164448633	164448854	chr3:164448659-164448828
chr3	164452148	164452250	chr3:164452188-164452217
chr3	164528038	164528345	chr3:164528066-164590725
chr3	164528463	164528894	chr3:164528066-164590725
chr3	164529133	164529922	chr3:164528066-164590725
chr3	164530263	164531664	chr3:164528066-164590725
chr3	164531688	164531989	chr3:164528066-164590725
chr3	164532043	164532465	chr3:164528066-164590725
chr3	164532508	164532607	chr3:164528066-164590725
chr3	164532673	164532769	chr3:164528066-164590725
chr3	164533658	164533745	chr3:164528066-164590725
chr3	164533818	164533903	chr3:164528066-164590725
chr3	164534008	164534089	chr3:164528066-164590725
chr3	164534123	164534200	chr3:164528066-164590725
chr3	164534223	164534352	chr3:164528066-164590725
chr3	164534403	164534548	chr3:164528066-164590725
chr3	164534568	164534641	chr3:164528066-164590725
chr3	164534843	164534967	chr3:164528066-164590725
chr3	164535048	164535154	chr3:164528066-164590725
chr3	164535393	164535460	chr3:164528066-164590725
chr3	164535768	164535869	chr3:164528066-164590725
chr3	164535903	164535978	chr3:164528066-164590725
chr3	164536038	164536176	chr3:164528066-164590725
chr3	164536208	164536423	chr3:164528066-164590725
chr3	164537348	164537566	chr3:164528066-164590725
chr3	164537623	164538599	chr3:164528066-164590725
chr3	164538898	164539640	chr3:164528066-164590725
chr3	164539678	164540495	chr3:164528066-164590725
chr3	164540498	164540961	chr3:164528066-164590725
chr3	164540968	164541216	chr3:164528066-164590725
chr3	164541243	164541348	chr3:164528066-164590725
chr3	164541353	164542812	chr3:164528066-164590725
chr3	164542848	164542945	chr3:164528066-164590725
chr3	164542963	164543098	chr3:164528066-164590725
chr3	164543113	164545356	chr3:164528066-164590725
chr3	164545368	164547602	chr3:164528066-164590725

chr3	164547603	164548396	chr3:164528066-164590725
chr3	164548553	164549389	chr3:164528066-164590725
chr3	164549433	164549521	chr3:164528066-164590725
chr3	164549533	164549610	chr3:164528066-164590725
chr3	164549738	164550690	chr3:164528066-164590725
chr3	164550698	164552769	chr3:164528066-164590725
chr3	164552778	164552963	chr3:164528066-164590725
chr3	164553253	164553348	chr3:164528066-164590725
chr3	164553353	164553655	chr3:164528066-164590725
chr3	164553663	164554931	chr3:164528066-164590725
chr3	164554938	164557586	chr3:164528066-164590725
chr3	164557743	164557825	chr3:164528066-164590725
chr3	164557878	164558673	chr3:164528066-164590725
chr3	164558678	164559129	chr3:164528066-164590725
chr3	164559183	164559324	chr3:164528066-164590725
chr3	164559338	164560068	chr3:164528066-164590725
chr3	164560088	164560213	chr3:164528066-164590725
chr3	164560233	164560771	chr3:164528066-164590725
chr3	164560818	164561637	chr3:164528066-164590725
chr3	164561668	164561959	chr3:164528066-164590725
chr3	164561963	164562065	chr3:164528066-164590725
chr3	164562068	164562688	chr3:164528066-164590725
chr3	164562693	164566936	chr3:164528066-164590725
chr3	164566943	164567457	chr3:164528066-164590725
chr3	164567748	164567847	chr3:164528066-164590725
chr3	164567848	164567915	chr3:164528066-164590725
chr3	164568183	164568837	chr3:164528066-164590725
chr3	164568873	164569416	chr3:164528066-164590725
chr3	164569428	164570198	chr3:164528066-164590725
chr3	164570208	164572174	chr3:164528066-164590725
chr3	164572493	164572607	chr3:164528066-164590725
chr3	164572613	164572942	chr3:164528066-164590725
chr3	164572953	164573071	chr3:164528066-164590725
chr3	164573078	164573573	chr3:164528066-164590725
chr3	164573853	164574316	chr3:164528066-164590725
chr3	164574318	164576090	chr3:164528066-164590725
chr3	164576118	164578454	chr3:164528066-164590725
chr3	164578648	164579100	chr3:164528066-164590725
chr3	164579103	164580931	chr3:164528066-164590725
chr3	164580948	164581160	chr3:164528066-164590725
chr3	164581168	164581276	chr3:164528066-164590725
chr3	164581358	164581475	chr3:164528066-164590725
chr3	164581483	164582661	chr3:164528066-164590725
chr3	164582693	164582853	chr3:164528066-164590725
chr3	164583163	164583276	chr3:164528066-164590725
chr3	164583283	164583437	chr3:164528066-164590725
chr3	164583443	164583714	chr3:164528066-164590725
chr3	164584015	164584563	chr3:164528066-164590725
chr3	164584840	164585018	chr3:164528066-164590725
chr3	164585025	164585354	chr3:164528066-164590725
chr3	164585370	164586749	chr3:164528066-164590725
chr3	164587055	164587425	chr3:164528066-164590725
chr3	164587735	164588212	chr3:164528066-164590725
chr3	164588520	164590168	chr3:164528066-164590725
chr3	164590175	164590493	chr3:164528066-164590725
chr3	164597050	164597152	chr3:164597071-164597134
chr3	164616175	164616285	chr3:164616210-164616259
chr3	164621365	164621470	chr3:164621396-164621457
chr3	165998158	166000890	chr3:165998188-166019936
chr3	166000903	166002089	chr3:165998188-166019936
chr3	166002118	166002234	chr3:165998188-166019936
chr3	166002238	166002425	chr3:165998188-166019936
chr3	166002448	166003490	chr3:165998188-166019936

chr3	166003588	166004292	chr3:165998188-166019936
chr3	166004603	166005311	chr3:165998188-166019936
chr3	166005318	166006183	chr3:165998188-166019936
chr3	166006203	166008711	chr3:165998188-166019936
chr3	166008733	166008906	chr3:165998188-166019936
chr3	166008908	166011279	chr3:165998188-166019936
chr3	166011298	166012127	chr3:165998188-166019936
chr3	166012163	166015171	chr3:165998188-166019936
chr3	166015178	166015926	chr3:165998188-166019936
chr3	166015943	166016264	chr3:165998188-166019936
chr3	166016273	166016420	chr3:165998188-166019936
chr3	166016428	166017310	chr3:165998188-166019936
chr3	166017573	166019464	chr3:165998188-166019936
chr3	166019483	166019801	chr3:165998188-166019936
chr3	166019828	166019963	chr3:165998188-166019936
chr3	166020083	166020637	chr3:166020105-166226563
chr3	166020653	166021954	chr3:166020105-166226563
chr3	166021958	166022952	chr3:166020105-166226563
chr3	166022968	166023617	chr3:166020105-166226563
chr3	166023628	166023773	chr3:166020105-166226563
chr3	166024053	166024427	chr3:166020105-166226563
chr3	166024793	166025023	chr3:166020105-166226563
chr3	166025033	166027369	chr3:166020105-166226563
chr3	166027373	166027556	chr3:166020105-166226563
chr3	166027608	166027715	chr3:166020105-166226563
chr3	166027793	166027910	chr3:166020105-166226563
chr3	166028233	166028870	chr3:166020105-166226563
chr3	166028983	166029091	chr3:166020105-166226563
chr3	166029238	166029648	chr3:166020105-166226563
chr3	166029653	166030010	chr3:166020105-166226563
chr3	166030018	166032567	chr3:166020105-166226563
chr3	166032623	166033891	chr3:166020105-166226563
chr3	166033898	166035399	chr3:166020105-166226563
chr3	166035453	166035697	chr3:166020105-166226563
chr3	166035698	166036498	chr3:166020105-166226563
chr3	166036868	166037012	chr3:166020105-166226563
chr3	166037278	166037742	chr3:166020105-166226563
chr3	166038063	166038926	chr3:166020105-166226563
chr3	166038933	166039470	chr3:166020105-166226563
chr3	166039488	166041769	chr3:166020105-166226563
chr3	166041823	166041904	chr3:166020105-166226563
chr3	166041953	166042038	chr3:166020105-166226563
chr3	166042158	166042315	chr3:166020105-166226563
chr3	166042368	166042527	chr3:166020105-166226563
chr3	166042593	166042665	chr3:166020105-166226563
chr3	166042708	166042783	chr3:166020105-166226563
chr3	166042853	166042979	chr3:166020105-166226563
chr3	166042983	166043052	chr3:166020105-166226563
chr3	166043268	166043374	chr3:166020105-166226563
chr3	166043383	166043571	chr3:166020105-166226563
chr3	166043573	166043782	chr3:166020105-166226563
chr3	166043883	166044065	chr3:166020105-166226563
chr3	166044323	166044398	chr3:166020105-166226563
chr3	166044403	166045061	chr3:166020105-166226563
chr3	166045068	166045162	chr3:166020105-166226563
chr3	166045363	166045447	chr3:166020105-166226563
chr3	166045533	166045613	chr3:166020105-166226563
chr3	166045638	166045874	chr3:166020105-166226563
chr3	166046308	166046746	chr3:166020105-166226563
chr3	166046753	166046842	chr3:166020105-166226563
chr3	166046903	166047001	chr3:166020105-166226563
chr3	166047238	166047802	chr3:166020105-166226563
chr3	166047813	166049452	chr3:166020105-166226563

chr3	166049453	166049526	chr3:166020105-166226563
chr3	166049583	166052806	chr3:166020105-166226563
chr3	166052813	166053795	chr3:166020105-166226563
chr3	166053798	166054317	chr3:166020105-166226563
chr3	166054318	166054534	chr3:166020105-166226563
chr3	166054543	166054629	chr3:166020105-166226563
chr3	166054913	166055195	chr3:166020105-166226563
chr3	166055263	166055514	chr3:166020105-166226563
chr3	166055523	166056006	chr3:166020105-166226563
chr3	166056278	166057753	chr3:166020105-166226563
chr3	166057758	166058338	chr3:166020105-166226563
chr3	166058343	166061291	chr3:166020105-166226563
chr3	166061668	166061847	chr3:166020105-166226563
chr3	166061948	166062445	chr3:166020105-166226563
chr3	166062453	166062647	chr3:166020105-166226563
chr3	166062853	166063354	chr3:166020105-166226563
chr3	166063363	166063456	chr3:166020105-166226563
chr3	166063468	166063550	chr3:166020105-166226563
chr3	166063553	166063873	chr3:166020105-166226563
chr3	166063883	166064392	chr3:166020105-166226563
chr3	166064438	166064616	chr3:166020105-166226563
chr3	166064628	166064700	chr3:166020105-166226563
chr3	166064713	166064849	chr3:166020105-166226563
chr3	166064903	166065166	chr3:166020105-166226563
chr3	166065228	166065305	chr3:166020105-166226563
chr3	166065308	166065532	chr3:166020105-166226563
chr3	166065598	166065836	chr3:166020105-166226563
chr3	166065863	166066052	chr3:166020105-166226563
chr3	166066068	166066442	chr3:166020105-166226563
chr3	166066463	166066545	chr3:166020105-166226563
chr3	166066818	166066892	chr3:166020105-166226563
chr3	166067408	166067619	chr3:166020105-166226563
chr3	166067983	166069529	chr3:166020105-166226563
chr3	166069548	166069715	chr3:166020105-166226563
chr3	166069758	166069939	chr3:166020105-166226563
chr3	166069968	166071092	chr3:166020105-166226563
chr3	166071293	166071379	chr3:166020105-166226563
chr3	166071383	166071479	chr3:166020105-166226563
chr3	166071648	166071731	chr3:166020105-166226563
chr3	166071793	166071876	chr3:166020105-166226563
chr3	166072103	166072253	chr3:166020105-166226563
chr3	166072258	166072416	chr3:166020105-166226563
chr3	166074403	166075706	chr3:166020105-166226563
chr3	166075728	166075941	chr3:166020105-166226563
chr3	166076123	166076236	chr3:166020105-166226563
chr3	166076243	166079412	chr3:166020105-166226563
chr3	166079438	166079576	chr3:166020105-166226563
chr3	166079648	166079732	chr3:166020105-166226563
chr3	166079868	166081200	chr3:166020105-166226563
chr3	166082588	166082684	chr3:166020105-166226563
chr3	166082973	166084279	chr3:166020105-166226563
chr3	166084368	166084494	chr3:166020105-166226563
chr3	166084508	166084696	chr3:166020105-166226563
chr3	166084698	166084777	chr3:166020105-166226563
chr3	166084783	166085713	chr3:166020105-166226563
chr3	166085718	166087943	chr3:166020105-166226563
chr3	166087953	166088523	chr3:166020105-166226563
chr3	166088528	166089338	chr3:166020105-166226563
chr3	166089483	166089839	chr3:166020105-166226563
chr3	166089848	166089926	chr3:166020105-166226563
chr3	166089988	166090927	chr3:166020105-166226563
chr3	166090948	166091046	chr3:166020105-166226563
chr3	166091048	166091959	chr3:166020105-166226563

chr3	166091973	166092495	chr3:166020105-166226563
chr3	166092523	166092742	chr3:166020105-166226563
chr3	166092743	166094651	chr3:166020105-166226563
chr3	166097243	166097317	chr3:166020105-166226563
chr3	166097718	166099760	chr3:166020105-166226563
chr3	166100088	166100194	chr3:166020105-166226563
chr3	166100613	166100969	chr3:166020105-166226563
chr3	166100978	166101254	chr3:166020105-166226563
chr3	166101563	166104548	chr3:166020105-166226563
chr3	166104853	166105467	chr3:166020105-166226563
chr3	166105468	166106933	chr3:166020105-166226563
chr3	166107193	166107262	chr3:166020105-166226563
chr3	166107548	166108461	chr3:166020105-166226563
chr3	166108713	166108792	chr3:166020105-166226563
chr3	166108798	166108873	chr3:166020105-166226563
chr3	166108968	166109236	chr3:166020105-166226563
chr3	166109273	166109347	chr3:166020105-166226563
chr3	166109488	166109582	chr3:166020105-166226563
chr3	166109638	166109726	chr3:166020105-166226563
chr3	166109733	166109883	chr3:166020105-166226563
chr3	166109918	166110004	chr3:166020105-166226563
chr3	166110048	166110142	chr3:166020105-166226563
chr3	166110178	166110308	chr3:166020105-166226563
chr3	166110418	166110526	chr3:166020105-166226563
chr3	166110533	166110650	chr3:166020105-166226563
chr3	166110673	166110777	chr3:166020105-166226563
chr3	166110843	166111021	chr3:166020105-166226563
chr3	166111388	166112107	chr3:166020105-166226563
chr3	166112143	166112456	chr3:166020105-166226563
chr3	166112498	166112596	chr3:166020105-166226563
chr3	166112613	166112953	chr3:166020105-166226563
chr3	166113033	166113102	chr3:166020105-166226563
chr3	166113938	166114012	chr3:166020105-166226563
chr3	166114818	166114895	chr3:166020105-166226563
chr3	166115488	166116138	chr3:166020105-166226563
chr3	166116443	166117410	chr3:166020105-166226563
chr3	166117773	166120405	chr3:166020105-166226563
chr3	166120408	166120591	chr3:166020105-166226563
chr3	166120598	166120804	chr3:166020105-166226563
chr3	166120893	166121230	chr3:166020105-166226563
chr3	166121233	166121705	chr3:166020105-166226563
chr3	166121708	166123023	chr3:166020105-166226563
chr3	166123033	166123288	chr3:166020105-166226563
chr3	166123308	166123520	chr3:166020105-166226563
chr3	166123533	166123963	chr3:166020105-166226563
chr3	166123968	166124051	chr3:166020105-166226563
chr3	166124083	166124283	chr3:166020105-166226563
chr3	166124318	166124605	chr3:166020105-166226563
chr3	166124643	166125177	chr3:166020105-166226563
chr3	166125198	166125751	chr3:166020105-166226563
chr3	166125753	166128076	chr3:166020105-166226563
chr3	166128328	166128702	chr3:166020105-166226563
chr3	166129058	166129220	chr3:166020105-166226563
chr3	166129228	166129861	chr3:166020105-166226563
chr3	166129983	166131311	chr3:166020105-166226563
chr3	166131333	166131767	chr3:166020105-166226563
chr3	166131783	166134079	chr3:166020105-166226563
chr3	166134088	166134414	chr3:166020105-166226563
chr3	166134488	166134681	chr3:166020105-166226563
chr3	166134703	166135250	chr3:166020105-166226563
chr3	166135258	166135512	chr3:166020105-166226563
chr3	166135513	166135716	chr3:166020105-166226563
chr3	166135718	166136133	chr3:166020105-166226563

chr3	166136148	166136294	chr3:166020105-166226563
chr3	166136658	166136747	chr3:166020105-166226563
chr3	166137168	166137262	chr3:166020105-166226563
chr3	166137428	166137498	chr3:166020105-166226563
chr3	166137533	166137629	chr3:166020105-166226563
chr3	166138128	166138244	chr3:166020105-166226563
chr3	166138248	166138333	chr3:166020105-166226563
chr3	166138368	166138445	chr3:166020105-166226563
chr3	166138598	166138800	chr3:166020105-166226563
chr3	166138833	166138977	chr3:166020105-166226563
chr3	166139198	166139273	chr3:166020105-166226563
chr3	166139328	166139452	chr3:166020105-166226563
chr3	166139763	166140677	chr3:166020105-166226563
chr3	166140743	166141592	chr3:166020105-166226563
chr3	166141788	166141904	chr3:166020105-166226563
chr3	166141998	166142082	chr3:166020105-166226563
chr3	166142683	166142944	chr3:166020105-166226563
chr3	166142968	166143223	chr3:166020105-166226563
chr3	166143233	166143971	chr3:166020105-166226563
chr3	166144008	166144742	chr3:166020105-166226563
chr3	166144798	166145561	chr3:166020105-166226563
chr3	166146073	166146190	chr3:166020105-166226563
chr3	166146208	166146688	chr3:166020105-166226563
chr3	166146728	166147432	chr3:166020105-166226563
chr3	166147443	166147558	chr3:166020105-166226563
chr3	166147698	166147953	chr3:166020105-166226563
chr3	166147958	166148071	chr3:166020105-166226563
chr3	166148078	166148518	chr3:166020105-166226563
chr3	166148593	166148670	chr3:166020105-166226563
chr3	166148708	166148858	chr3:166020105-166226563
chr3	166148868	166148947	chr3:166020105-166226563
chr3	166148968	166149137	chr3:166020105-166226563
chr3	166149138	166149319	chr3:166020105-166226563
chr3	166149388	166149491	chr3:166020105-166226563
chr3	166149653	166149733	chr3:166020105-166226563
chr3	166149933	166150034	chr3:166020105-166226563
chr3	166150088	166150173	chr3:166020105-166226563
chr3	166150283	166150524	chr3:166020105-166226563
chr3	166150593	166150690	chr3:166020105-166226563
chr3	166150743	166151120	chr3:166020105-166226563
chr3	166151123	166152036	chr3:166020105-166226563
chr3	166152043	166152906	chr3:166020105-166226563
chr3	166153168	166153979	chr3:166020105-166226563
chr3	166153988	166154204	chr3:166020105-166226563
chr3	166154333	166154833	chr3:166020105-166226563
chr3	166154838	166156167	chr3:166020105-166226563
chr3	166156193	166156669	chr3:166020105-166226563
chr3	166156678	166158376	chr3:166020105-166226563
chr3	166158388	166158553	chr3:166020105-166226563
chr3	166158648	166158764	chr3:166020105-166226563
chr3	166158813	166158957	chr3:166020105-166226563
chr3	166159138	166159481	chr3:166020105-166226563
chr3	166159523	166160533	chr3:166020105-166226563
chr3	166160813	166161209	chr3:166020105-166226563
chr3	166161243	166163885	chr3:166020105-166226563
chr3	166163888	166163991	chr3:166020105-166226563
chr3	166163993	166164078	chr3:166020105-166226563
chr3	166164213	166164292	chr3:166020105-166226563
chr3	166164298	166164426	chr3:166020105-166226563
chr3	166164433	166164498	chr3:166020105-166226563
chr3	166164528	166164598	chr3:166020105-166226563
chr3	166164613	166165439	chr3:166020105-166226563
chr3	166165448	166165669	chr3:166020105-166226563

chr3	166165688	166166218	chr3:166020105-166226563
chr3	166166273	166166887	chr3:166020105-166226563
chr3	166166983	166167080	chr3:166020105-166226563
chr3	166167208	166167307	chr3:166020105-166226563
chr3	166167398	166169403	chr3:166020105-166226563
chr3	166169708	166171303	chr3:166020105-166226563
chr3	166171323	166171936	chr3:166020105-166226563
chr3	166171998	166172229	chr3:166020105-166226563
chr3	166172233	166173260	chr3:166020105-166226563
chr3	166173278	166173455	chr3:166020105-166226563
chr3	166173478	166173583	chr3:166020105-166226563
chr3	166173608	166173699	chr3:166020105-166226563
chr3	166173738	166175060	chr3:166020105-166226563
chr3	166175203	166177489	chr3:166020105-166226563
chr3	166177778	166178732	chr3:166020105-166226563
chr3	166178798	166179380	chr3:166020105-166226563
chr3	166179388	166182329	chr3:166020105-166226563
chr3	166182343	166182805	chr3:166020105-166226563
chr3	166182808	166184219	chr3:166020105-166226563
chr3	166184348	166186496	chr3:166020105-166226563
chr3	166186533	166186705	chr3:166020105-166226563
chr3	166186733	166187020	chr3:166020105-166226563
chr3	166187028	166190095	chr3:166020105-166226563
chr3	166190128	166190418	chr3:166020105-166226563
chr3	166190423	166190796	chr3:166020105-166226563
chr3	166190803	166191086	chr3:166020105-166226563
chr3	166191098	166191657	chr3:166020105-166226563
chr3	166191893	166193061	chr3:166020105-166226563
chr3	166193163	166193266	chr3:166020105-166226563
chr3	166193268	166193349	chr3:166020105-166226563
chr3	166193353	166194170	chr3:166020105-166226563
chr3	166194348	166195110	chr3:166020105-166226563
chr3	166195113	166195336	chr3:166020105-166226563
chr3	166195353	166195664	chr3:166020105-166226563
chr3	166195693	166196882	chr3:166020105-166226563
chr3	166196918	166197077	chr3:166020105-166226563
chr3	166197128	166197320	chr3:166020105-166226563
chr3	166197508	166197604	chr3:166020105-166226563
chr3	166197623	166197972	chr3:166020105-166226563
chr3	166198043	166198148	chr3:166020105-166226563
chr3	166198158	166198373	chr3:166020105-166226563
chr3	166198398	166198616	chr3:166020105-166226563
chr3	166198648	166198915	chr3:166020105-166226563
chr3	166198973	166199061	chr3:166020105-166226563
chr3	166199118	166199235	chr3:166020105-166226563
chr3	166199238	166199302	chr3:166020105-166226563
chr3	166199333	166199571	chr3:166020105-166226563
chr3	166199573	166199681	chr3:166020105-166226563
chr3	166199718	166199903	chr3:166020105-166226563
chr3	166199908	166199973	chr3:166020105-166226563
chr3	166200213	166200361	chr3:166020105-166226563
chr3	166200548	166200621	chr3:166020105-166226563
chr3	166200663	166200893	chr3:166020105-166226563
chr3	166201213	166201343	chr3:166020105-166226563
chr3	166201348	166201637	chr3:166020105-166226563
chr3	166201668	166201822	chr3:166020105-166226563
chr3	166201828	166201927	chr3:166020105-166226563
chr3	166201953	166202094	chr3:166020105-166226563
chr3	166202103	166202507	chr3:166020105-166226563
chr3	166202528	166203918	chr3:166020105-166226563
chr3	166203968	166204526	chr3:166020105-166226563
chr3	166204533	166204706	chr3:166020105-166226563
chr3	166204748	166205861	chr3:166020105-166226563

chr3	166205913	166206059	chr3:166020105-166226563
chr3	166206068	166207648	chr3:166020105-166226563
chr3	166207808	166208064	chr3:166020105-166226563
chr3	166208393	166209627	chr3:166020105-166226563
chr3	166209663	166210322	chr3:166020105-166226563
chr3	166210368	166211968	chr3:166020105-166226563
chr3	166211973	166212227	chr3:166020105-166226563
chr3	166212248	166213210	chr3:166020105-166226563
chr3	166213508	166214700	chr3:166020105-166226563
chr3	166214738	166216550	chr3:166020105-166226563
chr3	166216593	166217080	chr3:166020105-166226563
chr3	166217098	166217173	chr3:166020105-166226563
chr3	166217178	166217348	chr3:166020105-166226563
chr3	166217558	166217659	chr3:166020105-166226563
chr3	166217913	166217986	chr3:166020105-166226563
chr3	166218028	166218101	chr3:166020105-166226563
chr3	166218113	166218197	chr3:166020105-166226563
chr3	166218203	166218272	chr3:166020105-166226563
chr3	166218818	166218886	chr3:166020105-166226563
chr3	166219463	166219541	chr3:166020105-166226563
chr3	166219658	166219766	chr3:166020105-166226563
chr3	166220508	166220579	chr3:166020105-166226563
chr3	166221593	166221666	chr3:166020105-166226563
chr3	166222443	166222508	chr3:166020105-166226563
chr3	166223213	166223288	chr3:166020105-166226563
chr3	166223643	166223833	chr3:166020105-166226563
chr3	166224663	166224779	chr3:166020105-166226563
chr3	166224918	166225616	chr3:166020105-166226563
chr3	166225628	166226325	chr3:166020105-166226563
chr3	166226328	166226604	chr3:166020105-166226563
chr3	166226673	166226751	chr3:166226725-166281774
chr3	166226783	166227232	chr3:166226725-166281774
chr3	166227233	166228105	chr3:166226725-166281774
chr3	166228113	166228637	chr3:166226725-166281774
chr3	166228638	166228799	chr3:166226725-166281774
chr3	166228818	166229135	chr3:166226725-166281774
chr3	166229353	166229436	chr3:166226725-166281774
chr3	166229478	166229628	chr3:166226725-166281774
chr3	166229763	166229908	chr3:166226725-166281774
chr3	166230123	166230192	chr3:166226725-166281774
chr3	166231283	166231425	chr3:166226725-166281774
chr3	166232443	166232512	chr3:166226725-166281774
chr3	166233178	166233253	chr3:166226725-166281774
chr3	166233263	166233382	chr3:166226725-166281774
chr3	166234533	166234605	chr3:166226725-166281774
chr3	166234663	166234899	chr3:166226725-166281774
chr3	166234903	166235488	chr3:166226725-166281774
chr3	166235793	166236346	chr3:166226725-166281774
chr3	166237468	166237554	chr3:166226725-166281774
chr3	166237588	166237846	chr3:166226725-166281774
chr3	166237863	166238061	chr3:166226725-166281774
chr3	166238063	166238213	chr3:166226725-166281774
chr3	166238258	166238353	chr3:166226725-166281774
chr3	166238358	166238597	chr3:166226725-166281774
chr3	166238623	166238720	chr3:166226725-166281774
chr3	166238723	166238874	chr3:166226725-166281774
chr3	166238918	166239329	chr3:166226725-166281774
chr3	166239393	166240188	chr3:166226725-166281774
chr3	166240193	166240335	chr3:166226725-166281774
chr3	166240348	166240902	chr3:166226725-166281774
chr3	166240933	166241375	chr3:166226725-166281774
chr3	166241383	166244436	chr3:166226725-166281774
chr3	166244443	166245429	chr3:166226725-166281774

chr3	166245453	166246508	chr3:166226725-166281774
chr3	166246518	166246968	chr3:166226725-166281774
chr3	166247173	166248731	chr3:166226725-166281774
chr3	166248818	166248892	chr3:166226725-166281774
chr3	166248998	166249305	chr3:166226725-166281774
chr3	166249313	166249466	chr3:166226725-166281774
chr3	166249633	166249718	chr3:166226725-166281774
chr3	166249773	166249855	chr3:166226725-166281774
chr3	166249873	166250059	chr3:166226725-166281774
chr3	166250268	166250346	chr3:166226725-166281774
chr3	166250698	166250805	chr3:166226725-166281774
chr3	166250958	166251305	chr3:166226725-166281774
chr3	166251313	166252333	chr3:166226725-166281774
chr3	166252358	166252663	chr3:166226725-166281774
chr3	166252758	166254215	chr3:166226725-166281774
chr3	166254473	166254908	chr3:166226725-166281774
chr3	166254953	166255154	chr3:166226725-166281774
chr3	166255173	166255539	chr3:166226725-166281774
chr3	166255853	166256390	chr3:166226725-166281774
chr3	166256393	166256623	chr3:166226725-166281774
chr3	166256643	166256723	chr3:166226725-166281774
chr3	166256838	166256943	chr3:166226725-166281774
chr3	166257348	166257913	chr3:166226725-166281774
chr3	166257923	166258221	chr3:166226725-166281774
chr3	166258248	166258363	chr3:166226725-166281774
chr3	166258413	166259212	chr3:166226725-166281774
chr3	166259238	166259638	chr3:166226725-166281774
chr3	166259643	166260064	chr3:166226725-166281774
chr3	166260113	166260347	chr3:166226725-166281774
chr3	166260523	166261632	chr3:166226725-166281774
chr3	166261933	166262028	chr3:166226725-166281774
chr3	166262043	166262214	chr3:166226725-166281774
chr3	166262218	166262966	chr3:166226725-166281774
chr3	166262968	166263362	chr3:166226725-166281774
chr3	166263428	166264394	chr3:166226725-166281774
chr3	166264683	166264959	chr3:166226725-166281774
chr3	166264973	166265300	chr3:166226725-166281774
chr3	166265513	166265587	chr3:166226725-166281774
chr3	166265593	166265709	chr3:166226725-166281774
chr3	166265718	166265823	chr3:166226725-166281774
chr3	166265908	166266141	chr3:166226725-166281774
chr3	166266218	166266391	chr3:166226725-166281774
chr3	166266418	166266488	chr3:166226725-166281774
chr3	166266498	166266581	chr3:166226725-166281774
chr3	166266663	166266951	chr3:166226725-166281774
chr3	166266963	166267090	chr3:166226725-166281774
chr3	166267163	166267366	chr3:166226725-166281774
chr3	166267368	166267864	chr3:166226725-166281774
chr3	166268018	166268198	chr3:166226725-166281774
chr3	166268373	166269414	chr3:166226725-166281774
chr3	166269418	166270058	chr3:166226725-166281774
chr3	166270348	166271438	chr3:166226725-166281774
chr3	166271473	166271680	chr3:166226725-166281774
chr3	166271683	166271829	chr3:166226725-166281774
chr3	166271893	166272975	chr3:166226725-166281774
chr3	166272978	166273230	chr3:166226725-166281774
chr3	166273368	166273653	chr3:166226725-166281774
chr3	166273848	166274027	chr3:166226725-166281774
chr3	166274033	166274131	chr3:166226725-166281774
chr3	166274198	166274291	chr3:166226725-166281774
chr3	166274508	166274657	chr3:166226725-166281774
chr3	166274753	166274838	chr3:166226725-166281774
chr3	166274853	166275119	chr3:166226725-166281774

chr3	166275298	166275388	chr3:166226725-166281774
chr3	166275458	166275542	chr3:166226725-166281774
chr3	166275548	166275697	chr3:166226725-166281774
chr3	166275853	166276137	chr3:166226725-166281774
chr3	166276218	166276381	chr3:166226725-166281774
chr3	166276383	166276576	chr3:166226725-166281774
chr3	166276578	166277113	chr3:166226725-166281774
chr3	166277118	166277564	chr3:166226725-166281774
chr3	166277618	166280236	chr3:166226725-166281774
chr3	166280263	166280914	chr3:166226725-166281774
chr3	166280918	166281015	chr3:166226725-166281774
chr3	166281018	166281781	chr3:166226725-166281774
chr3	166287488	166288189	chr3:166287480-166288146
chr3	166288438	166288576	chr3:166288462-166288551
chr3	180379634	180379812	chr3:180379659-180379795
chr3	180381629	180381803	chr3:180381654-180381774
chr3	180425354	180426028	chr3:180425376-180426692
chr3	180426049	180426710	chr3:180425376-180426692
chr3	180428944	180429052	chr3:180428980-180429007
chr3	180429064	180429212	chr3:180429097-180429191
chr3	180443279	180443387	chr3:180443302-180443361
chr3	180451239	180451498	chr3:180451266-180451470
chr3	180452244	180452413	chr3:180452274-180452394
chr3	180455259	180455637	chr3:180455280-180455602
chr3	180465644	180465849	chr3:180465667-180465831
chr3	180466009	180466139	chr3:180466033-180466107
chr3	180469114	180469213	chr3:180469135-180469198
chr3	180499749	180499931	chr3:180499771-180499912
chr3	180528164	180528236	chr3:180528197-180528258
chr3	180585899	180586117	chr3:180585928-180586083
chr3	180586504	180586664	chr3:180586538-180586624
chr3	180587809	180588431	chr3:180587834-180588427
chr3	180588564	180588805	chr3:180588587-180588793
chr3	180602089	180602583	chr3:180602054-180602553
chr3	180630054	180630552	chr3:180630089-180630524
chr3	180632409	180632798	chr3:180632439-180632783
chr3	180633189	180633435	chr3:180633221-180633419
chr3	180645404	180645623	chr3:180645435-180645601
chr3	180650799	180651205	chr3:180650823-180651174
chr3	180652904	180653033	chr3:180652925-180653019
chr3	180665624	180666011	chr3:180665652-180665980
chr3	180666109	180666316	chr3:180666134-180666283
chr3	180666484	180666623	chr3:180666508-180666602
chr3	180666989	180667156	chr3:180667014-180667131
chr3	180669054	180669270	chr3:180669085-180669256
chr3	180671514	180671906	chr3:180671545-180671893
chr3	180675304	180675748	chr3:180675314-180675716
chr3	180679249	180679385	chr3:180679255-180679342
chr3	180680649	180680882	chr3:180680670-180680878
chr3	180685814	180686229	chr3:180685838-180686193
chr3	180687924	180688170	chr3:180687945-180688449
chr3	180688209	180688483	chr3:180687945-180688449
chr3	180688834	180689627	chr3:180688862-180689604
chr3	180693079	180693204	chr3:180693100-180693192
chr3	180693874	180694855	chr3:180693909-180696341
chr3	180694874	180695978	chr3:180693909-180696341
chr3	180695989	180696377	chr3:180693909-180696341
chr3	180696499	180697344	chr3:180696522-180700541
chr3	180697349	180698719	chr3:180696522-180700541
chr3	180698724	180700573	chr3:180696522-180700541
chr3	180774439	180774609	chr3:180774467-180774574
chr3	180806639	180806750	chr3:180806664-180806705
chr3	180892644	180893179	chr3:180892677-180893166

chr3	180893279	180893381	chr3:180893301-180893371
chr3	181050024	181050134	chr3:181050065-181050098
chr3	181091644	181091777	chr3:181091666-181091748
chr3	181125644	181125814	chr3:181124842-181208277
chr3	181126094	181126173	chr3:181124842-181208277
chr3	181126184	181127190	chr3:181124842-181208277
chr3	181127194	181128976	chr3:181124842-181208277
chr3	181128979	181129052	chr3:181124842-181208277
chr3	181129054	181129204	chr3:181124842-181208277
chr3	181129209	181129539	chr3:181124842-181208277
chr3	181129569	181131352	chr3:181124842-181208277
chr3	181131379	181132189	chr3:181124842-181208277
chr3	181132244	181132378	chr3:181124842-181208277
chr3	181132379	181133072	chr3:181124842-181208277
chr3	181133074	181134307	chr3:181124842-181208277
chr3	181134314	181134937	chr3:181124842-181208277
chr3	181134939	181136068	chr3:181124842-181208277
chr3	181136074	181136870	chr3:181124842-181208277
chr3	181136874	181137194	chr3:181124842-181208277
chr3	181137249	181137519	chr3:181124842-181208277
chr3	181137524	181140992	chr3:181124842-181208277
chr3	181140999	181141492	chr3:181124842-181208277
chr3	181141874	181144965	chr3:181124842-181208277
chr3	181145179	181146389	chr3:181124842-181208277
chr3	181146479	181146715	chr3:181124842-181208277
chr3	181146724	181146800	chr3:181124842-181208277
chr3	181146804	181147377	chr3:181124842-181208277
chr3	181147494	181147818	chr3:181124842-181208277
chr3	181148369	181148918	chr3:181124842-181208277
chr3	181149014	181149530	chr3:181124842-181208277
chr3	181149534	181153202	chr3:181124842-181208277
chr3	181153204	181153907	chr3:181124842-181208277
chr3	181153934	181154056	chr3:181124842-181208277
chr3	181154099	181154183	chr3:181124842-181208277
chr3	181154184	181154410	chr3:181124842-181208277
chr3	181154414	181154502	chr3:181124842-181208277
chr3	181154589	181158077	chr3:181124842-181208277
chr3	181159024	181160987	chr3:181124842-181208277
chr3	181160989	181162748	chr3:181124842-181208277
chr3	181162754	181163139	chr3:181124842-181208277
chr3	181163434	181163600	chr3:181124842-181208277
chr3	181163604	181163861	chr3:181124842-181208277
chr3	181163864	181164318	chr3:181124842-181208277
chr3	181164329	181164825	chr3:181124842-181208277
chr3	181165044	181165404	chr3:181124842-181208277
chr3	181165514	181165744	chr3:181124842-181208277
chr3	181165799	181166751	chr3:181124842-181208277
chr3	181166814	181167115	chr3:181124842-181208277
chr3	181167119	181167194	chr3:181124842-181208277
chr3	181167469	181170234	chr3:181124842-181208277
chr3	181170364	181172224	chr3:181124842-181208277
chr3	181172244	181172887	chr3:181124842-181208277
chr3	181172889	181173682	chr3:181124842-181208277
chr3	181173689	181176036	chr3:181124842-181208277
chr3	181176039	181177902	chr3:181124842-181208277
chr3	181177909	181181178	chr3:181124842-181208277
chr3	181181184	181181471	chr3:181124842-181208277
chr3	181181474	181182122	chr3:181124842-181208277
chr3	181182124	181182726	chr3:181124842-181208277
chr3	181182749	181183616	chr3:181124842-181208277
chr3	181183619	181183838	chr3:181124842-181208277
chr3	181183859	181184040	chr3:181124842-181208277
chr3	181184224	181184329	chr3:181124842-181208277

chr3	181184419	181184561	chr3:181124842-181208277
chr3	181184574	181184682	chr3:181124842-181208277
chr3	181184689	181187931	chr3:181124842-181208277
chr3	181187954	181188277	chr3:181124842-181208277
chr3	181188534	181188715	chr3:181124842-181208277
chr3	181188769	181188869	chr3:181124842-181208277
chr3	181189499	181189564	chr3:181124842-181208277
chr3	181190029	181190103	chr3:181124842-181208277
chr3	181190814	181190912	chr3:181124842-181208277
chr3	181191439	181191544	chr3:181124842-181208277
chr3	181191809	181191885	chr3:181124842-181208277
chr3	181193244	181193354	chr3:181124842-181208277
chr3	181193779	181193854	chr3:181124842-181208277
chr3	181193954	181194048	chr3:181124842-181208277
chr3	181194164	181194239	chr3:181124842-181208277
chr3	181194269	181194545	chr3:181124842-181208277
chr3	181194589	181195322	chr3:181124842-181208277
chr3	181195379	181195995	chr3:181124842-181208277
chr3	181196914	181196987	chr3:181124842-181208277
chr3	181198544	181198614	chr3:181124842-181208277
chr3	181200664	181200985	chr3:181124842-181208277
chr3	181201274	181201601	chr3:181124842-181208277
chr3	181201609	181202496	chr3:181124842-181208277
chr3	181202499	181203169	chr3:181124842-181208277
chr3	181203174	181204030	chr3:181124842-181208277
chr3	181204044	181204498	chr3:181124842-181208277
chr3	181204499	181204818	chr3:181124842-181208277
chr3	181204844	181205099	chr3:181124842-181208277
chr3	181205104	181205400	chr3:181124842-181208277
chr3	181205469	181207230	chr3:181124842-181208277
chr3	181207254	181207979	chr3:181124842-181208277
chr3	181207984	181208300	chr3:181124842-181208277
chr3	181251794	181252263	chr3:181251820-181252249
chr3	181252419	181252865	chr3:181252399-181252835
chr3	181253549	181253687	chr3:181253572-181253669
chr3	181271569	181271670	chr3:181271592-181271661
chr3	181281474	181281727	chr3:181281507-181281716
chr3	181328639	181328737	chr3:181328648-181328717
chr3	181408594	181408810	chr3:181408621-181408790
chr3	181417354	181417760	chr3:181417385-181417832
chr3	181417779	181417853	chr3:181417385-181417832
chr3	181457332	181458115	chr3:181457356-181460261
chr3	181458147	181458803	chr3:181457356-181460261
chr3	181458807	181459022	chr3:181457356-181460261
chr3	181459027	181459190	chr3:181457356-181460261
chr3	181459202	181459698	chr3:181457356-181460261
chr3	181459707	181459986	chr3:181457356-181460261
chr3	181459987	181460243	chr3:181457356-181460261
chr3	182655837	182656153	chr3:182655861-182662961
chr3	182656162	182657261	chr3:182655861-182662961
chr3	182657272	182657351	chr3:182655861-182662961
chr3	182657352	182660906	chr3:182655861-182662961
chr3	182660927	182661027	chr3:182655861-182662961
chr3	182661052	182661141	chr3:182655861-182662961
chr3	182661142	182661940	chr3:182655861-182662961
chr3	182661957	182662355	chr3:182655861-182662961
chr3	182662357	182662964	chr3:182655861-182662961
chr3	182665002	182665132	chr3:182665025-182665122
chr3	182665307	182665447	chr3:182665337-182665420
chr3	182679007	182679173	chr3:182679013-182679144
chr3	182681647	182681863	chr3:182681668-182681837
chr3	182683297	182683575	chr3:182683324-182683541
chr3	182697842	182697966	chr3:182697863-182697939

chr3	182698252	182698333	chr3:182698274-182698431
chr3	182698337	182698460	chr3:182698274-182698431
chr3	182698567	182698755	chr3:182698594-182698723
chr3	182699052	182699304	chr3:182699081-182699275
chr3	182703557	182703774	chr3:182703579-182703741
chr3	182704322	182704524	chr3:182704346-182704620
chr3	182732982	182733377	chr3:182733005-182733354
chr3	182734837	182735461	chr3:182734858-182735596
chr3	182735477	182735559	chr3:182734858-182735596
chr3	182737887	182738058	chr3:182737917-182738025
chr3	182740172	182740361	chr3:182740204-182740505
chr3	182740372	182740471	chr3:182740204-182740505
chr3	182743512	182743618	chr3:182743542-182743592
chr3	182746852	182746990	chr3:182746880-182746977
chr3	182751757	182751890	chr3:182751778-182751865
chr3	182754982	182755256	chr3:182755005-182755222
chr3	182756782	182756959	chr3:182756813-182756923
chr3	182759332	182759581	chr3:182759354-182759538
chr3	182763177	182763342	chr3:182763200-182763328
chr3	182769917	182770054	chr3:182769946-182770028
chr3	182775077	182775250	chr3:182775098-182775210
chr3	182788762	182788971	chr3:182788786-182788932
chr3	182788972	182789184	chr3:182788997-182789145
chr3	182790132	182790300	chr3:182790153-182790275
chr3	182804447	182804602	chr3:182804480-182804576
chr3	182808697	182808844	chr3:182808723-182808819
chr3	182810137	182810343	chr3:182810160-182810333
chr3	182812322	182812422	chr3:182812346-182812395
chr3	182816252	182817052	chr3:182816275-182817031
chr3	182817132	182817406	chr3:182817139-182817375
chr3	182833231	182833369	chr3:182833261-182833863
chr3	182833656	182833889	chr3:182833261-182833863
chr3	182839976	182841083	chr3:182840000-182842002
chr3	182841361	182842035	chr3:182840000-182842002
chr3	182853476	182853687	chr3:182853504-182853675
chr3	182858291	182858404	chr3:182858325-182858383
chr3	182870131	182870308	chr3:182870162-182870291
chr3	182871436	182872208	chr3:182871469-182872179
chr3	182875851	182875958	chr3:182875900-182875920
chr3	182878611	182878792	chr3:182878644-182878750
chr3	182879676	182879830	chr3:182879708-182879803
chr3	182880361	182880718	chr3:182880394-182880699
chr3	182881386	182881660	chr3:182881407-182881627
chr3	190499992	190500350	chr3:190500006-190500320
chr3	190502127	190502452	chr3:190502162-190502427
chr3	190506377	190506551	chr3:190506410-190506561
chr3	190508242	190508353	chr3:190508265-190508347
chr3	190509342	190509474	chr3:190509363-190509444
chr3	190510952	190511079	chr3:190510979-190511050
chr3	190511332	190511508	chr3:190511364-190511474
chr3	190589827	190590150	chr3:190589848-190678987
chr3	190590157	190590692	chr3:190589848-190678987
chr3	190590697	190591145	chr3:190589848-190678987
chr3	190591147	190591273	chr3:190589848-190678987
chr3	190591447	190592503	chr3:190589848-190678987
chr3	190592827	190593565	chr3:190589848-190678987
chr3	190594142	190594571	chr3:190589848-190678987
chr3	190594577	190595340	chr3:190589848-190678987
chr3	190595732	190595977	chr3:190589848-190678987
chr3	190596017	190596879	chr3:190589848-190678987
chr3	190596932	190597013	chr3:190589848-190678987
chr3	190597167	190597452	chr3:190589848-190678987
chr3	190597462	190597768	chr3:190589848-190678987

chr3	190597772	190597850	chr3:190589848-190678987
chr3	190597882	190599601	chr3:190589848-190678987
chr3	190599697	190601370	chr3:190589848-190678987
chr3	190601747	190603184	chr3:190589848-190678987
chr3	190603497	190604459	chr3:190589848-190678987
chr3	190604467	190606203	chr3:190589848-190678987
chr3	190606527	190607183	chr3:190589848-190678987
chr3	190607187	190607892	chr3:190589848-190678987
chr3	190607977	190608648	chr3:190589848-190678987
chr3	190608652	190609475	chr3:190589848-190678987
chr3	190609497	190610430	chr3:190589848-190678987
chr3	190610437	190611431	chr3:190589848-190678987
chr3	190611507	190611816	chr3:190589848-190678987
chr3	190612147	190612683	chr3:190589848-190678987
chr3	190612687	190612840	chr3:190589848-190678987
chr3	190612847	190612925	chr3:190589848-190678987
chr3	190613152	190613307	chr3:190589848-190678987
chr3	190613337	190613546	chr3:190589848-190678987
chr3	190613607	190613746	chr3:190589848-190678987
chr3	190613757	190614017	chr3:190589848-190678987
chr3	190614137	190614232	chr3:190589848-190678987
chr3	190614267	190614441	chr3:190589848-190678987
chr3	190614562	190614707	chr3:190589848-190678987
chr3	190614712	190614787	chr3:190589848-190678987
chr3	190614812	190614908	chr3:190589848-190678987
chr3	190615197	190615282	chr3:190589848-190678987
chr3	190615307	190615401	chr3:190589848-190678987
chr3	190615617	190615692	chr3:190589848-190678987
chr3	190616267	190616342	chr3:190589848-190678987
chr3	190617807	190617992	chr3:190589848-190678987
chr3	190618482	190619260	chr3:190589848-190678987
chr3	190619387	190619889	chr3:190589848-190678987
chr3	190620032	190620132	chr3:190589848-190678987
chr3	190620222	190620340	chr3:190589848-190678987
chr3	190620452	190622250	chr3:190589848-190678987
chr3	190622277	190622350	chr3:190589848-190678987
chr3	190622442	190622522	chr3:190589848-190678987
chr3	190623072	190623154	chr3:190589848-190678987
chr3	190623307	190623394	chr3:190589848-190678987
chr3	190623487	190623646	chr3:190589848-190678987
chr3	190623647	190623723	chr3:190589848-190678987
chr3	190623792	190623900	chr3:190589848-190678987
chr3	190624082	190624167	chr3:190589848-190678987
chr3	190624312	190624386	chr3:190589848-190678987
chr3	190624552	190626466	chr3:190589848-190678987
chr3	190626472	190631414	chr3:190589848-190678987
chr3	190631462	190631853	chr3:190589848-190678987
chr3	190631952	190632206	chr3:190589848-190678987
chr3	190632277	190632462	chr3:190589848-190678987
chr3	190632467	190632985	chr3:190589848-190678987
chr3	190632987	190633221	chr3:190589848-190678987
chr3	190633302	190633437	chr3:190589848-190678987
chr3	190633512	190633679	chr3:190589848-190678987
chr3	190633727	190633801	chr3:190589848-190678987
chr3	190634277	190634368	chr3:190589848-190678987
chr3	190634587	190634674	chr3:190589848-190678987
chr3	190634912	190635062	chr3:190589848-190678987
chr3	190635067	190635179	chr3:190589848-190678987
chr3	190635202	190635537	chr3:190589848-190678987
chr3	190635547	190635653	chr3:190589848-190678987
chr3	190635767	190635902	chr3:190589848-190678987
chr3	190636067	190636308	chr3:190589848-190678987
chr3	190636322	190636440	chr3:190589848-190678987

chr3	190636442	190636618	chr3:190589848-190678987
chr3	190636767	190636844	chr3:190589848-190678987
chr3	190636942	190637100	chr3:190589848-190678987
chr3	190637152	190637587	chr3:190589848-190678987
chr3	190638997	190639065	chr3:190589848-190678987
chr3	190643617	190643723	chr3:190589848-190678987
chr3	190643787	190643897	chr3:190589848-190678987
chr3	190643907	190644041	chr3:190589848-190678987
chr3	190644042	190644874	chr3:190589848-190678987
chr3	190645097	190645245	chr3:190589848-190678987
chr3	190645522	190646029	chr3:190589848-190678987
chr3	190646467	190648866	chr3:190589848-190678987
chr3	190648907	190649016	chr3:190589848-190678987
chr3	190649252	190649496	chr3:190589848-190678987
chr3	190649662	190649791	chr3:190589848-190678987
chr3	190650032	190650285	chr3:190589848-190678987
chr3	190650292	190650558	chr3:190589848-190678987
chr3	190650857	190651637	chr3:190589848-190678987
chr3	190651852	190651936	chr3:190589848-190678987
chr3	190652837	190652913	chr3:190589848-190678987
chr3	190652987	190653079	chr3:190589848-190678987
chr3	190653082	190653197	chr3:190589848-190678987
chr3	190653262	190654097	chr3:190589848-190678987
chr3	190654142	190654224	chr3:190589848-190678987
chr3	190654267	190654487	chr3:190589848-190678987
chr3	190654507	190654584	chr3:190589848-190678987
chr3	190654587	190655454	chr3:190589848-190678987
chr3	190655477	190655762	chr3:190589848-190678987
chr3	190655772	190655867	chr3:190589848-190678987
chr3	190655872	190656232	chr3:190589848-190678987
chr3	190656237	190658341	chr3:190589848-190678987
chr3	190658347	190658792	chr3:190589848-190678987
chr3	190658797	190662217	chr3:190589848-190678987
chr3	190662252	190662873	chr3:190589848-190678987
chr3	190662877	190663344	chr3:190589848-190678987
chr3	190663632	190664532	chr3:190589848-190678987
chr3	190664552	190665464	chr3:190589848-190678987
chr3	190666152	190667119	chr3:190589848-190678987
chr3	190667127	190667647	chr3:190589848-190678987
chr3	190667667	190668468	chr3:190589848-190678987
chr3	190668507	190668905	chr3:190589848-190678987
chr3	190668997	190669111	chr3:190589848-190678987
chr3	190669117	190669819	chr3:190589848-190678987
chr3	190670122	190670367	chr3:190589848-190678987
chr3	190670402	190670568	chr3:190589848-190678987
chr3	190670747	190670832	chr3:190589848-190678987
chr3	190670952	190671030	chr3:190589848-190678987
chr3	190671112	190671341	chr3:190589848-190678987
chr3	190671352	190671854	chr3:190589848-190678987
chr3	190672142	190672425	chr3:190589848-190678987
chr3	190672612	190673127	chr3:190589848-190678987
chr3	190674272	190674382	chr3:190589848-190678987
chr3	190674392	190676099	chr3:190589848-190678987
chr3	190676267	190676384	chr3:190589848-190678987
chr3	190676662	190676770	chr3:190589848-190678987
chr3	190677027	190677176	chr3:190589848-190678987
chr3	190677632	190677824	chr3:190589848-190678987
chr3	190678117	190678235	chr3:190589848-190678987
chr3	190678377	190678466	chr3:190589848-190678987
chr3	190678787	190679027	chr3:190589848-190678987
chr3	195778122	195779069	chr3:195778154-195779055
chr3	195780262	195780459	chr3:195780288-195780429
chr3	195781927	195782214	chr3:195781950-195782172

chr3	195785132	195785267	chr3:195785154-195785236
chr3	195785422	195785525	chr3:195785444-195785503
chr3	195787027	195787129	chr3:195787050-195787118
chr3	195789417	195789522	chr3:195789452-195789516
chr3	195789697	195789837	chr3:195789724-195789810
chr3	195791157	195791307	chr3:195791179-195791299
chr3	195792292	195792508	chr3:195792313-195792471
chr3	195794357	195794540	chr3:195794388-195794528
chr3	195794867	195795012	chr3:195794902-195795001
chr3	195796302	195796472	chr3:195796325-195796439
chr3	195798237	195798375	chr3:195798266-195798369
chr3	195798852	195799057	chr3:195798873-195799023
chr3	195800772	195801024	chr3:195800800-195800996
chr3	195802007	195802251	chr3:195802029-195802231
chr3	195803912	195804022	chr3:195803934-195803993
chr3	195808667	195809057	chr3:195808701-195809032
chr4	843040	843603	chr4:843065-843562
chr4	843645	844206	chr4:843679-844197
chr4	844695	844899	chr4:844723-844872
chr4	845505	845796	chr4:845537-845762
chr4	853370	853543	chr4:853393-853510
chr4	858885	859064	chr4:858909-859032
chr4	860130	860334	chr4:860151-860322
chr4	860710	861249	chr4:860743-861220
chr4	862305	862511	chr4:862326-862473
chr4	864470	864716	chr4:864498-864692
chr4	866020	866603	chr4:866053-866561
chr4	870295	870442	chr4:870317-870397
chr4	870855	871030	chr4:870877-870995
chr4	871375	871630	chr4:871402-871597
chr4	875670	875850	chr4:875694-875828
chr4	876460	876631	chr4:876484-876607
chr4	877070	877296	chr4:877102-877251
chr4	877800	877901	chr4:877824-877874
chr4	882610	882786	chr4:882634-882758
chr4	884285	884446	chr4:884319-884410
chr4	887135	887316	chr4:887164-887277
chr4	887640	887814	chr4:887661-887797
chr4	890225	890360	chr4:890247-890337
chr4	891795	891966	chr4:891820-891946
chr4	898390	898605	chr4:898424-898567
chr4	900035	900592	chr4:900060-900559
chr4	905435	905638	chr4:905460-905630
chr4	905785	906005	chr4:905817-905962
chr4	906490	906606	chr4:906522-906582
chr4	907365	907479	chr4:907394-907456
chr4	909250	909501	chr4:909285-909466
chr4	925805	926354	chr4:925830-926161;chr4:926174-926328
chr4	939765	939937	chr4:939786-939914
chr4	941470	942420	chr4:941496-942403
chr4	944185	944322	chr4:944208-944306
chr4	944970	945069	chr4:944994-945047
chr4	945410	945790	chr4:945433-945761
chr4	946140	946248	chr4:946154-946238
chr4	946955	947161	chr4:946977-947142
chr4	949165	949311	chr4:949192-949279
chr4	949520	949767	chr4:949542-949732
chr4	950050	950237	chr4:950084-950680
chr4	950520	950706	chr4:950084-950680
chr4	951580	952465	chr4:951611-952444
chr4	952640	953291	chr4:952671-954509
chr4	953295	954549	chr4:952671-954509
chr4	954810	955019	chr4:954836-954989

chr4	955230	955404	chr4:955254-955366
chr4	955445	955657	chr4:955475-955622
chr4	955740	955898	chr4:955769-955870
chr4	956200	956441	chr4:956222-956401
chr4	956535	956740	chr4:956559-956708
chr4	956905	957114	chr4:956926-957078
chr4	958935	959115	chr4:958963-959079
chr4	959245	959363	chr4:959278-959317
chr4	959690	959899	chr4:959715-959866
chr4	960230	960342	chr4:960253-960315
chr4	960505	960618	chr4:960535-960590
chr4	960730	960871	chr4:960751-960842
chr4	960885	961172	chr4:960916-961149
chr4	961315	961460	chr4:961336-961437
chr4	961480	961621	chr4:961515-961590
chr4	961645	961849	chr4:961667-961815
chr4	962045	962226	chr4:962069-962195
chr4	962245	962384	chr4:962266-962352
chr4	962575	962715	chr4:962598-962698
chr4	964750	964900	chr4:964780-964860
chr4	966970	967359	chr4:966999-967348
chr4	980570	980705	chr4:980593-980683
chr4	15606136	15607462	chr4:15606161-15607422
chr4	15613866	15614066	chr4:15613888-15614037
chr4	15626851	15627620	chr4:15626874-15627600
chr4	15628471	15628616	chr4:15628495-15628578
chr4	15629476	15629697	chr4:15629507-15629656
chr4	15632266	15632438	chr4:15632288-15632414
chr4	15638091	15638312	chr4:15638116-15638299
chr4	15638741	15639000	chr4:15638767-15639266
chr4	15639006	15639289	chr4:15638767-15639266
chr4	15640106	15640345	chr4:15640130-15640317
chr4	15642386	15642519	chr4:15642410-15642506
chr4	15643136	15643213	chr4:15643146-15643250
chr4	15646091	15646341	chr4:15646115-15646331
chr4	15656791	15657053	chr4:15656826-15657035
chr4	15657816	15657999	chr4:15657851-15657965
chr4	15661321	15661568	chr4:15661349-15661545
chr4	15682871	15683167	chr4:15682902-15683147
chr4	15704596	15704979	chr4:15704572-15704955
chr4	15706511	15706616	chr4:15706542-15706587
chr4	15707116	15707284	chr4:15707137-15707264
chr4	15709106	15709292	chr4:15709131-15709269
chr4	15713406	15713545	chr4:15713428-15713512
chr4	15716886	15717024	chr4:15716907-15716984
chr4	15717296	15717458	chr4:15717329-15717422
chr4	15720506	15720652	chr4:15720529-15720616
chr4	15724476	15724584	chr4:15724497-15724557
chr4	15725156	15725328	chr4:15725057-15725289
chr4	15733341	15733971	chr4:15733362-15734410
chr4	15734226	15734327	chr4:15733362-15734410
chr4	15737651	15737785	chr4:15737675-15737754
chr4	15739376	15739892	chr4:15739409-15739936
chr4	15739901	15739970	chr4:15739409-15739936
chr4	23203131	23203281	chr4:23203158-23203272
chr4	23328849	23328974	chr4:23328880-23328948
chr4	23330974	23331075	chr4:23330998-23331066
chr4	23363814	23366129	chr4:23363843-23446906
chr4	23366169	23370863	chr4:23363843-23446906
chr4	23370869	23371715	chr4:23363843-23446906
chr4	23371724	23372853	chr4:23363843-23446906
chr4	23372854	23373758	chr4:23363843-23446906
chr4	23374194	23374461	chr4:23363843-23446906

chr4	23374844	23376879	chr4:23363843-23446906
chr4	23377169	23377730	chr4:23363843-23446906
chr4	23377739	23379646	chr4:23363843-23446906
chr4	23379649	23379749	chr4:23363843-23446906
chr4	23380049	23381403	chr4:23363843-23446906
chr4	23381404	23381778	chr4:23363843-23446906
chr4	23381804	23382131	chr4:23363843-23446906
chr4	23382429	23383268	chr4:23363843-23446906
chr4	23383269	23383421	chr4:23363843-23446906
chr4	23383424	23384149	chr4:23363843-23446906
chr4	23384154	23386821	chr4:23363843-23446906
chr4	23386829	23387123	chr4:23363843-23446906
chr4	23387139	23388197	chr4:23363843-23446906
chr4	23388204	23389868	chr4:23363843-23446906
chr4	23389879	23391199	chr4:23363843-23446906
chr4	23391204	23391333	chr4:23363843-23446906
chr4	23391379	23393470	chr4:23363843-23446906
chr4	23393479	23393562	chr4:23363843-23446906
chr4	23393574	23395637	chr4:23363843-23446906
chr4	23395664	23395743	chr4:23363843-23446906
chr4	23395854	23395933	chr4:23363843-23446906
chr4	23396104	23396284	chr4:23363843-23446906
chr4	23396319	23396397	chr4:23363843-23446906
chr4	23396434	23396648	chr4:23363843-23446906
chr4	23396714	23396932	chr4:23363843-23446906
chr4	23397824	23398065	chr4:23363843-23446906
chr4	23398074	23398201	chr4:23363843-23446906
chr4	23398289	23398391	chr4:23363843-23446906
chr4	23398494	23398570	chr4:23363843-23446906
chr4	23398669	23398885	chr4:23363843-23446906
chr4	23398899	23398982	chr4:23363843-23446906
chr4	23398994	23399155	chr4:23363843-23446906
chr4	23399159	23399223	chr4:23363843-23446906
chr4	23399239	23399522	chr4:23363843-23446906
chr4	23399524	23399672	chr4:23363843-23446906
chr4	23399954	23402079	chr4:23363843-23446906
chr4	23402124	23402604	chr4:23363843-23446906
chr4	23402609	23403178	chr4:23363843-23446906
chr4	23403294	23403375	chr4:23363843-23446906
chr4	23403484	23404642	chr4:23363843-23446906
chr4	23404729	23404844	chr4:23363843-23446906
chr4	23404849	23404966	chr4:23363843-23446906
chr4	23404984	23405093	chr4:23363843-23446906
chr4	23405299	23405561	chr4:23363843-23446906
chr4	23405944	23406013	chr4:23363843-23446906
chr4	23406159	23406298	chr4:23363843-23446906
chr4	23406649	23406764	chr4:23363843-23446906
chr4	23406819	23407118	chr4:23363843-23446906
chr4	23407244	23407348	chr4:23363843-23446906
chr4	23407449	23407581	chr4:23363843-23446906
chr4	23407614	23407690	chr4:23363843-23446906
chr4	23407699	23407853	chr4:23363843-23446906
chr4	23407904	23408094	chr4:23363843-23446906
chr4	23408189	23409630	chr4:23363843-23446906
chr4	23409634	23409715	chr4:23363843-23446906
chr4	23409734	23410536	chr4:23363843-23446906
chr4	23410664	23410758	chr4:23363843-23446906
chr4	23410889	23411422	chr4:23363843-23446906
chr4	23411429	23412026	chr4:23363843-23446906
chr4	23412029	23413780	chr4:23363843-23446906
chr4	23413789	23415341	chr4:23363843-23446906
chr4	23415359	23416449	chr4:23363843-23446906
chr4	23416459	23417133	chr4:23363843-23446906

chr4	23417154	23417823	chr4:23363843-23446906
chr4	23420159	23420762	chr4:23363843-23446906
chr4	23420764	23422350	chr4:23363843-23446906
chr4	23422379	23422535	chr4:23363843-23446906
chr4	23422594	23422901	chr4:23363843-23446906
chr4	23422944	23423345	chr4:23363843-23446906
chr4	23423364	23423550	chr4:23363843-23446906
chr4	23423569	23425231	chr4:23363843-23446906
chr4	23425234	23425516	chr4:23363843-23446906
chr4	23425519	23427118	chr4:23363843-23446906
chr4	23427199	23427341	chr4:23363843-23446906
chr4	23427354	23428037	chr4:23363843-23446906
chr4	23428049	23428164	chr4:23363843-23446906
chr4	23428169	23429229	chr4:23363843-23446906
chr4	23429504	23432766	chr4:23363843-23446906
chr4	23432814	23433215	chr4:23363843-23446906
chr4	23433224	23433623	chr4:23363843-23446906
chr4	23433624	23434056	chr4:23363843-23446906
chr4	23434074	23435404	chr4:23363843-23446906
chr4	23435414	23437549	chr4:23363843-23446906
chr4	23437574	23439803	chr4:23363843-23446906
chr4	23439894	23441212	chr4:23363843-23446906
chr4	23441489	23444036	chr4:23363843-23446906
chr4	23444369	23445968	chr4:23363843-23446906
chr4	23445974	23446930	chr4:23363843-23446906
chr4	77079857	77080608	chr4:77079889-77082904
chr4	77080872	77082599	chr4:77079889-77082904
chr4	77082602	77082910	chr4:77079889-77082904
chr4	77084347	77084559	chr4:77084377-77084536
chr4	77087367	77087476	chr4:77087402-77087454
chr4	77089532	77089665	chr4:77089555-77089629
chr4	77090987	77091173	chr4:77091019-77091138
chr4	77095267	77095483	chr4:77095296-77095466
chr4	77096917	77097098	chr4:77096943-77097063
chr4	77097567	77097704	chr4:77097589-77097681
chr4	77100647	77100904	chr4:77100669-77100858
chr4	77102072	77102278	chr4:77102106-77102254
chr4	77116827	77117035	chr4:77116859-77117017
chr4	77134557	77135070	chr4:77134579-77135046
chr4	77135167	77135594	chr4:77135192-77135577
chr4	77138707	77138802	chr4:77138731-77138841
chr4	77157392	77157619	chr4:77157421-77157805
chr4	77172852	77172988	chr4:77172873-77172973
chr4	77177297	77177698	chr4:77177330-77177676
chr4	77184827	77185003	chr4:77184856-77184996
chr4	77189787	77189993	chr4:77189812-77189966
chr4	77192687	77192944	chr4:77192720-77192921
chr4	77199192	77199417	chr4:77199221-77199377
chr4	77201392	77201522	chr4:77201416-77201494
chr4	77204507	77204962	chr4:77204533-77204933
chr4	77227152	77228172	chr4:77227178-77228142
chr4	77230272	77231339	chr4:77230296-77232752
chr4	77231362	77232780	chr4:77230296-77232752
chr4	90459410	90459558	chr4:90459432-90459537
chr4	90462000	90462206	chr4:90462022-90462178
chr4	90466450	90466569	chr4:90466476-90466719
chr4	90466575	90466727	chr4:90466476-90466719
chr4	90472180	90472762	chr4:90472210-90472469;chr4:90472506-90472721
chr4	90507045	90507284	chr4:90507066-90507855
chr4	90507345	90507871	chr4:90507066-90507855
chr4	90508940	90509134	chr4:90508971-90509091
chr4	90548900	90549082	chr4:90548932-90549068
chr4	90563545	90563683	chr4:90563567-90563671

chr4	90572245	90572392	chr4:90572266-90572345
chr4	90574390	90574573	chr4:90574421-90574536
chr4	90575710	90575850	chr4:90575731-90575812
chr4	90581340	90581450	chr4:90581364-90581506
chr4	90581460	90581533	chr4:90581364-90581506
chr4	90585615	90585697	chr4:90585649-90585684
chr4	90602640	90602842	chr4:90602631-90602835
chr4	90612420	90612587	chr4:90612435-90612556
chr4	90613765	90614025	chr4:90613565-90615087
chr4	90614160	90615111	chr4:90613565-90615087
chr4	90623390	90623527	chr4:90623424-90623535
chr4	90625515	90625706	chr4:90625547-90625677
chr4	90626065	90627769	chr4:90626098-90635338
chr4	90628055	90631012	chr4:90626098-90635338
chr4	90631310	90635353	chr4:90626098-90635338
chr4	90640950	90641339	chr4:90640976-90641323
chr4	90645270	90645836	chr4:90645249-90647811
chr4	90645845	90646234	chr4:90645249-90647811
chr4	90646245	90646477	chr4:90645249-90647811
chr4	90646545	90646690	chr4:90645249-90647811
chr4	90646695	90647195	chr4:90645249-90647811
chr4	90647200	90647822	chr4:90645249-90647811
chr4	90650320	90650454	chr4:90650344-90650428
chr4	90678250	90678819	chr4:90678283-90678782
chr4	90742975	90743572	chr4:90743002-90743539
chr4	90749255	90749363	chr4:90749293-90749335
chr4	90756665	90756882	chr4:90756697-90756846
chr4	90757255	90757394	chr4:90757277-90757364
chr4	90757525	90757946	chr4:90757558-90757843;chr4:90757893-90758379
chr4	90757955	90758416	chr4:90757893-90758379
chr4	90759370	90760179	chr4:90759402-90759466;chr4:90759516-90761746
chr4	90760190	90761298	chr4:90759516-90761746
chr4	90761305	90761517	chr4:90759516-90761746
chr4	90761530	90761775	chr4:90759516-90761746
chr4	90762470	90762584	chr4:90762491-90762551
chr4	90762850	90763170	chr4:90762861-90763129
chr4	90780850	90780926	chr4:90780803-90780902
chr4	118349826	118349961	chr4:118349849-118349923
chr4	118492876	118493309	chr4:118492909-118493290
chr4	118498081	118498180	chr4:118498121-118498145
chr4	118542001	118542178	chr4:118542032-118542147
chr4	118575926	118576066	chr4:118575961-118576065
chr4	118610061	118611192	chr4:118610090-118612343
chr4	118611216	118612375	chr4:118610090-118612343
chr4	118633116	118633220	chr4:118632597-118780942
chr4	118633481	118633547	chr4:118632597-118780942
chr4	118633641	118633791	chr4:118632597-118780942
chr4	118634046	118634180	chr4:118632597-118780942
chr4	118634471	118634546	chr4:118632597-118780942
chr4	118634726	118634803	chr4:118632597-118780942
chr4	118634901	118634980	chr4:118632597-118780942
chr4	118634986	118635083	chr4:118632597-118780942
chr4	118635111	118635864	chr4:118632597-118780942
chr4	118636156	118636290	chr4:118632597-118780942
chr4	118636296	118636549	chr4:118632597-118780942
chr4	118636556	118636951	chr4:118632597-118780942
chr4	118637016	118638997	chr4:118632597-118780942
chr4	118639001	118639089	chr4:118632597-118780942
chr4	118639091	118640506	chr4:118632597-118780942
chr4	118640566	118640794	chr4:118632597-118780942
chr4	118640796	118641016	chr4:118632597-118780942
chr4	118641021	118641125	chr4:118632597-118780942
chr4	118641186	118641314	chr4:118632597-118780942

chr4	118641321	118641398	chr4:118632597-118780942
chr4	118641406	118641473	chr4:118632597-118780942
chr4	118641846	118641960	chr4:118632597-118780942
chr4	118642006	118642216	chr4:118632597-118780942
chr4	118642231	118642372	chr4:118632597-118780942
chr4	118642391	118642481	chr4:118632597-118780942
chr4	118642526	118642871	chr4:118632597-118780942
chr4	118643001	118643100	chr4:118632597-118780942
chr4	118643191	118643267	chr4:118632597-118780942
chr4	118643406	118643479	chr4:118632597-118780942
chr4	118643591	118643672	chr4:118632597-118780942
chr4	118643781	118643857	chr4:118632597-118780942
chr4	118643911	118644094	chr4:118632597-118780942
chr4	118644126	118644393	chr4:118632597-118780942
chr4	118644441	118644574	chr4:118632597-118780942
chr4	118644576	118644670	chr4:118632597-118780942
chr4	118644916	118645064	chr4:118632597-118780942
chr4	118645131	118645623	chr4:118632597-118780942
chr4	118645631	118647612	chr4:118632597-118780942
chr4	118647906	118648855	chr4:118632597-118780942
chr4	118648861	118649319	chr4:118632597-118780942
chr4	118649586	118650862	chr4:118632597-118780942
chr4	118650871	118652419	chr4:118632597-118780942
chr4	118652426	118653198	chr4:118632597-118780942
chr4	118653211	118653316	chr4:118632597-118780942
chr4	118653321	118653728	chr4:118632597-118780942
chr4	118653736	118654797	chr4:118632597-118780942
chr4	118654801	118655245	chr4:118632597-118780942
chr4	118655251	118655444	chr4:118632597-118780942
chr4	118655771	118655916	chr4:118632597-118780942
chr4	118656236	118657055	chr4:118632597-118780942
chr4	118657196	118657273	chr4:118632597-118780942
chr4	118657286	118657360	chr4:118632597-118780942
chr4	118657386	118657598	chr4:118632597-118780942
chr4	118657646	118657785	chr4:118632597-118780942
chr4	118658116	118658189	chr4:118632597-118780942
chr4	118658196	118658274	chr4:118632597-118780942
chr4	118659956	118660059	chr4:118632597-118780942
chr4	118660586	118660662	chr4:118632597-118780942
chr4	118662251	118662372	chr4:118632597-118780942
chr4	118662381	118664032	chr4:118632597-118780942
chr4	118664341	118664893	chr4:118632597-118780942
chr4	118664896	118665009	chr4:118632597-118780942
chr4	118665286	118666187	chr4:118632597-118780942
chr4	118666191	118666394	chr4:118632597-118780942
chr4	118666891	118666965	chr4:118632597-118780942
chr4	118666971	118669481	chr4:118632597-118780942
chr4	118669491	118669940	chr4:118632597-118780942
chr4	118669941	118673191	chr4:118632597-118780942
chr4	118673336	118673418	chr4:118632597-118780942
chr4	118673426	118673502	chr4:118632597-118780942
chr4	118674101	118674249	chr4:118632597-118780942
chr4	118674271	118674357	chr4:118632597-118780942
chr4	118674481	118674555	chr4:118632597-118780942
chr4	118675451	118675663	chr4:118632597-118780942
chr4	118676051	118676634	chr4:118632597-118780942
chr4	118676636	118677055	chr4:118632597-118780942
chr4	118677071	118677158	chr4:118632597-118780942
chr4	118677186	118677471	chr4:118632597-118780942
chr4	118677481	118677901	chr4:118632597-118780942
chr4	118677991	118678062	chr4:118632597-118780942
chr4	118678266	118678333	chr4:118632597-118780942
chr4	118678761	118678838	chr4:118632597-118780942

chr4	118678906	118679082	chr4:118632597-118780942
chr4	118679341	118679501	chr4:118632597-118780942
chr4	118680201	118680519	chr4:118632597-118780942
chr4	118680576	118680829	chr4:118632597-118780942
chr4	118680831	118681376	chr4:118632597-118780942
chr4	118681416	118681596	chr4:118632597-118780942
chr4	118681666	118684623	chr4:118632597-118780942
chr4	118684626	118684759	chr4:118632597-118780942
chr4	118684851	118684992	chr4:118632597-118780942
chr4	118685371	118685453	chr4:118632597-118780942
chr4	118685521	118685603	chr4:118632597-118780942
chr4	118685726	118685812	chr4:118632597-118780942
chr4	118685901	118687979	chr4:118632597-118780942
chr4	118687981	118692872	chr4:118632597-118780942
chr4	118692886	118693802	chr4:118632597-118780942
chr4	118693811	118694836	chr4:118632597-118780942
chr4	118694841	118695030	chr4:118632597-118780942
chr4	118695126	118695300	chr4:118632597-118780942
chr4	118695311	118695567	chr4:118632597-118780942
chr4	118695571	118695692	chr4:118632597-118780942
chr4	118695696	118695927	chr4:118632597-118780942
chr4	118696076	118696333	chr4:118632597-118780942
chr4	118696461	118696558	chr4:118632597-118780942
chr4	118696606	118696692	chr4:118632597-118780942
chr4	118696811	118696919	chr4:118632597-118780942
chr4	118697126	118697272	chr4:118632597-118780942
chr4	118697361	118697429	chr4:118632597-118780942
chr4	118697556	118697638	chr4:118632597-118780942
chr4	118697746	118697855	chr4:118632597-118780942
chr4	118698071	118700304	chr4:118632597-118780942
chr4	118700306	118701554	chr4:118632597-118780942
chr4	118701561	118701727	chr4:118632597-118780942
chr4	118701731	118701911	chr4:118632597-118780942
chr4	118701921	118703257	chr4:118632597-118780942
chr4	118703281	118703800	chr4:118632597-118780942
chr4	118703801	118705731	chr4:118632597-118780942
chr4	118705736	118706481	chr4:118632597-118780942
chr4	118706491	118707304	chr4:118632597-118780942
chr4	118707326	118707550	chr4:118632597-118780942
chr4	118707851	118707932	chr4:118632597-118780942
chr4	118707951	118708101	chr4:118632597-118780942
chr4	118708296	118708449	chr4:118632597-118780942
chr4	118708481	118708561	chr4:118632597-118780942
chr4	118708846	118708952	chr4:118632597-118780942
chr4	118708966	118709098	chr4:118632597-118780942
chr4	118709146	118709232	chr4:118632597-118780942
chr4	118709366	118709459	chr4:118632597-118780942
chr4	118709461	118709862	chr4:118632597-118780942
chr4	118709916	118710238	chr4:118632597-118780942
chr4	118710251	118710449	chr4:118632597-118780942
chr4	118710451	118710870	chr4:118632597-118780942
chr4	118710976	118712156	chr4:118632597-118780942
chr4	118712161	118712961	chr4:118632597-118780942
chr4	118713231	118717104	chr4:118632597-118780942
chr4	118717111	118717466	chr4:118632597-118780942
chr4	118717536	118719895	chr4:118632597-118780942
chr4	118719901	118720177	chr4:118632597-118780942
chr4	118720386	118720781	chr4:118632597-118780942
chr4	118720796	118721367	chr4:118632597-118780942
chr4	118721376	118723344	chr4:118632597-118780942
chr4	118723396	118723545	chr4:118632597-118780942
chr4	118723551	118725794	chr4:118632597-118780942
chr4	118725846	118726341	chr4:118632597-118780942

chr4	118726351	118727666	chr4:118632597-118780942
chr4	118727671	118729777	chr4:118632597-118780942
chr4	118729781	118730051	chr4:118632597-118780942
chr4	118730406	118730482	chr4:118632597-118780942
chr4	118730491	118730806	chr4:118632597-118780942
chr4	118730821	118731932	chr4:118632597-118780942
chr4	118731946	118734844	chr4:118632597-118780942
chr4	118734851	118736049	chr4:118632597-118780942
chr4	118736056	118736497	chr4:118632597-118780942
chr4	118736516	118737040	chr4:118632597-118780942
chr4	118737041	118737278	chr4:118632597-118780942
chr4	118737281	118737814	chr4:118632597-118780942
chr4	118737821	118738163	chr4:118632597-118780942
chr4	118738166	118739813	chr4:118632597-118780942
chr4	118739981	118740062	chr4:118632597-118780942
chr4	118740166	118740246	chr4:118632597-118780942
chr4	118740356	118740577	chr4:118632597-118780942
chr4	118740586	118743586	chr4:118632597-118780942
chr4	118743891	118744520	chr4:118632597-118780942
chr4	118744546	118747929	chr4:118632597-118780942
chr4	118747961	118748693	chr4:118632597-118780942
chr4	118748731	118748996	chr4:118632597-118780942
chr4	118749016	118749137	chr4:118632597-118780942
chr4	118749141	118751792	chr4:118632597-118780942
chr4	118751826	118753504	chr4:118632597-118780942
chr4	118753531	118753597	chr4:118632597-118780942
chr4	118753616	118753697	chr4:118632597-118780942
chr4	118753986	118754320	chr4:118632597-118780942
chr4	118754341	118754446	chr4:118632597-118780942
chr4	118754491	118754676	chr4:118632597-118780942
chr4	118754711	118757040	chr4:118632597-118780942
chr4	118757106	118757645	chr4:118632597-118780942
chr4	118757816	118759589	chr4:118632597-118780942
chr4	118759591	118760097	chr4:118632597-118780942
chr4	118760371	118761004	chr4:118632597-118780942
chr4	118761496	118761646	chr4:118632597-118780942
chr4	118761841	118763992	chr4:118632597-118780942
chr4	118764246	118765645	chr4:118632597-118780942
chr4	118765646	118767179	chr4:118632597-118780942
chr4	118767241	118767705	chr4:118632597-118780942
chr4	118767706	118767926	chr4:118632597-118780942
chr4	118767971	118768258	chr4:118632597-118780942
chr4	118768341	118768433	chr4:118632597-118780942
chr4	118768451	118768570	chr4:118632597-118780942
chr4	118768631	118768781	chr4:118632597-118780942
chr4	118768791	118769710	chr4:118632597-118780942
chr4	118769731	118769843	chr4:118632597-118780942
chr4	118769911	118771837	chr4:118632597-118780942
chr4	118771846	118772004	chr4:118632597-118780942
chr4	118772311	118772626	chr4:118632597-118780942
chr4	118772696	118772999	chr4:118632597-118780942
chr4	118773001	118773083	chr4:118632597-118780942
chr4	118773086	118773303	chr4:118632597-118780942
chr4	118773321	118773554	chr4:118632597-118780942
chr4	118773561	118773681	chr4:118632597-118780942
chr4	118773756	118774060	chr4:118632597-118780942
chr4	118774081	118774179	chr4:118632597-118780942
chr4	118774181	118774297	chr4:118632597-118780942
chr4	118774391	118774573	chr4:118632597-118780942
chr4	118774671	118774778	chr4:118632597-118780942
chr4	118774811	118774907	chr4:118632597-118780942
chr4	118774911	118775243	chr4:118632597-118780942
chr4	118775341	118775474	chr4:118632597-118780942

chr4	118775686	118775762	chr4:118632597-118780942
chr4	118775856	118776130	chr4:118632597-118780942
chr4	118776241	118776346	chr4:118632597-118780942
chr4	118776366	118776507	chr4:118632597-118780942
chr4	118776716	118777733	chr4:118632597-118780942
chr4	118777736	118777952	chr4:118632597-118780942
chr4	118777976	118778046	chr4:118632597-118780942
chr4	118778116	118778234	chr4:118632597-118780942
chr4	118778831	118778916	chr4:118632597-118780942
chr4	118778936	118779016	chr4:118632597-118780942
chr4	118779066	118779153	chr4:118632597-118780942
chr4	118779261	118779346	chr4:118632597-118780942
chr4	118779506	118779595	chr4:118632597-118780942
chr4	118780436	118780514	chr4:118632597-118780942
chr4	118780836	118780973	chr4:118632597-118780942
chr4	118782286	118782399	chr4:118782316-118782376
chr4	118790281	118790443	chr4:118790302-118790435
chr4	118790591	118791144	chr4:118790613-118791123
chr4	168479587	168479658	chr4:168479622-168532118
chr4	168479752	168479861	chr4:168479622-168532118
chr4	168480217	168480296	chr4:168479622-168532118
chr4	168481092	168481170	chr4:168479622-168532118
chr4	168481327	168481443	chr4:168479622-168532118
chr4	168481467	168481541	chr4:168479622-168532118
chr4	168481542	168481630	chr4:168479622-168532118
chr4	168482182	168482262	chr4:168479622-168532118
chr4	168482312	168482394	chr4:168479622-168532118
chr4	168482652	168482758	chr4:168479622-168532118
chr4	168482782	168482942	chr4:168479622-168532118
chr4	168482947	168483031	chr4:168479622-168532118
chr4	168483397	168483533	chr4:168479622-168532118
chr4	168483537	168483702	chr4:168479622-168532118
chr4	168483777	168483900	chr4:168479622-168532118
chr4	168484032	168484116	chr4:168479622-168532118
chr4	168484472	168485366	chr4:168479622-168532118
chr4	168485367	168486484	chr4:168479622-168532118
chr4	168486937	168487013	chr4:168479622-168532118
chr4	168487542	168487622	chr4:168479622-168532118
chr4	168487647	168487720	chr4:168479622-168532118
chr4	168488897	168489664	chr4:168479622-168532118
chr4	168489782	168489885	chr4:168479622-168532118
chr4	168489927	168490435	chr4:168479622-168532118
chr4	168490642	168491390	chr4:168479622-168532118
chr4	168491422	168492607	chr4:168479622-168532118
chr4	168492662	168493892	chr4:168479622-168532118
chr4	168493902	168494193	chr4:168479622-168532118
chr4	168494197	168495112	chr4:168479622-168532118
chr4	168495122	168496280	chr4:168479622-168532118
chr4	168496287	168498603	chr4:168479622-168532118
chr4	168498607	168498808	chr4:168479622-168532118
chr4	168498907	168498986	chr4:168479622-168532118
chr4	168499087	168499178	chr4:168479622-168532118
chr4	168499307	168499389	chr4:168479622-168532118
chr4	168499392	168499504	chr4:168479622-168532118
chr4	168499507	168499712	chr4:168479622-168532118
chr4	168499717	168500216	chr4:168479622-168532118
chr4	168500217	168500632	chr4:168479622-168532118
chr4	168500682	168501039	chr4:168479622-168532118
chr4	168501057	168501268	chr4:168479622-168532118
chr4	168501282	168502110	chr4:168479622-168532118
chr4	168502122	168502261	chr4:168479622-168532118
chr4	168502347	168502628	chr4:168479622-168532118
chr4	168502712	168502828	chr4:168479622-168532118

chr4	168502847	168503177	chr4:168479622-168532118
chr4	168503517	168503598	chr4:168479622-168532118
chr4	168503887	168504098	chr4:168479622-168532118
chr4	168504112	168504300	chr4:168479622-168532118
chr4	168504302	168504776	chr4:168479622-168532118
chr4	168504807	168504919	chr4:168479622-168532118
chr4	168504957	168505033	chr4:168479622-168532118
chr4	168505097	168506421	chr4:168479622-168532118
chr4	168506422	168507375	chr4:168479622-168532118
chr4	168507382	168507593	chr4:168479622-168532118
chr4	168507877	168507951	chr4:168479622-168532118
chr4	168507952	168508272	chr4:168479622-168532118
chr4	168508327	168508619	chr4:168479622-168532118
chr4	168508622	168509138	chr4:168479622-168532118
chr4	168509377	168511344	chr4:168479622-168532118
chr4	168511352	168511778	chr4:168479622-168532118
chr4	168511782	168511973	chr4:168479622-168532118
chr4	168512017	168512457	chr4:168479622-168532118
chr4	168512497	168512674	chr4:168479622-168532118
chr4	168512947	168513157	chr4:168479622-168532118
chr4	168513257	168513356	chr4:168479622-168532118
chr4	168513357	168514320	chr4:168479622-168532118
chr4	168514717	168515320	chr4:168479622-168532118
chr4	168515327	168515821	chr4:168479622-168532118
chr4	168515832	168517535	chr4:168479622-168532118
chr4	168517542	168521812	chr4:168479622-168532118
chr4	168521822	168522255	chr4:168479622-168532118
chr4	168522257	168522838	chr4:168479622-168532118
chr4	168523042	168523901	chr4:168479622-168532118
chr4	168523912	168525342	chr4:168479622-168532118
chr4	168525397	168526614	chr4:168479622-168532118
chr4	168526622	168527969	chr4:168479622-168532118
chr4	168527987	168528066	chr4:168479622-168532118
chr4	168528072	168528252	chr4:168479622-168532118
chr4	168528262	168528558	chr4:168479622-168532118
chr4	168528587	168529244	chr4:168479622-168532118
chr4	168529842	168530651	chr4:168479622-168532118
chr4	168530932	168532130	chr4:168479622-168532118
chr4	170314402	170315690	chr4:170314425-170315674
chr4	170321352	170321525	chr4:170321375-170321508
chr4	170321647	170321816	chr4:170321671-170321802
chr4	170322772	170323018	chr4:170322802-170323011
chr4	170327717	170327926	chr4:170327746-170327898
chr4	170345682	170345762	chr4:170345703-170345951
chr4	170345767	170345980	chr4:170345703-170345951
chr4	170347262	170347413	chr4:170347296-170347385
chr4	170354672	170354849	chr4:170354695-170354816
chr4	170359207	170359426	chr4:170359233-170359410
chr4	170384372	170384572	chr4:170384393-170384546
chr4	170398252	170398526	chr4:170398274-170398503
chr4	170398557	170398661	chr4:170398582-170398648
chr4	170400532	170400698	chr4:170400553-170400685
chr4	170406062	170406615	chr4:170406086-170406585
chr4	170428157	170428294	chr4:170428187-170428283
chr4	170428837	170428977	chr4:170428865-170428943
chr4	170429377	170429509	chr4:170429398-170429482
chr4	170429887	170430032	chr4:170429919-170430003
chr4	170458937	170459068	chr4:170458959-170459062
chr4	170475592	170475952	chr4:170475193-170477002
chr4	170475957	170476388	chr4:170475193-170477002
chr4	170476392	170477021	chr4:170475193-170477002
chr4	170477047	170477271	chr4:170477082-170477246
chr4	170482607	170482732	chr4:170482630-170482705

chr4	170482812	170482919	chr4:170482837-170482888
chr4	170482997	170483063	chr4:170482982-170483042
chr4	170483262	170483369	chr4:170483287-170483347
chr4	170497722	170498095	chr4:170497754-170498230
chr4	170498097	170498265	chr4:170497754-170498230
chr4	170501957	170502067	chr4:170501992-170502053
chr4	170506477	170506715	chr4:170506499-170506700
chr4	170508687	170508787	chr4:170508709-170508764
chr4	170509772	170509911	chr4:170509799-170509886
chr4	170510572	170510678	chr4:170510597-170510665
chr4	170511852	170511993	chr4:170511876-170511960
chr4	170520222	170520361	chr4:170520250-170520348
chr4	170523137	170523272	chr4:170523158-170523255
chr4	170523642	170523844	chr4:170523664-170523829
chr4	170533147	170533857	chr4:170533170-170533780;chr4:170533783-170533828
chr4	170541647	170541917	chr4:170541671-170542214
chr4	170541922	170542243	chr4:170541671-170542214
chr4	170551307	170551412	chr4:170551315-170551382
chr4	170557042	170557268	chr4:170557063-170557239
chr4	170563957	170564349	chr4:170563858-170564319
chr4	170581187	170581482	chr4:170581212-170581633
chr4	170581487	170581672	chr4:170581212-170581633
chr4	170601172	170601376	chr4:170601200-170601358
chr4	170601507	170601913	chr4:170601529-170601882
chr4	170608787	170608920	chr4:170608808-170608908
chr4	170610172	170610415	chr4:170610193-170610381
chr4	170611652	170611828	chr4:170611680-170611803
chr4	170613237	170613474	chr4:170613264-170613471
chr4	170616727	170616866	chr4:170616762-170616843
chr4	170618332	170618922	chr4:170618339-170618885
chr4	170625122	170625377	chr4:170625148-170625335
chr4	170625972	170626537	chr4:170626007-170626506
chr4	170627987	170628428	chr4:170628018-170628417
chr4	170634202	170634484	chr4:170634229-170634446
chr4	170638912	170639059	chr4:170638942-170639018
chr4	170641032	170641687	chr4:170641057-170644824
chr4	170641702	170642314	chr4:170641057-170644824
chr4	170642327	170642682	chr4:170641057-170644824
chr4	170642692	170642998	chr4:170641057-170644824
chr4	170643257	170644634	chr4:170641057-170644824
chr4	170644637	170644841	chr4:170641057-170644824
chr4	170650582	170650659	chr4:170650615-170650860
chr4	170650662	170650868	chr4:170650615-170650860
chr4	170652822	170653176	chr4:170652854-170653169
chr4	170658782	170658919	chr4:170658810-170658898
chr4	170661557	170662003	chr4:170661578-170662077
chr4	170662007	170662112	chr4:170661578-170662077
chr4	170663082	170663289	chr4:170663107-170663258
chr4	170669872	170670017	chr4:170669894-170669993
chr4	170671592	170671908	chr4:170671622-170671876
chr4	170674792	170675007	chr4:170674826-170674986
chr4	170678957	170679128	chr4:170678980-170679104
chr4	170742322	170742409	chr4:170742332-170742347
chr4	170742847	170742956	chr4:170742880-170742910
chr5	1394372	1394904	chr5:1394404-1394873
chr5	1401007	1401123	chr5:1401029-1401101
chr5	1403012	1403224	chr5:1403036-1403204
chr5	1406277	1406417	chr5:1406302-1406403
chr5	1409117	1409253	chr5:1409140-1409240
chr5	1409807	1410000	chr5:1409835-1409964
chr5	1411327	1411506	chr5:1411357-1411470
chr5	1414777	1414963	chr5:1414805-1414930
chr5	1416187	1416321	chr5:1416212-1416316

chr5	1420662	1420855	chr5:1420683-1420818
chr5	1421957	1422145	chr5:1421990-1422129
chr5	1432552	1432832	chr5:1432578-1432813
chr5	1441452	1441624	chr5:1441473-1441605
chr5	1443017	1443397	chr5:1443026-1443357
chr5	1445437	1445581	chr5:1445462-1445543
chr5	4012325	4013600	chr5:4012356-4089499
chr5	4013605	4014162	chr5:4012356-4089499
chr5	4014165	4015223	chr5:4012356-4089499
chr5	4015465	4016448	chr5:4012356-4089499
chr5	4016730	4017043	chr5:4012356-4089499
chr5	4017085	4017157	chr5:4012356-4089499
chr5	4017360	4017988	chr5:4012356-4089499
chr5	4017990	4018247	chr5:4012356-4089499
chr5	4018250	4019554	chr5:4012356-4089499
chr5	4019560	4021595	chr5:4012356-4089499
chr5	4021615	4021695	chr5:4012356-4089499
chr5	4021710	4026296	chr5:4012356-4089499
chr5	4026300	4026361	chr5:4012356-4089499
chr5	4026365	4026434	chr5:4012356-4089499
chr5	4026480	4028011	chr5:4012356-4089499
chr5	4028015	4030368	chr5:4012356-4089499
chr5	4030755	4032817	chr5:4012356-4089499
chr5	4032840	4033274	chr5:4012356-4089499
chr5	4033340	4033439	chr5:4012356-4089499
chr5	4033770	4033858	chr5:4012356-4089499
chr5	4034265	4034954	chr5:4012356-4089499
chr5	4035230	4035328	chr5:4012356-4089499
chr5	4035330	4035949	chr5:4012356-4089499
chr5	4035955	4040520	chr5:4012356-4089499
chr5	4040545	4041777	chr5:4012356-4089499
chr5	4041780	4042005	chr5:4012356-4089499
chr5	4042280	4043675	chr5:4012356-4089499
chr5	4043690	4044389	chr5:4012356-4089499
chr5	4044400	4045725	chr5:4012356-4089499
chr5	4045840	4045947	chr5:4012356-4089499
chr5	4045960	4046232	chr5:4012356-4089499
chr5	4046340	4046508	chr5:4012356-4089499
chr5	4046630	4046701	chr5:4012356-4089499
chr5	4046725	4046806	chr5:4012356-4089499
chr5	4046855	4046935	chr5:4012356-4089499
chr5	4046940	4047295	chr5:4012356-4089499
chr5	4047465	4047578	chr5:4012356-4089499
chr5	4047645	4047758	chr5:4012356-4089499
chr5	4047840	4047953	chr5:4012356-4089499
chr5	4048015	4048173	chr5:4012356-4089499
chr5	4048175	4049065	chr5:4012356-4089499
chr5	4049070	4049439	chr5:4012356-4089499
chr5	4049440	4051527	chr5:4012356-4089499
chr5	4051535	4051859	chr5:4012356-4089499
chr5	4052165	4054751	chr5:4012356-4089499
chr5	4055705	4055779	chr5:4012356-4089499
chr5	4055835	4055938	chr5:4012356-4089499
chr5	4056515	4056581	chr5:4012356-4089499
chr5	4056875	4056962	chr5:4012356-4089499
chr5	4057500	4057613	chr5:4012356-4089499
chr5	4057635	4057759	chr5:4012356-4089499
chr5	4057770	4058713	chr5:4012356-4089499
chr5	4058820	4059854	chr5:4012356-4089499
chr5	4059950	4060878	chr5:4012356-4089499
chr5	4060890	4062061	chr5:4012356-4089499
chr5	4062095	4063338	chr5:4012356-4089499
chr5	4063345	4063536	chr5:4012356-4089499

chr5	4063550	4070380	chr5:4012356-4089499
chr5	4070400	4071428	chr5:4012356-4089499
chr5	4071430	4071570	chr5:4012356-4089499
chr5	4071625	4071806	chr5:4012356-4089499
chr5	4071815	4072065	chr5:4012356-4089499
chr5	4072105	4072662	chr5:4012356-4089499
chr5	4072970	4073079	chr5:4012356-4089499
chr5	4073120	4073238	chr5:4012356-4089499
chr5	4073280	4073560	chr5:4012356-4089499
chr5	4073600	4073669	chr5:4012356-4089499
chr5	4073670	4074199	chr5:4012356-4089499
chr5	4074205	4074502	chr5:4012356-4089499
chr5	4074515	4074666	chr5:4012356-4089499
chr5	4074945	4075343	chr5:4012356-4089499
chr5	4075360	4075738	chr5:4012356-4089499
chr5	4075750	4076166	chr5:4012356-4089499
chr5	4076405	4077119	chr5:4012356-4089499
chr5	4077120	4078490	chr5:4012356-4089499
chr5	4078520	4079134	chr5:4012356-4089499
chr5	4079140	4080482	chr5:4012356-4089499
chr5	4080660	4080953	chr5:4012356-4089499
chr5	4080995	4083385	chr5:4012356-4089499
chr5	4083390	4084367	chr5:4012356-4089499
chr5	4084665	4085518	chr5:4012356-4089499
chr5	4085520	4087350	chr5:4012356-4089499
chr5	4087500	4089530	chr5:4012356-4089499
chr5	4135720	4135977	chr5:4135750-4135958
chr5	4143335	4143910	chr5:4143359-4143881
chr5	4146630	4146755	chr5:4146673-4146707
chr5	4149335	4149451	chr5:4149375-4149391
chr5	25897718	25898902	chr5:25897743-25988138
chr5	25898903	25900106	chr5:25897743-25988138
chr5	25900128	25901109	chr5:25897743-25988138
chr5	25901413	25901705	chr5:25897743-25988138
chr5	25902013	25902596	chr5:25897743-25988138
chr5	25902603	25902916	chr5:25897743-25988138
chr5	25902928	25903106	chr5:25897743-25988138
chr5	25903133	25903901	chr5:25897743-25988138
chr5	25904238	25904642	chr5:25897743-25988138
chr5	25904973	25905309	chr5:25897743-25988138
chr5	25905453	25905530	chr5:25897743-25988138
chr5	25905653	25905835	chr5:25897743-25988138
chr5	25905868	25906860	chr5:25897743-25988138
chr5	25906878	25907167	chr5:25897743-25988138
chr5	25907223	25908202	chr5:25897743-25988138
chr5	25908318	25910656	chr5:25897743-25988138
chr5	25910663	25911711	chr5:25897743-25988138
chr5	25911713	25913392	chr5:25897743-25988138
chr5	25913463	25915946	chr5:25897743-25988138
chr5	25915973	25916126	chr5:25897743-25988138
chr5	25916258	25916694	chr5:25897743-25988138
chr5	25916708	25916844	chr5:25897743-25988138
chr5	25916993	25917179	chr5:25897743-25988138
chr5	25917843	25917941	chr5:25897743-25988138
chr5	25917963	25918052	chr5:25897743-25988138
chr5	25918123	25918311	chr5:25897743-25988138
chr5	25918378	25918490	chr5:25897743-25988138
chr5	25918588	25918663	chr5:25897743-25988138
chr5	25918738	25918827	chr5:25897743-25988138
chr5	25918893	25919116	chr5:25897743-25988138
chr5	25919193	25919304	chr5:25897743-25988138
chr5	25919339	25919408	chr5:25897743-25988138
chr5	25919574	25919642	chr5:25897743-25988138

chr5	25919684	25919764	chr5:25897743-25988138
chr5	25919879	25919957	chr5:25897743-25988138
chr5	25919969	25920052	chr5:25897743-25988138
chr5	25920059	25920202	chr5:25897743-25988138
chr5	25920224	25920297	chr5:25897743-25988138
chr5	25920414	25920528	chr5:25897743-25988138
chr5	25920669	25920752	chr5:25897743-25988138
chr5	25920854	25920937	chr5:25897743-25988138
chr5	25921349	25921434	chr5:25897743-25988138
chr5	25921719	25923256	chr5:25897743-25988138
chr5	25923259	25923387	chr5:25897743-25988138
chr5	25923404	25923856	chr5:25897743-25988138
chr5	25923859	25924575	chr5:25897743-25988138
chr5	25924584	25924677	chr5:25897743-25988138
chr5	25925014	25925229	chr5:25897743-25988138
chr5	25925239	25926465	chr5:25897743-25988138
chr5	25926664	25926805	chr5:25897743-25988138
chr5	25926819	25929772	chr5:25897743-25988138
chr5	25929864	25930035	chr5:25897743-25988138
chr5	25930064	25931609	chr5:25897743-25988138
chr5	25931634	25932043	chr5:25897743-25988138
chr5	25932044	25932267	chr5:25897743-25988138
chr5	25932574	25933897	chr5:25897743-25988138
chr5	25933899	25935388	chr5:25897743-25988138
chr5	25936189	25936405	chr5:25897743-25988138
chr5	25936409	25936997	chr5:25897743-25988138
chr5	25939579	25940656	chr5:25897743-25988138
chr5	25940659	25940881	chr5:25897743-25988138
chr5	25940889	25941929	chr5:25897743-25988138
chr5	25941934	25942229	chr5:25897743-25988138
chr5	25942559	25942643	chr5:25897743-25988138
chr5	25942659	25943855	chr5:25897743-25988138
chr5	25944059	25945327	chr5:25897743-25988138
chr5	25945334	25948483	chr5:25897743-25988138
chr5	25948489	25948711	chr5:25897743-25988138
chr5	25948714	25948857	chr5:25897743-25988138
chr5	25948869	25949147	chr5:25897743-25988138
chr5	25949174	25949553	chr5:25897743-25988138
chr5	25949569	25952600	chr5:25897743-25988138
chr5	25952864	25953277	chr5:25897743-25988138
chr5	25953339	25955433	chr5:25897743-25988138
chr5	25955434	25956509	chr5:25897743-25988138
chr5	25956514	25956948	chr5:25897743-25988138
chr5	25956949	25959571	chr5:25897743-25988138
chr5	25959579	25960444	chr5:25897743-25988138
chr5	25960449	25960597	chr5:25897743-25988138
chr5	25960649	25960869	chr5:25897743-25988138
chr5	25961199	25961417	chr5:25897743-25988138
chr5	25961459	25961523	chr5:25897743-25988138
chr5	25961524	25962896	chr5:25897743-25988138
chr5	25963244	25964076	chr5:25897743-25988138
chr5	25964079	25964543	chr5:25897743-25988138
chr5	25964564	25965393	chr5:25897743-25988138
chr5	25965429	25965639	chr5:25897743-25988138
chr5	25965664	25966516	chr5:25897743-25988138
chr5	25966529	25967008	chr5:25897743-25988138
chr5	25967314	25968737	chr5:25897743-25988138
chr5	25969314	25969505	chr5:25897743-25988138
chr5	25969704	25971007	chr5:25897743-25988138
chr5	25971044	25971318	chr5:25897743-25988138
chr5	25971334	25971433	chr5:25897743-25988138
chr5	25971689	25971927	chr5:25897743-25988138
chr5	25971929	25972794	chr5:25897743-25988138

chr5	25972799	25972920	chr5:25897743-25988138
chr5	25973219	25973622	chr5:25897743-25988138
chr5	25973889	25974615	chr5:25897743-25988138
chr5	25974619	25975544	chr5:25897743-25988138
chr5	25975554	25975912	chr5:25897743-25988138
chr5	25975919	25976451	chr5:25897743-25988138
chr5	25976459	25977486	chr5:25897743-25988138
chr5	25977489	25979038	chr5:25897743-25988138
chr5	25979054	25979548	chr5:25897743-25988138
chr5	25979599	25979840	chr5:25897743-25988138
chr5	25979934	25980019	chr5:25897743-25988138
chr5	25980064	25980291	chr5:25897743-25988138
chr5	25980294	25980371	chr5:25897743-25988138
chr5	25980439	25980516	chr5:25897743-25988138
chr5	25980819	25980899	chr5:25897743-25988138
chr5	25981294	25981508	chr5:25897743-25988138
chr5	25981514	25982472	chr5:25897743-25988138
chr5	25982479	25983996	chr5:25897743-25988138
chr5	25984084	25985162	chr5:25897743-25988138
chr5	25985379	25985462	chr5:25897743-25988138
chr5	25985484	25985594	chr5:25897743-25988138
chr5	25985724	25985804	chr5:25897743-25988138
chr5	25985834	25986153	chr5:25897743-25988138
chr5	25986184	25986524	chr5:25897743-25988138
chr5	25986544	25988149	chr5:25897743-25988138
chr5	25989304	25989997	chr5:25989311-25990124
chr5	25992354	25992832	chr5:25992378-25993084
chr5	25992839	25993119	chr5:25992378-25993084
chr5	25999804	26000264	chr5:25999820-26000252
chr5	26003309	26003384	chr5:26003273-26003360
chr5	26006379	26006508	chr5:26006401-26008482
chr5	26006514	26006885	chr5:26006401-26008482
chr5	26006894	26007808	chr5:26006401-26008482
chr5	26007809	26008501	chr5:26006401-26008482
chr5	26011999	26012100	chr5:26012038-26012066
chr5	26015409	26015583	chr5:26015343-26015854
chr5	26015654	26015823	chr5:26015343-26015854
chr5	26027719	26027935	chr5:26027740-26027915
chr5	26031779	26032276	chr5:26031802-26032243
chr5	26034824	26035521	chr5:26034858-26035517
chr5	26038644	26038990	chr5:26038670-26038982
chr5	26064704	26065019	chr5:26064728-26065002
chr5	26139919	26140000	chr5:26139960-26139973
chr5	27406614	27406896	chr5:27406639-27406877
chr5	27412679	27412792	chr5:27412712-27412796
chr5	27428239	27428340	chr5:27428266-27428320
chr5	27436224	27436550	chr5:27436259-27436532
chr5	27438494	27438598	chr5:27438517-27438637
chr5	27472359	27472644	chr5:27472387-27472626
chr5	27473204	27473958	chr5:27473229-27654445
chr5	27473979	27474581	chr5:27473229-27654445
chr5	27474589	27475260	chr5:27473229-27654445
chr5	27475279	27475440	chr5:27473229-27654445
chr5	27475554	27475633	chr5:27473229-27654445
chr5	27475634	27478277	chr5:27473229-27654445
chr5	27478284	27478695	chr5:27473229-27654445
chr5	27479494	27480358	chr5:27473229-27654445
chr5	27480364	27480930	chr5:27473229-27654445
chr5	27480939	27481030	chr5:27473229-27654445
chr5	27481324	27481874	chr5:27473229-27654445
chr5	27481879	27483301	chr5:27473229-27654445
chr5	27483324	27484679	chr5:27473229-27654445
chr5	27484819	27486335	chr5:27473229-27654445

chr5	27486339	27486473	chr5:27473229-27654445
chr5	27486509	27486584	chr5:27473229-27654445
chr5	27486709	27487301	chr5:27473229-27654445
chr5	27487314	27488177	chr5:27473229-27654445
chr5	27488179	27488481	chr5:27473229-27654445
chr5	27488489	27489118	chr5:27473229-27654445
chr5	27489129	27490311	chr5:27473229-27654445
chr5	27490404	27490517	chr5:27473229-27654445
chr5	27490519	27493491	chr5:27473229-27654445
chr5	27493779	27496034	chr5:27473229-27654445
chr5	27496314	27497152	chr5:27473229-27654445
chr5	27497154	27499083	chr5:27473229-27654445
chr5	27499094	27500367	chr5:27473229-27654445
chr5	27500939	27502046	chr5:27473229-27654445
chr5	27502199	27502279	chr5:27473229-27654445
chr5	27502339	27502411	chr5:27473229-27654445
chr5	27502599	27502669	chr5:27473229-27654445
chr5	27502684	27502765	chr5:27473229-27654445
chr5	27502774	27503020	chr5:27473229-27654445
chr5	27503039	27503132	chr5:27473229-27654445
chr5	27503149	27503237	chr5:27473229-27654445
chr5	27503279	27503493	chr5:27473229-27654445
chr5	27503559	27503671	chr5:27473229-27654445
chr5	27503899	27504085	chr5:27473229-27654445
chr5	27504309	27504386	chr5:27473229-27654445
chr5	27504394	27505276	chr5:27473229-27654445
chr5	27505534	27506483	chr5:27473229-27654445
chr5	27506719	27507079	chr5:27473229-27654445
chr5	27507084	27508085	chr5:27473229-27654445
chr5	27508364	27508685	chr5:27473229-27654445
chr5	27508724	27508806	chr5:27473229-27654445
chr5	27509094	27510433	chr5:27473229-27654445
chr5	27510544	27510666	chr5:27473229-27654445
chr5	27510704	27512016	chr5:27473229-27654445
chr5	27512334	27512607	chr5:27473229-27654445
chr5	27512609	27513497	chr5:27473229-27654445
chr5	27513499	27513890	chr5:27473229-27654445
chr5	27513894	27514236	chr5:27473229-27654445
chr5	27514399	27514489	chr5:27473229-27654445
chr5	27514639	27514720	chr5:27473229-27654445
chr5	27514724	27514873	chr5:27473229-27654445
chr5	27514879	27514997	chr5:27473229-27654445
chr5	27515024	27515154	chr5:27473229-27654445
chr5	27515194	27515306	chr5:27473229-27654445
chr5	27515314	27515439	chr5:27473229-27654445
chr5	27515469	27515679	chr5:27473229-27654445
chr5	27515879	27516067	chr5:27473229-27654445
chr5	27516074	27516229	chr5:27473229-27654445
chr5	27516369	27516458	chr5:27473229-27654445
chr5	27516534	27516721	chr5:27473229-27654445
chr5	27516774	27516858	chr5:27473229-27654445
chr5	27516864	27516950	chr5:27473229-27654445
chr5	27516964	27517056	chr5:27473229-27654445
chr5	27517064	27517151	chr5:27473229-27654445
chr5	27517184	27517316	chr5:27473229-27654445
chr5	27517349	27517429	chr5:27473229-27654445
chr5	27517434	27517765	chr5:27473229-27654445
chr5	27517774	27517926	chr5:27473229-27654445
chr5	27517929	27518012	chr5:27473229-27654445
chr5	27518144	27518231	chr5:27473229-27654445
chr5	27518364	27518885	chr5:27473229-27654445
chr5	27518909	27518999	chr5:27473229-27654445
chr5	27519119	27519200	chr5:27473229-27654445

chr5	27519219	27519483	chr5:27473229-27654445
chr5	27519554	27519688	chr5:27473229-27654445
chr5	27519704	27519779	chr5:27473229-27654445
chr5	27519859	27519977	chr5:27473229-27654445
chr5	27520024	27520128	chr5:27473229-27654445
chr5	27520154	27520304	chr5:27473229-27654445
chr5	27520364	27520441	chr5:27473229-27654445
chr5	27520779	27522301	chr5:27473229-27654445
chr5	27522594	27524107	chr5:27473229-27654445
chr5	27524364	27524739	chr5:27473229-27654445
chr5	27524864	27525243	chr5:27473229-27654445
chr5	27525289	27525830	chr5:27473229-27654445
chr5	27525889	27527027	chr5:27473229-27654445
chr5	27527034	27527886	chr5:27473229-27654445
chr5	27527894	27528198	chr5:27473229-27654445
chr5	27528214	27528289	chr5:27473229-27654445
chr5	27528339	27528597	chr5:27473229-27654445
chr5	27528599	27528976	chr5:27473229-27654445
chr5	27528979	27529106	chr5:27473229-27654445
chr5	27529109	27529453	chr5:27473229-27654445
chr5	27529459	27529535	chr5:27473229-27654445
chr5	27529539	27529760	chr5:27473229-27654445
chr5	27529764	27529895	chr5:27473229-27654445
chr5	27529899	27530533	chr5:27473229-27654445
chr5	27530534	27530613	chr5:27473229-27654445
chr5	27530624	27532748	chr5:27473229-27654445
chr5	27532754	27533719	chr5:27473229-27654445
chr5	27533724	27535225	chr5:27473229-27654445
chr5	27535269	27537030	chr5:27473229-27654445
chr5	27537179	27537563	chr5:27473229-27654445
chr5	27537564	27537876	chr5:27473229-27654445
chr5	27537904	27538327	chr5:27473229-27654445
chr5	27538334	27538484	chr5:27473229-27654445
chr5	27538554	27538658	chr5:27473229-27654445
chr5	27538979	27539563	chr5:27473229-27654445
chr5	27539569	27540256	chr5:27473229-27654445
chr5	27540264	27542162	chr5:27473229-27654445
chr5	27542164	27542397	chr5:27473229-27654445
chr5	27542404	27542912	chr5:27473229-27654445
chr5	27542979	27543061	chr5:27473229-27654445
chr5	27543499	27543581	chr5:27473229-27654445
chr5	27543689	27543769	chr5:27473229-27654445
chr5	27543829	27543929	chr5:27473229-27654445
chr5	27544099	27544235	chr5:27473229-27654445
chr5	27544354	27544481	chr5:27473229-27654445
chr5	27544504	27544584	chr5:27473229-27654445
chr5	27544614	27544803	chr5:27473229-27654445
chr5	27544969	27545072	chr5:27473229-27654445
chr5	27545159	27545947	chr5:27473229-27654445
chr5	27546004	27546107	chr5:27473229-27654445
chr5	27546114	27546320	chr5:27473229-27654445
chr5	27546324	27546539	chr5:27473229-27654445
chr5	27546594	27547112	chr5:27473229-27654445
chr5	27547114	27547321	chr5:27473229-27654445
chr5	27547369	27547482	chr5:27473229-27654445
chr5	27547599	27547670	chr5:27473229-27654445
chr5	27548504	27548580	chr5:27473229-27654445
chr5	27548679	27548900	chr5:27473229-27654445
chr5	27548949	27549166	chr5:27473229-27654445
chr5	27549174	27549480	chr5:27473229-27654445
chr5	27549509	27550041	chr5:27473229-27654445
chr5	27550049	27550313	chr5:27473229-27654445
chr5	27550339	27550502	chr5:27473229-27654445

chr5	27550539	27550994	chr5:27473229-27654445
chr5	27551054	27553012	chr5:27473229-27654445
chr5	27553054	27554410	chr5:27473229-27654445
chr5	27554414	27554500	chr5:27473229-27654445
chr5	27554544	27554780	chr5:27473229-27654445
chr5	27554784	27556486	chr5:27473229-27654445
chr5	27556504	27556983	chr5:27473229-27654445
chr5	27556989	27557105	chr5:27473229-27654445
chr5	27557139	27557705	chr5:27473229-27654445
chr5	27557729	27557822	chr5:27473229-27654445
chr5	27557989	27558100	chr5:27473229-27654445
chr5	27558289	27558390	chr5:27473229-27654445
chr5	27558524	27558829	chr5:27473229-27654445
chr5	27558849	27559319	chr5:27473229-27654445
chr5	27559324	27560126	chr5:27473229-27654445
chr5	27560129	27561645	chr5:27473229-27654445
chr5	27561979	27562086	chr5:27473229-27654445
chr5	27562364	27563311	chr5:27473229-27654445
chr5	27563319	27568154	chr5:27473229-27654445
chr5	27568159	27568397	chr5:27473229-27654445
chr5	27568399	27568757	chr5:27473229-27654445
chr5	27568764	27569696	chr5:27473229-27654445
chr5	27569699	27570147	chr5:27473229-27654445
chr5	27570149	27571508	chr5:27473229-27654445
chr5	27571544	27571920	chr5:27473229-27654445
chr5	27572069	27572497	chr5:27473229-27654445
chr5	27572514	27572801	chr5:27473229-27654445
chr5	27572869	27573059	chr5:27473229-27654445
chr5	27573074	27573307	chr5:27473229-27654445
chr5	27573309	27573392	chr5:27473229-27654445
chr5	27573439	27573531	chr5:27473229-27654445
chr5	27573634	27573730	chr5:27473229-27654445
chr5	27573754	27573902	chr5:27473229-27654445
chr5	27574019	27574151	chr5:27473229-27654445
chr5	27574204	27574271	chr5:27473229-27654445
chr5	27574369	27574591	chr5:27473229-27654445
chr5	27574964	27575037	chr5:27473229-27654445
chr5	27575044	27575694	chr5:27473229-27654445
chr5	27576014	27576291	chr5:27473229-27654445
chr5	27576304	27576980	chr5:27473229-27654445
chr5	27576984	27578549	chr5:27473229-27654445
chr5	27578554	27578629	chr5:27473229-27654445
chr5	27578639	27579226	chr5:27473229-27654445
chr5	27579244	27579524	chr5:27473229-27654445
chr5	27579534	27580372	chr5:27473229-27654445
chr5	27580609	27582512	chr5:27473229-27654445
chr5	27582514	27582820	chr5:27473229-27654445
chr5	27582824	27584673	chr5:27473229-27654445
chr5	27584674	27584787	chr5:27473229-27654445
chr5	27584849	27584996	chr5:27473229-27654445
chr5	27585109	27585192	chr5:27473229-27654445
chr5	27585204	27585285	chr5:27473229-27654445
chr5	27585544	27585647	chr5:27473229-27654445
chr5	27585769	27585847	chr5:27473229-27654445
chr5	27586064	27586177	chr5:27473229-27654445
chr5	27586289	27586477	chr5:27473229-27654445
chr5	27586479	27586754	chr5:27473229-27654445
chr5	27586809	27586891	chr5:27473229-27654445
chr5	27586934	27587009	chr5:27473229-27654445
chr5	27587079	27587159	chr5:27473229-27654445
chr5	27587194	27587355	chr5:27473229-27654445
chr5	27587574	27587893	chr5:27473229-27654445
chr5	27587929	27588243	chr5:27473229-27654445

chr5	27588244	27588756	chr5:27473229-27654445
chr5	27588794	27589254	chr5:27473229-27654445
chr5	27589264	27589710	chr5:27473229-27654445
chr5	27589714	27591947	chr5:27473229-27654445
chr5	27591949	27593898	chr5:27473229-27654445
chr5	27593904	27595647	chr5:27473229-27654445
chr5	27595679	27596182	chr5:27473229-27654445
chr5	27596244	27596308	chr5:27473229-27654445
chr5	27596369	27596462	chr5:27473229-27654445
chr5	27596544	27599641	chr5:27473229-27654445
chr5	27599664	27600399	chr5:27473229-27654445
chr5	27600429	27601009	chr5:27473229-27654445
chr5	27601104	27602425	chr5:27473229-27654445
chr5	27602429	27602733	chr5:27473229-27654445
chr5	27602734	27603235	chr5:27473229-27654445
chr5	27603299	27603393	chr5:27473229-27654445
chr5	27603399	27603833	chr5:27473229-27654445
chr5	27603834	27604138	chr5:27473229-27654445
chr5	27604139	27604683	chr5:27473229-27654445
chr5	27604684	27607033	chr5:27473229-27654445
chr5	27607039	27607278	chr5:27473229-27654445
chr5	27607554	27611952	chr5:27473229-27654445
chr5	27611994	27613462	chr5:27473229-27654445
chr5	27613624	27613699	chr5:27473229-27654445
chr5	27615009	27615091	chr5:27473229-27654445
chr5	27615144	27615219	chr5:27473229-27654445
chr5	27615594	27615684	chr5:27473229-27654445
chr5	27616729	27616813	chr5:27473229-27654445
chr5	27616814	27617127	chr5:27473229-27654445
chr5	27617139	27617221	chr5:27473229-27654445
chr5	27617224	27617311	chr5:27473229-27654445
chr5	27617319	27617395	chr5:27473229-27654445
chr5	27617404	27617483	chr5:27473229-27654445
chr5	27617539	27618228	chr5:27473229-27654445
chr5	27618249	27618907	chr5:27473229-27654445
chr5	27618929	27619039	chr5:27473229-27654445
chr5	27619314	27619976	chr5:27473229-27654445
chr5	27619979	27620332	chr5:27473229-27654445
chr5	27620339	27620432	chr5:27473229-27654445
chr5	27620434	27620519	chr5:27473229-27654445
chr5	27620629	27620956	chr5:27473229-27654445
chr5	27620959	27621260	chr5:27473229-27654445
chr5	27621269	27621751	chr5:27473229-27654445
chr5	27621769	27622962	chr5:27473229-27654445
chr5	27622974	27624257	chr5:27473229-27654445
chr5	27624259	27624443	chr5:27473229-27654445
chr5	27624479	27625533	chr5:27473229-27654445
chr5	27625884	27626936	chr5:27473229-27654445
chr5	27627224	27630420	chr5:27473229-27654445
chr5	27630479	27630822	chr5:27473229-27654445
chr5	27630909	27630989	chr5:27473229-27654445
chr5	27631054	27631135	chr5:27473229-27654445
chr5	27631214	27631345	chr5:27473229-27654445
chr5	27631349	27631434	chr5:27473229-27654445
chr5	27631479	27631565	chr5:27473229-27654445
chr5	27631909	27632016	chr5:27473229-27654445
chr5	27632059	27632235	chr5:27473229-27654445
chr5	27632434	27632745	chr5:27473229-27654445
chr5	27632804	27633126	chr5:27473229-27654445
chr5	27633204	27633649	chr5:27473229-27654445
chr5	27633744	27633825	chr5:27473229-27654445
chr5	27633829	27633950	chr5:27473229-27654445
chr5	27633994	27634074	chr5:27473229-27654445

chr5	27634094	27634557	chr5:27473229-27654445
chr5	27634574	27634812	chr5:27473229-27654445
chr5	27634829	27635088	chr5:27473229-27654445
chr5	27635089	27635277	chr5:27473229-27654445
chr5	27635284	27635437	chr5:27473229-27654445
chr5	27635469	27635798	chr5:27473229-27654445
chr5	27635904	27636124	chr5:27473229-27654445
chr5	27636129	27636276	chr5:27473229-27654445
chr5	27636609	27636699	chr5:27473229-27654445
chr5	27636934	27637334	chr5:27473229-27654445
chr5	27638059	27638420	chr5:27473229-27654445
chr5	27638424	27638551	chr5:27473229-27654445
chr5	27638649	27638782	chr5:27473229-27654445
chr5	27638809	27638968	chr5:27473229-27654445
chr5	27638999	27640211	chr5:27473229-27654445
chr5	27640214	27640412	chr5:27473229-27654445
chr5	27640539	27641279	chr5:27473229-27654445
chr5	27641304	27641520	chr5:27473229-27654445
chr5	27641569	27641692	chr5:27473229-27654445
chr5	27643644	27643948	chr5:27473229-27654445
chr5	27643954	27644065	chr5:27473229-27654445
chr5	27644074	27644416	chr5:27473229-27654445
chr5	27644419	27646246	chr5:27473229-27654445
chr5	27646249	27646326	chr5:27473229-27654445
chr5	27646334	27646479	chr5:27473229-27654445
chr5	27646499	27646933	chr5:27473229-27654445
chr5	27646949	27647155	chr5:27473229-27654445
chr5	27647174	27647402	chr5:27473229-27654445
chr5	27647409	27647766	chr5:27473229-27654445
chr5	27647814	27648248	chr5:27473229-27654445
chr5	27648254	27648614	chr5:27473229-27654445
chr5	27648944	27649051	chr5:27473229-27654445
chr5	27649064	27650066	chr5:27473229-27654445
chr5	27650074	27650638	chr5:27473229-27654445
chr5	27650649	27651102	chr5:27473229-27654445
chr5	27651104	27651180	chr5:27473229-27654445
chr5	27651189	27651290	chr5:27473229-27654445
chr5	27651619	27651729	chr5:27473229-27654445
chr5	27651929	27652005	chr5:27473229-27654445
chr5	27652269	27652376	chr5:27473229-27654445
chr5	27652579	27652656	chr5:27473229-27654445
chr5	27652709	27652797	chr5:27473229-27654445
chr5	27652804	27652896	chr5:27473229-27654445
chr5	27652909	27653122	chr5:27473229-27654445
chr5	27653179	27653312	chr5:27473229-27654445
chr5	27653384	27653457	chr5:27473229-27654445
chr5	27653464	27653653	chr5:27473229-27654445
chr5	27653674	27653848	chr5:27473229-27654445
chr5	27653849	27654463	chr5:27473229-27654445
chr5	40306178	40307424	chr5:40306202-40308804
chr5	40307428	40307554	chr5:40306202-40308804
chr5	40307813	40308005	chr5:40306202-40308804
chr5	40308023	40308833	chr5:40306202-40308804
chr5	40309058	40309196	chr5:40309085-40309174
chr5	40312933	40313117	chr5:40312968-40313094
chr5	40314948	40315056	chr5:40314997-40315002
chr5	40318273	40318411	chr5:40318302-40318374
chr5	40318613	40319175	chr5:40318639-40441645
chr5	40319178	40320186	chr5:40318639-40441645
chr5	40320508	40322849	chr5:40318639-40441645
chr5	40323013	40323329	chr5:40318639-40441645
chr5	40323668	40323857	chr5:40318639-40441645
chr5	40323863	40325756	chr5:40318639-40441645

chr5	40325828	40325925	chr5:40318639-40441645
chr5	40326183	40326458	chr5:40318639-40441645
chr5	40326563	40326642	chr5:40318639-40441645
chr5	40326718	40327184	chr5:40318639-40441645
chr5	40327218	40328032	chr5:40318639-40441645
chr5	40328033	40328150	chr5:40318639-40441645
chr5	40328253	40329408	chr5:40318639-40441645
chr5	40329463	40329735	chr5:40318639-40441645
chr5	40329738	40329813	chr5:40318639-40441645
chr5	40329823	40329914	chr5:40318639-40441645
chr5	40329923	40329990	chr5:40318639-40441645
chr5	40330018	40330373	chr5:40318639-40441645
chr5	40330383	40330513	chr5:40318639-40441645
chr5	40330518	40330736	chr5:40318639-40441645
chr5	40330818	40331576	chr5:40318639-40441645
chr5	40331638	40332173	chr5:40318639-40441645
chr5	40332183	40332312	chr5:40318639-40441645
chr5	40332328	40332414	chr5:40318639-40441645
chr5	40332473	40332626	chr5:40318639-40441645
chr5	40332648	40332767	chr5:40318639-40441645
chr5	40332768	40334262	chr5:40318639-40441645
chr5	40334293	40334397	chr5:40318639-40441645
chr5	40334443	40334553	chr5:40318639-40441645
chr5	40334608	40334712	chr5:40318639-40441645
chr5	40334718	40335573	chr5:40318639-40441645
chr5	40335613	40335899	chr5:40318639-40441645
chr5	40336128	40336216	chr5:40318639-40441645
chr5	40336303	40336383	chr5:40318639-40441645
chr5	40336508	40336629	chr5:40318639-40441645
chr5	40342278	40342360	chr5:40318639-40441645
chr5	40342893	40343029	chr5:40318639-40441645
chr5	40343118	40343716	chr5:40318639-40441645
chr5	40343753	40344221	chr5:40318639-40441645
chr5	40344233	40344716	chr5:40318639-40441645
chr5	40344808	40345921	chr5:40318639-40441645
chr5	40345923	40346905	chr5:40318639-40441645
chr5	40346913	40347606	chr5:40318639-40441645
chr5	40347608	40347973	chr5:40318639-40441645
chr5	40348048	40350174	chr5:40318639-40441645
chr5	40350178	40350549	chr5:40318639-40441645
chr5	40350558	40350760	chr5:40318639-40441645
chr5	40350763	40350834	chr5:40318639-40441645
chr5	40350883	40351007	chr5:40318639-40441645
chr5	40351218	40351298	chr5:40318639-40441645
chr5	40351303	40351386	chr5:40318639-40441645
chr5	40351388	40351483	chr5:40318639-40441645
chr5	40351488	40351621	chr5:40318639-40441645
chr5	40351653	40351735	chr5:40318639-40441645
chr5	40351893	40351962	chr5:40318639-40441645
chr5	40351963	40352080	chr5:40318639-40441645
chr5	40352458	40352536	chr5:40318639-40441645
chr5	40352693	40352822	chr5:40318639-40441645
chr5	40352823	40352902	chr5:40318639-40441645
chr5	40352908	40353041	chr5:40318639-40441645
chr5	40353223	40353319	chr5:40318639-40441645
chr5	40353323	40353435	chr5:40318639-40441645
chr5	40353678	40353767	chr5:40318639-40441645
chr5	40353828	40355130	chr5:40318639-40441645
chr5	40355133	40355449	chr5:40318639-40441645
chr5	40355463	40355896	chr5:40318639-40441645
chr5	40356078	40356195	chr5:40318639-40441645
chr5	40356338	40356736	chr5:40318639-40441645
chr5	40356743	40356831	chr5:40318639-40441645

chr5	40356858	40357593	chr5:40318639-40441645
chr5	40357618	40358142	chr5:40318639-40441645
chr5	40358423	40358501	chr5:40318639-40441645
chr5	40358523	40362470	chr5:40318639-40441645
chr5	40362748	40362900	chr5:40318639-40441645
chr5	40363098	40363182	chr5:40318639-40441645
chr5	40363188	40363961	chr5:40318639-40441645
chr5	40364103	40364180	chr5:40318639-40441645
chr5	40364318	40366041	chr5:40318639-40441645
chr5	40366053	40366590	chr5:40318639-40441645
chr5	40366628	40367186	chr5:40318639-40441645
chr5	40367188	40367402	chr5:40318639-40441645
chr5	40367518	40367594	chr5:40318639-40441645
chr5	40367733	40369426	chr5:40318639-40441645
chr5	40369438	40369513	chr5:40318639-40441645
chr5	40369523	40369838	chr5:40318639-40441645
chr5	40369848	40369920	chr5:40318639-40441645
chr5	40370428	40370580	chr5:40318639-40441645
chr5	40371368	40372715	chr5:40318639-40441645
chr5	40373088	40374743	chr5:40318639-40441645
chr5	40375018	40376944	chr5:40318639-40441645
chr5	40377073	40378414	chr5:40318639-40441645
chr5	40378438	40378626	chr5:40318639-40441645
chr5	40378628	40379918	chr5:40318639-40441645
chr5	40379993	40380560	chr5:40318639-40441645
chr5	40380568	40382941	chr5:40318639-40441645
chr5	40382943	40383763	chr5:40318639-40441645
chr5	40383778	40384413	chr5:40318639-40441645
chr5	40384423	40385342	chr5:40318639-40441645
chr5	40385348	40385550	chr5:40318639-40441645
chr5	40385553	40388014	chr5:40318639-40441645
chr5	40388298	40388403	chr5:40318639-40441645
chr5	40388478	40391881	chr5:40318639-40441645
chr5	40391888	40394662	chr5:40318639-40441645
chr5	40394668	40395798	chr5:40318639-40441645
chr5	40395803	40398694	chr5:40318639-40441645
chr5	40398698	40399030	chr5:40318639-40441645
chr5	40399033	40399186	chr5:40318639-40441645
chr5	40399203	40400086	chr5:40318639-40441645
chr5	40400088	40401272	chr5:40318639-40441645
chr5	40401363	40402552	chr5:40318639-40441645
chr5	40402563	40402716	chr5:40318639-40441645
chr5	40402803	40403476	chr5:40318639-40441645
chr5	40403488	40403846	chr5:40318639-40441645
chr5	40404288	40404390	chr5:40318639-40441645
chr5	40404538	40404971	chr5:40318639-40441645
chr5	40404973	40407716	chr5:40318639-40441645
chr5	40407993	40411971	chr5:40318639-40441645
chr5	40411973	40412475	chr5:40318639-40441645
chr5	40412613	40414488	chr5:40318639-40441645
chr5	40414493	40414966	chr5:40318639-40441645
chr5	40414993	40417345	chr5:40318639-40441645
chr5	40418473	40418552	chr5:40318639-40441645
chr5	40420818	40420891	chr5:40318639-40441645
chr5	40421313	40421393	chr5:40318639-40441645
chr5	40423428	40425393	chr5:40318639-40441645
chr5	40425823	40426502	chr5:40318639-40441645
chr5	40426558	40427131	chr5:40318639-40441645
chr5	40427213	40427378	chr5:40318639-40441645
chr5	40427668	40429484	chr5:40318639-40441645
chr5	40429498	40432000	chr5:40318639-40441645
chr5	40432018	40434938	chr5:40318639-40441645
chr5	40434943	40435428	chr5:40318639-40441645

chr5	40435453	40435733	chr5:40318639-40441645
chr5	40436003	40436575	chr5:40318639-40441645
chr5	40436603	40438303	chr5:40318639-40441645
chr5	40440163	40440870	chr5:40318639-40441645
chr5	40440878	40441678	chr5:40318639-40441645
chr5	45259398	45259648	chr5:45259348-45262912
chr5	45259673	45259838	chr5:45259348-45262912
chr5	45259843	45260237	chr5:45259348-45262912
chr5	45260243	45261862	chr5:45259348-45262912
chr5	45261863	45262942	chr5:45259348-45262912
chr5	45267168	45267374	chr5:45267190-45267355
chr5	45267888	45267985	chr5:45267792-45268115
chr5	45268003	45268151	chr5:45267792-45268115
chr5	45303668	45303954	chr5:45303700-45303941
chr5	45353168	45353365	chr5:45353201-45353348
chr5	45396558	45396846	chr5:45396593-45396812
chr5	45408973	45409541	chr5:45409003-45409502
chr5	45461923	45462134	chr5:45461947-45462109
chr5	45645263	45645741	chr5:45645286-45645710
chr5	45695748	45695982	chr5:45695770-45696253
chr5	45695983	45696264	chr5:45695770-45696253
chr5	60457769	60458020	chr5:60457800-60457993
chr5	60458329	60458427	chr5:60458351-60458403
chr5	60473149	60473254	chr5:60473189-60473211
chr5	60475509	60475679	chr5:60475530-60475654
chr5	60476154	60476289	chr5:60476178-60476265
chr5	60477709	60477778	chr5:60477736-60477917
chr5	60477834	60477947	chr5:60477736-60477917
chr5	60496359	60498830	chr5:60496392-60553181
chr5	60498904	60499225	chr5:60496392-60553181
chr5	60499239	60499662	chr5:60496392-60553181
chr5	60500609	60501049	chr5:60496392-60553181
chr5	60501154	60501238	chr5:60496392-60553181
chr5	60501259	60501374	chr5:60496392-60553181
chr5	60502609	60502718	chr5:60496392-60553181
chr5	60503034	60503112	chr5:60496392-60553181
chr5	60503629	60504975	chr5:60496392-60553181
chr5	60505084	60505188	chr5:60496392-60553181
chr5	60505254	60505752	chr5:60496392-60553181
chr5	60505769	60506002	chr5:60496392-60553181
chr5	60506294	60506393	chr5:60496392-60553181
chr5	60506394	60510431	chr5:60496392-60553181
chr5	60510714	60516775	chr5:60496392-60553181
chr5	60516784	60518373	chr5:60496392-60553181
chr5	60518509	60518605	chr5:60496392-60553181
chr5	60518629	60518697	chr5:60496392-60553181
chr5	60518734	60519641	chr5:60496392-60553181
chr5	60519649	60519764	chr5:60496392-60553181
chr5	60519799	60519897	chr5:60496392-60553181
chr5	60519909	60520040	chr5:60496392-60553181
chr5	60520044	60520559	chr5:60496392-60553181
chr5	60520564	60522003	chr5:60496392-60553181
chr5	60522144	60522700	chr5:60496392-60553181
chr5	60522704	60522974	chr5:60496392-60553181
chr5	60523064	60523868	chr5:60496392-60553181
chr5	60523919	60526297	chr5:60496392-60553181
chr5	60526339	60526531	chr5:60496392-60553181
chr5	60526639	60529617	chr5:60496392-60553181
chr5	60529854	60530299	chr5:60496392-60553181
chr5	60530314	60530765	chr5:60496392-60553181
chr5	60530769	60531453	chr5:60496392-60553181
chr5	60531749	60531932	chr5:60496392-60553181
chr5	60532764	60533002	chr5:60496392-60553181

chr5	60533019	60533413	chr5:60496392-60553181
chr5	60533434	60534290	chr5:60496392-60553181
chr5	60534369	60534690	chr5:60496392-60553181
chr5	60540659	60540729	chr5:60496392-60553181
chr5	60540829	60542213	chr5:60496392-60553181
chr5	60542224	60542650	chr5:60496392-60553181
chr5	60542659	60542965	chr5:60496392-60553181
chr5	60543254	60543338	chr5:60496392-60553181
chr5	60543339	60544066	chr5:60496392-60553181
chr5	60544069	60544263	chr5:60496392-60553181
chr5	60544544	60545289	chr5:60496392-60553181
chr5	60546674	60546894	chr5:60496392-60553181
chr5	60546959	60547036	chr5:60496392-60553181
chr5	60547269	60548636	chr5:60496392-60553181
chr5	60548639	60551739	chr5:60496392-60553181
chr5	60552064	60553034	chr5:60496392-60553181
chr5	60560579	60560782	chr5:60560603-60560756
chr5	60586604	60591597	chr5:60586625-60606371
chr5	60591599	60592079	chr5:60586625-60606371
chr5	60592294	60592862	chr5:60586625-60606371
chr5	60593239	60593345	chr5:60586625-60606371
chr5	60593359	60595382	chr5:60586625-60606371
chr5	60595399	60595898	chr5:60586625-60606371
chr5	60596219	60599258	chr5:60586625-60606371
chr5	60599259	60599658	chr5:60586625-60606371
chr5	60599864	60599947	chr5:60586625-60606371
chr5	60600234	60601446	chr5:60586625-60606371
chr5	60601709	60601804	chr5:60586625-60606371
chr5	60601809	60603102	chr5:60586625-60606371
chr5	60603144	60604005	chr5:60586625-60606371
chr5	60604039	60605434	chr5:60586625-60606371
chr5	60605439	60605552	chr5:60586625-60606371
chr5	60605664	60605777	chr5:60586625-60606371
chr5	60617239	60617628	chr5:60617266-60617614
chr5	60618139	60618453	chr5:60618168-60618616
chr5	60618454	60618633	chr5:60618168-60618616
chr5	60627559	60627839	chr5:60627591-60627805
chr5	62552694	62553244	chr5:62552717-62593623
chr5	62553519	62553686	chr5:62552717-62593623
chr5	62553979	62554335	chr5:62552717-62593623
chr5	62554609	62555031	chr5:62552717-62593623
chr5	62555109	62555303	chr5:62552717-62593623
chr5	62555344	62556958	chr5:62552717-62593623
chr5	62556959	62557109	chr5:62552717-62593623
chr5	62557139	62557220	chr5:62552717-62593623
chr5	62557599	62558306	chr5:62552717-62593623
chr5	62558309	62560455	chr5:62552717-62593623
chr5	62560459	62560795	chr5:62552717-62593623
chr5	62561559	62561639	chr5:62552717-62593623
chr5	62561649	62563341	chr5:62552717-62593623
chr5	62563399	62563762	chr5:62552717-62593623
chr5	62563849	62564347	chr5:62552717-62593623
chr5	62564354	62564430	chr5:62552717-62593623
chr5	62564434	62564782	chr5:62552717-62593623
chr5	62565209	62565363	chr5:62552717-62593623
chr5	62565364	62565568	chr5:62552717-62593623
chr5	62565584	62565744	chr5:62552717-62593623
chr5	62565754	62565993	chr5:62552717-62593623
chr5	62565994	62566321	chr5:62552717-62593623
chr5	62566329	62567317	chr5:62552717-62593623
chr5	62567514	62567693	chr5:62552717-62593623
chr5	62567759	62567829	chr5:62552717-62593623
chr5	62567924	62567997	chr5:62552717-62593623

chr5	62568134	62568212	chr5:62552717-62593623
chr5	62568224	62568296	chr5:62552717-62593623
chr5	62568299	62568428	chr5:62552717-62593623
chr5	62568464	62568687	chr5:62552717-62593623
chr5	62568689	62568769	chr5:62552717-62593623
chr5	62568774	62568923	chr5:62552717-62593623
chr5	62569034	62569124	chr5:62552717-62593623
chr5	62569279	62570697	chr5:62552717-62593623
chr5	62570824	62571688	chr5:62552717-62593623
chr5	62571959	62572371	chr5:62552717-62593623
chr5	62572379	62572930	chr5:62552717-62593623
chr5	62574984	62575082	chr5:62552717-62593623
chr5	62575154	62575275	chr5:62552717-62593623
chr5	62575279	62575408	chr5:62552717-62593623
chr5	62575474	62575580	chr5:62552717-62593623
chr5	62575974	62576148	chr5:62552717-62593623
chr5	62576149	62576229	chr5:62552717-62593623
chr5	62576264	62577738	chr5:62552717-62593623
chr5	62578424	62579076	chr5:62552717-62593623
chr5	62579084	62581845	chr5:62552717-62593623
chr5	62581874	62584522	chr5:62552717-62593623
chr5	62584529	62586154	chr5:62552717-62593623
chr5	62586164	62586704	chr5:62552717-62593623
chr5	62586794	62588292	chr5:62552717-62593623
chr5	62588294	62588982	chr5:62552717-62593623
chr5	62589104	62589533	chr5:62552717-62593623
chr5	62589534	62589791	chr5:62552717-62593623
chr5	62589794	62593648	chr5:62552717-62593623
chr5	88184844	88185332	chr5:88184867-88185297
chr5	88187959	88188139	chr5:88187983-88188094
chr5	88199944	88200057	chr5:88199972-88200020
chr5	88200999	88201116	chr5:88201025-88201087
chr5	88201484	88201625	chr5:88201518-88201590
chr5	88215419	88216119	chr5:88215454-88223420
chr5	88216559	88217505	chr5:88215454-88223420
chr5	88217549	88218902	chr5:88215454-88223420
chr5	88218909	88221841	chr5:88215454-88223420
chr5	88221884	88222092	chr5:88215454-88223420
chr5	88222124	88222200	chr5:88215454-88223420
chr5	88222209	88222271	chr5:88215454-88223420
chr5	88222544	88222722	chr5:88215454-88223420
chr5	88222774	88223441	chr5:88215454-88223420
chr5	88236604	88237204	chr5:88236629-88237187
chr5	88238799	88239189	chr5:88238832-88239160
chr5	88240139	88240594	chr5:88240172-88240561
chr5	88243924	88244060	chr5:88243952-88244032
chr5	88259714	88259828	chr5:88259766-88259771
chr5	88261634	88263131	chr5:88261657-88263096
chr5	88327549	88328142	chr5:88327574-88328110
chr5	88370889	88371165	chr5:88370912-88371148
chr5	88390754	88390875	chr5:88390775-88390846
chr5	88446314	88446427	chr5:88446349-88446412
chr5	88446519	88446620	chr5:88446540-88446594
chr5	88464344	88464515	chr5:88464066-88464485
chr5	88600539	88600799	chr5:88600566-88600988
chr5	88600804	88601011	chr5:88600566-88600988
chr5	88615979	88617583	chr5:88616004-88746745
chr5	88617584	88618738	chr5:88616004-88746745
chr5	88619064	88619550	chr5:88616004-88746745
chr5	88619564	88619748	chr5:88616004-88746745
chr5	88619769	88619853	chr5:88616004-88746745
chr5	88619854	88620696	chr5:88616004-88746745
chr5	88620699	88620845	chr5:88616004-88746745

chr5	88620884	88621274	chr5:88616004-88746745
chr5	88621639	88622088	chr5:88616004-88746745
chr5	88622094	88622852	chr5:88616004-88746745
chr5	88622859	88624131	chr5:88616004-88746745
chr5	88624164	88624260	chr5:88616004-88746745
chr5	88624954	88625345	chr5:88616004-88746745
chr5	88625354	88627363	chr5:88616004-88746745
chr5	88627859	88627934	chr5:88616004-88746745
chr5	88628524	88629226	chr5:88616004-88746745
chr5	88629279	88630383	chr5:88616004-88746745
chr5	88630389	88631060	chr5:88616004-88746745
chr5	88631069	88632500	chr5:88616004-88746745
chr5	88632529	88632618	chr5:88616004-88746745
chr5	88632999	88633120	chr5:88616004-88746745
chr5	88633134	88633513	chr5:88616004-88746745
chr5	88633519	88636934	chr5:88616004-88746745
chr5	88636944	88638688	chr5:88616004-88746745
chr5	88638689	88641984	chr5:88616004-88746745
chr5	88641989	88642347	chr5:88616004-88746745
chr5	88642354	88642852	chr5:88616004-88746745
chr5	88643139	88643761	chr5:88616004-88746745
chr5	88643769	88643914	chr5:88616004-88746745
chr5	88644539	88645783	chr5:88616004-88746745
chr5	88645794	88646133	chr5:88616004-88746745
chr5	88646139	88646496	chr5:88616004-88746745
chr5	88646504	88646893	chr5:88616004-88746745
chr5	88646904	88647990	chr5:88616004-88746745
chr5	88647999	88648219	chr5:88616004-88746745
chr5	88648224	88650949	chr5:88616004-88746745
chr5	88651229	88651745	chr5:88616004-88746745
chr5	88651749	88651820	chr5:88616004-88746745
chr5	88651934	88652123	chr5:88616004-88746745
chr5	88652184	88652704	chr5:88616004-88746745
chr5	88652969	88653410	chr5:88616004-88746745
chr5	88653464	88653562	chr5:88616004-88746745
chr5	88653579	88653656	chr5:88616004-88746745
chr5	88653679	88654193	chr5:88616004-88746745
chr5	88654199	88654873	chr5:88616004-88746745
chr5	88654879	88655846	chr5:88616004-88746745
chr5	88655869	88656094	chr5:88616004-88746745
chr5	88656109	88656362	chr5:88616004-88746745
chr5	88656364	88657562	chr5:88616004-88746745
chr5	88657569	88661383	chr5:88616004-88746745
chr5	88661394	88662312	chr5:88616004-88746745
chr5	88662319	88662964	chr5:88616004-88746745
chr5	88662979	88663069	chr5:88616004-88746745
chr5	88663119	88663813	chr5:88616004-88746745
chr5	88663814	88664450	chr5:88616004-88746745
chr5	88664459	88664536	chr5:88616004-88746745
chr5	88664594	88664662	chr5:88616004-88746745
chr5	88664669	88665036	chr5:88616004-88746745
chr5	88665064	88665143	chr5:88616004-88746745
chr5	88665149	88665475	chr5:88616004-88746745
chr5	88665614	88665854	chr5:88616004-88746745
chr5	88665974	88666154	chr5:88616004-88746745
chr5	88666219	88666323	chr5:88616004-88746745
chr5	88666569	88666649	chr5:88616004-88746745
chr5	88666749	88666822	chr5:88616004-88746745
chr5	88666874	88667175	chr5:88616004-88746745
chr5	88667214	88667411	chr5:88616004-88746745
chr5	88667434	88668262	chr5:88616004-88746745
chr5	88668269	88668363	chr5:88616004-88746745
chr5	88668549	88669850	chr5:88616004-88746745

chr5	88669874	88676748	chr5:88616004-88746745
chr5	88676759	88678465	chr5:88616004-88746745
chr5	88678624	88678700	chr5:88616004-88746745
chr5	88678799	88679424	chr5:88616004-88746745
chr5	88679529	88680033	chr5:88616004-88746745
chr5	88680034	88681781	chr5:88616004-88746745
chr5	88681794	88682117	chr5:88616004-88746745
chr5	88682199	88682782	chr5:88616004-88746745
chr5	88682789	88686785	chr5:88616004-88746745
chr5	88686864	88688068	chr5:88616004-88746745
chr5	88688069	88688596	chr5:88616004-88746745
chr5	88688609	88689757	chr5:88616004-88746745
chr5	88689759	88691301	chr5:88616004-88746745
chr5	88691304	88693659	chr5:88616004-88746745
chr5	88693664	88693776	chr5:88616004-88746745
chr5	88693799	88694247	chr5:88616004-88746745
chr5	88694254	88694506	chr5:88616004-88746745
chr5	88694509	88698954	chr5:88616004-88746745
chr5	88699234	88700116	chr5:88616004-88746745
chr5	88700134	88700641	chr5:88616004-88746745
chr5	88700679	88701177	chr5:88616004-88746745
chr5	88701184	88702414	chr5:88616004-88746745
chr5	88702419	88704579	chr5:88616004-88746745
chr5	88704584	88705284	chr5:88616004-88746745
chr5	88705339	88705425	chr5:88616004-88746745
chr5	88705449	88705656	chr5:88616004-88746745
chr5	88705669	88705900	chr5:88616004-88746745
chr5	88705909	88706074	chr5:88616004-88746745
chr5	88706079	88706167	chr5:88616004-88746745
chr5	88706209	88706431	chr5:88616004-88746745
chr5	88706464	88708227	chr5:88616004-88746745
chr5	88708229	88708638	chr5:88616004-88746745
chr5	88708639	88710887	chr5:88616004-88746745
chr5	88710889	88712522	chr5:88616004-88746745
chr5	88712669	88712753	chr5:88616004-88746745
chr5	88712839	88712965	chr5:88616004-88746745
chr5	88713294	88713372	chr5:88616004-88746745
chr5	88713444	88713571	chr5:88616004-88746745
chr5	88713724	88713835	chr5:88616004-88746745
chr5	88713839	88713911	chr5:88616004-88746745
chr5	88713954	88714073	chr5:88616004-88746745
chr5	88714509	88714591	chr5:88616004-88746745
chr5	88714729	88716784	chr5:88616004-88746745
chr5	88716789	88717386	chr5:88616004-88746745
chr5	88717914	88718235	chr5:88616004-88746745
chr5	88718264	88718337	chr5:88616004-88746745
chr5	88718344	88718446	chr5:88616004-88746745
chr5	88718474	88718551	chr5:88616004-88746745
chr5	88718684	88718754	chr5:88616004-88746745
chr5	88718789	88718904	chr5:88616004-88746745
chr5	88719429	88719492	chr5:88616004-88746745
chr5	88720104	88720187	chr5:88616004-88746745
chr5	88720379	88720450	chr5:88616004-88746745
chr5	88720489	88723619	chr5:88616004-88746745
chr5	88723664	88724209	chr5:88616004-88746745
chr5	88724214	88724362	chr5:88616004-88746745
chr5	88724369	88725353	chr5:88616004-88746745
chr5	88725634	88726253	chr5:88616004-88746745
chr5	88726259	88726617	chr5:88616004-88746745
chr5	88726634	88727433	chr5:88616004-88746745
chr5	88727439	88727866	chr5:88616004-88746745
chr5	88727874	88728861	chr5:88616004-88746745
chr5	88728894	88729954	chr5:88616004-88746745

chr5	88729959	88730636	chr5:88616004-88746745
chr5	88730639	88731348	chr5:88616004-88746745
chr5	88731354	88732484	chr5:88616004-88746745
chr5	88732489	88732792	chr5:88616004-88746745
chr5	88732799	88733681	chr5:88616004-88746745
chr5	88733689	88734482	chr5:88616004-88746745
chr5	88734484	88735329	chr5:88616004-88746745
chr5	88735359	88736041	chr5:88616004-88746745
chr5	88736049	88736367	chr5:88616004-88746745
chr5	88736369	88736436	chr5:88616004-88746745
chr5	88736474	88738272	chr5:88616004-88746745
chr5	88738274	88739224	chr5:88616004-88746745
chr5	88739234	88739408	chr5:88616004-88746745
chr5	88739409	88739657	chr5:88616004-88746745
chr5	88739669	88742582	chr5:88616004-88746745
chr5	88742589	88742880	chr5:88616004-88746745
chr5	88742914	88744127	chr5:88616004-88746745
chr5	88744134	88746750	chr5:88616004-88746745
chr5	88761459	88761632	chr5:88761482-88761592
chr5	88761704	88762227	chr5:88761732-88762215
chr5	88762329	88762506	chr5:88762353-88762488
chr5	102712196	102712924	chr5:102712223-102740931
chr5	102713061	102713139	chr5:102712223-102740931
chr5	102713471	102715116	chr5:102712223-102740931
chr5	102715161	102715531	chr5:102712223-102740931
chr5	102719936	102720034	chr5:102712223-102740931
chr5	102721011	102721087	chr5:102712223-102740931
chr5	102721576	102723198	chr5:102712223-102740931
chr5	102723946	102724476	chr5:102712223-102740931
chr5	102724481	102725571	chr5:102712223-102740931
chr5	102725576	102725909	chr5:102712223-102740931
chr5	102725921	102726912	chr5:102712223-102740931
chr5	102727186	102728991	chr5:102712223-102740931
chr5	102729011	102729891	chr5:102712223-102740931
chr5	102729916	102731268	chr5:102712223-102740931
chr5	102731546	102732254	chr5:102712223-102740931
chr5	102732261	102740211	chr5:102712223-102740931
chr5	102740216	102740759	chr5:102712223-102740931
chr5	102744606	102745107	chr5:102744641-102745088
chr5	102748076	102748259	chr5:102748104-102748212
chr5	102749441	102749608	chr5:102749462-102749572
chr5	151754543	151754796	chr5:151754574-151754774
chr5	151771078	151771612	chr5:151771092-151772062
chr5	151771623	151772075	chr5:151771092-151772062
chr5	151774998	151775166	chr5:151775019-151775145
chr5	151777598	151777739	chr5:151777620-151777705
chr5	151780083	151780648	chr5:151780114-151780613
chr5	151783923	151784736	chr5:151783948-151784840
chr5	151784758	151784856	chr5:151783948-151784840
chr5	151805758	151805869	chr5:151805779-151805843
chr5	151807883	151808030	chr5:151807917-151807998
chr5	151812713	151812828	chr5:151812738-151812929
chr5	151812888	151812966	chr5:151812738-151812929
chr5	151917243	151917394	chr5:151917264-151917370
chr5	151939883	151942081	chr5:151939905-151988543
chr5	151942083	151942387	chr5:151939905-151988543
chr5	151942393	151943011	chr5:151939905-151988543
chr5	151943338	151943815	chr5:151939905-151988543
chr5	151943833	151944612	chr5:151939905-151988543
chr5	151944618	151945466	chr5:151939905-151988543
chr5	151945473	151949274	chr5:151939905-151988543
chr5	151949573	151949928	chr5:151939905-151988543
chr5	151949938	151951000	chr5:151939905-151988543

chr5	151951043	151951135	chr5:151939905-151988543
chr5	151952438	151952590	chr5:151939905-151988543
chr5	151952683	151952765	chr5:151939905-151988543
chr5	151952783	151952864	chr5:151939905-151988543
chr5	151953523	151953600	chr5:151939905-151988543
chr5	151953773	151954220	chr5:151939905-151988543
chr5	151954223	151954549	chr5:151939905-151988543
chr5	151954568	151955077	chr5:151939905-151988543
chr5	151955323	151955857	chr5:151939905-151988543
chr5	151955903	151956002	chr5:151939905-151988543
chr5	151956023	151956884	chr5:151939905-151988543
chr5	151956893	151957719	chr5:151939905-151988543
chr5	151957728	151957965	chr5:151939905-151988543
chr5	151958003	151958670	chr5:151939905-151988543
chr5	151958718	151958873	chr5:151939905-151988543
chr5	151959173	151959271	chr5:151939905-151988543
chr5	151959408	151959494	chr5:151939905-151988543
chr5	151960543	151960624	chr5:151939905-151988543
chr5	151960658	151960797	chr5:151939905-151988543
chr5	151960973	151961088	chr5:151939905-151988543
chr5	151961183	151961300	chr5:151939905-151988543
chr5	151961533	151961606	chr5:151939905-151988543
chr5	151961923	151962040	chr5:151939905-151988543
chr5	151962163	151962497	chr5:151939905-151988543
chr5	151962523	151962644	chr5:151939905-151988543
chr5	151962688	151962869	chr5:151939905-151988543
chr5	151962963	151965479	chr5:151939905-151988543
chr5	151965763	151966297	chr5:151939905-151988543
chr5	151967463	151967533	chr5:151939905-151988543
chr5	151971943	151972805	chr5:151939905-151988543
chr5	151973028	151973713	chr5:151939905-151988543
chr5	151973763	151974230	chr5:151939905-151988543
chr5	151974233	151974806	chr5:151939905-151988543
chr5	151974838	151976951	chr5:151939905-151988543
chr5	151976968	151977991	chr5:151939905-151988543
chr5	151977998	151979357	chr5:151939905-151988543
chr5	151979358	151982414	chr5:151939905-151988543
chr5	151982428	151982793	chr5:151939905-151988543
chr5	151982803	151982875	chr5:151939905-151988543
chr5	151982913	151983763	chr5:151939905-151988543
chr5	151983773	151984289	chr5:151939905-151988543
chr5	151984308	151984601	chr5:151939905-151988543
chr5	151984868	151984947	chr5:151939905-151988543
chr5	151985023	151985245	chr5:151939905-151988543
chr5	151985278	151985392	chr5:151939905-151988543
chr5	151985418	151985531	chr5:151939905-151988543
chr5	151985583	151985718	chr5:151939905-151988543
chr5	151986063	151986209	chr5:151939905-151988543
chr5	151986228	151986312	chr5:151939905-151988543
chr5	151986318	151986969	chr5:151939905-151988543
chr5	151986973	151987687	chr5:151939905-151988543
chr5	151987803	151988561	chr5:151939905-151988543
chr5	151988648	151990678	chr5:151988682-152075050
chr5	151990838	151991415	chr5:151988682-152075050
chr5	151991433	151994104	chr5:151988682-152075050
chr5	151994108	151994491	chr5:151988682-152075050
chr5	151996858	151996939	chr5:151988682-152075050
chr5	151997003	151997260	chr5:151988682-152075050
chr5	151997313	151998059	chr5:151988682-152075050
chr5	151998063	151998197	chr5:151988682-152075050
chr5	151998303	151998387	chr5:151988682-152075050
chr5	151998393	151998616	chr5:151988682-152075050
chr5	151998628	151999595	chr5:151988682-152075050

chr5	151999598	152001710	chr5:151988682-152075050
chr5	152001713	152002667	chr5:151988682-152075050
chr5	152002923	152002997	chr5:151988682-152075050
chr5	152003278	152003647	chr5:151988682-152075050
chr5	152003658	152004022	chr5:151988682-152075050
chr5	152004323	152004395	chr5:151988682-152075050
chr5	152004678	152004796	chr5:151988682-152075050
chr5	152004798	152004986	chr5:151988682-152075050
chr5	152005033	152005145	chr5:151988682-152075050
chr5	152005148	152005311	chr5:151988682-152075050
chr5	152005318	152006086	chr5:151988682-152075050
chr5	152006093	152006833	chr5:151988682-152075050
chr5	152007123	152008555	chr5:151988682-152075050
chr5	152008753	152008873	chr5:151988682-152075050
chr5	152009503	152015106	chr5:151988682-152075050
chr5	152015113	152015339	chr5:151988682-152075050
chr5	152015428	152015840	chr5:151988682-152075050
chr5	152015843	152016697	chr5:151988682-152075050
chr5	152017013	152017755	chr5:151988682-152075050
chr5	152017763	152020047	chr5:151988682-152075050
chr5	152020053	152020219	chr5:151988682-152075050
chr5	152020478	152022324	chr5:151988682-152075050
chr5	152022333	152022423	chr5:151988682-152075050
chr5	152022438	152026839	chr5:151988682-152075050
chr5	152026853	152027860	chr5:151988682-152075050
chr5	152028058	152029917	chr5:151988682-152075050
chr5	152029923	152031885	chr5:151988682-152075050
chr5	152031943	152032887	chr5:151988682-152075050
chr5	152032943	152033464	chr5:151988682-152075050
chr5	152039448	152039647	chr5:151988682-152075050
chr5	152039648	152041986	chr5:151988682-152075050
chr5	152041988	152042342	chr5:151988682-152075050
chr5	152042353	152042443	chr5:151988682-152075050
chr5	152042528	152042783	chr5:151988682-152075050
chr5	152042798	152043119	chr5:151988682-152075050
chr5	152043123	152043668	chr5:151988682-152075050
chr5	152043678	152043844	chr5:151988682-152075050
chr5	152043848	152044054	chr5:151988682-152075050
chr5	152044188	152044687	chr5:151988682-152075050
chr5	152044693	152044808	chr5:151988682-152075050
chr5	152044813	152045295	chr5:151988682-152075050
chr5	152045298	152045449	chr5:151988682-152075050
chr5	152045648	152045726	chr5:151988682-152075050
chr5	152045778	152045851	chr5:151988682-152075050
chr5	152045923	152046183	chr5:151988682-152075050
chr5	152046423	152047045	chr5:151988682-152075050
chr5	152047138	152047739	chr5:151988682-152075050
chr5	152047748	152047844	chr5:151988682-152075050
chr5	152047848	152048276	chr5:151988682-152075050
chr5	152048308	152048563	chr5:151988682-152075050
chr5	152048568	152048825	chr5:151988682-152075050
chr5	152048828	152049555	chr5:151988682-152075050
chr5	152049558	152049651	chr5:151988682-152075050
chr5	152049673	152050053	chr5:151988682-152075050
chr5	152050398	152051796	chr5:151988682-152075050
chr5	152051798	152054011	chr5:151988682-152075050
chr5	152054018	152054910	chr5:151988682-152075050
chr5	152055028	152055169	chr5:151988682-152075050
chr5	152055178	152057010	chr5:151988682-152075050
chr5	152057283	152059724	chr5:151988682-152075050
chr5	152059728	152061727	chr5:151988682-152075050
chr5	152061733	152062039	chr5:151988682-152075050
chr5	152062088	152063704	chr5:151988682-152075050

chr5	152063708	152064718	chr5:151988682-152075050
chr5	152064763	152066416	chr5:151988682-152075050
chr5	152066483	152067227	chr5:151988682-152075050
chr5	152067268	152067525	chr5:151988682-152075050
chr5	152067838	152067940	chr5:151988682-152075050
chr5	152067948	152068028	chr5:151988682-152075050
chr5	152068038	152070235	chr5:151988682-152075050
chr5	152070238	152071431	chr5:151988682-152075050
chr5	152071683	152072139	chr5:151988682-152075050
chr5	152072178	152072255	chr5:151988682-152075050
chr5	152072273	152072651	chr5:151988682-152075050
chr5	152072698	152073729	chr5:151988682-152075050
chr5	152073733	152075073	chr5:151988682-152075050
chr5	152099988	152100204	chr5:152100014-152100170
chr5	152177113	152177184	chr5:152177113-152177823
chr5	152177523	152177598	chr5:152177113-152177823
chr5	152199783	152199885	chr5:152199804-152199860
chr5	152264963	152265221	chr5:152264985-152265188
chr5	152318373	152318575	chr5:152318397-152318551
chr5	152345768	152345925	chr5:152345800-152345894
chr5	152346608	152346714	chr5:152346652-152346666
chr5	152351248	152351383	chr5:152351273-152351346
chr5	152351543	152351767	chr5:152351573-152351793
chr5	152352608	152352863	chr5:152352637-152352834
chr5	152448243	152448401	chr5:152448271-152448373
chr5	152505623	152506582	chr5:152505645-152610222
chr5	152506628	152506870	chr5:152505645-152610222
chr5	152506873	152509820	chr5:152505645-152610222
chr5	152509898	152510191	chr5:152505645-152610222
chr5	152510193	152510908	chr5:152505645-152610222
chr5	152511393	152511515	chr5:152505645-152610222
chr5	152511763	152511835	chr5:152505645-152610222
chr5	152511838	152512303	chr5:152505645-152610222
chr5	152512603	152513646	chr5:152505645-152610222
chr5	152513663	152514865	chr5:152505645-152610222
chr5	152514883	152515227	chr5:152505645-152610222
chr5	152515228	152515572	chr5:152505645-152610222
chr5	152515573	152518434	chr5:152505645-152610222
chr5	152518543	152519914	chr5:152505645-152610222
chr5	152519923	152520491	chr5:152505645-152610222
chr5	152520498	152521097	chr5:152505645-152610222
chr5	152521368	152523759	chr5:152505645-152610222
chr5	152523768	152523897	chr5:152505645-152610222
chr5	152523898	152524159	chr5:152505645-152610222
chr5	152524173	152524496	chr5:152505645-152610222
chr5	152524498	152524590	chr5:152505645-152610222
chr5	152524613	152525691	chr5:152505645-152610222
chr5	152525728	152526378	chr5:152505645-152610222
chr5	152526393	152526645	chr5:152505645-152610222
chr5	152526708	152526909	chr5:152505645-152610222
chr5	152526918	152527001	chr5:152505645-152610222
chr5	152527018	152527166	chr5:152505645-152610222
chr5	152527183	152527282	chr5:152505645-152610222
chr5	152527408	152527477	chr5:152505645-152610222
chr5	152527503	152528748	chr5:152505645-152610222
chr5	152528758	152528838	chr5:152505645-152610222
chr5	152528843	152530232	chr5:152505645-152610222
chr5	152530258	152530392	chr5:152505645-152610222
chr5	152530438	152530534	chr5:152505645-152610222
chr5	152530553	152530847	chr5:152505645-152610222
chr5	152530908	152531082	chr5:152505645-152610222
chr5	152531223	152531332	chr5:152505645-152610222
chr5	152531343	152531477	chr5:152505645-152610222

chr5	152531593	152531658	chr5:152505645-152610222
chr5	152531678	152531870	chr5:152505645-152610222
chr5	152531918	152532006	chr5:152505645-152610222
chr5	152532178	152532252	chr5:152505645-152610222
chr5	152532648	152532732	chr5:152505645-152610222
chr5	152533288	152533402	chr5:152505645-152610222
chr5	152533453	152533536	chr5:152505645-152610222
chr5	152533728	152533814	chr5:152505645-152610222
chr5	152533848	152533926	chr5:152505645-152610222
chr5	152534083	152534447	chr5:152505645-152610222
chr5	152534448	152536676	chr5:152505645-152610222
chr5	152536678	152538055	chr5:152505645-152610222
chr5	152538068	152541122	chr5:152505645-152610222
chr5	152541123	152541344	chr5:152505645-152610222
chr5	152541348	152543458	chr5:152505645-152610222
chr5	152543463	152543560	chr5:152505645-152610222
chr5	152543698	152544979	chr5:152505645-152610222
chr5	152544983	152546771	chr5:152505645-152610222
chr5	152547038	152547754	chr5:152505645-152610222
chr5	152547873	152549421	chr5:152505645-152610222
chr5	152549473	152549691	chr5:152505645-152610222
chr5	152549788	152549926	chr5:152505645-152610222
chr5	152549963	152550493	chr5:152505645-152610222
chr5	152550498	152551070	chr5:152505645-152610222
chr5	152551093	152551530	chr5:152505645-152610222
chr5	152552273	152552443	chr5:152505645-152610222
chr5	152552453	152552693	chr5:152505645-152610222
chr5	152552968	152553416	chr5:152505645-152610222
chr5	152553553	152553669	chr5:152505645-152610222
chr5	152553718	152553962	chr5:152505645-152610222
chr5	152553973	152554047	chr5:152505645-152610222
chr5	152554193	152554499	chr5:152505645-152610222
chr5	152554518	152554794	chr5:152505645-152610222
chr5	152555108	152555294	chr5:152505645-152610222
chr5	152555373	152555523	chr5:152505645-152610222
chr5	152555568	152555653	chr5:152505645-152610222
chr5	152555663	152555738	chr5:152505645-152610222
chr5	152555743	152555896	chr5:152505645-152610222
chr5	152555918	152556240	chr5:152505645-152610222
chr5	152556473	152556554	chr5:152505645-152610222
chr5	152556678	152556748	chr5:152505645-152610222
chr5	152556768	152556847	chr5:152505645-152610222
chr5	152557358	152557493	chr5:152505645-152610222
chr5	152557578	152557826	chr5:152505645-152610222
chr5	152557838	152558017	chr5:152505645-152610222
chr5	152558023	152559411	chr5:152505645-152610222
chr5	152559438	152559624	chr5:152505645-152610222
chr5	152563438	152566619	chr5:152505645-152610222
chr5	152566628	152566742	chr5:152505645-152610222
chr5	152566748	152567617	chr5:152505645-152610222
chr5	152567948	152568971	chr5:152505645-152610222
chr5	152568978	152570990	chr5:152505645-152610222
chr5	152571228	152571893	chr5:152505645-152610222
chr5	152572053	152572176	chr5:152505645-152610222
chr5	152572428	152573222	chr5:152505645-152610222
chr5	152573228	152573540	chr5:152505645-152610222
chr5	152573858	152577145	chr5:152505645-152610222
chr5	152577158	152578935	chr5:152505645-152610222
chr5	152579293	152579428	chr5:152505645-152610222
chr5	152579443	152580495	chr5:152505645-152610222
chr5	152580503	152580580	chr5:152505645-152610222
chr5	152580588	152582189	chr5:152505645-152610222
chr5	152582498	152584467	chr5:152505645-152610222

chr5	152584838	152586285	chr5:152505645-152610222
chr5	152586543	152587077	chr5:152505645-152610222
chr5	152587083	152587583	chr5:152505645-152610222
chr5	152587588	152587686	chr5:152505645-152610222
chr5	152587718	152587830	chr5:152505645-152610222
chr5	152587833	152588150	chr5:152505645-152610222
chr5	152588178	152588982	chr5:152505645-152610222
chr5	152589068	152590098	chr5:152505645-152610222
chr5	152590103	152591301	chr5:152505645-152610222
chr5	152591308	152591820	chr5:152505645-152610222
chr5	152591828	152592111	chr5:152505645-152610222
chr5	152592118	152593500	chr5:152505645-152610222
chr5	152593508	152593622	chr5:152505645-152610222
chr5	152593633	152594761	chr5:152505645-152610222
chr5	152594778	152594901	chr5:152505645-152610222
chr5	152594958	152595049	chr5:152505645-152610222
chr5	152595058	152595209	chr5:152505645-152610222
chr5	152595218	152595398	chr5:152505645-152610222
chr5	152595438	152595512	chr5:152505645-152610222
chr5	152597338	152597416	chr5:152505645-152610222
chr5	152599448	152599512	chr5:152505645-152610222
chr5	152600618	152601875	chr5:152505645-152610222
chr5	152601918	152603787	chr5:152505645-152610222
chr5	152603793	152604677	chr5:152505645-152610222
chr5	152604688	152605675	chr5:152505645-152610222
chr5	152605748	152605907	chr5:152505645-152610222
chr5	152606098	152606218	chr5:152505645-152610222
chr5	152606553	152606690	chr5:152505645-152610222
chr5	152606758	152606923	chr5:152505645-152610222
chr5	152609058	152609307	chr5:152505645-152610222
chr5	152609508	152609802	chr5:152505645-152610222
chr5	152609808	152610262	chr5:152505645-152610222
chr5	152610743	152612465	chr5:152610767-152675341
chr5	152612843	152615277	chr5:152610767-152675341
chr5	152615623	152617593	chr5:152610767-152675341
chr5	152617598	152618761	chr5:152610767-152675341
chr5	152618768	152619825	chr5:152610767-152675341
chr5	152620103	152623125	chr5:152610767-152675341
chr5	152623138	152623797	chr5:152610767-152675341
chr5	152623813	152624171	chr5:152610767-152675341
chr5	152624258	152624399	chr5:152610767-152675341
chr5	152625283	152625359	chr5:152610767-152675341
chr5	152625428	152625516	chr5:152610767-152675341
chr5	152626278	152626359	chr5:152610767-152675341
chr5	152626853	152626923	chr5:152610767-152675341
chr5	152627043	152627119	chr5:152610767-152675341
chr5	152627218	152627296	chr5:152610767-152675341
chr5	152627483	152627556	chr5:152610767-152675341
chr5	152627878	152627991	chr5:152610767-152675341
chr5	152628008	152628179	chr5:152610767-152675341
chr5	152628213	152628285	chr5:152610767-152675341
chr5	152628298	152628398	chr5:152610767-152675341
chr5	152628423	152628593	chr5:152610767-152675341
chr5	152628743	152629162	chr5:152610767-152675341
chr5	152629448	152629533	chr5:152610767-152675341
chr5	152629633	152629741	chr5:152610767-152675341
chr5	152629813	152629966	chr5:152610767-152675341
chr5	152630008	152630098	chr5:152610767-152675341
chr5	152630158	152630291	chr5:152610767-152675341
chr5	152630588	152630706	chr5:152610767-152675341
chr5	152630788	152630855	chr5:152610767-152675341
chr5	152630868	152631011	chr5:152610767-152675341
chr5	152631283	152631369	chr5:152610767-152675341

chr5	152631373	152631666	chr5:152610767-152675341
chr5	152631833	152631940	chr5:152610767-152675341
chr5	152631978	152632134	chr5:152610767-152675341
chr5	152632148	152632400	chr5:152610767-152675341
chr5	152632693	152632812	chr5:152610767-152675341
chr5	152632863	152634460	chr5:152610767-152675341
chr5	152634473	152636933	chr5:152610767-152675341
chr5	152636938	152640275	chr5:152610767-152675341
chr5	152640278	152641131	chr5:152610767-152675341
chr5	152641133	152641752	chr5:152610767-152675341
chr5	152641773	152642273	chr5:152610767-152675341
chr5	152642278	152643790	chr5:152610767-152675341
chr5	152643833	152645156	chr5:152610767-152675341
chr5	152645163	152646009	chr5:152610767-152675341
chr5	152646308	152646473	chr5:152610767-152675341
chr5	152646478	152647561	chr5:152610767-152675341
chr5	152647648	152647887	chr5:152610767-152675341
chr5	152647888	152649393	chr5:152610767-152675341
chr5	152649718	152649897	chr5:152610767-152675341
chr5	152649908	152650036	chr5:152610767-152675341
chr5	152650043	152650437	chr5:152610767-152675341
chr5	152650708	152651383	chr5:152610767-152675341
chr5	152651408	152654059	chr5:152610767-152675341
chr5	152654078	152654394	chr5:152610767-152675341
chr5	152654403	152654703	chr5:152610767-152675341
chr5	152654848	152656223	chr5:152610767-152675341
chr5	152656228	152656324	chr5:152610767-152675341
chr5	152656673	152658814	chr5:152610767-152675341
chr5	152658823	152658991	chr5:152610767-152675341
chr5	152659008	152660937	chr5:152610767-152675341
chr5	152661213	152661421	chr5:152610767-152675341
chr5	152661513	152661639	chr5:152610767-152675341
chr5	152661728	152662138	chr5:152610767-152675341
chr5	152662233	152662315	chr5:152610767-152675341
chr5	152662333	152662431	chr5:152610767-152675341
chr5	152662693	152662786	chr5:152610767-152675341
chr5	152662818	152662901	chr5:152610767-152675341
chr5	152663138	152663285	chr5:152610767-152675341
chr5	152663843	152663929	chr5:152610767-152675341
chr5	152664683	152664765	chr5:152610767-152675341
chr5	152666133	152666206	chr5:152610767-152675341
chr5	152666903	152666972	chr5:152610767-152675341
chr5	152668903	152669082	chr5:152610767-152675341
chr5	152669523	152670147	chr5:152610767-152675341
chr5	152674923	152675158	chr5:152610767-152675341
chr5	152677393	152677462	chr5:152675342-152866451
chr5	152677468	152678097	chr5:152675342-152866451
chr5	152678098	152679564	chr5:152675342-152866451
chr5	152679583	152679996	chr5:152675342-152866451
chr5	152685248	152685327	chr5:152675342-152866451
chr5	152686123	152687811	chr5:152675342-152866451
chr5	152687818	152689536	chr5:152675342-152866451
chr5	152689543	152690029	chr5:152675342-152866451
chr5	152690058	152693693	chr5:152675342-152866451
chr5	152693728	152693860	chr5:152675342-152866451
chr5	152693863	152695025	chr5:152675342-152866451
chr5	152695468	152695725	chr5:152675342-152866451
chr5	152695728	152696976	chr5:152675342-152866451
chr5	152697253	152697417	chr5:152675342-152866451
chr5	152697423	152697522	chr5:152675342-152866451
chr5	152697523	152698391	chr5:152675342-152866451
chr5	152698393	152698942	chr5:152675342-152866451
chr5	152698958	152699319	chr5:152675342-152866451

chr5	152699323	152700072	chr5:152675342-152866451
chr5	152700358	152700972	chr5:152675342-152866451
chr5	152701013	152701681	chr5:152675342-152866451
chr5	152701708	152701779	chr5:152675342-152866451
chr5	152701823	152702182	chr5:152675342-152866451
chr5	152702233	152703603	chr5:152675342-152866451
chr5	152703843	152704897	chr5:152675342-152866451
chr5	152710948	152711140	chr5:152675342-152866451
chr5	152711413	152712684	chr5:152675342-152866451
chr5	152713238	152713310	chr5:152675342-152866451
chr5	152716048	152716121	chr5:152675342-152866451
chr5	152716921	152717001	chr5:152675342-152866451
chr5	152717156	152717223	chr5:152675342-152866451
chr5	152718171	152718251	chr5:152675342-152866451
chr5	152718496	152718600	chr5:152675342-152866451
chr5	152718696	152719290	chr5:152675342-152866451
chr5	152719291	152719577	chr5:152675342-152866451
chr5	152719581	152720734	chr5:152675342-152866451
chr5	152720736	152721053	chr5:152675342-152866451
chr5	152721106	152721379	chr5:152675342-152866451
chr5	152721381	152721630	chr5:152675342-152866451
chr5	152721636	152721703	chr5:152675342-152866451
chr5	152721736	152721980	chr5:152675342-152866451
chr5	152722016	152728029	chr5:152675342-152866451
chr5	152728036	152728566	chr5:152675342-152866451
chr5	152728576	152728653	chr5:152675342-152866451
chr5	152728676	152729020	chr5:152675342-152866451
chr5	152729031	152730896	chr5:152675342-152866451
chr5	152731191	152733529	chr5:152675342-152866451
chr5	152733531	152736506	chr5:152675342-152866451
chr5	152736511	152738521	chr5:152675342-152866451
chr5	152738531	152740495	chr5:152675342-152866451
chr5	152740501	152745042	chr5:152675342-152866451
chr5	152745046	152746390	chr5:152675342-152866451
chr5	152746401	152747012	chr5:152675342-152866451
chr5	152747026	152747679	chr5:152675342-152866451
chr5	152748061	152749750	chr5:152675342-152866451
chr5	152749801	152749996	chr5:152675342-152866451
chr5	152750301	152751846	chr5:152675342-152866451
chr5	152751881	152753693	chr5:152675342-152866451
chr5	152753741	152756651	chr5:152675342-152866451
chr5	152756676	152757220	chr5:152675342-152866451
chr5	152757511	152759174	chr5:152675342-152866451
chr5	152759176	152760969	chr5:152675342-152866451
chr5	152767096	152768065	chr5:152675342-152866451
chr5	152768071	152768342	chr5:152675342-152866451
chr5	152768376	152768625	chr5:152675342-152866451
chr5	152768711	152768785	chr5:152675342-152866451
chr5	152768786	152769323	chr5:152675342-152866451
chr5	152769326	152769713	chr5:152675342-152866451
chr5	152770061	152770430	chr5:152675342-152866451
chr5	152770431	152772206	chr5:152675342-152866451
chr5	152772211	152773061	chr5:152675342-152866451
chr5	152773066	152773700	chr5:152675342-152866451
chr5	152773706	152773961	chr5:152675342-152866451
chr5	152774236	152775021	chr5:152675342-152866451
chr5	152775041	152775898	chr5:152675342-152866451
chr5	152776226	152776778	chr5:152675342-152866451
chr5	152776811	152776941	chr5:152675342-152866451
chr5	152776961	152777193	chr5:152675342-152866451
chr5	152777201	152778052	chr5:152675342-152866451
chr5	152778066	152781617	chr5:152675342-152866451
chr5	152781876	152782024	chr5:152675342-152866451

chr5	152782031	152782631	chr5:152675342-152866451
chr5	152782636	152783216	chr5:152675342-152866451
chr5	152783256	152784787	chr5:152675342-152866451
chr5	152784791	152788086	chr5:152675342-152866451
chr5	152788091	152789407	chr5:152675342-152866451
chr5	152789416	152789537	chr5:152675342-152866451
chr5	152789551	152790312	chr5:152675342-152866451
chr5	152790316	152790696	chr5:152675342-152866451
chr5	152791026	152792542	chr5:152675342-152866451
chr5	152792741	152798631	chr5:152675342-152866451
chr5	152799256	152800319	chr5:152675342-152866451
chr5	152800346	152800497	chr5:152675342-152866451
chr5	152800501	152802425	chr5:152675342-152866451
chr5	152802436	152803281	chr5:152675342-152866451
chr5	152803336	152803424	chr5:152675342-152866451
chr5	152803481	152804046	chr5:152675342-152866451
chr5	152804411	152804539	chr5:152675342-152866451
chr5	152805341	152808049	chr5:152675342-152866451
chr5	152808271	152808981	chr5:152675342-152866451
chr5	152808986	152809314	chr5:152675342-152866451
chr5	152809316	152809946	chr5:152675342-152866451
chr5	152809956	152810718	chr5:152675342-152866451
chr5	152810721	152810857	chr5:152675342-152866451
chr5	152810871	152812318	chr5:152675342-152866451
chr5	152812336	152814077	chr5:152675342-152866451
chr5	152814366	152815772	chr5:152675342-152866451
chr5	152815776	152818289	chr5:152675342-152866451
chr5	152818311	152818384	chr5:152675342-152866451
chr5	152818386	152818536	chr5:152675342-152866451
chr5	152818541	152819355	chr5:152675342-152866451
chr5	152819361	152819714	chr5:152675342-152866451
chr5	152819726	152819854	chr5:152675342-152866451
chr5	152819876	152820585	chr5:152675342-152866451
chr5	152820586	152820890	chr5:152675342-152866451
chr5	152820891	152821051	chr5:152675342-152866451
chr5	152821061	152822099	chr5:152675342-152866451
chr5	152822137	152823081	chr5:152675342-152866451
chr5	152823127	152823200	chr5:152675342-152866451
chr5	152823497	152823645	chr5:152675342-152866451
chr5	152823757	152824100	chr5:152675342-152866451
chr5	152824147	152826149	chr5:152675342-152866451
chr5	152826157	152826526	chr5:152675342-152866451
chr5	152826532	152829273	chr5:152675342-152866451
chr5	152833887	152833962	chr5:152675342-152866451
chr5	152834427	152834501	chr5:152675342-152866451
chr5	152834982	152837308	chr5:152675342-152866451
chr5	152837317	152842892	chr5:152675342-152866451
chr5	152843137	152843209	chr5:152675342-152866451
chr5	152843227	152843322	chr5:152675342-152866451
chr5	152843332	152843571	chr5:152675342-152866451
chr5	152843747	152843946	chr5:152675342-152866451
chr5	152843957	152844076	chr5:152675342-152866451
chr5	152844092	152844175	chr5:152675342-152866451
chr5	152844177	152844448	chr5:152675342-152866451
chr5	152844467	152844785	chr5:152675342-152866451
chr5	152844797	152844929	chr5:152675342-152866451
chr5	152845022	152845476	chr5:152675342-152866451
chr5	152845727	152845872	chr5:152675342-152866451
chr5	152846162	152849470	chr5:152675342-152866451
chr5	152849512	152850163	chr5:152675342-152866451
chr5	152850172	152850673	chr5:152675342-152866451
chr5	152850962	152851061	chr5:152675342-152866451
chr5	152851167	152851460	chr5:152675342-152866451

chr5	152851582	152851694	chr5:152675342-152866451
chr5	152852437	152852530	chr5:152675342-152866451
chr5	152853362	152853451	chr5:152675342-152866451
chr5	152854217	152854292	chr5:152675342-152866451
chr5	152854482	152854552	chr5:152675342-152866451
chr5	152854597	152854692	chr5:152675342-152866451
chr5	152854717	152855398	chr5:152675342-152866451
chr5	152855422	152856252	chr5:152675342-152866451
chr5	152856257	152857575	chr5:152675342-152866451
chr5	152857667	152865318	chr5:152675342-152866451
chr5	152865327	152865444	chr5:152675342-152866451
chr5	152865447	152865600	chr5:152675342-152866451
chr5	152865617	152866220	chr5:152675342-152866451
chr5	152866237	152866485	chr5:152675342-152866451
chr5	152869152	152869442	chr5:152869174-152869397
chr5	152869792	152870563	chr5:152869827-152870009;chr5:152870041-152870071;chr5:152870083-152870530
chr5	152870722	152870905	chr5:152870744-152870892
chr5	152871707	152871889	chr5:152871731-152871870
chr5	152873452	152873916	chr5:152873487-152873894
chr5	152889282	152889392	chr5:152889309-152889366
chr5	152904717	152905016	chr5:152904739-152905082
chr5	152937322	152937646	chr5:152937357-152937655
chr5	152952757	152952865	chr5:152952787-152952832
chr5	152953882	152953978	chr5:152953908-152953951
chr5	152997467	152998034	chr5:152997500-152997999
chr5	153026452	153026744	chr5:153026487-153026727
chr5	153029857	153030111	chr5:153029889-153030074
chr5	153035352	153035471	chr5:153035378-153035432
chr5	153054027	153054248	chr5:153054059-153054221
chr5	153056522	153056741	chr5:153056553-153056721
chr5	153065757	153065895	chr5:153065784-153065889
chr5	153077572	153077756	chr5:153077596-153077714
chr5	153078397	153078642	chr5:153078426-153078633
chr5	153085247	153085678	chr5:153085256-153085732
chr5	153085697	153085770	chr5:153085256-153085732
chr5	153143972	153144213	chr5:153143993-153144192
chr5	153149702	153150011	chr5:153149727-153149975
chr5	153174152	153174320	chr5:153174180-153174295
chr5	153175007	153175180	chr5:153175035-153175150
chr5	153181892	153182067	chr5:153181915-153182050
chr5	153190557	153193453	chr5:153190584-153193429
chr5	158737699	158738058	chr5:158737728-158737766;chr5:158737808-158738022
chr5	158738194	158738432	chr5:158738215-158738422
chr5	158752519	158752688	chr5:158752541-158752662
chr5	158759764	158760037	chr5:158759789-158760011
chr5	158774494	158774634	chr5:158774523-158774613
chr5	158833674	158833826	chr5:158833708-158833791
chr5	158843509	158843679	chr5:158843531-158843649
chr5	158851009	158851170	chr5:158851030-158851138
chr5	158863424	158863534	chr5:158863454-158863501
chr5	158871624	158871765	chr5:158871657-158871731
chr5	158984774	158984880	chr5:158984803-158984865
chr5	158988234	158988375	chr5:158988268-158988371
chr5	158994494	158994610	chr5:158994532-158994566
chr5	159000244	159000379	chr5:159000268-159000344
chr5	159003089	159003196	chr5:159003110-159003175
chr5	159003284	159003461	chr5:159003311-159003439
chr5	159003824	159004084	chr5:159003852-159004054
chr5	159004219	159004335	chr5:159004249-159004286
chr5	159007184	159008150	chr5:159007210-159012921
chr5	159008164	159011518	chr5:159007210-159012921
chr5	159011549	159012320	chr5:159007210-159012921
chr5	159012339	159012494	chr5:159007210-159012921

chr5	159012499	159012615	chr5:159007210-159012921
chr5	159012619	159012961	chr5:159007210-159012921
chr5	159013849	159013959	chr5:159013872-159013933
chr5	159014029	159014150	chr5:159014057-159014097
chr5	159041519	159041625	chr5:159041551-159041602
chr5	159046329	159046635	chr5:159046350-159046604
chr5	159056399	159056515	chr5:159056411-159056486
chr6	1312650	1314708	chr6:1312674-1314993
chr6	1314710	1315018	chr6:1312674-1314993
chr6	21664974	21665255	chr6:21665002-21665244
chr6	21666644	21666883	chr6:21666674-21666876
chr6	21668854	21669166	chr6:21668877-21669130
chr6	21783839	21783973	chr6:21783862-21783939
chr6	21802864	21803010	chr6:21802887-21802971
chr6	21898919	21899034	chr6:21898950-21899015
chr6	21909679	21909852	chr6:21909700-21909834
chr6	21979149	21979330	chr6:21979173-21979291
chr6	22020534	22020782	chr6:22020567-22020771
chr6	22025519	22025627	chr6:22025548-22025586
chr6	22026169	22026266	chr6:22026197-22026236
chr6	22043969	22044152	chr6:22044002-22044123
chr6	22046729	22046830	chr6:22046770-22046786
chr6	22051684	22051857	chr6:22051706-22051819
chr6	22056749	22056963	chr6:22056774-22056920
chr6	22063224	22063515	chr6:22063248-22063469
chr6	22083784	22083966	chr6:22083811-22083947
chr6	22111014	22111182	chr6:22110975-22111157
chr6	22119789	22120603	chr6:22119819-22144059
chr6	22121929	22122088	chr6:22119819-22144059
chr6	22122089	22122863	chr6:22119819-22144059
chr6	22123164	22123877	chr6:22119819-22144059
chr6	22124164	22124385	chr6:22119819-22144059
chr6	22124719	22124820	chr6:22119819-22144059
chr6	22125109	22126579	chr6:22119819-22144059
chr6	22126649	22126727	chr6:22119819-22144059
chr6	22127399	22127475	chr6:22119819-22144059
chr6	22127479	22127555	chr6:22119819-22144059
chr6	22127809	22127883	chr6:22119819-22144059
chr6	22128289	22128506	chr6:22119819-22144059
chr6	22128854	22129005	chr6:22119819-22144059
chr6	22129009	22129434	chr6:22119819-22144059
chr6	22129444	22129643	chr6:22119819-22144059
chr6	22129644	22129715	chr6:22119819-22144059
chr6	22129804	22129947	chr6:22119819-22144059
chr6	22130424	22131056	chr6:22119819-22144059
chr6	22131359	22131787	chr6:22119819-22144059
chr6	22131854	22134056	chr6:22119819-22144059
chr6	22134069	22136893	chr6:22119819-22144059
chr6	22136894	22137225	chr6:22119819-22144059
chr6	22137239	22137797	chr6:22119819-22144059
chr6	22137799	22138971	chr6:22119819-22144059
chr6	22138974	22140095	chr6:22119819-22144059
chr6	22140409	22140998	chr6:22119819-22144059
chr6	22141299	22142227	chr6:22119819-22144059
chr6	22142524	22142890	chr6:22119819-22144059
chr6	22142919	22144063	chr6:22119819-22144059
chr6	22145704	22145833	chr6:22145742-22145786
chr6	22146829	22147533	chr6:22146862-22147521
chr6	22181524	22181604	chr6:22181551-22181654
chr6	22191789	22191923	chr6:22191795-22191885
chr6	22194244	22194612	chr6:22194273-22197448
chr6	22194629	22194926	chr6:22194273-22197448
chr6	22195174	22196516	chr6:22194273-22197448

chr6	22196849	22197470	chr6:22194273-22197448
chr6	22205389	22205527	chr6:22205413-22205488
chr6	22214504	22214755	chr6:22214535-22214734
chr6	22256579	22256715	chr6:22256602-22256698
chr6	22260554	22260769	chr6:22260564-22260727
chr6	22291084	22291259	chr6:22291106-22291217
chr6	22317034	22317142	chr6:22317055-22317108
chr6	22349429	22349741	chr6:22349457-22349722
chr6	22352754	22352909	chr6:22352789-22352880
chr6	22400729	22400912	chr6:22400757-22400872
chr6	22517889	22518033	chr6:22517913-22517999
chr6	22574339	22574622	chr6:22574347-22574584
chr6	22610764	22610909	chr6:22610792-22610869
chr6	28595179	28595282	chr6:28594286-28600433
chr6	28595304	28595411	chr6:28594286-28600433
chr6	28595539	28595920	chr6:28594286-28600433
chr6	28596234	28596903	chr6:28594286-28600433
chr6	28596904	28597103	chr6:28594286-28600433
chr6	28597439	28597650	chr6:28594286-28600433
chr6	28597994	28598102	chr6:28594286-28600433
chr6	28598199	28598319	chr6:28594286-28600433
chr6	28598649	28599222	chr6:28594286-28600433
chr6	28599509	28599661	chr6:28594286-28600433
chr6	28599964	28600458	chr6:28594286-28600433
chr6	28600574	28601102	chr6:28600599-28601807
chr6	28601114	28601837	chr6:28600599-28601807
chr6	28601954	28603584	chr6:28601978-28611171
chr6	28603589	28604157	chr6:28601978-28611171
chr6	28604269	28604491	chr6:28601978-28611171
chr6	28604494	28604929	chr6:28601978-28611171
chr6	28604959	28605074	chr6:28601978-28611171
chr6	28605079	28605992	chr6:28601978-28611171
chr6	28606304	28606469	chr6:28601978-28611171
chr6	28606569	28606975	chr6:28601978-28611171
chr6	28607579	28607659	chr6:28601978-28611171
chr6	28607769	28607924	chr6:28601978-28611171
chr6	28608064	28608198	chr6:28601978-28611171
chr6	28608511	28608824	chr6:28601978-28611171
chr6	28609256	28609385	chr6:28601978-28611171
chr6	28609386	28609908	chr6:28601978-28611171
chr6	28609911	28610091	chr6:28601978-28611171
chr6	28610381	28611154	chr6:28601978-28611171
chr6	28611321	28611535	chr6:28611343-28615933
chr6	28611941	28612726	chr6:28611343-28615933
chr6	28613156	28613264	chr6:28611343-28615933
chr6	28613286	28613368	chr6:28611343-28615933
chr6	28613386	28615250	chr6:28611343-28615933
chr6	28615266	28615956	chr6:28611343-28615933
chr6	28616086	28617703	chr6:28616107-28625963
chr6	28617711	28617843	chr6:28616107-28625963
chr6	28618341	28618747	chr6:28616107-28625963
chr6	28619026	28620873	chr6:28616107-28625963
chr6	28620931	28621166	chr6:28616107-28625963
chr6	28621211	28621326	chr6:28616107-28625963
chr6	28621726	28621797	chr6:28616107-28625963
chr6	28621831	28621935	chr6:28616107-28625963
chr6	28622141	28622297	chr6:28616107-28625963
chr6	28622301	28622486	chr6:28616107-28625963
chr6	28622751	28622956	chr6:28616107-28625963
chr6	28622971	28623728	chr6:28616107-28625963
chr6	28623736	28623830	chr6:28616107-28625963
chr6	28623831	28624440	chr6:28616107-28625963
chr6	28624461	28625080	chr6:28616107-28625963

chr6	28625086	28625640	chr6:28616107-28625963
chr6	28625866	28626000	chr6:28616107-28625963
chr6	28626086	28626150	chr6:28626135-28641562
chr6	28626161	28627230	chr6:28626135-28641562
chr6	28627241	28628046	chr6:28626135-28641562
chr6	28628276	28629774	chr6:28626135-28641562
chr6	28630026	28630148	chr6:28626135-28641562
chr6	28630441	28631177	chr6:28626135-28641562
chr6	28631456	28632035	chr6:28626135-28641562
chr6	28632606	28633211	chr6:28626135-28641562
chr6	28633441	28633929	chr6:28626135-28641562
chr6	28634211	28635280	chr6:28626135-28641562
chr6	28635561	28637260	chr6:28626135-28641562
chr6	28637866	28637965	chr6:28626135-28641562
chr6	28638006	28638428	chr6:28626135-28641562
chr6	28638701	28638807	chr6:28626135-28641562
chr6	28639571	28640346	chr6:28626135-28641562
chr6	28640431	28640791	chr6:28626135-28641562
chr6	28640901	28641554	chr6:28626135-28641562
chr6	28641701	28642667	chr6:28641734-28660936
chr6	28642716	28642813	chr6:28641734-28660936
chr6	28642861	28642944	chr6:28641734-28660936
chr6	28642976	28643131	chr6:28641734-28660936
chr6	28643161	28643326	chr6:28641734-28660936
chr6	28643391	28643482	chr6:28641734-28660936
chr6	28643491	28643746	chr6:28641734-28660936
chr6	28643796	28643960	chr6:28641734-28660936
chr6	28644266	28644546	chr6:28641734-28660936
chr6	28644866	28645232	chr6:28641734-28660936
chr6	28645246	28645586	chr6:28641734-28660936
chr6	28645896	28646338	chr6:28641734-28660936
chr6	28646416	28646507	chr6:28641734-28660936
chr6	28646536	28646669	chr6:28641734-28660936
chr6	28647251	28647324	chr6:28641734-28660936
chr6	28647331	28647773	chr6:28641734-28660936
chr6	28647801	28648793	chr6:28641734-28660936
chr6	28649066	28650353	chr6:28641734-28660936
chr6	28650716	28650853	chr6:28641734-28660936
chr6	28650861	28650943	chr6:28641734-28660936
chr6	28651201	28651355	chr6:28641734-28660936
chr6	28651446	28651640	chr6:28641734-28660936
chr6	28651641	28652013	chr6:28641734-28660936
chr6	28652041	28652385	chr6:28641734-28660936
chr6	28652436	28652584	chr6:28641734-28660936
chr6	28652596	28652851	chr6:28641734-28660936
chr6	28652936	28653264	chr6:28641734-28660936
chr6	28653266	28653369	chr6:28641734-28660936
chr6	28653461	28653786	chr6:28641734-28660936
chr6	28653846	28654193	chr6:28641734-28660936
chr6	28654236	28654495	chr6:28641734-28660936
chr6	28654651	28654726	chr6:28641734-28660936
chr6	28654946	28655092	chr6:28641734-28660936
chr6	28655126	28655209	chr6:28641734-28660936
chr6	28655376	28655461	chr6:28641734-28660936
chr6	28655641	28655793	chr6:28641734-28660936
chr6	28655881	28656013	chr6:28641734-28660936
chr6	28656106	28656178	chr6:28641734-28660936
chr6	28656211	28656418	chr6:28641734-28660936
chr6	28656481	28656693	chr6:28641734-28660936
chr6	28656731	28656807	chr6:28641734-28660936
chr6	28657041	28657121	chr6:28641734-28660936
chr6	28657181	28657258	chr6:28641734-28660936
chr6	28657271	28657366	chr6:28641734-28660936

chr6	28657576	28657651	chr6:28641734-28660936
chr6	28657736	28657897	chr6:28641734-28660936
chr6	28658016	28658153	chr6:28641734-28660936
chr6	28658226	28658340	chr6:28641734-28660936
chr6	28658451	28658889	chr6:28641734-28660936
chr6	28658936	28659074	chr6:28641734-28660936
chr6	28659106	28659195	chr6:28641734-28660936
chr6	28659241	28659316	chr6:28641734-28660936
chr6	28659321	28659419	chr6:28641734-28660936
chr6	28659596	28659670	chr6:28641734-28660936
chr6	28660176	28660248	chr6:28641734-28660936
chr6	28660356	28660477	chr6:28641734-28660936
chr6	28660801	28660946	chr6:28641734-28660936
chr6	28661076	28662808	chr6:28661110-28663658
chr6	28662861	28663242	chr6:28661110-28663658
chr6	28663526	28663704	chr6:28661110-28663658
chr6	28663836	28664925	chr6:28663830-28664893
chr6	28665021	28665946	chr6:28665043-28678315
chr6	28666231	28666724	chr6:28665043-28678315
chr6	28667076	28667801	chr6:28665043-28678315
chr6	28668086	28669396	chr6:28665043-28678315
chr6	28669691	28670181	chr6:28665043-28678315
chr6	28670426	28670523	chr6:28665043-28678315
chr6	28670526	28670777	chr6:28665043-28678315
chr6	28670831	28671883	chr6:28665043-28678315
chr6	28671891	28671958	chr6:28665043-28678315
chr6	28672036	28672324	chr6:28665043-28678315
chr6	28672331	28672682	chr6:28665043-28678315
chr6	28672736	28673383	chr6:28665043-28678315
chr6	28673396	28673878	chr6:28665043-28678315
chr6	28674186	28674503	chr6:28665043-28678315
chr6	28674791	28675107	chr6:28665043-28678315
chr6	28675111	28675206	chr6:28665043-28678315
chr6	28675576	28675953	chr6:28665043-28678315
chr6	28675956	28676384	chr6:28665043-28678315
chr6	28676391	28676743	chr6:28665043-28678315
chr6	28677051	28678346	chr6:28665043-28678315
chr6	28678466	28678705	chr6:28678487-28687430
chr6	28678706	28679559	chr6:28678487-28687430
chr6	28679831	28680372	chr6:28678487-28687430
chr6	28680866	28681171	chr6:28678487-28687430
chr6	28681746	28683641	chr6:28678487-28687430
chr6	28684246	28684968	chr6:28678487-28687430
chr6	28684971	28685334	chr6:28678487-28687430
chr6	28685621	28686271	chr6:28678487-28687430
chr6	28686601	28687450	chr6:28678487-28687430
chr6	28687571	28687820	chr6:28687602-28693744
chr6	28687846	28688375	chr6:28687602-28693744
chr6	28688661	28690016	chr6:28687602-28693744
chr6	28690291	28691344	chr6:28687602-28693744
chr6	28691356	28691575	chr6:28687602-28693744
chr6	28691581	28692289	chr6:28687602-28693744
chr6	28692321	28692710	chr6:28687602-28693744
chr6	28692991	28693755	chr6:28687602-28693744
chr6	28693886	28694098	chr6:28693918-28694804
chr6	28694101	28694840	chr6:28693918-28694804
chr6	28694951	28695155	chr6:28694977-28697041
chr6	28695266	28696338	chr6:28694977-28697041
chr6	28696511	28697062	chr6:28694977-28697041
chr6	28697186	28697541	chr6:28697213-28703154
chr6	28697566	28698089	chr6:28697213-28703154
chr6	28698356	28699987	chr6:28697213-28703154
chr6	28700016	28700100	chr6:28697213-28703154

chr6	28700226	28700750	chr6:28697213-28703154
chr6	28700836	28701896	chr6:28697213-28703154
chr6	28702156	28702596	chr6:28697213-28703154
chr6	28702606	28702677	chr6:28697213-28703154
chr6	28702901	28703138	chr6:28697213-28703154
chr6	28703306	28703617	chr6:28703327-28710678
chr6	28703631	28703857	chr6:28703327-28710678
chr6	28704066	28704142	chr6:28703327-28710678
chr6	28704311	28704396	chr6:28703327-28710678
chr6	28704416	28704562	chr6:28703327-28710678
chr6	28704716	28704937	chr6:28703327-28710678
chr6	28705231	28705596	chr6:28703327-28710678
chr6	28705706	28706615	chr6:28703327-28710678
chr6	28706626	28707143	chr6:28703327-28710678
chr6	28707876	28708049	chr6:28703327-28710678
chr6	28708336	28708409	chr6:28703327-28710678
chr6	28708721	28708868	chr6:28703327-28710678
chr6	28708871	28709341	chr6:28703327-28710678
chr6	28709361	28709809	chr6:28703327-28710678
chr6	28710426	28710655	chr6:28703327-28710678
chr6	28710816	28711417	chr6:28710851-28713877
chr6	28711446	28711607	chr6:28710851-28713877
chr6	28711611	28712412	chr6:28710851-28713877
chr6	28712416	28713129	chr6:28710851-28713877
chr6	28713171	28713283	chr6:28710851-28713877
chr6	28713351	28713489	chr6:28710851-28713877
chr6	28713491	28713560	chr6:28710851-28713877
chr6	28713626	28713796	chr6:28710851-28713877
chr6	28756541	28756823	chr6:28756564-28756777
chr6	28757231	28757327	chr6:28757261-28757297
chr6	29920201	29920780	chr6:29920224-29920755
chr6	29942856	29943321	chr6:29942888-29943294
chr6	29944251	29945634	chr6:29944272-29945600
chr6	29968766	29970540	chr6:29968787-29970509
chr6	29970911	29971184	chr6:29970943-29971145
chr6	29974971	29975185	chr6:29974999-29975173
chr6	29975266	29975456	chr6:29975300-29975419
chr6	29976376	29976487	chr6:29976408-29976451
chr6	29976636	29976955	chr6:29976669-29976948
chr6	29977471	29977640	chr6:29977502-29977608
chr6	29989436	29989543	chr6:29989443-29989538
chr6	30002651	30002895	chr6:30002684-30002887
chr6	30003661	30003781	chr6:30003692-30003760
chr6	30025866	30026001	chr6:30025891-30025967
chr6	30028496	30029018	chr6:30028517-30028980
chr6	31327301	31328011	chr6:31327334-31328475
chr6	31328126	31328247	chr6:31327334-31328475
chr6	31368446	31368558	chr6:31368478-31368540
chr6	31430866	31431079	chr6:31430899-31431056
chr6	31431126	31433612	chr6:31431147-31433586
chr6	31583001	31583159	chr6:31583032-31583131
chr6	31583276	31583376	chr6:31583297-31583359
chr6	31583421	31583521	chr6:31583446-31583513
chr6	31583766	31583944	chr6:31583788-31583922
chr6	31584096	31584322	chr6:31584120-31584283
chr6	31584561	31584807	chr6:31584592-31584798
chr6	32520291	32520950	chr6:32520313-32520940
chr6	32561546	32561960	chr6:32561576-32580894
chr6	32564546	32564763	chr6:32561576-32580894
chr6	32564771	32565513	chr6:32561576-32580894
chr6	32565771	32566316	chr6:32561576-32580894
chr6	32566341	32566424	chr6:32561576-32580894
chr6	32566441	32566516	chr6:32561576-32580894

chr6	32566541	32566832	chr6:32561576-32580894
chr6	32566836	32566928	chr6:32561576-32580894
chr6	32566996	32567072	chr6:32561576-32580894
chr6	32567461	32567616	chr6:32561576-32580894
chr6	32567901	32567984	chr6:32561576-32580894
chr6	32568411	32568493	chr6:32561576-32580894
chr6	32568866	32568933	chr6:32561576-32580894
chr6	32569001	32569091	chr6:32561576-32580894
chr6	32569096	32569166	chr6:32561576-32580894
chr6	32569516	32571350	chr6:32561576-32580894
chr6	32571611	32572690	chr6:32561576-32580894
chr6	32572691	32573040	chr6:32561576-32580894
chr6	32573346	32573805	chr6:32561576-32580894
chr6	32573811	32574854	chr6:32561576-32580894
chr6	32575221	32576460	chr6:32561576-32580894
chr6	32576461	32576817	chr6:32561576-32580894
chr6	32577111	32578849	chr6:32561576-32580894
chr6	32578851	32579141	chr6:32561576-32580894
chr6	32579161	32579259	chr6:32561576-32580894
chr6	32579606	32579777	chr6:32561576-32580894
chr6	32579881	32579968	chr6:32561576-32580894
chr6	32579981	32580089	chr6:32561576-32580894
chr6	32580101	32580216	chr6:32561576-32580894
chr6	32580236	32580926	chr6:32561576-32580894
chr6	32586576	32586921	chr6:32586478-32586908
chr6	32588971	32589423	chr6:32588995-32592359
chr6	32589481	32589656	chr6:32588995-32592359
chr6	32589666	32591321	chr6:32588995-32592359
chr6	32591326	32592049	chr6:32588995-32592359
chr6	32592056	32592378	chr6:32588995-32592359
chr6	32595921	32596077	chr6:32595955-32596047
chr6	32599826	32599899	chr6:32599718-32599869
chr6	32600491	32600710	chr6:32600520-32600686
chr6	32605111	32605357	chr6:32605133-32605317
chr6	32606666	32607712	chr6:32606688-32607682
chr6	32609061	32609373	chr6:32609086-32609335
chr6	32609716	32610052	chr6:32609748-32610030
chr6	32610356	32610917	chr6:32610386-32611429
chr6	32610931	32611444	chr6:32610386-32611429
chr6	32626026	32626553	chr6:32626051-32626537
chr6	32627211	32628066	chr6:32627243-32628026
chr6	32628106	32628515	chr6:32628131-32628506
chr6	32628591	32628705	chr6:32628635-32628659
chr6	32629101	32629277	chr6:32629123-32629234
chr6	32629711	32630065	chr6:32629743-32630025
chr6	32632551	32632868	chr6:32632574-32632844
chr6	32634251	32634504	chr6:32634275-32634471
chr6	32636011	32636188	chr6:32636044-32636160
chr6	32638621	32638903	chr6:32638643-32651923
chr6	32638906	32638990	chr6:32638643-32651923
chr6	32639851	32639918	chr6:32638643-32651923
chr6	32640731	32640800	chr6:32638643-32651923
chr6	32642231	32642335	chr6:32638643-32651923
chr6	32643066	32643141	chr6:32638643-32651923
chr6	32643291	32643359	chr6:32638643-32651923
chr6	32643456	32643559	chr6:32638643-32651923
chr6	32644261	32644339	chr6:32638643-32651923
chr6	32644496	32644577	chr6:32638643-32651923
chr6	32644641	32644723	chr6:32638643-32651923
chr6	32646196	32646292	chr6:32638643-32651923
chr6	32646386	32646525	chr6:32638643-32651923
chr6	32646551	32646682	chr6:32638643-32651923
chr6	32646741	32646827	chr6:32638643-32651923

chr6	32646936	32647053	chr6:32638643-32651923
chr6	32647056	32647191	chr6:32638643-32651923
chr6	32647206	32648275	chr6:32638643-32651923
chr6	32648376	32648939	chr6:32638643-32651923
chr6	32648961	32649142	chr6:32638643-32651923
chr6	32649256	32649904	chr6:32638643-32651923
chr6	32650171	32650323	chr6:32638643-32651923
chr6	32650326	32651776	chr6:32638643-32651923
chr6	32651836	32651945	chr6:32638643-32651923
chr6	32659536	32660064	chr6:32659536-32672430
chr6	32660111	32660198	chr6:32659536-32672430
chr6	32660361	32660436	chr6:32659536-32672430
chr6	32660731	32660888	chr6:32659536-32672430
chr6	32660921	32661018	chr6:32659536-32672430
chr6	32661096	32661179	chr6:32659536-32672430
chr6	32661276	32661347	chr6:32659536-32672430
chr6	32661546	32661698	chr6:32659536-32672430
chr6	32661761	32661976	chr6:32659536-32672430
chr6	32662211	32662316	chr6:32659536-32672430
chr6	32662501	32662585	chr6:32659536-32672430
chr6	32662876	32662956	chr6:32659536-32672430
chr6	32663246	32663430	chr6:32659536-32672430
chr6	32663441	32663512	chr6:32659536-32672430
chr6	32663521	32665590	chr6:32659536-32672430
chr6	32665591	32666059	chr6:32659536-32672430
chr6	32666196	32666307	chr6:32659536-32672430
chr6	32666496	32667413	chr6:32659536-32672430
chr6	32667736	32669127	chr6:32659536-32672430
chr6	32669131	32670159	chr6:32659536-32672430
chr6	32670186	32671020	chr6:32659536-32672430
chr6	32671081	32671732	chr6:32659536-32672430
chr6	32672076	32672464	chr6:32659536-32672430
chr6	32673551	32673640	chr6:32673574-32682137
chr6	32673886	32674144	chr6:32673574-32682137
chr6	32674151	32676377	chr6:32673574-32682137
chr6	32676381	32676477	chr6:32673574-32682137
chr6	32676576	32676644	chr6:32673574-32682137
chr6	32677021	32677540	chr6:32673574-32682137
chr6	32677661	32677728	chr6:32673574-32682137
chr6	32677731	32677954	chr6:32673574-32682137
chr6	32678116	32678321	chr6:32673574-32682137
chr6	32678336	32678490	chr6:32673574-32682137
chr6	32678521	32679368	chr6:32673574-32682137
chr6	32679376	32679634	chr6:32673574-32682137
chr6	32679641	32679846	chr6:32673574-32682137
chr6	32679861	32679957	chr6:32673574-32682137
chr6	32680016	32682167	chr6:32673574-32682137
chr6	32685746	32686634	chr6:32685781-32686612
chr6	32709091	32709342	chr6:32709118-32709302
chr6	32712911	32713223	chr6:32712935-32713184
chr6	32713536	32713884	chr6:32713567-32713849
chr6	32713986	32714205	chr6:32714016-32714191
chr6	32714331	32714964	chr6:32714358-32714992
chr6	32723841	32724809	chr6:32723874-32725081
chr6	32724986	32725101	chr6:32723874-32725081
chr6	32725526	32725703	chr6:32725549-32725660
chr6	32726591	32726953	chr6:32726626-32726908
chr6	32729411	32729726	chr6:32729436-32729703
chr6	32731121	32731335	chr6:32731150-32731311
chr6	41331381	41331457	chr6:41331101-41341101
chr6	41331466	41331782	chr6:41331101-41341101
chr6	41331811	41331888	chr6:41331101-41341101
chr6	41332166	41332540	chr6:41331101-41341101

chr6	41332616	41332814	chr6:41331101-41341101
chr6	41332816	41332896	chr6:41331101-41341101
chr6	41332906	41332997	chr6:41331101-41341101
chr6	41333051	41333166	chr6:41331101-41341101
chr6	41333171	41333429	chr6:41331101-41341101
chr6	41333431	41333535	chr6:41331101-41341101
chr6	41333906	41334291	chr6:41331101-41341101
chr6	41334571	41334869	chr6:41331101-41341101
chr6	41334926	41335098	chr6:41331101-41341101
chr6	41335196	41335779	chr6:41331101-41341101
chr6	41336011	41337314	chr6:41331101-41341101
chr6	41337606	41337695	chr6:41331101-41341101
chr6	41337806	41337879	chr6:41331101-41341101
chr6	41338266	41339008	chr6:41331101-41341101
chr6	41339061	41340041	chr6:41331101-41341101
chr6	41340046	41341134	chr6:41331101-41341101
chr6	41349141	41349698	chr6:41349165-41349684
chr6	41349756	41349889	chr6:41349782-41350213
chr6	41350776	41350888	chr6:41350805-41350867
chr6	72596374	72596566	chr6:72596405-72596890
chr6	72596589	72596902	chr6:72596405-72596890
chr6	72678654	72678796	chr6:72678685-72678766
chr6	72806619	72806905	chr6:72806651-72806865
chr6	72809634	72809730	chr6:72809677-72809689
chr6	72885019	72885109	chr6:72885063-72885075
chr6	72889254	72889644	chr6:72889277-72889618
chr6	72891959	72892869	chr6:72891986-72892852
chr6	72922489	72922946	chr6:72922513-72922925
chr6	72926119	72926377	chr6:72926144-72926341
chr6	72926429	72926961	chr6:72926462-72926946
chr6	72943454	72943551	chr6:72943475-72943543
chr6	72945299	72945468	chr6:72945320-72945431
chr6	72947504	72947636	chr6:72947525-72947625
chr6	72951994	72952160	chr6:72952016-72952140
chr6	72955484	72955602	chr6:72955517-72955564
chr6	72957694	72957861	chr6:72957717-72957830
chr6	72960009	72960175	chr6:72960032-72960163
chr6	72960589	72960803	chr6:72960623-72960795
chr6	72960894	72961097	chr6:72960917-72961071
chr6	72962434	72962581	chr6:72962463-72962535
chr6	72967804	72968008	chr6:72967827-72967984
chr6	72968659	72968840	chr6:72968688-72968814
chr6	72970369	72970594	chr6:72970401-72973707
chr6	72970604	72971888	chr6:72970401-72973707
chr6	72971934	72973444	chr6:72970401-72973707
chr6	72974644	72974788	chr6:72974677-72974755
chr6	72975069	72975241	chr6:72975092-72975206
chr6	72975634	72976055	chr6:72975662-72976016
chr6	72984029	72984179	chr6:72984051-72984135
chr6	72993724	72993862	chr6:72993749-72993821
chr6	73000354	73000602	chr6:73000381-73000564
chr6	73001604	73001786	chr6:73001636-73001749
chr6	73005599	73005696	chr6:73005639-73005669
chr6	73016934	73017105	chr6:73016960-73017073
chr6	73023179	73023403	chr6:73023208-73023375
chr6	73043279	73043563	chr6:73043302-73043538
chr6	73064334	73064883	chr6:73064359-73064858
chr6	73075444	73075555	chr6:73075475-73075518
chr6	73076404	73076577	chr6:73076431-73076568
chr6	73100274	73100623	chr6:73100299-73100587
chr6	73102374	73102538	chr6:73102399-73102512
chr6	73107924	73108073	chr6:73107950-73108052
chr6	73108629	73108801	chr6:73108656-73108796

chr6	73110164	73110701	chr6:73110197-73112845
chr6	73110724	73110893	chr6:73110197-73112845
chr6	73110894	73111858	chr6:73110197-73112845
chr6	73111884	73112883	chr6:73110197-73112845
chr6	73143294	73144721	chr6:73143315-73170267
chr6	73144814	73144966	chr6:73143315-73170267
chr6	73144979	73145813	chr6:73143315-73170267
chr6	73145814	73146192	chr6:73143315-73170267
chr6	73146514	73146719	chr6:73143315-73170267
chr6	73146964	73147033	chr6:73143315-73170267
chr6	73147099	73149193	chr6:73143315-73170267
chr6	73149194	73150229	chr6:73143315-73170267
chr6	73150254	73150386	chr6:73143315-73170267
chr6	73150389	73153613	chr6:73143315-73170267
chr6	73153889	73154225	chr6:73143315-73170267
chr6	73154234	73155223	chr6:73143315-73170267
chr6	73155649	73157343	chr6:73143315-73170267
chr6	73157344	73157871	chr6:73143315-73170267
chr6	73158009	73158093	chr6:73143315-73170267
chr6	73158199	73158770	chr6:73143315-73170267
chr6	73158789	73159323	chr6:73143315-73170267
chr6	73159644	73160226	chr6:73143315-73170267
chr6	73160544	73161155	chr6:73143315-73170267
chr6	73161159	73162619	chr6:73143315-73170267
chr6	73162684	73162887	chr6:73143315-73170267
chr6	73162899	73162981	chr6:73143315-73170267
chr6	73162994	73163071	chr6:73143315-73170267
chr6	73163139	73163247	chr6:73143315-73170267
chr6	73163269	73163359	chr6:73143315-73170267
chr6	73163414	73163490	chr6:73143315-73170267
chr6	73163754	73163836	chr6:73143315-73170267
chr6	73163839	73164110	chr6:73143315-73170267
chr6	73164114	73165001	chr6:73143315-73170267
chr6	73165269	73165375	chr6:73143315-73170267
chr6	73165384	73165608	chr6:73143315-73170267
chr6	73165884	73166063	chr6:73143315-73170267
chr6	73166104	73166317	chr6:73143315-73170267
chr6	73166629	73166760	chr6:73143315-73170267
chr6	73166779	73166887	chr6:73143315-73170267
chr6	73166914	73167053	chr6:73143315-73170267
chr6	73167054	73170290	chr6:73143315-73170267
chr6	84262571	84263305	chr6:84262598-84264004
chr6	84263321	84264018	chr6:84262598-84264004
chr6	84265831	84265974	chr6:84265861-84265964
chr6	84269801	84269960	chr6:84269822-84269927
chr6	84270551	84270705	chr6:84270582-84270666
chr6	84284706	84284866	chr6:84284728-84284840
chr6	84285026	84285368	chr6:84285050-84285340
chr6	84285706	84285786	chr6:84285741-84285772
chr6	84290146	84290354	chr6:84290168-84290318
chr6	84291911	84292084	chr6:84291940-84292075
chr6	84297896	84298451	chr6:84297921-84298420
chr6	84300906	84301051	chr6:84300929-84301013
chr6	84302141	84302305	chr6:84302173-84302257
chr6	84302636	84302771	chr6:84302664-84302736
chr6	84302876	84303019	chr6:84302900-84302978
chr6	84303161	84303478	chr6:84303196-84303460
chr6	84304061	84304241	chr6:84304092-84304247
chr6	84310961	84311183	chr6:84310989-84311157
chr6	84311261	84311359	chr6:84311303-84311318
chr6	84315371	84315554	chr6:84315403-84315526
chr6	84317391	84317570	chr6:84317417-84317527
chr6	84320351	84320417	chr6:84320368-84320396

chr6	84321101	84321210	chr6:84321156-84321162
chr6	84324531	84324628	chr6:84324575-84324581
chr6	84326666	84326781	chr6:84326687-84326758
chr6	84332991	84333088	chr6:84333019-84333061
chr6	84350786	84350956	chr6:84350814-84350921
chr6	84366441	84366622	chr6:84366472-84366584
chr6	84368696	84368828	chr6:84368717-84368811
chr6	84371196	84371341	chr6:84371220-84371323
chr6	84372041	84372158	chr6:84372065-84372141
chr6	84375136	84375325	chr6:84375157-84375300
chr6	84388466	84388638	chr6:84388496-84388622
chr6	84395776	84395898	chr6:84395808-84395942
chr6	84395906	84395978	chr6:84395808-84395942
chr6	84396496	84396614	chr6:84396547-84396558
chr6	84399286	84399396	chr6:84399311-84399364
chr6	84399916	84400188	chr6:84399941-84400155
chr6	84417486	84417696	chr6:84417516-84417676
chr6	84417776	84417943	chr6:84417799-84417908
chr6	84418041	84419447	chr6:84418064-84419410
chr6	89673368	89673863	chr6:89673398-89673849
chr6	89695943	89696088	chr6:89695972-89696045
chr6	89701068	89701138	chr6:89701080-89701122
chr6	89708048	89708284	chr6:89708076-89735137
chr6	89709018	89709087	chr6:89708076-89735137
chr6	89709188	89709799	chr6:89708076-89735137
chr6	89710043	89710431	chr6:89708076-89735137
chr6	89710538	89710677	chr6:89708076-89735137
chr6	89710683	89710899	chr6:89708076-89735137
chr6	89711163	89711953	chr6:89708076-89735137
chr6	89712528	89713291	chr6:89708076-89735137
chr6	89713958	89714052	chr6:89708076-89735137
chr6	89714388	89714526	chr6:89708076-89735137
chr6	89714808	89714905	chr6:89708076-89735137
chr6	89714933	89715150	chr6:89708076-89735137
chr6	89715408	89715551	chr6:89708076-89735137
chr6	89715848	89715944	chr6:89708076-89735137
chr6	89715968	89716220	chr6:89708076-89735137
chr6	89716513	89717101	chr6:89708076-89735137
chr6	89717113	89717353	chr6:89708076-89735137
chr6	89717358	89717575	chr6:89708076-89735137
chr6	89718003	89718077	chr6:89708076-89735137
chr6	89718083	89718221	chr6:89708076-89735137
chr6	89718408	89718496	chr6:89708076-89735137
chr6	89718523	89718641	chr6:89708076-89735137
chr6	89718643	89719436	chr6:89708076-89735137
chr6	89719443	89719964	chr6:89708076-89735137
chr6	89719993	89720223	chr6:89708076-89735137
chr6	89720398	89720797	chr6:89708076-89735137
chr6	89720798	89721415	chr6:89708076-89735137
chr6	89721418	89721937	chr6:89708076-89735137
chr6	89722193	89722689	chr6:89708076-89735137
chr6	89722948	89723453	chr6:89708076-89735137
chr6	89724303	89724387	chr6:89708076-89735137
chr6	89724403	89724470	chr6:89708076-89735137
chr6	89725578	89725658	chr6:89708076-89735137
chr6	89725678	89725777	chr6:89708076-89735137
chr6	89725973	89726107	chr6:89708076-89735137
chr6	89726318	89726430	chr6:89708076-89735137
chr6	89727168	89727315	chr6:89708076-89735137
chr6	89727703	89727915	chr6:89708076-89735137
chr6	89727938	89728472	chr6:89708076-89735137
chr6	89728778	89728979	chr6:89708076-89735137
chr6	89729263	89729661	chr6:89708076-89735137

chr6	89729848	89730896	chr6:89708076-89735137
chr6	89731193	89733193	chr6:89708076-89735137
chr6	89733468	89734285	chr6:89708076-89735137
chr6	89734293	89734443	chr6:89708076-89735137
chr6	95098619	95098818	chr6:95098573-95156859
chr6	95098829	95100582	chr6:95098573-95156859
chr6	95100594	95101580	chr6:95098573-95156859
chr6	95101664	95101782	chr6:95098573-95156859
chr6	95101799	95101983	chr6:95098573-95156859
chr6	95102124	95106029	chr6:95098573-95156859
chr6	95106099	95107095	chr6:95098573-95156859
chr6	95107169	95109121	chr6:95098573-95156859
chr6	95109269	95109405	chr6:95098573-95156859
chr6	95109459	95109526	chr6:95098573-95156859
chr6	95109559	95109631	chr6:95098573-95156859
chr6	95109639	95110503	chr6:95098573-95156859
chr6	95110509	95112002	chr6:95098573-95156859
chr6	95112024	95112378	chr6:95098573-95156859
chr6	95112449	95112768	chr6:95098573-95156859
chr6	95112779	95113322	chr6:95098573-95156859
chr6	95113349	95113420	chr6:95098573-95156859
chr6	95113424	95113690	chr6:95098573-95156859
chr6	95113704	95113789	chr6:95098573-95156859
chr6	95113799	95113877	chr6:95098573-95156859
chr6	95113879	95113955	chr6:95098573-95156859
chr6	95113979	95114956	chr6:95098573-95156859
chr6	95114969	95115286	chr6:95098573-95156859
chr6	95115299	95116484	chr6:95098573-95156859
chr6	95116499	95117446	chr6:95098573-95156859
chr6	95118324	95118405	chr6:95098573-95156859
chr6	95118599	95118666	chr6:95098573-95156859
chr6	95118939	95119070	chr6:95098573-95156859
chr6	95119179	95120454	chr6:95098573-95156859
chr6	95120609	95123133	chr6:95098573-95156859
chr6	95123144	95124443	chr6:95098573-95156859
chr6	95124454	95125957	chr6:95098573-95156859
chr6	95125999	95127426	chr6:95098573-95156859
chr6	95127429	95127577	chr6:95098573-95156859
chr6	95127579	95128906	chr6:95098573-95156859
chr6	95128934	95129070	chr6:95098573-95156859
chr6	95129189	95129282	chr6:95098573-95156859
chr6	95129319	95129976	chr6:95098573-95156859
chr6	95130354	95130434	chr6:95098573-95156859
chr6	95130439	95130636	chr6:95098573-95156859
chr6	95130714	95130911	chr6:95098573-95156859
chr6	95130914	95131060	chr6:95098573-95156859
chr6	95131334	95131522	chr6:95098573-95156859
chr6	95131539	95131754	chr6:95098573-95156859
chr6	95131764	95131983	chr6:95098573-95156859
chr6	95132049	95132119	chr6:95098573-95156859
chr6	95132129	95132438	chr6:95098573-95156859
chr6	95132444	95132631	chr6:95098573-95156859
chr6	95132634	95132968	chr6:95098573-95156859
chr6	95133024	95133406	chr6:95098573-95156859
chr6	95133714	95133831	chr6:95098573-95156859
chr6	95133914	95133994	chr6:95098573-95156859
chr6	95134074	95134238	chr6:95098573-95156859
chr6	95134279	95134345	chr6:95098573-95156859
chr6	95134399	95134548	chr6:95098573-95156859
chr6	95134549	95134628	chr6:95098573-95156859
chr6	95134654	95134837	chr6:95098573-95156859
chr6	95134839	95134944	chr6:95098573-95156859
chr6	95134974	95135221	chr6:95098573-95156859

chr6	95135309	95135477	chr6:95098573-95156859
chr6	95135479	95135702	chr6:95098573-95156859
chr6	95135704	95137518	chr6:95098573-95156859
chr6	95137519	95137669	chr6:95098573-95156859
chr6	95137914	95138322	chr6:95098573-95156859
chr6	95138329	95139897	chr6:95098573-95156859
chr6	95139899	95141481	chr6:95098573-95156859
chr6	95143094	95143906	chr6:95098573-95156859
chr6	95144064	95144387	chr6:95098573-95156859
chr6	95144454	95144812	chr6:95098573-95156859
chr6	95144814	95149235	chr6:95098573-95156859
chr6	95149239	95149911	chr6:95098573-95156859
chr6	95149919	95150803	chr6:95098573-95156859
chr6	95150809	95150990	chr6:95098573-95156859
chr6	95150999	95151546	chr6:95098573-95156859
chr6	95151554	95152331	chr6:95098573-95156859
chr6	95152454	95152968	chr6:95098573-95156859
chr6	95152974	95154470	chr6:95098573-95156859
chr6	95154784	95155548	chr6:95098573-95156859
chr6	95155554	95155944	chr6:95098573-95156859
chr6	95155954	95156889	chr6:95098573-95156859
chr6	95157009	95157734	chr6:95157031-95174880
chr6	95157739	95158517	chr6:95157031-95174880
chr6	95158524	95158656	chr6:95157031-95174880
chr6	95158659	95158798	chr6:95157031-95174880
chr6	95158814	95158929	chr6:95157031-95174880
chr6	95159244	95160936	chr6:95157031-95174880
chr6	95160939	95161176	chr6:95157031-95174880
chr6	95161569	95161846	chr6:95157031-95174880
chr6	95162114	95162241	chr6:95157031-95174880
chr6	95162354	95162439	chr6:95157031-95174880
chr6	95162649	95162901	chr6:95157031-95174880
chr6	95162909	95163017	chr6:95157031-95174880
chr6	95163024	95163925	chr6:95157031-95174880
chr6	95163934	95165158	chr6:95157031-95174880
chr6	95165184	95167493	chr6:95157031-95174880
chr6	95167789	95168682	chr6:95157031-95174880
chr6	95168689	95168765	chr6:95157031-95174880
chr6	95168804	95168908	chr6:95157031-95174880
chr6	95168934	95169112	chr6:95157031-95174880
chr6	95169124	95169294	chr6:95157031-95174880
chr6	95169339	95169456	chr6:95157031-95174880
chr6	95169564	95169644	chr6:95157031-95174880
chr6	95169704	95169791	chr6:95157031-95174880
chr6	95169829	95169902	chr6:95157031-95174880
chr6	95169944	95170098	chr6:95157031-95174880
chr6	95170124	95170202	chr6:95157031-95174880
chr6	95170239	95170402	chr6:95157031-95174880
chr6	95170424	95170604	chr6:95157031-95174880
chr6	95170764	95170828	chr6:95157031-95174880
chr6	95170914	95170995	chr6:95157031-95174880
chr6	95171014	95171123	chr6:95157031-95174880
chr6	95171134	95171211	chr6:95157031-95174880
chr6	95171289	95171367	chr6:95157031-95174880
chr6	95171404	95171620	chr6:95157031-95174880
chr6	95171644	95171858	chr6:95157031-95174880
chr6	95171869	95173595	chr6:95157031-95174880
chr6	95173604	95174818	chr6:95157031-95174880
chr6	97994154	97994298	chr6:97994176-97994271
chr6	98064699	98064849	chr6:98064726-98064807
chr6	98065979	98066139	chr6:98066001-98066107
chr6	98076754	98076854	chr6:98076776-98076837
chr6	98081334	98081506	chr6:98081363-98081479

chr6	98150134	98150280	chr6:98150158-98150240
chr6	98155529	98155716	chr6:98155562-98155680
chr6	98188009	98188159	chr6:98188042-98188130
chr6	98190024	98190162	chr6:98190047-98190134
chr6	98198074	98198177	chr6:98198099-98198155
chr6	98201574	98201686	chr6:98201598-98201647
chr6	98238929	98239055	chr6:98238963-98239032
chr6	98264304	98264666	chr6:98264338-98264632
chr6	98275899	98276107	chr6:98275924-98276087
chr6	98333354	98333552	chr6:98333376-98333531
chr6	98386799	98386871	chr6:98386664-98386849
chr6	98411704	98411876	chr6:98411729-98411844
chr6	98417304	98417375	chr6:98417281-98417342
chr6	98420374	98420588	chr6:98420401-98420549
chr6	98421344	98421492	chr6:98421379-98421473
chr6	98423824	98423939	chr6:98423859-98423923
chr6	98427424	98427525	chr6:98427386-98427509
chr6	98440514	98440617	chr6:98440570-98440571
chr6	98446724	98446798	chr6:98446734-98446784
chr6	98467369	98467484	chr6:98467403-98467470
chr6	98473729	98473839	chr6:98473772-98473799
chr6	98481069	98481173	chr6:98481092-98481148
chr6	98482339	98482445	chr6:98482370-98482438
chr6	98484664	98484929	chr6:98484698-98484885
chr6	98487409	98487612	chr6:98487433-98487587
chr6	98489269	98489402	chr6:98489294-98489388
chr6	98500854	98500982	chr6:98500875-98500964
chr6	98517114	98517399	chr6:98517140-98517217;chr6:98517271-98517362
chr6	98517509	98517658	chr6:98517496-98517638
chr6	98523919	98524057	chr6:98523942-98524021
chr6	98546524	98547404	chr6:98546547-98751343
chr6	98547454	98547601	chr6:98546547-98751343
chr6	98547774	98547860	chr6:98546547-98751343
chr6	98548124	98548205	chr6:98546547-98751343
chr6	98549134	98549255	chr6:98546547-98751343
chr6	98549584	98549691	chr6:98546547-98751343
chr6	98549759	98549839	chr6:98546547-98751343
chr6	98549934	98550028	chr6:98546547-98751343
chr6	98550034	98550112	chr6:98546547-98751343
chr6	98550119	98550193	chr6:98546547-98751343
chr6	98550214	98550331	chr6:98546547-98751343
chr6	98550384	98550551	chr6:98546547-98751343
chr6	98550659	98550732	chr6:98546547-98751343
chr6	98550994	98551079	chr6:98546547-98751343
chr6	98551209	98551543	chr6:98546547-98751343
chr6	98551544	98551635	chr6:98546547-98751343
chr6	98551674	98553313	chr6:98546547-98751343
chr6	98553319	98553420	chr6:98546547-98751343
chr6	98553449	98555040	chr6:98546547-98751343
chr6	98555049	98555121	chr6:98546547-98751343
chr6	98555164	98555260	chr6:98546547-98751343
chr6	98555549	98555653	chr6:98546547-98751343
chr6	98555654	98555854	chr6:98546547-98751343
chr6	98555859	98556059	chr6:98546547-98751343
chr6	98556089	98556666	chr6:98546547-98751343
chr6	98556669	98557031	chr6:98546547-98751343
chr6	98557049	98557166	chr6:98546547-98751343
chr6	98557274	98557390	chr6:98546547-98751343
chr6	98557394	98557632	chr6:98546547-98751343
chr6	98557689	98558988	chr6:98546547-98751343
chr6	98559009	98559896	chr6:98546547-98751343
chr6	98559899	98560194	chr6:98546547-98751343
chr6	98560214	98560358	chr6:98546547-98751343

chr6	98560384	98560537	chr6:98546547-98751343
chr6	98560544	98561332	chr6:98546547-98751343
chr6	98561369	98563158	chr6:98546547-98751343
chr6	98563194	98563514	chr6:98546547-98751343
chr6	98563524	98566156	chr6:98546547-98751343
chr6	98566174	98567114	chr6:98546547-98751343
chr6	98567129	98567542	chr6:98546547-98751343
chr6	98567574	98569356	chr6:98546547-98751343
chr6	98569714	98570272	chr6:98546547-98751343
chr6	98570539	98570735	chr6:98546547-98751343
chr6	98570744	98571045	chr6:98546547-98751343
chr6	98571309	98576421	chr6:98546547-98751343
chr6	98576444	98576803	chr6:98546547-98751343
chr6	98576809	98578072	chr6:98546547-98751343
chr6	98578364	98578631	chr6:98546547-98751343
chr6	98578644	98579950	chr6:98546547-98751343
chr6	98579969	98583492	chr6:98546547-98751343
chr6	98583499	98583699	chr6:98546547-98751343
chr6	98583704	98584724	chr6:98546547-98751343
chr6	98584734	98587294	chr6:98546547-98751343
chr6	98587299	98587531	chr6:98546547-98751343
chr6	98587649	98588228	chr6:98546547-98751343
chr6	98588614	98588855	chr6:98546547-98751343
chr6	98589164	98589949	chr6:98546547-98751343
chr6	98590019	98590350	chr6:98546547-98751343
chr6	98590424	98590601	chr6:98546547-98751343
chr6	98590609	98590772	chr6:98546547-98751343
chr6	98590774	98590928	chr6:98546547-98751343
chr6	98590959	98591124	chr6:98546547-98751343
chr6	98591134	98591295	chr6:98546547-98751343
chr6	98591304	98591377	chr6:98546547-98751343
chr6	98591454	98591548	chr6:98546547-98751343
chr6	98591599	98591700	chr6:98546547-98751343
chr6	98591869	98591949	chr6:98546547-98751343
chr6	98592094	98592254	chr6:98546547-98751343
chr6	98592264	98592866	chr6:98546547-98751343
chr6	98592869	98593037	chr6:98546547-98751343
chr6	98593039	98593826	chr6:98546547-98751343
chr6	98593919	98594023	chr6:98546547-98751343
chr6	98594084	98594455	chr6:98546547-98751343
chr6	98594474	98595319	chr6:98546547-98751343
chr6	98596239	98596373	chr6:98546547-98751343
chr6	98596389	98596620	chr6:98546547-98751343
chr6	98596629	98597369	chr6:98546547-98751343
chr6	98597379	98598296	chr6:98546547-98751343
chr6	98598304	98598401	chr6:98546547-98751343
chr6	98598404	98598756	chr6:98546547-98751343
chr6	98598769	98599278	chr6:98546547-98751343
chr6	98599469	98599539	chr6:98546547-98751343
chr6	98600869	98600952	chr6:98546547-98751343
chr6	98600954	98601034	chr6:98546547-98751343
chr6	98601644	98601718	chr6:98546547-98751343
chr6	98601834	98601935	chr6:98546547-98751343
chr6	98603609	98603683	chr6:98546547-98751343
chr6	98603884	98603955	chr6:98546547-98751343
chr6	98604049	98604156	chr6:98546547-98751343
chr6	98604164	98604238	chr6:98546547-98751343
chr6	98604554	98604913	chr6:98546547-98751343
chr6	98604919	98605002	chr6:98546547-98751343
chr6	98605019	98605137	chr6:98546547-98751343
chr6	98605139	98605371	chr6:98546547-98751343
chr6	98605434	98606231	chr6:98546547-98751343
chr6	98606524	98607369	chr6:98546547-98751343

chr6	98607379	98609222	chr6:98546547-98751343
chr6	98609229	98609798	chr6:98546547-98751343
chr6	98609834	98610216	chr6:98546547-98751343
chr6	98610229	98611539	chr6:98546547-98751343
chr6	98611959	98612438	chr6:98546547-98751343
chr6	98612444	98614511	chr6:98546547-98751343
chr6	98614939	98615011	chr6:98546547-98751343
chr6	98615084	98615189	chr6:98546547-98751343
chr6	98615279	98615358	chr6:98546547-98751343
chr6	98615504	98615584	chr6:98546547-98751343
chr6	98615604	98615860	chr6:98546547-98751343
chr6	98616239	98616324	chr6:98546547-98751343
chr6	98616369	98616454	chr6:98546547-98751343
chr6	98616509	98617031	chr6:98546547-98751343
chr6	98617059	98617132	chr6:98546547-98751343
chr6	98617204	98617288	chr6:98546547-98751343
chr6	98617319	98617401	chr6:98546547-98751343
chr6	98617479	98617748	chr6:98546547-98751343
chr6	98617759	98617987	chr6:98546547-98751343
chr6	98618034	98618130	chr6:98546547-98751343
chr6	98618229	98618354	chr6:98546547-98751343
chr6	98618459	98618611	chr6:98546547-98751343
chr6	98618649	98618721	chr6:98546547-98751343
chr6	98618824	98618901	chr6:98546547-98751343
chr6	98618989	98619331	chr6:98546547-98751343
chr6	98619344	98619425	chr6:98546547-98751343
chr6	98619489	98619752	chr6:98546547-98751343
chr6	98620204	98620278	chr6:98546547-98751343
chr6	98620294	98620435	chr6:98546547-98751343
chr6	98620464	98620539	chr6:98546547-98751343
chr6	98620594	98620675	chr6:98546547-98751343
chr6	98621234	98621562	chr6:98546547-98751343
chr6	98621564	98622395	chr6:98546547-98751343
chr6	98622399	98623416	chr6:98546547-98751343
chr6	98623494	98623600	chr6:98546547-98751343
chr6	98623839	98623911	chr6:98546547-98751343
chr6	98623919	98624110	chr6:98546547-98751343
chr6	98624134	98624338	chr6:98546547-98751343
chr6	98624919	98625018	chr6:98546547-98751343
chr6	98625039	98625120	chr6:98546547-98751343
chr6	98625389	98625459	chr6:98546547-98751343
chr6	98625529	98625609	chr6:98546547-98751343
chr6	98625744	98625863	chr6:98546547-98751343
chr6	98626144	98626269	chr6:98546547-98751343
chr6	98626514	98628010	chr6:98546547-98751343
chr6	98628014	98628149	chr6:98546547-98751343
chr6	98628179	98629118	chr6:98546547-98751343
chr6	98629444	98631680	chr6:98546547-98751343
chr6	98631974	98632171	chr6:98546547-98751343
chr6	98632174	98633094	chr6:98546547-98751343
chr6	98633529	98633606	chr6:98546547-98751343
chr6	98633674	98633772	chr6:98546547-98751343
chr6	98633774	98633866	chr6:98546547-98751343
chr6	98633874	98634010	chr6:98546547-98751343
chr6	98634029	98634873	chr6:98546547-98751343
chr6	98634874	98634974	chr6:98546547-98751343
chr6	98635079	98635567	chr6:98546547-98751343
chr6	98635569	98637032	chr6:98546547-98751343
chr6	98637034	98639104	chr6:98546547-98751343
chr6	98639394	98639929	chr6:98546547-98751343
chr6	98640234	98641862	chr6:98546547-98751343
chr6	98641939	98642146	chr6:98546547-98751343
chr6	98642179	98642426	chr6:98546547-98751343

chr6	98642464	98642546	chr6:98546547-98751343
chr6	98642559	98643860	chr6:98546547-98751343
chr6	98644154	98645856	chr6:98546547-98751343
chr6	98645864	98647697	chr6:98546547-98751343
chr6	98647709	98648049	chr6:98546547-98751343
chr6	98648054	98649093	chr6:98546547-98751343
chr6	98649099	98652989	chr6:98546547-98751343
chr6	98653054	98654146	chr6:98546547-98751343
chr6	98654149	98655296	chr6:98546547-98751343
chr6	98655734	98656573	chr6:98546547-98751343
chr6	98656574	98656968	chr6:98546547-98751343
chr6	98656984	98657139	chr6:98546547-98751343
chr6	98657144	98657344	chr6:98546547-98751343
chr6	98657349	98657939	chr6:98546547-98751343
chr6	98657944	98658352	chr6:98546547-98751343
chr6	98658354	98658431	chr6:98546547-98751343
chr6	98658494	98658643	chr6:98546547-98751343
chr6	98658664	98658782	chr6:98546547-98751343
chr6	98658844	98658943	chr6:98546547-98751343
chr6	98658944	98659059	chr6:98546547-98751343
chr6	98659094	98659391	chr6:98546547-98751343
chr6	98659429	98659564	chr6:98546547-98751343
chr6	98659749	98660888	chr6:98546547-98751343
chr6	98661199	98661273	chr6:98546547-98751343
chr6	98661284	98661466	chr6:98546547-98751343
chr6	98661474	98661571	chr6:98546547-98751343
chr6	98661874	98664032	chr6:98546547-98751343
chr6	98664334	98666083	chr6:98546547-98751343
chr6	98666119	98666306	chr6:98546547-98751343
chr6	98666359	98666440	chr6:98546547-98751343
chr6	98666459	98667226	chr6:98546547-98751343
chr6	98667824	98668464	chr6:98546547-98751343
chr6	98668469	98668790	chr6:98546547-98751343
chr6	98669259	98669754	chr6:98546547-98751343
chr6	98669864	98670038	chr6:98546547-98751343
chr6	98670039	98670142	chr6:98546547-98751343
chr6	98670219	98670293	chr6:98546547-98751343
chr6	98670319	98670641	chr6:98546547-98751343
chr6	98670824	98670974	chr6:98546547-98751343
chr6	98671069	98671205	chr6:98546547-98751343
chr6	98671624	98671754	chr6:98546547-98751343
chr6	98672434	98672675	chr6:98546547-98751343
chr6	98672709	98672798	chr6:98546547-98751343
chr6	98672844	98673022	chr6:98546547-98751343
chr6	98673404	98674564	chr6:98546547-98751343
chr6	98674854	98675816	chr6:98546547-98751343
chr6	98675834	98675910	chr6:98546547-98751343
chr6	98676084	98676289	chr6:98546547-98751343
chr6	98676294	98679636	chr6:98546547-98751343
chr6	98680164	98680333	chr6:98546547-98751343
chr6	98680344	98680456	chr6:98546547-98751343
chr6	98680604	98680689	chr6:98546547-98751343
chr6	98680799	98680918	chr6:98546547-98751343
chr6	98680929	98681114	chr6:98546547-98751343
chr6	98681484	98681579	chr6:98546547-98751343
chr6	98683639	98683812	chr6:98546547-98751343
chr6	98683854	98685002	chr6:98546547-98751343
chr6	98685034	98685515	chr6:98546547-98751343
chr6	98685579	98688425	chr6:98546547-98751343
chr6	98688829	98691208	chr6:98546547-98751343
chr6	98691524	98691918	chr6:98546547-98751343
chr6	98692104	98692211	chr6:98546547-98751343
chr6	98692219	98692392	chr6:98546547-98751343

chr6	98692414	98692661	chr6:98546547-98751343
chr6	98692669	98692872	chr6:98546547-98751343
chr6	98692874	98693548	chr6:98546547-98751343
chr6	98694194	98694313	chr6:98546547-98751343
chr6	98694334	98695022	chr6:98546547-98751343
chr6	98695029	98695975	chr6:98546547-98751343
chr6	98696254	98696493	chr6:98546547-98751343
chr6	98696494	98698482	chr6:98546547-98751343
chr6	98698484	98700176	chr6:98546547-98751343
chr6	98700179	98701038	chr6:98546547-98751343
chr6	98701049	98701359	chr6:98546547-98751343
chr6	98701599	98701781	chr6:98546547-98751343
chr6	98701969	98703603	chr6:98546547-98751343
chr6	98703609	98704252	chr6:98546547-98751343
chr6	98704284	98705347	chr6:98546547-98751343
chr6	98705584	98705773	chr6:98546547-98751343
chr6	98705849	98706396	chr6:98546547-98751343
chr6	98706399	98706467	chr6:98546547-98751343
chr6	98706469	98706935	chr6:98546547-98751343
chr6	98706954	98707586	chr6:98546547-98751343
chr6	98707864	98708436	chr6:98546547-98751343
chr6	98708499	98709364	chr6:98546547-98751343
chr6	98709369	98709567	chr6:98546547-98751343
chr6	98709574	98712033	chr6:98546547-98751343
chr6	98712089	98712821	chr6:98546547-98751343
chr6	98712844	98713548	chr6:98546547-98751343
chr6	98713564	98714168	chr6:98546547-98751343
chr6	98714484	98714704	chr6:98546547-98751343
chr6	98714719	98715191	chr6:98546547-98751343
chr6	98715194	98715369	chr6:98546547-98751343
chr6	98715494	98715664	chr6:98546547-98751343
chr6	98715669	98716322	chr6:98546547-98751343
chr6	98716374	98716613	chr6:98546547-98751343
chr6	98716629	98717709	chr6:98546547-98751343
chr6	98717989	98721712	chr6:98546547-98751343
chr6	98721714	98722588	chr6:98546547-98751343
chr6	98722589	98723341	chr6:98546547-98751343
chr6	98723354	98723432	chr6:98546547-98751343
chr6	98723439	98726041	chr6:98546547-98751343
chr6	98726044	98727779	chr6:98546547-98751343
chr6	98727789	98728354	chr6:98546547-98751343
chr6	98728374	98730174	chr6:98546547-98751343
chr6	98730189	98730441	chr6:98546547-98751343
chr6	98730449	98730584	chr6:98546547-98751343
chr6	98730614	98731132	chr6:98546547-98751343
chr6	98731134	98732210	chr6:98546547-98751343
chr6	98732259	98732586	chr6:98546547-98751343
chr6	98732589	98734261	chr6:98546547-98751343
chr6	98734264	98736166	chr6:98546547-98751343
chr6	98736199	98736980	chr6:98546547-98751343
chr6	98737134	98737386	chr6:98546547-98751343
chr6	98737524	98737721	chr6:98546547-98751343
chr6	98737959	98738037	chr6:98546547-98751343
chr6	98738079	98738211	chr6:98546547-98751343
chr6	98738214	98738351	chr6:98546547-98751343
chr6	98738664	98738725	chr6:98546547-98751343
chr6	98738744	98739110	chr6:98546547-98751343
chr6	98739124	98740196	chr6:98546547-98751343
chr6	98740204	98741644	chr6:98546547-98751343
chr6	98741894	98742035	chr6:98546547-98751343
chr6	98742199	98742269	chr6:98546547-98751343
chr6	98742324	98742582	chr6:98546547-98751343
chr6	98742584	98743748	chr6:98546547-98751343

chr6	98743749	98744131	chr6:98546547-98751343
chr6	98744144	98744454	chr6:98546547-98751343
chr6	98744519	98749826	chr6:98546547-98751343
chr6	98749864	98750153	chr6:98546547-98751343
chr6	98750234	98750402	chr6:98546547-98751343
chr6	98750769	98750844	chr6:98546547-98751343
chr6	98751024	98751102	chr6:98546547-98751343
chr6	98809214	98809326	chr6:98809213-98809288
chr6	98846324	98847776	chr6:98846346-98847748
chr6	137403608	137405079	chr6:137403629-137457242
chr6	137405088	137405327	chr6:137403629-137457242
chr6	137405363	137406008	chr6:137403629-137457242
chr6	137406018	137408689	chr6:137403629-137457242
chr6	137408693	137409413	chr6:137403629-137457242
chr6	137409718	137409797	chr6:137403629-137457242
chr6	137409823	137411537	chr6:137403629-137457242
chr6	137411593	137411989	chr6:137403629-137457242
chr6	137411993	137412775	chr6:137403629-137457242
chr6	137412908	137412983	chr6:137403629-137457242
chr6	137413083	137413867	chr6:137403629-137457242
chr6	137414268	137414866	chr6:137403629-137457242
chr6	137414948	137415043	chr6:137403629-137457242
chr6	137415078	137415176	chr6:137403629-137457242
chr6	137415183	137417321	chr6:137403629-137457242
chr6	137417328	137418161	chr6:137403629-137457242
chr6	137418163	137419131	chr6:137403629-137457242
chr6	137419723	137420273	chr6:137403629-137457242
chr6	137420563	137420989	chr6:137403629-137457242
chr6	137420998	137421079	chr6:137403629-137457242
chr6	137421083	137421709	chr6:137403629-137457242
chr6	137421978	137426709	chr6:137403629-137457242
chr6	137426718	137428015	chr6:137403629-137457242
chr6	137428508	137429515	chr6:137403629-137457242
chr6	137429968	137431371	chr6:137403629-137457242
chr6	137431463	137431532	chr6:137403629-137457242
chr6	137431798	137432023	chr6:137403629-137457242
chr6	137432113	137432219	chr6:137403629-137457242
chr6	137432288	137432478	chr6:137403629-137457242
chr6	137432633	137434335	chr6:137403629-137457242
chr6	137434568	137434769	chr6:137403629-137457242
chr6	137434828	137435313	chr6:137403629-137457242
chr6	137435318	137435760	chr6:137403629-137457242
chr6	137436038	137437188	chr6:137403629-137457242
chr6	137437248	137437717	chr6:137403629-137457242
chr6	137438128	137438256	chr6:137403629-137457242
chr6	137438318	137438528	chr6:137403629-137457242
chr6	137438538	137438773	chr6:137403629-137457242
chr6	137438778	137440449	chr6:137403629-137457242
chr6	137440468	137440650	chr6:137403629-137457242
chr6	137440673	137441134	chr6:137403629-137457242
chr6	137441243	137442753	chr6:137403629-137457242
chr6	137442758	137443059	chr6:137403629-137457242
chr6	137443068	137443512	chr6:137403629-137457242
chr6	137443518	137443724	chr6:137403629-137457242
chr6	137443863	137444225	chr6:137403629-137457242
chr6	137444238	137444911	chr6:137403629-137457242
chr6	137445203	137446064	chr6:137403629-137457242
chr6	137446073	137446255	chr6:137403629-137457242
chr6	137446308	137446867	chr6:137403629-137457242
chr6	137446923	137447172	chr6:137403629-137457242
chr6	137447178	137447543	chr6:137403629-137457242
chr6	137447823	137447911	chr6:137403629-137457242
chr6	137447918	137448692	chr6:137403629-137457242

chr6	137448803	137448901	chr6:137403629-137457242
chr6	137449028	137449110	chr6:137403629-137457242
chr6	137449218	137449295	chr6:137403629-137457242
chr6	137449983	137450060	chr6:137403629-137457242
chr6	137450593	137450670	chr6:137403629-137457242
chr6	137451053	137451236	chr6:137403629-137457242
chr6	137451273	137451358	chr6:137403629-137457242
chr6	137451403	137451769	chr6:137403629-137457242
chr6	137451783	137455746	chr6:137403629-137457242
chr6	137455838	137457235	chr6:137403629-137457242
chr6	137958233	137959522	chr6:137958265-138024094
chr6	137959533	137959793	chr6:137958265-138024094
chr6	137959833	137962682	chr6:137958265-138024094
chr6	137962688	137963062	chr6:137958265-138024094
chr6	137963063	137963131	chr6:137958265-138024094
chr6	137963428	137964091	chr6:137958265-138024094
chr6	137965338	137965876	chr6:137958265-138024094
chr6	137966073	137966183	chr6:137958265-138024094
chr6	137966643	137966727	chr6:137958265-138024094
chr6	137966728	137966897	chr6:137958265-138024094
chr6	137966958	137967801	chr6:137958265-138024094
chr6	137967823	137967908	chr6:137958265-138024094
chr6	137967998	137968132	chr6:137958265-138024094
chr6	137968338	137970049	chr6:137958265-138024094
chr6	137970078	137970352	chr6:137958265-138024094
chr6	137970598	137971204	chr6:137958265-138024094
chr6	137971648	137971906	chr6:137958265-138024094
chr6	137971948	137974006	chr6:137958265-138024094
chr6	137974013	137974545	chr6:137958265-138024094
chr6	137974553	137975220	chr6:137958265-138024094
chr6	137975273	137976027	chr6:137958265-138024094
chr6	137976078	137976166	chr6:137958265-138024094
chr6	137976203	137976410	chr6:137958265-138024094
chr6	137976498	137976684	chr6:137958265-138024094
chr6	137976698	137976859	chr6:137958265-138024094
chr6	137976923	137979361	chr6:137958265-138024094
chr6	137979363	137981382	chr6:137958265-138024094
chr6	137981388	137982238	chr6:137958265-138024094
chr6	137982758	137983628	chr6:137958265-138024094
chr6	137983923	137984145	chr6:137958265-138024094
chr6	137984148	137987181	chr6:137958265-138024094
chr6	137987188	137987628	chr6:137958265-138024094
chr6	137987668	137988152	chr6:137958265-138024094
chr6	137988183	137988596	chr6:137958265-138024094
chr6	137988598	137989099	chr6:137958265-138024094
chr6	137989343	137990967	chr6:137958265-138024094
chr6	137992258	137993526	chr6:137958265-138024094
chr6	137993533	137993667	chr6:137958265-138024094
chr6	137993673	137995517	chr6:137958265-138024094
chr6	137995523	137997566	chr6:137958265-138024094
chr6	137997573	137998367	chr6:137958265-138024094
chr6	137998368	137998820	chr6:137958265-138024094
chr6	137999088	137999858	chr6:137958265-138024094
chr6	137999868	138000210	chr6:137958265-138024094
chr6	138000498	138000615	chr6:137958265-138024094
chr6	138000648	138001312	chr6:137958265-138024094
chr6	138001593	138002902	chr6:137958265-138024094
chr6	138003233	138003648	chr6:137958265-138024094
chr6	138005028	138005903	chr6:137958265-138024094
chr6	138005928	138008927	chr6:137958265-138024094
chr6	138009018	138011728	chr6:137958265-138024094
chr6	138011733	138012088	chr6:137958265-138024094
chr6	138012143	138012652	chr6:137958265-138024094

chr6	138012853	138013291	chr6:137958265-138024094
chr6	138013338	138015411	chr6:137958265-138024094
chr6	138015443	138015596	chr6:137958265-138024094
chr6	138015598	138015905	chr6:137958265-138024094
chr6	138015908	138016389	chr6:137958265-138024094
chr6	138016393	138019308	chr6:137958265-138024094
chr6	138019623	138020409	chr6:137958265-138024094
chr6	138020423	138021068	chr6:137958265-138024094
chr6	138021203	138021477	chr6:137958265-138024094
chr6	138021478	138024127	chr6:137958265-138024094
chr6	138024128	138024750	chr6:138024158-138024776
chr6	138029003	138029108	chr6:138029038-138029085
chr6	138029138	138029253	chr6:138029171-138029227
chr6	138051273	138051773	chr6:138051306-138051734
chr6	138052323	138052393	chr6:138052348-138052382
chr6	138059818	138060133	chr6:138059845-138060120
chr6	165989956	165990041	chr6:165989987-165990027
chr6	166124481	166124654	chr6:166124506-166124641
chr6	166142701	166143448	chr6:166142456-166160771
chr6	166143451	166143614	chr6:166142456-166160771
chr6	166143651	166143984	chr6:166142456-166160771
chr6	166144536	166147364	chr6:166142456-166160771
chr6	166147366	166148446	chr6:166142456-166160771
chr6	166148491	166148560	chr6:166142456-166160771
chr6	166148616	166150072	chr6:166142456-166160771
chr6	166150076	166150280	chr6:166142456-166160771
chr6	166150306	166150773	chr6:166142456-166160771
chr6	166150846	166150927	chr6:166142456-166160771
chr6	166150931	166151033	chr6:166142456-166160771
chr6	166151071	166151179	chr6:166142456-166160771
chr6	166151296	166151489	chr6:166142456-166160771
chr6	166152366	166152633	chr6:166142456-166160771
chr6	166152691	166152769	chr6:166142456-166160771
chr6	166152891	166152968	chr6:166142456-166160771
chr6	166152991	166153071	chr6:166142456-166160771
chr6	166153121	166153309	chr6:166142456-166160771
chr6	166153371	166153636	chr6:166142456-166160771
chr6	166153641	166153726	chr6:166142456-166160771
chr6	166154161	166154363	chr6:166142456-166160771
chr6	166154381	166154550	chr6:166142456-166160771
chr6	166154581	166155155	chr6:166142456-166160771
chr6	166155156	166158343	chr6:166142456-166160771
chr6	166158351	166160602	chr6:166142456-166160771
chr6	166160606	166160679	chr6:166142456-166160771
chr6	166160706	166160808	chr6:166142456-166160771
chr6	166167646	166167897	chr6:166167676-166167870
chr6	166186416	166186782	chr6:166186449-166186759
chr6	166188331	166189337	chr6:166188340-166189315
chr6	166190036	166190528	chr6:166190061-166190509
chr6	166193721	166194499	chr6:166193756-166195411
chr6	166194526	166194780	chr6:166193756-166195411
chr6	166194786	166195442	chr6:166193756-166195411
chr6	166199931	166200124	chr6:166199966-166200079
chr6	166316111	166316365	chr6:166316144-166316338
chr6	166324266	166324451	chr6:166324295-166324422
chr6	166399566	166399999	chr6:166399595-166400091
chr7	22980883	22981550	chr7:22980877-22985782
chr7	22981573	22981820	chr7:22980877-22985782
chr7	22981848	22984849	chr7:22980877-22985782
chr7	22984853	22985798	chr7:22980877-22985782
chr7	22986548	22986905	chr7:22986570-22986866
chr7	22999848	23000069	chr7:22999874-23000034
chr7	23000828	23000967	chr7:23000853-23000941

chr7	23004008	23004174	chr7:23004033-23004150
chr7	23015803	23015935	chr7:23015828-23015924
chr7	23016283	23016460	chr7:23016311-23016427
chr7	23016938	23017067	chr7:23016959-23017040
chr7	23017758	23018103	chr7:23017782-23018067
chr7	23023528	23023673	chr7:23023562-23023664
chr7	23025143	23025378	chr7:23025166-23025354
chr7	23030653	23030786	chr7:23030679-23030758
chr7	23053518	23053771	chr7:23053543-23053749
chr7	23140660	23140890	chr7:23140690-23143197
chr7	23141045	23141294	chr7:23140690-23143197
chr7	23141295	23141372	chr7:23140690-23143197
chr7	23141375	23141765	chr7:23140690-23143197
chr7	23141770	23141851	chr7:23140690-23143197
chr7	23141870	23142128	chr7:23140690-23143197
chr7	23142160	23142401	chr7:23140690-23143197
chr7	23142455	23142571	chr7:23140690-23143197
chr7	23142625	23142796	chr7:23140690-23143197
chr7	23142910	23143067	chr7:23140690-23143197
chr7	23143840	23143944	chr7:23143868-23143937
chr7	23145150	23145292	chr7:23145171-23145252
chr7	23221420	23221844	chr7:23221445-23221825
chr7	23224665	23224938	chr7:23224688-23224917
chr7	23226660	23226793	chr7:23226670-23226765
chr7	23227630	23227880	chr7:23227651-23227838
chr7	23235430	23235567	chr7:23235457-23235534
chr7	23235975	23236081	chr7:23235997-23236061
chr7	23236275	23236417	chr7:23236298-23236385
chr7	23236770	23236902	chr7:23236791-23236866
chr7	23239055	23239197	chr7:23239076-23239161
chr7	23239780	23240653	chr7:23239786-23240628
chr7	23245605	23247066	chr7:23245631-23247664
chr7	23247085	23247159	chr7:23245631-23247664
chr7	23247195	23247283	chr7:23245631-23247664
chr7	23247325	23247401	chr7:23245631-23247664
chr7	23247410	23247528	chr7:23245631-23247664
chr7	23247530	23247697	chr7:23245631-23247664
chr7	23275555	23275770	chr7:23275585-23275742
chr7	23276260	23276467	chr7:23276286-23276442
chr7	23286155	23286585	chr7:23286181-23286546
chr7	23286600	23287022	chr7:23286627-23286986
chr7	23287325	23287648	chr7:23287359-23287612
chr7	23290355	23290500	chr7:23290380-23290481
chr7	23292900	23293104	chr7:23292925-23293078
chr7	23293755	23293971	chr7:23293787-23293931
chr7	23296475	23296907	chr7:23296510-23297667
chr7	23296935	23297030	chr7:23296510-23297667
chr7	23297305	23297585	chr7:23296510-23297667
chr7	23297595	23297689	chr7:23296510-23297667
chr7	23299575	23299793	chr7:23299598-23299757
chr7	23300050	23300421	chr7:23300074-23300392
chr7	23306075	23306260	chr7:23306099-23306234
chr7	23307480	23307623	chr7:23307504-23307607
chr7	23309560	23309811	chr7:23309585-23309794
chr7	23312705	23312810	chr7:23312699-23312795
chr7	23312865	23313005	chr7:23312887-23312990
chr7	23313105	23313575	chr7:23313139-23313544
chr7	23313660	23314742	chr7:23313683-23314727
chr7	23338325	23338793	chr7:23338357-23338768
chr7	23338905	23339258	chr7:23338939-23339227
chr7	23340425	23340674	chr7:23340457-23340636
chr7	23341825	23342386	chr7:23341856-23342355
chr7	23347465	23347602	chr7:23347486-23347568

chr7	23348940	23349198	chr7:23348974-23349180
chr7	51969146	51969294	chr7:51968975-51969273
chr7	51988801	51988971	chr7:51988822-51988934
chr7	51995541	51995649	chr7:51995464-51995622
chr7	51996756	51996872	chr7:51996786-51996826
chr7	52001391	52001491	chr7:52001415-52011415
chr7	52001696	52002017	chr7:52001415-52011415
chr7	52002201	52002298	chr7:52001415-52011415
chr7	52002306	52002574	chr7:52001415-52011415
chr7	52002596	52002914	chr7:52001415-52011415
chr7	52002936	52003048	chr7:52001415-52011415
chr7	52003056	52003638	chr7:52001415-52011415
chr7	52003686	52003966	chr7:52001415-52011415
chr7	52003981	52004203	chr7:52001415-52011415
chr7	52004461	52004711	chr7:52001415-52011415
chr7	52004721	52004819	chr7:52001415-52011415
chr7	52004836	52005085	chr7:52001415-52011415
chr7	52005111	52005323	chr7:52001415-52011415
chr7	52005331	52009021	chr7:52001415-52011415
chr7	52009686	52009791	chr7:52001415-52011415
chr7	52019856	52019956	chr7:52019880-52019932
chr7	52022596	52022699	chr7:52022633-52022659
chr7	52048161	52048338	chr7:52048193-52048309
chr7	52093136	52093264	chr7:52093160-52093231
chr7	65425638	65426058	chr7:65425671-65426050
chr7	65429383	65429462	chr7:65429309-65429445
chr7	65432688	65432811	chr7:65432717-65432894
chr7	65435258	65435336	chr7:65435268-65435353
chr7	65439248	65439466	chr7:65439281-65439428
chr7	65439478	65439731	chr7:65439512-65439691
chr7	65439883	65440098	chr7:65439905-65440058
chr7	65440968	65441198	chr7:65441001-65441189
chr7	65444358	65444564	chr7:65444385-65444528
chr7	65444678	65444942	chr7:65444713-65444898
chr7	65445183	65445431	chr7:65445210-65445396
chr7	65446928	65447322	chr7:65446960-65447301
chr7	82152555	82153033	chr7:82152384-82164963
chr7	82153070	82153151	chr7:82152384-82164963
chr7	82153155	82154013	chr7:82152384-82164963
chr7	82154125	82154807	chr7:82152384-82164963
chr7	82154810	82154903	chr7:82152384-82164963
chr7	82154905	82155254	chr7:82152384-82164963
chr7	82155500	82156025	chr7:82152384-82164963
chr7	82156330	82156741	chr7:82152384-82164963
chr7	82156745	82157492	chr7:82152384-82164963
chr7	82157495	82158678	chr7:82152384-82164963
chr7	82158680	82158854	chr7:82152384-82164963
chr7	82158855	82161277	chr7:82152384-82164963
chr7	82161295	82161418	chr7:82152384-82164963
chr7	82161450	82161738	chr7:82152384-82164963
chr7	82161740	82162146	chr7:82152384-82164963
chr7	82162220	82162828	chr7:82152384-82164963
chr7	82163130	82163719	chr7:82152384-82164963
chr7	82163985	82164488	chr7:82152384-82164963
chr7	82164510	82164673	chr7:82152384-82164963
chr7	86273197	86274323	chr7:86273229-86274287
chr7	86314197	86314607	chr7:86314176-86314572
chr7	86319692	86319909	chr7:86319726-86319870
chr7	86339387	86339553	chr7:86339414-86339521
chr7	86394297	86394970	chr7:86394321-86394929
chr7	86404367	86404613	chr7:86404396-86404587
chr7	86405237	86405351	chr7:86405260-86405495
chr7	86405357	86405534	chr7:86405260-86405495

chr7	86413182	86413285	chr7:86413212-86413271
chr7	86413532	86413985	chr7:86413541-86413973
chr7	86415337	86415488	chr7:86415372-86415463
chr7	86415542	86416452	chr7:86415576-86416432
chr7	86423902	86424089	chr7:86423935-86424061
chr7	86431982	86432488	chr7:86432016-86432515
chr7	86468132	86469245	chr7:86468154-86469221
chr7	86479657	86479883	chr7:86479685-86479860
chr7	86493562	86493955	chr7:86493597-86494200
chr7	86493997	86494232	chr7:86493597-86494200
chr7	101459193	101459409	chr7:101459183-101459373
chr7	101460848	101460970	chr7:101460881-101460949
chr7	101559373	101559543	chr7:101559394-101559505
chr7	101671348	101671455	chr7:101671377-101671425
chr7	101713603	101713713	chr7:101713618-101713697
chr7	101740618	101740782	chr7:101740643-101740781
chr7	101747653	101747755	chr7:101747615-101747739
chr7	101754943	101755090	chr7:101754977-101755054
chr7	101758463	101758572	chr7:101758486-101758553
chr7	101801818	101801920	chr7:101801839-101801888
chr7	101813693	101813842	chr7:101813725-101813830
chr7	101821718	101821966	chr7:101821748-101821937
chr7	101833058	101833166	chr7:101833092-101833151
chr7	101837088	101837203	chr7:101837121-101837170
chr7	101838758	101838901	chr7:101838786-101838883
chr7	101839883	101840622	chr7:101839913-101840585
chr7	101842058	101842157	chr7:101842081-101842147
chr7	101843318	101843469	chr7:101843350-101843452
chr7	101844613	101845521	chr7:101844639-101845484
chr7	101847648	101847861	chr7:101847670-101847836
chr7	101848358	101848475	chr7:101848393-101848450
chr7	101870613	101870964	chr7:101870646-101870949
chr7	101877298	101877546	chr7:101877331-101877520
chr7	101882568	101882893	chr7:101882599-101882864
chr7	101891663	101892134	chr7:101891691-101893513
chr7	101892138	101892989	chr7:101891691-101893513
chr7	101892993	101893330	chr7:101891691-101893513
chr7	101893353	101893539	chr7:101891691-101893513
chr7	101916603	101916800	chr7:101916636-101916764
chr7	101917493	101917599	chr7:101917514-101917581
chr7	101918493	101918665	chr7:101918517-101918630
chr7	101921193	101921374	chr7:101921219-101921336
chr7	101923298	101923436	chr7:101923328-101923412
chr7	101924063	101924173	chr7:101924095-101924152
chr7	101925108	101925254	chr7:101925131-101925212
chr7	101925968	101926085	chr7:101926003-101926068
chr7	101926278	101926678	chr7:101926312-101926650
chr7	109821001	109821106	chr7:109821034-109821067
chr7	109930326	109930452	chr7:109930348-109930419
chr7	109983786	109983900	chr7:109983824-109983845
chr7	109999956	110000078	chr7:110000004-110000019
chr7	110043651	110044366	chr7:110043681-110091388
chr7	110044411	110044535	chr7:110043681-110091388
chr7	110044621	110045225	chr7:110043681-110091388
chr7	110045321	110045411	chr7:110043681-110091388
chr7	110045511	110048129	chr7:110043681-110091388
chr7	110048131	110048899	chr7:110043681-110091388
chr7	110048906	110049415	chr7:110043681-110091388
chr7	110049426	110050063	chr7:110043681-110091388
chr7	110050086	110050538	chr7:110043681-110091388
chr7	110050546	110050866	chr7:110043681-110091388
chr7	110050886	110051285	chr7:110043681-110091388
chr7	110051371	110051715	chr7:110043681-110091388

chr7	110051716	110051798	chr7:110043681-110091388
chr7	110051801	110051881	chr7:110043681-110091388
chr7	110052156	110052432	chr7:110043681-110091388
chr7	110052551	110052627	chr7:110043681-110091388
chr7	110052636	110054745	chr7:110043681-110091388
chr7	110054746	110056066	chr7:110043681-110091388
chr7	110056151	110057101	chr7:110043681-110091388
chr7	110057386	110058008	chr7:110043681-110091388
chr7	110058011	110059862	chr7:110043681-110091388
chr7	110059951	110060902	chr7:110043681-110091388
chr7	110060911	110067686	chr7:110043681-110091388
chr7	110067936	110068563	chr7:110043681-110091388
chr7	110068581	110069044	chr7:110043681-110091388
chr7	110069066	110070875	chr7:110043681-110091388
chr7	110070876	110071094	chr7:110043681-110091388
chr7	110071101	110072098	chr7:110043681-110091388
chr7	110072426	110074126	chr7:110043681-110091388
chr7	110074136	110074413	chr7:110043681-110091388
chr7	110074676	110075915	chr7:110043681-110091388
chr7	110075981	110076133	chr7:110043681-110091388
chr7	110076146	110076284	chr7:110043681-110091388
chr7	110076326	110076406	chr7:110043681-110091388
chr7	110076471	110076569	chr7:110043681-110091388
chr7	110076596	110076683	chr7:110043681-110091388
chr7	110076686	110076837	chr7:110043681-110091388
chr7	110076926	110077009	chr7:110043681-110091388
chr7	110077021	110077312	chr7:110043681-110091388
chr7	110077346	110077420	chr7:110043681-110091388
chr7	110077461	110077543	chr7:110043681-110091388
chr7	110077591	110077969	chr7:110043681-110091388
chr7	110077971	110078080	chr7:110043681-110091388
chr7	110078091	110078411	chr7:110043681-110091388
chr7	110078421	110078601	chr7:110043681-110091388
chr7	110079836	110080715	chr7:110043681-110091388
chr7	110080731	110081811	chr7:110043681-110091388
chr7	110082106	110082211	chr7:110043681-110091388
chr7	110082216	110083500	chr7:110043681-110091388
chr7	110083506	110083584	chr7:110043681-110091388
chr7	110083936	110086476	chr7:110043681-110091388
chr7	110086771	110087975	chr7:110043681-110091388
chr7	110087976	110089038	chr7:110043681-110091388
chr7	110089041	110090506	chr7:110043681-110091388
chr7	110090551	110090729	chr7:110043681-110091388
chr7	110090741	110090817	chr7:110043681-110091388
chr7	110090826	110091427	chr7:110043681-110091388
chr7	110138861	110138971	chr7:110138900-110138938
chr7	110151091	110151184	chr7:110151116-110151171
chr7	110165486	110165647	chr7:110165512-110165618
chr7	110174621	110174976	chr7:110174645-110174939
chr7	110213486	110213624	chr7:110213508-110213586
chr7	110252121	110252275	chr7:110252147-110252253
chr7	131548493	131548872	chr7:131548277-131591828
chr7	131548873	131551044	chr7:131548277-131591828
chr7	131551363	131551723	chr7:131548277-131591828
chr7	131551728	131552614	chr7:131548277-131591828
chr7	131552648	131553090	chr7:131548277-131591828
chr7	131553128	131553236	chr7:131548277-131591828
chr7	131553588	131553717	chr7:131548277-131591828
chr7	131553718	131553800	chr7:131548277-131591828
chr7	131553808	131553998	chr7:131548277-131591828
chr7	131554013	131554339	chr7:131548277-131591828
chr7	131554343	131556123	chr7:131548277-131591828
chr7	131556423	131557483	chr7:131548277-131591828

chr7	131558118	131558196	chr7:131548277-131591828
chr7	131558203	131558715	chr7:131548277-131591828
chr7	131558723	131560139	chr7:131548277-131591828
chr7	131560153	131563500	chr7:131548277-131591828
chr7	131563528	131566237	chr7:131548277-131591828
chr7	131566243	131567775	chr7:131548277-131591828
chr7	131567778	131568183	chr7:131548277-131591828
chr7	131568478	131568626	chr7:131548277-131591828
chr7	131568628	131569232	chr7:131548277-131591828
chr7	131569238	131569444	chr7:131548277-131591828
chr7	131569448	131569519	chr7:131548277-131591828
chr7	131569523	131570901	chr7:131548277-131591828
chr7	131570903	131571117	chr7:131548277-131591828
chr7	131571128	131572413	chr7:131548277-131591828
chr7	131572458	131575304	chr7:131548277-131591828
chr7	131575608	131577125	chr7:131548277-131591828
chr7	131577143	131577832	chr7:131548277-131591828
chr7	131578063	131581869	chr7:131548277-131591828
chr7	131581883	131583441	chr7:131548277-131591828
chr7	131583443	131583701	chr7:131548277-131591828
chr7	131583703	131586530	chr7:131548277-131591828
chr7	131586533	131586922	chr7:131548277-131591828
chr7	131586953	131587074	chr7:131548277-131591828
chr7	131587093	131587173	chr7:131548277-131591828
chr7	131587328	131587669	chr7:131548277-131591828
chr7	131587673	131587878	chr7:131548277-131591828
chr7	131587888	131588288	chr7:131548277-131591828
chr7	131588293	131588604	chr7:131548277-131591828
chr7	131588618	131589785	chr7:131548277-131591828
chr7	131589848	131591849	chr7:131548277-131591828
chr7	131594933	131595020	chr7:131594861-131595330
chr7	131610963	131611193	chr7:131610997-131611154
chr7	131613128	131613388	chr7:131613163-131613365
chr7	131614113	131614280	chr7:131614148-131614256
chr7	131616848	131617102	chr7:131616881-131617068
chr7	131617748	131618096	chr7:131617783-131618070
chr7	131633468	131633745	chr7:131633489-131633712
chr7	131762396	131762574	chr7:131762423-131762557
chr7	131806691	131806946	chr7:131806726-131806915
chr7	131807586	131807739	chr7:131807612-131807693
chr7	142274710	142275771	chr7:142274732-142275743
chr8	16477924	16478033	chr8:16477963-16477992
chr8	16479714	16479823	chr8:16479749-16479799
chr8	16512499	16512673	chr8:16512524-16512647
chr8	16534389	16534603	chr8:16534413-16534583
chr8	16640669	16640817	chr8:16640696-16640791
chr8	16695394	16696643	chr8:16695418-16724432
chr8	16697109	16697247	chr8:16695418-16724432
chr8	16697489	16697848	chr8:16695418-16724432
chr8	16698004	16698137	chr8:16695418-16724432
chr8	16698244	16698729	chr8:16695418-16724432
chr8	16698994	16699061	chr8:16695418-16724432
chr8	16700604	16702405	chr8:16695418-16724432
chr8	16702409	16703086	chr8:16695418-16724432
chr8	16703194	16703627	chr8:16695418-16724432
chr8	16703644	16704198	chr8:16695418-16724432
chr8	16704199	16704554	chr8:16695418-16724432
chr8	16704799	16705736	chr8:16695418-16724432
chr8	16705744	16707475	chr8:16695418-16724432
chr8	16707804	16708962	chr8:16695418-16724432
chr8	16708969	16710441	chr8:16695418-16724432
chr8	16710449	16711436	chr8:16695418-16724432
chr8	16711439	16711593	chr8:16695418-16724432

chr8	16711879	16712302	chr8:16695418-16724432
chr8	16712344	16712609	chr8:16695418-16724432
chr8	16712914	16713371	chr8:16695418-16724432
chr8	16713384	16713591	chr8:16695418-16724432
chr8	16713594	16715196	chr8:16695418-16724432
chr8	16715199	16715351	chr8:16695418-16724432
chr8	16715354	16715500	chr8:16695418-16724432
chr8	16715519	16716104	chr8:16695418-16724432
chr8	16716109	16717465	chr8:16695418-16724432
chr8	16717469	16718258	chr8:16695418-16724432
chr8	16718519	16719884	chr8:16695418-16724432
chr8	16720159	16720339	chr8:16695418-16724432
chr8	16721124	16721191	chr8:16695418-16724432
chr8	16721389	16721603	chr8:16695418-16724432
chr8	16721679	16721774	chr8:16695418-16724432
chr8	16722064	16724208	chr8:16695418-16724432
chr8	16724214	16724459	chr8:16695418-16724432
chr8	27412949	27413083	chr8:27412628-27448127
chr8	27413129	27413732	chr8:27412628-27448127
chr8	27414019	27417202	chr8:27412628-27448127
chr8	27417204	27419417	chr8:27412628-27448127
chr8	27419499	27419577	chr8:27412628-27448127
chr8	27419899	27420798	chr8:27412628-27448127
chr8	27420949	27421108	chr8:27412628-27448127
chr8	27421554	27421634	chr8:27412628-27448127
chr8	27421674	27422690	chr8:27412628-27448127
chr8	27422774	27424429	chr8:27412628-27448127
chr8	27424719	27425279	chr8:27412628-27448127
chr8	27425574	27427169	chr8:27412628-27448127
chr8	27427924	27430154	chr8:27412628-27448127
chr8	27430264	27431112	chr8:27412628-27448127
chr8	27431129	27432344	chr8:27412628-27448127
chr8	27432494	27434380	chr8:27412628-27448127
chr8	27434654	27434805	chr8:27412628-27448127
chr8	27434809	27435450	chr8:27412628-27448127
chr8	27435454	27436226	chr8:27412628-27448127
chr8	27436569	27437769	chr8:27412628-27448127
chr8	27437804	27438024	chr8:27412628-27448127
chr8	27438104	27438178	chr8:27412628-27448127
chr8	27438179	27438324	chr8:27412628-27448127
chr8	27438409	27438510	chr8:27412628-27448127
chr8	27438654	27438836	chr8:27412628-27448127
chr8	27438874	27440304	chr8:27412628-27448127
chr8	27440554	27440709	chr8:27412628-27448127
chr8	27440714	27444731	chr8:27412628-27448127
chr8	27445004	27445529	chr8:27412628-27448127
chr8	27445739	27447660	chr8:27412628-27448127
chr8	27447784	27447901	chr8:27412628-27448127
chr8	27447944	27448159	chr8:27412628-27448127
chr8	27476564	27477478	chr8:27476347-27491006
chr8	27477774	27478302	chr8:27476347-27491006
chr8	27478374	27478458	chr8:27476347-27491006
chr8	27478519	27479727	chr8:27476347-27491006
chr8	27480014	27480192	chr8:27476347-27491006
chr8	27480489	27480704	chr8:27476347-27491006
chr8	27480759	27481036	chr8:27476347-27491006
chr8	27481139	27481298	chr8:27476347-27491006
chr8	27481324	27482664	chr8:27476347-27491006
chr8	27482669	27484320	chr8:27476347-27491006
chr8	27484389	27485549	chr8:27476347-27491006
chr8	27486204	27486788	chr8:27476347-27491006
chr8	27486804	27487913	chr8:27476347-27491006
chr8	27487994	27488245	chr8:27476347-27491006

chr8	27488249	27488326	chr8:27476347-27491006
chr8	27488384	27488457	chr8:27476347-27491006
chr8	27488524	27488631	chr8:27476347-27491006
chr8	27488659	27489738	chr8:27476347-27491006
chr8	27489764	27490497	chr8:27476347-27491006
chr8	27490779	27491021	chr8:27476347-27491006
chr8	60473860	60474134	chr8:60473885-60474119
chr8	60506110	60506388	chr8:60506136-60506373
chr8	60513455	60513561	chr8:60513481-60513522
chr8	60513895	60514039	chr8:60513919-60514011
chr8	60519055	60519470	chr8:60519088-60519564
chr8	60519510	60519590	chr8:60519088-60519564
chr8	60525355	60525481	chr8:60525377-60525448
chr8	60525910	60526016	chr8:60525939-60526334
chr8	60526025	60526346	chr8:60525939-60526334
chr8	60532435	60532748	chr8:60532470-60532719
chr8	60680910	60681391	chr8:60680901-60961769
chr8	60681440	60681527	chr8:60680901-60961769
chr8	60681550	60681734	chr8:60680901-60961769
chr8	60681765	60681877	chr8:60680901-60961769
chr8	60681935	60682828	chr8:60680901-60961769
chr8	60682830	60682945	chr8:60680901-60961769
chr8	60683010	60683494	chr8:60680901-60961769
chr8	60683540	60683939	chr8:60680901-60961769
chr8	60683965	60684571	chr8:60680901-60961769
chr8	60684575	60684948	chr8:60680901-60961769
chr8	60685025	60685102	chr8:60680901-60961769
chr8	60685155	60685705	chr8:60680901-60961769
chr8	60685765	60685974	chr8:60680901-60961769
chr8	60686005	60686604	chr8:60680901-60961769
chr8	60686610	60687618	chr8:60680901-60961769
chr8	60687635	60689511	chr8:60680901-60961769
chr8	60689580	60690313	chr8:60680901-60961769
chr8	60690340	60692298	chr8:60680901-60961769
chr8	60692355	60695997	chr8:60680901-60961769
chr8	60696090	60696824	chr8:60680901-60961769
chr8	60696825	60698581	chr8:60680901-60961769
chr8	60698620	60698707	chr8:60680901-60961769
chr8	60698990	60699071	chr8:60680901-60961769
chr8	60699210	60700617	chr8:60680901-60961769
chr8	60700925	60701772	chr8:60680901-60961769
chr8	60702065	60702846	chr8:60680901-60961769
chr8	60702895	60703448	chr8:60680901-60961769
chr8	60703450	60703649	chr8:60680901-60961769
chr8	60703655	60703799	chr8:60680901-60961769
chr8	60703855	60704040	chr8:60680901-60961769
chr8	60704120	60704237	chr8:60680901-60961769
chr8	60704240	60704814	chr8:60680901-60961769
chr8	60704825	60706354	chr8:60680901-60961769
chr8	60706360	60706566	chr8:60680901-60961769
chr8	60706575	60706637	chr8:60680901-60961769
chr8	60706645	60706709	chr8:60680901-60961769
chr8	60706710	60706805	chr8:60680901-60961769
chr8	60706885	60706969	chr8:60680901-60961769
chr8	60707130	60707214	chr8:60680901-60961769
chr8	60707340	60707561	chr8:60680901-60961769
chr8	60707565	60707657	chr8:60680901-60961769
chr8	60707665	60707759	chr8:60680901-60961769
chr8	60707825	60707901	chr8:60680901-60961769
chr8	60707995	60708286	chr8:60680901-60961769
chr8	60708450	60708521	chr8:60680901-60961769
chr8	60708530	60708638	chr8:60680901-60961769
chr8	60708645	60708896	chr8:60680901-60961769

chr8	60708930	60709382	chr8:60680901-60961769
chr8	60709395	60709471	chr8:60680901-60961769
chr8	60709475	60709929	chr8:60680901-60961769
chr8	60709930	60711351	chr8:60680901-60961769
chr8	60711360	60711828	chr8:60680901-60961769
chr8	60711935	60712102	chr8:60680901-60961769
chr8	60712120	60712478	chr8:60680901-60961769
chr8	60712485	60712565	chr8:60680901-60961769
chr8	60712570	60712917	chr8:60680901-60961769
chr8	60712930	60713446	chr8:60680901-60961769
chr8	60713450	60715224	chr8:60680901-60961769
chr8	60715225	60716528	chr8:60680901-60961769
chr8	60716530	60716662	chr8:60680901-60961769
chr8	60716680	60717063	chr8:60680901-60961769
chr8	60717350	60717767	chr8:60680901-60961769
chr8	60717775	60718390	chr8:60680901-60961769
chr8	60718410	60718509	chr8:60680901-60961769
chr8	60718515	60719370	chr8:60680901-60961769
chr8	60719445	60719568	chr8:60680901-60961769
chr8	60719610	60720802	chr8:60680901-60961769
chr8	60720810	60720976	chr8:60680901-60961769
chr8	60720980	60721439	chr8:60680901-60961769
chr8	60721450	60721966	chr8:60680901-60961769
chr8	60721970	60722472	chr8:60680901-60961769
chr8	60722475	60722841	chr8:60680901-60961769
chr8	60722850	60723665	chr8:60680901-60961769
chr8	60723670	60724035	chr8:60680901-60961769
chr8	60724100	60724424	chr8:60680901-60961769
chr8	60724425	60725501	chr8:60680901-60961769
chr8	60725825	60726346	chr8:60680901-60961769
chr8	60726360	60727163	chr8:60680901-60961769
chr8	60727220	60727444	chr8:60680901-60961769
chr8	60727505	60727693	chr8:60680901-60961769
chr8	60727695	60728467	chr8:60680901-60961769
chr8	60732225	60733442	chr8:60680901-60961769
chr8	60733455	60734603	chr8:60680901-60961769
chr8	60734605	60735364	chr8:60680901-60961769
chr8	60735380	60735454	chr8:60680901-60961769
chr8	60735455	60735669	chr8:60680901-60961769
chr8	60735695	60735893	chr8:60680901-60961769
chr8	60735945	60736601	chr8:60680901-60961769
chr8	60736680	60736794	chr8:60680901-60961769
chr8	60736795	60737459	chr8:60680901-60961769
chr8	60737535	60737627	chr8:60680901-60961769
chr8	60737635	60738602	chr8:60680901-60961769
chr8	60738715	60738845	chr8:60680901-60961769
chr8	60738955	60739105	chr8:60680901-60961769
chr8	60739110	60739187	chr8:60680901-60961769
chr8	60739360	60741847	chr8:60680901-60961769
chr8	60741855	60743598	chr8:60680901-60961769
chr8	60743900	60744656	chr8:60680901-60961769
chr8	60744660	60745479	chr8:60680901-60961769
chr8	60745480	60746621	chr8:60680901-60961769
chr8	60746630	60746778	chr8:60680901-60961769
chr8	60746785	60747264	chr8:60680901-60961769
chr8	60747285	60748377	chr8:60680901-60961769
chr8	60748385	60748930	chr8:60680901-60961769
chr8	60749270	60750309	chr8:60680901-60961769
chr8	60750310	60751373	chr8:60680901-60961769
chr8	60751670	60752966	chr8:60680901-60961769
chr8	60752970	60753124	chr8:60680901-60961769
chr8	60753125	60754419	chr8:60680901-60961769
chr8	60754425	60754793	chr8:60680901-60961769

chr8	60754795	60755113	chr8:60680901-60961769
chr8	60755125	60755876	chr8:60680901-60961769
chr8	60755955	60756028	chr8:60680901-60961769
chr8	60756050	60756119	chr8:60680901-60961769
chr8	60756160	60756229	chr8:60680901-60961769
chr8	60756315	60756391	chr8:60680901-60961769
chr8	60756490	60756569	chr8:60680901-60961769
chr8	60756580	60756672	chr8:60680901-60961769
chr8	60756685	60756873	chr8:60680901-60961769
chr8	60756895	60757084	chr8:60680901-60961769
chr8	60757105	60758338	chr8:60680901-60961769
chr8	60758400	60758490	chr8:60680901-60961769
chr8	60758560	60758770	chr8:60680901-60961769
chr8	60758815	60759548	chr8:60680901-60961769
chr8	60759795	60760290	chr8:60680901-60961769
chr8	60760300	60760451	chr8:60680901-60961769
chr8	60760465	60760785	chr8:60680901-60961769
chr8	60760920	60761079	chr8:60680901-60961769
chr8	60761225	60762256	chr8:60680901-60961769
chr8	60762300	60762703	chr8:60680901-60961769
chr8	60762730	60764420	chr8:60680901-60961769
chr8	60764450	60765716	chr8:60680901-60961769
chr8	60765720	60765792	chr8:60680901-60961769
chr8	60765860	60767307	chr8:60680901-60961769
chr8	60767310	60767581	chr8:60680901-60961769
chr8	60767590	60767966	chr8:60680901-60961769
chr8	60767980	60768102	chr8:60680901-60961769
chr8	60768140	60768420	chr8:60680901-60961769
chr8	60768555	60768804	chr8:60680901-60961769
chr8	60768815	60768896	chr8:60680901-60961769
chr8	60768960	60769059	chr8:60680901-60961769
chr8	60769375	60769491	chr8:60680901-60961769
chr8	60769640	60769747	chr8:60680901-60961769
chr8	60769775	60769853	chr8:60680901-60961769
chr8	60770065	60770138	chr8:60680901-60961769
chr8	60770180	60770270	chr8:60680901-60961769
chr8	60770530	60770603	chr8:60680901-60961769
chr8	60770900	60770982	chr8:60680901-60961769
chr8	60771055	60771132	chr8:60680901-60961769
chr8	60771645	60771718	chr8:60680901-60961769
chr8	60772070	60772142	chr8:60680901-60961769
chr8	60772185	60772274	chr8:60680901-60961769
chr8	60772750	60772830	chr8:60680901-60961769
chr8	60772845	60773232	chr8:60680901-60961769
chr8	60773245	60773353	chr8:60680901-60961769
chr8	60773410	60774436	chr8:60680901-60961769
chr8	60774440	60777474	chr8:60680901-60961769
chr8	60777480	60779081	chr8:60680901-60961769
chr8	60779200	60781933	chr8:60680901-60961769
chr8	60781960	60782278	chr8:60680901-60961769
chr8	60782290	60782366	chr8:60680901-60961769
chr8	60788375	60790160	chr8:60680901-60961769
chr8	60790250	60790491	chr8:60680901-60961769
chr8	60790615	60790689	chr8:60680901-60961769
chr8	60790695	60790806	chr8:60680901-60961769
chr8	60790915	60791066	chr8:60680901-60961769
chr8	60791235	60791332	chr8:60680901-60961769
chr8	60791710	60791880	chr8:60680901-60961769
chr8	60791900	60792052	chr8:60680901-60961769
chr8	60792135	60792371	chr8:60680901-60961769
chr8	60792380	60793277	chr8:60680901-60961769
chr8	60793280	60793503	chr8:60680901-60961769
chr8	60793535	60793689	chr8:60680901-60961769

chr8	60793690	60794254	chr8:60680901-60961769
chr8	60794280	60794426	chr8:60680901-60961769
chr8	60800600	60800833	chr8:60680901-60961769
chr8	60800930	60801166	chr8:60680901-60961769
chr8	60801230	60804441	chr8:60680901-60961769
chr8	60805260	60805514	chr8:60680901-60961769
chr8	60805780	60806512	chr8:60680901-60961769
chr8	60806520	60807694	chr8:60680901-60961769
chr8	60807725	60808198	chr8:60680901-60961769
chr8	60808210	60808306	chr8:60680901-60961769
chr8	60808340	60808549	chr8:60680901-60961769
chr8	60808575	60810789	chr8:60680901-60961769
chr8	60810820	60810932	chr8:60680901-60961769
chr8	60810970	60811804	chr8:60680901-60961769
chr8	60811845	60811983	chr8:60680901-60961769
chr8	60812175	60812750	chr8:60680901-60961769
chr8	60812975	60813261	chr8:60680901-60961769
chr8	60813310	60813406	chr8:60680901-60961769
chr8	60813535	60813968	chr8:60680901-60961769
chr8	60813970	60814087	chr8:60680901-60961769
chr8	60814245	60814336	chr8:60680901-60961769
chr8	60814340	60814457	chr8:60680901-60961769
chr8	60814610	60814729	chr8:60680901-60961769
chr8	60814745	60814826	chr8:60680901-60961769
chr8	60814830	60814909	chr8:60680901-60961769
chr8	60815090	60815168	chr8:60680901-60961769
chr8	60815500	60815673	chr8:60680901-60961769
chr8	60815830	60815907	chr8:60680901-60961769
chr8	60816830	60816905	chr8:60680901-60961769
chr8	60817080	60817153	chr8:60680901-60961769
chr8	60817595	60817692	chr8:60680901-60961769
chr8	60818125	60819871	chr8:60680901-60961769
chr8	60819875	60820820	chr8:60680901-60961769
chr8	60820860	60821149	chr8:60680901-60961769
chr8	60821150	60821285	chr8:60680901-60961769
chr8	60821435	60821702	chr8:60680901-60961769
chr8	60821770	60821965	chr8:60680901-60961769
chr8	60822020	60822193	chr8:60680901-60961769
chr8	60822195	60822293	chr8:60680901-60961769
chr8	60822370	60822461	chr8:60680901-60961769
chr8	60822530	60822614	chr8:60680901-60961769
chr8	60822615	60822710	chr8:60680901-60961769
chr8	60822715	60822946	chr8:60680901-60961769
chr8	60822985	60823081	chr8:60680901-60961769
chr8	60823085	60823163	chr8:60680901-60961769
chr8	60823165	60823243	chr8:60680901-60961769
chr8	60823340	60823485	chr8:60680901-60961769
chr8	60823495	60823591	chr8:60680901-60961769
chr8	60823660	60823976	chr8:60680901-60961769
chr8	60824100	60824177	chr8:60680901-60961769
chr8	60824185	60824337	chr8:60680901-60961769
chr8	60824370	60824604	chr8:60680901-60961769
chr8	60824625	60824702	chr8:60680901-60961769
chr8	60824725	60824805	chr8:60680901-60961769
chr8	60825065	60825184	chr8:60680901-60961769
chr8	60825305	60825389	chr8:60680901-60961769
chr8	60825405	60825503	chr8:60680901-60961769
chr8	60825520	60825639	chr8:60680901-60961769
chr8	60825640	60825747	chr8:60680901-60961769
chr8	60826065	60826569	chr8:60680901-60961769
chr8	60826575	60827441	chr8:60680901-60961769
chr8	60827475	60827609	chr8:60680901-60961769
chr8	60827895	60828828	chr8:60680901-60961769

chr8	60828835	60831451	chr8:60680901-60961769
chr8	60831455	60833007	chr8:60680901-60961769
chr8	60833010	60834472	chr8:60680901-60961769
chr8	60834475	60835038	chr8:60680901-60961769
chr8	60835050	60836556	chr8:60680901-60961769
chr8	60836560	60837220	chr8:60680901-60961769
chr8	60837225	60837405	chr8:60680901-60961769
chr8	60837410	60838094	chr8:60680901-60961769
chr8	60838095	60838544	chr8:60680901-60961769
chr8	60838545	60841651	chr8:60680901-60961769
chr8	60841945	60843232	chr8:60680901-60961769
chr8	60843250	60845481	chr8:60680901-60961769
chr8	60845500	60847817	chr8:60680901-60961769
chr8	60847870	60848043	chr8:60680901-60961769
chr8	60848385	60848810	chr8:60680901-60961769
chr8	60848820	60850640	chr8:60680901-60961769
chr8	60850875	60850952	chr8:60680901-60961769
chr8	60850965	60852312	chr8:60680901-60961769
chr8	60852370	60852456	chr8:60680901-60961769
chr8	60852605	60853366	chr8:60680901-60961769
chr8	60853400	60854220	chr8:60680901-60961769
chr8	60854305	60856117	chr8:60680901-60961769
chr8	60856240	60856460	chr8:60680901-60961769
chr8	60856510	60856657	chr8:60680901-60961769
chr8	60856665	60858983	chr8:60680901-60961769
chr8	60858985	60859064	chr8:60680901-60961769
chr8	60859075	60859465	chr8:60680901-60961769
chr8	60859475	60861782	chr8:60680901-60961769
chr8	60862060	60862284	chr8:60680901-60961769
chr8	60862560	60863133	chr8:60680901-60961769
chr8	60863180	60864047	chr8:60680901-60961769
chr8	60866525	60867668	chr8:60680901-60961769
chr8	60868620	60869208	chr8:60680901-60961769
chr8	60869210	60869344	chr8:60680901-60961769
chr8	60869345	60870839	chr8:60680901-60961769
chr8	60870845	60871504	chr8:60680901-60961769
chr8	60871520	60871613	chr8:60680901-60961769
chr8	60871620	60873304	chr8:60680901-60961769
chr8	60873595	60876704	chr8:60680901-60961769
chr8	60877005	60880093	chr8:60680901-60961769
chr8	60880110	60882491	chr8:60680901-60961769
chr8	60882515	60882796	chr8:60680901-60961769
chr8	60882835	60882914	chr8:60680901-60961769
chr8	60882920	60883416	chr8:60680901-60961769
chr8	60883425	60883658	chr8:60680901-60961769
chr8	60883705	60884375	chr8:60680901-60961769
chr8	60884385	60884836	chr8:60680901-60961769
chr8	60884880	60885186	chr8:60680901-60961769
chr8	60885190	60886067	chr8:60680901-60961769
chr8	60886085	60886378	chr8:60680901-60961769
chr8	60886385	60887911	chr8:60680901-60961769
chr8	60887920	60888589	chr8:60680901-60961769
chr8	60888600	60889363	chr8:60680901-60961769
chr8	60889365	60889674	chr8:60680901-60961769
chr8	60889705	60890586	chr8:60680901-60961769
chr8	60890595	60891719	chr8:60680901-60961769
chr8	60891955	60892177	chr8:60680901-60961769
chr8	60892180	60892310	chr8:60680901-60961769
chr8	60892315	60893094	chr8:60680901-60961769
chr8	60893205	60893868	chr8:60680901-60961769
chr8	60893875	60894237	chr8:60680901-60961769
chr8	60894260	60894394	chr8:60680901-60961769
chr8	60894410	60894538	chr8:60680901-60961769

chr8	60894575	60894841	chr8:60680901-60961769
chr8	60894895	60895306	chr8:60680901-60961769
chr8	60895385	60895559	chr8:60680901-60961769
chr8	60896020	60896601	chr8:60680901-60961769
chr8	60896755	60896842	chr8:60680901-60961769
chr8	60896855	60896995	chr8:60680901-60961769
chr8	60897110	60897295	chr8:60680901-60961769
chr8	60897375	60897456	chr8:60680901-60961769
chr8	60897460	60897632	chr8:60680901-60961769
chr8	60897705	60897817	chr8:60680901-60961769
chr8	60897825	60897958	chr8:60680901-60961769
chr8	60897990	60898065	chr8:60680901-60961769
chr8	60898075	60898147	chr8:60680901-60961769
chr8	60898150	60898266	chr8:60680901-60961769
chr8	60898435	60898658	chr8:60680901-60961769
chr8	60898745	60898832	chr8:60680901-60961769
chr8	60898910	60899166	chr8:60680901-60961769
chr8	60899170	60899261	chr8:60680901-60961769
chr8	60899300	60899371	chr8:60680901-60961769
chr8	60899385	60900048	chr8:60680901-60961769
chr8	60900055	60900239	chr8:60680901-60961769
chr8	60900255	60900459	chr8:60680901-60961769
chr8	60900475	60900673	chr8:60680901-60961769
chr8	60900675	60900847	chr8:60680901-60961769
chr8	60900850	60901080	chr8:60680901-60961769
chr8	60901350	60901812	chr8:60680901-60961769
chr8	60901830	60903021	chr8:60680901-60961769
chr8	60903025	60904049	chr8:60680901-60961769
chr8	60904115	60904424	chr8:60680901-60961769
chr8	60904425	60906117	chr8:60680901-60961769
chr8	60906120	60906441	chr8:60680901-60961769
chr8	60906445	60907519	chr8:60680901-60961769
chr8	60907525	60912276	chr8:60680901-60961769
chr8	60912370	60912552	chr8:60680901-60961769
chr8	60912570	60912792	chr8:60680901-60961769
chr8	60913680	60914235	chr8:60680901-60961769
chr8	60914295	60914471	chr8:60680901-60961769
chr8	60914570	60914688	chr8:60680901-60961769
chr8	60915125	60915211	chr8:60680901-60961769
chr8	60915225	60915332	chr8:60680901-60961769
chr8	60915460	60915550	chr8:60680901-60961769
chr8	60915590	60915680	chr8:60680901-60961769
chr8	60915750	60915866	chr8:60680901-60961769
chr8	60916155	60916380	chr8:60680901-60961769
chr8	60916425	60916517	chr8:60680901-60961769
chr8	60918205	60918285	chr8:60680901-60961769
chr8	60918320	60918403	chr8:60680901-60961769
chr8	60918595	60918673	chr8:60680901-60961769
chr8	60920305	60920391	chr8:60680901-60961769
chr8	60920550	60922548	chr8:60680901-60961769
chr8	60922665	60922883	chr8:60680901-60961769
chr8	60922950	60923461	chr8:60680901-60961769
chr8	60923755	60926753	chr8:60680901-60961769
chr8	60926755	60926974	chr8:60680901-60961769
chr8	60927050	60927132	chr8:60680901-60961769
chr8	60927290	60927791	chr8:60680901-60961769
chr8	60927795	60929235	chr8:60680901-60961769
chr8	60929305	60929418	chr8:60680901-60961769
chr8	60929440	60929524	chr8:60680901-60961769
chr8	60929670	60929764	chr8:60680901-60961769
chr8	60929810	60930268	chr8:60680901-60961769
chr8	60930280	60932686	chr8:60680901-60961769
chr8	60932690	60933330	chr8:60680901-60961769

chr8	60934090	60934430	chr8:60680901-60961769
chr8	60934440	60935268	chr8:60680901-60961769
chr8	60935275	60936170	chr8:60680901-60961769
chr8	60937540	60939963	chr8:60680901-60961769
chr8	60939965	60940849	chr8:60680901-60961769
chr8	60941135	60944295	chr8:60680901-60961769
chr8	60944330	60946304	chr8:60680901-60961769
chr8	60946835	60948533	chr8:60680901-60961769
chr8	60948580	60949110	chr8:60680901-60961769
chr8	60949125	60949240	chr8:60680901-60961769
chr8	60949470	60949710	chr8:60680901-60961769
chr8	60949800	60949896	chr8:60680901-60961769
chr8	60950360	60950439	chr8:60680901-60961769
chr8	60950480	60950666	chr8:60680901-60961769
chr8	60950845	60951018	chr8:60680901-60961769
chr8	60951075	60951353	chr8:60680901-60961769
chr8	60951420	60951578	chr8:60680901-60961769
chr8	60951595	60952169	chr8:60680901-60961769
chr8	60952170	60953053	chr8:60680901-60961769
chr8	60953075	60954209	chr8:60680901-60961769
chr8	60954495	60955380	chr8:60680901-60961769
chr8	60955445	60955961	chr8:60680901-60961769
chr8	60955965	60957361	chr8:60680901-60961769
chr8	60957370	60957591	chr8:60680901-60961769
chr8	60957625	60961782	chr8:60680901-60961769
chr8	60962050	60962209	chr8:60962084-60962178
chr8	60970785	60971102	chr8:60970809-60971087
chr8	60987125	60987387	chr8:60987152-60987353
chr8	61028335	61028551	chr8:61028357-61028506
chr8	61045395	61045496	chr8:61045432-61045455
chr8	89339897	89339984	chr8:89339931-89340088
chr8	89340042	89340120	chr8:89339931-89340088
chr8	89341132	89341342	chr8:89341157-89341326
chr8	89554797	89555095	chr8:89554832-89555059
chr8	89721532	89722133	chr8:89721558-89722107
chr8	89723697	89725299	chr8:89723723-89725271
chr8	89726407	89727171	chr8:89726431-89727163
chr8	89748202	89748348	chr8:89747953-89749363
chr8	89748352	89749326	chr8:89747953-89749363
chr8	89757877	89757959	chr8:89757688-89761163
chr8	89759747	89759979	chr8:89757688-89761163
chr8	89759987	89760874	chr8:89757688-89761163
chr8	89760877	89761187	chr8:89757688-89761163
chr8	91071402	91072529	chr8:91071435-91072514
chr8	91072827	91072948	chr8:91072856-91072928
chr8	91075302	91075403	chr8:91075327-91075381
chr8	91075482	91075576	chr8:91075508-91075548
chr8	91075612	91075717	chr8:91075633-91075689
chr8	91078092	91078230	chr8:91078125-91078203
chr8	91081192	91081305	chr8:91081225-91081282
chr8	91081347	91081495	chr8:91081381-91081465
chr8	91090577	91090707	chr8:91090600-91090675
chr8	91093612	91093781	chr8:91093643-91093749
chr8	91094227	91094369	chr8:91094253-91094330
chr8	91094822	91095139	chr8:91094846-91095109
chr8	91105647	91105737	chr8:91105625-91105903
chr8	98162503	98162631	chr8:98162534-98166913
chr8	98162783	98162877	chr8:98162534-98166913
chr8	98162973	98163122	chr8:98162534-98166913
chr8	98163128	98164406	chr8:98162534-98166913
chr8	98164503	98165267	chr8:98162534-98166913
chr8	98165268	98166949	chr8:98162534-98166913
chr8	111275219	111275391	chr8:111275243-111275358

chr8	111431029	111431187	chr8:111431060-111431152
chr8	111458244	111458542	chr8:111458276-111514682
chr8	111458549	111458820	chr8:111458276-111514682
chr8	111458824	111459105	chr8:111458276-111514682
chr8	111459399	111460266	chr8:111458276-111514682
chr8	111460299	111460819	chr8:111458276-111514682
chr8	111461104	111462306	chr8:111458276-111514682
chr8	111462319	111462787	chr8:111458276-111514682
chr8	111462844	111462920	chr8:111458276-111514682
chr8	111462984	111463099	chr8:111458276-111514682
chr8	111463259	111463440	chr8:111458276-111514682
chr8	111463519	111463595	chr8:111458276-111514682
chr8	111463624	111463697	chr8:111458276-111514682
chr8	111463699	111463773	chr8:111458276-111514682
chr8	111464114	111464528	chr8:111458276-111514682
chr8	111464539	111464725	chr8:111458276-111514682
chr8	111464734	111465361	chr8:111458276-111514682
chr8	111466604	111466719	chr8:111458276-111514682
chr8	111466754	111466940	chr8:111458276-111514682
chr8	111466959	111467407	chr8:111458276-111514682
chr8	111467409	111467897	chr8:111458276-111514682
chr8	111467939	111468011	chr8:111458276-111514682
chr8	111468079	111468155	chr8:111458276-111514682
chr8	111468194	111468372	chr8:111458276-111514682
chr8	111468444	111468787	chr8:111458276-111514682
chr8	111468819	111468903	chr8:111458276-111514682
chr8	111469159	111473954	chr8:111458276-111514682
chr8	111473959	111474254	chr8:111458276-111514682
chr8	111474259	111475138	chr8:111458276-111514682
chr8	111475159	111475903	chr8:111458276-111514682
chr8	111475909	111477146	chr8:111458276-111514682
chr8	111477149	111478331	chr8:111458276-111514682
chr8	111478334	111478681	chr8:111458276-111514682
chr8	111478694	111479087	chr8:111458276-111514682
chr8	111479094	111480571	chr8:111458276-111514682
chr8	111480874	111481027	chr8:111458276-111514682
chr8	111481029	111482403	chr8:111458276-111514682
chr8	111482479	111482554	chr8:111458276-111514682
chr8	111483299	111483381	chr8:111458276-111514682
chr8	111483984	111484050	chr8:111458276-111514682
chr8	111484214	111484453	chr8:111458276-111514682
chr8	111484454	111484607	chr8:111458276-111514682
chr8	111484609	111484827	chr8:111458276-111514682
chr8	111484839	111485205	chr8:111458276-111514682
chr8	111485224	111485396	chr8:111458276-111514682
chr8	111485409	111485813	chr8:111458276-111514682
chr8	111485814	111485917	chr8:111458276-111514682
chr8	111485964	111486036	chr8:111458276-111514682
chr8	111486074	111486769	chr8:111458276-111514682
chr8	111486784	111488693	chr8:111458276-111514682
chr8	111488764	111489107	chr8:111458276-111514682
chr8	111489109	111489397	chr8:111458276-111514682
chr8	111489524	111489754	chr8:111458276-111514682
chr8	111489764	111489946	chr8:111458276-111514682
chr8	111490214	111490395	chr8:111458276-111514682
chr8	111490424	111490591	chr8:111458276-111514682
chr8	111490594	111490965	chr8:111458276-111514682
chr8	111491049	111491203	chr8:111458276-111514682
chr8	111491204	111491593	chr8:111458276-111514682
chr8	111491634	111491817	chr8:111458276-111514682
chr8	111491824	111492139	chr8:111458276-111514682
chr8	111492239	111493043	chr8:111458276-111514682
chr8	111493044	111493633	chr8:111458276-111514682

chr8	111493634	111494119	chr8:111458276-111514682
chr8	111494464	111494759	chr8:111458276-111514682
chr8	111494804	111495033	chr8:111458276-111514682
chr8	111495074	111495216	chr8:111458276-111514682
chr8	111495254	111495588	chr8:111458276-111514682
chr8	111495689	111495778	chr8:111458276-111514682
chr8	111495909	111497871	chr8:111458276-111514682
chr8	111497924	111498992	chr8:111458276-111514682
chr8	111499089	111499334	chr8:111458276-111514682
chr8	111499419	111499506	chr8:111458276-111514682
chr8	111499564	111499644	chr8:111458276-111514682
chr8	111499889	111499999	chr8:111458276-111514682
chr8	111500039	111500189	chr8:111458276-111514682
chr8	111500204	111500278	chr8:111458276-111514682
chr8	111500354	111500501	chr8:111458276-111514682
chr8	111500514	111500589	chr8:111458276-111514682
chr8	111500639	111500717	chr8:111458276-111514682
chr8	111500749	111500820	chr8:111458276-111514682
chr8	111501274	111501398	chr8:111458276-111514682
chr8	111501434	111501599	chr8:111458276-111514682
chr8	111501699	111501789	chr8:111458276-111514682
chr8	111501839	111501935	chr8:111458276-111514682
chr8	111501969	111502081	chr8:111458276-111514682
chr8	111502194	111502320	chr8:111458276-111514682
chr8	111502364	111502468	chr8:111458276-111514682
chr8	111502529	111502600	chr8:111458276-111514682
chr8	111502719	111502793	chr8:111458276-111514682
chr8	111502799	111502886	chr8:111458276-111514682
chr8	111503159	111503344	chr8:111458276-111514682
chr8	111503649	111504514	chr8:111458276-111514682
chr8	111504519	111504979	chr8:111458276-111514682
chr8	111504989	111505211	chr8:111458276-111514682
chr8	111505214	111505640	chr8:111458276-111514682
chr8	111505959	111506079	chr8:111458276-111514682
chr8	111506244	111508926	chr8:111458276-111514682
chr8	111509494	111509595	chr8:111458276-111514682
chr8	111509619	111509692	chr8:111458276-111514682
chr8	111509759	111510055	chr8:111458276-111514682
chr8	111510919	111510998	chr8:111458276-111514682
chr8	111511024	111511123	chr8:111458276-111514682
chr8	111511184	111511841	chr8:111458276-111514682
chr8	111512174	111512774	chr8:111458276-111514682
chr8	111512809	111513074	chr8:111458276-111514682
chr8	111513249	111513363	chr8:111458276-111514682
chr8	111513374	111513542	chr8:111458276-111514682
chr8	111513859	111514412	chr8:111458276-111514682
chr8	111639579	111639652	chr8:111639594-111639687
chr8	111644549	111644824	chr8:111644579-111644794
chr8	111676974	111677089	chr8:111677021-111677035
chr8	128698552	128699874	chr8:128698587-128701668
chr8	128699882	128701710	chr8:128698587-128701668
chr8	128704012	128704153	chr8:128704034-128704111
chr8	128712822	128713105	chr8:128712852-128713084
chr8	128715272	128715514	chr8:128715294-128715488
chr8	128742197	128743182	chr8:128742231-128743168
chr8	128746042	128746246	chr8:128746070-128746213
chr8	128746272	128746383	chr8:128746309-128746328
chr8	128747267	128747367	chr8:128747291-128747342
chr8	128750122	128750228	chr8:128750147-128750197
chr8	128787272	128787729	chr8:128786990-128817883
chr8	128788017	128788183	chr8:128786990-128817883
chr8	128788352	128789349	chr8:128786990-128817883
chr8	128790022	128790794	chr8:128786990-128817883

chr8	128790877	128791027	chr8:128786990-128817883
chr8	128791177	128791612	chr8:128786990-128817883
chr8	128792007	128792273	chr8:128786990-128817883
chr8	128792477	128792555	chr8:128786990-128817883
chr8	128792677	128793072	chr8:128786990-128817883
chr8	128794009	128794399	chr8:128786990-128817883
chr8	128794679	128795373	chr8:128786990-128817883
chr8	128795629	128796227	chr8:128786990-128817883
chr8	128796509	128797227	chr8:128786990-128817883
chr8	128797519	128797855	chr8:128786990-128817883
chr8	128798064	128799249	chr8:128786990-128817883
chr8	128799534	128799961	chr8:128786990-128817883
chr8	128800879	128801561	chr8:128786990-128817883
chr8	128801714	128802398	chr8:128786990-128817883
chr8	128802694	128802926	chr8:128786990-128817883
chr8	128802934	128803015	chr8:128786990-128817883
chr8	128803289	128803438	chr8:128786990-128817883
chr8	128803664	128804124	chr8:128786990-128817883
chr8	128804644	128805606	chr8:128786990-128817883
chr8	128805909	128808494	chr8:128786990-128817883
chr8	128808499	128809118	chr8:128786990-128817883
chr8	128809559	128810269	chr8:128786990-128817883
chr8	128810559	128810833	chr8:128786990-128817883
chr8	128811129	128811332	chr8:128786990-128817883
chr8	128811364	128811539	chr8:128786990-128817883
chr8	128811669	128812795	chr8:128786990-128817883
chr8	128812799	128813488	chr8:128786990-128817883
chr8	128813764	128814147	chr8:128786990-128817883
chr8	128814744	128814843	chr8:128786990-128817883
chr8	128814844	128815360	chr8:128786990-128817883
chr8	128815664	128815864	chr8:128786990-128817883
chr8	128816484	128816591	chr8:128786990-128817883
chr8	128817084	128817180	chr8:128786990-128817883
chr8	128817464	128817617	chr8:128786990-128817883
chr8	128817624	128817908	chr8:128786990-128817883
chr8	128864154	128864611	chr8:128864179-128864593
chr8	128866949	128867065	chr8:128866973-128867041
chr8	128867364	128867578	chr8:128867393-128867565
chr8	128902799	128903277	chr8:128902834-128903244
chr8	128944274	128944931	chr8:128944298-128944941
chr8	128951724	128952746	chr8:128951753-128952700
chr8	128996394	128996467	chr8:128996149-128996450
chr8	129001374	129002113	chr8:129001407-129002289
chr8	129002114	129002322	chr8:129001407-129002289
chr8	129008779	129008883	chr8:129008806-129008844
chr8	129010419	129010573	chr8:129010445-129010605
chr8	129021814	129022001	chr8:129021835-129021964
chr8	129022539	129022714	chr8:129022563-129022690
chr8	129023699	129023816	chr8:129023722-129023791
chr8	129028854	129028970	chr8:129028880-129028923
chr8	129060669	129060879	chr8:129060693-129060855
chr8	129082374	129082642	chr8:129082405-129082607
chr8	129094899	129095122	chr8:129094932-129095094
chr8	129096744	129096882	chr8:129096766-129096867
chr8	129108004	129108111	chr8:129108058-129108063
chr8	129108744	129108936	chr8:129108763-129108902
chr8	129111594	129111990	chr8:129111615-129112144
chr8	129111994	129112125	chr8:129111615-129112144
chr8	129113229	129113525	chr8:129113225-129113503
chr8	129116674	129117455	chr8:129116707-129117437
chr8	129132274	129132844	chr8:129132282-129134260
chr8	129132849	129133652	chr8:129132282-129134260
chr8	129133719	129133880	chr8:129132282-129134260

chr8	129133889	129134239	chr8:129132282-129134260
chr8	129134539	129134878	chr8:129134564-129138308
chr8	129134894	129135279	chr8:129134564-129138308
chr8	129135349	129137508	chr8:129134564-129138308
chr8	129137524	129138345	chr8:129134564-129138308
chr8	129146314	129146594	chr8:129146341-129146578
chr8	129147299	129147477	chr8:129147320-129217547
chr8	129147484	129149623	chr8:129147320-129217547
chr8	129149649	129152745	chr8:129147320-129217547
chr8	129152759	129154861	chr8:129147320-129217547
chr8	129154864	129155109	chr8:129147320-129217547
chr8	129155299	129155929	chr8:129147320-129217547
chr8	129156054	129156158	chr8:129147320-129217547
chr8	129156639	129156725	chr8:129147320-129217547
chr8	129157139	129158980	chr8:129147320-129217547
chr8	129159009	129159254	chr8:129147320-129217547
chr8	129159264	129160357	chr8:129147320-129217547
chr8	129160359	129166195	chr8:129147320-129217547
chr8	129166199	129166629	chr8:129147320-129217547
chr8	129166679	129166760	chr8:129147320-129217547
chr8	129167094	129167176	chr8:129147320-129217547
chr8	129167204	129168227	chr8:129147320-129217547
chr8	129168519	129168953	chr8:129147320-129217547
chr8	129169149	129170212	chr8:129147320-129217547
chr8	129170494	129171576	chr8:129147320-129217547
chr8	129171859	129172262	chr8:129147320-129217547
chr8	129172344	129172551	chr8:129147320-129217547
chr8	129172554	129174433	chr8:129147320-129217547
chr8	129174704	129176955	chr8:129147320-129217547
chr8	129176964	129183562	chr8:129147320-129217547
chr8	129183569	129186112	chr8:129147320-129217547
chr8	129186114	129187065	chr8:129147320-129217547
chr8	129187334	129188746	chr8:129147320-129217547
chr8	129188749	129189142	chr8:129147320-129217547
chr8	129189154	129189582	chr8:129147320-129217547
chr8	129189619	129190122	chr8:129147320-129217547
chr8	129190214	129191369	chr8:129147320-129217547
chr8	129191389	129191565	chr8:129147320-129217547
chr8	129191579	129192108	chr8:129147320-129217547
chr8	129192114	129192819	chr8:129147320-129217547
chr8	129192829	129193281	chr8:129147320-129217547
chr8	129193559	129196080	chr8:129147320-129217547
chr8	129196109	129196258	chr8:129147320-129217547
chr8	129196274	129196427	chr8:129147320-129217547
chr8	129196434	129197110	chr8:129147320-129217547
chr8	129197114	129198782	chr8:129147320-129217547
chr8	129198789	129198983	chr8:129147320-129217547
chr8	129199279	129199370	chr8:129147320-129217547
chr8	129199379	129200369	chr8:129147320-129217547
chr8	129200374	129200955	chr8:129147320-129217547
chr8	129200964	129201161	chr8:129147320-129217547
chr8	129201169	129201773	chr8:129147320-129217547
chr8	129201779	129204044	chr8:129147320-129217547
chr8	129204049	129204626	chr8:129147320-129217547
chr8	129204634	129205488	chr8:129147320-129217547
chr8	129205489	129206313	chr8:129147320-129217547
chr8	129207014	129207101	chr8:129147320-129217547
chr8	129207354	129207637	chr8:129147320-129217547
chr8	129207974	129208898	chr8:129147320-129217547
chr8	129208899	129213983	chr8:129147320-129217547
chr8	129213989	129216337	chr8:129147320-129217547
chr8	129216339	129217461	chr8:129147320-129217547
chr8	129224474	129226216	chr8:129224496-129226187

chr8	129268219	129268432	chr8:129268253-129268400
chr8	130363819	130363920	chr8:130363847-130365226
chr8	130363924	130365261	chr8:130363847-130365226
chr8	130382529	130382669	chr8:130382552-130382625
chr8	130400264	130400403	chr8:130400290-130400364
chr8	130402099	130402200	chr8:130402143-130402173
chr8	130411854	130411955	chr8:130411759-130411930
chr8	130426924	130427077	chr8:130426950-130427058
chr8	130428164	130428304	chr8:130428185-130428273
chr8	130452189	130452298	chr8:130452219-130452274
chr8	130457929	130458139	chr8:130457951-130458098
chr8	130458749	130458907	chr8:130458779-130458872
chr8	130461194	130461612	chr8:130461174-130461574
chr8	130462669	130463360	chr8:130462690-130463351
chr8	130464969	130465373	chr8:130464992-130465363
chr8	130482624	130483729	chr8:130482617-130506952
chr8	130484004	130485589	chr8:130482617-130506952
chr8	130485614	130486062	chr8:130482617-130506952
chr8	130486064	130486277	chr8:130482617-130506952
chr8	130486284	130486956	chr8:130482617-130506952
chr8	130486964	130487048	chr8:130482617-130506952
chr8	130487069	130488358	chr8:130482617-130506952
chr8	130488359	130489477	chr8:130482617-130506952
chr8	130489494	130489682	chr8:130482617-130506952
chr8	130489684	130489752	chr8:130482617-130506952
chr8	130489754	130490496	chr8:130482617-130506952
chr8	130490804	130491267	chr8:130482617-130506952
chr8	130491334	130493001	chr8:130482617-130506952
chr8	130493009	130493192	chr8:130482617-130506952
chr8	130493199	130494568	chr8:130482617-130506952
chr8	130494664	130494799	chr8:130482617-130506952
chr8	130495059	130495238	chr8:130482617-130506952
chr8	130495249	130495332	chr8:130482617-130506952
chr8	130495484	130495909	chr8:130482617-130506952
chr8	130495919	130497611	chr8:130482617-130506952
chr8	130497839	130497952	chr8:130482617-130506952
chr8	130498014	130498264	chr8:130482617-130506952
chr8	130498349	130498486	chr8:130482617-130506952
chr8	130498494	130500196	chr8:130482617-130506952
chr8	130500204	130500312	chr8:130482617-130506952
chr8	130500589	130501782	chr8:130482617-130506952
chr8	130501794	130502154	chr8:130482617-130506952
chr8	130502159	130502281	chr8:130482617-130506952
chr8	130502309	130502462	chr8:130482617-130506952
chr8	130502734	130503108	chr8:130482617-130506952
chr8	130503114	130504465	chr8:130482617-130506952
chr8	130505174	130506128	chr8:130482617-130506952
chr8	130506134	130506503	chr8:130482617-130506952
chr8	130506619	130506731	chr8:130482617-130506952
chr8	130506739	130506992	chr8:130482617-130506952
chr8	130518489	130518958	chr8:130518516-130518918
chr8	130519574	130520031	chr8:130519608-130522444
chr8	130520064	130520534	chr8:130519608-130522444
chr8	130520549	130521068	chr8:130519608-130522444
chr8	130521069	130521572	chr8:130519608-130522444
chr8	130521589	130522462	chr8:130519608-130522444
chr8	130560444	130560952	chr8:130560479-130560917
chr8	130587104	130587413	chr8:130587127-130587396
chr8	130598604	130599295	chr8:130598637-130599268
chr8	130639379	130639731	chr8:130639359-130641491
chr8	130639744	130641516	chr8:130639359-130641491
chr8	130671209	130671295	chr8:130671234-130689686
chr8	130671314	130672085	chr8:130671234-130689686

chr8	130672149	130672233	chr8:130671234-130689686
chr8	130672249	130672329	chr8:130671234-130689686
chr8	130672344	130672427	chr8:130671234-130689686
chr8	130672434	130673005	chr8:130671234-130689686
chr8	130673009	130674156	chr8:130671234-130689686
chr8	130674169	130675107	chr8:130671234-130689686
chr8	130675109	130675712	chr8:130671234-130689686
chr8	130675824	130675902	chr8:130671234-130689686
chr8	130676059	130677272	chr8:130671234-130689686
chr8	130677294	130677531	chr8:130671234-130689686
chr8	130677534	130679590	chr8:130671234-130689686
chr8	130679894	130680692	chr8:130671234-130689686
chr8	130681619	130681695	chr8:130671234-130689686
chr8	130682119	130682225	chr8:130671234-130689686
chr8	130683119	130683203	chr8:130671234-130689686
chr8	130683204	130685774	chr8:130671234-130689686
chr8	130685844	130686427	chr8:130671234-130689686
chr8	130686734	130688346	chr8:130671234-130689686
chr8	130688354	130689409	chr8:130671234-130689686
chr8	130690759	130692651	chr8:130690788-130692626
chr8	130693839	130694779	chr8:130693819-130698684
chr8	130694884	130695070	chr8:130693819-130698684
chr8	130695089	130696306	chr8:130693819-130698684
chr8	130696309	130698639	chr8:130693819-130698684
chr9	22029409	22029613	chr9:22029431-22029593
chr9	22032649	22032997	chr9:22032672-22032985
chr9	22046294	22046477	chr9:22046315-22046448
chr9	22046724	22046933	chr9:22046749-22046899
chr9	22049069	22049247	chr9:22049104-22049227
chr9	22056349	22056424	chr9:22056250-22056386
chr9	22058334	22058830	chr9:22058357-22059053
chr9	22061929	22062054	chr9:22061951-22062025
chr9	22063919	22064055	chr9:22063942-22064017
chr9	22065639	22065784	chr9:22065660-22065756
chr9	22067564	22069521	chr9:22067593-22072638
chr9	22069524	22069867	chr9:22067593-22072638
chr9	22069959	22071294	chr9:22067593-22072638
chr9	22071364	22072666	chr9:22067593-22072638
chr9	22077644	22077934	chr9:22077677-22077890
chr9	22096344	22096540	chr9:22096370-22096513
chr9	22097229	22097391	chr9:22097256-22097363
chr9	22112294	22112426	chr9:22112318-22112394
chr9	22113639	22113810	chr9:22113664-22113798
chr9	22118609	22118803	chr9:22118642-22118766
chr9	22120174	22120451	chr9:22120198-22120409
chr9	22120479	22120678	chr9:22120502-22121096
chr9	22120679	22120793	chr9:22120502-22121096
chr9	22120809	22121118	chr9:22120502-22121096
chr9	33649113	33649218	chr9:33649152-33649167
chr9	33657488	33657595	chr9:33657523-33658572
chr9	33657598	33658142	chr9:33657523-33658572
chr9	33658153	33658537	chr9:33657523-33658572
chr9	33658788	33658983	chr9:33658821-33713591
chr9	33659018	33659730	chr9:33658821-33713591
chr9	33659883	33664273	chr9:33658821-33713591
chr9	33664293	33665473	chr9:33658821-33713591
chr9	33665733	33666042	chr9:33658821-33713591
chr9	33666253	33666332	chr9:33658821-33713591
chr9	33673493	33673585	chr9:33658821-33713591
chr9	33673593	33673750	chr9:33658821-33713591
chr9	33673763	33674348	chr9:33658821-33713591
chr9	33674353	33676045	chr9:33658821-33713591
chr9	33676048	33677019	chr9:33658821-33713591

chr9	33677023	33677155	chr9:33658821-33713591
chr9	33677158	33677394	chr9:33658821-33713591
chr9	33677398	33678063	chr9:33658821-33713591
chr9	33678073	33678708	chr9:33658821-33713591
chr9	33678713	33679316	chr9:33658821-33713591
chr9	33679368	33679727	chr9:33658821-33713591
chr9	33679733	33680425	chr9:33658821-33713591
chr9	33680438	33680646	chr9:33658821-33713591
chr9	33680678	33680760	chr9:33658821-33713591
chr9	33681028	33681120	chr9:33658821-33713591
chr9	33681208	33681394	chr9:33658821-33713591
chr9	33681543	33681749	chr9:33658821-33713591
chr9	33681753	33681842	chr9:33658821-33713591
chr9	33681958	33683592	chr9:33658821-33713591
chr9	33683598	33684061	chr9:33658821-33713591
chr9	33684068	33685470	chr9:33658821-33713591
chr9	33685748	33687955	chr9:33658821-33713591
chr9	33687968	33691017	chr9:33658821-33713591
chr9	33691283	33696194	chr9:33658821-33713591
chr9	33696213	33696536	chr9:33658821-33713591
chr9	33696553	33697411	chr9:33658821-33713591
chr9	33697458	33700198	chr9:33658821-33713591
chr9	33700438	33700537	chr9:33658821-33713591
chr9	33700553	33701612	chr9:33658821-33713591
chr9	33701903	33702895	chr9:33658821-33713591
chr9	33702898	33703318	chr9:33658821-33713591
chr9	33703328	33703641	chr9:33658821-33713591
chr9	33703743	33703915	chr9:33658821-33713591
chr9	33703928	33704041	chr9:33658821-33713591
chr9	33704078	33704397	chr9:33658821-33713591
chr9	33704453	33705461	chr9:33658821-33713591
chr9	33705463	33705844	chr9:33658821-33713591
chr9	33706158	33706633	chr9:33658821-33713591
chr9	33706638	33707075	chr9:33658821-33713591
chr9	33707258	33707474	chr9:33658821-33713591
chr9	33707498	33707643	chr9:33658821-33713591
chr9	33707663	33708205	chr9:33658821-33713591
chr9	33708233	33708405	chr9:33658821-33713591
chr9	33708573	33708668	chr9:33658821-33713591
chr9	33708818	33709719	chr9:33658821-33713591
chr9	33709783	33711049	chr9:33658821-33713591
chr9	33711053	33711278	chr9:33658821-33713591
chr9	33711308	33711892	chr9:33658821-33713591
chr9	33711898	33713284	chr9:33658821-33713591
chr9	33713308	33713493	chr9:33658821-33713591
chr9	33719658	33719905	chr9:33719687-33719866
chr9	33721553	33722580	chr9:33721588-33722553
chr9	33732938	33733361	chr9:33732972-33733329
chr9	33738253	33738435	chr9:33738285-33738414
chr9	33798333	33798451	chr9:33798365-33798433
chr9	33799763	33799940	chr9:33799784-33799911
chr9	33818593	33818805	chr9:33818624-33818793
chr9	35161954	35162323	chr9:35161988-35162302
chr9	35227979	35228073	chr9:35228011-35228041
chr9	35231094	35231236	chr9:35231116-35231216
chr9	35236444	35236619	chr9:35236465-35236583
chr9	35237674	35237838	chr9:35237699-35237823
chr9	35243279	35243389	chr9:35243287-35243361
chr9	35258964	35259076	chr9:35258989-35259047
chr9	35295664	35295951	chr9:35295692-35295927
chr9	35310429	35310781	chr9:35310463-35310778
chr9	35313874	35314014	chr9:35313895-35313986
chr9	35366914	35367026	chr9:35366943-35366990

chr9	35370289	35370433	chr9:35370314-35370393
chr9	35375099	35375234	chr9:35375123-35375198
chr9	35375994	35376286	chr9:35376024-35376244
chr9	35377439	35377723	chr9:35377464-35377692
chr9	35378259	35378466	chr9:35378291-35378433
chr9	35380439	35380646	chr9:35380466-35380636
chr9	35381069	35381246	chr9:35381096-35381212
chr9	35381529	35381753	chr9:35381552-35381716
chr9	35382324	35382546	chr9:35382353-35382504
chr9	35384219	35384324	chr9:35384242-35384311
chr9	35385699	35385833	chr9:35385720-35385810
chr9	35386139	35386313	chr9:35386161-35386290
chr9	35389819	35389992	chr9:35389842-35389970
chr9	35390604	35390750	chr9:35390625-35390711
chr9	35396449	35396626	chr9:35396472-35396599
chr9	35396814	35396947	chr9:35396837-35396934
chr9	35397139	35397341	chr9:35397163-35397307
chr9	35397609	35397740	chr9:35397631-35397709
chr9	35398184	35398319	chr9:35398207-35398285
chr9	35398529	35398665	chr9:35398550-35398639
chr9	35398849	35399071	chr9:35398878-35399031
chr9	35399134	35399305	chr9:35399157-35399281
chr9	35399359	35399477	chr9:35399388-35399445
chr9	35399619	35399757	chr9:35399645-35399726
chr9	35400259	35400474	chr9:35400292-35400440
chr9	35403139	35403274	chr9:35403163-35403256
chr9	35403414	35403611	chr9:35403436-35403596
chr9	35403719	35404346	chr9:35403744-35404331
chr9	84631309	84631477	chr9:84630942-84813641
chr9	84631784	84632294	chr9:84630942-84813641
chr9	84632929	84633109	chr9:84630942-84813641
chr9	84633164	84633840	chr9:84630942-84813641
chr9	84633944	84634092	chr9:84630942-84813641
chr9	84634379	84635779	chr9:84630942-84813641
chr9	84635934	84636088	chr9:84630942-84813641
chr9	84636089	84638714	chr9:84630942-84813641
chr9	84639034	84640306	chr9:84630942-84813641
chr9	84640309	84640412	chr9:84630942-84813641
chr9	84640414	84640536	chr9:84630942-84813641
chr9	84640919	84641024	chr9:84630942-84813641
chr9	84641109	84641290	chr9:84630942-84813641
chr9	84641344	84641461	chr9:84630942-84813641
chr9	84641469	84642354	chr9:84630942-84813641
chr9	84642394	84642577	chr9:84630942-84813641
chr9	84642584	84642875	chr9:84630942-84813641
chr9	84642879	84642951	chr9:84630942-84813641
chr9	84642964	84643230	chr9:84630942-84813641
chr9	84643554	84643636	chr9:84630942-84813641
chr9	84643684	84643761	chr9:84630942-84813641
chr9	84643769	84643966	chr9:84630942-84813641
chr9	84643974	84647445	chr9:84630942-84813641
chr9	84647494	84647784	chr9:84630942-84813641
chr9	84647789	84649300	chr9:84630942-84813641
chr9	84649304	84650281	chr9:84630942-84813641
chr9	84650289	84652919	chr9:84630942-84813641
chr9	84653004	84654494	chr9:84630942-84813641
chr9	84654789	84655569	chr9:84630942-84813641
chr9	84655604	84655774	chr9:84630942-84813641
chr9	84655819	84655926	chr9:84630942-84813641
chr9	84656024	84656913	chr9:84630942-84813641
chr9	84656964	84657106	chr9:84630942-84813641
chr9	84657114	84657343	chr9:84630942-84813641
chr9	84657419	84657521	chr9:84630942-84813641

chr9	84657704	84657882	chr9:84630942-84813641
chr9	84657994	84658126	chr9:84630942-84813641
chr9	84658129	84658245	chr9:84630942-84813641
chr9	84658279	84658392	chr9:84630942-84813641
chr9	84658609	84658718	chr9:84630942-84813641
chr9	84658834	84658935	chr9:84630942-84813641
chr9	84658979	84659146	chr9:84630942-84813641
chr9	84659149	84659399	chr9:84630942-84813641
chr9	84659409	84659563	chr9:84630942-84813641
chr9	84659959	84660060	chr9:84630942-84813641
chr9	84660344	84660509	chr9:84630942-84813641
chr9	84660514	84660860	chr9:84630942-84813641
chr9	84660874	84660957	chr9:84630942-84813641
chr9	84660959	84661183	chr9:84630942-84813641
chr9	84661244	84661460	chr9:84630942-84813641
chr9	84661484	84661825	chr9:84630942-84813641
chr9	84662099	84662276	chr9:84630942-84813641
chr9	84662389	84662470	chr9:84630942-84813641
chr9	84662554	84663827	chr9:84630942-84813641
chr9	84663829	84664060	chr9:84630942-84813641
chr9	84664099	84664517	chr9:84630942-84813641
chr9	84664804	84665440	chr9:84630942-84813641
chr9	84665444	84666245	chr9:84630942-84813641
chr9	84666249	84666529	chr9:84630942-84813641
chr9	84666784	84667717	chr9:84630942-84813641
chr9	84667724	84668350	chr9:84630942-84813641
chr9	84669309	84669441	chr9:84630942-84813641
chr9	84669464	84669575	chr9:84630942-84813641
chr9	84670144	84670746	chr9:84630942-84813641
chr9	84670759	84670850	chr9:84630942-84813641
chr9	84670924	84671311	chr9:84630942-84813641
chr9	84671334	84671452	chr9:84630942-84813641
chr9	84671469	84673783	chr9:84630942-84813641
chr9	84673829	84674887	chr9:84630942-84813641
chr9	84674889	84676040	chr9:84630942-84813641
chr9	84676059	84676432	chr9:84630942-84813641
chr9	84676434	84676607	chr9:84630942-84813641
chr9	84676619	84676833	chr9:84630942-84813641
chr9	84676869	84676954	chr9:84630942-84813641
chr9	84677064	84677460	chr9:84630942-84813641
chr9	84677784	84678226	chr9:84630942-84813641
chr9	84678279	84678489	chr9:84630942-84813641
chr9	84678494	84678887	chr9:84630942-84813641
chr9	84678889	84679000	chr9:84630942-84813641
chr9	84679004	84679104	chr9:84630942-84813641
chr9	84679534	84679603	chr9:84630942-84813641
chr9	84679864	84680175	chr9:84630942-84813641
chr9	84680489	84680777	chr9:84630942-84813641
chr9	84680839	84681078	chr9:84630942-84813641
chr9	84681089	84681165	chr9:84630942-84813641
chr9	84681279	84681386	chr9:84630942-84813641
chr9	84681394	84681564	chr9:84630942-84813641
chr9	84681579	84681793	chr9:84630942-84813641
chr9	84681794	84681966	chr9:84630942-84813641
chr9	84681979	84682855	chr9:84630942-84813641
chr9	84683154	84683306	chr9:84630942-84813641
chr9	84683624	84683890	chr9:84630942-84813641
chr9	84683899	84684291	chr9:84630942-84813641
chr9	84684299	84684602	chr9:84630942-84813641
chr9	84684684	84684771	chr9:84630942-84813641
chr9	84684889	84685146	chr9:84630942-84813641
chr9	84685294	84685406	chr9:84630942-84813641
chr9	84685464	84686356	chr9:84630942-84813641

chr9	84686719	84686830	chr9:84630942-84813641
chr9	84687114	84687201	chr9:84630942-84813641
chr9	84687259	84687472	chr9:84630942-84813641
chr9	84687994	84688061	chr9:84630942-84813641
chr9	84688139	84690660	chr9:84630942-84813641
chr9	84690674	84693347	chr9:84630942-84813641
chr9	84693699	84693865	chr9:84630942-84813641
chr9	84694639	84697551	chr9:84630942-84813641
chr9	84697554	84698458	chr9:84630942-84813641
chr9	84698754	84704427	chr9:84630942-84813641
chr9	84704579	84707211	chr9:84630942-84813641
chr9	84707504	84708771	chr9:84630942-84813641
chr9	84708959	84709659	chr9:84630942-84813641
chr9	84709924	84710593	chr9:84630942-84813641
chr9	84710599	84710907	chr9:84630942-84813641
chr9	84710924	84711053	chr9:84630942-84813641
chr9	84711684	84711753	chr9:84630942-84813641
chr9	84712064	84712150	chr9:84630942-84813641
chr9	84712459	84712552	chr9:84630942-84813641
chr9	84712714	84712796	chr9:84630942-84813641
chr9	84713114	84713208	chr9:84630942-84813641
chr9	84713279	84713407	chr9:84630942-84813641
chr9	84714149	84714227	chr9:84630942-84813641
chr9	84714279	84714458	chr9:84630942-84813641
chr9	84714459	84714869	chr9:84630942-84813641
chr9	84715219	84715472	chr9:84630942-84813641
chr9	84715534	84715899	chr9:84630942-84813641
chr9	84715934	84719369	chr9:84630942-84813641
chr9	84719434	84719790	chr9:84630942-84813641
chr9	84720074	84720139	chr9:84630942-84813641
chr9	84720149	84720270	chr9:84630942-84813641
chr9	84720374	84721816	chr9:84630942-84813641
chr9	84721839	84722912	chr9:84630942-84813641
chr9	84722929	84723212	chr9:84630942-84813641
chr9	84723354	84723417	chr9:84630942-84813641
chr9	84723439	84723574	chr9:84630942-84813641
chr9	84723579	84723664	chr9:84630942-84813641
chr9	84723674	84725553	chr9:84630942-84813641
chr9	84725829	84726184	chr9:84630942-84813641
chr9	84726194	84728527	chr9:84630942-84813641
chr9	84728549	84731931	chr9:84630942-84813641
chr9	84731939	84734292	chr9:84630942-84813641
chr9	84734524	84735900	chr9:84630942-84813641
chr9	84735949	84736465	chr9:84630942-84813641
chr9	84736469	84736600	chr9:84630942-84813641
chr9	84736604	84736708	chr9:84630942-84813641
chr9	84736709	84736944	chr9:84630942-84813641
chr9	84736949	84737021	chr9:84630942-84813641
chr9	84737309	84737916	chr9:84630942-84813641
chr9	84737954	84738345	chr9:84630942-84813641
chr9	84738684	84738760	chr9:84630942-84813641
chr9	84739039	84739321	chr9:84630942-84813641
chr9	84739634	84740416	chr9:84630942-84813641
chr9	84740459	84741597	chr9:84630942-84813641
chr9	84741599	84741886	chr9:84630942-84813641
chr9	84741964	84742101	chr9:84630942-84813641
chr9	84742209	84742399	chr9:84630942-84813641
chr9	84742459	84743768	chr9:84630942-84813641
chr9	84743774	84744280	chr9:84630942-84813641
chr9	84744564	84744919	chr9:84630942-84813641
chr9	84745189	84745531	chr9:84630942-84813641
chr9	84745554	84745972	chr9:84630942-84813641
chr9	84745994	84746593	chr9:84630942-84813641

chr9	84746604	84749608	chr9:84630942-84813641
chr9	84749609	84750617	chr9:84630942-84813641
chr9	84750619	84750741	chr9:84630942-84813641
chr9	84751084	84752161	chr9:84630942-84813641
chr9	84752239	84752387	chr9:84630942-84813641
chr9	84753409	84753490	chr9:84630942-84813641
chr9	84753659	84755733	chr9:84630942-84813641
chr9	84756004	84756071	chr9:84630942-84813641
chr9	84756364	84759234	chr9:84630942-84813641
chr9	84759584	84761871	chr9:84630942-84813641
chr9	84761879	84764461	chr9:84630942-84813641
chr9	84764464	84767535	chr9:84630942-84813641
chr9	84767539	84768581	chr9:84630942-84813641
chr9	84768659	84771668	chr9:84630942-84813641
chr9	84771694	84771830	chr9:84630942-84813641
chr9	84771849	84771938	chr9:84630942-84813641
chr9	84771969	84772145	chr9:84630942-84813641
chr9	84772474	84772880	chr9:84630942-84813641
chr9	84772889	84776047	chr9:84630942-84813641
chr9	84776279	84777170	chr9:84630942-84813641
chr9	84778999	84779741	chr9:84630942-84813641
chr9	84779749	84779825	chr9:84630942-84813641
chr9	84780829	84781039	chr9:84630942-84813641
chr9	84781064	84781505	chr9:84630942-84813641
chr9	84781509	84781587	chr9:84630942-84813641
chr9	84781589	84783067	chr9:84630942-84813641
chr9	84783444	84784351	chr9:84630942-84813641
chr9	84784604	84784828	chr9:84630942-84813641
chr9	84784859	84786711	chr9:84630942-84813641
chr9	84787009	84787184	chr9:84630942-84813641
chr9	84787194	84787560	chr9:84630942-84813641
chr9	84787574	84788558	chr9:84630942-84813641
chr9	84788559	84788834	chr9:84630942-84813641
chr9	84788844	84789007	chr9:84630942-84813641
chr9	84789054	84789514	chr9:84630942-84813641
chr9	84789799	84790594	chr9:84630942-84813641
chr9	84790604	84790782	chr9:84630942-84813641
chr9	84790839	84790990	chr9:84630942-84813641
chr9	84790999	84791264	chr9:84630942-84813641
chr9	84791314	84791597	chr9:84630942-84813641
chr9	84791639	84791716	chr9:84630942-84813641
chr9	84791994	84792228	chr9:84630942-84813641
chr9	84792234	84792346	chr9:84630942-84813641
chr9	84792354	84792480	chr9:84630942-84813641
chr9	84792739	84792842	chr9:84630942-84813641
chr9	84792844	84794068	chr9:84630942-84813641
chr9	84794349	84795082	chr9:84630942-84813641
chr9	84795379	84795513	chr9:84630942-84813641
chr9	84795514	84797276	chr9:84630942-84813641
chr9	84797474	84798520	chr9:84630942-84813641
chr9	84798529	84798797	chr9:84630942-84813641
chr9	84798839	84799094	chr9:84630942-84813641
chr9	84799144	84800371	chr9:84630942-84813641
chr9	84800384	84802543	chr9:84630942-84813641
chr9	84802549	84804209	chr9:84630942-84813641
chr9	84804219	84804721	chr9:84630942-84813641
chr9	84804734	84805450	chr9:84630942-84813641
chr9	84805994	84806798	chr9:84630942-84813641
chr9	84806814	84806895	chr9:84630942-84813641
chr9	84806964	84807160	chr9:84630942-84813641
chr9	84807244	84807621	chr9:84630942-84813641
chr9	84807679	84807986	chr9:84630942-84813641
chr9	84807994	84809152	chr9:84630942-84813641

chr9	84809499	84809618	chr9:84630942-84813641
chr9	84809669	84809748	chr9:84630942-84813641
chr9	84809799	84812794	chr9:84630942-84813641
chr9	84812809	84813654	chr9:84630942-84813641
chr9	84887649	84887902	chr9:84887670-84888396
chr9	84887924	84888072	chr9:84887670-84888396
chr9	84888099	84888429	chr9:84887670-84888396
chr9	84891939	84892113	chr9:84891963-84892077
chr9	84896469	84896580	chr9:84896496-84896565
chr9	84912289	84912492	chr9:84912296-84912455
chr9	84964694	84964801	chr9:84964718-84964763
chr9	85011594	85011735	chr9:85011622-85011715
chr9	85020074	85020254	chr9:85020102-85020225
chr9	85020789	85021179	chr9:85020822-85021154
chr9	85041174	85041333	chr9:85041206-85041291
chr9	85041869	85041975	chr9:85041896-85041941
chr9	85043174	85043421	chr9:85043113-85043395
chr9	85064524	85064734	chr9:85064555-85064700
chr9	85066494	85066660	chr9:85066517-85066644
chr9	85068249	85068385	chr9:85068259-85068373
chr9	85068519	85068941	chr9:85068552-85068914
chr9	85069814	85070159	chr9:85069821-85070140
chr9	85106844	85106991	chr9:85106866-85106969
chr9	85111649	85111792	chr9:85111629-85111773
chr9	85135564	85135877	chr9:85135588-85135855
chr9	85139409	85139588	chr9:85139433-85139570
chr9	85150944	85151185	chr9:85150968-85151147
chr9	85178964	85179051	chr9:85178943-85179056
chr9	121326214	121328140	chr9:121326224-121364867
chr9	121328149	121328630	chr9:121326224-121364867
chr9	121328634	121328732	chr9:121326224-121364867
chr9	121328749	121328844	chr9:121326224-121364867
chr9	121328849	121330656	chr9:121326224-121364867
chr9	121330979	121333127	chr9:121326224-121364867
chr9	121333399	121333721	chr9:121326224-121364867
chr9	121333724	121333812	chr9:121326224-121364867
chr9	121333814	121334463	chr9:121326224-121364867
chr9	121334494	121334565	chr9:121326224-121364867
chr9	121334569	121335971	chr9:121326224-121364867
chr9	121335984	121336168	chr9:121326224-121364867
chr9	121336174	121337122	chr9:121326224-121364867
chr9	121337169	121337249	chr9:121326224-121364867
chr9	121337364	121337465	chr9:121326224-121364867
chr9	121337569	121337654	chr9:121326224-121364867
chr9	121338074	121338158	chr9:121326224-121364867
chr9	121338159	121338259	chr9:121326224-121364867
chr9	121338289	121338370	chr9:121326224-121364867
chr9	121338424	121338537	chr9:121326224-121364867
chr9	121338864	121338943	chr9:121326224-121364867
chr9	121338974	121339158	chr9:121326224-121364867
chr9	121339214	121339468	chr9:121326224-121364867
chr9	121339504	121339805	chr9:121326224-121364867
chr9	121339814	121340263	chr9:121326224-121364867
chr9	121340264	121341098	chr9:121326224-121364867
chr9	121341109	121341230	chr9:121326224-121364867
chr9	121341249	121345435	chr9:121326224-121364867
chr9	121345509	121347969	chr9:121326224-121364867
chr9	121348019	121348205	chr9:121326224-121364867
chr9	121348294	121348446	chr9:121326224-121364867
chr9	121348694	121348771	chr9:121326224-121364867
chr9	121348914	121349067	chr9:121326224-121364867
chr9	121349539	121349734	chr9:121326224-121364867
chr9	121349799	121349900	chr9:121326224-121364867

chr9	121350049	121350130	chr9:121326224-121364867
chr9	121350224	121350477	chr9:121326224-121364867
chr9	121350479	121350775	chr9:121326224-121364867
chr9	121350779	121351148	chr9:121326224-121364867
chr9	121351194	121351420	chr9:121326224-121364867
chr9	121351809	121352861	chr9:121326224-121364867
chr9	121352969	121353078	chr9:121326224-121364867
chr9	121353089	121353171	chr9:121326224-121364867
chr9	121353554	121353677	chr9:121326224-121364867
chr9	121353714	121353839	chr9:121326224-121364867
chr9	121353884	121354237	chr9:121326224-121364867
chr9	121354319	121354405	chr9:121326224-121364867
chr9	121354414	121355930	chr9:121326224-121364867
chr9	121355934	121356014	chr9:121326224-121364867
chr9	121356034	121356682	chr9:121326224-121364867
chr9	121356694	121357748	chr9:121326224-121364867
chr9	121357759	121358662	chr9:121326224-121364867
chr9	121358664	121359166	chr9:121326224-121364867
chr9	121359169	121360814	chr9:121326224-121364867
chr9	121360819	121362830	chr9:121326224-121364867
chr9	121362899	121362976	chr9:121326224-121364867
chr9	121363074	121363590	chr9:121326224-121364867
chr9	121363894	121364893	chr9:121326224-121364867
chr9	121406579	121406725	chr9:121406609-121406687
chr9	121450159	121450307	chr9:121450182-121450281
chr9	121468069	121468253	chr9:121468104-121468235
chr9	121494969	121495071	chr9:121494991-121495055
chr9	129277012	129277105	chr9:129277037-129277089
chr9	129278672	129278989	chr9:129278695-129278960
chr9	129280627	129281220	chr9:129280633-129281208
chr9	129289867	129290118	chr9:129289896-129290075
chr9	129292437	129292645	chr9:129292462-129292622
chr9	129326907	129329130	chr9:129326937-129365987
chr9	129329427	129329635	chr9:129326937-129365987
chr9	129329677	129329915	chr9:129326937-129365987
chr9	129329937	129330767	chr9:129326937-129365987
chr9	129331027	129333401	chr9:129326937-129365987
chr9	129333402	129335038	chr9:129326937-129365987
chr9	129335077	129335439	chr9:129326937-129365987
chr9	129336387	129336472	chr9:129326937-129365987
chr9	129336727	129337953	chr9:129326937-129365987
chr9	129338172	129338245	chr9:129326937-129365987
chr9	129338542	129338626	chr9:129326937-129365987
chr9	129338642	129338863	chr9:129326937-129365987
chr9	129338867	129339297	chr9:129326937-129365987
chr9	129339592	129339948	chr9:129326937-129365987
chr9	129340397	129340806	chr9:129326937-129365987
chr9	129341082	129341282	chr9:129326937-129365987
chr9	129341332	129341841	chr9:129326937-129365987
chr9	129343127	129344223	chr9:129326937-129365987
chr9	129344242	129346049	chr9:129326937-129365987
chr9	129346172	129346442	chr9:129326937-129365987
chr9	129346762	129347124	chr9:129326937-129365987
chr9	129347412	129352110	chr9:129326937-129365987
chr9	129352412	129352730	chr9:129326937-129365987
chr9	129353012	129353285	chr9:129326937-129365987
chr9	129353287	129353814	chr9:129326937-129365987
chr9	129354092	129355207	chr9:129326937-129365987
chr9	129355522	129355710	chr9:129326937-129365987
chr9	129355742	129356135	chr9:129326937-129365987
chr9	129356442	129357006	chr9:129326937-129365987
chr9	129357017	129357115	chr9:129326937-129365987
chr9	129357117	129357253	chr9:129326937-129365987

chr9	129357687	129358973	chr9:129326937-129365987
chr9	129359247	129359470	chr9:129326937-129365987
chr9	129359572	129359958	chr9:129326937-129365987
chr9	129359962	129360357	chr9:129326937-129365987
chr9	129360367	129360831	chr9:129326937-129365987
chr9	129361232	129361388	chr9:129326937-129365987
chr9	129361512	129361592	chr9:129326937-129365987
chr9	129361657	129361732	chr9:129326937-129365987
chr9	129361752	129362469	chr9:129326937-129365987
chr9	129362737	129362912	chr9:129326937-129365987
chr9	129363557	129363674	chr9:129326937-129365987
chr9	129363872	129364264	chr9:129326937-129365987
chr9	129364557	129364765	chr9:129326937-129365987
chr9	129365037	129365353	chr9:129326937-129365987
chr9	129365442	129365524	chr9:129326937-129365987
chr9	129365812	129366029	chr9:129326937-129365987
chr9	129370367	129370751	chr9:129370391-129370740
chr9	129371397	129371538	chr9:129371422-129371493
chr9	129374702	129374830	chr9:129374724-129374798
chr9	129375702	129376006	chr9:129375727-129375985
chr9	129403002	129403147	chr9:129403034-129403111
chr9	129403227	129403628	chr9:129403255-129403572
chr10	2783515	2783690	chr10:2783509-2819554
chr10	2783700	2783780	chr10:2783509-2819554
chr10	2784055	2784206	chr10:2783509-2819554
chr10	2784315	2784430	chr10:2783509-2819554
chr10	2784690	2785640	chr10:2783509-2819554
chr10	2785755	2785883	chr10:2783509-2819554
chr10	2786370	2787513	chr10:2783509-2819554
chr10	2787815	2790314	chr10:2783509-2819554
chr10	2790590	2791503	chr10:2783509-2819554
chr10	2791620	2791730	chr10:2783509-2819554
chr10	2792340	2793348	chr10:2783509-2819554
chr10	2793350	2793897	chr10:2783509-2819554
chr10	2793905	2794459	chr10:2783509-2819554
chr10	2794475	2796115	chr10:2783509-2819554
chr10	2796385	2797139	chr10:2783509-2819554
chr10	2797660	2798530	chr10:2783509-2819554
chr10	2798695	2798971	chr10:2783509-2819554
chr10	2799055	2799501	chr10:2783509-2819554
chr10	2799565	2800282	chr10:2783509-2819554
chr10	2800310	2801115	chr10:2783509-2819554
chr10	2801215	2801611	chr10:2783509-2819554
chr10	2801825	2803095	chr10:2783509-2819554
chr10	2803175	2803768	chr10:2783509-2819554
chr10	2804080	2804160	chr10:2783509-2819554
chr10	2804180	2806122	chr10:2783509-2819554
chr10	2806405	2807715	chr10:2783509-2819554
chr10	2807730	2808921	chr10:2783509-2819554
chr10	2809215	2809756	chr10:2783509-2819554
chr10	2809910	2809985	chr10:2783509-2819554
chr10	2812315	2812381	chr10:2783509-2819554
chr10	2812510	2812629	chr10:2783509-2819554
chr10	2812920	2813915	chr10:2783509-2819554
chr10	2813925	2814219	chr10:2783509-2819554
chr10	2814265	2818409	chr10:2783509-2819554
chr10	2818685	2819153	chr10:2783509-2819554
chr10	2819455	2819593	chr10:2783509-2819554
chr10	18429575	18430171	chr10:18429605-18430144
chr10	18439805	18440213	chr10:18439811-18440198
chr10	18470840	18471056	chr10:18470735-18471036
chr10	18523075	18523212	chr10:18523096-18523188
chr10	18527295	18527477	chr10:18527322-18527449

chr10	18549295	18550288	chr10:18549328-18550246
chr10	18580330	18580718	chr10:18580361-18580686
chr10	18629581	18629935	chr10:18629613-18629906
chr10	18689496	18689576	chr10:18689503-18690029
chr10	18689581	18690065	chr10:18689503-18690029
chr10	18690151	18690287	chr10:18690186-18690266
chr10	18690831	18691326	chr10:18690852-18691334
chr10	18744226	18744776	chr10:18744249-18744748
chr10	18787256	18787750	chr10:18787283-18787722
chr10	18789716	18789906	chr10:18789740-18789877
chr10	18795376	18795521	chr10:18795399-18795476
chr10	18802051	18802477	chr10:18802043-18802589
chr10	18802511	18802588	chr10:18802043-18802589
chr10	18803131	18803305	chr10:18803164-18803298
chr10	18803396	18803512	chr10:18803439-18803459
chr10	18803881	18803992	chr10:18803908-18803970
chr10	18807231	18807377	chr10:18807264-18807345
chr10	18807816	18807920	chr10:18807838-18807897
chr10	18816491	18816660	chr10:18816516-18816626
chr10	18820751	18820931	chr10:18820777-18820890
chr10	18821936	18822297	chr10:18821965-18822265
chr10	18822981	18823194	chr10:18823004-18823156
chr10	18825006	18825145	chr10:18825029-18825125
chr10	18827076	18827312	chr10:18827108-18827294
chr10	18828101	18828670	chr10:18828122-18831106
chr10	18828686	18829260	chr10:18828122-18831106
chr10	18829266	18830521	chr10:18828122-18831106
chr10	18830821	18831004	chr10:18828122-18831106
chr10	18833226	18833332	chr10:18833252-18833316
chr10	26505211	26505830	chr10:26505235-26505814
chr10	26506516	26506616	chr10:26506538-26506598
chr10	26506736	26506950	chr10:26506770-26506920
chr10	26507941	26508226	chr10:26507971-26508205
chr10	26512781	26512933	chr10:26512815-26512906
chr10	26513446	26513612	chr10:26513467-26513580
chr10	26518556	26518742	chr10:26518590-26518706
chr10	26534841	26534958	chr10:26534849-26534929
chr10	26558026	26558136	chr10:26558047-26558102
chr10	26559536	26559721	chr10:26559568-26559685
chr10	26562531	26562648	chr10:26562564-26562629
chr10	26569916	26570062	chr10:26569937-26570016
chr10	26575246	26575457	chr10:26575273-26575423
chr10	26581366	26581541	chr10:26581393-26581501
chr10	26581801	26581955	chr10:26581830-26581920
chr10	26589681	26590061	chr10:26589716-26590048
chr10	26592931	26593530	chr10:26592962-26593491
chr10	61786021	61787051	chr10:61786055-61789584
chr10	61787071	61787798	chr10:61786055-61789584
chr10	61787801	61789448	chr10:61786055-61789584
chr10	61789456	61789602	chr10:61786055-61789584
chr10	61802396	61803639	chr10:61802429-61804561
chr10	61803646	61804581	chr10:61802429-61804561
chr10	61811206	61811383	chr10:61811232-61811362
chr10	61813411	61813507	chr10:61813434-61813485
chr10	61815381	61815809	chr10:61815415-61815796
chr10	61819061	61820531	chr10:61819091-61820501
chr10	61821656	61821755	chr10:61821678-61823012
chr10	61822016	61823032	chr10:61821678-61823012
chr10	61823881	61824059	chr10:61823914-61824050
chr10	61827661	61827807	chr10:61827692-61827767
chr10	61828371	61828729	chr10:61828394-61836213
chr10	61828736	61836229	chr10:61828394-61836213
chr10	61840271	61840387	chr10:61840294-61840378

chr10	61841871	61841990	chr10:61841907-61841934
chr10	61842231	61842520	chr10:61842265-61842495
chr10	61843221	61843392	chr10:61843249-61843375
chr10	61844336	61844619	chr10:61844359-61844588
chr10	61844881	61845028	chr10:61844914-61845011
chr10	61846266	61846655	chr10:61846300-61846642
chr10	61847881	61848153	chr10:61847904-61848118
chr10	61865636	61865849	chr10:61865662-61865817
chr10	61867921	61868054	chr10:61867945-61868044
chr10	61868566	61868842	chr10:61868587-61868812
chr10	61873961	61874112	chr10:61873982-61874089
chr10	61894006	61894151	chr10:61894028-61894131
chr10	61896186	61896306	chr10:61896221-61896720
chr10	61896331	61896752	chr10:61896221-61896720
chr10	61898241	61898497	chr10:61898274-61898845
chr10	61898501	61898885	chr10:61898274-61898845
chr10	61899071	61899538	chr10:61899095-61899158;chr10:61899172-61899514
chr10	61899721	61899827	chr10:61899742-61899798
chr10	61900076	61900781	chr10:61900109-61900774
chr10	61905701	61905797	chr10:61905725-61905779
chr10	61926316	61926436	chr10:61926348-61926411
chr10	61926556	61926694	chr10:61926581-61926654
chr10	61932031	61932176	chr10:61932065-61932161
chr10	61932631	61932779	chr10:61932657-61932756
chr10	61932811	61932959	chr10:61932845-61932944
chr10	61941051	61941214	chr10:61941086-61941185
chr10	61946451	61946706	chr10:61946472-61946670
chr10	61955871	61956030	chr10:61955902-61956001
chr10	61956256	61956394	chr10:61956284-61956383
chr10	61958076	61958316	chr10:61958097-61958295
chr10	61959856	61960012	chr10:61959886-61959985
chr10	61962731	61962869	chr10:61962759-61962858
chr10	61965526	61965660	chr10:61965549-61965648
chr10	61967771	61968015	chr10:61967793-61967991
chr10	61973136	61973284	chr10:61973169-61973268
chr10	61994421	61994557	chr10:61994445-61994544
chr10	62021581	62021734	chr10:62021616-62021715
chr10	62023571	62023821	chr10:62023592-62023778
chr10	62026371	62026682	chr10:62026404-62026445;chr10:62026499-62026645
chr10	62029866	62030001	chr10:62029888-62029987
chr10	62038501	62038659	chr10:62038531-62038630
chr10	62038781	62038919	chr10:62038807-62038906
chr10	62039271	62039407	chr10:62039295-62039397
chr10	62060041	62060259	chr10:62060076-62060240
chr10	62149151	62149648	chr10:62149182-62149634
chr10	62267236	62268419	chr10:62267258-62268383
chr10	62332231	62332647	chr10:62332222-62332714
chr10	62332676	62332756	chr10:62332222-62332714
chr10	62374916	62375019	chr10:62374943-62374982
chr10	62427796	62427902	chr10:62427825-62427892
chr10	62431481	62431664	chr10:62431505-62431625
chr10	62492991	62493085	chr10:62493020-62493284
chr10	62493096	62493309	chr10:62493020-62493284
chr10	94479811	94480949	chr10:94479832-94498436
chr10	94481516	94482186	chr10:94479832-94498436
chr10	94482196	94482539	chr10:94479832-94498436
chr10	94482541	94483553	chr10:94479832-94498436
chr10	94483896	94484244	chr10:94479832-94498436
chr10	94484271	94485086	chr10:94479832-94498436
chr10	94485111	94485587	chr10:94479832-94498436
chr10	94485871	94486090	chr10:94479832-94498436
chr10	94486376	94488105	chr10:94479832-94498436
chr10	94488216	94488699	chr10:94479832-94498436

chr10	94488701	94488906	chr10:94479832-94498436
chr10	94489371	94489515	chr10:94479832-94498436
chr10	94489521	94490241	chr10:94479832-94498436
chr10	94490701	94490972	chr10:94479832-94498436
chr10	94491201	94491668	chr10:94479832-94498436
chr10	94492011	94493699	chr10:94479832-94498436
chr10	94493991	94494835	chr10:94479832-94498436
chr10	94494846	94495042	chr10:94479832-94498436
chr10	94495471	94496242	chr10:94479832-94498436
chr10	94496566	94496650	chr10:94479832-94498436
chr10	94496716	94496793	chr10:94479832-94498436
chr10	94496971	94497510	chr10:94479832-94498436
chr10	94498171	94498451	chr10:94479832-94498436
chr10	103989914	103990886	chr10:103989945-103990858
chr10	103991314	103991555	chr10:103991344-103991547
chr10	103991684	103991877	chr10:103991719-103991849
chr10	104001064	104001268	chr10:104001089-104001231
chr10	121410850	121411378	chr10:121410881-121411367
chr10	121429335	121429721	chr10:121429362-121429689
chr10	121431745	121432205	chr10:121431766-121432168
chr10	121435950	121437293	chr10:121435975-121437331
chr10	129894899	129897551	chr10:129894924-129897519
chr10	129899499	129899994	chr10:129899521-129899965
chr10	129900809	129902203	chr10:129900842-129907687
chr10	129902214	129907708	chr10:129900842-129907687
chr10	129908614	129908822	chr10:129908641-129908797
chr10	129909884	129910089	chr10:129909908-129910080
chr10	129910159	129910340	chr10:129910190-129910309
chr10	129910369	129910722	chr10:129910396-129910709
chr10	129911664	129911885	chr10:129911690-129911866
chr10	129913169	129914290	chr10:129913191-129914271
chr10	129914729	129914839	chr10:129914754-129914800
chr10	129917489	129917603	chr10:129917516-129917583
chr10	129921119	129921287	chr10:129921144-129921260
chr10	129921329	129921462	chr10:129921354-129921433
chr10	129923804	129924053	chr10:129923839-129924020
chr10	129924339	129924495	chr10:129924362-129924468
chr11	22359887	22360201	chr11:22359916-22360165
chr11	22363042	22363360	chr11:22363073-22363326
chr11	22364757	22364940	chr11:22364792-22364911
chr11	22380932	22381112	chr11:22380958-22381073
chr11	22382407	22382567	chr11:22382442-22382530
chr11	22384262	22384401	chr11:22384284-22384371
chr11	22387057	22387273	chr11:22387092-22387235
chr11	22391562	22391765	chr11:22391584-22391734
chr11	22396277	22396462	chr11:22396300-22396433
chr11	22397502	22397653	chr11:22397527-22397638
chr11	22398057	22398240	chr11:22398090-22398218
chr11	22398927	22400691	chr11:22398950-22401046
chr11	22400697	22401076	chr11:22398950-22401046
chr11	24368002	24368998	chr11:24368035-24411930
chr11	24369042	24369205	chr11:24368035-24411930
chr11	24369222	24369438	chr11:24368035-24411930
chr11	24369457	24369841	chr11:24368035-24411930
chr11	24369842	24372705	chr11:24368035-24411930
chr11	24372707	24373254	chr11:24368035-24411930
chr11	24373262	24373355	chr11:24368035-24411930
chr11	24373372	24373447	chr11:24368035-24411930
chr11	24373562	24373702	chr11:24368035-24411930
chr11	24373732	24373951	chr11:24368035-24411930
chr11	24373957	24374114	chr11:24368035-24411930
chr11	24374117	24374572	chr11:24368035-24411930
chr11	24374862	24375425	chr11:24368035-24411930

chr11	24375442	24375733	chr11:24368035-24411930
chr11	24375782	24376610	chr11:24368035-24411930
chr11	24376887	24377414	chr11:24368035-24411930
chr11	24377977	24378513	chr11:24368035-24411930
chr11	24378682	24380423	chr11:24368035-24411930
chr11	24380692	24381361	chr11:24368035-24411930
chr11	24381482	24382264	chr11:24368035-24411930
chr11	24382542	24382844	chr11:24368035-24411930
chr11	24382877	24383004	chr11:24368035-24411930
chr11	24383182	24383259	chr11:24368035-24411930
chr11	24383267	24383552	chr11:24368035-24411930
chr11	24383577	24383853	chr11:24368035-24411930
chr11	24384042	24384110	chr11:24368035-24411930
chr11	24384137	24384262	chr11:24368035-24411930
chr11	24384322	24384505	chr11:24368035-24411930
chr11	24384552	24384673	chr11:24368035-24411930
chr11	24384677	24384957	chr11:24368035-24411930
chr11	24385012	24385219	chr11:24368035-24411930
chr11	24385292	24385618	chr11:24368035-24411930
chr11	24385677	24385828	chr11:24368035-24411930
chr11	24386067	24386501	chr11:24368035-24411930
chr11	24386502	24388296	chr11:24368035-24411930
chr11	24388302	24389568	chr11:24368035-24411930
chr11	24389577	24391131	chr11:24368035-24411930
chr11	24391132	24391408	chr11:24368035-24411930
chr11	24392752	24392826	chr11:24368035-24411930
chr11	24393097	24394866	chr11:24368035-24411930
chr11	24394872	24396901	chr11:24368035-24411930
chr11	24396942	24397304	chr11:24368035-24411930
chr11	24397347	24397917	chr11:24368035-24411930
chr11	24397922	24400698	chr11:24368035-24411930
chr11	24400702	24401079	chr11:24368035-24411930
chr11	24401092	24401271	chr11:24368035-24411930
chr11	24401272	24402274	chr11:24368035-24411930
chr11	24402577	24402731	chr11:24368035-24411930
chr11	24402752	24402941	chr11:24368035-24411930
chr11	24403037	24403179	chr11:24368035-24411930
chr11	24403277	24403348	chr11:24368035-24411930
chr11	24403372	24403830	chr11:24368035-24411930
chr11	24403842	24404304	chr11:24368035-24411930
chr11	24404307	24404389	chr11:24368035-24411930
chr11	24404407	24404995	chr11:24368035-24411930
chr11	24405267	24406696	chr11:24368035-24411930
chr11	24406732	24406882	chr11:24368035-24411930
chr11	24406917	24407074	chr11:24368035-24411930
chr11	24407087	24407541	chr11:24368035-24411930
chr11	24407542	24408889	chr11:24368035-24411930
chr11	24411057	24411929	chr11:24368035-24411930
chr11	31809815	31811120	chr11:31809839-31811567
chr11	31811140	31811462	chr11:31809839-31811567
chr11	31811480	31811583	chr11:31809839-31811567
chr11	31812235	31812447	chr11:31812257-31812408
chr11	31814950	31815141	chr11:31814985-31815101
chr11	31815170	31815387	chr11:31815199-31815350
chr11	31815545	31815700	chr11:31815579-31815662
chr11	31816145	31816368	chr11:31816177-31816336
chr11	31822215	31822422	chr11:31822238-31822404
chr11	31823085	31823358	chr11:31823108-31823324
chr11	31823390	31823503	chr11:31823418-31823460
chr11	31824230	31824400	chr11:31824251-31824382
chr11	31827915	31828032	chr11:31827949-31828010
chr11	31828365	31828504	chr11:31828396-31828473
chr11	31832350	31832950	chr11:31832375-31832901

chr11	31833615	31833760	chr11:31833640-31833731
chr11	31839335	31839538	chr11:31839356-31839509
chr11	60792157	60795575	chr11:60792181-60821968
chr11	60795967	60796694	chr11:60792181-60821968
chr11	60796982	60797095	chr11:60792181-60821968
chr11	60797187	60798285	chr11:60792181-60821968
chr11	60798922	60799503	chr11:60792181-60821968
chr11	60799777	60800023	chr11:60792181-60821968
chr11	60800052	60800152	chr11:60792181-60821968
chr11	60800177	60800325	chr11:60792181-60821968
chr11	60800632	60801040	chr11:60792181-60821968
chr11	60801042	60801894	chr11:60792181-60821968
chr11	60802157	60804180	chr11:60792181-60821968
chr11	60804417	60805655	chr11:60792181-60821968
chr11	60805962	60806043	chr11:60792181-60821968
chr11	60806322	60806792	chr11:60792181-60821968
chr11	60807117	60807998	chr11:60792181-60821968
chr11	60808277	60808793	chr11:60792181-60821968
chr11	60809097	60810090	chr11:60792181-60821968
chr11	60810197	60811516	chr11:60792181-60821968
chr11	60811532	60811634	chr11:60792181-60821968
chr11	60811637	60811889	chr11:60792181-60821968
chr11	60811892	60812185	chr11:60792181-60821968
chr11	60812202	60813383	chr11:60792181-60821968
chr11	60813387	60813503	chr11:60792181-60821968
chr11	60813782	60816642	chr11:60792181-60821968
chr11	60816647	60817031	chr11:60792181-60821968
chr11	60817337	60817985	chr11:60792181-60821968
chr11	60818257	60819265	chr11:60792181-60821968
chr11	60819327	60820933	chr11:60792181-60821968
chr11	60821232	60821841	chr11:60792181-60821968
chr11	60823047	60823292	chr11:60823073-60823265
chr11	60828412	60828754	chr11:60828436-60835322
chr11	60828757	60831064	chr11:60828436-60835322
chr11	60831342	60831600	chr11:60828436-60835322
chr11	60831887	60832327	chr11:60828436-60835322
chr11	60832642	60832887	chr11:60828436-60835322
chr11	60833187	60833260	chr11:60828436-60835322
chr11	60833272	60834370	chr11:60828436-60835322
chr11	60834402	60834809	chr11:60828436-60835322
chr11	60834862	60835164	chr11:60828436-60835322
chr11	60835217	60835362	chr11:60828436-60835322
chr11	60836277	60836386	chr11:60836313-60836347
chr11	60848062	60848180	chr11:60848087-60848143
chr11	60859747	60859881	chr11:60859768-60859862
chr11	61520077	61520293	chr11:61520108-61520262
chr11	61522837	61522936	chr11:61522860-61522914
chr11	61533067	61533212	chr11:61533096-61533184
chr11	61533402	61533713	chr11:61533429-61533693
chr11	61536707	61536805	chr11:61536731-61536793
chr11	61537682	61538019	chr11:61537717-61537997
chr11	61538942	61539257	chr11:61538971-61539222
chr11	61539267	61539451	chr11:61539300-61539424
chr11	61541417	61541659	chr11:61541438-61541634
chr11	61543492	61543637	chr11:61543527-61543604
chr11	61543772	61543936	chr11:61543793-61543904
chr11	61544232	61544376	chr11:61544258-61544349
chr11	61544707	61544953	chr11:61544735-61544936
chr11	61545182	61545354	chr11:61545205-61545317
chr11	61545822	61545990	chr11:61545851-61545961
chr11	61546712	61546914	chr11:61546734-61546895
chr11	61546942	61547077	chr11:61546969-61547042
chr11	61547292	61547439	chr11:61547313-61547402

chr11	61547662	61547778	chr11:61547693-61547762
chr11	61548152	61548302	chr11:61548183-61548264
chr11	61548402	61548548	chr11:61548428-61548517
chr11	61548587	61548830	chr11:61548609-61548801
chr11	61549012	61549334	chr11:61549044-61549296
chr11	61550947	61551087	chr11:61550969-61551072
chr11	61551287	61551440	chr11:61551322-61551397
chr11	61551717	61551894	chr11:61551751-61551857
chr11	61553247	61553381	chr11:61553271-61553346
chr11	61553512	61553758	chr11:61553534-61553740
chr11	78363846	78366451	chr11:78363875-78369861
chr11	78366581	78369902	chr11:78363875-78369861
chr11	78372466	78372681	chr11:78372493-78372636
chr11	78379956	78381635	chr11:78379981-78381596
chr11	78383051	78383397	chr11:78383077-78383374
chr11	78387171	78387464	chr11:78387196-78387432
chr11	78399076	78399283	chr11:78399098-78399271
chr11	78412541	78413491	chr11:78412570-78413448
chr11	78419381	78419587	chr11:78419405-78419560
chr11	78423491	78423789	chr11:78423526-78423759
chr11	78431376	78431490	chr11:78431414-78431435
chr11	78433691	78434001	chr11:78433712-78433962
chr11	78435946	78436091	chr11:78435977-78436052
chr11	78437101	78437311	chr11:78437123-78437267
chr11	78440396	78440719	chr11:78440420-78440688
chr11	78443336	78443647	chr11:78443360-78443622
chr11	78449471	78449653	chr11:78449495-78449615
chr11	78460261	78460404	chr11:78460294-78460378
chr11	78467261	78467612	chr11:78467289-78467575
chr11	78467826	78468120	chr11:78467849-78468066
chr11	78482011	78482217	chr11:78482036-78482183
chr11	78489611	78489713	chr11:78489646-78489673
chr11	78497911	78498167	chr11:78497942-78498128
chr11	78516306	78516563	chr11:78516336-78516537
chr11	78523131	78523396	chr11:78523166-78523361
chr11	78525306	78525489	chr11:78525338-78525466
chr11	78565126	78565401	chr11:78565148-78565359
chr11	78566976	78567256	chr11:78567008-78567223
chr11	78573981	78574199	chr11:78574006-78574177
chr11	78600806	78601088	chr11:78600829-78601065
chr11	78602261	78602404	chr11:78602282-78602381
chr11	78614201	78614604	chr11:78614232-78614568
chr11	78614826	78614960	chr11:78614850-78614931
chr11	78635471	78635675	chr11:78635500-78635791
chr11	78635676	78635817	chr11:78635500-78635791
chr11	78673166	78673298	chr11:78673187-78673264
chr11	78725581	78725828	chr11:78725615-78725795
chr11	78732596	78732879	chr11:78732619-78732838
chr11	78775611	78776077	chr11:78775643-78776052
chr11	78778926	78779531	chr11:78778947-78779501
chr11	78780736	78781089	chr11:78780766-78781054
chr11	78803851	78803965	chr11:78803892-78803928
chr11	78804216	78804434	chr11:78804249-78804396
chr11	78804481	78804663	chr11:78804509-78804631
chr11	78804751	78804891	chr11:78804779-78804880
chr11	78807031	78807979	chr11:78807055-78809048
chr11	78808001	78808410	chr11:78807055-78809048
chr11	78808416	78808877	chr11:78807055-78809048
chr11	78808886	78809060	chr11:78807055-78809048
chr11	78859726	78859864	chr11:78859754-78859851
chr11	78904031	78904210	chr11:78904066-78904205
chr11	78926821	78926982	chr11:78926852-78926954
chr11	78932001	78932209	chr11:78932024-78932168

chr11	78980596	78980753	chr11:78980630-78980718
chr11	79008501	79008614	chr11:79008532-79008588
chr11	79076011	79076575	chr11:79076038-79076537
chr11	79149981	79150262	chr11:79150005-79150244
chr11	79151521	79151894	chr11:79151552-79151992
chr11	79151921	79152036	chr11:79151552-79151992
chr11	85847851	85848738	chr11:85847881-85869944
chr11	85848761	85848836	chr11:85847881-85869944
chr11	85849441	85850742	chr11:85847881-85869944
chr11	85851366	85855104	chr11:85847881-85869944
chr11	85855371	85857021	chr11:85847881-85869944
chr11	85857226	85857419	chr11:85847881-85869944
chr11	85857716	85858590	chr11:85847881-85869944
chr11	85858891	85860105	chr11:85847881-85869944
chr11	85860386	85860737	chr11:85847881-85869944
chr11	85860761	85861584	chr11:85847881-85869944
chr11	85861606	85863843	chr11:85847881-85869944
chr11	85863856	85864227	chr11:85847881-85869944
chr11	85864231	85864312	chr11:85847881-85869944
chr11	85864391	85864926	chr11:85847881-85869944
chr11	85865141	85865468	chr11:85847881-85869944
chr11	85865756	85866494	chr11:85847881-85869944
chr11	85866556	85866805	chr11:85847881-85869944
chr11	85866951	85868210	chr11:85847881-85869944
chr11	85868526	85869964	chr11:85847881-85869944
chr11	85903794	85903967	chr11:85903828-85903956
chr11	85904449	85904526	chr11:85904474-85904524
chr11	91999700	92000741	chr11:91999722-92036512
chr11	92000745	92001223	chr11:91999722-92036512
chr11	92001230	92001640	chr11:91999722-92036512
chr11	92001660	92003561	chr11:91999722-92036512
chr11	92003865	92007840	chr11:91999722-92036512
chr11	92007845	92009156	chr11:91999722-92036512
chr11	92009160	92010531	chr11:91999722-92036512
chr11	92010620	92010704	chr11:91999722-92036512
chr11	92010710	92010848	chr11:91999722-92036512
chr11	92010875	92010993	chr11:91999722-92036512
chr11	92011220	92011774	chr11:91999722-92036512
chr11	92011790	92012685	chr11:91999722-92036512
chr11	92012710	92014513	chr11:91999722-92036512
chr11	92014520	92015087	chr11:91999722-92036512
chr11	92015330	92015559	chr11:91999722-92036512
chr11	92015830	92016114	chr11:91999722-92036512
chr11	92016125	92021342	chr11:91999722-92036512
chr11	92021365	92022667	chr11:91999722-92036512
chr11	92022730	92023420	chr11:91999722-92036512
chr11	92023425	92023659	chr11:91999722-92036512
chr11	92023960	92024251	chr11:91999722-92036512
chr11	92024280	92026092	chr11:91999722-92036512
chr11	92026260	92026684	chr11:91999722-92036512
chr11	92026720	92027187	chr11:91999722-92036512
chr11	92027250	92027838	chr11:91999722-92036512
chr11	92027840	92035201	chr11:91999722-92036512
chr11	92035230	92035635	chr11:91999722-92036512
chr11	92035640	92036409	chr11:91999722-92036512
chr11	92036415	92036553	chr11:91999722-92036512
chr11	113357911	113357976	chr11:113357963-113424241
chr11	113358111	113358868	chr11:113357963-113424241
chr11	113359141	113360075	chr11:113357963-113424241
chr11	113360081	113362433	chr11:113357963-113424241
chr11	113362436	113363337	chr11:113357963-113424241
chr11	113363341	113363595	chr11:113357963-113424241
chr11	113363616	113364851	chr11:113357963-113424241

chr11	113365171	113366444	chr11:113357963-113424241
chr11	113366446	113368534	chr11:113357963-113424241
chr11	113368536	113368831	chr11:113357963-113424241
chr11	113368836	113369205	chr11:113357963-113424241
chr11	113369241	113369750	chr11:113357963-113424241
chr11	113370066	113370151	chr11:113357963-113424241
chr11	113370156	113370604	chr11:113357963-113424241
chr11	113370871	113371380	chr11:113357963-113424241
chr11	113371616	113371844	chr11:113357963-113424241
chr11	113371846	113372879	chr11:113357963-113424241
chr11	113373056	113373589	chr11:113357963-113424241
chr11	113373866	113374106	chr11:113357963-113424241
chr11	113374411	113375691	chr11:113357963-113424241
chr11	113375696	113378089	chr11:113357963-113424241
chr11	113378196	113378320	chr11:113357963-113424241
chr11	113378436	113378583	chr11:113357963-113424241
chr11	113378666	113379977	chr11:113357963-113424241
chr11	113380001	113381970	chr11:113357963-113424241
chr11	113382301	113385078	chr11:113357963-113424241
chr11	113385081	113387858	chr11:113357963-113424241
chr11	113387861	113388185	chr11:113357963-113424241
chr11	113388186	113388259	chr11:113357963-113424241
chr11	113388326	113391411	chr11:113357963-113424241
chr11	113391416	113392096	chr11:113357963-113424241
chr11	113392161	113393970	chr11:113357963-113424241
chr11	113393971	113394211	chr11:113357963-113424241
chr11	113394296	113394362	chr11:113357963-113424241
chr11	113394371	113394446	chr11:113357963-113424241
chr11	113395561	113396092	chr11:113357963-113424241
chr11	113396146	113396335	chr11:113357963-113424241
chr11	113396351	113396486	chr11:113357963-113424241
chr11	113396501	113398185	chr11:113357963-113424241
chr11	113398191	113399147	chr11:113357963-113424241
chr11	113399151	113401569	chr11:113357963-113424241
chr11	113401581	113405894	chr11:113357963-113424241
chr11	113406191	113406263	chr11:113357963-113424241
chr11	113407381	113413999	chr11:113357963-113424241
chr11	113414006	113414146	chr11:113357963-113424241
chr11	113414156	113415323	chr11:113357963-113424241
chr11	113415326	113416557	chr11:113357963-113424241
chr11	113416566	113418764	chr11:113357963-113424241
chr11	113419031	113419626	chr11:113357963-113424241
chr11	113419636	113421445	chr11:113357963-113424241
chr11	113421746	113422383	chr11:113357963-113424241
chr11	113422391	113423433	chr11:113357963-113424241
chr11	113423441	113423515	chr11:113357963-113424241
chr11	113423846	113424260	chr11:113357963-113424241
chr11	130714053	130714218	chr11:130714085-130714931
chr11	130714668	130714940	chr11:130714085-130714931
chr11	130715323	130715759	chr11:130715348-130719061
chr11	130715783	130718104	chr11:130715348-130719061
chr11	130718128	130718208	chr11:130715348-130719061
chr11	130718228	130719101	chr11:130715348-130719061
chr11	130724598	130724820	chr11:130724621-130724787
chr11	130731648	130731744	chr11:130731670-130731723
chr11	130731838	130732675	chr11:130731860-130732661
chr11	130735333	130737520	chr11:130735364-130740142
chr11	130737528	130739812	chr11:130735364-130740142
chr11	130739813	130740165	chr11:130735364-130740142
chr11	130792013	130792190	chr11:130792048-130873953
chr11	130792388	130792854	chr11:130792048-130873953
chr11	130792943	130793016	chr11:130792048-130873953
chr11	130793018	130793092	chr11:130792048-130873953

chr11	130793193	130793711	chr11:130792048-130873953
chr11	130794018	130794135	chr11:130792048-130873953
chr11	130794198	130795725	chr11:130792048-130873953
chr11	130795733	130796147	chr11:130792048-130873953
chr11	130796148	130796213	chr11:130792048-130873953
chr11	130796528	130796604	chr11:130792048-130873953
chr11	130797138	130798084	chr11:130792048-130873953
chr11	130798093	130799983	chr11:130792048-130873953
chr11	130799988	130801425	chr11:130792048-130873953
chr11	130801438	130802415	chr11:130792048-130873953
chr11	130802518	130802601	chr11:130792048-130873953
chr11	130802618	130802717	chr11:130792048-130873953
chr11	130802773	130803049	chr11:130792048-130873953
chr11	130803053	130803916	chr11:130792048-130873953
chr11	130804278	130804699	chr11:130792048-130873953
chr11	130804713	130804899	chr11:130792048-130873953
chr11	130804958	130805036	chr11:130792048-130873953
chr11	130805323	130805462	chr11:130792048-130873953
chr11	130805513	130806186	chr11:130792048-130873953
chr11	130806203	130811198	chr11:130792048-130873953
chr11	130813708	130815785	chr11:130792048-130873953
chr11	130815788	130816482	chr11:130792048-130873953
chr11	130816558	130816744	chr11:130792048-130873953
chr11	130816773	130817118	chr11:130792048-130873953
chr11	130817123	130817422	chr11:130792048-130873953
chr11	130817428	130817874	chr11:130792048-130873953
chr11	130817938	130821835	chr11:130792048-130873953
chr11	130821943	130822999	chr11:130792048-130873953
chr11	130823008	130823710	chr11:130792048-130873953
chr11	130823713	130824058	chr11:130792048-130873953
chr11	130824103	130825647	chr11:130792048-130873953
chr11	130825658	130825747	chr11:130792048-130873953
chr11	130825753	130825856	chr11:130792048-130873953
chr11	130825858	130825935	chr11:130792048-130873953
chr11	130825943	130826037	chr11:130792048-130873953
chr11	130826043	130826293	chr11:130792048-130873953
chr11	130826563	130826643	chr11:130792048-130873953
chr11	130827418	130827703	chr11:130792048-130873953
chr11	130827713	130827897	chr11:130792048-130873953
chr11	130827898	130828165	chr11:130792048-130873953
chr11	130829323	130829392	chr11:130792048-130873953
chr11	130831153	130831268	chr11:130792048-130873953
chr11	130831693	130831772	chr11:130792048-130873953
chr11	130831793	130831955	chr11:130792048-130873953
chr11	130832168	130832270	chr11:130792048-130873953
chr11	130832988	130833059	chr11:130792048-130873953
chr11	130833103	130833200	chr11:130792048-130873953
chr11	130833563	130833702	chr11:130792048-130873953
chr11	130833738	130833840	chr11:130792048-130873953
chr11	130833843	130833926	chr11:130792048-130873953
chr11	130833953	130834103	chr11:130792048-130873953
chr11	130834228	130834637	chr11:130792048-130873953
chr11	130834663	130834952	chr11:130792048-130873953
chr11	130835008	130835156	chr11:130792048-130873953
chr11	130835203	130835351	chr11:130792048-130873953
chr11	130835393	130835470	chr11:130792048-130873953
chr11	130835718	130835798	chr11:130792048-130873953
chr11	130835958	130836028	chr11:130792048-130873953
chr11	130836168	130836299	chr11:130792048-130873953
chr11	130837313	130837445	chr11:130792048-130873953
chr11	130837473	130838426	chr11:130792048-130873953
chr11	130838433	130838861	chr11:130792048-130873953
chr11	130838868	130838942	chr11:130792048-130873953

chr11	130838953	130839500	chr11:130792048-130873953
chr11	130839508	130840070	chr11:130792048-130873953
chr11	130840348	130840544	chr11:130792048-130873953
chr11	130840563	130840641	chr11:130792048-130873953
chr11	130844853	130844942	chr11:130792048-130873953
chr11	130846363	130846447	chr11:130792048-130873953
chr11	130847133	130847210	chr11:130792048-130873953
chr11	130847428	130847691	chr11:130792048-130873953
chr11	130847703	130848803	chr11:130792048-130873953
chr11	130848843	130849068	chr11:130792048-130873953
chr11	130849128	130854171	chr11:130792048-130873953
chr11	130860188	130860258	chr11:130792048-130873953
chr11	130860273	130861354	chr11:130792048-130873953
chr11	130861368	130862256	chr11:130792048-130873953
chr11	130862258	130865522	chr11:130792048-130873953
chr11	130865528	130867515	chr11:130792048-130873953
chr11	130867518	130869275	chr11:130792048-130873953
chr11	130869278	130869959	chr11:130792048-130873953
chr11	130869973	130872415	chr11:130792048-130873953
chr11	130872448	130873491	chr11:130792048-130873953
chr11	130873523	130873706	chr11:130792048-130873953
chr11	130873713	130873987	chr11:130792048-130873953
chr11	130874638	130875902	chr11:130874374-130894976
chr11	130876228	130877227	chr11:130874374-130894976
chr11	130877238	130877707	chr11:130874374-130894976
chr11	130877708	130878466	chr11:130874374-130894976
chr11	130878743	130880935	chr11:130874374-130894976
chr11	130880978	130881477	chr11:130874374-130894976
chr11	130881478	130882717	chr11:130874374-130894976
chr11	130882718	130884541	chr11:130874374-130894976
chr11	130884548	130885885	chr11:130874374-130894976
chr11	130886088	130886324	chr11:130874374-130894976
chr11	130886398	130886615	chr11:130874374-130894976
chr11	130886618	130887696	chr11:130874374-130894976
chr11	130887983	130888729	chr11:130874374-130894976
chr11	130888803	130888994	chr11:130874374-130894976
chr11	130889063	130889166	chr11:130874374-130894976
chr11	130889828	130889916	chr11:130874374-130894976
chr11	130890008	130890129	chr11:130874374-130894976
chr11	130890143	130890280	chr11:130874374-130894976
chr11	130890283	130890356	chr11:130874374-130894976
chr11	130890358	130890556	chr11:130874374-130894976
chr11	130890563	130890785	chr11:130874374-130894976
chr11	130890828	130891334	chr11:130874374-130894976
chr11	130891348	130891494	chr11:130874374-130894976
chr11	130891498	130891929	chr11:130874374-130894976
chr11	130891933	130892058	chr11:130874374-130894976
chr11	130892093	130892161	chr11:130874374-130894976
chr11	130892403	130892595	chr11:130874374-130894976
chr11	130892608	130892960	chr11:130874374-130894976
chr11	130893088	130893174	chr11:130874374-130894976
chr11	130893723	130894087	chr11:130874374-130894976
chr11	130894088	130895006	chr11:130874374-130894976
chr11	130937358	130937880	chr11:130937379-130937842
chr11	130956323	130956534	chr11:130956344-130956516
chr11	130957353	130957533	chr11:130957376-130957497
chr11	133768154	133768723	chr11:133768188-133768686
chr11	133778539	133779209	chr11:133778458-133779172
chr11	133781759	133782047	chr11:133781780-133782013
chr11	133782164	133782268	chr11:133782189-133782257
chr11	133785419	133788231	chr11:133785184-133788197
chr11	133788894	133789027	chr11:133788918-133789000
chr11	133789614	133791329	chr11:133789636-133791292

chr11	133792034	133792143	chr11:133792071-133792117
chr11	133792434	133792655	chr11:133792463-133792625
chr11	133794679	133794829	chr11:133794714-133794799
chr11	133795599	133795893	chr11:133795633-133795860
chr11	133796789	133797012	chr11:133796810-133796986
chr11	133797634	133798201	chr11:133797660-133798159
chr11	133799544	133799714	chr11:133799565-133799677
chr11	133800854	133801067	chr11:133800878-133801029
chr11	133801319	133801495	chr11:133801347-133801464
chr11	133801514	133801732	chr11:133801549-133801690
chr11	133801934	133802140	chr11:133801965-133802108
chr11	133805479	133805698	chr11:133805511-133805657
chr11	133805924	133806125	chr11:133805947-133806089
chr11	133807244	133807423	chr11:133807270-133807388
chr11	133807679	133808030	chr11:133807704-133807995
chr11	133814089	133814297	chr11:133814114-133814261
chr11	133815934	133816210	chr11:133815955-133816187
chr11	133821799	133822151	chr11:133821827-133822111
chr11	133826564	133826920	chr11:133826585-133826880
chr12	2079929	2080024	chr12:2079951-2080367
chr12	2080029	2080387	chr12:2079951-2080367
chr12	2113759	2113937	chr12:2113793-2113902
chr12	2114059	2114133	chr12:2114051-2114125
chr12	2119974	2120046	chr12:2120000-2120776
chr12	2120344	2120795	chr12:2120000-2120776
chr12	2127354	2127488	chr12:2127378-2127458
chr12	2129069	2129179	chr12:2129091-2129698
chr12	2129189	2129714	chr12:2129091-2129698
chr12	2157489	2157813	chr12:2157517-2157776
chr12	2158274	2158658	chr12:2158299-2158629
chr12	2162169	2162802	chr12:2162200-2162777
chr12	2224354	2224754	chr12:2224389-2224711
chr12	2229469	2229618	chr12:2229490-2229596
chr12	2329119	2329338	chr12:2329140-2329305
chr12	2329669	2330068	chr12:2329702-2330020
chr12	2330749	2330894	chr12:2330780-2330861
chr12	2331344	2331624	chr12:2331365-2331586
chr12	2332504	2332680	chr12:2332530-2332645
chr12	2346234	2346799	chr12:2346269-2346768
chr12	2378909	2379128	chr12:2378941-2379107
chr12	2397154	2397401	chr12:2397184-2397370
chr12	2397809	2398120	chr12:2397833-2398103
chr12	2442209	2442399	chr12:2442241-2442370
chr12	2444324	2444499	chr12:2444353-2444478
chr12	2444634	2444885	chr12:2444669-2444879
chr12	2558119	2558296	chr12:2558141-2558281
chr12	2566699	2566880	chr12:2566732-2566872
chr12	2595234	2595458	chr12:2595269-2595428
chr12	2602334	2602580	chr12:2602355-2602552
chr12	2613574	2613719	chr12:2613601-2613705
chr12	2613979	2614125	chr12:2614007-2614111
chr12	2621944	2622174	chr12:2621977-2622150
chr12	2656594	2656729	chr12:2656615-2656690
chr12	2659084	2659218	chr12:2659108-2659199
chr12	2659674	2659860	chr12:2659709-2659822
chr12	2666099	2666173	chr12:2666116-2666143
chr12	2675564	2675778	chr12:2675587-2675748
chr12	2676699	2676989	chr12:2676734-2676960
chr12	2690734	2690976	chr12:2690755-2690963
chr12	2691964	2692135	chr12:2691987-2692108
chr12	2693644	2693753	chr12:2693668-2693783
chr12	2694514	2694694	chr12:2694541-2694662
chr12	2694969	2695076	chr12:2695000-2695070

chr12	2702344	2702528	chr12:2702378-2702511
chr12	2705014	2705405	chr12:2705039-2705372
chr12	2706369	2706469	chr12:2706395-2706455
chr12	2706569	2706693	chr12:2706602-2706662
chr12	2710984	2711172	chr12:2711019-2711126
chr12	2712494	2712976	chr12:2712515-2712939
chr12	2714214	2714356	chr12:2714246-2714334
chr12	2714819	2714987	chr12:2714844-2714952
chr12	2715744	2715858	chr12:2715776-2715829
chr12	2716114	2716333	chr12:2716149-2716296
chr12	2716544	2716655	chr12:2716586-2716606
chr12	2717014	2717274	chr12:2717045-2717229
chr12	2717644	2717894	chr12:2717676-2717878
chr12	2719684	2719903	chr12:2719706-2719865
chr12	2721039	2724147	chr12:2721068-2724137
chr12	2742769	2742908	chr12:2742794-2742878
chr12	2743439	2743574	chr12:2743462-2743546
chr12	2750589	2750947	chr12:2750623-2750925
chr12	2755524	2755726	chr12:2755549-2755698
chr12	2757599	2757708	chr12:2757640-2757673
chr12	2760199	2760969	chr12:2760228-2760934
chr12	2762974	2763088	chr12:2763000-2763066
chr12	2764279	2764435	chr12:2764312-2764404
chr12	2773964	2774179	chr12:2773990-2774156
chr12	2774719	2774902	chr12:2774746-2774874
chr12	2775754	2775962	chr12:2775781-2775948
chr12	2777779	2777952	chr12:2777665-2778201
chr12	2778014	2778224	chr12:2777665-2778201
chr12	2781269	2781407	chr12:2781294-2781386
chr12	2783624	2783837	chr12:2783649-2783808
chr12	2785119	2785899	chr12:2785152-2787063
chr12	2785904	2786109	chr12:2785152-2787063
chr12	2786114	2787091	chr12:2785152-2787063
chr12	2788584	2788999	chr12:2788609-2788962
chr12	2789494	2789787	chr12:2789528-2789741
chr12	2790794	2791037	chr12:2790819-2791014
chr12	2791084	2791240	chr12:2791115-2791220
chr12	2791629	2791897	chr12:2791600-2791844
chr12	2794874	2795037	chr12:2794901-2795008
chr12	2795304	2795449	chr12:2795331-2795435
chr12	2797579	2797976	chr12:2797612-2797945
chr12	2799004	2799396	chr12:2799028-2799376
chr12	2799944	2802138	chr12:2799972-2802108
chr12	7941966	7942320	chr12:7941991-7942361
chr12	7942326	7942400	chr12:7941991-7942361
chr12	7945516	7945589	chr12:7945545-7945808
chr12	7945616	7945686	chr12:7945545-7945808
chr12	7945766	7945840	chr12:7945545-7945808
chr12	7947091	7947164	chr12:7947052-7947139
chr12	7947241	7947878	chr12:7947274-7948657
chr12	7948206	7948689	chr12:7947274-7948657
chr12	40534633	40535568	chr12:40534663-40536678
chr12	40535578	40536669	chr12:40534663-40536678
chr12	40537813	40538129	chr12:40537836-40538106
chr12	40539238	40539354	chr12:40539275-40539298
chr12	40545803	40545977	chr12:40545827-40545948
chr12	40549868	40550185	chr12:40549878-40550145
chr12	40551633	40551864	chr12:40551655-40551863
chr12	40552203	40552415	chr12:40552237-40552379
chr12	40561023	40561629	chr12:40561049-40561611
chr12	40579778	40580015	chr12:40579810-40579985
chr12	40582948	40583017	chr12:40582884-40583007
chr12	40584033	40584231	chr12:40584054-40584196

chr12	40588023	40588131	chr12:40588056-40588098
chr12	40590513	40590616	chr12:40590545-40590575
chr12	40617428	40617824	chr12:40617460-40617821
chr12	40618778	40619099	chr12:40618812-40619084
chr12	40619333	40619475	chr12:40619356-40619442
chr12	40626053	40626222	chr12:40626075-40626185
chr12	40629403	40629537	chr12:40629427-40629516
chr12	40631738	40631922	chr12:40631770-40631905
chr12	40634258	40634441	chr12:40634284-40634419
chr12	40637328	40637499	chr12:40637351-40637483
chr12	40643603	40643778	chr12:40643627-40643747
chr12	40645008	40645213	chr12:40645033-40645176
chr12	40645243	40645377	chr12:40645266-40645346
chr12	40646688	40646858	chr12:40646711-40646818
chr12	40651028	40651198	chr12:40651049-40651179
chr12	40653258	40653436	chr12:40653281-40653406
chr12	40657568	40657738	chr12:40657590-40657703
chr12	40668358	40668573	chr12:40668384-40668529
chr12	40668648	40668819	chr12:40668655-40668795
chr12	40671668	40671849	chr12:40671689-40671818
chr12	40671858	40672079	chr12:40671892-40672063
chr12	40677668	40677934	chr12:40677676-40677935
chr12	40681128	40681364	chr12:40681152-40681341
chr12	40687313	40687499	chr12:40687346-40687465
chr12	40688623	40688724	chr12:40688646-40688716
chr12	40689213	40689462	chr12:40689228-40689446
chr12	40692023	40692329	chr12:40692044-40692295
chr12	40692883	40693091	chr12:40692910-40693059
chr12	40694583	40694903	chr12:40694614-40694866
chr12	40696583	40696708	chr12:40696590-40696684
chr12	40697728	40698805	chr12:40697749-40698778
chr12	40699563	40699801	chr12:40699586-40699768
chr12	40702243	40702516	chr12:40702268-40702498
chr12	40702883	40703059	chr12:40702907-40703035
chr12	40704203	40704476	chr12:40704232-40704451
chr12	40706488	40706739	chr12:40706515-40706710
chr12	40707748	40707993	chr12:40707773-40707975
chr12	40708983	40709118	chr12:40709013-40709102
chr12	40713763	40714001	chr12:40713789-40713977
chr12	40714808	40715018	chr12:40714835-40714990
chr12	40715813	40716010	chr12:40715836-40715983
chr12	40716088	40716346	chr12:40716120-40716312
chr12	40716938	40717138	chr12:40716961-40717108
chr12	40722138	40722274	chr12:40722161-40722262
chr12	40728738	40728984	chr12:40728768-40728959
chr12	40734063	40734293	chr12:40734095-40734256
chr12	40740533	40740735	chr12:40740554-40740725
chr12	40742178	40742317	chr12:40742210-40742311
chr12	40745318	40745559	chr12:40745340-40745535
chr12	40748073	40748320	chr12:40748100-40748294
chr12	40749893	40750025	chr12:40749916-40749989
chr12	40753053	40753256	chr12:40753061-40753246
chr12	40757193	40757359	chr12:40757203-40757356
chr12	40758618	40758859	chr12:40758643-40758852
chr12	40760783	40760909	chr12:40760807-40760879
chr12	40761433	40761620	chr12:40761445-40763087
chr12	40761628	40761705	chr12:40761445-40763087
chr12	40761713	40762497	chr12:40761445-40763087
chr12	40762498	40763111	chr12:40761445-40763087
chr12	56865142	56865470	chr12:56865177-56865427
chr12	56865532	56865632	chr12:56865556-56865620
chr12	56865877	56866018	chr12:56865902-56865979
chr12	56866452	56866567	chr12:56866473-56866535

chr12	56866977	56867124	chr12:56867011-56867104
chr12	56867212	56867396	chr12:56867246-56867378
chr12	56868292	56868549	chr12:56868327-56868504
chr12	56868597	56868710	chr12:56868629-56868680
chr12	56868797	56868972	chr12:56868827-56868967
chr12	56869372	56869484	chr12:56869407-56869466
chr12	56869692	56869776	chr12:56869728-56869761
chr12	56871412	56871521	chr12:56871443-56871502
chr12	56871682	56871912	chr12:56871716-56871880
chr12	56871942	56872078	chr12:56871966-56872046
chr12	56872812	56872991	chr12:56872835-56872965
chr12	56873542	56873724	chr12:56873563-56873685
chr12	56874037	56874188	chr12:56874071-56874171
chr12	56881697	56882051	chr12:56881720-56882198
chr12	56882062	56882239	chr12:56881720-56882198
chr12	68726479	68727006	chr12:68726511-68727444
chr12	68727024	68727488	chr12:68726511-68727444
chr12	68729399	68729470	chr12:68729434-68729561
chr12	68729474	68729580	chr12:68729434-68729561
chr12	68738419	68738695	chr12:68738443-68738683
chr12	68742254	68742562	chr12:68742280-68742533
chr12	68743479	68743712	chr12:68743503-68743739
chr12	68771429	68777141	chr12:68771452-68786416
chr12	68777259	68777713	chr12:68771452-68786416
chr12	68778004	68780706	chr12:68771452-68786416
chr12	68780714	68780815	chr12:68771452-68786416
chr12	68781214	68781729	chr12:68771452-68786416
chr12	68781754	68782601	chr12:68771452-68786416
chr12	68782984	68783228	chr12:68771452-68786416
chr12	68783234	68784121	chr12:68771452-68786416
chr12	68784469	68784552	chr12:68771452-68786416
chr12	68784574	68785264	chr12:68771452-68786416
chr12	68785329	68786448	chr12:68771452-68786416
chr12	68797479	68797913	chr12:68797512-68797881
chr12	68799629	68800643	chr12:68799657-68800605
chr12	68818294	68818402	chr12:68818318-68818375
chr12	68825594	68825921	chr12:68825625-68825911
chr12	68826259	68826481	chr12:68826294-68826459
chr12	68827034	68827177	chr12:68827060-68827151
chr12	68827559	68827809	chr12:68827588-68827787
chr12	68828709	68828965	chr12:68828742-68828924
chr12	68835644	68835742	chr12:68835667-68835996
chr12	68835859	68835932	chr12:68835667-68835996
chr12	68845124	68845540	chr12:68845146-68845510
chr12	84510537	84510896	chr12:84510561-84601640
chr12	84510942	84511776	chr12:84510561-84601640
chr12	84511782	84512314	chr12:84510561-84601640
chr12	84512317	84513057	chr12:84510561-84601640
chr12	84513067	84514090	chr12:84510561-84601640
chr12	84514097	84515627	chr12:84510561-84601640
chr12	84516187	84516784	chr12:84510561-84601640
chr12	84516797	84517911	chr12:84510561-84601640
chr12	84517942	84518459	chr12:84510561-84601640
chr12	84518747	84519873	chr12:84510561-84601640
chr12	84519877	84520219	chr12:84510561-84601640
chr12	84520477	84522726	chr12:84510561-84601640
chr12	84522727	84524746	chr12:84510561-84601640
chr12	84524747	84525838	chr12:84510561-84601640
chr12	84526387	84528134	chr12:84510561-84601640
chr12	84528207	84529191	chr12:84510561-84601640
chr12	84529247	84529379	chr12:84510561-84601640
chr12	84529382	84530431	chr12:84510561-84601640
chr12	84530432	84530927	chr12:84510561-84601640

chr12	84530932	84531793	chr12:84510561-84601640
chr12	84531802	84532129	chr12:84510561-84601640
chr12	84532277	84534586	chr12:84510561-84601640
chr12	84534607	84534923	chr12:84510561-84601640
chr12	84534937	84535334	chr12:84510561-84601640
chr12	84535337	84537113	chr12:84510561-84601640
chr12	84537117	84537950	chr12:84510561-84601640
chr12	84538012	84538431	chr12:84510561-84601640
chr12	84538432	84538738	chr12:84510561-84601640
chr12	84538802	84538867	chr12:84510561-84601640
chr12	84538872	84539146	chr12:84510561-84601640
chr12	84539147	84539348	chr12:84510561-84601640
chr12	84539362	84539474	chr12:84510561-84601640
chr12	84539532	84539887	chr12:84510561-84601640
chr12	84540482	84540565	chr12:84510561-84601640
chr12	84540567	84540803	chr12:84510561-84601640
chr12	84540807	84540896	chr12:84510561-84601640
chr12	84540902	84541159	chr12:84510561-84601640
chr12	84541167	84541253	chr12:84510561-84601640
chr12	84541262	84541387	chr12:84510561-84601640
chr12	84541407	84541504	chr12:84510561-84601640
chr12	84541682	84541752	chr12:84510561-84601640
chr12	84541757	84541836	chr12:84510561-84601640
chr12	84541892	84541969	chr12:84510561-84601640
chr12	84541977	84542102	chr12:84510561-84601640
chr12	84542132	84542530	chr12:84510561-84601640
chr12	84542602	84543038	chr12:84510561-84601640
chr12	84543137	84543283	chr12:84510561-84601640
chr12	84543322	84543393	chr12:84510561-84601640
chr12	84543697	84543922	chr12:84510561-84601640
chr12	84544322	84545020	chr12:84510561-84601640
chr12	84545047	84545126	chr12:84510561-84601640
chr12	84545127	84545211	chr12:84510561-84601640
chr12	84545377	84545523	chr12:84510561-84601640
chr12	84545547	84545681	chr12:84510561-84601640
chr12	84545682	84545792	chr12:84510561-84601640
chr12	84545802	84545871	chr12:84510561-84601640
chr12	84545912	84546015	chr12:84510561-84601640
chr12	84546137	84546214	chr12:84510561-84601640
chr12	84546327	84546487	chr12:84510561-84601640
chr12	84546812	84546890	chr12:84510561-84601640
chr12	84546957	84547111	chr12:84510561-84601640
chr12	84547192	84547271	chr12:84510561-84601640
chr12	84547367	84547486	chr12:84510561-84601640
chr12	84547487	84547555	chr12:84510561-84601640
chr12	84547812	84547987	chr12:84510561-84601640
chr12	84548002	84548192	chr12:84510561-84601640
chr12	84549452	84550233	chr12:84510561-84601640
chr12	84550237	84550852	chr12:84510561-84601640
chr12	84551037	84551431	chr12:84510561-84601640
chr12	84551512	84551883	chr12:84510561-84601640
chr12	84551907	84552228	chr12:84510561-84601640
chr12	84552232	84553596	chr12:84510561-84601640
chr12	84553607	84554113	chr12:84510561-84601640
chr12	84554432	84555827	chr12:84510561-84601640
chr12	84555972	84556265	chr12:84510561-84601640
chr12	84556312	84556729	chr12:84510561-84601640
chr12	84556787	84558525	chr12:84510561-84601640
chr12	84558907	84560483	chr12:84510561-84601640
chr12	84560487	84560678	chr12:84510561-84601640
chr12	84560712	84561069	chr12:84510561-84601640
chr12	84561077	84561327	chr12:84510561-84601640
chr12	84561337	84561744	chr12:84510561-84601640

chr12	84561747	84568358	chr12:84510561-84601640
chr12	84568362	84569295	chr12:84510561-84601640
chr12	84569302	84569495	chr12:84510561-84601640
chr12	84569507	84571116	chr12:84510561-84601640
chr12	84571117	84571441	chr12:84510561-84601640
chr12	84571457	84571810	chr12:84510561-84601640
chr12	84571817	84572279	chr12:84510561-84601640
chr12	84572287	84574738	chr12:84510561-84601640
chr12	84574742	84574964	chr12:84510561-84601640
chr12	84575012	84575461	chr12:84510561-84601640
chr12	84575622	84575815	chr12:84510561-84601640
chr12	84575817	84578983	chr12:84510561-84601640
chr12	84579027	84579489	chr12:84510561-84601640
chr12	84579497	84579783	chr12:84510561-84601640
chr12	84579867	84580993	chr12:84510561-84601640
chr12	84581002	84581260	chr12:84510561-84601640
chr12	84581467	84581610	chr12:84510561-84601640
chr12	84581652	84581924	chr12:84510561-84601640
chr12	84581967	84582490	chr12:84510561-84601640
chr12	84582492	84582913	chr12:84510561-84601640
chr12	84583102	84583212	chr12:84510561-84601640
chr12	84583507	84583583	chr12:84510561-84601640
chr12	84583622	84583794	chr12:84510561-84601640
chr12	84583807	84583945	chr12:84510561-84601640
chr12	84584077	84584311	chr12:84510561-84601640
chr12	84584392	84584467	chr12:84510561-84601640
chr12	84584477	84584705	chr12:84510561-84601640
chr12	84584737	84584919	chr12:84510561-84601640
chr12	84585017	84585269	chr12:84510561-84601640
chr12	84585377	84585491	chr12:84510561-84601640
chr12	84585572	84585697	chr12:84510561-84601640
chr12	84585702	84587693	chr12:84510561-84601640
chr12	84588292	84588367	chr12:84510561-84601640
chr12	84588567	84589677	chr12:84510561-84601640
chr12	84589992	84590094	chr12:84510561-84601640
chr12	84590662	84590737	chr12:84510561-84601640
chr12	84592142	84593331	chr12:84510561-84601640
chr12	84593332	84593729	chr12:84510561-84601640
chr12	84593732	84595728	chr12:84510561-84601640
chr12	84596057	84596319	chr12:84510561-84601640
chr12	84596322	84596523	chr12:84510561-84601640
chr12	84596617	84597901	chr12:84510561-84601640
chr12	84597932	84598466	chr12:84510561-84601640
chr12	84598477	84598989	chr12:84510561-84601640
chr12	84599537	84599664	chr12:84510561-84601640
chr12	84599732	84599849	chr12:84510561-84601640
chr12	84600092	84600327	chr12:84510561-84601640
chr12	84600362	84600444	chr12:84510561-84601640
chr12	84600462	84601433	chr12:84510561-84601640
chr12	84601437	84601671	chr12:84510561-84601640
chr12	84601757	84602492	chr12:84601782-84683509
chr12	84602502	84603767	chr12:84601782-84683509
chr12	84604107	84604229	chr12:84601782-84683509
chr12	84604232	84604298	chr12:84601782-84683509
chr12	84604302	84605238	chr12:84601782-84683509
chr12	84605242	84606298	chr12:84601782-84683509
chr12	84606352	84606575	chr12:84601782-84683509
chr12	84606577	84607705	chr12:84601782-84683509
chr12	84607707	84609183	chr12:84601782-84683509
chr12	84609192	84610699	chr12:84601782-84683509
chr12	84610832	84611550	chr12:84601782-84683509
chr12	84611607	84611701	chr12:84601782-84683509
chr12	84611707	84611779	chr12:84601782-84683509

chr12	84611822	84612601	chr12:84601782-84683509
chr12	84612632	84613101	chr12:84601782-84683509
chr12	84613202	84613275	chr12:84601782-84683509
chr12	84613387	84614515	chr12:84601782-84683509
chr12	84614527	84614723	chr12:84601782-84683509
chr12	84614757	84614922	chr12:84601782-84683509
chr12	84615207	84615289	chr12:84601782-84683509
chr12	84615667	84616346	chr12:84601782-84683509
chr12	84616362	84617533	chr12:84601782-84683509
chr12	84617562	84619671	chr12:84601782-84683509
chr12	84619672	84621490	chr12:84601782-84683509
chr12	84621537	84621775	chr12:84601782-84683509
chr12	84622062	84622288	chr12:84601782-84683509
chr12	84622437	84622588	chr12:84601782-84683509
chr12	84622592	84622862	chr12:84601782-84683509
chr12	84622882	84623062	chr12:84601782-84683509
chr12	84623147	84623742	chr12:84601782-84683509
chr12	84623767	84625592	chr12:84601782-84683509
chr12	84625597	84626062	chr12:84601782-84683509
chr12	84626067	84626221	chr12:84601782-84683509
chr12	84626222	84626574	chr12:84601782-84683509
chr12	84626602	84626718	chr12:84601782-84683509
chr12	84627022	84627282	chr12:84601782-84683509
chr12	84627297	84627874	chr12:84601782-84683509
chr12	84627887	84628211	chr12:84601782-84683509
chr12	84628287	84628370	chr12:84601782-84683509
chr12	84628372	84628473	chr12:84601782-84683509
chr12	84628502	84628922	chr12:84601782-84683509
chr12	84628972	84629109	chr12:84601782-84683509
chr12	84629562	84629670	chr12:84601782-84683509
chr12	84629677	84630195	chr12:84601782-84683509
chr12	84630197	84630446	chr12:84601782-84683509
chr12	84630457	84630676	chr12:84601782-84683509
chr12	84630682	84631495	chr12:84601782-84683509
chr12	84631502	84632017	chr12:84601782-84683509
chr12	84632022	84632580	chr12:84601782-84683509
chr12	84632592	84633216	chr12:84601782-84683509
chr12	84633262	84635779	chr12:84601782-84683509
chr12	84636067	84637097	chr12:84601782-84683509
chr12	84637102	84637426	chr12:84601782-84683509
chr12	84637707	84638162	chr12:84601782-84683509
chr12	84638182	84638525	chr12:84601782-84683509
chr12	84638537	84639708	chr12:84601782-84683509
chr12	84639712	84639930	chr12:84601782-84683509
chr12	84640297	84640394	chr12:84601782-84683509
chr12	84640917	84641948	chr12:84601782-84683509
chr12	84641952	84642048	chr12:84601782-84683509
chr12	84642052	84643647	chr12:84601782-84683509
chr12	84643657	84644419	chr12:84601782-84683509
chr12	84644437	84646206	chr12:84601782-84683509
chr12	84646217	84647482	chr12:84601782-84683509
chr12	84647492	84647830	chr12:84601782-84683509
chr12	84648257	84648827	chr12:84601782-84683509
chr12	84648832	84648908	chr12:84601782-84683509
chr12	84648912	84650221	chr12:84601782-84683509
chr12	84650222	84650549	chr12:84601782-84683509
chr12	84650557	84650767	chr12:84601782-84683509
chr12	84650887	84650991	chr12:84601782-84683509
chr12	84651022	84651107	chr12:84601782-84683509
chr12	84651122	84651294	chr12:84601782-84683509
chr12	84651387	84651739	chr12:84601782-84683509
chr12	84651742	84651995	chr12:84601782-84683509
chr12	84652037	84652168	chr12:84601782-84683509

chr12	84652177	84652253	chr12:84601782-84683509
chr12	84652262	84652794	chr12:84601782-84683509
chr12	84652817	84653015	chr12:84601782-84683509
chr12	84653022	84653514	chr12:84601782-84683509
chr12	84653527	84654146	chr12:84601782-84683509
chr12	84654227	84654673	chr12:84601782-84683509
chr12	84654677	84655349	chr12:84601782-84683509
chr12	84655392	84655461	chr12:84601782-84683509
chr12	84655467	84656581	chr12:84601782-84683509
chr12	84656582	84656875	chr12:84601782-84683509
chr12	84656877	84657826	chr12:84601782-84683509
chr12	84657832	84658327	chr12:84601782-84683509
chr12	84658622	84665723	chr12:84601782-84683509
chr12	84665972	84667354	chr12:84601782-84683509
chr12	84668167	84668836	chr12:84601782-84683509
chr12	84668877	84671512	chr12:84601782-84683509
chr12	84671882	84671967	chr12:84601782-84683509
chr12	84672077	84672148	chr12:84601782-84683509
chr12	84672167	84672381	chr12:84601782-84683509
chr12	84672407	84672661	chr12:84601782-84683509
chr12	84672727	84672848	chr12:84601782-84683509
chr12	84672862	84672959	chr12:84601782-84683509
chr12	84673007	84673206	chr12:84601782-84683509
chr12	84673262	84673340	chr12:84601782-84683509
chr12	84673427	84673496	chr12:84601782-84683509
chr12	84673852	84675116	chr12:84601782-84683509
chr12	84675117	84675299	chr12:84601782-84683509
chr12	84675577	84676047	chr12:84601782-84683509
chr12	84676182	84676284	chr12:84601782-84683509
chr12	84676287	84677532	chr12:84601782-84683509
chr12	84677572	84679632	chr12:84601782-84683509
chr12	84679707	84681663	chr12:84601782-84683509
chr12	84681702	84681995	chr12:84601782-84683509
chr12	84682032	84682930	chr12:84601782-84683509
chr12	84682937	84683145	chr12:84601782-84683509
chr12	84683187	84683534	chr12:84601782-84683509
chr12	84683612	84684433	chr12:84683646-84698234
chr12	84684442	84684970	chr12:84683646-84698234
chr12	84685037	84686687	chr12:84683646-84698234
chr12	84686712	84687030	chr12:84683646-84698234
chr12	84687317	84689698	chr12:84683646-84698234
chr12	84689762	84689963	chr12:84683646-84698234
chr12	84689982	84690202	chr12:84683646-84698234
chr12	84690232	84690304	chr12:84683646-84698234
chr12	84690722	84690814	chr12:84683646-84698234
chr12	84690832	84690984	chr12:84683646-84698234
chr12	84690992	84691236	chr12:84683646-84698234
chr12	84691552	84691920	chr12:84683646-84698234
chr12	84692217	84692745	chr12:84683646-84698234
chr12	84693077	84693899	chr12:84683646-84698234
chr12	84694412	84694933	chr12:84683646-84698234
chr12	84694997	84697885	chr12:84683646-84698234
chr12	84697892	84698279	chr12:84683646-84698234
chr12	90111021	90111162	chr12:90111042-90111140
chr12	90209676	90209774	chr12:90209717-90209729
chr12	90210776	90210917	chr12:90210804-90210877
chr12	90276416	90276698	chr12:90276441-90276668
chr12	90295021	90295750	chr12:90295044-90407660
chr12	90295766	90296927	chr12:90295044-90407660
chr12	90297216	90298202	chr12:90295044-90407660
chr12	90298346	90304418	chr12:90295044-90407660
chr12	90304426	90305695	chr12:90295044-90407660
chr12	90305696	90305783	chr12:90295044-90407660

chr12	90305811	90306253	chr12:90295044-90407660
chr12	90306261	90306404	chr12:90295044-90407660
chr12	90306426	90306508	chr12:90295044-90407660
chr12	90307386	90307602	chr12:90295044-90407660
chr12	90307886	90309297	chr12:90295044-90407660
chr12	90309301	90310014	chr12:90295044-90407660
chr12	90310211	90310674	chr12:90295044-90407660
chr12	90310681	90310782	chr12:90295044-90407660
chr12	90310886	90311067	chr12:90295044-90407660
chr12	90311076	90311310	chr12:90295044-90407660
chr12	90311316	90316904	chr12:90295044-90407660
chr12	90316921	90319528	chr12:90295044-90407660
chr12	90319806	90320496	chr12:90295044-90407660
chr12	90320791	90321564	chr12:90295044-90407660
chr12	90321566	90322918	chr12:90295044-90407660
chr12	90322971	90323187	chr12:90295044-90407660
chr12	90323491	90323678	chr12:90295044-90407660
chr12	90323701	90326221	chr12:90295044-90407660
chr12	90326271	90327646	chr12:90295044-90407660
chr12	90327651	90328185	chr12:90295044-90407660
chr12	90328186	90328954	chr12:90295044-90407660
chr12	90328956	90330466	chr12:90295044-90407660
chr12	90330471	90330759	chr12:90295044-90407660
chr12	90330781	90331962	chr12:90295044-90407660
chr12	90331971	90332409	chr12:90295044-90407660
chr12	90332571	90332826	chr12:90295044-90407660
chr12	90332831	90334224	chr12:90295044-90407660
chr12	90334526	90336002	chr12:90295044-90407660
chr12	90336021	90336347	chr12:90295044-90407660
chr12	90336371	90337019	chr12:90295044-90407660
chr12	90337031	90337478	chr12:90295044-90407660
chr12	90337481	90338532	chr12:90295044-90407660
chr12	90338541	90341921	chr12:90295044-90407660
chr12	90341931	90343040	chr12:90295044-90407660
chr12	90343046	90343233	chr12:90295044-90407660
chr12	90343246	90344803	chr12:90295044-90407660
chr12	90344866	90345468	chr12:90295044-90407660
chr12	90345476	90346067	chr12:90295044-90407660
chr12	90346361	90346799	chr12:90295044-90407660
chr12	90346801	90347144	chr12:90295044-90407660
chr12	90347146	90348745	chr12:90295044-90407660
chr12	90348751	90348929	chr12:90295044-90407660
chr12	90348946	90350732	chr12:90295044-90407660
chr12	90351091	90351341	chr12:90295044-90407660
chr12	90351351	90351701	chr12:90295044-90407660
chr12	90351726	90352335	chr12:90295044-90407660
chr12	90352336	90352870	chr12:90295044-90407660
chr12	90352891	90353356	chr12:90295044-90407660
chr12	90353391	90354943	chr12:90295044-90407660
chr12	90355001	90355437	chr12:90295044-90407660
chr12	90355441	90356182	chr12:90295044-90407660
chr12	90356191	90357055	chr12:90295044-90407660
chr12	90357061	90357882	chr12:90295044-90407660
chr12	90357891	90358400	chr12:90295044-90407660
chr12	90358711	90359205	chr12:90295044-90407660
chr12	90359216	90361076	chr12:90295044-90407660
chr12	90361086	90361272	chr12:90295044-90407660
chr12	90361651	90365503	chr12:90295044-90407660
chr12	90365551	90366192	chr12:90295044-90407660
chr12	90366506	90366675	chr12:90295044-90407660
chr12	90367006	90367259	chr12:90295044-90407660
chr12	90367281	90367416	chr12:90295044-90407660
chr12	90367461	90367752	chr12:90295044-90407660

chr12	90367766	90367835	chr12:90295044-90407660
chr12	90367866	90367953	chr12:90295044-90407660
chr12	90368041	90368135	chr12:90295044-90407660
chr12	90368161	90368642	chr12:90295044-90407660
chr12	90368646	90368724	chr12:90295044-90407660
chr12	90368761	90369730	chr12:90295044-90407660
chr12	90369751	90369855	chr12:90295044-90407660
chr12	90369881	90373920	chr12:90295044-90407660
chr12	90373921	90374674	chr12:90295044-90407660
chr12	90374676	90374743	chr12:90295044-90407660
chr12	90374751	90376122	chr12:90295044-90407660
chr12	90376406	90377674	chr12:90295044-90407660
chr12	90377701	90378333	chr12:90295044-90407660
chr12	90378346	90378585	chr12:90295044-90407660
chr12	90378691	90379311	chr12:90295044-90407660
chr12	90379316	90380234	chr12:90295044-90407660
chr12	90380371	90381083	chr12:90295044-90407660
chr12	90381371	90384025	chr12:90295044-90407660
chr12	90384026	90386212	chr12:90295044-90407660
chr12	90386226	90386947	chr12:90295044-90407660
chr12	90386951	90388750	chr12:90295044-90407660
chr12	90388751	90390335	chr12:90295044-90407660
chr12	90390341	90390970	chr12:90295044-90407660
chr12	90390971	90393151	chr12:90295044-90407660
chr12	90393156	90393933	chr12:90295044-90407660
chr12	90393941	90395087	chr12:90295044-90407660
chr12	90395091	90396813	chr12:90295044-90407660
chr12	90396816	90396980	chr12:90295044-90407660
chr12	90396981	90398553	chr12:90295044-90407660
chr12	90398616	90398843	chr12:90295044-90407660
chr12	90398871	90399642	chr12:90295044-90407660
chr12	90399646	90399896	chr12:90295044-90407660
chr12	90399916	90401585	chr12:90295044-90407660
chr12	90401596	90403287	chr12:90295044-90407660
chr12	90403291	90404159	chr12:90295044-90407660
chr12	90404221	90404553	chr12:90295044-90407660
chr12	90404836	90405090	chr12:90295044-90407660
chr12	90405096	90406349	chr12:90295044-90407660
chr12	90406351	90407707	chr12:90295044-90407660
chr12	90415266	90415374	chr12:90415296-90415334
chr12	90460751	90460969	chr12:90460772-90460937
chr12	90462541	90462652	chr12:90462565-90462619
chr12	90488186	90488283	chr12:90488212-90488256
chr12	90494446	90494637	chr12:90494477-90494618
chr12	90501496	90501635	chr12:90501503-90501598
chr12	90505686	90506592	chr12:90505713-90506556
chr12	92087900	92087999	chr12:92087864-92087962
chr12	92125830	92126052	chr12:92125853-92126013
chr12	92208090	92208862	chr12:92208114-92208844
chr12	92209345	92209565	chr12:92209375-92209532
chr12	92212230	92212475	chr12:92212255-92212449
chr12	92214315	92214428	chr12:92214339-92214439
chr12	92228820	92228962	chr12:92228853-92228931
chr12	92231805	92232038	chr12:92231831-92231997
chr12	92241350	92242932	chr12:92241372-92261220
chr12	92242935	92243102	chr12:92241372-92261220
chr12	92243105	92243285	chr12:92241372-92261220
chr12	92243290	92243805	chr12:92241372-92261220
chr12	92244100	92244340	chr12:92241372-92261220
chr12	92244385	92244758	chr12:92241372-92261220
chr12	92244790	92244904	chr12:92241372-92261220
chr12	92244910	92245048	chr12:92241372-92261220
chr12	92245080	92245160	chr12:92241372-92261220

chr12	92245225	92245568	chr12:92241372-92261220
chr12	92245570	92245704	chr12:92241372-92261220
chr12	92245765	92246044	chr12:92241372-92261220
chr12	92246050	92246314	chr12:92241372-92261220
chr12	92246330	92246403	chr12:92241372-92261220
chr12	92246460	92246821	chr12:92241372-92261220
chr12	92246830	92249542	chr12:92241372-92261220
chr12	92249565	92251158	chr12:92241372-92261220
chr12	92251165	92252786	chr12:92241372-92261220
chr12	92253070	92254329	chr12:92241372-92261220
chr12	92254390	92255183	chr12:92241372-92261220
chr12	92255190	92256374	chr12:92241372-92261220
chr12	92256420	92258599	chr12:92241372-92261220
chr12	92258885	92259509	chr12:92241372-92261220
chr12	92259620	92261253	chr12:92241372-92261220
chr12	92262295	92262478	chr12:92262316-92262447
chr12	92266435	92266523	chr12:92266459-92266526
chr12	92270090	92271644	chr12:92270014-92272331
chr12	92271645	92272334	chr12:92270014-92272331
chr12	92273320	92275595	chr12:92273342-92275582
chr12	92276830	92276934	chr12:92276873-92276886
chr12	92278485	92278668	chr12:92278520-92278639
chr12	92281950	92282082	chr12:92281971-92282043
chr12	92284995	92285165	chr12:92284972-92285155
chr12	92297250	92297360	chr12:92297244-92297329
chr12	92299050	92299308	chr12:92299071-92300005
chr12	92299310	92300039	chr12:92299071-92300005
chr12	109249836	109249990	chr12:109249857-109249963
chr12	109252676	109252825	chr12:109252707-109252811
chr12	109270436	109270711	chr12:109270445-109270689
chr12	109273306	109274023	chr12:109273338-109274000
chr12	109274101	109274202	chr12:109274126-109274167
chr12	109277381	109277563	chr12:109277411-109277547
chr12	109278741	109278999	chr12:109278773-109278976
chr12	109281191	109281372	chr12:109281225-109281340
chr12	109283216	109283354	chr12:109283244-109283321
chr12	109283956	109284061	chr12:109283983-109284049
chr12	109286736	109286841	chr12:109286757-109286812
chr12	109286926	109287238	chr12:109286948-109287197
chr12	109287386	109287700	chr12:109287418-109287667
chr12	109288016	109288161	chr12:109288038-109288143
chr12	109290751	109290900	chr12:109290781-109290864
chr12	109292221	109292606	chr12:109292247-109292572
chr12	109293121	109294842	chr12:109293152-109294819
chr12	123258848	123259267	chr12:123258873-123259253
chr12	123261073	123261365	chr12:123261104-123262230
chr12	123261633	123262271	chr12:123261104-123262230
chr12	123265678	123265891	chr12:123265710-123265877
chr12	123270243	123270378	chr12:123270265-123270367
chr12	123273283	123273489	chr12:123273304-123273476
chr12	123276543	123276681	chr12:123276566-123276668
chr12	123281828	123281970	chr12:123281853-123281942
chr12	123282603	123282784	chr12:123282631-123282747
chr12	123285648	123286423	chr12:123285670-123286399
chr12	123290673	123290880	chr12:123290697-123290842
chr12	123297788	123298013	chr12:123297816-123297966
chr12	123307878	123308032	chr12:123307912-123308006
chr12	123310943	123311337	chr12:123310968-123312075
chr12	123311343	123312110	chr12:123310968-123312075
chr12	123319228	123319580	chr12:123319261-123319553
chr12	123319963	123320207	chr12:123319984-123320190
chr12	123332548	123332661	chr12:123332577-123332641
chr12	123332988	123333196	chr12:123333012-123333155

chr12	123333318	123333426	chr12:123333342-123333399
chr12	123334398	123334534	chr12:123334421-123334502
chr12	123335358	123335499	chr12:123335381-123335458
chr12	123335758	123335866	chr12:123335782-123335844
chr12	123338568	123338762	chr12:123338589-123338730
chr12	123339418	123339537	chr12:123339451-123339509
chr12	123339578	123339706	chr12:123339599-123339675
chr12	123339783	123339978	chr12:123339811-123339952
chr12	123340073	123340172	chr12:123340097-123340159
chr12	123340343	123340487	chr12:123340377-123340450
chr12	123340503	123340747	chr12:123340526-123340710
chr12	123340773	123340927	chr12:123340802-123340891
chr12	123340948	123341123	chr12:123340978-123341095
chr12	123341148	123341301	chr12:123341171-123341273
chr12	123341533	123342215	chr12:123341567-123342447
chr12	123342308	123342426	chr12:123341567-123342447
chr12	123342613	123342828	chr12:123342648-123342796
chr12	123342873	123343008	chr12:123342895-123342982
chr12	123343358	123343514	chr12:123343384-123343492
chr12	123343583	123343770	chr12:123343607-123343744
chr12	123343938	123344125	chr12:123343972-123344083
chr12	123344283	123344395	chr12:123344318-123344377
chr12	123344558	123344784	chr12:123344593-123344624;chr12:123344694-123344757
chr12	123344948	123345088	chr12:123344969-123345070
chr12	123345203	123345364	chr12:123345225-123345331
chr12	123345438	123345616	chr12:123345462-123345586
chr12	123345643	123345719	chr12:123345677-123345739
chr12	123345823	123346067	chr12:123345854-123346061
chr12	123346228	123347522	chr12:123346252-123347507
chr13	79360501	79360945	chr13:79360530-79360932
chr13	79361416	79361636	chr13:79361440-79361614
chr13	79363616	79363734	chr13:79363651-79363719
chr13	79365001	79365193	chr13:79365033-79365155
chr13	79366261	79366480	chr13:79366295-79366442
chr13	79370086	79370254	chr13:79370109-79370234
chr13	79398516	79398583	chr13:79398550-79417762
chr13	79398606	79398706	chr13:79398550-79417762
chr13	79398821	79398910	chr13:79398550-79417762
chr13	79398996	79400484	chr13:79398550-79417762
chr13	79400486	79400988	chr13:79398550-79417762
chr13	79401021	79402274	chr13:79398550-79417762
chr13	79402286	79404068	chr13:79398550-79417762
chr13	79404126	79405348	chr13:79398550-79417762
chr13	79405591	79407244	chr13:79398550-79417762
chr13	79407251	79408938	chr13:79398550-79417762
chr13	79408946	79410493	chr13:79398550-79417762
chr13	79410511	79411976	chr13:79398550-79417762
chr13	79412026	79412198	chr13:79398550-79417762
chr13	79413061	79413347	chr13:79398550-79417762
chr13	79413351	79413753	chr13:79398550-79417762
chr13	79413811	79414062	chr13:79398550-79417762
chr13	79414081	79414203	chr13:79398550-79417762
chr13	79414226	79414380	chr13:79398550-79417762
chr13	79414686	79414885	chr13:79398550-79417762
chr13	79414891	79416549	chr13:79398550-79417762
chr13	79416551	79417000	chr13:79398550-79417762
chr13	79417001	79417119	chr13:79398550-79417762
chr13	79417151	79417419	chr13:79398550-79417762
chr13	79417456	79417798	chr13:79398550-79417762
chr14	22849002	22850194	chr14:22849025-22850808
chr14	22850202	22850843	chr14:22849025-22850808
chr14	22931872	22932261	chr14:22931899-22932227
chr14	22934207	22934303	chr14:22934235-22934278

chr14	22950207	22950380	chr14:22950229-22950347
chr14	22951817	22951988	chr14:22951841-22951948
chr14	22980397	22983346	chr14:22980408-23017301
chr14	22983652	22983794	chr14:22980408-23017301
chr14	22984077	22986023	chr14:22980408-23017301
chr14	22986062	22987365	chr14:22980408-23017301
chr14	22987372	22988188	chr14:22980408-23017301
chr14	22988457	22991853	chr14:22980408-23017301
chr14	22991907	22994911	chr14:22980408-23017301
chr14	22994912	22996779	chr14:22980408-23017301
chr14	22997057	22999701	chr14:22980408-23017301
chr14	22999737	23002231	chr14:22980408-23017301
chr14	23002232	23003521	chr14:22980408-23017301
chr14	23003687	23004624	chr14:22980408-23017301
chr14	23004627	23005684	chr14:22980408-23017301
chr14	23005692	23009866	chr14:22980408-23017301
chr14	23009947	23012600	chr14:22980408-23017301
chr14	23012887	23016369	chr14:22980408-23017301
chr14	23016412	23017320	chr14:22980408-23017301
chr14	23018547	23018655	chr14:23018581-23018626
chr14	23018852	23018965	chr14:23018901-23018916
chr14	23025117	23025444	chr14:23025151-23025416
chr14	55308692	55309842	chr14:55308723-55310861
chr14	55309852	55310882	chr14:55308723-55310861
chr14	55312452	55312613	chr14:55312485-55312570
chr14	55313782	55313875	chr14:55313816-55313848
chr14	55326372	55326485	chr14:55326398-55326454
chr14	55332017	55332199	chr14:55332044-55332154
chr14	55369007	55369569	chr14:55369038-55369542
chr14	56185214	56187434	chr14:56185061-56206340
chr14	56187439	56188654	chr14:56185061-56206340
chr14	56188689	56190306	chr14:56185061-56206340
chr14	56190309	56190571	chr14:56185061-56206340
chr14	56190574	56193356	chr14:56185061-56206340
chr14	56194839	56194943	chr14:56185061-56206340
chr14	56194944	56195042	chr14:56185061-56206340
chr14	56195044	56196959	chr14:56185061-56206340
chr14	56196964	56198236	chr14:56185061-56206340
chr14	56198254	56199094	chr14:56185061-56206340
chr14	56199164	56199290	chr14:56185061-56206340
chr14	56199549	56201855	chr14:56185061-56206340
chr14	56201964	56202218	chr14:56185061-56206340
chr14	56202249	56202995	chr14:56185061-56206340
chr14	56203014	56203088	chr14:56185061-56206340
chr14	56203299	56203964	chr14:56185061-56206340
chr14	56203974	56204719	chr14:56185061-56206340
chr14	56204889	56205161	chr14:56185061-56206340
chr14	56205434	56205978	chr14:56185061-56206340
chr14	56205999	56206353	chr14:56185061-56206340
chr14	56213899	56214015	chr14:56213934-56213978
chr14	56214844	56215031	chr14:56214871-56215000
chr14	56228454	56228690	chr14:56228500-56228519;chr14:56228604-56228654
chr14	56238154	56238449	chr14:56238185-56238423
chr14	56239389	56239601	chr14:56239420-56239567
chr14	56239684	56240043	chr14:56239706-56240007
chr14	56247829	56249136	chr14:56247853-56249712
chr14	56249154	56249747	chr14:56247853-56249712
chr14	56254799	56255126	chr14:56254644-56255094
chr14	56259979	56260192	chr14:56260014-56260078;chr14:56260085-56260174
chr14	56260969	56261096	chr14:56261001-56261063
chr14	85606668	85606864	chr14:85606692-85619189
chr14	85606978	85608694	chr14:85606692-85619189
chr14	85608993	85611252	chr14:85606692-85619189

chr14	85611258	85611794	chr14:85606692-85619189
chr14	85612073	85613065	chr14:85606692-85619189
chr14	85613098	85613182	chr14:85606692-85619189
chr14	85613183	85614785	chr14:85606692-85619189
chr14	85614788	85616002	chr14:85606692-85619189
chr14	85616003	85617705	chr14:85606692-85619189
chr14	85617713	85618175	chr14:85606692-85619189
chr14	85618483	85618755	chr14:85606692-85619189
chr14	85618888	85618988	chr14:85606692-85619189
chr14	85619038	85619157	chr14:85606692-85619189
chr14	99639956	99640144	chr14:99639874-99642532
chr14	99640146	99640210	chr14:99639874-99642532
chr14	99640211	99642574	chr14:99639874-99642532
chr14	99697656	99697929	chr14:99697681-99697894
chr14	99723781	99724202	chr14:99723807-99724176
chr14	99737476	99737660	chr14:99737497-99738050
chr14	99737666	99737732	chr14:99737497-99738050
chr14	99737796	99737858	chr14:99737497-99738050
chr14	99737861	99738071	chr14:99737497-99738050
chr15	26147473	26147743	chr15:26147506-26147713
chr15	26260468	26261202	chr15:26260480-26261168
chr15	26272678	26272841	chr15:26272711-26281253
chr15	26273218	26274296	chr15:26272711-26281253
chr15	26274303	26274513	chr15:26272711-26281253
chr15	26274753	26274906	chr15:26272711-26281253
chr15	26275313	26275556	chr15:26272711-26281253
chr15	26275748	26276038	chr15:26272711-26281253
chr15	26276173	26276543	chr15:26272711-26281253
chr15	26276823	26278446	chr15:26272711-26281253
chr15	26278473	26278612	chr15:26272711-26281253
chr15	26278733	26279204	chr15:26272711-26281253
chr15	26279478	26279689	chr15:26272711-26281253
chr15	26279948	26280590	chr15:26272711-26281253
chr15	26280593	26281193	chr15:26272711-26281253
chr15	26281218	26281295	chr15:26272711-26281253
chr15	26295653	26295974	chr15:26295684-26295941
chr15	26296378	26296523	chr15:26296413-26296491
chr15	26296778	26297442	chr15:26296803-26297423
chr15	26297478	26298284	chr15:26297513-26298267
chr15	40509633	40509879	chr15:40509628-40509868
chr15	40510668	40510786	chr15:40510700-40510763
chr15	40512743	40512942	chr15:40512764-40512918
chr15	40530928	40532330	chr15:40530959-40532003;chr15:40532070-40532296
chr15	40532448	40532559	chr15:40532490-40532512
chr15	40532633	40532767	chr15:40532663-40532882
chr15	40532778	40532891	chr15:40532663-40532882
chr15	40533673	40534245	chr15:40533706-40534205
chr15	40544368	40544514	chr15:40544390-40544464
chr15	40544718	40544920	chr15:40544748-40544892
chr15	40545053	40545520	chr15:40545088-40545490
chr15	40549013	40549294	chr15:40549043-40549287
chr15	40556608	40556751	chr15:40556643-40556729
chr15	40556768	40556879	chr15:40556803-40556848
chr15	40556958	40557197	chr15:40556981-40557190
chr15	40558008	40558728	chr15:40558042-40558696
chr15	40558778	40558871	chr15:40558812-40559198
chr15	40558888	40559238	chr15:40558812-40559198
chr15	40564393	40564959	chr15:40564424-40564922
chr15	40565038	40565228	chr15:40565066-40565200
chr15	40565513	40565699	chr15:40565546-40565673
chr15	40565728	40565895	chr15:40565751-40565877
chr15	40566108	40566529	chr15:40566130-40566496
chr15	40568093	40568953	chr15:40568127-40569688

chr15	40568983	40569718	chr15:40568127-40569688
chr15	53267341	53268076	chr15:53267306-53268046
chr15	53269881	53270312	chr15:53269895-53300631
chr15	53270316	53277566	chr15:53269895-53300631
chr15	53277581	53278094	chr15:53269895-53300631
chr15	53278101	53278685	chr15:53269895-53300631
chr15	53278706	53279652	chr15:53269895-53300631
chr15	53279666	53279843	chr15:53269895-53300631
chr15	53279851	53279937	chr15:53269895-53300631
chr15	53280036	53280207	chr15:53269895-53300631
chr15	53280241	53280320	chr15:53269895-53300631
chr15	53280966	53281083	chr15:53269895-53300631
chr15	53281226	53281416	chr15:53269895-53300631
chr15	53283116	53283195	chr15:53269895-53300631
chr15	53283641	53283724	chr15:53269895-53300631
chr15	53284741	53284813	chr15:53269895-53300631
chr15	53285141	53285278	chr15:53269895-53300631
chr15	53285681	53285762	chr15:53269895-53300631
chr15	53285826	53285924	chr15:53269895-53300631
chr15	53286346	53286423	chr15:53269895-53300631
chr15	53286636	53286752	chr15:53269895-53300631
chr15	53286786	53286986	chr15:53269895-53300631
chr15	53287006	53287482	chr15:53269895-53300631
chr15	53287491	53287960	chr15:53269895-53300631
chr15	53287961	53289807	chr15:53269895-53300631
chr15	53289856	53290103	chr15:53269895-53300631
chr15	53291501	53291685	chr15:53269895-53300631
chr15	53291846	53292001	chr15:53269895-53300631
chr15	53292016	53292464	chr15:53269895-53300631
chr15	53292466	53293523	chr15:53269895-53300631
chr15	53293531	53293798	chr15:53269895-53300631
chr15	53294146	53294444	chr15:53269895-53300631
chr15	53294446	53294538	chr15:53269895-53300631
chr15	53294546	53297523	chr15:53269895-53300631
chr15	53297551	53300341	chr15:53269895-53300631
chr15	53301861	53302240	chr15:53301882-53302208
chr15	61591983	61592271	chr15:61591932-61592738
chr15	61592308	61592424	chr15:61591932-61592738
chr15	61687238	61687347	chr15:61687265-61687306
chr15	61692558	61692641	chr15:61692580-61692630
chr15	61693888	61693995	chr15:61693936-61693958
chr15	61759558	61759661	chr15:61759564-61759629
chr15	61771273	61771416	chr15:61771298-61771378
chr15	61786133	61786232	chr15:61786154-61786205
chr15	61799348	61799462	chr15:61799376-61799439
chr15	61827828	61829894	chr15:61827850-61872197
chr15	61829903	61830127	chr15:61827850-61872197
chr15	61830168	61830256	chr15:61827850-61872197
chr15	61830273	61830609	chr15:61827850-61872197
chr15	61830638	61830717	chr15:61827850-61872197
chr15	61830753	61830892	chr15:61827850-61872197
chr15	61830948	61831320	chr15:61827850-61872197
chr15	61831328	61831410	chr15:61827850-61872197
chr15	61831558	61831690	chr15:61827850-61872197
chr15	61831723	61831834	chr15:61827850-61872197
chr15	61832098	61832374	chr15:61827850-61872197
chr15	61832658	61832743	chr15:61827850-61872197
chr15	61832803	61833025	chr15:61827850-61872197
chr15	61833118	61833192	chr15:61827850-61872197
chr15	61833408	61834358	chr15:61827850-61872197
chr15	61834408	61835213	chr15:61827850-61872197
chr15	61835403	61835490	chr15:61827850-61872197
chr15	61835623	61836057	chr15:61827850-61872197

chr15	61836058	61836381	chr15:61827850-61872197
chr15	61836388	61838690	chr15:61827850-61872197
chr15	61838963	61840225	chr15:61827850-61872197
chr15	61840473	61842583	chr15:61827850-61872197
chr15	61842588	61845772	chr15:61827850-61872197
chr15	61845783	61846302	chr15:61827850-61872197
chr15	61846303	61846386	chr15:61827850-61872197
chr15	61846403	61846745	chr15:61827850-61872197
chr15	61846748	61852059	chr15:61827850-61872197
chr15	61852168	61853417	chr15:61827850-61872197
chr15	61853423	61853803	chr15:61827850-61872197
chr15	61854473	61855401	chr15:61827850-61872197
chr15	61855418	61858124	chr15:61827850-61872197
chr15	61858138	61858227	chr15:61827850-61872197
chr15	61858423	61859701	chr15:61827850-61872197
chr15	61859753	61859948	chr15:61827850-61872197
chr15	61860053	61861249	chr15:61827850-61872197
chr15	61861258	61861431	chr15:61827850-61872197
chr15	61861433	61863922	chr15:61827850-61872197
chr15	61863993	61864111	chr15:61827850-61872197
chr15	61864533	61864941	chr15:61827850-61872197
chr15	61864943	61868315	chr15:61827850-61872197
chr15	61868333	61868607	chr15:61827850-61872197
chr15	61868648	61868750	chr15:61827850-61872197
chr15	61869313	61869745	chr15:61827850-61872197
chr15	61869753	61870189	chr15:61827850-61872197
chr15	61870203	61871450	chr15:61827850-61872197
chr15	61872088	61872232	chr15:61827850-61872197
chr15	61881598	61881744	chr15:61881622-61881706
chr15	61882088	61882232	chr15:61882114-61882202
chr15	61890703	61890843	chr15:61890710-61890800
chr15	61896568	61896835	chr15:61896603-61897450
chr15	61896838	61897462	chr15:61896603-61897450
chr15	61927583	61927720	chr15:61927613-61927689
chr15	61931513	61931951	chr15:61931547-61931911
chr15	61935708	61935960	chr15:61935729-61935917
chr15	61938813	61938906	chr15:61938837-61938890
chr15	61945353	61945644	chr15:61945387-61945624
chr15	61946048	61946299	chr15:61946078-61946259
chr15	61987968	61988495	chr15:61987998-62004796
chr15	61988508	61988618	chr15:61987998-62004796
chr15	61988783	61992186	chr15:61987998-62004796
chr15	61992193	61992377	chr15:61987998-62004796
chr15	61992503	61995890	chr15:61987998-62004796
chr15	61995893	61996127	chr15:61987998-62004796
chr15	61996133	61996713	chr15:61987998-62004796
chr15	61996783	61997334	chr15:61987998-62004796
chr15	61997623	61997870	chr15:61987998-62004796
chr15	61997883	61998015	chr15:61987998-62004796
chr15	61998088	61998518	chr15:61987998-62004796
chr15	61998528	62000354	chr15:61987998-62004796
chr15	62000558	62000656	chr15:61987998-62004796
chr15	62000658	62001182	chr15:61987998-62004796
chr15	62001238	62001319	chr15:61987998-62004796
chr15	62001398	62001499	chr15:61987998-62004796
chr15	62001583	62001803	chr15:61987998-62004796
chr15	62001813	62001888	chr15:61987998-62004796
chr15	62002163	62002367	chr15:61987998-62004796
chr15	62002388	62003422	chr15:61987998-62004796
chr15	62003433	62003737	chr15:61987998-62004796
chr15	62004143	62004216	chr15:61987998-62004796
chr15	62004243	62004841	chr15:61987998-62004796
chr15	62007288	62007430	chr15:62007313-62007418

chr15	62022013	62022234	chr15:62022048-62022206
chr15	62022748	62023470	chr15:62022780-62023589
chr15	62023483	62023613	chr15:62022780-62023589
chr15	62047723	62047845	chr15:62047756-62047825
chr15	62126908	62127022	chr15:62126950-62126976
chr15	62141563	62141711	chr15:62141595-62141671
chr15	62144033	62144383	chr15:62144054-62144351
chr15	62144558	62145968	chr15:62144589-62146757
chr15	62146263	62146789	chr15:62144589-62146757
chr15	62147048	62147180	chr15:62147069-62147153
chr15	62148453	62148629	chr15:62148484-62148608
chr15	62155613	62155745	chr15:62155638-62155727
chr15	62156478	62157722	chr15:62156504-62160146
chr15	62158008	62160170	chr15:62156504-62160146
chr15	62160833	62161011	chr15:62160857-62160972
chr15	62161673	62161852	chr15:62161698-62161822
chr15	62164163	62164267	chr15:62164187-62164233
chr15	62165413	62165630	chr15:62165444-62165608
chr15	62167053	62167166	chr15:62167074-62167150
chr15	62167903	62168052	chr15:62167930-62168044
chr15	62169148	62169285	chr15:62169171-62169253
chr15	62170778	62170956	chr15:62170805-62170945
chr15	62172783	62172951	chr15:62172807-62172921
chr15	62173008	62173162	chr15:62173041-62173153
chr15	62173738	62173841	chr15:62173761-62173831
chr15	62173913	62174059	chr15:62173945-62174027
chr15	62174768	62174955	chr15:62174794-62174935
chr15	62176303	62176502	chr15:62176326-62176468
chr15	62182328	62182617	chr15:62182363-62182599
chr15	62199438	62199614	chr15:62199462-62199589
chr15	62201168	62201336	chr15:62201190-62201324
chr15	62202353	62202519	chr15:62202375-62202504
chr15	62204013	62204215	chr15:62204038-62204203
chr15	62205488	62205626	chr15:62205509-62205614
chr15	62207798	62208227	chr15:62207831-62208221
chr15	62209518	62209874	chr15:62209539-62209834
chr15	62210313	62210479	chr15:62210334-62210456
chr15	62211458	62211677	chr15:62211487-62211648
chr15	62212243	62212551	chr15:62212265-62212530
chr15	62212663	62212873	chr15:62212696-62212846
chr15	62214123	62214266	chr15:62214145-62214232
chr15	62214563	62214987	chr15:62214595-62214961
chr15	62217623	62217765	chr15:62217654-62217747
chr15	62219268	62219547	chr15:62219289-62219519
chr15	62221673	62221972	chr15:62221699-62221947
chr15	62223263	62223471	chr15:62223288-62223458
chr15	62226393	62226561	chr15:62226417-62226530
chr15	62228773	62228986	chr15:62228795-62228949
chr15	62232823	62233024	chr15:62232845-62232993
chr15	62233938	62234283	chr15:62233961-62234266
chr15	62237903	62238103	chr15:62237913-62238081
chr15	62238483	62238627	chr15:62238505-62238609
chr15	62239358	62239545	chr15:62239391-62239508
chr15	62241618	62241831	chr15:62241641-62241804
chr15	62242523	62242639	chr15:62242556-62242616
chr15	62243118	62243257	chr15:62243143-62243223
chr15	62243998	62244196	chr15:62244022-62244179
chr15	62246598	62246772	chr15:62246619-62246753
chr15	62250783	62250943	chr15:62250806-62250915
chr15	62251623	62251835	chr15:62251646-62251794
chr15	62253763	62254118	chr15:62253787-62254092
chr15	62254548	62254751	chr15:62254569-62254737
chr15	62254923	62255059	chr15:62254947-62255051

chr15	62256003	62256189	chr15:62256033-62256150
chr15	62256918	62257083	chr15:62256897-62257060
chr15	62258248	62258356	chr15:62258281-62258341
chr15	62259543	62259660	chr15:62259566-62259646
chr15	62261473	62261684	chr15:62261497-62261651
chr15	62264798	62264973	chr15:62264823-62264963
chr15	62265628	62265756	chr15:62265652-62265731
chr15	62266463	62266635	chr15:62266486-62266616
chr15	62269258	62269408	chr15:62269280-62269398
chr15	62270793	62270972	chr15:62270824-62270948
chr15	62273513	62273688	chr15:62273540-62273677
chr15	62274623	62274797	chr15:62274657-62274772
chr15	62275988	62276233	chr15:62276018-62276211
chr15	62277033	62277233	chr15:62277055-62277198
chr15	62283173	62283309	chr15:62283198-62283293
chr15	62283848	62284013	chr15:62283871-62284001
chr15	62292738	62292844	chr15:62292762-62292825
chr15	62299478	62299691	chr15:62299506-62299678
chr15	62300828	62300974	chr15:62300853-62300960
chr15	62302643	62302826	chr15:62302670-62302798
chr15	62304283	62304390	chr15:62304305-62304363
chr15	62305203	62305362	chr15:62305237-62305318
chr15	62306108	62306219	chr15:62306131-62306191
chr15	62312653	62312752	chr15:62312677-62312737
chr15	62315583	62315732	chr15:62315609-62315719
chr15	62315943	62316045	chr15:62315978-62316044
chr15	62320528	62320647	chr15:62320556-62320619
chr15	62325618	62325750	chr15:62325639-62325741
chr15	62327133	62327264	chr15:62327155-62327251
chr15	62333498	62333600	chr15:62333522-62333565
chr15	62336378	62336463	chr15:62336410-62336454
chr15	62352448	62352697	chr15:62352473-62352664
chr15	70580046	70580306	chr15:70580076-70630120
chr15	70580321	70580923	chr15:70580076-70630120
chr15	70580931	70581037	chr15:70580076-70630120
chr15	70581101	70582394	chr15:70580076-70630120
chr15	70582396	70585324	chr15:70580076-70630120
chr15	70585326	70585780	chr15:70580076-70630120
chr15	70585786	70587991	chr15:70580076-70630120
chr15	70588306	70589289	chr15:70580076-70630120
chr15	70589491	70589906	chr15:70580076-70630120
chr15	70589911	70590674	chr15:70580076-70630120
chr15	70590676	70591914	chr15:70580076-70630120
chr15	70591916	70592053	chr15:70580076-70630120
chr15	70592061	70594734	chr15:70580076-70630120
chr15	70594811	70594987	chr15:70580076-70630120
chr15	70595011	70595466	chr15:70580076-70630120
chr15	70595516	70597026	chr15:70580076-70630120
chr15	70597091	70597976	chr15:70580076-70630120
chr15	70597981	70598341	chr15:70580076-70630120
chr15	70598346	70598527	chr15:70580076-70630120
chr15	70598536	70598776	chr15:70580076-70630120
chr15	70599106	70600045	chr15:70580076-70630120
chr15	70600356	70601619	chr15:70580076-70630120
chr15	70601856	70601942	chr15:70580076-70630120
chr15	70602091	70602362	chr15:70580076-70630120
chr15	70602406	70602602	chr15:70580076-70630120
chr15	70602616	70603100	chr15:70580076-70630120
chr15	70603236	70603628	chr15:70580076-70630120
chr15	70603636	70605645	chr15:70580076-70630120
chr15	70605676	70606873	chr15:70580076-70630120
chr15	70606966	70607379	chr15:70580076-70630120
chr15	70607381	70607667	chr15:70580076-70630120

chr15	70607676	70608314	chr15:70580076-70630120
chr15	70608336	70609299	chr15:70580076-70630120
chr15	70609581	70610391	chr15:70580076-70630120
chr15	70610396	70610648	chr15:70580076-70630120
chr15	70610656	70612050	chr15:70580076-70630120
chr15	70612051	70614582	chr15:70580076-70630120
chr15	70614631	70618020	chr15:70580076-70630120
chr15	70618066	70619283	chr15:70580076-70630120
chr15	70619586	70619765	chr15:70580076-70630120
chr15	70620001	70620833	chr15:70580076-70630120
chr15	70620836	70620987	chr15:70580076-70630120
chr15	70620991	70622245	chr15:70580076-70630120
chr15	70622246	70622659	chr15:70580076-70630120
chr15	70622666	70622958	chr15:70580076-70630120
chr15	70622966	70623270	chr15:70580076-70630120
chr15	70623271	70623636	chr15:70580076-70630120
chr15	70623686	70623885	chr15:70580076-70630120
chr15	70623891	70624179	chr15:70580076-70630120
chr15	70624196	70624409	chr15:70580076-70630120
chr15	70624451	70624766	chr15:70580076-70630120
chr15	70624826	70624984	chr15:70580076-70630120
chr15	70625011	70625078	chr15:70580076-70630120
chr15	70625371	70626928	chr15:70580076-70630120
chr15	70626936	70629546	chr15:70580076-70630120
chr15	70629556	70630154	chr15:70580076-70630120
chr16	9847236	9848499	chr16:9847261-9858805
chr16	9848511	9849710	chr16:9847261-9858805
chr16	9849746	9850468	chr16:9847261-9858805
chr16	9850486	9850882	chr16:9847261-9858805
chr16	9850886	9851220	chr16:9847261-9858805
chr16	9851261	9851737	chr16:9847261-9858805
chr16	9851746	9854238	chr16:9847261-9858805
chr16	9854256	9854367	chr16:9847261-9858805
chr16	9854376	9855576	chr16:9847261-9858805
chr16	9855581	9856881	chr16:9847261-9858805
chr16	9856886	9858858	chr16:9847261-9858805
chr16	9862686	9863105	chr16:9862707-9863085
chr16	9892106	9892352	chr16:9892133-9892321
chr16	9916096	9916300	chr16:9916120-9916281
chr16	9923256	9923542	chr16:9923279-9923509
chr16	9927936	9928114	chr16:9927961-9928087
chr16	9934476	9934673	chr16:9934503-9934657
chr16	9934766	9934977	chr16:9934792-9934961
chr16	9943581	9943835	chr16:9943612-9943818
chr16	9947846	9948409	chr16:9947869-9948368
chr16	9984811	9984987	chr16:9984842-9984957
chr16	10031788	10032423	chr16:10031815-10032408
chr16	10064578	10064754	chr16:10064599-10064715
chr16	10122458	10122569	chr16:10122498-10122524
chr16	10125158	10125477	chr16:10125181-10125437
chr16	10125478	10125592	chr16:10125509-10125548
chr16	10127483	10127741	chr16:10127515-10127716
chr16	10130818	10131172	chr16:10130842-10131154
chr16	10273323	10274300	chr16:10273347-10274289
chr16	10275703	10276301	chr16:10275733-10276263
chr16	10276478	10276634	chr16:10276510-10276611
chr16	13747560	13747823	chr16:13747590-13791392
chr16	13747880	13747991	chr16:13747590-13791392
chr16	13748310	13749528	chr16:13747590-13791392
chr16	13749535	13749918	chr16:13747590-13791392
chr16	13749920	13752630	chr16:13747590-13791392
chr16	13752660	13754748	chr16:13747590-13791392
chr16	13755125	13755202	chr16:13747590-13791392

chr16	13755205	13756192	chr16:13747590-13791392
chr16	13756215	13756433	chr16:13747590-13791392
chr16	13756525	13756607	chr16:13747590-13791392
chr16	13756715	13757598	chr16:13747590-13791392
chr16	13757620	13757721	chr16:13747590-13791392
chr16	13757735	13758376	chr16:13747590-13791392
chr16	13758380	13758543	chr16:13747590-13791392
chr16	13758850	13759233	chr16:13747590-13791392
chr16	13759560	13759766	chr16:13747590-13791392
chr16	13759870	13760735	chr16:13747590-13791392
chr16	13760915	13760982	chr16:13747590-13791392
chr16	13761045	13761121	chr16:13747590-13791392
chr16	13761285	13761440	chr16:13747590-13791392
chr16	13761525	13762618	chr16:13747590-13791392
chr16	13762620	13762704	chr16:13747590-13791392
chr16	13762720	13762958	chr16:13747590-13791392
chr16	13762975	13764054	chr16:13747590-13791392
chr16	13764055	13765762	chr16:13747590-13791392
chr16	13765765	13766475	chr16:13747590-13791392
chr16	13766765	13767230	chr16:13747590-13791392
chr16	13767485	13767742	chr16:13747590-13791392
chr16	13767750	13768113	chr16:13747590-13791392
chr16	13768115	13768317	chr16:13747590-13791392
chr16	13768320	13768458	chr16:13747590-13791392
chr16	13768560	13768766	chr16:13747590-13791392
chr16	13769005	13769086	chr16:13747590-13791392
chr16	13769150	13769280	chr16:13747590-13791392
chr16	13769320	13769389	chr16:13747590-13791392
chr16	13769395	13769684	chr16:13747590-13791392
chr16	13769700	13769783	chr16:13747590-13791392
chr16	13770325	13770400	chr16:13747590-13791392
chr16	13770470	13770548	chr16:13747590-13791392
chr16	13770565	13770702	chr16:13747590-13791392
chr16	13770895	13770978	chr16:13747590-13791392
chr16	13771130	13771201	chr16:13747590-13791392
chr16	13771500	13771566	chr16:13747590-13791392
chr16	13771805	13771882	chr16:13747590-13791392
chr16	13772225	13772498	chr16:13747590-13791392
chr16	13772500	13772751	chr16:13747590-13791392
chr16	13772755	13772984	chr16:13747590-13791392
chr16	13773095	13778037	chr16:13747590-13791392
chr16	13778055	13780009	chr16:13747590-13791392
chr16	13780535	13780629	chr16:13747590-13791392
chr16	13780650	13781688	chr16:13747590-13791392
chr16	13781695	13782247	chr16:13747590-13791392
chr16	13782910	13783145	chr16:13747590-13791392
chr16	13783165	13784239	chr16:13747590-13791392
chr16	13784380	13784907	chr16:13747590-13791392
chr16	13785010	13785124	chr16:13747590-13791392
chr16	13785125	13785207	chr16:13747590-13791392
chr16	13785255	13785328	chr16:13747590-13791392
chr16	13785520	13785622	chr16:13747590-13791392
chr16	13785695	13785791	chr16:13747590-13791392
chr16	13785825	13785907	chr16:13747590-13791392
chr16	13786735	13786863	chr16:13747590-13791392
chr16	13786870	13787206	chr16:13747590-13791392
chr16	13787240	13791424	chr16:13747590-13791392
chr16	13791535	13794687	chr16:13791559-13804648
chr16	13794950	13795504	chr16:13791559-13804648
chr16	13795790	13797288	chr16:13791559-13804648
chr16	13797580	13798493	chr16:13791559-13804648
chr16	13798780	13800320	chr16:13791559-13804648
chr16	13800695	13801229	chr16:13791559-13804648

chr16	13801265	13801541	chr16:13791559-13804648
chr16	13801670	13801765	chr16:13791559-13804648
chr16	13802045	13803248	chr16:13791559-13804648
chr16	13803550	13803642	chr16:13791559-13804648
chr16	13803860	13803955	chr16:13791559-13804648
chr16	13803990	13804170	chr16:13791559-13804648
chr16	13804450	13804655	chr16:13791559-13804648
chr16	13823860	13824175	chr16:13823887-13824293
chr16	13860870	13861258	chr16:13860893-13861223
chr16	13871555	13871738	chr16:13871576-13871701
chr16	13872780	13872987	chr16:13872757-13872952
chr16	13873525	13873622	chr16:13873554-13873617
chr16	13874615	13874724	chr16:13874638-13874684
chr16	24621512	24621835	chr16:24621535-24621805
chr16	24652172	24652365	chr16:24652204-24652330
chr16	24652607	24652719	chr16:24652655-24652671
chr16	24673017	24673285	chr16:24672742-24679125
chr16	24673352	24673500	chr16:24672742-24679125
chr16	24673822	24674200	chr16:24672742-24679125
chr16	24674202	24674602	chr16:24672742-24679125
chr16	24674887	24675369	chr16:24672742-24679125
chr16	24676022	24676096	chr16:24672742-24679125
chr16	24676127	24676383	chr16:24672742-24679125
chr16	24676687	24677043	chr16:24672742-24679125
chr16	24677047	24677385	chr16:24672742-24679125
chr16	24678002	24678163	chr16:24672742-24679125
chr16	24678452	24679093	chr16:24672742-24679125
chr16	24682222	24682409	chr16:24682247-24682383
chr16	24696632	24696703	chr16:24696665-24696716
chr16	24716019	24716218	chr16:24715880-24717945
chr16	24716334	24716661	chr16:24715880-24717945
chr16	24716944	24717736	chr16:24715880-24717945
chr16	24718764	24718913	chr16:24718365-24721546
chr16	24718919	24718997	chr16:24718365-24721546
chr16	24719004	24719109	chr16:24718365-24721546
chr16	24719399	24719511	chr16:24718365-24721546
chr16	24719874	24720033	chr16:24718365-24721546
chr16	24720744	24720841	chr16:24718365-24721546
chr16	24721159	24721256	chr16:24718365-24721546
chr16	24733094	24733208	chr16:24733121-24733162
chr16	26405166	26409022	chr16:26405197-26494157
chr16	26409071	26409238	chr16:26405197-26494157
chr16	26409541	26413455	chr16:26405197-26494157
chr16	26414986	26416345	chr16:26405197-26494157
chr16	26416601	26417485	chr16:26405197-26494157
chr16	26417486	26417788	chr16:26405197-26494157
chr16	26417816	26418226	chr16:26405197-26494157
chr16	26418321	26418434	chr16:26405197-26494157
chr16	26418486	26418869	chr16:26405197-26494157
chr16	26418956	26419117	chr16:26405197-26494157
chr16	26419141	26419239	chr16:26405197-26494157
chr16	26419241	26419585	chr16:26405197-26494157
chr16	26419616	26419830	chr16:26405197-26494157
chr16	26422646	26423516	chr16:26405197-26494157
chr16	26423741	26424081	chr16:26405197-26494157
chr16	26424086	26424744	chr16:26405197-26494157
chr16	26424746	26425578	chr16:26405197-26494157
chr16	26425866	26428019	chr16:26405197-26494157
chr16	26428306	26428569	chr16:26405197-26494157
chr16	26428616	26429016	chr16:26405197-26494157
chr16	26429031	26429900	chr16:26405197-26494157
chr16	26430161	26430538	chr16:26405197-26494157
chr16	26430696	26430879	chr16:26405197-26494157

chr16	26434576	26434698	chr16:26405197-26494157
chr16	26436721	26436894	chr16:26405197-26494157
chr16	26436911	26437393	chr16:26405197-26494157
chr16	26437396	26437898	chr16:26405197-26494157
chr16	26437946	26438508	chr16:26405197-26494157
chr16	26438586	26438744	chr16:26405197-26494157
chr16	26439026	26439443	chr16:26405197-26494157
chr16	26439446	26439794	chr16:26405197-26494157
chr16	26439806	26439988	chr16:26405197-26494157
chr16	26440176	26440254	chr16:26405197-26494157
chr16	26440256	26440365	chr16:26405197-26494157
chr16	26440746	26440904	chr16:26405197-26494157
chr16	26441176	26441250	chr16:26405197-26494157
chr16	26441261	26441785	chr16:26405197-26494157
chr16	26442186	26442333	chr16:26405197-26494157
chr16	26442641	26443926	chr16:26405197-26494157
chr16	26444006	26444156	chr16:26405197-26494157
chr16	26444161	26445222	chr16:26405197-26494157
chr16	26445256	26445500	chr16:26405197-26494157
chr16	26445846	26447184	chr16:26405197-26494157
chr16	26447271	26447504	chr16:26405197-26494157
chr16	26447706	26447817	chr16:26405197-26494157
chr16	26448066	26448142	chr16:26405197-26494157
chr16	26448231	26448345	chr16:26405197-26494157
chr16	26448391	26448578	chr16:26405197-26494157
chr16	26448671	26448770	chr16:26405197-26494157
chr16	26448821	26448945	chr16:26405197-26494157
chr16	26449001	26449807	chr16:26405197-26494157
chr16	26449831	26450809	chr16:26405197-26494157
chr16	26451016	26451097	chr16:26405197-26494157
chr16	26451146	26451960	chr16:26405197-26494157
chr16	26451981	26455038	chr16:26405197-26494157
chr16	26455346	26456753	chr16:26405197-26494157
chr16	26456756	26457410	chr16:26405197-26494157
chr16	26457416	26459544	chr16:26405197-26494157
chr16	26459616	26460032	chr16:26405197-26494157
chr16	26460036	26460654	chr16:26405197-26494157
chr16	26460976	26461367	chr16:26405197-26494157
chr16	26461536	26462468	chr16:26405197-26494157
chr16	26462746	26462835	chr16:26405197-26494157
chr16	26462961	26463116	chr16:26405197-26494157
chr16	26463211	26465834	chr16:26405197-26494157
chr16	26465841	26468043	chr16:26405197-26494157
chr16	26468321	26469010	chr16:26405197-26494157
chr16	26469016	26469275	chr16:26405197-26494157
chr16	26469286	26470345	chr16:26405197-26494157
chr16	26470651	26471950	chr16:26405197-26494157
chr16	26471956	26472200	chr16:26405197-26494157
chr16	26472476	26475593	chr16:26405197-26494157
chr16	26475596	26475873	chr16:26405197-26494157
chr16	26475881	26476073	chr16:26405197-26494157
chr16	26476121	26476394	chr16:26405197-26494157
chr16	26476411	26476506	chr16:26405197-26494157
chr16	26476571	26476737	chr16:26405197-26494157
chr16	26476766	26476900	chr16:26405197-26494157
chr16	26476956	26477023	chr16:26405197-26494157
chr16	26477091	26477204	chr16:26405197-26494157
chr16	26477236	26477307	chr16:26405197-26494157
chr16	26477591	26477665	chr16:26405197-26494157
chr16	26477711	26477784	chr16:26405197-26494157
chr16	26478781	26478859	chr16:26405197-26494157
chr16	26478971	26479040	chr16:26405197-26494157
chr16	26479281	26479347	chr16:26405197-26494157

chr16	26479431	26479510	chr16:26405197-26494157
chr16	26479576	26479667	chr16:26405197-26494157
chr16	26480066	26480137	chr16:26405197-26494157
chr16	26480156	26480236	chr16:26405197-26494157
chr16	26480371	26480460	chr16:26405197-26494157
chr16	26480461	26480706	chr16:26405197-26494157
chr16	26480791	26480867	chr16:26405197-26494157
chr16	26480871	26481061	chr16:26405197-26494157
chr16	26481076	26481361	chr16:26405197-26494157
chr16	26481431	26481723	chr16:26405197-26494157
chr16	26481741	26481836	chr16:26405197-26494157
chr16	26481841	26481912	chr16:26405197-26494157
chr16	26481921	26481992	chr16:26405197-26494157
chr16	26482001	26482126	chr16:26405197-26494157
chr16	26482131	26482195	chr16:26405197-26494157
chr16	26482201	26482296	chr16:26405197-26494157
chr16	26482451	26482655	chr16:26405197-26494157
chr16	26482666	26482737	chr16:26405197-26494157
chr16	26482766	26482885	chr16:26405197-26494157
chr16	26482886	26483316	chr16:26405197-26494157
chr16	26483371	26483446	chr16:26405197-26494157
chr16	26483501	26483615	chr16:26405197-26494157
chr16	26483621	26483835	chr16:26405197-26494157
chr16	26483936	26484074	chr16:26405197-26494157
chr16	26484256	26484331	chr16:26405197-26494157
chr16	26484371	26485005	chr16:26405197-26494157
chr16	26485016	26485399	chr16:26405197-26494157
chr16	26485411	26485495	chr16:26405197-26494157
chr16	26485751	26485831	chr16:26405197-26494157
chr16	26486121	26487412	chr16:26405197-26494157
chr16	26487596	26488058	chr16:26405197-26494157
chr16	26488061	26488426	chr16:26405197-26494157
chr16	26488491	26490290	chr16:26405197-26494157
chr16	26490291	26490441	chr16:26405197-26494157
chr16	26490446	26491063	chr16:26405197-26494157
chr16	26491071	26491638	chr16:26405197-26494157
chr16	26491646	26492707	chr16:26405197-26494157
chr16	26492801	26493016	chr16:26405197-26494157
chr16	26493086	26493218	chr16:26405197-26494157
chr16	26493251	26493437	chr16:26405197-26494157
chr16	26493486	26493974	chr16:26405197-26494157
chr16	26494011	26494133	chr16:26405197-26494157
chr16	29911672	29913263	chr16:29911695-29913241
chr16	29916142	29916486	chr16:29916172-29916286;chr16:29916332-29916472
chr16	29917082	29917469	chr16:29917108-29917377;chr16:29917399-29917448
chr16	29917632	29918462	chr16:29917656-29918429
chr16	29922277	29922904	chr16:29922298-29922878
chr16	29922957	29923194	chr16:29922981-29923188
chr16	29923252	29923716	chr16:29923280-29923691
chr16	29930712	29931009	chr16:29930740-29931185
chr16	29931072	29931202	chr16:29930740-29931185
chr16	29933327	29934042	chr16:29933354-29934682
chr16	29934047	29934704	chr16:29933354-29934682
chr16	29935522	29935848	chr16:29935532-29936031
chr16	29935852	29936050	chr16:29935532-29936031
chr16	29936827	29936929	chr16:29936859-29936902
chr16	29937087	29937934	chr16:29937110-29939252
chr16	29938092	29939026	chr16:29937110-29939252
chr16	29939557	29939817	chr16:29939588-29940254
chr16	30996497	30996638	chr16:30996518-30996618
chr16	30996747	30997291	chr16:30996780-30997268
chr16	30997347	30997566	chr16:30997369-30997525
chr16	30997717	30997888	chr16:30997743-30997852

chr16	30997902	30998040	chr16:30997925-30998028
chr16	30998132	30998342	chr16:30998160-30998323
chr16	30999067	30999661	chr16:30999088-31000473
chr16	30999942	31000499	chr16:30999088-31000473
chr16	31000537	31000636	chr16:31000576-31000590
chr16	31003567	31004590	chr16:31003590-31004561
chr16	31004642	31004823	chr16:31004667-31004805
chr16	31007972	31008112	chr16:31008003-31008077
chr16	31008247	31008412	chr16:31008271-31008380
chr16	31008792	31008934	chr16:31008822-31008896
chr16	31012217	31012366	chr16:31012248-31012323
chr16	31012392	31012536	chr16:31012414-31012514
chr16	31012827	31012964	chr16:31012849-31012924
chr16	31021662	31021986	chr16:31021687-31021949
chr16	31044167	31044281	chr16:31044209-31044230
chr16	31044287	31045193	chr16:31044320-31044397;chr16:31044415-31045156
chr16	31045312	31045442	chr16:31045333-31045435
chr16	31045502	31045671	chr16:31045524-31045646
chr16	31045747	31045897	chr16:31045782-31045857
chr16	31046267	31046378	chr16:31046290-31046361
chr16	31046987	31047098	chr16:31047019-31047082
chr16	31047382	31047913	chr16:31047377-31047876
chr16	31049222	31049398	chr16:31049246-31049355
chr16	31049412	31049554	chr16:31049434-31049511
chr16	31049797	31049981	chr16:31049830-31049968
chr16	31050377	31050784	chr16:31050400-31050599;chr16:31050640-31050758
chr16	31050837	31051003	chr16:31050861-31050972
chr16	31051022	31051164	chr16:31051043-31051139
chr16	31051197	31051460	chr16:31051232-31051489
chr16	31053437	31054322	chr16:31053462-31054296
chr16	31054442	31054587	chr16:31054470-31054563
chr16	31060812	31061074	chr16:31060833-31061201
chr16	31085707	31085964	chr16:31085742-31085952
chr16	31087187	31094660	chr16:31087222-31094635
chr16	31117402	31117571	chr16:31117427-31117534
chr16	31119092	31119492	chr16:31119127-31119466
chr16	31119592	31119828	chr16:31119614-31119800
chr16	31120332	31120756	chr16:31120367-31120739
chr16	31120802	31120920	chr16:31120831-31120900
chr16	31120972	31121143	chr16:31120993-31121104
chr16	31121367	31121479	chr16:31121397-31121445
chr16	31121502	31121672	chr16:31121525-31121645
chr16	31121697	31121839	chr16:31121721-31121820
chr16	31121977	31122122	chr16:31122008-31122082
chr16	31122382	31122566	chr16:31122411-31122540
chr16	31122587	31122748	chr16:31122620-31122710
chr16	31123167	31124147	chr16:31123189-31124110
chr16	31127137	31127211	chr16:31127074-31127190
chr16	31128762	31129241	chr16:31128783-31129213
chr16	31131472	31131619	chr16:31131506-31131581
chr16	31131637	31131862	chr16:31131659-31131835
chr16	31138322	31138438	chr16:31138355-31138409
chr16	31138487	31138695	chr16:31138509-31138674
chr16	31139347	31139482	chr16:31139370-31139460
chr16	31141312	31141510	chr16:31141337-31141478
chr16	31141557	31141693	chr16:31141587-31141681
chr16	31141747	31141952	chr16:31141776-31141927
chr16	31142042	31142733	chr16:31142066-31142714
chr16	31225307	31225626	chr16:31225341-31225618
chr16	31226027	31226480	chr16:31226052-31226449
chr16	31230387	31230540	chr16:31230415-31230511
chr16	31230577	31230863	chr16:31230609-31230840
chr16	31232197	31232273	chr16:31232216-31232239

chr16	31234122	31234295	chr16:31234147-31234266
chr16	31235467	31236241	chr16:31235501-31236510
chr16	52580358	52580473	chr16:52580394-52580424
chr16	52585953	52586385	chr16:52585985-52586346
chr16	52587943	52588125	chr16:52587975-52588091
chr16	52596693	52596803	chr16:52596725-52596774
chr16	52607068	52607279	chr16:52607090-52607256
chr16	52612258	52612409	chr16:52612292-52612380
chr16	52615143	52615281	chr16:52615166-52615253
chr16	52621328	52621474	chr16:52621353-52621452
chr16	52624513	52626586	chr16:52624535-52650827
chr16	52626588	52627060	chr16:52624535-52650827
chr16	52627273	52628105	chr16:52624535-52650827
chr16	52628563	52630468	chr16:52624535-52650827
chr16	52630828	52631465	chr16:52624535-52650827
chr16	52631518	52633696	chr16:52624535-52650827
chr16	52633698	52633863	chr16:52624535-52650827
chr16	52633868	52635886	chr16:52624535-52650827
chr16	52636233	52636349	chr16:52624535-52650827
chr16	52636353	52636778	chr16:52624535-52650827
chr16	52636788	52637308	chr16:52624535-52650827
chr16	52637593	52642090	chr16:52624535-52650827
chr16	52642368	52646752	chr16:52624535-52650827
chr16	52646753	52647213	chr16:52624535-52650827
chr16	52647223	52647298	chr16:52624535-52650827
chr16	52647353	52648155	chr16:52624535-52650827
chr16	52648453	52650855	chr16:52624535-52650827
chr16	52655993	52656841	chr16:52656026-52656806
chr16	52660068	52660159	chr16:52660108-52660131
chr16	52663073	52663290	chr16:52663099-52663270
chr16	52663813	52664017	chr16:52663820-52663980
chr16	52664573	52664820	chr16:52664598-52664795
chr16	52664838	52665012	chr16:52664861-52665000
chr16	52681938	52682050	chr16:52681963-52682024
chr16	52685828	52686043	chr16:52685863-52686017
chr16	85979003	85979285	chr16:85979024-85979236
chr16	85979503	85979615	chr16:85979553-85979571
chr16	85981438	85981754	chr16:85981470-85981747
chr16	85982093	85982787	chr16:85982101-85984378
chr16	85983043	85983158	chr16:85982101-85984378
chr16	85983428	85984404	chr16:85982101-85984378
chr16	85995048	85995214	chr16:85995072-85995181
chr16	85995448	85995843	chr16:85995479-85995800
chr16	86005803	86005945	chr16:86005838-86018633
chr16	86005953	86006226	chr16:86005838-86018633
chr16	86007698	86009726	chr16:86005838-86018633
chr16	86009728	86009833	chr16:86005838-86018633
chr16	86010123	86013452	chr16:86005838-86018633
chr16	86013463	86014458	chr16:86005838-86018633
chr16	86014548	86015157	chr16:86005838-86018633
chr16	86015473	86017970	chr16:86005838-86018633
chr16	86017973	86018672	chr16:86005838-86018633
chr16	86052173	86052446	chr16:86052197-86052414
chr16	86054473	86054599	chr16:86054488-86054571
chr16	86060863	86061316	chr16:86060887-86061287
chr17	1963109	1965303	chr17:1963132-1965551
chr17	1965309	1965583	chr17:1963132-1965551
chr17	1968334	1968486	chr17:1968366-1968448
chr17	1968734	1969019	chr17:1968761-1968973
chr17	1969274	1969412	chr17:1969296-1969383
chr17	1972039	1972265	chr17:1972071-1972225
chr17	1975194	1975336	chr17:1975216-1975297
chr17	1985069	1985284	chr17:1985103-1985250

chr17	1985494	1985633	chr17:1985520-1985604
chr17	1988984	1989237	chr17:1989018-1989195
chr17	1990334	1992094	chr17:1990368-1992055
chr17	1992939	1993085	chr17:1992974-1993063
chr17	1998054	1998447	chr17:1998078-1998406
chr17	2004679	2004894	chr17:2004415-2004854
chr17	2034424	2034602	chr17:2034454-2034671
chr17	2036739	2036887	chr17:2036770-2037048
chr17	2036969	2037086	chr17:2036770-2037048
chr17	2053854	2053965	chr17:2053875-2053944
chr17	2075919	2076516	chr17:2075951-2076365;chr17:2076391-2076472
chr17	2089934	2090144	chr17:2089956-2090125
chr17	2091669	2091858	chr17:2091692-2091809
chr17	2114244	2114388	chr17:2114297-2114310;chr17:2114317-2114383
chr17	2115884	2116063	chr17:2115908-2116031
chr17	2116969	2117166	chr17:2116990-2117138
chr17	2117409	2117691	chr17:2117432-2117708
chr17	2118754	2119002	chr17:2118775-2118987
chr17	2119174	2119363	chr17:2119198-2119309
chr17	2132584	2132781	chr17:2132616-2132758
chr17	2133919	2134027	chr17:2133940-2133995
chr17	2135939	2136055	chr17:2135973-2136022
chr17	2136219	2136925	chr17:2136243-2136904
chr17	2139764	2139976	chr17:2139785-2139931
chr17	2140059	2140337	chr17:2140090-2140331
chr17	2140644	2140715	chr17:2140592-2140685
chr17	2140759	2140911	chr17:2140788-2140874
chr17	2143264	2143519	chr17:2143296-2143485
chr17	2146054	2146617	chr17:2146089-2146588
chr17	2147929	2148027	chr17:2147951-2148013
chr17	2148134	2148252	chr17:2148166-2148206
chr17	2169199	2169507	chr17:2169223-2169493
chr17	2185909	2186185	chr17:2185940-2186153
chr17	2186884	2187069	chr17:2186918-2187029
chr17	2195824	2195956	chr17:2195845-2195924
chr17	2196129	2196310	chr17:2196164-2196271
chr17	2200514	2200660	chr17:2200536-2200647
chr17	2201134	2201395	chr17:2201156-2201349
chr17	2202169	2203983	chr17:2202199-2203958
chr17	2206274	2206834	chr17:2206307-2206813
chr17	2206904	2207325	chr17:2206926-2207311
chr17	2208064	2208625	chr17:2208090-2208589
chr17	2210379	2210454	chr17:2210329-2210611
chr17	2210549	2210629	chr17:2210329-2210611
chr17	2213039	2213328	chr17:2213070-2213284
chr17	2218639	2219038	chr17:2218671-2219022
chr17	2221134	2221307	chr17:2221156-2221290
chr17	2222094	2222240	chr17:2222119-2222223
chr17	2224574	2224750	chr17:2224599-2224719
chr17	2224814	2224954	chr17:2224835-2224910
chr17	2226399	2226659	chr17:2226429-2226639
chr17	2226914	2228569	chr17:2226948-2228554
chr17	17576409	17576518	chr17:17576462-17576475
chr17	17578649	17578871	chr17:17578679-17578836
chr17	17579494	17579703	chr17:17579523-17579685
chr17	17580854	17580937	chr17:17580888-17580943
chr17	17584754	17585143	chr17:17584786-17585107
chr17	17585784	17585884	chr17:17585808-17585877
chr17	17586829	17587204	chr17:17586859-17588364
chr17	17587484	17587994	chr17:17586859-17588364
chr17	17588034	17588220	chr17:17586859-17588364
chr17	17588224	17588413	chr17:17586859-17588364
chr17	17597714	17597822	chr17:17597760-17597780

chr17	17627319	17627485	chr17:17627341-17627473
chr17	17662324	17662747	chr17:17662345-17662700
chr17	17669109	17669633	chr17:17669131-17669616
chr17	17674079	17674184	chr17:17674129-17674135
chr17	17677589	17677721	chr17:17677611-17677714
chr17	17680104	17680189	chr17:17679999-17682843
chr17	17680269	17680769	chr17:17679999-17682843
chr17	17680774	17681060	chr17:17679999-17682843
chr17	17681069	17681226	chr17:17679999-17682843
chr17	17681229	17682879	chr17:17679999-17682843
chr17	17685414	17685564	chr17:17685445-17685529
chr17	17696224	17697098	chr17:17696246-17701827
chr17	17697124	17700004	chr17:17696246-17701827
chr17	17700009	17701854	chr17:17696246-17701827
chr17	17702724	17703297	chr17:17702757-17703256
chr17	17707044	17707472	chr17:17707069-17707428
chr17	17712429	17712538	chr17:17712480-17712483
chr17	17712564	17712782	chr17:17712595-17712753
chr17	17713249	17714549	chr17:17713283-17715044
chr17	17714579	17714724	chr17:17713283-17715044
chr17	17714734	17715139	chr17:17713283-17715044;chr17:17715068-17715098
chr17	17715259	17716453	chr17:17715286-17715370;chr17:17715386-17716165;chr17:17716223-17716793
chr17	17716564	17716803	chr17:17716223-17716793
chr17	17716854	17717106	chr17:17716882-17717083
chr17	17717534	17717746	chr17:17717558-17717724
chr17	17717904	17718077	chr17:17717928-17718061
chr17	17718114	17718299	chr17:17718148-17718258
chr17	17718509	17718675	chr17:17718534-17718643
chr17	17719149	17719370	chr17:17719173-17719342
chr17	17719499	17719709	chr17:17719520-17719687
chr17	17719739	17720073	chr17:17719770-17720032
chr17	17720244	17720491	chr17:17720271-17720450
chr17	17720544	17720788	chr17:17720569-17720771
chr17	17720844	17720955	chr17:17720879-17720905
chr17	17720984	17721272	chr17:17721009-17721230
chr17	17721544	17721724	chr17:17721573-17721688
chr17	17722299	17722581	chr17:17722326-17722548
chr17	17722604	17722794	chr17:17722633-17722768
chr17	17722829	17723288	chr17:17722851-17723265
chr17	17723379	17723874	chr17:17723403-17723835
chr17	17726804	17726947	chr17:17726831-17726921
chr17	17727914	17728441	chr17:17727913-17728412
chr17	17740014	17740356	chr17:17740040-17740325
chr17	17746834	17751112	chr17:17746827-17751097
chr17	17752109	17752210	chr17:17752136-17752173
chr17	17754184	17754292	chr17:17754206-17754266
chr17	17761054	17761192	chr17:17761082-17761169
chr17	17764764	17764903	chr17:17764789-17764865
chr17	17766009	17766202	chr17:17766044-17766162
chr17	17766554	17766736	chr17:17766587-17766724
chr17	17769574	17769758	chr17:17769609-17769733
chr17	17770164	17770265	chr17:17770189-17770238
chr17	17772629	17773264	chr17:17772653-17773228
chr17	17775244	17775402	chr17:17775278-17775378
chr17	17782914	17783088	chr17:17782940-17783057
chr17	17785984	17786209	chr17:17786018-17786177
chr17	17787924	17788109	chr17:17787947-17788082
chr17	17796939	17797155	chr17:17796974-17797124
chr17	17801874	17802024	chr17:17801909-17801988
chr17	17810739	17810875	chr17:17810760-17810845
chr17	17875554	17875753	chr17:17875575-17875734
chr17	17919489	17919601	chr17:17919520-17919574
chr17	17921299	17922039	chr17:17921323-17922000

chr17	17924414	17924587	chr17:17924436-17924552
chr17	17924714	17925003	chr17:17924735-17924977
chr17	17925029	17925217	chr17:17925058-17925171
chr17	17927914	17928045	chr17:17927937-17928018
chr17	17928529	17928682	chr17:17928564-17928654
chr17	17929609	17929748	chr17:17929632-17929730
chr17	17931519	17931727	chr17:17931545-17931691
chr17	17931899	17932003	chr17:17931928-17931973
chr17	17942169	17942518	chr17:17942194-17942482
chr17	26557401	26557582	chr17:26557425-26562307
chr17	26557706	26557787	chr17:26557425-26562307
chr17	26558001	26558383	chr17:26557425-26562307
chr17	26558441	26558637	chr17:26557425-26562307
chr17	26558691	26558783	chr17:26557425-26562307
chr17	26558806	26559005	chr17:26557425-26562307
chr17	26559071	26559150	chr17:26557425-26562307
chr17	26559171	26559448	chr17:26557425-26562307
chr17	26559481	26559974	chr17:26557425-26562307
chr17	26560026	26560105	chr17:26557425-26562307
chr17	26560261	26560371	chr17:26557425-26562307
chr17	26560486	26560564	chr17:26557425-26562307
chr17	26560646	26560728	chr17:26557425-26562307
chr17	26560971	26561086	chr17:26557425-26562307
chr17	26561096	26561225	chr17:26557425-26562307
chr17	26561281	26561359	chr17:26557425-26562307
chr17	26561421	26561500	chr17:26557425-26562307
chr17	26561686	26561788	chr17:26557425-26562307
chr17	26561951	26562031	chr17:26557425-26562307
chr17	26575521	26575883	chr17:26575555-26575844
chr17	26577571	26577934	chr17:26577603-26578253
chr17	26583431	26583580	chr17:26583463-26583549
chr17	26588976	26589134	chr17:26588841-26596731
chr17	26589296	26589413	chr17:26588841-26596731
chr17	26589611	26589683	chr17:26588841-26596731
chr17	26589966	26590655	chr17:26588841-26596731
chr17	26590676	26591005	chr17:26588841-26596731
chr17	26591041	26591105	chr17:26588841-26596731
chr17	26591151	26591230	chr17:26588841-26596731
chr17	26591286	26591363	chr17:26588841-26596731
chr17	26591556	26591752	chr17:26588841-26596731
chr17	26591766	26593165	chr17:26588841-26596731
chr17	26593166	26593885	chr17:26588841-26596731
chr17	26593896	26595755	chr17:26588841-26596731
chr17	26595761	26596775	chr17:26588841-26596731
chr17	26597901	26598749	chr17:26597935-26598725
chr17	26599221	26599429	chr17:26599243-26599391
chr17	26610186	26610539	chr17:26610220-26610513
chr17	43697663	43697772	chr17:43697693-43697738
chr17	43697953	43698529	chr17:43697975-43698518
chr17	43699243	43699440	chr17:43699266-43699407
chr17	43707243	43707815	chr17:43707269-43707822
chr17	43713623	43714186	chr17:43713648-43715325
chr17	43714513	43715354	chr17:43713648-43715325
chr17	43716513	43716922	chr17:43716340-43716886
chr17	43717733	43718170	chr17:43717754-43719666
chr17	43718448	43718573	chr17:43717754-43719666
chr17	43718858	43719012	chr17:43717754-43719666
chr17	43719013	43719706	chr17:43717754-43719666
chr17	43723243	43723613	chr17:43723266-43723598
chr17	43750168	43750291	chr17:43750192-43750268
chr17	43809033	43809347	chr17:43809068-43809310
chr17	43810038	43810360	chr17:43810068-43810343
chr17	43810693	43810905	chr17:43810718-43810892

chr17	43811623	43812061	chr17:43811649-43812035
chr17	43861648	43861956	chr17:43861672-43861943
chr17	43884343	43884496	chr17:43884375-43884463
chr17	43893793	43893982	chr17:43893828-43893948
chr17	43898698	43898842	chr17:43898720-43898806
chr17	43902828	43902999	chr17:43902851-43902961
chr17	43906558	43906709	chr17:43906580-43906687
chr17	43906893	43907047	chr17:43906926-43907013
chr17	43907433	43907608	chr17:43907459-43907580
chr17	43907753	43907972	chr17:43907782-43907936
chr17	43908223	43908334	chr17:43908245-43908306
chr17	43910478	43910611	chr17:43910503-43910576
chr17	43910783	43910927	chr17:43910817-43910903
chr17	43911058	43911230	chr17:43911079-43911215
chr17	43911343	43911440	chr17:43911372-43911414
chr17	43911958	43913221	chr17:43911989-43913194
chr17	43920993	43921558	chr17:43921016-43921527
chr17	43922223	43924470	chr17:43922255-43924438
chr17	43971723	43972423	chr17:43971747-43972966
chr17	43972448	43973007	chr17:43971747-43972966
chr17	43985013	43985777	chr17:43985035-43985749
chr17	43988073	43988171	chr17:43988100-43988145
chr17	44039658	44039876	chr17:44039686-44039836
chr17	44046503	44046688	chr17:44046530-44046665
chr17	44049203	44049334	chr17:44049224-44049311
chr17	44049398	44049517	chr17:44049445-44049449
chr17	44050118	44051290	chr17:44050148-44055806
chr17	44051293	44053263	chr17:44050148-44055806
chr17	44053268	44055272	chr17:44050148-44055806
chr17	44055278	44055845	chr17:44050148-44055806
chr17	44060508	44061325	chr17:44060543-44061296
chr17	44063003	44063149	chr17:44063024-44063110
chr17	44064373	44064490	chr17:44064405-44064461
chr17	44067218	44067463	chr17:44067243-44067441
chr17	44068793	44068979	chr17:44068825-44068952
chr17	44071258	44071369	chr17:44071289-44071343
chr17	44073753	44074070	chr17:44073764-44074030
chr17	44076593	44077100	chr17:44076615-44077060
chr17	44087653	44087787	chr17:44087675-44087768
chr17	44091583	44091725	chr17:44091608-44091690
chr17	44095953	44096123	chr17:44095983-44096096
chr17	44101298	44101776	chr17:44101321-44103714
chr17	44101783	44102071	chr17:44101321-44103714
chr17	44102078	44103457	chr17:44101321-44103714
chr17	44103468	44103747	chr17:44101321-44103714
chr17	44107328	44107469	chr17:44107281-44109069
chr17	44107513	44107612	chr17:44107281-44109069
chr17	44107658	44107965	chr17:44107281-44109069
chr17	44107968	44108674	chr17:44107281-44109069
chr17	44108698	44109083	chr17:44107281-44109069
chr17	44109388	44109703	chr17:44109412-44109665
chr17	44110423	44110600	chr17:44110445-44110558
chr17	44110733	44110854	chr17:44110768-44110826
chr17	44111503	44111672	chr17:44111526-44111651
chr17	44112643	44112753	chr17:44112678-44112732
chr17	44112863	44113171	chr17:44112886-44113136
chr17	44115873	44116097	chr17:44115903-44116052
chr17	44116363	44116617	chr17:44116392-44116581
chr17	44117043	44117289	chr17:44117067-44117250
chr17	44127873	44128081	chr17:44127898-44128070
chr17	44143878	44144134	chr17:44143902-44144098
chr17	44144893	44145045	chr17:44144914-44145033
chr17	44159783	44159922	chr17:44159806-44159908

chr17	44171903	44172102	chr17:44171925-44172067
chr17	44230248	44230357	chr17:44230269-44230332
chr17	44248188	44249634	chr17:44248220-44249598
chr17	44269753	44270099	chr17:44269784-44270166
chr17	44270158	44270291	chr17:44270188-44270272
chr17	44270918	44271272	chr17:44270941-44271251
chr17	44273708	44274117	chr17:44273733-44274087
chr17	44301003	44301181	chr17:44301036-44301174
chr17	44370073	44370314	chr17:44370098-44371395
chr17	44370603	44370718	chr17:44370098-44371395
chr17	44370738	44371258	chr17:44370098-44371395
chr17	44372433	44372609	chr17:44372464-44375108
chr17	44372613	44373116	chr17:44372464-44375108
chr17	44373208	44373424	chr17:44372464-44375108
chr17	44373438	44373663	chr17:44372464-44375108
chr17	44373668	44373976	chr17:44372464-44375108
chr17	44373983	44374137	chr17:44372464-44375108
chr17	44374213	44374451	chr17:44372464-44375108
chr17	44374508	44374596	chr17:44372464-44375108
chr17	44374608	44374698	chr17:44372464-44375108
chr17	44374723	44374867	chr17:44372464-44375108
chr17	44374983	44375071	chr17:44372464-44375108
chr17	44377158	44377272	chr17:44377159-44377231
chr17	44379978	44380056	chr17:44380003-44380075
chr17	44382903	44382974	chr17:44382874-44382946
chr17	44383578	44383651	chr17:44383594-44383675
chr17	44399658	44399803	chr17:44399687-44399759
chr17	44400298	44400374	chr17:44400318-44400393
chr17	44405823	44405908	chr17:44405757-44405876
chr17	44407813	44407947	chr17:44407815-44409347
chr17	44407953	44408142	chr17:44407815-44409347
chr17	44408163	44408245	chr17:44407815-44409347
chr17	44408248	44408360	chr17:44407815-44409347
chr17	44408363	44408698	chr17:44407815-44409347
chr17	44408723	44408911	chr17:44407815-44409347
chr17	44409053	44409379	chr17:44407815-44409347
chr17	44409938	44410018	chr17:44409917-44410022
chr17	44412888	44412985	chr17:44412910-44412960
chr17	44414638	44414751	chr17:44414661-44414716
chr17	44414773	44414949	chr17:44414794-44414934
chr17	44415013	44415188	chr17:44415040-44415160
chr17	77086648	77086852	chr17:77086676-77086967
chr17	77086918	77086985	chr17:77086676-77086967
chr17	77090508	77090641	chr17:77090532-77090611
chr17	77091568	77091680	chr17:77091594-77091656
chr17	77092708	77092807	chr17:77092734-77092774
chr17	77093338	77093517	chr17:77093373-77093506
chr17	77093748	77093852	chr17:77093773-77093827
chr17	77097643	77097751	chr17:77097665-77097726
chr17	77099213	77099352	chr17:77099243-77099336
chr17	77100133	77100236	chr17:77100154-77100208
chr17	77102703	77102884	chr17:77102732-77102870
chr17	77111553	77111872	chr17:77111575-77111830
chr17	77231818	77231898	chr17:77231847-77231887
chr17	77303773	77303932	chr17:77303805-77303906
chr17	77478504	77478714	chr17:77478535-77478680
chr17	77512009	77512280	chr17:77512036-77512230
chr18	6432279	6433109	chr18:6432227-6438578
chr18	6433394	6434044	chr18:6432227-6438578
chr18	6434059	6434152	chr18:6432227-6438578
chr18	6434379	6434479	chr18:6432227-6438578
chr18	6434484	6436239	chr18:6432227-6438578
chr18	6436349	6436685	chr18:6432227-6438578

chr18	6436784	6437041	chr18:6432227-6438578
chr18	6437329	6437571	chr18:6432227-6438578
chr18	6437599	6438607	chr18:6432227-6438578
chr18	40323160	40323719	chr18:40323191-40323685
chr18	40500630	40500750	chr18:40500661-40500725
chr18	40503505	40503741	chr18:40503536-40503728
chr18	40528930	40529032	chr18:40528951-40529013
chr18	40554010	40554152	chr18:40554038-40554112
chr18	40613740	40613852	chr18:40613775-40613832
chr18	40695360	40695678	chr18:40695381-40695657
chr18	52716280	52716771	chr18:52716306-52762988
chr18	52716860	52716973	chr18:52716306-52762988
chr18	52716980	52718616	chr18:52716306-52762988
chr18	52718630	52718824	chr18:52716306-52762988
chr18	52718985	52719061	chr18:52716306-52762988
chr18	52719100	52719374	chr18:52716306-52762988
chr18	52719375	52723022	chr18:52716306-52762988
chr18	52723040	52724222	chr18:52716306-52762988
chr18	52724225	52727945	chr18:52716306-52762988
chr18	52727960	52728105	chr18:52716306-52762988
chr18	52728165	52728301	chr18:52716306-52762988
chr18	52728605	52728775	chr18:52716306-52762988
chr18	52728790	52728878	chr18:52716306-52762988
chr18	52728890	52729121	chr18:52716306-52762988
chr18	52729490	52729854	chr18:52716306-52762988
chr18	52729915	52730049	chr18:52716306-52762988
chr18	52730095	52732844	chr18:52716306-52762988
chr18	52733135	52733273	chr18:52716306-52762988
chr18	52733570	52734994	chr18:52716306-52762988
chr18	52735010	52735476	chr18:52716306-52762988
chr18	52735480	52735756	chr18:52716306-52762988
chr18	52736055	52736293	chr18:52716306-52762988
chr18	52736295	52739147	chr18:52716306-52762988
chr18	52739150	52739934	chr18:52716306-52762988
chr18	52739950	52741944	chr18:52716306-52762988
chr18	52741945	52742222	chr18:52716306-52762988
chr18	52742250	52743244	chr18:52716306-52762988
chr18	52743255	52743515	chr18:52716306-52762988
chr18	52743855	52745202	chr18:52716306-52762988
chr18	52745370	52747897	chr18:52716306-52762988
chr18	52747905	52748144	chr18:52716306-52762988
chr18	52748145	52748554	chr18:52716306-52762988
chr18	52748950	52749272	chr18:52716306-52762988
chr18	52749295	52749501	chr18:52716306-52762988
chr18	52749595	52749829	chr18:52716306-52762988
chr18	52750145	52751009	chr18:52716306-52762988
chr18	52751010	52751982	chr18:52716306-52762988
chr18	52751985	52754537	chr18:52716306-52762988
chr18	52754555	52757007	chr18:52716306-52762988
chr18	52757015	52758841	chr18:52716306-52762988
chr18	52759140	52759554	chr18:52716306-52762988
chr18	52759595	52760774	chr18:52716306-52762988
chr18	52760780	52763027	chr18:52716306-52762988
chr18	52773000	52773158	chr18:52772990-52775884
chr18	52773165	52773737	chr18:52772990-52775884
chr18	52773740	52774789	chr18:52772990-52775884
chr18	52774810	52775913	chr18:52772990-52775884
chr18	52784145	52784335	chr18:52784179-52784314
chr18	52786705	52786843	chr18:52786726-52786829
chr18	52791315	52791566	chr18:52791350-52791546
chr18	53428510	53428825	chr18:53428533-53428794
chr18	53429740	53429865	chr18:53429775-53429829
chr18	53452100	53452243	chr18:53452129-53452205

chr18	53453790	53453924	chr18:53453815-53453887
chr18	53455820	53456033	chr18:53455843-53455992
chr18	53458925	53459165	chr18:53458947-53459129
chr18	53497590	53497731	chr18:53497614-53497703
chr18	53497870	53497973	chr18:53497894-53497964
chr18	53505040	53506435	chr18:53505061-53506416
chr18	53527925	53528114	chr18:53527960-53557813
chr18	53528150	53528247	chr18:53527960-53557813
chr18	53528280	53528361	chr18:53527960-53557813
chr18	53528395	53528764	chr18:53527960-53557813
chr18	53528785	53529648	chr18:53527960-53557813
chr18	53529665	53530058	chr18:53527960-53557813
chr18	53530060	53531366	chr18:53527960-53557813
chr18	53531370	53531569	chr18:53527960-53557813
chr18	53531580	53532703	chr18:53527960-53557813
chr18	53532710	53533797	chr18:53527960-53557813
chr18	53533820	53534090	chr18:53527960-53557813
chr18	53534095	53536833	chr18:53527960-53557813
chr18	53536835	53537527	chr18:53527960-53557813
chr18	53537545	53538427	chr18:53527960-53557813
chr18	53538715	53538955	chr18:53527960-53557813
chr18	53538960	53539330	chr18:53527960-53557813
chr18	53539605	53541139	chr18:53527960-53557813
chr18	53541350	53541762	chr18:53527960-53557813
chr18	53541765	53543374	chr18:53527960-53557813
chr18	53543385	53544426	chr18:53527960-53557813
chr18	53544430	53547662	chr18:53527960-53557813
chr18	53547675	53550764	chr18:53527960-53557813
chr18	53550770	53551325	chr18:53527960-53557813
chr18	53551625	53553734	chr18:53527960-53557813
chr18	53553735	53553871	chr18:53527960-53557813
chr18	53553875	53554642	chr18:53527960-53557813
chr18	53565210	53565454	chr18:53565235-53565434
chr18	53565545	53565686	chr18:53565567-53565658
chr18	53587360	53587485	chr18:53587392-53587430
chr18	53670815	53671165	chr18:53670843-53671135
chr18	53684935	53685043	chr18:53684958-53685010
chr18	53690600	53690677	chr18:53690632-53690693
chr18	53692550	53692658	chr18:53692571-53692626
chr18	53694860	53694976	chr18:53694882-53696473
chr18	53695305	53696499	chr18:53694882-53696473
chr18	53707825	53707891	chr18:53707562-53707881
chr18	53709335	53709523	chr18:53709365-53709575
chr18	53709525	53709606	chr18:53709365-53709575
chr18	53717285	53717426	chr18:53717310-53717408
chr18	53723155	53723313	chr18:53723135-53723285
chr18	53727310	53727448	chr18:53727334-53727435
chr18	53727640	53728541	chr18:53727671-53728509
chr18	53729905	53730367	chr18:53729927-53730334
chr18	53735435	53735625	chr18:53735467-53735601
chr18	53744720	53744976	chr18:53744752-53744959
chr18	53745305	53745492	chr18:53745339-53745463
chr18	53746775	53746893	chr18:53746811-53746852
chr18	53750475	53750912	chr18:53750503-53751181
chr18	53750915	53751193	chr18:53750503-53751181
chr18	53752400	53752655	chr18:53752435-53752622
chr18	53754445	53755001	chr18:53754467-53754991
chr18	53755605	53755824	chr18:53755640-53755814
chr18	53757380	53757558	chr18:53757415-53757534
chr18	53759150	53759335	chr18:53759178-53759288
chr18	53763120	53763567	chr18:53763145-53763550
chr18	53766760	53768438	chr18:53766792-53797273
chr18	53768445	53768903	chr18:53766792-53797273

chr18	53768915	53770218	chr18:53766792-53797273
chr18	53770510	53771043	chr18:53766792-53797273
chr18	53771085	53771518	chr18:53766792-53797273
chr18	53771555	53771834	chr18:53766792-53797273
chr18	53771835	53772129	chr18:53766792-53797273
chr18	53772140	53772864	chr18:53766792-53797273
chr18	53772875	53772997	chr18:53766792-53797273
chr18	53773000	53773409	chr18:53766792-53797273
chr18	53773410	53774660	chr18:53766792-53797273
chr18	53774665	53776182	chr18:53766792-53797273
chr18	53776190	53776800	chr18:53766792-53797273
chr18	53776860	53780287	chr18:53766792-53797273
chr18	53780310	53785408	chr18:53766792-53797273
chr18	53785410	53785683	chr18:53766792-53797273
chr18	53785715	53786260	chr18:53766792-53797273
chr18	53786270	53792123	chr18:53766792-53797273
chr18	53792205	53792304	chr18:53766792-53797273
chr18	53792485	53793295	chr18:53766792-53797273
chr18	53793315	53795844	chr18:53766792-53797273
chr18	53795865	53796079	chr18:53766792-53797273
chr18	53796150	53796493	chr18:53766792-53797273
chr18	53796755	53797250	chr18:53766792-53797273
chr18	53804515	53804863	chr18:53804536-53804845
chr18	53804950	53805096	chr18:53804984-53805056
chr18	53805800	53805974	chr18:53805821-53805946
chr18	53806990	53807093	chr18:53807018-53807058
chr18	53810075	53810459	chr18:53810102-53810452
chr18	53817050	53817333	chr18:53817084-53817316
chr18	53836345	53836719	chr18:53836379-53836710
chr18	53848555	53848724	chr18:53848579-53848705
chr18	53855485	53855622	chr18:53855508-53855581
chr18	53857960	53858523	chr18:53857994-53858493
chr20	3052235	3052453	chr20:3052265-3052421
chr20	3052700	3052940	chr20:3052722-3052924
chr20	3052985	3053195	chr20:3053008-3053163
chr20	3063180	3063481	chr20:3063201-3063448
chr20	3063600	3063841	chr20:3063622-3063824
chr20	3065175	3065400	chr20:3065200-3065370
chr20	3087525	3088762	chr20:3087558-3088217;chr20:3088218-3091099
chr20	3089040	3089323	chr20:3088218-3091099
chr20	3089595	3091134	chr20:3088218-3091099
chr20	3091255	3091373	chr20:3091277-3091363
chr20	3092255	3092435	chr20:3092289-3092398
chr20	3095925	3096150	chr20:3095950-3096112
chr20	3097110	3097390	chr20:3097134-3097369
chr20	3101995	3103268	chr20:3102029-3103230
chr20	3103935	3104070	chr20:3103957-3104052
chr20	3116625	3116838	chr20:3116648-3116804
chr20	3131160	3131538	chr20:3131183-3131513
chr20	3137305	3137474	chr20:3137329-3137436
chr20	3140380	3140715	chr20:3140411-3140842
chr20	16046195	16050745	chr20:16046227-16057739
chr20	16050765	16050943	chr20:16046227-16057739
chr20	16051525	16051602	chr20:16046227-16057739
chr20	16051740	16051830	chr20:16046227-16057739
chr20	16051910	16051989	chr20:16046227-16057739
chr20	16052715	16053645	chr20:16046227-16057739
chr20	16053710	16054030	chr20:16046227-16057739
chr20	16054040	16056599	chr20:16046227-16057739
chr20	16056925	16057750	chr20:16046227-16057739
chr20	16089685	16089961	chr20:16089707-16089934
chr20	16091220	16091325	chr20:16091243-16091308
chr20	22561606	22561973	chr20:22561641-22563792

chr20	22561981	22562106	chr20:22561641-22563792
chr20	22562156	22563832	chr20:22561641-22563792
chr20	22564806	22564972	chr20:22564829-22565101
chr20	22565021	22565113	chr20:22564829-22565101
chr20	22565891	22566114	chr20:22565912-22566101
chr20	47934997	47935170	chr20:47935022-47935138
chr20	47946072	47946221	chr20:47946095-47946199
chr20	47947197	47947282	chr20:47947240-47947267
chr20	47948457	47948590	chr20:47948482-47948561
chr20	47980382	47980584	chr20:47980413-47991529
chr20	47980607	47982751	chr20:47980413-47991529
chr20	47982782	47983389	chr20:47980413-47991529
chr20	47983397	47984038	chr20:47980413-47991529
chr20	47984337	47985013	chr20:47980413-47991529
chr20	47985037	47986853	chr20:47980413-47991529
chr20	47986862	47987019	chr20:47980413-47991529
chr20	47987022	47988494	chr20:47980413-47991529
chr20	47988507	47991563	chr20:47980413-47991529
chr20	48003377	48003544	chr20:48003399-48003535
chr20	48061527	48062094	chr20:48061560-48062059
chr20	48098427	48099202	chr20:48098450-48099184
chr20	48100427	48100535	chr20:48100478-48100490
chr21	38996503	38996777	chr21:38996524-38997786
chr21	38996778	38997382	chr21:38996524-38997786
chr21	38997388	38997823	chr21:38996524-38997786
chr21	39086488	39087459	chr21:39086513-39087434
chr21	39212938	39213039	chr21:39212959-39213011
chr21	39288163	39288297	chr21:39288186-39288741
chr21	39288298	39288760	chr21:39288186-39288741
chr21	43036599	43037266	chr21:43036634-43037448
chr22	39925067	39925665	chr22:39925097-39925628
chr22	39925797	39925940	chr22:39925824-39925922
chr22	39927177	39927719	chr22:39927211-39927689
chr22	39928377	39928545	chr22:39928399-39928528
chr22	39928667	39928866	chr22:39928691-39928860
chr22	39966732	39967013	chr22:39966757-39966993
chr22	39967067	39967182	chr22:39967109-39967129
chr22	39982663	39983227	chr22:39982697-39983196
chr22	39983698	39983768	chr22:39983747-39983756
chr22	39994128	39994288	chr22:39994155-39994267
chr22	39996498	39996673	chr22:39996524-39996658
chr22	40015293	40015440	chr22:40015314-40015412
chr22	40030538	40030762	chr22:40030569-40030729
chr22	40036843	40037210	chr22:40036871-40037187
chr22	40038778	40038923	chr22:40038801-40038894
chr22	40039418	40039564	chr22:40039442-40039517
chr22	40042548	40042891	chr22:40042573-40042886
chr22	40043798	40043970	chr22:40043826-40043951
chr22	40045473	40045975	chr22:40045505-40045934
chr22	40052373	40052477	chr22:40052422-40052424
chr22	40052668	40052746	chr22:40052712-40052714
chr22	40054133	40054339	chr22:40054156-40054308
chr22	40054913	40055166	chr22:40054935-40055121
chr22	40055403	40055587	chr22:40055437-40055555
chr22	40055673	40055886	chr22:40055701-40055857
chr22	40056318	40056468	chr22:40056348-40056442
chr22	40057083	40057334	chr22:40057112-40057315
chr22	40057938	40058462	chr22:40057969-40058440
chr22	40058758	40058892	chr22:40058780-40058881
chr22	40059698	40059876	chr22:40059722-40059846
chr22	40060068	40060177	chr22:40060095-40060164
chr22	40060718	40060954	chr22:40060743-40060928
chr22	40061468	40061657	chr22:40061502-40061629

chr22	40061863	40062036	chr22:40061885-40062011
chr22	40064273	40064423	chr22:40064296-40064386
chr22	40066008	40066270	chr22:40066042-40066235
chr22	40066773	40067000	chr22:40066807-40066959
chr22	40068173	40068353	chr22:40068203-40068313
chr22	40068928	40069102	chr22:40068953-40069087
chr22	40069943	40070077	chr22:40069967-40070038
chr22	40073318	40073462	chr22:40073345-40073424
chr22	40073968	40074136	chr22:40073991-40074113
chr22	40075088	40075480	chr22:40075111-40075450
chr22	40075703	40075912	chr22:40075726-40075873
chr22	40076913	40077085	chr22:40076934-40077057
chr22	40078243	40078694	chr22:40078283-40078297;chr22:40078308-40078666
chr22	40080273	40080781	chr22:40080306-40081216
chr22	40080788	40081244	chr22:40080306-40081216
chr22	40081733	40082619	chr22:40081765-40085742
chr22	40082698	40085769	chr22:40081765-40085742
chr22	50925182	50925367	chr22:50925212-50925341
chr22	50925782	50925926	chr22:50925813-50925894
chr22	50926062	50926207	chr22:50926090-50926171
chr22	50926287	50926500	chr22:50926314-50926477
chr22	50926677	50926775	chr22:50926703-50926771
chr22	50927437	50927616	chr22:50927468-50927578
chr22	50927632	50927738	chr22:50927656-50927724
chr22	50927792	50927899	chr22:50927825-50927875
chr22	50927927	50928108	chr22:50927960-50928073
chr22	50928152	50928749	chr22:50928176-50928750
chrX	5758683	5758957	chrX:5758677-5758932
chrX	5762448	5762552	chrX:5762474-5762542
chrX	5808033	5808275	chrX:5808066-5811707
chrX	5808313	5810080	chrX:5808066-5811707
chrX	5810108	5810765	chrX:5808066-5811707
chrX	5810783	5811722	chrX:5808066-5811707
chrX	5821083	5821941	chrX:5821117-5821907
chrX	5827063	5827311	chrX:5827094-5827280
chrX	5947298	5947503	chrX:5947320-5947473
chrX	5950703	5950809	chrX:5950731-5950791
chrX	5970153	5970312	chrX:5970185-5970259
chrX	6002983	6003551	chrX:6003016-6003515
chrX	6069010	6069848	chrX:6069035-6069812
chrX	6104785	6105030	chrX:6104812-6105079
chrX	6105490	6105851	chrX:6105523-6105830
chrX	6144645	6145269	chrX:6144675-6145244
chrX	6145705	6145910	chrX:6145729-6145888
chrX	6146550	6147119	chrX:6146581-6147086
chrX	21277634	21278033	chrX:21277666-21278006
chrX	21318959	21319098	chrX:21318984-21319063
chrX	21392079	21392258	chrX:21392100-21392237
chrX	21392509	21392724	chrX:21392535-21393079
chrX	21392764	21393108	chrX:21392535-21393079
chrX	21424669	21425228	chrX:21424697-21425196
chrX	21444589	21444807	chrX:21444614-21444778
chrX	21450704	21450960	chrX:21450729-21450932
chrX	21458779	21458925	chrX:21458811-21458899
chrX	21488859	21489030	chrX:21488883-21488996
chrX	21508554	21508727	chrX:21508576-21508696
chrX	21515879	21515979	chrX:21515904-21515964
chrX	21519614	21519721	chrX:21519637-21519706
chrX	21534579	21534796	chrX:21534602-21534749
chrX	21536594	21536693	chrX:21536634-21536646
chrX	21544959	21545139	chrX:21544984-21545118
chrX	21549944	21550219	chrX:21549973-21550185
chrX	21579554	21579710	chrX:21579588-21579678

chrX	21581329	21581609	chrX:21581355-21581570
chrX	21608654	21608765	chrX:21608689-21608738
chrX	21609114	21609325	chrX:21609139-21609312
chrX	21613069	21613200	chrX:21613091-21613165
chrX	21613419	21613553	chrX:21613441-21613513
chrX	21619374	21619478	chrX:21619399-21619467
chrX	21624864	21625015	chrX:21624896-21624997
chrX	21627159	21627668	chrX:21627188-21627735
chrX	21627694	21627763	chrX:21627188-21627735
chrX	21659629	21660826	chrX:21659650-21660980
chrX	21660829	21661008	chrX:21659650-21660980
chrX	21666934	21667179	chrX:21666948-21667145
chrX	21670399	21671475	chrX:21670423-21672813
chrX	21671479	21672396	chrX:21670423-21672813
chrX	21672419	21672837	chrX:21670423-21672813
chrX	68377430	68379070	chrX:68377126-68379036
chrX	68399360	68399511	chrX:68399388-68399488
chrX	68421295	68421513	chrX:68421319-68421475
chrX	68424145	68424326	chrX:68424174-68424292
chrX	68428355	68429540	chrX:68428094-68429859
chrX	68429595	68429871	chrX:68428094-68429859
