# Supplementary material for: Targeted RNA sequencing enhances gene expression profiling of ultra-low input samples
Source: RNA Biol. 2020 Jun 28;17(12):1741–53. doi: 10.1080/15476286.2020.1777768 (PMC7746246; doi:10.1080/15476286.2020.1777768)
Supplement: Supplemental Material [file KRNB_A_1777768_SM6200.zip › TableS10_isoforms_summary.pdf]

| agg_code | study | Capture Panel | Pre-capture number of isoforms (median) | Post-capture number of isoforms (median) | GffCompare class_code | Enrichment Factor |
|---|---|---|---|---|---|---|
| known.isoforms | Bulks | NF Capture | 114 | 167 | (= + c) | 1.46 |
| known.isoforms | Mini-bulks | NF Capture | 18.5 | 100.5 | (= + c) | 5.43 |
| novel | Bulks | NF Capture | 64.5 | 129 | j | 2.00 |
| novel | Mini-bulks | NF Capture | 1 | 83.5 | j | 83.50 |
| | | | | | | |
| = | Bulks | NF Capture | 99.5 | 160 | = | 1.61 |
| = | Mini-bulks | NF Capture | 7.5 | 74 | = | 9.87 |
| c | Bulks | NF Capture | 15 | 8 | c | 0.53 |
| c | Mini-bulks | NF Capture | 11 | 28 | c | 2.55 |
| | | | | | | |
| known.isoforms | Bulks | TF Capture | 1065.5 | 1348 | (= + c) | 1.27 |
| known.isoforms | Mini-bulks | TF Capture 850 | 87 | 776 | (= + c) | 8.92 |
| known.isoforms | Mini-bulks | TF Capture 150 | 87 | 771 | (= + c) | 8.86 |
| novel | Bulks | TF Capture | 394 | 727.5 | j | 1.85 |
| novel | Mini-bulks | TF Capture 850 | 3 | 352.5 | j | 117.50 |
| novel | Mini-bulks | TF Capture 150 | 3 | 313 | j | 104.33 |
| | | | | | | |
| = | Bulks | TF Capture | 997 | 1262 | = | 1.27 |
| = | Mini-bulks | TF Capture 850 | 47 | 550.5 | = | 11.71 |
| = | Mini-bulks | TF Capture 150 | 47 | 535.5 | = | 11.39 |
| c | Bulks | Pre-TFCapture | 64.5 | 85.5 | c | 1.33 |
| c | Mini-bulks | TF Capture 850 | 37.5 | 229.5 | c | 6.12 |
| c | Mini-bulks | TF Capture 150 | 37.5 | 231.5 | c | 6.17 |
