# Supplementary material for: Targeted RNA sequencing enhances gene expression profiling of ultra-low input samples
Source: RNA Biol. 2020 Jun 28;17(12):1741–53. doi: 10.1080/15476286.2020.1777768 (PMC7746246; doi:10.1080/15476286.2020.1777768)
Supplement: Supplemental Material [file KRNB_A_1777768_SM6200.zip › TableS2_NG_Capture_ERCC_targets.pdf]

FRAC_PROBE_COVERAGE is fraction of spike-in directly targeted by a probe.

FRAC_ESTIMATED_PROBE_COVERAGE is fraction of spike-in estimated to be directly and indirectly targeted by probes

Spike_in_target	FRAC_PROBE_COVERAGE	FRAC_ESTIMATED_PROBE_COVERAGE
ERCC_00004	1	1
ERCC_00012	0.99	1
ERCC_00014	0.99	1
ERCC_00019	1	1
ERCC_00022	1	1
ERCC_00024	0.98	1
ERCC_00028	1	1
ERCC_00031	0.99	1
ERCC_00034	1	1
ERCC_00035	0.99	1
ERCC_00041	1	1
ERCC_00042	0.99	1
ERCC_00043	0.99	1
ERCC_00044	1	1
ERCC_00046	1	1
ERCC_00048	1	1
ERCC_00051	0.97	1
ERCC_00057	0.99	1
ERCC_00058	0.99	1
ERCC_00059	1	1
ERCC_00069	0.99	1
ERCC_00071	0.98	1
ERCC_00078	1	1
ERCC_00083	1	1
ERCC_00084	0.99	1
ERCC_00085	0.98	1
ERCC_00086	1	1
ERCC_00092	0.99	1
ERCC_00098	1	1
ERCC_00099	0.99	1
ERCC_00104	1	1
ERCC_00108	0.99	1
ERCC_00109	0.98	1
ERCC_00111	1	1
ERCC_00117	1	1
ERCC_00120	0.97	1
ERCC_00123	1	1
ERCC_00131	1	1
ERCC_00134	1	1
ERCC_00137	1	1
ERCC_00138	1	1
ERCC_00143	0.98	1
ERCC_00144	0.99	1
ERCC_00145	1	1
ERCC_00147	0.94	1

ERCC_00148	1	1
ERCC_00150	1	1
ERCC_00156	0.99	1
ERCC_00157	1	1
ERCC_00158	1	1
ERCC_00160	0.98	1
ERCC_00164	0.98	1
ERCC_00165	1	1
ERCC_00168	1	1
ERCC_00170	0.99	1
ERCC_00171	0.99	1
