# Supplementary material for: Targeted RNA sequencing enhances gene expression profiling of ultra-low input samples
Source: RNA Biol. 2020 Jun 28;17(12):1741–53. doi: 10.1080/15476286.2020.1777768 (PMC7746246; doi:10.1080/15476286.2020.1777768)
Supplement: Supplemental Material [file KRNB_A_1777768_SM6200.zip › TableS3_TF_capture_targets_hg38.pdf]

Chromosome	Start_coordinate	End_coordinate	Original_target_region
chr1	998968	999457	chr1:998961-999432
chr1	999528	999642	chr1:999525-999613
chr1	999693	1000182	chr1:999691-1000172
chr1	2528757	2529520	chr1:2528744-2529749
chr1	2529522	2529761	chr1:2528744-2529749
chr1	2529847	2530035	chr1:2529845-2530011
chr1	2530112	2530253	chr1:2530110-2530245
chr1	3069175	3069316	chr1:3069167-3069296
chr1	3080210	3080411	chr1:3080201-3080401
chr1	3186125	3186484	chr1:3186124-3186474
chr1	3237940	3238117	chr1:3237930-3238095
chr1	3244090	3244169	chr1:3244086-3244137
chr1	3385150	3385303	chr1:3385148-3385286
chr1	3386675	3386938	chr1:3386675-3386918
chr1	3396490	3396602	chr1:3396490-3396593
chr1	3402800	3403011	chr1:3402790-3402998
chr1	3404750	3404916	chr1:3404738-3404886
chr1	3405495	3405685	chr1:3405494-3405648
chr1	3411395	3412839	chr1:3411383-3412800
chr1	3414560	3414668	chr1:3414559-3414647
chr1	3417830	3418021	chr1:3417827-3417997
chr1	3418670	3418780	chr1:3418666-3418744
chr1	3424930	3425075	chr1:3424918-3425059
chr1	3425580	3425762	chr1:3425580-3425750
chr1	3426055	3426229	chr1:3426050-3426225
chr1	3430875	3431135	chr1:3430871-3431108
chr1	3431965	3432149	chr1:3431965-3432140
chr1	3433680	3434309	chr1:3433676-3438621
chr1	3434330	3435563	chr1:3433676-3438621
chr1	3435565	3436194	chr1:3433676-3438621
chr1	3436205	3437705	chr1:3433676-3438621
chr1	3437765	3438037	chr1:3433676-3438621
chr1	3438040	3438648	chr1:3433676-3438621
chr1	3652530	3652600	chr1:3652519-3652562
chr1	3682345	3682449	chr1:3682332-3682430
chr1	3683065	3683211	chr1:3683059-3683180
chr1	3688910	3689127	chr1:3688892-3689092
chr1	3690675	3690972	chr1:3690671-3690944
chr1	3698070	3698181	chr1:3698057-3698151
chr1	3707550	3707808	chr1:3707548-3707791
chr1	3722025	3722231	chr1:3722020-3722207
chr1	3723365	3723506	chr1:3723353-3723469
chr1	3727120	3727262	chr1:3727114-3727224
chr1	3727635	3727808	chr1:3727627-3727770
chr1	3728140	3728250	chr1:3728128-3728217
chr1	3729340	3729487	chr1:3729326-3729448
chr1	3730000	3730182	chr1:3729999-3730148
chr1	3730930	3731072	chr1:3730926-3731065
chr1	3731465	3731570	chr1:3731462-3731556
chr1	3732755	3733919	chr1:3732746-3736201
chr1	3733935	3736218	chr1:3732746-3736201
chr1	6065925	6066136	chr1:6065917-6066117
chr1	6085205	6085278	chr1:6085203-6085248
chr1	6096660	6096774	chr1:6096656-6096756
chr1	6097280	6097454	chr1:6097268-6097418
chr1	6098490	6098667	chr1:6098484-6098634
chr1	6099830	6100004	chr1:6099822-6099972
chr1	6105290	6105460	chr1:6105277-6105427
chr1	6106240	6106317	chr1:6106233-6106287
chr1	6108215	6108420	chr1:6108187-6108387
chr1	6244195	6244264	chr1:6244191-6244241
chr1	6244350	6244467	chr1:6244350-6244446

chr1	6244560	6244658	chr1:6244547-6244629
chr1	6245120	6245605	chr1:6245109-6245578
chr1	6412715	6413327	chr1:6412417-6413282
chr1	6415275	6415788	chr1:6415231-6419340
chr1	6416075	6417585	chr1:6415231-6419340
chr1	6417875	6419382	chr1:6415231-6419340
chr1	6419615	6419730	chr1:6419606-6419702
chr1	6419775	6419951	chr1:6419775-6419919
chr1	6423380	6423595	chr1:6423380-6423580
chr1	6424490	6424664	chr1:6424477-6424637
chr1	6785335	6785596	chr1:6785323-6785575
chr1	6806990	6807113	chr1:6806982-6807088
chr1	6820190	6820263	chr1:6820180-6820250
chr1	6825095	6825233	chr1:6825091-6825210
chr1	6853060	6853196	chr1:6853047-6853189
chr1	6871765	6871864	chr1:6871756-6872053
chr1	6871870	6872072	chr1:6871756-6872053
chr1	6874055	6874454	chr1:6874055-6874423
chr1	6886250	6886380	chr1:6886239-6886346
chr1	6887655	6888236	chr1:6887653-6888201
chr1	6908505	6908652	chr1:6908504-6908704
chr1	6908665	6908740	chr1:6908504-6908704
chr1	7091315	7091386	chr1:7091303-7091371
chr1	7249490	7249641	chr1:7249490-7249626
chr1	7467830	7467938	chr1:7467829-7467901
chr1	7640405	7640581	chr1:7640399-7640553
chr1	7661735	7661902	chr1:7661725-7661866
chr1	7663355	7665214	chr1:7663352-7665199
chr1	7670915	7671061	chr1:7670910-7671037
chr1	7677600	7677742	chr1:7677598-7677733
chr1	7732455	7732641	chr1:7732447-7732599
chr1	7736355	7736564	chr1:7736343-7736540
chr1	7736930	7737035	chr1:7736930-7737009
chr1	7737265	7737616	chr1:7737254-7737570
chr1	7737965	7738503	chr1:7737958-7738482
chr1	7744835	7745051	chr1:7744834-7745022
chr1	7745850	7746126	chr1:7745844-7746091
chr1	7747710	7747818	chr1:7747709-7747781
chr1	7751200	7751421	chr1:7751198-7751392
chr1	7752470	7752569	chr1:7752458-7752533
chr1	7755640	7755715	chr1:7755637-7755668
chr1	7766465	7766583	chr1:7766458-7769706
chr1	7766600	7767050	chr1:7766458-7769706
chr1	7767065	7767696	chr1:7766458-7769706
chr1	7767715	7767834	chr1:7766458-7769706
chr1	7767905	7767967	chr1:7766458-7769706
chr1	7767970	7768109	chr1:7766458-7769706
chr1	7768130	7769738	chr1:7766458-7769706
chr1	8861287	8861464	chr1:8861279-8861429
chr1	8862897	8862973	chr1:8862886-8862945
chr1	10636675	10636844	chr1:10636603-10640059
chr1	10636930	10637113	chr1:10636603-10640059
chr1	10637175	10637812	chr1:10636603-10640059
chr1	10637825	10639103	chr1:10636603-10640059
chr1	10639125	10640093	chr1:10636603-10640059
chr1	10642095	10642278	chr1:10642081-10642322
chr1	10642865	10643040	chr1:10642858-10643000
chr1	10643170	10643343	chr1:10643159-10643311
chr1	10644925	10645108	chr1:10644916-10645088
chr1	10646130	10646345	chr1:10646127-10646326
chr1	10647225	10648169	chr1:10647212-10648139
chr1	10649070	10649224	chr1:10649069-10649192
chr1	10649285	10649481	chr1:10649282-10649437

chr1	10650695	10650772	chr1:10650691-10650755
chr1	10650950	10651081	chr1:10650940-10651076
chr1	10653385	10654233	chr1:10653376-10654218
chr1	10654420	10654602	chr1:10654418-10654591
chr1	10655660	10655837	chr1:10655648-10655813
chr1	10656645	10656756	chr1:10656645-10656736
chr1	10658515	10658591	chr1:10658507-10658576
chr1	10659710	10660552	chr1:10659701-10660536
chr1	10660750	10660892	chr1:10660736-10660856
chr1	10665095	10665582	chr1:10665082-10665571
chr1	10693880	10693951	chr1:10693873-10693912
chr1	10697685	10697895	chr1:10697673-10697873
chr1	10704195	10704575	chr1:10704195-10704562
chr1	10705505	10705614	chr1:10705491-10705575
chr1	10760710	10760885	chr1:10760700-10760857
chr1	10796585	10796690	chr1:10796563-10796650
chr1	11020508	11020634	chr1:11020499-11020599
chr1	11022143	11022222	chr1:11022123-11022273
chr1	11022358	11022446	chr1:11022352-11022502
chr1	11023278	11023376	chr1:11023192-11023342
chr1	13700254	13700326	chr1:13700197-13700300
chr1	13704859	13705665	chr1:13704854-13705645
chr1	13707969	13708178	chr1:13707964-13708164
chr1	13715544	13715652	chr1:13715540-13715614
chr1	13731004	13731156	chr1:13730999-13731117
chr1	13732779	13732890	chr1:13732778-13732882
chr1	13738759	13738972	chr1:13738749-13738940
chr1	13742004	13742183	chr1:13742004-13742157
chr1	13749369	13749504	chr1:13749360-13749487
chr1	13769049	13769186	chr1:13769038-13769173
chr1	13771739	13772232	chr1:13771725-13772214
chr1	13773089	13773232	chr1:13773077-13773188
chr1	13778419	13778602	chr1:13778417-13782831
chr1	13778644	13780879	chr1:13778417-13782831
chr1	13780884	13780956	chr1:13778417-13782831
chr1	13780974	13781049	chr1:13778417-13782831
chr1	13781054	13782695	chr1:13778417-13782831
chr1	13782709	13782851	chr1:13778417-13782831
chr1	13786539	13787055	chr1:13786514-13788079
chr1	13787059	13788107	chr1:13786514-13788079
chr1	13816429	13816612	chr1:13816426-13816570
chr1	13823164	13824007	chr1:13823158-13825079
chr1	13824019	13825102	chr1:13823158-13825079
chr1	15941879	15942265	chr1:15941868-15942252
chr1	15942334	15942757	chr1:15942330-15942738
chr1	15942764	15942938	chr1:15942755-15942901
chr1	15943074	15943243	chr1:15943063-15943519
chr1	15943244	15943556	chr1:15943063-15943519
chr1	15943599	15944628	chr1:15943598-15944600
chr1	15944699	15944877	chr1:15944696-15944839
chr1	15944939	15945230	chr1:15944936-15945202
chr1	15945714	15946350	chr1:15945714-15946316
chr1	15946944	15947316	chr1:15946934-15947123;chr1:15947156-15947292
chr1	15948299	15948522	chr1:15948290-15948497
chr1	15949644	15949857	chr1:15949634-15949834
chr1	15952914	15952989	chr1:15952909-15952973
chr1	15973049	15973150	chr1:15973038-15973125
chr1	15975984	15976169	chr1:15975982-15976132
chr1	16974883	16975057	chr1:16974873-16975023
chr1	16975268	16975377	chr1:16975268-16975342
chr1	18631018	18631092	chr1:18631005-18631688
chr1	18631093	18631433	chr1:18631005-18631688
chr1	18631453	18631722	chr1:18631005-18631688

chr1	18634313	18634559	chr1:18634302-18634538
chr1	18635118	18635267	chr1:18635110-18635240
chr1	18636238	18636379	chr1:18636236-18636371
chr1	18640133	18640324	chr1:18640103-18640303
chr1	18691763	18691976	chr1:18691753-18691953
chr1	18700653	18700830	chr1:18700652-18700818
chr1	18703103	18703309	chr1:18703093-18703296
chr1	18735633	18736162	chr1:18735631-18736138
chr1	18744818	18747798	chr1:18744813-18748866
chr1	18747823	18747988	chr1:18744813-18748866
chr1	18747993	18748899	chr1:18744813-18748866
chr1	18871648	18871830	chr1:18871640-18871790
chr1	18872803	18872981	chr1:18872803-18872957
chr1	18874473	18874586	chr1:18874462-18874562
chr1	18879973	18880184	chr1:18879972-18880172
chr1	23506434	23507240	chr1:23506429-23510148
chr1	23507539	23510168	chr1:23506429-23510148
chr1	23516339	23516550	chr1:23516334-23516527
chr1	23519029	23519423	chr1:23519015-23519396
chr1	23520914	23521104	chr1:23520912-23521071
chr1	23521844	23522084	chr1:23521836-23522056
chr1	23524394	23524498	chr1:23524382-23524488
chr1	23525924	23526141	chr1:23525920-23526120
chr1	23530544	23531252	chr1:23530541-23531220
chr1	24319322	24319777	chr1:24319321-24319747
chr1	24319817	24319970	chr1:24319813-24319941
chr1	24323042	24323152	chr1:24323039-24323117
chr1	24331427	24331652	chr1:24331425-24331612
chr1	24332667	24332870	chr1:24332656-24332856
chr1	24334647	24334722	chr1:24334644-24334706
chr1	24336482	24336843	chr1:24336481-24336827
chr1	24337087	24337181	chr1:24337077-24337151
chr1	24337637	24337827	chr1:24337635-24337789
chr1	24337997	24338138	chr1:24337991-24338103
chr1	24339677	24339781	chr1:24339667-24339762
chr1	24342127	24342306	chr1:24342114-24342273
chr1	24342702	24342806	chr1:24342693-24342772
chr1	24342902	24343042	chr1:24342891-24343025
chr1	24344902	24344977	chr1:24344896-24344931
chr1	24346562	24346666	chr1:24346552-24346641
chr1	24347467	24347577	chr1:24347467-24347553
chr1	24350062	24350138	chr1:24350057-24350122
chr1	24354387	24355117	chr1:24354373-24355318
chr1	24355147	24355360	chr1:24354373-24355318
chr1	24364197	24364517	chr1:24364184-24364482
chr1	24899510	24901010	chr1:24899510-24902666
chr1	24901050	24902686	chr1:24899510-24902666
chr1	24907270	24907443	chr1:24907258-24907417
chr1	24919240	24919347	chr1:24919239-24919344
chr1	24927580	24927757	chr1:24927573-24927730
chr1	24928745	24928815	chr1:24928742-24928798
chr1	24929590	24929776	chr1:24929586-24929877
chr1	24929800	24929903	chr1:24929586-24929877
chr1	24930830	24931049	chr1:24930823-24931023
chr1	24964525	24964706	chr1:24964513-24965010
chr1	24964800	24965139	chr1:24964513-24965010;chr1:24965019-24965121
chr1	28668744	28668872	chr1:28668731-28668839
chr1	28669604	28669819	chr1:28669596-28669796
chr1	28683564	28683778	chr1:28683563-28683740
chr1	28690079	28690184	chr1:28690073-28690186
chr1	28691584	28691728	chr1:28691584-28691709
chr1	28692944	28693070	chr1:28692941-28693045
chr1	28696934	28697117	chr1:28696926-28697084

chr1	28702439	28702593	chr1:28702437-28702569
chr1	28704204	28704353	chr1:28704191-28704329
chr1	28710524	28710669	chr1:28710519-28710642
chr1	28714074	28714814	chr1:28714072-28719353
chr1	28714889	28715311	chr1:28714072-28719353
chr1	28715614	28715821	chr1:28714072-28719353
chr1	28716104	28716758	chr1:28714072-28719353
chr1	28716764	28717029	chr1:28714072-28719353
chr1	28717039	28717176	chr1:28714072-28719353
chr1	28717479	28717554	chr1:28714072-28719353
chr1	28717569	28718307	chr1:28714072-28719353
chr1	28718564	28718636	chr1:28714072-28719353
chr1	28718649	28719389	chr1:28714072-28719353
chr1	31908820	31908953	chr1:31908810-31908960
chr1	31910040	31910154	chr1:31910037-31910112
chr1	31915905	31916000	chr1:31915894-31915987
chr1	31936840	31937044	chr1:31936829-31937029
chr1	35573381	35573633	chr1:35573369-35573604
chr1	35573936	35574421	chr1:35573926-35574409
chr1	35574951	35575027	chr1:35574948-35575000
chr1	35588341	35588576	chr1:35588329-35588552
chr1	35589931	35590088	chr1:35589929-35590048
chr1	35590646	35590791	chr1:35590633-35590775
chr1	35591336	35591552	chr1:35591331-35591531
chr1	35594406	35595361	chr1:35594393-35595328
chr1	37809608	37811496	chr1:37809604-37815566
chr1	37811503	37812994	chr1:37809604-37815566
chr1	37813283	37813662	chr1:37809604-37815566
chr1	37813663	37813834	chr1:37809604-37815566
chr1	37813863	37813990	chr1:37809604-37815566
chr1	37814003	37814919	chr1:37809604-37815566
chr1	37814948	37815597	chr1:37809604-37815566
chr1	37817418	37817493	chr1:37817418-37817482
chr1	37822128	37822727	chr1:37822120-37822716
chr1	37823718	37823829	chr1:37823709-37823812
chr1	37832248	37832356	chr1:37832244-37832322
chr1	37835078	37835227	chr1:37835078-37835215
chr1	37835673	37835785	chr1:37835670-37835744
chr1	37838638	37838775	chr1:37838624-37838756
chr1	37839923	37840183	chr1:37839919-37840158
chr1	37848698	37848906	chr1:37848684-37848884
chr1	37857253	37857750	chr1:37857250-37857709
chr1	37858488	37858565	chr1:37858487-37858539
chr1	37859543	37859646	chr1:37859530-37859620
chr1	38044623	38045278	chr1:38044610-38046794
chr1	38045283	38045417	chr1:38044610-38046794
chr1	38045418	38046641	chr1:38044610-38046794
chr1	38046653	38046748	chr1:38044610-38046794
chr1	39624157	39624516	chr1:39624152-39627180
chr1	39624537	39627202	chr1:39624152-39627180
chr1	39630227	39630338	chr1:39630226-39630308
chr1	39631507	39631611	chr1:39631495-39631579
chr1	39632652	39632730	chr1:39632648-39632715
chr1	39639557	39639970	chr1:39639545-39639945
chr1	39895452	39895614	chr1:39895425-39897970
chr1	39895622	39895824	chr1:39895425-39897970
chr1	39895832	39896778	chr1:39895425-39897970
chr1	39896782	39898007	chr1:39895425-39897970
chr1	39899622	39902041	chr1:39899611-39902013
chr1	39902152	39902293	chr1:39902140-39902256
chr1	40477227	40477931	chr1:40477214-40477894
chr1	40479047	40479492	chr1:40479035-40479488
chr1	40480617	40480690	chr1:40480609-40480809

chr1	40480712	40480787	chr1:40480609-40480809
chr1	40481772	40481876	chr1:40481762-40481854
chr1	40489092	40489243	chr1:40489087-40489214
chr1	40489537	40489643	chr1:40489528-40489624
chr1	40490792	40491180	chr1:40490779-40491156
chr1	40494932	40495351	chr1:40494920-40496343
chr1	40495362	40495794	chr1:40494920-40496343
chr1	40495802	40496368	chr1:40494920-40496343
chr1	40691697	40691912	chr1:40691688-40691867
chr1	40692007	40692226	chr1:40692007-40692200
chr1	40692297	40692366	chr1:40692289-40692347
chr1	40695707	40695777	chr1:40695605-40695745
chr1	40709362	40709634	chr1:40709356-40709616
chr1	40712407	40712512	chr1:40712397-40712469
chr1	40723212	40723289	chr1:40723207-40723274
chr1	40734857	40735060	chr1:40734847-40735033
chr1	40735327	40735777	chr1:40735314-40735750
chr1	40738847	40738978	chr1:40738833-40738948
chr1	40747537	40747614	chr1:40747533-40747605
chr1	40749572	40749718	chr1:40749572-40749686
chr1	40753157	40753272	chr1:40753150-40753246
chr1	40755602	40755820	chr1:40755591-40755791
chr1	40758127	40758329	chr1:40758120-40758294
chr1	40762887	40763086	chr1:40762887-40763046
chr1	40766097	40766249	chr1:40766089-40766231
chr1	40766602	40766750	chr1:40766595-40766703
chr1	40766922	40766996	chr1:40766917-40766974
chr1	40769357	40769437	chr1:40769355-40769415
chr1	40770397	40771156	chr1:40770396-40771603
chr1	40771177	40771617	chr1:40770396-40771603
chr1	41361927	41362367	chr1:41361921-41362344
chr1	41373952	41374179	chr1:41373949-41374149
chr1	41381627	41383615	chr1:41381615-41383590
chr1	41506407	41506585	chr1:41506364-41511266
chr1	41506622	41506903	chr1:41506364-41511266
chr1	41506912	41509813	chr1:41506364-41511266
chr1	41509842	41510223	chr1:41506364-41511266
chr1	41510252	41511298	chr1:41506364-41511266
chr1	41512822	41513770	chr1:41512815-41513750
chr1	41518412	41518516	chr1:41518401-41518488
chr1	41524737	41524929	chr1:41524734-41524910
chr1	41575547	41575724	chr1:41575543-41575689
chr1	41579737	41580311	chr1:41579736-41585318
chr1	41580317	41580990	chr1:41579736-41585318
chr1	41581007	41585347	chr1:41579736-41585318
chr1	41628752	41628982	chr1:41628748-41628947
chr1	41700917	41701032	chr1:41700915-41700995
chr1	41847202	41848474	chr1:41847190-41848445
chr1	41848872	41849124	chr1:41848870-41849089
chr1	41861732	41861939	chr1:41861726-41861926
chr1	41864587	41864696	chr1:41864577-41864648
chr1	41865547	41865624	chr1:41865547-41865615
chr1	41869547	41869653	chr1:41869546-41869630
chr1	41918412	41918746	chr1:41918412-41918707
chr1	42035817	42035952	chr1:42035806-42035925
chr1	42176547	42177740	chr1:42176538-42179825
chr1	42177812	42178012	chr1:42176538-42179825
chr1	42178017	42178513	chr1:42176538-42179825
chr1	42178802	42179867	chr1:42176538-42179825
chr1	42181922	42182059	chr1:42181916-42182024
chr1	42188747	42189829	chr1:42188736-42189809
chr1	42191307	42191729	chr1:42191302-42191719
chr1	42194892	42195076	chr1:42194889-42195064

chr1	42199122	42199240	chr1:42199101-42199230
chr1	42205762	42206047	chr1:42205761-42206011
chr1	42227882	42227990	chr1:42227882-42227966
chr1	42264932	42265220	chr1:42264930-42265189
chr1	42278347	42278705	chr1:42278347-42278672
chr1	42311062	42311140	chr1:42311049-42311110
chr1	42315357	42315488	chr1:42315345-42315459
chr1	42320332	42320526	chr1:42320319-42320519
chr1	42323687	42323867	chr1:42323683-42323827
chr1	42334927	42335005	chr1:42334924-42334965
chr1	42335072	42335251	chr1:42335058-42335232
chr1	42335637	42335912	chr1:42335625-42335877
chr1	42547177	42547398	chr1:42547165-42547365
chr1	42653387	42653461	chr1:42653375-42653443
chr1	42653822	42654013	chr1:42653817-42653967
chr1	42690717	42690933	chr1:42690717-42690917
chr1	42701007	42701087	chr1:42700946-42701046
chr1	42702062	42702136	chr1:42701980-42702130
chr1	43997617	43997755	chr1:43997589-43997739
chr1	43997857	43997969	chr1:43997854-43997954
chr1	44000767	44001097	chr1:44000766-44001055
chr1	44013287	44013499	chr1:44013287-44013487
chr1	44118862	44119005	chr1:44118849-44118988
chr1	44129352	44130232	chr1:44129352-44130196
chr1	44130522	44130775	chr1:44130511-44130756
chr1	44133247	44134262	chr1:44133237-44135140
chr1	44134517	44135024	chr1:44133237-44135140
chr1	46507002	46507188	chr1:46506995-46507164
chr1	46508287	46508504	chr1:46508273-46508473
chr1	46510462	46510688	chr1:46510460-46510654
chr1	46510937	46511295	chr1:46510934-46511283
chr1	46512052	46512860	chr1:46512042-46514226
chr1	46512862	46513941	chr1:46512042-46514226
chr1	47216343	47216796	chr1:47216289-47220174
chr1	47216798	47217111	chr1:47216289-47220174
chr1	47217408	47220192	chr1:47216289-47220174
chr1	47221203	47221432	chr1:47221191-47221391
chr1	47223383	47223632	chr1:47223383-47223619
chr1	47224003	47224110	chr1:47224003-47224098
chr1	47225448	47225906	chr1:47225442-47225889
chr1	47228358	47228543	chr1:47228346-47228522
chr1	47229203	47229411	chr1:47229195-47229771
chr1	47229428	47229803	chr1:47229195-47229771
chr1	47231058	47231224	chr1:47231046-47231218
chr1	47231668	47231743	chr1:47231656-47231715
chr1	47232168	47232235	chr1:47232166-47232220
chr1	47416083	47416492	chr1:47416071-47418052
chr1	47416528	47416709	chr1:47416071-47418052
chr1	47416733	47418004	chr1:47416071-47418052
chr1	47436023	47438530	chr1:47436016-47440691
chr1	47438553	47438632	chr1:47436016-47440691
chr1	47438783	47440107	chr1:47436016-47440691
chr1	47440123	47440731	chr1:47436016-47440691
chr1	52345733	52345936	chr1:52345722-52345909
chr1	52349303	52349521	chr1:52349299-52349499
chr1	52350603	52350747	chr1:52350592-52353647
chr1	52351033	52351607	chr1:52350592-52353647
chr1	52351893	52352547	chr1:52350592-52353647
chr1	52352553	52353289	chr1:52350592-52353647
chr1	52353313	52353666	chr1:52350592-52353647
chr1	52354613	52354727	chr1:52354607-52354698
chr1	52354848	52354948	chr1:52354839-52354939
chr1	52355403	52355477	chr1:52355397-52355449

chr1	52355608	52355679	chr1:52355607-52355666
chr1	52355783	52355888	chr1:52355770-52355844
chr1	52356188	52356341	chr1:52356185-52356302
chr1	52356388	52356469	chr1:52356383-52356442
chr1	52356768	52356840	chr1:52356754-52356813
chr1	52357013	52357189	chr1:52357000-52357174
chr1	52357528	52357724	chr1:52357525-52357698
chr1	52357793	52357927	chr1:52357780-52357916
chr1	52358338	52358485	chr1:52358330-52358461
chr1	52358693	52358773	chr1:52358685-52358758
chr1	52359028	52359150	chr1:52359026-52359157
chr1	52359258	52359376	chr1:52359249-52359357
chr1	52359468	52359572	chr1:52359458-52359534
chr1	52359708	52359921	chr1:52359704-52359883
chr1	52360073	52360259	chr1:52360073-52360233
chr1	52360433	52360569	chr1:52360423-52360549
chr1	52360983	52361164	chr1:52360973-52361132
chr1	52361553	52361653	chr1:52361512-52361616
chr1	52362608	52362788	chr1:52362601-52362746
chr1	52364563	52364680	chr1:52364551-52364634
chr1	52366068	52366227	chr1:52366068-52366193
chr1	53506263	53506794	chr1:53506236-53506776
chr1	53509128	53509307	chr1:53509119-53509287
chr1	53509848	53510062	chr1:53509848-53510027
chr1	53514633	53514819	chr1:53514624-53514781
chr1	53520643	53520789	chr1:53520633-53520766
chr1	53524778	53524924	chr1:53524776-53524887
chr1	53529793	53529981	chr1:53529790-53529952
chr1	53594108	53595026	chr1:53594107-53594990
chr1	53600113	53600318	chr1:53600100-53600278
chr1	53730243	53730461	chr1:53730243-53730443
chr1	53733903	53734054	chr1:53733903-53734204
chr1	53734078	53734215	chr1:53733903-53734204
chr1	53737813	53738129	chr1:53737805-53738106
chr1	58780799	58781542	chr1:58780787-58784327
chr1	58781554	58782436	chr1:58780787-58784327
chr1	58782459	58782637	chr1:58780787-58784327
chr1	58782644	58784348	chr1:58780787-58784327
chr1	61242835	61243055	chr1:61242833-61243033
chr1	61406630	61406745	chr1:61406627-61406727
chr1	61426465	61426579	chr1:61426464-61426556
chr1	61455305	61455490	chr1:61455302-61455452
chr1	63323054	63323501	chr1:63323040-63325126
chr1	63323504	63323606	chr1:63323040-63325126
chr1	63323664	63324867	chr1:63323040-63325126
chr1	63324879	63325109	chr1:63323040-63325126
chr1	77944057	77944299	chr1:77944054-77944269
chr1	77945512	77945931	chr1:77945505-77945895
chr1	77946492	77947785	chr1:77946481-77948333
chr1	77947792	77948353	chr1:77946481-77948333
chr1	77948407	77948623	chr1:77948404-77948914
chr1	77948637	77948918	chr1:77948404-77948914
chr1	77948997	77949309	chr1:77948969-77949300
chr1	77955257	77955333	chr1:77955254-77955458
chr1	77955347	77955494	chr1:77955254-77955458
chr1	77956577	77956717	chr1:77956571-77956700
chr1	77960197	77960294	chr1:77960183-77960263
chr1	77960352	77960717	chr1:77960343-77960785
chr1	77960732	77960800	chr1:77960343-77960785
chr1	77962782	77962956	chr1:77962769-77962930
chr1	77963587	77963720	chr1:77963573-77963715
chr1	77964062	77964173	chr1:77964061-77964162
chr1	77964297	77964372	chr1:77964253-77964356

chr1	77964657	77964757	chr1:77964645-77964747
chr1	77964872	77964986	chr1:77964869-77964968
chr1	77965082	77965246	chr1:77965068-77965231
chr1	77966697	77966768	chr1:77966693-77966751
chr1	77966887	77966967	chr1:77966883-77966955
chr1	77967052	77967129	chr1:77967048-77967101
chr1	77968162	77968236	chr1:77968164-77968203
chr1	77969022	77969096	chr1:77969021-77969084
chr1	77969937	77970013	chr1:77969924-77970015
chr1	77975812	77975882	chr1:77975812-77976012
chr1	77975887	77976019	chr1:77975812-77976012
chr1	77978897	77979046	chr1:77978884-77979024
chr1	79716859	79717530	chr1:79716850-79916850
chr1	79717534	79717605	chr1:79716850-79916850
chr1	79717614	79718137	chr1:79716850-79916850
chr1	79718299	79718372	chr1:79716850-79916850
chr1	79718609	79718684	chr1:79716850-79916850
chr1	79718699	79718769	chr1:79716850-79916850
chr1	79718944	79719015	chr1:79716850-79916850
chr1	79719334	79719506	chr1:79716850-79916850
chr1	79720284	79720357	chr1:79716850-79916850
chr1	79720399	79720469	chr1:79716850-79916850
chr1	79720479	79720591	chr1:79716850-79916850
chr1	79720609	79721119	chr1:79716850-79916850
chr1	79721174	79721306	chr1:79716850-79916850
chr1	79721339	79721963	chr1:79716850-79916850
chr1	79721964	79722343	chr1:79716850-79916850
chr1	79722354	79722601	chr1:79716850-79916850
chr1	79722659	79722729	chr1:79716850-79916850
chr1	79722844	79722925	chr1:79716850-79916850
chr1	79722939	79723043	chr1:79716850-79916850
chr1	79723294	79723398	chr1:79716850-79916850
chr1	79723424	79723767	chr1:79716850-79916850
chr1	79723844	79723917	chr1:79716850-79916850
chr1	79723984	79724411	chr1:79716850-79916850
chr1	79724414	79724735	chr1:79716850-79916850
chr1	79724754	79724964	chr1:79716850-79916850
chr1	79724974	79726161	chr1:79716850-79916850
chr1	79726189	79726993	chr1:79716850-79916850
chr1	79727034	79728252	chr1:79716850-79916850
chr1	79728254	79728422	chr1:79716850-79916850
chr1	79728439	79729212	chr1:79716850-79916850
chr1	79729219	79731166	chr1:79716850-79916850
chr1	79731174	79731443	chr1:79716850-79916850
chr1	79731449	79732138	chr1:79716850-79916850
chr1	79732204	79732302	chr1:79716850-79916850
chr1	79732404	79732544	chr1:79716850-79916850
chr1	79732559	79732764	chr1:79716850-79916850
chr1	79732779	79735177	chr1:79716850-79916850
chr1	79735184	79735285	chr1:79716850-79916850
chr1	79735734	79736010	chr1:79716850-79916850
chr1	79736244	79738818	chr1:79716850-79916850
chr1	79738824	79738991	chr1:79716850-79916850
chr1	79739024	79739648	chr1:79716850-79916850
chr1	79739649	79739994	chr1:79716850-79916850
chr1	79740009	79740563	chr1:79716850-79916850
chr1	79740594	79742506	chr1:79716850-79916850
chr1	79742509	79743677	chr1:79716850-79916850
chr1	79743679	79743958	chr1:79716850-79916850
chr1	79743969	79744814	chr1:79716850-79916850
chr1	79744834	79745295	chr1:79716850-79916850
chr1	79745314	79745420	chr1:79716850-79916850
chr1	79745444	79745512	chr1:79716850-79916850

chr1	79745524	79745650	chr1:79716850-79916850
chr1	79745659	79745934	chr1:79716850-79916850
chr1	79746034	79746114	chr1:79716850-79916850
chr1	79746119	79746229	chr1:79716850-79916850
chr1	79746264	79746472	chr1:79716850-79916850
chr1	79746519	79746595	chr1:79716850-79916850
chr1	79746644	79746741	chr1:79716850-79916850
chr1	79746789	79746863	chr1:79716850-79916850
chr1	79746894	79747073	chr1:79716850-79916850
chr1	79747149	79747268	chr1:79716850-79916850
chr1	79747289	79747386	chr1:79716850-79916850
chr1	79747404	79747504	chr1:79716850-79916850
chr1	79747534	79747751	chr1:79716850-79916850
chr1	79747754	79747827	chr1:79716850-79916850
chr1	79747859	79747990	chr1:79716850-79916850
chr1	79748114	79748358	chr1:79716850-79916850
chr1	79748364	79748525	chr1:79716850-79916850
chr1	79748774	79748909	chr1:79716850-79916850
chr1	79748974	79749368	chr1:79716850-79916850
chr1	79749394	79749566	chr1:79716850-79916850
chr1	79749579	79749677	chr1:79716850-79916850
chr1	79749754	79750550	chr1:79716850-79916850
chr1	79750554	79750682	chr1:79716850-79916850
chr1	79750704	79751055	chr1:79716850-79916850
chr1	79751349	79751726	chr1:79716850-79916850
chr1	79752029	79753076	chr1:79716850-79916850
chr1	79753149	79753937	chr1:79716850-79916850
chr1	79753949	79754334	chr1:79716850-79916850
chr1	79754569	79754737	chr1:79716850-79916850
chr1	79754739	79755534	chr1:79716850-79916850
chr1	79755554	79755686	chr1:79716850-79916850
chr1	79755969	79756477	chr1:79716850-79916850
chr1	79756489	79756768	chr1:79716850-79916850
chr1	79756779	79757089	chr1:79716850-79916850
chr1	79757099	79757343	chr1:79716850-79916850
chr1	79757669	79759339	chr1:79716850-79916850
chr1	79759424	79759726	chr1:79716850-79916850
chr1	79759744	79759884	chr1:79716850-79916850
chr1	79759954	79760161	chr1:79716850-79916850
chr1	79760174	79762101	chr1:79716850-79916850
chr1	79762104	79762384	chr1:79716850-79916850
chr1	79762389	79762520	chr1:79716850-79916850
chr1	79762539	79763256	chr1:79716850-79916850
chr1	79763279	79763455	chr1:79716850-79916850
chr1	79763459	79764035	chr1:79716850-79916850
chr1	79764049	79764225	chr1:79716850-79916850
chr1	79764229	79764299	chr1:79716850-79916850
chr1	79764314	79764553	chr1:79716850-79916850
chr1	79764824	79765525	chr1:79716850-79916850
chr1	79765534	79765768	chr1:79716850-79916850
chr1	79765774	79766570	chr1:79716850-79916850
chr1	79766664	79768303	chr1:79716850-79916850
chr1	79768314	79769674	chr1:79716850-79916850
chr1	79769774	79770996	chr1:79716850-79916850
chr1	79771059	79771225	chr1:79716850-79916850
chr1	79771259	79771641	chr1:79716850-79916850
chr1	79771939	79772636	chr1:79716850-79916850
chr1	79772649	79773414	chr1:79716850-79916850
chr1	79773419	79773874	chr1:79716850-79916850
chr1	79773904	79774100	chr1:79716850-79916850
chr1	79774104	79774485	chr1:79716850-79916850
chr1	79774629	79774705	chr1:79716850-79916850
chr1	79774734	79774809	chr1:79716850-79916850

chr1	79774854	79774935	chr1:79716850-79916850
chr1	79774964	79775034	chr1:79716850-79916850
chr1	79775044	79776485	chr1:79716850-79916850
chr1	79776519	79779645	chr1:79716850-79916850
chr1	79779954	79780368	chr1:79716850-79916850
chr1	79780724	79780925	chr1:79716850-79916850
chr1	79781240	79781630	chr1:79716850-79916850
chr1	79781680	79782241	chr1:79716850-79916850
chr1	79782265	79784592	chr1:79716850-79916850
chr1	79784615	79787196	chr1:79716850-79916850
chr1	79787615	79787705	chr1:79716850-79916850
chr1	79787730	79788404	chr1:79716850-79916850
chr1	79788520	79788594	chr1:79716850-79916850
chr1	79788690	79792327	chr1:79716850-79916850
chr1	79792335	79792510	chr1:79716850-79916850
chr1	79792515	79793718	chr1:79716850-79916850
chr1	79793730	79793870	chr1:79716850-79916850
chr1	79794030	79794106	chr1:79716850-79916850
chr1	79794110	79794185	chr1:79716850-79916850
chr1	79794195	79794269	chr1:79716850-79916850
chr1	79794415	79794492	chr1:79716850-79916850
chr1	79794515	79794620	chr1:79716850-79916850
chr1	79794630	79794895	chr1:79716850-79916850
chr1	79794940	79795043	chr1:79716850-79916850
chr1	79795085	79795401	chr1:79716850-79916850
chr1	79795460	79795554	chr1:79716850-79916850
chr1	79795555	79795818	chr1:79716850-79916850
chr1	79796155	79796234	chr1:79716850-79916850
chr1	79796475	79797552	chr1:79716850-79916850
chr1	79797565	79798074	chr1:79716850-79916850
chr1	79798100	79798303	chr1:79716850-79916850
chr1	79798305	79800317	chr1:79716850-79916850
chr1	79800325	79801160	chr1:79716850-79916850
chr1	79801175	79802087	chr1:79716850-79916850
chr1	79802095	79802372	chr1:79716850-79916850
chr1	79802640	79803157	chr1:79716850-79916850
chr1	79803160	79805679	chr1:79716850-79916850
chr1	79805690	79806727	chr1:79716850-79916850
chr1	79806790	79806866	chr1:79716850-79916850
chr1	79806910	79807196	chr1:79716850-79916850
chr1	79807275	79807351	chr1:79716850-79916850
chr1	79807685	79807759	chr1:79716850-79916850
chr1	79807870	79807942	chr1:79716850-79916850
chr1	79808490	79808650	chr1:79716850-79916850
chr1	79808720	79808792	chr1:79716850-79916850
chr1	79808845	79809941	chr1:79716850-79916850
chr1	79810025	79810341	chr1:79716850-79916850
chr1	79810345	79813676	chr1:79716850-79916850
chr1	79813690	79814246	chr1:79716850-79916850
chr1	79814260	79814392	chr1:79716850-79916850
chr1	79814410	79814576	chr1:79716850-79916850
chr1	79814610	79815909	chr1:79716850-79916850
chr1	79815920	79816373	chr1:79716850-79916850
chr1	79816385	79816672	chr1:79716850-79916850
chr1	79816675	79818254	chr1:79716850-79916850
chr1	79818260	79820665	chr1:79716850-79916850
chr1	79820670	79821263	chr1:79716850-79916850
chr1	79821560	79821668	chr1:79716850-79916850
chr1	79821705	79822730	chr1:79716850-79916850
chr1	79822755	79824078	chr1:79716850-79916850
chr1	79824080	79824289	chr1:79716850-79916850
chr1	79824295	79824574	chr1:79716850-79916850
chr1	79824575	79824848	chr1:79716850-79916850

chr1	79824850	79825257	chr1:79716850-79916850
chr1	79825280	79825800	chr1:79716850-79916850
chr1	79825825	79825969	chr1:79716850-79916850
chr1	79826000	79826077	chr1:79716850-79916850
chr1	79826115	79826742	chr1:79716850-79916850
chr1	79826745	79828820	chr1:79716850-79916850
chr1	79828845	79828978	chr1:79716850-79916850
chr1	79828980	79829157	chr1:79716850-79916850
chr1	79829180	79829262	chr1:79716850-79916850
chr1	79829305	79829413	chr1:79716850-79916850
chr1	79829430	79829496	chr1:79716850-79916850
chr1	79829500	79832845	chr1:79716850-79916850
chr1	79832905	79833394	chr1:79716850-79916850
chr1	79833400	79833638	chr1:79716850-79916850
chr1	79833640	79833750	chr1:79716850-79916850
chr1	79833880	79835181	chr1:79716850-79916850
chr1	79835200	79835516	chr1:79716850-79916850
chr1	79835525	79835833	chr1:79716850-79916850
chr1	79835985	79836337	chr1:79716850-79916850
chr1	79836345	79838438	chr1:79716850-79916850
chr1	79838440	79838849	chr1:79716850-79916850
chr1	79838870	79838967	chr1:79716850-79916850
chr1	79838970	79839151	chr1:79716850-79916850
chr1	79839155	79841703	chr1:79716850-79916850
chr1	79841705	79841940	chr1:79716850-79916850
chr1	79841945	79843199	chr1:79716850-79916850
chr1	79843240	79844081	chr1:79716850-79916850
chr1	79844090	79844294	chr1:79716850-79916850
chr1	79844295	79844911	chr1:79716850-79916850
chr1	79844915	79844989	chr1:79716850-79916850
chr1	79844995	79846002	chr1:79716850-79916850
chr1	79846005	79846241	chr1:79716850-79916850
chr1	79846290	79847140	chr1:79716850-79916850
chr1	79847365	79848341	chr1:79716850-79916850
chr1	79848460	79848907	chr1:79716850-79916850
chr1	79848920	79850280	chr1:79716850-79916850
chr1	79850340	79851452	chr1:79716850-79916850
chr1	79851740	79852057	chr1:79716850-79916850
chr1	79852395	79853961	chr1:79716850-79916850
chr1	79853980	79854684	chr1:79716850-79916850
chr1	79854690	79856322	chr1:79716850-79916850
chr1	79856335	79857191	chr1:79716850-79916850
chr1	79857235	79857304	chr1:79716850-79916850
chr1	79857320	79857387	chr1:79716850-79916850
chr1	79857710	79858024	chr1:79716850-79916850
chr1	79858205	79858275	chr1:79716850-79916850
chr1	79858295	79858397	chr1:79716850-79916850
chr1	79858440	79858900	chr1:79716850-79916850
chr1	79858905	79858981	chr1:79716850-79916850
chr1	79858985	79859064	chr1:79716850-79916850
chr1	79859065	79859228	chr1:79716850-79916850
chr1	79859235	79860225	chr1:79716850-79916850
chr1	79860240	79860583	chr1:79716850-79916850
chr1	79860640	79861356	chr1:79716850-79916850
chr1	79861385	79861940	chr1:79716850-79916850
chr1	79862000	79862384	chr1:79716850-79916850
chr1	79862405	79862708	chr1:79716850-79916850
chr1	79863000	79864047	chr1:79716850-79916850
chr1	79864060	79864266	chr1:79716850-79916850
chr1	79864550	79865243	chr1:79716850-79916850
chr1	79865535	79866342	chr1:79716850-79916850
chr1	79866355	79866433	chr1:79716850-79916850
chr1	79866450	79867110	chr1:79716850-79916850

chr1	79870710	79872629	chr1:79716850-79916850
chr1	79872750	79872828	chr1:79716850-79916850
chr1	79872965	79873620	chr1:79716850-79916850
chr1	79873650	79873753	chr1:79716850-79916850
chr1	79873755	79873858	chr1:79716850-79916850
chr1	79873870	79874932	chr1:79716850-79916850
chr1	79874980	79877449	chr1:79716850-79916850
chr1	79877525	79877728	chr1:79716850-79916850
chr1	79877790	79877873	chr1:79716850-79916850
chr1	79877885	79877951	chr1:79716850-79916850
chr1	79878110	79878179	chr1:79716850-79916850
chr1	79878240	79878419	chr1:79716850-79916850
chr1	79878920	79879061	chr1:79716850-79916850
chr1	79879130	79879225	chr1:79716850-79916850
chr1	79879275	79879406	chr1:79716850-79916850
chr1	79879500	79879578	chr1:79716850-79916850
chr1	79879760	79879832	chr1:79716850-79916850
chr1	79879915	79880018	chr1:79716850-79916850
chr1	79880025	79880159	chr1:79716850-79916850
chr1	79880210	79880312	chr1:79716850-79916850
chr1	79880565	79880665	chr1:79716850-79916850
chr1	79880885	79880959	chr1:79716850-79916850
chr1	79881415	79881481	chr1:79716850-79916850
chr1	79881980	79882051	chr1:79716850-79916850
chr1	79882270	79882345	chr1:79716850-79916850
chr1	79882395	79882496	chr1:79716850-79916850
chr1	79882630	79883269	chr1:79716850-79916850
chr1	79884660	79884732	chr1:79716850-79916850
chr1	79885500	79887033	chr1:79716850-79916850
chr1	79887040	79887346	chr1:79716850-79916850
chr1	79887350	79887603	chr1:79716850-79916850
chr1	79887630	79887844	chr1:79716850-79916850
chr1	79887865	79888600	chr1:79716850-79916850
chr1	79894160	79894237	chr1:79716850-79916850
chr1	79894720	79896850	chr1:79716850-79916850
chr1	79896885	79898086	chr1:79716850-79916850
chr1	79898095	79898823	chr1:79716850-79916850
chr1	79898825	79899206	chr1:79716850-79916850
chr1	79899220	79900292	chr1:79716850-79916850
chr1	79900300	79900864	chr1:79716850-79916850
chr1	79900870	79903680	chr1:79716850-79916850
chr1	79903695	79904636	chr1:79716850-79916850
chr1	79904680	79904774	chr1:79716850-79916850
chr1	79905840	79905922	chr1:79716850-79916850
chr1	79906520	79906591	chr1:79716850-79916850
chr1	79907235	79909500	chr1:79716850-79916850
chr1	79909505	79909611	chr1:79716850-79916850
chr1	79909620	79911050	chr1:79716850-79916850
chr1	79911120	79911365	chr1:79716850-79916850
chr1	79911375	79911688	chr1:79716850-79916850
chr1	79911700	79912357	chr1:79716850-79916850
chr1	79912660	79914299	chr1:79716850-79916850
chr1	79914315	79915759	chr1:79716850-79916850
chr1	79915760	79916035	chr1:79716850-79916850
chr1	79916110	79916427	chr1:79716850-79916850
chr1	79916435	79916638	chr1:79716850-79916850
chr1	79916655	79916862	chr1:79716850-79916850
chr1	89995125	89995297	chr1:89995111-89995273
chr1	89996045	89996259	chr1:89996033-89996233
chr1	89998115	89998193	chr1:89998109-89998154
chr1	90005010	90005113	chr1:90005002-90005092
chr1	90005135	90005596	chr1:90005132-90006449
chr1	90005610	90005774	chr1:90005132-90006449

chr1	90005780	90006482	chr1:90005132-90006449
chr1	90007345	90007776	chr1:90007344-90007750
chr1	90010100	90010301	chr1:90010087-90010286
chr1	90013135	90013271	chr1:90013125-90013237
chr1	90017330	90017498	chr1:90017316-90017464
chr1	90018685	90018789	chr1:90018684-90018784
chr1	90020805	90020943	chr1:90020791-90020922
chr1	90022260	90022364	chr1:90022249-90022345
chr1	90027360	90028349	chr1:90027353-90035531
chr1	90028385	90028650	chr1:90027353-90035531
chr1	90028655	90028821	chr1:90027353-90035531
chr1	90029090	90029521	chr1:90027353-90035531
chr1	90029530	90030329	chr1:90027353-90035531
chr1	90030345	90030658	chr1:90027353-90035531
chr1	90030945	90031473	chr1:90027353-90035531
chr1	90031475	90031566	chr1:90027353-90035531
chr1	90031845	90033948	chr1:90027353-90035531
chr1	90033950	90034619	chr1:90027353-90035531
chr1	90034640	90034982	chr1:90027353-90035531
chr1	90034985	90035544	chr1:90027353-90035531
chr1	90711550	90711960	chr1:90711538-90712624
chr1	90711995	90712662	chr1:90711538-90712624
chr1	90713480	90713694	chr1:90713477-90713677
chr1	90714535	90714782	chr1:90714530-90714756
chr1	90716575	90716813	chr1:90716570-90717237
chr1	90716835	90717264	chr1:90716570-90717237
chr1	92474770	92476208	chr1:92474761-92476207
chr1	92478595	92478751	chr1:92478587-92478753
chr1	92480360	92480493	chr1:92480347-92480485
chr1	92480610	92481093	chr1:92480600-92481088
chr1	92482110	92482330	chr1:92482108-92482308
chr1	92482875	92483087	chr1:92482863-92483046
chr1	92483375	92483620	chr1:92483372-92483586
chr1	92483740	92483991	chr1:92483734-92483954
chr1	92484450	92484705	chr1:92484445-92484682
chr1	92486025	92486099	chr1:92486013-92486071
chr1	92486735	92486909	chr1:92486725-92486876
chr1	93079235	93079558	chr1:93079234-93079531
chr1	93080975	93081042	chr1:93080964-93081019
chr1	93110240	93110448	chr1:93110229-93110428
chr1	93110555	93110656	chr1:93110544-93110630
chr1	93111405	93111608	chr1:93111398-93111598
chr1	93114700	93114800	chr1:93114687-93114783
chr1	93114995	93115100	chr1:93114987-93115088
chr1	93115480	93115649	chr1:93115469-93115618
chr1	93118350	93118463	chr1:93118344-93118440
chr1	93119345	93119421	chr1:93119332-93119401
chr1	93120550	93120698	chr1:93120548-93120672
chr1	93120805	93120904	chr1:93120792-93120894
chr1	93127235	93127314	chr1:93127231-93127299
chr1	93128805	93129250	chr1:93128794-93129448
chr1	93129270	93129481	chr1:93128794-93129448
chr1	93133730	93133829	chr1:93133702-93133808
chr1	93133930	93133997	chr1:93133927-93133980
chr1	93134090	93134224	chr1:93134090-93134459
chr1	93134230	93134476	chr1:93134090-93134459
chr1	93136680	93137123	chr1:93136669-93139081
chr1	93137130	93137623	chr1:93136669-93139081
chr1	93137635	93138269	chr1:93136669-93139081
chr1	93138285	93139117	chr1:93136669-93139081
chr1	110060004	110061083	chr1:110059993-110061041
chr1	110061444	110061579	chr1:110061434-110061563
chr1	110064589	110064922	chr1:110064586-110064903

chr1	110068179	110068387	chr1:110068168-110068368
chr1	110070349	110070727	chr1:110070335-110070700
chr1	114837231	114837721	chr1:114837226-114837692
chr1	114838381	114838596	chr1:114838381-114838581
chr1	114839276	114839422	chr1:114839270-114839406
chr1	114845751	114846041	chr1:114845750-114846005
chr1	114846226	114846341	chr1:114846223-114846323
chr1	114847891	114848029	chr1:114847891-114847997
chr1	114848686	114848791	chr1:114848674-114848777
chr1	114851246	114851457	chr1:114851242-114851453
chr1	115836386	115836491	chr1:115836376-115843917
chr1	115836501	115836670	chr1:115836376-115843917
chr1	115836746	115838354	chr1:115836376-115843917
chr1	115838381	115840722	chr1:115836376-115843917
chr1	115840751	115842016	chr1:115836376-115843917
chr1	115842021	115843049	chr1:115836376-115843917
chr1	115843066	115843697	chr1:115836376-115843917
chr1	115843701	115843915	chr1:115836376-115843917
chr1	118883056	118884332	chr1:118883045-118885516
chr1	118884336	118884613	chr1:118883045-118885516
chr1	118884616	118885528	chr1:118883045-118885516
chr1	118890876	118890982	chr1:118890866-118890965
chr1	118899041	118899158	chr1:118899027-118899125
chr1	118914116	118914185	chr1:118914114-118914179
chr1	118923441	118923632	chr1:118923435-118923603
chr1	118924651	118924844	chr1:118924645-118924817
chr1	118926511	118926629	chr1:118926509-118926611
chr1	118931631	118931878	chr1:118931618-118931832
chr1	118944611	118944836	chr1:118944608-118944808
chr1	118987601	118987840	chr1:118987590-118987805
chr1	118989361	118989566	chr1:118989354-118989556
chr1	149887249	149887378	chr1:149887164-149887364
chr1	150809709	150810241	chr1:150809704-150812110
chr1	150810249	150810641	chr1:150809704-150812110
chr1	150810644	150811447	chr1:150809704-150812110
chr1	150811489	150811907	chr1:150809704-150812110
chr1	150811919	150812131	chr1:150809704-150812110
chr1	150813174	150813364	chr1:150813171-150813338
chr1	150814079	150814259	chr1:150814076-150814239
chr1	150816264	150816437	chr1:150816258-150816406
chr1	150816789	150816911	chr1:150816787-150816890
chr1	150817094	150817235	chr1:150817081-150817202
chr1	150817364	150817448	chr1:150817360-150817433
chr1	150817929	150818060	chr1:150817919-150818030
chr1	150823204	150823373	chr1:150823193-150823345
chr1	150826544	150826652	chr1:150826542-150826617
chr1	150829099	150829242	chr1:150829092-150829227
chr1	150829914	150830012	chr1:150829903-150829980
chr1	150831494	150831921	chr1:150831485-150831903
chr1	150832339	150832416	chr1:150832333-150832399
chr1	150834539	150834649	chr1:150834537-150834640
chr1	150836289	150836535	chr1:150836279-150836499
chr1	150839514	150839684	chr1:150839440-150839654
chr1	150842424	150842502	chr1:150842423-150842468
chr1	150846264	150846342	chr1:150846262-150846307
chr1	150852774	150852870	chr1:150852761-150852840
chr1	150858349	150858495	chr1:150858348-150858460
chr1	150875359	150875574	chr1:150875355-150875555
chr1	150876544	150876802	chr1:150876542-150876768
chr1	151281629	151282119	chr1:151281617-151282079
chr1	151282279	151282410	chr1:151282266-151282395
chr1	151283879	151284081	chr1:151283868-151284068
chr1	151285219	151285421	chr1:151285205-151286074

chr1	151286009	151286110	chr1:151285205-151286074
chr1	151286284	151288428	chr1:151286274-151288406
chr1	151288534	151288742	chr1:151288527-151288706
chr1	151289104	151289306	chr1:151289094-151289271
chr1	151289379	151289560	chr1:151289377-151289540
chr1	151289684	151290036	chr1:151289677-151290007
chr1	151290124	151290268	chr1:151290121-151290234
chr1	151290374	151290594	chr1:151290370-151290573
chr1	151290694	151291641	chr1:151290694-151291646
chr1	151340649	151343194	chr1:151340639-151343178
chr1	151343354	151343460	chr1:151343341-151343442
chr1	151343694	151343910	chr1:151343680-151343882
chr1	151344204	151344319	chr1:151344196-151344278
chr1	151344429	151344636	chr1:151344416-151344536;chr1:151344559-151344604
chr1	151344734	151344883	chr1:151344727-151344847
chr1	151345119	151345228	chr1:151345105-151345188
chr1	151345929	151346003	chr1:151345927-151345961
chr1	151346204	151346366	chr1:151346204-151346333
chr1	151346434	151346635	chr1:151346421-151346606
chr1	151346754	151347040	chr1:151346754-151347021
chr1	151347154	151347375	chr1:151347150-151347357
chr1	151806312	151807651	chr1:151806304-151807633
chr1	151811327	151812278	chr1:151811324-151814789
chr1	151812282	151812360	chr1:151811324-151814789
chr1	151812447	151812518	chr1:151811324-151814789
chr1	151812527	151814812	chr1:151811324-151814789
chr1	151814912	151815449	chr1:151814912-151815425
chr1	151816667	151816818	chr1:151816663-151816805
chr1	151817207	151817309	chr1:151817194-151817280
chr1	151825932	151826126	chr1:151825929-151826091
chr1	151827887	151828112	chr1:151827885-151828085
chr1	151829427	151829505	chr1:151829428-151829458
chr1	151830937	151831157	chr1:151830927-151831132
chr1	151831727	151831910	chr1:151831724-151831872
chr1	152797827	152797895	chr1:152797772-152797922
chr1	153967887	153967963	chr1:153967891-153967933
chr1	153968522	153969484	chr1:153968521-153969458
chr1	153970392	153970617	chr1:153970383-153970583
chr1	153972747	153973103	chr1:153972743-153973080
chr1	153973207	153973289	chr1:153973194-153973263
chr1	153973382	153973493	chr1:153973379-153973464
chr1	153973627	153973734	chr1:153973619-153973716
chr1	153973872	153974155	chr1:153973871-153974118
chr1	155002629	155002961	chr1:155002629-155002943
chr1	155007189	155007419	chr1:155007186-155007386
chr1	155010264	155010402	chr1:155010257-155010397
chr1	155010919	155011034	chr1:155010915-155011015
chr1	155014019	155014135	chr1:155014014-155014093
chr1	155014474	155015838	chr1:155014447-155015814
chr1	155016224	155018397	chr1:155016219-155018369
chr1	156077533	156077712	chr1:156077532-156077682
chr1	156078663	156078865	chr1:156078650-156078850
chr1	156081553	156081665	chr1:156081544-156081644
chr1	156244258	156244450	chr1:156244253-156244403
chr1	156244693	156244881	chr1:156244689-156244860
chr1	156290098	156290275	chr1:156290088-156290360
chr1	156290898	156291004	chr1:156290540-156291106
chr1	156293348	156293729	chr1:156293335-156293710
chr1	156294018	156294268	chr1:156294017-156294236
chr1	156294378	156294582	chr1:156294364-156294565
chr1	156294678	156295036	chr1:156294675-156295016
chr1	156295308	156295730	chr1:156295296-156295689
chr1	156404463	156404628	chr1:156404451-156404601

chr1	156407083	156407197	chr1:156407079-156407179
chr1	156423598	156423819	chr1:156423596-156423796
chr1	156463728	156464951	chr1:156463726-156467656
chr1	156465013	156465648	chr1:156463726-156467656
chr1	156465698	156466288	chr1:156463726-156467656
chr1	156466303	156467461	chr1:156463726-156467656
chr1	156467483	156467692	chr1:156463726-156467656
chr1	156467998	156468323	chr1:156467992-156468299
chr1	156468783	156469046	chr1:156468779-156469020
chr1	156475108	156475264	chr1:156475107-156475237
chr1	156477013	156477234	chr1:156477011-156477202
chr1	156479303	156479379	chr1:156479289-156479346
chr1	156479598	156479811	chr1:156479585-156479796
chr1	156480653	156480788	chr1:156480642-156480777
chr1	156480833	156480976	chr1:156480833-156480971
chr1	156482438	156482662	chr1:156482436-156482640
chr1	156483243	156483461	chr1:156483238-156483430
chr1	156490513	156490621	chr1:156490511-156490599
chr1	156497533	156497749	chr1:156497532-156497732
chr1	156499443	156499573	chr1:156499431-156499560
chr1	156500498	156500839	chr1:156500485-156500828
chr1	156673058	156673245	chr1:156673055-156673205
chr1	156673458	156673560	chr1:156673453-156673527
chr1	156742118	156742526	chr1:156742106-156743878
chr1	156742533	156743894	chr1:156742106-156743878
chr1	156744168	156744395	chr1:156744162-156744348
chr1	156744503	156744573	chr1:156744500-156744570
chr1	156745008	156745407	chr1:156745007-156745373
chr1	156747438	156747513	chr1:156747434-156747471
chr1	156749323	156749530	chr1:156749309-156749509
chr1	156751353	156751733	chr1:156751342-156751744
chr1	156752048	156752473	chr1:156752037-156752448
chr1	156755538	156755687	chr1:156755536-156755664
chr1	156758993	156759237	chr1:156758982-156759219
chr1	156766798	156766941	chr1:156766789-156766925
chr1	157121203	157121298	chr1:157121190-157125979
chr1	157121333	157124198	chr1:157121190-157125979
chr1	157124258	157126002	chr1:157121190-157125979
chr1	157133233	157134259	chr1:157133144-157134227
chr1	157135093	157135733	chr1:157135091-157135708
chr1	157136318	157136395	chr1:157136306-157136365
chr1	157137048	157137251	chr1:157137037-157137237
chr1	157138318	157138495	chr1:157138315-157138474
chr1	158999968	159000423	chr1:158999967-159000395
chr1	159005963	159006075	chr1:159005961-159006041
chr1	159009008	159009252	chr1:159008994-159009225
chr1	159009903	159010180	chr1:159009891-159010161
chr1	159014663	159014939	chr1:159014660-159014945
chr1	159015683	159016003	chr1:159015672-159015987
chr1	159016543	159016722	chr1:159016532-159016700
chr1	159018233	159018682	chr1:159018228-159018651
chr1	159020353	159020564	chr1:159020340-159020529
chr1	159032533	159032705	chr1:159032523-159032691
chr1	159037338	159037562	chr1:159037325-159037525
chr1	159045308	159045489	chr1:159045296-159045464
chr1	159049443	159049624	chr1:159049431-159049599
chr1	159051678	159052109	chr1:159051678-159052098
chr1	159052318	159052396	chr1:159052319-159052350
chr1	159053543	159053744	chr1:159053532-159053724
chr1	159054823	159055069	chr1:159054820-159055155
chr1	159055118	159055189	chr1:159054820-159055155
chr1	160367093	160367359	chr1:160367066-160367337
chr1	160368238	160368455	chr1:160368236-160368436

chr1	160370558	160371895	chr1:160370556-160372848
chr1	160371898	160372337	chr1:160370556-160372848
chr1	160372383	160372809	chr1:160370556-160372848
chr1	160372813	160372884	chr1:160370556-160372848
chr1	161039308	161040047	chr1:161039250-161040009
chr1	161040203	161040350	chr1:161040201-161040330
chr1	161040583	161040693	chr1:161040575-161040670
chr1	161040813	161040890	chr1:161040813-161040872
chr1	161041328	161041444	chr1:161041323-161041411
chr1	161041658	161041882	chr1:161041650-161041846
chr1	161042118	161042235	chr1:161042115-161042217
chr1	161042563	161042699	chr1:161042554-161042670
chr1	161042833	161042908	chr1:161042832-161042882
chr1	161043258	161043402	chr1:161043252-161043360
chr1	161044903	161044970	chr1:161044911-161044941
chr1	161045868	161046013	chr1:161045857-161045977
chr1	161230898	161231506	chr1:161230894-161231474
chr1	161232808	161232970	chr1:161232806-161232946
chr1	161233178	161233369	chr1:161233168-161233338
chr1	161235848	161236004	chr1:161235846-161235977
chr1	161236458	161236612	chr1:161236458-161236598
chr1	161238048	161238325	chr1:161238040-161238302
chr1	161261108	161261318	chr1:161261106-161261306
chr1	161283668	161283738	chr1:161283667-161283722
chr1	161284378	161284522	chr1:161284338-161284488
chr1	161766293	161766470	chr1:161766293-161766442
chr1	161778243	161778359	chr1:161778243-161778320
chr1	161781923	161782024	chr1:161781911-161781999
chr1	161783998	161784117	chr1:161783989-161784096
chr1	161791418	161791563	chr1:161791407-161791537
chr1	161792133	161792345	chr1:161792123-161792327
chr1	161802058	161802306	chr1:161802051-161802272
chr1	161819633	161819857	chr1:161819632-161819818
chr1	161821083	161821187	chr1:161821069-161821161
chr1	161836553	161836772	chr1:161836548-161836748
chr1	161840078	161840504	chr1:161840074-161840468
chr1	161846458	161846601	chr1:161846448-161846580
chr1	161851728	161851870	chr1:161851721-161851835
chr1	161853228	161853334	chr1:161853223-161853323
chr1	161860213	161860295	chr1:161860206-161860277
chr1	161863203	161863343	chr1:161863197-161863312
chr1	161912298	161912415	chr1:161912295-161912380
chr1	161958453	161959393	chr1:161958445-161964070
chr1	161959698	161960075	chr1:161958445-161964070
chr1	161960093	161961567	chr1:161958445-161964070
chr1	161961583	161962120	chr1:161958445-161964070
chr1	161962138	161964088	chr1:161958445-161964070
chr1	163147359	163147540	chr1:163147353-163147503
chr1	163152544	163152690	chr1:163152537-163152649
chr1	163193259	163193478	chr1:163193254-163193454
chr1	164555684	164555759	chr1:164555583-164555719
chr1	164559209	164559547	chr1:164559199-164559529
chr1	164559674	164560033	chr1:164559634-164560013
chr1	164560149	164560226	chr1:164560145-164560357
chr1	164560239	164560371	chr1:164560145-164560357
chr1	164562814	164562949	chr1:164562804-164562930
chr1	164563244	164563345	chr1:164563237-164563311
chr1	164563929	164564042	chr1:164563927-164564015
chr1	164576794	164577049	chr1:164576783-164577004
chr1	164590359	164590468	chr1:164590349-164590434
chr1	164605194	164605299	chr1:164605192-164605419
chr1	164605304	164605446	chr1:164605192-164605419
chr1	164625929	164626112	chr1:164625928-164626096

chr1	164630994	164631127	chr1:164630980-164631116
chr1	164641039	164642013	chr1:164641028-164642193
chr1	164642024	164642230	chr1:164641028-164642193
chr1	164642704	164642853	chr1:164642692-164642829
chr1	164651964	164652070	chr1:164651895-164652139
chr1	164657114	164657405	chr1:164657112-164657372
chr1	164674489	164674844	chr1:164674489-164674816
chr1	164682319	164682486	chr1:164682309-164682483
chr1	164764444	164764648	chr1:164764434-164764634
chr1	164772919	164773058	chr1:164772911-164773022
chr1	164787654	164787788	chr1:164787642-164787753
chr1	164792494	164792749	chr1:164792491-164792738
chr1	164799704	164799919	chr1:164799698-164799889
chr1	164807544	164807703	chr1:164807541-164807677
chr1	164811989	164812166	chr1:164811989-164812149
chr1	164820079	164820223	chr1:164820071-164820184
chr1	164821549	164821651	chr1:164821536-164821626
chr1	164846594	164848181	chr1:164846583-164851831
chr1	164848194	164849809	chr1:164846583-164851831
chr1	164849814	164849903	chr1:164846583-164851831
chr1	164849914	164850050	chr1:164846583-164851831
chr1	164850094	164850330	chr1:164846583-164851831
chr1	164850354	164850425	chr1:164846583-164851831
chr1	164850484	164851458	chr1:164846583-164851831
chr1	164851489	164851861	chr1:164846583-164851831
chr1	165201879	165203465	chr1:165201866-165204040
chr1	165203479	165204081	chr1:165201866-165204040
chr1	165205864	165206054	chr1:165205863-165206034
chr1	165207494	165207632	chr1:165207481-165207614
chr1	165210709	165210814	chr1:165210698-165210776
chr1	165212964	165213075	chr1:165212958-165213055
chr1	165213209	165213314	chr1:165213207-165213280
chr1	165213649	165213828	chr1:165213640-165213813
chr1	165249419	165249661	chr1:165249407-165249640
chr1	165347934	165348032	chr1:165347926-165348126
chr1	165348054	165348155	chr1:165347926-165348126
chr1	165353079	165353303	chr1:165353075-165353262
chr1	165355494	165355786	chr1:165355483-165355746
chr1	165356079	165356256	chr1:165356069-165356241
chr1	165356354	165356752	chr1:165356354-165356715
chr1	165400919	165401459	chr1:165400916-165401410
chr1	165406819	165406923	chr1:165406811-165406917
chr1	165408234	165408340	chr1:165408226-165408318
chr1	165409559	165409708	chr1:165409557-165409690
chr1	165410714	165410851	chr1:165410701-165410831
chr1	165410959	165411144	chr1:165410948-165411109
chr1	165417049	165417258	chr1:165417040-165417220
chr1	165419874	165420056	chr1:165419869-165420014
chr1	165423044	165423258	chr1:165423030-165423230
chr1	165424759	165425014	chr1:165424750-165424972
chr1	165428729	165429005	chr1:165428718-165428966
chr1	165436939	165437216	chr1:165436927-165437201
chr1	165444844	165445151	chr1:165444844-165445355
chr1	165445159	165445364	chr1:165444844-165445355
chr1	167220838	167220995	chr1:167220828-167220958
chr1	167222298	167222483	chr1:167222292-167222492
chr1	167241408	167241620	chr1:167241406-167241610
chr1	167303473	167303581	chr1:167303473-167303574
chr1	167329043	167329342	chr1:167329043-167329306
chr1	167332478	167332556	chr1:167332469-167332535
chr1	167338043	167338111	chr1:167337969-167338258
chr1	167338123	167338198	chr1:167337969-167338258
chr1	167365472	167365587	chr1:167365466-167365569

chr1	167370172	167370246	chr1:167370160-167370214
chr1	167371917	167372071	chr1:167371916-167372036
chr1	167374107	167374339	chr1:167374107-167374296
chr1	167376032	167376191	chr1:167376028-167376155
chr1	167376552	167376624	chr1:167376552-167376587
chr1	167383857	167383969	chr1:167383856-167383951
chr1	167387192	167387284	chr1:167387180-167387255
chr1	167389597	167389776	chr1:167389587-167389761
chr1	167396287	167396469	chr1:167396285-167396427
chr1	167396532	167396683	chr1:167396532-167396825
chr1	167396702	167396856	chr1:167396532-167396825
chr1	167398002	167398151	chr1:167397993-167398133
chr1	167399197	167399378	chr1:167399185-167399365
chr1	167401462	167401568	chr1:167401450-167401556
chr1	167411967	167412045	chr1:167411958-167412304
chr1	167412067	167412344	chr1:167411958-167412304
chr1	167413032	167413139	chr1:167413025-167413114
chr1	167415502	167415928	chr1:167415499-167427345
chr1	167415987	167416083	chr1:167415499-167427345
chr1	167416132	167416229	chr1:167415499-167427345
chr1	167416257	167416391	chr1:167415499-167427345
chr1	167416397	167418061	chr1:167415499-167427345
chr1	167418067	167418898	chr1:167415499-167427345
chr1	167418932	167419312	chr1:167415499-167427345
chr1	167419357	167420150	chr1:167415499-167427345
chr1	167420452	167421949	chr1:167415499-167427345
chr1	167421967	167425040	chr1:167415499-167427345
chr1	167425057	167425231	chr1:167415499-167427345
chr1	167425242	167426225	chr1:167415499-167427345
chr1	167426242	167426420	chr1:167415499-167427345
chr1	167426427	167426565	chr1:167415499-167427345
chr1	167426607	167427246	chr1:167415499-167427345
chr1	167427262	167427370	chr1:167415499-167427345
chr1	168281042	168281330	chr1:168281039-168281293
chr1	168282472	168282691	chr1:168282471-168282671
chr1	168291162	168291452	chr1:168291159-168291424
chr1	168293152	168293293	chr1:168293143-168293278
chr1	168297627	168297800	chr1:168297623-168297785
chr1	168300422	168300503	chr1:168300421-168300483
chr1	168305007	168305224	chr1:168305007-168305196
chr1	168308017	168308092	chr1:168308016-168308877
chr1	168308257	168308914	chr1:168308016-168308877
chr1	168312712	168313660	chr1:168312707-168314426
chr1	168313942	168314466	chr1:168312707-168314426
chr1	170662737	170662950	chr1:170662727-170662924
chr1	170663147	170663421	chr1:170663133-170664459
chr1	170663422	170663487	chr1:170663133-170664459
chr1	170663502	170663733	chr1:170663133-170664459
chr1	170663737	170664050	chr1:170663133-170664459
chr1	170664097	170664483	chr1:170663133-170664459
chr1	170719752	170719924	chr1:170719725-170719903
chr1	170722627	170722702	chr1:170722616-170722668
chr1	170726227	170726849	chr1:170726219-170729021
chr1	170726897	170727072	chr1:170726219-170729021
chr1	170727117	170728866	chr1:170726219-170729021
chr1	170728877	170729054	chr1:170726219-170729021
chr1	170730217	170730383	chr1:170730205-170730348
chr1	170730447	170730645	chr1:170730445-170730621
chr1	170732247	170732460	chr1:170732236-170732436
chr1	170736052	170736546	chr1:170736047-170739419
chr1	170736567	170736918	chr1:170736047-170739419
chr1	170736942	170737126	chr1:170736047-170739419
chr1	170737172	170737624	chr1:170736047-170739419

chr1	170737632	170739432	chr1:170736047-170739419
chr1	175323330	175323513	chr1:175323326-175323476
chr1	175324365	175324469	chr1:175324355-175324455
chr1	175636775	175636995	chr1:175636771-175636971
chr1	179551304	179551494	chr1:179551301-179551451
chr1	179552614	179552727	chr1:179552602-179552681
chr1	179556214	179556429	chr1:179556205-179556405
chr1	180230289	180230437	chr1:180230285-180230605
chr1	180230449	180230616	chr1:180230285-180230605
chr1	180238284	180238514	chr1:180238284-180238484
chr1	180248294	180248499	chr1:180248284-180248469
chr1	180266399	180266611	chr1:180266391-180266594
chr1	180274264	180274527	chr1:180274262-180274500
chr1	180276384	180278824	chr1:180276374-180278982
chr1	180278844	180279012	chr1:180276374-180278982
chr1	182646769	182646948	chr1:182646767-182646917
chr1	182648149	182648249	chr1:182648136-182648236
chr1	182675709	182675915	chr1:182675699-182675899
chr1	185045369	185045685	chr1:185045363-185045649
chr1	185046099	185046315	chr1:185046095-185046295
chr1	185047154	185047365	chr1:185047147-185047347
chr1	185087559	185087664	chr1:185087551-185087640
chr1	185091579	185091760	chr1:185091578-185091739
chr1	185093069	185093315	chr1:185093060-185093276
chr1	185098084	185098336	chr1:185098071-185098308
chr1	185099799	185099979	chr1:185099790-185099962
chr1	185100209	185100508	chr1:185100199-185102608
chr1	185100549	185100903	chr1:185100199-185102608
chr1	185100909	185101805	chr1:185100199-185102608
chr1	185101844	185102234	chr1:185100199-185102608
chr1	185102254	185102535	chr1:185100199-185102608
chr1	185102544	185102640	chr1:185100199-185102608
chr1	197084290	197084425	chr1:197084276-197084426
chr1	197086805	197086920	chr1:197086802-197086902
chr1	197145150	197145366	chr1:197145149-197145349
chr1	197911905	197912056	chr1:197911901-197912044
chr1	197912145	197912256	chr1:197912140-197912244
chr1	197912495	197912569	chr1:197912487-197912559
chr1	197913015	197913169	chr1:197913014-197913120
chr1	197915875	197916792	chr1:197915836-197916751
chr1	197917365	197917440	chr1:197917354-197917997
chr1	197917445	197918032	chr1:197917354-197917997
chr1	197918325	197918781	chr1:197918315-197918766
chr1	197919985	197920189	chr1:197919971-197920174
chr1	197921310	197921696	chr1:197921303-197921659
chr1	197924615	197924828	chr1:197924605-197924805
chr1	197927600	197927821	chr1:197927590-197927793
chr1	197929005	197929275	chr1:197929001-197935478
chr1	197929350	197929484	chr1:197929001-197935478
chr1	197929485	197929579	chr1:197929001-197935478
chr1	197929600	197930245	chr1:197929001-197935478
chr1	197930270	197930411	chr1:197929001-197935478
chr1	197930415	197930519	chr1:197929001-197935478
chr1	197930520	197930760	chr1:197929001-197935478
chr1	197930775	197932064	chr1:197929001-197935478
chr1	197932095	197932795	chr1:197929001-197935478
chr1	197932805	197933324	chr1:197929001-197935478
chr1	197933340	197933682	chr1:197929001-197935478
chr1	197933775	197934714	chr1:197929001-197935478
chr1	197934715	197934880	chr1:197929001-197935478
chr1	197934900	197935279	chr1:197929001-197935478
chr1	197935295	197935509	chr1:197929001-197935478
chr1	198253093	198253192	chr1:198253080-198253180

chr1	198256333	198256506	chr1:198256327-198256477
chr1	198297143	198297266	chr1:198297140-198297240
chr1	198304308	198304524	chr1:198304306-198304506
chr1	198319418	198319600	chr1:198319411-198319561
chr1	200027603	200027676	chr1:200027601-200027911
chr1	200027708	200027945	chr1:200027601-200027911
chr1	200029033	200029732	chr1:200029032-200029728
chr1	200038713	200038794	chr1:200038700-200038772
chr1	200039658	200039803	chr1:200039657-200039795
chr1	200042833	200042976	chr1:200042824-200042965
chr1	200043783	200044510	chr1:200043773-200044535
chr1	200045453	200045619	chr1:200045442-200045584
chr1	200048183	200048845	chr1:200048171-200048818
chr1	200058083	200058450	chr1:200057503-200058424
chr1	200080743	200080954	chr1:200080732-200080932
chr1	200111213	200111344	chr1:200111201-200111321
chr1	200120813	200120989	chr1:200120807-200120955
chr1	200173973	200174247	chr1:200173962-200177424
chr1	200174263	200176665	chr1:200173962-200177424
chr1	200176763	200177357	chr1:200173962-200177424
chr1	200177383	200177457	chr1:200173962-200177424
chr1	200404944	200405849	chr1:200404939-200409723
chr1	200405869	200406695	chr1:200404939-200409723
chr1	200406714	200406967	chr1:200404939-200409723
chr1	200406969	200409271	chr1:200404939-200409723
chr1	200409274	200409581	chr1:200404939-200409723
chr1	200409614	200409745	chr1:200404939-200409723
chr1	200409959	200410093	chr1:200409945-200410056
chr1	201039944	201040117	chr1:201039932-201040082
chr1	201040234	201040348	chr1:201040230-201040330
chr1	201095804	201096019	chr1:201095796-201095996
chr1	202007944	202008721	chr1:202007944-202011808
chr1	202009084	202009618	chr1:202007944-202011808
chr1	202009619	202011577	chr1:202007944-202011808
chr1	202011969	202012327	chr1:202011956-202012759
chr1	202012329	202012792	chr1:202011956-202012759
chr1	202012859	202013072	chr1:202012856-202013036
chr1	202013184	202013327	chr1:202013181-202013298
chr1	202013789	202014067	chr1:202013777-202014024
chr1	202015209	202016246	chr1:202015208-202017188
chr1	202016259	202016514	chr1:202015208-202017188
chr1	202016544	202016618	chr1:202015208-202017188
chr1	202016774	202017225	chr1:202015208-202017188
chr1	203083204	203083346	chr1:203083131-203084031
chr1	203083354	203084057	chr1:203083131-203084031
chr1	203084649	203084771	chr1:203084646-203084728
chr1	203085504	203086070	chr1:203085490-203086036
chr1	203305504	203305781	chr1:203305490-203305748
chr1	203307109	203307529	chr1:203307103-203309602
chr1	203307604	203309619	chr1:203307103-203309602
chr1	204073124	204073748	chr1:204073114-204073711
chr1	204074039	204074318	chr1:204074025-204074389
chr1	204074329	204074428	chr1:204074025-204074389
chr1	204078129	204078211	chr1:204078128-204078185
chr1	204083849	204084050	chr1:204083836-204084036
chr1	204112924	204113173	chr1:204112914-204113134
chr1	204114324	204114468	chr1:204114320-204114432
chr1	204114529	204114623	chr1:204114518-204114605
chr1	204116064	204116392	chr1:204116064-204116349
chr1	204116509	204116720	chr1:204116503-204116679
chr1	204117134	204117218	chr1:204117121-204117190
chr1	204117594	204117986	chr1:204117592-204119859
chr1	204118629	204119083	chr1:204117592-204119859

chr1	204119094	204119373	chr1:204117592-204119859
chr1	204119384	204119482	chr1:204117592-204119859
chr1	204119509	204119731	chr1:204117592-204119859
chr1	204121874	204122016	chr1:204121870-204121985
chr1	204122249	204122426	chr1:204122236-204122399
chr1	204122854	204122990	chr1:204122853-204122963
chr1	204123124	204123242	chr1:204123111-204123208
chr1	204123674	204123851	chr1:204123660-204123804
chr1	204124654	204124896	chr1:204124640-204124857
chr1	204125869	204126718	chr1:204125857-204127743
chr1	204126719	204127775	chr1:204125857-204127743
chr1	205046724	205046939	chr1:205046711-205046911
chr1	205073144	205073250	chr1:205073136-205073236
chr1	205073664	205073840	chr1:205073655-205073805
chr1	205074109	205074284	chr1:205074096-205074246
chr1	205505840	205506071	chr1:205505840-205506040
chr1	205530635	205530736	chr1:205530627-205530705
chr1	205531350	205531530	chr1:205531343-205531493
chr1	205599830	205600227	chr1:205599824-205600194
chr1	205603200	205603384	chr1:205603200-205603353
chr1	205607970	205608107	chr1:205607942-205616644
chr1	205608120	205608566	chr1:205607942-205616644
chr1	205608570	205609748	chr1:205607942-205616644
chr1	205609795	205610779	chr1:205607942-205616644
chr1	205610785	205613020	chr1:205607942-205616644
chr1	205613085	205613195	chr1:205607942-205616644
chr1	205613355	205613427	chr1:205607942-205616644
chr1	205613430	205615104	chr1:205607942-205616644
chr1	205615415	205616281	chr1:205607942-205616644
chr1	205616315	205616661	chr1:205607942-205616644
chr1	205618960	205619110	chr1:205618956-205619073
chr1	205619335	205620849	chr1:205619267-205620838
chr1	205623675	205623940	chr1:205623675-205623891
chr1	205628350	205628562	chr1:205628348-205628548
chr1	205631645	205631992	chr1:205631631-205631962
chr1	209590414	209590631	chr1:209590406-209590606
chr1	209612824	209612930	chr1:209612812-209612912
chr1	209613039	209613231	chr1:209613039-209613189
chr1	209785624	209786998	chr1:209785622-209788644
chr1	209787214	209787625	chr1:209785622-209788644
chr1	209787629	209788683	chr1:209785622-209788644
chr1	209789674	209789829	chr1:209789666-209789785
chr1	209790494	209790924	chr1:209790494-209790887
chr1	209792279	209792458	chr1:209792268-209792427
chr1	209795289	209795442	chr1:209795289-209795418
chr1	209796349	209796562	chr1:209796347-209796552
chr1	209801244	209801452	chr1:209801239-209801416
chr1	209801984	209802189	chr1:209801971-209802166
chr1	209804779	209804988	chr1:209804772-209804972
chr1	209805959	209806201	chr1:209805946-209806175
chr1	212090039	212090253	chr1:212090029-212090229
chr1	212100994	212101092	chr1:212100984-212101084
chr1	212102854	212103023	chr1:212102841-212102991
chr1	212565344	212565513	chr1:212565333-212565483
chr1	212596289	212596496	chr1:212596281-212596481
chr1	212608634	212608943	chr1:212608627-212608930
chr1	212613444	212613553	chr1:212613435-212614127
chr1	212613564	212614165	chr1:212613435-212614127
chr1	212615019	212615280	chr1:212615017-212615261
chr1	212618139	212618274	chr1:212618126-212618234
chr1	212619054	212620804	chr1:212619045-212620777
chr1	212686424	212687023	chr1:212686417-212686979
chr1	212687474	212687549	chr1:212687466-212687509

chr1	212690394	212690596	chr1:212690383-212690583
chr1	212696964	212698365	chr1:212696960-212698755
chr1	212698414	212698793	chr1:212696960-212698755
chr1	212699684	212699988	chr1:212699672-212699985
chr1	213983186	213983362	chr1:213983180-213983323
chr1	213987936	213988238	chr1:213987934-213988483
chr1	213988266	213988408	chr1:213987934-213988483
chr1	213988446	213988749	chr1:213987934-213988483;chr1:213988500-213988721
chr1	213989731	213989806	chr1:213989717-213989781
chr1	213996481	213998291	chr1:213996468-213998262
chr1	214005166	214005303	chr1:214005164-214005272
chr1	214011531	214011737	chr1:214011520-214011715
chr1	214019041	214019244	chr1:214019029-214019229
chr1	214035661	214036420	chr1:214035648-214041502
chr1	214036426	214038385	chr1:214035648-214041502
chr1	214038396	214038525	chr1:214035648-214041502
chr1	214038531	214039064	chr1:214035648-214041502
chr1	214039111	214039424	chr1:214035648-214041502
chr1	214039466	214039772	chr1:214035648-214041502
chr1	214039831	214040458	chr1:214035648-214041502
chr1	214040476	214040755	chr1:214035648-214041502
chr1	214040766	214040948	chr1:214035648-214041502
chr1	214041041	214041500	chr1:214035648-214041502
chr1	214635801	214635875	chr1:214635800-214636000
chr1	214635891	214635992	chr1:214635800-214636000
chr1	214652891	214653000	chr1:214652889-214652989
chr1	214658936	214659053	chr1:214658928-214659028
chr1	214663601	214663775	chr1:214663590-214663740
chr1	216503257	216503502	chr1:216503245-216507183
chr1	216503512	216503713	chr1:216503245-216507183
chr1	216503717	216503850	chr1:216503245-216507183
chr1	216503852	216504195	chr1:216503245-216507183
chr1	216504207	216505461	chr1:216503245-216507183
chr1	216505462	216505911	chr1:216503245-216507183
chr1	216505917	216506402	chr1:216503245-216507183
chr1	216506522	216506589	chr1:216503245-216507183
chr1	216506597	216506695	chr1:216503245-216507183
chr1	216506702	216507231	chr1:216503245-216507183
chr1	216519157	216519455	chr1:216519151-216519421
chr1	216564222	216564442	chr1:216564218-216564401
chr1	216567992	216568132	chr1:216567987-216568098
chr1	216607742	216607953	chr1:216607732-216607932
chr1	216650972	216651121	chr1:216650972-216651089
chr1	216677082	216677511	chr1:216677075-216677491
chr1	216681902	216682044	chr1:216681895-216682026
chr1	216686087	216686227	chr1:216686081-216686188
chr1	216701437	216701543	chr1:216701424-216701509
chr1	216707372	216707506	chr1:216707358-216707486
chr1	216714522	216714656	chr1:216714515-216714634
chr1	216715082	216715296	chr1:216715073-216715253
chr1	216723252	216723490	chr1:216723239-216723472
chr1	216804947	216805414	chr1:216804942-216805367
chr1	216855042	216855147	chr1:216855041-216855125
chr1	216862437	216863142	chr1:216862229-216865178
chr1	216863147	216863381	chr1:216862229-216865178
chr1	216863387	216863662	chr1:216862229-216865178
chr1	216863677	216864338	chr1:216862229-216865178
chr1	216864347	216864553	chr1:216862229-216865178
chr1	216864572	216864823	chr1:216862229-216865178
chr1	216864827	216865197	chr1:216862229-216865178
chr1	216939588	216939707	chr1:216939581-216939673
chr1	216985763	216985845	chr1:216985757-216985817
chr1	217000278	217000763	chr1:217000252-217000724

chr1	217076853	217076966	chr1:217076851-217076931
chr1	217078138	217078393	chr1:217078135-217078360
chr1	217089518	217089653	chr1:217089506-217089645
chr1	217090443	217090589	chr1:217090430-217090547
chr1	217137668	217137785	chr1:217137666-217137755
chr1	220879484	220880459	chr1:220879399-220880449
chr1	220881064	220881133	chr1:220881068-220881099
chr1	220881204	220881405	chr1:220881193-220881373
chr1	220882164	220882384	chr1:220882163-220882348
chr1	220884194	220885065	chr1:220884194-220885059
chr1	225846795	225846971	chr1:225846782-225846932
chr1	225847045	225847127	chr1:225847033-225847133
chr1	225860855	225861043	chr1:225860847-225860997
chr1	225862225	225862345	chr1:225862217-225862317
chr1	225875305	225875514	chr1:225875304-225875504
chr1	226223625	226224093	chr1:226223617-226224074
chr1	226225495	226226402	chr1:226225482-226227054
chr1	226226700	226227077	chr1:226225482-226227054
chr1	226361395	226361584	chr1:226361391-226361541
chr1	226361970	226362074	chr1:226361968-226362068
chr1	226392265	226392349	chr1:226392257-226392314
chr1	226392925	226393000	chr1:226392916-226392980
chr1	226402220	226402393	chr1:226402213-226402379
chr1	226404300	226404514	chr1:226404296-226404496
chr1	226407815	226407934	chr1:226407809-226407909
chr1	230703192	230703373	chr1:230703179-230703329
chr1	230704202	230704317	chr1:230704192-230704292
chr1	230712282	230712497	chr1:230712269-230712469
chr1	244048940	244049009	chr1:244048938-244048993
chr1	244049580	244049782	chr1:244049566-244049766
chr1	244051290	244051466	chr1:244051282-244051444
chr1	244053800	244054777	chr1:244053787-244057476
chr1	244054805	244055363	chr1:244053787-244057476
chr1	244055405	244055716	chr1:244053787-244057476
chr1	244055745	244056298	chr1:244053787-244057476
chr1	244056315	244056594	chr1:244053787-244057476
chr1	244056620	244056929	chr1:244053787-244057476
chr1	244056930	244057098	chr1:244053787-244057476
chr1	244057100	244057493	chr1:244053787-244057476
chr1	247121985	247122438	chr1:247121974-247123871
chr1	247122660	247123191	chr1:247121974-247123871
chr1	247123485	247123913	chr1:247121974-247123871
chr1	247138540	247138786	chr1:247138144-247138757
chr1	247142240	247142432	chr1:247142202-247142402
chr1	247149870	247149979	chr1:247149857-247149950
chr1	247155905	247156644	chr1:247155900-247157403
chr1	247156680	247157000	chr1:247155900-247157403
chr1	247157080	247157175	chr1:247155900-247157403
chr1	247157180	247157432	chr1:247155900-247157403
chr1	247159015	247159086	chr1:247159005-247159066
chr1	247159690	247159766	chr1:247159686-247159867
chr1	247159780	247159879	chr1:247159686-247159867
chr1	247171850	247172030	chr1:247171847-247172016
chr1	247297425	247298332	chr1:247297411-247301276
chr1	247298600	247299165	chr1:247297411-247301276
chr1	247299185	247301321	chr1:247297411-247301276
chr1	247308480	247310583	chr1:247308474-247313852
chr1	247310610	247311094	chr1:247308474-247313852
chr1	247311455	247311526	chr1:247308474-247313852
chr1	247311530	247312479	chr1:247308474-247313852
chr1	247312755	247313098	chr1:247308474-247313852
chr1	247313100	247313245	chr1:247308474-247313852
chr1	247313270	247313479	chr1:247308474-247313852

chr1	247313485	247313877	chr1:247308474-247313852
chr1	247322705	247322846	chr1:247322697-247322805
chr1	247323165	247323270	chr1:247323153-247323230
chr1	247324970	247325193	chr1:247324961-247325161
chr1	247328675	247328898	chr1:247328675-247328866
chr1	247329190	247329307	chr1:247329188-247329615
chr1	247329330	247329653	chr1:247329188-247329615
chr1	247329975	247330127	chr1:247329965-247330081
chr1	247331445	247331753	chr1:247331431-247331743
chr1	247331795	247331865	chr1:247331789-247331846
chr1	248850012	248850541	chr1:248850005-248850516
chr1	248850692	248850797	chr1:248850681-248850781
chr1	248850872	248850976	chr1:248850862-248850947
chr1	248853947	248854085	chr1:248853936-248854051
chr1	248855382	248855503	chr1:248855379-248855458
chr1	248855557	248855981	chr1:248855557-248855946
chr1	248856297	248856437	chr1:248856287-248856422
chr1	248856527	248856607	chr1:248856513-248856562
chr1	248857237	248858080	chr1:248857233-248858065
chr1	248858132	248858386	chr1:248858130-248858860
chr1	248858412	248858899	chr1:248858130-248858860
chr1	248858927	248859097	chr1:248858917-248859085
chr2	1789115	1789957	chr2:1789112-1792007
chr2	1789975	1790214	chr2:1789112-1792007
chr2	1790230	1791272	chr2:1789112-1792007
chr2	1791275	1791464	chr2:1789112-1792007
chr2	1791485	1792045	chr2:1789112-1792007
chr2	1792345	1792494	chr2:1792320-1792464
chr2	1801690	1801834	chr2:1801689-1801799
chr2	1809085	1809201	chr2:1809075-1809167
chr2	1816180	1817042	chr2:1816180-1817016
chr2	1839160	1839404	chr2:1839148-1839370
chr2	1840760	1840902	chr2:1840759-1842907
chr2	1841400	1841949	chr2:1840759-1842907
chr2	1841985	1842927	chr2:1840759-1842907
chr2	1851645	1851723	chr2:1851640-1851703
chr2	1853890	1854090	chr2:1853880-1854080
chr2	1883880	1884131	chr2:1883876-1884091
chr2	1886540	1886618	chr2:1886538-1886607
chr2	1887490	1887651	chr2:1887487-1887609
chr2	1889240	1889497	chr2:1889240-1889477
chr2	1892050	1892325	chr2:1892036-1892287
chr2	1903080	1903332	chr2:1903079-1903294
chr2	1910240	1910390	chr2:1910239-1910347
chr2	1912030	1912143	chr2:1912019-1912110
chr2	1917215	1917356	chr2:1917204-1917339
chr2	1922285	1923274	chr2:1922285-1923263
chr2	1943090	1943306	chr2:1942981-1943334
chr2	1974415	1974778	chr2:1974414-1974751
chr2	1979165	1979248	chr2:1979164-1979227
chr2	1979525	1979603	chr2:1979520-1979554
chr2	1979730	1979810	chr2:1979722-1979777
chr2	1997200	1997381	chr2:1997190-1997347
chr2	2053980	2054159	chr2:2053977-2054123
chr2	2172875	2173018	chr2:2172871-2172988
chr2	2284410	2284518	chr2:2284403-2284503
chr2	2303490	2304052	chr2:2303482-2304016
chr2	2305950	2306184	chr2:2305938-2306155
chr2	2324340	2324559	chr2:2324331-2324535
chr2	2326035	2326563	chr2:2326035-2326554
chr2	2330970	2331288	chr2:2330966-2331260
chr2	5693860	5694076	chr2:5693847-5694047
chr2	9951750	9951886	chr2:9951692-9951853

chr2	9952910	9953087	chr2:9952906-9953060
chr2	9954915	9955130	chr2:9954914-9955101
chr2	9958795	9958996	chr2:9958785-9958964
chr2	9961045	9961464	chr2:9961045-9961436
chr2	9962465	9962562	chr2:9962454-9962531
chr2	9963895	9964080	chr2:9963885-9964042
chr2	9964240	9964382	chr2:9964234-9964346
chr2	9965300	9965401	chr2:9965286-9965381
chr2	9968790	9969003	chr2:9968788-9968988
chr2	9986135	9986318	chr2:9986123-9986282
chr2	9990705	9990808	chr2:9990695-9990773
chr2	9992010	9992160	chr2:9992006-9992146
chr2	9993200	9993268	chr2:9993206-9993244
chr2	9995885	9995995	chr2:9995878-9995970
chr2	9996320	9996427	chr2:9996315-9996401
chr2	9998975	9999047	chr2:9998964-9999029
chr2	10000595	10001410	chr2:10000592-10002277
chr2	10001420	10002144	chr2:10000592-10002277
chr2	10002180	10002307	chr2:10000592-10002277
chr2	10042860	10043099	chr2:10042848-10043071
chr2	10043555	10043627	chr2:10043554-10043758
chr2	10043630	10043769	chr2:10043554-10043758
chr2	10043830	10043903	chr2:10043828-10043887
chr2	10044180	10044458	chr2:10044174-10044451
chr2	10046150	10046437	chr2:10046149-10046419
chr2	10047660	10048635	chr2:10047649-10048595
chr2	10049540	10049748	chr2:10049531-10049731
chr2	10052235	10052656	chr2:10052226-10054836
chr2	10052660	10052724	chr2:10052226-10054836
chr2	10052735	10054876	chr2:10052226-10054836
chr2	10129065	10129166	chr2:10129054-10129154
chr2	10129245	10129417	chr2:10129233-10129383
chr2	11444375	11445339	chr2:11444374-11446523
chr2	11445425	11445844	chr2:11444374-11446523
chr2	11445875	11446089	chr2:11444374-11446523
chr2	11446115	11446227	chr2:11444374-11446523
chr2	11446230	11446481	chr2:11444374-11446523
chr2	11447640	11447814	chr2:11447626-11447774
chr2	11450025	11450169	chr2:11450011-11450126
chr2	11451650	11451821	chr2:11451650-11451806
chr2	11453595	11453829	chr2:11453581-11453798
chr2	11455355	11455494	chr2:11455341-11455474
chr2	11457180	11457252	chr2:11457178-11457233
chr2	11458280	11458388	chr2:11458280-11458351
chr2	11459105	11459285	chr2:11459069-11459269
chr2	11465775	11466215	chr2:11465771-11466177
chr2	15940564	15940778	chr2:15940563-15940743
chr2	15941959	15942870	chr2:15941947-15942854
chr2	15943729	15943939	chr2:15943717-15943917
chr2	15945504	15946139	chr2:15945492-15947007
chr2	15946144	15947017	chr2:15945492-15947007
chr2	17816499	17817134	chr2:17816495-17817101
chr2	19351527	19351730	chr2:19351484-19352410
chr2	19351757	19351912	chr2:19351484-19352410
chr2	19351927	19352453	chr2:19351484-19352410
chr2	19353152	19353847	chr2:19353140-19353837
chr2	19357707	19357969	chr2:19357705-19357946
chr2	19358287	19358673	chr2:19358283-19358653
chr2	27130964	27131531	chr2:27130962-27131508
chr2	27131674	27131861	chr2:27131671-27131831
chr2	27132019	27132122	chr2:27132009-27132082
chr2	27132214	27132389	chr2:27132203-27132403
chr2	27132609	27132747	chr2:27132602-27132727

chr2	27132844	27132950	chr2:27132842-27132923
chr2	27133119	27133370	chr2:27133116-27133337
chr2	27133534	27133751	chr2:27133531-27133721
chr2	27134294	27134720	chr2:27134286-27134675
chr2	27255324	27255497	chr2:27255310-27255460
chr2	27256399	27256500	chr2:27256385-27256485
chr2	27265149	27265350	chr2:27265142-27265342
chr2	27377689	27378404	chr2:27377683-27378371
chr2	27378479	27379081	chr2:27378466-27379054
chr2	27380149	27380454	chr2:27380092-27380444
chr2	27380479	27380690	chr2:27380471-27380725
chr2	27380754	27380826	chr2:27380760-27380790
chr2	28392449	28392554	chr2:28392447-28392544
chr2	28392794	28392939	chr2:28392801-28392836;chr2:28392857-28393822
chr2	28392964	28393843	chr2:28392857-28393822
chr2	28395514	28395797	chr2:28395510-28395776
chr2	28402214	28402437	chr2:28402213-28402413
chr2	28404109	28404398	chr2:28404106-28404358
chr2	28408769	28408905	chr2:28408758-28408866
chr2	28411879	28415685	chr2:28411878-28417312
chr2	28415704	28416465	chr2:28411878-28417312
chr2	28416479	28416954	chr2:28411878-28417312
chr2	28416974	28417102	chr2:28411878-28417312
chr2	28417124	28417330	chr2:28411878-28417312
chr2	37211006	37211112	chr2:37210998-37211082
chr2	37211846	37212055	chr2:37211842-37212039
chr2	37212336	37212420	chr2:37212334-37212392
chr2	37213881	37213983	chr2:37213875-37213961
chr2	37214886	37214958	chr2:37214885-37214952
chr2	37216346	37216449	chr2:37216343-37216418
chr2	37216986	37217056	chr2:37216983-37217037
chr2	37220386	37220489	chr2:37220384-37220473
chr2	37221576	37221786	chr2:37221563-37221763
chr2	37222391	37222600	chr2:37222379-37222563
chr2	37223171	37223425	chr2:37223169-37223401
chr2	37227551	37228812	chr2:37227543-37229036
chr2	37228826	37229066	chr2:37227543-37229036
chr2	37231286	37231391	chr2:37231272-37231369
chr2	37231411	37231484	chr2:37231411-37231480
chr2	44942046	44942213	chr2:44941897-44942910
chr2	44942226	44942299	chr2:44941897-44942910
chr2	44942311	44942929	chr2:44941897-44942910
chr2	44943746	44943970	chr2:44943742-44943942
chr2	44944576	44944816	chr2:44944567-44946077
chr2	44944851	44945218	chr2:44944567-44946077
chr2	44945436	44945617	chr2:44944567-44946077
chr2	44945621	44945733	chr2:44944567-44946077
chr2	44945751	44946098	chr2:44944567-44946077
chr2	45005161	45005717	chr2:45005160-45006485
chr2	45005721	45005817	chr2:45005160-45006485
chr2	45005826	45005891	chr2:45005160-45006485
chr2	45005896	45006491	chr2:45005160-45006485
chr2	45007431	45007638	chr2:45007418-45007618
chr2	45008551	45009465	chr2:45008550-45009430
chr2	46293676	46294129	chr2:46293666-46294098
chr2	46296621	46296753	chr2:46296607-46296725
chr2	46297401	46297554	chr2:46297401-46297937
chr2	46297576	46297958	chr2:46297401-46297937
chr2	46325416	46325623	chr2:46325405-46325605
chr2	46335701	46335944	chr2:46335689-46335906
chr2	46346886	46347107	chr2:46346872-46347063
chr2	46347241	46347564	chr2:46347237-46347537
chr2	46356151	46356333	chr2:46356150-46356302

chr2	46356726	46356835	chr2:46356723-46356808
chr2	46360651	46360784	chr2:46360637-46360756
chr2	46360886	46361111	chr2:46360884-46361090
chr2	46369826	46369973	chr2:46369826-46369933
chr2	46375691	46375881	chr2:46375689-46375837
chr2	46376546	46376789	chr2:46376538-46376753
chr2	46377896	46378047	chr2:46377893-46378087
chr2	46378661	46378813	chr2:46378656-46378767
chr2	46380226	46380759	chr2:46380226-46380717
chr2	46381601	46381747	chr2:46381595-46381722
chr2	46381986	46382123	chr2:46381974-46382089
chr2	46382426	46382618	chr2:46382424-46382598
chr2	46384476	46385177	chr2:46384475-46386703
chr2	46385196	46385336	chr2:46384475-46386703
chr2	46385346	46385667	chr2:46384475-46386703
chr2	46385701	46386720	chr2:46384475-46386703
chr2	47354476	47354699	chr2:47354469-47354669
chr2	47385174	47385245	chr2:47385165-47385210
chr2	47386579	47386753	chr2:47386571-47386721
chr2	48314639	48314845	chr2:48314636-48314814
chr2	48328589	48328724	chr2:48328560-48328702
chr2	48330719	48330924	chr2:48330705-48330905
chr2	48346209	48346769	chr2:48346200-48346751
chr2	48359054	48359163	chr2:48359046-48359147
chr2	48362654	48362732	chr2:48362642-48362707
chr2	48373294	48373376	chr2:48373291-48373360
chr2	48374919	48375575	chr2:48374919-48379294
chr2	48375589	48375791	chr2:48374919-48379294
chr2	48375794	48375934	chr2:48374919-48379294
chr2	48375944	48376993	chr2:48374919-48379294
chr2	48377029	48378368	chr2:48374919-48379294
chr2	48378399	48378679	chr2:48374919-48379294
chr2	48378699	48378979	chr2:48374919-48379294
chr2	48378994	48379314	chr2:48374919-48379294
chr2	55867095	55867264	chr2:55867084-55867234
chr2	55870730	55870836	chr2:55870719-55870819
chr2	55899385	55899592	chr2:55899373-55899573
chr2	60452519	60452695	chr2:60452516-60452666
chr2	60460689	60460797	chr2:60460681-60460781
chr2	60462274	60462469	chr2:60462274-60462424
chr2	60468734	60468850	chr2:60468731-60468831
chr2	60487339	60487549	chr2:60487333-60487533
chr2	60541824	60541960	chr2:60541784-60541925
chr2	60545979	60546085	chr2:60545970-60546070
chr2	60881524	60881879	chr2:60881520-60881850
chr2	60891694	60891863	chr2:60891682-60891825
chr2	60893434	60893647	chr2:60893424-60893624
chr2	60894409	60894581	chr2:60894396-60894545
chr2	60901004	60901101	chr2:60900991-60901083
chr2	60916879	60917031	chr2:60916876-60917017
chr2	60918194	60918302	chr2:60918190-60918295
chr2	60918404	60918646	chr2:60918393-60918606
chr2	60920044	60920116	chr2:60920040-60920109
chr2	60920574	60920643	chr2:60920573-60920642
chr2	60921769	60922509	chr2:60921762-60931610
chr2	60922514	60922772	chr2:60921762-60931610
chr2	60923074	60923168	chr2:60921762-60931610
chr2	60923169	60924599	chr2:60921762-60931610
chr2	60924604	60925376	chr2:60921762-60931610
chr2	60925394	60925537	chr2:60921762-60931610
chr2	60925564	60925701	chr2:60921762-60931610
chr2	60925714	60928055	chr2:60921762-60931610
chr2	60929999	60931634	chr2:60921762-60931610

chr2	63050057	63050162	chr2:63050056-63050152
chr2	63050812	63050915	chr2:63050801-63050897
chr2	63051252	63051328	chr2:63051248-63051317
chr2	63052892	63053097	chr2:63052879-63053087
chr2	63053937	63054220	chr2:63053931-63054198
chr2	63054752	63054963	chr2:63054749-63054949
chr2	63055502	63056118	chr2:63055500-63057836
chr2	63056147	63057135	chr2:63055500-63057836
chr2	63057182	63057482	chr2:63055500-63057836
chr2	63057537	63057856	chr2:63055500-63057836
chr2	66433454	66433600	chr2:66433451-66433567
chr2	66435144	66435275	chr2:66435132-66435868
chr2	66435324	66435535	chr2:66435132-66435868
chr2	66435574	66435745	chr2:66435132-66435868
chr2	66435759	66435887	chr2:66435132-66435868
chr2	66437449	66437991	chr2:66437392-66437963
chr2	66439334	66439677	chr2:66439320-66439671
chr2	66439849	66440021	chr2:66439842-66439984
chr2	66440099	66440384	chr2:66440096-66440345
chr2	66440564	66440633	chr2:66440561-66440612
chr2	66440679	66440995	chr2:66440677-66441007
chr2	66441414	66441487	chr2:66441413-66441464
chr2	66442914	66443084	chr2:66442901-66443048
chr2	66445194	66445308	chr2:66445192-66445296
chr2	66454709	66454824	chr2:66454708-66454794
chr2	66461799	66461918	chr2:66461797-66461887
chr2	66464114	66464260	chr2:66464108-66464220
chr2	66493189	66493405	chr2:66493182-66493382
chr2	66496034	66496217	chr2:66496025-66496200
chr2	66508929	66509045	chr2:66508926-66509123
chr2	66509054	66509155	chr2:66508926-66509123
chr2	66512154	66512334	chr2:66512148-66512294
chr2	66547949	66548052	chr2:66547942-66548019
chr2	66562079	66562151	chr2:66562081-66562119
chr2	66567454	66568097	chr2:66567452-66568536
chr2	66568134	66568560	chr2:66567452-66568536
chr2	66568669	66570807	chr2:66568666-66573869
chr2	66570824	66571204	chr2:66568666-66573869
chr2	66571219	66572009	chr2:66568666-66573869
chr2	66572029	66572723	chr2:66568666-66573869
chr2	66572739	66573893	chr2:66568666-66573869
chr2	69897696	69897774	chr2:69897687-69897742
chr2	69915051	69915436	chr2:69915040-69915403
chr2	69916121	69916231	chr2:69916120-69916220
chr2	69918791	69919000	chr2:69918780-69918980
chr2	69921731	69921806	chr2:69921735-69921765
chr2	69935356	69935493	chr2:69935350-69935465
chr2	69937031	69937414	chr2:69937018-69937394
chr2	69938096	69939990	chr2:69938096-69942945
chr2	69939991	69940087	chr2:69938096-69942945
chr2	69940166	69941575	chr2:69938096-69942945
chr2	69941591	69941862	chr2:69938096-69942945
chr2	69941881	69941979	chr2:69938096-69942945
chr2	69941986	69942964	chr2:69938096-69942945
chr2	70777321	70777415	chr2:70777309-70777382
chr2	70777631	70777705	chr2:70777636-70777671
chr2	70779726	70779945	chr2:70779726-70779926
chr2	70785416	70785669	chr2:70785414-70785639
chr2	70787661	70787833	chr2:70787648-70787801
chr2	70790416	70790667	chr2:70790407-70790643
chr2	70900601	70900875	chr2:70900589-70900868
chr2	70905006	70905217	chr2:70904996-70905196
chr2	70921101	70921321	chr2:70921097-70921285

chr2	70932766	70933380	chr2:70932766-70933446
chr2	70933391	70933467	chr2:70932766-70933446
chr2	71188396	71188562	chr2:71188385-71188535
chr2	71189846	71189962	chr2:71189844-71189944
chr2	71191111	71191326	chr2:71191108-71191308
chr2	72916271	72917069	chr2:72916259-72917060
chr2	72917476	72918404	chr2:72917474-72918372
chr2	72921311	72921532	chr2:72921310-72921510
chr2	72923416	72923751	chr2:72923413-72923721
chr2	72924321	72924527	chr2:72924308-72924493
chr2	72925441	72925562	chr2:72925438-72925541
chr2	72933796	72934924	chr2:72933786-72934891
chr2	73290931	73291005	chr2:73290928-73292781
chr2	73291021	73292831	chr2:73290928-73292781
chr2	73293191	73293718	chr2:73293181-73293705
chr2	74513470	74513780	chr2:74513462-74513745
chr2	74514005	74514263	chr2:74513996-74514234
chr2	74514485	74515222	chr2:74514483-74515206
chr2	74515640	74515885	chr2:74515632-74515870
chr2	74515975	74517177	chr2:74515972-74517147
chr2	75653235	75653445	chr2:75653226-75653426
chr2	75662705	75662912	chr2:75662705-75664783
chr2	75662920	75663583	chr2:75662705-75664783
chr2	75663880	75664783	chr2:75662705-75664783
chr2	75665935	75666078	chr2:75665928-75666053
chr2	75670150	75670316	chr2:75670137-75670284
chr2	75671955	75672029	chr2:75671949-75672016
chr2	75673445	75673553	chr2:75673443-75673520
chr2	75680205	75680344	chr2:75680192-75680314
chr2	75687840	75688004	chr2:75687826-75687977
chr2	75689030	75689246	chr2:75689025-75689225
chr2	75689975	75690109	chr2:75689968-75690081
chr2	75691980	75692139	chr2:75691976-75692100
chr2	75694250	75694419	chr2:75694240-75694427
chr2	75696210	75696340	chr2:75696199-75696315
chr2	75701195	75701298	chr2:75701189-75701287
chr2	75701800	75702431	chr2:75701795-75702423
chr2	75706535	75706671	chr2:75706522-75706651
chr2	75710445	75711025	chr2:75710443-75710989
chr2	85133418	85133692	chr2:85133409-85133933
chr2	85133728	85133964	chr2:85133409-85133933
chr2	85134028	85134100	chr2:85134015-85134079
chr2	85134333	85134465	chr2:85134322-85134450
chr2	85167083	85167300	chr2:85166919-85167373
chr2	85213623	85213732	chr2:85213622-85213693
chr2	85217028	85217238	chr2:85217019-85217206
chr2	85233798	85233918	chr2:85233797-85233888
chr2	85237308	85237461	chr2:85237237-85237437
chr2	85283503	85283617	chr2:85283494-85283578
chr2	85302484	85302629	chr2:85302483-85302616
chr2	85303899	85304004	chr2:85303894-85303997
chr2	85304264	85304360	chr2:85304254-85304338
chr2	85305259	85305438	chr2:85305259-85305403
chr2	85306214	85306388	chr2:85306205-85306365
chr2	85306454	85306600	chr2:85306451-85306559
chr2	85307664	85307745	chr2:85307641-85307717
chr2	85309029	85309653	chr2:85309028-85310388
chr2	85309659	85310297	chr2:85309028-85310388
chr2	85310299	85310397	chr2:85309028-85310388
chr2	86815569	86815748	chr2:86815568-86815718
chr2	86822324	86822404	chr2:86822320-86822374
chr2	86837629	86837836	chr2:86837617-86837817
chr2	86842179	86842348	chr2:86842169-86842319

chr2	86844919	86844992	chr2:86844921-86844958
chr2	86853009	86853120	chr2:86852996-86853086
chr2	95297309	95297488	chr2:95297303-95297453
chr2	95298694	95299155	chr2:95298682-95299120
chr2	95303054	95303538	chr2:95303043-95303503
chr2	95310359	95310540	chr2:95310354-95310520
chr2	95323379	95323603	chr2:95323376-95323576
chr2	95346734	95346833	chr2:95346727-95347122
chr2	95346839	95347150	chr2:95346727-95347122
chr2	95355039	95355305	chr2:95355036-95355282
chr2	95374304	95374441	chr2:95374295-95374420
chr2	95374849	95374921	chr2:95374847-95374917
chr2	95375144	95375216	chr2:95375137-95375208
chr2	95381609	95381709	chr2:95381595-95381703
chr2	95382389	95382498	chr2:95382376-95382481
chr2	95383234	95383306	chr2:95383231-95383294
chr2	95384004	95384147	chr2:95384001-95386083
chr2	95384194	95386110	chr2:95384001-95386083
chr2	96536749	96536859	chr2:96536748-96536830
chr2	96537344	96538333	chr2:96537344-96538321
chr2	96542169	96542382	chr2:96542155-96542355
chr2	96547414	96547554	chr2:96547401-96547517
chr2	96549349	96549487	chr2:96549320-96549459
chr2	96549764	96549841	chr2:96549752-96549805
chr2	96550189	96550309	chr2:96550187-96550285
chr2	96550579	96550754	chr2:96550573-96550733
chr2	96551099	96552646	chr2:96551098-96552638
chr2	100820151	100820258	chr2:100820151-100820414
chr2	100820261	100820421	chr2:100820151-100820414
chr2	100821026	100821212	chr2:100821024-100821199
chr2	100863331	100863554	chr2:100863322-100863522
chr2	100904736	100904821	chr2:100904732-100904786
chr2	100925146	100925319	chr2:100925145-100925300
chr2	100932921	100933024	chr2:100932909-100933001
chr2	100934121	100934196	chr2:100934110-100934160
chr2	100937766	100937873	chr2:100937752-100937842
chr2	100948236	100948374	chr2:100948234-100948355
chr2	100949366	100949513	chr2:100949366-100949480
chr2	100962761	100962879	chr2:100962754-100962833
chr2	100964071	100964218	chr2:100964057-100964176
chr2	100964861	100964970	chr2:100964860-100964943
chr2	100965661	100965810	chr2:100965659-100965766
chr2	100968291	100968469	chr2:100968280-100968428
chr2	100970996	100971106	chr2:100970989-100971074
chr2	100974816	100974957	chr2:100974802-100974944
chr2	100975461	100975603	chr2:100975457-100975567
chr2	100977721	100977829	chr2:100977709-100977799
chr2	100982241	100982414	chr2:100982230-100982377
chr2	100988081	100988314	chr2:100988078-100988276
chr2	100990256	100990486	chr2:100990255-100990446
chr2	100990791	100990889	chr2:100990779-100990872
chr2	100993356	100993563	chr2:100993346-100993527
chr2	100994451	100994591	chr2:100994442-100994580
chr2	100995401	100996038	chr2:100995399-100996829
chr2	100996126	100996858	chr2:100995399-100996829
chr2	104855510	104855621	chr2:104855510-104858574
chr2	104855635	104855814	chr2:104855510-104858574
chr2	104855845	104856080	chr2:104855510-104858574
chr2	104856120	104856226	chr2:104855510-104858574
chr2	104856235	104856309	chr2:104855510-104858574
chr2	104856330	104856402	chr2:104855510-104858574
chr2	104856410	104857035	chr2:104855510-104858574
chr2	104857110	104857396	chr2:104855510-104858574

chr2	104857405	104857515	chr2:104855510-104858574
chr2	104857535	104858084	chr2:104855510-104858574
chr2	104858085	104858603	chr2:104855510-104858574
chr2	110638013	110638198	chr2:110638009-110638159
chr2	110639753	110639865	chr2:110639741-110639841
chr2	110668628	110668840	chr2:110668617-110668817
chr2	112211531	112211743	chr2:112211524-112216468
chr2	112211756	112212027	chr2:112211524-112216468
chr2	112212071	112212148	chr2:112211524-112216468
chr2	112212231	112212973	chr2:112211524-112216468
chr2	112213261	112213501	chr2:112211524-112216468
chr2	112213801	112213939	chr2:112211524-112216468
chr2	112214236	112214471	chr2:112211524-112216468
chr2	112214486	112215173	chr2:112211524-112216468
chr2	112215181	112216506	chr2:112211524-112216468
chr2	112230706	112230962	chr2:112230702-112230950
chr2	112231841	112231980	chr2:112231837-112231947
chr2	112233261	112233396	chr2:112233257-112233371
chr2	112234131	112234234	chr2:112234119-112234236
chr2	112236566	112236673	chr2:112236561-112236695
chr2	112238341	112238543	chr2:112238314-112238528
chr2	112240381	112240524	chr2:112240367-112240492
chr2	112250201	112250303	chr2:112250190-112250272
chr2	112251221	112251432	chr2:112251214-112251414
chr2	112254011	112254110	chr2:112253969-112254099
chr2	112254901	112255157	chr2:112254899-112255111
chr2	113216001	113216874	chr2:113215996-113218609
chr2	113216891	113218012	chr2:113215996-113218609
chr2	113218031	113218625	chr2:113215996-113218609
chr2	113220101	113220202	chr2:113220091-113220178
chr2	113227156	113227295	chr2:113227098-113227256
chr2	113235396	113235617	chr2:113235393-113235582
chr2	113236611	113236760	chr2:113236600-113236721
chr2	113241551	113241807	chr2:113241550-113241779
chr2	113242016	113242152	chr2:113242007-113242130
chr2	113242701	113242812	chr2:113242689-113242778
chr2	113244426	113244651	chr2:113244426-113244624
chr2	113246761	113246941	chr2:113246753-113246919
chr2	113249301	113249509	chr2:113249289-113249489
chr2	113278381	113278591	chr2:113278369-113278566
chr2	113278841	113278972	chr2:113278830-113278950
chr2	113498676	113499580	chr2:113498664-113501155
chr2	113499596	113499698	chr2:113498664-113501155
chr2	113499746	113501165	chr2:113498664-113501155
chr2	118842171	118842526	chr2:118842170-118843254
chr2	118842556	118843226	chr2:118842170-118843254
chr2	118843936	118844314	chr2:118843923-118844352
chr2	118844316	118844384	chr2:118843923-118844352
chr2	118845236	118845452	chr2:118845229-118845429
chr2	118846306	118846522	chr2:118846305-118847678
chr2	118846566	118847420	chr2:118846305-118847678
chr2	118847426	118847497	chr2:118846305-118847678
chr2	118847536	118847717	chr2:118846305-118847678
chr2	120735626	120735692	chr2:120735622-120735681
chr2	120736241	120736316	chr2:120736246-120736285
chr2	120777626	120777843	chr2:120777613-120777813
chr2	120792286	120792473	chr2:120792282-120792438
chr2	120797291	120797510	chr2:120797290-120797468
chr2	120927371	120927514	chr2:120927360-120927466
chr2	120951246	120951508	chr2:120951242-120951466
chr2	120955256	120955461	chr2:120955244-120955430
chr2	120968716	120968935	chr2:120968713-120968915
chr2	120970406	120970654	chr2:120970392-120970606

chr2	120971946	120972088	chr2:120971940-120972063
chr2	120974926	120975006	chr2:120974923-120975109
chr2	120975031	120975139	chr2:120974923-120975109
chr2	120978436	120978619	chr2:120978433-120978583
chr2	120982726	120982893	chr2:120982715-120982880
chr2	120984471	120984765	chr2:120984470-120984743
chr2	120986291	120986630	chr2:120986277-120986614
chr2	120988216	120991377	chr2:120988207-120992653
chr2	120991396	120991999	chr2:120988207-120992653
chr2	120992046	120992670	chr2:120988207-120992653
chr2	121216596	121218264	chr2:121216586-121224387
chr2	121218301	121219658	chr2:121216586-121224387
chr2	121219761	121221397	chr2:121216586-121224387
chr2	121221411	121221592	chr2:121216586-121224387
chr2	121221601	121221945	chr2:121216586-121224387
chr2	121221976	121222084	chr2:121216586-121224387
chr2	121222091	121222200	chr2:121216586-121224387
chr2	121222246	121222316	chr2:121216586-121224387
chr2	121222541	121224425	chr2:121216586-121224387
chr2	121225571	121225650	chr2:121225561-121225613
chr2	121231826	121232006	chr2:121231825-121231968
chr2	121234101	121234217	chr2:121234090-121234194
chr2	121235221	121235331	chr2:121235220-121235311
chr2	121237626	121237747	chr2:121237622-121237716
chr2	121237811	121237883	chr2:121237801-121237850
chr2	121239571	121239688	chr2:121239557-121239649
chr2	121242361	121242507	chr2:121242358-121242469
chr2	121246831	121247008	chr2:121246817-121246970
chr2	121248166	121248279	chr2:121248163-121248270
chr2	121248991	121249126	chr2:121248981-121249087
chr2	121249576	121249677	chr2:121249570-121249647
chr2	121281121	121281309	chr2:121281119-121281271
chr2	121283046	121283262	chr2:121283042-121283242
chr2	121285051	121285238	chr2:121285047-121285207
chr2	138671526	138671698	chr2:138671515-138671665
chr2	138741141	138741375	chr2:138741138-138741338
chr2	138780201	138780309	chr2:138780190-138780290
chr2	144388732	144388884	chr2:144388732-144388882
chr2	144389607	144389792	chr2:144389603-144389753
chr2	144389867	144390044	chr2:144389865-144390028
chr2	144396412	144396532	chr2:144396411-144396511
chr2	144401212	144401314	chr2:144401198-144401298
chr2	144452717	144452791	chr2:144452714-144452914
chr2	144452807	144452937	chr2:144452714-144452914
chr2	144513657	144513837	chr2:144513647-144513797
chr2	144517287	144517411	chr2:144517277-144517377
chr2	156324433	156325074	chr2:156324431-156326000
chr2	156325093	156325308	chr2:156324431-156326000
chr2	156325348	156325582	chr2:156324431-156326000
chr2	156325583	156326039	chr2:156324431-156326000
chr2	156326163	156326364	chr2:156326149-156326328
chr2	156326718	156326937	chr2:156326717-156326920
chr2	156327853	156328027	chr2:156327850-156328014
chr2	156328403	156328548	chr2:156328403-156328533
chr2	156329323	156330219	chr2:156329322-156330188
chr2	156330668	156330817	chr2:156330667-156330791
chr2	156331783	156332002	chr2:156331783-156331965
chr2	156332483	156332747	chr2:156332479-156332720
chr2	156333663	156333871	chr2:156333657-156333857
chr2	156342043	156342353	chr2:156342039-156342348
chr2	158533223	158533342	chr2:158533216-158533316
chr2	158533428	158533507	chr2:158533437-158533467
chr2	158653493	158653692	chr2:158653479-158653679

chr2	158678583	158678696	chr2:158678580-158678654
chr2	158680428	158680791	chr2:158680428-158680674;chr2:158680677-158680827
chr2	161416099	161416273	chr2:161416093-161417102
chr2	161416274	161417115	chr2:161416093-161417102
chr2	161417689	161417863	chr2:161417675-161417830
chr2	161417894	161418075	chr2:161417894-161418087
chr2	161418209	161418351	chr2:161418200-161418322
chr2	161418899	161419071	chr2:161418891-161419050
chr2	161419209	161419318	chr2:161419209-161419291
chr2	161419519	161419689	chr2:161419510-161419919
chr2	161419694	161419932	chr2:161419510-161419919
chr2	161420209	161420278	chr2:161420195-161420257
chr2	161423369	161424702	chr2:161423368-161425870
chr2	161424704	161424839	chr2:161423368-161425870
chr2	161424869	161425884	chr2:161423368-161425870
chr2	165469659	165469757	chr2:165469646-165469740
chr2	165494759	165494947	chr2:165494758-165494928
chr2	165517879	165517993	chr2:165517872-165517961
chr2	165566784	165567004	chr2:165566776-165566981
chr2	165572349	165572619	chr2:165572338-165572587
chr2	165574114	165574470	chr2:165574108-165574431
chr2	165577329	165577546	chr2:165577316-165577516
chr2	165595044	165595234	chr2:165595042-165595213
chr2	165657774	165658038	chr2:165657760-165658020
chr2	165676314	165676642	chr2:165676311-165676608
chr2	165678714	165678957	chr2:165678700-165680980
chr2	165679029	165679111	chr2:165678700-165680980
chr2	165679139	165680680	chr2:165678700-165680980
chr2	165680704	165681011	chr2:165678700-165680980
chr2	170715350	170715594	chr2:170715350-170715563
chr2	170716270	170718093	chr2:170716258-170718078
chr2	172085890	172086001	chr2:172085890-172085990
chr2	172086765	172086868	chr2:172086753-172086853
chr2	172088005	172088182	chr2:172088002-172088152
chr2	172099450	172099612	chr2:172099438-172101646
chr2	172099620	172100584	chr2:172099438-172101646
chr2	172100590	172101668	chr2:172099438-172101646
chr2	172102145	172102290	chr2:172102138-172102900
chr2	172102315	172102388	chr2:172102138-172102900
chr2	172102410	172102941	chr2:172102138-172102900
chr2	173906459	173907407	chr2:173906458-173910257
chr2	173907409	173908528	chr2:173906458-173910257
chr2	173908539	173908776	chr2:173906458-173910257
chr2	173908779	173908911	chr2:173906458-173910257
chr2	173908914	173909710	chr2:173906458-173910257
chr2	173909714	173910267	chr2:173906458-173910257
chr2	173913079	173913282	chr2:173913069-173913266
chr2	173918594	173918806	chr2:173918592-173918785
chr2	173954884	173956248	chr2:173954872-173956232
chr2	173957384	173957596	chr2:173957375-173957575
chr2	173962979	173963395	chr2:173962971-173963379
chr2	173963774	173964022	chr2:173963760-173964219
chr2	173964029	173964231	chr2:173963760-173964219
chr2	173964404	173964577	chr2:173964404-173964553
chr2	173964844	173964925	chr2:173964850-173964885
chr2	173965164	173965738	chr2:173965164-173965702
chr2	174334954	174335145	chr2:174334945-174335113
chr2	174336119	174337107	chr2:174336106-174338492
chr2	174337199	174337437	chr2:174336106-174338492
chr2	174337484	174338066	chr2:174336106-174338492
chr2	174338074	174338518	chr2:174336106-174338492
chr2	175072259	175072775	chr2:175072249-175074867
chr2	175072809	175073456	chr2:175072249-175074867

chr2	175073489	175073766	chr2:175072249-175074867
chr2	175073779	175073913	chr2:175072249-175074867
chr2	175073934	175074484	chr2:175072249-175074867
chr2	175074504	175074853	chr2:175072249-175074867
chr2	175075004	175075109	chr2:175074999-175075087
chr2	175080659	175080805	chr2:175080659-175080765
chr2	175093074	175093281	chr2:175093060-175093267
chr2	175097449	175097622	chr2:175097443-175097593
chr2	175111569	175111680	chr2:175111567-175111654
chr2	175113999	175114133	chr2:175113993-175114108
chr2	175114254	175114886	chr2:175114242-175114868
chr2	175117999	175118136	chr2:175117989-175118118
chr2	175118259	175118401	chr2:175118250-175118369
chr2	175119674	175119835	chr2:175119660-175119860
chr2	175121444	175121554	chr2:175121443-175121540
chr2	175126799	175126985	chr2:175126792-175127058
chr2	175130139	175130218	chr2:175130137-175130207
chr2	175132694	175132768	chr2:175132694-175132757
chr2	175136414	175136519	chr2:175136411-175136486
chr2	175147944	175148022	chr2:175147945-175147980
chr2	175151059	175151167	chr2:175151059-175151158
chr2	175168049	175168236	chr2:175168049-175168206
chr2	175168299	175168408	chr2:175168285-175168382
chr2	176092899	176092970	chr2:176092890-176093671
chr2	176092984	176093057	chr2:176092890-176093671
chr2	176093094	176093677	chr2:176092890-176093671
chr2	176094489	176095074	chr2:176094479-176095938
chr2	176095094	176095170	chr2:176094479-176095938
chr2	176095189	176095960	chr2:176094479-176095938
chr2	176104219	176104640	chr2:176104215-176104626
chr2	176105429	176105642	chr2:176105417-176105617
chr2	176107289	176107575	chr2:176107285-176108136
chr2	176107589	176108170	chr2:176107285-176108136
chr2	176108789	176109779	chr2:176108789-176109754
chr2	176111389	176111531	chr2:176111382-176111516
chr2	176114224	176114328	chr2:176114219-176114419
chr2	176114329	176114438	chr2:176114219-176114419
chr2	176116579	176117601	chr2:176116578-176117578
chr2	176118954	176119425	chr2:176118953-176119942
chr2	176119459	176119571	chr2:176118953-176119942
chr2	176119574	176119706	chr2:176118953-176119942
chr2	176119709	176119954	chr2:176118953-176119942
chr2	176122729	176123625	chr2:176122719-176123585
chr2	176123944	176124292	chr2:176123933-176124937
chr2	176124299	176124965	chr2:176123933-176124937
chr2	176129699	176130428	chr2:176129693-176130943
chr2	176130429	176130958	chr2:176129693-176130943
chr2	176131319	176132016	chr2:176131316-176132695
chr2	176132024	176132719	chr2:176131316-176132695
chr2	176136614	176137036	chr2:176136611-176136999
chr2	176139009	176139666	chr2:176138996-176139663
chr2	176146669	176146891	chr2:176146669-176146869
chr2	176152614	176152766	chr2:176152607-176153226
chr2	176152784	176153245	chr2:176152607-176153226
chr2	176160904	176161110	chr2:176160890-176161080
chr2	176169044	176169687	chr2:176169030-176169655
chr2	176171529	176173141	chr2:176171516-176173102
chr2	176188584	176189457	chr2:176188578-176189453
chr2	176189809	176190938	chr2:176189807-176190907
chr2	177227604	177227848	chr2:177227594-177227953
chr2	177230359	177232031	chr2:177230304-177232008
chr2	177232279	177232396	chr2:177232276-177233339
chr2	177232404	177233372	chr2:177232276-177233339

chr2	177234009	177234290	chr2:177234004-177234271
chr2	177263414	177263820	chr2:177263402-177263800
chr2	177264534	177265169	chr2:177264531-177265131
chr2	177283609	177283814	chr2:177283599-177283804
chr2	177287624	177287798	chr2:177287610-177287777
chr2	177323239	177323401	chr2:177323238-177323373
chr2	177334394	177334541	chr2:177334392-177334529
chr2	177338599	177338722	chr2:177338597-177338699
chr2	177377154	177377367	chr2:177377154-177377354
chr2	181673099	181673584	chr2:181673087-181673547
chr2	181675164	181675399	chr2:181675161-181675361
chr2	181676474	181677107	chr2:181676466-181678871
chr2	181677149	181677394	chr2:181676466-181678871
chr2	181677419	181677550	chr2:181676466-181678871
chr2	181677569	181678647	chr2:181676466-181678871
chr2	181678649	181678901	chr2:181676466-181678871
chr2	181680429	181680895	chr2:181680429-181680876
chr2	184598374	184599068	chr2:184598365-184599070
chr2	184806844	184807057	chr2:184806833-184807033
chr2	184866374	184866550	chr2:184866364-184866512
chr2	184933604	184933756	chr2:184933602-184933733
chr2	184935784	184937183	chr2:184935782-184939492
chr2	184937189	184939517	chr2:184935782-184939492
chr2	190908203	190908414	chr2:190908197-190908397
chr2	190931553	190931654	chr2:190931544-190931637
chr2	190932723	190932895	chr2:190932715-190932865
chr2	190954768	190954836	chr2:190954754-190954818
chr2	190962843	190963009	chr2:190962829-190962979
chr2	190964358	190964724	chr2:190964357-190964694
chr2	190969043	190970752	chr2:190969033-190970717
chr2	190974838	190974942	chr2:190974829-190974935
chr2	190975318	190975425	chr2:190975309-190975388
chr2	190975538	190975918	chr2:190975536-190975887
chr2	190976843	190977057	chr2:190976839-190977025
chr2	190978858	190979048	chr2:190978855-190979001
chr2	190979773	190979879	chr2:190979771-190979866
chr2	190980623	190980690	chr2:190980619-190980669
chr2	190982393	190982541	chr2:190982382-190982518
chr2	190983653	190983754	chr2:190983641-190983740
chr2	190984313	190984418	chr2:190984309-190984393
chr2	190985618	190985693	chr2:190985618-190985660
chr2	190986863	190986964	chr2:190986853-190986947
chr2	190989618	190989690	chr2:190989614-190989674
chr2	190991228	190991352	chr2:190991227-190991320
chr2	190995118	190995265	chr2:190995060-190995219
chr2	190996258	190996467	chr2:190996244-190996444
chr2	190997868	190998036	chr2:190997855-190998007
chr2	190998228	190998332	chr2:190998216-190998308
chr2	190999628	190999744	chr2:190999625-190999704
chr2	191001073	191001197	chr2:191001073-191001163
chr2	191007573	191007681	chr2:191007562-191007661
chr2	191008973	191009146	chr2:191008962-191009107
chr2	191009878	191010024	chr2:191009875-191010004
chr2	191010378	191010486	chr2:191010366-191010449
chr2	191013538	191013987	chr2:191013524-191013955
chr2	191014028	191014273	chr2:191014017-191014250
chr2	191020203	191020345	chr2:191020199-191020320
chr2	191020758	191020976	chr2:191020757-191020960
chr2	191029588	191029869	chr2:191029575-191029866
chr2	191030973	191031120	chr2:191030971-191031080
chr2	191031463	191031529	chr2:191031449-191031516
chr2	191032963	191033167	chr2:191032957-191033149
chr2	191033498	191033635	chr2:191033489-191033626

chr2	191033923	191034032	chr2:191033910-191034005
chr2	191034558	191034635	chr2:191034547-191034597
chr2	191036173	191036309	chr2:191036163-191036299
chr2	191039198	191039314	chr2:191039198-191039297
chr2	191041068	191041190	chr2:191041064-191041148
chr2	191041483	191041683	chr2:191041473-191041683
chr2	191054488	191054557	chr2:191054489-191054534
chr2	191058023	191058133	chr2:191058017-191058111
chr2	191058713	191058786	chr2:191058709-191058769
chr2	191061733	191061840	chr2:191061728-191061821
chr2	191062763	191062941	chr2:191062761-191062920
chr2	191064808	191064987	chr2:191064806-191064958
chr2	191066433	191066561	chr2:191066429-191066515
chr2	191069693	191069807	chr2:191069692-191069771
chr2	191073098	191073204	chr2:191073097-191073190
chr2	191076233	191076334	chr2:191076226-191076325
chr2	191127943	191128160	chr2:191127941-191128141
chr2	191131493	191131565	chr2:191131490-191131900
chr2	191131568	191131922	chr2:191131490-191131900
chr2	191146613	191146757	chr2:191146612-191146757
chr2	191148078	191148223	chr2:191148075-191148204
chr2	191150953	191151268	chr2:191150946-191151260
chr2	191151343	191151625	chr2:191151333-191151596
chr2	197728432	197728616	chr2:197728424-197728578
chr2	197743072	197743177	chr2:197743060-197743159
chr2	197780162	197780362	chr2:197780149-197780349
chr2	199269505	199269903	chr2:199269499-199272672
chr2	199269905	199270362	chr2:199269499-199272672
chr2	199270370	199270508	chr2:199269499-199272672
chr2	199270520	199271107	chr2:199269499-199272672
chr2	199271110	199271465	chr2:199269499-199272672
chr2	199271470	199271565	chr2:199269499-199272672
chr2	199271570	199271776	chr2:199269499-199272672
chr2	199271810	199272085	chr2:199269499-199272672
chr2	199272135	199272692	chr2:199269499-199272672
chr2	199308770	199308988	chr2:199308759-199308957
chr2	199323810	199323980	chr2:199323802-199323958
chr2	199328710	199328947	chr2:199328697-199328910
chr2	199348710	199349190	chr2:199348700-199349173
chr2	199368615	199368719	chr2:199368604-199368707
chr2	199380370	199380520	chr2:199380363-199380487
chr2	199381700	199381843	chr2:199381693-199381820
chr2	199395840	199396871	chr2:199395830-199397006
chr2	199396880	199397017	chr2:199395830-199397006
chr2	199397540	199397611	chr2:199397529-199397840
chr2	199423685	199423953	chr2:199423685-199423915
chr2	199433350	199433532	chr2:199433337-199433514
chr2	199448770	199448981	chr2:199448760-199448960
chr2	199455880	199456112	chr2:199455868-199456096
chr2	199457345	199457555	chr2:199457338-199458745
chr2	199457570	199457733	chr2:199457338-199458745
chr2	199457740	199458779	chr2:199457338-199458745
chr2	199460460	199460561	chr2:199460451-199460532
chr2	199464845	199465089	chr2:199464835-199465167
chr2	199465100	199465197	chr2:199464835-199465167
chr2	199471015	199471304	chr2:199471013-199471266
chr2	202912601	202913136	chr2:202912600-202913114
chr2	202917886	202918051	chr2:202917876-202918043
chr2	202924351	202924450	chr2:202924296-202924415
chr2	202941871	202942004	chr2:202941859-202941980
chr2	202942746	202942992	chr2:202942739-202942967
chr2	202944621	202944794	chr2:202944617-202944817
chr2	202952571	202952702	chr2:202952558-202952679

chr2	202954006	202954154	chr2:202954004-202954134
chr2	202955681	202955795	chr2:202955673-202955758
chr2	202961241	202962186	chr2:202961236-202963168
chr2	202962501	202962670	chr2:202961236-202963168
chr2	202962731	202963048	chr2:202961236-202963168
chr2	202966981	202967123	chr2:202966977-202967098
chr2	202969931	202970095	chr2:202969918-202970062
chr2	202971506	202971758	chr2:202971504-202971738
chr2	202973491	202973591	chr2:202973481-202973584
chr2	202974341	202974521	chr2:202974333-202974496
chr2	202977271	202977347	chr2:202977268-202977332
chr2	202981556	202981700	chr2:202981554-202981685
chr2	202982081	202982466	chr2:202982071-202982441
chr2	202983516	202983960	chr2:202983505-202987063
chr2	202984041	202984225	chr2:202983505-202987063
chr2	202984241	202984386	chr2:202983505-202987063
chr2	202984391	202984784	chr2:202983505-202987063
chr2	202985071	202985164	chr2:202983505-202987063
chr2	202985171	202986867	chr2:202983505-202987063
chr2	202986896	202986969	chr2:202983505-202987063
chr2	202986986	202987097	chr2:202983505-202987063
chr2	207081120	207081296	chr2:207081114-207081264
chr2	207088470	207088575	chr2:207088457-207088557
chr2	207132190	207132403	chr2:207132190-207132390
chr2	207529750	207530170	chr2:207529736-207530134
chr2	207534610	207534708	chr2:207534601-207534683
chr2	207547195	207547405	chr2:207547182-207547382
chr2	207550260	207550334	chr2:207550260-207550595
chr2	207550350	207550616	chr2:207550260-207550595
chr2	207555630	207555783	chr2:207555627-207555749
chr2	207559125	207559275	chr2:207559121-207559255
chr2	207560225	207560407	chr2:207560225-207560372
chr2	207561115	207561183	chr2:207561118-207561160
chr2	207567475	207567570	chr2:207567462-207567563
chr2	207570180	207570360	chr2:207570178-207570321
chr2	207571735	207571816	chr2:207571733-207571777
chr2	207575285	207575488	chr2:207575271-207575454
chr2	207576630	207576705	chr2:207576630-207576693
chr2	207577120	207577253	chr2:207577109-207577224
chr2	207577505	207578103	chr2:207577504-207578065
chr2	207596915	207597124	chr2:207596913-207603431
chr2	207597130	207597365	chr2:207596913-207603431
chr2	207597370	207598485	chr2:207596913-207603431
chr2	207598500	207598568	chr2:207596913-207603431
chr2	207598850	207599692	chr2:207596913-207603431
chr2	207599700	207600220	chr2:207596913-207603431
chr2	207600265	207600440	chr2:207596913-207603431
chr2	207600450	207602223	chr2:207596913-207603431
chr2	207602245	207603444	chr2:207596913-207603431
chr2	212999702	212999943	chr2:212999690-213008084
chr2	212999972	213000246	chr2:212999690-213008084
chr2	213000277	213000338	chr2:212999690-213008084
chr2	213000357	213000592	chr2:212999690-213008084
chr2	213000597	213000776	chr2:212999690-213008084
chr2	213000782	213001058	chr2:212999690-213008084
chr2	213001072	213001905	chr2:212999690-213008084
chr2	213001952	213002885	chr2:212999690-213008084
chr2	213002892	213003353	chr2:212999690-213008084
chr2	213003357	213004164	chr2:212999690-213008084
chr2	213004172	213005273	chr2:212999690-213008084
chr2	213005312	213006010	chr2:212999690-213008084
chr2	213006022	213007242	chr2:212999690-213008084
chr2	213007257	213008100	chr2:212999690-213008084

chr2	213013797	213013970	chr2:213013790-213013934
chr2	213016922	213017058	chr2:213016922-213017044
chr2	213021657	213021756	chr2:213021643-213021720
chr2	213021992	213022149	chr2:213021992-213022130
chr2	213049712	213049890	chr2:213049712-213049880
chr2	213056572	213056778	chr2:213056565-213056744
chr2	213056842	213057123	chr2:213056832-213057099
chr2	213061747	213061949	chr2:213061735-213061935
chr2	213081172	213081250	chr2:213081169-213081238
chr2	213097877	213097980	chr2:213097876-213097960
chr2	213147412	213147662	chr2:213147371-213147812
chr2	213147682	213147850	chr2:213147371-213147812
chr2	213148602	213148680	chr2:213148595-213148644
chr2	213150147	213150448	chr2:213150143-213150408
chr2	213151412	213151620	chr2:213151412-213151609
chr2	213152137	213152453	chr2:213152125-213152427
chr2	214414490	214414596	chr2:214414483-214414583
chr2	214436665	214436782	chr2:214436658-214436758
chr2	214546810	214547028	chr2:214546809-214547009
chr2	214575675	214575855	chr2:214575671-214575821
chr2	218981090	218982294	chr2:218981086-218982256
chr2	218983165	218983382	chr2:218983162-218983362
chr2	218984230	218984338	chr2:218984230-218984305
chr2	218985025	218985691	chr2:218985023-218985657
chr2	219245630	219245818	chr2:219245628-219245796
chr2	219245905	219246112	chr2:219245891-219246085
chr2	219246530	219246920	chr2:219246521-219246876
chr2	219247115	219247275	chr2:219247112-219247246
chr2	219247415	219247564	chr2:219247414-219247535
chr2	219247675	219247791	chr2:219247661-219247757
chr2	219248195	219248356	chr2:219248192-219248314
chr2	219248420	219249582	chr2:219248420-219249560
chr2	222199900	222201021	chr2:222199887-222202190
chr2	222201045	222201818	chr2:222199887-222202190
chr2	222201835	222202218	chr2:222199887-222202190
chr2	222220149	222220392	chr2:222220139-222220354
chr2	222221224	222221404	chr2:222221221-222221387
chr2	222221484	222221728	chr2:222221483-222221699
chr2	222232089	222232299	chr2:222232077-222232283
chr2	222239834	222239999	chr2:222239779-222239979
chr2	222293639	222293712	chr2:222293629-222294301
chr2	222293719	222293855	chr2:222293629-222294301
chr2	222293869	222294321	chr2:222293629-222294301
chr2	222295539	222295670	chr2:222295527-222295657
chr2	222296984	222297230	chr2:222296977-222297213
chr2	222298544	222299032	chr2:222298530-222298996
chr2	223599156	223599323	chr2:223599146-223599296
chr2	223600476	223600688	chr2:223600468-223600668
chr2	223602291	223602409	chr2:223602284-223602384
chr2	230168927	230169242	chr2:230168917-230169237
chr2	230170627	230170763	chr2:230170620-230170761
chr2	230171482	230171639	chr2:230171478-230171616
chr2	230171697	230171810	chr2:230171695-230171767
chr2	230172077	230172218	chr2:230172065-230172174
chr2	230172852	230173001	chr2:230172843-230172959
chr2	230176497	230176744	chr2:230176472-230176724
chr2	230177537	230177717	chr2:230177537-230177680
chr2	230178167	230178261	chr2:230178156-230178255
chr2	230183572	230183643	chr2:230183571-230183640
chr2	230186007	230186181	chr2:230185993-230186143
chr2	230200897	230201003	chr2:230200884-230200965
chr2	230202592	230202760	chr2:230202578-230202728
chr2	230209942	230210052	chr2:230209930-230210008

chr2	230211482	230211591	chr2:230211469-230211553
chr2	230212357	230212467	chr2:230212346-230212430
chr2	230212772	230213058	chr2:230212760-230213027
chr2	230214957	230215138	chr2:230214949-230215118
chr2	230216782	230216971	chr2:230216780-230216928
chr2	230219557	230219701	chr2:230219555-230219673
chr2	230219877	230220142	chr2:230219873-230220112
chr2	230221717	230221822	chr2:230221704-230221784
chr2	230223907	230224121	chr2:230223895-230224095
chr2	230225542	230225614	chr2:230225534-230225579
chr2	233837514	233837651	chr2:233837502-233837652
chr2	233838669	233838880	chr2:233838662-233838862
chr2	233840609	233840716	chr2:233840608-233840708
chr2	236165236	236166470	chr2:236165235-236166437
chr2	236167146	236167357	chr2:236167137-236167329
chr2	236167451	236167780	chr2:236167448-236168369
chr2	236167801	236168401	chr2:236167448-236168369
chr2	237627587	237627762	chr2:237627575-237627740
chr2	237683412	237683524	chr2:237683403-237683495
chr2	237692157	237692545	chr2:237692144-237692521
chr2	237700767	237700984	chr2:237700761-237700961
chr2	237708547	237708669	chr2:237708543-237708630
chr2	237717762	237717835	chr2:237717761-237717809
chr2	237719527	237719604	chr2:237719522-237719567
chr2	237720772	237720855	chr2:237720771-237720822
chr2	237723542	237723619	chr2:237723547-237723586
chr2	237727882	237727963	chr2:237727875-237727935
chr2	237733772	237733846	chr2:237733773-237733818
chr2	237735277	237735351	chr2:237735267-237735333
chr2	237735602	237735703	chr2:237735588-237735676
chr2	237739237	237739347	chr2:237739231-237739309
chr2	237748362	237748430	chr2:237748363-237748399
chr2	237749202	237749363	chr2:237749198-237749324
chr2	237751212	237751290	chr2:237751199-237751271
chr2	237753312	237753501	chr2:237753308-237753479
chr2	237756102	237756208	chr2:237756094-237756187
chr2	237757457	237757564	chr2:237757455-237757548
chr2	237758737	237758841	chr2:237758728-237758821
chr2	237760077	237760217	chr2:237760063-237760205
chr2	237762602	237763415	chr2:237762602-237765915
chr2	237763442	237764977	chr2:237762602-237765915
chr2	237765457	237765696	chr2:237762602-237765915
chr2	237765712	237765952	chr2:237762602-237765915
chr2	237769627	237769848	chr2:237769625-237769818
chr2	237769952	237770019	chr2:237769942-237769992
chr2	237772087	237772227	chr2:237772080-237772198
chr2	237772867	237772987	chr2:237772865-237772945
chr2	237773862	237774250	chr2:237773853-237774227
chr2	237774367	237774463	chr2:237774357-237774462
chr2	237779422	237779886	chr2:237779421-237780315
chr2	237779897	237780302	chr2:237779421-237780315
chr2	237813592	237813705	chr2:237813591-237813682
chr2	238238277	238239607	chr2:238238266-238239568
chr2	238239672	238240160	chr2:238239660-238240124
chr2	238240397	238240670	chr2:238240391-238240662
chr2	240676606	240676778	chr2:240676594-240676744
chr2	240680356	240680461	chr2:240680351-240680451
chr2	240681291	240681498	chr2:240681283-240681483
chr3	4979126	4979827	chr3:4979115-4979798
chr3	4979961	4980035	chr3:4979961-4980031
chr3	4980301	4980443	chr3:4980300-4980408
chr3	4981401	4981556	chr3:4981391-4981515
chr3	4982846	4983834	chr3:4982835-4985323

chr3	4983851	4984923	chr3:4982835-4985323
chr3	4984926	4985030	chr3:4982835-4985323
chr3	4985031	4985345	chr3:4982835-4985323
chr3	12287377	12287685	chr3:12287367-12287675
chr3	12287872	12288038	chr3:12287859-12288020
chr3	12288847	12289171	chr3:12288837-12289134
chr3	12305782	12306002	chr3:12305772-12305959
chr3	12312387	12312487	chr3:12312379-12312453
chr3	12351472	12351699	chr3:12351471-12351674
chr3	12371862	12372145	chr3:12371850-12372111
chr3	12379712	12379956	chr3:12379703-12379931
chr3	12381322	12381504	chr3:12381321-12381491
chr3	12392617	12392775	chr3:12392613-12392752
chr3	12394727	12394944	chr3:12394720-12394920
chr3	12399352	12399426	chr3:12399347-12399490
chr3	12405882	12406109	chr3:12405881-12406081
chr3	12416717	12417174	chr3:12416703-12417154
chr3	12433907	12434379	chr3:12433897-12434356
chr3	14990816	14991030	chr3:14990812-14991012
chr3	15039136	15039257	chr3:15039127-15039227
chr3	15042846	15043029	chr3:15042833-15042983
chr3	18345398	18345608	chr3:18345386-18349682
chr3	18345618	18346561	chr3:18345386-18349682
chr3	18346603	18348521	chr3:18345386-18349682
chr3	18348548	18348724	chr3:18345386-18349682
chr3	18348738	18348869	chr3:18345386-18349682
chr3	18348873	18349647	chr3:18345386-18349682
chr3	18351313	18351424	chr3:18351312-18351408
chr3	18352003	18352218	chr3:18351991-18352195
chr3	18372158	18372378	chr3:18372150-18372350
chr3	18378173	18378353	chr3:18378169-18378325
chr3	18386403	18386648	chr3:18386398-18386611
chr3	18394473	18394932	chr3:18394461-18394916
chr3	18397178	18397333	chr3:18397178-18397290
chr3	18410798	18410989	chr3:18410797-18411054
chr3	18411008	18411083	chr3:18410797-18411054
chr3	18415123	18415257	chr3:18415110-18415234
chr3	18416013	18416154	chr3:18416003-18416133
chr3	18416913	18417432	chr3:18416900-18417393
chr3	18417463	18417532	chr3:18417457-18417510
chr3	18417578	18417722	chr3:18417575-18417699
chr3	18420758	18421427	chr3:18420755-18421388
chr3	18423638	18423732	chr3:18423626-18425369
chr3	18423753	18424126	chr3:18423626-18425369
chr3	18424128	18424577	chr3:18423626-18425369
chr3	18424588	18425151	chr3:18423626-18425369
chr3	18425188	18425352	chr3:18423626-18425369
chr3	18434913	18435270	chr3:18434910-18435253
chr3	18436798	18436896	chr3:18436788-18436871
chr3	18438573	18438795	chr3:18438564-18438773
chr3	18444608	18444721	chr3:18444606-18444883
chr3	18444733	18444904	chr3:18444606-18444883
chr3	18445528	18445622	chr3:18445517-18445588
chr3	19148453	19148813	chr3:19148453-19148795
chr3	19253653	19253902	chr3:19253653-19253887
chr3	19281208	19281338	chr3:19281197-19281329
chr3	19329533	19329748	chr3:19329524-19329724
chr3	19342588	19342735	chr3:19342586-19342714
chr3	19346658	19346763	chr3:19346651-19346734
chr3	19347728	19347974	chr3:19347720-19347965
chr3	19390493	19390673	chr3:19390480-19390638
chr3	19391693	19391806	chr3:19391690-19391765
chr3	19395113	19395324	chr3:19395103-19395311

chr3	19438168	19438388	chr3:19438163-19438361
chr3	19450118	19451442	chr3:19450105-19451404
chr3	19456773	19457019	chr3:19456767-19456982
chr3	19510358	19510438	chr3:19510362-19510401
chr3	19513043	19513362	chr3:19512969-19513325
chr3	19515328	19515466	chr3:19515321-19515428
chr3	19518003	19518116	chr3:19517997-19518074
chr3	19533403	19535684	chr3:19533394-19535646
chr3	23945259	23945518	chr3:23945259-23945594
chr3	23945524	23945620	chr3:23945259-23945594
chr3	23946134	23946206	chr3:23946120-23946172
chr3	23954544	23954820	chr3:23954536-23954803
chr3	23956049	23956155	chr3:23956036-23956125
chr3	23959674	23959853	chr3:23959670-23959815
chr3	23961989	23962620	chr3:23961976-23962605
chr3	23963234	23963363	chr3:23963223-23963330
chr3	23964989	23965193	chr3:23964976-23965162
chr3	23967824	23968042	chr3:23967812-23968023
chr3	23972684	23972889	chr3:23972681-23972881
chr3	23977224	23978700	chr3:23977222-23980618
chr3	23978709	23979333	chr3:23977222-23980618
chr3	23979334	23979541	chr3:23977222-23980618
chr3	23979554	23980636	chr3:23977222-23980618
chr3	24117171	24118977	chr3:24117159-24123125
chr3	24119001	24121115	chr3:24117159-24123125
chr3	24121141	24122722	chr3:24117159-24123125
chr3	24122736	24123158	chr3:24117159-24123125
chr3	24127506	24127799	chr3:24127498-24127757
chr3	24133326	24133499	chr3:24133315-24133462
chr3	24143501	24143718	chr3:24143500-24143706
chr3	24146676	24146852	chr3:24146674-24146822
chr3	24152401	24152505	chr3:24152389-24152490
chr3	24165036	24165628	chr3:24165026-24165595
chr3	24190081	24190363	chr3:24190073-24190334
chr3	24228941	24229014	chr3:24228937-24229001
chr3	24297227	24297416	chr3:24297225-24297371
chr3	24318362	24318503	chr3:24318357-24318494
chr3	24337302	24337379	chr3:24337299-24337371
chr3	24348562	24348643	chr3:24348557-24348614
chr3	24430107	24430216	chr3:24430100-24430172
chr3	24448367	24448578	chr3:24448365-24448565
chr3	24494652	24495039	chr3:24494651-24495005
chr3	24495142	24495311	chr3:24495132-24495282
chr3	25174332	25174596	chr3:25174331-25174575
chr3	25428267	25428901	chr3:25428262-25428888
chr3	25432682	25432899	chr3:25432669-25432869
chr3	25458322	25458422	chr3:25458309-25458393
chr3	25461197	25461378	chr3:25461192-25461341
chr3	25462322	25462569	chr3:25462308-25462545
chr3	25501187	25501356	chr3:25501181-25501323
chr3	25569762	25569935	chr3:25569757-25569918
chr3	25580557	25580764	chr3:25580545-25580722
chr3	25593507	25593725	chr3:25593502-25593707
chr3	25594522	25594719	chr3:25594519-25594678
chr3	25596422	25597410	chr3:25596419-25597754
chr3	25597422	25597774	chr3:25596419-25597754
chr3	27715961	27716375	chr3:27715948-27717808
chr3	27716391	27716870	chr3:27715948-27717808
chr3	27716876	27717819	chr3:27715948-27717808
chr3	27718586	27718662	chr3:27718586-27718648
chr3	27718741	27718914	chr3:27718734-27718893
chr3	27719371	27719502	chr3:27719359-27719481
chr3	27720171	27720351	chr3:27720170-27720325

chr3	27721416	27721913	chr3:27721413-27722711
chr3	27721926	27722736	chr3:27721413-27722711
chr3	33388345	33389004	chr3:33388335-33390368
chr3	33389035	33390382	chr3:33388335-33390368
chr3	33392570	33392642	chr3:33392562-33392614
chr3	33393325	33393463	chr3:33393311-33393454
chr3	33396175	33396318	chr3:33396161-33396280
chr3	33396755	33396896	chr3:33396744-33396878
chr3	33397045	33397152	chr3:33397044-33397135
chr3	33400200	33400306	chr3:33400188-33400282
chr3	33400975	33401050	chr3:33400961-33401016
chr3	33402800	33402908	chr3:33402800-33402904
chr3	33406735	33406952	chr3:33406722-33406922
chr3	33408700	33408840	chr3:33408689-33408797
chr3	33409240	33409381	chr3:33409235-33409346
chr3	33409460	33409633	chr3:33409448-33409601
chr3	33411590	33411722	chr3:33411580-33411687
chr3	33412735	33412854	chr3:33412721-33412827
chr3	33416005	33416122	chr3:33416005-33416100
chr3	33416770	33416867	chr3:33416757-33416834
chr3	33425590	33425771	chr3:33425589-33425741
chr3	33439735	33440625	chr3:33439735-33440587
chr3	33440680	33440935	chr3:33440676-33440923
chr3	33441270	33441389	chr3:33441268-33441371
chr3	39141857	39143211	chr3:39141854-39144044
chr3	39143232	39143592	chr3:39141854-39144044
chr3	39143612	39144078	chr3:39141854-39144044
chr3	39144137	39144469	chr3:39144136-39144451
chr3	39145007	39145283	chr3:39144996-39145256
chr3	39146477	39146601	chr3:39146477-39146722
chr3	39146617	39146755	chr3:39146477-39146722
chr3	39148057	39148264	chr3:39148054-39148254
chr3	39153437	39153584	chr3:39153437-39153575
chr3	39154502	39154579	chr3:39154496-39154562
chr3	40412852	40413076	chr3:40412848-40413048
chr3	40423867	40423969	chr3:40423863-40423963
chr3	40424777	40424948	chr3:40424766-40424916
chr3	40427282	40427460	chr3:40427271-40427421
chr3	44648730	44648847	chr3:44648726-44648834
chr3	44650950	44651267	chr3:44650940-44651259
chr3	44652565	44652741	chr3:44652556-44652701
chr3	44653790	44654008	chr3:44653782-44653982
chr3	44658700	44659090	chr3:44658700-44660791
chr3	44659175	44659761	chr3:44658700-44660791
chr3	44659775	44660828	chr3:44658700-44660791
chr3	44852250	44852349	chr3:44852239-44852339
chr3	44852715	44852850	chr3:44852672-44852822
chr3	44871080	44871310	chr3:44871078-44871278
chr3	44894460	44894671	chr3:44894454-44894654
chr3	44911310	44911433	chr3:44911306-44911406
chr3	44913585	44913773	chr3:44913583-44913733
chr3	45823320	45824240	chr3:45823315-45826332
chr3	45824255	45824321	chr3:45823315-45826332
chr3	45824385	45824546	chr3:45823315-45826332
chr3	45824560	45826352	chr3:45823315-45826332
chr3	45827355	45827470	chr3:45827355-45827459
chr3	45828440	45828655	chr3:45828438-45828615
chr3	45830920	45831027	chr3:45830912-45830990
chr3	45831075	45831147	chr3:45831072-45831138
chr3	45833050	45833159	chr3:45833049-45833121
chr3	45834240	45834315	chr3:45834237-45834298
chr3	45835595	45835816	chr3:45835589-45835784
chr3	45837930	45838081	chr3:45837926-45838051

chr3	45841695	45841873	chr3:45841694-45841860
chr3	45842000	45842207	chr3:45841988-45842166
chr3	45854995	45855095	chr3:45854985-45855074
chr3	45858950	45859044	chr3:45858939-45859016
chr3	45904470	45904677	chr3:45904457-45904657
chr3	45913140	45913215	chr3:45913119-45913177
chr3	45915430	45916080	chr3:45915420-45916042
chr3	48241108	48241250	chr3:48241099-48241214
chr3	48247633	48247707	chr3:48247624-48247677
chr3	48257928	48258003	chr3:48257928-48257961
chr3	48260823	48260969	chr3:48260812-48260939
chr3	48267928	48270924	chr3:48267914-48270990
chr3	48279503	48279713	chr3:48279495-48279695
chr3	48286258	48287301	chr3:48286245-48287627
chr3	48298968	48299286	chr3:48298954-48299253
chr3	49859530	49859723	chr3:49859526-49859676
chr3	49859735	49859920	chr3:49859731-49859881
chr3	49860175	49860294	chr3:49860170-49860258
chr3	49867420	49867624	chr3:49867415-49867615
chr3	52410661	52411006	chr3:52410656-52411247
chr3	52411011	52411285	chr3:52410656-52411247
chr3	52412811	52412953	chr3:52412810-52412920
chr3	52413996	52414065	chr3:52413995-52414048
chr3	52414496	52414620	chr3:52414495-52414587
chr3	52419836	52419955	chr3:52419832-52419934
chr3	52420316	52420454	chr3:52420310-52420435
chr3	52420906	52421084	chr3:52420902-52421062
chr3	52421651	52421761	chr3:52421647-52421754
chr3	52422231	52422383	chr3:52422221-52422338
chr3	52422771	52422911	chr3:52422759-52422881
chr3	52423091	52423317	chr3:52423090-52423641
chr3	52423321	52423663	chr3:52423090-52423641
chr3	57197856	57197983	chr3:57197842-57198295
chr3	57197991	57198298	chr3:57197842-57198295
chr3	57198396	57198500	chr3:57198390-57198492
chr3	57198761	57198965	chr3:57198752-57198952
chr3	57199771	57200055	chr3:57199761-57200028
chr3	57200881	57201088	chr3:57200868-57201068
chr3	57226296	57226535	chr3:57226295-57226521
chr3	62369701	62369988	chr3:62369680-62370342
chr3	62369996	62370370	chr3:62369680-62370342
chr3	62371226	62371361	chr3:62371216-62371349
chr3	62371546	62371680	chr3:62371532-62371667
chr3	62372016	62372515	chr3:62372016-62373015
chr3	62372556	62373037	chr3:62372016-62373015
chr3	62373291	62373529	chr3:62373278-62373515
chr3	63239791	63240017	chr3:63239787-63239987
chr3	63480866	63480978	chr3:63480856-63480956
chr3	63556551	63556755	chr3:63556542-63556741
chr3	63609226	63609327	chr3:63609216-63609316
chr3	63610476	63610596	chr3:63610473-63610553
chr3	63615236	63615412	chr3:63615223-63615373
chr3	69739436	69739724	chr3:69739434-69739701
chr3	69752066	69752148	chr3:69752039-69752385
chr3	69758716	69758787	chr3:69758703-69758756
chr3	69762731	69762814	chr3:69762723-69762789
chr3	69763556	69763700	chr3:69763555-69763692
chr3	69763811	69763961	chr3:69763810-69763942
chr3	69778831	69779077	chr3:69778817-69779042
chr3	69838591	69838662	chr3:69838593-69838632
chr3	69866216	69866364	chr3:69866211-69866346
chr3	69879146	69879420	chr3:69879133-69879383
chr3	69882126	69882337	chr3:69882121-69882321

chr3	69936586	69936772	chr3:69936586-69936755
chr3	69936836	69936948	chr3:69936828-69937172
chr3	69936956	69937200	chr3:69936828-69937172
chr3	69937831	69938071	chr3:69937821-69938049
chr3	69938391	69939088	chr3:69938351-69939065
chr3	69939101	69939232	chr3:69939097-69939181
chr3	69941236	69941346	chr3:69941235-69941331
chr3	69949051	69949203	chr3:69949050-69949168
chr3	69951816	69951920	chr3:69951811-69951886
chr3	69956466	69956567	chr3:69956454-69956530
chr3	69959286	69959456	chr3:69959272-69959420
chr3	69964851	69965660	chr3:69964846-69968337
chr3	69965671	69965801	chr3:69964846-69968337
chr3	69965821	69966066	chr3:69964846-69968337
chr3	69966076	69967716	chr3:69964846-69968337
chr3	69967721	69968352	chr3:69964846-69968337
chr3	70954696	70954881	chr3:70954692-70959391
chr3	70954891	70955706	chr3:70954692-70959391
chr3	70955721	70955826	chr3:70954692-70959391
chr3	70955846	70956416	chr3:70954692-70959391
chr3	70956421	70956494	chr3:70954692-70959391
chr3	70956501	70956740	chr3:70954692-70959391
chr3	70956767	70956934	chr3:70954692-70959391
chr3	70956952	70957158	chr3:70954692-70959391
chr3	70957162	70957277	chr3:70954692-70959391
chr3	70957287	70958126	chr3:70954692-70959391
chr3	70958132	70958207	chr3:70954692-70959391
chr3	70958222	70958289	chr3:70954692-70959391
chr3	70958312	70958623	chr3:70954692-70959391
chr3	70958652	70958742	chr3:70954692-70959391
chr3	70958767	70959190	chr3:70954692-70959391
chr3	70959197	70959412	chr3:70954692-70959391
chr3	70965892	70966075	chr3:70965889-70966056
chr3	70968847	70969064	chr3:70968847-70969051
chr3	70970742	70970819	chr3:70970735-70970805
chr3	70972022	70972205	chr3:70972010-70972180
chr3	70972567	70972708	chr3:70972554-70972676
chr3	70976947	70977057	chr3:70976940-70977042
chr3	70977652	70977755	chr3:70977642-70977722
chr3	70977827	70978044	chr3:70977827-70978029
chr3	70988005	70988111	chr3:70987993-70988077
chr3	71000975	71001099	chr3:71000971-71001059
chr3	71015550	71015664	chr3:71015548-71015653
chr3	71040285	71040424	chr3:71040277-71040403
chr3	71041330	71041554	chr3:71041327-71041532
chr3	71046940	71047124	chr3:71046936-71047095
chr3	71052550	71052652	chr3:71052536-71052626
chr3	71053635	71053795	chr3:71053635-71053773
chr3	71064825	71064943	chr3:71064823-71064926
chr3	71065635	71065807	chr3:71065624-71065767
chr3	71112535	71112649	chr3:71112535-71112637
chr3	71130505	71130853	chr3:71130497-71130837
chr3	71197890	71198111	chr3:71197881-71198068
chr3	71198215	71198419	chr3:71198201-71198392
chr3	71210820	71210902	chr3:71210818-71210869
chr3	71244865	71244968	chr3:71244853-71245165
chr3	71244980	71245189	chr3:71244853-71245165
chr3	71296085	71296434	chr3:71296084-71296445
chr3	71299825	71299897	chr3:71299819-71299880
chr3	71304690	71304792	chr3:71304678-71304760
chr3	71305890	71306062	chr3:71305877-71306029
chr3	71329775	71329854	chr3:71329780-71329820
chr3	71332745	71333554	chr3:71332193-71335281

chr3	71333845	71333912	chr3:71332193-71335281
chr3	71333920	71334018	chr3:71332193-71335281
chr3	71334025	71334131	chr3:71332193-71335281
chr3	71334410	71335002	chr3:71332193-71335281
chr3	71359150	71359272	chr3:71359149-71359244
chr3	71360490	71360663	chr3:71360479-71360760
chr3	71360675	71360785	chr3:71360479-71360760
chr3	71391685	71391911	chr3:71391682-71391882
chr3	71493435	71493568	chr3:71493425-71493555
chr3	71543430	71543575	chr3:71543423-71543557
chr3	71581550	71581975	chr3:71581548-71581953
chr3	71583580	71583788	chr3:71583570-71583753
chr3	71583915	71583985	chr3:71583909-71583989
chr3	87259408	87259751	chr3:87259403-87260104
chr3	87259758	87260142	chr3:87259403-87260104
chr3	87261283	87261354	chr3:87261272-87261333
chr3	87262073	87262258	chr3:87262070-87262235
chr3	87264298	87264531	chr3:87264287-87264512
chr3	87271398	87271625	chr3:87271397-87271597
chr3	87273348	87273530	chr3:87273346-87273496
chr3	87276333	87276626	chr3:87276320-87276587
chr3	88051947	88056836	chr3:88051943-88057309
chr3	88056847	88057047	chr3:88051943-88057309
chr3	88057067	88057333	chr3:88051943-88057309
chr3	88057642	88058238	chr3:88057637-88058223
chr3	88058872	88059078	chr3:88058814-88059068
chr3	88061607	88061745	chr3:88061598-88061798
chr3	88061747	88061813	chr3:88061598-88061798
chr3	101827996	101828205	chr3:101827990-101828184
chr3	101829621	101829727	chr3:101829612-101829688
chr3	101833336	101833549	chr3:101833329-101833529
chr3	101848891	101849923	chr3:101848891-101849917
chr3	101850276	101850449	chr3:101850262-101850426
chr3	101852091	101852238	chr3:101852084-101852224
chr3	101852736	101852807	chr3:101852737-101852768
chr3	101852886	101853007	chr3:101852885-101852989
chr3	101853101	101853900	chr3:101853090-101853863
chr3	101854591	101854706	chr3:101854577-101854683
chr3	101855061	101855254	chr3:101855061-101855208
chr3	101855396	101855473	chr3:101855394-101855458
chr3	101855746	101855916	chr3:101855732-101855902
chr3	101857076	101857223	chr3:101857072-101857183
chr3	101857291	101857471	chr3:101857291-101857459
chr3	101859331	101859530	chr3:101859317-101861022
chr3	101859536	101859982	chr3:101859317-101861022
chr3	101860001	101860181	chr3:101859317-101861022
chr3	101860466	101860663	chr3:101859317-101861022
chr3	101860671	101860796	chr3:101859317-101861022
chr3	101860821	101861054	chr3:101859317-101861022
chr3	107522936	107523116	chr3:107522935-107523107
chr3	107523456	107523671	chr3:107523453-107523651
chr3	107524111	107524217	chr3:107524106-107524194
chr3	107524356	107524428	chr3:107524356-107524567
chr3	107524431	107524601	chr3:107524356-107524567
chr3	107525396	107525506	chr3:107525390-107525490
chr3	107526336	107526433	chr3:107526326-107526398
chr3	107599326	107599459	chr3:107599312-107599588
chr3	107599476	107599612	chr3:107599312-107599588
chr3	107638506	107638582	chr3:107638501-107638545
chr3	107641801	107641892	chr3:107641790-107641864
chr3	107645846	107646028	chr3:107645835-107646087
chr3	107646046	107646113	chr3:107645835-107646087
chr3	107657061	107657206	chr3:107657061-107657172

chr3	107659576	107659797	chr3:107659567-107659770
chr3	107661841	107661985	chr3:107661827-107661944
chr3	107662661	107662898	chr3:107662588-107662880
chr3	107674801	107674873	chr3:107674804-107674836
chr3	107677426	107677501	chr3:107677426-107677470
chr3	107681896	107682101	chr3:107681890-107682090
chr3	107684566	107684739	chr3:107684555-107684709
chr3	107710451	107710652	chr3:107710451-107710622
chr3	107711276	107711354	chr3:107711274-107711364
chr3	107716616	107716866	chr3:107716606-107716849
chr3	107728776	107728991	chr3:107728764-107728960
chr3	107732956	107733037	chr3:107732955-107733023
chr3	107744641	107744739	chr3:107744629-107744710
chr3	107747976	107748074	chr3:107747964-107748039
chr3	107755601	107755716	chr3:107755597-107755678
chr3	107772631	107772733	chr3:107772627-107773636
chr3	107772741	107773060	chr3:107772627-107773636
chr3	107773081	107773672	chr3:107772627-107773636
chr3	107774731	107774865	chr3:107774718-107774857
chr3	107776206	107776384	chr3:107776204-107776349
chr3	107778371	107778547	chr3:107778370-107778519
chr3	107789796	107789899	chr3:107789786-107789876
chr3	107791246	107791321	chr3:107791239-107791299
chr3	107798536	107798742	chr3:107798522-107798720
chr3	107801096	107801323	chr3:107801094-107801281
chr3	107805381	107805831	chr3:107805369-107811324
chr3	107805861	107806105	chr3:107805369-107811324
chr3	107806131	107806339	chr3:107805369-107811324
chr3	107806351	107807530	chr3:107805369-107811324
chr3	107807531	107807707	chr3:107805369-107811324
chr3	107807731	107808115	chr3:107805369-107811324
chr3	107808141	107810203	chr3:107805369-107811324
chr3	107810226	107810752	chr3:107805369-107811324
chr3	107810761	107810912	chr3:107805369-107811324
chr3	107810926	107811350	chr3:107805369-107811324
chr3	114234672	114235156	chr3:114234630-114237578
chr3	114235172	114235811	chr3:114234630-114237578
chr3	114235857	114236212	chr3:114234630-114237578
chr3	114236217	114237134	chr3:114234630-114237578
chr3	114338102	114338655	chr3:114338095-114339426
chr3	114338677	114338879	chr3:114338095-114339426
chr3	114338882	114338947	chr3:114338095-114339426
chr3	114338987	114339443	chr3:114338095-114339426
chr3	114350277	114351904	chr3:114350273-114351878
chr3	114380222	114380432	chr3:114380216-114380404
chr3	114380777	114380891	chr3:114380776-114380940
chr3	114387332	114387589	chr3:114387274-114387564
chr3	114388077	114388175	chr3:114388064-114388145
chr3	114389007	114389124	chr3:114389004-114389105
chr3	114419067	114419203	chr3:114419053-114419167
chr3	114454592	114454719	chr3:114454578-114454817
chr3	114454772	114454847	chr3:114454578-114454817
chr3	114500347	114500418	chr3:114500351-114500391
chr3	114502822	114502889	chr3:114502797-114502864
chr3	114556817	114557028	chr3:114556806-114557006
chr3	114582124	114582262	chr3:114582113-114582266
chr3	114599254	114599365	chr3:114599250-114599346
chr3	114624119	114624241	chr3:114624115-114624206
chr3	114624889	114624971	chr3:114624876-114624945
chr3	114658399	114658881	chr3:114658340-114658859
chr3	114687229	114687583	chr3:114687225-114688375
chr3	114687629	114687948	chr3:114687225-114688375
chr3	114693529	114693607	chr3:114693527-114693575

chr3	114710269	114710344	chr3:114710267-114710313
chr3	114713984	114714055	chr3:114713975-114714015
chr3	114743719	114743796	chr3:114743711-114743779
chr3	114758899	114759305	chr3:114758895-114759271
chr3	114791684	114792207	chr3:114791684-114792177
chr3	114801104	114801211	chr3:114801100-114801177
chr3	114861754	114861831	chr3:114861761-114861793
chr3	114874044	114874121	chr3:114874038-114874108
chr3	114900299	114900374	chr3:114900303-114900342
chr3	114904329	114904397	chr3:114904326-114904368
chr3	114974369	114974435	chr3:114974365-114974416
chr3	115027464	115027607	chr3:115027455-115027574
chr3	115071219	115071410	chr3:115071218-115071375
chr3	115100184	115100326	chr3:115100174-115100295
chr3	115144814	115144926	chr3:115144812-115144909
chr3	115147229	115147295	chr3:115147218-115147271
chr3	119780483	119781011	chr3:119780483-119782300
chr3	119781028	119782295	chr3:119780483-119782300
chr3	119782718	119782873	chr3:119782709-119782852
chr3	119795993	119796213	chr3:119795985-119796185
chr3	119807228	119807482	chr3:119807228-119807447
chr3	119810063	119810216	chr3:119810060-119810194
chr3	119811538	119811752	chr3:119811538-119811726
chr3	119812698	119812979	chr3:119812685-119812960
chr3	119814978	119815162	chr3:119814978-119815121
chr3	119815323	119815473	chr3:119815322-119815439
chr3	119815728	119815847	chr3:119815725-119815831
chr3	119817068	119818495	chr3:119817067-119818485
chr3	121835238	121835417	chr3:121835229-121835391
chr3	121836188	121836259	chr3:121836188-121836256
chr3	121836658	121836826	chr3:121836644-121836794
chr3	121837423	121837814	chr3:121837423-121837776
chr3	121844458	121844562	chr3:121844452-121844547
chr3	121852063	121852162	chr3:121852050-121852250
chr3	121852163	121852265	chr3:121852050-121852250
chr3	121854688	121854832	chr3:121854686-121854823
chr3	121857048	121857196	chr3:121857010-121857156
chr3	121872543	121872821	chr3:121872536-121872788
chr3	121872993	121873067	chr3:121872988-121873163
chr3	121873078	121873189	chr3:121872988-121873163
chr3	121886343	121886560	chr3:121886341-121886526
chr3	125225573	125225687	chr3:125225560-125233939
chr3	125225703	125226018	chr3:125225560-125233939
chr3	125226043	125226767	chr3:125225560-125233939
chr3	125226783	125227883	chr3:125225560-125233939
chr3	125227903	125229195	chr3:125225560-125233939
chr3	125229223	125230921	chr3:125225560-125233939
chr3	125230943	125231359	chr3:125225560-125233939
chr3	125231373	125231895	chr3:125225560-125233939
chr3	125231913	125232056	chr3:125225560-125233939
chr3	125232073	125232315	chr3:125225560-125233939
chr3	125232338	125233967	chr3:125225560-125233939
chr3	125234223	125234360	chr3:125234210-125234329
chr3	125277728	125277838	chr3:125277725-125277809
chr3	125279123	125279262	chr3:125279123-125279247
chr3	125288103	125288246	chr3:125288102-125288228
chr3	125292473	125292828	chr3:125292464-125292793
chr3	125313318	125313663	chr3:125313307-125313656
chr3	125323308	125323452	chr3:125323308-125323444
chr3	125331158	125331267	chr3:125331157-125331238
chr3	125338828	125338947	chr3:125338821-125338911
chr3	125356988	125357260	chr3:125356976-125357280
chr3	125357748	125357851	chr3:125357735-125357818

chr3	125358798	125359019	chr3:125358791-125358991
chr3	125375108	125375181	chr3:125375101-125375354
chr3	125375188	125375260	chr3:125375101-125375354
chr3	125375288	125375393	chr3:125375101-125375354
chr3	126103708	126103881	chr3:126103696-126103846
chr3	126105738	126105851	chr3:126105725-126105825
chr3	126147898	126148106	chr3:126147886-126148086
chr3	126342648	126343147	chr3:126342634-126343895
chr3	126343178	126343916	chr3:126342634-126343895
chr3	126350923	126351125	chr3:126350912-126351112
chr3	126351853	126352969	chr3:126351840-126352947
chr3	126354013	126354306	chr3:126354011-126354285
chr3	126357238	126357468	chr3:126357236-126357442
chr3	128479428	128480162	chr3:128479426-128481318
chr3	128480168	128481354	chr3:128479426-128481318
chr3	128481818	128482107	chr3:128481818-128482078
chr3	128483863	128484042	chr3:128483859-128484005
chr3	128485728	128486405	chr3:128485726-128486368
chr3	128486813	128487088	chr3:128486802-128487076
chr3	128487723	128487939	chr3:128487710-128487921
chr3	128488363	128488565	chr3:128488351-128488530
chr3	128490308	128490663	chr3:128490299-128490648
chr3	128491233	128491436	chr3:128491226-128491426
chr3	128492013	128492247	chr3:128492003-128492222
chr3	128492898	128493217	chr3:128492898-128493185
chr3	129170433	129170607	chr3:129170420-129170570
chr3	129171078	129171206	chr3:129171078-129171178
chr3	129182623	129182842	chr3:129182622-129182822
chr3	136338052	136338296	chr3:136338046-136338277
chr3	136338372	136338480	chr3:136338369-136338450
chr3	136340502	136340637	chr3:136340490-136340605
chr3	136341447	136341589	chr3:136341440-136341551
chr3	136343832	136344026	chr3:136343831-136344006
chr3	136349157	136349374	chr3:136349157-136349363
chr3	136357732	136357864	chr3:136357719-136357848
chr3	136359152	136359293	chr3:136359147-136359296
chr3	136363367	136363482	chr3:136363365-136363467
chr3	136366942	136367091	chr3:136366942-136367082
chr3	136369112	136369299	chr3:136369107-136369282
chr3	136377667	136377769	chr3:136377659-136377752
chr3	136398757	136398838	chr3:136398748-136398829
chr3	136417557	136417982	chr3:136417557-136417972
chr3	136421095	136421170	chr3:136421092-136421163
chr3	136422410	136422626	chr3:136422409-136422613
chr3	136422770	136422882	chr3:136422767-136422857
chr3	136422955	136423061	chr3:136422951-136423044
chr3	136433555	136433670	chr3:136433555-136433659
chr3	136443290	136443433	chr3:136443286-136443404
chr3	136452045	136452185	chr3:136452032-136452147
chr3	136464880	136465026	chr3:136464880-136464988
chr3	136472415	136472529	chr3:136472412-136472492
chr3	136473550	136473652	chr3:136473538-136473637
chr3	136477290	136477419	chr3:136477288-136477412
chr3	136500230	136500306	chr3:136500222-136500296
chr3	136502640	136502817	chr3:136502627-136502779
chr3	136518055	136518433	chr3:136518042-136518415
chr3	136521225	136521424	chr3:136521212-136521417
chr3	136542130	136542233	chr3:136542118-136542195
chr3	136568765	136568875	chr3:136568764-136568861
chr3	136586805	136586879	chr3:136586801-136586926
chr3	136586880	136586954	chr3:136586801-136586926
chr3	136591490	136591562	chr3:136591444-136591549
chr3	136604320	136604488	chr3:136604308-136604473

chr3	136623170	136623276	chr3:136623145-136623248
chr3	136630870	136630978	chr3:136630869-136630981
chr3	136658805	136659022	chr3:136658801-136659001
chr3	136752205	136752410	chr3:136752194-136752403
chr3	137764285	137764849	chr3:137764283-137766338
chr3	137764880	137766361	chr3:137764283-137766338
chr3	138944281	138944800	chr3:138944223-138947083
chr3	138944816	138946389	chr3:138944223-138947083
chr3	138946401	138947086	chr3:138944223-138947083
chr3	141944427	141944614	chr3:141944427-141952696
chr3	141944622	141945136	chr3:141944427-141952696
chr3	141945427	141946756	chr3:141944427-141952696
chr3	141947017	141947405	chr3:141944427-141952696
chr3	141947582	141948241	chr3:141944427-141952696
chr3	141948552	141948832	chr3:141944427-141952696
chr3	141949102	141949809	chr3:141944427-141952696
chr3	141950077	141951169	chr3:141944427-141952696
chr3	141951177	141952711	chr3:141944427-141952696
chr3	141952842	141953041	chr3:141952828-141953016
chr3	141959677	141959860	chr3:141959673-141959840
chr3	141963812	141963993	chr3:141963811-141963963
chr3	141970072	141970148	chr3:141970072-141970141
chr3	141974102	141974206	chr3:141974047-141974194
chr3	141978522	141978694	chr3:141978519-141978682
chr3	141989077	141989524	chr3:141989066-141989514
chr3	141993537	141993604	chr3:141993537-141993585
chr3	141995032	141995167	chr3:141995019-141995141
chr3	142000257	142000389	chr3:142000251-142000354
chr3	142005447	142005558	chr3:142005440-142005544
chr3	142028582	142028696	chr3:142028578-142028665
chr3	142029062	142029173	chr3:142029062-142029157
chr3	142093067	142093141	chr3:142093060-142093127
chr3	142101802	142101864	chr3:142101734-142101841
chr3	142126312	142126380	chr3:142126148-142126370
chr3	142147637	142147844	chr3:142147623-142147823
chr3	142149192	142149326	chr3:142149182-142149537
chr3	142149342	142149561	chr3:142149182-142149537
chr3	146515188	146516125	chr3:146515179-146516101
chr3	146516453	146516731	chr3:146516439-146516696
chr3	146516913	146517184	chr3:146516902-146517394
chr3	146517188	146517427	chr3:146516902-146517394
chr3	146521543	146522065	chr3:146521543-146522053
chr3	146525603	146525682	chr3:146525604-146525647
chr3	146528618	146528862	chr3:146528613-146528831
chr3	146532688	146532895	chr3:146532679-146532879
chr3	146533473	146533587	chr3:146533469-146533550
chr3	146537998	146538070	chr3:146537995-146538044
chr3	146540853	146541174	chr3:146540852-146541180
chr3	146544478	146544886	chr3:146544466-146544864
chr3	147386059	147387532	chr3:147386045-147388859
chr3	147387539	147388899	chr3:147386045-147388859
chr3	147389004	147389398	chr3:147388991-147389372
chr3	147390939	147391272	chr3:147390930-147391246
chr3	147391969	147392462	chr3:147391969-147392430
chr3	147393434	147393958	chr3:147393421-147393942
chr3	147395859	147396503	chr3:147395851-147396469
chr3	147398589	147398806	chr3:147398578-147398778
chr3	147402729	147402841	chr3:147402727-147402812
chr3	147403554	147403828	chr3:147403546-147403798
chr3	147403969	147404324	chr3:147403963-147404284
chr3	147405384	147405877	chr3:147405372-147405849
chr3	147405914	147406152	chr3:147405907-147406133
chr3	147406364	147406863	chr3:147406362-147406860

chr3	147409394	147409492	chr3:147409383-147411094
chr3	147409539	147409609	chr3:147409383-147411094
chr3	147409619	147409997	chr3:147409383-147411094
chr3	147410029	147411112	chr3:147409383-147411094
chr3	147412519	147412706	chr3:147412517-147412681
chr3	147413354	147413752	chr3:147413353-147416719
chr3	147413759	147414023	chr3:147413353-147416719
chr3	147414059	147416755	chr3:147413353-147416719
chr3	147421339	147421583	chr3:147421326-147421559
chr3	147489904	147490112	chr3:147489899-147490099
chr3	147507954	147508089	chr3:147507940-147508052
chr3	147509594	147509875	chr3:147509594-147510293
chr3	147509894	147510204	chr3:147509594-147510293
chr3	147510219	147510330	chr3:147509594-147510293
chr3	149030154	149030327	chr3:149030126-149032372
chr3	149030334	149031143	chr3:149030126-149032372
chr3	149031169	149032326	chr3:149030126-149032372
chr3	149034924	149035041	chr3:149034917-149034998
chr3	149039049	149039258	chr3:149039048-149039229
chr3	149039584	149039732	chr3:149039580-149039693
chr3	149040034	149040196	chr3:149040030-149040156
chr3	149041539	149041719	chr3:149041489-149041680
chr3	149042179	149042315	chr3:149042165-149042290
chr3	149046079	149046288	chr3:149046079-149046259
chr3	149048029	149048176	chr3:149048027-149048163
chr3	149048874	149049014	chr3:149048862-149049001
chr3	149050244	149050411	chr3:149050231-149050375
chr3	149055304	149055417	chr3:149055302-149055400
chr3	149059729	149059833	chr3:149059717-149059807
chr3	149060779	149060886	chr3:149060778-149060858
chr3	149063444	149063546	chr3:149063430-149063524
chr3	149064799	149064903	chr3:149064790-149064866
chr3	149068249	149068345	chr3:149068239-149068335
chr3	149071254	149071469	chr3:149071251-149071443
chr3	149071584	149071689	chr3:149071582-149071657
chr3	149073229	149073334	chr3:149073224-149073322
chr3	149074214	149074361	chr3:149074214-149074348
chr3	149075889	149076062	chr3:149075880-149076047
chr3	149081532	149081633	chr3:149081529-149081729
chr3	149081642	149081749	chr3:149081529-149081729
chr3	149084682	149084896	chr3:149084681-149084889
chr3	149086322	149086580	chr3:149086316-149086554
chr3	157261220	157261401	chr3:157261220-157261370
chr3	157265535	157265630	chr3:157265525-157265625
chr3	157330350	157330563	chr3:157330341-157330541
chr3	157459790	157459962	chr3:157459776-157459926
chr3	157460185	157460371	chr3:157460180-157460355
chr3	157470320	157470425	chr3:157470313-157470413
chr3	158096010	158096290	chr3:158096010-158098320
chr3	158096310	158096407	chr3:158096010-158098320
chr3	158096440	158096888	chr3:158096010-158098320
chr3	158096960	158097508	chr3:158096010-158098320
chr3	158097515	158098355	chr3:158096010-158098320
chr3	158099870	158099968	chr3:158099859-158099948
chr3	158100255	158100324	chr3:158100253-158100311
chr3	158101510	158101590	chr3:158101507-158101707
chr3	158101595	158101751	chr3:158101507-158101707
chr3	158102690	158102893	chr3:158102677-158102887
chr3	158105060	158105130	chr3:158105057-158105129
chr3	158106290	158106521	chr3:158106160-158106503
chr3	165773365	165773540	chr3:165773356-165773506
chr3	165786155	165786254	chr3:165786144-165786244
chr3	165813025	165813236	chr3:165813021-165813221

chr3	169083505	169083643	chr3:169083498-169085043
chr3	169083645	169083927	chr3:169083498-169085043
chr3	169083950	169084118	chr3:169083498-169085043
chr3	169084135	169084735	chr3:169083498-169085043
chr3	169084755	169085072	chr3:169083498-169085043
chr3	169086520	169086592	chr3:169086512-169086565
chr3	169089010	169089210	chr3:169088999-169089183
chr3	169090010	169090249	chr3:169089999-169090236
chr3	169092970	169093142	chr3:169092957-169093102
chr3	169095075	169095261	chr3:169095075-169095245
chr3	169100895	169101001	chr3:169100884-169100962
chr3	169102060	169102248	chr3:169102059-169102226
chr3	169112790	169112902	chr3:169112786-169112874
chr3	169115385	169116757	chr3:169115382-169116739
chr3	169121055	169121242	chr3:169121055-169121212
chr3	169122590	169122768	chr3:169122579-169122727
chr3	169127855	169128097	chr3:169127843-169128060
chr3	169131440	169131644	chr3:169131428-169131880
chr3	169131660	169131898	chr3:169131428-169131880
chr3	169133355	169133490	chr3:169133349-169133474
chr3	169133920	169133996	chr3:169133911-169133970
chr3	169143700	169143850	chr3:169143697-169143832
chr3	169145005	169145106	chr3:169144996-169146305
chr3	169145185	169145522	chr3:169144996-169146305
chr3	169145560	169145831	chr3:169144996-169146305
chr3	169145835	169146179	chr3:169144996-169146305
chr3	169146210	169146345	chr3:169144996-169146305
chr3	169146490	169146693	chr3:169146476-169146675
chr3	169146780	169146875	chr3:169146722-169147734
chr3	169146880	169147718	chr3:169146722-169147734
chr3	169149485	169149776	chr3:169149481-169149755
chr3	169269135	169269249	chr3:169269134-169269238
chr3	169354730	169354957	chr3:169354729-169354929
chr3	169381195	169381546	chr3:169381186-169381524
chr3	169663340	169663443	chr3:169663335-169663618
chr3	170357678	170357888	chr3:170357677-170357856
chr3	170358518	170358648	chr3:170358508-170358645
chr3	170359623	170361450	chr3:170359622-170361429
chr3	170374358	170374456	chr3:170374346-170374546
chr3	170374458	170374568	chr3:170374346-170374546
chr3	170381243	170381352	chr3:170381243-170381341
chr3	170384543	170384778	chr3:170384532-170384765
chr3	170390233	170390476	chr3:170390222-170390464
chr3	170391048	170391298	chr3:170391035-170391260
chr3	170392268	170392624	chr3:170392258-170396835
chr3	170392628	170394118	chr3:170392258-170396835
chr3	170394288	170395010	chr3:170392258-170396835
chr3	170395013	170396012	chr3:170392258-170396835
chr3	170396018	170396151	chr3:170392258-170396835
chr3	170396153	170396844	chr3:170392258-170396835
chr3	179322994	179323319	chr3:179322990-179323291
chr3	179323769	179323873	chr3:179323762-179323844
chr3	179327574	179327645	chr3:179327560-179327631
chr3	179327754	179327922	chr3:179327743-179327906
chr3	179328314	179328381	chr3:179328282-179328351
chr3	179329619	179329760	chr3:179329617-179329728
chr3	179332999	179333129	chr3:179332988-179333123
chr3	179333274	179334932	chr3:179333268-179338583
chr3	179334949	179335126	chr3:179333268-179338583
chr3	179335369	179335556	chr3:179333268-179338583
chr3	179335559	179335776	chr3:179333268-179338583
chr3	179336064	179336201	chr3:179333268-179338583
chr3	179336229	179336982	chr3:179333268-179338583

chr3	179337274	179337349	chr3:179333268-179338583
chr3	179337624	179337756	chr3:179333268-179338583
chr3	179338054	179338115	chr3:179333268-179338583
chr3	179338154	179338615	chr3:179333268-179338583
chr3	181711924	181712041	chr3:181711923-181714436
chr3	181712104	181712496	chr3:181711923-181714436
chr3	181712549	181713416	chr3:181711923-181714436
chr3	181713444	181714459	chr3:181711923-181714436
chr3	183253373	183253485	chr3:183253372-183253472
chr3	183254133	183254240	chr3:183254133-183254233
chr3	183265433	183265544	chr3:183265423-183265523
chr3	183269503	183269671	chr3:183269497-183269647
chr3	183274848	183275073	chr3:183274836-183275036
chr3	185645560	185645662	chr3:185645473-185645623
chr3	185647070	185647147	chr3:185647024-185647124
chr3	185818120	185818331	chr3:185818110-185818310
chr3	186046320	186046467	chr3:186046307-186048860
chr3	186046480	186046662	chr3:186046307-186048860
chr3	186046685	186047272	chr3:186046307-186048860
chr3	186047275	186047786	chr3:186046307-186048860
chr3	186047800	186048895	chr3:186046307-186048860
chr3	186052030	186052146	chr3:186052029-186052131
chr3	186057075	186057249	chr3:186057074-186057244
chr3	186057425	186057499	chr3:186057422-186057491
chr3	186064215	186064324	chr3:186064212-186064560
chr3	186064385	186064602	chr3:186064212-186064560
chr3	186065815	186066102	chr3:186065812-186066072
chr3	186077995	186078239	chr3:186077992-186078214
chr3	186079035	186079138	chr3:186079031-186079105
chr3	186079820	186080072	chr3:186079816-186080104
chr3	186081045	186081204	chr3:186081045-186081175
chr3	186084170	186084277	chr3:186084168-186084280
chr3	186095125	186095334	chr3:186095119-186095313
chr3	186099830	186100041	chr3:186099820-186100020
chr3	186105310	186105383	chr3:186105304-186105355
chr3	186105450	186105527	chr3:186105448-186105496
chr3	186105635	186105745	chr3:186105624-186105712
chr3	186105825	186105966	chr3:186105823-186105942
chr3	186108460	186108710	chr3:186108456-186108675
chr3	186108840	186108933	chr3:186108827-186108929
chr3	186108950	186109125	chr3:186108939-186109112
chr3	186110275	186110352	chr3:186110268-186110318
chr3	187721390	187721692	chr3:187721376-187722601
chr3	187721715	187721921	chr3:187721376-187722601
chr3	187721980	187722645	chr3:187721376-187722601
chr3	187724950	187725092	chr3:187724940-187725078
chr3	187725500	187725646	chr3:187725498-187725629
chr3	187726730	187726914	chr3:187726730-187726898
chr3	187727535	187727744	chr3:187727526-187727726
chr3	187728360	187728580	chr3:187728359-187728544
chr3	187729050	187730037	chr3:187729049-187730021
chr3	187731715	187731959	chr3:187731708-187731930
chr3	187733540	187733718	chr3:187733532-187733703
chr3	187734870	187734983	chr3:187734868-187734947
chr3	187736100	187737294	chr3:187736089-187737274
chr3	187737605	187737739	chr3:187737596-187737944
chr3	187737770	187737981	chr3:187737596-187737944
chr3	189631446	189631597	chr3:189631426-189631577
chr3	189640271	189640481	chr3:189640259-189640459
chr3	189737751	189737886	chr3:189737739-189737868
chr3	189738651	189738793	chr3:189738641-189738774
chr3	189789666	189789880	chr3:189789659-189789842
chr3	189808276	189808558	chr3:189808271-189808526

chr3	189864231	189864458	chr3:189864231-189864418
chr3	189866691	189866822	chr3:189866681-189866797
chr3	189867841	189867979	chr3:189867832-189867942
chr3	189868581	189868736	chr3:189868579-189868716
chr3	189869336	189869435	chr3:189869323-189869406
chr3	189872866	189873017	chr3:189872858-189872995
chr3	189880076	189880209	chr3:189880063-189881496
chr3	189880256	189881265	chr3:189880063-189881496
chr3	189881311	189881512	chr3:189880063-189881496
chr3	189886396	189886592	chr3:189886393-189886551
chr3	189889341	189889525	chr3:189889339-189889484
chr3	189890801	189890902	chr3:189890788-189890882
chr3	189894216	189896273	chr3:189894205-189897279
chr3	189896296	189896432	chr3:189894205-189897279
chr3	189896466	189896976	chr3:189894205-189897279
chr3	189896981	189897047	chr3:189894205-189897279
chr3	189897051	189897252	chr3:189894205-189897279
chr3	194136155	194136350	chr3:194136144-194136488
chr3	194136370	194136516	chr3:194136144-194136488
chr3	194136625	194136744	chr3:194136616-194136712
chr3	194136970	194137072	chr3:194136960-194137048
chr3	194137695	194138755	chr3:194137682-194138732
chr3	196052043	196052216	chr3:196052034-196052184
chr3	196053418	196053542	chr3:196053417-196053517
chr4	1725799	1726024	chr4:1725796-1725996
chr4	1744739	1744857	chr4:1744732-1744832
chr4	1744949	1745126	chr4:1744947-1745097
chr4	1797489	1797703	chr4:1797476-1797676
chr4	1806844	1806948	chr4:1806834-1806934
chr4	1807119	1807301	chr4:1807115-1807265
chr4	2061069	2061179	chr4:2061062-2061162
chr4	2062544	2062767	chr4:2062544-2062744
chr4	2063764	2063938	chr4:2063759-2063909
chr4	2247434	2249430	chr4:2247431-2250701
chr4	2249459	2250730	chr4:2247431-2250701
chr4	2251084	2251278	chr4:2251083-2251246
chr4	2252409	2252560	chr4:2252407-2252522
chr4	2255014	2255370	chr4:2255008-2255351
chr4	2257974	2258051	chr4:2257981-2258011
chr4	2258884	2259068	chr4:2258884-2259057
chr4	2259779	2259996	chr4:2259772-2259972
chr4	2261734	2261839	chr4:2261724-2261824
chr4	2261919	2262320	chr4:2261916-2262294
chr4	4859679	4860382	chr4:4859665-4860368
chr4	4862434	4862647	chr4:4862427-4862608
chr4	4862714	4863383	chr4:4862700-4863936
chr4	4863384	4863559	chr4:4862700-4863936
chr4	4863589	4863761	chr4:4862700-4863936
chr4	4863774	4863963	chr4:4862700-4863936
chr4	8846077	8846367	chr4:8846075-8846324
chr4	8867057	8868380	chr4:8867047-8868345
chr4	8869697	8869910	chr4:8869683-8869883
chr4	8871222	8871859	chr4:8871220-8871817
chr4	13540830	13542538	chr4:13540829-13542528
chr4	13543145	13543362	chr4:13543138-13543338
chr4	13543950	13545075	chr4:13543948-13545050
chr4	17460751	17460833	chr4:17460685-17460835
chr4	17487091	17487259	chr4:17487086-17487236
chr4	17490661	17490780	chr4:17490661-17490745
chr4	17498376	17498579	chr4:17498365-17498565
chr4	17501731	17501852	chr4:17501718-17501818
chr4	17826307	17826530	chr4:17826303-17826503
chr4	17841212	17841880	chr4:17841198-17845901

chr4	17841887	17845913	chr4:17841198-17845901
chr4	17873387	17875291	chr4:17873387-17878213
chr4	17875297	17877043	chr4:17873387-17878213
chr4	17877057	17878208	chr4:17873387-17878213
chr4	17880622	17881143	chr4:17880594-17884752
chr4	17881172	17881976	chr4:17880594-17884752
chr4	17881987	17882923	chr4:17880594-17884752
chr4	17882947	17883658	chr4:17880594-17884752
chr4	17883692	17884792	chr4:17880594-17884752
chr4	17886067	17886178	chr4:17886067-17886161
chr4	17909102	17909378	chr4:17909093-17909345
chr4	17945552	17945771	chr4:17945544-17945744
chr4	17960257	17960331	chr4:17960245-17960311
chr4	17961912	17962051	chr4:17961902-17962032
chr4	17962972	17963081	chr4:17962969-17963049
chr4	17972822	17972891	chr4:17972819-17972885
chr4	18020572	18020756	chr4:18020530-18020734
chr4	18021597	18021673	chr4:18021597-18021876
chr4	18021702	18021901	chr4:18021597-18021876
chr4	26163464	26163640	chr4:26163454-26163614
chr4	26272614	26272783	chr4:26272606-26272761
chr4	26319674	26319911	chr4:26319661-26319888
chr4	26320564	26321058	chr4:26320562-26321048
chr4	26321374	26321908	chr4:26321364-26321871
chr4	26322164	26322323	chr4:26322163-26322297
chr4	26322874	26322983	chr4:26322869-26322950
chr4	26343144	26343246	chr4:26343139-26343223
chr4	26359704	26359814	chr4:26359690-26359775
chr4	26362449	26362526	chr4:26362435-26362628
chr4	26362574	26362647	chr4:26362435-26362628
chr4	26367884	26368168	chr4:26367873-26368145
chr4	26384529	26384636	chr4:26384528-26384608
chr4	26386349	26386420	chr4:26386352-26386391
chr4	26406174	26406304	chr4:26406174-26406270
chr4	26410044	26410326	chr4:26410041-26410292
chr4	26412934	26413008	chr4:26412929-26412987
chr4	26415529	26415602	chr4:26415474-26415640
chr4	26417654	26417863	chr4:26417648-26417848
chr4	26420559	26420642	chr4:26420550-26420725
chr4	26424394	26424500	chr4:26424341-26424479
chr4	26424634	26424783	chr4:26424630-26424743
chr4	26428769	26428845	chr4:26428719-26428860
chr4	26429924	26430066	chr4:26429897-26430053
chr4	26430419	26430532	chr4:26430418-26430522
chr4	26430699	26431188	chr4:26430691-26435131
chr4	26431279	26431584	chr4:26430691-26435131
chr4	26431619	26431828	chr4:26430691-26435131
chr4	26431839	26432392	chr4:26430691-26435131
chr4	26432414	26433593	chr4:26430691-26435131
chr4	26433604	26433920	chr4:26430691-26435131
chr4	26433929	26434253	chr4:26430691-26435131
chr4	26434269	26435002	chr4:26430691-26435131
chr4	26435014	26435152	chr4:26430691-26435131
chr4	38664198	38664490	chr4:38664195-38664461
chr4	38673113	38673313	chr4:38673101-38673301
chr4	38680593	38680704	chr4:38680586-38680682
chr4	38688588	38689092	chr4:38688584-38689071
chr4	38689768	38690770	chr4:38689728-38690751
chr4	38694758	38694934	chr4:38694745-38694906
chr4	38697088	38697470	chr4:38697081-38701042
chr4	38697488	38699228	chr4:38697081-38701042
chr4	38699278	38701086	chr4:38697081-38701042
chr4	40426005	40426177	chr4:40425993-40426143

chr4	40432690	40432782	chr4:40432650-40432750
chr4	40474470	40474699	chr4:40474466-40474666
chr4	41744093	41745308	chr4:41744081-41746322
chr4	41745353	41745761	chr4:41744081-41746322
chr4	41745793	41746351	chr4:41744081-41746322
chr4	41747353	41747563	chr4:41747348-41747536
chr4	41747658	41747728	chr4:41747647-41747709
chr4	41748378	41749009	chr4:41748369-41748970
chr4	47847238	47848355	chr4:47847232-47848336
chr4	47851098	47851173	chr4:47851094-47851148
chr4	47851858	47851965	chr4:47851855-47851942
chr4	47855068	47855171	chr4:47855058-47855163
chr4	47862848	47862920	chr4:47862845-47862915
chr4	47875133	47875315	chr4:47875126-47875293
chr4	47877023	47877232	chr4:47877011-47877191
chr4	47878533	47878671	chr4:47878524-47878665
chr4	47884353	47884459	chr4:47884345-47884437
chr4	47885498	47885677	chr4:47885497-47885657
chr4	47885883	47886023	chr4:47885878-47885999
chr4	47887243	47887452	chr4:47887231-47887431
chr4	47890623	47890722	chr4:47890612-47890703
chr4	47894193	47894326	chr4:47894179-47894302
chr4	47896523	47896665	chr4:47896522-47896647
chr4	47897978	47898115	chr4:47897966-47898081
chr4	47898758	47898877	chr4:47898756-47898857
chr4	47898963	47899135	chr4:47898958-47899120
chr4	47899383	47899581	chr4:47899369-47899548
chr4	47903193	47903340	chr4:47903192-47903323
chr4	47905238	47905380	chr4:47905236-47905346
chr4	47910823	47911002	chr4:47910823-47910994
chr4	48066393	48067723	chr4:48066392-48067705
chr4	48071528	48071700	chr4:48071516-48071674
chr4	48073948	48074083	chr4:48073934-48074053
chr4	48076403	48076478	chr4:48076401-48076466
chr4	48079913	48080202	chr4:48079911-48080178
chr4	48086468	48086645	chr4:48086465-48086637
chr4	48089683	48089866	chr4:48089384-48089824
chr4	48094083	48094224	chr4:48094076-48094204
chr4	48095148	48095252	chr4:48095142-48095222
chr4	48104903	48104978	chr4:48104900-48104955
chr4	48110548	48110618	chr4:48110537-48110603
chr4	48112318	48112529	chr4:48112306-48112512
chr4	48113213	48113325	chr4:48113206-48113309
chr4	48114358	48114562	chr4:48114347-48114545
chr4	48127593	48127804	chr4:48127582-48127782
chr4	48134158	48134282	chr4:48134154-48134256
chr4	48485053	48485150	chr4:48485042-48485142
chr4	48486813	48486897	chr4:48486813-48487013
chr4	48486903	48487045	chr4:48486813-48487013
chr4	48488433	48488613	chr4:48488426-48488576
chr4	54099530	54099676	chr4:54099522-54099636
chr4	54100035	54100735	chr4:54100030-54100918
chr4	54100810	54100940	chr4:54100030-54100918
chr4	54101595	54102444	chr4:54101581-54102505
chr4	54667660	54667882	chr4:54667648-54667848
chr4	54737180	54737309	chr4:54737180-54737280
chr4	54738430	54738621	chr4:54738428-54738578
chr4	55427916	55428048	chr4:55427902-55435594
chr4	55428051	55428777	chr4:55427902-55435594
chr4	55428796	55428975	chr4:55427902-55435594
chr4	55428996	55429480	chr4:55427902-55435594
chr4	55429481	55430068	chr4:55427902-55435594
chr4	55430076	55430430	chr4:55427902-55435594

chr4	55430441	55431690	chr4:55427902-55435594
chr4	55431711	55432473	chr4:55427902-55435594
chr4	55432491	55434593	chr4:55427902-55435594
chr4	55434601	55435621	chr4:55427902-55435594
chr4	55438281	55438366	chr4:55438281-55438537
chr4	55438371	55438543	chr4:55438281-55438537
chr4	55442431	55442650	chr4:55442431-55442634
chr4	55443686	55443897	chr4:55443686-55443896
chr4	55444646	55444818	chr4:55444632-55444785
chr4	55448781	55448892	chr4:55448778-55448868
chr4	55449396	55449517	chr4:55449395-55449496
chr4	55450101	55450272	chr4:55450090-55450232
chr4	55453681	55453850	chr4:55453676-55453824
chr4	55455896	55456036	chr4:55455896-55456003
chr4	55456231	55456327	chr4:55456217-55456300
chr4	55458891	55459039	chr4:55458891-55459010
chr4	55459161	55459296	chr4:55459147-55459261
chr4	55463686	55463830	chr4:55463684-55463805
chr4	55470726	55470830	chr4:55470716-55470806
chr4	55475976	55476094	chr4:55475962-55476054
chr4	55478826	55478999	chr4:55478814-55478963
chr4	55479641	55479714	chr4:55479639-55479699
chr4	55482741	55482844	chr4:55482738-55482828
chr4	55486401	55486578	chr4:55486392-55486549
chr4	55489386	55489482	chr4:55489373-55489465
chr4	55497966	55498185	chr4:55497941-55498141
chr4	55509911	55510100	chr4:55509911-55510065
chr4	55545466	55545536	chr4:55545452-55545932
chr4	55545546	55545964	chr4:55545452-55545932
chr4	55546486	55546667	chr4:55546481-55546655
chr4	55546781	55547158	chr4:55546781-55547138
chr4	56907886	56908224	chr4:56907875-56908213
chr4	56908916	56909060	chr4:56908912-56909039
chr4	56909201	56909610	chr4:56909189-56909587
chr4	56910631	56911547	chr4:56910629-56911536
chr4	56919786	56919899	chr4:56919786-56919870
chr4	56927616	56927692	chr4:56927614-56927664
chr4	56929851	56930490	chr4:56929840-56935844
chr4	56930491	56931431	chr4:56929840-56935844
chr4	56931446	56932591	chr4:56929840-56935844
chr4	56932596	56932771	chr4:56929840-56935844
chr4	56932796	56932866	chr4:56929840-56935844
chr4	56932876	56933212	chr4:56929840-56935844
chr4	56933221	56933851	chr4:56929840-56935844
chr4	56933856	56935011	chr4:56929840-56935844
chr4	56935021	56935510	chr4:56929840-56935844
chr4	56935541	56935611	chr4:56929840-56935844
chr4	56935691	56935758	chr4:56929840-56935844
chr4	56935771	56935866	chr4:56929840-56935844
chr4	56952561	56952780	chr4:56952554-56952754
chr4	71238775	71238979	chr4:71238764-71238964
chr4	71466490	71466592	chr4:71466477-71466577
chr4	71472705	71472886	chr4:71472698-71472848
chr4	71567015	71567111	chr4:71567003-71567083
chr4	71567800	71567963	chr4:71567787-71567937
chr4	84491994	84493333	chr4:84491986-84493549
chr4	84493339	84493544	chr4:84491986-84493549
chr4	84494634	84494853	chr4:84494631-84494831
chr4	84495674	84495850	chr4:84495671-84495844
chr4	84496494	84496989	chr4:84496492-84496950
chr4	84497564	84497708	chr4:84497558-84498450
chr4	84497719	84498486	chr4:84497558-84498450
chr4	93828753	93830659	chr4:93828752-93830964

chr4	93830683	93830749	chr4:93828752-93830964
chr4	93830753	93830998	chr4:93828752-93830964
chr4	95058698	95058925	chr4:95058697-95058897
chr4	95114728	95114847	chr4:95114727-95114827
chr4	95115698	95115796	chr4:95115684-95115789
chr4	95152678	95152789	chr4:95152673-95152773
chr4	95154558	95154728	chr4:95154547-95154697
chr4	102501330	102501816	chr4:102501328-102501788
chr4	102501910	102502197	chr4:102501897-102502167
chr4	102503380	102503510	chr4:102503374-102503490
chr4	102510880	102510953	chr4:102510879-102510949
chr4	102522065	102522273	chr4:102522059-102522259
chr4	102525510	102525589	chr4:102525511-102525557
chr4	102529845	102529950	chr4:102529835-102529914
chr4	102533840	102533910	chr4:102533844-102533885
chr4	102537585	102537665	chr4:102537586-102537628
chr4	102537860	102537965	chr4:102537857-102537956
chr4	102557115	102557220	chr4:102557104-102557206
chr4	102567045	102567144	chr4:102566986-102567135
chr4	102576880	102577066	chr4:102576875-102577039
chr4	102577725	102578422	chr4:102577713-102579039
chr4	102578435	102578549	chr4:102577713-102579039
chr4	102578550	102579076	chr4:102577713-102579039
chr4	102580540	102580644	chr4:102580534-102580639
chr4	102582865	102582972	chr4:102582865-102582957
chr4	102584690	102584824	chr4:102584681-102584820
chr4	102593425	102593597	chr4:102593424-102593568
chr4	102594900	102595014	chr4:102594891-102594981
chr4	102596140	102596355	chr4:102596137-102596332
chr4	102597525	102597704	chr4:102597519-102597661
chr4	102600895	102601051	chr4:102600894-102601009
chr4	102606500	102606713	chr4:102606495-102606697
chr4	102607150	102607348	chr4:102607149-102607319
chr4	102607650	102607781	chr4:102607648-102607751
chr4	102610585	102610731	chr4:102610574-102610699
chr4	102612055	102612131	chr4:102612043-102612110
chr4	102612435	102612622	chr4:102612433-102612606
chr4	102613435	102613585	chr4:102613424-102613581
chr4	102616445	102617102	chr4:102616433-102617302
chr4	102617120	102617256	chr4:102616433-102617302
chr4	102617265	102617338	chr4:102616433-102617302
chr4	108047569	108047877	chr4:108047544-108048774
chr4	108047894	108048162	chr4:108047544-108048774
chr4	108048254	108048778	chr4:108047544-108048774
chr4	108063619	108063695	chr4:108063622-108063663
chr4	108070669	108070785	chr4:108070661-108070770
chr4	108071669	108071814	chr4:108071657-108071778
chr4	108072014	108072283	chr4:108072002-108072251
chr4	108072839	108072907	chr4:108072836-108072896
chr4	108075374	108075486	chr4:108075374-108075477
chr4	108078219	108078504	chr4:108078219-108078462
chr4	108079494	108079638	chr4:108079491-108079614
chr4	108081589	108081706	chr4:108081583-108081669
chr4	108083224	108083476	chr4:108083212-108083446
chr4	108089124	108089280	chr4:108089124-108089257
chr4	108140884	108141104	chr4:108140883-108141083
chr4	108163569	108163722	chr4:108163567-108163701
chr4	108165104	108165281	chr4:108165096-108165257
chr4	108166284	108166803	chr4:108166271-108166797
chr4	108167564	108168367	chr4:108167554-108168956
chr4	108168379	108168980	chr4:108167554-108168956
chr4	110617424	110618716	chr4:110617422-110618688
chr4	110620004	110620247	chr4:110619999-110621369

chr4	110620264	110620545	chr4:110619999-110621369
chr4	110620554	110621391	chr4:110619999-110621369
chr4	110622269	110623134	chr4:110622255-110623103
chr4	110632354	110632488	chr4:110632342-110632480
chr4	110632954	110633029	chr4:110632952-110633008
chr4	110634159	110634410	chr4:110634157-110634391
chr4	110636794	110638196	chr4:110636780-110638154
chr4	110640269	110640471	chr4:110640255-110640455
chr4	110641929	110642148	chr4:110641918-110642123
chr4	112513526	112515505	chr4:112513515-112515476
chr4	112515846	112516026	chr4:112515846-112516172
chr4	112516041	112516210	chr4:112515846-112516172
chr4	119318955	119319121	chr4:119318941-119319091
chr4	119319535	119319642	chr4:119319535-119319635
chr4	120684930	120685062	chr4:120684918-120685025
chr4	120691955	120692348	chr4:120691914-120695275
chr4	120692365	120694725	chr4:120691914-120695275
chr4	120694745	120695302	chr4:120691914-120695275
chr4	120710320	120710429	chr4:120710308-120710413
chr4	120754560	120754669	chr4:120754552-120754638
chr4	120762380	120762797	chr4:120762379-120762766
chr4	120777190	120777298	chr4:120777187-120777281
chr4	120781155	120781324	chr4:120781142-120781303
chr4	120785010	120785108	chr4:120784997-120785091
chr4	120798280	120798444	chr4:120798266-120798424
chr4	120799660	120799783	chr4:120799660-120799745
chr4	120811375	120811486	chr4:120811369-120811449
chr4	120812705	120812848	chr4:120812550-120812814
chr4	120816460	120816609	chr4:120816452-120816574
chr4	120816845	120816942	chr4:120816831-120816924
chr4	120818365	120818545	chr4:120818352-120818527
chr4	120821175	120821350	chr4:120821170-120821345
chr4	120837345	120838322	chr4:120837344-120839274
chr4	120838660	120838734	chr4:120837344-120839274
chr4	120838750	120839115	chr4:120837344-120839274
chr4	120839170	120839305	chr4:120837344-120839274
chr4	120853430	120853558	chr4:120853417-120853540
chr4	120869650	120869874	chr4:120869646-120869846
chr4	120907485	120907587	chr4:120907473-120907557
chr4	120922515	120922903	chr4:120922515-120922870
chr4	139028124	139028373	chr4:139028111-139028349
chr4	139057219	139058005	chr4:139057219-139059607
chr4	139058024	139058167	chr4:139057219-139059607
chr4	139058199	139058273	chr4:139057219-139059607
chr4	139058299	139058395	chr4:139057219-139059607
chr4	139058489	139058599	chr4:139057219-139059607
chr4	139058619	139058762	chr4:139057219-139059607
chr4	139058819	139058890	chr4:139057219-139059607
chr4	139059074	139059217	chr4:139057219-139059607
chr4	139059264	139059497	chr4:139057219-139059607
chr4	139059514	139059625	chr4:139057219-139059607
chr4	139060404	139060616	chr4:139060323-139060674
chr4	139060629	139060697	chr4:139060323-139060674
chr4	139061869	139062079	chr4:139061864-139062057
chr4	139067689	139067790	chr4:139067683-139067770
chr4	139071874	139072051	chr4:139071865-139072039
chr4	139073454	139073602	chr4:139073453-139073567
chr4	139084139	139084411	chr4:139084132-139084496
chr4	139084449	139084524	chr4:139084132-139084496
chr4	139101599	139101980	chr4:139101590-139102353
chr4	139102029	139102240	chr4:139101590-139102353
chr4	139116649	139116827	chr4:139116648-139116807
chr4	139125169	139125346	chr4:139125163-139125329

chr4	139137639	139137879	chr4:139137629-139137867
chr4	139139424	139139518	chr4:139139412-139139497
chr4	139175764	139175974	chr4:139175755-139175955
chr4	139176979	139177251	chr4:139176966-139177218
chr4	145481203	145481410	chr4:145481193-145481400
chr4	145481698	145481774	chr4:145481697-145481752
chr4	145481793	145482067	chr4:145481791-145482038
chr4	145482498	145483202	chr4:145482485-145483176
chr4	145484513	145484799	chr4:145484512-145484764
chr4	145487653	145487793	chr4:145487579-145487779
chr4	145503893	145503992	chr4:145503851-145503979
chr4	145513243	145513312	chr4:145513240-145513378
chr4	145513318	145513409	chr4:145513240-145513378
chr4	145514438	145515038	chr4:145514437-145515013
chr4	145525728	145525834	chr4:145525719-145525815
chr4	145539813	145540104	chr4:145539803-145540061
chr4	145542593	145542726	chr4:145542581-145542698
chr4	145546088	145546190	chr4:145546076-145546171
chr4	145546703	145546945	chr4:145546702-145546924
chr4	145553793	145554077	chr4:145553783-145554040
chr4	145557803	145558078	chr4:145557790-145558079
chr4	146638893	146639294	chr4:146638892-146639428
chr4	146639328	146639467	chr4:146638892-146639428
chr4	146639868	146640083	chr4:146639866-146642474
chr4	146640113	146641334	chr4:146639866-146642474
chr4	146641348	146641690	chr4:146639866-146642474
chr4	146641693	146641865	chr4:146639866-146642474
chr4	146641878	146642508	chr4:146639866-146642474
chr4	148078763	148080457	chr4:148078761-148081499
chr4	148080463	148080879	chr4:148078761-148081499
chr4	148080893	148081528	chr4:148078761-148081499
chr4	148114113	148114282	chr4:148114103-148114261
chr4	148120158	148120311	chr4:148120157-148120288
chr4	148146588	148146772	chr4:148146587-148146757
chr4	148152478	148152647	chr4:148152468-148152613
chr4	148154563	148154805	chr4:148154550-148154901
chr4	148154833	148154927	chr4:148154550-148154901
chr4	148165653	148165859	chr4:148165642-148165842
chr4	148194758	148194866	chr4:148194745-148194862
chr4	148201048	148201153	chr4:148201038-148201132
chr4	148259968	148260144	chr4:148259965-148260117
chr4	148368268	148368484	chr4:148368264-148368470
chr4	148435113	148436888	chr4:148435103-148436862
chr4	148442163	148442416	chr4:148442159-148442520
chr4	148442458	148442553	chr4:148442159-148442520
chr4	148444528	148444721	chr4:148444525-148444698
chr4	173524982	173525055	chr4:173524968-173525028
chr4	173525272	173525388	chr4:173525272-173525377
chr4	173526097	173526614	chr4:173526090-173527375
chr4	173526622	173527255	chr4:173526090-173527375
chr4	173527262	173527406	chr4:173526090-173527375
chr4	173528087	173529176	chr4:173528078-173530229
chr4	173529217	173529385	chr4:173528078-173530229
chr4	173529387	173529559	chr4:173528078-173530229
chr4	173529572	173529919	chr4:173528078-173530229
chr4	173529932	173530251	chr4:173528078-173530229
chr4	184387725	184387824	chr4:184387712-184389066
chr4	184387825	184387956	chr4:184387712-184389066
chr4	184387975	184388220	chr4:184387712-184389066
chr4	184388230	184388679	chr4:184387712-184389066
chr4	184388710	184389091	chr4:184387712-184389066
chr4	184390710	184390790	chr4:184390702-184390749
chr4	184398915	184399108	chr4:184398914-184399079

chr4	184407190	184407292	chr4:184407178-184407283
chr4	184408160	184408272	chr4:184408157-184408275
chr4	184418165	184418234	chr4:184418166-184418213
chr4	184418510	184418719	chr4:184418504-184418708
chr4	184419480	184419578	chr4:184419468-184419568
chr4	184428980	184429096	chr4:184428977-184429070
chr4	184448655	184449090	chr4:184448655-184449050
chr4	184462440	184462648	chr4:184462427-184462627
chr4	184473980	184474085	chr4:184473968-184474049
chr4	184474115	184474230	chr4:184474105-184474186
chr4	184474380	184474590	chr4:184474378-184474580
chr4	185018850	185019118	chr4:185018840-185019087
chr4	185019395	185019502	chr4:185019386-185019491
chr4	185019625	185019868	chr4:185019623-185019843
chr4	185020275	185020843	chr4:185020272-185020804
chr4	187995770	187995839	chr4:187995770-187995840
chr4	187999185	187999304	chr4:187999107-187999273
chr4	187999610	187999733	chr4:187999608-187999685
chr4	188001205	188001418	chr4:188001192-188001392
chr4	188002715	188003900	chr4:188002707-188005050
chr4	188003950	188004190	chr4:188002707-188005050
chr4	188004805	188005065	chr4:188002707-188005050
chr4	190175746	190175823	chr4:190175646-190175845
chr4	190180636	190180858	chr4:190180636-190180836
chr4	190182116	190182192	chr4:190182116-190182181
chr4	190183261	190183335	chr4:190183235-190183303
chr4	190185341	190185412	chr4:190185340-190185439
chr5	1394622	1394789	chr5:1394608-1394758
chr5	1400917	1400992	chr5:1400914-1400986
chr5	1423712	1423924	chr5:1423708-1423908
chr5	2745847	2745919	chr5:2745844-2747616
chr5	2745937	2746014	chr5:2745844-2747616
chr5	2746017	2746261	chr5:2745844-2747616
chr5	2746272	2746755	chr5:2745844-2747616
chr5	2746757	2747212	chr5:2745844-2747616
chr5	2747327	2747638	chr5:2745844-2747616
chr5	2748347	2749089	chr5:2748344-2749052
chr5	2749382	2749525	chr5:2749381-2749787
chr5	2749632	2749802	chr5:2749381-2749787
chr5	2751192	2751682	chr5:2751164-2751662
chr5	3596066	3596413	chr5:3596053-3596381
chr5	3597706	3597930	chr5:3597703-3597903
chr5	3599231	3599376	chr5:3599224-3600260
chr5	3599491	3600291	chr5:3599224-3600260
chr5	3600621	3600719	chr5:3600608-3600681
chr5	3600991	3601059	chr5:3600982-3601403
chr5	3601076	3601431	chr5:3600982-3601403
chr5	21752096	21752281	chr5:21752086-21752236
chr5	21755591	21755716	chr5:21755590-21755690
chr5	22157456	22157684	chr5:22157453-22157653
chr5	32531636	32531958	chr5:32531632-32531916
chr5	32538731	32538952	chr5:32538717-32538917
chr5	32571096	32571212	chr5:32571090-32571174
chr5	32585511	32585997	chr5:32585498-32585971
chr5	32586371	32586512	chr5:32586368-32586501
chr5	32588546	32588626	chr5:32588511-32588584
chr5	32591576	32591684	chr5:32591562-32591687
chr5	32594571	32594648	chr5:32594570-32594872
chr5	32594766	32594836	chr5:32594570-32594872
chr5	32598971	32599102	chr5:32598960-32599069
chr5	32601061	32601132	chr5:32601004-32604079
chr5	32601156	32603789	chr5:32601004-32604079
chr5	32603796	32603900	chr5:32601004-32604079

chr5	32603901	32604108	chr5:32601004-32604079
chr5	43067583	43067799	chr5:43067580-43067786
chr5	43067973	43068235	chr5:43067969-43068217
chr5	43080368	43080530	chr5:43080205-43080503
chr5	43093033	43093593	chr5:43093022-43093558
chr5	43096598	43096827	chr5:43096595-43096795
chr5	43099023	43099236	chr5:43099019-43099338
chr5	43116453	43116668	chr5:43116446-43116630
chr5	43120883	43121134	chr5:43120882-43121123
chr5	43121528	43121955	chr5:43121532-43121568;chr5:43121595-43122177
chr5	43121963	43122209	chr5:43121595-43122177
chr5	43123208	43123313	chr5:43123208-43123310
chr5	43139178	43139346	chr5:43139164-43139309
chr5	43161258	43161948	chr5:43161248-43161931
chr5	43167933	43168019	chr5:43167929-43168029
chr5	43173323	43173487	chr5:43173317-43173448
chr5	43174448	43175134	chr5:43174446-43176324
chr5	43175148	43175704	chr5:43174446-43176324
chr5	43175733	43176346	chr5:43174446-43176324
chr5	43191753	43191932	chr5:43191752-43191920
chr5	51383403	51383720	chr5:51383390-51383699
chr5	51384548	51384755	chr5:51384540-51384730
chr5	51386213	51386386	chr5:51386203-51386626
chr5	51386408	51386660	chr5:51386203-51386626
chr5	51387498	51387790	chr5:51387489-51387749
chr5	51388603	51388811	chr5:51388597-51388797
chr5	51389658	51389972	chr5:51389645-51389932
chr5	51391283	51391462	chr5:51391273-51391441
chr5	51393498	51394418	chr5:51393493-51394738
chr5	51394468	51394716	chr5:51393493-51394738
chr5	58825075	58825177	chr5:58825062-58825162
chr5	58851175	58851357	chr5:58851163-58851313
chr5	73444840	73445176	chr5:73444826-73445177
chr5	73446265	73447314	chr5:73446255-73448527
chr5	73447320	73447454	chr5:73446255-73448527
chr5	73447470	73447778	chr5:73446255-73448527
chr5	73447865	73448038	chr5:73446255-73448527
chr5	73448045	73448155	chr5:73446255-73448527
chr5	73448210	73448558	chr5:73446255-73448527
chr5	77628739	77629397	chr5:77628711-77630794
chr5	77629399	77630831	chr5:77628711-77630794
chr5	77634729	77634797	chr5:77634728-77634928
chr5	77634804	77634938	chr5:77634728-77634928
chr5	77635934	77636745	chr5:77635930-77636712
chr5	77636824	77637249	chr5:77636820-77637230
chr5	77638514	77639709	chr5:77638512-77639688
chr5	80319624	80319740	chr5:80319624-80319701
chr5	80319974	80321838	chr5:80319974-80321842
chr5	88717128	88719032	chr5:88717116-88722925
chr5	88719043	88720196	chr5:88717116-88722925
chr5	88720223	88720337	chr5:88717116-88722925
chr5	88720353	88720796	chr5:88717116-88722925
chr5	88720803	88721926	chr5:88717116-88722925
chr5	88721983	88722560	chr5:88717116-88722925
chr5	88722583	88722965	chr5:88717116-88722925
chr5	88726403	88726602	chr5:88726393-88726593
chr5	88728493	88728643	chr5:88728492-88728628
chr5	88729223	88729356	chr5:88729217-88729347
chr5	88731728	88731912	chr5:88731728-88731901
chr5	88732473	88732588	chr5:88732471-88732543
chr5	88749082	88749157	chr5:88749069-88749117
chr5	88751857	88752039	chr5:88751856-88752043
chr5	88761037	88761151	chr5:88760987-88761125

chr5	88761197	88761365	chr5:88761184-88761328
chr5	88761727	88761944	chr5:88761717-88761912
chr5	88771787	88771897	chr5:88771778-88771866
chr5	88772267	88772372	chr5:88772265-88772361
chr5	88785342	88785572	chr5:88785338-88785542
chr5	88804607	88804822	chr5:88804597-88804801
chr5	88823737	88823843	chr5:88823734-88824275
chr5	88823847	88824379	chr5:88823734-88824275;chr5:88824309-88824340
chr5	88824882	88824951	chr5:88824879-88824914
chr5	88825722	88825904	chr5:88825718-88825864
chr5	88827047	88827220	chr5:88827035-88827192
chr5	88839252	88839329	chr5:88839239-88839309
chr5	88839387	88839628	chr5:88839378-88839623
chr5	88877947	88878041	chr5:88877937-88878020
chr5	88880887	88881297	chr5:88880843-88881264
chr5	88883027	88883308	chr5:88882954-88883466
chr5	88883347	88883481	chr5:88882954-88883466
chr5	88883652	88883760	chr5:88883651-88883747
chr5	88884332	88884445	chr5:88884327-88884426
chr5	88884492	88884566	chr5:88884488-88884568
chr5	88887512	88887623	chr5:88887501-88887598
chr5	88888177	88888255	chr5:88888166-88888232
chr5	88888892	88888996	chr5:88888878-88888964
chr5	88889062	88889355	chr5:88889050-88889324
chr5	88903922	88904270	chr5:88903915-88904257
chr5	93583401	93583534	chr5:93583336-93585486
chr5	93583546	93583795	chr5:93583336-93585486
chr5	93583841	93584928	chr5:93583336-93585486
chr5	93584941	93585109	chr5:93583336-93585486
chr5	93585126	93585510	chr5:93583336-93585486
chr5	93587101	93587271	chr5:93587100-93587243
chr5	93587466	93587557	chr5:93587459-93587571
chr5	93587916	93588487	chr5:93587916-93588444
chr5	93590166	93590380	chr5:93590163-93590363
chr5	93591986	93592228	chr5:93591972-93592186
chr5	93593566	93594070	chr5:93593561-93594615
chr5	93594126	93594269	chr5:93593561-93594615
chr5	93594316	93594641	chr5:93593561-93594615
chr5	102371947	102372167	chr5:102371944-102372123
chr5	102373347	102373446	chr5:102373336-102373436
chr5	102443897	102444107	chr5:102443890-102444090
chr5	119629571	119629838	chr5:119629558-119629838
chr5	119630931	119631145	chr5:119630924-119631124
chr5	119632761	119632895	chr5:119632747-119632888
chr5	119633961	119634605	chr5:119633959-119634734
chr5	119634611	119634757	chr5:119633959-119634734
chr5	119635031	119635109	chr5:119635027-119635086
chr5	132481619	132482056	chr5:132481608-132484075
chr5	132482934	132484102	chr5:132481608-132484075
chr5	132484374	132484509	chr5:132484361-132484497
chr5	132485669	132485750	chr5:132485666-132485716
chr5	132489394	132489679	chr5:132489391-132489645
chr5	132490014	132490329	chr5:132490001-132490304
chr5	132490544	132490826	chr5:132490544-132490798
chr5	134114716	134115162	chr5:134114710-134115155
chr5	134115206	134115418	chr5:134115200-134115387
chr5	134115576	134116109	chr5:134115562-134116086
chr5	134123621	134123784	chr5:134123617-134123756
chr5	134129706	134129930	chr5:134129703-134129903
chr5	134138061	134138272	chr5:134138058-134138237
chr5	134138951	134139064	chr5:134138950-134139038
chr5	134140796	134140911	chr5:134140795-134140888
chr5	134141026	134141381	chr5:134141020-134141350

chr5	134142186	134142347	chr5:134142184-134142304
chr5	134142726	134142910	chr5:134142720-134142883
chr5	134142996	134143121	chr5:134142992-134143100
chr5	134143591	134143667	chr5:134143591-134143640
chr5	134144806	134144903	chr5:134144796-134144869
chr5	134145271	134145374	chr5:134145259-134145340
chr5	134145741	134147023	chr5:134145740-134146185;chr5:134146200-134148229
chr5	134147036	134147656	chr5:134146200-134148229
chr5	134147996	134148236	chr5:134146200-134148229
chr5	135027736	135028552	chr5:135027734-135029321
chr5	135028571	135029337	chr5:135027734-135029321
chr5	135029896	135030118	chr5:135029894-135030094
chr5	135031286	135031523	chr5:135031275-135031508
chr5	135032846	135033162	chr5:135032844-135033135
chr5	135033716	135034309	chr5:135033712-135034298
chr5	135034511	135034829	chr5:135034501-135034813
chr5	135534291	135535984	chr5:135534281-135535949
chr5	136132844	136132991	chr5:136132844-136132962
chr5	136133224	136133718	chr5:136133223-136133761
chr5	136133719	136133789	chr5:136133223-136133761
chr5	136142784	136142998	chr5:136142781-136142981
chr5	136147834	136147943	chr5:136147831-136147906
chr5	136153604	136154192	chr5:136153591-136154163
chr5	136160864	136161138	chr5:136160855-136161107
chr5	136163284	136163421	chr5:136163271-136163391
chr5	136172439	136172863	chr5:136172433-136172829
chr5	136174379	136174667	chr5:136174375-136174632
chr5	136177349	136177931	chr5:136177336-136182734
chr5	136177934	136178150	chr5:136177336-136182734
chr5	136178159	136178762	chr5:136177336-136182734
chr5	136179064	136179467	chr5:136177336-136182734
chr5	136179474	136182234	chr5:136177336-136182734
chr5	136182244	136182360	chr5:136177336-136182734
chr5	136182364	136182747	chr5:136177336-136182734
chr5	136187164	136187350	chr5:136187160-136187316
chr5	136188544	136188766	chr5:136188544-136188747
chr5	138465493	138465919	chr5:138465489-138466068
chr5	138466008	138466105	chr5:138465489-138466068
chr5	138466758	138466988	chr5:138466756-138469315
chr5	138466998	138468582	chr5:138466756-138469315
chr5	138468593	138468666	chr5:138466756-138469315
chr5	138468668	138468770	chr5:138466756-138469315
chr5	138468798	138469342	chr5:138466756-138469315
chr5	140107879	140108126	chr5:140107776-140108100
chr5	140111844	140112049	chr5:140111831-140112031
chr5	140112929	140113250	chr5:140112927-140113209
chr5	140114054	140114287	chr5:140114040-140125619
chr5	140114324	140115165	chr5:140114040-140125619
chr5	140115274	140115413	chr5:140114040-140125619
chr5	140115414	140116031	chr5:140114040-140125619
chr5	140116039	140116828	chr5:140114040-140125619
chr5	140116839	140118172	chr5:140114040-140125619
chr5	140118179	140118392	chr5:140114040-140125619
chr5	140118414	140119859	chr5:140114040-140125619
chr5	140119874	140120187	chr5:140114040-140125619
chr5	140120259	140120887	chr5:140114040-140125619
chr5	140120899	140122497	chr5:140114040-140125619
chr5	140122509	140123716	chr5:140114040-140125619
chr5	140123734	140124159	chr5:140114040-140125619
chr5	140124164	140124857	chr5:140114040-140125619
chr5	140124859	140125656	chr5:140114040-140125619
chr5	143277939	143278553	chr5:143277930-143282041
chr5	143278594	143279636	chr5:143277930-143282041

chr5	143279649	143281101	chr5:143277930-143282041
chr5	143281129	143281786	chr5:143277930-143282041
chr5	143281789	143282074	chr5:143277930-143282041
chr5	143282574	143282756	chr5:143282567-143282725
chr5	143295464	143295610	chr5:143295459-143295590
chr5	143298674	143298846	chr5:143298667-143298812
chr5	143300494	143300733	chr5:143300484-143300763
chr5	143310109	143310252	chr5:143310096-143310213
chr5	143314009	143314193	chr5:143313998-143314168
chr5	143356144	143356217	chr5:143356141-143356341
chr5	143356219	143356348	chr5:143356141-143356341
chr5	143389879	143390029	chr5:143389877-143390001
chr5	143399664	143400857	chr5:143399655-143400852
chr5	143401264	143401410	chr5:143401257-143401397
chr5	143402604	143402748	chr5:143402601-143402737
chr5	143403219	143403320	chr5:143403210-143403700
chr5	143403324	143403739	chr5:143403210-143403700
chr5	143403784	143403891	chr5:143403784-143403882
chr5	143403989	143404170	chr5:143403985-143404129
chr5	143404389	143404549	chr5:143404375-143404544
chr5	143404619	143404690	chr5:143404618-143404667
chr5	143405159	143405337	chr5:143405149-143405323
chr5	143408999	143409278	chr5:143408989-143409239
chr5	143433719	143433861	chr5:143433718-143433827
chr5	143434534	143434960	chr5:143434531-143435512
chr5	143434964	143435532	chr5:143434531-143435512
chr5	146339559	146340536	chr5:146339547-146340520
chr5	147322084	147322294	chr5:147322081-147322281
chr5	147343004	147343086	chr5:147343005-147343043
chr5	147348694	147348851	chr5:147348694-147348830
chr5	147383449	147383522	chr5:147383440-147383505
chr5	147383899	147384085	chr5:147383889-147384039
chr5	147384374	147384564	chr5:147384374-147384524
chr5	150166795	150167360	chr5:150166794-150167321
chr5	150180380	150180596	chr5:150180374-150180574
chr5	150182770	150182961	chr5:150182767-150182913
chr5	150183475	150184569	chr5:150183473-150184558
chr5	150223075	150223255	chr5:150223067-150223217
chr5	150228195	150228314	chr5:150228191-150228286
chr5	150270960	150271188	chr5:150270957-150271157
chr5	150894395	150895663	chr5:150894391-150898184
chr5	150895680	150895835	chr5:150894391-150898184
chr5	150895850	150895930	chr5:150894391-150898184
chr5	150895940	150896084	chr5:150894391-150898184
chr5	150896100	150896174	chr5:150894391-150898184
chr5	150896190	150896267	chr5:150894391-150898184
chr5	150896285	150897878	chr5:150894391-150898184
chr5	150897880	150898205	chr5:150894391-150898184
chr5	150898510	150898585	chr5:150898427-150898554
chr5	150902060	150902129	chr5:150902057-150902257
chr5	150902180	150902293	chr5:150902057-150902257
chr5	150903140	150903214	chr5:150903140-150903182
chr5	150903330	150903410	chr5:150903328-150903383
chr5	150903875	150904009	chr5:150903863-150903977
chr5	150904705	150905000	chr5:150904703-150904983
chr5	153618252	153618474	chr5:153618248-153618448
chr5	153802397	153802507	chr5:153802390-153802490
chr5	153811027	153811212	chr5:153811024-153811174
chr5	154474982	154475177	chr5:154474971-154475910
chr5	154475277	154475586	chr5:154474971-154475910
chr5	154475587	154475933	chr5:154474971-154475910
chr5	154476427	154476642	chr5:154476422-154476622
chr5	154477472	154477966	chr5:154477465-154478264

chr5	154477977	154478285	chr5:154477465-154478264
chr5	157625681	157625943	chr5:157625678-157626721
chr5	157625961	157626265	chr5:157625678-157626721
chr5	157626271	157626755	chr5:157625678-157626721
chr5	157638231	157638761	chr5:157638229-157638722
chr5	157639491	157639712	chr5:157639490-157639690
chr5	157646646	157646852	chr5:157646636-157646816
chr5	157648656	157648909	chr5:157648656-157648896
chr5	157651121	157652453	chr5:157651111-157652420
chr5	157667801	157667873	chr5:157667797-157667852
chr5	157671341	157671439	chr5:157671329-157671402
chr5	158695916	158695984	chr5:158695915-158699142
chr5	158696001	158696631	chr5:158695915-158699142
chr5	158696641	158696889	chr5:158695915-158699142
chr5	158696931	158697233	chr5:158695915-158699142
chr5	158697286	158698650	chr5:158695915-158699142
chr5	158698676	158698819	chr5:158695915-158699142
chr5	158698841	158699155	chr5:158695915-158699142
chr5	158707581	158707658	chr5:158707567-158707636
chr5	158707986	158708209	chr5:158707978-158708173
chr5	158712166	158712365	chr5:158712153-158712333
chr5	158712971	158713186	chr5:158712969-158713147
chr5	158714116	158714194	chr5:158714116-158714182
chr5	158716466	158716691	chr5:158716466-158716666
chr5	158731046	158731190	chr5:158731043-158731157
chr5	158777416	158777572	chr5:158777412-158777539
chr5	158796356	158796498	chr5:158796344-158796475
chr5	158823176	158823358	chr5:158823175-158823317
chr5	158840031	158840147	chr5:158840028-158840123
chr5	159018961	159019141	chr5:159018959-159019129
chr5	159073396	159073477	chr5:159073395-159073464
chr5	159084671	159084774	chr5:159084665-159084739
chr5	159095621	159095693	chr5:159095619-159095675
chr5	159096356	159096430	chr5:159096342-159096406
chr5	159096976	159097165	chr5:159096973-159097130
chr5	159097496	159097635	chr5:159097484-159097623
chr5	159099356	159099520	chr5:159099344-159099761
chr5	159099591	159099660	chr5:159099344-159099761
chr5	159099666	159099794	chr5:159099344-159099761
chr5	162069536	162069740	chr5:162069525-162069725
chr5	162142216	162142332	chr5:162142216-162142316
chr5	162149121	162149323	chr5:162149107-162149313
chr5	162151731	162151804	chr5:162151729-162151879
chr5	162151836	162151916	chr5:162151729-162151879
chr5	162153096	162153284	chr5:162153092-162153242
chr5	170105905	170106466	chr5:170105896-170106531
chr5	170106485	170106554	chr5:170105896-170106531
chr5	170108050	170109696	chr5:170108048-170109725
chr5	173056360	173056507	chr5:173056351-173056479
chr5	173057355	173057708	chr5:173057343-173057683
chr5	173080585	173080727	chr5:173080584-173080784
chr5	173080730	173080802	chr5:173080584-173080784
chr5	173086500	173086650	chr5:173086500-173086626
chr5	173090320	173091465	chr5:173090314-173092330
chr5	173091470	173091617	chr5:173090314-173092330
chr5	173091680	173091813	chr5:173090314-173092330
chr5	173092100	173092345	chr5:173090314-173092330
chr5	173103365	173103572	chr5:173103357-173103557
chr5	173108635	173108843	chr5:173108623-173108818
chr5	173110535	173110737	chr5:173110521-173110711
chr5	173112305	173112408	chr5:173112305-173112379
chr5	173123090	173123224	chr5:173123079-173123202
chr5	173133630	173134240	chr5:173133629-173139284

chr5	173134295	173134549	chr5:173133629-173139284
chr5	173134560	173134736	chr5:173133629-173139284
chr5	173134760	173135077	chr5:173133629-173139284
chr5	173135100	173135508	chr5:173133629-173139284
chr5	173135555	173135625	chr5:173133629-173139284
chr5	173135635	173136219	chr5:173133629-173139284
chr5	173136240	173136364	chr5:173133629-173139284
chr5	173136375	173136960	chr5:173133629-173139284
chr5	173136965	173137689	chr5:173133629-173139284
chr5	173137695	173138996	chr5:173133629-173139284
chr5	173139000	173139253	chr5:173133629-173139284
chr5	173232120	173232894	chr5:173232108-173233501
chr5	173232915	173233057	chr5:173232108-173233501
chr5	173233060	173233508	chr5:173232108-173233501
chr5	173234050	173234153	chr5:173234047-173234140
chr5	173234750	173235354	chr5:173234749-173235357
chr5	174724545	174725096	chr5:174724532-174725067
chr5	174727815	174728038	chr5:174727814-174728014
chr5	174729160	174729831	chr5:174729158-174730893
chr5	174729905	174730560	chr5:174729158-174730893
chr5	174730585	174730896	chr5:174729158-174730893
chr5	177307120	177307994	chr5:177307109-177307964
chr5	177310425	177310574	chr5:177310425-177310540
chr5	177310660	177310745	chr5:177310667-177310697
chr5	177311385	177311529	chr5:177311378-177311514
chr5	177311760	177312333	chr5:177311760-177312309
chr5	177992235	177993081	chr5:177992234-177993047
chr5	177994105	177994355	chr5:177994105-177994338
chr5	177994995	177995206	chr5:177994982-177995182
chr5	177995830	177996175	chr5:177995824-177996242
chr5	177996180	177996279	chr5:177995824-177996242
chr5	178204515	178205070	chr5:178204506-178205046
chr5	178205850	178206035	chr5:178205841-178206010
chr5	178206745	178206926	chr5:178206731-178206890
chr5	178207095	178207239	chr5:178207093-178207225
chr5	178209335	178209482	chr5:178209329-178209447
chr5	178210140	178210282	chr5:178210131-178210272
chr5	178210555	178210856	chr5:178210552-178210817
chr5	178694615	178694962	chr5:178694604-178694947
chr5	178695290	178695605	chr5:178695286-178695598
chr5	178696105	178696178	chr5:178696096-178696181
chr5	178697585	178697727	chr5:178697585-178697695
chr5	178706940	178707189	chr5:178706938-178707172
chr5	178711605	178712131	chr5:178711591-178713621
chr5	178712155	178712299	chr5:178711591-178713621
chr5	178712320	178712723	chr5:178711591-178713621
chr5	178712745	178713053	chr5:178711591-178713621
chr5	178713070	178713633	chr5:178711591-178713621
chr5	178715220	178715443	chr5:178715219-178715419
chr5	178725385	178725488	chr5:178725375-178725471
chr5	178726130	178726235	chr5:178726074-178726227
chr5	178727035	178727142	chr5:178726998-178727125
chr5	178729000	178729421	chr5:178728989-178729383
chr5	178730555	178730731	chr5:178730555-178730702
chr5	179060425	179060697	chr5:179060414-179060666
chr5	179062025	179062141	chr5:179062014-179062095
chr5	179062720	179062924	chr5:179062706-179062906
chr5	179076455	179076568	chr5:179076444-179076571
chr5	179077075	179077185	chr5:179077070-179077166
chr5	179078685	179079222	chr5:179078682-179082529
chr5	179079240	179079375	chr5:179078682-179082529
chr5	179079400	179079479	chr5:179078682-179082529
chr5	179079495	179079813	chr5:179078682-179082529

chr5	179079820	179080630	chr5:179078682-179082529
chr5	179080655	179081736	chr5:179078682-179082529
chr5	179081745	179081815	chr5:179078682-179082529
chr5	179081830	179082484	chr5:179078682-179082529
chr5	179083360	179083573	chr5:179083344-179083537
chr6	391745	391819	chr6:391738-391809
chr6	393100	393384	chr6:393097-393368
chr6	394830	395038	chr6:394820-395007
chr6	395860	396101	chr6:395846-396074
chr6	397110	397570	chr6:397107-397546
chr6	398840	398978	chr6:398827-398935
chr6	401435	401819	chr6:401423-401777
chr6	403735	403943	chr6:403722-403922
chr6	405020	405173	chr6:405017-405130
chr6	406770	406918	chr6:406765-406899
chr6	407460	407632	chr6:407454-411447
chr6	407790	408745	chr6:407454-411447
chr6	408770	411433	chr6:407454-411447
chr6	1312440	1314469	chr6:1312439-1314748
chr6	1314485	1314651	chr6:1312439-1314748
chr6	1314655	1314763	chr6:1312439-1314748
chr6	1389840	1390054	chr6:1389833-1391118
chr6	1390065	1390745	chr6:1389833-1391118
chr6	1390755	1390847	chr6:1389833-1391118
chr6	1390855	1391139	chr6:1389833-1391118
chr6	1393335	1393559	chr6:1393332-1393532
chr6	1394705	1395195	chr6:1394695-1395597
chr6	1395220	1395626	chr6:1394695-1395597
chr6	1609980	1610816	chr6:1609971-1613897
chr6	1610925	1611218	chr6:1609971-1613897
chr6	1611240	1611566	chr6:1609971-1613897
chr6	1611580	1611786	chr6:1609971-1613897
chr6	1611800	1611900	chr6:1609971-1613897
chr6	1611915	1612182	chr6:1609971-1613897
chr6	1612190	1612607	chr6:1609971-1613897
chr6	1612620	1612758	chr6:1609971-1613897
chr6	1612770	1613929	chr6:1609971-1613897
chr6	7107598	7107664	chr6:7107596-7107768
chr6	7107688	7107802	chr6:7107596-7107768
chr6	7107808	7108084	chr6:7107798-7108064
chr6	7140648	7140826	chr6:7140647-7140808
chr6	7165058	7165279	chr6:7165058-7165258
chr6	7176658	7176812	chr6:7176654-7176773
chr6	7181128	7181306	chr6:7181123-7181289
chr6	7181883	7182093	chr6:7181869-7182082
chr6	7189163	7189346	chr6:7189158-7189322
chr6	7210803	7210990	chr6:7210803-7210948
chr6	7211578	7211721	chr6:7211572-7211709
chr6	7226473	7226685	chr6:7226466-7226656
chr6	7228998	7231946	chr6:7228996-7231907
chr6	7240448	7240619	chr6:7240437-7240602
chr6	7246423	7247238	chr6:7246423-7247221
chr6	7248518	7249054	chr6:7248510-7251980
chr6	7249103	7249798	chr6:7248510-7251980
chr6	7249813	7250967	chr6:7248510-7251980
chr6	7251008	7251278	chr6:7248510-7251980
chr6	7251283	7251349	chr6:7248510-7251980
chr6	7251368	7251438	chr6:7248510-7251980
chr6	7251503	7251998	chr6:7248510-7251980
chr6	10393188	10393587	chr6:10393185-10393561
chr6	10395078	10395246	chr6:10395019-10395219
chr6	10396703	10397221	chr6:10396676-10398705
chr6	10397238	10397333	chr6:10396676-10398705

chr6	10397338	10397467	chr6:10396676-10398705
chr6	10397473	10397907	chr6:10396676-10398705
chr6	10397928	10398004	chr6:10396676-10398705
chr6	10398088	10398290	chr6:10396676-10398705
chr6	10398298	10398746	chr6:10396676-10398705
chr6	10400458	10400627	chr6:10400447-10400589
chr6	10400723	10400822	chr6:10400713-10400791
chr6	10402493	10402645	chr6:10402491-10402610
chr6	10404518	10404757	chr6:10404507-10404739
chr6	10406803	10406881	chr6:10406792-10406844
chr6	10409903	10410368	chr6:10409900-10410335
chr6	10410873	10411039	chr6:10410859-10411018
chr6	10411578	10411885	chr6:10411569-10412374
chr6	10411888	10412416	chr6:10411569-10412374
chr6	10412603	10412889	chr6:10412601-10412879
chr6	10414953	10415093	chr6:10414940-10415248
chr6	10415178	10415283	chr6:10414940-10415248
chr6	10415928	10416206	chr6:10415921-10416190
chr6	10416928	10417241	chr6:10416920-10417207
chr6	10418198	10418722	chr6:10418188-10418684
chr6	10419428	10419687	chr6:10419417-10419659
chr6	12008818	12008883	chr6:12008761-12008875
chr6	12012013	12012078	chr6:12011999-12012054
chr6	12012348	12012453	chr6:12012337-12012566
chr6	12012458	12012601	chr6:12012337-12012566
chr6	12012713	12012811	chr6:12012701-12012789
chr6	12015528	12015707	chr6:12015525-12015668
chr6	12020248	12020427	chr6:12020247-12020418
chr6	12037783	12037853	chr6:12037725-12039245
chr6	12037998	12038387	chr6:12037725-12039245
chr6	12038753	12039277	chr6:12037725-12039245
chr6	12048593	12048818	chr6:12048591-12048791
chr6	12089193	12089260	chr6:12089183-12089237
chr6	12119898	12125822	chr6:12119889-12125870
chr6	12125833	12125910	chr6:12119889-12125870
chr6	12129758	12129902	chr6:12129758-12129896
chr6	12130778	12130943	chr6:12130766-12130942
chr6	12135803	12135908	chr6:12135790-12135892
chr6	12161448	12161964	chr6:12161438-12161929
chr6	12163288	12164465	chr6:12163282-12164999
chr6	12164473	12164713	chr6:12163282-12164999
chr6	12164723	12165041	chr6:12163282-12164999
chr6	14129113	14129334	chr6:14129111-14129311
chr6	14133658	14133770	chr6:14133655-14133755
chr6	14135108	14135298	chr6:14135107-14135257
chr6	15246323	15246607	chr6:15246295-15246584
chr6	15248898	15248997	chr6:15248854-15248964
chr6	15374123	15374274	chr6:15374116-15374252
chr6	15410228	15410395	chr6:15410223-15410365
chr6	15452013	15452194	chr6:15452005-15452175
chr6	15468553	15468750	chr6:15468541-15468718
chr6	15487308	15487557	chr6:15487306-15487542
chr6	15489798	15490025	chr6:15489794-15489994
chr6	15496133	15497193	chr6:15496131-15497170
chr6	15500918	15501441	chr6:15500906-15501409
chr6	15504513	15504618	chr6:15504499-15504592
chr6	15505168	15505309	chr6:15505164-15505295
chr6	15507148	15507286	chr6:15507135-15507254
chr6	15507348	15507423	chr6:15507345-15507416
chr6	15508348	15508495	chr6:15508339-15508454
chr6	15511298	15511418	chr6:15511295-15511401
chr6	15512218	15512424	chr6:15512207-15512390
chr6	15512918	15513067	chr6:15512914-15513045

chr6	15513238	15513466	chr6:15513238-15513422
chr6	15517163	15517308	chr6:15517160-15517268
chr6	15520068	15520547	chr6:15520068-15522040
chr6	15520558	15521041	chr6:15520068-15522040
chr6	15521098	15521336	chr6:15520068-15522040
chr6	15521348	15521532	chr6:15520068-15522040
chr6	15521553	15522075	chr6:15520068-15522040
chr6	20401907	20402089	chr6:20401905-20402625
chr6	20402102	20402338	chr6:20401905-20402625
chr6	20402372	20402585	chr6:20401905-20402625
chr6	20403692	20403867	chr6:20403678-20403832
chr6	20458147	20458350	chr6:20458134-20458334
chr6	20479847	20479994	chr6:20479845-20479957
chr6	20481207	20481460	chr6:20481205-20481425
chr6	20482767	20482942	chr6:20482761-20482920
chr6	20486692	20486845	chr6:20486688-20486803
chr6	20488122	20488251	chr6:20488112-20488248
chr6	20490167	20492158	chr6:20490167-20493715
chr6	20492162	20493207	chr6:20490167-20493715
chr6	20493217	20493533	chr6:20490167-20493715
chr6	20493572	20493745	chr6:20490167-20493715
chr6	21595768	21595981	chr6:21595759-21595959
chr6	26156851	26157060	chr6:26156850-26157050
chr6	26158511	26158582	chr6:26158350-26158559
chr6	26165491	26165713	chr6:26165484-26165684
chr6	26170981	26171157	chr6:26170974-26171124
chr6	26634391	26635059	chr6:26634382-26638728
chr6	26635071	26636750	chr6:26634382-26638728
chr6	26636756	26638759	chr6:26634382-26638728
chr6	26641101	26641312	chr6:26641092-26641292
chr6	26643661	26643740	chr6:26643658-26643728
chr6	26658561	26658678	chr6:26658557-26658646
chr6	26658786	26658886	chr6:26658776-26658851
chr6	26659451	26659762	chr6:26659440-26659752
chr6	27866849	27867069	chr6:27866848-27867048
chr6	28080986	28081539	chr6:28080974-28081530
chr6	28084011	28084183	chr6:28083970-28084170
chr6	28085491	28085923	chr6:28085480-28085891
chr6	28086171	28086317	chr6:28086171-28086310
chr6	28088576	28089502	chr6:28088562-28089563
chr6	28089526	28089600	chr6:28088562-28089563
chr6	28224886	28225399	chr6:28224885-28225366
chr6	28226921	28227534	chr6:28226920-28227504
chr6	28227666	28228300	chr6:28227664-28229230
chr6	28228406	28229257	chr6:28227664-28229230
chr6	28230336	28230527	chr6:28230335-28230488
chr6	28232566	28232829	chr6:28232561-28233482
chr6	28232841	28233395	chr6:28232561-28233482
chr6	28233421	28233502	chr6:28232561-28233482
chr6	28267011	28267225	chr6:28267009-28267213
chr6	28267361	28267471	chr6:28267358-28267453
chr6	28271856	28272182	chr6:28271853-28272339
chr6	28272191	28272362	chr6:28271853-28272339
chr6	28272676	28272829	chr6:28272669-28272787
chr6	28276196	28278255	chr6:28276194-28278224
chr6	28324696	28325197	chr6:28324692-28326854
chr6	28325206	28326573	chr6:28324692-28326854
chr6	28326591	28326876	chr6:28324692-28326854
chr6	28327386	28327568	chr6:28327382-28327533
chr6	28329306	28329384	chr6:28329302-28329778
chr6	28329396	28329816	chr6:28329302-28329778
chr6	28332296	28332431	chr6:28332284-28332393
chr6	28335366	28335439	chr6:28335366-28335451

chr6	28335841	28336403	chr6:28335829-28336375
chr6	28336801	28336974	chr6:28336789-28336943
chr6	28341756	28341828	chr6:28341745-28341795
chr6	28349926	28350058	chr6:28349913-28350067
chr6	28350276	28350412	chr6:28350271-28350388
chr6	28353321	28353501	chr6:28353317-28353482
chr6	28353961	28354181	chr6:28353861-28354204
chr6	28355326	28355408	chr6:28355322-28355674
chr6	28355446	28355595	chr6:28355322-28355674
chr6	28356046	28356294	chr6:28356045-28356271
chr6	28357651	28357867	chr6:28357644-28357844
chr6	28359526	28359831	chr6:28359524-28359988
chr6	28359856	28360029	chr6:28359524-28359988
chr6	28361331	28361509	chr6:28361323-28361471
chr6	28363316	28363416	chr6:28363302-28363385
chr6	28363701	28363845	chr6:28363691-28363815
chr6	28365431	28366932	chr6:28365425-28369177
chr6	28367206	28367312	chr6:28365425-28369177
chr6	28367441	28367720	chr6:28365425-28369177
chr6	28368271	28369186	chr6:28365425-28369177
chr6	28378966	28379314	chr6:28378954-28382609
chr6	28379611	28379817	chr6:28378954-28382609
chr6	28379831	28380813	chr6:28378954-28382609
chr6	28380821	28381302	chr6:28378954-28382609
chr6	28381306	28381886	chr6:28378954-28382609
chr6	28381906	28382634	chr6:28378954-28382609
chr6	28383536	28383736	chr6:28383526-28383726
chr6	28390651	28390899	chr6:28390595-28391742
chr6	28390906	28391287	chr6:28390595-28391742
chr6	28391301	28391777	chr6:28390595-28391742
chr6	28392911	28393091	chr6:28392901-28393046
chr6	28398006	28398123	chr6:28398003-28398473
chr6	28398131	28398512	chr6:28398003-28398473
chr6	28399666	28399757	chr6:28399656-28399734
chr6	30061264	30061689	chr6:30061253-30061669
chr6	30061924	30062043	chr6:30061916-30062017
chr6	30062234	30062369	chr6:30062223-30062333
chr6	30064684	30064918	chr6:30064672-30064909
chr6	31164366	31164890	chr6:31164365-31164867
chr6	31165131	31165305	chr6:31165127-31165286
chr6	31165656	31165938	chr6:31165570-31166129
chr6	31166021	31166161	chr6:31165570-31166129
chr6	31166676	31166886	chr6:31166515-31166847
chr6	31170256	31170327	chr6:31170215-31170693
chr6	31170351	31170422	chr6:31170215-31170693
chr6	31170511	31170581	chr6:31170215-31170693
chr6	31170616	31170722	chr6:31170215-31170693
chr6	31172381	31172598	chr6:31172378-31172578
chr6	31180621	31180770	chr6:31180618-31180731
chr6	31616469	31616545	chr6:31616406-31616506
chr6	31616819	31616996	chr6:31616815-31616965
chr6	32107554	32107764	chr6:32107551-32107751
chr6	32115494	32115980	chr6:32115484-32115968
chr6	32116489	32116591	chr6:32116479-32116564
chr6	32116714	32116852	chr6:32116703-32116815
chr6	32117044	32117146	chr6:32117036-32117130
chr6	32117329	32117439	chr6:32117322-32117404
chr6	32117589	32117736	chr6:32117586-32117694
chr6	32117859	32118083	chr6:32117858-32118038
chr6	32118784	32118884	chr6:32118774-32118866
chr6	32118969	32119187	chr6:32118955-32119141
chr6	32119824	32119982	chr6:32119823-32119957
chr6	32120784	32120923	chr6:32120770-32120902

chr6	32120994	32121132	chr6:32120988-32121124
chr6	32121269	32121390	chr6:32121262-32121348
chr6	32126054	32126267	chr6:32126041-32126252
chr6	32127114	32127223	chr6:32127102-32127194
chr6	32127454	32127559	chr6:32127441-32127520
chr6	32127674	32127785	chr6:32127670-32127750
chr6	32128129	32128258	chr6:32128116-32128253
chr6	32184754	32185533	chr6:32184740-32186474
chr6	32185709	32186057	chr6:32184740-32186474
chr6	32186059	32186512	chr6:32184740-32186474
chr6	32186614	32186729	chr6:32186603-32186690
chr6	32186814	32186934	chr6:32186812-32186901
chr6	32187254	32187430	chr6:32187241-32187395
chr6	32187659	32187793	chr6:32187646-32187782
chr6	32187974	32188198	chr6:32187965-32188156
chr6	32188264	32188546	chr6:32188256-32188504
chr6	32188729	32188828	chr6:32188722-32188796
chr6	32189694	32190185	chr6:32189694-32190186
chr6	33193599	33194095	chr6:33193587-33194829
chr6	33194109	33194843	chr6:33193587-33194829
chr6	33194949	33195089	chr6:33194944-33195050
chr6	33195364	33195487	chr6:33195362-33195466
chr6	33195574	33195688	chr6:33195569-33195702
chr6	33195919	33196063	chr6:33195906-33196036
chr6	33196434	33196508	chr6:33196433-33196606
chr6	33196509	33196638	chr6:33196433-33196606
chr6	33197769	33197973	chr6:33197761-33197941
chr6	33198309	33198495	chr6:33198307-33198464
chr6	33199179	33199286	chr6:33199168-33199416
chr6	33199294	33199432	chr6:33199168-33199416
chr6	33200249	33200319	chr6:33200241-33200294
chr6	34236884	34237018	chr6:34236872-34237317
chr6	34237024	34237339	chr6:34236872-34237317
chr6	34238799	34238981	chr6:34238790-34238957
chr6	34240744	34240948	chr6:34240736-34240915
chr6	34242719	34242818	chr6:34242711-34242795
chr6	34243469	34243536	chr6:34243467-34243518
chr6	34244859	34244940	chr6:34244830-34246229
chr6	34244969	34245042	chr6:34244830-34246229
chr6	34245094	34245167	chr6:34244830-34246229
chr6	34245309	34245384	chr6:34244830-34246229
chr6	34245434	34245615	chr6:34244830-34246229
chr6	34245624	34245741	chr6:34244830-34246229
chr6	34245744	34245842	chr6:34244830-34246229
chr6	34245849	34245927	chr6:34244830-34246229
chr6	34246064	34246136	chr6:34244830-34246229
chr6	34246184	34246255	chr6:34244830-34246229
chr6	34516151	34516369	chr6:34516142-34516342
chr6	34531701	34531802	chr6:34531687-34531787
chr6	34532426	34532595	chr6:34532420-34532570
chr6	34537801	34538472	chr6:34537801-34538452
chr6	34539251	34539432	chr6:34539249-34539396
chr6	34539516	34539590	chr6:34539514-34539562
chr6	34540986	34541211	chr6:34540983-34541181
chr6	34544031	34544527	chr6:34544019-34544484
chr6	34549926	34550134	chr6:34549914-34550114
chr6	34555931	34556367	chr6:34555928-34556333
chr6	35258981	35259416	chr6:35258981-35259386
chr6	35259481	35259870	chr6:35259470-35259841
chr6	35262871	35263097	chr6:35262871-35263071
chr6	35270421	35270561	chr6:35270411-35270523
chr6	35281066	35281241	chr6:35281055-35281224
chr6	35286136	35286241	chr6:35286127-35286208

chr6	35286331	35286435	chr6:35286321-35286399
chr6	35287656	35287883	chr6:35287645-35287845
chr6	35290266	35290416	chr6:35290252-35290382
chr6	35290641	35291428	chr6:35290640-35291403
chr6	35291566	35291781	chr6:35291557-35291737
chr6	35292551	35292830	chr6:35292539-35292787
chr6	35292881	35293065	chr6:35292880-35293044
chr6	35293751	35295890	chr6:35293750-35295985
chr6	35295891	35295994	chr6:35293750-35295985
chr6	35342561	35342716	chr6:35342557-35342681
chr6	35347071	35347189	chr6:35347066-35347150
chr6	35362921	35363139	chr6:35362916-35363116
chr6	35397501	35397623	chr6:35397497-35397593
chr6	35410986	35411246	chr6:35410986-35411217
chr6	35420126	35420305	chr6:35420126-35420281
chr6	35421821	35421972	chr6:35421819-35421958
chr6	35423951	35424165	chr6:35423945-35424148
chr6	35424331	35424984	chr6:35424328-35425393
chr6	35425276	35425422	chr6:35424328-35425393
chr6	35425831	35428210	chr6:35425831-35428191
chr6	35497876	35498140	chr6:35497873-35498460
chr6	35498156	35498472	chr6:35497873-35498460
chr6	35498996	35499202	chr6:35498992-35499192
chr6	35499981	35500167	chr6:35499980-35500152
chr6	35503571	35503671	chr6:35503558-35503657
chr6	35503746	35503875	chr6:35503736-35503848
chr6	35505741	35505882	chr6:35505740-35505853
chr6	35506006	35506176	chr6:35506002-35506173
chr6	35509276	35509347	chr6:35509208-35509312
chr6	35509636	35509781	chr6:35509633-35509750
chr6	35509826	35509939	chr6:35509826-35509928
chr6	35510871	35510946	chr6:35510860-35511025
chr6	35511656	35511838	chr6:35511647-35511806
chr6	35512186	35512295	chr6:35512179-35512270
chr6	35512651	35512725	chr6:35512638-35512690
chr6	35512811	35512952	chr6:35512811-35512938
chr6	36354207	36354343	chr6:36354090-36354687
chr6	36354467	36354604	chr6:36354090-36354687
chr6	36354607	36354682	chr6:36354090-36354687
chr6	36361077	36361292	chr6:36361073-36361273
chr6	36366197	36366270	chr6:36366193-36366762
chr6	36366272	36366794	chr6:36366193-36366762
chr6	36366882	36366987	chr6:36366874-36366975
chr6	36368942	36369086	chr6:36368928-36369071
chr6	36371342	36371597	chr6:36371329-36371560
chr6	36373462	36373599	chr6:36373452-36373578
chr6	36375837	36376064	chr6:36375829-36376035
chr6	36385537	36385683	chr6:36385533-36385669
chr6	36387537	36387816	chr6:36387535-36387800
chr6	37819507	37820032	chr6:37819498-37820016
chr6	37929955	37930027	chr6:37929958-37929999
chr6	38033950	38034151	chr6:38033924-38034124
chr6	38061600	38061815	chr6:38061592-38061775
chr6	38082395	38082480	chr6:38082391-38082457
chr6	38116585	38116770	chr6:38116571-38116739
chr6	38142270	38142375	chr6:38142258-38142331
chr6	38143000	38143222	chr6:38142996-38143204
chr6	38152240	38152624	chr6:38152234-38154624
chr6	38152625	38154575	chr6:38152234-38154624
chr6	41072950	41073088	chr6:41072944-41073084
chr6	41079030	41079187	chr6:41079028-41079164
chr6	41080820	41080937	chr6:41080810-41080897
chr6	41082115	41082332	chr6:41082112-41082312

chr6	41084045	41084232	chr6:41084045-41084192
chr6	41089585	41089745	chr6:41089578-41089710
chr6	41090215	41090330	chr6:41090203-41090309
chr6	41091535	41091714	chr6:41091527-41091694
chr6	41092925	41093095	chr6:41092911-41093085
chr6	41094395	41094512	chr6:41094395-41094497
chr6	41097360	41097580	chr6:41097356-41097562
chr6	41097945	41098376	chr6:41097937-41099976
chr6	41098395	41099708	chr6:41097937-41099976
chr6	41099730	41099936	chr6:41097937-41099976
chr6	41546425	41546888	chr6:41546425-41546867
chr6	41551630	41551834	chr6:41551618-41551818
chr6	41565745	41566002	chr6:41565744-41565964
chr6	41570185	41570430	chr6:41570173-41570394
chr6	41577990	41578112	chr6:41577985-41578081
chr6	41584770	41584926	chr6:41584768-41584891
chr6	41585440	41585547	chr6:41585430-41585517
chr6	41587010	41587184	chr6:41587008-41587156
chr6	41587305	41587552	chr6:41587298-41587512
chr6	41587795	41587907	chr6:41587792-41587897
chr6	41588645	41588761	chr6:41588643-41588731
chr6	41589770	41589887	chr6:41589770-41589854
chr6	41589965	41590184	chr6:41589962-41590170
chr6	41590280	41590375	chr6:41590270-41590347
chr6	41591230	41591333	chr6:41591220-41591322
chr6	41594870	41595023	chr6:41594869-41594991
chr6	41597185	41597269	chr6:41597175-41597242
chr6	41597780	41597965	chr6:41597780-41597950
chr6	41598790	41601101	chr6:41598788-41602384
chr6	41601130	41601478	chr6:41598788-41602384
chr6	41601760	41602418	chr6:41598788-41602384
chr6	41683980	41685122	chr6:41683977-41685078
chr6	41686090	41686279	chr6:41686089-41686237
chr6	41686790	41687186	chr6:41686528-41687169
chr6	41687755	41687834	chr6:41687752-41687809
chr6	41687920	41688066	chr6:41687907-41688028
chr6	41689740	41689843	chr6:41689730-41689811
chr6	41690665	41690948	chr6:41690662-41690917
chr6	41691000	41691070	chr6:41691000-41691476
chr6	41691105	41691483	chr6:41691000-41691476
chr6	41705660	41705988	chr6:41705646-41705940
chr6	41713310	41713532	chr6:41713307-41713507
chr6	41723460	41723747	chr6:41723460-41723726
chr6	41732660	41733890	chr6:41732648-41733853
chr6	41734095	41734227	chr6:41734084-41734194
chr6	41734325	41734423	chr6:41734312-41734401
chr6	41734910	41735081	chr6:41734899-41735060
chr6	41735350	41735634	chr6:41735349-41735608
chr6	41736155	41736285	chr6:41736145-41736259
chr6	43171303	43172181	chr6:43171298-43172169
chr6	43172958	43173173	chr6:43172950-43173150
chr6	43173858	43174136	chr6:43173846-43174113
chr6	43175708	43176007	chr6:43175705-43175967
chr6	43176553	43176698	chr6:43176547-43176667
chr6	43178303	43178513	chr6:43178293-43178485
chr6	43178818	43178921	chr6:43178805-43178882
chr6	43179103	43179927	chr6:43179094-43181507
chr6	43179943	43181309	chr6:43179094-43181507
chr6	43181323	43181527	chr6:43179094-43181507
chr6	44387533	44387880	chr6:44387524-44387868
chr6	44390278	44390393	chr6:44390267-44390371
chr6	44392668	44392852	chr6:44392666-44392828
chr6	44393483	44393595	chr6:44393445-44393573

chr6	44396343	44396458	chr6:44396340-44396440
chr6	44403808	44404038	chr6:44403808-44404027
chr6	44406348	44406479	chr6:44406322-44406467
chr6	44408453	44408662	chr6:44408443-44408632
chr6	44419458	44419624	chr6:44419448-44419597
chr6	44422648	44422828	chr6:44422646-44422809
chr6	44424418	44424616	chr6:44424418-44424583
chr6	44426108	44426213	chr6:44426102-44426183
chr6	44426488	44426740	chr6:44426481-44426724
chr6	44429718	44429923	chr6:44429712-44429910
chr6	44431508	44431677	chr6:44431449-44431649
chr6	44445663	44445874	chr6:44445654-44445867
chr6	44446613	44447024	chr6:44446606-44447890
chr6	44447033	44447643	chr6:44446606-44447890
chr6	44447653	44447931	chr6:44446606-44447890
chr6	44448173	44449388	chr6:44448163-44450426
chr6	44449508	44449848	chr6:44448163-44450426
chr6	44450153	44450395	chr6:44448163-44450426
chr6	45328168	45328524	chr6:45328156-45328784
chr6	45328588	45328799	chr6:45328156-45328784
chr6	45336138	45336361	chr6:45336134-45336334
chr6	45361573	45361734	chr6:45361572-45361688
chr6	45371898	45372077	chr6:45371888-45372055
chr6	45422048	45422681	chr6:45422035-45422957
chr6	45422778	45422982	chr6:45422035-45422957
chr6	45431863	45432052	chr6:45431862-45432019
chr6	45437958	45438059	chr6:45437946-45438051
chr6	45491953	45492133	chr6:45491940-45492114
chr6	45512248	45512438	chr6:45512245-45512407
chr6	45513463	45513669	chr6:45513449-45513653
chr6	45545228	45545305	chr6:45545216-45545282
chr6	45546833	45547367	chr6:45546826-45551082
chr6	45547388	45547585	chr6:45546826-45551082
chr6	45547593	45548116	chr6:45546826-45551082
chr6	45548133	45549974	chr6:45546826-45551082
chr6	45550013	45551080	chr6:45546826-45551082
chr6	45663118	45664177	chr6:45663114-45664349
chr6	45664203	45664376	chr6:45663114-45664349
chr6	50713838	50713938	chr6:50713827-50714094
chr6	50713968	50714106	chr6:50713827-50714094
chr6	50715118	50715651	chr6:50715115-50715613
chr6	50718048	50718121	chr6:50718041-50718186
chr6	50718133	50718204	chr6:50718041-50718186
chr6	50719103	50719176	chr6:50719089-50719150
chr6	50728868	50729051	chr6:50728855-50729021
chr6	50729198	50729334	chr6:50729193-50729312
chr6	50745113	50745291	chr6:50745106-50745248
chr6	50749213	50749422	chr6:50749213-50749413
chr6	50751213	50751356	chr6:50751210-50751324
chr6	50772658	50772995	chr6:50772644-50772988
chr6	50818728	50819014	chr6:50818722-50818972
chr6	50821788	50821999	chr6:50821784-50822160
chr6	50822003	50822193	chr6:50821784-50822160
chr6	50823413	50823874	chr6:50823406-50823865
chr6	50825323	50825525	chr6:50825316-50825516
chr6	50828618	50828695	chr6:50828618-50828679
chr6	50836073	50836285	chr6:50836060-50836280
chr6	50837983	50838127	chr6:50837974-50838093
chr6	50840168	50840304	chr6:50840155-50840297
chr6	50843093	50843403	chr6:50843091-50847613
chr6	50843428	50843529	chr6:50843091-50847613
chr6	50843543	50844276	chr6:50843091-50847613
chr6	50844308	50847191	chr6:50843091-50847613

chr6	50847193	50847640	chr6:50843091-50847613
chr6	53126973	53127735	chr6:53126963-53128946
chr6	53128128	53128954	chr6:53126963-53128946
chr6	53130803	53130954	chr6:53130802-53130931
chr6	53132008	53132154	chr6:53132006-53132119
chr6	53134078	53134159	chr6:53134071-53134324
chr6	53134163	53134338	chr6:53134071-53134324
chr6	53143483	53143687	chr6:53143469-53143669
chr6	53145568	53145803	chr6:53145557-53145768
chr6	53148753	53148871	chr6:53148753-53148829
chr6	83512607	83512710	chr6:83512594-83512694
chr6	83517922	83518141	chr6:83517921-83518121
chr6	83523432	83523610	chr6:83523421-83523571
chr6	84687383	84687695	chr6:84687372-84687678
chr6	84688588	84688696	chr6:84688580-84688667
chr6	84732613	84732822	chr6:84732601-84737409
chr6	84732833	84734912	chr6:84732601-84737409
chr6	84734918	84736626	chr6:84732601-84737409
chr6	84736638	84737435	chr6:84732601-84737409
chr6	84738508	84738608	chr6:84738496-84738591
chr6	84741068	84741819	chr6:84741066-84741802
chr6	84742023	84742171	chr6:84742023-84742548
chr6	84742183	84742565	chr6:84742023-84742548
chr6	84744268	84744342	chr6:84744260-84744325
chr6	84747923	84748105	chr6:84747919-84748087
chr6	84752023	84752192	chr6:84752012-84752212
chr6	84756703	84756886	chr6:84756697-84756869
chr6	84760263	84760382	chr6:84760254-84760356
chr6	84762543	84762763	chr6:84762543-84762748
chr6	84762918	84763389	chr6:84762917-84763355
chr6	84763418	84763483	chr6:84763423-84763453
chr6	84763903	84764540	chr6:84763889-84764519
chr6	87155670	87155778	chr6:87155659-87155759
chr6	87156840	87157045	chr6:87156833-87157033
chr6	87245545	87245648	chr6:87245544-87245644
chr6	87254660	87254835	chr6:87254649-87254799
chr6	88143855	88144079	chr6:88143855-88144055
chr6	88144855	88145144	chr6:88144854-88145113
chr6	88145175	88145353	chr6:88145171-88145337
chr6	88153120	88153295	chr6:88153061-88153261
chr6	88162965	88163046	chr6:88162956-88163024
chr6	88164270	88164361	chr6:88164256-88164343
chr6	88165815	88165917	chr6:88165802-88165902
chr6	89926540	89928110	chr6:89926528-89932890
chr6	89928140	89928447	chr6:89926528-89932890
chr6	89928465	89930098	chr6:89926528-89932890
chr6	89930175	89930234	chr6:89926528-89932890
chr6	89930250	89931588	chr6:89926528-89932890
chr6	89931640	89931922	chr6:89926528-89932890
chr6	89931975	89932914	chr6:89926528-89932890
chr6	89938145	89938362	chr6:89938143-89938350
chr6	89950280	89951892	chr6:89950269-89951862
chr6	90008090	90008272	chr6:90008089-90008258
chr6	90008605	90008870	chr6:90008601-90008856
chr6	90026355	90026573	chr6:90026341-90026541
chr6	90088960	90089134	chr6:90088960-90089109
chr6	90104375	90104477	chr6:90104364-90104435
chr6	90206580	90206723	chr6:90206568-90206681
chr6	90217985	90218264	chr6:90217978-90218245
chr6	90252515	90252636	chr6:90252512-90252590
chr6	90271860	90271967	chr6:90271848-90271941
chr6	90296480	90296774	chr6:90296479-90296908
chr6	90296810	90296946	chr6:90296479-90296908

chr6	98834603	98834680	chr6:98834591-98839470
chr6	98834683	98835096	chr6:98834591-98839470
chr6	98835098	98835241	chr6:98834591-98839470
chr6	98835308	98835792	chr6:98834591-98839470
chr6	98835823	98836309	chr6:98834591-98839470
chr6	98836338	98836478	chr6:98834591-98839470
chr6	98836578	98837176	chr6:98834591-98839470
chr6	98837178	98837678	chr6:98834591-98839470
chr6	98837683	98837964	chr6:98834591-98839470
chr6	98837973	98838491	chr6:98834591-98839470
chr6	98838503	98838826	chr6:98834591-98839470
chr6	98838833	98839000	chr6:98834591-98839470
chr6	98839003	98839484	chr6:98834591-98839470
chr6	99606743	99607232	chr6:99606729-99607206
chr6	99608753	99608892	chr6:99608740-99608872
chr6	99609188	99609339	chr6:99609186-99609307
chr6	99610938	99611154	chr6:99610916-99611116
chr6	99613043	99613702	chr6:99613032-99615578
chr6	99613763	99615612	chr6:99613032-99615578
chr6	100385018	100385398	chr6:100385014-100391091
chr6	100385403	100385639	chr6:100385014-100391091
chr6	100385698	100385781	chr6:100385014-100391091
chr6	100385783	100386587	chr6:100385014-100391091
chr6	100386593	100391114	chr6:100385014-100391091
chr6	100393488	100393918	chr6:100393486-100393889
chr6	100420803	100420989	chr6:100420789-100420958
chr6	100447273	100447449	chr6:100447267-100447415
chr6	100448148	100448265	chr6:100448145-100448252
chr6	100448488	100448710	chr6:100448478-100448678
chr6	100449373	100449488	chr6:100449362-100449448
chr6	100449598	100449738	chr6:100449590-100449699
chr6	100450268	100450382	chr6:100450266-100450356
chr6	100453768	100453885	chr6:100453761-100453844
chr6	100456033	100456230	chr6:100456020-100456220
chr6	100463303	100463972	chr6:100463293-100463935
chr6	100464623	100464959	chr6:100464613-100464929
chr6	106086321	106086602	chr6:106086319-106086595
chr6	106087581	106087941	chr6:106087579-106087907
chr6	106088206	106088485	chr6:106088200-106088449
chr6	106095626	106095752	chr6:106095614-106095734
chr6	106098611	106098748	chr6:106098605-106098721
chr6	106098951	106099123	chr6:106098939-106099094
chr6	106099301	106099562	chr6:106099299-106099552
chr6	106101696	106101897	chr6:106101690-106101890
chr6	106104826	106105954	chr6:106104824-106105933
chr6	106106381	106106525	chr6:106106370-106106499
chr6	106106916	106107684	chr6:106106910-106109939
chr6	106107716	106108517	chr6:106106910-106109939
chr6	106108541	106108617	chr6:106106910-106109939
chr6	106108626	106109006	chr6:106106910-106109939
chr6	106109026	106109096	chr6:106106910-106109939
chr6	106109111	106109960	chr6:106106910-106109939
chr6	107704108	107704220	chr6:107704103-107705325
chr6	107704228	107704642	chr6:107704103-107705325
chr6	107704658	107705366	chr6:107704103-107705325
chr6	107707868	107708055	chr6:107707865-107708011
chr6	107718953	107719061	chr6:107718953-107719045
chr6	107719758	107721015	chr6:107719729-107720993
chr6	107731983	107732438	chr6:107731973-107732395
chr6	107740068	107740176	chr6:107740064-107740156
chr6	107743953	107744093	chr6:107743941-107744054
chr6	107744953	107745168	chr6:107744948-107745143
chr6	107746693	107746913	chr6:107746688-107746889

chr6	107749683	107749839	chr6:107749683-107749813
chr6	107755578	107755676	chr6:107755570-107755664
chr6	107772183	107772401	chr6:107772171-107772386
chr6	107806078	107806297	chr6:107806068-107806268
chr6	107824133	107824364	chr6:107824125-107824317
chr6	108166058	108166664	chr6:108166057-108166790
chr6	108166673	108166807	chr6:108166057-108166790
chr6	108168023	108168171	chr6:108168022-108168162
chr6	108168763	108168956	chr6:108168762-108168921
chr6	108171463	108171639	chr6:108171457-108171603
chr6	108173128	108173337	chr6:108173119-108173319
chr6	108174843	108174948	chr6:108174835-108174923
chr6	108176113	108176364	chr6:108176103-108176325
chr6	108176503	108176766	chr6:108176502-108176738
chr6	108178103	108178282	chr6:108178094-108178241
chr6	108180328	108180436	chr6:108180322-108180419
chr6	108180818	108180987	chr6:108180806-108180956
chr6	108181558	108181674	chr6:108181545-108181651
chr6	108187308	108187906	chr6:108187300-108188809
chr6	108187913	108188053	chr6:108187300-108188809
chr6	108188098	108188515	chr6:108187300-108188809
chr6	108188543	108188812	chr6:108187300-108188809
chr6	108559848	108560048	chr6:108559834-108560015
chr6	108560878	108561081	chr6:108560865-108561829
chr6	108561098	108561867	chr6:108560865-108561829
chr6	108609238	108609449	chr6:108609231-108609431
chr6	108656348	108656566	chr6:108656345-108656538
chr6	108663468	108664915	chr6:108663454-108664889
chr6	108679838	108680389	chr6:108679826-108684774
chr6	108680393	108680747	chr6:108679826-108684774
chr6	108680768	108682381	chr6:108679826-108684774
chr6	108682403	108683480	chr6:108679826-108684774
chr6	108683693	108683793	chr6:108679826-108684774
chr6	108683823	108684813	chr6:108679826-108684774
chr6	116877264	116877518	chr6:116877259-116877498
chr6	116877809	116877993	chr6:116877795-116877952
chr6	116880549	116880698	chr6:116880543-116880667
chr6	116882379	116882449	chr6:116882366-116882428
chr6	116884794	116885078	chr6:116884788-116885045
chr6	116888534	116888763	chr6:116888530-116888730
chr6	116893994	116894096	chr6:116893986-116894064
chr6	116910939	116911080	chr6:116910934-116911042
chr6	116916009	116916117	chr6:116916007-116916085
chr6	116916214	116916349	chr6:116916200-116916314
chr6	116918039	116918147	chr6:116918036-116918110
chr6	116919144	116919332	chr6:116919136-116919296
chr6	116920319	116920491	chr6:116920309-116920454
chr6	116922054	116922185	chr6:116922041-116922151
chr6	116923119	116923257	chr6:116923106-116923224
chr6	116924674	116924810	chr6:116924668-116924791
chr6	116925459	116925684	chr6:116925452-116925659
chr6	116927029	116927560	chr6:116927026-116927539
chr6	116928759	116929008	chr6:116928758-116928971
chr6	116931334	116932139	chr6:116931330-116932163
chr6	122399549	122399866	chr6:122399545-122399830
chr6	122404179	122404390	chr6:122404169-122404369
chr6	122412384	122412527	chr6:122412372-122412481
chr6	122412639	122412784	chr6:122412636-122412764
chr6	122413534	122413661	chr6:122413524-122413649
chr6	122416229	122416333	chr6:122416220-122416296
chr6	122419169	122419234	chr6:122419167-122419229
chr6	122420134	122420242	chr6:122420134-122420222
chr6	122422149	122422329	chr6:122422149-122422298

chr6	122422719	122422976	chr6:122422717-122422957
chr6	122423584	122423706	chr6:122423580-122423686
chr6	122427914	122427995	chr6:122427902-122427956
chr6	122431429	122431539	chr6:122431429-122431514
chr6	122431924	122432530	chr6:122431924-122433119
chr6	122432539	122433135	chr6:122431924-122433119
chr6	125747665	125747737	chr6:125747663-125747726
chr6	125749585	125749865	chr6:125749579-125749859
chr6	125751800	125751904	chr6:125751800-125751879
chr6	125752010	125752117	chr6:125752006-125752090
chr6	125754465	125754572	chr6:125754464-125754546
chr6	125755540	125755741	chr6:125755530-125755730
chr6	125759120	125761298	chr6:125759116-125761269
chr6	133889149	133889319	chr6:133889137-133889847
chr6	133889379	133889861	chr6:133889137-133889847
chr6	133890884	133891099	chr6:133890881-133891081
chr6	133891719	133892070	chr6:133891712-133895553
chr6	133892084	133892186	chr6:133891712-133895553
chr6	133892199	133892267	chr6:133891712-133895553
chr6	133892279	133894240	chr6:133891712-133895553
chr6	133894249	133895025	chr6:133891712-133895553
chr6	133895029	133895576	chr6:133891712-133895553
chr6	133952174	133952763	chr6:133952169-133952730
chr6	133953189	133953437	chr6:133953184-133953425
chr6	133954644	133954864	chr6:133954637-133954837
chr6	133980084	133980297	chr6:133980081-133980260
chr6	133982579	133982673	chr6:133982567-133982650
chr6	133982819	133982888	chr6:133982816-133982880
chr6	133984384	133984491	chr6:133984375-133984479
chr6	133984579	133984764	chr6:133984576-133984723
chr6	133985884	133985994	chr6:133985883-133985968
chr6	133986964	133987465	chr6:133986960-133988546
chr6	133987474	133987609	chr6:133986960-133988546
chr6	133987664	133988584	chr6:133986960-133988546
chr6	135181345	135181553	chr6:135181314-135181536
chr6	135185885	135186053	chr6:135185876-135186020
chr6	135187840	135187938	chr6:135187833-135187905
chr6	135189800	135189896	chr6:135189790-135189883
chr6	135190130	135190387	chr6:135190126-135190347
chr6	135192330	135192576	chr6:135192323-135192558
chr6	135193845	135193957	chr6:135193837-135193918
chr6	135194365	135194708	chr6:135194355-135194686
chr6	135194970	135195102	chr6:135194959-135195081
chr6	135195760	135196037	chr6:135195747-135196002
chr6	135196725	135196952	chr6:135196725-135197323
chr6	135196985	135197336	chr6:135196725-135197323
chr6	135198915	135199085	chr6:135198907-135199050
chr6	135199525	135199602	chr6:135199525-135199581
chr6	135200095	135200225	chr6:135200084-135200199
chr6	135200290	135200585	chr6:135200289-135200674
chr6	135200595	135200696	chr6:135200289-135200674
chr6	135201645	135201789	chr6:135201638-135201749
chr6	135203230	135203356	chr6:135203216-135203950
chr6	135203395	135203961	chr6:135203216-135203950
chr6	135211405	135211618	chr6:135211405-135211605
chr6	135217865	135218380	chr6:135217860-135219173
chr6	135218395	135218557	chr6:135217860-135219173
chr6	135218580	135218752	chr6:135217860-135219173
chr6	135218765	135219175	chr6:135217860-135219173
chr6	136260965	136261109	chr6:136260965-136261115
chr6	136261265	136261382	chr6:136261264-136261364
chr6	136265355	136265574	chr6:136265351-136265551
chr6	136269260	136269329	chr6:136269263-136269303

chr6	136269450	136269549	chr6:136269436-136269536
chr6	137492215	137492801	chr6:137492201-137494250
chr6	137492835	137494064	chr6:137492201-137494250
chr6	137494085	137494244	chr6:137492201-137494250
chr6	142751480	142752749	chr6:142751466-142753931
chr6	142752755	142753049	chr6:142751466-142753931
chr6	142753055	142753649	chr6:142751466-142753931
chr6	142753665	142753950	chr6:142751466-142753931
chr6	142759780	142760522	chr6:142759771-142760667
chr6	142760545	142760696	chr6:142759771-142760667
chr6	142761520	142761591	chr6:142761463-142761565
chr6	142764800	142764979	chr6:142764798-142764974
chr6	142768390	142768574	chr6:142768381-142768536
chr6	142769555	142775135	chr6:142769551-142775125
chr6	142776145	142776220	chr6:142776146-142776191
chr6	142783530	142783633	chr6:142783520-142783615
chr6	142836935	142837074	chr6:142836934-142837047
chr6	142941850	142942057	chr6:142941839-142942039
chr6	142944800	142944940	chr6:142944800-142945031
chr6	142944945	142945050	chr6:142944800-142945031
chr6	142945110	142945214	chr6:142945098-142945201
chr6	142946000	142946378	chr6:142945943-142946365
chr6	142956450	142956727	chr6:142956443-142956698
chr6	143940305	143940583	chr6:143940299-143942663
chr6	143940615	143940723	chr6:143940299-143942663
chr6	143940745	143942701	chr6:143940299-143942663
chr6	143947990	143948481	chr6:143947984-143948460
chr6	143960480	143960722	chr6:143960468-143961412
chr6	143960735	143961430	chr6:143960468-143961412
chr6	143964795	143964870	chr6:143964786-143968978
chr6	143964885	143966364	chr6:143964786-143968978
chr6	143966405	143966827	chr6:143964786-143968978
chr6	143966850	143967954	chr6:143964786-143968978
chr6	143968015	143968468	chr6:143964786-143968978
chr6	143968470	143968998	chr6:143964786-143968978
chr6	143973720	143973936	chr6:143973718-143973918
chr6	144008095	144008444	chr6:144008089-144008416
chr6	144008740	144008858	chr6:144008734-144008823
chr6	144064455	144064624	chr6:144064450-144064599
chr6	151364130	151364365	chr6:151364116-151366892
chr6	151364400	151366914	chr6:151364116-151366892
chr6	151373475	151373641	chr6:151373464-151373649
chr6	151380370	151380597	chr6:151380368-151380568
chr6	151391420	151391496	chr6:151391419-151391548
chr6	151656694	151656768	chr6:151656690-151656763
chr6	151690509	151690688	chr6:151690495-151690664
chr6	151701879	151702023	chr6:151701874-151702005
chr6	151804434	151805245	chr6:151804430-151805820
chr6	151805264	151805837	chr6:151804430-151805820
chr6	151807209	151807524	chr6:151807195-151807509
chr6	151807554	151808394	chr6:151807550-151808364
chr6	151809109	151809295	chr6:151809106-151809271
chr6	151811009	151811303	chr6:151811008-151811278
chr6	151842599	151842814	chr6:151842596-151842787
chr6	151880654	151880800	chr6:151880654-151880771
chr6	151944174	151944543	chr6:151944171-151944508
chr6	151983454	151983587	chr6:151983447-151983560
chr6	151983974	151984191	chr6:151983974-151984165
chr6	152007534	152007754	chr6:152007530-152007730
chr6	152011664	152011817	chr6:152011655-152011794
chr6	152060999	152061151	chr6:152060990-152061124
chr6	152094394	152094611	chr6:152094384-152094568
chr6	152098734	152099757	chr6:152098731-152103274

chr6	152099769	152100778	chr6:152098731-152103274
chr6	152100779	152100908	chr6:152098731-152103274
chr6	152100929	152101026	chr6:152098731-152103274
chr6	152101044	152101353	chr6:152098731-152103274
chr6	152101384	152103280	chr6:152098731-152103274
chr6	152125269	152126610	chr6:152125265-152129619
chr6	152126619	152129552	chr6:152125265-152129619
chr6	158232244	158232318	chr6:158232235-158232303
chr6	158282264	158282408	chr6:158282262-158282378
chr6	158285749	158285967	chr6:158285735-158285935
chr6	158312064	158312372	chr6:158312050-158314268
chr6	158312374	158314291	chr6:158312050-158314268
chr6	158413074	158413216	chr6:158413064-158413193
chr6	158429739	158429919	chr6:158429735-158429897
chr6	158449000	158449214	chr6:158448995-158449176
chr6	158452145	158452287	chr6:158452133-158452268
chr6	158461575	158461752	chr6:158461562-158461729
chr6	158479760	158480002	chr6:158479750-158479975
chr6	158481060	158481308	chr6:158481054-158481289
chr6	158489590	158489767	chr6:158489587-158489732
chr6	158493575	158493752	chr6:158493572-158493717
chr6	158494755	158494861	chr6:158494752-158494846
chr6	158498670	158498851	chr6:158498668-158498812
chr6	158501690	158504202	chr6:158501677-158504178
chr6	158506580	158508141	chr6:158506577-158508747
chr6	158508180	158508780	chr6:158506577-158508747
chr6	166157659	166158611	chr6:166157655-166158588
chr6	166159904	166160123	chr6:166159904-166160104
chr6	166160849	166160996	chr6:166160836-166160966
chr6	166162449	166162634	chr6:166162446-166162623
chr6	166164604	166164680	chr6:166164604-166164666
chr6	166164804	166164877	chr6:166164799-166164861
chr6	166165709	166165876	chr6:166165705-166165840
chr6	166166599	166166886	chr6:166166591-166166856
chr6	166167389	166167884	chr6:166167385-166167877
chr6	166168339	166168734	chr6:166168327-166168700
chr6	169705140	169705354	chr6:169705132-169705321
chr6	169705615	169705762	chr6:169705615-169705724
chr6	169707055	169709078	chr6:169707027-169710391
chr6	169709080	169709982	chr6:169707027-169710391
chr6	169710000	169710421	chr6:169707027-169710391
chr6	169712385	169712566	chr6:169712385-169712539
chr6	169714735	169714871	chr6:169714732-169714842
chr6	169715710	169715863	chr6:169715707-169715857
chr6	169715960	169716103	chr6:169715954-169716088
chr6	169717755	169717924	chr6:169717741-169717906
chr6	169718800	169719328	chr6:169718787-169719298
chr6	169721005	169721149	chr6:169721004-169721111
chr6	169723015	169723228	chr6:169723005-169723205
chr6	169723850	169723953	chr6:169723844-169724055
chr6	169724010	169724072	chr6:169723844-169724055
chr6	169725420	169725607	chr6:169725420-169725566
chr7	1082207	1082279	chr7:1082207-1082274
chr7	1082912	1083139	chr7:1082908-1083108
chr7	1086812	1087267	chr7:1086806-1087244
chr7	1088087	1088344	chr7:1088086-1088321
chr7	1091412	1093830	chr7:1091406-1093815
chr7	1530727	1530927	chr7:1530713-1530898
chr7	1536392	1536605	chr7:1536380-1536580
chr7	1537497	1538483	chr7:1537486-1538457
chr7	1538712	1538857	chr7:1538707-1538826
chr7	1539162	1539263	chr7:1539148-1539228
chr7	1539942	1541009	chr7:1539940-1542084

chr7	1541012	1542097	chr7:1539940-1542084
chr7	4682311	4682395	chr7:4682308-4682868
chr7	4682396	4682462	chr7:4682308-4682868
chr7	4682466	4682886	chr7:4682308-4682868
chr7	4722686	4722915	chr7:4722682-4722882
chr7	4740841	4741053	chr7:4740837-4741023
chr7	4754466	4755055	chr7:4754458-4755051
chr7	4755236	4755410	chr7:4755236-4755383
chr7	4756996	4757219	chr7:4756993-4757187
chr7	4759056	4759224	chr7:4759050-4759217
chr7	4759316	4759635	chr7:4759310-4759595
chr7	4761066	4761312	chr7:4761063-4761288
chr7	4762191	4765552	chr7:4762183-4771443
chr7	4765581	4768125	chr7:4762183-4771443
chr7	4768431	4768528	chr7:4762183-4771443
chr7	4768546	4769450	chr7:4762183-4771443
chr7	4769786	4770107	chr7:4762183-4771443
chr7	4770121	4770215	chr7:4762183-4771443
chr7	4770536	4770875	chr7:4762183-4771443
chr7	4770911	4771317	chr7:4762183-4771443
chr7	4771326	4771465	chr7:4762183-4771443
chr7	13891238	13891577	chr7:13891227-13896087
chr7	13891583	13891863	chr7:13891227-13896087
chr7	13891873	13892183	chr7:13891227-13896087
chr7	13892198	13892645	chr7:13891227-13896087
chr7	13892688	13893599	chr7:13891227-13896087
chr7	13893638	13894158	chr7:13891227-13896087
chr7	13894163	13895762	chr7:13891227-13896087
chr7	13895763	13896112	chr7:13891227-13896087
chr7	13900748	13900856	chr7:13900737-13900839
chr7	13906433	13906610	chr7:13906429-13906599
chr7	13907793	13907864	chr7:13907792-13907856
chr7	13909638	13909712	chr7:13909631-13909700
chr7	13911248	13911328	chr7:13911238-13911307
chr7	13931508	13931784	chr7:13931501-13931749
chr7	13935718	13935931	chr7:13935707-13935896
chr7	13939118	13939262	chr7:13939116-13939246
chr7	13974863	13975114	chr7:13974863-13975086
chr7	13983218	13983441	chr7:13983216-13983416
chr7	13985363	13985551	chr7:13985354-13985520
chr7	13986118	13986555	chr7:13986115-13986514
chr7	13986638	13986712	chr7:13986637-13986685
chr7	13986983	13987057	chr7:13986970-13987025
chr7	13988093	13988197	chr7:13988085-13988173
chr7	13988683	13988905	chr7:13988679-13988873
chr7	13989008	13989164	chr7:13989007-13989139
chr7	13989268	13989979	chr7:13989267-13989947
chr7	13990423	13990525	chr7:13990410-13990489
chr7	13990553	13990737	chr7:13990552-13990702
chr7	13991123	13991273	chr7:13991121-13991249
chr7	13991328	13991440	chr7:13991327-13991425
chr7	15611253	15612642	chr7:15611211-15612611
chr7	15626748	15626924	chr7:15626745-15626918
chr7	15647628	15647839	chr7:15647626-15647826
chr7	15685888	15686154	chr7:15685885-15686812
chr7	15686193	15686849	chr7:15685885-15686812
chr7	16462813	16462897	chr7:16462813-16462963
chr7	16462908	16462980	chr7:16462813-16462963
chr7	16465463	16465579	chr7:16465463-16465563
chr7	16504343	16504559	chr7:16504340-16504540
chr7	17298633	17299361	chr7:17298621-17299329
chr7	17309938	17310153	chr7:17309935-17310123
chr7	17313948	17314171	chr7:17313946-17314146

chr7	17322503	17322612	chr7:17322500-17322607
chr7	17327758	17327868	chr7:17327758-17327848
chr7	17329858	17330110	chr7:17329857-17330075
chr7	17330763	17330902	chr7:17330755-17330886
chr7	17333923	17334134	chr7:17333911-17334114
chr7	17334893	17335034	chr7:17334886-17334996
chr7	17335658	17336069	chr7:17335644-17336075
chr7	17338988	17340251	chr7:17338985-17340228
chr7	17342928	17343661	chr7:17342920-17346152
chr7	17343678	17344610	chr7:17342920-17346152
chr7	17344913	17345024	chr7:17342920-17346152
chr7	17345333	17346134	chr7:17342920-17346152
chr7	19020993	19021137	chr7:19020990-19021101
chr7	19056053	19056204	chr7:19056050-19056165
chr7	19110158	19110374	chr7:19110150-19110350
chr7	19113048	19113171	chr7:19113046-19113140
chr7	19113888	19113966	chr7:19113876-19113923
chr7	19115493	19115593	chr7:19115467-19116131
chr7	19115638	19115819	chr7:19115467-19116131
chr7	19115878	19116045	chr7:19115467-19116131
chr7	19116068	19116162	chr7:19115467-19116131
chr7	19116683	19117038	chr7:19116670-19117672
chr7	19117073	19117700	chr7:19116670-19117672
chr7	19144783	19145435	chr7:19144781-19145421
chr7	20134668	20137873	chr7:20134654-20141158
chr7	20138183	20138556	chr7:20134654-20141158
chr7	20138578	20138686	chr7:20134654-20141158
chr7	20138963	20139830	chr7:20134654-20141158
chr7	20139863	20140273	chr7:20134654-20141158
chr7	20140278	20140618	chr7:20134654-20141158
chr7	20140623	20140792	chr7:20134654-20141158
chr7	20140893	20141173	chr7:20134654-20141158
chr7	20154193	20154415	chr7:20154192-20154381
chr7	20158203	20160272	chr7:20158203-20160245
chr7	20161758	20161889	chr7:20161747-20161870
chr7	20164258	20164331	chr7:20164255-20164399
chr7	20170713	20170790	chr7:20170713-20170781
chr7	20174773	20174841	chr7:20174769-20174969
chr7	20174848	20174992	chr7:20174769-20174969
chr7	20184988	20185099	chr7:20184988-20185078
chr7	20198533	20198706	chr7:20198524-20198670
chr7	20217308	20217444	chr7:20217298-20217404
chr7	20782288	20782854	chr7:20782282-20785795
chr7	20782858	20784426	chr7:20782282-20785795
chr7	20784438	20784544	chr7:20782282-20785795
chr7	20784578	20785318	chr7:20782282-20785795
chr7	20785343	20785410	chr7:20782282-20785795
chr7	20785438	20785819	chr7:20782282-20785795
chr7	20785958	20786056	chr7:20785947-20786043
chr7	20786783	20786925	chr7:20786777-20786886
chr7	21428043	21428281	chr7:21428033-21428258
chr7	21428693	21428795	chr7:21428676-21428792
chr7	21429298	21430868	chr7:21429288-21430843
chr7	21446868	21447094	chr7:21446867-21447067
chr7	21477088	21477323	chr7:21477078-21477307
chr7	21481923	21482144	chr7:21481923-21482123
chr7	21511028	21511440	chr7:21511021-21514822
chr7	21511448	21512562	chr7:21511021-21514822
chr7	21512853	21513641	chr7:21511021-21514822
chr7	21513643	21514536	chr7:21511021-21514822
chr7	21514548	21514860	chr7:21511021-21514822
chr7	23245626	23245850	chr7:23245621-23245821
chr7	23254226	23254331	chr7:23254212-23254312

chr7	23256891	23257078	chr7:23256891-23257041
chr7	23273531	23273632	chr7:23273520-23273614
chr7	23274076	23274234	chr7:23274064-23274214
chr7	26152253	26152673	chr7:26152239-26153068
chr7	26152678	26153113	chr7:26152239-26153068
chr7	26167458	26167531	chr7:26167454-26167654
chr7	26167533	26167671	chr7:26167454-26167654
chr7	26177953	26178158	chr7:26177942-26178122
chr7	26183713	26183884	chr7:26183700-26183874
chr7	26184568	26185066	chr7:26184532-26187125
chr7	26185073	26185331	chr7:26184532-26187125
chr7	26185348	26185514	chr7:26184532-26187125
chr7	26185523	26186159	chr7:26184532-26187125
chr7	26186218	26187127	chr7:26184532-26187125
chr7	27092998	27094510	chr7:27092992-27094795
chr7	27094513	27094827	chr7:27092992-27094795
chr7	27095263	27095711	chr7:27095260-27095973
chr7	27095718	27095996	chr7:27095260-27095973
chr7	27100353	27100635	chr7:27100353-27101465
chr7	27100648	27101481	chr7:27100353-27101465
chr7	27102153	27102845	chr7:27102109-27102811
chr7	27106183	27107106	chr7:27106183-27108720
chr7	27107113	27107490	chr7:27106183-27108720
chr7	27107523	27107786	chr7:27106183-27108720
chr7	27107843	27108746	chr7:27106183-27108720
chr7	27110128	27110795	chr7:27110114-27110760
chr7	27113628	27113873	chr7:27113624-27113845
chr7	27119528	27119630	chr7:27119515-27119595
chr7	27122563	27122670	chr7:27122558-27122642
chr7	27124443	27124585	chr7:27124433-27124549
chr7	27126888	27127096	chr7:27126885-27127070
chr7	27128518	27129588	chr7:27128506-27129571
chr7	27130123	27130813	chr7:27130117-27130799
chr7	27136293	27136511	chr7:27136290-27136490
chr7	27140088	27140266	chr7:27140082-27140225
chr7	27141138	27141524	chr7:27141051-27142085
chr7	27141543	27142003	chr7:27141051-27142085
chr7	27142013	27142118	chr7:27141051-27142085
chr7	27142733	27142802	chr7:27142725-27142775
chr7	27143048	27143695	chr7:27143045-27143668
chr7	27145488	27145743	chr7:27145395-27145917
chr7	27145753	27145936	chr7:27145395-27145917
chr7	27147313	27147818	chr7:27147307-27147774
chr7	27149053	27149265	chr7:27149053-27149253
chr7	27150313	27150633	chr7:27150307-27150603
chr7	27152288	27152611	chr7:27152287-27152581
chr7	27153718	27154779	chr7:27153715-27155222
chr7	27154818	27155146	chr7:27153715-27155222
chr7	27155153	27155264	chr7:27153715-27155222
chr7	27155958	27156035	chr7:27155966-27155998
chr7	27156178	27156783	chr7:27156166-27156754
chr7	27157118	27157297	chr7:27157117-27157288
chr7	27157823	27157965	chr7:27157823-27157936
chr7	27162448	27162901	chr7:27162434-27163841
chr7	27162928	27163299	chr7:27162434-27163841
chr7	27163308	27163542	chr7:27162434-27163841
chr7	27163548	27163868	chr7:27162434-27163841
chr7	27164888	27165543	chr7:27164877-27165530
chr7	27167223	27167422	chr7:27167212-27167412
chr7	27169303	27169583	chr7:27169293-27169444;chr7:27169466-27169737
chr7	27169603	27169743	chr7:27169466-27169737
chr7	27170433	27170502	chr7:27170430-27170498
chr7	27170598	27170810	chr7:27170590-27172173

chr7	27170823	27170955	chr7:27170590-27172173
chr7	27170973	27171213	chr7:27170590-27172173
chr7	27171228	27171363	chr7:27170590-27172173
chr7	27171388	27172193	chr7:27170590-27172173
chr7	27172423	27172986	chr7:27172413-27172963
chr7	27173353	27173596	chr7:27173348-27174306
chr7	27173613	27174343	chr7:27173348-27174306
chr7	27174713	27175208	chr7:27174701-27175180
chr7	27177283	27177512	chr7:27177274-27177474
chr7	27179658	27180287	chr7:27179645-27180261
chr7	27181523	27181697	chr7:27181509-27183028
chr7	27181723	27182845	chr7:27181509-27183028
chr7	27182853	27183053	chr7:27181509-27183028
chr7	27184448	27184552	chr7:27184435-27185223
chr7	27184608	27185247	chr7:27184435-27185223
chr7	27193503	27193570	chr7:27193502-27193558
chr7	27194388	27194523	chr7:27194363-27198442
chr7	27194538	27194742	chr7:27194363-27198442
chr7	27194758	27196200	chr7:27194363-27198442
chr7	27196218	27196943	chr7:27194363-27198442
chr7	27196963	27197235	chr7:27194363-27198442
chr7	27197253	27198086	chr7:27194363-27198442
chr7	27198088	27198479	chr7:27194363-27198442
chr7	27199158	27199452	chr7:27199155-27200106
chr7	27199473	27199684	chr7:27199155-27200106
chr7	27199693	27199835	chr7:27199155-27200106
chr7	27199838	27200127	chr7:27199155-27200106
chr7	28299322	28299469	chr7:28299320-28299441
chr7	28409337	28409438	chr7:28409334-28409423
chr7	28409657	28410397	chr7:28409657-28410770
chr7	28410412	28410803	chr7:28409657-28410770
chr7	28412532	28412846	chr7:28412524-28412917
chr7	28412847	28412953	chr7:28412524-28412917
chr7	28435627	28435727	chr7:28435614-28435688
chr7	28488177	28488259	chr7:28488174-28488246
chr7	28491132	28491276	chr7:28491129-28491263
chr7	28494907	28495024	chr7:28494905-28494999
chr7	28507617	28507770	chr7:28507615-28507737
chr7	28513477	28513861	chr7:28513463-28513838
chr7	28570367	28570554	chr7:28570364-28570537
chr7	28609062	28609138	chr7:28609060-28609120
chr7	28685982	28686211	chr7:28685980-28686197
chr7	28718757	28718909	chr7:28718752-28718879
chr7	28724227	28724372	chr7:28724221-28724332
chr7	28726682	28726763	chr7:28726680-28726731
chr7	28749302	28749583	chr7:28749294-28749545
chr7	28778947	28779261	chr7:28778944-28779228
chr7	28784062	28784288	chr7:28784054-28784254
chr7	28804212	28804559	chr7:28804198-28804522
chr7	28809197	28809448	chr7:28809186-28809414
chr7	28818082	28818216	chr7:28818070-28818179
chr7	28819122	28819953	chr7:28819115-28825894
chr7	28820007	28820490	chr7:28819115-28825894
chr7	28820502	28820612	chr7:28819115-28825894
chr7	28820887	28821132	chr7:28819115-28825894
chr7	28821152	28821451	chr7:28819115-28825894
chr7	28821492	28821795	chr7:28819115-28825894
chr7	28821822	28822420	chr7:28819115-28825894
chr7	28822437	28823231	chr7:28819115-28825894
chr7	28823262	28823928	chr7:28819115-28825894
chr7	28823942	28824085	chr7:28819115-28825894
chr7	28824092	28824607	chr7:28819115-28825894
chr7	28824612	28824744	chr7:28819115-28825894

chr7	28824762	28825930	chr7:28819115-28825894
chr7	31339151	31339317	chr7:31339139-31339289
chr7	31340606	31340725	chr7:31340592-31340692
chr7	35202432	35202789	chr7:35202429-35202770
chr7	35204482	35204621	chr7:35204469-35204582
chr7	35231517	35231618	chr7:35231503-35231580
chr7	35240887	35241067	chr7:35240878-35241037
chr7	35244952	35245093	chr7:35244948-35245057
chr7	35248687	35248855	chr7:35248676-35248841
chr7	35249957	35250246	chr7:35249950-35250203
chr7	35251882	35252102	chr7:35251879-35252079
chr7	35253502	35254183	chr7:35253493-35254147
chr7	38978002	38978085	chr7:38977997-38978058
chr7	39006757	39006904	chr7:39006756-39006867
chr7	39034337	39034525	chr7:39034334-39034491
chr7	39083562	39083819	chr7:39083560-39083802
chr7	39085067	39085159	chr7:39085054-39085129
chr7	39085792	39086069	chr7:39085764-39086031
chr7	39120842	39120922	chr7:39120832-39120902
chr7	39132377	39132699	chr7:39132377-39132666
chr7	39204237	39204351	chr7:39204234-39204326
chr7	39207392	39207658	chr7:39207391-39207620
chr7	39249902	39250124	chr7:39249899-39250099
chr7	39339712	39340025	chr7:39339641-39340015
chr7	39373477	39373729	chr7:39373476-39373708
chr7	39406602	39406747	chr7:39406599-39406740
chr7	39433077	39433295	chr7:39433076-39433283
chr7	39451542	39451719	chr7:39451532-39451701
chr7	39460552	39460741	chr7:39460546-39460715
chr7	39464187	39464755	chr7:39464181-39468601
chr7	39464777	39465055	chr7:39464181-39468601
chr7	39465082	39465430	chr7:39464181-39468601
chr7	39465442	39465950	chr7:39464181-39468601
chr7	39465967	39467083	chr7:39464181-39468601
chr7	39467097	39467193	chr7:39464181-39468601
chr7	39467197	39467714	chr7:39464181-39468601
chr7	39467772	39467949	chr7:39464181-39468601
chr7	39467957	39468196	chr7:39464181-39468601
chr7	39468197	39468470	chr7:39464181-39468601
chr7	39468472	39468574	chr7:39464181-39468601
chr7	39471087	39471272	chr7:39471087-39471242
chr7	39490379	39490634	chr7:39490369-39490609
chr7	39493049	39493123	chr7:39493043-39493095
chr7	41960992	41961194	chr7:41960949-41966641
chr7	41961212	41961551	chr7:41960949-41966641
chr7	41961557	41961975	chr7:41960949-41966641
chr7	41961987	41963838	chr7:41960949-41966641
chr7	41963867	41964068	chr7:41960949-41966641
chr7	41964072	41966660	chr7:41960949-41966641
chr7	41967597	41967957	chr7:41967595-41967923
chr7	41972337	41972662	chr7:41972336-41972627
chr7	41977557	41977735	chr7:41977557-41977722
chr7	41978602	41978779	chr7:41978598-41978748
chr7	42023477	42023640	chr7:42023467-42023608
chr7	42025277	42025427	chr7:42025263-42025377
chr7	42026212	42026450	chr7:42026198-42026412
chr7	42040047	42040256	chr7:42040037-42040239
chr7	42045397	42045572	chr7:42045383-42045530
chr7	42048502	42048711	chr7:42048490-42048696
chr7	42076752	42076897	chr7:42076751-42076857
chr7	42148237	42148483	chr7:42148225-42148468
chr7	42152362	42152466	chr7:42152352-42152453
chr7	42172502	42172713	chr7:42172490-42172686

chr7	42223132	42223312	chr7:42223129-42223295
chr7	42227662	42227738	chr7:42227650-42227721
chr7	42229647	42229740	chr7:42229634-42229834
chr7	42229747	42229856	chr7:42229634-42229834
chr7	42236977	42237078	chr7:42236970-42237059
chr7	44104363	44104926	chr7:44104360-44104918
chr7	44106173	44106274	chr7:44106160-44106240
chr7	44106553	44106896	chr7:44106545-44106887
chr7	44107448	44107551	chr7:44107438-44107510
chr7	44107633	44107738	chr7:44107628-44107700
chr7	44107808	44107956	chr7:44107808-44107931
chr7	44108013	44108125	chr7:44108006-44108084
chr7	44108898	44109012	chr7:44108898-44108976
chr7	44109108	44109219	chr7:44109106-44109184
chr7	44109293	44109361	chr7:44109287-44109341
chr7	44109658	44110154	chr7:44109644-44110124
chr7	44110213	44110369	chr7:44110206-44110346
chr7	44110728	44111262	chr7:44110724-44111239
chr7	44111518	44112076	chr7:44111506-44112050
chr7	44112148	44112363	chr7:44112141-44112321
chr7	44112558	44112940	chr7:44112557-44112909
chr7	44112993	44113144	chr7:44112990-44113130
chr7	44113263	44113376	chr7:44113251-44113351
chr7	44113593	44114164	chr7:44113593-44114530
chr7	44114173	44114564	chr7:44113593-44114530
chr7	44876333	44877510	chr7:44876292-44885361
chr7	44877818	44878382	chr7:44876292-44885361
chr7	44878388	44879028	chr7:44876292-44885361
chr7	44879313	44879408	chr7:44876292-44885361
chr7	44879418	44880655	chr7:44876292-44885361
chr7	44880678	44881910	chr7:44876292-44885361
chr7	44881918	44882372	chr7:44876292-44885361
chr7	44882378	44883759	chr7:44876292-44885361
chr7	44883763	44885368	chr7:44876292-44885361
chr7	50304128	50304377	chr7:50304123-50304359
chr7	50304728	50304951	chr7:50304727-50304922
chr7	50308723	50308939	chr7:50308720-50308906
chr7	50319058	50319141	chr7:50319047-50319101
chr7	50327648	50328005	chr7:50327637-50327985
chr7	50367678	50367917	chr7:50367664-50369444
chr7	50367918	50368659	chr7:50367664-50369444
chr7	50368663	50369465	chr7:50367664-50369444
chr7	50369523	50369869	chr7:50369521-50369839
chr7	50376538	50376818	chr7:50376532-50376793
chr7	50382543	50382721	chr7:50382539-50382707
chr7	50384598	50384826	chr7:50384595-50384795
chr7	50387358	50387499	chr7:50387344-50387470
chr7	50391738	50391873	chr7:50391728-50391863
chr7	50399928	50400763	chr7:50399917-50405101
chr7	50400793	50402366	chr7:50399917-50405101
chr7	50402378	50402554	chr7:50399917-50405101
chr7	50402563	50402778	chr7:50399917-50405101
chr7	50402798	50403815	chr7:50399917-50405101
chr7	50403838	50405138	chr7:50399917-50405101
chr7	63450439	63450516	chr7:63450422-63450518
chr7	63454719	63454794	chr7:63454660-63454860
chr7	63454799	63454904	chr7:63454660-63454860
chr7	63455379	63455810	chr7:63455378-63456687
chr7	63455829	63455908	chr7:63455378-63456687
chr7	63455914	63455990	chr7:63455378-63456687
chr7	63456169	63456244	chr7:63455378-63456687
chr7	63456254	63456333	chr7:63455378-63456687
chr7	63456344	63456423	chr7:63455378-63456687

chr7	63456594	63456674	chr7:63455378-63456687
chr7	64794394	64794571	chr7:64794387-64794571
chr7	64807834	64808059	chr7:64807820-64808020
chr7	64814069	64814144	chr7:64814058-64814147
chr7	64815614	64815698	chr7:64815571-64815653
chr7	64830949	64831028	chr7:64830939-64831076
chr7	64831504	64831801	chr7:64831450-64833681
chr7	64831814	64831904	chr7:64831450-64833681
chr7	64831914	64832037	chr7:64831450-64833681
chr7	64832074	64832240	chr7:64831450-64833681
chr7	64832339	64832412	chr7:64831450-64833681
chr7	64832414	64832567	chr7:64831450-64833681
chr7	64832649	64832722	chr7:64831450-64833681
chr7	64832744	64832837	chr7:64831450-64833681
chr7	64832844	64832986	chr7:64831450-64833681
chr7	64833009	64833215	chr7:64831450-64833681
chr7	64833244	64833403	chr7:64831450-64833681
chr7	64833459	64833715	chr7:64831450-64833681
chr7	64971779	64971899	chr7:64971775-64979536
chr7	64971924	64972259	chr7:64971775-64979536
chr7	64972274	64972416	chr7:64971775-64979536
chr7	64972439	64973414	chr7:64971775-64979536
chr7	64973424	64974071	chr7:64971775-64979536
chr7	64974074	64974678	chr7:64971775-64979536
chr7	64974694	64974907	chr7:64971775-64979536
chr7	64975004	64975210	chr7:64971775-64979536
chr7	64975239	64975643	chr7:64971775-64979536
chr7	64975659	64976175	chr7:64971775-64979536
chr7	64976454	64976858	chr7:64971775-64979536
chr7	64976879	64976955	chr7:64971775-64979536
chr7	64977069	64977144	chr7:64971775-64979536
chr7	64977159	64977291	chr7:64971775-64979536
chr7	64977304	64977401	chr7:64971775-64979536
chr7	64977404	64977480	chr7:64971775-64979536
chr7	64977919	64977991	chr7:64971775-64979536
chr7	64977999	64978072	chr7:64971775-64979536
chr7	64978079	64978219	chr7:64971775-64979536
chr7	64978334	64978468	chr7:64971775-64979536
chr7	64978799	64978930	chr7:64971775-64979536
chr7	64979099	64979198	chr7:64971775-64979536
chr7	64979199	64979550	chr7:64971775-64979536
chr7	64983699	64983873	chr7:64983647-64983847
chr7	64990154	64990230	chr7:64989946-64990658
chr7	64990309	64990408	chr7:64989946-64990658
chr7	64990439	64990687	chr7:64989946-64990658
chr7	65373839	65373981	chr7:65373798-65374000
chr7	65383924	65384102	chr7:65383923-65384123
chr7	65388859	65388934	chr7:65388805-65388901
chr7	65398399	65398627	chr7:65398340-65401135
chr7	65398634	65398708	chr7:65398340-65401135
chr7	65398979	65399050	chr7:65398340-65401135
chr7	65399064	65399134	chr7:65398340-65401135
chr7	65399384	65399482	chr7:65398340-65401135
chr7	65399574	65399706	chr7:65398340-65401135
chr7	65399719	65400044	chr7:65398340-65401135
chr7	65400079	65400459	chr7:65398340-65401135
chr7	65400499	65401018	chr7:65398340-65401135
chr7	65401019	65401161	chr7:65398340-65401135
chr7	65960917	65961057	chr7:65960913-65961063
chr7	65972722	65972795	chr7:65972597-65972797
chr7	73593265	73593642	chr7:73593193-73594403
chr7	73593660	73593977	chr7:73593193-73594403
chr7	73594000	73594421	chr7:73593193-73594403

chr7	73595640	73595789	chr7:73595636-73595760
chr7	73595850	73595999	chr7:73595841-73595969
chr7	73596165	73596306	chr7:73596152-73596272
chr7	73596365	73596517	chr7:73596363-73596479
chr7	73596650	73596822	chr7:73596638-73596789
chr7	73596875	73596950	chr7:73596864-73596932
chr7	73597185	73597752	chr7:73597181-73597713
chr7	73599535	73599714	chr7:73599525-73599695
chr7	73603220	73603424	chr7:73603210-73603410
chr7	73605690	73605815	chr7:73605687-73605768
chr7	73605920	73606134	chr7:73605909-73606111
chr7	73606985	73607062	chr7:73606973-73607018
chr7	73607140	73607450	chr7:73607135-73607420
chr7	73607600	73607709	chr7:73607589-73607672
chr7	73616070	73616214	chr7:73616070-73616177
chr7	73624200	73624567	chr7:73624199-73624543
chr7	82758579	82758739	chr7:82758565-82758715
chr7	82760644	82760744	chr7:82760638-82760738
chr7	82771964	82772182	chr7:82771964-82772164
chr7	82822549	82822718	chr7:82822539-82822689
chr7	82824244	82824361	chr7:82824235-82824335
chr7	82916734	82916906	chr7:82916723-82916873
chr7	82949489	82949602	chr7:82949475-82949575
chr7	87152366	87152965	chr7:87152360-87152944
chr7	87154231	87154305	chr7:87154227-87154278
chr7	87154401	87154549	chr7:87154389-87154528
chr7	87155461	87155570	chr7:87155454-87155559
chr7	87162826	87162967	chr7:87162797-87163617
chr7	87162976	87163224	chr7:87162797-87163617
chr7	87163231	87163658	chr7:87162797-87163617
chr7	87164261	87164362	chr7:87164253-87164331
chr7	87164936	87165091	chr7:87164933-87165050
chr7	87166496	87166640	chr7:87166482-87166605
chr7	87167516	87167736	chr7:87167515-87167715
chr7	87171001	87171086	chr7:87170994-87171042
chr7	87173536	87173684	chr7:87173534-87173649
chr7	87174601	87174706	chr7:87174592-87174669
chr7	87179546	87179733	chr7:87179544-87179702
chr7	87181301	87181370	chr7:87181308-87181341
chr7	87182021	87182373	chr7:87182016-87182337
chr7	87184411	87184664	chr7:87184396-87184625
chr7	87185836	87186008	chr7:87185828-87185980
chr7	87188041	87188346	chr7:87188038-87188301
chr7	87190956	87191056	chr7:87190944-87191027
chr7	87193201	87193387	chr7:87193197-87193353
chr7	87193731	87194114	chr7:87193724-87194102
chr7	87194691	87194896	chr7:87194683-87194892
chr7	87195031	87196017	chr7:87195030-87196009
chr7	93922143	93922253	chr7:93922133-93922233
chr7	93923343	93923561	chr7:93923340-93923540
chr7	93926103	93926267	chr7:93926090-93926240
chr7	97005548	97005768	chr7:97005547-97006413
chr7	97005783	97005849	chr7:97005547-97006413
chr7	97005858	97006037	chr7:97005547-97006413
chr7	97006128	97006213	chr7:97005547-97006413
chr7	97006243	97006451	chr7:97005547-97006413
chr7	97006498	97007862	chr7:97006492-97007831
chr7	97008883	97009109	chr7:97008883-97009083
chr7	97009798	97010193	chr7:97009795-97011039
chr7	97010278	97010354	chr7:97009795-97011039
chr7	97010358	97011064	chr7:97009795-97011039
chr7	97020923	97021099	chr7:97020915-97021065
chr7	97022188	97022408	chr7:97022184-97022369

chr7	97023528	97023737	chr7:97023522-97023722
chr7	97024278	97024388	chr7:97024268-97024368
chr7	97736298	97736371	chr7:97736298-97736352
chr7	97737408	97737619	chr7:97737397-97737597
chr7	97739873	97740052	chr7:97739873-97740023
chr7	98211433	98211532	chr7:98211426-98211498
chr7	98212253	98212749	chr7:98212253-98212979
chr7	98212778	98213014	chr7:98212253-98212979
chr7	99504657	99504758	chr7:99504650-99504733
chr7	99504962	99505171	chr7:99504949-99505160
chr7	99505257	99506323	chr7:99505253-99506458
chr7	99506327	99506504	chr7:99505253-99506458
chr7	99512452	99512612	chr7:99512452-99512591
chr7	99519827	99519951	chr7:99519826-99519909
chr7	99520177	99520312	chr7:99520168-99520304
chr7	99521947	99522162	chr7:99521936-99522136
chr7	99525812	99526444	chr7:99525812-99526418
chr7	99531107	99533433	chr7:99531107-99534700
chr7	99533442	99534213	chr7:99531107-99534700
chr7	100049777	100049848	chr7:100049773-100049841
chr7	100051572	100051645	chr7:100051477-100051610
chr7	100056912	100057276	chr7:100056910-100057405
chr7	100057282	100057429	chr7:100056910-100057405
chr7	100057697	100057923	chr7:100057697-100057890
chr7	100063792	100063906	chr7:100063787-100063882
chr7	101815917	101816126	chr7:101815903-101816093
chr7	101817612	101817688	chr7:101817601-101817669
chr7	101818922	101818996	chr7:101818920-101818979
chr7	101913157	101913472	chr7:101913103-101913438
chr7	101916117	101916263	chr7:101916114-101916225
chr7	101932342	101932636	chr7:101932342-101932622
chr7	101933932	101934141	chr7:101933923-101934123
chr7	101959397	101959617	chr7:101959389-101959601
chr7	102028107	102028182	chr7:102028097-102028145
chr7	102070347	102070449	chr7:102070338-102070417
chr7	102097377	102097517	chr7:102097363-102097501
chr7	102104377	102104481	chr7:102104335-102104459
chr7	102111707	102111809	chr7:102111697-102111774
chr7	102115217	102115290	chr7:102115206-102115273
chr7	102125537	102125638	chr7:102125527-102125778
chr7	102125702	102125773	chr7:102125527-102125778
chr7	102158572	102158640	chr7:102158559-102158608
chr7	102170457	102170561	chr7:102170445-102170550
chr7	102178472	102178694	chr7:102178468-102178657
chr7	102189812	102189886	chr7:102189812-102189871
chr7	102193842	102193920	chr7:102193841-102193890
chr7	102195517	102195619	chr7:102195506-102195603
chr7	102196647	102197345	chr7:102196633-102197305
chr7	102198802	102198877	chr7:102198801-102198867
chr7	102200072	102200188	chr7:102200070-102200172
chr7	102201367	102202241	chr7:102201359-102202204
chr7	102204402	102204581	chr7:102204390-102204556
chr7	102205127	102205212	chr7:102205113-102205170
chr7	102227367	102227684	chr7:102227366-102227669
chr7	102234052	102234265	chr7:102234051-102234240
chr7	102239322	102239607	chr7:102239319-102239584
chr7	102248412	102248840	chr7:102248411-102258233
chr7	102248862	102249569	chr7:102248411-102258233
chr7	102249577	102249701	chr7:102248411-102258233
chr7	102249712	102250050	chr7:102248411-102258233
chr7	102250072	102250975	chr7:102248411-102258233
chr7	102250977	102251217	chr7:102248411-102258233
chr7	102251232	102252103	chr7:102248411-102258233

chr7	102252132	102255386	chr7:102248411-102258233
chr7	102255397	102255809	chr7:102248411-102258233
chr7	102255842	102256324	chr7:102248411-102258233
chr7	102256327	102256855	chr7:102248411-102258233
chr7	102256877	102257705	chr7:102248411-102258233
chr7	102257707	102257875	chr7:102248411-102258233
chr7	102257907	102258249	chr7:102248411-102258233
chr7	102273377	102273529	chr7:102273365-102273493
chr7	102274252	102274328	chr7:102274243-102274310
chr7	102275252	102275394	chr7:102275246-102275359
chr7	102277962	102278103	chr7:102277948-102278065
chr7	102280047	102280145	chr7:102280036-102280120
chr7	102280812	102280884	chr7:102280803-102280860
chr7	102281842	102281963	chr7:102281839-102281920
chr7	102282712	102282794	chr7:102282711-102282776
chr7	102283022	102283971	chr7:102283020-102283957
chr7	103472763	103472939	chr7:103472758-103472910
chr7	103482883	103482991	chr7:103482872-103482971
chr7	103669908	103670007	chr7:103669895-103670095
chr7	103670023	103670128	chr7:103669895-103670095
chr7	107168974	107169211	chr7:107168960-107169185
chr7	107169719	107170142	chr7:107169719-107170130
chr7	107173459	107173603	chr7:107173456-107173591
chr7	107174454	107174768	chr7:107174447-107174745
chr7	107179789	107180099	chr7:107179625-107180062
chr7	107182384	107182626	chr7:107182372-107182601
chr7	107184099	107184276	chr7:107184045-107184245
chr7	107185809	107185953	chr7:107185800-107185942
chr7	107186364	107186477	chr7:107186360-107186472
chr7	107186569	107186711	chr7:107186568-107186681
chr7	107189304	107189467	chr7:107189291-107189448
chr7	107190179	107190346	chr7:107190172-107190317
chr7	107195834	107196283	chr7:107195833-107197122
chr7	107196559	107196739	chr7:107195833-107197122
chr7	107196894	107197103	chr7:107195833-107197122
chr7	107200169	107200339	chr7:107200159-107200331
chr7	107923945	107924085	chr7:107923937-107924087
chr7	107924240	107924339	chr7:107924229-107924329
chr7	107938715	107938923	chr7:107938710-107938910
chr7	107959685	107959864	chr7:107959684-107959834
chr7	107960455	107960563	chr7:107960444-107960544
chr7	114086326	114086646	chr7:114086326-114086611
chr7	114087756	114087864	chr7:114087746-114087838
chr7	114088166	114088278	chr7:114088165-114088270
chr7	114162956	114163128	chr7:114162943-114163088
chr7	114285411	114285483	chr7:114285404-114285456
chr7	114288031	114288124	chr7:114288018-114288109
chr7	114366506	114366655	chr7:114366504-114366640
chr7	114414276	114414575	chr7:114414273-114414542
chr7	114415006	114415379	chr7:114414996-114415360
chr7	114416291	114416463	chr7:114416285-114416427
chr7	114426511	114426718	chr7:114426499-114426679
chr7	114498891	114499002	chr7:114498882-114499728
chr7	114499116	114499703	chr7:114498882-114499728
chr7	114534621	114534728	chr7:114534616-114534706
chr7	114538326	114538407	chr7:114538325-114538383
chr7	114570816	114570925	chr7:114570806-114570881
chr7	114622811	114622946	chr7:114622726-114622926
chr7	114628551	114628858	chr7:114628539-114628982
chr7	114628861	114629003	chr7:114628539-114628982
chr7	114629596	114629672	chr7:114629591-114629642
chr7	114629791	114629872	chr7:114629786-114630005
chr7	114629966	114630048	chr7:114629786-114630005

chr7	114631536	114631747	chr7:114631524-114631705
chr7	114642411	114642665	chr7:114642409-114642623
chr7	114644691	114644795	chr7:114644684-114645830
chr7	114644796	114645138	chr7:114644684-114645830
chr7	114645171	114645438	chr7:114644684-114645830
chr7	114645456	114645835	chr7:114644684-114645830
chr7	114652206	114652321	chr7:114652202-114652290
chr7	114653926	114654172	chr7:114653925-114654143
chr7	114656441	114656635	chr7:114656439-114656594
chr7	114658066	114658299	chr7:114658065-114658267
chr7	114659366	114659465	chr7:114659355-114659432
chr7	114659576	114659691	chr7:114659571-114659673
chr7	114661796	114661936	chr7:114661791-114661913
chr7	114662066	114662214	chr7:114662064-114662186
chr7	114663461	114663533	chr7:114663449-114663519
chr7	114664286	114666237	chr7:114664272-114669984
chr7	114666251	114667152	chr7:114664272-114669984
chr7	114667336	114667417	chr7:114664272-114669984
chr7	114667431	114668653	chr7:114664272-114669984
chr7	114668666	114669047	chr7:114664272-114669984
chr7	114669061	114670021	chr7:114664272-114669984
chr7	114689781	114690106	chr7:114689781-114693772
chr7	114690176	114691939	chr7:114689781-114693772
chr7	114691951	114693801	chr7:114689781-114693772
chr7	115935154	115936656	chr7:115935147-115942040
chr7	115936659	115937146	chr7:115935147-115942040
chr7	115937159	115939147	chr7:115935147-115942040
chr7	115939149	115939630	chr7:115935147-115942040
chr7	115939649	115939764	chr7:115935147-115942040
chr7	115939779	115941613	chr7:115935147-115942040
chr7	115941684	115942079	chr7:115935147-115942040
chr7	115950874	115950979	chr7:115950873-115950949
chr7	115954589	115954660	chr7:115954585-115954642
chr7	115956689	115956819	chr7:115956678-115956793
chr7	115968184	115968327	chr7:115968184-115968313
chr7	115974179	115974276	chr7:115974169-115974256
chr7	115984274	115984555	chr7:115984261-115984513
chr7	116030639	116030845	chr7:116030632-116030813
chr7	116110719	116110955	chr7:116110707-116110926
chr7	116117489	116117570	chr7:116117481-116117549
chr7	116120124	116120447	chr7:116120119-116120429
chr7	116121424	116121506	chr7:116121424-116121468
chr7	116124349	116124523	chr7:116124349-116124549
chr7	116157304	116157375	chr7:116157301-116157360
chr7	116159789	116159928	chr7:116159789-116159896
chr7	122301407	122301579	chr7:122301393-122302355
chr7	122301592	122301667	chr7:122301393-122302355
chr7	122301747	122302369	chr7:122301393-122302355
chr7	122302807	122302948	chr7:122302798-122302931
chr7	122303177	122303321	chr7:122303176-122303311
chr7	122303637	122304516	chr7:122303636-122304511
chr7	122310197	122310714	chr7:122310184-122310691
chr7	124747198	124747380	chr7:124747193-124747343
chr7	124754263	124754469	chr7:124754253-124754453
chr7	124763953	124764071	chr7:124763953-124764053
chr7	127610365	127610547	chr7:127610291-127611206
chr7	127610565	127611230	chr7:127610291-127611206
chr7	127611540	127611886	chr7:127611534-127611848
chr7	127611950	127612026	chr7:127611944-127612000
chr7	127613035	127613111	chr7:127613021-127613091
chr7	127613460	127613564	chr7:127613449-127613532
chr7	127613755	127613911	chr7:127613755-127613881
chr7	127614485	127614599	chr7:127614481-127614557

chr7	127614885	127615761	chr7:127614879-127615726
chr7	127615920	127616055	chr7:127615915-127616027
chr7	127616565	127616779	chr7:127616551-127616751
chr7	127617275	127617345	chr7:127617274-127617400
chr7	127617365	127617443	chr7:127617274-127617400
chr7	127617975	127618157	chr7:127617961-127618114
chr7	128242700	128242910	chr7:128242700-128242900
chr7	128252075	128252178	chr7:128252062-128252162
chr7	128254415	128254585	chr7:128254403-128254553
chr7	128937615	128937864	chr7:128937611-128937834
chr7	128937945	128938090	chr7:128937939-128938049
chr7	128939125	128939337	chr7:128939125-128939325
chr7	128940205	128940490	chr7:128940205-128940463
chr7	128940675	128940897	chr7:128940671-128940875
chr7	128941280	128941461	chr7:128941278-128941424
chr7	128942075	128942294	chr7:128942070-128942276
chr7	128945855	128946068	chr7:128945844-128946034
chr7	128947025	128947099	chr7:128947022-128947056
chr7	128947240	128947566	chr7:128947229-128947535
chr7	128947730	128948158	chr7:128947728-128948121
chr7	128948215	128948353	chr7:128948209-128948328
chr7	128948580	128949136	chr7:128948572-128950035
chr7	128949150	128949788	chr7:128948572-128950035
chr7	128949850	128949995	chr7:128948572-128950035
chr7	129611719	129611864	chr7:129611713-129611824
chr7	129630074	129630177	chr7:129630067-129630168
chr7	129633454	129633765	chr7:129633444-129633780
chr7	129657344	129657611	chr7:129657342-129657574
chr7	129671434	129671573	chr7:129671428-129671543
chr7	129677644	129677788	chr7:129677631-129677758
chr7	129690409	129690553	chr7:129690405-129690546
chr7	129703269	129703472	chr7:129703256-129703456
chr7	129709079	129709268	chr7:129709074-129709233
chr7	129710379	129710591	chr7:129710373-129710571
chr7	129711484	129711586	chr7:129711474-129711576
chr7	129717219	129717416	chr7:129717218-129717376
chr7	129727254	129727390	chr7:129727240-129727365
chr7	129744174	129744252	chr7:129744172-129744229
chr7	129755019	129755299	chr7:129755017-129756145
chr7	129755304	129755474	chr7:129755017-129756145
chr7	129755564	129756087	chr7:129755017-129756145
chr7	129756484	129756860	chr7:129756473-129757082
chr7	129756879	129757119	chr7:129756473-129757082
chr7	130731234	130734071	chr7:130731234-130734061
chr7	132784887	132785669	chr7:132784867-132785660
chr7	132796442	132796627	chr7:132796441-132796577
chr7	132804152	132804383	chr7:132804150-132804350
chr7	132813427	132813580	chr7:132813424-132813561
chr7	132815482	132815651	chr7:132815474-132815628
chr7	132838412	132838495	chr7:132838398-132838469
chr7	132885672	132885781	chr7:132885661-132885745
chr7	132973427	132974060	chr7:132973427-132974024
chr7	132975157	132975315	chr7:132975153-132975286
chr7	133022407	133022513	chr7:133022405-133022495
chr7	133024552	133024661	chr7:133024545-133024627
chr7	133051992	133052106	chr7:133051983-133052062
chr7	133070152	133070253	chr7:133070141-133070234
chr7	133081867	133082106	chr7:133081856-133082088
chr7	135681552	135681734	chr7:135681550-135681700
chr7	135684127	135684255	chr7:135684123-135684223
chr7	135715067	135715157	chr7:135714956-135715156
chr7	137875096	137875899	chr7:137874978-137880551
chr7	137875911	137876334	chr7:137874978-137880551

chr7	137876366	137877179	chr7:137874978-137880551
chr7	137877186	137878928	chr7:137874978-137880551
chr7	137879006	137879170	chr7:137874978-137880551
chr7	137879176	137880307	chr7:137874978-137880551
chr7	137880336	137880571	chr7:137874978-137880551
chr7	137882421	137882671	chr7:137882411-137882628
chr7	137884536	137885137	chr7:137884266-137885121
chr7	137885416	137885518	chr7:137885402-137885502
chr7	137901361	137901433	chr7:137901353-137901422
chr7	137905701	137905886	chr7:137905701-137905848
chr7	137908251	137908478	chr7:137908251-137908436
chr7	137912851	137913092	chr7:137912810-137913078
chr7	137915846	137916053	chr7:137915836-137916012
chr7	137922596	137922794	chr7:137922589-137922767
chr7	137928151	137928397	chr7:137928149-137928366
chr7	137933766	137933913	chr7:137933762-137933890
chr7	137948171	137948389	chr7:137948161-137948361
chr7	137999671	137999843	chr7:137999658-137999810
chr7	138001606	138002099	chr7:138001603-138002067
chr7	139573249	139573440	chr7:139573247-139573397
chr7	139575134	139575256	chr7:139575127-139575227
chr7	139583924	139584097	chr7:139583914-139584064
chr7	139600434	139600563	chr7:139600434-139600534
chr7	139700019	139700235	chr7:139700016-139700216
chr7	144397249	144397565	chr7:144397239-144397541
chr7	144398284	144398607	chr7:144398281-144398586
chr7	144398949	144399198	chr7:144398949-144399178
chr7	144399399	144399529	chr7:144399396-144399482
chr7	144399759	144399870	chr7:144399756-144399863
chr7	144400109	144400328	chr7:144400109-144400312
chr7	144401054	144401618	chr7:144401045-144401597
chr7	144401874	144401980	chr7:144401868-144401950
chr7	144404569	144404712	chr7:144404555-144404680
chr7	144407449	144407675	chr7:144407449-144407649
chr7	144410154	144410255	chr7:144410142-144410227
chr7	149472794	149474118	chr7:149472793-149475483
chr7	149474129	149475487	chr7:149472793-149475483
chr7	149476934	149477063	chr7:149476921-149477047
chr7	149477564	149477784	chr7:149477563-149477755
chr7	149487619	149488250	chr7:149487619-149491953
chr7	149488284	149489227	chr7:149487619-149491953
chr7	149489264	149491974	chr7:149487619-149491953
chr7	149492869	149493003	chr7:149492858-149492972
chr7	149494029	149494109	chr7:149493988-149494070
chr7	149494209	149494284	chr7:149494203-149494503
chr7	149494294	149494544	chr7:149494203-149494503
chr7	149496344	149496565	chr7:149496341-149496541
chr7	149497514	149497844	chr7:149497512-149497817
chr7	149764190	149765772	chr7:149764181-149766239
chr7	149765780	149766267	chr7:149764181-149766239
chr7	149769095	149769236	chr7:149769089-149769200
chr7	149770450	149770596	chr7:149770439-149770556
chr7	149771010	149771108	chr7:149770998-149771074
chr7	149771815	149772033	chr7:149771812-149772012
chr7	149773120	149773497	chr7:149773107-149773479
chr7	151148601	151149092	chr7:151148588-151149142
chr7	151149101	151149183	chr7:151148588-151149142
chr7	151149781	151150033	chr7:151149776-151150021
chr7	151160726	151160939	chr7:151160717-151160917
chr7	151167016	151167162	chr7:151167010-151167548
chr7	151167171	151167584	chr7:151167010-151167548
chr7	151174616	151174768	chr7:151174615-151174745
chr7	155458129	155459077	chr7:155458128-155459062

chr7	155461064	155461290	chr7:155461062-155461262
chr7	155462384	155462931	chr7:155462370-155464831
chr7	155462939	155463406	chr7:155462370-155464831
chr7	155463519	155463867	chr7:155462370-155464831
chr7	155463909	155463995	chr7:155462370-155464831
chr7	155464009	155464151	chr7:155462370-155464831
chr7	155464169	155464237	chr7:155462370-155464831
chr7	155464249	155464627	chr7:155462370-155464831
chr7	155464629	155464864	chr7:155462370-155464831
chr8	1697200	1697312	chr8:1697199-1697299
chr8	1701190	1701382	chr8:1701187-1701337
chr8	10723772	10724723	chr8:10723767-10726666
chr8	10725092	10726704	chr8:10723767-10726666
chr8	10730202	10730548	chr8:10730195-10730512
chr8	10898772	10898948	chr8:10898766-10898916
chr8	10924637	10924758	chr8:10924633-10924733
chr8	10993547	10993776	chr8:10993546-10993746
chr8	11195072	11195248	chr8:11195071-11195221
chr8	11200582	11200687	chr8:11200575-11200675
chr8	11676958	11677070	chr8:11676958-11677063
chr8	11692618	11692687	chr8:11692625-11692660
chr8	11700518	11700795	chr8:11700507-11700778
chr8	11704153	11704336	chr8:11704150-11704304
chr8	11705923	11706050	chr8:11705913-11706033
chr8	11707868	11708952	chr8:11707855-11708928
chr8	11718453	11718673	chr8:11718452-11718652
chr8	11748918	11749114	chr8:11748915-11749085
chr8	11750123	11750274	chr8:11750110-11750236
chr8	11755053	11755156	chr8:11755045-11755133
chr8	11756948	11757121	chr8:11756934-11757083
chr8	11758293	11760017	chr8:11758292-11760002
chr8	12104392	12104458	chr8:12104388-12104433
chr8	12108122	12108199	chr8:12108122-12108205
chr8	12109012	12109084	chr8:12108952-12109152
chr8	12109902	12109980	chr8:12109899-12110026
chr8	12110802	12110913	chr8:12110799-12110895
chr8	12111467	12111549	chr8:12111431-12111514
chr8	12112632	12113586	chr8:12112573-12115516
chr8	12113602	12115549	chr8:12112573-12115516
chr8	22114426	22115795	chr8:22114414-22115762
chr8	22116301	22116466	chr8:22116299-22116428
chr8	22116886	22117055	chr8:22116874-22117039
chr8	22118956	22119094	chr8:22118949-22119065
chr8	22119176	22119320	chr8:22119163-22119283
chr8	22119771	22119904	chr8:22119759-22119890
chr8	22120116	22120192	chr8:22120103-22120173
chr8	22120351	22120521	chr8:22120341-22120507
chr8	22120716	22120975	chr8:22120715-22120958
chr8	22121076	22121250	chr8:22121064-22121228
chr8	22121616	22121717	chr8:22121612-22121694
chr8	22122501	22122612	chr8:22122492-22122608
chr8	22122796	22122912	chr8:22122789-22122879
chr8	22123661	22123836	chr8:22123648-22123813
chr8	22125321	22125532	chr8:22125310-22125504
chr8	22125586	22125761	chr8:22125581-22125732
chr8	22127036	22127845	chr8:22127036-22127829
chr8	22127941	22128154	chr8:22127938-22128116
chr8	22128561	22129240	chr8:22128558-22129210
chr8	22130431	22131304	chr8:22130427-22131868
chr8	22131326	22131881	chr8:22130427-22131868
chr8	22132526	22132740	chr8:22132515-22132715
chr8	22133266	22133411	chr8:22133262-22133384
chr8	22687671	22688118	chr8:22687658-22691482

chr8	22688136	22688580	chr8:22687658-22691482
chr8	22688616	22688963	chr8:22687658-22691482
chr8	22689036	22689289	chr8:22687658-22691482
chr8	22689306	22689899	chr8:22687658-22691482
chr8	22689946	22691489	chr8:22687658-22691482
chr8	22692046	22692250	chr8:22692037-22692224
chr8	22692301	22692443	chr8:22692301-22692414
chr8	22692511	22692640	chr8:22692498-22692717
chr8	22692801	22692951	chr8:22692790-22693302
chr8	22692966	22693077	chr8:22692790-22693302
chr8	22693081	22693333	chr8:22692790-22693302
chr8	23678706	23679044	chr8:23678692-23681639
chr8	23679051	23680124	chr8:23678692-23681639
chr8	23680146	23681656	chr8:23678692-23681639
chr8	23682606	23682955	chr8:23682603-23682927
chr8	23702461	23703088	chr8:23702450-23703082
chr8	23704836	23705035	chr8:23704822-23705022
chr8	23706336	23706612	chr8:23706324-23706598
chr8	24915640	24915749	chr8:24915629-24915729
chr8	24917060	24917236	chr8:24917060-24917210
chr8	24917530	24917626	chr8:24917516-24917616
chr8	24917645	24917721	chr8:24917631-24917731
chr8	24917860	24918027	chr8:24917848-24917998
chr8	24918280	24918447	chr8:24918270-24918420
chr8	25841740	25844002	chr8:25841729-25844640
chr8	25844055	25844679	chr8:25841729-25844640
chr8	25850605	25850790	chr8:25850593-25850761
chr8	25858060	25858171	chr8:25858058-25858154
chr8	25858320	25858539	chr8:25858318-25858504
chr8	25861050	25861274	chr8:25861048-25861226
chr8	25861310	25861390	chr8:25861308-25861374
chr8	25862715	25862832	chr8:25862708-25862797
chr8	25886765	25886912	chr8:25886754-25886881
chr8	25887845	25887991	chr8:25887841-25887972
chr8	25889765	25889897	chr8:25889751-25889869
chr8	25908475	25908545	chr8:25908473-25908555
chr8	25919925	25920126	chr8:25919913-25920113
chr8	26033085	26033163	chr8:26033084-26033153
chr8	26040030	26040140	chr8:26040027-26040101
chr8	26040625	26040699	chr8:26040615-26040671
chr8	26040940	26041017	chr8:26040938-26041002
chr8	26041295	26041510	chr8:26041287-26041488
chr8	26042100	26042268	chr8:26042094-26042251
chr8	26044740	26045275	chr8:26044728-26045397
chr8	26045290	26045429	chr8:26044728-26045397
chr8	28318290	28318504	chr8:28318285-28318485
chr8	28339405	28339510	chr8:28339391-28339491
chr8	28342955	28343128	chr8:28342941-28343091
chr8	28345590	28345662	chr8:28345584-28348830
chr8	28345675	28345771	chr8:28345584-28348830
chr8	28345775	28346927	chr8:28345584-28348830
chr8	28346970	28348264	chr8:28345584-28348830
chr8	28348290	28348524	chr8:28345584-28348830
chr8	28348545	28348861	chr8:28345584-28348830
chr8	28349130	28349241	chr8:28349124-28349228
chr8	28350070	28350191	chr8:28350063-28350156
chr8	28351495	28351714	chr8:28351494-28351807
chr8	28351725	28351833	chr8:28351494-28351807
chr8	28352585	28352687	chr8:28352572-28352673
chr8	28353175	28353431	chr8:28353172-28353408
chr8	28356675	28356820	chr8:28356669-28356779
chr8	28359595	28359855	chr8:28359591-28359824
chr8	28360885	28361210	chr8:28360884-28361182

chr8	28365360	28365563	chr8:28365347-28365526
chr8	28368450	28368606	chr8:28368448-28368565
chr8	28382045	28382268	chr8:28382036-28382236
chr8	28385080	28385429	chr8:28385079-28385397
chr8	28386005	28386146	chr8:28386003-28386138
chr8	28386400	28386480	chr8:28386392-28386466
chr8	28402645	28402725	chr8:28402637-28402701
chr8	28890394	28891036	chr8:28890393-28891019
chr8	28891319	28891491	chr8:28891315-28891484
chr8	28891754	28891830	chr8:28891759-28891790
chr8	28893909	28894073	chr8:28893896-28894096
chr8	28963810	28963916	chr8:28963810-28963890
chr8	28970055	28970546	chr8:28970042-28970519
chr8	28980075	28980178	chr8:28980070-28980156
chr8	29009085	29009217	chr8:29009071-29009182
chr8	29009685	29009838	chr8:29009683-29009821
chr8	29018760	29018944	chr8:29018759-29018913
chr8	29042650	29042748	chr8:29042638-29042718
chr8	29045370	29045485	chr8:29045360-29045443
chr8	29046305	29046437	chr8:29046299-29046416
chr8	29047365	29047482	chr8:29047357-29047453
chr8	29048960	29049313	chr8:29048953-29049591
chr8	29049325	29049617	chr8:29048953-29049591
chr8	29050215	29050500	chr8:29050201-29050654
chr8	29050565	29050680	chr8:29050201-29050654
chr8	29051020	29051353	chr8:29051017-29053270
chr8	29051365	29051886	chr8:29051017-29053270
chr8	29051920	29052965	chr8:29051017-29053270
chr8	29052980	29053295	chr8:29051017-29053270
chr8	29053660	29053762	chr8:29053657-29053759
chr8	29063895	29063991	chr8:29063881-29063963
chr8	29064175	29064786	chr8:29064175-29064764
chr8	31639395	31639469	chr8:31639385-31639431
chr8	31639760	31640284	chr8:31639754-31640729
chr8	31640285	31640745	chr8:31639754-31640729
chr8	32220928	32221164	chr8:32220927-32221133
chr8	32228148	32228367	chr8:32228147-32228347
chr8	32287113	32287597	chr8:32287100-32287566
chr8	32548219	32548845	chr8:32548209-32548826
chr8	32595839	32596040	chr8:32595827-32596005
chr8	32605564	32605715	chr8:32605561-32605683
chr8	32614514	32614593	chr8:32614513-32614564
chr8	32616839	32616915	chr8:32616834-32616885
chr8	32647724	32648388	chr8:32647717-32648384
chr8	32721764	32722076	chr8:32721752-32722042
chr8	32727949	32728102	chr8:32727948-32728078
chr8	32742009	32742086	chr8:32742007-32742075
chr8	32742679	32743236	chr8:32742674-32743224
chr8	32748829	32749110	chr8:32748823-32749903
chr8	32749194	32749517	chr8:32748823-32749903
chr8	32749539	32749923	chr8:32748823-32749903
chr8	32751064	32751175	chr8:32751062-32751155
chr8	32754384	32754492	chr8:32754371-32754474
chr8	32756404	32756556	chr8:32756402-32756529
chr8	32759314	32759456	chr8:32759305-32759436
chr8	32760199	32761019	chr8:32760199-32760989
chr8	32763219	32763391	chr8:32763217-32763359
chr8	32763749	32764481	chr8:32763747-32767959
chr8	32764519	32767296	chr8:32763747-32767959
chr8	32767319	32767708	chr8:32763747-32767959
chr8	32767749	32768002	chr8:32763747-32767959
chr8	40532101	40532272	chr8:40532088-40532238
chr8	40581176	40581281	chr8:40581164-40581261

chr8	40873261	40873481	chr8:40873260-40873460
chr8	41645191	41646267	chr8:41645177-41646694
chr8	41646271	41646723	chr8:41645177-41646694
chr8	41647196	41647393	chr8:41647193-41647359
chr8	41648076	41648251	chr8:41648065-41648235
chr8	41649306	41649510	chr8:41649295-41649495
chr8	41650121	41650686	chr8:41650110-41650669
chr8	47736918	47737025	chr8:47736908-47739086
chr8	47737033	47739112	chr8:47736908-47739086
chr8	48917823	48918191	chr8:48917767-48918988
chr8	48918193	48918669	chr8:48917767-48918988
chr8	48918678	48919017	chr8:48917767-48918988
chr8	48919898	48920462	chr8:48919895-48920441
chr8	48921188	48921551	chr8:48921186-48921529
chr8	48921648	48921762	chr8:48921646-48921740
chr8	52110848	52111952	chr8:52110838-52113338
chr8	52111998	52112629	chr8:52110838-52113338
chr8	52112643	52112915	chr8:52110838-52113338
chr8	52112953	52113027	chr8:52110838-52113338
chr8	52113033	52113351	chr8:52110838-52113338
chr8	52116283	52116461	chr8:52116274-52116418
chr8	52118338	52118459	chr8:52118337-52118441
chr8	52126063	52126171	chr8:52126051-52126140
chr8	52131968	52132215	chr8:52131957-52132179
chr8	52133068	52133165	chr8:52133056-52133137
chr8	52133238	52133314	chr8:52133238-52133301
chr8	52136598	52136677	chr8:52136589-52136658
chr8	52137428	52137815	chr8:52137420-52137812
chr8	52142933	52143042	chr8:52142929-52143045
chr8	52149743	52149981	chr8:52149731-52149977
chr8	52158898	52159151	chr8:52158897-52159109
chr8	52161383	52161595	chr8:52161374-52161568
chr8	52163998	52164099	chr8:52163985-52164090
chr8	52165148	52165256	chr8:52165134-52165225
chr8	52166858	52167009	chr8:52166851-52166986
chr8	52171573	52171647	chr8:52171568-52171623
chr8	52171793	52172600	chr8:52171791-52172583
chr8	52180133	52180345	chr8:52180121-52180312
chr8	52201068	52201387	chr8:52201061-52201376
chr8	52206428	52206768	chr8:52206415-52206750
chr8	52210033	52210145	chr8:52210029-52210117
chr8	52214208	52214318	chr8:52214202-52214290
chr8	52217743	52217929	chr8:52217741-52217901
chr8	52220743	52220890	chr8:52220740-52220848
chr8	52221643	52221835	chr8:52221639-52221793
chr8	52230033	52230107	chr8:52230031-52230077
chr8	52246588	52246697	chr8:52246585-52246664
chr8	52248483	52248564	chr8:52248478-52248526
chr8	52249368	52249446	chr8:52249357-52249412
chr8	52403328	52404163	chr8:52403326-52403686;chr8:52403700-52407916
chr8	52404193	52404845	chr8:52403700-52407916
chr8	52404853	52407660	chr8:52403700-52407916
chr8	52407663	52407944	chr8:52403700-52407916
chr8	52409333	52409467	chr8:52409327-52409452
chr8	52409558	52409974	chr8:52409547-52409945
chr8	52413333	52413556	chr8:52413330-52413530
chr8	54457934	54458476	chr8:54457934-54458445
chr8	54459059	54459698	chr8:54459057-54460888
chr8	54459709	54460088	chr8:54459057-54460888
chr8	54460099	54460892	chr8:54459057-54460888
chr8	56160914	56161300	chr8:56160903-56167503
chr8	56161324	56162660	chr8:56160903-56167503
chr8	56162664	56163213	chr8:56160903-56167503

chr8	56163234	56163661	chr8:56160903-56167503
chr8	56163704	56164308	chr8:56160903-56167503
chr8	56164339	56164546	chr8:56160903-56167503
chr8	56164574	56165992	chr8:56160903-56167503
chr8	56165999	56167118	chr8:56160903-56167503
chr8	56167124	56167508	chr8:56160903-56167503
chr8	56168034	56168382	chr8:56168027-56168386
chr8	56171104	56171201	chr8:56171090-56171189
chr8	56173164	56173373	chr8:56173160-56173360
chr8	56179419	56179519	chr8:56179408-56179513
chr8	56209254	56209511	chr8:56209252-56209483
chr8	56211124	56211309	chr8:56211120-56211324
chr8	64580377	64580475	chr8:64580366-64583628
chr8	64580492	64580569	chr8:64580366-64583628
chr8	64580632	64580733	chr8:64580366-64583628
chr8	64580747	64581057	chr8:64580366-64583628
chr8	64581072	64581380	chr8:64580366-64583628
chr8	64581392	64581469	chr8:64580366-64583628
chr8	64581492	64582145	chr8:64580366-64583628
chr8	64582152	64582492	chr8:64580366-64583628
chr8	64582512	64583169	chr8:64580366-64583628
chr8	64583187	64583277	chr8:64580366-64583628
chr8	64583287	64583668	chr8:64580366-64583628
chr8	66177349	66177529	chr8:66177341-66177491
chr8	66178304	66178407	chr8:66178292-66178392
chr8	66562174	66562678	chr8:66562174-66564825
chr8	66562699	66562902	chr8:66562174-66564825
chr8	66562934	66563518	chr8:66562174-66564825
chr8	66563524	66563702	chr8:66562174-66564825
chr8	66563709	66563948	chr8:66562174-66564825
chr8	66563964	66564066	chr8:66562174-66564825
chr8	66564079	66564176	chr8:66562174-66564825
chr8	66564179	66564480	chr8:66562174-66564825
chr8	66564489	66564836	chr8:66562174-66564825
chr8	66565139	66565527	chr8:66565132-66565507
chr8	66566074	66566251	chr8:66566063-66566243
chr8	66566684	66566791	chr8:66566683-66566788
chr8	66566879	66566992	chr8:66566875-66566992
chr8	66572494	66572627	chr8:66572481-66572596
chr8	66573364	66573538	chr8:66573363-66573506
chr8	66576019	66576401	chr8:66576006-66576375
chr8	66580134	66580383	chr8:66580132-66580366
chr8	66592439	66592549	chr8:66592439-66592544
chr8	66593119	66593234	chr8:66593119-66593194
chr8	66595594	66595766	chr8:66595582-66595757
chr8	66597334	66597571	chr8:66597329-66597550
chr8	66599054	66599161	chr8:66599049-66599142
chr8	66601699	66601802	chr8:66601697-66601769
chr8	66602429	66602533	chr8:66602417-66602523
chr8	66608224	66608439	chr8:66608210-66608410
chr8	66612519	66612656	chr8:66612513-66612621
chr8	66612819	66613322	chr8:66612818-66613294
chr8	70051659	70051767	chr8:70051650-70052304
chr8	70052049	70052343	chr8:70051650-70052304
chr8	70055304	70055411	chr8:70055299-70055401
chr8	70058649	70058868	chr8:70058639-70058842
chr8	70061389	70061604	chr8:70061383-70061583
chr8	70066244	70066528	chr8:70066234-70066505
chr8	70068239	70068409	chr8:70068229-70068387
chr8	70068479	70068552	chr8:70068478-70068532
chr8	70069174	70069913	chr8:70069160-70069884
chr8	70071159	70071358	chr8:70071149-70071327
chr8	70071604	70071710	chr8:70071594-70071693

chr8	71841589	71842784	chr8:71841548-71842747
chr8	71842944	71843018	chr8:71842936-71842992
chr8	71843384	71843460	chr8:71843379-71843413
chr8	71843644	71844488	chr8:71843644-71844468
chr8	75407914	75407986	chr8:75407913-75407964
chr8	75408044	75408184	chr8:75408035-75408162
chr8	75485784	75485861	chr8:75485784-75485816
chr8	75490089	75490244	chr8:75490088-75490208
chr8	75495409	75495521	chr8:75495404-75495496
chr8	75527064	75527139	chr8:75526959-75527134
chr8	75535419	75535633	chr8:75535410-75535610
chr8	75539869	75540119	chr8:75539868-75540080
chr8	75543824	75543996	chr8:75543810-75543979
chr8	75547594	75547697	chr8:75547586-75547681
chr8	75551389	75551534	chr8:75551387-75551494
chr8	75553044	75553224	chr8:75553041-75553197
chr8	75555984	75556066	chr8:75555981-75556069
chr8	75558529	75558696	chr8:75558517-75558670
chr8	75558809	75559050	chr8:75558800-75559037
chr8	75560349	75560494	chr8:75560343-75560466
chr8	75563984	75564504	chr8:75563974-75566843
chr8	75564519	75565659	chr8:75563974-75566843
chr8	75565664	75566292	chr8:75563974-75566843
chr8	75566309	75566689	chr8:75563974-75566843
chr8	75566694	75566825	chr8:75563974-75566843
chr8	79764022	79765059	chr8:79764009-79765771
chr8	79765067	79765792	chr8:79764009-79765771
chr8	79766202	79766529	chr8:79766202-79766493
chr8	79766662	79766773	chr8:79766650-79766732
chr8	79766997	79767116	chr8:79766996-79767092
chr8	79767227	79767299	chr8:79767218-79767472
chr8	79767307	79767483	chr8:79767218-79767472
chr8	79767587	79767891	chr8:79767574-79767863
chr8	80485622	80485904	chr8:80485618-80485883
chr8	80486222	80486777	chr8:80486212-80487782
chr8	80486792	80487809	chr8:80486212-80487782
chr8	80499502	80500411	chr8:80499493-80500382
chr8	80504557	80504770	chr8:80504554-80504754
chr8	80513912	80514037	chr8:80513909-80514008
chr8	80518407	80518611	chr8:80518402-80518579
chr8	80518792	80518963	chr8:80518781-80518954
chr8	80519222	80519962	chr8:80519222-80526265
chr8	80519987	80521516	chr8:80519222-80526265
chr8	80521522	80523035	chr8:80519222-80526265
chr8	80523037	80523111	chr8:80519222-80526265
chr8	80523132	80523610	chr8:80519222-80526265
chr8	80523632	80525718	chr8:80519222-80526265
chr8	80525722	80526065	chr8:80519222-80526265
chr8	80526067	80526269	chr8:80519222-80526265
chr8	85177238	85177688	chr8:85177224-85177654
chr8	85187688	85187827	chr8:85187684-85187796
chr8	85196708	85196924	chr8:85196704-85196904
chr8	85202148	85202287	chr8:85202146-85202256
chr8	85203093	85203273	chr8:85203093-85203255
chr8	85206168	85206242	chr8:85206162-85206224
chr8	85207438	85207512	chr8:85207424-85207489
chr8	85209153	85209436	chr8:85209141-85209409
chr8	85212158	85212892	chr8:85212156-85213925
chr8	85213188	85213255	chr8:85212156-85213925
chr8	85213563	85213936	chr8:85212156-85213925
chr8	85215313	85215730	chr8:85215284-85216307
chr8	85215803	85216345	chr8:85215284-85216307
chr8	98944411	98944833	chr8:98944402-98944823

chr8	98946041	98946330	chr8:98946030-98946302
chr8	98947426	98947625	chr8:98947416-98947616
chr8	98948186	98948496	chr8:98948172-98948686
chr8	98948511	98948727	chr8:98948172-98948686
chr8	98948841	98949624	chr8:98948838-98949608
chr8	98950666	98951045	chr8:98950655-98951022
chr8	98951521	98952133	chr8:98951518-98952104
chr8	101492433	101492863	chr8:101492431-101492849
chr8	101543243	101543462	chr8:101543240-101543436
chr8	101552718	101552793	chr8:101552714-101552782
chr8	101558418	101558852	chr8:101558418-101558812
chr8	101570338	101570418	chr8:101570338-101570394
chr8	101573673	101573857	chr8:101573667-101573824
chr8	101577413	101577558	chr8:101577407-101577519
chr8	101584658	101584863	chr8:101584646-101584846
chr8	101599063	101599173	chr8:101599056-101599151
chr8	101619543	101619719	chr8:101619538-101619697
chr8	101631643	101631758	chr8:101631636-101631724
chr8	101632228	101632382	chr8:101632225-101632365
chr8	101636893	101636969	chr8:101636896-101636928
chr8	101644133	101644242	chr8:101644130-101644225
chr8	101649413	101649532	chr8:101649413-101649499
chr8	101664458	101664529	chr8:101664453-101664518
chr8	101666598	101669251	chr8:101666588-101669726
chr8	101669268	101669585	chr8:101666588-101669726
chr8	101669593	101669655	chr8:101666588-101669726
chr8	101669673	101669742	chr8:101666588-101669726
chr8	102648788	102648858	chr8:102648778-102650391
chr8	102648868	102650399	chr8:102648778-102650391
chr8	102651158	102652095	chr8:102651148-102652061
chr8	102652168	102652409	chr8:102652163-102652397
chr8	102653473	102653542	chr8:102653469-102654375
chr8	102653548	102654405	chr8:102653469-102654375
chr8	102655568	102655927	chr8:102655565-102655902
chr8	115408496	115408624	chr8:115408495-115415084
chr8	115408636	115409159	chr8:115408495-115415084
chr8	115409171	115409268	chr8:115408495-115415084
chr8	115409271	115409906	chr8:115408495-115415084
chr8	115409926	115410232	chr8:115408495-115415084
chr8	115410251	115410782	chr8:115408495-115415084
chr8	115410786	115410950	chr8:115408495-115415084
chr8	115410971	115411097	chr8:115408495-115415084
chr8	115411121	115411290	chr8:115408495-115415084
chr8	115411291	115411777	chr8:115408495-115415084
chr8	115411821	115412170	chr8:115408495-115415084
chr8	115412181	115412320	chr8:115408495-115415084
chr8	115412331	115412536	chr8:115408495-115415084
chr8	115412551	115412999	chr8:115408495-115415084
chr8	115413031	115415096	chr8:115408495-115415084
chr8	115418331	115418470	chr8:115418329-115418452
chr8	115492181	115492256	chr8:115492187-115492221
chr8	115515201	115515310	chr8:115515189-115515283
chr8	115587001	115587641	chr8:115587000-115587604
chr8	115603886	115605034	chr8:115603872-115605002
chr8	115619141	115620075	chr8:115619131-115620070
chr8	115623611	115623788	chr8:115623600-115623758
chr8	115637236	115637345	chr8:115637233-115637330
chr8	115661581	115661714	chr8:115661569-115661678
chr8	115667946	115668251	chr8:115667873-115668294
chr8	115668546	115668722	chr8:115668544-115669001
chr8	115668741	115668807	chr8:115668544-115669001
chr8	115668821	115669031	chr8:115668544-115669001
chr8	115669356	115669487	chr8:115669344-115669483

chr8	115698826	115699047	chr8:115698824-115699024
chr8	115700936	115701082	chr8:115700935-115701072
chr8	115809631	115809701	chr8:115809632-115809673
chr8	116845943	116847062	chr8:116845934-116847691
chr8	116847078	116847705	chr8:116845934-116847691
chr8	116850643	116852277	chr8:116850617-116852252
chr8	116852558	116852721	chr8:116852548-116852708
chr8	116854253	116854495	chr8:116854244-116854468
chr8	116856178	116856286	chr8:116856165-116856288
chr8	116856648	116856802	chr8:116856645-116856771
chr8	116857273	116857487	chr8:116857266-116857473
chr8	116858363	116858499	chr8:116858351-116858458
chr8	116861853	116861951	chr8:116861840-116861940
chr8	116863143	116863280	chr8:116863129-116863259
chr8	116864923	116865129	chr8:116864912-116865112
chr8	116866588	116866765	chr8:116866585-116866761
chr8	116874118	116874549	chr8:116874118-116874528
chr8	116874613	116874894	chr8:116874610-116874866
chr8	122781398	122781708	chr8:122781393-122781946
chr8	122781768	122781973	chr8:122781393-122781946
chr8	122820023	122820240	chr8:122820023-122820223
chr8	122863398	122863564	chr8:122863384-122863539
chr8	122951298	122954068	chr8:122951291-122954028
chr8	122973243	122973633	chr8:122973241-122974512
chr8	122973663	122974228	chr8:122973241-122974512
chr8	122974248	122974528	chr8:122973241-122974512
chr8	127735442	127736630	chr8:127735433-127736623
chr8	127738257	127739058	chr8:127738247-127739019
chr8	127740407	127741133	chr8:127740395-127741434
chr8	127741197	127741327	chr8:127740395-127741434
chr8	127741392	127741462	chr8:127740395-127741434
chr8	134477788	134478743	chr8:134477787-134478721
chr8	134502353	134502572	chr8:134502351-134502551
chr8	134509628	134509767	chr8:134509618-134509749
chr8	134510073	134510211	chr8:134510061-134510182
chr8	134510698	134511093	chr8:134510695-134511082
chr8	134512483	134512630	chr8:134512474-134512601
chr8	134520893	134521027	chr8:134520882-134521001
chr8	134532833	134532984	chr8:134532833-134532972
chr8	134564993	134565182	chr8:134564986-134565152
chr8	134565333	134565450	chr8:134565332-134565421
chr8	134583833	134584022	chr8:134583831-134584005
chr8	134588248	134588437	chr8:134588245-134588395
chr8	134590268	134590373	chr8:134590267-134590355
chr8	134594808	134594919	chr8:134594806-134594885
chr8	134599733	134599885	chr8:134599729-134599862
chr8	134600488	134600700	chr8:134600486-134600668
chr8	134601478	134602958	chr8:134601476-134602933
chr8	134608733	134608924	chr8:134608728-134608879
chr8	134610473	134610684	chr8:134610469-134610655
chr8	134637473	134637747	chr8:134637460-134637712
chr8	134639778	134639889	chr8:134639774-134639880
chr8	134657568	134657740	chr8:134657560-134657737
chr8	134696433	134696587	chr8:134696430-134696558
chr8	134712848	134712954	chr8:134712844-134713049
chr8	134712973	134713077	chr8:134712844-134713049
chr8	143020893	143021009	chr8:143020891-143020991
chr8	143021318	143021492	chr8:143021313-143021463
chr8	143021553	143021740	chr8:143021548-143021715
chr8	143023678	143023849	chr8:143023668-143023818
chr8	143419193	143419362	chr8:143419181-143419338
chr8	143421618	143421840	chr8:143421618-143421818
chr8	143428103	143428379	chr8:143428059-143430406

chr8	143428388	143428457	chr8:143428059-143430406
chr8	143428458	143429779	chr8:143428059-143430406
chr8	143429803	143430148	chr8:143428059-143430406
chr8	143430168	143430440	chr8:143428059-143430406
chr8	144266568	144267200	chr8:144266559-144267180
chr8	144268103	144268493	chr8:144268103-144268481
chr8	144291603	144291911	chr8:144291590-144291874
chr8	144292493	144292668	chr8:144292482-144292648
chr8	144297373	144297592	chr8:144297362-144297562
chr8	144308908	144309066	chr8:144308905-144309014
chr8	144309458	144309613	chr8:144309454-144309594
chr8	144309773	144309934	chr8:144309771-144309896
chr8	144311173	144311281	chr8:144311173-144311249
chr8	144311323	144311397	chr8:144311320-144311382
chr8	144311508	144311617	chr8:144311504-144311601
chr8	144311708	144311851	chr8:144311699-144311836
chr8	144311973	144312283	chr8:144311962-144312244
chr8	144312628	144312747	chr8:144312627-144312711
chr8	144313518	144313630	chr8:144313510-144313616
chr8	144313848	144313927	chr8:144313845-144313911
chr8	144313988	144314063	chr8:144313984-144314054
chr8	144314138	144314456	chr8:144314124-144314433
chr8	144330568	144331557	chr8:144330564-144334116
chr8	144331583	144334147	chr8:144330564-144334116
chr8	144335318	144335542	chr8:144335314-144335514
chr8	144336068	144336320	chr8:144336054-144336281
chr8	144474363	144475090	chr8:144474352-144475056
chr8	144475158	144475266	chr8:144475156-144475261
chr8	144475588	144475897	chr8:144475582-144476335
chr8	144475903	144476357	chr8:144475582-144476335
chr8	144720908	144721035	chr8:144720906-144721022
chr8	144721138	144722436	chr8:144721128-144723382
chr8	144722453	144723383	chr8:144721128-144723382
chr8	144730613	144730826	chr8:144730606-144730806
chr8	144753473	144753838	chr8:144753453-144753796
chr8	144754193	144754347	chr8:144754191-144754321
chr8	144754708	144754842	chr8:144754695-144754803
chr8	144755418	144755621	chr8:144755404-144755605
chr8	144773123	144773341	chr8:144773113-144774605
chr8	144773343	144773684	chr8:144773113-144774605
chr8	144773698	144774641	chr8:144773113-144774605
chr8	144777468	144777607	chr8:144777457-144777577
chr8	144778038	144778187	chr8:144778037-144778164
chr8	144778448	144778552	chr8:144778438-144778525
chr8	144780228	144780307	chr8:144780227-144780282
chr8	144787288	144787357	chr8:144787278-144787345
chr8	144798878	144798951	chr8:144798875-144798937
chr8	144800383	144800591	chr8:144800370-144800570
chr8	144802873	144802985	chr8:144802869-144802947
chr8	144803648	144803754	chr8:144803640-144803767
chr8	144804128	144804281	chr8:144804124-144804238
chr8	144807193	144808862	chr8:144807190-144809144
chr8	144809318	144809521	chr8:144809217-144810076
chr8	144809523	144809693	chr8:144809217-144810076
chr8	144810108	144811214	chr8:144810105-144811169
chr8	144827473	144827839	chr8:144827463-144827609;chr8:144827617-144827812
chr8	144828008	144828077	chr8:144828007-144828053
chr8	144828733	144828977	chr8:144828728-144828950
chr8	144829053	144829132	chr8:144829041-144829090
chr8	144829458	144829630	chr8:144829444-144829604
chr8	144830943	144831047	chr8:144830930-144831029
chr8	144837393	144837541	chr8:144837390-144837507
chr8	144838013	144838666	chr8:144838007-144838848

chr8	144841898	144843304	chr8:144841893-144843772
chr8	144843608	144843791	chr8:144841893-144843772
chr8	144844873	144845091	chr8:144844869-144845069
chr8	144845988	144846711	chr8:144845978-144847509
chr8	144846983	144847300	chr8:144845978-144847509
chr8	144847428	144847539	chr8:144845978-144847509
chr8	144876583	144876693	chr8:144876496-144876661
chr8	144876953	144877131	chr8:144876950-144882836
chr8	144877358	144879420	chr8:144876950-144882836
chr8	144879568	144879774	chr8:144876950-144882836
chr8	144880073	144880586	chr8:144876950-144882836
chr8	144880873	144882861	chr8:144876950-144882836
chr8	144886843	144886918	chr8:144886839-144886902
chr8	144889583	144889732	chr8:144889580-144889694
chr8	144889938	144890115	chr8:144889932-144890074
chr8	144890313	144890418	chr8:144890307-144890403
chr8	144894293	144894504	chr8:144894284-144894484
chr8	144895148	144895267	chr8:144895142-144895232
chr8	144901343	144901515	chr8:144901336-144901479
chr8	144901758	144902041	chr8:144901748-144902168
chr8	144902063	144902204	chr8:144901748-144902168
chr8	144973588	144974291	chr8:144973588-144978553
chr8	144974413	144974811	chr8:144973588-144978553
chr8	144975108	144975835	chr8:144973588-144978553
chr8	144975868	144977106	chr8:144973588-144978553
chr8	144977123	144977370	chr8:144973588-144978553
chr8	144977373	144978568	chr8:144973588-144978553
chr8	144994478	144994765	chr8:144994478-144994735
chr8	144995698	144996328	chr8:144995690-144996607
chr8	144996338	144996417	chr8:144995690-144996607
chr8	144996508	144996588	chr8:144995690-144996607
chr8	144998233	144998895	chr8:144997981-144999769
chr8	144998908	144999180	chr8:144997981-144999769
chr8	144999423	144999810	chr8:144997981-144999769
chr8	145000353	145000581	chr8:145000351-145000538
chr8	145001663	145001889	chr8:145001662-145001862
chr8	145002828	145002921	chr8:145002816-145002895
chr9	116230	116485	chr9:116230-118204
chr9	116495	117609	chr9:116230-118204
chr9	117685	117762	chr9:116230-118204
chr9	117775	118234	chr9:116230-118204
chr9	841703	842229	chr9:841689-842192
chr9	842713	842966	chr9:842713-842954
chr9	846973	847087	chr9:846959-847143
chr9	847088	847167	chr9:846959-847143
chr9	893923	894235	chr9:893911-894195
chr9	916768	916941	chr9:916762-916907
chr9	966163	966343	chr9:966155-966355
chr9	967998	968514	chr9:967984-969090
chr9	968533	969124	chr9:967984-969090
chr9	1049868	1050574	chr9:1049857-1050775
chr9	1050578	1050779	chr9:1049857-1050775
chr9	1051573	1052175	chr9:1051569-1052138
chr9	1053008	1053149	chr9:1052999-1053108
chr9	1053733	1053841	chr9:1053721-1053824
chr9	1054623	1054695	chr9:1054623-1054653
chr9	1055723	1057590	chr9:1055721-1057552
chr9	3218301	3218470	chr9:3218296-3225280
chr9	3218476	3219440	chr9:3218296-3225280
chr9	3219461	3219526	chr9:3218296-3225280
chr9	3219571	3220330	chr9:3218296-3225280
chr9	3220351	3220490	chr9:3218296-3225280
chr9	3220501	3222094	chr9:3218296-3225280

chr9	3222111	3222742	chr9:3218296-3225280
chr9	3222751	3225298	chr9:3218296-3225280
chr9	3228856	3228931	chr9:3228846-3228889
chr9	3247046	3248205	chr9:3247036-3248185
chr9	3256996	3257213	chr9:3256990-3257199
chr9	3262936	3263117	chr9:3262934-3263084
chr9	3266221	3266315	chr9:3266207-3266305
chr9	3268241	3268312	chr9:3268238-3268438
chr9	3268316	3268463	chr9:3268238-3268438
chr9	3270371	3270775	chr9:3270370-3270738
chr9	3270941	3271147	chr9:3270929-3271118
chr9	3275501	3275654	chr9:3275499-3275612
chr9	3277341	3277484	chr9:3277339-3277461
chr9	3288141	3288275	chr9:3288130-3288250
chr9	3293081	3293296	chr9:3293076-3293258
chr9	3301556	3301658	chr9:3301542-3301620
chr9	3323971	3324147	chr9:3323960-3324129
chr9	3330271	3330510	chr9:3330258-3330517
chr9	3344731	3344914	chr9:3344731-3344880
chr9	3346666	3346782	chr9:3346666-3346764
chr9	3395471	3395621	chr9:3395471-3395596
chr9	3488781	3488920	chr9:3488775-3488895
chr9	3489381	3489520	chr9:3489368-3489487
chr9	3490281	3490354	chr9:3490278-3490345
chr9	3525831	3525933	chr9:3525746-3526004
chr9	3525961	3526027	chr9:3525746-3526004
chr9	3824136	3824378	chr9:3824126-3828408
chr9	3824406	3824894	chr9:3824126-3828408
chr9	3824936	3825242	chr9:3824126-3828408
chr9	3825246	3825419	chr9:3824126-3828408
chr9	3825431	3826803	chr9:3824126-3828408
chr9	3826816	3827614	chr9:3824126-3828408
chr9	3827616	3828420	chr9:3824126-3828408
chr9	3829321	3829530	chr9:3829309-3829492
chr9	3856021	3856202	chr9:3856008-3856184
chr9	3864076	3864292	chr9:3864067-3864267
chr9	3879436	3879616	chr9:3879426-3879595
chr9	3898691	3898876	chr9:3898690-3898835
chr9	3932371	3932469	chr9:3932359-3932470
chr9	3932806	3932938	chr9:3932794-3932918
chr9	3937036	3937206	chr9:3937027-3937189
chr9	3975146	3975223	chr9:3975133-3975199
chr9	4034529	4035067	chr9:4034526-4035042
chr9	4035849	4036033	chr9:4035837-4035998
chr9	4081239	4081375	chr9:4081227-4081361
chr9	4117774	4118892	chr9:4117767-4118881
chr9	4125734	4125955	chr9:4125733-4125941
chr9	4144849	4145234	chr9:4144838-4145193
chr9	4147304	4147590	chr9:4147296-4147644
chr9	4147609	4147689	chr9:4147296-4147644
chr9	4152079	4152215	chr9:4152066-4152183
chr9	4285549	4286103	chr9:4285538-4286523
chr9	4286119	4286532	chr9:4285538-4286523
chr9	4298384	4298536	chr9:4298383-4298496
chr9	4299424	4299717	chr9:4299420-4299916
chr9	4299734	4299938	chr9:4299420-4299916
chr9	4308789	4308998	chr9:4308776-4308968
chr9	4310404	4310544	chr9:4310400-4310528
chr9	4348244	4348422	chr9:4348231-4348392
chr9	14088187	14088357	chr9:14088176-14088326
chr9	14102432	14102544	chr9:14102421-14102510
chr9	14109672	14109882	chr9:14109662-14109862
chr9	14113007	14113088	chr9:14112998-14113081

chr9	14120452	14120556	chr9:14120439-14120539
chr9	14305872	14306046	chr9:14305868-14306020
chr9	14306992	14307110	chr9:14306988-14307089
chr9	16409505	16409711	chr9:16409502-16419649
chr9	16409735	16411404	chr9:16409502-16419649
chr9	16411410	16411769	chr9:16409502-16419649
chr9	16411770	16412709	chr9:16409502-16419649
chr9	16412755	16413168	chr9:16409502-16419649
chr9	16413200	16413347	chr9:16409502-16419649
chr9	16413365	16414912	chr9:16409502-16419649
chr9	16414930	16417129	chr9:16409502-16419649
chr9	16417160	16417307	chr9:16409502-16419649
chr9	16417325	16418288	chr9:16409502-16419649
chr9	16418295	16418653	chr9:16409502-16419649
chr9	16418710	16418842	chr9:16409502-16419649
chr9	16418895	16419660	chr9:16409502-16419649
chr9	16421260	16421338	chr9:16421261-16421295
chr9	16429300	16429373	chr9:16429299-16429999
chr9	16429375	16430033	chr9:16429299-16429999
chr9	16431405	16431512	chr9:16431402-16431487
chr9	16435040	16435117	chr9:16435037-16435099
chr9	16435565	16437565	chr9:16435554-16437524
chr9	16448435	16448660	chr9:16448435-16448635
chr9	16552530	16552785	chr9:16552529-16552765
chr9	16579790	16580191	chr9:16579790-16580148
chr9	16582990	16583096	chr9:16582982-16583085
chr9	16704756	16705108	chr9:16704752-16705082
chr9	16727806	16727940	chr9:16727796-16728214
chr9	16727956	16728244	chr9:16727796-16728214
chr9	16738371	16738512	chr9:16738359-16738485
chr9	16867366	16867676	chr9:16867358-16867692
chr9	16867961	16868211	chr9:16867948-16868170
chr9	16870646	16870866	chr9:16870645-16870843
chr9	33290520	33290620	chr9:33290510-33290597
chr9	33294430	33295448	chr9:33294419-33295427
chr9	33301265	33301455	chr9:33301262-33301421
chr9	33303200	33303298	chr9:33303190-33303268
chr9	33307200	33307315	chr9:33307193-33307299
chr9	33311115	33311207	chr9:33311105-33311177
chr9	33313655	33313819	chr9:33313653-33313793
chr9	33318730	33318838	chr9:33318730-33318830
chr9	33318910	33319155	chr9:33318909-33319127
chr9	33328580	33328701	chr9:33328580-33328678
chr9	33330405	33330618	chr9:33330395-33330595
chr9	33332475	33332548	chr9:33332471-33332502
chr9	33338520	33338632	chr9:33338509-33338589
chr9	33342755	33342892	chr9:33342745-33342854
chr9	33344070	33344219	chr9:33344068-33344188
chr9	33347050	33347157	chr9:33347037-33347117
chr9	33348015	33348082	chr9:33347662-33348723
chr9	33348510	33348765	chr9:33347662-33348723
chr9	33351565	33351828	chr9:33351559-33351790
chr9	33354085	33354194	chr9:33354085-33354187
chr9	33354850	33354924	chr9:33354850-33354892
chr9	33364010	33364128	chr9:33364009-33364108
chr9	33364715	33364785	chr9:33364707-33364774
chr9	33365630	33366017	chr9:33365232-33366009
chr9	33366635	33366813	chr9:33366628-33366774
chr9	33367525	33367632	chr9:33367514-33367619
chr9	33369905	33371181	chr9:33369905-33371157
chr9	34621386	34621596	chr9:34621378-34621558
chr9	34622021	34622146	chr9:34622019-34622109
chr9	34622356	34622564	chr9:34622346-34622529

chr9	34623426	34623745	chr9:34623424-34623714
chr9	34623876	34624089	chr9:34623863-34624047
chr9	34625741	34625855	chr9:34625741-34625814
chr9	34626656	34626883	chr9:34626655-34626855
chr9	34627701	34628111	chr9:34627696-34628107
chr9	35403501	35403611	chr9:35403499-35403599
chr9	35403756	35403944	chr9:35403747-35403897
chr9	35736236	35736345	chr9:35736226-35736311
chr9	35736401	35736580	chr9:35736391-35736541
chr9	36622202	36622411	chr9:36622192-36622392
chr9	36674842	36674947	chr9:36674837-36674937
chr9	36677172	36677343	chr9:36677159-36677309
chr9	36833287	36833523	chr9:36833274-36840636
chr9	36833537	36833775	chr9:36833274-36840636
chr9	36833782	36833853	chr9:36833274-36840636
chr9	36833877	36834406	chr9:36833274-36840636
chr9	36834442	36834539	chr9:36833274-36840636
chr9	36834597	36838558	chr9:36833274-36840636
chr9	36838582	36840644	chr9:36833274-36840636
chr9	36846852	36846949	chr9:36846842-36846929
chr9	36882007	36882121	chr9:36882003-36882105
chr9	36923367	36923516	chr9:36923354-36923484
chr9	36930887	36930967	chr9:36930887-36930944
chr9	36966557	36966730	chr9:36966548-36966724
chr9	37002647	37002793	chr9:37002647-37002776
chr9	37006482	37006555	chr9:37006472-37006537
chr9	37014997	37015208	chr9:37014996-37015194
chr9	37020642	37020821	chr9:37020635-37020801
chr9	37022522	37022729	chr9:37022517-37022717
chr9	37033992	37034093	chr9:37033985-37034185
chr9	37034122	37034193	chr9:37033985-37034185
chr9	37438127	37439983	chr9:37438113-37442555
chr9	37439987	37440194	chr9:37438113-37442555
chr9	37440227	37442569	chr9:37438113-37442555
chr9	37446552	37446761	chr9:37446542-37446742
chr9	37465217	37465438	chr9:37465214-37465399
chr9	41126433	41126684	chr9:41126429-41128463
chr9	41126698	41127883	chr9:41126429-41128463
chr9	41127938	41128014	chr9:41126429-41128463
chr9	41128033	41128499	chr9:41126429-41128463
chr9	65282712	65285220	chr9:65282100-65285209
chr9	68302877	68303291	chr9:68302866-68304905
chr9	68303292	68303394	chr9:68302866-68304905
chr9	68303442	68304632	chr9:68302866-68304905
chr9	68304637	68304916	chr9:68302866-68304905
chr9	70384637	70386161	chr9:70384596-70388005
chr9	70386182	70386766	chr9:70384596-70388005
chr9	70386787	70387467	chr9:70384596-70388005
chr9	70387492	70388018	chr9:70384596-70388005
chr9	70392897	70393124	chr9:70392897-70393097
chr9	70412872	70414655	chr9:70412858-70414624
chr9	74497366	74497447	chr9:74497364-74497983
chr9	74497471	74497665	chr9:74497364-74497983
chr9	74497676	74497999	chr9:74497364-74497983
chr9	74522186	74522400	chr9:74522176-74522376
chr9	74615601	74615677	chr9:74615599-74615639
chr9	74630281	74630400	chr9:74630281-74630367
chr9	74634641	74634809	chr9:74634630-74634772
chr9	74642416	74642841	chr9:74642413-74642815
chr9	74660616	74660762	chr9:74660616-74660738
chr9	74662481	74662635	chr9:74662473-74662606
chr9	74665491	74665605	chr9:74665487-74665595
chr9	74667796	74667937	chr9:74667790-74667901

chr9	74671791	74671941	chr9:74671788-74671901
chr9	74685471	74685788	chr9:74685462-74693177
chr9	74685876	74687171	chr9:74685462-74693177
chr9	74687181	74687284	chr9:74685462-74693177
chr9	74687326	74688589	chr9:74685462-74693177
chr9	74688611	74689093	chr9:74685462-74693177
chr9	74689396	74691535	chr9:74685462-74693177
chr9	74691541	74691951	chr9:74685462-74693177
chr9	74691956	74693172	chr9:74685462-74693177
chr9	77019660	77019936	chr9:77019654-77020953
chr9	77019960	77020067	chr9:77019654-77020953
chr9	77020155	77020332	chr9:77019654-77020953
chr9	77020340	77020965	chr9:77019654-77020953
chr9	79571785	79572863	chr9:79571772-79572835
chr9	79573265	79573401	chr9:79573261-79573370
chr9	79573690	79573809	chr9:79573688-79573786
chr9	79573935	79574226	chr9:79573932-79574196
chr9	79574880	79574964	chr9:79574872-79574936
chr9	79575780	79576061	chr9:79575778-79576027
chr9	79576140	79576317	chr9:79576132-79576283
chr9	79576475	79576808	chr9:79576474-79576802
chr9	79580065	79580223	chr9:79580059-79580185
chr9	79580235	79580406	chr9:79580226-79580372
chr9	79601415	79601493	chr9:79601414-79601509
chr9	79612660	79612737	chr9:79612655-79612718
chr9	79627380	79627486	chr9:79627373-79627448
chr9	79649480	79649738	chr9:79649480-79649724
chr9	79649820	79649889	chr9:79649819-79649858
chr9	79652590	79652808	chr9:79652589-79652799
chr9	79663455	79663635	chr9:79663449-79663603
chr9	79664995	79665204	chr9:79664983-79665183
chr9	79671230	79671594	chr9:79671208-79671557
chr9	79704785	79704929	chr9:79704782-79704902
chr9	79705890	79705964	chr9:79705888-79705942
chr9	79706760	79706931	chr9:79706746-79706899
chr9	79707135	79707232	chr9:79707122-79707218
chr9	79708120	79708262	chr9:79708117-79708250
chr9	79708595	79708825	chr9:79708592-79708786
chr9	79709635	79709733	chr9:79709622-79709699
chr9	79718000	79718112	chr9:79717996-79718071
chr9	79718725	79719015	chr9:79718721-79718971
chr9	79720050	79720333	chr9:79720045-79720293
chr9	79721750	79721926	chr9:79721740-79721888
chr9	79722455	79722641	chr9:79722450-79722601
chr9	79722960	79723067	chr9:79722958-79723035
chr9	79725040	79725679	chr9:79725036-79726882
chr9	79725690	79725831	chr9:79725036-79726882
chr9	79725840	79726294	chr9:79725036-79726882
chr9	79726335	79726874	chr9:79725036-79726882
chr9	83969233	83969378	chr9:83969230-83969440
chr9	83970173	83970286	chr9:83970161-83970261
chr9	85547148	85547319	chr9:85547136-85547286
chr9	85575323	85575427	chr9:85575314-85575414
chr9	85659203	85659334	chr9:85659189-85659389
chr9	85659338	85659410	chr9:85659189-85659389
chr9	91409047	91410938	chr9:91409044-91410906
chr9	91420887	91421103	chr9:91420882-91421082
chr9	91423642	91423893	chr9:91423639-91423862
chr9	93951622	93952369	chr9:93951621-93952328
chr9	93952742	93952848	chr9:93952728-93952813
chr9	93952897	93953469	chr9:93952895-93953431
chr9	93953952	93954152	chr9:93953939-93954139
chr9	93954927	93955176	chr9:93954923-93955372

chr9	93955177	93955385	chr9:93954923-93955372
chr9	96385953	96386965	chr9:96385940-96388459
chr9	96386973	96388486	chr9:96385940-96388459
chr9	96392398	96392554	chr9:96392397-96392536
chr9	96394828	96394985	chr9:96394822-96394942
chr9	96395878	96396083	chr9:96395866-96396066
chr9	96398163	96398342	chr9:96398163-96398314
chr9	96417613	96417974	chr9:96417612-96418329
chr9	96417983	96418358	chr9:96417612-96418329
chr9	97853254	97854057	chr9:97853253-97856715
chr9	97854079	97854411	chr9:97853253-97856715
chr9	97854439	97856542	chr9:97853253-97856715
chr9	97856549	97856683	chr9:97853253-97856715
chr9	99821854	99822422	chr9:99821854-99822407
chr9	99823464	99823684	chr9:99823463-99823663
chr9	99825669	99825850	chr9:99825658-99825832
chr9	99826729	99826844	chr9:99826726-99826801
chr9	99828054	99828330	chr9:99828040-99828993
chr9	99828359	99829017	chr9:99828040-99828993
chr9	99832699	99832834	chr9:99832688-99832818
chr9	99833289	99834101	chr9:99833281-99834059
chr9	99844649	99844863	chr9:99844648-99844848
chr9	99847439	99847660	chr9:99847436-99847615
chr9	99863624	99864004	chr9:99863619-99866891
chr9	99864009	99865297	chr9:99863619-99866891
chr9	99865344	99866317	chr9:99863619-99866891
chr9	99866334	99866927	chr9:99863619-99866891
chr9	100474835	100475052	chr9:100474828-100475028
chr9	100572580	100572698	chr9:100572576-100572676
chr9	100576525	100576692	chr9:100576515-100576665
chr9	101398875	101399201	chr9:101398872-101399189
chr9	101399395	101399547	chr9:101399394-101399530
chr9	101399910	101400048	chr9:101399883-101400010
chr9	101401080	101401292	chr9:101401070-101401270
chr9	101407940	101410688	chr9:101407928-101410660
chr9	107484863	107485940	chr9:107484851-107485926
chr9	107487028	107487208	chr9:107487027-107487192
chr9	107487298	107488294	chr9:107487294-107488267
chr9	107488943	107489142	chr9:107488929-107489130
chr9	107489168	107489798	chr9:107489167-107489766
chr9	107490123	107490517	chr9:107490118-107490482
chr9	110875577	110875754	chr9:110875572-110875722
chr9	110877532	110877734	chr9:110877521-110877721
chr9	110941427	110941533	chr9:110941420-110941520
chr9	113038389	113038461	chr9:113038379-113044268
chr9	113038464	113038536	chr9:113038379-113044268
chr9	113038804	113039296	chr9:113038379-113044268
chr9	113039334	113039677	chr9:113038379-113044268
chr9	113039679	113039757	chr9:113038379-113044268
chr9	113039759	113039826	chr9:113038379-113044268
chr9	113039874	113040789	chr9:113038379-113044268
chr9	113040819	113040951	chr9:113038379-113044268
chr9	113041224	113042807	chr9:113038379-113044268
chr9	113042809	113042990	chr9:113038379-113044268
chr9	113042994	113044309	chr9:113038379-113044268
chr9	113048094	113048310	chr9:113048081-113048281
chr9	113049369	113049512	chr9:113049361-113049496
chr9	113049799	113049882	chr9:113049790-113049860
chr9	113056569	113056783	chr9:113056556-113056759
chr9	114334195	114335488	chr9:114334155-114337306
chr9	114335810	114336157	chr9:114334155-114337306
chr9	114336160	114336998	chr9:114334155-114337306
chr9	114337010	114337334	chr9:114334155-114337306

chr9	114341545	114341758	chr9:114341532-114341725
chr9	114342020	114342164	chr9:114342008-114342125
chr9	114343710	114343895	chr9:114343707-114346009
chr9	114343910	114343999	chr9:114343707-114346009
chr9	114344000	114345053	chr9:114343707-114346009
chr9	114345370	114345651	chr9:114343707-114346009
chr9	114345680	114346024	chr9:114343707-114346009
chr9	114346670	114346825	chr9:114346668-114346784
chr9	114347730	114347945	chr9:114347723-114347900
chr9	114348900	114349038	chr9:114348886-114348999
chr9	114350860	114351048	chr9:114350858-114351021
chr9	114355925	114356178	chr9:114355924-114356136
chr9	114356865	114356977	chr9:114356862-114356969
chr9	114357930	114358350	chr9:114357920-114358337
chr9	114359355	114359709	chr9:114359086-114359794
chr9	114359745	114359826	chr9:114359086-114359794
chr9	114359900	114360077	chr9:114359895-114360062
chr9	114361705	114361931	chr9:114361703-114361911
chr9	114362410	114362548	chr9:114362405-114362533
chr9	114364560	114364635	chr9:114364559-114364619
chr9	114367550	114367733	chr9:114367542-114367697
chr9	114368210	114368289	chr9:114368199-114368246
chr9	114368450	114368630	chr9:114368438-114368595
chr9	114374105	114374211	chr9:114374092-114374167
chr9	114376465	114377657	chr9:114376465-114377635
chr9	114381060	114381492	chr9:114381059-114381446
chr9	114383875	114384087	chr9:114383863-114384063
chr9	114387860	114388110	chr9:114387859-114388081
chr9	114394370	114394441	chr9:114394356-114394405
chr9	115021125	115021275	chr9:115021117-115021267
chr9	115023975	115024096	chr9:115023972-115024072
chr9	115097855	115098064	chr9:115097843-115098043
chr9	122202577	122204793	chr9:122202576-122204780
chr9	122209622	122209727	chr9:122209613-122209717
chr9	122213607	122213959	chr9:122213605-122213922
chr9	122213977	122214088	chr9:122213973-122214069
chr9	122214282	122214387	chr9:122214282-122214383
chr9	122217077	122217323	chr9:122217067-122217288
chr9	122221052	122221769	chr9:122221048-122221740
chr9	122222992	122223066	chr9:122222978-122223178
chr9	122223087	122223177	chr9:122222978-122223178
chr9	122226377	122226520	chr9:122226375-122226497
chr9	122226847	122227066	chr9:122226847-122227030
chr9	122227412	122227601	chr9:122227408-122227586
chr9	122227802	122228019	chr9:122227799-122228431
chr9	122228032	122228445	chr9:122227799-122228431
chr9	122228667	122228974	chr9:122228656-122228941
chr9	122229382	122229651	chr9:122229370-122229626
chr9	124004063	124004292	chr9:124004061-124004261
chr9	124011623	124011722	chr9:124011609-124012468
chr9	124011753	124012485	chr9:124011609-124012468
chr9	124013973	124014181	chr9:124013960-124014163
chr9	124015108	124015560	chr9:124015097-124015525
chr9	124021098	124021318	chr9:124021098-124021304
chr9	124021823	124021900	chr9:124021818-124021867
chr9	124032428	124033103	chr9:124032419-124033301
chr9	124033183	124033315	chr9:124032419-124033301
chr9	124481238	124483033	chr9:124481235-124483005
chr9	124491088	124491267	chr9:124491080-124491228
chr9	124493038	124493188	chr9:124493029-124493149
chr9	124498588	124498805	chr9:124498588-124498788
chr9	124500098	124500726	chr9:124500089-124500715
chr9	124500948	124501020	chr9:124500947-124500986

chr9	124503078	124503264	chr9:124503078-124503220
chr9	124503298	124503436	chr9:124503293-124503410
chr9	124506958	124507425	chr9:124506957-124507235;chr9:124507248-124507430
chr9	124517278	124517957	chr9:124517274-124522793
chr9	124517963	124518751	chr9:124517274-124522793
chr9	124518778	124519607	chr9:124517274-124522793
chr9	124519923	124522548	chr9:124517274-124522793
chr9	124522558	124522833	chr9:124517274-124522793
chr9	124524728	124524876	chr9:124524720-124524873
chr9	124526788	124526928	chr9:124526778-124526900
chr9	124535878	124536169	chr9:124535877-124536132
chr9	124538093	124538344	chr9:124538091-124538319
chr9	124540043	124540228	chr9:124540032-124540187
chr9	124543813	124543893	chr9:124543801-124543857
chr9	124554338	124554590	chr9:124554327-124554570
chr9	124595758	124595839	chr9:124595748-124595808
chr9	124733303	124733375	chr9:124733307-124733349
chr9	124755293	124755514	chr9:124755287-124755487
chr9	124771033	124771349	chr9:124771019-124771310
chr9	125747403	125747684	chr9:125747344-125747653
chr9	125748193	125748647	chr9:125748181-125748623
chr9	125797408	125797475	chr9:125797394-125797465
chr9	125843713	125843837	chr9:125843712-125843812
chr9	125844568	125844999	chr9:125844565-125844982
chr9	125865268	125865346	chr9:125865270-125865305
chr9	125909653	125909870	chr9:125909651-125909851
chr9	125915693	125915955	chr9:125915685-125915927
chr9	125929658	125929877	chr9:125929654-125929845
chr9	125935473	125935633	chr9:125935471-125935607
chr9	125949378	125949451	chr9:125949377-125949440
chr9	125960693	125960870	chr9:125960683-125960849
chr9	125962113	125962245	chr9:125962101-125962214
chr9	125963023	125963135	chr9:125963011-125963101
chr9	125965843	125967094	chr9:125965830-125967377
chr9	125967098	125967366	chr9:125965830-125967377
chr9	126614453	126614623	chr9:126614442-126614588
chr9	126615388	126615594	chr9:126615382-126615569
chr9	126659108	126659333	chr9:126659107-126659307
chr9	126690848	126691089	chr9:126690835-126691068
chr9	126693153	126693351	chr9:126693141-126693323
chr9	126693528	126693630	chr9:126693523-126693601
chr9	126693748	126693858	chr9:126693745-126693845
chr9	126695838	126696020	chr9:126695838-126696003
chr9	126696303	126697434	chr9:126696293-126701032
chr9	126697443	126697828	chr9:126696293-126701032
chr9	126698168	126700578	chr9:126696293-126701032
chr9	126700593	126700979	chr9:126696293-126701032
chr9	126700983	126701056	chr9:126696293-126701032
chr9	128434524	128434630	chr9:128434513-128434613
chr9	128435659	128435926	chr9:128435652-128435802;chr9:128435805-128435905
chr9	128436849	128437036	chr9:128436848-128436998
chr9	128437119	128437190	chr9:128437117-128437183
chr9	128437194	128437335	chr9:128437184-128437334
chr9	129665649	129666147	chr9:129665640-129666126
chr9	129674099	129674321	chr9:129674093-129674293
chr9	129719244	129719449	chr9:129719230-129719418
chr9	129720604	129720812	chr9:129720595-129720774
chr9	129722229	129722702	chr9:129722216-129722674
chr9	130578964	130579783	chr9:130578964-130579764
chr9	130580659	130582106	chr9:130580658-130582086
chr9	130595484	130595594	chr9:130595482-130595588
chr9	130609949	130610014	chr9:130609953-130609987
chr9	130612459	130612529	chr9:130612455-130612505

chr9	130612969	130613044	chr9:130612955-130613027
chr9	130614289	130614356	chr9:130614287-130614345
chr9	130616354	130616542	chr9:130616354-130616517
chr9	130617809	130617918	chr9:130617796-130617895
chr9	130620364	130620472	chr9:130620353-130620458
chr9	130622714	130622830	chr9:130622707-130622810
chr9	130623619	130623743	chr9:130623610-130623711
chr9	130626364	130626545	chr9:130626363-130626505
chr9	130627384	130627611	chr9:130627383-130627583
chr9	130630634	130630818	chr9:130630627-130630788
chr9	130631174	130631306	chr9:130631160-130631293
chr9	130631569	130631675	chr9:130631556-130631630
chr9	130631944	130632056	chr9:130631941-130632022
chr9	130632214	130632316	chr9:130632201-130632278
chr9	130634624	130634773	chr9:130634620-130634738
chr9	130635864	130636160	chr9:130635863-130636126
chr9	130637014	130637755	chr9:130637013-130638352
chr9	130637774	130637910	chr9:130637013-130638352
chr9	130637924	130638383	chr9:130637013-130638352
chr9	130664599	130664914	chr9:130664593-130664876
chr9	130666619	130666830	chr9:130666607-130666798
chr9	130668159	130668309	chr9:130668157-130668313
chr9	130670204	130670416	chr9:130670202-130670402
chr9	130678529	130678674	chr9:130678528-130678640
chr9	130681249	130681609	chr9:130681247-130682981
chr9	130681634	130682434	chr9:130681247-130682981
chr9	130682444	130683011	chr9:130681247-130682981
chr9	132582188	132583278	chr9:132582184-132583263
chr9	132585818	132586032	chr9:132585806-132586006
chr9	132587328	132587582	chr9:132587328-132587551
chr9	132589228	132590290	chr9:132589227-132590266
chr9	132944003	132944082	chr9:132943999-132944046
chr9	132945533	132945683	chr9:132945532-132945669
chr9	132946603	132947028	chr9:132946596-132947001
chr9	132947063	132947286	chr9:132947060-132947246
chr9	132949703	132949840	chr9:132949689-132949804
chr9	132951208	132951768	chr9:132951208-132953763
chr9	132952058	132952128	chr9:132951208-132953763
chr9	132952138	132952246	chr9:132951208-132953763
chr9	132952248	132952321	chr9:132951208-132953763
chr9	132952578	132953376	chr9:132951208-132953763
chr9	132975088	132975219	chr9:132975016-132975204
chr9	132976558	132976637	chr9:132976460-132976595
chr9	132978508	132978872	chr9:132978506-132978841
chr9	132982703	132982872	chr9:132982645-132982845
chr9	132986668	132986814	chr9:132986658-132986778
chr9	132987293	132987425	chr9:132987281-132987419
chr9	132988203	132988485	chr9:132988196-132988468
chr9	132989073	132989212	chr9:132989060-132989198
chr9	132989653	132989936	chr9:132989645-132989907
chr9	132990883	132991201	chr9:132990871-132991687
chr9	132991208	132991705	chr9:132990871-132991687
chr9	134317101	134317534	chr9:134317097-134317502
chr9	134319446	134319523	chr9:134319437-134319484
chr9	134326591	134326689	chr9:134326579-134326659
chr9	134330821	134331050	chr9:134330819-134331019
chr9	134371971	134372148	chr9:134371970-134372139
chr9	134374006	134374155	chr9:134374006-134374130
chr9	134401406	134401574	chr9:134401395-134401540
chr9	134401631	134406101	chr9:134401631-134406394
chr9	134408151	134408335	chr9:134408148-134408299
chr9	134408941	134409153	chr9:134408939-134409119
chr9	134417166	134417348	chr9:134417157-134417327

chr9	134421686	134421826	chr9:134421675-134421805
chr9	134429111	134429258	chr9:134429107-134429240
chr9	134431911	134432019	chr9:134431904-134431996
chr9	134434111	134434242	chr9:134434101-134434207
chr9	134436466	134437733	chr9:134436466-134440585
chr9	134437741	134438854	chr9:134436466-134440585
chr9	134438856	134439131	chr9:134436466-134440585
chr9	134439141	134440092	chr9:134436466-134440585
chr9	134440106	134440598	chr9:134436466-134440585
chr9	135693411	135694498	chr9:135693406-135694457
chr9	135695051	135695300	chr9:135695049-135695263
chr9	135695851	135696058	chr9:135695837-135696037
chr9	135696621	135696831	chr9:135696611-135696805
chr9	135697506	135697650	chr9:135697505-135697627
chr9	135698331	135698514	chr9:135698328-135698476
chr9	135699001	135699151	chr9:135698994-135699126
chr9	135699411	135699560	chr9:135699402-135699528
chr9	136006536	136006815	chr9:136006536-136012024
chr9	136006831	136007229	chr9:136006536-136012024
chr9	136007261	136007326	chr9:136006536-136012024
chr9	136007551	136008887	chr9:136006536-136012024
chr9	136008891	136010909	chr9:136006536-136012024
chr9	136010946	136011080	chr9:136006536-136012024
chr9	136011081	136012065	chr9:136006536-136012024
chr9	136013201	136013323	chr9:136013198-136013296
chr9	136013876	136013991	chr9:136013863-136013969
chr9	136016276	136016459	chr9:136016264-136016429
chr9	136019176	136019502	chr9:136019167-136019480
chr9	136049636	136050588	chr9:136049635-136050580
chr9	136060196	136060405	chr9:136060194-136060394
chr9	136095201	136095309	chr9:136095188-136095285
chr9	136196261	136197769	chr9:136196249-136197743
chr9	136198661	136198825	chr9:136198651-136198820
chr9	136198921	136199094	chr9:136198907-136199059
chr9	136199681	136199901	chr9:136199677-136199880
chr9	136200581	136200777	chr9:136200581-136200753
chr9	136201176	136201695	chr9:136201164-136201674
chr9	136202946	136203195	chr9:136202945-136203158
chr9	136203921	136204131	chr9:136203909-136204109
chr9	136204936	136205114	chr9:136204933-136205109
chr9	137007826	137008001	chr9:137007814-137007964
chr9	137008421	137008523	chr9:137008415-137008515
chr9	137025616	137025830	chr9:137025609-137025809
chr10	3776007	3776703	chr10:3775995-3779590
chr10	3776747	3776923	chr10:3775995-3779590
chr10	3776947	3777188	chr10:3775995-3779590
chr10	3777217	3777732	chr10:3775995-3779590
chr10	3777797	3779020	chr10:3775995-3779590
chr10	3779062	3779629	chr10:3775995-3779590
chr10	3780117	3782258	chr10:3780105-3782214
chr10	3783217	3783292	chr10:3783215-3783281
chr10	3784912	3785294	chr10:3784912-3785281
chr10	8053607	8053792	chr10:8053603-8053778
chr10	8054777	8054909	chr10:8054692-8054891
chr10	8055287	8055457	chr10:8055286-8055896
chr10	8055467	8055910	chr10:8055286-8055896
chr10	8058307	8058877	chr10:8058304-8058841
chr10	8062372	8062501	chr10:8062286-8062486
chr10	8063997	8064166	chr10:8063992-8064138
chr10	8069472	8069633	chr10:8069472-8069598
chr10	8073752	8074490	chr10:8073738-8075198
chr10	8074542	8075234	chr10:8073738-8075198
chr10	23192337	23192821	chr10:23192326-23193314

chr10	23192822	23193453	chr10:23192326-23193314;chr10:23193331-23193423
chr10	23193717	23194068	chr10:23193703-23194252
chr10	23194092	23194264	chr10:23193703-23194252
chr10	26223887	26224004	chr10:26223886-26223977
chr10	26224542	26224717	chr10:26224538-26224688
chr10	26274902	26275112	chr10:26274890-26275090
chr10	26292912	26293026	chr10:26292901-26292991
chr10	26300797	26301002	chr10:26300787-26300995
chr10	26304047	26304223	chr10:26304033-26304183
chr10	27672902	27673100	chr10:27672874-27675415
chr10	27673107	27673628	chr10:27672874-27675415
chr10	27673652	27673868	chr10:27672874-27675415
chr10	27673902	27674204	chr10:27672874-27675415
chr10	27674227	27674457	chr10:27672874-27675415
chr10	27674467	27675441	chr10:27672874-27675415
chr10	27675512	27675582	chr10:27675520-27675554
chr10	27718562	27718632	chr10:27718562-27718596
chr10	27734467	27734818	chr10:27734455-27734791
chr10	27735232	27735396	chr10:27735220-27735374
chr10	27739302	27739502	chr10:27739277-27739477
chr10	27741357	27741529	chr10:27741344-27741504
chr10	27743227	27743582	chr10:27743227-27743545
chr10	27745432	27745604	chr10:27745419-27745594
chr10	27745717	27746092	chr10:27745706-27746060
chr10	31319179	31319319	chr10:31319124-31319292
chr10	31320139	31320235	chr10:31320125-31320207
chr10	31320619	31320727	chr10:31320605-31320698
chr10	31321144	31321564	chr10:31321134-31321532
chr10	31321629	31321875	chr10:31321620-31321851
chr10	31322014	31322123	chr10:31322009-31322084
chr10	31351154	31351353	chr10:31351143-31351343
chr10	31355154	31355229	chr10:31355143-31355192
chr10	31358149	31358609	chr10:31358144-31358590
chr10	31373029	31373197	chr10:31373017-31373173
chr10	31387124	31387308	chr10:31387123-31387266
chr10	31387729	31387826	chr10:31387719-31387798
chr10	31389604	31389681	chr10:31389604-31389660
chr10	31461049	31461253	chr10:31461036-31461237
chr10	31495784	31495864	chr10:31495775-31495838
chr10	31502354	31502540	chr10:31502347-31502509
chr10	31510674	31510896	chr10:31510672-31510875
chr10	31514604	31514713	chr10:31514602-31514708
chr10	31520129	31520801	chr10:31520125-31521936
chr10	31520814	31521969	chr10:31520125-31521936
chr10	31523944	31524157	chr10:31523932-31524113
chr10	31526679	31527003	chr10:31526671-31529814
chr10	31527009	31527081	chr10:31526671-31529814
chr10	31527089	31527397	chr10:31526671-31529814
chr10	31527444	31529848	chr10:31526671-31529814
chr10	35126802	35127213	chr10:35126790-35127193
chr10	35127437	35127599	chr10:35127423-35127567
chr10	35137782	35137898	chr10:35137781-35137879
chr10	35148367	35148701	chr10:35148367-35148663
chr10	35152282	35152436	chr10:35152279-35152398
chr10	35154067	35154290	chr10:35154060-35154565
chr10	35154292	35154592	chr10:35154060-35154565
chr10	35167527	35167836	chr10:35167515-35167803
chr10	35175587	35175743	chr10:35175586-35175705
chr10	35175862	35176032	chr10:35175852-35175999
chr10	35178882	35179010	chr10:35178871-35178986
chr10	35179147	35179884	chr10:35179133-35179847
chr10	35182752	35182967	chr10:35182743-35182943
chr10	35188212	35188452	chr10:35188199-35188423

chr10	35195132	35195242	chr10:35195126-35195214
chr10	35195877	35196168	chr10:35195864-35196142
chr10	35196402	35196546	chr10:35196398-35196539
chr10	35201452	35201526	chr10:35201450-35201486
chr10	35206907	35207087	chr10:35206894-35207051
chr10	35211262	35212985	chr10:35211253-35212958
chr10	38094338	38094508	chr10:38094333-38094490
chr10	38094938	38095247	chr10:38094930-38095224
chr10	38095363	38095646	chr10:38095357-38095633
chr10	38095743	38095819	chr10:38095743-38095804
chr10	38096573	38096648	chr10:38096573-38096632
chr10	38114758	38114839	chr10:38114754-38114881
chr10	38114843	38114923	chr10:38114754-38114881
chr10	38115208	38115549	chr10:38115194-38115693
chr10	38115553	38115725	chr10:38115194-38115693
chr10	38117403	38118204	chr10:38117389-38125544
chr10	38118208	38120128	chr10:38117389-38125544
chr10	38120438	38120863	chr10:38117389-38125544
chr10	38120888	38121201	chr10:38117389-38125544
chr10	38121213	38121358	chr10:38117389-38125544
chr10	38121363	38121707	chr10:38117389-38125544
chr10	38121723	38121976	chr10:38117389-38125544
chr10	38122248	38122343	chr10:38117389-38125544
chr10	38122468	38122573	chr10:38117389-38125544
chr10	38123078	38123220	chr10:38117389-38125544
chr10	38123313	38123560	chr10:38117389-38125544
chr10	38123938	38124012	chr10:38117389-38125544
chr10	38124218	38124399	chr10:38117389-38125544
chr10	38124598	38125576	chr10:38117389-38125544
chr10	38127013	38127222	chr10:38127003-38127203
chr10	38146743	38146876	chr10:38146731-38150293
chr10	38147053	38147129	chr10:38146731-38150293
chr10	38147233	38147708	chr10:38146731-38150293
chr10	38147768	38148828	chr10:38146731-38150293
chr10	38149123	38149376	chr10:38146731-38150293
chr10	38149378	38149577	chr10:38146731-38150293
chr10	38149858	38150320	chr10:38146731-38150293
chr10	43556343	43558198	chr10:43556343-43558171
chr10	43567898	43568494	chr10:43567898-43568459
chr10	43570978	43571183	chr10:43570965-43571165
chr10	43573638	43573707	chr10:43573636-43573677
chr10	43574553	43574651	chr10:43574539-43574618
chr10	45000483	45001008	chr10:45000474-45000978
chr10	45004238	45004679	chr10:45004225-45005326
chr10	45004703	45005294	chr10:45004225-45005326
chr10	49364193	49364674	chr10:49364180-49366381
chr10	49364678	49366394	chr10:49364180-49366381
chr10	49374953	49375161	chr10:49374939-49375139
chr10	49386478	49386626	chr10:49386477-49386590
chr10	49386693	49386896	chr10:49386679-49386858
chr10	49390143	49390247	chr10:49390132-49390234
chr10	49391168	49391270	chr10:49391163-49391261
chr10	49395408	49395557	chr10:49395406-49395521
chr10	49395938	49396053	chr10:49395934-49396016
chr10	52768370	52768534	chr10:52768360-52768510
chr10	52769250	52769329	chr10:52769246-52769315
chr10	58385033	58385672	chr10:58385021-58385648
chr10	58386223	58386371	chr10:58386219-58386338
chr10	58386663	58386737	chr10:58386649-58386716
chr10	58388198	58388269	chr10:58388189-58388260
chr10	58388678	58388814	chr10:58388669-58388819
chr10	58389538	58389745	chr10:58389528-58389728
chr10	58390778	58390877	chr10:58390764-58390860

chr10	58394368	58394569	chr10:58394357-58394539
chr10	58394938	58395797	chr10:58394927-58399221
chr10	58395818	58396062	chr10:58394927-58399221
chr10	58396063	58397523	chr10:58394927-58399221
chr10	58397543	58397748	chr10:58394927-58399221
chr10	58398198	58398428	chr10:58394927-58399221
chr10	58398438	58399238	chr10:58394927-58399221
chr10	61901303	61901765	chr10:61901299-61901730
chr10	61902168	61902444	chr10:61902158-61902413
chr10	61910803	61911019	chr10:61910796-61910996
chr10	61940188	61940428	chr10:61940182-61940408
chr10	62000103	62000338	chr10:62000090-62000321
chr10	62049223	62049513	chr10:62049210-62049483
chr10	62050898	62051037	chr10:62050887-62051000
chr10	62057118	62057333	chr10:62057116-62057318
chr10	62059243	62059315	chr10:62059242-62059295
chr10	62069713	62069825	chr10:62069699-62069797
chr10	62085708	62085846	chr10:62085701-62085900
chr10	62085863	62085939	chr10:62085701-62085900
chr10	62090863	62093660	chr10:62090861-62096944
chr10	62093728	62095093	chr10:62090861-62096944
chr10	62095118	62095369	chr10:62090861-62096944
chr10	62095373	62096979	chr10:62090861-62096944
chr10	62812007	62813699	chr10:62811995-62814468
chr10	62813727	62814507	chr10:62811995-62814468
chr10	62815867	62816177	chr10:62815860-62816366
chr10	62816182	62816394	chr10:62815860-62816366
chr10	62818552	62818680	chr10:62818540-62818647
chr10	62819112	62819189	chr10:62819105-62819167
chr10	62893512	62893585	chr10:62893506-62893550
chr10	62894462	62894575	chr10:62894456-62894537
chr10	62899547	62899753	chr10:62899535-62899735
chr10	62901602	62901739	chr10:62901594-62901720
chr10	62916967	62917082	chr10:62916960-62917057
chr10	62919102	62919249	chr10:62919100-62919210
chr10	62919827	62919928	chr10:62919817-62919900
chr10	68230635	68232145	chr10:68230623-68232103
chr10	69571709	69572410	chr10:69571697-69573044
chr10	69572429	69573061	chr10:69571697-69573044
chr10	86756614	86756945	chr10:86756600-86756919
chr10	86795724	86795945	chr10:86795720-86795920
chr10	86838874	86839006	chr10:86838861-86838979
chr10	86843409	86843526	chr10:86843405-86843510
chr10	86875869	86876119	chr10:86875866-86876085
chr10	86890074	86890258	chr10:86890061-86890224
chr10	86892134	86892240	chr10:86892126-86892229
chr10	86899799	86899909	chr10:86899793-86899890
chr10	86900034	86900147	chr10:86900026-86900126
chr10	86912244	86912414	chr10:86912239-86912384
chr10	86917139	86917351	chr10:86917133-86917326
chr10	86919174	86919504	chr10:86919171-86919469
chr10	86921589	86921724	chr10:86921519-86921695
chr10	86923379	86923525	chr10:86923375-86923506
chr10	86923604	86925726	chr10:86923593-86932838
chr10	86925989	86926225	chr10:86923593-86932838
chr10	86926544	86928209	chr10:86923593-86932838
chr10	86928514	86928665	chr10:86923593-86932838
chr10	86928944	86930282	chr10:86923593-86932838
chr10	86930564	86930743	chr10:86923593-86932838
chr10	86930884	86930963	chr10:86923593-86932838
chr10	86931309	86931802	chr10:86923593-86932838
chr10	86931854	86932066	chr10:86923593-86932838
chr10	86932114	86932387	chr10:86923593-86932838

chr10	86932529	86932877	chr10:86923593-86932838
chr10	92689954	92690372	chr10:92689950-92690347
chr10	92691819	92692566	chr10:92691819-92692546
chr10	92692704	92692775	chr10:92692701-92692752
chr10	92693554	92693768	chr10:92693549-92693749
chr10	92694559	92695677	chr10:92694546-92695646
chr10	99532941	99533500	chr10:99532932-99533489
chr10	99534146	99534359	chr10:99534137-99534337
chr10	99534996	99535129	chr10:99534984-99536524
chr10	99535131	99535444	chr10:99534984-99536524
chr10	99535531	99536432	chr10:99534984-99536524
chr10	99536436	99536545	chr10:99534984-99536524
chr10	100735616	100735760	chr10:100735602-100735733
chr10	100745721	100746325	chr10:100745710-100746303
chr10	100748856	100749953	chr10:100748849-100749914
chr10	100750706	100750926	chr10:100750693-100750891
chr10	100753761	100753973	chr10:100753747-100753947
chr10	100779501	100779618	chr10:100779497-100779583
chr10	100781246	100781388	chr10:100781245-100781365
chr10	100786946	100787023	chr10:100786942-100787011
chr10	100806441	100806614	chr10:100806429-100806605
chr10	100809111	100809256	chr10:100809109-100809236
chr10	100824651	100824773	chr10:100824647-100824749
chr10	100824911	100825022	chr10:100824901-100824984
chr10	100827011	100827119	chr10:100827008-100827095
chr10	100827551	100829096	chr10:100827542-100829941
chr10	100829106	100829235	chr10:100827542-100829941
chr10	100829271	100829907	chr10:100827542-100829941
chr10	101130516	101131181	chr10:101130504-101132109
chr10	101131191	101131869	chr10:101130504-101132109
chr10	101131881	101132130	chr10:101130504-101132109
chr10	101132786	101133101	chr10:101132781-101133074
chr10	101134146	101134389	chr10:101134138-101134376
chr10	101135356	101135527	chr10:101135353-101135516
chr10	101136676	101137165	chr10:101136667-101137789
chr10	101137181	101137767	chr10:101136667-101137789
chr10	101226196	101226548	chr10:101226194-101227790
chr10	101226561	101226944	chr10:101226194-101227790
chr10	101226946	101227825	chr10:101226194-101227790
chr10	101228501	101229374	chr10:101228490-101229794
chr10	101229421	101229797	chr10:101228490-101229794
chr10	102230197	102230681	chr10:102230185-102231101
chr10	102230687	102231119	chr10:102230185-102231101
chr10	102231597	102231798	chr10:102231587-102231790
chr10	102231967	102232120	chr10:102231962-102232092
chr10	102234287	102234506	chr10:102234285-102234485
chr10	102241332	102241511	chr10:102241332-102241474
chr10	102394112	102394789	chr10:102394109-102394774
chr10	102395692	102396014	chr10:102395692-102395980
chr10	102396252	102396368	chr10:102396252-102396334
chr10	102396457	102396532	chr10:102396448-102396489
chr10	102396737	102396837	chr10:102396724-102396823
chr10	102396907	102397092	chr10:102396903-102397055
chr10	102397302	102397443	chr10:102397301-102397408
chr10	102397537	102397720	chr10:102397526-102397685
chr10	102397992	102398106	chr10:102397980-102398085
chr10	102398212	102398320	chr10:102398211-102398297
chr10	102398387	102398530	chr10:102398384-102398523
chr10	102398742	102398884	chr10:102398738-102398864
chr10	102399297	102399508	chr10:102399287-102399497
chr10	102399577	102399756	chr10:102399576-102399718
chr10	102399932	102400073	chr10:102399926-102400061
chr10	102400087	102400223	chr10:102400079-102400194

chr10	102400287	102400532	chr10:102400277-102400491
chr10	102400657	102400846	chr10:102400654-102400824
chr10	102400947	102401067	chr10:102400946-102401049
chr10	102401187	102401364	chr10:102401179-102401331
chr10	102401452	102401535	chr10:102401448-102401518
chr10	102401747	102401934	chr10:102401744-102401917
chr10	102402057	102402192	chr10:102402047-102402159
chr10	102402252	102402495	chr10:102402251-102402466
chr10	103302801	103303971	chr10:103302795-103303961
chr10	103314196	103314302	chr10:103314185-103314272
chr10	103320866	103321064	chr10:103320853-103321053
chr10	103326541	103326651	chr10:103326533-103326632
chr10	103345031	103345167	chr10:103345023-103345132
chr10	103347241	103347345	chr10:103347237-103347319
chr10	103347396	103347469	chr10:103347394-103347450
chr10	103348726	103348832	chr10:103348715-103348812
chr10	103348906	103349018	chr10:103348899-103348999
chr10	103350706	103350781	chr10:103350706-103351134
chr10	103350786	103351146	chr10:103350706-103351134
chr10	103603651	103603825	chr10:103603639-103603789
chr10	103605806	103605911	chr10:103605797-103605897
chr10	103732536	103732749	chr10:103732531-103732731
chr10	110207605	110208102	chr10:110207604-110208082
chr10	110210240	110210345	chr10:110210230-110210315
chr10	110225995	110226323	chr10:110225992-110226283
chr10	110227550	110228358	chr10:110227540-110228321
chr10	110244825	110244902	chr10:110244827-110244873
chr10	110245745	110245821	chr10:110245742-110245818
chr10	110259100	110259310	chr10:110259088-110259288
chr10	110279185	110279329	chr10:110279179-110279294
chr10	110279925	110280108	chr10:110279913-110280085
chr10	110284825	110285082	chr10:110284823-110287365
chr10	110285100	110285525	chr10:110284823-110287365
chr10	110285535	110286497	chr10:110284823-110287365
chr10	110286505	110287397	chr10:110284823-110287365
chr10	112950250	112950456	chr10:112950249-112950945
chr10	112950490	112950563	chr10:112950249-112950945
chr10	112950575	112950956	chr10:112950249-112950945
chr10	112951210	112951274	chr10:112951206-112951273
chr10	112951490	112951624	chr10:112951482-112951607
chr10	112964560	112964637	chr10:112964555-112964624
chr10	113040025	113040145	chr10:113040024-113040126
chr10	113089410	113089580	chr10:113089396-113089540
chr10	113122995	113123210	chr10:113122985-113123185
chr10	113125540	113125961	chr10:113125535-113125938
chr10	113126035	113126106	chr10:113126021-113126102
chr10	113126690	113126904	chr10:113126689-113126881
chr10	113126970	113127081	chr10:113126956-113127053
chr10	113129210	113129488	chr10:113129205-113129987
chr10	113129565	113129984	chr10:113129205-113129987
chr10	113132025	113132136	chr10:113132021-113132101
chr10	113132950	113133047	chr10:113132895-113133020
chr10	113133245	113133316	chr10:113133239-113133289
chr10	113141185	113141322	chr10:113141183-113141316
chr10	113143925	113144032	chr10:113143922-113144025
chr10	113146015	113146128	chr10:113146010-113146097
chr10	113150995	113151142	chr10:113150982-113151123
chr10	113151750	113151915	chr10:113151724-113151884
chr10	113152345	113152476	chr10:113152332-113152440
chr10	113158030	113158104	chr10:113158020-113158069
chr10	113158670	113158744	chr10:113158666-113158717
chr10	113159930	113160035	chr10:113159919-113159992
chr10	113160625	113160730	chr10:113160618-113160691

chr10	113165565	113166234	chr10:113165554-113167678
chr10	113166315	113166452	chr10:113165554-113167678
chr10	113166465	113166769	chr10:113165554-113167678
chr10	113166820	113167344	chr10:113165554-113167678
chr10	113167355	113167704	chr10:113165554-113167678
chr10	117128528	117129648	chr10:117128520-117132302
chr10	117129668	117129855	chr10:117128520-117132302
chr10	117129863	117131147	chr10:117128520-117132302
chr10	117131158	117132281	chr10:117128520-117132302
chr10	117133298	117133818	chr10:117133289-117134583
chr10	117133823	117134313	chr10:117133289-117134583
chr10	117134323	117134595	chr10:117133289-117134583
chr10	117135673	117135893	chr10:117135672-117135872
chr10	117136478	117136699	chr10:117136471-117136659
chr10	117137818	117138071	chr10:117137815-117138301
chr10	117138143	117138327	chr10:117137815-117138301
chr10	117542454	117542833	chr10:117542443-117543673
chr10	117542834	117542924	chr10:117542443-117543673
chr10	117542964	117543059	chr10:117542443-117543673
chr10	117543139	117543420	chr10:117542443-117543673
chr10	117543439	117543693	chr10:117542443-117543673
chr10	117544879	117545147	chr10:117544865-117545230
chr10	117545154	117545253	chr10:117544865-117545230
chr10	117545639	117545852	chr10:117545631-117545816
chr10	117546854	117547061	chr10:117546840-117547040
chr10	117548074	117548523	chr10:117548064-117549546
chr10	117548564	117548898	chr10:117548064-117549546
chr10	117548934	117549565	chr10:117548064-117549546
chr10	122990819	122991055	chr10:122990805-122994492
chr10	122991059	122992408	chr10:122990805-122994492
chr10	122992414	122993714	chr10:122990805-122994492
chr10	122993734	122994499	chr10:122990805-122994492
chr10	122995999	122996214	chr10:122995993-122996176
chr10	122998234	122998680	chr10:122998226-122998671
chr10	123004029	123004253	chr10:123004023-123004223
chr10	123007039	123007209	chr10:123007025-123007176
chr10	123008574	123008843	chr10:123008574-123008687;chr10:123008693-123008817
chr10	123136354	123136464	chr10:123136350-123136450
chr10	123137064	123137237	chr10:123137057-123137207
chr10	124445252	124447604	chr10:124445238-124448147
chr10	124447622	124448184	chr10:124445238-124448147
chr10	124449737	124450198	chr10:124449729-124450184
chr10	124984322	124984503	chr10:124984316-124984623
chr10	124984512	124984651	chr10:124984316-124984623
chr10	124988457	124988549	chr10:124988339-124989698
chr10	124988562	124988637	chr10:124988339-124989698
chr10	124988652	124988724	chr10:124988339-124989698
chr10	124988732	124988830	chr10:124988339-124989698
chr10	124988892	124989391	chr10:124988339-124989698
chr10	124989442	124989548	chr10:124988339-124989698
chr10	124989567	124989644	chr10:124988339-124989698
chr10	124989652	124989729	chr10:124988339-124989698
chr10	124992777	124992848	chr10:124992694-124992812
chr10	124993287	124993360	chr10:124993201-124993329
chr10	124993857	124993934	chr10:124993854-124993985
chr10	124994552	124994724	chr10:124994468-124994683
chr10	124997967	124998083	chr10:124997963-124998170
chr10	124998092	124998205	chr10:124997963-124998170
chr10	125003057	125003132	chr10:125002959-125003104
chr10	125003407	125003507	chr10:125003337-125003492
chr10	125005547	125006063	chr10:125005546-125006013
chr10	125026082	125027895	chr10:125026081-125027890
chr10	125027957	125028064	chr10:125027957-125028028

chr10	125039082	125039194	chr10:125038996-125039155
chr10	125057277	125057485	chr10:125057265-125057465
chr10	125088202	125088332	chr10:125088190-125088319
chr10	125100587	125100811	chr10:125100586-125100772
chr10	125111022	125111091	chr10:125110989-125111093
chr10	125122667	125122906	chr10:125122656-125122868
chr10	125133522	125133632	chr10:125133511-125133612
chr10	125138007	125138129	chr10:125138006-125138104
chr10	125138217	125138294	chr10:125138210-125138250
chr10	125158647	125158746	chr10:125158634-125158716
chr10	125159972	125160174	chr10:125159856-125160145
chr10	125160367	125160550	chr10:125160318-125160520
chr10	125160837	125161207	chr10:125160826-125161170
chr10	127737242	127737732	chr10:127737234-127737784
chr10	127737747	127737821	chr10:127737234-127737784
chr10	127738527	127739784	chr10:127738519-127741186
chr10	127740207	127740785	chr10:127738519-127741186
chr10	127740807	127741227	chr10:127738519-127741186
chr10	128099107	128099272	chr10:128099105-128099255
chr10	128100157	128100370	chr10:128100156-128100356
chr10	128101267	128101377	chr10:128101257-128101357
chr10	129835287	129835851	chr10:129835282-129837960
chr10	129835862	129836119	chr10:129835282-129837960
chr10	129836132	129836377	chr10:129835282-129837960
chr10	129836402	129836562	chr10:129835282-129837960
chr10	129836602	129837368	chr10:129835282-129837960
chr10	129837377	129837973	chr10:129835282-129837960
chr10	129839092	129839193	chr10:129839082-129839195
chr10	129840247	129840461	chr10:129840244-129840442
chr10	129840857	129841066	chr10:129840843-129841032
chr10	129842117	129842337	chr10:129842115-129842293
chr10	129843137	129843208	chr10:129843136-129843202
chr10	129848402	129848514	chr10:129848391-129848480
chr10	129862202	129862408	chr10:129862188-129862388
chr10	129867147	129867291	chr10:129867140-129867267
chr10	129867787	129867942	chr10:129867781-129867912
chr10	129873457	129873633	chr10:129873451-129873596
chr10	129877767	129877844	chr10:129877767-129877849
chr10	129957262	129957337	chr10:129957257-129957326
chr10	129958937	129959046	chr10:129958933-129959007
chr10	129962182	129962265	chr10:129962170-129962226
chr10	129962942	129963014	chr10:129962941-129963005
chr10	129963372	129963547	chr10:129963366-129963523
chr10	129963647	129963862	chr10:129963634-129963841
chr10	132783630	132783948	chr10:132783630-132785170
chr10	132783950	132785204	chr10:132783630-132785170
chr10	132785290	132785460	chr10:132785279-132785452
chr10	132785545	132786069	chr10:132785542-132786052
chr10	133237415	133237977	chr10:133237403-133238155
chr10	133237995	133238095	chr10:133237403-133238155
chr10	133238100	133238170	chr10:133237403-133238155
chr10	133238835	133239052	chr10:133238815-133239015
chr10	133239680	133239752	chr10:133239675-133239836
chr10	133239760	133239868	chr10:133239675-133239836
chr10	133239935	133240429	chr10:133239931-133241929
chr10	133240830	133241952	chr10:133239931-133241929
chr11	314270	314347	chr11:314256-314356
chr11	314995	315099	chr11:314921-315071
chr11	612565	612842	chr11:612552-612800
chr11	613005	613160	chr11:612998-613117
chr11	613205	613633	chr11:613205-613595
chr11	613795	614066	chr11:613784-614037
chr11	614175	614435	chr11:614173-614399

chr11	614475	614553	chr11:614475-614534
chr11	614790	615727	chr11:614782-615728
chr11	615915	616028	chr11:615912-615999
chr11	644240	644667	chr11:644232-644654
chr11	646330	646634	chr11:646326-646596
chr11	647740	647975	chr11:647739-647939
chr11	651245	651319	chr11:651161-651407
chr11	653945	654090	chr11:653940-654051
chr11	674545	674817	chr11:674535-674783
chr11	678695	678841	chr11:678693-678822
chr11	679700	679837	chr11:679687-679816
chr11	679990	680096	chr11:679977-680074
chr11	680975	681108	chr11:680962-681089
chr11	684910	685012	chr11:684897-684971
chr11	686860	687007	chr11:686857-686997
chr11	687920	688087	chr11:687910-688057
chr11	688340	688471	chr11:688330-688460
chr11	689670	689843	chr11:689662-689813
chr11	691500	691626	chr11:691500-691598
chr11	692260	692364	chr11:692249-692350
chr11	693495	693666	chr11:693488-693644
chr11	694685	695314	chr11:694678-695277
chr11	697485	698020	chr11:697485-697999
chr11	1199870	1199987	chr11:1199869-1199969
chr11	1200445	1200623	chr11:1200437-1200587
chr11	2268495	2269161	chr11:2268494-2269126
chr11	2269735	2270958	chr11:2269732-2270952
chr11	6926406	6926899	chr11:6926403-6926861
chr11	6927671	6927838	chr11:6927661-6927807
chr11	6932101	6932698	chr11:6932093-6932672
chr11	6941581	6941694	chr11:6941570-6941653
chr11	6943091	6943229	chr11:6943082-6943215
chr11	6943546	6943659	chr11:6943545-6943641
chr11	6955696	6956194	chr11:6955689-6957848
chr11	6956201	6957192	chr11:6955689-6957848
chr11	6957201	6957680	chr11:6955689-6957848
chr11	6957696	6957877	chr11:6955689-6957848
chr11	6980086	6980223	chr11:6980072-6980272
chr11	6984396	6984669	chr11:6984128-6984632
chr11	6988226	6988890	chr11:6988215-6988930
chr11	6999321	7000174	chr11:6999317-7001555
chr11	7000196	7000530	chr11:6999317-7001555
chr11	7000531	7001586	chr11:6999317-7001555
chr11	7002711	7002891	chr11:7002708-7002855
chr11	7004001	7004072	chr11:7004000-7004200
chr11	7004106	7004198	chr11:7004000-7004200
chr11	7015831	7016036	chr11:7015819-7016024
chr11	7019696	7019769	chr11:7019682-7019745
chr11	7020076	7020326	chr11:7020072-7020295
chr11	8937589	8938253	chr11:8937578-8938211
chr11	9460329	9460471	chr11:9460318-9460458
chr11	9460529	9460886	chr11:9460521-9460855
chr11	9460974	9461104	chr11:9460964-9461076
chr11	9461654	9461982	chr11:9461647-9461940
chr11	9461999	9462069	chr11:9462001-9462040
chr11	9463829	9463901	chr11:9463770-9463870
chr11	9471309	9471460	chr11:9471301-9471420
chr11	9472689	9472796	chr11:9472676-9472769
chr11	9473779	9473888	chr11:9473776-9473849
chr11	9473944	9474051	chr11:9473940-9474024
chr11	9474559	9474656	chr11:9474549-9474633
chr11	9478389	9478620	chr11:9478389-9478586
chr11	9479479	9479581	chr11:9479471-9479546

chr11	9489119	9489338	chr11:9489113-9489313
chr11	9494654	9494801	chr11:9494645-9494765
chr11	9496314	9496396	chr11:9496302-9496378
chr11	9497674	9497830	chr11:9497674-9497800
chr11	9501099	9501307	chr11:9501090-9501270
chr11	9508624	9508873	chr11:9508618-9508846
chr11	9512459	9512628	chr11:9512447-9512596
chr11	9516204	9516380	chr11:9516200-9516362
chr11	9525244	9525498	chr11:9525239-9525479
chr11	9527534	9528317	chr11:9527529-9528524
chr11	9528319	9528513	chr11:9527529-9528524
chr11	10511689	10512245	chr11:10511677-10515066
chr11	10512254	10512769	chr11:10511677-10515066
chr11	10512774	10513675	chr11:10511677-10515066
chr11	10513959	10514406	chr11:10511677-10515066
chr11	10514424	10515088	chr11:10511677-10515066
chr11	10518329	10518406	chr11:10518317-10519141
chr11	10518514	10519181	chr11:10518317-10519141
chr11	10525199	10525385	chr11:10525191-10525373
chr11	10530644	10530785	chr11:10530642-10530751
chr11	10531829	10532053	chr11:10531826-10532015
chr11	10534024	10534232	chr11:10534015-10534205
chr11	10539404	10539579	chr11:10539402-10539602
chr11	10540759	10540937	chr11:10540752-10540906
chr11	10541124	10541264	chr11:10541121-10541230
chr11	10559154	10559331	chr11:10559147-10559297
chr11	10559824	10559936	chr11:10559815-10559894
chr11	10590079	10590254	chr11:10590071-10590271
chr11	12674604	12674846	chr11:12674590-12674834
chr11	12675414	12675577	chr11:12675408-12675561
chr11	12677329	12677542	chr11:12677329-12677529
chr11	12764182	12764477	chr11:12764178-12764437
chr11	12862257	12862331	chr11:12862249-12862314
chr11	12864837	12864913	chr11:12864837-12864900
chr11	12879447	12879877	chr11:12879442-12879842
chr11	12881012	12881087	chr11:12881004-12881051
chr11	12881897	12881974	chr11:12881895-12881957
chr11	12883012	12883162	chr11:12883000-12883125
chr11	12901952	12902120	chr11:12901939-12902113
chr11	12924922	12925088	chr11:12924911-12925052
chr11	12930182	12930359	chr11:12930173-12930326
chr11	12937117	12937263	chr11:12937108-12944483
chr11	12937282	12937391	chr11:12937108-12944483
chr11	12937392	12937668	chr11:12937108-12944483
chr11	12937692	12937784	chr11:12937108-12944483
chr11	12937807	12937969	chr11:12937108-12944483
chr11	12937992	12940231	chr11:12937108-12944483
chr11	12940252	12943191	chr11:12937108-12944483
chr11	12943192	12943912	chr11:12937108-12944483
chr11	12943947	12944465	chr11:12937108-12944483
chr11	13276662	13277114	chr11:13276651-13277072
chr11	13277792	13277862	chr11:13277792-13277923
chr11	13277867	13277940	chr11:13277792-13277923
chr11	13309992	13310091	chr11:13309985-13310059
chr11	13326417	13326499	chr11:13326412-13326467
chr11	13348542	13348755	chr11:13348530-13348730
chr11	13349952	13350092	chr11:13349944-13350070
chr11	13354307	13354483	chr11:13354300-13354448
chr11	13355237	13355318	chr11:13355225-13355296
chr11	13356742	13356821	chr11:13356738-13356777
chr11	13356887	13357128	chr11:13356877-13357096
chr11	13358437	13358617	chr11:13358433-13358591
chr11	13360347	13360455	chr11:13360344-13360424

chr11	13365512	13365617	chr11:13365499-13365592
chr11	13366667	13366816	chr11:13366664-13366782
chr11	13369622	13369797	chr11:13369617-13369767
chr11	13372172	13372446	chr11:13372162-13372409
chr11	13374092	13374242	chr11:13374092-13374201
chr11	13375622	13375790	chr11:13375611-13375762
chr11	13376557	13376771	chr11:13376544-13376728
chr11	13378342	13378482	chr11:13378336-13378445
chr11	13381162	13381279	chr11:13381160-13381254
chr11	13385702	13385813	chr11:13385688-13385791
chr11	13386607	13387294	chr11:13386595-13387266
chr11	13492057	13492676	chr11:13492054-13492666
chr11	13492772	13492886	chr11:13492769-13492860
chr11	13494387	13494590	chr11:13494382-13494582
chr11	13495922	13496191	chr11:13495910-13496020;chr11:13496041-13496181
chr11	15966462	15966679	chr11:15966448-15973112
chr11	15966682	15966920	chr11:15966448-15973112
chr11	15966967	15969007	chr11:15966448-15973112
chr11	15969097	15970228	chr11:15966448-15973112
chr11	15970242	15970338	chr11:15966448-15973112
chr11	15970342	15972479	chr11:15966448-15973112
chr11	15972497	15972595	chr11:15966448-15973112
chr11	15972627	15973149	chr11:15966448-15973112
chr11	15986212	15986454	chr11:15986203-15986420
chr11	15988997	15989252	chr11:15988996-15989230
chr11	16014947	16015082	chr11:16014941-16015050
chr11	16015262	16015367	chr11:16015252-16015327
chr11	16046517	16046738	chr11:16046513-16046701
chr11	16049717	16049966	chr11:16049712-16049938
chr11	16055752	16055950	chr11:16055751-16055901
chr11	16096002	16096137	chr11:16095995-16096118
chr11	16097617	16097729	chr11:16097608-16097688
chr11	16110327	16110402	chr11:16110319-16110399
chr11	16111807	16111963	chr11:16111802-16111923
chr11	16183887	16183971	chr11:16183885-16183954
chr11	16186782	16186965	chr11:16186780-16186955
chr11	16234587	16234695	chr11:16234581-16234671
chr11	16252377	16252545	chr11:16252372-16252536
chr11	16264462	16264782	chr11:16264448-16264846
chr11	16264787	16264857	chr11:16264448-16264846
chr11	16269952	16270101	chr11:16269943-16270068
chr11	16283757	16283931	chr11:16283755-16283918
chr11	16295997	16296214	chr11:16295988-16296188
chr11	16318052	16318117	chr11:16318025-16318653
chr11	16318132	16318372	chr11:16318025-16318653
chr11	16318387	16318659	chr11:16318025-16318653
chr11	16341022	16341295	chr11:16341010-16341269
chr11	16382287	16382431	chr11:16382287-16382398
chr11	16397457	16397575	chr11:16397457-16397562
chr11	16402672	16402885	chr11:16402658-16402863
chr11	16408822	16408893	chr11:16408697-16408884
chr11	16434147	16434478	chr11:16434143-16434450
chr11	16465702	16465821	chr11:16465700-16465816
chr11	16476327	16476400	chr11:16476314-16476388
chr11	16605912	16606018	chr11:16605900-16605991
chr11	16607177	16607429	chr11:16607177-16607727
chr11	16607442	16607758	chr11:16607177-16607727
chr11	16612082	16612233	chr11:16612080-16612260
chr11	16714842	16714932	chr11:16714829-16714905
chr11	16736342	16736486	chr11:16736338-16736472
chr11	16739467	16739612	chr11:16739458-16739591
chr11	17719572	17720447	chr11:17719567-17720412
chr11	17720902	17721009	chr11:17720901-17720980

chr11	17721257	17722135	chr11:17721254-17722131
chr11	19224102	19224477	chr11:19224062-19224840
chr11	19224512	19224851	chr11:19224062-19224840
chr11	19225232	19225652	chr11:19225220-19225615
chr11	19225732	19226090	chr11:19225731-19226078
chr11	19228412	19228632	chr11:19228409-19228609
chr11	19229467	19230029	chr11:19229453-19229988
chr11	19230242	19230361	chr11:19230240-19230328
chr11	19230632	19230846	chr11:19230630-19230834
chr11	19232247	19232381	chr11:19232233-19232371
chr11	19234372	19234547	chr11:19234359-19234521
chr11	19234757	19235068	chr11:19234743-19235058
chr11	19237322	19237497	chr11:19237313-19237470
chr11	19237857	19238137	chr11:19237853-19238132
chr11	19240107	19240243	chr11:19240106-19240230
chr11	19240547	19240790	chr11:19240547-19240951
chr11	19240797	19240964	chr11:19240547-19240951
chr11	19241407	19241653	chr11:19241406-19241620
chr11	20156162	20156610	chr11:20156154-20156573
chr11	20157042	20157250	chr11:20157036-20157239
chr11	20157822	20157891	chr11:20157821-20158021
chr11	20157897	20158042	chr11:20157821-20158021
chr11	20159192	20159296	chr11:20159190-20159292
chr11	20159957	20160640	chr11:20159957-20160613
chr11	20363697	20364464	chr11:20363684-20364432
chr11	20367177	20367676	chr11:20367173-20367655
chr11	20373007	20373227	chr11:20373007-20373207
chr11	20376582	20376735	chr11:20376579-20376717
chr11	20382177	20382246	chr11:20382177-20382239
chr11	20382982	20383801	chr11:20382979-20383783
chr11	22340081	22340302	chr11:22340078-22340278
chr11	22376581	22376689	chr11:22376572-22376672
chr11	22377406	22377583	chr11:22377404-22377554
chr11	28021136	28021321	chr11:28021132-28021282
chr11	28023751	28023860	chr11:28023740-28023840
chr11	28050451	28050659	chr11:28050441-28050641
chr11	31790713	31790901	chr11:31790709-31790860
chr11	31793233	31793589	chr11:31793219-31793553
chr11	31793663	31793836	chr11:31793651-31793802
chr11	31794038	31794150	chr11:31794031-31794114
chr11	31794643	31794821	chr11:31794629-31794788
chr11	31796513	31796720	chr11:31796503-31796703
chr11	31800538	31800690	chr11:31800534-31800667
chr11	31800703	31800874	chr11:31800690-31800856
chr11	31801238	31801801	chr11:31801229-31801776
chr11	31801878	31801957	chr11:31801870-31801912
chr11	31802703	31802882	chr11:31802703-31804415
chr11	31802958	31803756	chr11:31802703-31804415
chr11	31803758	31803992	chr11:31802703-31804415
chr11	31804013	31804425	chr11:31802703-31804415
chr11	31804463	31804637	chr11:31804451-31804619
chr11	31805393	31806013	chr11:31805388-31806013
chr11	31806413	31806665	chr11:31806401-31806629
chr11	31806848	31806959	chr11:31806848-31806925
chr11	31807293	31808148	chr11:31807289-31808802
chr11	31808153	31808814	chr11:31807289-31808802
chr11	31809318	31809415	chr11:31809304-31809401
chr11	31809748	31810675	chr11:31809745-31810667
chr11	31810828	31811712	chr11:31810827-31811689
chr11	31811818	31811948	chr11:31811807-31811952
chr11	31812103	31812263	chr11:31812092-31812240
chr11	31812648	31813145	chr11:31812647-31813131
chr11	31814373	31814978	chr11:31814367-31814976

chr11	31817808	31818090	chr11:31817808-31818062
chr11	32387787	32387987	chr11:32387774-32389179
chr11	32388032	32389183	chr11:32387774-32389179
chr11	32391972	32392077	chr11:32391971-32392064
chr11	32392677	32392773	chr11:32392665-32392755
chr11	32396257	32396444	chr11:32396256-32396407
chr11	32399957	32400061	chr11:32399947-32400044
chr11	32400147	32400646	chr11:32400145-32400615
chr11	32416492	32416569	chr11:32416489-32416540
chr11	32417432	32417681	chr11:32417423-32417654
chr11	32422042	32422252	chr11:32422030-32422230
chr11	32427957	32428079	chr11:32427955-32428058
chr11	32428507	32428646	chr11:32428496-32428619
chr11	32428952	32429067	chr11:32428945-32429029
chr11	32430542	32430844	chr11:32430529-32430813
chr11	32434702	32435415	chr11:32434699-32435587
chr11	32435422	32435623	chr11:32434699-32435587
chr11	33858577	33859595	chr11:33858575-33859575
chr11	33864602	33864958	chr11:33864601-33864929
chr11	33869347	33869487	chr11:33869345-33869987
chr11	33869492	33869659	chr11:33869345-33869987
chr11	33869687	33870001	chr11:33869345-33869987
chr11	33870347	33870572	chr11:33870344-33870529
chr11	33881827	33881903	chr11:33881823-33881887
chr11	33884767	33884990	chr11:33884765-33884965
chr11	33891802	33892325	chr11:33891794-33892289
chr11	34478792	34479565	chr11:34478792-34480314
chr11	34479702	34480120	chr11:34478792-34480314
chr11	34480132	34480332	chr11:34478792-34480314
chr11	34480777	34480984	chr11:34480771-34480967
chr11	34482432	34482513	chr11:34482429-34482499
chr11	34490012	34490081	chr11:34490008-34490059
chr11	34491892	34493240	chr11:34491596-34493712
chr11	34493262	34493751	chr11:34491596-34493712
chr11	34496262	34496480	chr11:34496262-34496462
chr11	34505642	34505781	chr11:34505628-34505753
chr11	34511557	34511863	chr11:34511543-34511828
chr11	34513687	34513820	chr11:34513676-34513805
chr11	34621092	34621242	chr11:34621092-34621228
chr11	34622062	34622453	chr11:34622052-34622189;chr11:34622202-34622416
chr11	34624262	34624333	chr11:34624259-34624317
chr11	34632472	34632680	chr11:34632463-34632652
chr11	34642007	34642283	chr11:34642007-34642165;chr11:34642176-34642348
chr11	34642302	34642375	chr11:34642176-34642348
chr11	34642397	34642497	chr11:34642384-34642463
chr11	34642627	34642747	chr11:34642626-34642727
chr11	34646452	34646901	chr11:34646438-34646894
chr11	34649027	34649104	chr11:34649018-34649081
chr11	34650212	34650421	chr11:34650211-34650411
chr11	34651547	34651622	chr11:34651541-34651610
chr11	34651737	34651813	chr11:34651736-34651805
chr11	34656917	34656993	chr11:34656907-34656970
chr11	34658537	34658757	chr11:34658532-34658728
chr11	34658842	34660434	chr11:34658831-34661057
chr11	34660472	34661102	chr11:34658831-34661057
chr11	44260448	44260722	chr11:44260443-44265183
chr11	44260758	44265199	chr11:44260443-44265183
chr11	44267498	44267641	chr11:44267493-44267622
chr11	44275358	44275677	chr11:44275347-44275658
chr11	44303193	44303407	chr11:44303179-44303379
chr11	44309648	44309724	chr11:44309596-44310166
chr11	44309733	44310196	chr11:44309596-44310166
chr11	46277661	46278233	chr11:46277660-46278213

chr11	46293701	46293928	chr11:46293693-46293893
chr11	46295136	46295343	chr11:46295125-46295333
chr11	46299946	46300194	chr11:46299934-46300163
chr11	46307821	46308035	chr11:46307815-46308000
chr11	46309996	46310095	chr11:46309988-46310067
chr11	46311031	46311220	chr11:46311031-46311189
chr11	46312326	46312502	chr11:46312324-46312474
chr11	46312616	46312686	chr11:46312611-46312670
chr11	46312851	46312927	chr11:46312850-46312919
chr11	46315171	46315429	chr11:46315170-46315387
chr11	46315481	46315558	chr11:46315478-46316080
chr11	46315841	46316123	chr11:46315478-46316080
chr11	46316296	46316398	chr11:46316285-46316385
chr11	46317371	46317510	chr11:46317360-46317487
chr11	46320266	46320545	chr11:46320263-46320528
chr11	46320721	46321393	chr11:46320709-46321422
chr11	47248296	47248369	chr11:47248299-47248334
chr11	47250466	47250576	chr11:47250465-47250551
chr11	47254471	47254687	chr11:47254459-47254659
chr11	47257606	47257748	chr11:47257603-47257735
chr11	47257916	47258624	chr11:47257916-47259259
chr11	47258636	47258797	chr11:47257916-47259259
chr11	47258831	47258904	chr11:47257916-47259259
chr11	47259006	47259285	chr11:47257916-47259259
chr11	47259361	47259646	chr11:47259360-47259606
chr11	47259786	47260009	chr11:47259785-47259979
chr11	47260396	47260717	chr11:47260396-47260678
chr11	47261241	47261461	chr11:47261240-47261449
chr11	47261556	47262040	chr11:47261546-47262018
chr11	47267921	47268069	chr11:47267912-47268026
chr11	47268266	47268385	chr11:47268260-47268355
chr11	47268551	47269043	chr11:47268549-47269010
chr11	47354861	47355572	chr11:47354859-47355546
chr11	47358186	47358666	chr11:47358180-47359006
chr11	47358791	47359039	chr11:47358180-47359006
chr11	47359856	47360071	chr11:47359852-47360040
chr11	47374316	47374535	chr11:47374310-47374510
chr11	47375646	47375751	chr11:47375632-47375732
chr11	47378321	47378599	chr11:47378308-47378576
chr11	60761240	60761315	chr11:60761213-60761413
chr11	60773850	60773965	chr11:60773850-60773950
chr11	60775610	60775785	chr11:60775604-60775754
chr11	61752655	61752832	chr11:61752648-61752790
chr11	61755390	61755464	chr11:61755388-61755442
chr11	61765630	61765740	chr11:61765624-61765712
chr11	61765970	61766241	chr11:61765957-61766221
chr11	61769260	61769333	chr11:61769259-61769321
chr11	61770245	61770547	chr11:61770245-61770525
chr11	61771510	61771785	chr11:61771499-61771750
chr11	61771840	61771979	chr11:61771828-61771952
chr11	61775065	61775279	chr11:61775065-61775265
chr11	61776065	61776165	chr11:61776055-61776132
chr11	61776335	61776464	chr11:61776321-61776432
chr11	61776790	61776904	chr11:61776786-61776877
chr11	61777265	61777481	chr11:61777263-61777464
chr11	61777605	61777860	chr11:61777605-61777845
chr11	61778390	61778518	chr11:61778379-61778489
chr11	61779270	61779442	chr11:61779262-61779423
chr11	61779500	61779605	chr11:61779497-61779570
chr11	61779850	61779967	chr11:61779841-61779930
chr11	61780225	61780306	chr11:61780221-61780290
chr11	61780715	61780830	chr11:61780711-61780792
chr11	61780965	61781076	chr11:61780956-61781045

chr11	61781145	61781358	chr11:61781137-61781329
chr11	61781585	61781862	chr11:61781572-61781824
chr11	61783500	61783615	chr11:61783497-61783600
chr11	61783855	61783968	chr11:61783850-61783925
chr11	61784290	61784422	chr11:61784279-61784385
chr11	61785800	61785909	chr11:61785799-61785874
chr11	61786070	61788556	chr11:61786062-61788518
chr11	64305571	64305746	chr11:64305571-64305736
chr11	64306226	64306867	chr11:64306226-64306846
chr11	64307181	64307536	chr11:64307167-64307508
chr11	64313951	64314100	chr11:64313950-64314067
chr11	64314241	64314395	chr11:64314238-64314367
chr11	64314751	64314929	chr11:64314740-64314911
chr11	64315026	64315307	chr11:64315000-64315270
chr11	64315721	64316331	chr11:64315706-64316309
chr11	65653606	65654200	chr11:65653595-65655762
chr11	65654206	65655775	chr11:65653595-65655762
chr11	65655866	65655960	chr11:65655854-65655935
chr11	65658286	65658540	chr11:65658286-65658499
chr11	65658731	65658844	chr11:65658717-65658822
chr11	65659666	65659827	chr11:65659665-65659797
chr11	65660136	65660244	chr11:65660123-65660215
chr11	65661686	65661902	chr11:65661686-65661868
chr11	65661946	65662126	chr11:65661936-65662088
chr11	65662186	65662815	chr11:65662178-65662793
chr11	65662836	65663111	chr11:65662825-65663094
chr11	65787021	65787485	chr11:65787021-65787473
chr11	65790131	65790529	chr11:65790128-65790488
chr11	65791506	65791579	chr11:65791493-65791693
chr11	65791586	65791724	chr11:65791493-65791693
chr11	65792331	65792479	chr11:65792317-65792450
chr11	65793721	65794293	chr11:65793708-65794248
chr11	65794551	65794757	chr11:65794537-65794727
chr11	65795056	65795905	chr11:65795045-65797219
chr11	65795946	65797240	chr11:65795045-65797219
chr11	65892051	65893330	chr11:65892048-65893296
chr11	65894021	65894159	chr11:65894013-65894121
chr11	65896811	65897040	chr11:65896808-65897006
chr11	65900246	65900588	chr11:65900240-65900573
chr11	66292001	66292102	chr11:66291988-66292088
chr11	66293256	66293477	chr11:66293256-66293456
chr11	66294331	66294496	chr11:66294321-66294471
chr11	66421006	66421384	chr11:66421003-66421354
chr11	66422131	66422311	chr11:66422119-66422271
chr11	66422451	66422565	chr11:66422450-66422553
chr11	66422686	66422966	chr11:66422673-66422941
chr11	66423126	66423275	chr11:66423122-66423232
chr11	66423591	66423726	chr11:66423577-66423713
chr11	66423846	66425287	chr11:66423834-66425270
chr11	66425971	66426739	chr11:66425960-66426707
chr11	67631302	67631669	chr11:67631302-67631894
chr11	67631682	67631924	chr11:67631302-67631894
chr11	67632317	67632431	chr11:67632317-67632412
chr11	67632607	67632684	chr11:67632602-67632670
chr11	67632952	67633128	chr11:67632947-67633103
chr11	67634192	67634386	chr11:67634188-67634360
chr11	67634822	67634925	chr11:67634815-67634917
chr11	67635002	67635295	chr11:67634995-67635263
chr11	67639467	67639577	chr11:67639465-67639560
chr11	68903997	68904073	chr11:68903985-68904038
chr11	68906082	68906396	chr11:68906068-68906384
chr11	68908147	68908361	chr11:68908144-68908337
chr11	68908547	68908654	chr11:68908533-68908631

chr11	68911447	68911624	chr11:68911439-68911603
chr11	68914822	68915050	chr11:68914822-68915023
chr11	68916492	68916699	chr11:68916479-68916679
chr11	68917747	68917923	chr11:68917735-68917883
chr11	68929182	68929376	chr11:68929182-68929357
chr11	68930097	68930455	chr11:68930090-68930434
chr11	68933312	68933520	chr11:68933298-68933481
chr11	68933692	68933942	chr11:68933681-68933913
chr11	68934477	68934575	chr11:68934463-68934558
chr11	68935312	68935449	chr11:68935298-68935422
chr11	68936242	68937062	chr11:68936236-68937091
chr11	68938182	68938367	chr11:68938181-68938354
chr11	68939547	68940555	chr11:68939533-68940599
chr11	72239256	72240209	chr11:72239255-72240198
chr11	72241111	72241316	chr11:72241101-72241289
chr11	72242071	72242289	chr11:72242069-72242269
chr11	72243791	72244216	chr11:72243787-72244176
chr11	72245586	72245695	chr11:72245579-72245664
chr11	86244557	86245376	chr11:86244543-86245343
chr11	86250307	86250488	chr11:86250295-86250448
chr11	86251212	86251423	chr11:86251198-86251398
chr11	86252157	86252257	chr11:86252147-86252240
chr11	86255232	86255308	chr11:86255221-86255287
chr11	86256397	86256530	chr11:86256386-86256512
chr11	86257522	86257629	chr11:86257514-86257596
chr11	86264177	86264292	chr11:86264171-86264263
chr11	86266087	86266231	chr11:86266082-86266216
chr11	86268467	86268576	chr11:86268455-86268561
chr11	86276142	86277152	chr11:86276131-86277138
chr11	86277917	86278822	chr11:86277917-86278813
chr11	101029627	101029696	chr11:101029623-101039271
chr11	101029702	101031595	chr11:101029623-101039271
chr11	101031602	101031709	chr11:101029623-101039271
chr11	101031822	101037429	chr11:101029623-101039271
chr11	101037432	101039307	chr11:101029623-101039271
chr11	101041957	101042130	chr11:101041944-101042102
chr11	101049942	101050087	chr11:101049928-101050059
chr11	101051427	101051602	chr11:101051423-101051568
chr11	101062457	101062767	chr11:101062446-101062752
chr11	101091767	101091905	chr11:101091759-101091876
chr11	101092777	101092981	chr11:101092767-101092967
chr11	101126007	101126191	chr11:101126006-101126158
chr11	101126992	101127105	chr11:101126990-101127088
chr11	101127437	101127891	chr11:101127433-101130524
chr11	101127897	101129191	chr11:101127433-101130524
chr11	101129202	101130558	chr11:101127433-101130524
chr11	111908824	111908997	chr11:111908817-111908967
chr11	111910309	111910480	chr11:111910299-111910449
chr11	111911534	111911632	chr11:111911523-111911623
chr11	114059604	114059914	chr11:114059592-114059882
chr11	114060254	114060327	chr11:114060256-114060294
chr11	114060519	114060625	chr11:114060506-114060584
chr11	114061374	114061597	chr11:114061370-114061561
chr11	114063214	114064598	chr11:114063210-114064568
chr11	114121824	114121935	chr11:114121817-114121914
chr11	114151634	114151845	chr11:114151623-114151823
chr11	114156344	114156455	chr11:114156336-114156434
chr11	114180774	114180963	chr11:114180765-114180942
chr11	114186959	114187066	chr11:114186951-114187038
chr11	114237209	114237358	chr11:114237206-114237340
chr11	114242179	114242355	chr11:114242166-114242337
chr11	114247209	114247384	chr11:114247197-114247365
chr11	114250329	114250646	chr11:114250325-114250676

chr11	118353126	118353266	chr11:118353118-118353268
chr11	118436491	118436697	chr11:118436489-118436944
chr11	118436706	118436972	chr11:118436489-118436944
chr11	118439036	118439137	chr11:118439023-118439122
chr11	118447636	118449280	chr11:118447624-118450746
chr11	118449606	118450314	chr11:118447624-118450746
chr11	118450316	118450778	chr11:118447624-118450746
chr11	118467786	118468006	chr11:118467783-118467983
chr11	118468786	118468859	chr11:118468774-118468844
chr11	118471661	118473349	chr11:118471661-118474315
chr11	118473361	118474337	chr11:118471661-118474315
chr11	118476806	118477018	chr11:118476804-118476982
chr11	118477966	118478223	chr11:118477966-118478202
chr11	118480186	118480259	chr11:118480173-118480238
chr11	118481726	118482104	chr11:118481714-118482092
chr11	118482421	118482489	chr11:118482421-118482495
chr11	118484186	118484364	chr11:118484182-118484331
chr11	118484871	118485012	chr11:118484861-118484975
chr11	118488616	118488794	chr11:118488613-118488760
chr11	118489801	118489909	chr11:118489791-118489887
chr11	118490131	118490278	chr11:118490128-118490249
chr11	118491206	118491339	chr11:118491195-118491318
chr11	118491746	118491961	chr11:118491743-118491928
chr11	118493066	118493244	chr11:118493056-118493230
chr11	118494301	118494435	chr11:118494287-118494398
chr11	118494696	118494799	chr11:118494693-118494767
chr11	118495701	118495924	chr11:118495699-118495893
chr11	118496271	118496408	chr11:118496260-118496367
chr11	118497936	118498097	chr11:118497935-118498073
chr11	118498376	118498551	chr11:118498369-118498528
chr11	118499316	118499451	chr11:118499302-118499420
chr11	118499846	118499951	chr11:118499834-118499913
chr11	118500991	118501178	chr11:118500986-118501147
chr11	118501676	118501893	chr11:118501671-118501857
chr11	118502411	118506678	chr11:118502397-118506646
chr11	118507541	118507647	chr11:118507528-118507609
chr11	118509146	118509217	chr11:118509135-118509200
chr11	118509961	118510145	chr11:118509947-118510118
chr11	118511956	118512066	chr11:118511950-118512025
chr11	118519631	118519807	chr11:118519617-118519792
chr11	118520006	118520082	chr11:118519956-118520064
chr11	118520806	118520906	chr11:118520801-118520885
chr11	118521291	118521439	chr11:118521287-118521417
chr11	118521901	118524696	chr11:118521896-118526832
chr11	118524711	118525591	chr11:118521896-118526832
chr11	118525656	118525757	chr11:118521896-118526832
chr11	118525796	118525946	chr11:118521896-118526832
chr11	118525966	118526587	chr11:118521896-118526832
chr11	118526591	118526867	chr11:118521896-118526832
chr11	118971706	118972022	chr11:118971706-118971992
chr11	118978781	118978890	chr11:118978778-118978856
chr11	118978956	118979234	chr11:118978956-118979204
chr11	118979441	118979690	chr11:118979441-118979668
chr11	118980241	118980343	chr11:118980228-118980303
chr11	118980501	118980756	chr11:118980489-118980728
chr11	118981221	118981321	chr11:118981207-118981291
chr11	119121586	119121665	chr11:119121586-119121639
chr11	119121791	119122043	chr11:119121782-119122013
chr11	119124096	119124306	chr11:119123732-119127125
chr11	119124311	119124702	chr11:119123732-119127125
chr11	119125306	119125382	chr11:119123732-119127125
chr11	119125686	119126066	chr11:119123732-119127125
chr11	119126366	119127102	chr11:119123732-119127125

chr11	119130726	119130986	chr11:119130724-119130954
chr11	119131536	119131683	chr11:119131534-119131646
chr11	119131831	119132013	chr11:119131829-119131997
chr11	119132501	119132613	chr11:119132495-119132573
chr11	119132661	119132801	chr11:119132660-119132781
chr11	119132866	119133015	chr11:119132863-119133002
chr11	119133106	119133238	chr11:119133094-119133219
chr11	119134031	119135747	chr11:119134021-119136044
chr11	120236651	120236719	chr11:120236639-120236707
chr11	120240206	120240420	chr11:120240193-120240371
chr11	120242466	120242683	chr11:120242464-120242664
chr11	120246451	120246532	chr11:120246448-120246517
chr11	120269206	120269277	chr11:120269209-120269244
chr11	120298266	120298414	chr11:120298264-120298390
chr11	120299631	120299710	chr11:120299623-120299726
chr11	120301051	120301128	chr11:120301042-120301089
chr11	120302291	120302390	chr11:120302285-120302368
chr11	120305031	120305242	chr11:120305029-120305212
chr11	120305656	120305825	chr11:120305643-120305785
chr11	120307486	120307630	chr11:120307478-120307615
chr11	120309426	120309605	chr11:120309424-120309586
chr11	120315371	120315445	chr11:120315360-120315427
chr11	120317236	120317377	chr11:120317228-120317364
chr11	120318356	120319440	chr11:120318352-120319944
chr11	120319731	120319969	chr11:120318352-120319944
chr11	122977573	122977925	chr11:122977569-122977891
chr11	122979273	122979494	chr11:122979260-122979457
chr11	122980333	122980557	chr11:122980333-122980533
chr11	122981413	122981737	chr11:122981409-122981720
chr11	123724178	123725263	chr11:123724176-123726991
chr11	123725268	123727014	chr11:123724176-123726991
chr11	123727483	123727638	chr11:123727475-123727595
chr11	123728133	123728284	chr11:123728132-123728262
chr11	123729138	123729250	chr11:123729125-123729214
chr11	123729628	123729843	chr11:123729614-123729825
chr11	123730493	123731025	chr11:123730486-123730985
chr11	123736108	123736187	chr11:123736094-123736294
chr11	123736188	123736331	chr11:123736094-123736294
chr11	123738623	123738947	chr11:123738623-123738910
chr11	123739433	123739574	chr11:123739433-123739543
chr11	123740118	123740559	chr11:123740116-123740536
chr11	123741548	123741697	chr11:123741548-123741675
chr11	125164699	125164812	chr11:125164686-125164776
chr11	125184194	125184415	chr11:125184186-125184385
chr11	125235054	125235156	chr11:125235044-125235115
chr11	125239629	125239764	chr11:125239615-125239728
chr11	125240069	125240293	chr11:125240062-125240268
chr11	125287739	125288189	chr11:125287725-125288150
chr11	125330259	125330408	chr11:125330258-125330388
chr11	125331819	125331963	chr11:125331818-125331925
chr11	125332624	125332694	chr11:125332615-125332688
chr11	125351284	125351426	chr11:125351283-125351392
chr11	125353299	125353513	chr11:125353288-125353488
chr11	125367849	125368008	chr11:125367845-125367985
chr11	125385559	125385741	chr11:125385550-125385722
chr11	125397884	125398097	chr11:125397873-125398063
chr11	125410199	125410347	chr11:125410195-125410325
chr11	125410779	125410890	chr11:125410778-125410876
chr11	125411759	125411904	chr11:125411745-125411865
chr11	125429024	125429128	chr11:125429011-125429088
chr11	125429964	125430177	chr11:125429962-125430141
chr11	125431169	125431296	chr11:125431165-125433389
chr11	125431309	125433192	chr11:125431165-125433389

chr11	125433194	125433399	chr11:125431165-125433389
chr11	128458768	128458887	chr11:128458760-128462576
chr11	128458933	128459027	chr11:128458760-128462576
chr11	128459048	128459142	chr11:128458760-128462576
chr11	128459218	128460055	chr11:128458760-128462576
chr11	128460133	128461776	chr11:128458760-128462576
chr11	128461788	128462583	chr11:128458760-128462576
chr11	128463508	128463669	chr11:128463508-128463627
chr11	128467253	128467466	chr11:128467248-128467448
chr11	128480203	128480473	chr11:128480190-128480451
chr11	128484833	128485109	chr11:128484822-128485071
chr11	128486073	128486178	chr11:128486068-128486146
chr11	128489298	128489521	chr11:128489289-128489490
chr11	128490468	128490609	chr11:128490456-128490576
chr11	128504998	128505432	chr11:128504990-128505394
chr11	128521923	128522340	chr11:128521912-128522310
chr11	128536423	128536819	chr11:128536411-128536783
chr11	128556303	128556473	chr11:128556290-128556435
chr11	128573073	128573194	chr11:128573061-128573144
chr11	128587488	128587599	chr11:128587485-128587558
chr11	128686548	128686727	chr11:128686534-128686701
chr11	128692988	128693161	chr11:128692981-128693143
chr11	128693773	128693955	chr11:128693769-128694276
chr11	128693983	128694293	chr11:128693769-128694276
chr11	128748238	128748308	chr11:128748236-128748290
chr11	128755123	128755195	chr11:128755112-128755312
chr11	128755218	128755320	chr11:128755112-128755312
chr11	128758123	128758339	chr11:128758114-128758326
chr11	128764663	128764838	chr11:128764650-128764821
chr11	128768128	128768297	chr11:128768117-128768272
chr11	128772793	128773004	chr11:128772781-128772985
chr11	128781963	128782036	chr11:128781957-128782023
chr11	128805368	128805443	chr11:128805365-128805431
chr11	128807188	128807264	chr11:128807179-128807239
chr11	128809158	128809231	chr11:128809156-128809204
chr11	128810468	128811125	chr11:128810458-128813267
chr11	128811143	128811692	chr11:128810458-128813267
chr11	128811698	128811871	chr11:128810458-128813267
chr11	128811883	128812294	chr11:128810458-128813267
chr11	128812298	128813275	chr11:128810458-128813267
chr11	129375953	129376229	chr11:129375939-129376222
chr11	129386733	129386938	chr11:129386728-129386928
chr11	129436753	129437081	chr11:129436750-129437051
chr11	129442848	129442948	chr11:129442834-129442919
chr11	129451138	129452031	chr11:129451135-129452279
chr11	129452138	129452244	chr11:129451135-129452279
chr11	129899748	129900345	chr11:129899705-129902516
chr11	129900363	129901447	chr11:129899705-129902516
chr11	129901458	129902359	chr11:129899705-129902516
chr11	129902363	129902532	chr11:129899705-129902516
chr11	129905638	129905751	chr11:129905637-129905741
chr11	129910548	129910693	chr11:129910475-129910656
chr11	129912098	129912238	chr11:129912084-129912225
chr11	129914713	129915034	chr11:129914703-129915018
chr11	129915663	129915892	chr11:129915659-129915860
chr11	129917138	129917269	chr11:129917126-129917237
chr11	129918538	129918751	chr11:129918538-129918718
chr11	129923248	129923428	chr11:129923247-129923403
chr11	129924883	129925241	chr11:129924881-129925229
chr11	129931028	129931280	chr11:129931015-129931258
chr11	129932103	129932255	chr11:129932101-129932231
chr11	129935113	129935260	chr11:129935100-129935218
chr11	129937608	129937717	chr11:129937597-129937670

chr11	129942428	129942638	chr11:129942425-129942629
chr11	129944778	129945028	chr11:129944770-129945012
chr11	129947148	129947652	chr11:129947144-129947622
chr11	129948063	129948143	chr11:129948055-129948107
chr11	129955513	129955588	chr11:129955511-129955571
chr11	129957753	129957939	chr11:129957745-129957910
chr11	129960903	129961121	chr11:129960895-129961082
chr11	129978623	129978838	chr11:129978610-129978810
chr11	130002723	130002870	chr11:130002721-130002835
chr12	2857693	2859695	chr12:2857680-2859663
chr12	2861303	2861374	chr12:2861298-2861412
chr12	2864328	2864513	chr12:2864319-2864495
chr12	2864693	2864796	chr12:2864682-2864755
chr12	2864893	2864976	chr12:2864884-2864945
chr12	2865353	2865430	chr12:2865354-2865399
chr12	2866393	2866546	chr12:2866392-2866521
chr12	2868573	2868788	chr12:2868562-2868754
chr12	2872108	2872284	chr12:2872095-2872247
chr12	2873988	2874559	chr12:2873976-2874525
chr12	2876923	2877056	chr12:2876919-2877048
chr12	2959343	2959515	chr12:2959329-2959481
chr12	2959798	2960370	chr12:2959795-2960353
chr12	2994738	2995023	chr12:2994737-2994992
chr12	3003438	3003648	chr12:3003438-3003638
chr12	3011008	3011084	chr12:3011003-3011068
chr12	3012173	3012251	chr12:3012169-3012232
chr12	3017058	3017134	chr12:3017062-3017095
chr12	3017398	3017548	chr12:3017397-3017526
chr12	3018543	3018618	chr12:3018544-3018588
chr12	3019123	3019202	chr12:3019114-3019170
chr12	3020633	3020792	chr12:3020633-3020773
chr12	3021843	3022014	chr12:3021843-3022017
chr12	3037973	3038145	chr12:3037967-3038108
chr12	3040108	3040297	chr12:3040106-3040259
chr12	3040378	3040691	chr12:3040364-3040673
chr12	5949057	5949246	chr12:5949053-5949203
chr12	5949792	5949912	chr12:5949785-5949883
chr12	5989257	5989353	chr12:5989255-5989455
chr12	5989367	5989467	chr12:5989255-5989455
chr12	6230447	6230668	chr12:6230443-6230643
chr12	6236197	6236305	chr12:6236191-6236275
chr12	6237762	6237881	chr12:6237762-6237912
chr12	6666477	6666542	chr12:6666476-6668115
chr12	6666652	6666811	chr12:6666476-6668115
chr12	6666822	6667895	chr12:6666476-6668115
chr12	6667942	6668149	chr12:6666476-6668115
chr12	6669032	6669209	chr12:6669030-6669189
chr12	6670772	6670883	chr12:6670759-6670838
chr12	6672362	6672573	chr12:6672349-6672532
chr12	6673227	6673472	chr12:6673215-6673440
chr12	6677172	6677277	chr12:6677166-6677259
chr12	6678127	6678479	chr12:6678126-6678460
chr12	6678662	6678738	chr12:6678660-6678710
chr12	6678947	6679201	chr12:6678945-6679183
chr12	6679457	6679530	chr12:6679454-6679525
chr12	6687072	6687278	chr12:6687060-6687260
chr12	6688177	6688703	chr12:6688166-6688689
chr12	6688707	6688912	chr12:6688702-6688896
chr12	6689097	6689516	chr12:6689097-6689510
chr12	7787805	7787880	chr12:7787793-7787843
chr12	7788425	7788597	chr12:7788418-7788578
chr12	7789400	7789540	chr12:7789395-7789765
chr12	7789565	7789698	chr12:7789395-7789765

chr12	7789730	7789804	chr12:7789395-7789765
chr12	7790360	7790578	chr12:7790357-7790557
chr12	7792975	7793085	chr12:7792949-7793212
chr12	7793165	7793240	chr12:7792949-7793212
chr12	7794495	7794568	chr12:7794456-7794543
chr12	7794690	7795282	chr12:7794678-7799141
chr12	7795605	7796125	chr12:7794678-7799141
chr12	7796325	7796741	chr12:7794678-7799141
chr12	7797670	7797746	chr12:7794678-7799141
chr12	7797975	7798105	chr12:7794678-7799141
chr12	7798375	7799163	chr12:7794678-7799141
chr12	8032710	8033846	chr12:8032702-8033833
chr12	8035040	8035252	chr12:8035026-8035226
chr12	8039830	8040180	chr12:8039818-8040165
chr12	8042660	8042765	chr12:8042657-8042732
chr12	8043700	8043775	chr12:8043700-8043769
chr12	8043950	8044137	chr12:8043950-8044091
chr12	8044770	8044978	chr12:8044759-8044958
chr12	8047885	8047966	chr12:8047881-8048289
chr12	8047970	8048327	chr12:8047881-8048289
chr12	8048710	8048826	chr12:8048696-8048798
chr12	8049370	8049971	chr12:8049361-8050253
chr12	8050355	8050466	chr12:8050349-8050440
chr12	8050525	8050644	chr12:8050521-8050620
chr12	8052775	8053078	chr12:8052761-8055503
chr12	8053100	8055497	chr12:8052761-8055503
chr12	10699096	10699204	chr12:10699088-10699654
chr12	10699221	10699675	chr12:10699088-10699654
chr12	10701261	10701342	chr12:10701253-10701353
chr12	10701971	10702151	chr12:10701959-10702134
chr12	10702526	10702653	chr12:10702258-10702642
chr12	10704036	10704182	chr12:10704022-10704148
chr12	10708206	10708315	chr12:10708192-10708392
chr12	10708321	10708429	chr12:10708192-10708392
chr12	10709911	10710096	chr12:10709907-10710120
chr12	10710351	10710822	chr12:10710350-10710788
chr12	10713216	10713365	chr12:10713210-10713333
chr12	10715696	10715807	chr12:10715693-10715783
chr12	10717726	10717799	chr12:10717712-10717777
chr12	10718086	10718159	chr12:10718087-10718121
chr12	10718361	10718438	chr12:10718361-10718430
chr12	10719091	10719166	chr12:10719079-10719143
chr12	10720801	10720963	chr12:10720790-10720932
chr12	10721921	10722068	chr12:10721917-10722046
chr12	10722071	10722409	chr12:10722059-10722396
chr12	10722851	10723069	chr12:10722849-10723312
chr12	10723081	10723326	chr12:10722849-10723312
chr12	11649856	11650181	chr12:11649853-11650160
chr12	11716586	11716778	chr12:11716586-11716743
chr12	11747951	11748181	chr12:11747951-11748151
chr12	11752451	11752804	chr12:11752449-11752875
chr12	11752816	11752896	chr12:11752449-11752875
chr12	11825651	11825730	chr12:11825643-11825713
chr12	11826406	11826589	chr12:11826401-11826556
chr12	11839151	11839339	chr12:11839139-11839304
chr12	11840471	11840881	chr12:11840460-11840868
chr12	11853426	11853582	chr12:11853426-11853561
chr12	11869436	11869860	chr12:11869423-11869969
chr12	11869861	11870014	chr12:11869423-11869969
chr12	11884451	11884625	chr12:11884444-11884587
chr12	11885931	11886038	chr12:11885925-11886026
chr12	11890941	11891364	chr12:11890940-11895402
chr12	11891366	11891607	chr12:11890940-11895402

chr12	11891646	11892213	chr12:11890940-11895402
chr12	11892231	11892374	chr12:11890940-11895402
chr12	11892376	11892509	chr12:11890940-11895402
chr12	11892516	11893694	chr12:11890940-11895402
chr12	11893921	11895286	chr12:11890940-11895402
chr12	11895306	11895379	chr12:11890940-11895402
chr12	12611836	12612228	chr12:12611826-12612187
chr12	12631426	12631639	chr12:12631412-12631612
chr12	12632496	12632607	chr12:12632488-12632591
chr12	12635781	12636000	chr12:12635776-12635974
chr12	12637581	12637754	chr12:12637569-12637714
chr12	12642006	12642818	chr12:12641993-12645108
chr12	12642836	12643670	chr12:12641993-12645108
chr12	12643671	12645133	chr12:12641993-12645108
chr12	12725996	12726107	chr12:12725996-12726096
chr12	12737116	12737330	chr12:12737113-12737313
chr12	12785611	12785720	chr12:12785606-12785694
chr12	12786911	12787100	chr12:12786908-12787058
chr12	19404056	19404226	chr12:19404044-19404216
chr12	19439681	19439818	chr12:19439673-19440370
chr12	19439831	19440392	chr12:19439673-19440370
chr12	19440591	19440761	chr12:19440580-19440727
chr12	19462521	19462731	chr12:19462509-19462717
chr12	19473261	19473368	chr12:19473247-19473355
chr12	19493801	19494023	chr12:19493799-19493986
chr12	19500101	19500237	chr12:19500092-19500221
chr12	19512401	19512478	chr12:19512397-19512465
chr12	19514671	19514810	chr12:19514670-19514784
chr12	19518126	19518222	chr12:19518086-19522239
chr12	19518241	19518444	chr12:19518086-19522239
chr12	19518446	19519219	chr12:19518086-19522239
chr12	19519241	19519753	chr12:19518086-19522239
chr12	19519761	19519938	chr12:19518086-19522239
chr12	19519976	19520477	chr12:19518086-19522239
chr12	19520501	19520784	chr12:19518086-19522239
chr12	19520961	19521038	chr12:19518086-19522239
chr12	19521111	19521520	chr12:19518086-19522239
chr12	19521526	19522069	chr12:19518086-19522239
chr12	19522076	19522250	chr12:19518086-19522239
chr12	19553121	19553195	chr12:19552852-19553272
chr12	19553201	19553314	chr12:19552852-19553272
chr12	19623216	19623421	chr12:19623202-19623402
chr12	19720736	19720839	chr12:19720632-19720801
chr12	21373346	21373452	chr12:21373336-21373431
chr12	21373646	21373744	chr12:21373641-21373733
chr12	21375431	21375639	chr12:21375419-21375619
chr12	21378246	21378418	chr12:21378236-21378386
chr12	23529500	23529573	chr12:23529499-23534522
chr12	23529590	23530172	chr12:23529499-23534522
chr12	23530175	23530794	chr12:23529499-23534522
chr12	23530855	23531129	chr12:23529499-23534522
chr12	23531130	23531328	chr12:23529499-23534522
chr12	23531350	23531585	chr12:23529499-23534522
chr12	23531605	23531674	chr12:23529499-23534522
chr12	23531700	23531905	chr12:23529499-23534522
chr12	23531950	23532054	chr12:23529499-23534522
chr12	23532210	23533237	chr12:23529499-23534522
chr12	23533360	23533694	chr12:23529499-23534522
chr12	23533790	23533902	chr12:23529499-23534522
chr12	23533920	23534551	chr12:23529499-23534522
chr12	23536455	23536707	chr12:23536452-23536669
chr12	23543215	23543398	chr12:23543210-23543384
chr12	23546325	23546425	chr12:23546315-23546424

chr12	23563260	23563446	chr12:23563257-23563403
chr12	23575670	23575885	chr12:23575658-23575838
chr12	23584555	23584633	chr12:23584546-23584612
chr12	23604390	23604556	chr12:23604386-23604533
chr12	23632145	23632228	chr12:23632145-23632194
chr12	23640825	23640932	chr12:23640811-23640897
chr12	23665450	23665595	chr12:23665443-23665564
chr12	23734690	23734778	chr12:23734683-23734752
chr12	23738390	23738535	chr12:23738385-23738506
chr12	23740855	23741068	chr12:23740850-23741039
chr12	23755645	23755740	chr12:23755637-23755724
chr12	23762475	23762614	chr12:23762474-23762597
chr12	23813305	23813524	chr12:23813301-23813501
chr12	23845990	23846203	chr12:23845982-23846193
chr12	23895805	23896049	chr12:23895792-23896027
chr12	23920040	23920149	chr12:23920037-23920614
chr12	23920150	23920641	chr12:23920037-23920614
chr12	23944175	23944385	chr12:23944170-23944352
chr12	23949575	23949709	chr12:23949563-23949703
chr12	23950880	23950964	chr12:23950868-23951032
chr12	23950995	23951075	chr12:23950868-23951032
chr12	26120039	26120424	chr12:26120025-26121840
chr12	26120444	26121045	chr12:26120025-26121840
chr12	26121069	26121864	chr12:26120025-26121840
chr12	26122314	26123183	chr12:26122302-26123168
chr12	26123639	26123773	chr12:26123629-26123741
chr12	26124084	26124208	chr12:26124071-26124179
chr12	26124519	26124594	chr12:26124518-26124582
chr12	26124729	26124972	chr12:26124717-26125127
chr12	26124974	26125143	chr12:26124717-26125127
chr12	27332854	27333171	chr12:27332853-27333136
chr12	27343019	27343229	chr12:27343018-27343218
chr12	27368264	27368438	chr12:27368261-27368412
chr12	27370129	27370240	chr12:27370128-27370230
chr12	27376349	27376425	chr12:27376345-27376387
chr12	27377454	27377538	chr12:27377440-27377503
chr12	27380259	27380437	chr12:27380246-27380404
chr12	27385484	27385599	chr12:27385480-27385560
chr12	27387229	27387335	chr12:27387227-27387320
chr12	27389179	27389327	chr12:27389178-27389296
chr12	27390104	27390283	chr12:27390095-27390239
chr12	27400534	27400779	chr12:27400533-27400774
chr12	27401259	27401374	chr12:27401256-27401363
chr12	27401524	27401698	chr12:27401523-27401674
chr12	27402619	27402699	chr12:27402618-27402677
chr12	27403439	27403578	chr12:27403432-27403541
chr12	27415874	27415980	chr12:27415868-27415947
chr12	27418094	27418234	chr12:27418087-27418196
chr12	27420399	27421277	chr12:27420395-27425289
chr12	27421399	27421477	chr12:27420395-27425289
chr12	27421629	27423311	chr12:27420395-27425289
chr12	27423619	27423895	chr12:27420395-27425289
chr12	27423909	27424105	chr12:27420395-27425289
chr12	27424109	27425187	chr12:27420395-27425289
chr12	27425189	27425317	chr12:27420395-27425289
chr12	45014682	45015968	chr12:45014671-45016618
chr12	45016012	45016640	chr12:45014671-45016618
chr12	45023797	45023914	chr12:45023788-45023894
chr12	45036032	45036146	chr12:45036018-45036114
chr12	45046792	45047001	chr12:45046783-45046983
chr12	45050532	45051122	chr12:45050524-45051099
chr12	47841548	47842489	chr12:47841536-47845005
chr12	47842758	47845030	chr12:47841536-47845005

chr12	47846338	47846493	chr12:47846334-47846451
chr12	47846658	47846814	chr12:47846656-47846808
chr12	47855633	47855821	chr12:47855629-47855801
chr12	47857128	47857283	chr12:47857128-47857249
chr12	47857508	47857722	chr12:47857503-47857688
chr12	47865048	47865198	chr12:47865046-47865177
chr12	47878828	47879141	chr12:47878815-47879115
chr12	47882703	47882950	chr12:47882693-47882935
chr12	47899843	47899985	chr12:47899836-47899958
chr12	47904563	47905066	chr12:47904563-47905040
chr12	47929868	47930087	chr12:47929865-47930065
chr12	47942873	47943090	chr12:47942862-47943048
chr12	48995178	48995286	chr12:48995148-48998666
chr12	48995288	48995368	chr12:48995148-48998666
chr12	48995383	48996334	chr12:48995148-48998666
chr12	48996338	48998684	chr12:48995148-48998666
chr12	48999083	48999325	chr12:48999078-48999309
chr12	50108038	50108150	chr12:50108030-50108130
chr12	50109433	50109605	chr12:50109422-50109572
chr12	50763703	50763778	chr12:50763709-50763741
chr12	50764078	50764330	chr12:50764077-50764307
chr12	50764493	50764663	chr12:50764479-50764647
chr12	50775583	50775689	chr12:50775581-50775658
chr12	50780143	50780263	chr12:50780139-50780238
chr12	50792843	50793066	chr12:50792843-50793043
chr12	50795913	50796032	chr12:50795908-50796009
chr12	50809463	50809615	chr12:50809455-50809589
chr12	50814023	50814230	chr12:50814009-50814192
chr12	50814283	50814463	chr12:50814279-50814439
chr12	50819638	50819785	chr12:50819634-50821122
chr12	50819798	50820103	chr12:50819634-50821122
chr12	50820108	50820174	chr12:50819634-50821122
chr12	50820183	50820636	chr12:50819634-50821122
chr12	50820638	50821162	chr12:50819634-50821122
chr12	51061213	51062260	chr12:51061204-51064669
chr12	51062288	51064710	chr12:51061204-51064669
chr12	51067678	51068009	chr12:51067672-51067969
chr12	51073823	51074115	chr12:51073822-51074082
chr12	51076423	51076659	chr12:51076410-51076647
chr12	51077948	51078171	chr12:51077948-51078148
chr12	51081428	51081508	chr12:51081427-51081496
chr12	51082523	51082701	chr12:51082520-51082694
chr12	51083008	51083121	chr12:51083006-51083110
chr12	51083228	51083685	chr12:51083228-51083664
chr12	51093663	51093838	chr12:51093662-51095278
chr12	51093858	51093995	chr12:51093662-51095278
chr12	51094003	51095279	chr12:51093662-51095278
chr12	51095988	51096065	chr12:51095988-51096040
chr12	51098788	51098961	chr12:51098775-51098918
chr12	51099698	51099794	chr12:51099654-51099779
chr12	51101943	51102049	chr12:51101934-51102025
chr12	51103678	51103783	chr12:51103669-51103763
chr12	51104168	51104240	chr12:51104154-51104203
chr12	51106538	51106646	chr12:51106524-51106613
chr12	51107308	51107378	chr12:51107294-51107346
chr12	51109128	51109314	chr12:51109120-51109273
chr12	51110888	51111023	chr12:51110876-51110983
chr12	51113138	51113356	chr12:51113132-51113332
chr12	51116318	51116439	chr12:51116314-51116420
chr12	51117683	51117782	chr12:51117670-51117747
chr12	51118628	51118809	chr12:51118620-51118772
chr12	51172313	51172934	chr12:51172300-51172912
chr12	51173063	51173169	chr12:51173052-51173134

chr12	51186938	51187289	chr12:51186935-51190592
chr12	51187293	51187434	chr12:51186935-51190592
chr12	51187443	51187939	chr12:51186935-51190592
chr12	51188213	51188464	chr12:51186935-51190592
chr12	51188498	51189291	chr12:51186935-51190592
chr12	51189313	51190609	chr12:51186935-51190592
chr12	51191598	51191780	chr12:51191595-51191764
chr12	51192333	51192489	chr12:51192329-51192471
chr12	51195983	51196191	chr12:51195969-51196173
chr12	51196798	51196984	chr12:51196798-51196946
chr12	51197773	51198203	chr12:51197769-51198167
chr12	51198563	51198801	chr12:51198549-51198775
chr12	51199753	51199893	chr12:51199746-51199868
chr12	51204173	51204401	chr12:51204172-51204368
chr12	51206793	51206970	chr12:51206788-51206947
chr12	51207323	51207699	chr12:51207222-51207693
chr12	51216768	51216997	chr12:51216768-51216968
chr12	51217648	51217722	chr12:51217641-51217708
chr12	52022838	52022977	chr12:52022831-52022939
chr12	52037178	52037457	chr12:52037176-52037443
chr12	52037728	52037895	chr12:52037714-52038749
chr12	52037928	52038062	chr12:52037714-52038749
chr12	52038348	52038776	chr12:52037714-52038749
chr12	52040468	52040677	chr12:52040456-52040656
chr12	52041818	52041959	chr12:52041804-52041929
chr12	52043813	52043949	chr12:52043801-52043911
chr12	52045493	52045606	chr12:52045479-52045568
chr12	52051403	52051582	chr12:52051401-52051572
chr12	52052473	52052663	chr12:52052473-52052645
chr12	52054328	52054621	chr12:52054326-52055306
chr12	52054623	52055332	chr12:52054326-52055306
chr12	52056038	52056180	chr12:52056029-52056159
chr12	52056493	52056672	chr12:52056493-52056645
chr12	52056903	52057288	chr12:52056889-52057259
chr12	52057363	52057570	chr12:52057351-52057530
chr12	52058693	52059531	chr12:52058687-52059507
chr12	53210578	53211871	chr12:53210566-53211863
chr12	53213088	53213273	chr12:53213084-53213243
chr12	53213503	53213714	chr12:53213495-53213700
chr12	53214058	53214275	chr12:53214058-53214235
chr12	53214448	53214631	chr12:53214445-53214606
chr12	53215298	53215476	chr12:53215292-53215434
chr12	53215653	53215837	chr12:53215645-53215794
chr12	53219953	53220438	chr12:53219949-53220413
chr12	53223128	53223339	chr12:53223116-53223316
chr12	53227363	53227690	chr12:53227361-53227687
chr12	53229848	53230097	chr12:53229841-53230074
chr12	53231168	53231246	chr12:53231167-53231235
chr12	53231398	53231582	chr12:53231390-53231550
chr12	53231983	53232223	chr12:53231973-53232256
chr12	53232513	53233018	chr12:53232510-53232980
chr12	53326603	53329436	chr12:53326574-53329420
chr12	53335628	53335787	chr12:53335625-53335754
chr12	53336158	53336415	chr12:53336145-53336382
chr12	53340608	53340821	chr12:53340608-53340808
chr12	53345123	53345339	chr12:53345113-53345315
chr12	53380183	53380328	chr12:53380175-53380298
chr12	53380638	53380708	chr12:53380638-53380684
chr12	53381313	53381858	chr12:53381301-53381813
chr12	53382123	53383658	chr12:53382109-53383622
chr12	53389913	53390115	chr12:53389903-53390103
chr12	53406588	53406729	chr12:53406584-53406753
chr12	53409373	53409587	chr12:53409361-53409561

chr12	53410938	53411632	chr12:53410926-53416446
chr12	53411678	53411864	chr12:53410926-53416446
chr12	53411878	53412936	chr12:53410926-53416446
chr12	53412958	53413407	chr12:53410926-53416446
chr12	53413408	53414167	chr12:53410926-53416446
chr12	53414203	53415638	chr12:53410926-53416446
chr12	53415653	53416350	chr12:53410926-53416446
chr12	53416363	53416463	chr12:53410926-53416446
chr12	53512128	53517367	chr12:53512058-53517354
chr12	53523168	53523423	chr12:53523156-53523269;chr12:53523275-53523384
chr12	53524563	53524778	chr12:53524563-53524761
chr12	53524873	53525012	chr12:53524864-53524976
chr12	53531753	53531922	chr12:53531743-53531896
chr12	53532523	53532624	chr12:53532509-53532623
chr12	53533163	53533292	chr12:53533159-53533259
chr12	53534513	53534688	chr12:53534501-53534659
chr12	53537428	53537573	chr12:53537414-53537552
chr12	53542898	53543031	chr12:53542886-53543448
chr12	53543063	53543476	chr12:53542886-53543448
chr12	53543848	53543988	chr12:53543839-53543951
chr12	53552553	53552656	chr12:53552540-53552637
chr12	53600953	53601032	chr12:53600952-53601021
chr12	53602368	53602588	chr12:53602367-53602567
chr12	53625963	53626030	chr12:53625950-53626000
chr12	53626278	53626425	chr12:53626278-53626410
chr12	53938773	53938991	chr12:53938764-53939642
chr12	53938998	53939655	chr12:53938764-53939642
chr12	53940708	53940928	chr12:53940704-53940904
chr12	53945013	53946032	chr12:53944999-53946544
chr12	53946053	53946504	chr12:53944999-53946544
chr12	53973138	53973935	chr12:53973125-53973927
chr12	53975193	53975462	chr12:53975180-53977643
chr12	53975478	53976117	chr12:53975180-53977643
chr12	53976138	53976863	chr12:53975180-53977643
chr12	53976868	53977677	chr12:53975180-53977643
chr12	53985063	53985139	chr12:53985064-53985097
chr12	53985198	53986029	chr12:53985185-53986010
chr12	53986133	53986210	chr12:53986131-53986193
chr12	53986348	53986422	chr12:53986337-53986380
chr12	53986443	53986713	chr12:53986429-53986686
chr12	53987838	53988041	chr12:53987827-53988027
chr12	53989173	53990309	chr12:53989168-53990279
chr12	53990628	53990843	chr12:53990623-53990814
chr12	54009108	54009741	chr12:54009105-54009720
chr12	54010318	54010523	chr12:54010304-54010504
chr12	54011098	54011232	chr12:54011088-54012362
chr12	54011243	54011344	chr12:54011088-54012362
chr12	54011348	54011864	chr12:54011088-54012362
chr12	54011943	54012227	chr12:54011088-54012362
chr12	54015898	54016015	chr12:54015896-54017414
chr12	54016023	54016362	chr12:54015896-54017414
chr12	54016368	54016965	chr12:54015896-54017414
chr12	54016988	54017264	chr12:54015896-54017414
chr12	54017298	54017384	chr12:54015896-54017414
chr12	54026338	54026567	chr12:54026334-54026534
chr12	54028368	54028958	chr12:54028357-54028922
chr12	54029633	54030626	chr12:54029630-54030823
chr12	54030733	54030828	chr12:54029630-54030823
chr12	54032858	54033600	chr12:54032852-54033576
chr12	54034283	54035379	chr12:54034277-54035358
chr12	54037853	54038034	chr12:54037853-54038014
chr12	54047778	54047950	chr12:54047768-54047917
chr12	54053173	54053454	chr12:54053159-54053424

chr12	54053883	54054379	chr12:54053876-54054361
chr12	54054858	54055278	chr12:54054849-54056030
chr12	54055308	54055594	chr12:54054849-54056030
chr12	54055628	54056050	chr12:54054849-54056030
chr12	54292113	54292605	chr12:54292110-54293381
chr12	54292633	54293417	chr12:54292110-54293381
chr12	54295138	54295802	chr12:54295134-54295760
chr12	54297763	54297904	chr12:54297763-54297884
chr12	54299203	54299411	chr12:54299190-54299390
chr12	54300153	54300304	chr12:54300146-54300276
chr12	54300813	54301083	chr12:54300800-54301051
chr12	55019953	55020338	chr12:55019944-55020313
chr12	55021283	55021491	chr12:55021277-55021477
chr12	55026443	55026580	chr12:55026430-55030014
chr12	55026623	55029488	chr12:55026430-55030014
chr12	55029503	55030047	chr12:55026430-55030014
chr12	55757428	55757573	chr12:55757428-55757553
chr12	55760463	55760667	chr12:55760423-55760640
chr12	55789078	55789151	chr12:55789074-55789143
chr12	55794813	55794913	chr12:55794806-55794880
chr12	55796028	55796100	chr12:55796024-55796076
chr12	55797373	55797584	chr12:55797364-55797564
chr12	55800573	55800640	chr12:55800561-55800629
chr12	55800863	55800940	chr12:55800853-55800900
chr12	55803633	55803740	chr12:55803628-55803728
chr12	56341603	56342022	chr12:56341596-56343531
chr12	56342593	56343567	chr12:56341596-56343531
chr12	56343838	56344154	chr12:56343824-56344135
chr12	56346153	56346230	chr12:56346145-56346203
chr12	56346453	56346662	chr12:56346441-56346624
chr12	56346828	56346976	chr12:56346818-56346955
chr12	56348533	56348649	chr12:56348528-56348623
chr12	56348753	56348837	chr12:56348751-56348804
chr12	56348923	56349066	chr12:56348923-56349059
chr12	56349163	56349275	chr12:56349162-56349261
chr12	56349353	56349536	chr12:56349344-56349509
chr12	56349588	56349766	chr12:56349588-56349775
chr12	56350053	56350255	chr12:56349859-56350432
chr12	56350258	56350439	chr12:56349859-56350432
chr12	56350828	56350905	chr12:56350828-56350888
chr12	56351098	56351488	chr12:56351097-56351190;chr12:56351202-56351450
chr12	56354418	56354641	chr12:56354418-56354614
chr12	56354778	56354889	chr12:56354777-56354872
chr12	56355273	56355389	chr12:56355272-56355351
chr12	56355443	56355563	chr12:56355442-56355532
chr12	56355713	56355823	chr12:56355707-56355803
chr12	56356143	56356318	chr12:56356131-56356285
chr12	56356448	56356602	chr12:56356440-56356578
chr12	56360058	56360132	chr12:56360057-56360126
chr12	57095638	57096271	chr12:57095626-57096761
chr12	57096278	57096805	chr12:57095626-57096761
chr12	57096858	57097014	chr12:57096849-57096978
chr12	57097078	57097157	chr12:57097067-57097133
chr12	57098508	57098613	chr12:57098504-57098597
chr12	57098793	57098937	chr12:57098791-57098902
chr12	57099018	57099095	chr12:57099014-57099078
chr12	57099293	57099482	chr12:57099293-57099440
chr12	57099778	57099917	chr12:57099766-57099903
chr12	57099998	57100105	chr12:57099995-57100090
chr12	57102293	57102507	chr12:57102289-57102496
chr12	57102828	57102940	chr12:57102828-57102990
chr12	57104464	57104613	chr12:57104463-57104586
chr12	57104729	57104856	chr12:57104725-57104813

chr12	57105159	57105367	chr12:57105150-57105339
chr12	57105469	57105622	chr12:57105467-57105599
chr12	57106204	57106387	chr12:57106190-57106339
chr12	57106539	57106618	chr12:57106527-57106580
chr12	57106704	57106842	chr12:57106692-57106831
chr12	57107244	57107346	chr12:57107230-57107314
chr12	57107614	57107757	chr12:57107604-57107743
chr12	57108169	57108319	chr12:57108162-57108299
chr12	57109944	57110363	chr12:57109940-57110340
chr12	57110389	57110562	chr12:57110381-57110543
chr12	57111109	57111254	chr12:57111109-57111433
chr12	57111314	57111453	chr12:57111109-57111433
chr12	57126959	57127177	chr12:57126948-57127148
chr12	57132034	57132183	chr12:57132020-57132139
chr12	57460134	57460209	chr12:57460134-57460201
chr12	57460494	57460569	chr12:57460490-57460555
chr12	57461989	57462200	chr12:57461989-57462189
chr12	57463674	57463814	chr12:57463664-57463791
chr12	57463999	57464119	chr12:57463998-57464091
chr12	57464679	57464896	chr12:57464672-57464868
chr12	57465114	57465292	chr12:57465110-57465255
chr12	57465609	57465721	chr12:57465606-57465696
chr12	57465799	57465939	chr12:57465787-57465925
chr12	57466239	57466433	chr12:57466239-57466389
chr12	57467334	57467524	chr12:57467332-57467497
chr12	57467999	57468248	chr12:57467993-57468224
chr12	57469444	57469712	chr12:57469430-57469698
chr12	57470319	57472145	chr12:57470316-57472104
chr12	57516814	57517021	chr12:57516805-57517132
chr12	57517034	57517148	chr12:57516805-57517132
chr12	57517719	57517794	chr12:57517719-57517753
chr12	57520424	57520542	chr12:57520417-57520517
chr12	65051320	65051510	chr12:65051320-65051470
chr12	65055120	65055235	chr12:65055117-65055213
chr12	65082710	65082924	chr12:65082709-65082909
chr12	65824130	65824413	chr12:65824130-65825381
chr12	65824445	65824719	chr12:65824130-65825381
chr12	65824765	65825045	chr12:65824130-65825381
chr12	65825055	65825402	chr12:65824130-65825381
chr12	65828005	65828115	chr12:65828000-65828087
chr12	65838520	65838591	chr12:65838518-65838569
chr12	65842145	65842240	chr12:65842133-65842254
chr12	65842590	65843539	chr12:65842590-65843523
chr12	65867500	65867845	chr12:65867488-65867807
chr12	65881745	65882130	chr12:65881742-65882126
chr12	65909710	65909922	chr12:65909702-65909902
chr12	65914955	65915554	chr12:65914719-65915527
chr12	65951385	65952623	chr12:65951382-65958854
chr12	65952645	65953398	chr12:65951382-65958854
chr12	65953400	65954934	chr12:65951382-65958854
chr12	65955190	65955295	chr12:65951382-65958854
chr12	65955345	65957369	chr12:65951382-65958854
chr12	65957485	65958864	chr12:65951382-65958854
chr12	65963250	65963402	chr12:65963244-65966295
chr12	65963420	65964278	chr12:65963244-65966295
chr12	65964345	65964726	chr12:65963244-65966295
chr12	65964755	65965127	chr12:65963244-65966295
chr12	65965135	65966329	chr12:65963244-65966295
chr12	77021305	77021718	chr12:77021246-77024185
chr12	77021720	77022339	chr12:77021246-77024185
chr12	77022340	77022551	chr12:77021246-77024185
chr12	77022565	77022633	chr12:77021246-77024185
chr12	77022675	77024185	chr12:77021246-77024185

chr12	77025560	77026022	chr12:77025557-77025982
chr12	77027885	77028167	chr12:77027882-77028138
chr12	77029830	77030355	chr12:77029830-77030332
chr12	77033060	77033160	chr12:77033049-77033122
chr12	77033860	77034084	chr12:77033856-77034042
chr12	77043075	77043219	chr12:77043064-77043199
chr12	77044545	77044825	chr12:77044533-77044795
chr12	77046040	77046369	chr12:77046037-77046328
chr12	77048285	77048494	chr12:77048274-77048474
chr12	77050580	77050763	chr12:77050575-77050746
chr12	77055860	77056136	chr12:77055854-77056130
chr12	77064555	77064686	chr12:77064542-77064653
chr12	77065350	77065605	chr12:77065344-77065580
chr12	80707506	80708274	chr12:80707497-80708238
chr12	80708536	80708634	chr12:80708523-80708614
chr12	80708851	80709223	chr12:80708841-80709474
chr12	80709231	80709500	chr12:80708841-80709474
chr12	80716916	80717595	chr12:80716911-80717564
chr12	80718361	80718471	chr12:80718357-80718433
chr12	80718861	80719358	chr12:80718860-80719673
chr12	80719421	80719689	chr12:80718860-80719673
chr12	85280109	85280178	chr12:85280106-85280487
chr12	85280184	85280504	chr12:85280106-85280487
chr12	85283574	85283900	chr12:85283571-85283876
chr12	85286864	85287004	chr12:85286852-85286981
chr12	85299554	85299687	chr12:85299471-85299671
chr12	85301154	85301488	chr12:85301154-85301784
chr12	85301489	85301804	chr12:85301154-85301784
chr12	85875294	85875498	chr12:85875280-85875480
chr12	85876639	85876714	chr12:85876639-85876701
chr12	85878479	85878583	chr12:85878469-85878569
chr12	85882224	85882403	chr12:85882222-85882372
chr12	95020235	95020931	chr12:95020228-95022403
chr12	95020935	95021205	chr12:95020228-95022403
chr12	95021495	95022408	chr12:95020228-95022403
chr12	95025150	95025294	chr12:95025149-95025255
chr12	95026825	95026894	chr12:95026832-95026868
chr12	95028390	95028533	chr12:95028386-95028524
chr12	95030420	95030903	chr12:95030408-95030873
chr12	95031350	95031494	chr12:95031348-95031488
chr12	95037120	95037322	chr12:95037112-95037312
chr12	95040485	95040635	chr12:95040475-95040598
chr12	95049080	95049272	chr12:95049067-95049233
chr12	95051765	95051975	chr12:95051761-95051943
chr12	95057560	95057675	chr12:95057552-95057643
chr12	95057730	95057909	chr12:95057730-95057878
chr12	95058310	95058524	chr12:95058309-95058489
chr12	95059915	95059988	chr12:95059905-95059984
chr12	95062510	95062767	chr12:95062507-95062738
chr12	95062855	95062926	chr12:95062849-95062887
chr12	95067330	95067407	chr12:95067330-95067391
chr12	95072640	95073028	chr12:95072639-95073017
chr12	95073380	95073741	chr12:95073379-95073703
chr12	96194393	96194725	chr12:96194381-96194705
chr12	96198028	96198177	chr12:96198025-96198142
chr12	96212738	96212877	chr12:96212724-96212841
chr12	96223578	96223812	chr12:96223564-96223773
chr12	96233723	96233938	chr12:96233711-96233911
chr12	96246943	96247762	chr12:96246939-96247734
chr12	96255463	96255583	chr12:96255459-96255550
chr12	96259733	96259889	chr12:96259730-96259853
chr12	96267093	96268938	chr12:96267081-96269835
chr12	96268943	96269216	chr12:96267081-96269835

chr12	96269218	96269567	chr12:96267081-96269835
chr12	96269618	96269862	chr12:96267081-96269835
chr12	100473708	100474092	chr12:100473707-100474059
chr12	100484423	100484639	chr12:100484420-100484620
chr12	100492503	100492648	chr12:100492502-100492637
chr12	100493283	100493415	chr12:100493269-100493402
chr12	100499863	100500038	chr12:100499852-100500024
chr12	100503368	100503519	chr12:100503359-100503496
chr12	100505603	100505846	chr12:100505574-100505824
chr12	100510778	100511173	chr12:100510777-100511143
chr12	100532463	100532641	chr12:100532457-100532610
chr12	100534903	100535042	chr12:100534889-100535023
chr12	100536518	100536619	chr12:100536506-100536610
chr12	100536953	100537053	chr12:100536947-100537047
chr12	100540683	100540848	chr12:100540671-100540818
chr12	100561898	100562034	chr12:100561884-100561998
chr12	100563263	100564247	chr12:100563250-100564413
chr12	100564253	100564430	chr12:100563250-100564413
chr12	101475426	101475530	chr12:101475420-101475502
chr12	101476841	101476944	chr12:101476827-101476907
chr12	101477601	101477677	chr12:101477557-101477651
chr12	101479581	101479717	chr12:101479581-101479694
chr12	101482806	101482931	chr12:101482791-101482900
chr12	101485151	101485378	chr12:101485151-101485351
chr12	101486346	101486421	chr12:101486343-101486997
chr12	101486466	101486948	chr12:101486343-101486997
chr12	101486951	101487021	chr12:101486343-101486997
chr12	102957946	102958076	chr12:102957944-102958044
chr12	102959361	102959433	chr12:102959361-102959511
chr12	102959451	102959521	chr12:102959361-102959511
chr12	106582919	106583084	chr12:106582906-106583363
chr12	106583239	106583380	chr12:106582906-106583363
chr12	106583644	106583755	chr12:106583644-106583734
chr12	106585729	106586010	chr12:106585726-106585976
chr12	106593379	106593601	chr12:106593367-106593567
chr12	106601144	106601350	chr12:106601136-106601346
chr12	106608799	106608925	chr12:106608796-106608883
chr12	106639344	106639419	chr12:106639331-106639392
chr12	106645884	106645964	chr12:106645881-106645936
chr12	106654234	106654380	chr12:106654227-106654351
chr12	106682004	106682073	chr12:106681992-106682054
chr12	106684709	106684931	chr12:106684695-106684908
chr12	106686884	106687133	chr12:106686883-106687097
chr12	106689299	106689400	chr12:106689286-106689364
chr12	106693074	106693181	chr12:106693065-106693159
chr12	106696284	106696471	chr12:106696282-106696446
chr12	106709334	106709448	chr12:106709329-106709430
chr12	106711459	106711535	chr12:106711452-106711511
chr12	106715409	106715579	chr12:106715399-106715544
chr12	106719969	106720075	chr12:106719959-106720054
chr12	106720769	106720902	chr12:106720758-106720876
chr12	106732134	106732288	chr12:106732129-106732249
chr12	106732924	106733110	chr12:106732923-106733085
chr12	106747439	106747615	chr12:106747436-106747599
chr12	106750654	106750815	chr12:106750654-106750793
chr12	106761199	106761500	chr12:106761196-106762803
chr12	106761514	106762242	chr12:106761196-106762803
chr12	106762244	106762836	chr12:106761196-106762803
chr12	107318418	107319926	chr12:107318412-107320075
chr12	107319938	107320113	chr12:107318412-107320075
chr12	107320598	107320672	chr12:107320587-107320635
chr12	107494773	107494987	chr12:107494772-107494972
chr12	107520498	107520682	chr12:107520486-107520636

chr12	107543938	107544150	chr12:107543934-107544139
chr12	107580658	107580905	chr12:107580649-107581007
chr12	107580918	107581025	chr12:107580649-107581007
chr12	107581153	107581333	chr12:107581151-107581304
chr12	107610203	107610385	chr12:107610189-107610356
chr12	107612793	107612902	chr12:107612779-107612869
chr12	107615073	107615185	chr12:107615060-107615162
chr12	107617088	107617203	chr12:107617088-107617182
chr12	107617303	107617447	chr12:107617300-107617436
chr12	107618158	107618417	chr12:107618157-107618378
chr12	107619998	107620185	chr12:107619985-107620164
chr12	107634833	107634973	chr12:107634831-107634958
chr12	107635298	107635398	chr12:107635284-107635390
chr12	107640318	107640428	chr12:107640310-107640413
chr12	107642093	107642208	chr12:107642089-107642171
chr12	107649208	107650303	chr12:107649205-107650690
chr12	107650313	107650517	chr12:107649205-107650690
chr12	107651683	107651831	chr12:107651673-107651787
chr12	107657513	107658208	chr12:107657508-107659642
chr12	107658228	107658330	chr12:107657508-107659642
chr12	107658343	107659679	chr12:107657508-107659642
chr12	107732878	107734540	chr12:107732865-107734522
chr12	107739383	107739573	chr12:107739382-107739551
chr12	107740948	107741269	chr12:107740945-107741260
chr12	107742223	107742382	chr12:107742220-107742348
chr12	107743203	107743328	chr12:107743196-107743282
chr12	107744543	107744701	chr12:107744542-107744682
chr12	107746288	107746462	chr12:107746274-107746424
chr12	107751423	107752226	chr12:107751414-107752209
chr12	107753933	107754129	chr12:107753923-107754109
chr12	107754833	107754906	chr12:107754828-107754860
chr12	107756838	107756996	chr12:107756831-107756965
chr12	107760513	107760964	chr12:107760504-107760928
chr12	107760968	107761289	chr12:107760956-107761272
chr12	108912593	108912775	chr12:108912591-108912741
chr12	108915783	108915901	chr12:108915782-108915872
chr12	109019648	109019726	chr12:109019646-109019846
chr12	109019733	109019877	chr12:109019646-109019846
chr12	109277978	109279944	chr12:109277978-109279930
chr12	109281418	109281837	chr12:109281406-109281799
chr12	109285303	109285522	chr12:109285303-109285511
chr12	109286658	109286767	chr12:109286647-109286861
chr12	109286773	109286878	chr12:109286647-109286861
chr12	109287443	109287561	chr12:109287396-109287524
chr12	109287843	109287993	chr12:109287843-109287957
chr12	109288063	109288213	chr12:109288055-109288180
chr12	109290153	109290320	chr12:109290140-109290286
chr12	109291933	109292153	chr12:109291924-109292124
chr12	109308248	109308349	chr12:109308235-109308324
chr12	109309118	109309233	chr12:109309118-109309220
chr12	111034023	111034274	chr12:111034023-111034240
chr12	111037288	111037493	chr12:111037278-111037478
chr12	111099448	111099634	chr12:111099445-111099636
chr12	111099943	111100152	chr12:111099929-111100117
chr12	111214203	111214343	chr12:111214199-111214310
chr12	111217893	111217969	chr12:111217889-111217937
chr12	111263788	111263862	chr12:111263760-111263839
chr12	111270233	111270649	chr12:111270229-111270654
chr12	111291418	111291569	chr12:111291417-111291552
chr12	111293458	111293588	chr12:111293445-111293569
chr12	111295338	111295449	chr12:111295332-111295409
chr12	111296473	111296557	chr12:111296472-111296539
chr12	111298543	111298620	chr12:111298540-111298589

chr12	111304213	111304325	chr12:111304209-111304314
chr12	111306923	111307138	chr12:111306920-111307112
chr12	111307198	111307272	chr12:111307198-111307257
chr12	111308288	111308368	chr12:111308284-111308333
chr12	111308433	111308545	chr12:111308426-111308526
chr12	111310043	111310717	chr12:111310040-111310682
chr12	111312113	111312225	chr12:111312099-111312201
chr12	111320018	111320786	chr12:111320011-111320775
chr12	111322433	111322619	chr12:111322420-111322580
chr12	111334448	111334730	chr12:111334440-111334710
chr12	111338293	111338505	chr12:111338285-111338474
chr12	111341783	111342066	chr12:111341779-111342053
chr12	111347533	111348623	chr12:111347523-111350554
chr12	111348628	111350436	chr12:111347523-111350554
chr12	111350503	111350573	chr12:111347523-111350554
chr12	114353935	114354142	chr12:114353930-114356106
chr12	114354220	114355299	chr12:114353930-114356106
chr12	114355310	114355449	chr12:114353930-114356106
chr12	114355490	114356118	chr12:114353930-114356106
chr12	114366110	114366416	chr12:114366096-114366391
chr12	114385485	114385593	chr12:114385475-114385567
chr12	114390545	114390761	chr12:114390531-114390731
chr12	114394740	114394920	chr12:114394740-114394893
chr12	114398580	114398766	chr12:114398572-114398720
chr12	114399525	114399654	chr12:114399512-114399632
chr12	114401825	114401935	chr12:114401825-114401920
chr12	114403755	114403970	chr12:114403751-114403949
chr12	114405630	114406048	chr12:114405627-114406084
chr12	114407820	114408458	chr12:114407813-114408442
chr12	114672154	114672337	chr12:114672152-114672302
chr12	114674164	114674280	chr12:114674164-114674264
chr12	114681729	114681938	chr12:114681722-114681922
chr12	120978545	120979109	chr12:120978542-120979094
chr12	120988845	120989049	chr12:120988832-120989032
chr12	120993530	120993739	chr12:120993519-120993706
chr12	120994170	120994419	chr12:120994163-120994405
chr12	120996265	120996452	chr12:120996261-120996413
chr12	120996550	120996754	chr12:120996540-120996742
chr12	120997475	120998006	chr12:120997473-120998300
chr12	120999280	120999664	chr12:120999267-120999627
chr12	122078735	122079290	chr12:122078721-122079266
chr12	122099710	122099934	chr12:122099707-122099907
chr12	122127260	122127403	chr12:122127255-122127362
chr12	122127880	122129252	chr12:122127879-122129226
chr12	122129595	122129674	chr12:122129587-122129629
chr12	122129945	122130123	chr12:122129940-122130112
chr12	122130850	122130958	chr12:122130843-122130933
chr12	122132295	122132408	chr12:122132291-122132383
chr12	122132530	122134074	chr12:122132524-122134156
chr12	122134085	122134187	chr12:122132524-122134156
chr12	122134960	122135379	chr12:122134320-122135345
chr12	122135465	122135705	chr12:122135453-122135666
chr12	122137480	122137612	chr12:122137468-122137590
chr12	122138200	122138321	chr12:122138193-122138295
chr12	122138425	122138585	chr12:122138423-122138551
chr12	122138825	122138954	chr12:122138814-122138941
chr12	122140830	122141104	chr12:122140826-122140948;chr12:122140953-122141083
chr12	122141690	122143886	chr12:122141690-122147347
chr12	122143910	122144288	chr12:122141690-122147347
chr12	122144905	122146610	chr12:122141690-122147347
chr12	122146630	122147366	chr12:122141690-122147347
chr12	133037292	133037580	chr12:133037291-133037554
chr12	133037587	133037950	chr12:133037579-133037969

chr12	133038932	133039064	chr12:133038874-133039047
chr12	133041107	133041505	chr12:133041099-133041485
chr12	133047957	133048113	chr12:133047954-133048081
chr12	133048752	133048864	chr12:133048752-133048848
chr12	133050527	133050605	chr12:133050523-133050586
chr12	133052907	133053108	chr12:133052897-133053097
chr12	133056967	133057667	chr12:133056953-133063304
chr12	133057672	133057893	chr12:133056953-133063304
chr12	133057907	133058330	chr12:133056953-133063304
chr12	133058342	133058488	chr12:133056953-133063304
chr12	133058502	133060098	chr12:133056953-133063304
chr12	133060132	133060411	chr12:133056953-133063304
chr12	133060492	133061049	chr12:133056953-133063304
chr12	133061052	133061193	chr12:133056953-133063304
chr12	133061472	133063330	chr12:133056953-133063304
chr12	133079932	133081082	chr12:133079837-133081072
chr12	133081272	133081351	chr12:133081272-133081329
chr12	133081542	133083268	chr12:133081531-133083229
chr12	133083467	133083575	chr12:133083462-133083561
chr12	133084722	133084933	chr12:133084716-133084916
chr12	133093442	133093536	chr12:133093414-133093505
chr12	133100992	133101060	chr12:133100986-133101053
chr12	133105512	133106000	chr12:133105509-133106666
chr12	133106002	133106698	chr12:133105509-133106666
chr12	133130627	133131051	chr12:133130623-133131014
chr12	133134897	133135110	chr12:133134897-133135097
chr12	133139202	133139274	chr12:133139198-133139249
chr12	133143532	133143627	chr12:133143519-133143622
chr12	133143912	133144541	chr12:133143868-133144525
chr12	133151037	133151172	chr12:133151027-133151154
chr12	133151817	133151930	chr12:133151808-133151904
chr12	133155507	133156037	chr12:133155502-133159465
chr12	133156047	133156354	chr12:133155502-133159465
chr12	133156377	133159130	chr12:133155502-133159465
chr12	133159147	133159487	chr12:133155502-133159465
chr12	133181417	133181700	chr12:133181408-133181686
chr12	133181957	133182048	chr12:133181945-133182030
chr12	133187877	133188090	chr12:133187864-133188072
chr12	133191492	133191646	chr12:133191488-133191615
chr12	133191912	133192031	chr12:133191907-133192003
chr12	133194152	133194356	chr12:133194139-133194339
chr12	133202157	133203569	chr12:133202143-133214831
chr12	133203597	133205152	chr12:133202143-133214831
chr12	133205172	133205298	chr12:133202143-133214831
chr12	133205307	133207512	chr12:133202143-133214831
chr12	133207837	133208071	chr12:133202143-133214831
chr12	133208647	133208927	chr12:133202143-133214831
chr12	133209217	133209289	chr12:133202143-133214831
chr12	133209382	133209561	chr12:133202143-133214831
chr12	133209817	133211209	chr12:133202143-133214831
chr12	133211212	133211310	chr12:133202143-133214831
chr12	133211612	133211751	chr12:133202143-133214831
chr12	133211762	133212435	chr12:133202143-133214831
chr12	133212602	133212757	chr12:133202143-133214831
chr12	133213212	133213288	chr12:133202143-133214831
chr12	133213322	133213391	chr12:133202143-133214831
chr12	133213997	133214069	chr12:133202143-133214831
chr12	133214082	133214393	chr12:133202143-133214831
chr12	133214487	133214845	chr12:133202143-133214831
chr13	27792652	27793144	chr13:27792642-27793102
chr13	27793577	27794476	chr13:27793565-27794768
chr13	27794477	27794767	chr13:27793565-27794768
chr13	27920032	27920271	chr13:27920019-27920544

chr13	27920272	27920577	chr13:27920019-27920544
chr13	27922617	27922829	chr13:27922609-27922809
chr13	27924257	27924541	chr13:27924255-27926231
chr13	27924572	27925238	chr13:27924255-27926231
chr13	27925487	27925555	chr13:27924255-27926231
chr13	27925627	27926255	chr13:27924255-27926231
chr13	27962162	27963388	chr13:27962136-27963369
chr13	27964872	27965052	chr13:27964869-27965015
chr13	27967262	27967340	chr13:27967256-27967325
chr13	27968467	27969206	chr13:27968465-27971139
chr13	27969217	27971158	chr13:27968465-27971139
chr13	36168208	36169088	chr13:36168207-36169054
chr13	36170533	36170827	chr13:36170530-36170787
chr13	36173698	36173834	chr13:36173691-36173810
chr13	36174488	36174585	chr13:36174475-36174567
chr13	36174733	36174910	chr13:36174721-36174869
chr13	36181993	36182907	chr13:36181991-36183197
chr13	36189958	36190092	chr13:36189945-36190056
chr13	36191798	36191904	chr13:36191794-36191894
chr13	36193623	36193743	chr13:36193620-36193725
chr13	36193813	36193883	chr13:36193805-36193867
chr13	36201878	36202132	chr13:36201878-36202093
chr13	36213448	36213659	chr13:36213448-36213648
chr13	36214478	36214636	chr13:36214478-36214615
chr13	37009773	37009854	chr13:37009763-37009809
chr13	37010563	37010671	chr13:37010551-37010655
chr13	37012203	37012305	chr13:37012191-37012297
chr13	37017258	37017390	chr13:37017244-37017364
chr13	37019343	37019409	chr13:37019341-37019397
chr13	37021448	37021638	chr13:37021447-37021602
chr13	37022023	37022811	chr13:37022010-37023044
chr13	37022828	37022967	chr13:37022010-37023044
chr13	37022983	37023049	chr13:37022010-37023044
chr13	37024048	37024214	chr13:37024034-37024193
chr13	37024348	37024452	chr13:37024339-37024442
chr13	37025228	37025471	chr13:37025190-37025437
chr13	37026198	37026267	chr13:37026203-37026236
chr13	37028158	37028337	chr13:37028147-37028308
chr13	37029768	37029848	chr13:37029764-37029836
chr13	37031568	37031638	chr13:37031566-37031623
chr13	37031793	37031928	chr13:37031738-37031895
chr13	37033448	37033600	chr13:37033448-37033588
chr13	37040403	37040474	chr13:37040403-37040458
chr13	37040583	37040721	chr13:37040575-37040692
chr13	37044088	37044193	chr13:37044077-37044181
chr13	37045258	37045401	chr13:37045246-37045376
chr13	37047358	37047436	chr13:37047345-37047408
chr13	37047538	37047607	chr13:37047534-37047601
chr13	37047878	37047948	chr13:37047877-37047936
chr13	37048558	37048631	chr13:37048563-37048600
chr13	37049918	37050121	chr13:37049904-37050104
chr13	37051493	37051628	chr13:37051487-37051646
chr13	37056878	37057030	chr13:37056877-37057133
chr13	37059208	37059727	chr13:37059194-37059468;chr13:37059479-37059713
chr13	40469965	40470182	chr13:40469952-40470162
chr13	40555670	40558371	chr13:40555666-40559034
chr13	40558410	40558921	chr13:40555666-40559034
chr13	40558930	40559067	chr13:40555666-40559034
chr13	40559520	40560885	chr13:40559508-40560860
chr13	40562695	40562806	chr13:40562688-40562764
chr13	40572360	40572563	chr13:40572334-40572534
chr13	40611030	40611174	chr13:40611020-40611129
chr13	40665625	40666226	chr13:40665582-40666597

chr13	40666230	40666626	chr13:40665582-40666597
chr13	40932035	40934067	chr13:40932027-40934028
chr13	40940920	40941382	chr13:40940920-40941370
chr13	40942955	40943164	chr13:40942951-40943144
chr13	40943855	40943951	chr13:40943841-40943925
chr13	40949810	40949986	chr13:40949805-40949973
chr13	40951330	40951472	chr13:40951328-40951436
chr13	40958840	40959046	chr13:40958835-40959016
chr13	40981985	40982300	chr13:40981982-40982282
chr13	40982865	40982943	chr13:40982857-40982899
chr13	41006260	41006472	chr13:41006255-41006455
chr13	41019240	41019351	chr13:41019227-41019314
chr13	41060935	41061107	chr13:41060929-41061123
chr13	44433868	44434054	chr13:44433864-44434014
chr13	44434743	44434917	chr13:44434733-44434883
chr13	44436043	44436118	chr13:44436043-44436095
chr13	44563793	44564018	chr13:44563785-44563985
chr13	44574423	44574531	chr13:44574411-44574511
chr13	45783958	45784073	chr13:45783907-45784057
chr13	45848483	45848700	chr13:45848481-45848681
chr13	45851503	45851612	chr13:45851494-45851594
chr13	71438009	71439927	chr13:71437965-71440692
chr13	71439959	71440160	chr13:71437965-71440692
chr13	71440164	71440502	chr13:71437965-71440692
chr13	71440509	71440714	chr13:71437965-71440692
chr13	71475144	71475225	chr13:71475140-71475209
chr13	71475709	71475853	chr13:71475705-71475849
chr13	71479179	71479350	chr13:71479168-71479316
chr13	71488999	71489181	chr13:71488996-71489148
chr13	71557024	71557178	chr13:71557023-71557158
chr13	71559819	71559975	chr13:71559819-71559955
chr13	71572839	71573020	chr13:71572839-71573012
chr13	71573429	71573604	chr13:71573417-71573573
chr13	71604584	71604799	chr13:71604573-71604773
chr13	71630559	71630752	chr13:71630555-71630717
chr13	71681804	71681911	chr13:71681794-71681910
chr13	71865934	71866273	chr13:71865921-71867192
chr13	71866414	71866489	chr13:71865921-71867192
chr13	71866584	71867063	chr13:71865921-71867192
chr13	71867069	71867200	chr13:71865921-71867192
chr13	73063824	73063897	chr13:73063823-73063883
chr13	73066119	73066326	chr13:73066111-73066311
chr13	73075709	73075903	chr13:73075707-73075857
chr13	73686094	73686926	chr13:73686088-73695671
chr13	73686929	73687558	chr13:73686088-73695671
chr13	73687569	73687818	chr13:73686088-73695671
chr13	73687824	73689289	chr13:73686088-73695671
chr13	73689304	73690107	chr13:73686088-73695671
chr13	73690134	73692025	chr13:73686088-73695671
chr13	73692034	73693810	chr13:73686088-73695671
chr13	73693814	73695694	chr13:73686088-73695671
chr13	73715369	73715547	chr13:73715367-73715525
chr13	73764949	73765024	chr13:73764937-73765000
chr13	73813154	73813309	chr13:73813151-73813287
chr13	73837489	73837701	chr13:73837479-73837679
chr13	73845839	73846388	chr13:73845826-73846373
chr13	73943994	73944100	chr13:73943980-73944070
chr13	73994999	73995080	chr13:73994989-73995049
chr13	77761017	77761117	chr13:77761010-77761110
chr13	77763157	77763322	chr13:77763144-77763294
chr13	78598372	78598471	chr13:78598361-78602551
chr13	78598472	78599700	chr13:78598361-78602551
chr13	78599702	78600921	chr13:78598361-78602551

chr13	78600937	78601961	chr13:78598361-78602551
chr13	78601977	78602209	chr13:78598361-78602551
chr13	78602212	78602356	chr13:78598361-78602551
chr13	78602377	78602588	chr13:78598361-78602551
chr13	78603217	78603454	chr13:78603203-78603560
chr13	78603472	78603584	chr13:78603203-78603560
chr13	94709627	94709884	chr13:94709621-94712399
chr13	94709887	94710418	chr13:94709621-94712399
chr13	94710422	94711552	chr13:94709621-94712399
chr13	94711567	94711954	chr13:94709621-94712399
chr13	94712002	94712389	chr13:94709621-94712399
chr13	95548972	95549186	chr13:95548968-95549168
chr13	95560207	95560305	chr13:95560193-95560293
chr13	95560382	95560563	chr13:95560381-95560531
chr13	95577242	95577354	chr13:95577238-95577338
chr13	95577902	95578085	chr13:95577899-95578049
chr13	99962966	99963398	chr13:99962963-99965819
chr13	99963436	99963641	chr13:99962963-99965819
chr13	99963671	99963767	chr13:99962963-99965819
chr13	99963771	99964494	chr13:99962963-99965819
chr13	99964496	99965007	chr13:99962963-99965819
chr13	99965031	99965161	chr13:99962963-99965819
chr13	99965181	99965844	chr13:99962963-99965819
chr13	99967051	99967190	chr13:99967043-99967243
chr13	99967191	99967274	chr13:99967043-99967243
chr13	99970126	99970410	chr13:99970126-99971909
chr13	99970441	99970578	chr13:99970126-99971909
chr13	99970591	99971152	chr13:99970126-99971909
chr13	99971196	99971937	chr13:99970126-99971909
chr13	99981836	99982150	chr13:99981771-99983139
chr13	99982151	99982756	chr13:99981771-99983139
chr13	99982836	99983171	chr13:99981771-99983139
chr13	99984296	99984427	chr13:99984288-99984409
chr13	99984701	99984773	chr13:99984688-99984737
chr13	99984791	99985146	chr13:99984788-99985109
chr13	99985326	99985822	chr13:99985322-99986773
chr13	99985846	99985917	chr13:99985322-99986773
chr13	99985941	99986790	chr13:99985322-99986773
chr13	112067656	112067748	chr13:112067646-112070488
chr13	112067771	112067868	chr13:112067646-112070488
chr13	112067886	112068235	chr13:112067646-112070488
chr13	112068261	112068506	chr13:112067646-112070488
chr13	112068561	112069423	chr13:112067646-112070488
chr13	112069446	112070412	chr13:112067646-112070488
chr13	112070421	112070524	chr13:112067646-112070488
chr13	113584721	113584902	chr13:113584720-113584888
chr13	113585286	113585707	chr13:113585272-113585677
chr13	113585786	113585893	chr13:113585773-113585849
chr13	113585966	113586213	chr13:113585965-113586180
chr13	113611026	113611095	chr13:113610995-113611062
chr13	113623186	113623312	chr13:113623179-113623286
chr13	113631706	113631937	chr13:113631622-113631905
chr13	113633126	113633271	chr13:113633119-113633287
chr13	113633901	113634059	chr13:113633889-113634033
chr13	113634561	113634634	chr13:113634533-113634602
chr13	113635986	113636067	chr13:113635976-113636128
chr13	113636091	113636169	chr13:113635976-113636128
chr13	113636546	113636741	chr13:113636533-113636700
chr13	113637151	113637257	chr13:113637150-113637242
chr13	113637631	113637843	chr13:113637619-113637896
chr13	113637846	113637922	chr13:113637619-113637896
chr13	113640121	113640379	chr13:113640119-113641470
chr13	113640401	113640912	chr13:113640119-113641470

chr13	113640926	113641000	chr13:113640119-113641470
chr13	113641036	113641498	chr13:113640119-113641470
chr14	20455588	20455685	chr14:20455577-20455672
chr14	20456768	20456882	chr14:20456760-20456860
chr14	20456993	20457274	chr14:20456990-20457272
chr14	21090403	21091168	chr14:21090390-21091140
chr14	21091418	21091562	chr14:21091410-21091542
chr14	21091873	21093331	chr14:21091864-21093304
chr14	21093448	21093522	chr14:21093428-21093492
chr14	21093598	21093835	chr14:21093585-21093828
chr14	21094393	21094574	chr14:21094390-21094535
chr14	21097338	21097430	chr14:21097327-21097412
chr14	21098353	21098658	chr14:21098311-21098640
chr14	21104513	21104743	chr14:21104510-21104722
chr14	21521088	21523187	chr14:21521080-21525654
chr14	21523203	21523415	chr14:21521080-21525654
chr14	21523423	21524931	chr14:21521080-21525654
chr14	21524953	21525378	chr14:21521080-21525654
chr14	21525383	21525696	chr14:21521080-21525654
chr14	21526068	21526531	chr14:21526060-21526500
chr14	21533603	21533773	chr14:21533548-21533748
chr14	21536848	21537078	chr14:21536848-21537216
chr14	21537123	21537223	chr14:21536848-21537216
chr14	23117308	23117849	chr14:23117303-23117822
chr14	23118583	23119644	chr14:23118581-23119616
chr14	23272448	23272697	chr14:23272421-23272672
chr14	23273628	23274614	chr14:23273627-23277187
chr14	23274913	23275565	chr14:23273627-23277187
chr14	23275673	23277220	chr14:23273627-23277187
chr14	23284998	23285072	chr14:23284990-23285028
chr14	23285293	23285646	chr14:23285291-23285611
chr14	23285913	23286121	chr14:23285912-23286117
chr14	23289123	23289339	chr14:23289110-23289310
chr14	23293543	23293720	chr14:23293530-23293688
chr14	24080113	24081586	chr14:24080106-24081568
chr14	24081683	24082011	chr14:24081679-24081986
chr14	24082468	24082907	chr14:24082467-24082875
chr14	24084523	24084771	chr14:24084519-24084745
chr14	24086903	24087116	chr14:24086896-24087096
chr14	24093188	24093404	chr14:24093184-24093508
chr14	24162148	24162355	chr14:24162143-24162324
chr14	24162973	24163187	chr14:24162965-24163149
chr14	24163388	24163595	chr14:24163377-24163765
chr14	24164063	24164173	chr14:24164062-24164134
chr14	24164598	24164991	chr14:24164586-24164955
chr14	24165063	24165219	chr14:24165056-24165183
chr14	24165848	24166006	chr14:24165846-24165962
chr14	24365673	24365750	chr14:24365672-24365718
chr14	24366913	24367198	chr14:24366910-24367214
chr14	24367268	24367687	chr14:24367259-24367351;chr14:24367363-24367653
chr14	24368028	24368228	chr14:24368019-24368440
chr14	24368253	24368458	chr14:24368019-24368440
chr14	24369008	24370616	chr14:24368998-24370594
chr14	24372448	24372634	chr14:24372440-24372603
chr14	24372828	24373389	chr14:24372814-24373370
chr14	24373708	24373892	chr14:24373694-24373867
chr14	24374123	24374480	chr14:24374122-24374466
chr14	24375663	24375742	chr14:24375659-24375715
chr14	24375983	24376123	chr14:24375974-24376101
chr14	24376298	24377487	chr14:24376293-24379604
chr14	24377488	24379617	chr14:24376293-24379604
chr14	28763549	28763771	chr14:28763548-28763748
chr14	28765389	28766896	chr14:28765387-28770277

chr14	28766929	28767029	chr14:28765387-28770277
chr14	28767069	28767416	chr14:28765387-28770277
chr14	28767509	28769229	chr14:28765387-28770277
chr14	28769249	28770054	chr14:28765387-28770277
chr14	28770059	28770316	chr14:28765387-28770277
chr14	32934945	32935020	chr14:32934932-32935003
chr14	32939255	32939402	chr14:32939242-32939366
chr14	33055915	33056028	chr14:33055904-33055994
chr14	33213685	33214010	chr14:33213677-33215426
chr14	33214025	33214197	chr14:33213677-33215426
chr14	33214200	33215451	chr14:33213677-33215426
chr14	33215540	33215614	chr14:33215536-33215587
chr14	33367185	33367291	chr14:33367185-33367268
chr14	33560120	33560236	chr14:33560120-33560210
chr14	33567660	33567881	chr14:33567656-33567856
chr14	33582095	33582205	chr14:33582081-33582166
chr14	33583295	33583437	chr14:33583292-33583405
chr14	33608500	33608663	chr14:33608486-33608643
chr14	33610985	33611133	chr14:33610983-33611114
chr14	33612410	33612622	chr14:33612404-33612589
chr14	33676210	33676403	chr14:33676210-33676385
chr14	33680605	33681073	chr14:33680602-33681042
chr14	33735225	33735359	chr14:33735213-33735332
chr14	33774340	33774526	chr14:33774336-33774530
chr14	33778465	33778608	chr14:33778465-33778572
chr14	33780640	33780879	chr14:33780630-33780871
chr14	33793910	33794075	chr14:33793896-33794044
chr14	33797470	33797604	chr14:33797456-33797581
chr14	33799745	33800605	chr14:33799733-33804176
chr14	33800615	33801734	chr14:33799733-33804176
chr14	33801740	33801855	chr14:33799733-33804176
chr14	33801870	33802360	chr14:33799733-33804176
chr14	33802420	33803707	chr14:33799733-33804176
chr14	33803720	33803889	chr14:33799733-33804176
chr14	33803890	33803957	chr14:33799733-33804176
chr14	33803985	33804202	chr14:33799733-33804176
chr14	36516400	36516821	chr14:36516391-36518020
chr14	36516830	36517046	chr14:36516391-36518020
chr14	36517075	36518045	chr14:36516391-36518020
chr14	36518990	36519062	chr14:36518984-36519693
chr14	36519085	36519728	chr14:36518984-36519693
chr14	36520065	36520306	chr14:36520052-36520286
chr14	36520560	36521157	chr14:36520556-36521149
chr14	36580605	36581056	chr14:36580578-36581464
chr14	36581070	36581247	chr14:36580578-36581464
chr14	36581300	36581483	chr14:36580578-36581464
chr14	36582235	36582622	chr14:36582232-36582607
chr14	36657575	36657927	chr14:36657567-36657900
chr14	36660155	36660363	chr14:36660149-36660349
chr14	36661710	36662128	chr14:36661696-36662093
chr14	36662575	36663566	chr14:36662573-36663523
chr14	36665990	36666622	chr14:36665989-36666601
chr14	36671055	36671169	chr14:36671048-36671143
chr14	36671985	36672125	chr14:36671979-36672109
chr14	36676210	36677192	chr14:36676197-36678280
chr14	36677200	36678326	chr14:36676197-36678280
chr14	36678635	36679176	chr14:36678634-36679138
chr14	37589995	37591000	chr14:37589983-37592711
chr14	37591005	37591227	chr14:37589983-37592711
chr14	37591235	37592741	chr14:37589983-37592711
chr14	37593885	37594651	chr14:37593874-37594613
chr14	37594900	37595148	chr14:37594900-37595348
chr14	37595150	37595354	chr14:37594900-37595348

chr14	56799906	56800290	chr14:56799904-56802355
chr14	56800296	56800578	chr14:56799904-56802355
chr14	56800586	56801391	chr14:56799904-56802355
chr14	56801401	56802381	chr14:56799904-56802355
chr14	56804196	56804368	chr14:56804187-56804363
chr14	56805371	56805579	chr14:56805359-56805663
chr14	56805591	56805700	chr14:56805359-56805663
chr14	56808796	56809003	chr14:56808786-56808986
chr14	56810161	56810301	chr14:56810158-56810479
chr14	56810306	56810514	chr14:56810158-56810479
chr14	60508952	60510006	chr14:60508950-60509970
chr14	60511097	60511588	chr14:60511083-60512850
chr14	60511667	60512864	chr14:60511083-60512850
chr14	60643427	60645205	chr14:60643414-60646577
chr14	60645207	60645515	chr14:60643414-60646577
chr14	60645547	60645821	chr14:60643414-60646577
chr14	60645837	60646016	chr14:60643414-60646577
chr14	60646037	60646604	chr14:60643414-60646577
chr14	60647462	60647675	chr14:60647460-60647660
chr14	60648632	60649481	chr14:60648629-60649462
chr14	60651947	60652153	chr14:60651934-60652118
chr14	60652972	60653048	chr14:60652962-60653026
chr14	60658192	60658302	chr14:60658182-60658259
chr14	60709527	60710996	chr14:60709527-60714203
chr14	60711017	60711509	chr14:60709527-60714203
chr14	60711797	60713059	chr14:60709527-60714203
chr14	60713077	60714240	chr14:60709527-60714203
chr14	60719772	60720471	chr14:60719759-60720445
chr14	60720982	60721156	chr14:60720979-60721144
chr14	60722057	60722265	chr14:60722049-60722249
chr14	60723062	60723192	chr14:60723050-60723165
chr14	60723212	60724093	chr14:60723211-60724134
chr14	60724097	60724167	chr14:60723211-60724134
chr14	60724257	60724367	chr14:60724255-60724348
chr14	61695517	61695862	chr14:61695512-61695839
chr14	61697622	61697831	chr14:61697621-61697954
chr14	61697837	61697975	chr14:61697621-61697954
chr14	61698907	61698984	chr14:61698894-61698946
chr14	61705822	61706039	chr14:61705821-61706021
chr14	61720382	61720602	chr14:61720378-61720572
chr14	61721522	61721695	chr14:61721508-61721654
chr14	61721747	61721859	chr14:61721738-61721824
chr14	61726432	61726852	chr14:61726420-61726818
chr14	61727452	61727669	chr14:61727452-61727655
chr14	61732422	61732567	chr14:61732417-61732524
chr14	61734137	61734315	chr14:61734137-61734285
chr14	61736897	61736971	chr14:61736888-61737109
chr14	61736987	61737132	chr14:61736888-61737109
chr14	61738087	61738407	chr14:61738086-61738373
chr14	61740507	61740646	chr14:61740504-61740627
chr14	61740767	61741216	chr14:61740754-61741188
chr14	61744717	61744851	chr14:61744704-61744813
chr14	61745692	61745831	chr14:61745690-61745817
chr14	61746942	61747358	chr14:61746933-61748259
chr14	61747362	61747960	chr14:61746933-61748259
chr14	61747967	61748275	chr14:61746933-61748259
chr14	64084259	64084773	chr14:64084231-64085052
chr14	64084779	64085077	chr14:64084231-64085052
chr14	64226974	64228074	chr14:64226706-64228045
chr14	64231199	64231961	chr14:64230917-64235150
chr14	64231974	64233648	chr14:64230917-64235150
chr14	64233784	64233917	chr14:64230917-64235150
chr14	64233934	64234295	chr14:64230917-64235150

chr14	64234309	64235193	chr14:64230917-64235150
chr14	64249554	64249698	chr14:64249545-64249679
chr14	64257234	64257449	chr14:64257225-64257415
chr14	64260459	64260776	chr14:64260448-64260748
chr14	64268804	64268940	chr14:64268794-64268911
chr14	64279994	64280169	chr14:64279980-64280153
chr14	64282624	64283085	chr14:64282623-64283075
chr14	64291404	64291548	chr14:64291402-64291602
chr14	64291549	64291623	chr14:64291402-64291602
chr14	64294039	64294422	chr14:64294032-64294410
chr14	64297534	64297745	chr14:64297532-64297718
chr14	64298404	64298520	chr14:64298390-64298484
chr14	64301374	64301619	chr14:64301367-64301580
chr14	64303489	64303698	chr14:64303475-64303659
chr14	64336419	64336737	chr14:64336412-64336720
chr14	64337899	64338149	chr14:64337897-64338112
chr14	64504004	64504114	chr14:64503995-64504091
chr14	64511254	64511403	chr14:64511248-64511384
chr14	64515059	64515276	chr14:64515051-64515251
chr14	64516599	64516759	chr14:64516599-64516751
chr14	64521489	64523928	chr14:64521486-64523898
chr14	64531864	64532995	chr14:64531860-64533690
chr14	64533004	64533380	chr14:64531860-64533690
chr14	64533384	64533722	chr14:64531860-64533690
chr14	65006174	65006307	chr14:65006173-65006284
chr14	65029219	65029456	chr14:65029218-65029418
chr14	65075133	65075964	chr14:65075123-65078036
chr14	65075968	65076342	chr14:65075123-65078036
chr14	65076363	65077301	chr14:65075123-65078036
chr14	65077303	65078073	chr14:65075123-65078036
chr14	65083743	65084309	chr14:65083743-65084299
chr14	65093721	65093827	chr14:65093707-65093815
chr14	65102306	65102447	chr14:65102303-65102695
chr14	65102481	65102718	chr14:65102303-65102695
chr14	67019756	67019971	chr14:67019747-67019947
chr14	67023641	67023721	chr14:67023632-67023675
chr14	67058651	67058828	chr14:67058648-67058798
chr14	67179591	67179694	chr14:67179577-67179674
chr14	67180806	67180999	chr14:67180803-67180953
chr14	67554001	67554222	chr14:67553999-67554199
chr14	67586001	67586121	chr14:67585997-67586097
chr14	67587076	67587256	chr14:67587073-67587223
chr14	72619307	72619414	chr14:72619295-72619367
chr14	72619912	72620021	chr14:72619902-72619984
chr14	72629637	72629771	chr14:72629623-72629736
chr14	72661052	72661851	chr14:72661042-72662833
chr14	72661867	72662445	chr14:72661042-72662833
chr14	72662452	72662581	chr14:72661042-72662833
chr14	72662582	72662725	chr14:72661042-72662833
chr14	72662737	72662872	chr14:72661042-72662833
chr14	72669952	72670629	chr14:72669951-72671338
chr14	72670647	72670899	chr14:72669951-72671338
chr14	72670982	72671048	chr14:72669951-72671338
chr14	72671062	72671385	chr14:72669951-72671338
chr14	72674022	72674385	chr14:72674021-72674368
chr14	72675452	72675808	chr14:72675446-72675771
chr14	72679212	72679315	chr14:72679201-72679302
chr14	72693087	72693226	chr14:72693075-72693213
chr14	72714422	72714546	chr14:72714422-72714504
chr14	72723632	72723738	chr14:72723631-72723771
chr14	72723967	72724104	chr14:72723964-72724105
chr14	72731427	72731702	chr14:72731417-72731667
chr14	72731807	72731955	chr14:72731806-72731934

chr14	72742452	72742741	chr14:72742441-72742711
chr14	72753267	72753392	chr14:72753263-72753371
chr14	72771737	72771915	chr14:72771732-72771893
chr14	72806022	72806091	chr14:72806009-72806057
chr14	72806782	72806885	chr14:72806774-72806872
chr14	72836092	72836519	chr14:72836083-72836483
chr14	72877517	72877736	chr14:72877507-72877707
chr14	72879817	72879984	chr14:72879803-72879947
chr14	72892157	72892672	chr14:72892146-72892649
chr14	72894067	72894144	chr14:72894056-72894116
chr14	74239473	74239969	chr14:74239471-74239931
chr14	74241193	74241288	chr14:74241181-74241266
chr14	74245168	74245312	chr14:74245164-74245288
chr14	74258268	74258476	chr14:74258268-74258468
chr14	74259613	74259818	chr14:74259601-74259782
chr14	74260593	74262771	chr14:74260593-74262738
chr14	74763378	74764115	chr14:74763365-74764362
chr14	74764118	74764402	chr14:74763365-74764362
chr14	74778448	74778703	chr14:74778446-74778683
chr14	74780418	74780628	chr14:74780404-74780584
chr14	74781343	74782346	chr14:74781333-74782325
chr14	74797593	74799725	chr14:74797579-74799697
chr14	74802563	74802709	chr14:74802555-74802676
chr14	74809383	74809838	chr14:74809379-74809797
chr14	74809918	74810020	chr14:74809909-74810002
chr14	74810228	74810445	chr14:74810224-74810420
chr14	74811628	74811776	chr14:74811619-74811738
chr14	74812638	74812817	chr14:74812627-74812782
chr14	74816208	74816285	chr14:74816202-74816265
chr14	74816573	74816724	chr14:74816570-74816690
chr14	74816938	74817115	chr14:74816930-74817107
chr14	74817193	74817308	chr14:74817193-74817277
chr14	74818243	74818352	chr14:74818230-74818314
chr14	74821098	74822139	chr14:74821056-74822115
chr14	74822958	74823173	chr14:74822946-74823146
chr14	74824258	74824330	chr14:74824255-74824307
chr14	74827373	74828044	chr14:74827371-74828032
chr14	74829213	74829362	chr14:74829212-74829343
chr14	74835278	74835482	chr14:74835264-74835447
chr14	74835783	74836212	chr14:74835774-74836281
chr14	74836218	74836318	chr14:74835774-74836281
chr14	74844028	74844283	chr14:74844023-74844260
chr14	74855458	74856690	chr14:74855452-74856650
chr14	74857148	74857720	chr14:74857145-74858256
chr14	74858053	74858269	chr14:74857145-74858256
chr14	74858673	74858782	chr14:74858664-74859435
chr14	74858883	74858981	chr14:74858664-74859435
chr14	74858983	74859404	chr14:74858664-74859435
chr14	75278773	75279132	chr14:75278773-75279123
chr14	75279643	75282069	chr14:75279642-75282230
chr14	75282118	75282262	chr14:75279642-75282230
chr14	75427718	75427863	chr14:75427715-75427849
chr14	75428053	75428266	chr14:75428045-75428252
chr14	75428368	75428638	chr14:75428356-75428606
chr14	75432143	75432353	chr14:75432133-75432336
chr14	75437898	75438155	chr14:75437897-75438121
chr14	75445098	75445423	chr14:75445089-75445379
chr14	75449463	75449682	chr14:75449460-75449660
chr14	75461433	75461541	chr14:75461425-75461530
chr14	75469298	75469486	chr14:75469289-75474111
chr14	75469503	75470169	chr14:75469289-75474111
chr14	75470193	75470963	chr14:75469289-75474111
chr14	75470988	75473084	chr14:75469289-75474111

chr14	75473348	75473550	chr14:75469289-75474111
chr14	75473553	75473967	chr14:75469289-75474111
chr14	75473968	75474144	chr14:75469289-75474111
chr14	75522448	75522765	chr14:75522424-75522745
chr14	75525088	75525199	chr14:75525083-75525188
chr14	75526253	75526467	chr14:75526245-75526445
chr14	75546473	75547031	chr14:75546461-75547015
chr14	76382173	76382388	chr14:76382170-76382370
chr14	76491618	76491724	chr14:76491616-76491716
chr14	76498213	76498410	chr14:76498213-76498363
chr14	76499978	76500095	chr14:76499966-76500066
chr14	76500698	76500882	chr14:76500694-76500844
chr14	81059297	81059507	chr14:81059287-81059487
chr14	81096652	81096730	chr14:81096638-81096707
chr14	81108387	81108487	chr14:81108374-81108524
chr14	81108552	81108787	chr14:81108548-81108761
chr14	81139777	81139874	chr14:81139767-81139867
chr14	81142952	81143120	chr14:81142939-81143089
chr14	89124941	89125270	chr14:89124870-89125226
chr14	89125341	89125481	chr14:89125327-89125440
chr14	89156196	89156637	chr14:89156171-89162969
chr14	89156661	89160872	chr14:89156171-89162969
chr14	89160951	89161115	chr14:89156171-89162969
chr14	89161136	89161275	chr14:89156171-89162969
chr14	89161316	89161386	chr14:89156171-89162969
chr14	89161396	89161527	chr14:89156171-89162969
chr14	89161591	89162247	chr14:89156171-89162969
chr14	89162326	89162797	chr14:89156171-89162969
chr14	89162816	89163009	chr14:89156171-89162969
chr14	89175411	89175640	chr14:89175410-89175610
chr14	89180711	89180843	chr14:89180700-89180806
chr14	89183946	89184168	chr14:89183945-89184154
chr14	89185546	89185899	chr14:89185536-89185873
chr14	89190381	89190463	chr14:89190381-89190449
chr14	89223121	89223373	chr14:89223116-89223342
chr14	89230731	89230871	chr14:89230727-89230867
chr14	89263606	89263785	chr14:89263592-89264055
chr14	89280951	89281027	chr14:89280949-89281014
chr14	89284436	89284550	chr14:89284427-89284522
chr14	89349536	89349791	chr14:89349532-89349761
chr14	89350671	89350817	chr14:89350671-89350808
chr14	89351066	89351173	chr14:89351062-89351321
chr14	89351176	89351346	chr14:89351062-89351321
chr14	89401581	89401679	chr14:89401569-89401665
chr14	89411946	89412511	chr14:89411933-89412492
chr14	89416871	89417129	chr14:89416870-89417112
chr14	89486571	89486697	chr14:89486568-89486671
chr14	89493956	89494095	chr14:89493943-89494051
chr14	89494131	89494219	chr14:89494129-89494185
chr14	89619041	89619179	chr14:89619027-89619149
chr14	94768226	94768672	chr14:94768215-94768649
chr14	94768961	94769243	chr14:94768957-94769217
chr14	94769671	94770264	chr14:94769660-94770230
chr14	99169294	99170513	chr14:99169286-99176195
chr14	99170579	99171417	chr14:99169286-99176195
chr14	99171429	99171604	chr14:99169286-99176195
chr14	99171614	99171958	chr14:99169286-99176195
chr14	99171959	99172185	chr14:99169286-99176195
chr14	99172189	99172396	chr14:99169286-99176195
chr14	99172409	99172472	chr14:99169286-99176195
chr14	99172489	99172593	chr14:99169286-99176195
chr14	99172604	99172841	chr14:99169286-99176195
chr14	99172854	99173545	chr14:99169286-99176195

chr14	99173624	99173802	chr14:99169286-99176195
chr14	99173809	99173873	chr14:99169286-99176195
chr14	99173874	99175202	chr14:99169286-99176195
chr14	99175229	99176218	chr14:99169286-99176195
chr14	99231344	99231592	chr14:99231344-99231557
chr14	99245479	99245691	chr14:99245470-99245670
chr14	99257474	99257865	chr14:99257470-99257839
chr14	99271174	99271308	chr14:99271160-99271524
chr14	99271324	99271389	chr14:99271160-99271524
chr14	99271459	99271521	chr14:99271160-99271524
chr14	100238299	100238589	chr14:100238297-100238790
chr14	100238609	100238826	chr14:100238297-100238790
chr14	100238989	100239378	chr14:100238984-100239923
chr14	100239389	100239457	chr14:100238984-100239923
chr14	100239479	100239940	chr14:100238984-100239923
chr14	100246549	100246764	chr14:100246538-100246738
chr14	100262314	100262491	chr14:100262303-100262466
chr14	100274709	100274783	chr14:100274697-100274758
chr14	100276494	100276668	chr14:100276489-100276648
chr14	100277419	100277735	chr14:100277417-100282792
chr14	100277764	100277972	chr14:100277417-100282792
chr14	100277974	100278078	chr14:100277417-100282792
chr14	100278079	100280962	chr14:100277417-100282792
chr14	100281079	100282824	chr14:100277417-100282792
chr14	102592669	102592958	chr14:102592660-102593187
chr14	102592964	102593216	chr14:102592660-102593187
chr14	102593269	102593338	chr14:102593265-102593325
chr14	102681904	102682005	chr14:102681894-102681978
chr14	102683779	102683995	chr14:102683778-102683978
chr14	102701279	102701354	chr14:102701277-102701330
chr14	102707354	102707533	chr14:102707350-102707512
chr14	102708474	102708612	chr14:102708464-102708583
chr14	102710934	102711036	chr14:102710934-102711013
chr14	102714434	102714650	chr14:102714422-102714617
chr14	102721019	102721126	chr14:102721006-102721084
chr14	102721324	102721398	chr14:102721319-102721377
chr14	102722189	102722436	chr14:102722186-102722416
chr14	102726479	102727943	chr14:102726467-102730576
chr14	102727944	102728080	chr14:102726467-102730576
chr14	102728094	102730587	chr14:102726467-102730576
chr14	103940963	103941132	chr14:103940953-103941103
chr14	103941398	103941509	chr14:103941396-103941496
chr14	104800608	104800711	chr14:104800595-104800700
chr14	104800913	104804145	chr14:104800912-104804712
chr14	104804168	104804734	chr14:104800912-104804712
chr15	27328811	27328930	chr15:27328805-27328888
chr15	27457550	27457769	chr15:27457548-27457748
chr15	27480650	27480831	chr15:27480649-27480799
chr15	27527945	27528021	chr15:27527932-27527992
chr15	27532610	27532791	chr15:27532599-27532749
chr15	31326867	31327073	chr15:31326854-31327789
chr15	31327087	31327816	chr15:31326854-31327789
chr15	31339942	31340017	chr15:31339932-31339987
chr15	31343967	31344179	chr15:31343953-31344153
chr15	31366157	31366587	chr15:31366153-31366554
chr15	31372012	31372441	chr15:31372009-31377899
chr15	31372467	31374725	chr15:31372009-31377899
chr15	31374732	31377939	chr15:31372009-31377899
chr15	31392852	31393267	chr15:31392842-31393255
chr15	31393587	31393735	chr15:31393574-31393691
chr15	31403437	31404340	chr15:31403427-31404729
chr15	31404627	31404702	chr15:31403427-31404729
chr15	31435377	31435707	chr15:31435369-31435665

chr15	35084258	35084334	chr15:35084192-35085110
chr15	35084433	35084586	chr15:35084192-35085110
chr15	35084598	35084676	chr15:35084192-35085110
chr15	35084798	35084936	chr15:35084192-35085110
chr15	35084988	35085130	chr15:35084192-35085110
chr15	36889206	36890373	chr15:36889203-36892459
chr15	36890381	36892479	chr15:36889203-36892459
chr15	36894726	36894831	chr15:36894716-36894812
chr15	36895161	36895301	chr15:36895148-36895261
chr15	36896636	36896716	chr15:36896627-36896686
chr15	36898366	36898444	chr15:36898363-36898420
chr15	36898736	36898806	chr15:36898726-36898768
chr15	36901001	36901177	chr15:36900999-36901156
chr15	36903946	36904094	chr15:36903940-36904067
chr15	36950336	36950430	chr15:36950323-36950400
chr15	36994396	36994623	chr15:36994393-36994593
chr15	37036816	37036992	chr15:37036813-37036959
chr15	37066306	37066542	chr15:37066296-37066537
chr15	37083781	37083922	chr15:37083770-37083885
chr15	37093591	37093766	chr15:37093580-37093730
chr15	37094536	37094610	chr15:37094526-37094577
chr15	37095576	37095648	chr15:37095563-37095614
chr15	37096316	37096460	chr15:37096288-37096506
chr15	37096466	37096536	chr15:37096288-37096506
chr15	37097271	37097382	chr15:37097270-37097367
chr15	37097966	37098326	chr15:37097966-37098321
chr15	37098901	37099162	chr15:37098900-37099396
chr15	37099176	37099436	chr15:37098900-37099396
chr15	37099456	37099561	chr15:37099454-37100530
chr15	37099566	37099654	chr15:37099454-37100530
chr15	37099751	37099815	chr15:37099454-37100530
chr15	37099836	37100067	chr15:37099454-37100530
chr15	37100076	37100239	chr15:37099454-37100530
chr15	37100316	37100560	chr15:37099454-37100530
chr15	37100901	37101287	chr15:37100891-37101299
chr15	40212562	40212667	chr15:40212548-40212648
chr15	40213337	40213514	chr15:40213331-40213474
chr15	40214212	40214417	chr15:40214206-40214406
chr15	41335432	41335538	chr15:41335421-41335621
chr15	41377212	41377283	chr15:41377204-41377304
chr15	41380092	41380231	chr15:41380092-41380242
chr15	41621237	41621334	chr15:41621223-41621298
chr15	41621547	41621624	chr15:41621535-41621589
chr15	41660422	41660557	chr15:41660411-41660525
chr15	41668827	41669986	chr15:41668827-41669958
chr15	41684397	41684718	chr15:41684393-41684690
chr15	41696082	41697057	chr15:41696074-41697023
chr15	41698862	41698971	chr15:41698862-41698941
chr15	41699077	41699182	chr15:41699063-41699159
chr15	41707737	41707877	chr15:41707727-41707859
chr15	41708107	41708217	chr15:41708103-41708208
chr15	41710702	41711378	chr15:41710690-41711349
chr15	41713157	41713442	chr15:41713150-41713496
chr15	41727192	41727427	chr15:41727179-41727406
chr15	41729167	41729381	chr15:41729163-41729349
chr15	41731087	41731294	chr15:41731087-41731287
chr15	41734532	41734631	chr15:41734521-41734594
chr15	41736192	41736711	chr15:41736180-41736698
chr15	41739912	41740232	chr15:41739905-41740203
chr15	41742552	41743192	chr15:41742545-41743172
chr15	41748682	41748958	chr15:41748636-41748927
chr15	41749117	41750633	chr15:41749110-41750615
chr15	41754447	41754583	chr15:41754436-41754567

chr15	41757787	41757865	chr15:41757787-41757839
chr15	41760322	41760545	chr15:41760322-41760529
chr15	41761742	41761886	chr15:41761738-41761850
chr15	41762132	41762372	chr15:41762128-41762362
chr15	41764887	41765102	chr15:41764885-41765062
chr15	41766012	41766549	chr15:41766003-41773081
chr15	41766567	41769296	chr15:41766003-41773081
chr15	41769322	41769802	chr15:41766003-41773081
chr15	41769822	41770840	chr15:41766003-41773081
chr15	41771132	41771566	chr15:41766003-41773081
chr15	41771577	41771890	chr15:41766003-41773081
chr15	41771892	41772353	chr15:41766003-41773081
chr15	41772647	41773100	chr15:41766003-41773081
chr15	48121074	48121180	chr15:48121065-48121165
chr15	48121859	48122050	chr15:48121856-48122006
chr15	48130349	48130565	chr15:48130339-48130539
chr15	48134634	48135700	chr15:48134630-48143071
chr15	48135719	48136454	chr15:48134630-48143071
chr15	48136474	48139090	chr15:48134630-48143071
chr15	48139099	48139512	chr15:48134630-48143071
chr15	48139544	48140353	chr15:48134630-48143071
chr15	48140384	48141287	chr15:48134630-48143071
chr15	48141604	48141869	chr15:48134630-48143071
chr15	48141874	48142194	chr15:48134630-48143071
chr15	48142204	48143105	chr15:48134630-48143071
chr15	48149034	48149105	chr15:48149031-48149083
chr15	48149169	48149388	chr15:48149162-48149371
chr15	48151099	48151207	chr15:48151099-48151171
chr15	48151479	48151593	chr15:48151472-48151571
chr15	48151884	48151957	chr15:48151873-48151942
chr15	48152234	48152308	chr15:48152233-48152284
chr15	48152319	48152590	chr15:48152306-48153893
chr15	48152609	48153228	chr15:48152306-48153893
chr15	48153249	48153920	chr15:48152306-48153893
chr15	48157149	48157463	chr15:48157135-48157898
chr15	48157469	48157932	chr15:48157135-48157898
chr15	48157994	48158070	chr15:48157992-48158056
chr15	48158179	48158258	chr15:48158174-48158224
chr15	48158769	48158945	chr15:48158768-48158922
chr15	48159599	48159826	chr15:48159595-48159804
chr15	48162764	48162833	chr15:48162694-48162893
chr15	48165934	48166039	chr15:48165932-48166027
chr15	48167344	48167417	chr15:48167341-48167401
chr15	48168634	48168842	chr15:48168629-48168839
chr15	48176864	48177077	chr15:48176859-48177059
chr15	48178079	48178544	chr15:48178076-48178517
chr15	52756994	52757487	chr15:52756988-52757847
chr15	52757514	52757857	chr15:52756988-52757847
chr15	52780599	52780933	chr15:52780595-52781183
chr15	52780934	52781203	chr15:52780595-52781183
chr15	52783659	52783872	chr15:52783658-52783858
chr15	52785724	52785976	chr15:52785724-52785969
chr15	52787014	52787163	chr15:52787006-52787127
chr15	52787764	52788546	chr15:52787760-52788514
chr15	52788789	52789488	chr15:52788779-52790012
chr15	52789509	52789654	chr15:52788779-52790012
chr15	52789669	52790001	chr15:52788779-52790012
chr15	52791039	52791117	chr15:52791028-52791078
chr15	56087291	56088538	chr15:56087279-56096620
chr15	56088581	56090573	chr15:56087279-56096620
chr15	56090576	56095458	chr15:56087279-56096620
chr15	56095476	56096628	chr15:56087279-56096620
chr15	56098081	56098404	chr15:56098080-56098376

chr15	56101371	56101578	chr15:56101358-56101566
chr15	56102176	56102278	chr15:56102168-56102251
chr15	56103556	56103704	chr15:56103551-56103670
chr15	56142781	56142941	chr15:56142777-56142900
chr15	56144411	56144504	chr15:56144400-56144483
chr15	56160706	56160783	chr15:56160715-56160747
chr15	56179066	56179166	chr15:56179058-56179133
chr15	56179261	56179331	chr15:56179269-56179303
chr15	56189846	56189923	chr15:56189834-56189886
chr15	56226131	56226355	chr15:56226131-56226331
chr15	56243126	56243313	chr15:56243124-56243266
chr15	56918731	56918931	chr15:56918721-56918906
chr15	56919161	56919259	chr15:56919147-56919228
chr15	56919521	56920015	chr15:56919516-56919988
chr15	56921036	56921137	chr15:56921025-56921098
chr15	57001351	57001423	chr15:57001348-57001384
chr15	57063761	57063860	chr15:57063749-57063823
chr15	57072656	57072730	chr15:57072652-57072714
chr15	57091801	57091904	chr15:57091788-57091891
chr15	57109266	57109341	chr15:57109257-57109317
chr15	57118361	57118461	chr15:57118349-57118433
chr15	57129796	57129999	chr15:57129782-57129972
chr15	57134321	57134533	chr15:57134315-57134500
chr15	57166401	57166479	chr15:57166401-57166466
chr15	57192171	57192314	chr15:57192157-57192293
chr15	57197776	57197844	chr15:57197772-57197825
chr15	57214081	57214436	chr15:57214078-57214396
chr15	57219036	57219144	chr15:57219036-57219590
chr15	57219176	57219623	chr15:57219036-57219590
chr15	57228256	57228454	chr15:57228246-57228446
chr15	57231161	57231297	chr15:57231151-57231257
chr15	57232291	57232449	chr15:57232290-57232430
chr15	57232721	57232896	chr15:57232711-57232856
chr15	57234056	57234141	chr15:57234042-57234107
chr15	57243471	57243581	chr15:57243471-57243553
chr15	57248041	57248116	chr15:57248043-57248081
chr15	57251181	57251459	chr15:57251168-57251423
chr15	57252201	57252521	chr15:57252198-57252492
chr15	57253266	57253483	chr15:57253261-57253468
chr15	57262106	57262237	chr15:57262093-57262208
chr15	57263111	57263293	chr15:57263111-57263274
chr15	57273031	57273290	chr15:57273029-57273262
chr15	57282196	57282616	chr15:57282182-57282598
chr15	57286156	57286793	chr15:57286156-57289853
chr15	57286806	57289607	chr15:57286156-57289853
chr15	57289631	57289810	chr15:57286156-57289853
chr15	57290831	57291160	chr15:57290828-57291124
chr15	57299236	57299318	chr15:57299225-57299281
chr15	59110371	59110588	chr15:59110359-59110559
chr15	59117281	59117392	chr15:59117268-59117368
chr15	59123516	59123694	chr15:59123516-59123666
chr15	59124771	59124952	chr15:59124766-59124916
chr15	60004231	60004681	chr15:60004221-60004643
chr15	60004906	60005245	chr15:60004906-60007441
chr15	60005266	60006907	chr15:60004906-60007441
chr15	60006936	60007240	chr15:60004906-60007441
chr15	60007246	60007445	chr15:60004906-60007441
chr15	60011946	60012152	chr15:60011937-60012137
chr15	60025766	60025945	chr15:60025755-60025909
chr15	60036206	60036274	chr15:60036109-60036242
chr15	60041616	60041934	chr15:60041611-60041899
chr15	60061661	60061759	chr15:60061647-60061730
chr15	60488286	60488496	chr15:60488283-60497619

chr15	60488516	60490164	chr15:60488283-60497619
chr15	60490181	60492038	chr15:60488283-60497619
chr15	60492051	60492637	chr15:60488283-60497619
chr15	60492641	60493295	chr15:60488283-60497619
chr15	60493326	60493636	chr15:60488283-60497619
chr15	60493681	60493842	chr15:60488283-60497619
chr15	60493851	60493957	chr15:60488283-60497619
chr15	60493981	60495087	chr15:60488283-60497619
chr15	60495141	60495865	chr15:60488283-60497619
chr15	60495866	60496457	chr15:60488283-60497619
chr15	60496461	60496594	chr15:60488283-60497619
chr15	60496611	60497391	chr15:60488283-60497619
chr15	60497406	60497649	chr15:60488283-60497619
chr15	60499891	60500040	chr15:60499891-60500004
chr15	60500961	60501101	chr15:60500958-60501069
chr15	60502761	60502907	chr15:60502759-60502867
chr15	60503546	60503685	chr15:60503534-60503667
chr15	60505521	60505661	chr15:60505507-60505629
chr15	60511236	60511660	chr15:60511225-60511621
chr15	60512041	60512117	chr15:60512032-60512097
chr15	60514626	60514796	chr15:60514615-60514757
chr15	60531776	60531884	chr15:60531765-60531851
chr15	60556864	60556965	chr15:60556852-60556928
chr15	60592389	60592567	chr15:60592386-60592541
chr15	60592814	60593159	chr15:60592807-60593126
chr15	60614904	60615044	chr15:60614893-60615027
chr15	60627249	60627503	chr15:60627237-60627463
chr15	60650629	60650728	chr15:60650620-60650708
chr15	60678659	60678735	chr15:60678656-60678686
chr15	60706244	60706387	chr15:60706231-60706352
chr15	60746659	60746865	chr15:60746648-60746848
chr15	61038829	61039142	chr15:61038825-61039127
chr15	61041099	61041174	chr15:61041103-61041134
chr15	61176079	61176495	chr15:61176067-61176456
chr15	61229064	61229308	chr15:61229052-61229319
chr15	65658048	65658479	chr15:65658045-65658437
chr15	65659123	65659329	chr15:65659122-65660128
chr15	65659608	65660163	chr15:65659122-65660128
chr15	65660503	65660575	chr15:65660488-65660542
chr15	65661153	65661255	chr15:65661145-65661987
chr15	65661258	65661384	chr15:65661145-65661987
chr15	65661393	65661626	chr15:65661145-65661987
chr15	65661648	65662002	chr15:65661145-65661987
chr15	65664343	65664418	chr15:65664329-65664394
chr15	65664563	65664749	chr15:65664559-65664722
chr15	65665348	65665503	chr15:65665344-65665462
chr15	65667458	65667727	chr15:65667448-65667703
chr15	65667938	65668139	chr15:65667924-65668123
chr15	65669778	65669957	chr15:65669778-65669925
chr15	65670013	65670228	chr15:65670012-65670188
chr15	65671793	65671903	chr15:65671791-65671886
chr15	65676458	65676660	chr15:65676444-65676634
chr15	65680648	65680908	chr15:65680634-65681314
chr15	65680913	65681030	chr15:65680634-65681314
chr15	65681228	65681334	chr15:65680634-65681314
chr15	65690418	65691543	chr15:65690414-65691511
chr15	65696373	65696517	chr15:65696365-65696497
chr15	65697268	65697387	chr15:65697266-65697383
chr15	65700553	65700709	chr15:65700543-65700675
chr15	65701053	65701204	chr15:65701050-65701192
chr15	65701773	65701916	chr15:65701761-65701890
chr15	65702308	65702520	chr15:65702304-65702511
chr15	65702878	65703014	chr15:65702872-65703008

chr15	65706103	65706235	chr15:65706090-65706224
chr15	65710113	65710334	chr15:65710113-65710313
chr15	65715483	65715656	chr15:65715477-65715623
chr15	65717788	65718037	chr15:65717777-65717996
chr15	65722853	65722958	chr15:65722847-65722948
chr15	65729083	65729262	chr15:65729071-65729247
chr15	65729543	65729709	chr15:65729533-65729678
chr15	65731643	65731722	chr15:65731641-65731700
chr15	65732753	65732826	chr15:65732751-65732818
chr15	65737708	65737962	chr15:65737706-65737945
chr15	65738708	65738887	chr15:65738705-65738875
chr15	65741718	65741791	chr15:65741714-65741784
chr15	65752378	65752629	chr15:65752378-65752628
chr15	65756153	65756502	chr15:65756139-65756472
chr15	65761363	65761474	chr15:65761359-65761438
chr15	65792018	65792331	chr15:65792009-65792293
chr15	66716431	66716538	chr15:66716420-66716498
chr15	66762826	66763043	chr15:66762812-66763012
chr15	66780996	66781182	chr15:66780996-66781146
chr15	67063766	67063979	chr15:67063762-67063944
chr15	67065846	67066135	chr15:67065844-67066360
chr15	67066146	67066385	chr15:67065844-67066360
chr15	67082951	67083168	chr15:67082944-67083144
chr15	67098586	67099048	chr15:67098581-67099024
chr15	67125716	67126009	chr15:67125715-67125984
chr15	67128116	67128188	chr15:67128102-67128151
chr15	67138011	67138110	chr15:67138000-67138100
chr15	67138236	67138552	chr15:67138234-67138522
chr15	67164896	67165113	chr15:67164894-67165088
chr15	67165256	67165411	chr15:67165252-67165384
chr15	67166021	67166206	chr15:67166018-67166193
chr15	67166531	67166896	chr15:67166530-67166853
chr15	67170561	67170635	chr15:67170553-67170604
chr15	67181251	67181491	chr15:67181240-67181453
chr15	67184726	67184875	chr15:67184726-67184864
chr15	67187031	67187169	chr15:67187019-67187130
chr15	67187371	67187548	chr15:67187364-67187509
chr15	67190416	67191939	chr15:67190412-67195195
chr15	67191941	67193697	chr15:67190412-67195195
chr15	67193701	67195231	chr15:67190412-67195195
chr15	71792643	71792760	chr15:71792637-71792722
chr15	71807028	71807236	chr15:71807017-71807204
chr15	71808318	71808542	chr15:71808311-71808511
chr15	71809278	71809354	chr15:71809275-71809345
chr15	71810553	71810883	chr15:71810553-71810861
chr15	71811493	71811634	chr15:71811482-71811609
chr15	71811778	71811878	chr15:71811765-71811869
chr15	71811958	71812216	chr15:71811954-71812176
chr15	71812338	71812518	chr15:71812335-71812511
chr15	71813388	71813674	chr15:71813388-71813635
chr15	71814018	71814947	chr15:71814011-71814929
chr15	71817473	71817828	chr15:71817459-71818259
chr15	71817838	71818224	chr15:71817459-71818259
chr15	74410844	74411015	chr15:74410835-74410985
chr15	74411304	74411381	chr15:74411294-74411356
chr15	74420824	74421046	chr15:74420821-74421021
chr15	74541179	74541359	chr15:74541176-74541330
chr15	74543859	74543937	chr15:74543859-74544488
chr15	74543964	74544529	chr15:74543859-74544488
chr15	74563729	74563942	chr15:74563719-74563919
chr15	74572874	74572952	chr15:74572861-74572933
chr15	74573134	74573251	chr15:74573131-74573204
chr15	74589824	74590039	chr15:74589819-74590003

chr15	74591154	74591470	chr15:74591150-74591434
chr15	74591569	74591854	chr15:74591559-74591814
chr15	74593144	74593251	chr15:74593134-74593236
chr15	74595684	74596795	chr15:74595610-74598131
chr15	74596809	74597046	chr15:74595610-74598131
chr15	74597049	74597505	chr15:74595610-74598131
chr15	74597524	74598161	chr15:74595610-74598131
chr15	76336734	76336942	chr15:76336723-76336941
chr15	76337779	76337993	chr15:76337777-76337967
chr15	76338264	76338544	chr15:76338251-76338514
chr15	76340284	76340609	chr15:76340275-76340559
chr15	76341144	76341314	chr15:76341133-76341301
chr15	76341719	76342504	chr15:76341718-76342476
chr15	77420422	77421123	chr15:77420411-77421004;chr15:77421010-77421094
chr15	77447722	77447835	chr15:77447718-77447811
chr15	77458222	77458290	chr15:77458216-77458284
chr15	77458407	77458583	chr15:77458403-77458558
chr15	77464242	77464428	chr15:77464239-77464387
chr15	77467102	77467341	chr15:77467094-77467307
chr15	77470922	77471052	chr15:77470909-77471042
chr15	77471782	77471868	chr15:77471782-77471814
chr15	77475197	77475417	chr15:77475185-77475385
chr15	77477562	77477664	chr15:77477554-77477630
chr15	77478297	77478555	chr15:77478292-77478510
chr15	77479182	77479505	chr15:77479178-77479604
chr15	77482977	77484094	chr15:77482969-77485607
chr15	77484232	77484323	chr15:77482969-77485607
chr15	77484337	77485384	chr15:77482969-77485607
chr15	77485387	77485623	chr15:77482969-77485607
chr15	78173691	78173882	chr15:78173689-78173839
chr15	78174396	78174510	chr15:78174384-78174484
chr15	78201581	78201789	chr15:78201572-78201772
chr15	80466979	80467192	chr15:80466969-80467169
chr15	80591614	80591721	chr15:80591604-80591704
chr15	80593609	80593789	chr15:80593599-80593749
chr15	80597194	80597378	chr15:80597194-80597344
chr15	83255916	83256741	chr15:83255902-83258126
chr15	83256746	83258141	chr15:83255902-83258126
chr15	83262961	83264851	chr15:83262950-83264815
chr15	83266841	83267084	chr15:83266835-83267071
chr15	83268146	83268240	chr15:83268132-83268232
chr15	83280851	83281074	chr15:83280850-83281050
chr15	83283146	83283350	chr15:83283134-83283319
chr15	83284536	83284752	chr15:83284529-83284714
chr15	83580928	83581149	chr15:83580920-83581120
chr15	83588683	83588786	chr15:83588671-83588771
chr15	83618093	83618264	chr15:83618081-83618231
chr15	84764706	84764852	chr15:84764706-84764815
chr15	84771776	84771993	chr15:84771762-84771962
chr15	84778261	84778336	chr15:84778182-84778312
chr15	84782656	84784906	chr15:84782653-84784895
chr15	84790711	84790927	chr15:84790704-84790883
chr15	84797876	84798060	chr15:84797868-84798045
chr15	84798321	84798499	chr15:84798314-84798474
chr15	84798601	84798916	chr15:84798587-84798875
chr15	84799111	84799245	chr15:84799097-84799210
chr15	84799851	84799987	chr15:84799841-84799977
chr15	84801866	84802126	chr15:84801862-84806432
chr15	84802146	84805711	chr15:84801862-84806432
chr15	84805741	84805916	chr15:84801862-84806432
chr15	84805941	84806448	chr15:84801862-84806432
chr15	89748670	89748886	chr15:89748660-89750227
chr15	89748890	89750264	chr15:89748660-89750227

chr15	89750520	89751330	chr15:89750508-89751310
chr15	89760600	89760679	chr15:89760590-89760638
chr15	89767535	89767747	chr15:89767522-89767722
chr15	89776370	89776897	chr15:89776357-89777281
chr15	89776920	89777306	chr15:89776357-89777281
chr15	89778065	89778732	chr15:89778064-89778754
chr15	96325947	96326019	chr15:96325937-96326352
chr15	96326032	96326379	chr15:96325937-96326352
chr15	96328777	96328952	chr15:96328736-96328936
chr15	96330722	96330794	chr15:96330716-96332621
chr15	96330847	96330949	chr15:96330716-96332621
chr15	96330967	96331043	chr15:96330716-96332621
chr15	96331092	96331271	chr15:96330716-96332621
chr15	96331292	96331566	chr15:96330716-96332621
chr15	96331697	96331801	chr15:96330716-96332621
chr15	96331802	96331867	chr15:96330716-96332621
chr15	96331912	96332289	chr15:96330716-96332621
chr15	96332322	96332634	chr15:96330716-96332621
chr15	96333172	96333868	chr15:96333162-96333857
chr15	96334087	96334643	chr15:96334075-96334603
chr15	96337357	96337630	chr15:96337347-96340263
chr15	96337632	96337771	chr15:96337347-96340263
chr15	96337827	96337893	chr15:96337347-96340263
chr15	96337907	96337979	chr15:96337347-96340263
chr15	96337987	96338358	chr15:96337347-96340263
chr15	96338367	96338672	chr15:96337347-96340263
chr15	96338677	96338977	chr15:96337347-96340263
chr15	96338982	96339628	chr15:96337347-96340263
chr15	96339652	96339999	chr15:96337347-96340263
chr15	96340022	96340295	chr15:96337347-96340263
chr15	99565417	99565705	chr15:99565416-99565669
chr15	99565712	99566141	chr15:99565699-99566104
chr15	99566492	99566625	chr15:99566482-99566599
chr15	99598442	99598540	chr15:99598429-99598511
chr15	99632987	99633191	chr15:99632977-99633173
chr15	99645572	99645790	chr15:99645560-99645764
chr15	99649002	99649071	chr15:99649002-99649202
chr15	99649152	99649224	chr15:99649002-99649202
chr15	99657412	99657557	chr15:99657412-99657531
chr15	99671332	99671464	chr15:99671322-99671454
chr15	99671522	99671601	chr15:99671519-99671657
chr15	99671612	99671689	chr15:99671519-99671657
chr15	99674402	99674650	chr15:99674392-99674612
chr15	99675412	99675489	chr15:99675398-99675458
chr15	99690252	99690423	chr15:99690240-99690428
chr15	99706742	99706889	chr15:99706728-99706855
chr15	99710637	99710796	chr15:99710633-99710760
chr15	99712392	99712499	chr15:99712389-99715546
chr15	99712532	99712848	chr15:99712389-99715546
chr15	99712917	99713329	chr15:99712389-99715546
chr15	99713332	99715573	chr15:99712389-99715546
chr16	981810	982375	chr16:981807-982344
chr16	983740	983993	chr16:983727-983960
chr16	984710	985982	chr16:984700-986979
chr16	985995	986791	chr16:984700-986979
chr16	986810	987015	chr16:984700-986979
chr16	2223565	2223961	chr16:2223565-2223929
chr16	2226485	2226699	chr16:2226475-2226675
chr16	2228300	2228544	chr16:2228291-2228523
chr16	2229580	2229722	chr16:2229569-2229675
chr16	2232170	2232390	chr16:2232170-2232364
chr16	2232460	2232600	chr16:2232455-2232576
chr16	2232765	2232943	chr16:2232755-2232908

chr16	2233020	2233195	chr16:2233010-2233183
chr16	2233450	2233670	chr16:2233437-2233647
chr16	2233890	2234029	chr16:2233881-2233990
chr16	2234180	2234424	chr16:2234170-2234388
chr16	2234595	2234800	chr16:2234582-2234781
chr16	2234870	2235038	chr16:2234858-2235001
chr16	2235080	2235159	chr16:2235080-2235143
chr16	2235225	2235680	chr16:2235215-2235665
chr16	3088895	3090672	chr16:3088889-3090646
chr16	3091545	3091625	chr16:3091539-3091597
chr16	3091765	3091842	chr16:3091763-3091828
chr16	3092050	3092335	chr16:3092048-3092316
chr16	3092550	3092668	chr16:3092541-3093004
chr16	3092670	3093024	chr16:3092541-3093004
chr16	3096450	3096543	chr16:3096449-3096481
chr16	3097410	3097616	chr16:3097403-3097603
chr16	3099200	3099341	chr16:3099189-3099317
chr16	3112560	3112983	chr16:3112559-3112950
chr16	3113360	3113494	chr16:3113348-3113487
chr16	3115355	3115601	chr16:3115354-3115568
chr16	3115840	3115953	chr16:3115828-3115920
chr16	3116435	3116574	chr16:3116426-3116547
chr16	3118905	3119053	chr16:3118904-3119015
chr16	3119255	3120550	chr16:3119255-3120517
chr16	3263810	3263912	chr16:3263799-3263883
chr16	3282945	3284211	chr16:3282942-3284205
chr16	3285060	3285269	chr16:3285058-3285239
chr16	3285680	3285789	chr16:3285680-3285754
chr16	3286030	3286901	chr16:3286022-3286884
chr16	3288455	3288611	chr16:3288453-3288570
chr16	3289395	3291485	chr16:3289392-3291460
chr16	3295475	3295685	chr16:3295473-3295673
chr16	3401240	3402429	chr16:3401234-3402406
chr16	3404425	3405099	chr16:3404425-3405079
chr16	3408330	3409377	chr16:3408320-3409370
chr16	3725055	3726585	chr16:3725053-3729874
chr16	3726605	3727345	chr16:3725053-3729874
chr16	3727365	3727456	chr16:3725053-3729874
chr16	3727460	3728408	chr16:3725053-3729874
chr16	3728450	3729915	chr16:3725053-3729874
chr16	3730520	3730592	chr16:3730511-3730552
chr16	3731195	3731454	chr16:3731191-3731473
chr16	3731775	3731971	chr16:3731775-3731937
chr16	3736045	3736216	chr16:3736035-3736203
chr16	3736660	3736847	chr16:3736649-3736815
chr16	3738570	3738710	chr16:3738558-3738672
chr16	3739450	3739729	chr16:3739445-3739724
chr16	3740400	3740579	chr16:3740398-3740549
chr16	3743665	3743742	chr16:3743662-3743732
chr16	3744905	3744977	chr16:3744893-3744961
chr16	3745285	3745393	chr16:3745276-3745354
chr16	3745550	3745618	chr16:3745555-3745595
chr16	3749635	3749702	chr16:3749626-3749683
chr16	3751730	3751880	chr16:3751725-3751836
chr16	3757300	3757400	chr16:3757287-3757376
chr16	3757820	3758058	chr16:3757808-3758048
chr16	3758865	3758999	chr16:3758853-3758972
chr16	3767730	3767934	chr16:3767719-3767909
chr16	3769185	3769392	chr16:3769173-3769353
chr16	3770575	3771009	chr16:3770569-3770986
chr16	3773690	3773974	chr16:3773635-3773930
chr16	3774580	3774718	chr16:3774568-3774693
chr16	3778010	3778187	chr16:3778010-3778182

chr16	3778705	3778850	chr16:3778699-3778817
chr16	3780735	3780917	chr16:3780731-3780878
chr16	3781215	3781319	chr16:3781203-3781306
chr16	3782695	3782947	chr16:3782683-3782926
chr16	3784480	3784551	chr16:3784472-3784515
chr16	3791195	3791266	chr16:3791192-3791255
chr16	3791980	3792128	chr16:3791980-3792094
chr16	3793395	3793647	chr16:3793385-3793626
chr16	3810605	3810789	chr16:3810602-3810779
chr16	3812945	3813108	chr16:3812937-3812977;chr16:3812997-3813072
chr16	3850310	3851040	chr16:3850296-3851009
chr16	3872330	3872550	chr16:3872323-3872523
chr16	3879840	3880127	chr16:3879831-3880726
chr16	3880150	3880288	chr16:3879831-3880726
chr16	3880305	3880408	chr16:3879831-3880726
chr16	3880520	3880721	chr16:3879831-3880726
chr16	4257185	4257321	chr16:4257185-4258249
chr16	4257450	4257614	chr16:4257185-4258249
chr16	4257620	4258285	chr16:4257185-4258249
chr16	4260100	4260278	chr16:4260089-4260245
chr16	4260465	4260633	chr16:4260454-4260595
chr16	4261785	4261958	chr16:4261778-4261949
chr16	4262330	4262452	chr16:4262323-4262422
chr16	4262545	4262720	chr16:4262535-4262701
chr16	4263670	4263753	chr16:4263666-4263737
chr16	4268045	4268120	chr16:4268047-4268081
chr16	4270450	4270652	chr16:4270436-4270636
chr16	4272670	4272942	chr16:4272657-4273075
chr16	4273000	4273106	chr16:4272657-4273075
chr16	4314770	4315460	chr16:4314760-4315455
chr16	4319280	4319505	chr16:4319278-4319478
chr16	4329120	4329197	chr16:4329109-4329169
chr16	4332215	4332475	chr16:4332214-4332452
chr16	4333355	4333529	chr16:4333346-4333519
chr16	4334800	4335018	chr16:4334800-4334977
chr16	4335060	4335204	chr16:4335059-4335193
chr16	4335275	4335427	chr16:4335274-4335393
chr16	4336735	4339632	chr16:4336724-4339597
chr16	9763291	9763461	chr16:9763278-9763428
chr16	9763781	9763895	chr16:9763771-9763871
chr16	9764801	9764996	chr16:9764798-9764948
chr16	9768861	9768980	chr16:9768850-9768950
chr16	9835776	9835990	chr16:9835774-9835974
chr16	11547733	11548578	chr16:11547721-11549746
chr16	11548598	11548707	chr16:11547721-11549746
chr16	11548818	11549056	chr16:11547721-11549746
chr16	11549058	11549760	chr16:11547721-11549746
chr16	11553423	11553791	chr16:11553422-11553748
chr16	11556283	11556747	chr16:11556283-11556736
chr16	11561153	11561610	chr16:11561145-11561574
chr16	11575418	11575616	chr16:11575405-11575586
chr16	11583088	11583310	chr16:11583084-11583284
chr16	11584203	11584342	chr16:11584189-11584332
chr16	11586148	11586401	chr16:11586145-11586373
chr16	11586888	11586958	chr16:11586885-11586950
chr16	11587153	11587292	chr16:11587150-11587289
chr16	11587348	11587594	chr16:11587337-11587563
chr16	11598398	11598536	chr16:11598387-11598500
chr16	11629133	11629243	chr16:11629122-11629210
chr16	11635828	11635983	chr16:11635824-11635953
chr16	11636078	11636228	chr16:11636069-11636381
chr16	11636233	11636413	chr16:11636069-11636381
chr16	21258695	21258885	chr16:21258695-21258845

chr16	21261255	21261365	chr16:21261253-21261338
chr16	21290905	21291113	chr16:21290897-21291097
chr16	28938686	28938799	chr16:28938672-28938768
chr16	28938871	28939015	chr16:28938871-28938978
chr16	28939151	28939234	chr16:28939140-28939290
chr16	29806131	29806203	chr16:29806105-29806180
chr16	29806536	29806927	chr16:29806536-29806893
chr16	29806991	29807890	chr16:29806977-29807885
chr16	29807941	29808043	chr16:29807928-29808013
chr16	29808061	29808303	chr16:29808050-29808293
chr16	29808571	29808971	chr16:29808569-29808935
chr16	29809541	29809797	chr16:29809539-29809771
chr16	29810116	29810665	chr16:29810076-29811164
chr16	29810671	29811197	chr16:29810076-29811164
chr16	30085801	30086477	chr16:30085792-30086438
chr16	30086516	30086721	chr16:30086511-30086695
chr16	30086786	30086862	chr16:30086777-30086882
chr16	30088271	30088485	chr16:30087915-30088615
chr16	30088506	30088658	chr16:30087915-30088615
chr16	30088681	30088867	chr16:30088677-30088839
chr16	30088951	30089246	chr16:30088942-30089210
chr16	30090761	30091014	chr16:30090757-30090992
chr16	30091076	30091918	chr16:30091075-30091887
chr16	31060856	31062286	chr16:31060842-31062280
chr16	31063816	31064518	chr16:31063812-31064481
chr16	31064581	31064720	chr16:31064571-31064685
chr16	31064806	31065130	chr16:31064799-31065088
chr16	31067921	31068132	chr16:31067912-31068112
chr16	31072751	31072951	chr16:31072739-31073502
chr16	31072971	31073524	chr16:31072739-31073502
chr16	31073661	31074307	chr16:31073658-31074271
chr16	31471581	31471659	chr16:31471584-31471623
chr16	31472036	31472202	chr16:31472029-31472201
chr16	31473086	31473577	chr16:31473079-31473556
chr16	31473691	31473761	chr16:31473681-31473734
chr16	31473841	31474020	chr16:31473834-31473977
chr16	31474151	31474274	chr16:31474151-31474239
chr16	31474361	31474476	chr16:31474349-31474455
chr16	31474571	31474780	chr16:31474562-31474759
chr16	31476011	31476205	chr16:31476011-31476185
chr16	31476481	31476594	chr16:31476480-31476562
chr16	31476861	31477045	chr16:31476861-31477010
chr16	31477311	31477992	chr16:31477309-31477960
chr16	31713241	31713315	chr16:31713228-31713507
chr16	31713321	31713522	chr16:31713228-31713507
chr16	31714366	31714446	chr16:31714366-31714417
chr16	31722651	31722729	chr16:31722625-31722752
chr16	31723316	31723399	chr16:31723257-31723353
chr16	31733516	31733655	chr16:31733510-31733627
chr16	31753776	31754785	chr16:31753765-31761565
chr16	31754886	31755025	chr16:31753765-31761565
chr16	31755041	31755117	chr16:31753765-31761565
chr16	31755121	31755291	chr16:31753765-31761565
chr16	31755301	31755612	chr16:31753765-31761565
chr16	31755636	31757173	chr16:31753765-31761565
chr16	31757451	31757804	chr16:31753765-31761565
chr16	31757971	31758487	chr16:31753765-31761565
chr16	31758786	31759559	chr16:31753765-31761565
chr16	31759561	31761348	chr16:31753765-31761565
chr16	31761351	31761585	chr16:31753765-31761565
chr16	31769276	31769495	chr16:31769263-31769463
chr16	31792641	31792752	chr16:31792629-31792730
chr16	31793346	31793450	chr16:31793334-31793411

chr16	31793706	31794893	chr16:31793701-31794869
chr16	31873771	31873980	chr16:31873757-31873969
chr16	31874076	31874298	chr16:31874065-31874264
chr16	31875156	31875328	chr16:31875151-31875302
chr16	31883246	31883318	chr16:31883242-31883442
chr16	31883351	31883470	chr16:31883242-31883442
chr16	31884506	31884611	chr16:31884497-31884624
chr16	31885161	31885287	chr16:31885160-31885256
chr16	31890206	31890279	chr16:31890206-31890250
chr16	31914476	31915466	chr16:31914475-31917357
chr16	31915561	31915877	chr16:31914475-31917357
chr16	31915891	31915967	chr16:31914475-31917357
chr16	31915971	31916128	chr16:31914475-31917357
chr16	31916311	31916392	chr16:31914475-31917357
chr16	31916406	31917243	chr16:31914475-31917357
chr16	31917256	31917387	chr16:31914475-31917357
chr16	49487525	49490777	chr16:49487523-49491304
chr16	49490780	49490875	chr16:49487523-49491304
chr16	49490925	49491347	chr16:49487523-49491304
chr16	49523635	49523778	chr16:49523623-49523739
chr16	49525365	49525524	chr16:49525362-49525494
chr16	49626170	49626279	chr16:49626169-49626254
chr16	49635665	49638891	chr16:49635659-49638874
chr16	49640740	49640898	chr16:49640737-49640852
chr16	49664100	49664236	chr16:49664090-49664225
chr16	49730600	49730989	chr16:49730575-49730971
chr16	49780555	49780633	chr16:49780544-49780609
chr16	49789490	49789568	chr16:49789486-49789546
chr16	49822670	49822741	chr16:49822669-49822738
chr16	49826765	49827049	chr16:49826762-49827010
chr16	49852565	49852701	chr16:49852561-49852761
chr16	49852710	49852776	chr16:49852561-49852761
chr16	49855735	49856120	chr16:49855734-49856135
chr16	49857750	49857945	chr16:49857740-49857919
chr16	51135987	51136534	chr16:51135974-51137552
chr16	51136557	51137571	chr16:51135974-51137552
chr16	51138687	51141740	chr16:51138687-51142145
chr16	51141767	51142177	chr16:51138687-51142145
chr16	51143217	51143422	chr16:51143203-51143408
chr16	51144402	51144610	chr16:51144391-51144591
chr16	51150382	51150636	chr16:51150380-51150597
chr16	51151177	51151308	chr16:51151165-51151367
chr16	51151322	51151383	chr16:51151165-51151367
chr16	52438012	52438394	chr16:52438004-52439968
chr16	52438397	52439602	chr16:52438004-52439968
chr16	52439617	52440011	chr16:52438004-52439968
chr16	52444277	52444385	chr16:52444275-52444356
chr16	52446007	52446264	chr16:52445993-52446221
chr16	52450277	52450566	chr16:52450276-52450546
chr16	52463937	52464220	chr16:52463933-52464188
chr16	52468512	52468589	chr16:52468508-52468574
chr16	52475532	52475684	chr16:52475530-52475640
chr16	52482227	52482440	chr16:52482217-52482417
chr16	52519407	52519594	chr16:52519405-52519579
chr16	52546637	52547032	chr16:52546636-52547008
chr16	52547377	52547447	chr16:52547367-52547419
chr16	52547717	52547837	chr16:52547713-52547802
chr16	54283303	54283380	chr16:54283303-54283740
chr16	54283408	54283539	chr16:54283303-54283740
chr16	54283543	54283665	chr16:54283303-54283740
chr16	54283678	54283778	chr16:54283303-54283740
chr16	54284248	54284321	chr16:54284245-54284312
chr16	54284498	54284711	chr16:54284496-54285613

chr16	54284713	54285340	chr16:54284496-54285613
chr16	54285428	54285637	chr16:54284496-54285613
chr16	54285783	54286790	chr16:54285783-54286763
chr16	54930863	54930936	chr16:54930861-54931447
chr16	54930953	54931402	chr16:54930861-54931447
chr16	54932063	54932234	chr16:54932053-54932209
chr16	54932498	54932634	chr16:54932497-54932903
chr16	54932743	54932910	chr16:54932497-54932903
chr16	54933083	54933951	chr16:54933076-54934485
chr16	54933958	54934054	chr16:54933076-54934485
chr16	54934073	54934206	chr16:54933076-54934485
chr16	54934218	54934508	chr16:54933076-54934485
chr16	55323763	55324018	chr16:55323759-55325136
chr16	55324023	55324277	chr16:55323759-55325136
chr16	55324298	55324496	chr16:55323759-55325136
chr16	55324503	55325157	chr16:55323759-55325136
chr16	55325808	55326015	chr16:55325799-55326010
chr16	55326338	55326633	chr16:55326335-55326593
chr16	55326878	55326953	chr16:55326876-55326936
chr16	55327298	55327444	chr16:55327295-55327405
chr16	55327598	55327692	chr16:55327585-55327893
chr16	55327763	55327915	chr16:55327585-55327893
chr16	55328773	55329340	chr16:55328699-55329311
chr16	55330303	55330792	chr16:55330297-55330760
chr16	67165796	67165876	chr16:67165792-67165846
chr16	67165946	67166091	chr16:67165945-67166070
chr16	67166321	67166431	chr16:67166319-67166395
chr16	67166561	67166641	chr16:67166557-67166622
chr16	67167126	67167261	chr16:67167119-67167226
chr16	67167306	67167417	chr16:67167302-67167402
chr16	67167461	67167606	chr16:67167460-67167599
chr16	67167721	67167987	chr16:67167719-67167947
chr16	67168841	67168978	chr16:67168830-67168936
chr16	67169046	67169120	chr16:67169035-67169101
chr16	67169291	67169942	chr16:67169278-67169945
chr16	67192181	67192387	chr16:67192168-67192362
chr16	67192506	67192689	chr16:67192494-67192664
chr16	67192771	67192907	chr16:67192760-67192870
chr16	67193021	67193197	chr16:67193008-67193170
chr16	67193426	67193536	chr16:67193421-67193515
chr16	67194401	67194476	chr16:67194397-67194459
chr16	67194686	67195022	chr16:67194685-67194980
chr16	67195781	67195898	chr16:67195781-67196008
chr16	67195921	67196015	chr16:67195781-67196008
chr16	67196896	67197108	chr16:67196885-67197085
chr16	67197611	67197688	chr16:67197598-67197646
chr16	67197866	67197944	chr16:67197866-67197911
chr16	67198021	67198934	chr16:67198007-67198918
chr16	67562408	67562751	chr16:67562406-67562724
chr16	67571148	67571289	chr16:67571147-67571264
chr16	67610828	67611611	chr16:67610823-67611613
chr16	67611953	67612138	chr16:67611950-67612121
chr16	67616753	67616895	chr16:67616744-67616878
chr16	67620698	67620850	chr16:67620696-67620817
chr16	67621448	67621624	chr16:67621441-67621591
chr16	67626568	67626738	chr16:67626554-67626715
chr16	67628378	67628582	chr16:67628369-67628552
chr16	67629408	67629545	chr16:67629397-67629533
chr16	67632476	67632678	chr16:67632463-67632663
chr16	67636691	67636867	chr16:67636689-67636851
chr16	67637701	67637938	chr16:67637687-67639183
chr16	67637956	67639212	chr16:67637687-67639183
chr16	67842101	67842842	chr16:67842100-67842807

chr16	67843491	67844218	chr16:67843487-67844195
chr16	68084756	68085203	chr16:68084750-68085180
chr16	68085356	68085635	chr16:68085343-68085784
chr16	68085646	68085817	chr16:68085343-68085784
chr16	68086651	68086816	chr16:68086637-68086698;chr16:68086700-68086798
chr16	68088946	68089234	chr16:68088700-68089202
chr16	68090756	68090970	chr16:68090754-68090954
chr16	68121991	68123156	chr16:68121986-68123121
chr16	68126451	68126633	chr16:68126447-68126610
chr16	68138521	68138795	chr16:68138510-68138784
chr16	68157871	68158084	chr16:68157868-68158073
chr16	68166846	68167039	chr16:68166842-68167015
chr16	68174376	68174521	chr16:68174373-68174514
chr16	68181476	68181543	chr16:68181474-68181530
chr16	68183241	68183384	chr16:68183239-68183366
chr16	68190776	68191937	chr16:68190767-68192210
chr16	68214331	68214451	chr16:68214328-68214432
chr16	68217711	68218058	chr16:68217710-68218090
chr16	68221201	68221308	chr16:68221195-68221284
chr16	68226351	68226905	chr16:68226349-68228960
chr16	68226961	68227802	chr16:68226349-68228960
chr16	68227811	68228132	chr16:68226349-68228960
chr16	68228136	68228970	chr16:68226349-68228960
chr16	68530101	68530558	chr16:68530089-68530534
chr16	68533816	68533886	chr16:68533697-68533855
chr16	68539226	68539501	chr16:68539212-68539487
chr16	68539711	68539852	chr16:68539702-68539825
chr16	68541781	68541996	chr16:68541769-68541969
chr16	68555076	68555252	chr16:68555072-68555240
chr16	68558006	68558084	chr16:68557997-68558124
chr16	68558086	68558165	chr16:68557997-68558124
chr16	68558376	68558606	chr16:68558374-68558568
chr16	68563001	68564348	chr16:68562988-68567136
chr16	68564356	68564832	chr16:68562988-68567136
chr16	68564856	68565864	chr16:68562988-68567136
chr16	68566121	68567173	chr16:68562988-68567136
chr16	68575801	68576042	chr16:68575790-68576072
chr16	69565208	69565349	chr16:69565093-69566374
chr16	69565373	69566387	chr16:69565093-69566374
chr16	69568498	69568575	chr16:69568494-69568548
chr16	69618763	69618973	chr16:69618749-69618949
chr16	69626408	69626560	chr16:69626402-69626528
chr16	69644798	69644877	chr16:69644796-69644856
chr16	69646518	69646593	chr16:69646516-69646578
chr16	69647038	69647600	chr16:69647027-69647586
chr16	69653248	69653452	chr16:69653235-69653428
chr16	69655613	69655821	chr16:69655608-69655799
chr16	69659728	69659916	chr16:69659726-69659899
chr16	69669978	69670125	chr16:69669976-69670111
chr16	69670238	69670307	chr16:69670235-69670288
chr16	69677213	69677353	chr16:69677202-69677335
chr16	69684888	69685000	chr16:69684886-69686075
chr16	69685003	69685180	chr16:69684886-69686075
chr16	69685618	69685729	chr16:69684886-69686075
chr16	69690943	69691119	chr16:69690939-69691088
chr16	69691758	69693620	chr16:69691748-69694239
chr16	69693648	69694272	chr16:69691748-69694239
chr16	69695138	69695572	chr16:69695135-69695676
chr16	69696363	69696437	chr16:69696359-69704666
chr16	69696438	69697448	chr16:69696359-69704666
chr16	69697463	69697942	chr16:69696359-69704666
chr16	69697948	69698080	chr16:69696359-69704666
chr16	69698128	69698377	chr16:69696359-69704666

chr16	69698398	69698746	chr16:69696359-69704666
chr16	69698758	69699805	chr16:69696359-69704666
chr16	69699863	69700092	chr16:69696359-69704666
chr16	69700118	69700184	chr16:69696359-69704666
chr16	69700243	69700944	chr16:69696359-69704666
chr16	69701238	69701337	chr16:69696359-69704666
chr16	69701338	69702666	chr16:69696359-69704666
chr16	69702678	69703889	chr16:69696359-69704666
chr16	69703908	69704012	chr16:69696359-69704666
chr16	69704023	69704338	chr16:69696359-69704666
chr16	69704348	69704690	chr16:69696359-69704666
chr16	71381055	71381262	chr16:71381045-71381245
chr16	71385580	71385659	chr16:71385576-71385648
chr16	71389750	71389933	chr16:71389748-71389898
chr16	71447605	71448597	chr16:71447596-71449885
chr16	71448605	71448753	chr16:71447596-71449885
chr16	71448765	71448837	chr16:71447596-71449885
chr16	71448850	71449002	chr16:71447596-71449885
chr16	71449030	71449925	chr16:71447596-71449885
chr16	71453255	71453988	chr16:71453242-71453973
chr16	71454070	71454246	chr16:71454041-71454218
chr16	71455000	71455171	chr16:71454942-71455142
chr16	71456080	71456153	chr16:71456071-71456121
chr16	71456775	71456854	chr16:71456763-71456830
chr16	71461660	71462793	chr16:71461658-71462759
chr16	71463000	71463134	chr16:71462987-71463095
chr16	71464555	71464871	chr16:71464554-71467096
chr16	71465175	71465673	chr16:71464554-71467096
chr16	71466030	71466527	chr16:71464554-71467096
chr16	71466530	71466979	chr16:71464554-71467096
chr16	71466995	71467131	chr16:71464554-71467096
chr16	71473870	71475627	chr16:71473582-71476272
chr16	71475650	71475725	chr16:71473582-71476272
chr16	71475730	71476311	chr16:71473582-71476272
chr16	71477890	71478374	chr16:71477885-71478341
chr16	71478440	71478544	chr16:71478434-71478507
chr16	71478880	71479138	chr16:71478878-71479100
chr16	71482085	71482166	chr16:71482081-71482143
chr16	71482245	71482359	chr16:71482241-71482322
chr16	71484595	71484761	chr16:71484588-71484748
chr16	71487250	71487333	chr16:71487079-71487410
chr16	71489010	71489392	chr16:71488997-71489351
chr16	71494955	71495035	chr16:71494953-71495018
chr16	71514010	71514188	chr16:71514003-71514157
chr16	71523860	71524075	chr16:71523849-71524049
chr16	71546210	71546346	chr16:71546201-71546335
chr16	71563685	71565121	chr16:71563678-71565089
chr16	71859690	71860695	chr16:71859679-71860672
chr16	71861775	71861979	chr16:71861775-71861942
chr16	71864150	71864263	chr16:71864137-71864242
chr16	71864905	71865082	chr16:71864898-71865048
chr16	71867905	71868075	chr16:71867892-71868037
chr16	71871305	71871530	chr16:71871304-71871504
chr16	71879920	71880086	chr16:71879906-71880065
chr16	71880435	71880516	chr16:71880422-71880476
chr16	71881320	71881439	chr16:71881316-71881434
chr16	71883550	71883621	chr16:71883553-71883585
chr16	71883920	71884235	chr16:71883907-71884204
chr16	71884595	71884846	chr16:71884593-71884809
chr16	71895010	71895119	chr16:71895008-71895098
chr16	72788705	72788883	chr16:72788698-72788848
chr16	72793255	72793367	chr16:72793254-72793354
chr16	72817490	72817700	chr16:72817479-72817679

chr16	75148495	75148682	chr16:75148491-75148662
chr16	75148900	75149045	chr16:75148900-75149012
chr16	75149060	75149296	chr16:75149050-75149267
chr16	75152910	75152983	chr16:75152908-75152966
chr16	75157145	75157248	chr16:75157136-75157211
chr16	75166780	75166948	chr16:75166769-75166913
chr16	75169265	75170084	chr16:75169252-75172236
chr16	75170155	75171699	chr16:75169252-75172236
chr16	75171705	75172012	chr16:75169252-75172236
chr16	75172045	75172174	chr16:75169252-75172236
chr16	75172195	75172268	chr16:75169252-75172236
chr16	79585854	79585953	chr16:79585842-79585941
chr16	79590289	79590508	chr16:79590287-79590487
chr16	79593849	79594137	chr16:79593837-79600714
chr16	79594154	79594385	chr16:79593837-79600714
chr16	79594419	79594634	chr16:79593837-79600714
chr16	79594644	79594713	chr16:79593837-79600714
chr16	79594719	79595116	chr16:79593837-79600714
chr16	79595134	79595406	chr16:79593837-79600714
chr16	79595439	79595919	chr16:79593837-79600714
chr16	79595939	79596190	chr16:79593837-79600714
chr16	79596199	79596885	chr16:79593837-79600714
chr16	79596889	79596961	chr16:79593837-79600714
chr16	79596964	79597347	chr16:79593837-79600714
chr16	79597419	79597758	chr16:79593837-79600714
chr16	79597809	79598238	chr16:79593837-79600714
chr16	79598339	79598466	chr16:79593837-79600714
chr16	79598469	79598573	chr16:79593837-79600714
chr16	79598604	79599190	chr16:79593837-79600714
chr16	79599224	79599329	chr16:79593837-79600714
chr16	79599354	79599883	chr16:79593837-79600714
chr16	79599919	79600483	chr16:79593837-79600714
chr16	79600489	79600592	chr16:79593837-79600714
chr16	79600604	79600744	chr16:79593837-79600714
chr16	85898808	85899258	chr16:85898802-85899223
chr16	85900938	85901285	chr16:85900927-85901246
chr16	85902688	85903217	chr16:85902688-85903189
chr16	85906093	85906326	chr16:85906092-85906292
chr16	85908993	85909907	chr16:85908987-85909882
chr16	85911573	85911690	chr16:85911569-85911658
chr16	85913133	85913256	chr16:85913130-85913236
chr16	85913983	85914195	chr16:85913973-85914171
chr16	85914318	85914526	chr16:85914312-85914520
chr16	85918418	85918842	chr16:85918416-85918803
chr16	85920118	85920248	chr16:85920108-85920224
chr16	85921108	85922284	chr16:85921105-85922609
chr16	85922298	85922642	chr16:85921105-85922609
chr16	86510538	86510617	chr16:86510526-86511548
chr16	86510618	86510722	chr16:86510526-86511548
chr16	86510728	86511566	chr16:86510526-86511548
chr16	86512138	86512365	chr16:86512136-86512336
chr16	86512933	86513534	chr16:86512924-86515418
chr16	86513553	86514219	chr16:86512924-86515418
chr16	86514263	86514389	chr16:86512924-86515418
chr16	86514398	86514787	chr16:86512924-86515418
chr16	86514843	86515439	chr16:86512924-86515418
chr16	86567263	86567696	chr16:86567250-86569728
chr16	86567708	86569316	chr16:86567250-86569728
chr16	86569333	86569496	chr16:86567250-86569728
chr16	86569518	86569615	chr16:86567250-86569728
chr16	86569633	86569760	chr16:86567250-86569728
chr16	86576368	86576484	chr16:86576367-86576454
chr16	86577593	86577803	chr16:86577587-86577787

chr16	86578518	86579009	chr16:86578505-86582160
chr16	86579078	86581932	chr16:86578505-86582160
chr16	86581938	86582177	chr16:86578505-86582160
chr16	88504370	88504595	chr16:88504358-88504558
chr16	88514425	88514529	chr16:88514420-88514520
chr16	88516120	88516291	chr16:88516112-88516262
chr16	88516570	88516682	chr16:88516558-88516646
chr16	88532835	88532951	chr16:88532835-88532935
chr16	88533150	88533332	chr16:88533147-88533297
chr16	88677685	88678637	chr16:88677681-88678629
chr16	88681105	88681739	chr16:88681093-88681714
chr16	88682280	88682500	chr16:88682268-88682468
chr16	88686335	88686522	chr16:88686330-88686493
chr17	1270561	1271478	chr17:1270558-1271460
chr17	2055140	2055246	chr17:2055138-2055238
chr17	2056310	2056381	chr17:2056309-2056346
chr17	2056675	2056855	chr17:2056670-2056820
chr17	2384060	2384238	chr17:2384059-2387649
chr17	2384250	2384573	chr17:2384059-2387649
chr17	2384625	2387661	chr17:2384059-2387649
chr17	2387870	2388085	chr17:2387856-2388049
chr17	2391405	2391841	chr17:2391402-2391815
chr17	2392715	2392792	chr17:2392703-2392760
chr17	2393760	2393832	chr17:2393760-2393830
chr17	2394055	2394191	chr17:2394042-2394154
chr17	2394300	2394374	chr17:2394304-2394346
chr17	2394880	2395478	chr17:2394874-2395454
chr17	2396460	2396872	chr17:2396449-2396853
chr17	2398010	2398227	chr17:2398006-2398206
chr17	2400315	2400463	chr17:2400307-2400446
chr17	2400645	2400920	chr17:2400639-2401118
chr17	2400945	2401123	chr17:2400639-2401118
chr17	5012711	5012921	chr17:5012705-5012905
chr17	5022611	5022731	chr17:5022609-5022709
chr17	5023471	5023657	chr17:5023467-5023617
chr17	6686191	6686374	chr17:6686188-6686338
chr17	6687531	6687651	chr17:6687528-6687628
chr17	6690791	6690898	chr17:6690778-6690878
chr17	6697071	6697292	chr17:6697067-6697267
chr17	7023020	7023177	chr17:7023019-7023099;chr17:7023101-7023147
chr17	7023480	7023871	chr17:7023476-7023850
chr17	7024090	7024339	chr17:7024082-7024304
chr17	7024400	7024682	chr17:7024400-7024763
chr17	7024715	7024796	chr17:7024400-7024763
chr17	7025080	7025224	chr17:7025075-7025200
chr17	7026460	7026638	chr17:7026456-7026621
chr17	7026710	7026853	chr17:7026704-7026835
chr17	7026950	7027103	chr17:7026949-7027087
chr17	7027515	7029681	chr17:7027502-7029642
chr17	7029815	7030314	chr17:7029815-7030290
chr17	7459365	7459766	chr17:7459365-7463890
chr17	7459785	7460004	chr17:7459365-7463890
chr17	7460035	7460103	chr17:7459365-7463890
chr17	7460130	7463029	chr17:7459365-7463890
chr17	7463045	7463915	chr17:7459365-7463890
chr17	7465720	7466478	chr17:7465710-7466810
chr17	7466540	7466823	chr17:7465710-7466810
chr17	7467270	7467343	chr17:7467256-7467327
chr17	7477720	7477938	chr17:7477710-7477910
chr17	7479455	7479595	chr17:7479455-7479625
chr17	7484015	7484246	chr17:7484003-7484215
chr17	7588345	7588571	chr17:7588342-7588546
chr17	7589000	7590200	chr17:7588998-7590170

chr17	7666915	7667243	chr17:7666901-7667425
chr17	7667270	7667441	chr17:7666901-7667425
chr17	7668410	7668559	chr17:7668401-7669690
chr17	7668860	7669696	chr17:7668401-7669690
chr17	7670620	7670759	chr17:7670608-7670715
chr17	7673215	7673351	chr17:7673206-7673339
chr17	7673545	7673643	chr17:7673534-7673608
chr17	7673705	7673854	chr17:7673700-7673837
chr17	7674185	7674329	chr17:7674180-7674290
chr17	7674870	7675007	chr17:7674858-7674971
chr17	7675055	7675388	chr17:7675052-7675493
chr17	7676005	7676287	chr17:7675993-7676272
chr17	7676385	7676641	chr17:7676381-7676622
chr17	7677335	7677468	chr17:7677324-7677434
chr17	7679855	7680068	chr17:7679844-7680044
chr17	8120591	8120843	chr17:8120589-8120804
chr17	8121136	8121393	chr17:8121132-8122037
chr17	8121401	8122061	chr17:8121132-8122037
chr17	8122356	8122449	chr17:8122342-8122430
chr17	8123041	8123145	chr17:8123030-8123126
chr17	8123326	8123538	chr17:8123319-8123505
chr17	8124056	8124131	chr17:8124042-8124092
chr17	11977441	11978769	chr17:11977438-11978744
chr17	11983296	11983446	chr17:11983296-11983407
chr17	11984116	11984195	chr17:11984112-11984197
chr17	11987401	11987567	chr17:11987389-11987589
chr17	11990461	11990580	chr17:11990461-11990550
chr17	11990976	11991205	chr17:11990973-11991163
chr17	11992451	11992947	chr17:11992442-11992917
chr17	11996811	11997129	chr17:11996809-11997110
chr17	11997286	11997388	chr17:11997272-11997372
chr17	11997441	11997541	chr17:11997430-11997510
chr17	12665891	12666282	chr17:12665889-12666243
chr17	12705141	12705214	chr17:12705127-12705193
chr17	12710491	12710565	chr17:12710494-12710538
chr17	12713211	12713413	chr17:12713200-12713400
chr17	12715526	12715600	chr17:12715518-12715574
chr17	12717351	12717465	chr17:12717345-12717421
chr17	12722856	12723035	chr17:12722846-12723008
chr17	12736171	12736340	chr17:12736160-12736336
chr17	12739206	12739358	chr17:12739202-12739328
chr17	12744191	12744374	chr17:12744182-12744436
chr17	12744381	12744460	chr17:12744182-12744436
chr17	12745931	12746099	chr17:12745918-12746072
chr17	12752421	12753386	chr17:12752413-12753346
chr17	12756416	12756589	chr17:12756413-12756557
chr17	12758086	12758233	chr17:12758084-12758213
chr17	12760661	12760740	chr17:12760649-12760707
chr17	12763081	12763721	chr17:12763072-12768949
chr17	12763736	12764993	chr17:12763072-12768949
chr17	12764996	12765274	chr17:12763072-12768949
chr17	12765331	12765502	chr17:12763072-12768949
chr17	12765576	12766969	chr17:12763072-12768949
chr17	12766986	12767052	chr17:12763072-12768949
chr17	12767066	12767619	chr17:12763072-12768949
chr17	12767621	12768885	chr17:12763072-12768949
chr17	17681475	17681829	chr17:17681472-17681793
chr17	17682500	17682570	chr17:17682494-17682563
chr17	17683550	17683863	chr17:17683545-17685050
chr17	17684165	17684364	chr17:17683545-17685050
chr17	17684365	17684676	chr17:17683545-17685050
chr17	17684710	17685099	chr17:17683545-17685050
chr17	17724030	17724171	chr17:17724027-17724159

chr17	17782135	17782250	chr17:17782131-17782215
chr17	17791145	17791364	chr17:17791139-17791339
chr17	17792945	17793784	chr17:17792932-17798513
chr17	17793810	17796672	chr17:17792932-17798513
chr17	17796690	17798536	chr17:17792932-17798513
chr17	17803755	17804158	chr17:17803755-17804114
chr17	17809295	17809468	chr17:17809281-17809439
chr17	17809975	17811235	chr17:17809969-17811784
chr17	17811265	17811398	chr17:17809969-17811784
chr17	17811415	17811798	chr17:17809969-17811784
chr17	17811985	17812896	chr17:17811972-17812056;chr17:17812072-17812851
chr17	17812915	17813128	chr17:17812909-17813479
chr17	17813245	17813484	chr17:17812909-17813479
chr17	17813575	17813792	chr17:17813568-17813769
chr17	17814245	17814432	chr17:17814244-17814410
chr17	17814625	17814763	chr17:17814614-17814747
chr17	17814840	17814985	chr17:17814834-17814944
chr17	17815225	17815361	chr17:17815220-17815329
chr17	17815860	17816056	chr17:17815859-17816028
chr17	17816210	17816395	chr17:17816206-17816373
chr17	17816460	17816759	chr17:17816456-17816718
chr17	17816970	17817177	chr17:17816957-17817136
chr17	17817255	17817474	chr17:17817255-17817457
chr17	17817560	17817950	chr17:17817547-17817916
chr17	17818260	17818410	chr17:17818259-17818374
chr17	17819025	17819267	chr17:17819012-17819234
chr17	17819330	17819480	chr17:17819319-17819454
chr17	17819540	17820887	chr17:17819537-17820864
chr17	17823520	17823633	chr17:17823517-17823618
chr17	17830730	17830940	chr17:17830716-17830916
chr17	17836730	17837010	chr17:17836726-17837002
chr17	19411125	19411484	chr17:19411124-19411462
chr17	19411630	19411878	chr17:19411629-19411670;chr17:19411689-19411831
chr17	19412500	19412823	chr17:19412497-19412783
chr17	19412945	19413158	chr17:19412937-19413144
chr17	19413280	19413432	chr17:19413279-19413411
chr17	19413590	19413709	chr17:19413576-19413681
chr17	19414095	19414174	chr17:19414094-19414145
chr17	19414450	19414530	chr17:19414448-19414505
chr17	19414595	19414701	chr17:19414585-19414660
chr17	19414770	19414933	chr17:19414769-19414887
chr17	19415040	19415229	chr17:19415037-19415207
chr17	19415295	19415372	chr17:19415285-19415339
chr17	19415525	19415631	chr17:19415517-19415592
chr17	19415705	19417295	chr17:19415704-19417276
chr17	28506245	28506464	chr17:28506242-28506443
chr17	28516655	28516732	chr17:28516588-28516788
chr17	28523940	28524122	chr17:28523940-28524092
chr17	28524505	28525004	chr17:28524502-28524967
chr17	28527255	28527395	chr17:28527250-28527361
chr17	28529095	28529249	chr17:28529093-28529224
chr17	28530755	28530863	chr17:28530748-28530845
chr17	28534340	28534552	chr17:28534330-28534538
chr17	28534710	28535213	chr17:28534706-28535198
chr17	28537130	28538346	chr17:28537116-28538896
chr17	28538380	28538935	chr17:28537116-28538896
chr17	29003945	29004158	chr17:29003933-29004133
chr17	29055815	29055928	chr17:29055812-29055888
chr17	29056185	29056357	chr17:29056174-29056324
chr17	31937020	31937088	chr17:31937017-31937520
chr17	31937110	31937416	chr17:31937017-31937520
chr17	31937420	31937557	chr17:31937017-31937520
chr17	31940285	31940363	chr17:31940285-31940332

chr17	31940430	31940506	chr17:31940421-31940486
chr17	31947625	31947703	chr17:31947616-31947685
chr17	31966146	31966217	chr17:31966146-31966196
chr17	31973146	31973251	chr17:31973145-31973231
chr17	31975481	31975724	chr17:31975481-31975713
chr17	31976521	31976641	chr17:31976520-31976614
chr17	31983011	31983121	chr17:31982998-31983104
chr17	31984106	31984326	chr17:31984101-31984301
chr17	31988326	31988530	chr17:31988319-31988497
chr17	31993241	31993354	chr17:31993241-31993333
chr17	31993876	31994047	chr17:31993864-31994008
chr17	31994576	31994745	chr17:31994563-31994721
chr17	31995576	31995778	chr17:31995563-31995762
chr17	31996806	31996910	chr17:31996797-31996877
chr17	31998666	31999449	chr17:31998657-32001045
chr17	31999451	31999755	chr17:31998657-32001045
chr17	31999791	32000250	chr17:31998657-32001045
chr17	32000256	32000635	chr17:31998657-32001045
chr17	32000646	32001055	chr17:31998657-32001045
chr17	36936793	36937040	chr17:36936784-36937245
chr17	36937048	36937268	chr17:36936784-36937245
chr17	36937478	36937811	chr17:36937474-36938367
chr17	36937823	36938380	chr17:36937474-36938367
chr17	36939333	36939554	chr17:36939330-36939530
chr17	36940293	36940542	chr17:36940289-36940516
chr17	36940623	36940916	chr17:36940609-36940887
chr17	36942203	36942381	chr17:36942199-36942365
chr17	36942753	36943176	chr17:36942751-36944612
chr17	36943203	36943704	chr17:36942751-36944612
chr17	36943738	36943807	chr17:36942751-36944612
chr17	36943818	36943921	chr17:36942751-36944612
chr17	36943943	36944260	chr17:36942751-36944612
chr17	36944303	36944580	chr17:36942751-36944612
chr17	37686443	37687431	chr17:37686431-37687392
chr17	37699078	37699236	chr17:37699075-37699194
chr17	37700983	37701213	chr17:37700982-37701177
chr17	37704923	37705066	chr17:37704916-37705050
chr17	37710503	37710687	chr17:37710502-37710663
chr17	37731603	37731846	chr17:37731594-37731830
chr17	37733563	37733854	chr17:37733556-37733821
chr17	37739448	37739670	chr17:37739439-37739639
chr17	37743353	37743564	chr17:37743340-37743540
chr17	37744553	37745277	chr17:37744540-37745247
chr17	39404288	39404619	chr17:39404284-39405308
chr17	39404638	39405344	chr17:39404284-39405308
chr17	39406603	39410740	chr17:39406600-39410721
chr17	39415028	39415144	chr17:39415025-39415131
chr17	39415248	39415355	chr17:39415243-39415339
chr17	39416268	39416494	chr17:39416254-39416454
chr17	39419728	39419940	chr17:39419716-39419918
chr17	39423328	39423483	chr17:39423326-39423445
chr17	39423703	39423838	chr17:39423696-39423821
chr17	39424633	39424773	chr17:39424626-39424738
chr17	39427708	39427812	chr17:39427700-39427790
chr17	39431118	39431228	chr17:39431114-39431188
chr17	39431953	39432055	chr17:39431941-39432016
chr17	39434253	39434348	chr17:39434248-39434320
chr17	39440398	39440542	chr17:39440385-39440518
chr17	39440628	39440705	chr17:39440622-39440677
chr17	39443563	39443668	chr17:39443549-39443628
chr17	39445498	39445603	chr17:39445410-39445569
chr17	39447808	39447944	chr17:39447797-39447904
chr17	39451048	39451318	chr17:39451037-39451286

chr17	39604808	39606341	chr17:39604779-39606604
chr17	39606343	39606619	chr17:39604779-39606604
chr17	39607753	39607963	chr17:39607727-39607943
chr17	39757728	39759138	chr17:39757714-39766493
chr17	39759438	39760161	chr17:39757714-39766493
chr17	39760618	39760855	chr17:39757714-39766493
chr17	39761068	39761309	chr17:39757714-39766493
chr17	39761323	39761462	chr17:39757714-39766493
chr17	39761608	39761701	chr17:39757714-39766493
chr17	39761978	39762601	chr17:39757714-39766493
chr17	39762903	39763487	chr17:39757714-39766493
chr17	39763503	39763820	chr17:39757714-39766493
chr17	39764478	39764933	chr17:39757714-39766493
chr17	39764943	39766516	chr17:39757714-39766493
chr17	39777653	39777799	chr17:39777650-39777767
chr17	39777888	39778253	chr17:39777884-39778225
chr17	39788268	39788405	chr17:39788257-39788374
chr17	39791418	39791592	chr17:39791415-39791583
chr17	39792673	39792970	chr17:39792672-39792933
chr17	39808963	39809181	chr17:39808954-39809154
chr17	39829393	39829496	chr17:39829386-39829488
chr17	39832098	39832175	chr17:39832097-39832151
chr17	39864123	39864205	chr17:39864119-39864188
chr17	40058293	40058510	chr17:40058289-40058478
chr17	40062198	40062582	chr17:40062192-40062548
chr17	40062813	40063104	chr17:40062809-40063092
chr17	40068913	40069030	chr17:40068902-40069007
chr17	40070673	40070879	chr17:40070663-40070863
chr17	40072093	40072204	chr17:40072090-40072184
chr17	40074193	40074553	chr17:40074191-40074541
chr17	40076873	40076947	chr17:40076870-40076938
chr17	40077508	40077621	chr17:40077507-40077608
chr17	40083838	40084020	chr17:40083834-40083982
chr17	40084613	40084828	chr17:40084609-40084815
chr17	40086713	40086894	chr17:40086706-40086853
chr17	40088253	40088538	chr17:40088241-40088500
chr17	40089218	40090014	chr17:40089205-40092627
chr17	40090043	40090965	chr17:40089205-40092627
chr17	40090968	40091111	chr17:40089205-40092627
chr17	40091118	40091216	chr17:40089205-40092627
chr17	40091263	40091448	chr17:40089205-40092627
chr17	40091463	40092644	chr17:40089205-40092627
chr17	40092793	40093884	chr17:40092786-40093867
chr17	40093923	40094134	chr17:40093911-40094122
chr17	40094938	40095155	chr17:40094934-40095120
chr17	40095448	40096125	chr17:40095443-40096087
chr17	40096448	40096623	chr17:40096442-40096587
chr17	40096698	40096808	chr17:40096690-40096779
chr17	40097078	40097155	chr17:40097064-40097403
chr17	40097158	40097444	chr17:40097064-40097403
chr17	40098098	40098314	chr17:40098085-40098285
chr17	40100068	40100739	chr17:40100063-40100725
chr17	40309193	40309312	chr17:40309191-40309286
chr17	40318283	40318459	chr17:40318280-40318448
chr17	40322993	40323290	chr17:40322990-40323259
chr17	40326478	40326594	chr17:40326474-40326551
chr17	40330858	40331424	chr17:40330856-40331396
chr17	40341398	40341643	chr17:40341387-40341615
chr17	40342368	40342888	chr17:40342362-40342867
chr17	40345253	40345342	chr17:40345240-40345316
chr17	40346708	40346935	chr17:40346706-40346906
chr17	40348328	40348507	chr17:40348315-40348464
chr17	40349783	40349966	chr17:40349783-40349925

chr17	40351398	40351563	chr17:40351386-40351531
chr17	40351913	40352094	chr17:40351909-40352070
chr17	40352333	40352522	chr17:40352330-40352507
chr17	40354313	40354530	chr17:40354301-40354506
chr17	40355268	40355452	chr17:40355262-40355421
chr17	40356008	40356744	chr17:40356008-40357643
chr17	40356758	40357324	chr17:40356008-40357643
chr17	40357343	40357671	chr17:40356008-40357643
chr17	40389508	40389676	chr17:40389497-40389647
chr17	40389973	40390092	chr17:40389964-40390064
chr17	42199171	42199247	chr17:42199167-42201864
chr17	42199261	42199367	chr17:42199167-42201864
chr17	42199376	42200453	chr17:42199167-42201864
chr17	42200471	42201523	chr17:42199167-42201864
chr17	42201546	42201890	chr17:42199167-42201864
chr17	42202341	42202492	chr17:42202339-42202447
chr17	42202761	42202839	chr17:42202756-42202808
chr17	42207561	42207740	chr17:42207557-42207728
chr17	42210176	42210318	chr17:42210170-42210301
chr17	42210416	42210515	chr17:42210402-42210497
chr17	42211986	42212222	chr17:42211983-42212190
chr17	42216021	42216131	chr17:42216013-42216106
chr17	42217171	42217307	chr17:42217159-42217282
chr17	42217376	42217502	chr17:42217376-42217464
chr17	42218156	42218377	chr17:42218150-42218330
chr17	42218731	42218912	chr17:42218722-42218878
chr17	42219316	42219506	chr17:42219311-42219463
chr17	42219716	42219855	chr17:42219711-42219842
chr17	42223381	42223565	chr17:42223381-42223556
chr17	42224791	42224899	chr17:42224778-42224868
chr17	42227531	42227707	chr17:42227528-42227685
chr17	42232001	42232161	chr17:42231999-42232137
chr17	42237176	42237401	chr17:42237172-42237372
chr17	42276261	42276435	chr17:42276247-42276406
chr17	42276631	42276741	chr17:42276627-42276707
chr17	42287546	42287660	chr17:42287546-42287635
chr17	42287876	42288012	chr17:42287866-42288598
chr17	42288026	42288132	chr17:42287866-42288598
chr17	42288136	42288627	chr17:42287866-42288598
chr17	42289371	42289556	chr17:42289369-42289539
chr17	42289871	42290047	chr17:42289865-42290022
chr17	42291971	42292085	chr17:42291971-42292061
chr17	42295631	42295808	chr17:42295618-42295793
chr17	42297731	42297967	chr17:42297731-42297931
chr17	42299751	42299902	chr17:42299750-42299881
chr17	42300136	42300308	chr17:42300129-42300281
chr17	42300716	42300907	chr17:42300714-42300870
chr17	42301276	42301489	chr17:42301274-42301454
chr17	42304351	42304450	chr17:42304341-42304429
chr17	42304531	42304679	chr17:42304529-42304652
chr17	42305611	42305727	chr17:42305609-42305702
chr17	42306181	42306456	chr17:42306169-42306447
chr17	42307401	42307507	chr17:42307401-42307496
chr17	42307606	42307745	chr17:42307592-42307723
chr17	42308181	42308365	chr17:42308177-42308333
chr17	42309046	42309126	chr17:42309046-42309098
chr17	42309376	42309712	chr17:42309376-42309686
chr17	42310511	42310772	chr17:42310506-42311943
chr17	42310796	42311077	chr17:42310506-42311943
chr17	42311116	42311564	chr17:42310506-42311943
chr17	42311586	42311963	chr17:42310506-42311943
chr17	42313396	42314856	chr17:42313323-42315800
chr17	42315146	42315396	chr17:42313323-42315800

chr17	42315401	42315823	chr17:42313323-42315800
chr17	42316801	42316938	chr17:42316788-42316901
chr17	42317191	42317262	chr17:42317181-42317224
chr17	42322291	42322530	chr17:42322281-42322494
chr17	42323006	42323154	chr17:42323003-42323143
chr17	42323261	42323376	chr17:42323259-42323354
chr17	42323581	42323656	chr17:42323572-42323625
chr17	42324711	42324850	chr17:42324710-42324846
chr17	42324966	42325087	chr17:42324962-42325061
chr17	42326116	42326230	chr17:42326115-42326199
chr17	42329411	42329484	chr17:42329409-42329457
chr17	42329556	42329671	chr17:42329553-42329647
chr17	42329736	42329811	chr17:42329746-42329776
chr17	42331471	42331544	chr17:42331471-42331531
chr17	42333676	42333806	chr17:42333672-42333765
chr17	42333896	42334082	chr17:42333890-42334049
chr17	42337436	42337616	chr17:42337434-42337586
chr17	42337776	42337889	chr17:42337762-42337857
chr17	42338731	42338837	chr17:42338730-42338812
chr17	42339316	42339436	chr17:42339313-42339409
chr17	42345571	42345673	chr17:42345558-42345657
chr17	42346571	42346755	chr17:42346568-42346713
chr17	42348396	42348575	chr17:42348388-42348539
chr17	42374066	42374247	chr17:42374058-42374206
chr17	42374986	42375161	chr17:42374981-42375181
chr17	42386941	42387154	chr17:42386938-42388568
chr17	42387171	42387583	chr17:42386938-42388568
chr17	42387601	42388592	chr17:42386938-42388568
chr17	42567068	42567365	chr17:42567067-42567328
chr17	42567618	42567695	chr17:42567618-42567655
chr17	42568473	42568582	chr17:42568469-42568559
chr17	42568838	42568989	chr17:42568836-42568943
chr17	42569203	42569324	chr17:42569203-42569303
chr17	42569508	42569615	chr17:42569506-42569606
chr17	42569983	42570210	chr17:42569981-42570183
chr17	42571548	42572183	chr17:42571546-42572164
chr17	42761833	42761907	chr17:42761833-42761899
chr17	42762373	42762476	chr17:42762363-42762463
chr17	42762598	42762781	chr17:42762596-42762746
chr17	43527845	43528759	chr17:43527843-43528743
chr17	43529135	43529253	chr17:43529134-43529236
chr17	43529515	43529692	chr17:43529503-43529676
chr17	43529885	43529965	chr17:43529883-43529952
chr17	43530110	43530475	chr17:43530106-43530434
chr17	43532675	43532970	chr17:43532673-43532939
chr17	43533195	43533378	chr17:43533186-43533348
chr17	43533860	43534001	chr17:43533858-43533985
chr17	43544975	43545052	chr17:43544974-43545022
chr17	43545280	43545396	chr17:43545273-43545367
chr17	43545560	43545744	chr17:43545557-43545713
chr17	43545870	43545943	chr17:43545860-43545937
chr17	43546185	43546459	chr17:43546184-43546432
chr17	43574065	43574277	chr17:43574063-43574263
chr17	43579350	43579660	chr17:43579291-43579620
chr17	43640400	43642037	chr17:43640387-43642032
chr17	43643490	43643669	chr17:43643487-43643660
chr17	43651810	43652019	chr17:43651800-43652000
chr17	43661065	43661599	chr17:43661065-43661954
chr17	43661610	43661672	chr17:43661065-43661954
chr17	43661685	43661978	chr17:43661065-43661954
chr17	44076745	44078432	chr17:44076745-44078415
chr17	44078505	44078685	chr17:44078499-44078665
chr17	44078795	44078908	chr17:44078794-44078879

chr17	44079145	44079296	chr17:44079143-44079277
chr17	44080115	44080263	chr17:44080106-44080225
chr17	44080400	44080514	chr17:44080400-44080498
chr17	44080775	44080912	chr17:44080762-44080882
chr17	44082585	44082695	chr17:44082584-44082672
chr17	44082765	44082845	chr17:44082764-44082820
chr17	44083555	44083697	chr17:44083544-44083652
chr17	44083815	44083899	chr17:44083804-44083854
chr17	44084560	44084710	chr17:44084554-44084675
chr17	44085025	44085178	chr17:44085021-44085155
chr17	44086575	44086769	chr17:44086571-44086737
chr17	44087425	44087499	chr17:44087411-44087696
chr17	44087505	44087713	chr17:44087411-44087696
chr17	44088390	44088609	chr17:44088386-44088598
chr17	44091270	44091529	chr17:44091269-44091492
chr17	44091710	44091850	chr17:44091699-44091831
chr17	44092180	44092322	chr17:44092171-44092284
chr17	44092385	44092557	chr17:44092380-44092527
chr17	44092675	44092812	chr17:44092675-44092806
chr17	44093105	44093248	chr17:44093091-44093206
chr17	44093315	44093514	chr17:44093313-44093485
chr17	44093575	44093872	chr17:44093574-44093834
chr17	44110735	44111364	chr17:44110728-44111341
chr17	44114250	44114469	chr17:44114243-44114443
chr17	44117495	44117713	chr17:44117493-44117704
chr17	44123255	44123702	chr17:44123253-44123702
chr17	47733252	47733956	chr17:47733243-47733945
chr17	47734957	47735161	chr17:47734948-47735148
chr17	47742622	47742806	chr17:47742609-47742764
chr17	47743082	47743218	chr17:47743070-47743192
chr17	47744197	47744375	chr17:47744194-47744353
chr17	47744492	47744559	chr17:47744481-47744543
chr17	47744747	47745985	chr17:47744747-47746119
chr17	47746022	47746161	chr17:47744747-47746119
chr17	47844917	47845412	chr17:47844907-47848486
chr17	47845417	47845660	chr17:47844907-47848486
chr17	47845697	47847538	chr17:47844907-47848486
chr17	47847557	47848506	chr17:47844907-47848486
chr17	47850932	47851207	chr17:47850918-47851193
chr17	47853592	47853804	chr17:47853582-47853782
chr17	47855662	47855912	chr17:47855653-47855874
chr17	47896152	47896326	chr17:47896149-47896293
chr17	47897822	47897958	chr17:47897814-47897927
chr17	47906172	47906388	chr17:47906164-47906364
chr17	47909677	47909753	chr17:47909663-47909730
chr17	47914727	47914874	chr17:47914725-47914861
chr17	47915317	47915425	chr17:47915311-47915388
chr17	47916162	47917153	chr17:47916155-47917130
chr17	47917787	47917865	chr17:47917784-47917856
chr17	47922962	47923288	chr17:47922961-47923274
chr17	47924932	47925114	chr17:47924918-47925093
chr17	47925357	47925563	chr17:47925347-47925541
chr17	47927727	47928653	chr17:47927723-47928957
chr17	47928657	47928965	chr17:47927723-47928957
chr17	48048367	48048583	chr17:48048358-48048462;chr17:48048477-48048548
chr17	48048612	48048683	chr17:48048606-48048648
chr17	48048777	48048881	chr17:48048772-48048877
chr17	48050607	48050716	chr17:48050606-48051628
chr17	48050717	48051656	chr17:48050606-48051628
chr17	48053502	48053715	chr17:48053489-48053689
chr17	48054327	48054451	chr17:48054327-48054419
chr17	48054562	48054656	chr17:48054549-48054638
chr17	48054747	48055163	chr17:48054674-48055150

chr17	48055952	48056057	chr17:48055951-48056040
chr17	48056387	48056640	chr17:48056385-48056598
chr17	48057042	48057158	chr17:48057031-48057121
chr17	48057352	48057540	chr17:48057343-48057502
chr17	48058297	48058825	chr17:48058294-48061487
chr17	48058837	48061327	chr17:48058294-48061487
chr17	48528532	48531025	chr17:48528525-48530997
chr17	48540907	48541643	chr17:48540893-48541631
chr17	48542657	48543787	chr17:48542654-48543747
chr17	48543877	48543946	chr17:48543873-48543909
chr17	48544532	48545015	chr17:48544520-48544989
chr17	48548882	48549184	chr17:48548869-48551181
chr17	48549237	48549876	chr17:48548869-48551181
chr17	48549907	48551125	chr17:48548869-48551181
chr17	48552037	48552807	chr17:48552026-48553922
chr17	48552812	48553448	chr17:48552026-48553922
chr17	48553457	48553620	chr17:48552026-48553922
chr17	48553627	48553696	chr17:48552026-48553922
chr17	48553707	48553944	chr17:48552026-48553922
chr17	48554357	48554875	chr17:48554284-48554840
chr17	48555532	48555641	chr17:48555530-48555618
chr17	48556432	48556528	chr17:48556419-48556521
chr17	48567192	48567416	chr17:48567188-48567388
chr17	48573837	48574509	chr17:48573836-48574487
chr17	48575047	48575222	chr17:48575042-48575208
chr17	48575522	48575654	chr17:48575512-48577020
chr17	48575697	48576427	chr17:48575512-48577020
chr17	48576472	48576849	chr17:48575512-48577020
chr17	48576852	48577052	chr17:48575512-48577020
chr17	48577862	48579808	chr17:48577862-48580111
chr17	48579822	48580140	chr17:48577862-48580111
chr17	48582302	48582436	chr17:48582291-48582622
chr17	48582452	48582666	chr17:48582291-48582622
chr17	48590127	48590309	chr17:48590124-48590272
chr17	48591267	48591672	chr17:48591256-48592456
chr17	48591687	48591867	chr17:48591256-48592456
chr17	48591892	48591995	chr17:48591256-48592456
chr17	48591997	48592286	chr17:48591256-48592456
chr17	48592302	48592466	chr17:48591256-48592456
chr17	48593122	48593974	chr17:48593120-48593961
chr17	48595817	48596691	chr17:48595750-48596672
chr17	48596922	48597163	chr17:48596908-48597134
chr17	48597737	48598266	chr17:48597735-48598260
chr17	48603722	48604138	chr17:48603714-48604132
chr17	48604532	48604813	chr17:48604479-48604992
chr17	48604827	48605032	chr17:48604479-48604992
chr17	48607227	48607675	chr17:48607226-48608095
chr17	48607732	48607833	chr17:48607226-48608095
chr17	48607847	48608122	chr17:48607226-48608095
chr17	48609157	48609362	chr17:48609146-48609346
chr17	48610522	48611048	chr17:48610518-48611020
chr17	48611377	48611622	chr17:48611376-48611604
chr17	48612347	48612703	chr17:48612345-48613509
chr17	48612732	48613328	chr17:48612345-48613509
chr17	48613332	48613540	chr17:48612345-48613509
chr17	48614282	48614713	chr17:48614280-48614939
chr17	48614722	48614786	chr17:48614280-48614939
chr17	48614797	48614864	chr17:48614280-48614939
chr17	48614877	48614947	chr17:48614280-48614939
chr17	48621202	48623163	chr17:48621158-48623135
chr17	48625752	48626381	chr17:48625752-48626356
chr17	48633517	48633592	chr17:48633513-48633572
chr17	48724917	48727059	chr17:48724908-48727043

chr17	48727992	48728844	chr17:48727992-48729178
chr17	48728972	48729134	chr17:48727992-48729178
chr17	48729142	48729214	chr17:48727992-48729178
chr17	49289232	49291705	chr17:49289205-49291695
chr17	49294567	49295101	chr17:49294564-49298924
chr17	49295197	49295407	chr17:49294564-49298924
chr17	49295432	49295671	chr17:49294564-49298924
chr17	49296002	49296496	chr17:49294564-49298924
chr17	49296497	49296592	chr17:49294564-49298924
chr17	49296737	49297712	chr17:49294564-49298924
chr17	49297727	49298948	chr17:49294564-49298924
chr17	49311322	49311496	chr17:49311311-49311456
chr17	49311932	49312081	chr17:49311926-49312042
chr17	49312697	49312874	chr17:49312697-49312845
chr17	49316837	49317258	chr17:49316825-49317983
chr17	49317267	49317998	chr17:49316825-49317983
chr17	49322397	49322505	chr17:49322384-49322508
chr17	49360182	49360256	chr17:49360181-49360381
chr17	49360257	49360401	chr17:49360181-49360381
chr17	49361922	49362137	chr17:49361908-49362116
chr17	49362422	49362496	chr17:49362412-49362473
chr17	49404087	49404863	chr17:49404048-49405204
chr17	49404917	49404991	chr17:49404048-49405204
chr17	49405157	49405236	chr17:49404048-49405204
chr17	49406817	49406897	chr17:49406758-49406854
chr17	49409042	49409120	chr17:49409041-49409158
chr17	49409382	49409510	chr17:49409330-49409473
chr17	49411722	49411794	chr17:49411677-49411839
chr17	49413242	49413313	chr17:49413176-49413291
chr17	49414252	49414355	chr17:49414241-49414333
chr17	49414752	49414930	chr17:49414739-49414905
chr17	49970391	49970620	chr17:49970389-49970589
chr17	49973151	49973378	chr17:49973150-49973350
chr17	49973701	49973883	chr17:49973700-49973850
chr17	49990006	49990297	chr17:49990004-49991864
chr17	49990351	49991881	chr17:49990004-49991864
chr17	49992391	49992596	chr17:49992378-49992578
chr17	49993401	49993724	chr17:49993399-49993703
chr17	49994686	49995255	chr17:49994673-49995224
chr17	50834936	50835055	chr17:50834932-50835032
chr17	50836051	50836163	chr17:50836047-50836147
chr17	50837711	50837923	chr17:50837698-50837898
chr17	50839511	50839692	chr17:50839498-50839648
chr17	55265025	55265157	chr17:55265011-55265599
chr17	55265160	55265297	chr17:55265011-55265599
chr17	55265355	55265610	chr17:55265011-55265599
chr17	55265810	55265925	chr17:55265809-55265886
chr17	55266235	55266340	chr17:55266221-55266315
chr17	55267600	55267698	chr17:55267590-55268086
chr17	55267715	55268107	chr17:55267590-55268086
chr17	55283495	55283986	chr17:55283481-55283697;chr17:55283699-55283953
chr17	55284010	55284084	chr17:55284011-55284057
chr17	55285545	55285777	chr17:55285545-55285745
chr17	55288890	55288983	chr17:55288880-55288983
chr17	55315230	55315480	chr17:55315226-55315447
chr17	55318955	55319070	chr17:55318952-55319054
chr17	55320660	55321017	chr17:55320660-55325065
chr17	55321030	55321963	chr17:55320660-55325065
chr17	55322010	55322388	chr17:55320660-55325065
chr17	55322390	55324797	chr17:55320660-55325065
chr17	55324825	55325058	chr17:55320660-55325065
chr17	57971558	57972799	chr17:57971546-57974900
chr17	57972808	57973018	chr17:57971546-57974900

chr17	57973023	57973088	chr17:57971546-57974900
chr17	57973093	57973303	chr17:57971546-57974900
chr17	57973313	57973734	chr17:57971546-57974900
chr17	57973758	57974911	chr17:57971546-57974900
chr17	57979153	57979230	chr17:57979151-57979313
chr17	57979273	57979353	chr17:57979151-57979313
chr17	57980613	57980829	chr17:57980602-57980804
chr17	57981873	57981950	chr17:57981872-57981936
chr17	57982708	57983417	chr17:57982698-57983393
chr17	57984138	57984349	chr17:57984130-57984330
chr17	57985253	57985365	chr17:57985253-57985348
chr17	57985998	57986159	chr17:57985984-57986144
chr17	57988078	57988190	chr17:57988078-57988259
chr17	57988203	57988279	chr17:57988078-57988259
chr17	61399899	61400356	chr17:61399895-61400571
chr17	61400374	61400583	chr17:61399895-61400571
chr17	61401694	61401972	chr17:61401683-61401951
chr17	61403064	61403246	chr17:61403060-61403207
chr17	61404429	61404528	chr17:61404420-61404497
chr17	61404614	61404795	chr17:61404605-61404769
chr17	61405204	61405877	chr17:61405201-61405836
chr17	61408054	61409072	chr17:61408053-61409466
chr17	61409089	61409500	chr17:61408053-61409466
chr17	61452404	61452582	chr17:61452403-61452577
chr17	61454539	61454748	chr17:61454527-61454727
chr17	61456449	61456704	chr17:61456445-61456676
chr17	61457539	61457656	chr17:61457536-61457631
chr17	61464219	61464439	chr17:61464213-61464411
chr17	61465829	61465966	chr17:61465818-61465938
chr17	61467509	61467694	chr17:61467509-61467657
chr17	61478354	61478463	chr17:61478350-61478429
chr17	61478634	61478820	chr17:61478626-61478789
chr17	61479894	61479995	chr17:61479880-61479969
chr17	61480089	61480349	chr17:61480089-61480319
chr17	61481324	61481399	chr17:61481324-61481455
chr17	61481414	61481490	chr17:61481324-61481455
chr17	61482909	61483611	chr17:61482896-61485110
chr17	61483649	61484733	chr17:61482896-61485110
chr17	61484759	61484925	chr17:61482896-61485110
chr17	61484974	61485153	chr17:61482896-61485110
chr17	61681279	61682856	chr17:61681265-61684140
chr17	61683149	61684048	chr17:61681265-61684140
chr17	61684054	61684155	chr17:61681265-61684140
chr17	61684994	61685067	chr17:61684771-61686165
chr17	61685074	61685214	chr17:61684771-61686165
chr17	61685244	61686187	chr17:61684771-61686165
chr17	61693429	61693538	chr17:61693429-61693512
chr17	61715964	61716063	chr17:61715950-61716063
chr17	61739064	61739447	chr17:61739056-61739410
chr17	61743024	61743159	chr17:61743012-61743134
chr17	61744434	61744607	chr17:61744431-61744591
chr17	61776404	61776585	chr17:61776400-61776562
chr17	61780274	61780410	chr17:61780260-61780401
chr17	61780839	61781026	chr17:61780839-61781005
chr17	61784279	61784449	chr17:61784269-61784424
chr17	61793604	61793743	chr17:61793596-61793729
chr17	61799109	61799315	chr17:61799099-61799299
chr17	61801264	61801505	chr17:61801252-61801474
chr17	61808479	61808783	chr17:61808466-61808757
chr17	61810094	61810311	chr17:61810083-61810283
chr17	61847104	61847246	chr17:61847100-61847220
chr17	61849129	61849273	chr17:61849128-61849256
chr17	61857059	61857242	chr17:61857057-61857231

chr17	61859809	61859944	chr17:61859795-61859907
chr17	61861454	61861596	chr17:61861446-61861569
chr17	61861909	61862088	chr17:61861909-61862075
chr17	61863294	61863530	chr17:61863283-61863521
chr17	72121032	72121849	chr17:72121019-72121822
chr17	72122732	72123011	chr17:72122718-72122972
chr17	72123547	72124485	chr17:72123542-72126420
chr17	72124522	72125005	chr17:72123542-72126420
chr17	72125007	72125198	chr17:72123542-72126420
chr17	72125207	72125543	chr17:72123542-72126420
chr17	72125567	72125888	chr17:72123542-72126420
chr17	72125917	72126085	chr17:72123542-72126420
chr17	72126102	72126365	chr17:72123542-72126420
chr17	72126377	72126449	chr17:72123542-72126420
chr17	76136357	76138143	chr17:76136332-76138120
chr17	76139902	76140598	chr17:76139897-76140564
chr17	76141117	76141337	chr17:76141113-76141299
chr17	78214337	78214438	chr17:78214327-78214427
chr17	78214692	78214867	chr17:78214679-78214829
chr17	78216682	78216786	chr17:78216681-78216781
chr17	78222827	78223008	chr17:78222827-78222977
chr17	78223467	78223651	chr17:78223464-78223614
chr17	78378642	78378823	chr17:78378639-78378808
chr17	78384437	78384641	chr17:78384426-78384626
chr17	78392482	78392686	chr17:78392475-78392665
chr17	78396307	78396418	chr17:78396307-78396385
chr17	78398252	78398366	chr17:78398251-78398351
chr17	78399302	78399582	chr17:78399288-78399537
chr17	78400687	78400894	chr17:78400676-78400855
chr17	78403567	78404103	chr17:78403567-78404089
chr17	78414887	78415065	chr17:78414878-78415027
chr17	78419552	78419943	chr17:78419545-78419927
chr17	78420222	78420340	chr17:78420221-78420323
chr17	78424307	78424601	chr17:78424303-78424557
chr17	78424927	78425141	chr17:78424917-78425114
chr17	79090839	79090908	chr17:79090735-79090885
chr17	79094389	79094557	chr17:79094379-79094529
chr17	79095514	79095596	chr17:79095512-79095574
chr17	79192414	79192627	chr17:79192413-79192613
chr17	80470889	80471064	chr17:80470884-80471034
chr17	80471744	80471847	chr17:80471731-80471831
chr17	80472459	80472687	chr17:80472455-80472655
chr17	81918358	81921567	chr17:81918344-81923057
chr17	81921573	81921674	chr17:81918344-81923057
chr17	81921703	81923096	chr17:81918344-81923057
chr17	81923163	81923239	chr17:81923149-81923214
chr17	81923353	81923563	chr17:81923352-81923532
chr17	81926343	81926556	chr17:81926338-81926538
chr17	81927528	81927704	chr17:81927527-81927714
chr17	82519718	82520329	chr17:82519712-82520307
chr17	82559353	82559493	chr17:82559339-82559480
chr17	82563358	82563568	chr17:82563353-82563548
chr17	82568063	82568230	chr17:82568053-82568201
chr17	82571723	82571908	chr17:82571723-82571870
chr17	82582753	82582960	chr17:82582740-82582934
chr17	82584023	82584226	chr17:82584012-82584188
chr17	82585903	82586224	chr17:82585903-82586200
chr17	82587063	82587594	chr17:82587062-82587562
chr17	82593413	82593972	chr17:82593399-82593954
chr17	82595793	82596223	chr17:82595781-82596191
chr17	82597093	82597320	chr17:82597093-82597293
chr17	82600103	82600276	chr17:82600091-82600244
chr17	82601313	82601727	chr17:82601302-82604607

chr17	82601753	82602324	chr17:82601302-82604607
chr17	82602328	82603719	chr17:82601302-82604607
chr17	82603733	82604568	chr17:82601302-82604607
chr17	82644178	82644670	chr17:82644173-82644662
chr17	82829448	82829995	chr17:82829434-82830877
chr17	82830018	82830896	chr17:82829434-82830877
chr17	82831018	82832676	chr17:82831018-82832636
chr17	82837788	82838003	chr17:82837774-82837974
chr17	82839938	82840601	chr17:82839926-82840578
chr18	3411615	3411756	chr18:3411607-3412254
chr18	3411770	3412286	chr18:3411607-3412254
chr18	3412995	3413068	chr18:3412853-3413049
chr18	3418150	3418248	chr18:3418142-3418215
chr18	3437370	3437581	chr18:3437366-3437566
chr18	3447620	3447824	chr18:3447608-3447797
chr18	3448510	3448616	chr18:3448498-3448597
chr18	3449415	3450544	chr18:3449412-3450505
chr18	3451595	3452410	chr18:3451592-3452382
chr18	3453775	3453887	chr18:3453773-3453856
chr18	3455165	3455263	chr18:3455151-3455258
chr18	3455420	3457131	chr18:3455413-3457113
chr18	3457370	3458009	chr18:3457364-3459978
chr18	3458015	3458206	chr18:3457364-3459978
chr18	3458215	3459999	chr18:3457364-3459978
chr18	5289030	5289229	chr18:5289018-5292204
chr18	5289265	5290581	chr18:5289018-5292204
chr18	5290600	5292205	chr18:5289018-5292204
chr18	5293250	5293364	chr18:5293243-5293327
chr18	5293970	5294043	chr18:5293971-5294001
chr18	5295660	5295737	chr18:5295651-5295702
chr18	5295820	5296197	chr18:5295812-5296195
chr18	5297010	5297089	chr18:5297011-5297053
chr18	22169449	22169695	chr18:22169442-22169682
chr18	22170944	22172106	chr18:22170932-22172279
chr18	22172139	22172302	chr18:22170932-22172279
chr18	22176959	22177143	chr18:22176954-22177121
chr18	22181454	22181615	chr18:22181452-22181578
chr18	22182759	22182874	chr18:22182756-22182844
chr18	22182939	22183053	chr18:22182939-22183043
chr18	22185899	22186127	chr18:22185898-22186098
chr18	22200664	22201130	chr18:22200655-22202528
chr18	22201134	22201242	chr18:22200655-22202528
chr18	22201249	22201456	chr18:22200655-22202528
chr18	22201469	22201567	chr18:22200655-22202528
chr18	22201584	22201894	chr18:22200655-22202528
chr18	22201954	22202545	chr18:22200655-22202528
chr18	35332211	35332911	chr18:35332211-35339073
chr18	35332931	35333527	chr18:35332211-35339073
chr18	35333596	35335200	chr18:35332211-35339073
chr18	35335201	35335753	chr18:35332211-35339073
chr18	35335771	35337210	chr18:35332211-35339073
chr18	35337216	35337908	chr18:35332211-35339073
chr18	35337916	35338091	chr18:35332211-35339073
chr18	35338116	35339095	chr18:35332211-35339073
chr18	35339831	35340005	chr18:35339828-35339976
chr18	35340241	35340761	chr18:35340230-35340733
chr18	35342476	35342617	chr18:35342474-35342675
chr18	35343756	35343971	chr18:35343743-35343932
chr18	35344366	35344468	chr18:35344359-35344456
chr18	35344676	35344753	chr18:35344668-35344721
chr18	35345291	35345394	chr18:35345278-35345482
chr18	35345406	35345521	chr18:35345278-35345482
chr18	47808963	47810325	chr18:47808956-47841950

chr18	47810328	47810635	chr18:47808956-47841950
chr18	47810773	47811188	chr18:47808956-47841950
chr18	47811488	47811970	chr18:47808956-47841950
chr18	47812663	47812938	chr18:47808956-47841950
chr18	47813208	47813272	chr18:47808956-47841950
chr18	47813753	47816720	chr18:47808956-47841950
chr18	47817038	47817477	chr18:47808956-47841950
chr18	47817588	47818242	chr18:47808956-47841950
chr18	47818243	47818951	chr18:47808956-47841950
chr18	47819008	47819453	chr18:47808956-47841950
chr18	47819813	47819892	chr18:47808956-47841950
chr18	47820018	47820905	chr18:47808956-47841950
chr18	47820933	47821404	chr18:47808956-47841950
chr18	47821413	47821590	chr18:47808956-47841950
chr18	47821598	47822670	chr18:47808956-47841950
chr18	47822978	47824559	chr18:47808956-47841950
chr18	47824568	47824735	chr18:47808956-47841950
chr18	47824738	47825536	chr18:47808956-47841950
chr18	47825553	47827442	chr18:47808956-47841950
chr18	47828958	47830138	chr18:47808956-47841950
chr18	47830168	47830306	chr18:47808956-47841950
chr18	47830588	47833066	chr18:47808956-47841950
chr18	47833073	47834829	chr18:47808956-47841950
chr18	47834833	47836597	chr18:47808956-47841950
chr18	47836623	47837292	chr18:47808956-47841950
chr18	47837583	47838102	chr18:47808956-47841950
chr18	47838123	47839010	chr18:47808956-47841950
chr18	47839033	47840070	chr18:47808956-47841950
chr18	47840138	47841146	chr18:47808956-47841950
chr18	47841163	47841336	chr18:47808956-47841950
chr18	47841348	47841972	chr18:47808956-47841950
chr18	47844913	47845510	chr18:47844911-47845484
chr18	47845668	47845809	chr18:47845662-47845800
chr18	47848478	47848695	chr18:47848474-47848687
chr18	47851273	47851345	chr18:47851273-47851327
chr18	47865058	47865176	chr18:47865058-47865133
chr18	47868273	47868474	chr18:47868262-47868457
chr18	47869243	47869457	chr18:47869242-47869436
chr18	47870483	47870586	chr18:47870472-47870564
chr18	47886818	47886928	chr18:47886818-47886923
chr18	47895273	47895768	chr18:47895266-47895725
chr18	47896523	47896850	chr18:47896520-47896811
chr18	47920083	47920194	chr18:47920071-47920169
chr18	47922548	47922696	chr18:47922546-47922655
chr18	47923568	47923680	chr18:47923565-47923642
chr18	47928893	47929097	chr18:47928887-47929087
chr18	47930263	47930566	chr18:47930251-47930599
chr18	47930803	47931148	chr18:47930795-47931146
chr18	51028393	51028540	chr18:51028393-51028665
chr18	51028573	51028687	chr18:51028393-51028665
chr18	51029618	51029767	chr18:51029613-51029744
chr18	51030113	51030641	chr18:51030099-51030623
chr18	51044963	51045084	chr18:51044958-51045051
chr18	51046923	51047313	chr18:51046919-51047295
chr18	51048688	51048882	chr18:51048685-51048860
chr18	51049288	51049360	chr18:51049294-51049324
chr18	51054783	51055035	chr18:51054780-51054993
chr18	51058133	51058274	chr18:51058124-51058244
chr18	51058343	51058488	chr18:51058339-51058456
chr18	51059868	51059946	chr18:51059865-51059916
chr18	51065433	51065640	chr18:51065422-51065606
chr18	51067018	51067201	chr18:51067018-51067187
chr18	51069898	51070110	chr18:51069889-51070089

chr18	51076638	51076796	chr18:51076637-51077130
chr18	51076798	51077143	chr18:51076637-51077130
chr18	51077263	51077434	chr18:51077250-51077419
chr18	51078268	51078644	chr18:51078255-51085045
chr18	51078648	51079317	chr18:51078255-51085045
chr18	51079323	51079809	chr18:51078255-51085045
chr18	51079818	51079966	chr18:51078255-51085045
chr18	51079983	51080551	chr18:51078255-51085045
chr18	51080823	51082721	chr18:51078255-51085045
chr18	51082743	51083257	chr18:51078255-51085045
chr18	51083263	51083433	chr18:51078255-51085045
chr18	51083463	51083711	chr18:51078255-51085045
chr18	51083718	51083995	chr18:51078255-51085045
chr18	51084098	51084209	chr18:51078255-51085045
chr18	51084213	51085082	chr18:51078255-51085045
chr18	55227884	55228031	chr18:55227880-55228030
chr18	55228224	55228376	chr18:55228220-55228361
chr18	55228849	55228975	chr18:55228846-55228946
chr18	55336609	55336836	chr18:55336609-55336809
chr18	57435694	57436230	chr18:57435684-57436944
chr18	57436259	57436954	chr18:57435684-57436944
chr18	57439704	57439901	chr18:57439694-57439894
chr18	57444389	57444468	chr18:57444375-57444427
chr18	57467159	57467236	chr18:57467159-57467220
chr18	57476439	57477414	chr18:57476436-57491297
chr18	57477419	57478453	chr18:57476436-57491297
chr18	57478459	57479324	chr18:57476436-57491297
chr18	57479344	57480569	chr18:57476436-57491297
chr18	57480589	57481136	chr18:57476436-57491297
chr18	57481174	57483117	chr18:57476436-57491297
chr18	57483154	57484087	chr18:57476436-57491297
chr18	57484089	57484186	chr18:57476436-57491297
chr18	57484219	57485955	chr18:57476436-57491297
chr18	57485979	57486602	chr18:57476436-57491297
chr18	57486619	57491334	chr18:57476436-57491297
chr18	59267054	59269540	chr18:59267034-59269501
chr18	59270564	59270777	chr18:59270557-59270757
chr18	59272369	59272648	chr18:59272360-59272614
chr18	59272929	59273414	chr18:59272917-59273393
chr18	59273874	59274113	chr18:59273861-59274086
chr18	75210768	75210877	chr18:75210754-75211243
chr18	75210898	75210994	chr18:75210754-75211243
chr18	75210998	75211274	chr18:75210754-75211243
chr18	75211538	75211668	chr18:75211528-75211916
chr18	75211673	75211946	chr18:75211528-75211916
chr18	75221068	75221171	chr18:75221057-75221154
chr18	75244588	75244661	chr18:75244575-75244622
chr18	75245238	75245412	chr18:75245227-75245400
chr18	75248283	75248494	chr18:75248270-75248470
chr18	75285458	75289062	chr18:75285447-75289950
chr18	75289088	75289272	chr18:75285447-75289950
chr18	75289283	75289783	chr18:75285447-75289950
chr18	75289803	75289974	chr18:75285447-75289950
chr18	78980283	78980385	chr18:78980274-78980356
chr18	78985018	78985089	chr18:78985009-78985070
chr18	78988723	78988929	chr18:78988711-78988911
chr18	78991693	78995473	chr18:78991681-78995462
chr18	78996903	78997883	chr18:78996890-78999974
chr18	78997893	78998024	chr18:78996890-78999974
chr18	78998028	78998295	chr18:78996890-78999974
chr18	78998303	78998616	chr18:78996890-78999974
chr18	78998628	78999360	chr18:78996890-78999974
chr18	78999413	79000002	chr18:78996890-78999974

chr18	79002288	79002538	chr18:79002288-79002677
chr18	79002553	79002691	chr18:79002288-79002677
chr18	79395868	79396383	chr18:79395855-79396351
chr18	79400283	79400482	chr18:79400273-79400479
chr18	79410403	79411525	chr18:79410402-79411501
chr18	79433588	79433767	chr18:79433578-79433738
chr18	79448278	79448419	chr18:79448269-79448401
chr18	79448793	79449003	chr18:79448781-79448984
chr18	79450953	79451133	chr18:79450953-79451126
chr18	79451683	79451829	chr18:79451675-79451816
chr18	79461323	79461400	chr18:79461310-79461366
chr18	79467463	79468187	chr18:79467449-79470014
chr18	79468193	79468893	chr18:79467449-79470014
chr18	79469038	79470045	chr18:79467449-79470014
chr18	79474518	79474597	chr18:79474480-79474680
chr18	79486253	79486958	chr18:79486247-79486937
chr18	79493398	79493864	chr18:79493395-79493823
chr18	79523923	79524070	chr18:79523923-79524035
chr18	79527118	79527438	chr18:79527118-79527402
chr18	79527528	79529363	chr18:79527527-79529325
chr19	925780	925932	chr19:925780-925920
chr19	927070	927268	chr19:927059-927259
chr19	928680	929184	chr19:928677-929145
chr19	929270	929482	chr19:929261-929896
chr19	929500	929926	chr19:929261-929896
chr19	932500	932781	chr19:932417-932742
chr19	960095	960170	chr19:960091-960164
chr19	964250	964465	chr19:964247-964431
chr19	964835	965115	chr19:964832-965080
chr19	966575	966913	chr19:966571-966868
chr19	968415	968526	chr19:968404-968503
chr19	971945	972193	chr19:971877-975934
chr19	972250	972601	chr19:971877-975934
chr19	972610	972803	chr19:971877-975934
chr19	972820	973109	chr19:971877-975934
chr19	973390	975596	chr19:971877-975934
chr19	975615	975743	chr19:971877-975934
chr19	975775	975955	chr19:971877-975934
chr19	1609300	1609430	chr19:1609289-1611849
chr19	1609435	1610422	chr19:1609289-1611849
chr19	1610425	1611158	chr19:1609289-1611849
chr19	1611185	1611856	chr19:1609289-1611849
chr19	1612220	1612473	chr19:1612206-1612433
chr19	1615295	1615853	chr19:1615284-1615821
chr19	1619130	1619485	chr19:1619110-1619474
chr19	1619790	1619887	chr19:1619779-1619853
chr19	1620970	1621081	chr19:1620967-1621046
chr19	1621135	1621212	chr19:1621132-1621191
chr19	1621840	1621989	chr19:1621837-1621970
chr19	1622055	1622235	chr19:1622053-1622223
chr19	1622320	1622423	chr19:1622312-1622415
chr19	1623950	1624018	chr19:1623950-1624000
chr19	1625575	1625718	chr19:1625575-1625708
chr19	1627365	1627440	chr19:1627358-1627426
chr19	1631955	1632134	chr19:1631950-1632116
chr19	1632340	1632449	chr19:1632331-1632405
chr19	1646355	1646424	chr19:1646354-1646427
chr19	1650180	1650778	chr19:1650176-1650745
chr19	1651050	1651368	chr19:1651049-1651356
chr19	1652300	1652408	chr19:1652299-1652605
chr19	1652425	1652630	chr19:1652299-1652605
chr19	1752385	1753822	chr19:1752372-1754854
chr19	1753840	1754894	chr19:1752372-1754854

chr19	1761900	1762117	chr19:1761898-1762098
chr19	1775165	1776378	chr19:1775152-1780988
chr19	1776385	1777010	chr19:1775152-1780988
chr19	1777070	1777326	chr19:1775152-1780988
chr19	1777485	1777694	chr19:1775152-1780988
chr19	1778010	1778111	chr19:1775152-1780988
chr19	1778220	1778292	chr19:1775152-1780988
chr19	1778370	1778719	chr19:1775152-1780988
chr19	1778905	1779404	chr19:1775152-1780988
chr19	1779455	1779557	chr19:1775152-1780988
chr19	1779600	1780977	chr19:1775152-1780988
chr19	1852400	1853977	chr19:1852398-1854760
chr19	1853995	1854759	chr19:1852398-1854760
chr19	1856865	1857071	chr19:1856854-1857054
chr19	1862805	1862874	chr19:1862793-1862858
chr19	1863040	1863601	chr19:1863040-1863568
chr19	3267430	3267651	chr19:3267428-3267628
chr19	3290275	3290386	chr19:3290274-3290374
chr19	3293370	3293509	chr19:3293318-3293486
chr19	3296760	3296938	chr19:3296757-3296907
chr19	3359563	3359717	chr19:3359562-3359685
chr19	3366558	3366686	chr19:3366548-3366666
chr19	3381713	3382281	chr19:3381711-3382243
chr19	3404788	3405001	chr19:3404774-3404974
chr19	3410608	3411025	chr19:3410595-3411178
chr19	3425108	3425187	chr19:3425105-3425177
chr19	3433528	3433632	chr19:3433517-3433592
chr19	3434283	3434431	chr19:3434276-3434400
chr19	3435083	3435232	chr19:3435082-3435207
chr19	3449013	3449163	chr19:3449013-3449139
chr19	3452493	3452700	chr19:3452481-3452666
chr19	3453763	3453945	chr19:3453762-3453916
chr19	3456558	3456661	chr19:3456549-3456635
chr19	3457888	3458031	chr19:3457882-3457989
chr19	3462753	3462930	chr19:3462751-3469217
chr19	3462963	3465157	chr19:3462751-3469217
chr19	3465178	3465322	chr19:3462751-3469217
chr19	3465448	3466042	chr19:3462751-3469217
chr19	3466068	3466212	chr19:3462751-3469217
chr19	3466243	3467746	chr19:3462751-3469217
chr19	3467783	3468621	chr19:3462751-3469217
chr19	3468708	3468779	chr19:3462751-3469217
chr19	3468798	3468943	chr19:3462751-3469217
chr19	3468953	3469084	chr19:3462751-3469217
chr19	3469178	3469248	chr19:3462751-3469217
chr19	3769408	3770980	chr19:3769088-3770959
chr19	3771538	3772003	chr19:3771526-3772010
chr19	3772168	3772243	chr19:3772162-3772221
chr19	4044388	4044592	chr19:4044363-4048244
chr19	4044603	4044676	chr19:4044363-4048244
chr19	4044733	4044829	chr19:4044363-4048244
chr19	4044833	4045489	chr19:4044363-4048244
chr19	4045493	4046349	chr19:4044363-4048244
chr19	4046448	4046579	chr19:4044363-4048244
chr19	4046618	4046763	chr19:4044363-4048244
chr19	4046828	4047601	chr19:4044363-4048244
chr19	4047713	4048275	chr19:4044363-4048244
chr19	4053973	4055272	chr19:4053970-4055247
chr19	4058783	4059002	chr19:4058781-4058981
chr19	4065443	4065744	chr19:4065429-4065732
chr19	4066693	4066961	chr19:4066681-4066945
chr19	4153608	4153791	chr19:4153600-4153774
chr19	4154903	4155045	chr19:4154898-4155027

chr19	4156998	4157332	chr19:4156994-4157295
chr19	4159673	4159805	chr19:4159663-4159782
chr19	4164508	4164652	chr19:4164502-4164640
chr19	4168353	4168470	chr19:4168350-4168457
chr19	4170143	4170227	chr19:4170139-4170208
chr19	4171103	4171211	chr19:4171090-4171175
chr19	4171388	4171487	chr19:4171382-4171479
chr19	4171658	4172265	chr19:4171655-4173054
chr19	4172578	4172658	chr19:4171655-4173054
chr19	4172713	4173089	chr19:4171655-4173054
chr19	5993170	5994289	chr19:5993163-5994950
chr19	5994290	5994429	chr19:5993163-5994950
chr19	5994435	5994969	chr19:5993163-5994950
chr19	5995600	5995680	chr19:5995600-5995643
chr19	5997065	5997243	chr19:5997059-5997213
chr19	6001815	6002039	chr19:6001814-6002023
chr19	6002720	6002912	chr19:6002720-6002870
chr19	6004200	6004313	chr19:6004200-6004298
chr19	6007015	6007191	chr19:6007011-6007166
chr19	6007700	6007837	chr19:6007689-6007802
chr19	6008110	6008255	chr19:6008105-6008226
chr19	6010135	6010286	chr19:6010135-6010251
chr19	6012995	6013126	chr19:6012985-6013105
chr19	6016100	6016305	chr19:6016089-6016271
chr19	6023095	6023245	chr19:6023095-6023205
chr19	6026165	6026269	chr19:6026162-6026237
chr19	6027170	6027355	chr19:6027163-6027334
chr19	6039990	6040276	chr19:6039979-6040241
chr19	6042050	6042166	chr19:6042043-6042123
chr19	6044200	6044303	chr19:6044192-6044282
chr19	6047420	6047530	chr19:6047406-6047504
chr19	6049145	6049260	chr19:6049135-6049232
chr19	6057165	6057320	chr19:6057161-6057283
chr19	6072015	6072186	chr19:6072004-6072147
chr19	6078140	6078337	chr19:6078100-6078334
chr19	6110405	6110613	chr19:6110392-6110601
chr19	6113575	6113770	chr19:6113561-6113761
chr19	6132790	6132974	chr19:6132747-6132947
chr19	6148230	6148353	chr19:6148230-6148329
chr19	6175875	6176483	chr19:6175872-6176443
chr19	6190050	6190263	chr19:6189727-6190433
chr19	6190275	6190459	chr19:6189727-6190433
chr19	6190855	6191244	chr19:6190842-6192053
chr19	6191250	6191382	chr19:6190842-6192053
chr19	6191415	6191527	chr19:6190842-6192053
chr19	6191555	6192076	chr19:6190842-6192053
chr19	6413350	6413641	chr19:6413347-6414120
chr19	6413665	6413767	chr19:6413347-6414120
chr19	6413840	6414045	chr19:6413347-6414120
chr19	6414075	6414154	chr19:6413347-6414120
chr19	6414460	6414636	chr19:6414456-6415301
chr19	6414715	6415091	chr19:6414456-6415301
chr19	6415110	6415317	chr19:6414456-6415301
chr19	6415380	6415492	chr19:6415379-6415457
chr19	6415535	6415756	chr19:6415533-6415734
chr19	6415810	6415927	chr19:6415807-6415896
chr19	6416310	6416439	chr19:6416297-6416407
chr19	6416490	6416676	chr19:6416489-6416650
chr19	6416745	6416888	chr19:6416737-6416882
chr19	6416995	6417116	chr19:6416986-6417087
chr19	6417740	6417856	chr19:6417738-6417841
chr19	6417980	6418103	chr19:6417980-6418079
chr19	6418485	6418598	chr19:6418482-6418581

chr19	6418705	6418918	chr19:6418700-6418876
chr19	6419215	6419292	chr19:6419202-6419260
chr19	6420075	6420153	chr19:6420072-6420144
chr19	6420435	6420508	chr19:6420421-6420471
chr19	6421285	6421360	chr19:6421277-6421317
chr19	6421645	6421725	chr19:6421649-6421688
chr19	6422350	6422456	chr19:6422339-6422436
chr19	6424145	6424348	chr19:6424137-6424327
chr19	6424455	6424750	chr19:6424452-6424794
chr19	6424755	6424828	chr19:6424452-6424794
chr19	9140382	9140624	chr19:9140379-9140592
chr19	9150397	9150607	chr19:9150394-9150594
chr19	9155932	9156078	chr19:9155924-9156041
chr19	9156612	9156765	chr19:9156611-9156748
chr19	9157267	9157380	chr19:9157267-9157394
chr19	9157837	9158100	chr19:9157833-9158075
chr19	9158837	9158941	chr19:9158825-9158908
chr19	9160122	9162148	chr19:9160113-9163424
chr19	9162152	9163440	chr19:9160113-9163424
chr19	9363022	9363318	chr19:9363019-9363291
chr19	9371612	9371787	chr19:9371609-9371752
chr19	9376382	9376452	chr19:9376315-9376423
chr19	9380057	9380166	chr19:9380056-9380139
chr19	11887793	11887977	chr19:11887783-11887986
chr19	11903613	11903686	chr19:11903572-11903699
chr19	11903928	11904000	chr19:11903904-11903965
chr19	11904683	11905059	chr19:11904648-11906267
chr19	11905153	11905385	chr19:11904648-11906267
chr19	11905388	11905467	chr19:11904648-11906267
chr19	11905468	11905542	chr19:11904648-11906267
chr19	11905728	11905828	chr19:11904648-11906267
chr19	11905998	11906248	chr19:11904648-11906267
chr19	11913673	11914063	chr19:11913408-11914329
chr19	12014834	12015080	chr19:12014731-12016666
chr19	12015174	12015253	chr19:12014731-12016666
chr19	12015279	12015581	chr19:12014731-12016666
chr19	12015614	12015688	chr19:12014731-12016666
chr19	12015709	12016698	chr19:12014731-12016666
chr19	12017884	12017953	chr19:12017875-12017936
chr19	12018169	12018245	chr19:12018165-12018677
chr19	12018279	12018709	chr19:12018165-12018677
chr19	12021934	12022017	chr19:12021929-12022189
chr19	12022084	12022159	chr19:12021929-12022189
chr19	12030139	12030218	chr19:12030139-12030198
chr19	12034839	12034916	chr19:12034827-12034874
chr19	12035539	12035753	chr19:12035536-12035741
chr19	12163064	12163241	chr19:12163063-12163206
chr19	12163764	12163837	chr19:12163759-12163816
chr19	12164714	12164853	chr19:12164628-12164874
chr19	12165519	12165734	chr19:12165510-12165710
chr19	12169284	12169430	chr19:12169281-12169391
chr19	12185854	12185934	chr19:12185784-12185911
chr19	12186119	12186190	chr19:12186113-12186174
chr19	12186569	12187019	chr19:12186569-12188440
chr19	12187114	12187213	chr19:12186569-12188440
chr19	12187214	12187283	chr19:12186569-12188440
chr19	12187299	12187442	chr19:12186569-12188440
chr19	12187459	12187592	chr19:12186569-12188440
chr19	12187624	12187698	chr19:12186569-12188440
chr19	12187734	12187871	chr19:12186569-12188440
chr19	12187884	12188461	chr19:12186569-12188440
chr19	12188799	12189325	chr19:12188755-12189881
chr19	12189664	12189914	chr19:12188755-12189881

chr19	12791504	12792205	chr19:12791495-12792173
chr19	12792744	12793257	chr19:12792732-12793315
chr19	12884434	12884811	chr19:12884422-12885060
chr19	12884829	12885075	chr19:12884422-12885060
chr19	12885329	12886170	chr19:12885316-12886142
chr19	12887054	12887215	chr19:12887053-12887181
chr19	12995619	12995892	chr19:12995607-12995864
chr19	13012189	13012298	chr19:13012187-13012266
chr19	13017149	13017363	chr19:13017148-13017348
chr19	13023989	13024059	chr19:13023990-13024034
chr19	13024579	13024737	chr19:13024579-13024723
chr19	13024944	13025571	chr19:13024938-13025552
chr19	13070004	13070081	chr19:13070002-13070069
chr19	13073049	13073124	chr19:13073046-13073109
chr19	13073424	13073537	chr19:13073421-13073496
chr19	13073909	13074061	chr19:13073905-13074026
chr19	13075539	13075683	chr19:13075534-13075671
chr19	13078619	13078759	chr19:13078612-13078735
chr19	13081689	13081896	chr19:13081679-13081855
chr19	13087989	13088171	chr19:13087988-13088136
chr19	13090299	13090410	chr19:13090298-13090390
chr19	13094634	13095164	chr19:13094634-13098796
chr19	13095199	13096211	chr19:13094634-13098796
chr19	13096214	13096953	chr19:13094634-13098796
chr19	13096989	13097062	chr19:13094634-13098796
chr19	13097064	13097191	chr19:13094634-13098796
chr19	13097204	13098681	chr19:13094634-13098796
chr19	13098684	13098785	chr19:13094634-13098796
chr19	13118349	13118482	chr19:13118336-13118454
chr19	13120384	13120592	chr19:13120370-13120570
chr19	13135209	13136189	chr19:13135199-13136153
chr19	13136234	13136413	chr19:13136231-13136405
chr19	13137274	13137389	chr19:13137270-13137376
chr19	13137489	13137590	chr19:13137477-13137575
chr19	13138159	13138439	chr19:13138146-13141141
chr19	13138449	13139145	chr19:13138146-13141141
chr19	13139169	13139474	chr19:13138146-13141141
chr19	13139524	13139785	chr19:13138146-13141141
chr19	13139829	13141169	chr19:13138146-13141141
chr19	13151984	13153028	chr19:13151974-13154908
chr19	13153044	13154198	chr19:13151974-13154908
chr19	13154209	13154939	chr19:13151974-13154908
chr19	13909829	13909971	chr19:13909822-13909958
chr19	13912334	13912478	chr19:13912322-13912438
chr19	13912534	13912609	chr19:13912527-13912593
chr19	13913179	13913316	chr19:13913167-13913302
chr19	13913409	13913654	chr19:13913403-13913638
chr19	13918079	13918224	chr19:13918069-13918194
chr19	13918509	13918612	chr19:13918503-13918576
chr19	13918759	13918828	chr19:13918745-13918817
chr19	13918924	13919061	chr19:13918911-13919042
chr19	13919129	13919236	chr19:13919129-13919202
chr19	13919829	13919964	chr19:13919817-13919951
chr19	13920559	13920699	chr19:13920556-13920668
chr19	13920759	13920934	chr19:13920749-13920922
chr19	13923329	13923475	chr19:13923327-13923455
chr19	13923554	13923632	chr19:13923547-13923606
chr19	13923694	13923844	chr19:13923694-13923811
chr19	13926519	13926628	chr19:13926516-13926590
chr19	13926674	13926745	chr19:13926666-13926725
chr19	13926824	13926900	chr19:13926820-13926872
chr19	13926989	13927087	chr19:13926977-13927077
chr19	13927179	13927303	chr19:13927174-13927265

chr19	13927894	13928044	chr19:13927892-13928030
chr19	13928134	13928205	chr19:13928123-13928188
chr19	13929389	13929468	chr19:13929378-13929442
chr19	13929539	13929689	chr19:13929533-13929660
chr19	13930079	13930191	chr19:13930077-13930154
chr19	13930244	13930320	chr19:13930241-13930289
chr19	13930384	13930918	chr19:13930374-13930879
chr19	13961599	13961742	chr19:13961599-13962864
chr19	13961744	13962839	chr19:13961599-13962864
chr19	13963004	13963074	chr19:13962993-13963039
chr19	13963129	13963308	chr19:13963121-13963275
chr19	13963544	13963759	chr19:13963537-13963746
chr19	13963859	13964046	chr19:13963857-13964007
chr19	13965449	13965563	chr19:13965448-13965546
chr19	13965639	13965808	chr19:13965625-13965777
chr19	13966434	13966576	chr19:13966420-13966530
chr19	13966634	13966787	chr19:13966632-13966751
chr19	13968564	13968713	chr19:13968564-13968680
chr19	13968774	13968918	chr19:13968774-13968894
chr19	13970004	13970211	chr19:13969993-13970175
chr19	13972744	13973149	chr19:13972742-13973127
chr19	13977994	13978118	chr19:13977991-13978086
chr19	13979449	13979565	chr19:13979446-13979542
chr19	13980584	13980722	chr19:13980572-13980689
chr19	13982129	13982238	chr19:13982120-13982228
chr19	13983199	13983308	chr19:13983186-13983270
chr19	13983319	13983400	chr19:13983312-13983357
chr19	13983499	13983634	chr19:13983485-13983595
chr19	13986239	13986458	chr19:13986229-13986429
chr19	13993529	13993882	chr19:13993524-13993895
chr19	13998129	13998208	chr19:13998117-13998317
chr19	14006114	14006205	chr19:14006102-14006285
chr19	14006214	14006311	chr19:14006102-14006285
chr19	14006749	14007072	chr19:14006749-14007039
chr19	14693449	14693524	chr19:14693450-14693494
chr19	14695019	14695165	chr19:14695009-14695133
chr19	14695579	14695687	chr19:14695565-14695661
chr19	14699204	14699309	chr19:14699198-14699281
chr19	14700294	14700396	chr19:14700205-14700355
chr19	14701609	14701921	chr19:14701583-14701897
chr19	14705064	14705215	chr19:14705053-14705170
chr19	14706699	14706807	chr19:14706685-14706773
chr19	14707924	14707998	chr19:14707916-14707965
chr19	14708329	14708636	chr19:14708327-14709220
chr19	14708659	14708872	chr19:14708327-14709220
chr19	14714739	14715032	chr19:14714737-14715470
chr19	14715054	14715141	chr19:14714737-14715470
chr19	14715269	14715510	chr19:14714737-14715470
chr19	14716124	14716208	chr19:14716111-14716238
chr19	14716999	14717120	chr19:14716993-14717089
chr19	14717664	14717770	chr19:14717656-14717733
chr19	14718229	14719912	chr19:14718227-14721908
chr19	14720199	14720610	chr19:14718227-14721908
chr19	14720614	14720887	chr19:14718227-14721908
chr19	14720899	14721284	chr19:14718227-14721908
chr19	14721299	14721689	chr19:14718227-14721908
chr19	14721869	14721947	chr19:14718227-14721908
chr19	14723999	14724212	chr19:14723989-14724189
chr19	14731184	14732239	chr19:14731174-14732242
chr19	16324854	16325027	chr19:16324816-16324998
chr19	16325219	16326064	chr19:16325215-16326032
chr19	16326869	16327267	chr19:16326855-16327874
chr19	16327279	16327501	chr19:16326855-16327874

chr19	16327504	16327886	chr19:16326855-16327874
chr19	17231888	17232659	chr19:17231882-17232626
chr19	17235498	17235767	chr19:17235498-17236065
chr19	17235788	17236104	chr19:17235498-17236065
chr19	17240683	17240789	chr19:17240670-17240765
chr19	17243533	17243738	chr19:17243519-17243719
chr19	17244243	17244350	chr19:17244232-17244504
chr19	17244358	17244536	chr19:17244232-17244504
chr19	17244948	17245958	chr19:17244942-17245940
chr19	17816923	17817098	chr19:17816909-17817059
chr19	17818038	17818145	chr19:17818024-17818224
chr19	17818153	17818268	chr19:17818024-17818224
chr19	17821328	17821439	chr19:17821316-17821416
chr19	18279760	18279827	chr19:18279759-18281622
chr19	18279850	18279912	chr19:18279759-18281622
chr19	18279975	18280046	chr19:18279759-18281622
chr19	18280070	18280970	chr19:18279759-18281622
chr19	18281005	18281640	chr19:18279759-18281622
chr19	19145566	19146415	chr19:19145566-19146405
chr19	19146561	19146679	chr19:19146554-19146648
chr19	19146746	19146885	chr19:19146741-19146875
chr19	19147041	19147219	chr19:19147035-19147183
chr19	19147711	19147858	chr19:19147697-19147832
chr19	19149221	19149463	chr19:19149216-19149429
chr19	19150686	19150796	chr19:19150681-19150764
chr19	19155776	19155994	chr19:19155776-19155976
chr19	19157081	19157186	chr19:19157024-19157142
chr19	19157221	19157296	chr19:19157215-19157283
chr19	19170216	19170324	chr19:19170204-19170289
chr19	19172251	19172461	chr19:19172251-19172451
chr19	19186041	19186123	chr19:19186033-19186098
chr19	19561716	19562142	chr19:19561706-19562117
chr19	19563516	19563621	chr19:19563508-19563615
chr19	19564776	19565100	chr19:19564774-19565089
chr19	19569461	19569608	chr19:19569448-19569584
chr19	19570111	19570322	chr19:19570098-19570299
chr19	19570591	19570870	chr19:19570585-19570833
chr19	19591346	19591561	chr19:19591334-19591534
chr19	19599296	19599406	chr19:19599291-19599365
chr19	19618511	19618935	chr19:19618510-19618916
chr19	19900916	19901032	chr19:19900912-19901091
chr19	19909351	19909430	chr19:19909296-19909520
chr19	19913906	19914005	chr19:19913773-19913973
chr19	19914771	19914843	chr19:19914733-19914822
chr19	19916611	19916688	chr19:19916559-19916655
chr19	19927121	19927195	chr19:19927110-19927313
chr19	19932246	19932405	chr19:19932007-19932997
chr19	19932406	19932483	chr19:19932007-19932997
chr19	19932491	19932695	chr19:19932007-19932997
chr19	19932741	19932813	chr19:19932007-19932997
chr19	19932851	19932952	chr19:19932007-19932997
chr19	19932961	19933031	chr19:19932007-19932997
chr19	19933236	19933375	chr19:19933181-19935575
chr19	19933416	19933490	chr19:19933181-19935575
chr19	19933631	19933731	chr19:19933181-19935575
chr19	19934001	19934078	chr19:19933181-19935575
chr19	19934241	19934316	chr19:19933181-19935575
chr19	19934636	19934829	chr19:19933181-19935575
chr19	19934836	19935107	chr19:19933181-19935575
chr19	19935156	19935589	chr19:19933181-19935575
chr19	20923246	20923349	chr19:20923221-20923416
chr19	20932251	20932436	chr19:20932214-20932414
chr19	20934971	20935090	chr19:20934948-20935047

chr19	20936621	20936693	chr19:20936591-20937361
chr19	20936876	20937011	chr19:20936591-20937361
chr19	20937296	20937388	chr19:20936591-20937361
chr19	20943051	20943688	chr19:20942800-20943977
chr19	20943696	20943769	chr19:20942800-20943977
chr19	20943791	20943993	chr19:20942800-20943977
chr19	20946311	20946411	chr19:20946299-20946389
chr19	20948751	20948939	chr19:20948743-20950697
chr19	20948941	20949019	chr19:20948743-20950697
chr19	20949021	20949128	chr19:20948743-20950697
chr19	20949376	20949444	chr19:20948743-20950697
chr19	20949541	20949614	chr19:20948743-20950697
chr19	20949701	20949768	chr19:20948743-20950697
chr19	20949896	20949968	chr19:20948743-20950697
chr19	20950216	20950351	chr19:20948743-20950697
chr19	20950356	20950701	chr19:20948743-20950697
chr19	21142026	21142106	chr19:21142023-21142186
chr19	21143556	21143659	chr19:21143550-21143643
chr19	21160401	21160509	chr19:21160343-21160543
chr19	21166406	21166479	chr19:21166334-21166461
chr19	21169846	21169920	chr19:21169802-21169882
chr19	21182651	21182823	chr19:21182622-21196053
chr19	21182836	21182910	chr19:21182622-21196053
chr19	21182916	21183019	chr19:21182622-21196053
chr19	21183266	21183338	chr19:21182622-21196053
chr19	21183906	21183977	chr19:21182622-21196053
chr19	21184001	21184073	chr19:21182622-21196053
chr19	21184351	21184800	chr19:21182622-21196053
chr19	21184801	21185449	chr19:21182622-21196053
chr19	21185751	21185852	chr19:21182622-21196053
chr19	21185891	21186004	chr19:21182622-21196053
chr19	21186326	21186702	chr19:21182622-21196053
chr19	21186706	21187226	chr19:21182622-21196053
chr19	21187736	21187982	chr19:21182622-21196053
chr19	21188271	21188368	chr19:21182622-21196053
chr19	21188391	21188622	chr19:21182622-21196053
chr19	21188631	21189224	chr19:21182622-21196053
chr19	21189511	21189858	chr19:21182622-21196053
chr19	21190206	21190490	chr19:21182622-21196053
chr19	21190501	21190664	chr19:21182622-21196053
chr19	21190961	21191180	chr19:21182622-21196053
chr19	21191461	21191814	chr19:21182622-21196053
chr19	21191866	21192171	chr19:21182622-21196053
chr19	21192436	21193060	chr19:21182622-21196053
chr19	21193066	21193234	chr19:21182622-21196053
chr19	21193591	21193810	chr19:21182622-21196053
chr19	21193811	21193887	chr19:21182622-21196053
chr19	21193896	21194032	chr19:21182622-21196053
chr19	21194046	21194139	chr19:21182622-21196053
chr19	21194221	21194469	chr19:21182622-21196053
chr19	21194731	21194805	chr19:21182622-21196053
chr19	21194811	21196069	chr19:21182622-21196053
chr19	21496681	21496759	chr19:21496681-21496722
chr19	21498271	21498348	chr19:21498198-21498349
chr19	21505566	21505750	chr19:21505563-21506080
chr19	21505761	21505895	chr19:21505563-21506080
chr19	21505956	21506097	chr19:21505563-21506080
chr19	21507526	21507598	chr19:21507519-21507675
chr19	21507611	21507716	chr19:21507519-21507675
chr19	21518841	21518918	chr19:21518574-21518988
chr19	21527846	21527981	chr19:21527842-21528042
chr19	21529091	21529160	chr19:21529083-21529279
chr19	21529206	21529305	chr19:21529083-21529279

chr19	21529681	21529764	chr19:21529657-21529784
chr19	21530601	21530678	chr19:21530588-21530684
chr19	21536311	21536768	chr19:21536279-21538078
chr19	21536826	21536900	chr19:21536279-21538078
chr19	21536906	21536982	chr19:21536279-21538078
chr19	21537011	21537079	chr19:21536279-21538078
chr19	21537171	21537318	chr19:21536279-21538078
chr19	21537321	21537393	chr19:21536279-21538078
chr19	21537511	21537581	chr19:21536279-21538078
chr19	21537751	21537824	chr19:21536279-21538078
chr19	21537906	21537976	chr19:21536279-21538078
chr19	21555366	21555443	chr19:21554909-21556270
chr19	21555616	21555685	chr19:21554909-21556270
chr19	21555756	21555859	chr19:21554909-21556270
chr19	21556051	21556284	chr19:21554909-21556270
chr19	21804951	21805134	chr19:21804948-21809807
chr19	21805636	21805976	chr19:21804948-21809807
chr19	21805981	21806150	chr19:21804948-21809807
chr19	21806451	21806965	chr19:21804948-21809807
chr19	21806966	21807664	chr19:21804948-21809807
chr19	21808871	21808974	chr19:21804948-21809807
chr19	21809256	21809325	chr19:21804948-21809807
chr19	21809356	21809794	chr19:21804948-21809807
chr19	21812016	21812096	chr19:21812012-21812056
chr19	21817891	21817968	chr19:21817887-21817986
chr19	21821801	21821872	chr19:21821761-21821961
chr19	21821906	21821980	chr19:21821761-21821961
chr19	21836046	21836189	chr19:21836035-21836208
chr19	21837916	21838070	chr19:21837853-21838034
chr19	21838881	21838993	chr19:21838838-21838947
chr19	21839811	21840112	chr19:21839808-21840086
chr19	21843156	21843365	chr19:21843144-21843326
chr19	21845391	21845518	chr19:21845391-21845506
chr19	21849916	21849996	chr19:21849913-21849978
chr19	21850951	21851238	chr19:21850937-21851360
chr19	21851931	21852142	chr19:21851904-21852125
chr19	22391028	22391228	chr19:22391018-22392981
chr19	22391233	22391675	chr19:22391018-22392981
chr19	22392113	22392185	chr19:22391018-22392981
chr19	22392473	22392593	chr19:22391018-22392981
chr19	22392638	22392737	chr19:22391018-22392981
chr19	22392788	22392995	chr19:22391018-22392981
chr19	22402383	22402583	chr19:22402381-22402564
chr19	22422278	22422355	chr19:22422194-22422357
chr19	22435673	22435817	chr19:22435661-22436006
chr19	22435838	22435943	chr19:22435661-22436006
chr19	22475823	22475957	chr19:22475813-22476013
chr19	22475968	22476037	chr19:22475813-22476013
chr19	22514368	22514649	chr19:22514363-22514630
chr19	22532338	22532495	chr19:22532338-22532485
chr19	22634323	22634403	chr19:22634323-22634474
chr19	22657523	22657632	chr19:22657523-22657723
chr19	22657648	22657715	chr19:22657523-22657723
chr19	22663808	22664016	chr19:22663799-22667670
chr19	22664183	22664309	chr19:22663799-22667670
chr19	22664598	22664673	chr19:22663799-22667670
chr19	22665158	22666031	chr19:22663799-22667670
chr19	22666058	22666164	chr19:22663799-22667670
chr19	22666473	22666747	chr19:22663799-22667670
chr19	22666748	22666952	chr19:22663799-22667670
chr19	22667238	22667366	chr19:22663799-22667670
chr19	22667373	22667591	chr19:22663799-22667670
chr19	23331586	23331802	chr19:23331583-23331783

chr19	23362616	23362726	chr19:23362575-23362725
chr19	30228289	30228584	chr19:30228289-30228673
chr19	30228589	30228687	chr19:30228289-30228673
chr19	30284074	30284145	chr19:30284071-30284141
chr19	30296154	30296267	chr19:30296154-30296243
chr19	30300429	30300678	chr19:30300425-30300669
chr19	30308734	30308947	chr19:30308723-30308923
chr19	30352379	30352513	chr19:30352367-30352484
chr19	30372424	30372564	chr19:30372411-30372556
chr19	30443569	30445323	chr19:30443560-30445732
chr19	30445334	30445748	chr19:30443560-30445732
chr19	30534859	30535040	chr19:30534846-30534999
chr19	30547949	30549524	chr19:30547942-30549514
chr19	30557169	30558037	chr19:30557156-30558059
chr19	30710764	30711141	chr19:30710756-30713538
chr19	30711164	30711753	chr19:30710756-30713538
chr19	30711769	30711901	chr19:30710756-30713538
chr19	30711919	30712434	chr19:30710756-30713538
chr19	30712439	30712924	chr19:30710756-30713538
chr19	30713029	30713342	chr19:30710756-30713538
chr19	30713354	30713554	chr19:30710756-30713538
chr19	31274954	31275432	chr19:31274944-31279752
chr19	31275439	31275811	chr19:31274944-31279752
chr19	31275814	31276095	chr19:31274944-31279752
chr19	31276124	31279317	chr19:31274944-31279752
chr19	31279349	31279768	chr19:31274944-31279752
chr19	31290724	31290954	chr19:31290720-31290920
chr19	31305499	31305782	chr19:31305487-31305763
chr19	31308344	31308744	chr19:31308344-31308709
chr19	31349249	31349479	chr19:31349179-31349547
chr19	31349509	31349582	chr19:31349179-31349547
chr19	33299944	33300291	chr19:33299933-33302564
chr19	33300304	33301839	chr19:33299933-33302564
chr19	33301864	33302102	chr19:33299933-33302564
chr19	33302119	33302597	chr19:33299933-33302564
chr19	33373339	33373927	chr19:33373329-33373895
chr19	33374319	33374641	chr19:33374311-33374613
chr19	33376799	33377011	chr19:33376796-33376996
chr19	33379149	33381070	chr19:33379143-33382686
chr19	33381089	33382621	chr19:33379143-33382686
chr19	34677648	34678132	chr19:34677638-34678103
chr19	34678748	34678848	chr19:34678736-34678813
chr19	34679828	34679980	chr19:34679827-34679959
chr19	34682793	34682909	chr19:34682776-34682897
chr19	34683153	34683270	chr19:34683151-34683238
chr19	34683998	34684170	chr19:34683991-34686397
chr19	34684217	34684707	chr19:34683991-34686397
chr19	34684717	34684796	chr19:34683991-34686397
chr19	34684827	34685036	chr19:34683991-34686397
chr19	34685057	34685373	chr19:34683991-34686397
chr19	34685382	34685456	chr19:34683991-34686397
chr19	34685482	34686423	chr19:34683991-34686397
chr19	35268977	35269043	chr19:35268977-35269163
chr19	35269052	35269183	chr19:35268977-35269163
chr19	35269447	35269730	chr19:35269445-35269699
chr19	35269802	35270128	chr19:35269802-35270097
chr19	35270262	35270623	chr19:35270262-35270597
chr19	35270722	35270825	chr19:35270717-35270805
chr19	35271007	35271152	chr19:35271000-35271141
chr19	35275332	35275432	chr19:35275081-35275425
chr19	35275782	35275942	chr19:35275782-35275900
chr19	35278702	35279711	chr19:35278697-35279815
chr19	35279762	35279835	chr19:35278697-35279815

chr19	35641747	35642740	chr19:35641744-35642698
chr19	35642967	35643076	chr19:35642964-35643045
chr19	35643282	35643778	chr19:35643273-35643753
chr19	35644247	35644909	chr19:35644234-35644871
chr19	36604817	36605002	chr19:36604816-36604975
chr19	36605437	36605760	chr19:36605426-36605730
chr19	36607562	36607661	chr19:36607551-36607622
chr19	36608422	36609938	chr19:36608419-36610053
chr19	36609962	36610071	chr19:36608419-36610053
chr19	36610507	36610747	chr19:36610497-36610742
chr19	36620297	36620415	chr19:36620296-36620496
chr19	36620422	36620532	chr19:36620296-36620496
chr19	36626137	36627190	chr19:36626129-36634113
chr19	36627197	36627686	chr19:36626129-36634113
chr19	36627702	36628955	chr19:36626129-36634113
chr19	36629237	36629548	chr19:36626129-36634113
chr19	36629557	36629615	chr19:36626129-36634113
chr19	36629942	36630352	chr19:36626129-36634113
chr19	36630622	36630793	chr19:36626129-36634113
chr19	36631062	36631143	chr19:36626129-36634113
chr19	36631147	36631236	chr19:36626129-36634113
chr19	36631702	36632192	chr19:36626129-36634113
chr19	36632487	36633048	chr19:36626129-36634113
chr19	36633327	36633554	chr19:36626129-36634113
chr19	36633562	36633664	chr19:36626129-36634113
chr19	36633667	36633837	chr19:36626129-36634113
chr19	36633852	36634089	chr19:36626129-36634113
chr19	38211017	38211686	chr19:38211005-38212133
chr19	38211712	38211854	chr19:38211005-38212133
chr19	38211857	38212168	chr19:38211005-38212133
chr19	38212287	38212421	chr19:38212279-38212391
chr19	38213657	38213786	chr19:38213643-38213756
chr19	38216142	38216293	chr19:38216139-38216259
chr19	38216357	38216426	chr19:38216352-38216403
chr19	38217462	38217610	chr19:38217459-38217591
chr19	38217797	38217910	chr19:38217797-38217876
chr19	38218572	38218693	chr19:38218572-38218662
chr19	38218932	38219071	chr19:38218930-38219058
chr19	38220937	38221162	chr19:38220937-38221137
chr19	38222357	38222493	chr19:38222356-38222464
chr19	38222557	38222731	chr19:38222547-38222708
chr19	38222847	38223231	chr19:38222834-38223298
chr19	38223257	38223334	chr19:38222834-38223298
chr19	38223767	38223984	chr19:38223755-38223961
chr19	38224127	38224269	chr19:38224113-38224250
chr19	38227957	38228066	chr19:38227945-38228042
chr19	38228652	38228723	chr19:38228661-38228693
chr19	38229562	38229743	chr19:38229558-38229714
chr19	39706609	39706711	chr19:39706596-39706673
chr19	39707189	39707403	chr19:39707177-39707388
chr19	39708504	39708702	chr19:39708495-39708695
chr19	42086138	42086942	chr19:42086109-42092270
chr19	42086948	42087074	chr19:42086109-42092270
chr19	42087083	42087144	chr19:42086109-42092270
chr19	42087163	42087598	chr19:42086109-42092270
chr19	42087608	42088157	chr19:42086109-42092270
chr19	42088168	42088499	chr19:42086109-42092270
chr19	42088513	42089221	chr19:42086109-42092270
chr19	42089273	42089696	chr19:42086109-42092270
chr19	42089778	42090193	chr19:42086109-42092270
chr19	42090208	42090974	chr19:42086109-42092270
chr19	42091003	42091098	chr19:42086109-42092270
chr19	42091103	42092295	chr19:42086109-42092270

chr19	42093478	42093924	chr19:42093466-42093895
chr19	42095293	42095483	chr19:42095285-42095462
chr19	42095558	42095732	chr19:42095544-42095693
chr19	42095838	42095944	chr19:42095787-42095929
chr19	42096083	42096264	chr19:42096081-42096243
chr19	42099538	42099641	chr19:42099526-42099618
chr19	42099723	42099846	chr19:42099714-42099821
chr19	42110558	42110631	chr19:42110546-42110605
chr19	42110713	42110789	chr19:42110700-42110756
chr19	42116708	42116850	chr19:42116708-42117005
chr19	42116893	42117051	chr19:42116708-42117005
chr19	42117253	42117470	chr19:42117249-42117432
chr19	42122138	42122206	chr19:42122125-42122182
chr19	42122338	42122416	chr19:42122343-42122378
chr19	42122513	42122590	chr19:42122510-42122576
chr19	42123158	42123271	chr19:42123155-42123229
chr19	42132393	42132500	chr19:42132383-42132478
chr19	42160333	42160409	chr19:42160331-42160392
chr19	42174213	42174436	chr19:42174200-42174400
chr19	42175973	42176052	chr19:42175962-42176025
chr19	42177153	42177357	chr19:42177152-42177325
chr19	42196383	42196595	chr19:42196382-42196585
chr19	42247573	42247676	chr19:42247571-42249738
chr19	42247678	42248999	chr19:42247571-42249738
chr19	42249003	42249767	chr19:42247571-42249738
chr19	42249828	42249979	chr19:42249826-42249942
chr19	42250338	42250475	chr19:42250330-42250565
chr19	42250488	42250591	chr19:42250330-42250565
chr19	42251388	42251456	chr19:42251388-42251440
chr19	42252923	42253124	chr19:42252909-42253109
chr19	42254543	42254642	chr19:42254536-42254635
chr19	42254978	42255184	chr19:42254977-42255157
chr19	42268548	42268622	chr19:42268536-42268594
chr19	42269228	42269390	chr19:42269223-42269381
chr19	42271778	42271966	chr19:42271773-42274577
chr19	42271968	42274591	chr19:42271773-42274577
chr19	42278498	42278715	chr19:42278484-42278684
chr19	42280003	42280233	chr19:42279991-42280203
chr19	42284023	42284800	chr19:42284019-42284771
chr19	42286778	42286954	chr19:42286770-42286920
chr19	42287043	42287216	chr19:42287005-42287240
chr19	42287333	42287426	chr19:42287319-42287449
chr19	42287548	42287627	chr19:42287544-42287727
chr19	42287628	42287699	chr19:42287544-42287727
chr19	42287868	42288009	chr19:42287809-42287975
chr19	42288893	42289098	chr19:42288887-42289090
chr19	42289233	42289452	chr19:42289180-42289406
chr19	42289848	42289930	chr19:42289847-42289951
chr19	42290233	42291272	chr19:42290232-42291466
chr19	42291338	42291476	chr19:42290232-42291466
chr19	42291563	42291782	chr19:42291557-42291745
chr19	42292088	42292246	chr19:42292085-42292207
chr19	42292303	42292484	chr19:42292299-42292466
chr19	42292568	42292896	chr19:42292565-42292859
chr19	42292958	42293317	chr19:42292955-42293281
chr19	42293603	42293843	chr19:42293591-42293836
chr19	42293938	42294120	chr19:42293934-42294089
chr19	42294173	42294329	chr19:42294172-42294304
chr19	42294588	42294759	chr19:42294574-42294735
chr19	42294823	42295526	chr19:42294823-42295797
chr19	42295573	42295826	chr19:42294823-42295797
chr19	43912628	43915203	chr19:43912628-43915200
chr19	43917978	43918172	chr19:43917966-43918166

chr19	43918873	43918995	chr19:43918869-43918962
chr19	43919608	43919777	chr19:43919572-43919739
chr19	43922173	43922278	chr19:43922170-43922287
chr19	43924238	43924348	chr19:43924237-43924312
chr19	43924403	43924590	chr19:43924401-43924561
chr19	43925338	43925481	chr19:43925324-43925458
chr19	43932413	43932730	chr19:43932402-43932690
chr19	43934433	43934713	chr19:43934425-43934694
chr19	43934923	43935313	chr19:43934923-43935278
chr19	43967863	43968119	chr19:43967861-43968084
chr19	43984178	43984379	chr19:43984169-43984348
chr19	43988468	43988566	chr19:43988458-43988558
chr19	43990028	43990130	chr19:43990023-43990112
chr19	43991638	43991721	chr19:43991547-43991674
chr19	43991843	43991914	chr19:43991841-43991934
chr19	43993968	43994196	chr19:43993965-43994165
chr19	43996103	43996510	chr19:43996092-43998325
chr19	43996553	43996700	chr19:43996092-43998325
chr19	43996708	43996965	chr19:43996092-43998325
chr19	43996978	43997211	chr19:43996092-43998325
chr19	43997213	43997481	chr19:43996092-43998325
chr19	43997493	43997571	chr19:43996092-43998325
chr19	43997578	43997760	chr19:43996092-43998325
chr19	43997783	43997878	chr19:43996092-43998325
chr19	43997913	43998010	chr19:43996092-43998325
chr19	43998013	43998118	chr19:43996092-43998325
chr19	43998218	43998358	chr19:43996092-43998325
chr19	44002953	44003123	chr19:44002947-44003107
chr19	44006703	44007123	chr19:44006699-44007093
chr19	44008808	44009021	chr19:44008789-44008991
chr19	44009148	44009350	chr19:44009083-44009322
chr19	44010273	44010712	chr19:44010268-44013926
chr19	44010713	44011198	chr19:44010268-44013926
chr19	44011298	44011395	chr19:44010268-44013926
chr19	44011428	44011910	chr19:44010268-44013926
chr19	44011953	44012060	chr19:44010268-44013926
chr19	44012083	44013949	chr19:44010268-44013926
chr19	44072148	44072319	chr19:44072143-44072291
chr19	44076333	44076441	chr19:44076321-44076404
chr19	44077813	44078039	chr19:44077812-44078012
chr19	44081098	44081181	chr19:44081014-44081141
chr19	44082013	44082086	chr19:44082012-44082105
chr19	44085723	44087300	chr19:44085713-44089613
chr19	44087378	44087592	chr19:44085713-44089613
chr19	44088203	44088352	chr19:44085713-44089613
chr19	44088408	44088518	chr19:44085713-44089613
chr19	44088558	44088826	chr19:44085713-44089613
chr19	44089268	44089649	chr19:44085713-44089613
chr19	44094348	44094545	chr19:44094338-44094530
chr19	44094853	44094957	chr19:44094784-44095158
chr19	44094973	44095178	chr19:44094784-44095158
chr19	44096058	44096167	chr19:44096045-44096431
chr19	44096183	44096465	chr19:44096045-44096431
chr19	44097708	44097917	chr19:44097708-44097888
chr19	44100808	44100889	chr19:44100800-44100927
chr19	44101133	44101253	chr19:44101132-44101225
chr19	44102968	44103116	chr19:44102968-44103168
chr19	44103128	44103198	chr19:44102968-44103168
chr19	44106398	44106793	chr19:44106395-44109886
chr19	44106928	44107005	chr19:44106395-44109886
chr19	44107023	44107180	chr19:44106395-44109886
chr19	44107183	44107354	chr19:44106395-44109886
chr19	44107363	44107680	chr19:44106395-44109886

chr19	44107698	44107867	chr19:44106395-44109886
chr19	44107868	44108921	chr19:44106395-44109886
chr19	44108923	44109319	chr19:44106395-44109886
chr19	44109328	44109927	chr19:44106395-44109886
chr19	45001433	45001713	chr19:45001429-45001685
chr19	45002958	45003042	chr19:45002948-45002996
chr19	45008458	45008751	chr19:45008457-45008721
chr19	45011938	45012296	chr19:45011935-45012276
chr19	45022053	45022241	chr19:45022052-45022210
chr19	45025328	45025445	chr19:45025328-45025420
chr19	45025608	45025763	chr19:45025605-45025737
chr19	45028893	45028998	chr19:45028887-45028992
chr19	45032538	45032786	chr19:45032533-45032749
chr19	45034243	45034315	chr19:45034243-45034312
chr19	45034453	45034560	chr19:45034450-45034528
chr19	45037408	45037948	chr19:45037404-45038198
chr19	45037968	45038211	chr19:45037404-45038198
chr19	45468613	45468729	chr19:45468612-45468712
chr19	45470628	45470813	chr19:45470628-45470778
chr19	45470858	45470969	chr19:45470849-45470949
chr19	45471203	45471313	chr19:45471201-45471301
chr19	45472553	45472738	chr19:45472550-45472706
chr19	45472848	45473033	chr19:45472846-45472997
chr19	45764788	45767174	chr19:45764784-45767155
chr19	45768053	45768706	chr19:45768041-45769226
chr19	45768788	45768858	chr19:45768041-45769226
chr19	45768868	45769241	chr19:45768041-45769226
chr19	45863993	45864210	chr19:45863988-45864191
chr19	45864268	45864555	chr19:45864259-45864525
chr19	45865433	45865648	chr19:45865430-45865630
chr19	45872083	45873829	chr19:45872074-45873797
chr19	45890033	45890864	chr19:45890019-45891323
chr19	45890883	45891087	chr19:45890019-45891323
chr19	45891088	45891371	chr19:45890019-45891323
chr19	45901278	45901843	chr19:45901274-45901825
chr19	45902563	45902633	chr19:45902569-45902604
chr19	46297058	46297135	chr19:46297045-46297102
chr19	46298398	46298541	chr19:46298389-46298514
chr19	46300943	46301151	chr19:46300929-46301129
chr19	46303608	46303716	chr19:46303598-46303687
chr19	46303848	46304116	chr19:46303848-46304088
chr19	46305043	46305149	chr19:46305041-46305135
chr19	46305258	46305436	chr19:46305244-46305390
chr19	46308233	46308343	chr19:46308220-46308305
chr19	46308548	46308625	chr19:46308551-46308586
chr19	46308673	46308803	chr19:46308662-46308775
chr19	46309153	46309368	chr19:46309150-46309359
chr19	46310548	46310728	chr19:46310541-46310691
chr19	46312173	46312284	chr19:46312160-46312267
chr19	46312513	46312686	chr19:46312505-46312653
chr19	46320443	46320590	chr19:46320442-46320561
chr19	46321788	46321990	chr19:46321775-46321966
chr19	46325538	46325657	chr19:46325534-46325639
chr19	46329218	46329493	chr19:46329206-46329478
chr19	46331168	46331310	chr19:46331155-46331273
chr19	46334908	46335017	chr19:46334904-46334986
chr19	46338478	46338578	chr19:46338226-46338538
chr19	46339533	46340056	chr19:46339524-46343433
chr19	46340058	46340450	chr19:46339524-46343433
chr19	46340798	46341184	chr19:46339524-46343433
chr19	46341473	46341644	chr19:46339524-46343433
chr19	46341933	46342381	chr19:46339524-46343433
chr19	46342528	46343477	chr19:46339524-46343433

chr19	46918685	46920099	chr19:46918675-46922356
chr19	46920120	46922388	chr19:46918675-46922356
chr19	46937275	46937445	chr19:46937263-46937408
chr19	46987995	46988110	chr19:46987988-46988066
chr19	46989545	46989697	chr19:46989543-46989675
chr19	46991025	46991246	chr19:46991023-46991223
chr19	46997505	46997792	chr19:46997496-46997750
chr19	46999095	46999172	chr19:46999084-46999144
chr19	46999310	46999836	chr19:46999303-46999815
chr19	47000340	47001414	chr19:47000330-47005077
chr19	47001430	47003926	chr19:47000330-47005077
chr19	47003940	47004497	chr19:47000330-47005077
chr19	47004510	47004928	chr19:47000330-47005077
chr19	47004955	47005092	chr19:47000330-47005077
chr19	47019840	47020024	chr19:47019819-47019997
chr19	47020895	47021199	chr19:47020885-47021169
chr19	47021625	47021876	chr19:47021611-47021847
chr19	47027710	47027886	chr19:47027655-47027855
chr19	47032280	47032394	chr19:47032277-47032351
chr19	47032650	47032755	chr19:47032642-47032732
chr19	47035327	47035408	chr19:47035328-47035358
chr19	47035867	47036082	chr19:47035859-47036129
chr19	47036092	47036165	chr19:47035859-47036129
chr19	47039047	47039184	chr19:47039035-47039151
chr19	47039407	47039588	chr19:47039403-47039564
chr19	47040452	47040568	chr19:47040443-47040550
chr19	47040977	47041166	chr19:47040977-47041125
chr19	47042812	47042923	chr19:47042809-47042904
chr19	47045202	47045793	chr19:47045190-47045756
chr19	47403407	47403592	chr19:47403403-47403553
chr19	47406382	47406541	chr19:47406376-47406526
chr19	47406887	47407010	chr19:47406887-47406971
chr19	47819782	47820039	chr19:47819778-47820004
chr19	47821842	47822028	chr19:47821838-47822010
chr19	47834422	47834563	chr19:47834408-47834543
chr19	47836252	47836429	chr19:47836242-47836394
chr19	47839322	47840401	chr19:47839319-47843330
chr19	47840997	47841563	chr19:47839319-47843330
chr19	47841582	47842313	chr19:47839319-47843330
chr19	47842597	47843356	chr19:47839319-47843330
chr19	48630867	48631088	chr19:48630867-48631052
chr19	48633217	48633664	chr19:48633085-48633655
chr19	48634417	48634658	chr19:48634406-48634636
chr19	48635587	48636014	chr19:48635579-48635990
chr19	48636857	48637456	chr19:48636855-48637438
chr19	48880977	48881155	chr19:48880964-48881126
chr19	48882007	48882214	chr19:48881998-48882203
chr19	48883767	48883872	chr19:48883753-48883852
chr19	48883942	48884084	chr19:48883931-48884046
chr19	48885452	48885593	chr19:48885447-48885560
chr19	48886452	48886666	chr19:48886442-48886642
chr19	48887962	48888279	chr19:48887949-48888261
chr19	48889522	48889634	chr19:48889509-48889631
chr19	48895007	48895175	chr19:48894997-48895162
chr19	48895367	48895512	chr19:48895365-48895503
chr19	48896432	48896578	chr19:48896429-48896556
chr19	48897357	48897494	chr19:48897344-48897459
chr19	48897832	48897921	chr19:48897836-48897869
chr19	48898282	48898390	chr19:48898278-48898382
chr19	48898492	48898666	chr19:48898486-48898733
chr19	48898677	48898747	chr19:48898486-48898733
chr19	49341023	49341476	chr19:49341010-49341437
chr19	49342448	49342621	chr19:49342437-49342590

chr19	49344778	49344996	chr19:49344777-49344977
chr19	49347193	49347453	chr19:49347189-49347424
chr19	49348703	49348876	chr19:49348702-49348845
chr19	49348983	49349078	chr19:49348956-49349059
chr19	49351303	49351378	chr19:49351300-49351365
chr19	49355153	49355225	chr19:49355147-49355206
chr19	49355313	49355447	chr19:49355302-49355419
chr19	49357258	49357328	chr19:49357251-49357314
chr19	49359438	49359512	chr19:49359434-49359499
chr19	49359843	49360160	chr19:49359843-49360438
chr19	49361398	49361513	chr19:49361395-49361495
chr19	49362333	49362469	chr19:49362328-49362457
chr19	49659568	49659860	chr19:49659568-49659833
chr19	49660713	49660860	chr19:49660712-49660844
chr19	49661463	49661564	chr19:49661449-49662329
chr19	49661878	49662371	chr19:49661449-49662329
chr19	49662433	49662638	chr19:49662424-49662617
chr19	49663198	49663294	chr19:49663187-49663258
chr19	49663348	49663526	chr19:49663342-49663514
chr19	49664443	49665000	chr19:49664442-49664991
chr19	49930228	49930460	chr19:49930218-49930432
chr19	49930733	49931252	chr19:49930731-49931229
chr19	49932428	49933192	chr19:49932421-49933935
chr19	49933313	49933974	chr19:49932421-49933935
chr19	50314850	50315021	chr19:50314840-50314990
chr19	50315945	50316128	chr19:50315941-50316091
chr19	50320230	50320328	chr19:50320222-50320322
chr19	50326230	50326444	chr19:50326230-50326430
chr19	50329655	50329830	chr19:50329652-50329795
chr19	50338015	50338106	chr19:50338001-50338076
chr19	50352495	50352574	chr19:50352490-50352606
chr19	50357460	50357672	chr19:50357456-50357656
chr19	50371365	50371465	chr19:50371354-50371440
chr19	50376240	50376846	chr19:50376234-50376826
chr19	50377255	50377351	chr19:50377244-50377328
chr19	50377435	50377682	chr19:50377433-50377648
chr19	50377745	50377899	chr19:50377732-50377870
chr19	50378150	50378475	chr19:50378148-50378439
chr19	50378525	50378817	chr19:50378521-50378796
chr19	50379015	50379224	chr19:50379001-50379181
chr19	50379790	50379890	chr19:50379779-50379879
chr19	50381970	50382184	chr19:50381965-50382174
chr19	50382465	50382980	chr19:50382455-50382982
chr19	50418940	50419013	chr19:50418937-50418985
chr19	50419900	50420002	chr19:50419892-50419973
chr19	50422475	50422585	chr19:50422472-50422545
chr19	50422830	50423071	chr19:50422822-50423037
chr19	50423615	50423780	chr19:50423604-50423755
chr19	50428040	50429427	chr19:50428037-50431313
chr19	50430010	50430455	chr19:50428037-50431313
chr19	50430745	50431179	chr19:50428037-50431313
chr19	51571300	51571514	chr19:51571297-51571475
chr19	51573160	51574166	chr19:51573149-51574210
chr19	51574170	51574243	chr19:51573149-51574210
chr19	51581395	51581575	chr19:51581390-51581546
chr19	51581795	51581899	chr19:51581786-51581882
chr19	51583455	51583657	chr19:51583443-51583643
chr19	51586630	51588905	chr19:51586626-51592508
chr19	51588965	51589040	chr19:51586626-51592508
chr19	51589115	51589214	chr19:51586626-51592508
chr19	51589280	51589700	chr19:51586626-51592508
chr19	51589720	51589854	chr19:51586626-51592508
chr19	51589900	51591611	chr19:51586626-51592508

chr19	51591625	51591765	chr19:51586626-51592508
chr19	51592025	51592522	chr19:51586626-51592508
chr19	51964355	51965296	chr19:51964342-51966214
chr19	51965340	51965482	chr19:51964342-51966214
chr19	51965490	51965568	chr19:51964342-51966214
chr19	51965570	51966231	chr19:51964342-51966214
chr19	51968585	51968692	chr19:51968577-51968673
chr19	51969090	51969166	chr19:51969004-51969145
chr19	51974350	51974563	chr19:51974345-51974531
chr19	51974685	51974869	chr19:51974677-51974853
chr19	51981975	51982047	chr19:51981924-51982031
chr19	51986510	51986690	chr19:51986509-51986670
chr19	51986770	51986874	chr19:51986769-51986856
chr19	52838015	52838193	chr19:52838007-52842151
chr19	52838195	52838711	chr19:52838007-52842151
chr19	52838715	52838956	chr19:52838007-52842151
chr19	52839380	52839497	chr19:52838007-52842151
chr19	52839505	52839645	chr19:52838007-52842151
chr19	52839745	52839957	chr19:52838007-52842151
chr19	52839960	52840038	chr19:52838007-52842151
chr19	52840040	52840558	chr19:52838007-52842151
chr19	52840570	52840646	chr19:52838007-52842151
chr19	52840650	52840792	chr19:52838007-52842151
chr19	52841155	52841304	chr19:52838007-52842151
chr19	52841400	52841478	chr19:52838007-52842151
chr19	52841505	52841853	chr19:52838007-52842151
chr19	52841880	52842183	chr19:52838007-52842151
chr19	52846560	52846634	chr19:52846557-52846611
chr19	52849220	52849457	chr19:52849086-52849630
chr19	52849460	52849603	chr19:52849086-52849630
chr19	52853875	52853962	chr19:52853871-52854126
chr19	52854055	52854157	chr19:52853871-52854126
chr19	52854285	52854356	chr19:52854257-52854345
chr19	52855825	52855900	chr19:52855823-52856023
chr19	52855960	52856055	chr19:52855823-52856023
chr19	52857580	52857687	chr19:52857571-52857649
chr19	54137740	54138029	chr19:54137727-54137993
chr19	54140395	54140614	chr19:54140392-54140592
chr19	54141395	54142423	chr19:54141395-54143003
chr19	54142445	54143039	chr19:54141395-54143003
chr19	54143120	54143199	chr19:54143118-54143186
chr19	54143445	54143552	chr19:54143441-54143516
chr19	54143670	54143774	chr19:54143659-54143749
chr19	54144005	54144154	chr19:54144004-54144134
chr19	54144245	54144359	chr19:54144236-54144332
chr19	54145555	54145830	chr19:54145541-54145817
chr19	54145920	54146062	chr19:54145909-54146043
chr19	54146600	54146676	chr19:54146600-54146657
chr19	54148150	54148573	chr19:54148147-54148535
chr19	54148620	54148769	chr19:54148619-54148743
chr19	54149560	54149777	chr19:54149559-54149758
chr19	54152235	54152340	chr19:54152225-54152325
chr19	54152440	54152646	chr19:54152427-54152626
chr19	54152880	54153016	chr19:54152866-54152999
chr19	54153185	54153428	chr19:54153180-54153393
chr19	54153500	54153857	chr19:54153496-54153840
chr19	54153950	54154472	chr19:54153943-54154455
chr19	55401620	55401800	chr19:55401612-55401762
chr19	55404290	55404406	chr19:55404287-55404387
chr19	55635027	55635729	chr19:55635015-55635712
chr19	55640907	55641206	chr19:55640895-55641183
chr19	55641772	55643144	chr19:55641762-55643396
chr19	55643147	55643419	chr19:55641762-55643396

chr19	56567522	56568083	chr19:56567510-56568038
chr19	56568757	56568913	chr19:56568757-56568883
chr19	56570177	56570387	chr19:56570174-56570371
chr19	56574402	56574547	chr19:56574393-56574520
chr19	56574647	56574753	chr19:56574637-56574733
chr19	56576712	56577180	chr19:56576712-56582895
chr19	56577197	56577349	chr19:56576712-56582895
chr19	56577352	56577433	chr19:56576712-56582895
chr19	56577447	56577849	chr19:56576712-56582895
chr19	56577947	56578026	chr19:56576712-56582895
chr19	56578032	56578761	chr19:56576712-56582895
chr19	56578767	56579151	chr19:56576712-56582895
chr19	56579387	56579733	chr19:56576712-56582895
chr19	56579747	56580507	chr19:56576712-56582895
chr19	56580532	56580602	chr19:56576712-56582895
chr19	56580607	56581268	chr19:56576712-56582895
chr19	56581282	56581515	chr19:56576712-56582895
chr19	56581522	56582690	chr19:56576712-56582895
chr19	56582697	56582814	chr19:56576712-56582895
chr19	56584027	56584140	chr19:56584019-56584112
chr19	56586637	56586716	chr19:56586634-56588911
chr19	56587117	56587205	chr19:56586634-56588911
chr19	56587482	56588388	chr19:56586634-56588911
chr19	56588422	56588560	chr19:56586634-56588911
chr19	56588642	56588801	chr19:56586634-56588911
chr19	56810096	56810826	chr19:56810082-56817579
chr19	56810841	56811785	chr19:56810082-56817579
chr19	56811796	56812103	chr19:56810082-56817579
chr19	56812131	56812413	chr19:56810082-56817579
chr19	56812426	56812815	chr19:56810082-56817579
chr19	56812836	56813210	chr19:56810082-56817579
chr19	56813216	56814331	chr19:56810082-56817579
chr19	56814366	56817589	chr19:56810082-56817579
chr19	56817746	56817868	chr19:56817745-56817838
chr19	56818611	56818714	chr19:56818599-56818702
chr19	56821661	56821766	chr19:56821650-56821754
chr19	56823596	56823705	chr19:56823589-56823679
chr19	56824261	56825041	chr19:56824261-56825006
chr19	56826401	56826502	chr19:56826387-56826463
chr19	56831806	56831992	chr19:56831794-56831994
chr19	56833301	56833398	chr19:56833289-56833420
chr19	56833651	56833801	chr19:56833642-56833763
chr19	56836031	56836138	chr19:56836017-56836104
chr19	57351306	57351737	chr19:57351306-57351697
chr19	57352661	57352765	chr19:57352654-57352735
chr19	57353726	57353885	chr19:57353724-57353851
chr19	57355271	57355449	chr19:57355270-57355411
chr19	57356041	57357026	chr19:57356029-57359898
chr19	57357046	57357293	chr19:57356029-57359898
chr19	57357301	57357443	chr19:57356029-57359898
chr19	57357456	57357538	chr19:57356029-57359898
chr19	57357541	57357618	chr19:57356029-57359898
chr19	57357636	57359939	chr19:57356029-57359898
chr19	57614232	57614520	chr19:57614232-57614503
chr19	57616137	57616337	chr19:57616123-57616323
chr19	57618802	57618905	chr19:57618791-57618877
chr19	57619417	57619658	chr19:57619411-57619628
chr19	57620172	57620666	chr19:57620159-57624723
chr19	57620727	57620810	chr19:57620159-57624723
chr19	57620812	57621297	chr19:57620159-57624723
chr19	57621312	57622967	chr19:57620159-57624723
chr19	57622997	57623277	chr19:57620159-57624723
chr19	57623282	57623556	chr19:57620159-57624723

chr19	57623562	57624316	chr19:57620159-57624723
chr19	57624427	57624560	chr19:57620159-57624723
chr19	57624562	57624742	chr19:57620159-57624723
chr19	57668937	57669044	chr19:57668934-57669203
chr19	57670242	57670595	chr19:57670241-57670567
chr19	57671707	57671926	chr19:57671707-57671907
chr19	57676052	57676578	chr19:57676040-57676541
chr19	57677917	57678106	chr19:57677913-57678079
chr19	57678172	57679113	chr19:57678165-57679152
chr19	57697372	57697577	chr19:57697366-57701145
chr19	57697582	57698060	chr19:57697366-57701145
chr19	57698067	57698686	chr19:57697366-57701145
chr19	57698697	57698768	chr19:57697366-57701145
chr19	57699377	57699477	chr19:57697366-57701145
chr19	57699597	57699697	chr19:57697366-57701145
chr19	57700027	57701150	chr19:57697366-57701145
chr19	57701242	57701945	chr19:57701237-57702788
chr19	57701972	57702106	chr19:57701237-57702788
chr19	57702127	57702201	chr19:57701237-57702788
chr19	57702212	57702811	chr19:57701237-57702788
chr19	57704877	57704979	chr19:57704852-57704979
chr19	57707912	57708142	chr19:57707909-57708109
chr19	57708942	57709226	chr19:57708938-57709194
chr19	57940857	57941071	chr19:57940832-57942647
chr19	57941072	57941248	chr19:57940832-57942647
chr19	57941262	57941407	chr19:57940832-57942647
chr19	57941422	57941570	chr19:57940832-57942647
chr19	57941577	57941819	chr19:57940832-57942647
chr19	57941852	57942661	chr19:57940832-57942647
chr19	57943942	57944092	chr19:57943933-57944060
chr19	57945807	57946032	chr19:57945804-57946004
chr19	57947452	57947693	chr19:57947441-57947675
chr19	58059242	58059321	chr19:58059238-58059310
chr19	58059737	58060406	chr19:58059733-58060398
chr19	58061582	58061736	chr19:58061579-58061706
chr19	58063447	58063560	chr19:58063445-58063541
chr19	58066717	58067716	chr19:58066704-58069755
chr19	58067722	58069600	chr19:58066704-58069755
chr19	58076817	58077039	chr19:58076804-58077004
chr19	58081862	58082048	chr19:58081692-58082008
chr19	58183032	58184021	chr19:58183028-58183442;chr19:58183453-58183998
chr19	58185697	58185800	chr19:58185696-58185838
chr19	58186952	58187061	chr19:58186946-58187042
chr19	58202237	58202476	chr19:58202226-58202468
chr19	58204102	58204322	chr19:58204099-58204299
chr19	58206732	58207045	chr19:58206719-58207202
chr19	58207072	58207239	chr19:58206719-58207202
chr19	58209962	58210113	chr19:58209960-58210073
chr19	58211567	58211708	chr19:58211559-58211686
chr19	58212172	58213607	chr19:58212160-58213562
chr19	58288767	58288986	chr19:58288760-58288960
chr19	58294107	58296408	chr19:58294097-58302805
chr19	58296682	58296944	chr19:58294097-58302805
chr19	58297252	58297419	chr19:58294097-58302805
chr19	58297677	58297992	chr19:58294097-58302805
chr19	58298612	58298822	chr19:58294097-58302805
chr19	58299407	58299482	chr19:58294097-58302805
chr19	58299772	58301870	chr19:58294097-58302805
chr19	58301897	58302074	chr19:58294097-58302805
chr19	58302082	58302280	chr19:58294097-58302805
chr19	58302302	58302447	chr19:58294097-58302805
chr19	58302467	58302823	chr19:58294097-58302805
chr19	58432822	58433711	chr19:58432813-58435211

chr19	58433732	58435226	chr19:58432813-58435211
chr19	58437032	58437255	chr19:58437025-58437215
chr19	58437882	58438101	chr19:58437878-58438078
chr19	58439767	58440259	chr19:58439758-58440222
chr19	58513532	58514350	chr19:58513529-58514310
chr19	58516397	58517721	chr19:58516394-58517673
chr19	58519027	58519591	chr19:58519018-58519559
chr19	58519617	58520255	chr19:58519616-58520222
chr19	58537167	58537374	chr19:58537154-58537354
chr19	58538707	58538923	chr19:58538700-58538911
chr19	58561942	58563517	chr19:58561931-58563504
chr19	58569212	58569429	chr19:58569204-58569397
chr19	58569527	58569600	chr19:58569515-58569586
chr19	58570347	58570564	chr19:58570343-58570527
chr19	58570997	58571468	chr19:58570993-58571429
chr19	58572557	58572807	chr19:58572549-58572775
chr19	58573062	58573382	chr19:58573054-58573352
chr20	325605	326550	chr20:325594-330224
chr20	326620	326929	chr20:325594-330224
chr20	326950	328526	chr20:325594-330224
chr20	328560	330140	chr20:325594-330224
chr20	330175	330251	chr20:325594-330224
chr20	603800	604289	chr20:603796-604665
chr20	604310	604698	chr20:603796-604665
chr20	608530	608751	chr20:608529-608729
chr20	609725	610037	chr20:609712-610398
chr20	610050	610425	chr20:609712-610398
chr20	661595	662624	chr20:661595-664461
chr20	662645	663000	chr20:661595-664461
chr20	663020	663203	chr20:661595-664461
chr20	663205	664492	chr20:661595-664461
chr20	669930	670146	chr20:669916-670116
chr20	675475	676177	chr20:675468-676179
chr20	2692935	2693107	chr20:2692877-2693148
chr20	2693575	2693803	chr20:2693573-2693782
chr20	2698420	2698631	chr20:2698415-2698615
chr20	2705590	2705763	chr20:2705576-2705733
chr20	2705980	2706051	chr20:2705973-2706037
chr20	2706220	2706304	chr20:2706208-2706264
chr20	2707950	2708074	chr20:2707946-2708020
chr20	2709575	2709654	chr20:2709573-2709642
chr20	2748550	2748661	chr20:2748548-2748630
chr20	2749400	2749540	chr20:2749400-2749518
chr20	2749630	2749762	chr20:2749619-2749753
chr20	2749860	2749990	chr20:2749846-2749973
chr20	2751695	2751798	chr20:2751690-2751788
chr20	2751930	2752276	chr20:2751921-2752263
chr20	2752370	2752577	chr20:2752356-2752545
chr20	2755090	2755834	chr20:2755087-2755824
chr20	2758915	2759014	chr20:2758908-2758984
chr20	2759275	2760119	chr20:2759267-2760108
chr20	11890722	11890977	chr20:11890722-11890954
chr20	11891327	11891470	chr20:11891326-11891447
chr20	11892502	11892576	chr20:11892492-11892543
chr20	11901442	11901542	chr20:11901435-11901635
chr20	11901592	11901665	chr20:11901435-11901635
chr20	11917787	11918106	chr20:11917777-11918601
chr20	11918127	11918606	chr20:11917777-11918601
chr20	11918812	11919200	chr20:11918809-11919176
chr20	11919277	11919858	chr20:11919276-11919836
chr20	11921567	11921679	chr20:11921565-11921662
chr20	11922637	11926635	chr20:11922633-11926609
chr20	17996564	17996779	chr20:17996560-17996752

chr20	18024152	18024990	chr20:18024151-18024952
chr20	18041547	18041755	chr20:18041533-18041723
chr20	18050652	18050750	chr20:18050640-18050739
chr20	18055437	18055638	chr20:18055426-18055626
chr20	18056657	18056899	chr20:18056656-18056877
chr20	18057537	18057898	chr20:18057534-18057877
chr20	18058902	18059078	chr20:18058889-18059050
chr20	18059082	18059222	chr20:18059079-18059188
chr20	18288287	18288640	chr20:18288282-18288604
chr20	18289902	18290091	chr20:18289898-18290074
chr20	18297987	18298098	chr20:18297984-18298062
chr20	18298302	18298479	chr20:18298288-18298464
chr20	18305007	18305196	chr20:18305007-18305178
chr20	18305667	18305787	chr20:18305667-18305807
chr20	18306282	18306424	chr20:18306270-18306393
chr20	18306707	18306780	chr20:18306705-18306747
chr20	18308217	18308428	chr20:18308192-18308392
chr20	18310172	18310312	chr20:18310168-18310306
chr20	18312637	18312714	chr20:18312635-18314767
chr20	18313027	18313126	chr20:18312635-18314767
chr20	18313127	18314803	chr20:18312635-18314767
chr20	18315082	18317033	chr20:18315068-18316996
chr20	18814097	18814299	chr20:18814086-18814286
chr20	20368120	20368696	chr20:20368120-20370949
chr20	20368700	20369753	chr20:20368120-20370949
chr20	20369760	20370960	chr20:20368120-20370949
chr20	21511022	21511222	chr20:21511009-21512485
chr20	21511272	21511439	chr20:21511009-21512485
chr20	21511457	21511814	chr20:21511009-21512485
chr20	21511827	21512492	chr20:21511009-21512485
chr20	21513412	21513825	chr20:21513410-21514026
chr20	21513837	21514039	chr20:21513410-21514026
chr20	21705662	21706023	chr20:21705658-21705998
chr20	21706222	21707101	chr20:21706222-21707067
chr20	21708562	21708734	chr20:21708557-21708700
chr20	21709227	21709481	chr20:21709221-21709444
chr20	21711262	21711463	chr20:21711249-21711449
chr20	21714482	21714925	chr20:21714470-21718486
chr20	21714947	21715360	chr20:21714470-21718486
chr20	21715372	21715757	chr20:21714470-21718486
chr20	21715797	21716490	chr20:21714470-21718486
chr20	21716522	21718492	chr20:21714470-21718486
chr20	22581018	22581147	chr20:22581004-22583154
chr20	22581153	22581329	chr20:22581004-22583154
chr20	22581353	22581457	chr20:22581004-22583154
chr20	22581518	22582047	chr20:22581004-22583154
chr20	22582048	22582715	chr20:22581004-22583154
chr20	22582723	22583174	chr20:22581004-22583154
chr20	22584203	22584334	chr20:22584191-22584463
chr20	22584383	22584475	chr20:22584191-22584463
chr20	22585278	22585464	chr20:22585274-22585455
chr20	23362184	23362258	chr20:23362181-23362237
chr20	23362324	23362884	chr20:23362319-23362850
chr20	23363234	23363336	chr20:23363220-23363317
chr20	23364374	23365282	chr20:23364362-23365747
chr20	23365294	23365783	chr20:23364362-23365747
chr20	23367014	23367124	chr20:23367002-23367097
chr20	23367839	23368064	chr20:23367839-23368039
chr20	23368774	23368945	chr20:23368761-23368929
chr20	23369584	23369766	chr20:23369583-23369741
chr20	23370099	23373073	chr20:23370090-23373063
chr20	31748115	31748341	chr20:31748115-31748315
chr20	31798455	31798572	chr20:31798452-31798552

chr20	31800980	31801154	chr20:31800969-31801119
chr20	31844310	31845324	chr20:31844300-31845619
chr20	31845335	31845645	chr20:31844300-31845619
chr20	32192504	32193768	chr20:32192502-32197682
chr20	32193769	32193831	chr20:32192502-32197682
chr20	32193849	32194689	chr20:32192502-32197682
chr20	32194699	32197697	chr20:32192502-32197682
chr20	32201919	32202322	chr20:32201918-32202292
chr20	32203024	32203239	chr20:32203011-32203211
chr20	32207654	32207762	chr20:32207640-32207729
chr20	33408459	33408634	chr20:33408449-33408599
chr20	33408709	33408838	chr20:33408700-33408800
chr20	33433969	33434179	chr20:33433969-33434169
chr20	33675694	33677011	chr20:33675682-33676979
chr20	33677109	33677357	chr20:33677104-33677330
chr20	33677429	33677579	chr20:33677425-33677540
chr20	33678209	33678385	chr20:33678200-33678353
chr20	33679754	33680004	chr20:33679754-33679974
chr20	33680329	33680447	chr20:33680325-33680416
chr20	33685024	33685239	chr20:33685013-33685213
chr20	33686014	33686298	chr20:33686003-33686404
chr20	33686304	33686441	chr20:33686003-33686404
chr20	35771980	35772090	chr20:35771973-35772079
chr20	35774500	35775093	chr20:35774487-35775058
chr20	35782565	35782645	chr20:35782327-35782609
chr20	35800095	35800206	chr20:35800093-35800173
chr20	35801490	35801624	chr20:35801490-35801605
chr20	35838360	35838458	chr20:35838354-35838442
chr20	35842575	35842768	chr20:35842572-35842744
chr20	35847355	35847457	chr20:35847349-35847434
chr20	35849390	35849512	chr20:35849388-35849483
chr20	35855230	35855302	chr20:35855230-35855291
chr20	35855305	35855478	chr20:35855300-35855572
chr20	35856360	35856560	chr20:35856355-35856530
chr20	35858310	35858417	chr20:35858301-35858381
chr20	35863015	35863438	chr20:35863012-35863400
chr20	35869450	35869584	chr20:35869437-35869551
chr20	35870965	35871172	chr20:35870954-35871134
chr20	35871650	35871859	chr20:35871649-35871829
chr20	35899380	35899665	chr20:35899369-35899648
chr20	35901955	35902181	chr20:35901950-35902150
chr20	35913250	35913319	chr20:35913248-35913347
chr20	35914045	35914228	chr20:35914032-35914197
chr20	35917190	35917406	chr20:35917179-35917387
chr20	35917490	35917703	chr20:35917483-35917662
chr20	35927790	35927899	chr20:35927779-35927879
chr20	35931260	35931491	chr20:35931248-35931444
chr20	35938710	35939130	chr20:35938696-35939108
chr20	35940865	35941082	chr20:35940863-35941047
chr20	35947490	35948744	chr20:35947484-35950381
chr20	35948760	35949777	chr20:35947484-35950381
chr20	35950075	35950418	chr20:35947484-35950381
chr20	37693961	37694169	chr20:37693954-37694152
chr20	37694931	37695042	chr20:37694926-37695033
chr20	37727321	37727429	chr20:37727301-37727402
chr20	37729401	37729474	chr20:37729387-37729587
chr20	37729481	37729624	chr20:37729387-37729587
chr20	37732886	37733064	chr20:37732878-37733067
chr20	37737391	37737496	chr20:37737377-37737484
chr20	37744656	37744732	chr20:37744644-37744713
chr20	37746471	37746690	chr20:37746467-37746647
chr20	37749856	37751154	chr20:37749850-37751130
chr20	37751496	37751700	chr20:37751485-37751666

chr20	37756301	37756408	chr20:37756290-37756391
chr20	37757566	37757679	chr20:37757558-37757656
chr20	37765206	37765322	chr20:37765196-37765290
chr20	37767956	37768069	chr20:37767952-37768044
chr20	37777286	37777465	chr20:37777272-37777417
chr20	37777656	37777735	chr20:37777653-37777712
chr20	37779191	37779368	chr20:37779186-37779335
chr20	37802876	37803082	chr20:37802866-37803048
chr20	37832256	37832375	chr20:37832247-37832345
chr20	37840101	37840222	chr20:37840101-37840199
chr20	37842261	37842434	chr20:37842253-37842419
chr20	37847861	37847935	chr20:37847857-37847891
chr20	37859906	37860052	chr20:37859898-37860036
chr20	37860271	37860390	chr20:37860271-37860344
chr20	37871926	37872133	chr20:37871924-37872129
chr20	38725026	38725133	chr20:38725014-38725114
chr20	38726381	38726584	chr20:38726367-38726567
chr20	38727461	38727634	chr20:38727451-38727601
chr20	40685851	40686269	chr20:40685847-40689240
chr20	40686291	40686670	chr20:40685847-40689240
chr20	40686671	40686951	chr20:40685847-40689240
chr20	40686961	40687235	chr20:40685847-40689240
chr20	40687261	40688358	chr20:40685847-40689240
chr20	40688361	40688434	chr20:40685847-40689240
chr20	40688456	40689267	chr20:40685847-40689240
chr20	41178461	41179637	chr20:41178447-41180305
chr20	41179921	41180034	chr20:41178447-41180305
chr20	41180041	41180322	chr20:41178447-41180305
chr20	41180821	41182813	chr20:41180814-41182780
chr20	41183196	41184712	chr20:41183139-41185201
chr20	41184741	41185232	chr20:41183139-41185201
chr20	41201286	41201396	chr20:41201285-41201384
chr20	41202066	41205067	chr20:41202056-41205066
chr20	41213736	41213813	chr20:41213733-41213899
chr20	41238701	41238833	chr20:41238687-41238796
chr20	41239606	41239711	chr20:41239597-41239704
chr20	41267471	41267649	chr20:41267462-41267612
chr20	41268991	41269100	chr20:41268978-41269083
chr20	41269421	41269522	chr20:41269410-41269485
chr20	41271926	41272299	chr20:41271926-41272273
chr20	41299971	41300156	chr20:41299965-41300127
chr20	41307726	41307945	chr20:41307725-41307925
chr20	41316866	41317218	chr20:41316861-41317186
chr20	41317521	41317617	chr20:41317508-41317672
chr20	41317621	41317697	chr20:41317508-41317672
chr20	43667031	43667334	chr20:43667018-43667303
chr20	43670501	43670721	chr20:43670500-43670700
chr20	43673811	43673922	chr20:43673805-43673899
chr20	43681796	43681873	chr20:43681783-43681855
chr20	43682806	43682901	chr20:43682793-43682886
chr20	43686851	43687103	chr20:43686851-43687072
chr20	43692161	43692345	chr20:43692156-43692319
chr20	43699756	43700082	chr20:43699756-43700044
chr20	43702491	43702914	chr20:43702489-43702903
chr20	43705221	43705376	chr20:43705218-43705358
chr20	43709976	43710072	chr20:43709962-43710062
chr20	43711501	43711634	chr20:43711487-43711601
chr20	43713011	43713125	chr20:43713001-43713106
chr20	43715146	43715320	chr20:43715133-43715283
chr20	43715966	43716534	chr20:43715958-43716496
chr20	43914871	43915009	chr20:43914863-43914990
chr20	43916156	43916234	chr20:43916149-43916208
chr20	43945706	43946069	chr20:43945704-43946038

chr20	43955151	43955305	chr20:43955147-43955272
chr20	43969106	43969284	chr20:43969095-43969295
chr20	43973366	43973444	chr20:43973366-43973432
chr20	44006546	44006840	chr20:44006546-44006792
chr20	44039006	44039267	chr20:44039003-44039229
chr20	44051311	44051565	chr20:44051305-44051545
chr20	44054301	44054567	chr20:44054298-44054526
chr20	44064786	44064885	chr20:44064776-44064857
chr20	44065721	44066141	chr20:44065711-44066107
chr20	44066741	44066887	chr20:44066729-44066857
chr20	44068656	44069636	chr20:44068649-44069616
chr20	44355701	44355875	chr20:44355699-44355853
chr20	44388646	44388862	chr20:44388637-44388837
chr20	44390461	44390709	chr20:44390457-44390679
chr20	44401281	44401524	chr20:44401269-44401487
chr20	44402556	44402669	chr20:44402553-44402644
chr20	44406066	44406248	chr20:44406057-44406232
chr20	44407391	44407493	chr20:44407380-44407475
chr20	44413696	44413820	chr20:44413693-44413800
chr20	44414516	44414692	chr20:44414506-44414662
chr20	44418436	44418543	chr20:44418424-44418512
chr20	44419721	44419907	chr20:44419720-44419876
chr20	44424031	44424137	chr20:44424017-44424639
chr20	44424146	44424390	chr20:44424017-44424639
chr20	44424396	44424673	chr20:44424017-44424639
chr20	44428346	44428521	chr20:44428334-44428487
chr20	44429536	44430947	chr20:44429522-44434596
chr20	44430991	44432347	chr20:44429522-44434596
chr20	44432366	44432917	chr20:44429522-44434596
chr20	44432921	44433345	chr20:44429522-44434596
chr20	44433681	44434625	chr20:44429522-44434596
chr20	45306851	45306964	chr20:45306850-45306944
chr20	45309576	45309714	chr20:45309566-45309692
chr20	45311601	45311675	chr20:45311588-45311659
chr20	45311851	45311990	chr20:45311838-45311954
chr20	45312221	45312406	chr20:45312220-45312395
chr20	45313471	45313623	chr20:45313467-45313605
chr20	45314046	45314123	chr20:45314034-45314144
chr20	45314416	45314604	chr20:45314412-45314565
chr20	45316196	45316371	chr20:45316186-45316342
chr20	45316481	45316615	chr20:45316476-45316590
chr20	45316691	45317220	chr20:45316685-45317822
chr20	45317241	45317847	chr20:45316685-45317822
chr20	45815856	45815934	chr20:45815853-45815913
chr20	45816751	45816846	chr20:45816708-45816858
chr20	46021786	46021889	chr20:46021786-46021886
chr20	46023026	46023099	chr20:46022971-46023071
chr20	46023371	46023549	chr20:46023368-46023518
chr20	46031081	46031290	chr20:46031075-46031275
chr20	46037296	46037400	chr20:46037285-46037385
chr20	46043636	46043714	chr20:46043632-46043692
chr20	46057211	46057320	chr20:46057203-46057303
chr20	46057521	46057697	chr20:46057513-46057663
chr20	46058486	46058840	chr20:46058480-46058630;chr20:46058655-46058805
chr20	46059601	46059786	chr20:46059598-46059748
chr20	47209216	47209463	chr20:47209213-47210897
chr20	47209466	47210398	chr20:47209213-47210897
chr20	47210461	47210919	chr20:47209213-47210897
chr20	47212651	47212761	chr20:47212641-47212725
chr20	47220266	47220338	chr20:47220257-47220324
chr20	47221326	47221501	chr20:47221313-47221474
chr20	47224326	47224572	chr20:47224316-47224556
chr20	47227206	47227332	chr20:47227202-47227281

chr20	47229726	47229850	chr20:47229725-47229806
chr20	47236326	47236555	chr20:47236325-47236516
chr20	47237441	47238300	chr20:47237309-47239138
chr20	47238306	47238384	chr20:47237309-47239138
chr20	47238386	47238594	chr20:47237309-47239138
chr20	47238596	47238667	chr20:47237309-47239138
chr20	47238671	47238805	chr20:47237309-47239138
chr20	47238836	47239154	chr20:47237309-47239138
chr20	47246016	47246408	chr20:47246007-47246517
chr20	47246416	47246549	chr20:47246007-47246517
chr20	47249286	47249479	chr20:47249286-47249439
chr20	47262291	47262445	chr20:47262287-47262428
chr20	47276321	47276815	chr20:47276313-47276795
chr20	47282106	47282254	chr20:47282101-47282217
chr20	47283571	47283685	chr20:47283570-47283648
chr20	47287231	47287304	chr20:47287228-47287284
chr20	47290196	47290303	chr20:47290186-47290274
chr20	47291796	47291920	chr20:47291795-47291888
chr20	47294676	47294811	chr20:47294665-47294779
chr20	47298731	47298978	chr20:47298728-47298947
chr20	47310091	47310235	chr20:47310055-47310204
chr20	47318951	47319100	chr20:47318950-47319061
chr20	47338221	47338426	chr20:47338215-47338415
chr20	47347856	47347966	chr20:47347855-47347926
chr20	47348016	47348087	chr20:47348016-47348072
chr20	47349866	47350011	chr20:47349860-47349988
chr20	47351711	47351891	chr20:47351709-47351877
chr20	47355461	47355561	chr20:47355449-47355657
chr20	47355576	47355686	chr20:47355449-47355657
chr20	47356386	47356460	chr20:47356379-47356414
chr20	47356656	47356916	chr20:47356656-47356889
chr20	49983006	49983179	chr20:49982998-49983141
chr20	49983831	49984391	chr20:49983823-49984351
chr20	49985611	49985833	chr20:49985607-49985807
chr20	49987881	49988899	chr20:49987871-49988886
chr20	50190741	50191063	chr20:50190733-50192689
chr20	50191086	50192224	chr20:50190733-50192689
chr20	50192236	50192519	chr20:50190733-50192689
chr20	50192531	50192695	chr20:50190733-50192689
chr20	51386956	51387865	chr20:51386956-51391451
chr20	51387871	51388490	chr20:51386956-51391451
chr20	51388511	51389135	chr20:51386956-51391451
chr20	51389151	51390697	chr20:51386956-51391451
chr20	51390701	51391462	chr20:51386956-51391451
chr20	51394166	51394370	chr20:51394155-51394355
chr20	51398651	51398759	chr20:51398642-51398730
chr20	51432066	51432781	chr20:51432066-51432756
chr20	51435201	51435339	chr20:51435187-51435314
chr20	51435706	51435781	chr20:51435705-51435761
chr20	51454556	51454708	chr20:51454547-51454688
chr20	51473991	51474173	chr20:51473979-51474152
chr20	51475466	51475687	chr20:51475457-51475660
chr20	51516786	51516970	chr20:51516783-51516955
chr20	51523091	51524132	chr20:51523080-51524110
chr20	51542371	51542737	chr20:51542369-51542719
chr20	51562561	51562847	chr20:51562559-51562831
chr20	51782341	51782519	chr20:51782330-51784684
chr20	51782566	51782699	chr20:51782330-51784684
chr20	51782706	51782777	chr20:51782330-51784684
chr20	51782781	51782881	chr20:51782330-51784684
chr20	51782911	51783009	chr20:51782330-51784684
chr20	51783011	51783732	chr20:51782330-51784684
chr20	51784056	51784717	chr20:51782330-51784684

chr20	51788901	51789175	chr20:51788860-51789141
chr20	51790026	51791206	chr20:51790021-51792352
chr20	51791221	51791617	chr20:51790021-51792352
chr20	51791651	51792188	chr20:51790021-51792352
chr20	51792191	51792376	chr20:51790021-51792352
chr20	51792761	51793118	chr20:51792758-51793078
chr20	51797426	51797604	chr20:51797425-51797573
chr20	51800146	51800370	chr20:51800142-51800342
chr20	51801496	51801602	chr20:51801490-51801586
chr20	51802291	51802526	chr20:51802278-51802520
chr20	52972541	52972701	chr20:52972406-52973333
chr20	52972836	52972941	chr20:52972406-52973333
chr20	52973036	52973355	chr20:52972406-52973333
chr20	53185296	53185404	chr20:53185282-53185676
chr20	53185636	53185714	chr20:53185282-53185676
chr20	53253556	53256603	chr20:53253498-53256571
chr20	53427731	53427954	chr20:53427730-53427930
chr20	53487156	53487265	chr20:53487143-53495330
chr20	53487286	53488709	chr20:53487143-53495330
chr20	53488716	53488844	chr20:53487143-53495330
chr20	53488846	53490824	chr20:53487143-53495330
chr20	53490831	53491066	chr20:53487143-53495330
chr20	53491076	53492073	chr20:53487143-53495330
chr20	53492086	53492777	chr20:53487143-53495330
chr20	53492781	53493729	chr20:53487143-53495330
chr20	53493751	53494000	chr20:53487143-53495330
chr20	53494306	53494381	chr20:53487143-53495330
chr20	53494386	53494524	chr20:53487143-53495330
chr20	53494536	53494635	chr20:53487143-53495330
chr20	53494651	53494827	chr20:53487143-53495330
chr20	53494836	53495340	chr20:53487143-53495330
chr20	53567066	53567211	chr20:53567064-53569264
chr20	53567271	53567833	chr20:53567064-53569264
chr20	53567841	53568744	chr20:53567064-53569264
chr20	53568761	53569281	chr20:53567064-53569264
chr20	53571731	53571867	chr20:53571720-53571853
chr20	53575726	53577312	chr20:53575726-53577280
chr20	53578336	53578443	chr20:53578333-53578450
chr20	53581466	53583179	chr20:53581460-53583168
chr20	53587031	53587205	chr20:53586988-53587188
chr20	53593776	53593849	chr20:53593755-53593839
chr20	53597641	53597743	chr20:53597630-53597703
chr20	53608366	53608472	chr20:53608353-53608444
chr20	53608841	53608917	chr20:53608831-53608892
chr20	53609751	53609933	chr20:53609747-53609907
chr20	56629306	56629619	chr20:56629301-56629592
chr20	56630236	56630388	chr20:56630233-56630372
chr20	56631206	56631716	chr20:56631204-56631690
chr20	56631816	56631900	chr20:56631804-56631856
chr20	56633366	56633604	chr20:56633352-56633569
chr20	56634151	56634303	chr20:56634149-56634268
chr20	56635116	56635331	chr20:56635109-56635309
chr20	56636611	56636800	chr20:56636609-56636754
chr20	56637741	56638666	chr20:56637727-56639283
chr20	56638676	56639304	chr20:56637727-56639283
chr20	57488781	57488852	chr20:57488391-57489027
chr20	57495971	57496042	chr20:57495965-57496364
chr20	57496151	57496298	chr20:57495965-57496364
chr20	57497166	57498712	chr20:57497163-57498701
chr20	57499821	57500085	chr20:57499553-57500410
chr20	57500101	57500174	chr20:57499553-57500410
chr20	57501826	57502049	chr20:57501826-57502026
chr20	57503446	57503622	chr20:57503435-57503601

chr20	57506741	57507180	chr20:57506741-57507876
chr20	57507481	57507893	chr20:57506741-57507876
chr20	57508616	57508826	chr20:57508605-57508788
chr20	57512601	57512793	chr20:57512591-57512752
chr20	57513246	57513908	chr20:57513235-57513872
chr20	57514601	57514775	chr20:57514591-57514741
chr20	57515471	57515869	chr20:57515464-57515834
chr20	57518501	57518922	chr20:57518494-57518891
chr20	57519206	57519389	chr20:57519206-57519377
chr20	57523081	57523289	chr20:57523067-57523278
chr20	57523676	57524889	chr20:57523662-57524867
chr20	57525031	57525606	chr20:57525027-57525652
chr20	62463501	62465020	chr20:62463496-62464991
chr20	62465351	62465490	chr20:62465339-62465464
chr20	62465846	62465947	chr20:62465833-62465921
chr20	62466436	62466572	chr20:62466425-62466551
chr20	62467976	62468158	chr20:62467953-62468153
chr20	62473406	62473583	chr20:62473402-62473578
chr20	62475001	62475576	chr20:62474998-62475542
chr20	62475936	62476004	chr20:62475929-62475970
chr20	62841311	62842129	chr20:62841309-62842097
chr20	62845401	62845550	chr20:62845137-62845513
chr20	62846066	62846140	chr20:62846062-62846117
chr20	62850816	62851029	chr20:62850808-62851008
chr20	62854016	62854201	chr20:62854015-62854157
chr20	62857396	62857656	chr20:62857394-62857638
chr20	62859366	62859548	chr20:62859363-62859526
chr20	62860126	62860348	chr20:62860124-62860308
chr20	62861036	62861797	chr20:62861023-62861763
chr20	62877746	62878160	chr20:62877737-62882414
chr20	62878166	62879033	chr20:62877737-62882414
chr20	62879051	62882438	chr20:62877737-62882414
chr20	62887226	62891175	chr20:62887213-62891155
chr20	62891986	62892100	chr20:62891986-62892076
chr20	62892811	62892984	chr20:62892808-62892962
chr20	62893666	62894232	chr20:62893665-62894194
chr20	62894421	62894562	chr20:62894412-62894548
chr20	62894811	62894930	chr20:62894809-62894914
chr20	62895061	62895194	chr20:62895048-62895165
chr20	62896236	62896414	chr20:62896232-62896392
chr20	62896536	62897034	chr20:62896530-62896996
chr20	62898576	62898794	chr20:62898573-62898773
chr20	62905011	62906107	chr20:62904997-62906100
chr20	62907151	62907400	chr20:62907146-62907359
chr20	62909711	62910055	chr20:62909698-62910020
chr20	62910786	62911628	chr20:62910773-62911614
chr20	62914221	62914422	chr20:62914209-62914406
chr20	62926441	62926537	chr20:62926431-62926503
chr20	62937796	62937977	chr20:62937795-62937952
chr20	63005931	63006476	chr20:63005926-63007035
chr20	63006481	63007068	chr20:63005926-63007035
chr20	63587611	63590767	chr20:63587601-63590729
chr20	63592026	63592180	chr20:63592021-63592144
chr20	63592546	63592682	chr20:63592532-63592670
chr20	63593021	63593122	chr20:63593010-63593082
chr20	63595621	63595797	chr20:63595609-63595767
chr20	63597761	63597865	chr20:63597756-63597860
chr20	63602971	63603124	chr20:63602964-63603092
chr20	63604751	63604938	chr20:63604742-63604902
chr20	63619276	63619909	chr20:63619266-63619876
chr20	63626961	63627030	chr20:63626955-63627041
chr20	63708556	63709197	chr20:63708552-63709164
chr20	63733226	63733377	chr20:63733218-63733352

chr20	63733586	63733980	chr20:63733586-63733970
chr20	63734651	63734940	chr20:63734644-63735564
chr20	63735126	63735578	chr20:63734644-63735564
chr20	63956711	63956888	chr20:63956701-63960139
chr20	63956896	63957832	chr20:63956701-63960139
chr20	63957836	63960176	chr20:63956701-63960139
chr20	63961321	63961426	chr20:63961308-63961407
chr20	63961941	63962024	chr20:63961941-63962004
chr20	63962276	63962391	chr20:63962272-63962374
chr20	63962591	63962811	chr20:63962586-63962781
chr20	63963096	63963276	chr20:63963094-63963265
chr20	63963351	63963452	chr20:63963341-63963440
chr20	63963626	63963743	chr20:63963617-63963710
chr20	63963796	63963948	chr20:63963788-63963913
chr20	63964081	63964259	chr20:63964070-63964217
chr20	63964321	63964419	chr20:63964319-63964391
chr20	63964491	63964749	chr20:63964489-63964716
chr20	63966141	63966811	chr20:63966140-63966781
chr20	63966886	63967021	chr20:63966875-63967004
chr20	63967381	63967566	chr20:63967380-63967523
chr20	63967831	63967990	chr20:63967829-63967952
chr20	63968786	63969004	chr20:63968783-63968983
chr20	63969816	63969880	chr20:63969813-63969865
chr20	64047586	64047756	chr20:64047581-64048962
chr20	64047761	64048990	chr20:64047581-64048962
chr20	64049166	64049677	chr20:64049158-64049641
chr20	64151801	64151933	chr20:64151790-64151910
chr20	64164486	64164758	chr20:64164473-64164739
chr20	64190066	64190192	chr20:64190062-64190160
chr20	64191496	64191918	chr20:64191486-64191884
chr20	64198861	64198937	chr20:64198861-64198916
chr20	64199896	64199977	chr20:64199891-64199922
chr20	64205046	64205120	chr20:64205034-64205097
chr20	64205566	64205818	chr20:64205552-64205800
chr20	64207606	64207991	chr20:64207593-64208487
chr20	64208106	64208525	chr20:64207593-64208487
chr20	64211206	64211349	chr20:64211205-64211340
chr20	64212061	64212165	chr20:64212047-64212138
chr20	64213546	64213693	chr20:64213533-64213647
chr20	64217066	64217311	chr20:64217066-64217281
chr20	64218831	64219062	chr20:64218829-64219035
chr20	64219716	64220014	chr20:64219712-64219982
chr20	64221906	64222078	chr20:64221892-64222047
chr20	64223121	64223195	chr20:64223110-64223173
chr20	64223296	64223379	chr20:64223290-64223359
chr20	64225301	64225516	chr20:64225287-64225487
chr20	64227421	64227498	chr20:64227414-64227477
chr20	64227901	64228135	chr20:64227887-64228102
chr20	64232176	64232423	chr20:64232163-64232385
chr20	64236566	64236667	chr20:64236554-64236646
chr20	64237286	64237402	chr20:64237286-64237390
chr20	64239761	64239943	chr20:64239759-64239903
chr20	64240321	64241623	chr20:64240319-64242253
chr20	64241636	64241801	chr20:64240319-64242253
chr20	64241836	64242292	chr20:64240319-64242253
chr21	25734574	25734712	chr21:25734569-25734702
chr21	25741584	25741690	chr21:25741572-25741675
chr21	25745209	25745360	chr21:25745209-25745354
chr21	25749039	25749145	chr21:25749035-25749120
chr21	25750824	25751033	chr21:25750815-25751015
chr21	25751999	25752384	chr21:25751988-25752348
chr21	25758009	25758221	chr21:25758009-25758204
chr21	25762314	25762391	chr21:25762311-25762365

chr21	25764209	25764357	chr21:25764209-25764350
chr21	25764604	25764805	chr21:25764594-25764787
chr21	25769009	25770081	chr21:25769003-25772460
chr21	25770089	25770484	chr21:25769003-25772460
chr21	25770489	25771108	chr21:25769003-25772460
chr21	25771124	25771353	chr21:25769003-25772460
chr21	25771359	25771432	chr21:25769003-25772460
chr21	25771454	25771621	chr21:25769003-25772460
chr21	25771624	25771757	chr21:25769003-25772460
chr21	25771774	25772319	chr21:25769003-25772460
chr21	25772354	25772447	chr21:25769003-25772460
chr21	29194082	29194286	chr21:29194070-29194260
chr21	29218862	29218937	chr21:29218860-29218926
chr21	29287987	29288130	chr21:29287983-29288095
chr21	29298877	29298988	chr21:29298870-29298953
chr21	29299417	29299633	chr21:29299415-29299598
chr21	29299692	29299802	chr21:29299682-29299764
chr21	29300142	29300214	chr21:29300132-29300175
chr21	29300797	29300872	chr21:29300801-29300832
chr21	29305202	29305357	chr21:29305198-29305322
chr21	29321232	29321550	chr21:29321220-29321514
chr21	29326072	29327422	chr21:29326058-29327393
chr21	29329492	29329706	chr21:29329486-29329693
chr21	29342412	29344622	chr21:29342398-29346148
chr21	29344682	29345799	chr21:29342398-29346148
chr21	29345802	29346180	chr21:29342398-29346148
chr21	29582317	29582390	chr21:29582311-29582351
chr21	29596052	29596410	chr21:29596039-29596375
chr21	29596722	29596793	chr21:29596720-29596778
chr21	29597572	29597730	chr21:29597569-29597694
chr21	29616672	29616889	chr21:29616663-29616863
chr21	29630577	29630727	chr21:29630538-29630751
chr21	33025856	33026726	chr21:33025844-33029196
chr21	33026771	33028788	chr21:33025844-33029196
chr21	33028806	33029184	chr21:33025844-33029196
chr21	33070156	33070362	chr21:33070143-33072420
chr21	33070396	33070545	chr21:33070143-33072420
chr21	33070556	33072398	chr21:33070143-33072420
chr21	33557227	33557335	chr21:33557216-33557316
chr21	33557547	33557739	chr21:33557545-33557695
chr21	33559882	33560059	chr21:33559876-33560026
chr21	33563587	33563812	chr21:33563579-33563779
chr21	33576377	33576556	chr21:33576364-33576514
chr21	34787803	34788364	chr21:34787800-34792610
chr21	34788368	34788533	chr21:34787800-34792610
chr21	34788553	34789564	chr21:34787800-34792610
chr21	34789608	34789880	chr21:34787800-34792610
chr21	34789883	34789976	chr21:34787800-34792610
chr21	34789998	34791299	chr21:34787800-34792610
chr21	34791303	34791436	chr21:34787800-34792610
chr21	34791483	34791548	chr21:34787800-34792610
chr21	34791578	34792630	chr21:34787800-34792610
chr21	34799303	34799495	chr21:34799300-34799462
chr21	34821278	34821440	chr21:34821278-34821696
chr21	34821453	34821734	chr21:34821278-34821696
chr21	34829853	34830009	chr21:34829851-34829970
chr21	34834413	34834627	chr21:34834409-34834601
chr21	34856298	34856479	chr21:34856288-34856447
chr21	34859473	34859584	chr21:34859473-34859578
chr21	34880558	34880747	chr21:34880556-34880713
chr21	34886843	34887700	chr21:34886842-34888690
chr21	34887703	34888497	chr21:34886842-34888690
chr21	34888518	34888727	chr21:34886842-34888690

chr21	34889678	34889814	chr21:34889676-34889790
chr21	34892923	34892995	chr21:34892924-34892963
chr21	35048853	35049118	chr21:35048841-35049344
chr21	35049128	35049375	chr21:35048841-35049344
chr21	35064718	35064794	chr21:35064715-35064829
chr21	35064943	35065086	chr21:35064935-35065072
chr21	35146358	35146526	chr21:35146346-35146546
chr21	35372508	35372828	chr21:35372506-35372793
chr21	35435718	35436584	chr21:35435660-35439071
chr21	35436588	35436841	chr21:35435660-35439071
chr21	35437033	35437242	chr21:35435660-35439071
chr21	35437258	35437775	chr21:35435660-35439071
chr21	35437808	35438222	chr21:35435660-35439071
chr21	35438223	35439105	chr21:35435660-35439071
chr21	35461968	35462039	chr21:35461960-35462025
chr21	35465193	35465366	chr21:35465183-35465329
chr21	35580663	35580955	chr21:35580659-35580929
chr21	35654828	35654901	chr21:35654819-35654875
chr21	35864298	35864495	chr21:35864284-35864467
chr21	35954788	35954857	chr21:35954659-35954825
chr21	35981753	35981936	chr21:35981750-35981916
chr21	35984573	35984860	chr21:35984571-35984830
chr21	36004563	36004685	chr21:36004563-36004667
chr21	36699138	36699417	chr21:36699132-36699921
chr21	36699433	36699951	chr21:36699132-36699921
chr21	36709168	36709810	chr21:36709167-36710613
chr21	36710088	36710161	chr21:36709167-36710613
chr21	36710163	36710591	chr21:36709167-36710613
chr21	36712543	36712653	chr21:36712532-36712622
chr21	36715918	36716124	chr21:36715905-36716105
chr21	36719823	36719939	chr21:36719820-36720587
chr21	36719948	36720612	chr21:36719820-36720587
chr21	36723058	36723155	chr21:36723044-36723130
chr21	36726118	36726339	chr21:36726118-36726318
chr21	36731053	36731161	chr21:36731044-36731151
chr21	36741718	36741905	chr21:36741716-36741864
chr21	36743388	36743575	chr21:36743386-36743555
chr21	36744728	36745156	chr21:36744727-36745136
chr21	36745798	36745905	chr21:36745794-36745871
chr21	36747673	36748625	chr21:36747664-36749917
chr21	36748648	36749451	chr21:36747664-36749917
chr21	36749463	36749953	chr21:36747664-36749917
chr21	38380027	38383950	chr21:38380026-38383923
chr21	38391002	38391077	chr21:38390994-38391042
chr21	38391672	38391749	chr21:38391658-38391715
chr21	38392387	38392460	chr21:38392375-38392444
chr21	38395372	38395591	chr21:38395369-38395580
chr21	38400577	38400650	chr21:38400573-38400645
chr21	38402562	38402642	chr21:38402556-38402637
chr21	38403517	38403724	chr21:38403505-38403709
chr21	38423417	38423601	chr21:38423409-38423561
chr21	38445407	38445657	chr21:38445403-38445621
chr21	38449027	38449137	chr21:38449021-38449105
chr21	38470777	38472117	chr21:38470774-38472080
chr21	38474032	38474141	chr21:38474026-38474121
chr21	38498367	38498510	chr21:38498362-38498483
chr21	38575662	38575778	chr21:38575661-38575747
chr21	38584847	38584963	chr21:38584843-38584945
chr21	38657202	38657409	chr21:38657202-38657402
chr21	38660522	38660699	chr21:38660521-38660667
chr21	38661667	38661809	chr21:38661657-38661780
chr21	38805307	38805635	chr21:38805306-38805616
chr21	38805872	38806148	chr21:38805830-38806120

chr21	38806452	38806846	chr21:38806450-38806808
chr21	38809647	38809825	chr21:38809635-38809796
chr21	38810037	38810111	chr21:38810034-38810106
chr21	38811347	38811576	chr21:38811347-38811547
chr21	38813007	38813152	chr21:38813002-38813114
chr21	38814272	38814418	chr21:38814272-38814392
chr21	38814792	38815008	chr21:38814780-38814981
chr21	38817017	38817121	chr21:38817007-38817091
chr21	38818432	38818686	chr21:38818424-38818646
chr21	38819502	38819793	chr21:38819502-38819766
chr21	38821597	38821740	chr21:38821585-38821704
chr21	38822687	38823619	chr21:38822673-38824955
chr21	38823642	38824971	chr21:38822673-38824955
chr21	41798232	41799274	chr21:41798224-41801722
chr21	41799277	41800496	chr21:41798224-41801722
chr21	41800512	41800830	chr21:41798224-41801722
chr21	41800852	41801225	chr21:41798224-41801722
chr21	41801257	41801747	chr21:41798224-41801722
chr21	41802717	41802823	chr21:41802711-41803118
chr21	41802842	41803123	chr21:41802711-41803118
chr21	41804542	41804758	chr21:41804533-41804723
chr21	41810162	41810492	chr21:41810153-41810466
chr21	41810752	41810869	chr21:41810752-41810836
chr21	41815707	41815863	chr21:41815704-41815836
chr21	41819582	41819815	chr21:41819581-41819783
chr21	41820102	41820213	chr21:41820094-41820174
chr21	41821067	41821249	chr21:41821066-41821230
chr21	41821912	41822049	chr21:41821902-41822037
chr21	41823317	41823468	chr21:41823317-41823449
chr21	41825967	41826068	chr21:41825959-41826054
chr21	41828167	41828353	chr21:41828165-41828333
chr21	41832668	41832882	chr21:41832668-41832868
chr21	41834513	41834585	chr21:41834504-41834564
chr21	41835448	41835555	chr21:41835436-41835524
chr21	41836123	41836225	chr21:41836112-41836207
chr21	41836468	41836684	chr21:41836467-41836649
chr21	41836878	41836951	chr21:41836875-41836932
chr21	41837933	41838091	chr21:41837933-41838063
chr21	41839633	41839885	chr21:41839622-41839853
chr21	41847103	41847201	chr21:41847089-41847191
chr21	41854573	41854849	chr21:41854565-41854818
chr21	41857183	41857357	chr21:41857175-41857329
chr21	41859043	41859262	chr21:41859030-41859228
chr21	41859603	41859719	chr21:41859591-41859685
chr21	41860338	41860421	chr21:41860326-41860378
chr21	41861563	41861790	chr21:41861562-41861751
chr21	41861928	41862041	chr21:41861924-41862007
chr21	41863238	41863318	chr21:41863234-41863302
chr21	41867303	41867407	chr21:41867299-41867377
chr21	41871503	41871648	chr21:41871493-41871621
chr21	41878698	41878869	chr21:41878694-41878860
chr21	41879008	41879173	chr21:41878995-41879140
chr21	41879278	41879482	chr21:41879269-41879482
chr21	41986843	41988751	chr21:41986830-41994108
chr21	41988798	41990797	chr21:41986830-41994108
chr21	41990818	41992210	chr21:41986830-41994108
chr21	41992213	41994132	chr21:41986830-41994108
chr21	41998753	41998927	chr21:41998753-41998953
chr21	42002363	42002602	chr21:42002349-42002563
chr21	42002898	42002973	chr21:42002896-42002961
chr21	42007823	42008082	chr21:42007819-42008057
chr21	42009478	42009609	chr21:42009466-42009597
chr21	42010263	42010419	chr21:42010251-42010387

chr21	42974509	42974695	chr21:42974509-42974664
chr21	43004324	43004474	chr21:43004321-43004432
chr21	43007494	43007644	chr21:43007490-43007618
chr21	43010064	43010232	chr21:43010052-43010224
chr21	43013074	43013249	chr21:43013067-43013238
chr21	43016909	43017026	chr21:43016907-43017007
chr21	43018139	43018252	chr21:43018132-43018230
chr21	43020464	43020566	chr21:43020450-43020548
chr21	43021304	43021452	chr21:43021302-43021431
chr21	43024444	43024762	chr21:43024434-43024734
chr21	43024884	43024989	chr21:43024870-43024947
chr21	43025824	43026030	chr21:43025812-43026012
chr21	43028704	43028891	chr21:43028701-43028875
chr21	43029894	43030256	chr21:43029889-43033931
chr21	43030299	43031873	chr21:43029889-43033931
chr21	43032154	43032507	chr21:43029889-43033931
chr21	43032609	43033546	chr21:43029889-43033931
chr21	43033564	43033956	chr21:43029889-43033931
chr21	44285890	44286160	chr21:44285879-44286138
chr21	44286565	44286741	chr21:44286556-44286731
chr21	44286990	44287170	chr21:44286977-44287133
chr21	44287520	44287633	chr21:44287516-44287591
chr21	44288345	44288497	chr21:44288344-44288458
chr21	44289665	44289837	chr21:44289656-44289802
chr21	44290000	44290096	chr21:44289987-44290068
chr21	44291095	44291246	chr21:44291094-44291210
chr21	44292305	44292410	chr21:44292301-44292401
chr21	44293005	44293211	chr21:44292992-44293175
chr21	44293795	44293942	chr21:44293788-44293910
chr21	44294400	44294516	chr21:44294400-44294503
chr21	44295355	44295570	chr21:44295343-44295543
chr21	44296385	44296463	chr21:44296382-44296445
chr21	44297655	44297973	chr21:44297655-44298219
chr21	44298155	44298265	chr21:44297655-44298219
chr22	17575511	17575719	chr22:17575509-17575709
chr22	17589591	17589704	chr22:17589589-17589665
chr22	17590096	17590280	chr22:17590094-17590244
chr22	18916899	18917014	chr22:18916889-18916988
chr22	18918319	18918506	chr22:18918315-18918465
chr22	18929014	18929223	chr22:18929000-18929200
chr22	19148589	19149106	chr22:19148575-19149095
chr22	19149674	19149951	chr22:19149662-19149916
chr22	19150079	19150309	chr22:19150024-19150283
chr22	19523599	19523826	chr22:19523598-19523798
chr22	19524394	19524583	chr22:19524392-19524542
chr22	19525159	19525260	chr22:19525154-19525254
chr22	19526524	19526641	chr22:19526516-19526616
chr22	19756709	19756776	chr22:19756702-19756745
chr22	19759559	19759716	chr22:19759557-19759677
chr22	19760914	19761010	chr22:19760904-19761280
chr22	19761059	19761299	chr22:19760904-19761280
chr22	19763254	19763359	chr22:19763240-19763342
chr22	19764164	19764338	chr22:19764154-19764326
chr22	19764964	19765148	chr22:19764957-19765113
chr22	19765764	19765839	chr22:19765757-19765825
chr22	19765904	19766013	chr22:19765901-19766002
chr22	19766389	19766464	chr22:19766388-19767334
chr22	19766494	19767370	chr22:19766388-19767334
chr22	19770714	19770932	chr22:19770712-19770912
chr22	19779219	19779580	chr22:19779219-19779546
chr22	19782924	19783059	chr22:19782912-19783032
chr22	21417403	21417578	chr22:21417403-21417560
chr22	21420638	21420795	chr22:21420635-21420757

chr22	21441248	21441474	chr22:21441247-21441447
chr22	21442463	21442890	chr22:21442459-21442857
chr22	21444933	21446076	chr22:21444921-21451463
chr22	21446083	21447136	chr22:21444921-21451463
chr22	21447143	21447359	chr22:21444921-21451463
chr22	21447363	21447784	chr22:21444921-21451463
chr22	21447793	21448539	chr22:21444921-21451463
chr22	21448543	21449212	chr22:21444921-21451463
chr22	21449258	21449427	chr22:21444921-21451463
chr22	21449428	21450230	chr22:21444921-21451463
chr22	21450243	21451139	chr22:21444921-21451463
chr22	21451143	21451503	chr22:21444921-21451463
chr22	27748285	27748364	chr22:27748276-27751096
chr22	27748385	27749822	chr22:27748276-27751096
chr22	27749830	27750453	chr22:27748276-27751096
chr22	27750545	27751138	chr22:27748276-27751096
chr22	27790010	27790224	chr22:27790006-27790206
chr22	27791750	27791903	chr22:27791746-27791883
chr22	27796765	27798888	chr22:27796762-27801498
chr22	27798960	27799629	chr22:27796762-27801498
chr22	27799650	27801534	chr22:27796762-27801498
chr22	28794560	28795752	chr22:28794554-28795732
chr22	28796050	28796228	chr22:28796046-28796192
chr22	28797090	28797220	chr22:28797076-28797205
chr22	28799050	28799194	chr22:28799041-28799153
chr22	28799960	28800138	chr22:28799959-28800133
chr22	28800305	28800629	chr22:28800297-28800597
chr22	29270857	29271083	chr22:29270849-29271049
chr22	29288687	29288800	chr22:29288686-29288786
chr22	29290422	29290589	chr22:29290412-29290562
chr22	29299752	29299866	chr22:29299751-29299851
chr22	29300122	29300304	chr22:29300121-29300271
chr22	31325817	31325986	chr22:31325803-31327309
chr22	31325987	31326161	chr22:31325803-31327309
chr22	31326182	31327342	chr22:31325803-31327309
chr22	31328787	31328932	chr22:31328786-31328924
chr22	31332702	31332777	chr22:31332690-31332890
chr22	31332797	31332901	chr22:31332690-31332890
chr22	31335702	31335876	chr22:31335691-31335863
chr22	31340687	31341694	chr22:31340676-31341683
chr22	31342897	31342979	chr22:31342896-31342960
chr22	31344342	31346220	chr22:31344331-31346232
chr22	36991132	36991315	chr22:36991126-36991276
chr22	36991482	36991608	chr22:36991480-36991580
chr22	36998367	36998597	chr22:36998366-36998566
chr22	37970685	37971320	chr22:37970685-37971298
chr22	37972465	37974220	chr22:37972460-37974198
chr22	37977880	37978169	chr22:37977866-37978135
chr22	37983360	37983445	chr22:37983356-37983868
chr22	37983450	37983799	chr22:37983356-37983868
chr22	37983810	37983878	chr22:37983356-37983868
chr22	37984200	37984559	chr22:37984198-37984537
chr22	38201895	38202268	chr22:38201881-38202238
chr22	38202795	38203111	chr22:38202795-38203084
chr22	38213545	38213730	chr22:38213534-38213706
chr22	38213835	38213913	chr22:38213822-38213889
chr22	38214420	38216107	chr22:38214419-38216511
chr22	38216390	38216531	chr22:38214419-38216511
chr22	38738610	38738795	chr22:38738604-38738754
chr22	38738880	38738994	chr22:38738872-38738972
chr22	38761025	38761242	chr22:38761019-38761219
chr22	39521585	39521691	chr22:39521571-39521671
chr22	39521780	39521955	chr22:39521772-39521922

chr22	41091786	41093125	chr22:41091785-41093098
chr22	41094271	41094488	chr22:41094265-41094465
chr22	41117191	41117862	chr22:41117186-41117821
chr22	41125866	41126056	chr22:41125863-41126040
chr22	41127496	41127782	chr22:41127486-41127748
chr22	41129896	41130039	chr22:41129889-41130003
chr22	41131391	41131660	chr22:41131387-41131633
chr22	41135821	41135935	chr22:41135812-41135906
chr22	41137666	41137813	chr22:41137652-41137790
chr22	41140151	41140287	chr22:41140139-41140257
chr22	41141061	41141237	chr22:41141047-41141222
chr22	41146751	41146830	chr22:41146738-41146816
chr22	41147836	41147981	chr22:41147836-41147946
chr22	41149051	41149196	chr22:41149037-41149175
chr22	41149761	41150234	chr22:41149760-41150198
chr22	41151841	41152051	chr22:41151832-41152012
chr22	41152206	41152382	chr22:41152205-41152350
chr22	41154996	41155132	chr22:41154994-41155113
chr22	41157176	41157420	chr22:41157168-41157408
chr22	41158411	41158522	chr22:41158411-41158500
chr22	41160641	41160751	chr22:41160641-41160722
chr22	41162736	41162808	chr22:41162722-41162779
chr22	41164056	41164167	chr22:41164052-41164130
chr22	41166601	41166670	chr22:41166598-41166666
chr22	41168471	41168611	chr22:41168466-41168599
chr22	41168721	41168906	chr22:41168720-41168867
chr22	41169291	41169361	chr22:41169282-41169329
chr22	41169511	41169646	chr22:41169502-41169616
chr22	41170411	41170588	chr22:41170405-41170571
chr22	41172511	41172689	chr22:41172498-41172663
chr22	41173626	41173810	chr22:41173622-41173784
chr22	41176246	41176569	chr22:41176246-41176528
chr22	41176781	41179281	chr22:41176772-41180079
chr22	41179296	41179458	chr22:41176772-41180079
chr22	41179541	41179860	chr22:41176772-41180079
chr22	41179911	41180114	chr22:41176772-41180079
chr22	41367336	41367628	chr22:41367332-41367599
chr22	41368961	41369176	chr22:41368948-41369148
chr22	41381931	41382211	chr22:41381928-41382201
chr22	41382936	41383007	chr22:41382934-41382988
chr22	41387421	41387707	chr22:41387350-41387668
chr22	41394096	41394340	chr22:41394095-41394316
chr22	41395751	41399346	chr22:41395744-41399326
chr22	41883562	41883778	chr22:41883558-41883758
chr22	41904887	41904991	chr22:41904874-41904974
chr22	41905442	41905628	chr22:41905439-41905589
chr22	42160067	42160169	chr22:42160012-42161358
chr22	42160227	42160638	chr22:42160012-42161358
chr22	42160682	42161054	chr22:42160012-42161358
chr22	42161057	42161121	chr22:42160012-42161358
chr22	42161127	42161401	chr22:42160012-42161358
chr22	42168622	42168755	chr22:42168608-42168736
chr22	42169847	42169926	chr22:42169846-42169896
chr22	42179612	42179719	chr22:42179608-42179702
chr22	42209662	42209973	chr22:42209650-42215442
chr22	42209987	42214335	chr22:42209650-42215442
chr22	42214367	42214577	chr22:42209650-42215442
chr22	42214587	42214765	chr22:42209650-42215442
chr22	42214772	42215469	chr22:42209650-42215442
chr22	42305647	42305865	chr22:42305641-42305841
chr22	42343482	42343635	chr22:42343478-42343616
chr22	46150524	46150661	chr22:46150520-46150652
chr22	46150839	46151180	chr22:46150827-46151177

chr22	46151894	46151994	chr22:46151883-46151970
chr22	46156234	46156612	chr22:46156229-46156593
chr22	46171294	46171508	chr22:46171283-46171492
chr22	46176774	46176880	chr22:46176747-46176836
chr22	46188734	46188847	chr22:46188722-46188822
chr22	46198344	46198627	chr22:46198341-46198591
chr22	46215174	46215400	chr22:46215172-46215364
chr22	46218264	46218413	chr22:46218262-46218401
chr22	46219819	46220027	chr22:46219811-46220014
chr22	46224554	46224760	chr22:46224526-46224726
chr22	46231794	46232274	chr22:46231791-46232239
chr22	46235144	46235978	chr22:46235132-46243756
chr22	46236264	46237270	chr22:46235132-46243756
chr22	46237619	46241910	chr22:46235132-46243756
chr22	46241934	46242002	chr22:46235132-46243756
chr22	46242014	46242717	chr22:46235132-46243756
chr22	46242754	46243553	chr22:46235132-46243756
chr22	46243569	46243734	chr22:46235132-46243756
chr22	50489230	50489310	chr22:50489227-50489295
chr22	50489535	50489677	chr22:50489531-50489644
chr22	50489755	50489931	chr22:50489747-50489897
chrX	624401	624636	chrX:624343-624602
chrX	630466	631202	chrX:630465-631174
chrX	632936	633149	chrX:632924-633124
chrX	634631	634837	chrX:634617-634826
chrX	640821	640895	chrX:640820-640878
chrX	641011	641108	chrX:640998-641087
chrX	644401	645398	chrX:644390-646823
chrX	645426	645539	chrX:644390-646823
chrX	645556	645899	chrX:644390-646823
chrX	646156	646531	chrX:644390-646823
chrX	646551	646719	chrX:644390-646823
chrX	646721	646793	chrX:644390-646823
chrX	659351	659444	chrX:658784-659411
chrX	2486423	2486521	chrX:2486413-2490772
chrX	2486523	2487375	chrX:2486413-2490772
chrX	2487613	2488197	chrX:2486413-2490772
chrX	2488443	2490790	chrX:2486413-2490772
chrX	2498778	2498976	chrX:2498768-2498968
chrX	9725694	9725880	chrX:9725690-9725840
chrX	9734614	9734820	chrX:9734606-9734806
chrX	9739484	9739597	chrX:9739484-9739584
chrX	21855986	21858619	chrX:21855986-21858727
chrX	24149175	24149279	chrX:24149172-24149253
chrX	24149630	24149805	chrX:24149628-24149794
chrX	24149855	24150145	chrX:24149853-24150130
chrX	24150515	24150819	chrX:24150505-24150819
chrX	24151435	24151504	chrX:24151425-24151481
chrX	24151730	24151837	chrX:24151690-24151800
chrX	24152720	24152863	chrX:24152719-24152830
chrX	24172715	24172942	chrX:24172714-24172914
chrX	24175390	24175473	chrX:24175388-24175443
chrX	24177690	24177878	chrX:24177683-24177866
chrX	24179185	24179798	chrX:24179182-24179770
chrX	24202265	24202487	chrX:24202259-24202459
chrX	24207335	24207514	chrX:24207325-24207475
chrX	24207725	24207893	chrX:24207711-24207855
chrX	24208220	24208407	chrX:24208217-24208370
chrX	24208900	24209087	chrX:24208899-24209040
chrX	24210200	24212875	chrX:24210192-24216255
chrX	24213190	24213439	chrX:24210192-24216255
chrX	24213470	24213675	chrX:24210192-24216255
chrX	24213690	24213899	chrX:24210192-24216255

chrX	24213910	24215829	chrX:24210192-24216255
chrX	24215860	24216062	chrX:24210192-24216255
chrX	24216065	24216283	chrX:24210192-24216255
chrX	25003718	25003990	chrX:25003693-25004910
chrX	25003993	25004264	chrX:25003693-25004910
chrX	25004343	25004945	chrX:25003693-25004910
chrX	25005973	25006050	chrX:25005969-25006005
chrX	25006548	25006661	chrX:25006544-25006627
chrX	25007113	25007224	chrX:25007110-25007439
chrX	25007253	25007466	chrX:25007110-25007439
chrX	25008898	25009113	chrX:25008895-25009095
chrX	25010263	25010338	chrX:25010259-25010305
chrX	25012923	25013662	chrX:25012921-25013798
chrX	25013678	25013823	chrX:25012921-25013798
chrX	25015543	25015981	chrX:25015541-25015948
chrX	25016173	25016245	chrX:25016170-25016205
chrX	25016358	25016431	chrX:25016353-25016420
chrX	30304217	30304470	chrX:30304205-30304823
chrX	30304497	30304856	chrX:30304205-30304823
chrX	30305687	30305885	chrX:30305673-30305855
chrX	30307092	30307297	chrX:30307080-30307280
chrX	30308197	30308269	chrX:30308195-30309598
chrX	30308297	30309627	chrX:30308195-30309598
chrX	41689756	41689863	chrX:41689742-41689835
chrX	41694731	41694951	chrX:41694727-41694927
chrX	41695561	41695905	chrX:41695554-41695898
chrX	46446857	46447352	chrX:46446856-46447346
chrX	46448212	46448353	chrX:46448199-46448310
chrX	46450432	46450506	chrX:46450430-46450475
chrX	46462757	46462893	chrX:46462747-46462874
chrX	46463197	46463492	chrX:46463197-46463449
chrX	46472027	46472554	chrX:46472024-46472526
chrX	46472752	46473323	chrX:46472749-46474639
chrX	46473332	46473665	chrX:46472749-46474639
chrX	46473937	46474174	chrX:46472749-46474639
chrX	46474292	46474636	chrX:46472749-46474639
chrX	46476902	46477124	chrX:46476902-46477102
chrX	46497132	46497339	chrX:46497121-46497422
chrX	46497727	46498616	chrX:46497726-46501335
chrX	46498907	46499041	chrX:46497726-46501335
chrX	46499447	46500170	chrX:46497726-46501335
chrX	46500187	46501376	chrX:46497726-46501335
chrX	46522942	46523318	chrX:46522936-46523618
chrX	46528347	46528484	chrX:46528334-46528445
chrX	46528782	46528946	chrX:46528782-46528909
chrX	46542082	46542161	chrX:46542072-46542116
chrX	46543587	46543806	chrX:46543577-46543777
chrX	46544512	46544652	chrX:46544500-46544611
chrX	46545382	46545490	chrX:46545370-46545457
chrX	47370593	47370764	chrX:47370582-47370740
chrX	47410278	47410441	chrX:47410275-47410402
chrX	47410693	47410806	chrX:47410679-47410775
chrX	47412378	47412806	chrX:47412368-47414305
chrX	47412808	47412885	chrX:47412368-47414305
chrX	47412888	47413194	chrX:47412368-47414305
chrX	47413223	47413380	chrX:47412368-47414305
chrX	47413388	47414007	chrX:47412368-47414305
chrX	47445918	47446017	chrX:47445878-47449474
chrX	47446018	47446126	chrX:47445878-47449474
chrX	47446148	47447871	chrX:47445878-47449474
chrX	47447873	47448366	chrX:47445878-47449474
chrX	47448378	47448606	chrX:47445878-47449474
chrX	47448628	47448707	chrX:47445878-47449474

chrX	47448708	47449504	chrX:47445878-47449474
chrX	47455933	47456043	chrX:47455920-47456016
chrX	47456273	47456465	chrX:47456271-47456438
chrX	47467323	47467786	chrX:47467323-47467760
chrX	47473463	47473668	chrX:47473451-47473651
chrX	47482468	47482956	chrX:47482458-47482946
chrX	47635573	47636544	chrX:47635520-47636927
chrX	47636558	47636816	chrX:47635520-47636927
chrX	47636818	47636960	chrX:47635520-47636927
chrX	47637013	47637127	chrX:47637012-47637114
chrX	47637753	47638215	chrX:47637750-47638182
chrX	47638898	47639352	chrX:47638894-47639338
chrX	47640273	47640477	chrX:47640266-47640466
chrX	47641233	47641494	chrX:47641231-47641475
chrX	47648898	47649381	chrX:47648886-47649367
chrX	47649923	47650030	chrX:47649920-47650026
chrX	47650423	47650636	chrX:47650422-47650604
chrX	48786563	48786664	chrX:48786553-48786645
chrX	48788338	48788551	chrX:48788315-48788515
chrX	48791098	48791354	chrX:48791090-48791329
chrX	48791843	48792236	chrX:48791843-48792221
chrX	48792328	48792506	chrX:48792322-48792468
chrX	48793173	48793321	chrX:48793171-48793297
chrX	48793793	48794326	chrX:48793792-48794311
chrX	49028738	49030636	chrX:49028725-49030601
chrX	49031398	49031587	chrX:49031396-49031544
chrX	49033473	49033577	chrX:49033464-49033540
chrX	49033733	49033806	chrX:49033725-49033782
chrX	49034138	49034292	chrX:49034133-49034251
chrX	49037743	49038140	chrX:49037738-49038114
chrX	49038198	49038453	chrX:49038196-49038442
chrX	49039108	49039440	chrX:49039106-49039410
chrX	49039743	49039827	chrX:49039734-49039803
chrX	49040468	49040607	chrX:49040454-49040568
chrX	49042153	49042366	chrX:49042139-49042339
chrX	49043113	49043292	chrX:49043110-49043486
chrX	49043308	49043464	chrX:49043110-49043486
chrX	49250688	49251141	chrX:49250676-49251765
chrX	49251148	49251263	chrX:49250676-49251765
chrX	49251278	49251801	chrX:49250676-49251765
chrX	49253133	49253241	chrX:49253125-49253202
chrX	49253918	49254107	chrX:49253916-49254067
chrX	49255433	49255561	chrX:49255428-49255509
chrX	49255728	49255843	chrX:49255714-49255802
chrX	49256753	49257038	chrX:49256750-49257012
chrX	49257438	49257579	chrX:49257426-49257565
chrX	49257663	49257783	chrX:49257663-49257768
chrX	49258303	49258557	chrX:49258295-49258527
chrX	49261028	49261229	chrX:49261017-49261217
chrX	49264663	49264844	chrX:49264660-49264826
chrX	55623404	55624433	chrX:55623399-55625325
chrX	55624444	55625361	chrX:55623399-55625325
chrX	56232436	56232752	chrX:56232436-56232745
chrX	56233051	56233372	chrX:56233046-56233341
chrX	56250241	56250344	chrX:56250230-56250304
chrX	56261386	56261605	chrX:56261383-56261583
chrX	56265186	56265781	chrX:56265179-56265744
chrX	56268921	56269000	chrX:56268912-56268991
chrX	56269391	56269533	chrX:56269377-56269489
chrX	56270181	56270333	chrX:56270181-56270321
chrX	56284326	56285171	chrX:56284312-56287889
chrX	56285186	56286139	chrX:56284312-56287889
chrX	56286441	56287897	chrX:56284312-56287889

chrX	67544041	67544740	chrX:67544031-67546762
chrX	67544751	67545312	chrX:67544031-67546762
chrX	67545406	67546520	chrX:67544031-67546762
chrX	67546551	67546795	chrX:67544031-67546762
chrX	67568896	67569047	chrX:67568888-67569022
chrX	67570616	67570831	chrX:67570613-67570813
chrX	67643266	67643441	chrX:67643255-67643407
chrX	67680726	67681037	chrX:67680719-67681003
chrX	67685941	67686153	chrX:67685940-67686126
chrX	67689566	67689766	chrX:67689555-67689763
chrX	67694676	67695743	chrX:67694672-67696075
chrX	67695836	67696105	chrX:67694672-67696075
chrX	67711411	67711720	chrX:67711401-67711689
chrX	67717486	67717664	chrX:67717477-67717622
chrX	67721836	67721986	chrX:67721832-67721963
chrX	67722831	67723015	chrX:67722826-67722984
chrX	67723686	67724011	chrX:67723685-67730619
chrX	67724021	67724260	chrX:67723685-67730619
chrX	67724381	67726746	chrX:67723685-67730619
chrX	67726751	67728180	chrX:67723685-67730619
chrX	67728181	67728563	chrX:67723685-67730619
chrX	67728621	67729984	chrX:67723685-67730619
chrX	67729996	67730167	chrX:67723685-67730619
chrX	67730191	67730633	chrX:67723685-67730619
chrX	71096207	71097011	chrX:71096196-71096981
chrX	71097677	71097900	chrX:71097673-71097873
chrX	71099312	71099494	chrX:71099307-71099453
chrX	71100697	71101777	chrX:71100683-71101740
chrX	71102077	71102433	chrX:71102076-71103535
chrX	71102457	71103536	chrX:71102076-71103535
chrX	73447255	73447798	chrX:73447253-73447755
chrX	73450965	73451182	chrX:73450960-73451160
chrX	73453525	73453699	chrX:73453516-73453662
chrX	73454390	73455261	chrX:73454378-73455245
chrX	80014756	80014915	chrX:80014755-80014887
chrX	80021001	80021219	chrX:80020998-80021198
chrX	80022241	80022457	chrX:80022241-80022444
chrX	80023061	80023316	chrX:80023059-80023303
chrX	80024076	80024187	chrX:80024062-80024164
chrX	80025631	80025804	chrX:80025602-80025777
chrX	80026706	80026891	chrX:80026703-80026868
chrX	80027266	80027339	chrX:80027255-80027320
chrX	80027991	80028094	chrX:80027990-80028076
chrX	80030511	80031658	chrX:80030497-80031769
chrX	80031676	80031769	chrX:80030497-80031769
chrX	81115090	81115266	chrX:81115080-81115230
chrX	81116215	81116325	chrX:81116203-81116303
chrX	81125010	81125084	chrX:81124999-81125199
chrX	81125090	81125229	chrX:81124999-81125199
chrX	83508255	83509460	chrX:83508249-83512127
chrX	83509715	83511074	chrX:83508249-83512127
chrX	83511120	83511199	chrX:83508249-83512127
chrX	83511210	83511522	chrX:83508249-83512127
chrX	83511540	83511637	chrX:83508249-83512127
chrX	83511700	83512104	chrX:83508249-83512127
chrX	83871475	83871577	chrX:83871470-83871570
chrX	83874545	83874643	chrX:83874531-83874631
chrX	83878555	83878664	chrX:83878425-83878625
chrX	83886565	83886734	chrX:83886551-83886699
chrX	85244035	85244110	chrX:85244031-85244191
chrX	85244155	85244235	chrX:85244031-85244191
chrX	85244765	85244919	chrX:85244764-85244887
chrX	85245945	85246046	chrX:85245902-85246022

chrX	85246930	85247674	chrX:85246929-85247651
chrX	85255260	85255837	chrX:85255258-85255801
chrX	85262600	85262773	chrX:85262541-85262741
chrX	85264275	85264464	chrX:85264274-85264430
chrX	85265130	85265267	chrX:85265117-85265255
chrX	85267285	85267432	chrX:85267277-85267415
chrX	85268305	85268384	chrX:85268293-85268341
chrX	85270005	85270180	chrX:85270002-85270146
chrX	85270655	85273382	chrX:85270650-85273362
chrX	86148460	86149134	chrX:86148457-86149108
chrX	86153965	86154190	chrX:86153963-86154163
chrX	86262975	86263048	chrX:86262967-86263011
chrX	86514275	86514436	chrX:86514273-86514391
chrX	86651035	86651187	chrX:86651035-86651167
chrX	86695020	86695212	chrX:86695020-86695179
chrX	86714560	86714745	chrX:86714547-86714720
chrX	86739750	86739908	chrX:86739746-86739882
chrX	86742645	86742747	chrX:86742642-86742726
chrX	86812855	86813068	chrX:86812855-86813034
chrX	86813140	86813307	chrX:86813129-86813277
chrX	86814700	86814869	chrX:86814687-86814834
chrX	86816035	86816115	chrX:86816033-86816099
chrX	86827775	86831493	chrX:86827765-86832604
chrX	86831510	86832080	chrX:86827765-86832604
chrX	86832095	86832444	chrX:86827765-86832604
chrX	86832455	86832625	chrX:86827765-86832604
chrX	89921885	89922901	chrX:89921881-89922883
chrX	103782386	103782603	chrX:103782375-103782575
chrX	103789336	103789410	chrX:103789332-103789398
chrX	103790526	103790711	chrX:103790526-103790676
chrX	104113151	104113267	chrX:104113146-104113246
chrX	104113446	104113610	chrX:104113433-104113583
chrX	104250046	104250336	chrX:104250037-104250896
chrX	104250496	104250928	chrX:104250037-104250896
chrX	104251601	104251776	chrX:104251544-104251744
chrX	104252781	104252859	chrX:104252782-104252828
chrX	104254161	104254613	chrX:104254153-104254577
chrX	104254776	104254959	chrX:104254767-104254933
chrX	118824617	118824734	chrX:118824613-118824713
chrX	118825142	118825320	chrX:118825130-118825280
chrX	119588407	119590023	chrX:119588336-119589977
chrX	119591242	119591341	chrX:119591233-119591309
chrX	119592392	119593158	chrX:119592382-119593150
chrX	119598737	119598949	chrX:119598734-119598934
chrX	119603472	119603576	chrX:119603461-119603559
chrX	119605737	119605914	chrX:119605730-119605895
chrX	120070672	120071377	chrX:120070671-120072845
chrX	120071392	120071811	chrX:120070671-120072845
chrX	120071822	120072058	chrX:120070671-120072845
chrX	120072087	120072860	chrX:120070671-120072845
chrX	120074187	120074404	chrX:120074174-120074374
chrX	120075902	120075983	chrX:120075899-120075945
chrX	120076882	120077306	chrX:120076876-120077288
chrX	120077477	120077699	chrX:120077473-120077705
chrX	120158597	120158857	chrX:120158560-120158829
chrX	120159017	120159454	chrX:120159014-120159426
chrX	120160357	120160434	chrX:120160357-120160403
chrX	120161897	120162106	chrX:120161892-120162092
chrX	120163457	120164195	chrX:120163457-120165630
chrX	120164212	120164480	chrX:120163457-120165630
chrX	120164487	120164905	chrX:120163457-120165630
chrX	120164932	120165669	chrX:120163457-120165630
chrX	120250752	120251002	chrX:120250751-120250977

chrX	120251727	120251938	chrX:120251723-120251923
chrX	120252672	120252777	chrX:120252668-120252770
chrX	120253427	120253984	chrX:120253413-120258398
chrX	120253997	120254382	chrX:120253413-120258398
chrX	120254407	120256790	chrX:120253413-120258398
chrX	120256797	120258082	chrX:120253413-120258398
chrX	120258102	120258413	chrX:120253413-120258398
chrX	123960222	123960322	chrX:123960211-123960288
chrX	123960532	123960605	chrX:123960518-123960942
chrX	123960642	123960960	chrX:123960518-123960942
chrX	123961322	123961457	chrX:123961313-123961435
chrX	123961717	123961870	chrX:123961717-123961856
chrX	123962037	123962144	chrX:123962032-123962106
chrX	123963182	123963436	chrX:123963169-123963401
chrX	124003712	124003777	chrX:124003466-124003747
chrX	124021367	124021434	chrX:124021366-124021431
chrX	124022542	124022678	chrX:124022530-124022671
chrX	124025852	124025945	chrX:124025839-124025918
chrX	124026577	124026704	chrX:124026564-124026687
chrX	124030967	124031143	chrX:124030960-124031125
chrX	124037527	124037635	chrX:124037526-124037623
chrX	124042572	124042678	chrX:124042568-124042645
chrX	124045167	124045385	chrX:124045163-124045368
chrX	124047362	124047534	chrX:124047353-124047505
chrX	124049017	124049115	chrX:124049004-124049078
chrX	124050187	124050345	chrX:124050185-124050309
chrX	124051122	124051221	chrX:124051120-124051219
chrX	124051322	124051429	chrX:124051314-124051394
chrX	124056127	124056274	chrX:124056127-124056235
chrX	124057867	124058003	chrX:124057865-124057977
chrX	124061237	124061370	chrX:124061223-124061341
chrX	124061772	124061886	chrX:124061770-124061874
chrX	124062912	124063009	chrX:124062901-124062994
chrX	124063127	124063223	chrX:124063115-124063205
chrX	124063852	124064063	chrX:124063847-124064051
chrX	124065877	124065959	chrX:124065875-124065946
chrX	124066187	124066289	chrX:124066174-124066262
chrX	124066367	124066464	chrX:124066355-124066436
chrX	124068572	124068677	chrX:124068563-124068656
chrX	124071152	124071340	chrX:124071148-124071323
chrX	124076332	124076483	chrX:124076331-124076471
chrX	124077967	124078079	chrX:124077956-124078058
chrX	124081392	124081571	chrX:124081379-124081528
chrX	124083422	124083575	chrX:124083420-124083549
chrX	124086557	124086797	chrX:124086546-124086770
chrX	124090577	124090796	chrX:124090574-124090764
chrX	124090862	124091008	chrX:124090853-124090964
chrX	124093922	124094161	chrX:124093912-124094144
chrX	124095372	124095524	chrX:124095371-124095495
chrX	124100577	124100865	chrX:124100573-124102656
chrX	124100892	124101476	chrX:124100573-124102656
chrX	124101492	124101639	chrX:124100573-124102656
chrX	124101647	124102685	chrX:124100573-124102656
chrX	124400907	124401134	chrX:124400907-124401107
chrX	124411827	124412120	chrX:124411821-124412090
chrX	130064881	130067542	chrX:130064873-130067525
chrX	130069301	130069689	chrX:130069299-130069677
chrX	130071051	130071264	chrX:130071039-130071232
chrX	130071346	130071451	chrX:130071335-130071419
chrX	130072226	130072444	chrX:130072225-130072417
chrX	130074056	130074162	chrX:130074048-130074141
chrX	130074591	130074764	chrX:130074580-130074752
chrX	130081256	130081578	chrX:130081255-130081539

chrX	130084816	130085037	chrX:130084804-130085004
chrX	130110326	130110511	chrX:130110324-130110495
chrX	130110631	130110734	chrX:130110619-130110716
chrX	130172231	130172331	chrX:130172220-130172320
chrX	130173761	130173979	chrX:130173758-130173958
chrX	130184296	130184469	chrX:130184284-130184434
chrX	133216668	133216775	chrX:133216668-133218348
chrX	133216778	133216892	chrX:133216668-133218348
chrX	133216983	133217633	chrX:133216668-133218348
chrX	133217678	133217989	chrX:133216668-133218348
chrX	133218003	133218379	chrX:133216668-133218348
chrX	134373263	134373499	chrX:134373252-134373467
chrX	134377578	134377798	chrX:134377571-134377755
chrX	134378008	134378124	chrX:134378004-134378106
chrX	134388298	134388409	chrX:134388287-134388487
chrX	134388413	134388509	chrX:134388287-134388487
chrX	134393508	134393648	chrX:134393500-134393634
chrX	134393908	134393981	chrX:134393908-134393952
chrX	134413498	134413668	chrX:134413487-134413657
chrX	134413828	134414001	chrX:134413822-134413966
chrX	134415023	134415302	chrX:134415015-134415292
chrX	134417178	134417574	chrX:134417168-134420323
chrX	134417873	134418812	chrX:134417168-134420323
chrX	134419173	134419245	chrX:134417168-134420323
chrX	134419373	134419929	chrX:134417168-134420323
chrX	134425208	134426574	chrX:134425200-134428791
chrX	134426583	134428086	chrX:134425200-134428791
chrX	134428133	134428825	chrX:134425200-134428791
chrX	137566142	137566457	chrX:137566141-137567751
chrX	137566462	137566840	chrX:137566141-137567751
chrX	137566842	137566954	chrX:137566141-137567751
chrX	137566972	137567768	chrX:137566141-137567751
chrX	137568817	137569098	chrX:137568804-137569065
chrX	137569897	137570100	chrX:137569890-137571585
chrX	137570107	137571613	chrX:137569890-137571585
chrX	137576292	137576504	chrX:137576290-137576490
chrX	137577122	137577709	chrX:137577121-137577691
chrX	140502987	140505145	chrX:140502984-140505116
chrX	151578930	151579094	chrX:151578886-151579086
chrX	151674090	151674201	chrX:151674086-151674186
chrX	151676000	151676183	chrX:151675996-151676146
chrX	153947561	153948478	chrX:153947552-153949386
chrX	153948561	153948730	chrX:153947552-153949386
chrX	153948746	153949196	chrX:153947552-153949386
chrX	153949206	153949414	chrX:153947552-153949386
chrX	153949561	153949638	chrX:153949552-153949616
chrX	153950251	153950577	chrX:153950242-153950543
chrX	153950816	153951032	chrX:153950812-153950998
chrX	153951361	153951495	chrX:153951349-153951487
chrX	153951591	153951736	chrX:153951588-153951707
chrX	153951851	153952198	chrX:153951840-153952161
chrX	153952516	153953120	chrX:153952513-153953090
chrX	153953616	153953789	chrX:153953606-153953770
chrX	153954066	153955441	chrX:153954065-153955542
chrX	153955461	153955570	chrX:153954065-153955542
chrX	153956201	153956441	chrX:153956190-153956411
chrX	153956631	153956780	chrX:153956624-153956763
chrX	153956931	153957011	chrX:153956917-153957060
chrX	153957021	153957101	chrX:153956917-153957060
chrX	153957321	153957568	chrX:153957313-153957533
chrX	153957791	153957896	chrX:153957781-153957886
chrX	153958036	153958296	chrX:153958024-153958249
chrX	153958576	153958808	chrX:153958568-153958766

chrX	153959336	153959512	chrX:153959330-153959491
chrX	153959801	153960188	chrX:153959801-153960161
chrX	153960241	153960455	chrX:153960234-153960414
chrX	153961546	153961686	chrX:153961541-153961648
chrX	153962221	153962339	chrX:153962221-153962306
chrX	153963226	153963452	chrX:153963224-153963433
chrX	153964131	153964310	chrX:153964123-153964284
chrX	153964581	153964762	chrX:153964577-153964726
chrX	153965376	153965584	chrX:153965372-153965572
chrX	153970651	153971848	chrX:153970647-153971807
chrX	154303891	154304117	chrX:154303890-154304090
chrX	154327861	154327985	chrX:154327858-154327958
chrX	154329528	154329706	chrX:154329515-154329665
chrY	2786864	2787734	chrY:2786854-2787699
chrY	2935294	2935463	chrY:2935280-2935446
chrY	2935479	2935809	chrY:2935476-2935769
chrY	2951194	2951393	chrY:2951183-2951383
chrY	2953909	2954027	chrY:2953908-2953997
chrY	2961074	2961677	chrY:2961073-2961646
chrY	2975104	2975285	chrY:2975094-2975244
chrY	2975524	2975665	chrY:2975510-2975654
chrY	2976679	2976855	chrY:2976669-2976822
chrY	2977823	2978110	chrY:2977818-2978080
chrY	2978813	2978887	chrY:2978809-2982506
chrY	2978903	2981346	chrY:2978809-2982506
chrY	2981348	2982442	chrY:2978809-2982506
chrY	2982468	2982536	chrY:2978809-2982506
