# Supplementary material for: Targeted RNA sequencing enhances gene expression profiling of ultra-low input samples
Source: RNA Biol. 2020 Jun 28;17(12):1741–53. doi: 10.1080/15476286.2020.1777768 (PMC7746246; doi:10.1080/15476286.2020.1777768)
Supplement: Supplemental Material [file KRNB_A_1777768_SM6200.zip › TableS4_TF_Capture_ERCC_SIRV_Sequin_targets.pdf]

FRAC_PROBE_COVERAGE is fraction of spike-in directly targeted by a probe.

FRAC_ESTIMATED_PROBE_COVERAGE is fraction of spike-in estimated to be directly and indirectly targeted by probes

Spike_in_target	FRAC_PROBE_COVERAGE	FRAC_ESTIMATED_PROBE_COVERAGE
ERCC_00004	0.93	1
ERCC_00012	0.98	1
ERCC_00014	0.98	1
ERCC_00019	0.93	1
ERCC_00022	0.98	1
ERCC_00024	0.91	1
ERCC_00028	0.99	1
ERCC_00031	0.97	1
ERCC_00034	0.98	1
ERCC_00035	0.96	1
ERCC_00041	0.98	1
ERCC_00042	0.96	1
ERCC_00043	0.97	1
ERCC_00044	0.97	1
ERCC_00046	0.96	1
ERCC_00048	0.99	1
ERCC_00051	0.88	1
ERCC_00057	0.96	1
ERCC_00058	0.97	1
ERCC_00059	0.95	1
ERCC_00069	0.98	1
ERCC_00071	0.96	1
ERCC_00078	0.98	1
ERCC_00083	0.96	1
ERCC_00084	0.97	1
ERCC_00085	0.95	1
ERCC_00086	0.97	1
ERCC_00092	0.97	1
ERCC_00098	0.98	1
ERCC_00099	0.98	1
ERCC_00104	0.99	1
ERCC_00108	0.97	1
ERCC_00109	0.9	1
ERCC_00111	0.97	1
ERCC_00117	0.99	1
ERCC_00120	0.95	1
ERCC_00123	0.97	1
ERCC_00131	0.96	1
ERCC_00134	0.91	1
ERCC_00137	0.98	1
ERCC_00138	0.97	1
ERCC_00143	0.95	1
ERCC_00144	0.93	1
ERCC_00145	0.98	1
ERCC_00147	0.91	1

ERCC_00148	0.96	1
ERCC_00150	0.95	1
ERCC_00156	0.95	1
ERCC_00157	0.96	1
ERCC_00158	0.99	1
ERCC_00160	0.95	1
ERCC_00164	0.96	1
ERCC_00165	0.98	1
ERCC_00168	0.97	1
ERCC_00170	0.95	1
ERCC_00171	0.96	1
SIRV1_1000_1484	1	1
SIRV1_6337_6813	0.98	1
SIRV1_7552_7814	1	1
SIRV1_10282_10366	0.93	1
SIRV1_10444_10791	1	1
SIRV1_10882_11242	0.98	1
SIRV1_11403_11643	0.95	1
SIRV2_1000_1661	0.98	1
SIRV2_1741_1853	0.97	1
SIRV2_1973_2064	0.88	1
SIRV2_2674_2802	0.9	1
SIRV2_2881_3010	1	1
SIRV2_3105_3374	1	1
SIRV2_3643_3825	0.95	1
SIRV2_3966_4479	0.98	1
SIRV2_4687_4800	1	1
SIRV2_5751_5911	0.93	1
SIRV3_1000_1167	0.92	1
SIRV3_1532_1764	0.97	1
SIRV3_1902_2005	0.96	1
SIRV3_4003_4080	0.9	1
SIRV3_4568_4779	0.98	1
SIRV3_6057_8292	1	1
SIRV3_8755_8975	0.95	1
SIRV3_9189_9324	0.9	1
SIRV3_9434_9943	0.97	1
SIRV4_1000_1346	1	1
SIRV4_1455_1885	0.97	1
SIRV4_2251_3403	0.99	1
SIRV4_3637_4103	0.97	1
SIRV4_5007_5158	0.9	1
SIRV4_8322_8372	1	1
SIRV4_8629_8990	0.93	1
SIRV4_13672_13937	1	1
SIRV4_14582_14633	0.98	1

SIRV4_15019_15122	0.92	1
SIRV5_1000_1149	0.86	1
SIRV5_1925_2488	0.98	1
SIRV5_3298_3404	1	1
SIRV5_3483_3643	1	1
SIRV5_5380_5450	1	1
SIRV5_5543_5626	0.86	1
SIRV5_6111_6169	1	1
SIRV5_6327_6452	1	1
SIRV5_6658_6722	0.84	1
SIRV5_6826_6957	0.92	1
SIRV5_7144_7307	0.99	1
SIRV5_7681_7762	0.83	1
SIRV5_7870_8585	0.98	1
SIRV5_10858_10991	1	1
SIRV5_11133_13606	1	1
SIRV6_1000_1186	0.98	1
SIRV6_1303_1381	0.82	1
SIRV6_1468_2120	1	1
SIRV6_2285_2620	1	1
SIRV6_2740_2828	0.93	1
SIRV6_3023_3164	0.9	1
SIRV6_7104_7448	0.96	1
SIRV6_7805_7923	0.91	1
SIRV6_8999_11837	1	1
SIRV7_1000_2675	0.99	1
SIRV7_2993_3111	0.79	1
SIRV7_3809_3896	1	1
SIRV7_4095_4179	0.86	1
SIRV7_4725_4810	1	1
SIRV7_43027_43078	1	1
SIRV7_55849_56097	1	1
SIRV7_70883_70987	0.96	1
SIRV7_78841_78963	0.94	1
SIRV7_114680_114988	0.95	1
SIRV7_147608_147957	0.96	1
chrIS_94350_94401	0.98	1
chrIS_95208_95292	0.88	1
chrIS_95607_96462	1	1
chrIS_144925_145141	0.92	1
chrIS_151619_151736	0.92	1
chrIS_234952_235060	0.87	1
chrIS_642800_642850	1	1
chrIS_834174_834288	0.86	1
chrIS_1082117_1082439	0.99	1
chrIS_1084006_1084129	0.95	1

chrIS_1091539_1091714	0.99	1
chrIS_1114691_1114840	0.95	1
chrIS_1122578_1122733	0.94	1
chrIS_1123184_1123408	0.98	1
chrIS_1124564_1124651	0.94	1
chrIS_1130611_1130733	0.91	1
chrIS_1138984_1139085	0.87	1
chrIS_1140597_1140725	0.91	1
chrIS_1141283_1141399	0.97	1
chrIS_1144270_1144452	0.92	1
chrIS_1146820_1146966	0.96	1
chrIS_1151783_1151957	0.92	1
chrIS_1153238_1153331	0.89	1
chrIS_1154783_1154900	0.86	1
chrIS_1156111_1156291	0.92	1
chrIS_1157345_1157423	0.97	1
chrIS_1159841_1159948	0.99	1
chrIS_1160764_1160971	1	1
chrIS_1163084_1163193	0.98	1
chrIS_1166112_1166229	0.91	1
chrIS_1171309_1171485	0.97	1
chrIS_1175695_1175779	0.88	1
chrIS_1177068_1177160	0.95	1
chrIS_1190799_1190849	1	1
chrIS_1191641_1191827	0.93	1
chrIS_1198654_1198734	0.9	1
chrIS_1239336_1239452	0.83	1
chrIS_1379268_1379391	0.92	1
chrIS_1580725_1580775	1	1
chrIS_1635426_1635480	1	1
chrIS_1635701_1635820	0.88	1
chrIS_1637937_1638309	1	1
chrIS_1638433_1638621	0.96	1
chrIS_1649105_1649178	0.81	1
chrIS_2012075_2012156	0	0
chrIS_2035092_2035142	1	1
chrIS_2082605_2082668	1	1
chrIS_2086805_2086875	0.9	1
chrIS_2116883_2116960	0.88	1
chrIS_2135118_2135293	1	1
chrIS_2136057_2136132	0.88	1
chrIS_2136396_2136489	0.87	1
chrIS_2190531_2190609	0.83	1
chrIS_2193901_2193970	0	0
chrIS_2199163_2199400	0.99	1
chrIS_2226971_2227517	0.99	1

chrIS_2238020_2238070	1	1
chrIS_2245193_2245253	1	1
chrIS_2252439_2252615	0.98	1
chrIS_2274605_2274720	0.96	1
chrIS_2283051_2283228	0.98	1
chrIS_2294426_2294602	1	1
chrIS_2298169_2298310	1	1
chrIS_2298808_2299117	1	1
chrIS_2304090_2304689	0.98	1
chrIS_2305167_2305532	1	1
chrIS_2319441_2319640	0.98	1
chrIS_2321085_2321205	1	1
chrIS_2330814_2330938	0.99	1
chrIS_2331966_2332170	0.97	1
chrIS_2332295_2332412	0.88	1
chrIS_2332638_2332719	0.98	1
chrIS_2334176_2334287	0.89	1
chrIS_2336306_2336369	0.81	1
chrIS_2340481_2340580	0.91	1
chrIS_2342821_2342890	0.87	1
chrIS_2349699_2350203	0.96	1
chrIS_2350846_2350923	0.92	1
chrIS_2352226_2352542	0.99	1
chrIS_2353030_2353197	0.96	1
chrIS_2353572_2353640	0.99	1
chrIS_2354656_2354936	0.97	1
chrIS_2379319_2380586	0.99	1
chrIS_2381452_2381737	0.99	1
chrIS_2382196_2382463	0.99	1
chrIS_2382758_2382911	0.89	1
chrIS_2386327_2386699	0.96	1
chrIS_2456887_2456938	0.98	1
chrIS_2466123_2466180	0.88	1
chrIS_2470905_2471007	1	1
chrIS_2483103_2483165	1	1
chrIS_2499186_2499350	0.81	1
chrIS_2504838_2505699	0.99	1
chrIS_2529478_2530101	0.98	1
chrIS_2541035_2541136	0.94	1
chrIS_2543832_2544003	0.99	1
chrIS_2545554_2545634	0.84	1
chrIS_2547607_2547796	0.95	1
chrIS_2553117_2553188	0.82	1
chrIS_2554763_2554890	0.99	1
chrIS_2555890_2556327	0.97	1
chrIS_2567183_2567269	0.93	1

chrIS_2567553_2567790	0.99	1
chrIS_2575076_2575188	0.84	1
chrIS_2599318_2599880	1	1
chrIS_2611160_2611257	0.86	1
chrIS_2614432_2614514	0.83	1
chrIS_2615222_2615379	1	1
chrIS_2619008_2619134	0.99	1
chrIS_2619410_2619581	0.92	1
chrIS_2619792_2619918	0.9	1
chrIS_2620506_2620727	0.99	1
chrIS_2620819_2621042	0.96	1
chrIS_2621526_2621634	0.87	1
chrIS_2621718_2621838	0.9	1
chrIS_2622259_2622471	0.99	1
chrIS_2623093_2623363	0.99	1
chrIS_2623685_2623909	0.93	1
chrIS_2707443_2707532	0.94	1
chrIS_2708687_2709121	0.99	1
chrIS_2936138_2936636	0.99	1
chrIS_2936765_2936858	0.95	1
chrIS_2936981_2937110	0.91	1
chrIS_2937522_2937665	1	1
chrIS_2937762_2937844	0.9	1
chrIS_2938014_2938111	0.86	1
chrIS_2938190_2938267	0.82	1
chrIS_2938361_2938432	0.82	1
chrIS_2938536_2938672	0.98	1
chrIS_2939121_2939299	0.98	1
chrIS_2972191_2972738	0.97	1
chrIS_2973595_2973658	0.95	1
chrIS_2975750_2976023	1	1
chrIS_2977329_2977650	0.97	1
chrIS_2986487_2986595	0.95	1
chrIS_3000315_3001061	0.99	1
chrIS_3002793_3003530	0.98	1
chrIS_3009696_3009837	0.96	1
chrIS_3009936_3010078	0.75	1
chrIS_3010186_3010353	0.93	1
chrIS_3010571_3010692	0.95	1
chrIS_3010778_3010926	0.99	1
chrIS_3011353_3011535	0.92	1
chrIS_3011837_3011972	1	1
chrIS_3012122_3012266	0.94	1
chrIS_3013625_3013706	0.88	1
chrIS_3014252_3014390	0.9	1
chrIS_3014542_3014639	0.97	1

chrIS_3017645_3017698	1	1
chrIS_3019315_3020018	0.98	1
chrIS_3020100_3020159	0.92	1
chrIS_3020306_3020396	1	1
chrIS_3020691_3020873	0.92	1
chrIS_3020962_3021399	0.98	1
chrIS_3253315_3253554	0.97	1
chrIS_3254620_3254694	1	1
chrIS_3309013_3309208	0.81	1
chrIS_3313966_3314193	0.95	1
chrIS_3332080_3332196	0.93	1
chrIS_3333673_3333831	0.91	1
chrIS_3335390_3335652	1	1
chrIS_3621203_3621284	0.86	1
chrIS_3625758_3625960	0.94	1
chrIS_3762243_3762414	0.99	1
chrIS_3765664_3765776	0.88	1
chrIS_3766592_3766770	0.99	1
chrIS_3767612_3767833	0.94	1
chrIS_3769405_3769575	0.93	1
chrIS_3777278_3777367	1	1
chrIS_3779731_3779805	0.86	1
chrIS_3784211_3784371	0.97	1
chrIS_3789500_3789560	0.88	1
chrIS_3792663_3792748	0.84	1
chrIS_3793972_3794118	0.9	1
chrIS_3930109_3930311	0.94	1
chrIS_3934002_3934172	0.99	1
chrIS_3934896_3934947	0.98	1
chrIS_3939679_3940292	0.98	1
chrIS_3964838_3965000	0.99	1
chrIS_3966323_3966404	0.86	1
chrIS_3966508_3966604	1	1
chrIS_3966712_3966830	0.99	1
chrIS_3968589_3968656	0.99	1
chrIS_3987946_3988081	0.93	1
chrIS_3990035_3990137	0.96	1
chrIS_3991835_3991972	0.97	1
chrIS_3992081_3992175	0.85	1
chrIS_3993287_3993417	1	1
chrIS_3994204_3994301	1	1
chrIS_3996987_3997068	0.89	1
chrIS_3998393_3998496	0.86	1
chrIS_4010232_4010293	1	1
chrIS_4016746_4016865	0.92	1
chrIS_4018335_4018470	0.94	1

chrIS_4018576_4018642	0.79	1
chrIS_4021961_4022056	0.99	1
chrIS_4022379_4022456	0.95	1
chrIS_4030203_4030333	0.99	1
chrIS_4049798_4050293	0.98	1
chrIS_4051177_4051338	0.99	1
chrIS_4054301_4054511	0.96	1
chrIS_4057808_4057886	0.86	1
chrIS_4100465_4100769	0.96	1
chrIS_4101674_4101751	0.91	1
chrIS_4101878_4102201	1	1
chrIS_4102307_4102463	0.93	1
chrIS_4102554_4102790	1	1
chrIS_4103042_4103157	0.86	1
chrIS_4104245_4104386	0.9	1
chrIS_4104525_4104724	0.97	1
chrIS_4105691_4105787	0.85	1
chrIS_4106581_4106724	0.92	1
chrIS_4107087_4107235	1	1
chrIS_4107318_4107442	0.9	1
chrIS_4107663_4107780	0.92	1
chrIS_4107881_4108025	0.9	1
chrIS_4108394_4108474	0.86	1
chrIS_4108923_4109001	0.82	1
chrIS_4109142_4109228	1	1
chrIS_4109344_4109395	0.98	1
chrIS_4109478_4109592	0.98	1
chrIS_4109758_4109891	1	1
chrIS_4111656_4111738	0.87	1
chrIS_4111931_4112006	0.83	1
chrIS_4116809_4116864	0.91	1
chrIS_4117581_4117680	0.91	1
chrIS_4119182_4119261	0.86	1
chrIS_4119340_4119390	1	1
chrIS_4123436_4123589	0.91	1
chrIS_4124907_4124973	0.82	1
chrIS_4129975_4130060	1	1
chrIS_4131449_4131525	0.83	1
chrIS_4134639_4134700	1	1
chrIS_4134901_4134984	0.89	1
chrIS_4135586_4135680	0.85	1
chrIS_4136033_4136230	0.99	1
chrIS_4138023_4138296	1	1
chrIS_4139062_4139266	1	1
chrIS_4144390_4145563	0.99	1
chrIS_4145915_4146273	1	1

chrIS_4147371_4147520	0.94	1
chrIS_4147746_4148312	0.99	1
chrIS_4159998_4160177	0.99	1
chrIS_4160322_4160510	1	1
chrIS_4163915_4164055	0.97	1
chrIS_4177666_4177926	0.99	1
chrIS_4180707_4180779	1	1
chrIS_4189915_4190014	0.86	1
chrIS_4191517_4191584	0.94	1
chrIS_4191998_4192102	1	1
chrIS_4203624_4204060	0.99	1
chrIS_4207145_4207258	0.96	1
chrIS_4215843_4215915	0.94	1
chrIS_4217882_4217996	0.94	1
chrIS_4221460_4221556	0.93	1
chrIS_4226430_4226604	0.98	1
chrIS_4231197_4231356	0.95	1
chrIS_4234905_4235013	0.99	1
chrIS_4237665_4237725	0.98	1
chrIS_4238706_4239513	1	1
chrIS_4304595_4304946	0.96	1
chrIS_4306873_4307088	1	1
chrIS_4323096_4323157	1	1
chrIS_4326810_4326923	0.9	1
chrIS_4328120_4328283	1	1
chrIS_4330276_4330442	0.92	1
chrIS_4331573_4331708	0.9	1
chrIS_4332767_4332940	0.97	1
chrIS_4340862_4341036	0.93	1
chrIS_4342635_4342812	0.96	1
chrIS_4343177_4343389	0.95	1
chrIS_4343481_4343602	0.9	1
chrIS_4343695_4343877	0.9	1
chrIS_4350009_4350798	0.99	1
chrIS_4351662_4352444	0.98	1
chrIS_4386849_4387303	0.99	1
chrIS_4485879_4486727	0.99	1
chrIS_4486808_4486858	1	1
chrIS_4487689_4487813	0.98	1
chrIS_4488078_4488128	1	1
chrIS_4488225_4488415	1	1
chrIS_4488529_4488680	0.88	1
chrIS_4488764_4488888	0.96	1
chrIS_4489990_4490417	0.97	1
chrIS_4492605_4493278	0.98	1
chrIS_4494298_4494362	1	1

chrIS_4496107_4496432	0.96	1
chrIS_4496970_4497051	0.91	1
chrIS_4554813_4555021	0.96	1
chrIS_4771416_4771602	0.87	1
chrIS_4772277_4772373	1	1
chrIS_4777703_4778235	0.99	1
chrIS_4967074_4967140	0.83	1
chrIS_5022066_5022141	0.83	1
chrIS_5042004_5042122	0.91	1
chrIS_5048701_5048829	0.92	1
chrIS_5072791_5072920	1	1
chrIS_5093658_5093837	0.94	1
chrIS_5096116_5096282	1	1
chrIS_5116019_5116111	0.98	1
chrIS_5144857_5145117	1	1
chrIS_5187407_5187504	0.86	1
chrIS_5187657_5187792	1	1
chrIS_5188651_5188949	1	1
chrIS_5189005_5189463	0.97	1
chrIS_5192002_5192222	0.94	1
chrIS_5193920_5194047	1	1
chrIS_5196624_5196805	1	1
chrIS_5200617_5200689	0.82	1
chrIS_5203024_5203269	0.94	1
chrIS_5203540_5203913	1	1
chrIS_5289117_5289365	0.97	1
chrIS_5290387_5290451	1	1
chrIS_5296917_5297057	0.7	1
chrIS_5298508_5298643	0.9	1
chrIS_5301020_5301254	1	1
chrIS_5301965_5302130	0.92	1
chrIS_5304722_5304997	1	1
chrIS_5317753_5317814	0.97	1
chrIS_5318328_5318446	0.88	1
chrIS_5323605_5323722	0.98	1
chrIS_5328168_5328311	0.98	1
chrIS_5331753_5331870	0.99	1
chrIS_5332996_5333614	1	1
chrIS_5333793_5334003	0.94	1
chrIS_5343228_5343343	0.88	1
chrIS_5345474_5345582	0.91	1
chrIS_5354332_5354811	0.97	1
chrIS_5389912_5392534	1	1
chrIS_5447619_5447694	0.81	1
chrIS_5469045_5469171	0.94	1
chrIS_5473380_5473535	1	1

chrIS_5473657_5473833	0.92	1
chrIS_5476673_5476746	0.81	1
chrIS_5481390_5481500	0.89	1
chrIS_5481620_5481818	1	1
chrIS_5486379_5487896	0.99	1
chrIS_5503296_5503538	0.95	1
chrIS_5510798_5510916	0.88	1
chrIS_5513043_5513468	0.98	1
chrIS_5517507_5517677	0.99	1
chrIS_5519455_5519558	0.86	1
chrIS_5520522_5520710	0.94	1
chrIS_5522852_5523122	0.99	1
chrIS_5523400_5523590	0.94	1
chrIS_5529237_5529326	0.88	1
chrIS_5556300_5556512	0.72	1
chrIS_5561111_5561383	0.95	1
chrIS_5569536_5570470	0.99	1
chrIS_5582250_5582320	0.81	1
chrIS_5583159_5583473	0.96	1
chrIS_5584604_5584749	0.92	1
chrIS_5584851_5585061	1	1
chrIS_5586795_5586898	0.93	1
chrIS_5587732_5587947	0.96	1
chrIS_5589006_5589079	0.96	1
chrIS_5589543_5589749	0.87	1
chrIS_5591063_5591115	1	1
chrIS_5594307_5594471	0.97	1
chrIS_5595512_5595564	0.98	1
chrIS_5596214_5596387	0.92	1
chrIS_5597093_5597145	1	1
chrIS_5597760_5597933	0.92	1
chrIS_5598647_5598699	0.98	1
chrIS_5599306_5601169	0.99	1
chrIS_5972393_5973338	1	1
chrIS_6108674_6108937	0.95	1
chrIS_6109104_6109199	0.92	1
chrIS_6109364_6109499	0.95	1
chrIS_6109635_6109751	0.84	1
chrIS_6110259_6110392	0.97	1
chrIS_6110696_6110841	0.87	1
chrIS_6111257_6111448	0.93	1
chrIS_6111924_6111985	0.82	1
chrIS_6112170_6112289	0.82	1
chrIS_6112673_6112784	0.86	1
chrIS_6112884_6113034	0.98	1
chrIS_6113167_6113218	1	1

chrIS_6113298_6113483	0.92	1
chrIS_6119113_6119382	0.99	1
chrIS_6165873_6166537	1	1
chrIS_6179427_6179540	1	1
chrIS_6181033_6181145	0.98	1
chrIS_6181713_6181764	0.98	1
chrIS_6184928_6185058	0.98	1
chrIS_6188138_6188272	0.96	1
chrIS_6209805_6209984	0.98	1
chrIS_6211256_6211352	1	1
chrIS_6223312_6223572	0.97	1
chrIS_6226646_6227344	1	1
chrIS_6227384_6227908	0.98	1
chrIS_6259933_6260193	0.95	1
chrIS_6260790_6260904	0.71	1
chrIS_6261642_6261779	0.9	1
chrIS_6262593_6262818	1	1
chrIS_6262874_6262934	1	1
chrIS_6264075_6264138	0.81	1
chrIS_6264761_6264897	0.91	1
chrIS_6266512_6266650	0.95	1
chrIS_6266759_6266900	0.98	1
chrIS_6269229_6269301	0.81	1
chrIS_6278661_6278757	1	1
chrIS_6278846_6278896	1	1
chrIS_6279378_6279436	1	1
chrIS_6279586_6279734	0.89	1
chrIS_6281864_6282267	0.97	1
chrIS_6283114_6283215	0.87	1
chrIS_6285939_6286217	0.96	1
chrIS_6298771_6298908	0.9	1
chrIS_6301790_6302048	0.98	1
chrIS_6303437_6303511	0	0
chrIS_6304032_6304117	1	1
chrIS_6309621_6309684	0.98	1
chrIS_6311240_6311425	0.89	1
chrIS_6311516_6311572	0.91	1
chrIS_6314656_6314769	0.88	1
chrIS_6319171_6319249	0.82	1
chrIS_6321933_6322146	0.94	1
chrIS_6327071_6327495	0.95	1
chrIS_6328885_6329009	0.9	1
chrIS_6335369_6335504	0.9	1
chrIS_6336490_6336638	0.98	1
chrIS_6338548_6338719	0.96	1
chrIS_6343646_6343756	0.98	1

chrIS_6344958_6345028	0.96	1
chrIS_6347512_6347649	0.92	1
chrIS_6350708_6350774	0.88	1
chrIS_6352149_6352251	0.89	1
chrIS_6358963_6359141	0.92	1
chrIS_6387264_6387358	1	1
chrIS_6412428_6412927	0.98	1
chrIS_6413537_6413588	1	1
chrIS_6415167_6415217	1	1
chrIS_6419955_6420058	0.99	1
chrIS_6426901_6426961	1	1
chrIS_6428960_6429109	0.98	1
chrIS_6433335_6433481	1	1
chrIS_6437392_6437487	0.87	1
chrIS_6440045_6440150	0.93	1
chrIS_6441350_6441438	0.99	1
chrIS_6446047_6446148	0.94	1
chrIS_6447833_6447908	0.81	1
chrIS_6448131_6448285	0.86	1
chrIS_6448937_6449236	1	1
chrIS_6449849_6450090	0.75	1
chrIS_6450753_6451743	0.99	1
chrIS_6471917_6472391	1	1
chrIS_6474670_6474800	0.91	1
chrIS_6476582_6476686	0.88	1
chrIS_6478335_6478551	0.94	1
chrIS_6495392_6496217	1	1
chrIS_6535368_6535835	0.98	1
chrIS_6539108_6539232	0.93	1
chrIS_6539915_6539965	1	1
chrIS_6545613_6545680	0.97	1
chrIS_6547531_6547776	1	1
chrIS_6555180_6555589	0.99	1
chrIS_6556643_6557165	0.99	1
chrIS_6583066_6583237	1	1
chrIS_6592651_6592829	0.93	1
chrIS_6593719_6593848	0.89	1
chrIS_6594510_6594561	0.98	1
chrIS_6596381_6596431	1	1
chrIS_6596553_6596603	1	1
chrIS_6596841_6596963	0.98	1
chrIS_6597068_6597189	0.93	1
chrIS_6597349_6597486	0.99	1
chrIS_6597818_6597899	0.83	1
chrIS_6598021_6598199	0.98	1
chrIS_6598379_6598474	0.86	1

chrIS_6599007_6599070	0.94	1
chrIS_6599205_6599301	0.92	1
chrIS_6599498_6599634	0.9	1
chrIS_6600665_6600778	0.87	1
chrIS_6600918_6601316	0.99	1
chrIS_6655220_6656127	0.99	1
chrIS_6662715_6662859	0.97	1
chrIS_6696454_6700868	1	1
chrIS_6706097_6706208	0.96	1
chrIS_6729798_6729925	0.89	1
chrIS_6737128_6737281	0.84	1
chrIS_6746403_6746507	0.93	1
chrIS_6748168_6748286	0.83	1
chrIS_6763671_6763745	0.81	1
chrIS_6773640_6774431	0.98	1
chrIS_6778985_6779046	0.89	1
chrIS_6790135_6790296	1	1
chrIS_6794367_6794699	1	1
chrIS_6798855_6798958	0.91	1
chrIS_6799684_6799856	0.97	1
chrIS_6802313_6802437	0.89	1
chrIS_6807647_6808198	0.97	1
chrIS_6822105_6822297	1	1
chrIS_6829318_6829759	1	1
chrIS_6929221_6929838	0.99	1
chrIS_6931093_6931237	0.91	1
chrIS_6931598_6931697	0.99	1
chrIS_6932695_6932875	1	1
chrIS_6937949_6938060	0.94	1
chrIS_6940455_6940632	0.92	1
chrIS_6940721_6940840	0.95	1
chrIS_6942256_6942456	0.93	1
chrIS_6942568_6942661	0.9	1
chrIS_6949423_6949776	0.96	1
chrIS_6950162_6950307	0.9	1
chrIS_6951407_6951607	0.94	1
chrIS_6955486_6955729	0.99	1
chrIS_6959789_6960050	0.95	1
chrIS_6960383_6960499	0.9	1
chrIS_6962456_6962816	1	1
chrIS_7337725_7337841	0.96	1
chrIS_7338171_7338269	0.87	1
chrIS_7338447_7338599	0.88	1
chrIS_7339899_7340079	0.89	1
chrIS_7341856_7341936	0.82	1
chrIS_7342677_7342767	0.92	1

chrIS_7342993_7343057	0.91	1
chrIS_7343794_7343915	0.88	1
chrIS_7346929_7347003	1	1
chrIS_7348133_7348268	0.93	1
chrIS_7445531_7445906	0.98	1
chrIS_7549499_7550494	1	1
chrIS_7625867_7626425	1	1
chrIS_7635628_7635775	1	1
chrIS_7636006_7636153	0.9	1
chrIS_7656829_7657013	0.96	1
chrIS_7657290_7657390	0.96	1
chrIS_7658259_7658322	0.86	1
chrIS_7662079_7662174	0.97	1
chrIS_7718245_7718338	0.86	1
chrIS_7741619_7741670	1	1
chrIS_7764407_7764457	1	1
chrIS_7820132_7820325	0.94	1
chrIS_7848864_7849011	0.93	1
chrIS_7853954_7854138	0.96	1
chrIS_7900736_7900907	1	1
chrIS_7903352_7903793	0.97	1
chrIS_7905439_7905585	0.92	1
chrIS_7908465_7908639	0.98	1
chrIS_7911537_7911651	0.8	1
chrIS_7914928_7915248	0.96	1
chrIS_8045965_8046873	0.99	1
chrIS_8057728_8058532	1	1
chrIS_8062352_8062433	0.83	1
chrIS_8062956_8063025	0.8	1
chrIS_8064889_8065442	0.99	1
chrIS_8065890_8066036	0.95	1
chrIS_8071624_8071759	0.9	1
chrIS_8072775_8072867	0.99	1
chrIS_8080502_8081234	0.98	1
chrIS_8123647_8124022	0.98	1
chrIS_8127433_8127676	1	1
chrIS_8130039_8130231	0.93	1
chrIS_8141772_8141885	1	1
chrIS_8143042_8143225	0.99	1
chrIS_8148301_8148423	0.91	1
chrIS_8150428_8150600	0.99	1
chrIS_8164483_8164613	0.98	1
chrIS_8167698_8168114	1	1
chrIS_8168354_8168506	1	1
chrIS_8169141_8169317	0.97	1
chrIS_8169654_8170561	1	1

chrIS_8176595_8176706	0.79	1
chrIS_8211993_8212093	0.86	1
chrIS_8276227_8276315	0.93	1
chrIS_8300036_8300148	1	1
chrIS_8361097_8361247	0.95	1
chrIS_8365334_8366421	1	1
chrIS_8368031_8368106	0.91	1
chrIS_8373434_8373581	0.97	1
chrIS_8379851_8380113	0.96	1
chrIS_8382717_8382857	0.94	1
chrIS_8390408_8390459	0.98	1
chrIS_8390552_8390635	0.88	1
chrIS_8392317_8392453	0.94	1
chrIS_8392874_8392972	0.91	1
chrIS_8397649_8397729	0.82	1
chrIS_8400682_8401345	0.99	1
chrIS_8460727_8461954	0.99	1
chrIS_8483368_8483608	0.96	1
chrIS_8484114_8484280	0.95	1
chrIS_8484443_8484567	0.9	1
chrIS_8485068_8485123	0.91	1
chrIS_8499332_8499650	0.97	1
chrIS_8531300_8531471	0.99	1
chrIS_8531860_8531939	0.84	1
chrIS_8532087_8532870	0.99	1
chrIS_8623258_8623397	1	1
chrIS_8623842_8624065	0.95	1
chrIS_8624983_8625071	0.88	1
chrIS_8626141_8626346	0.97	1
chrIS_8626454_8626515	1	1
chrIS_8627310_8627558	0.97	1
chrIS_8815650_8816170	0.97	1
chrIS_8823107_8823225	0.88	1
chrIS_8823558_8823639	0.83	1
chrIS_8825658_8825741	0.87	1
chrIS_8826539_8826627	1	1
chrIS_8831062_8831143	0.88	1
chrIS_8833123_8833230	0.89	1
chrIS_8847460_8847643	1	1
chrIS_8850130_8850180	1	1
chrIS_8851141_8851291	0.92	1
chrIS_8854705_8854880	0.97	1
chrIS_8855127_8855225	0.86	1
chrIS_8857002_8857155	0.95	1
chrIS_8857738_8857825	1	1
chrIS_8860165_8860385	0.98	1

chrIS_8863154_8863228	0.81	1
chrIS_8866824_8866875	0.98	1
chrIS_8867445_8867550	0.87	1
chrIS_8869786_8869951	0.98	1
chrIS_8871547_8871738	1	1
chrIS_8890605_8890752	0.97	1
chrIS_8919974_8920058	0.88	1
chrIS_8945051_8945161	0.88	1
chrIS_8964884_8964944	0.97	1
chrIS_8969730_8969780	1	1
chrIS_8971398_8971455	1	1
chrIS_8971857_8971971	0.96	1
chrIS_8980094_8980194	1	1
chrIS_8982284_8982364	0.96	1
chrIS_8982984_8983034	1	1
chrIS_8983510_8983605	0.99	1
chrIS_8983740_8983826	1	1
chrIS_8984900_8985002	0.94	1
chrIS_8985752_8985992	0.94	1
chrIS_8988587_8988671	0.92	1
chrIS_8989589_8989639	1	1
chrIS_8990763_8990859	1	1
chrIS_8992832_8992928	1	1
chrIS_8996305_8996408	0.86	1
chrIS_8996904_8996987	0.86	1
chrIS_8999689_8999740	0.98	1
chrIS_8999828_8999900	0.96	1
chrIS_9004519_9004618	1	1
chrIS_9007466_9007571	0.96	1
chrIS_9008298_9008391	0.85	1
chrIS_9009680_9009731	1	1
chrIS_9010152_9010245	0.88	1
chrIS_9012776_9012847	0.97	1
chrIS_9013888_9013949	1	1
chrIS_9014886_9015138	0.96	1
chrIS_9034678_9035211	0.99	1
chrIS_9035493_9035600	1	1
chrIS_9035683_9035739	0.89	1
chrIS_9036039_9036233	1	1
chrIS_9044917_9045040	1	1
chrIS_9048028_9048093	0.78	1
chrIS_9133607_9133746	1	1
chrIS_9192124_9192188	0.8	1
chrIS_9225302_9225549	0.94	1
chrIS_9270062_9270302	0.95	1
chrIS_9273055_9273282	1	1

chrIS_9283532_9283613	0.91	1
chrIS_9284467_9284862	0.99	1
chrIS_9285484_9285613	0.98	1
chrIS_9287405_9287869	0.98	1
chrIS_9353175_9354087	0.98	1
chrIS_9354309_9354398	1	1
chrIS_9407564_9407740	0.91	1
chrIS_9412302_9412378	0.91	1
chrIS_9552545_9552605	1	1
chrIS_9557472_9557802	0.97	1
chrIS_9559325_9559449	0.99	1
chrIS_9579656_9579865	0.99	1
chrIS_9583447_9583593	1	1
chrIS_9585659_9585804	0.91	1
chrIS_9588391_9588682	0.93	1
chrIS_9588842_9588960	0.91	1
chrIS_9592037_9592237	0.99	1
chrIS_9595752_9595901	0.96	1
chrIS_9596946_9597106	0.95	1
chrIS_9597213_9597296	0.84	1
chrIS_9603007_9603224	1	1
chrIS_9604963_9605146	0.99	1
chrIS_9606927_9607023	0.85	1
chrIS_9608352_9608458	1	1
chrIS_9609905_9609981	0.96	1
chrIS_9613121_9613171	1	1
chrIS_9623190_9624102	0.98	1
chrIS_9715006_9715057	1	1
chrIS_9748907_9749074	0.92	1
chrIS_9753629_9753834	0.93	1
chrIS_9760856_9761013	0.99	1
chrIS_9763219_9763356	0.9	1
chrIS_9767307_9767573	1	1
chrIS_9805054_9805525	0.97	1
chrIS_9808902_9809098	0.99	1
chrIS_9811221_9811301	0.84	1
chrIS_9811592_9811824	1	1
chrIS_9903250_9903595	1	1
chrIS_9904339_9904481	0.92	1
chrIS_9904568_9904688	0.93	1
chrIS_9905129_9905315	0.94	1
chrIS_9905698_9905822	0.91	1
chrIS_9906002_9906127	0.98	1
chrIS_9908372_9908538	0.92	1
chrIS_9909211_9909268	0.89	1
chrIS_9911416_9911618	0.94	1

chrIS_9912079_9912129	1	1
chrIS_9913315_9913408	1	1
chrIS_9932857_9932946	1	1
chrIS_9941035_9941390	0.96	1
chrIS_9952142_9952284	0.98	1
chrIS_9955925_9956054	1	1
chrIS_9958958_9959092	0.96	1
chrIS_9960731_9960824	0.85	1
chrIS_9964815_9964866	0	0
chrIS_9971020_9971110	0.98	1
chrIS_9971497_9971598	0.96	1
chrIS_9978626_9978855	0.94	1
chrIS_9979546_9979807	0.97	1
chrIS_10217767_10218434	1	1
chrIS_10219555_10219627	0.85	1
chrIS_10220285_10220346	0.95	1
chrIS_10221077_10221133	0.89	1
chrIS_10223138_10223189	0.98	1
chrIS_10224651_10224711	0.83	1
chrIS_10227006_10227100	0.94	1
chrIS_10227795_10227881	0.84	1
chrIS_10228692_10228794	0.97	1
chrIS_10229347_10229519	0.93	1
chrIS_10229799_10229908	0.96	1
chrIS_10230358_10230421	0.98	1
chrIS_10233077_10233186	0.87	1
chrIS_10375811_10376263	1	1
chrIS_10379459_10379582	0.98	1
chrIS_10380834_10380897	0.79	1
chrIS_10382493_10382664	0.94	1
chrIS_10387047_10387195	0.88	1
chrIS_10388366_10389391	1	1
chrIS_10389976_10390029	0.98	1
chrIS_10390347_10390403	1	1
chrIS_10391263_10391944	0.98	1
chrIS_10394052_10394934	0.98	1
