# Supplementary material for: Targeted RNA sequencing enhances gene expression profiling of ultra-low input samples
Source: RNA Biol. 2020 Jun 28;17(12):1741–53. doi: 10.1080/15476286.2020.1777768 (PMC7746246; doi:10.1080/15476286.2020.1777768)
Supplement: Supplemental Material [file KRNB_A_1777768_SM6200.zip › TableS5_RawReadFastq.pdf]

Bulks

Collective.SampleID	idx	RawReads.Pre-Capture	RawReads.Post-Capture.NF Panel	RawReads.Post-Capture.TF Panel
CON_1_A_FP+25_LY_UOXZ-0	277	36207415	8500817	9718155
CON_1_A_FP+55_LY_UOXZ-0	282	27671566	8225844	8367110
CON_2_A_FP+25_LY_UOXZ-0	278	31538986	8128323	9397533
CON_2_A_FP+55_LY_UOXZ-0	283	19204479	6263470	5935660
CON_3_A_FP+25_LY_UOXZ-0	279	35265490	7825754	9480992
CON_3_A_FP+55_LY_UOXZ-0	284	31229456	8725069	9106667
PS1_1_A_FP+25_LY_UOXZ-0	280	34963928	7913769	10445146
PS1_1_A_FP+55_LY_UOXZ-0	285	24757688	7248225	7996664
PS1_2_A_FP+25_LY_UOXZ-0	281	31629302	9184925	10035714
PS1_2_A_FP+55_LY_UOXZ-0	286	31353171	7866397	8873935
CON_1_A_FP+25_LY_UOXC-0	265	28406253	8831389	9113147
CON_1_A_FP+55_LY_UOXC-0	271	25041012	7245583	6655244
CON_2_A_FP+25_LY_UOXC-0	266	27641114	8153219	8612721
CON_2_A_FP+55_LY_UOXC-0	272	33924571	8924420	8375111
CON_3_A_FP+25_LY_UOXC-0	267	29652386	7415382	7463009
CON_3_A_FP+55_LY_UOXC-0	273	37557109	12454461	10963700
PS1_1_A_FP+25_LY_UOXC-0	268	32668656	7856882	8709108
PS1_1_A_FP+55_LY_UOXC-0	274	30715805	9091580	8620367
PS1_2_A_FP+25_LY_UOXC-0	269	34623910	8333572	8437298
PS1_2_A_FP+55_LY_UOXC-0	275	25009365	9347129	8943278
PS1_3_A_FP+25_LY_UOXC-0	270	33869990	7587111	8403762
PS1_3_A_FP+55_LY_UOXC-0	276	27049621	7728347	6989640
CON_1_A_FP+25_LY_UCAM-0	253	20386493	7116435	7069877
CON_1_A_FP+55_LY_UCAM-0	259	16599088	5579268	5315552
CON_2_A_FP+25_LY_UCAM-0	254	23791478	6899623	7040031
CON_2_A_FP+55_LY_UCAM-0	260	32243124	7779752	7607379
CON_3_A_FP+25_LY_UCAM-0	255	24082582	6223361	6191394
CON_3_A_FP+55_LY_UCAM-0	261	18871353	5917281	5772809
PS1_1_A_FP+25_LY_UCAM-0	256	25548855	5776228	5942195
PS1_1_A_FP+55_LY_UCAM-0	262	20695349	4411374	4782030
PS1_2_A_FP+25_LY_UCAM-0	257	25652440	6526471	6868744
PS1_2_A_FP+55_LY_UCAM-0	263	22865978	6561077	6754086
PS1_3_A_FP+25_LY_UCAM-0	258	21182380	5783111	5721559
PS1_3_A_FP+55_LY_UCAM-0	264	23146135	5075266	4907433
CON_1_A_FP+25_LY_JANS-0	241	18109539	5151499	4577461
CON_1_A_FP+55_LY_JANS-0	247	14735921	5378423	4626424
CON_2_A_FP+25_LY_JANS-0	242	18563357	4929623	4513895
CON_2_A_FP+55_LY_JANS-0	248	18676366	5675919	4668006
CON_3_A_FP+25_LY_JANS-0	243	14629140	5522543	4863431
CON_3_A_FP+55_LY_JANS-0	249	20605013	6389124	5232026
PS1_1_A_FP+25_LY_JANS-0	244	16055128	4671407	4244033
PS1_1_A_FP+55_LY_JANS-0	250	18939608	4808218	4911342
PS1_2_A_FP+25_LY_JANS-0	245	15976576	4578073	4184141
PS1_2_A_FP+55_LY_JANS-0	251	18792376	5830322	5588807
PS1_3_A_FP+25_LY_JANS-0	246	21326223	6079711	5486511
PS1_3_A_FP+55_LY_JANS-0	252	19236636	6337440	5370766
CON_1_A_FP+25_LY_ABBV-0	229	13017267	3999160	3306730
CON_1_A_FP+55_LY_ABBV-0	235	13626405	4845032	3729684
CON_2_A_FP+25_LY_ABBV-0	230	14542258	3325406	2903075
CON_2_A_FP+55_LY_ABBV-0	236	15653328	3984839	3514180
CON_3_A_FP+25_LY_ABBV-0	231	15439798	3217934	3454016
CON_3_A_FP+55_LY_ABBV-0	237	15087597	4698349	4041005
PS1_1_A_FP+25_LY_ABBV-0	232	13701214	3745996	3432457

PS1_1_A_FP+55_LY_ABBV-0	238	14744494	4982662	4323683
PS1_2_A_FP+25_LY_ABBV-0	233	15137280	4886583	4342444
PS1_2_A_FP+55_LY_ABBV-0	239	18851116	5754440	4705317
PS1_3_A_FP+25_LY_ABBV-0	234	13774086	3807551	3293684
PS1_3_A_FP+55_LY_ABBV-0	240	20057736	6278151	5336113

Mini-bulks

idx	RawReads.Pre-Cap	RawReads.Post-Cap 850ng	Panel	Raw.reads.Post-Cap 150ng	Panel_150ng
227256	984218	1388206	NFcapture_850ng	NA	
227280	917614	1189459	NFcapture_850ng	NA	
228256	1033218	1552013	NFcapture_850ng	NA	
228280	872678	1151244	NFcapture_850ng	NA	
255256	1061998	1313130	NFcapture_850ng	NA	
255280	898342	1075954	NFcapture_850ng	NA	
256256	1048659	1346513	NFcapture_850ng	NA	
256280	1011816	1205846	NFcapture_850ng	NA	
227208	887120	1762347	TFcapture_850ng	1573893	TFcapture_150ng
227232	814233	1522308	TFcapture_850ng	1375514	TFcapture_150ng
228208	878375	1901931	TFcapture_850ng	1709439	TFcapture_150ng
228232	785458	1462990	TFcapture_850ng	1311566	TFcapture_150ng
255208	1093996	317606	TFcapture_850ng	377105	TFcapture_150ng
255232	597677	1116437	TFcapture_850ng	1027126	TFcapture_150ng
256208	721697	351965	TFcapture_850ng	382883	TFcapture_150ng
256232	639240	1400749	TFcapture_850ng	1277482	TFcapture_150ng
