# Supplementary material for: Targeted RNA sequencing enhances gene expression profiling of ultra-low input samples
Source: RNA Biol. 2020 Jun 28;17(12):1741–53. doi: 10.1080/15476286.2020.1777768 (PMC7746246; doi:10.1080/15476286.2020.1777768)
Supplement: Supplemental Material [file KRNB_A_1777768_SM6200.zip › TableS6_Usable_reads_dedup_deMM.pdf]

Bulks

| library.Pre-Capture | Pre-Capture - PassQC reads mapping to the whole transcriptome | Pre-Capture - PassQC reads mapping to the Capture Panel | panel.Pre-Capture | idx | library.Post-Capture | Post-Capture - PassQC reads mapping to the whole transcriptome | Post-Capture - PassQC reads mapping to the Capture Panel | panel.Post-Capture | Difference (Pre-Capture - Post-Capture on target) |
|---|---|---|---|---|---|---|---|---|---|
| CON_1_A_FP+25_LY_ABBV-0 | 21661820 | 106633 | NFcapture | 229 | CON_1_A_FP+25_LY_ABBV-0 | 4994437 | 4810777 | TFcapture | 16851043 |
| CON_1_A_FP+25_LY_ABBV-0 | 21661820 | 106633 | NFcapture | 229 | CON_1_A_FP+25_LY_ABBV-0 | 3308596 | 2791954 | NFcapture | 18869866 |
| CON_1_A_FP+25_LY_ABBV-0 | 21661820 | 670221 | TFcapture | 229 | CON_1_A_FP+25_LY_ABBV-0 | 4994437 | 4810777 | TFcapture | 16851043 |
| CON_1_A_FP+25_LY_ABBV-0 | 21661820 | 670221 | TFcapture | 229 | CON_1_A_FP+25_LY_ABBV-0 | 3308596 | 2791954 | NFcapture | 18869866 |
| CON_1_A_FP+25_LY_JANS-0 | 31277033 | 171579 | NFcapture | 241 | CON_1_A_FP+25_LY_JANS-0 | 6949841 | 6674114 | TFcapture | 24602919 |
| CON_1_A_FP+25_LY_JANS-0 | 31277033 | 171579 | NFcapture | 241 | CON_1_A_FP+25_LY_JANS-0 | 4794794 | 4176045 | NFcapture | 27100988 |
| CON_1_A_FP+25_LY_JANS-0 | 31277033 | 1024752 | TFcapture | 241 | CON_1_A_FP+25_LY_JANS-0 | 6949841 | 6674114 | TFcapture | 24602919 |
| CON_1_A_FP+25_LY_JANS-0 | 31277033 | 1024752 | TFcapture | 241 | CON_1_A_FP+25_LY_JANS-0 | 4794794 | 4176045 | NFcapture | 27100988 |
| CON_1_A_FP+25_LY_UCAM-0 | 34645443 | 184221 | NFcapture | 253 | CON_1_A_FP+25_LY_UCAM-0 | 10469753 | 10107023 | TFcapture | 24538420 |
| CON_1_A_FP+25_LY_UCAM-0 | 34645443 | 184221 | NFcapture | 253 | CON_1_A_FP+25_LY_UCAM-0 | 6645530 | 5811713 | NFcapture | 28833730 |
| CON_1_A_FP+25_LY_UCAM-0 | 34645443 | 1300691 | TFcapture | 253 | CON_1_A_FP+25_LY_UCAM-0 | 10469753 | 10107023 | TFcapture | 24538420 |
| CON_1_A_FP+25_LY_UCAM-0 | 34645443 | 1300691 | TFcapture | 253 | CON_1_A_FP+25_LY_UCAM-0 | 6645530 | 5811713 | NFcapture | 28833730 |
| CON_1_A_FP+25_LY_UOXC-0 | 47898745 | 254523 | NFcapture | 265 | CON_1_A_FP+25_LY_UOXC-0 | 13023936 | 12424979 | TFcapture | 35473766 |
| CON_1_A_FP+25_LY_UOXC-0 | 47898745 | 254523 | NFcapture | 265 | CON_1_A_FP+25_LY_UOXC-0 | 8028232 | 6869625 | NFcapture | 41029120 |
| CON_1_A_FP+25_LY_UOXC-0 | 47898745 | 1734691 | TFcapture | 265 | CON_1_A_FP+25_LY_UOXC-0 | 13023936 | 12424979 | TFcapture | 35473766 |
| CON_1_A_FP+25_LY_UOXC-0 | 47898745 | 1734691 | TFcapture | 265 | CON_1_A_FP+25_LY_UOXC-0 | 8028232 | 6869625 | NFcapture | 41029120 |
| CON_1_A_FP+25_LY_UOXZ-0 | 60553382 | 308206 | NFcapture | 277 | CON_1_A_FP+25_LY_UOXZ-0 | 14125723 | 13646164 | TFcapture | 46907218 |
| CON_1_A_FP+25_LY_UOXZ-0 | 60553382 | 308206 | NFcapture | 277 | CON_1_A_FP+25_LY_UOXZ-0 | 7920255 | 6822816 | NFcapture | 53730566 |
| CON_1_A_FP+25_LY_UOXZ-0 | 60553382 | 2373434 | TFcapture | 277 | CON_1_A_FP+25_LY_UOXZ-0 | 14125723 | 13646164 | TFcapture | 46907218 |
| CON_1_A_FP+25_LY_UOXZ-0 | 60553382 | 2373434 | TFcapture | 277 | CON_1_A_FP+25_LY_UOXZ-0 | 7920255 | 6822816 | NFcapture | 53730566 |
| CON_1_A_FP+55_LY_ABBV-0 | 23746668 | 136728 | NFcapture | 235 | CON_1_A_FP+55_LY_ABBV-0 | 5708638 | 5489138 | TFcapture | 18257530 |
| CON_1_A_FP+55_LY_ABBV-0 | 23746668 | 136728 | NFcapture | 235 | CON_1_A_FP+55_LY_ABBV-0 | 4298039 | 3744846 | NFcapture | 20001822 |
| CON_1_A_FP+55_LY_ABBV-0 | 23746668 | 758638 | TFcapture | 235 | CON_1_A_FP+55_LY_ABBV-0 | 5708638 | 5489138 | TFcapture | 18257530 |
| CON_1_A_FP+55_LY_ABBV-0 | 23746668 | 758638 | TFcapture | 235 | CON_1_A_FP+55_LY_ABBV-0 | 4298039 | 3744846 | NFcapture | 20001822 |
| CON_1_A_FP+55_LY_JANS-0 | 26186297 | 147536 | NFcapture | 247 | CON_1_A_FP+55_LY_JANS-0 | 7121226 | 6858304 | TFcapture | 19327993 |
| CON_1_A_FP+55_LY_JANS-0 | 26186297 | 147536 | NFcapture | 247 | CON_1_A_FP+55_LY_JANS-0 | 5199584 | 4557804 | NFcapture | 21628493 |
| CON_1_A_FP+55_LY_JANS-0 | 26186297 | 826006 | TFcapture | 247 | CON_1_A_FP+55_LY_JANS-0 | 7121226 | 6858304 | TFcapture | 19327993 |
| CON_1_A_FP+55_LY_JANS-0 | 26186297 | 826006 | TFcapture | 247 | CON_1_A_FP+55_LY_JANS-0 | 5199584 | 4557804 | NFcapture | 21628493 |
| CON_1_A_FP+55_LY_UCAM-0 | 28148195 | 137186 | NFcapture | 259 | CON_1_A_FP+55_LY_UCAM-0 | 8070652 | 7762594 | TFcapture | 20385601 |
| CON_1_A_FP+55_LY_UCAM-0 | 28148195 | 137186 | NFcapture | 259 | CON_1_A_FP+55_LY_UCAM-0 | 5291944 | 4551056 | NFcapture | 23597139 |
| CON_1_A_FP+55_LY_UCAM-0 | 28148195 | 950642 | TFcapture | 259 | CON_1_A_FP+55_LY_UCAM-0 | 8070652 | 7762594 | TFcapture | 20385601 |
| CON_1_A_FP+55_LY_UCAM-0 | 28148195 | 950642 | TFcapture | 259 | CON_1_A_FP+55_LY_UCAM-0 | 5291944 | 4551056 | NFcapture | 23597139 |
| CON_1_A_FP+55_LY_UOXC-0 | 43025400 | 229169 | NFcapture | 271 | CON_1_A_FP+55_LY_UOXC-0 | 10178510 | 9782969 | TFcapture | 33242431 |
| CON_1_A_FP+55_LY_UOXC-0 | 43025400 | 229169 | NFcapture | 271 | CON_1_A_FP+55_LY_UOXC-0 | 7155159 | 6232931 | NFcapture | 36792469 |
| CON_1_A_FP+55_LY_UOXC-0 | 43025400 | 1365750 | TFcapture | 271 | CON_1_A_FP+55_LY_UOXC-0 | 10178510 | 9782969 | TFcapture | 33242431 |
| CON_1_A_FP+55_LY_UOXC-0 | 43025400 | 1365750 | TFcapture | 271 | CON_1_A_FP+55_LY_UOXC-0 | 7155159 | 6232931 | NFcapture | 36792469 |
| CON_1_A_FP+55_LY_UOXZ-0 | 46748969 | 249845 | NFcapture | 282 | CON_1_A_FP+55_LY_UOXZ-0 | 12294948 | 11849371 | TFcapture | 34899598 |
| CON_1_A_FP+55_LY_UOXZ-0 | 46748969 | 249845 | NFcapture | 282 | CON_1_A_FP+55_LY_UOXZ-0 | 7546439 | 6523425 | NFcapture | 40225544 |
| CON_1_A_FP+55_LY_UOXZ-0 | 46748969 | 1696941 | TFcapture | 282 | CON_1_A_FP+55_LY_UOXZ-0 | 12294948 | 11849371 | TFcapture | 34899598 |
| CON_1_A_FP+55_LY_UOXZ-0 | 46748969 | 1696941 | TFcapture | 282 | CON_1_A_FP+55_LY_UOXZ-0 | 7546439 | 6523425 | NFcapture | 40225544 |
| CON_2_A_FP+25_LY_ABBV-0 | 23027647 | 91367 | NFcapture | 230 | CON_2_A_FP+25_LY_ABBV-0 | 4149130 | 3980140 | TFcapture | 19047507 |
| CON_2_A_FP+25_LY_ABBV-0 | 23027647 | 91367 | NFcapture | 230 | CON_2_A_FP+25_LY_ABBV-0 | 2351894 | 1836110 | NFcapture | 21191537 |
| CON_2_A_FP+25_LY_ABBV-0 | 23027647 | 629403 | TFcapture | 230 | CON_2_A_FP+25_LY_ABBV-0 | 4149130 | 3980140 | TFcapture | 19047507 |
| CON_2_A_FP+25_LY_ABBV-0 | 23027647 | 629403 | TFcapture | 230 | CON_2_A_FP+25_LY_ABBV-0 | 2351894 | 1836110 | NFcapture | 21191537 |
| CON_2_A_FP+25_LY_JANS-0 | 32460428 | 171517 | NFcapture | 242 | CON_2_A_FP+25_LY_JANS-0 | 6905015 | 6615694 | TFcapture | 25844734 |
| CON_2_A_FP+25_LY_JANS-0 | 32460428 | 171517 | NFcapture | 242 | CON_2_A_FP+25_LY_JANS-0 | 4742069 | 4128477 | NFcapture | 28331951 |
| CON_2_A_FP+25_LY_JANS-0 | 32460428 | 1003205 | TFcapture | 242 | CON_2_A_FP+25_LY_JANS-0 | 6905015 | 6615694 | TFcapture | 25844734 |
| CON_2_A_FP+25_LY_JANS-0 | 32460428 | 1003205 | TFcapture | 242 | CON_2_A_FP+25_LY_JANS-0 | 4742069 | 4128477 | NFcapture | 28331951 |
| CON_2_A_FP+25_LY_UCAM-0 | 39721095 | 214999 | NFcapture | 254 | CON_2_A_FP+25_LY_UCAM-0 | 10476994 | 10115304 | TFcapture | 29605791 |
| CON_2_A_FP+25_LY_UCAM-0 | 39721095 | 214999 | NFcapture | 254 | CON_2_A_FP+25_LY_UCAM-0 | 6554322 | 5732994 | NFcapture | 33988101 |
| CON_2_A_FP+25_LY_UCAM-0 | 39721095 | 1498192 | TFcapture | 254 | CON_2_A_FP+25_LY_UCAM-0 | 10476994 | 10115304 | TFcapture | 29605791 |
| CON_2_A_FP+25_LY_UCAM-0 | 39721095 | 1498192 | TFcapture | 254 | CON_2_A_FP+25_LY_UCAM-0 | 6554322 | 5732994 | NFcapture | 33988101 |
| CON_2_A_FP+25_LY_UOXC-0 | 46674474 | 240524 | NFcapture | 266 | CON_2_A_FP+25_LY_UOXC-0 | 12512896 | 12050553 | TFcapture | 34623921 |
| CON_2_A_FP+25_LY_UOXC-0 | 46674474 | 240524 | NFcapture | 266 | CON_2_A_FP+25_LY_UOXC-0 | 7714226 | 6687351 | NFcapture | 39987123 |
| CON_2_A_FP+25_LY_UOXC-0 | 46674474 | 1714771 | TFcapture | 266 | CON_2_A_FP+25_LY_UOXC-0 | 12512896 | 12050553 | TFcapture | 34623921 |
| CON_2_A_FP+25_LY_UOXC-0 | 46674474 | 1714771 | TFcapture | 266 | CON_2_A_FP+25_LY_UOXC-0 | 7714226 | 6687351 | NFcapture | 39987123 |
| CON_2_A_FP+25_LY_UOXZ-0 | 53474219 | 285339 | NFcapture | 278 | CON_2_A_FP+25_LY_UOXZ-0 | 13647746 | 13169378 | TFcapture | 40304841 |
| CON_2_A_FP+25_LY_UOXZ-0 | 53474219 | 285339 | NFcapture | 278 | CON_2_A_FP+25_LY_UOXZ-0 | 7756924 | 6780206 | NFcapture | 46694013 |
| CON_2_A_FP+25_LY_UOXZ-0 | 53474219 | 2179919 | TFcapture | 278 | CON_2_A_FP+25_LY_UOXZ-0 | 13647746 | 13169378 | TFcapture | 40304841 |
| CON_2_A_FP+25_LY_UOXZ-0 | 53474219 | 2179919 | TFcapture | 278 | CON_2_A_FP+25_LY_UOXZ-0 | 7756924 | 6780206 | NFcapture | 46694013 |
| CON_2_A_FP+55_LY_ABBV-0 | 25015248 | 108570 | NFcapture | 236 | CON_2_A_FP+55_LY_ABBV-0 | 5014457 | 4808624 | TFcapture | 20206624 |
| CON_2_A_FP+55_LY_ABBV-0 | 25015248 | 108570 | NFcapture | 236 | CON_2_A_FP+55_LY_ABBV-0 | 2920805 | 2366474 | NFcapture | 22648774 |
| CON_2_A_FP+55_LY_ABBV-0 | 25015248 | 744450 | TFcapture | 236 | CON_2_A_FP+55_LY_ABBV-0 | 5014457 | 4808624 | TFcapture | 20206624 |
| CON_2_A_FP+55_LY_ABBV-0 | 25015248 | 744450 | TFcapture | 236 | CON_2_A_FP+55_LY_ABBV-0 | 2920805 | 2366474 | NFcapture | 22648774 |
| CON_2_A_FP+55_LY_JANS-0 | 32796945 | 187775 | NFcapture | 248 | CON_2_A_FP+55_LY_JANS-0 | 7131485 | 6841920 | TFcapture | 25955025 |
| CON_2_A_FP+55_LY_JANS-0 | 32796945 | 187775 | NFcapture | 248 | CON_2_A_FP+55_LY_JANS-0 | 5274621 | 4627019 | NFcapture | 28169926 |
| CON_2_A_FP+55_LY_JANS-0 | 32796945 | 994471 | TFcapture | 248 | CON_2_A_FP+55_LY_JANS-0 | 7131485 | 6841920 | TFcapture | 25955025 |
| CON_2_A_FP+55_LY_JANS-0 | 32796945 | 994471 | TFcapture | 248 | CON_2_A_FP+55_LY_JANS-0 | 5274621 | 4627019 | NFcapture | 28169926 |
| CON_2_A_FP+55_LY_UCAM-0 | 51998114 | 251652 | NFcapture | 260 | CON_2_A_FP+55_LY_UCAM-0 | 11048522 | 10623317 | TFcapture | 41374797 |
| CON_2_A_FP+55_LY_UCAM-0 | 51998114 | 251652 | NFcapture | 260 | CON_2_A_FP+55_LY_UCAM-0 | 6763403 | 5805392 | NFcapture | 46192722 |
| CON_2_A_FP+55_LY_UCAM-0 | 51998114 | 1727917 | TFcapture | 260 | CON_2_A_FP+55_LY_UCAM-0 | 11048522 | 10623317 | TFcapture | 41374797 |
| CON_2_A_FP+55_LY_UCAM-0 | 51998114 | 1727917 | TFcapture | 260 | CON_2_A_FP+55_LY_UCAM-0 | 6763403 | 5805392 | NFcapture | 46192722 |

| | | | | | | | | | |
|---|---|---|---|---|---|---|---|---|---|
| CON_2_A_FP+55_LY_UOXC-0 | 56140233 | 301896 | NFcapture | 272 | CON_2_A_FP+55_LY_UOXC-0 | 12084218 | 11613021 | TFcapture | 44527212 |
| CON_2_A_FP+55_LY_UOXC-0 | 56140233 | 301896 | NFcapture | 272 | CON_2_A_FP+55_LY_UOXC-0 | 7849550 | 6811625 | NFcapture | 49328608 |
| CON_2_A_FP+55_LY_UOXC-0 | 56140233 | 1865620 | TFcapture | 272 | CON_2_A_FP+55_LY_UOXC-0 | 12084218 | 11613021 | TFcapture | 44527212 |
| CON_2_A_FP+55_LY_UOXC-0 | 56140233 | 1865620 | TFcapture | 272 | CON_2_A_FP+55_LY_UOXC-0 | 7849550 | 6811625 | NFcapture | 49328608 |
| CON_2_A_FP+55_LY_UOXZ-0 | 33694666 | 192651 | NFcapture | 283 | CON_2_A_FP+55_LY_UOXZ-0 | 9094036 | 8777655 | TFcapture | 24917011 |
| CON_2_A_FP+55_LY_UOXZ-0 | 33694666 | 192651 | NFcapture | 283 | CON_2_A_FP+55_LY_UOXZ-0 | 6248056 | 5489145 | NFcapture | 28205521 |
| CON_2_A_FP+55_LY_UOXZ-0 | 33694666 | 1224056 | TFcapture | 283 | CON_2_A_FP+55_LY_UOXZ-0 | 9094036 | 8777655 | TFcapture | 24917011 |
| CON_2_A_FP+55_LY_UOXZ-0 | 33694666 | 1224056 | TFcapture | 283 | CON_2_A_FP+55_LY_UOXZ-0 | 6248056 | 5489145 | NFcapture | 28205521 |
| CON_3_A_FP+25_LY_ABBV-0 | 24690131 | 90576 | NFcapture | 231 | CON_3_A_FP+25_LY_ABBV-0 | 4963587 | 4782518 | TFcapture | 19907613 |
| CON_3_A_FP+25_LY_ABBV-0 | 24690131 | 90576 | NFcapture | 231 | CON_3_A_FP+25_LY_ABBV-0 | 2409171 | 1872594 | NFcapture | 22817537 |
| CON_3_A_FP+25_LY_ABBV-0 | 24690131 | 783862 | TFcapture | 231 | CON_3_A_FP+25_LY_ABBV-0 | 4963587 | 4782518 | TFcapture | 19907613 |
| CON_3_A_FP+25_LY_ABBV-0 | 24690131 | 783862 | TFcapture | 231 | CON_3_A_FP+25_LY_ABBV-0 | 2409171 | 1872594 | NFcapture | 22817537 |
| CON_3_A_FP+25_LY_JANS-0 | 25914348 | 140169 | NFcapture | 243 | CON_3_A_FP+25_LY_JANS-0 | 7377954 | 7079090 | TFcapture | 18835258 |
| CON_3_A_FP+25_LY_JANS-0 | 25914348 | 140169 | NFcapture | 243 | CON_3_A_FP+25_LY_JANS-0 | 5133266 | 4469201 | NFcapture | 21445147 |
| CON_3_A_FP+25_LY_JANS-0 | 25914348 | 802904 | TFcapture | 243 | CON_3_A_FP+25_LY_JANS-0 | 7377954 | 7079090 | TFcapture | 18835258 |
| CON_3_A_FP+25_LY_JANS-0 | 25914348 | 802904 | TFcapture | 243 | CON_3_A_FP+25_LY_JANS-0 | 5133266 | 4469201 | NFcapture | 21445147 |
| CON_3_A_FP+25_LY_UCAM-0 | 39702426 | 212453 | NFcapture | 255 | CON_3_A_FP+25_LY_UCAM-0 | 9380617 | 9061805 | TFcapture | 30640621 |
| CON_3_A_FP+25_LY_UCAM-0 | 39702426 | 212453 | NFcapture | 255 | CON_3_A_FP+25_LY_UCAM-0 | 6128655 | 5355176 | NFcapture | 34347250 |
| CON_3_A_FP+25_LY_UCAM-0 | 39702426 | 1498445 | TFcapture | 255 | CON_3_A_FP+25_LY_UCAM-0 | 9380617 | 9061805 | TFcapture | 30640621 |
| CON_3_A_FP+25_LY_UCAM-0 | 39702426 | 1498445 | TFcapture | 255 | CON_3_A_FP+25_LY_UCAM-0 | 6128655 | 5355176 | NFcapture | 34347250 |
| CON_3_A_FP+25_LY_UOXC-0 | 49572257 | 253824 | NFcapture | 267 | CON_3_A_FP+25_LY_UOXC-0 | 11027041 | 10614684 | TFcapture | 38957573 |
| CON_3_A_FP+25_LY_UOXC-0 | 49572257 | 253824 | NFcapture | 267 | CON_3_A_FP+25_LY_UOXC-0 | 7100729 | 6146775 | NFcapture | 43425482 |
| CON_3_A_FP+25_LY_UOXC-0 | 49572257 | 1782366 | TFcapture | 267 | CON_3_A_FP+25_LY_UOXC-0 | 11027041 | 10614684 | TFcapture | 38957573 |
| CON_3_A_FP+25_LY_UOXC-0 | 49572257 | 1782366 | TFcapture | 267 | CON_3_A_FP+25_LY_UOXC-0 | 7100729 | 6146775 | NFcapture | 43425482 |
| CON_3_A_FP+25_LY_UOXZ-0 | 58669257 | 296768 | NFcapture | 279 | CON_3_A_FP+25_LY_UOXZ-0 | 13649424 | 13194865 | TFcapture | 45474392 |
| CON_3_A_FP+25_LY_UOXZ-0 | 58669257 | 296768 | NFcapture | 279 | CON_3_A_FP+25_LY_UOXZ-0 | 7413056 | 6393053 | NFcapture | 52276204 |
| CON_3_A_FP+25_LY_UOXZ-0 | 58669257 | 2314647 | TFcapture | 279 | CON_3_A_FP+25_LY_UOXZ-0 | 13649424 | 13194865 | TFcapture | 45474392 |
| CON_3_A_FP+25_LY_UOXZ-0 | 58669257 | 2314647 | TFcapture | 279 | CON_3_A_FP+25_LY_UOXZ-0 | 7413056 | 6393053 | NFcapture | 52276204 |
| CON_3_A_FP+55_LY_ABBV-0 | 24881333 | 123705 | NFcapture | 237 | CON_3_A_FP+55_LY_ABBV-0 | 5743200 | 5529377 | TFcapture | 19351956 |
| CON_3_A_FP+55_LY_ABBV-0 | 24881333 | 123705 | NFcapture | 237 | CON_3_A_FP+55_LY_ABBV-0 | 3461053 | 2933448 | NFcapture | 21947885 |
| CON_3_A_FP+55_LY_ABBV-0 | 24881333 | 759977 | TFcapture | 237 | CON_3_A_FP+55_LY_ABBV-0 | 5743200 | 5529377 | TFcapture | 19351956 |
| CON_3_A_FP+55_LY_ABBV-0 | 24881333 | 759977 | TFcapture | 237 | CON_3_A_FP+55_LY_ABBV-0 | 3461053 | 2933448 | NFcapture | 21947885 |
| CON_3_A_FP+55_LY_JANS-0 | 35966025 | 206424 | NFcapture | 249 | CON_3_A_FP+55_LY_JANS-0 | 7782588 | 7466277 | TFcapture | 28499748 |
| CON_3_A_FP+55_LY_JANS-0 | 35966025 | 206424 | NFcapture | 249 | CON_3_A_FP+55_LY_JANS-0 | 5546528 | 4841125 | NFcapture | 31124900 |
| CON_3_A_FP+55_LY_JANS-0 | 35966025 | 1112830 | TFcapture | 249 | CON_3_A_FP+55_LY_JANS-0 | 7782588 | 7466277 | TFcapture | 28499748 |
| CON_3_A_FP+55_LY_JANS-0 | 35966025 | 1112830 | TFcapture | 249 | CON_3_A_FP+55_LY_JANS-0 | 5546528 | 4841125 | NFcapture | 31124900 |
| CON_3_A_FP+55_LY_UCAM-0 | 31924501 | 157166 | NFcapture | 261 | CON_3_A_FP+55_LY_UCAM-0 | 8711534 | 8393357 | TFcapture | 23531144 |
| CON_3_A_FP+55_LY_UCAM-0 | 31924501 | 157166 | NFcapture | 261 | CON_3_A_FP+55_LY_UCAM-0 | 5599692 | 4840456 | NFcapture | 27084045 |
| CON_3_A_FP+55_LY_UCAM-0 | 31924501 | 1092310 | TFcapture | 261 | CON_3_A_FP+55_LY_UCAM-0 | 8711534 | 8393357 | TFcapture | 23531144 |
| CON_3_A_FP+55_LY_UCAM-0 | 31924501 | 1092310 | TFcapture | 261 | CON_3_A_FP+55_LY_UCAM-0 | 5599692 | 4840456 | NFcapture | 27084045 |
| CON_3_A_FP+55_LY_UOXC-0 | 62954375 | 367298 | NFcapture | 273 | CON_3_A_FP+55_LY_UOXC-0 | 15353822 | 14741349 | TFcapture | 48213026 |
| CON_3_A_FP+55_LY_UOXC-0 | 62954375 | 367298 | NFcapture | 273 | CON_3_A_FP+55_LY_UOXC-0 | 10133159 | 8848671 | NFcapture | 54105704 |
| CON_3_A_FP+55_LY_UOXC-0 | 62954375 | 2120586 | TFcapture | 273 | CON_3_A_FP+55_LY_UOXC-0 | 15353822 | 14741349 | TFcapture | 48213026 |
| CON_3_A_FP+55_LY_UOXC-0 | 62954375 | 2120586 | TFcapture | 273 | CON_3_A_FP+55_LY_UOXC-0 | 10133159 | 8848671 | NFcapture | 54105704 |
| CON_3_A_FP+55_LY_UOXZ-0 | 52111750 | 263435 | NFcapture | 284 | CON_3_A_FP+55_LY_UOXZ-0 | 12852253 | 12379962 | TFcapture | 39731788 |
| CON_3_A_FP+55_LY_UOXZ-0 | 52111750 | 263435 | NFcapture | 284 | CON_3_A_FP+55_LY_UOXZ-0 | 7431234 | 6409557 | NFcapture | 45702193 |
| CON_3_A_FP+55_LY_UOXZ-0 | 52111750 | 1788617 | TFcapture | 284 | CON_3_A_FP+55_LY_UOXZ-0 | 12852253 | 12379962 | TFcapture | 39731788 |
| CON_3_A_FP+55_LY_UOXZ-0 | 52111750 | 1788617 | TFcapture | 284 | CON_3_A_FP+55_LY_UOXZ-0 | 7431234 | 6409557 | NFcapture | 45702193 |
| PS1_1_A_FP+25_LY_ABBV-0 | 22143874 | 99633 | NFcapture | 232 | PS1_1_A_FP+25_LY_ABBV-0 | 4903348 | 4699404 | TFcapture | 17444470 |
| PS1_1_A_FP+25_LY_ABBV-0 | 22143874 | 99633 | NFcapture | 232 | PS1_1_A_FP+25_LY_ABBV-0 | 2665449 | 2188891 | NFcapture | 19954983 |
| PS1_1_A_FP+25_LY_ABBV-0 | 22143874 | 707751 | TFcapture | 232 | PS1_1_A_FP+25_LY_ABBV-0 | 4903348 | 4699404 | TFcapture | 17444470 |
| PS1_1_A_FP+25_LY_ABBV-0 | 22143874 | 707751 | TFcapture | 232 | PS1_1_A_FP+25_LY_ABBV-0 | 2665449 | 2188891 | NFcapture | 19954983 |
| PS1_1_A_FP+25_LY_JANS-0 | 27396140 | 149445 | NFcapture | 244 | PS1_1_A_FP+25_LY_JANS-0 | 6434058 | 6212853 | TFcapture | 21183287 |
| PS1_1_A_FP+25_LY_JANS-0 | 27396140 | 149445 | NFcapture | 244 | PS1_1_A_FP+25_LY_JANS-0 | 4107492 | 3556758 | NFcapture | 23839382 |
| PS1_1_A_FP+25_LY_JANS-0 | 27396140 | 1002286 | TFcapture | 244 | PS1_1_A_FP+25_LY_JANS-0 | 6434058 | 6212853 | TFcapture | 21183287 |
| PS1_1_A_FP+25_LY_JANS-0 | 27396140 | 1002286 | TFcapture | 244 | PS1_1_A_FP+25_LY_JANS-0 | 4107492 | 3556758 | NFcapture | 23839382 |
| PS1_1_A_FP+25_LY_UCAM-0 | 41821280 | 187215 | NFcapture | 256 | PS1_1_A_FP+25_LY_UCAM-0 | 8757136 | 8445290 | TFcapture | 33375990 |
| PS1_1_A_FP+25_LY_UCAM-0 | 41821280 | 187215 | NFcapture | 256 | PS1_1_A_FP+25_LY_UCAM-0 | 5446316 | 4666688 | NFcapture | 37154592 |
| PS1_1_A_FP+25_LY_UCAM-0 | 41821280 | 1343513 | TFcapture | 256 | PS1_1_A_FP+25_LY_UCAM-0 | 8757136 | 8445290 | TFcapture | 33375990 |
| PS1_1_A_FP+25_LY_UCAM-0 | 41821280 | 1343513 | TFcapture | 256 | PS1_1_A_FP+25_LY_UCAM-0 | 5446316 | 4666688 | NFcapture | 37154592 |
| PS1_1_A_FP+25_LY_UOXC-0 | 54872159 | 295282 | NFcapture | 268 | PS1_1_A_FP+25_LY_UOXC-0 | 12371003 | 11923641 | TFcapture | 42948518 |
| PS1_1_A_FP+25_LY_UOXC-0 | 54872159 | 295282 | NFcapture | 268 | PS1_1_A_FP+25_LY_UOXC-0 | 7571170 | 6636925 | NFcapture | 48235234 |
| PS1_1_A_FP+25_LY_UOXC-0 | 54872159 | 2120614 | TFcapture | 268 | PS1_1_A_FP+25_LY_UOXC-0 | 12371003 | 11923641 | TFcapture | 42948518 |
| PS1_1_A_FP+25_LY_UOXC-0 | 54872159 | 2120614 | TFcapture | 268 | PS1_1_A_FP+25_LY_UOXC-0 | 7571170 | 6636925 | NFcapture | 48235234 |
| PS1_1_A_FP+25_LY_UOXZ-0 | 58478861 | 262266 | NFcapture | 280 | PS1_1_A_FP+25_LY_UOXZ-0 | 14847995 | 14352244 | TFcapture | 44126617 |
| PS1_1_A_FP+25_LY_UOXZ-0 | 58478861 | 262266 | NFcapture | 280 | PS1_1_A_FP+25_LY_UOXZ-0 | 7373972 | 6318605 | NFcapture | 52160256 |
| PS1_1_A_FP+25_LY_UOXZ-0 | 58478861 | 2248822 | TFcapture | 280 | PS1_1_A_FP+25_LY_UOXZ-0 | 14847995 | 14352244 | TFcapture | 44126617 |
| PS1_1_A_FP+25_LY_UOXZ-0 | 58478861 | 2248822 | TFcapture | 280 | PS1_1_A_FP+25_LY_UOXZ-0 | 7373972 | 6318605 | NFcapture | 52160256 |
| PS1_1_A_FP+55_LY_ABBV-0 | 25300314 | 144816 | NFcapture | 238 | PS1_1_A_FP+55_LY_ABBV-0 | 6373197 | 6128178 | TFcapture | 19172136 |
| PS1_1_A_FP+55_LY_ABBV-0 | 25300314 | 144816 | NFcapture | 238 | PS1_1_A_FP+55_LY_ABBV-0 | 4182739 | 3647694 | NFcapture | 21652620 |
| PS1_1_A_FP+55_LY_ABBV-0 | 25300314 | 874814 | TFcapture | 238 | PS1_1_A_FP+55_LY_ABBV-0 | 6373197 | 6128178 | TFcapture | 19172136 |
| PS1_1_A_FP+55_LY_ABBV-0 | 25300314 | 874814 | TFcapture | 238 | PS1_1_A_FP+55_LY_ABBV-0 | 4182739 | 3647694 | NFcapture | 21652620 |
| PS1_1_A_FP+55_LY_JANS-0 | 31947689 | 146255 | NFcapture | 250 | PS1_1_A_FP+55_LY_JANS-0 | 7025562 | 6743172 | TFcapture | 25204517 |
| PS1_1_A_FP+55_LY_JANS-0 | 31947689 | 146255 | NFcapture | 250 | PS1_1_A_FP+55_LY_JANS-0 | 3750644 | 3173943 | NFcapture | 28773746 |
| PS1_1_A_FP+55_LY_JANS-0 | 31947689 | 1029285 | TFcapture | 250 | PS1_1_A_FP+55_LY_JANS-0 | 7025562 | 6743172 | TFcapture | 25204517 |
| PS1_1_A_FP+55_LY_JANS-0 | 31947689 | 1029285 | TFcapture | 250 | PS1_1_A_FP+55_LY_JANS-0 | 3750644 | 3173943 | NFcapture | 28773746 |
| PS1_1_A_FP+55_LY_UCAM-0 | 30911387 | 111217 | NFcapture | 262 | PS1_1_A_FP+55_LY_UCAM-0 | 6422705 | 6113945 | TFcapture | 24797442 |
| PS1_1_A_FP+55_LY_UCAM-0 | 30911387 | 111217 | NFcapture | 262 | PS1_1_A_FP+55_LY_UCAM-0 | 3134471 | 2487952 | NFcapture | 28423435 |
| PS1_1_A_FP+55_LY_UCAM-0 | 30911387 | 903870 | TFcapture | 262 | PS1_1_A_FP+55_LY_UCAM-0 | 6422705 | 6113945 | TFcapture | 24797442 |
| PS1_1_A_FP+55_LY_UCAM-0 | 30911387 | 903870 | TFcapture | 262 | PS1_1_A_FP+55_LY_UCAM-0 | 3134471 | 2487952 | NFcapture | 28423435 |
| PS1_1_A_FP+55_LY_UOXC-0 | 52213900 | 306352 | NFcapture | 274 | PS1_1_A_FP+55_LY_UOXC-0 | 12697656 | 12207282 | TFcapture | 40006618 |

| | | | | | | | | | |
|---|---|---|---|---|---|---|---|---|---|
| PS1_1_A_FP+55_LY_UOXC-0 | 52213900 | 306352 | NFcapture | 274 | PS1_1_A_FP+55_LY_UOXC-0 | 8492166 | 7471083 | NFcapture | 44742817 |
| PS1_1_A_FP+55_LY_UOXC-0 | 52213900 | 1879524 | TFcapture | 274 | PS1_1_A_FP+55_LY_UOXC-0 | 12697656 | 12207282 | TFcapture | 40006618 |
| PS1_1_A_FP+55_LY_UOXC-0 | 52213900 | 1879524 | TFcapture | 274 | PS1_1_A_FP+55_LY_UOXC-0 | 8492166 | 7471083 | NFcapture | 44742817 |
| PS1_1_A_FP+55_LY_UOXZ-0 | 42727456 | 214816 | NFcapture | 285 | PS1_1_A_FP+55_LY_UOXZ-0 | 11908252 | 11501854 | TFcapture | 31225602 |
| PS1_1_A_FP+55_LY_UOXZ-0 | 42727456 | 214816 | NFcapture | 285 | PS1_1_A_FP+55_LY_UOXZ-0 | 7147774 | 6216164 | NFcapture | 36511292 |
| PS1_1_A_FP+55_LY_UOXZ-0 | 42727456 | 1458620 | TFcapture | 285 | PS1_1_A_FP+55_LY_UOXZ-0 | 11908252 | 11501854 | TFcapture | 31225602 |
| PS1_1_A_FP+55_LY_UOXZ-0 | 42727456 | 1458620 | TFcapture | 285 | PS1_1_A_FP+55_LY_UOXZ-0 | 7147774 | 6216164 | NFcapture | 36511292 |
| PS1_2_A_FP+25_LY_ABBV-0 | 25889270 | 143303 | NFcapture | 233 | PS1_2_A_FP+25_LY_ABBV-0 | 6303256 | 6076142 | TFcapture | 19813128 |
| PS1_2_A_FP+25_LY_ABBV-0 | 25889270 | 143303 | NFcapture | 233 | PS1_2_A_FP+25_LY_ABBV-0 | 3827804 | 3296122 | NFcapture | 22593148 |
| PS1_2_A_FP+25_LY_ABBV-0 | 25889270 | 942414 | TFcapture | 233 | PS1_2_A_FP+25_LY_ABBV-0 | 6303256 | 6076142 | TFcapture | 19813128 |
| PS1_2_A_FP+25_LY_ABBV-0 | 25889270 | 942414 | TFcapture | 233 | PS1_2_A_FP+25_LY_ABBV-0 | 3827804 | 3296122 | NFcapture | 22593148 |
| PS1_2_A_FP+25_LY_JANS-0 | 28018243 | 149713 | NFcapture | 245 | PS1_2_A_FP+25_LY_JANS-0 | 6457074 | 6203007 | TFcapture | 21815236 |
| PS1_2_A_FP+25_LY_JANS-0 | 28018243 | 149713 | NFcapture | 245 | PS1_2_A_FP+25_LY_JANS-0 | 4420762 | 3850703 | NFcapture | 24167540 |
| PS1_2_A_FP+25_LY_JANS-0 | 28018243 | 920602 | TFcapture | 245 | PS1_2_A_FP+25_LY_JANS-0 | 6457074 | 6203007 | TFcapture | 21815236 |
| PS1_2_A_FP+25_LY_JANS-0 | 28018243 | 920602 | TFcapture | 245 | PS1_2_A_FP+25_LY_JANS-0 | 4420762 | 3850703 | NFcapture | 24167540 |
| PS1_2_A_FP+25_LY_UCAM-0 | 41797374 | 194082 | NFcapture | 257 | PS1_2_A_FP+25_LY_UCAM-0 | 10002712 | 9645366 | TFcapture | 32152008 |
| PS1_2_A_FP+25_LY_UCAM-0 | 41797374 | 194082 | NFcapture | 257 | PS1_2_A_FP+25_LY_UCAM-0 | 5934085 | 5073119 | NFcapture | 36724255 |
| PS1_2_A_FP+25_LY_UCAM-0 | 41797374 | 1407227 | TFcapture | 257 | PS1_2_A_FP+25_LY_UCAM-0 | 10002712 | 9645366 | TFcapture | 32152008 |
| PS1_2_A_FP+25_LY_UCAM-0 | 41797374 | 1407227 | TFcapture | 257 | PS1_2_A_FP+25_LY_UCAM-0 | 5934085 | 5073119 | NFcapture | 36724255 |
| PS1_2_A_FP+25_LY_UOXC-0 | 59112619 | 323894 | NFcapture | 269 | PS1_2_A_FP+25_LY_UOXC-0 | 12385480 | 11918101 | TFcapture | 47194518 |
| PS1_2_A_FP+25_LY_UOXC-0 | 59112619 | 323894 | NFcapture | 269 | PS1_2_A_FP+25_LY_UOXC-0 | 8086479 | 7115661 | NFcapture | 51996958 |
| PS1_2_A_FP+25_LY_UOXC-0 | 59112619 | 2095715 | TFcapture | 269 | PS1_2_A_FP+25_LY_UOXC-0 | 12385480 | 11918101 | TFcapture | 47194518 |
| PS1_2_A_FP+25_LY_UOXC-0 | 59112619 | 2095715 | TFcapture | 269 | PS1_2_A_FP+25_LY_UOXC-0 | 8086479 | 7115661 | NFcapture | 51996958 |
| PS1_2_A_FP+25_LY_UOXZ-0 | 52820527 | 280755 | NFcapture | 281 | PS1_2_A_FP+25_LY_UOXZ-0 | 14223802 | 13712618 | TFcapture | 39107909 |
| PS1_2_A_FP+25_LY_UOXZ-0 | 52820527 | 280755 | NFcapture | 281 | PS1_2_A_FP+25_LY_UOXZ-0 | 8180116 | 7063753 | NFcapture | 45756774 |
| PS1_2_A_FP+25_LY_UOXZ-0 | 52820527 | 1986258 | TFcapture | 281 | PS1_2_A_FP+25_LY_UOXZ-0 | 14223802 | 13712618 | TFcapture | 39107909 |
| PS1_2_A_FP+25_LY_UOXZ-0 | 52820527 | 1986258 | TFcapture | 281 | PS1_2_A_FP+25_LY_UOXZ-0 | 8180116 | 7063753 | NFcapture | 45756774 |
| PS1_2_A_FP+55_LY_ABBV-0 | 32113090 | 196224 | NFcapture | 239 | PS1_2_A_FP+55_LY_ABBV-0 | 6968220 | 6716820 | TFcapture | 25396270 |
| PS1_2_A_FP+55_LY_ABBV-0 | 32113090 | 196224 | NFcapture | 239 | PS1_2_A_FP+55_LY_ABBV-0 | 4748329 | 4156328 | NFcapture | 27956762 |
| PS1_2_A_FP+55_LY_ABBV-0 | 32113090 | 1080873 | TFcapture | 239 | PS1_2_A_FP+55_LY_ABBV-0 | 6968220 | 6716820 | TFcapture | 25396270 |
| PS1_2_A_FP+55_LY_ABBV-0 | 32113090 | 1080873 | TFcapture | 239 | PS1_2_A_FP+55_LY_ABBV-0 | 4748329 | 4156328 | NFcapture | 27956762 |
| PS1_2_A_FP+55_LY_JANS-0 | 32617059 | 181072 | NFcapture | 251 | PS1_2_A_FP+55_LY_JANS-0 | 8348903 | 8021627 | TFcapture | 24595432 |
| PS1_2_A_FP+55_LY_JANS-0 | 32617059 | 181072 | NFcapture | 251 | PS1_2_A_FP+55_LY_JANS-0 | 5105122 | 4423398 | NFcapture | 28193661 |
| PS1_2_A_FP+55_LY_JANS-0 | 32617059 | 1173611 | TFcapture | 251 | PS1_2_A_FP+55_LY_JANS-0 | 8348903 | 8021627 | TFcapture | 24595432 |
| PS1_2_A_FP+55_LY_JANS-0 | 32617059 | 1173611 | TFcapture | 251 | PS1_2_A_FP+55_LY_JANS-0 | 5105122 | 4423398 | NFcapture | 28193661 |
| PS1_2_A_FP+55_LY_UCAM-0 | 36649414 | 155057 | NFcapture | 263 | PS1_2_A_FP+55_LY_UCAM-0 | 9743092 | 9364027 | TFcapture | 27285387 |
| PS1_2_A_FP+55_LY_UCAM-0 | 36649414 | 155057 | NFcapture | 263 | PS1_2_A_FP+55_LY_UCAM-0 | 5377914 | 4462562 | NFcapture | 32186852 |
| PS1_2_A_FP+55_LY_UCAM-0 | 36649414 | 1178928 | TFcapture | 263 | PS1_2_A_FP+55_LY_UCAM-0 | 9743092 | 9364027 | TFcapture | 27285387 |
| PS1_2_A_FP+55_LY_UCAM-0 | 36649414 | 1178928 | TFcapture | 263 | PS1_2_A_FP+55_LY_UCAM-0 | 5377914 | 4462562 | NFcapture | 32186852 |
| PS1_2_A_FP+55_LY_UOXC-0 | 42881241 | 251870 | NFcapture | 275 | PS1_2_A_FP+55_LY_UOXC-0 | 13076351 | 12571960 | TFcapture | 30309281 |
| PS1_2_A_FP+55_LY_UOXC-0 | 42881241 | 251870 | NFcapture | 275 | PS1_2_A_FP+55_LY_UOXC-0 | 8579696 | 7550746 | NFcapture | 35330495 |
| PS1_2_A_FP+55_LY_UOXC-0 | 42881241 | 1531232 | TFcapture | 275 | PS1_2_A_FP+55_LY_UOXC-0 | 13076351 | 12571960 | TFcapture | 30309281 |
| PS1_2_A_FP+55_LY_UOXC-0 | 42881241 | 1531232 | TFcapture | 275 | PS1_2_A_FP+55_LY_UOXC-0 | 8579696 | 7550746 | NFcapture | 35330495 |
| PS1_2_A_FP+55_LY_UOXZ-0 | 52731176 | 259279 | NFcapture | 286 | PS1_2_A_FP+55_LY_UOXZ-0 | 12841783 | 12370607 | TFcapture | 40360569 |
| PS1_2_A_FP+55_LY_UOXZ-0 | 52731176 | 259279 | NFcapture | 286 | PS1_2_A_FP+55_LY_UOXZ-0 | 7239121 | 6234063 | NFcapture | 46497113 |
| PS1_2_A_FP+55_LY_UOXZ-0 | 52731176 | 1827799 | TFcapture | 286 | PS1_2_A_FP+55_LY_UOXZ-0 | 12841783 | 12370607 | TFcapture | 40360569 |
| PS1_2_A_FP+55_LY_UOXZ-0 | 52731176 | 1827799 | TFcapture | 286 | PS1_2_A_FP+55_LY_UOXZ-0 | 7239121 | 6234063 | NFcapture | 46497113 |
| PS1_3_A_FP+25_LY_ABBV-0 | 22684652 | 110313 | NFcapture | 234 | PS1_3_A_FP+25_LY_ABBV-0 | 4814408 | 4624444 | TFcapture | 18060208 |
| PS1_3_A_FP+25_LY_ABBV-0 | 22684652 | 110313 | NFcapture | 234 | PS1_3_A_FP+25_LY_ABBV-0 | 2988394 | 2501722 | NFcapture | 20182930 |
| PS1_3_A_FP+25_LY_ABBV-0 | 22684652 | 712030 | TFcapture | 234 | PS1_3_A_FP+25_LY_ABBV-0 | 4814408 | 4624444 | TFcapture | 18060208 |
| PS1_3_A_FP+25_LY_ABBV-0 | 22684652 | 712030 | TFcapture | 234 | PS1_3_A_FP+25_LY_ABBV-0 | 2988394 | 2501722 | NFcapture | 20182930 |
| PS1_3_A_FP+25_LY_JANS-0 | 36630816 | 199994 | NFcapture | 246 | PS1_3_A_FP+25_LY_JANS-0 | 8162634 | 7842217 | TFcapture | 28788599 |
| PS1_3_A_FP+25_LY_JANS-0 | 36630816 | 199994 | NFcapture | 246 | PS1_3_A_FP+25_LY_JANS-0 | 5449427 | 4750226 | NFcapture | 31880590 |
| PS1_3_A_FP+25_LY_JANS-0 | 36630816 | 1168255 | TFcapture | 246 | PS1_3_A_FP+25_LY_JANS-0 | 8162634 | 7842217 | TFcapture | 28788599 |
| PS1_3_A_FP+25_LY_JANS-0 | 36630816 | 1168255 | TFcapture | 246 | PS1_3_A_FP+25_LY_JANS-0 | 5449427 | 4750226 | NFcapture | 31880590 |
| PS1_3_A_FP+25_LY_UCAM-0 | 35492295 | 176024 | NFcapture | 258 | PS1_3_A_FP+25_LY_UCAM-0 | 8680799 | 8364347 | TFcapture | 27127948 |
| PS1_3_A_FP+25_LY_UCAM-0 | 35492295 | 176024 | NFcapture | 258 | PS1_3_A_FP+25_LY_UCAM-0 | 5608180 | 4865332 | NFcapture | 30626963 |
| PS1_3_A_FP+25_LY_UCAM-0 | 35492295 | 1192840 | TFcapture | 258 | PS1_3_A_FP+25_LY_UCAM-0 | 8680799 | 8364347 | TFcapture | 27127948 |
| PS1_3_A_FP+25_LY_UCAM-0 | 35492295 | 1192840 | TFcapture | 258 | PS1_3_A_FP+25_LY_UCAM-0 | 5608180 | 4865332 | NFcapture | 30626963 |
| PS1_3_A_FP+25_LY_UOXC-0 | 56688211 | 308046 | NFcapture | 270 | PS1_3_A_FP+25_LY_UOXC-0 | 11947970 | 11509935 | TFcapture | 45178276 |
| PS1_3_A_FP+25_LY_UOXC-0 | 56688211 | 308046 | NFcapture | 270 | PS1_3_A_FP+25_LY_UOXC-0 | 7437459 | 6522025 | NFcapture | 50166186 |
| PS1_3_A_FP+25_LY_UOXC-0 | 56688211 | 2172627 | TFcapture | 270 | PS1_3_A_FP+25_LY_UOXC-0 | 11947970 | 11509935 | TFcapture | 45178276 |
| PS1_3_A_FP+25_LY_UOXC-0 | 56688211 | 2172627 | TFcapture | 270 | PS1_3_A_FP+25_LY_UOXC-0 | 7437459 | 6522025 | NFcapture | 50166186 |
| PS1_3_A_FP+55_LY_ABBV-0 | 33626086 | 199826 | NFcapture | 240 | PS1_3_A_FP+55_LY_ABBV-0 | 7690617 | 7411403 | TFcapture | 26214683 |
| PS1_3_A_FP+55_LY_ABBV-0 | 33626086 | 199826 | NFcapture | 240 | PS1_3_A_FP+55_LY_ABBV-0 | 4764557 | 4134156 | NFcapture | 29491930 |
| PS1_3_A_FP+55_LY_ABBV-0 | 33626086 | 1210557 | TFcapture | 240 | PS1_3_A_FP+55_LY_ABBV-0 | 7690617 | 7411403 | TFcapture | 26214683 |
| PS1_3_A_FP+55_LY_ABBV-0 | 33626086 | 1210557 | TFcapture | 240 | PS1_3_A_FP+55_LY_ABBV-0 | 4764557 | 4134156 | NFcapture | 29491930 |
| PS1_3_A_FP+55_LY_JANS-0 | 33434318 | 187284 | NFcapture | 252 | PS1_3_A_FP+55_LY_JANS-0 | 7919617 | 7606885 | TFcapture | 25827433 |
| PS1_3_A_FP+55_LY_JANS-0 | 33434318 | 187284 | NFcapture | 252 | PS1_3_A_FP+55_LY_JANS-0 | 5308874 | 4620739 | NFcapture | 28813579 |
| PS1_3_A_FP+55_LY_JANS-0 | 33434318 | 1061015 | TFcapture | 252 | PS1_3_A_FP+55_LY_JANS-0 | 7919617 | 7606885 | TFcapture | 25827433 |
| PS1_3_A_FP+55_LY_JANS-0 | 33434318 | 1061015 | TFcapture | 252 | PS1_3_A_FP+55_LY_JANS-0 | 5308874 | 4620739 | NFcapture | 28813579 |
| PS1_3_A_FP+55_LY_UCAM-0 | 36136097 | 155304 | NFcapture | 264 | PS1_3_A_FP+55_LY_UCAM-0 | 6869109 | 6582889 | TFcapture | 29553208 |
| PS1_3_A_FP+55_LY_UCAM-0 | 36136097 | 155304 | NFcapture | 264 | PS1_3_A_FP+55_LY_UCAM-0 | 3547645 | 2881716 | NFcapture | 33254381 |
| PS1_3_A_FP+55_LY_UCAM-0 | 36136097 | 1101538 | TFcapture | 264 | PS1_3_A_FP+55_LY_UCAM-0 | 6869109 | 6582889 | TFcapture | 29553208 |
| PS1_3_A_FP+55_LY_UCAM-0 | 36136097 | 1101538 | TFcapture | 264 | PS1_3_A_FP+55_LY_UCAM-0 | 3547645 | 2881716 | NFcapture | 33254381 |
| PS1_3_A_FP+55_LY_UOXC-0 | 46305654 | 268653 | NFcapture | 276 | PS1_3_A_FP+55_LY_UOXC-0 | 10589070 | 10191663 | TFcapture | 36113991 |
| PS1_3_A_FP+55_LY_UOXC-0 | 46305654 | 268653 | NFcapture | 276 | PS1_3_A_FP+55_LY_UOXC-0 | 7521378 | 6622607 | NFcapture | 39683047 |
| PS1_3_A_FP+55_LY_UOXC-0 | 46305654 | 1617853 | TFcapture | 276 | PS1_3_A_FP+55_LY_UOXC-0 | 10589070 | 10191663 | TFcapture | 36113991 |
| PS1_3_A_FP+55_LY_UOXC-0 | 46305654 | 1617853 | TFcapture | 276 | PS1_3_A_FP+55_LY_UOXC-0 | 7521378 | 6622607 | NFcapture | 39683047 |

Mini-bulks

| library | PassQC reads mapping to the whole transcriptome | PassQC reads mapping to the Capture Panel | panel | lane | group | NF_Difference (Pre-Capture - Post--Capture on target) | TF850_Difference (Pre-Capture - Post--Capture on target) | TF150_Difference (Pre-Capture - Post--Capture on target) |
|---|---|---|---|---|---|---|---|---|
| WTCHG_255094_227256 | 1578400 | 4225 | NFcapture | 255094 | Pre NF-Capture | 1189247 | -36724 | 164634 |
| WTCHG_255094_227280 | 1440238 | 3971 | NFcapture | 255094 | Pre NF-Capture | 1017663 | 22704 | 183122 |
| WTCHG_255094_228256 | 1693371 | 4868 | NFcapture | 255094 | Pre NF-Capture | 1213622 | 40946 | 227791 |
| WTCHG_255094_228280 | 1390597 | 3744 | NFcapture | 255094 | Pre NF-Capture | 974922 | 168009 | 292578 |
| WTCHG_255094_255256 | 1610314 | 3969 | NFcapture | 255094 | Pre NF-Capture | 1240576 | 216119 | 219225 |
| WTCHG_255094_255280 | 1391426 | 3100 | NFcapture | 255094 | Pre NF-Capture | 1045298 | 57336 | 160349 |
| WTCHG_255094_256256 | 1568610 | 3710 | NFcapture | 255094 | Pre NF-Capture | 1173125 | 191361 | 195545 |
| WTCHG_255094_256280 | 1597989 | 3768 | NFcapture | 255094 | Pre NF-Capture | 1224346 | 95306 | 196602 |
| WTCHG_264983_227208 | 1375502 | 20320 | TFcapture | 264983 | Pre TF-Capture | | | |
| WTCHG_264983_227232 | 1237324 | 17380 | TFcapture | 264983 | Pre TF-Capture | | | |
| WTCHG_264983_228208 | 1383494 | 21596 | TFcapture | 264983 | Pre TF-Capture | | | |
| WTCHG_264983_228232 | 1182356 | 16795 | TFcapture | 264983 | Pre TF-Capture | | | |
| WTCHG_264983_255208 | 254537 | 1780 | TFcapture | 264983 | Pre TF-Capture | | | |
| WTCHG_264983_255232 | 920064 | 12843 | TFcapture | 264983 | Pre TF-Capture | | | |
| WTCHG_264983_256208 | 250093 | 2254 | TFcapture | 264983 | Pre TF-Capture | | | |
| WTCHG_264983_256232 | 1014475 | 15328 | TFcapture | 264983 | Pre TF-Capture | | | |
| WTCHG_422059_227256 | 590017 | 389153 | NFcapture | 422059 | Post-Capture NF | | | |
| WTCHG_422059_227280 | 588543 | 422575 | NFcapture | 422059 | Post-Capture NF | | | |
| WTCHG_422059_228256 | 700014 | 479749 | NFcapture | 422059 | Post-Capture NF | | | |
| WTCHG_422059_228280 | 580165 | 415675 | NFcapture | 422059 | Post-Capture NF | | | |
| WTCHG_422059_255256 | 559617 | 369738 | NFcapture | 422059 | Post-Capture NF | | | |
| WTCHG_422059_255280 | 510320 | 346128 | NFcapture | 422059 | Post-Capture NF | | | |
| WTCHG_422059_256256 | 593044 | 395485 | NFcapture | 422059 | Post-Capture NF | | | |
| WTCHG_422059_256280 | 554925 | 373643 | NFcapture | 422059 | Post-Capture NF | | | |
| WTCHG_473873_227208 | 1576206 | 1412226 | TFcapture | 473873 | Post-Capture TF850 | | | |
| WTCHG_473873_227232 | 1383328 | 1214620 | TFcapture | 473873 | Post-Capture TF850 | | | |
| WTCHG_473873_228208 | 1505934 | 1342548 | TFcapture | 473873 | Post-Capture TF850 | | | |
| WTCHG_473873_228232 | 1162072 | 1014347 | TFcapture | 473873 | Post-Capture TF850 | | | |
| WTCHG_473873_255208 | 49701 | 38418 | TFcapture | 473873 | Post-Capture TF850 | | | |
| WTCHG_473873_255232 | 991639 | 862728 | TFcapture | 473873 | Post-Capture TF850 | | | |
| WTCHG_473873_256208 | 74890 | 58732 | TFcapture | 473873 | Post-Capture TF850 | | | |
| WTCHG_473873_256232 | 1073710 | 919169 | TFcapture | 473873 | Post-Capture TF850 | | | |
| WTCHG_473874_227208 | 1330204 | 1210868 | TFcapture | 473874 | Post-Capture TF150 | | | |
| WTCHG_473874_227232 | 1185911 | 1054202 | TFcapture | 473874 | Post-Capture TF150 | | | |
| WTCHG_473874_228208 | 1269232 | 1155703 | TFcapture | 473874 | Post-Capture TF150 | | | |
| WTCHG_473874_228232 | 1000916 | 889778 | TFcapture | 473874 | Post-Capture TF150 | | | |
| WTCHG_473874_255208 | 43329 | 35312 | TFcapture | 473874 | Post-Capture TF150 | | | |
| WTCHG_473874_255232 | 867658 | 759715 | TFcapture | 473874 | Post-Capture TF150 | | | |
| WTCHG_473874_256208 | 66646 | 54548 | TFcapture | 473874 | Post-Capture TF150 | | | |
| WTCHG_473874_256232 | 942744 | 817873 | TFcapture | 473874 | Post-Capture TF150 | | | |
