# Supplementary material for: Targeted RNA sequencing enhances gene expression profiling of ultra-low input samples
Source: RNA Biol. 2020 Jun 28;17(12):1741–53. doi: 10.1080/15476286.2020.1777768 (PMC7746246; doi:10.1080/15476286.2020.1777768)
Supplement: Supplemental Material [file KRNB_A_1777768_SM6200.zip › TableS7_Global_Capture_metrics.pdf]

Bulks

| Sample ID | panel | % on-target reads Pre-capture | % on-target reads Post-capture | Fold Enrichment |
|---|---|---|---|---|
| CON_1_A_FP+25_LY_ABBV-0_NFcapture_mapping.proportion.dedup | NFcapture | 0.49 | 84.38 | 172.20 |
| CON_1_A_FP+25_LY_JANS-0_NFcapture_mapping.proportion.dedup | NFcapture | 0.54 | 87.09 | 161.28 |
| CON_1_A_FP+25_LY_UCAM-0_NFcapture_mapping.proportion.dedup | NFcapture | 0.53 | 87.45 | 165.00 |
| CON_1_A_FP+25_LY_UOXC-0_NFcapture_mapping.proportion.dedup | NFcapture | 0.53 | 85.56 | 161.43 |
| CON_1_A_FP+25_LY_UOXZ-0_NFcapture_mapping.proportion.dedup | NFcapture | 0.5 | 86.14 | 172.28 |
| CON_1_A_FP+55_LY_ABBV-0_NFcapture_mapping.proportion.dedup | NFcapture | 0.57 | 87.12 | 152.84 |
| CON_1_A_FP+55_LY_JANS-0_NFcapture_mapping.proportion.dedup | NFcapture | 0.56 | 87.65 | 156.52 |
| CON_1_A_FP+55_LY_UCAM-0_NFcapture_mapping.proportion.dedup | NFcapture | 0.48 | 85.99 | 179.15 |
| CON_1_A_FP+55_LY_UOXC-0_NFcapture_mapping.proportion.dedup | NFcapture | 0.53 | 87.11 | 164.36 |
| CON_1_A_FP+55_LY_UOXZ-0_NFcapture_mapping.proportion.dedup | NFcapture | 0.53 | 86.44 | 163.09 |
| CON_2_A_FP+25_LY_ABBV-0_NFcapture_mapping.proportion.dedup | NFcapture | 0.39 | 78.06 | 200.15 |
| CON_2_A_FP+25_LY_JANS-0_NFcapture_mapping.proportion.dedup | NFcapture | 0.52 | 87.06 | 167.42 |
| CON_2_A_FP+25_LY_UCAM-0_NFcapture_mapping.proportion.dedup | NFcapture | 0.54 | 87.46 | 161.96 |
| CON_2_A_FP+25_LY_UOXC-0_NFcapture_mapping.proportion.dedup | NFcapture | 0.51 | 86.68 | 169.96 |
| CON_2_A_FP+25_LY_UOXZ-0_NFcapture_mapping.proportion.dedup | NFcapture | 0.53 | 87.4 | 164.91 |
| CON_2_A_FP+55_LY_ABBV-0_NFcapture_mapping.proportion.dedup | NFcapture | 0.43 | 81.02 | 188.42 |
| CON_2_A_FP+55_LY_JANS-0_NFcapture_mapping.proportion.dedup | NFcapture | 0.57 | 87.72 | 153.89 |
| CON_2_A_FP+55_LY_UCAM-0_NFcapture_mapping.proportion.dedup | NFcapture | 0.48 | 85.83 | 178.81 |
| CON_2_A_FP+55_LY_UOXC-0_NFcapture_mapping.proportion.dedup | NFcapture | 0.53 | 86.77 | 163.72 |
| CON_2_A_FP+55_LY_UOXZ-0_NFcapture_mapping.proportion.dedup | NFcapture | 0.57 | 87.85 | 154.12 |
| CON_3_A_FP+25_LY_ABBV-0_NFcapture_mapping.proportion.dedup | NFcapture | 0.36 | 77.72 | 215.89 |
| CON_3_A_FP+25_LY_JANS-0_NFcapture_mapping.proportion.dedup | NFcapture | 0.54 | 87.06 | 161.22 |
| CON_3_A_FP+25_LY_UCAM-0_NFcapture_mapping.proportion.dedup | NFcapture | 0.53 | 87.37 | 164.85 |
| CON_3_A_FP+25_LY_UOXC-0_NFcapture_mapping.proportion.dedup | NFcapture | 0.51 | 86.56 | 169.73 |
| CON_3_A_FP+25_LY_UOXZ-0_NFcapture_mapping.proportion.dedup | NFcapture | 0.5 | 86.24 | 172.48 |
| CON_3_A_FP+55_LY_ABBV-0_NFcapture_mapping.proportion.dedup | NFcapture | 0.49 | 84.75 | 172.96 |
| CON_3_A_FP+55_LY_JANS-0_NFcapture_mapping.proportion.dedup | NFcapture | 0.57 | 87.28 | 153.12 |
| CON_3_A_FP+55_LY_UCAM-0_NFcapture_mapping.proportion.dedup | NFcapture | 0.49 | 86.44 | 176.41 |
| CON_3_A_FP+55_LY_UOXC-0_NFcapture_mapping.proportion.dedup | NFcapture | 0.58 | 87.32 | 150.55 |
| CON_3_A_FP+55_LY_UOXZ-0_NFcapture_mapping.proportion.dedup | NFcapture | 0.5 | 86.25 | 172.50 |
| PS1_1_A_FP+25_LY_ABBV-0_NFcapture_mapping.proportion.dedup | NFcapture | 0.44 | 82.12 | 186.64 |
| PS1_1_A_FP+25_LY_JANS-0_NFcapture_mapping.proportion.dedup | NFcapture | 0.54 | 86.59 | 160.35 |
| PS1_1_A_FP+25_LY_UCAM-0_NFcapture_mapping.proportion.dedup | NFcapture | 0.44 | 85.68 | 194.73 |
| PS1_1_A_FP+25_LY_UOXC-0_NFcapture_mapping.proportion.dedup | NFcapture | 0.53 | 87.66 | 165.40 |
| PS1_1_A_FP+25_LY_UOXZ-0_NFcapture_mapping.proportion.dedup | NFcapture | 0.44 | 85.68 | 194.73 |
| PS1_1_A_FP+55_LY_ABBV-0_NFcapture_mapping.proportion.dedup | NFcapture | 0.57 | 87.2 | 152.98 |
| PS1_1_A_FP+55_LY_JANS-0_NFcapture_mapping.proportion.dedup | NFcapture | 0.45 | 84.62 | 188.04 |
| PS1_1_A_FP+55_LY_UCAM-0_NFcapture_mapping.proportion.dedup | NFcapture | 0.35 | 79.37 | 226.77 |
| PS1_1_A_FP+55_LY_UOXC-0_NFcapture_mapping.proportion.dedup | NFcapture | 0.58 | 87.97 | 151.67 |
| PS1_1_A_FP+55_LY_UOXZ-0_NFcapture_mapping.proportion.dedup | NFcapture | 0.5 | 86.96 | 173.92 |
| PS1_2_A_FP+25_LY_ABBV-0_NFcapture_mapping.proportion.dedup | NFcapture | 0.55 | 86.1 | 156.55 |
| PS1_2_A_FP+25_LY_JANS-0_NFcapture_mapping.proportion.dedup | NFcapture | 0.53 | 87.1 | 164.34 |
| PS1_2_A_FP+25_LY_UCAM-0_NFcapture_mapping.proportion.dedup | NFcapture | 0.46 | 85.49 | 185.85 |
| PS1_2_A_FP+25_LY_UOXC-0_NFcapture_mapping.proportion.dedup | NFcapture | 0.54 | 87.99 | 162.94 |
| PS1_2_A_FP+25_LY_UOXZ-0_NFcapture_mapping.proportion.dedup | NFcapture | 0.53 | 86.35 | 162.92 |
| PS1_2_A_FP+55_LY_ABBV-0_NFcapture_mapping.proportion.dedup | NFcapture | 0.61 | 87.53 | 143.49 |
| PS1_2_A_FP+55_LY_JANS-0_NFcapture_mapping.proportion.dedup | NFcapture | 0.55 | 86.64 | 157.53 |
| PS1_2_A_FP+55_LY_UCAM-0_NFcapture_mapping.proportion.dedup | NFcapture | 0.42 | 82.97 | 197.55 |
| PS1_2_A_FP+55_LY_UOXC-0_NFcapture_mapping.proportion.dedup | NFcapture | 0.58 | 88 | 151.72 |
| PS1_2_A_FP+55_LY_UOXZ-0_NFcapture_mapping.proportion.dedup | NFcapture | 0.49 | 86.11 | 175.73 |
| PS1_3_A_FP+25_LY_ABBV-0_NFcapture_mapping.proportion.dedup | NFcapture | 0.48 | 83.71 | 174.40 |
| PS1_3_A_FP+25_LY_JANS-0_NFcapture_mapping.proportion.dedup | NFcapture | 0.54 | 87.16 | 161.41 |

| | | | | |
|---|---|---|---|---|
| PS1_3_A_FP+25_LY_UCAM-0_NFcapture_mapping.proportion.dedup | NFcapture | 0.49 | 86.75 | 177.04 |
| PS1_3_A_FP+25_LY_UOXC-0_NFcapture_mapping.proportion.dedup | NFcapture | 0.54 | 87.69 | 162.39 |
| PS1_3_A_FP+55_LY_ABBV-0_NFcapture_mapping.proportion.dedup | NFcapture | 0.59 | 86.76 | 147.05 |
| PS1_3_A_FP+55_LY_JANS-0_NFcapture_mapping.proportion.dedup | NFcapture | 0.56 | 87.03 | 155.41 |
| PS1_3_A_FP+55_LY_UCAM-0_NFcapture_mapping.proportion.dedup | NFcapture | 0.42 | 81.22 | 193.38 |
| PS1_3_A_FP+55_LY_UOXC-0_NFcapture_mapping.proportion.dedup | NFcapture | 0.58 | 88.05 | 151.81 |
| CON_1_A_FP+25_LY_ABBV-0_TFcapture_mapping.proportion.dedup | TFcapture | 3.09 | 96.32 | 31.17 |
| CON_1_A_FP+25_LY_JANS-0_TFcapture_mapping.proportion.dedup | TFcapture | 3.27 | 96.03 | 29.37 |
| CON_1_A_FP+25_LY_UCAM-0_TFcapture_mapping.proportion.dedup | TFcapture | 3.75 | 96.53 | 25.74 |
| CON_1_A_FP+25_LY_UOXC-0_TFcapture_mapping.proportion.dedup | TFcapture | 3.62 | 95.4 | 26.35 |
| CON_1_A_FP+25_LY_UOXZ-0_TFcapture_mapping.proportion.dedup | TFcapture | 3.91 | 96.6 | 24.71 |
| CON_1_A_FP+55_LY_ABBV-0_TFcapture_mapping.proportion.dedup | TFcapture | 3.19 | 96.15 | 30.14 |
| CON_1_A_FP+55_LY_JANS-0_TFcapture_mapping.proportion.dedup | TFcapture | 3.15 | 96.3 | 30.57 |
| CON_1_A_FP+55_LY_UCAM-0_TFcapture_mapping.proportion.dedup | TFcapture | 3.37 | 96.18 | 28.54 |
| CON_1_A_FP+55_LY_UOXC-0_TFcapture_mapping.proportion.dedup | TFcapture | 3.17 | 96.11 | 30.32 |
| CON_1_A_FP+55_LY_UOXZ-0_TFcapture_mapping.proportion.dedup | TFcapture | 3.62 | 96.37 | 26.62 |
| CON_2_A_FP+25_LY_ABBV-0_TFcapture_mapping.proportion.dedup | TFcapture | 2.73 | 95.92 | 35.14 |
| CON_2_A_FP+25_LY_JANS-0_TFcapture_mapping.proportion.dedup | TFcapture | 3.09 | 95.8 | 31.00 |
| CON_2_A_FP+25_LY_UCAM-0_TFcapture_mapping.proportion.dedup | TFcapture | 3.77 | 96.54 | 25.61 |
| CON_2_A_FP+25_LY_UOXC-0_TFcapture_mapping.proportion.dedup | TFcapture | 3.67 | 96.3 | 26.24 |
| CON_2_A_FP+25_LY_UOXZ-0_TFcapture_mapping.proportion.dedup | TFcapture | 4.07 | 96.49 | 23.71 |
| CON_2_A_FP+55_LY_ABBV-0_TFcapture_mapping.proportion.dedup | TFcapture | 2.97 | 95.89 | 32.29 |
| CON_2_A_FP+55_LY_JANS-0_TFcapture_mapping.proportion.dedup | TFcapture | 3.03 | 95.93 | 31.66 |
| CON_2_A_FP+55_LY_UCAM-0_TFcapture_mapping.proportion.dedup | TFcapture | 3.32 | 96.15 | 28.96 |
| CON_2_A_FP+55_LY_UOXC-0_TFcapture_mapping.proportion.dedup | TFcapture | 3.32 | 96.1 | 28.95 |
| CON_2_A_FP+55_LY_UOXZ-0_TFcapture_mapping.proportion.dedup | TFcapture | 3.63 | 96.52 | 26.59 |
| CON_3_A_FP+25_LY_ABBV-0_TFcapture_mapping.proportion.dedup | TFcapture | 3.17 | 96.35 | 30.39 |
| CON_3_A_FP+25_LY_JANS-0_TFcapture_mapping.proportion.dedup | TFcapture | 3.09 | 95.94 | 31.05 |
| CON_3_A_FP+25_LY_UCAM-0_TFcapture_mapping.proportion.dedup | TFcapture | 3.77 | 96.6 | 25.62 |
| CON_3_A_FP+25_LY_UOXC-0_TFcapture_mapping.proportion.dedup | TFcapture | 3.59 | 96.26 | 26.81 |
| CON_3_A_FP+25_LY_UOXZ-0_TFcapture_mapping.proportion.dedup | TFcapture | 3.94 | 96.66 | 24.53 |
| CON_3_A_FP+55_LY_ABBV-0_TFcapture_mapping.proportion.dedup | TFcapture | 3.05 | 96.27 | 31.56 |
| CON_3_A_FP+55_LY_JANS-0_TFcapture_mapping.proportion.dedup | TFcapture | 3.09 | 95.93 | 31.05 |
| CON_3_A_FP+55_LY_UCAM-0_TFcapture_mapping.proportion.dedup | TFcapture | 3.42 | 96.34 | 28.17 |
| CON_3_A_FP+55_LY_UOXC-0_TFcapture_mapping.proportion.dedup | TFcapture | 3.36 | 96.01 | 28.57 |
| CON_3_A_FP+55_LY_UOXZ-0_TFcapture_mapping.proportion.dedup | TFcapture | 3.43 | 96.32 | 28.08 |
| PS1_1_A_FP+25_LY_ABBV-0_TFcapture_mapping.proportion.dedup | TFcapture | 3.19 | 95.84 | 30.04 |
| PS1_1_A_FP+25_LY_JANS-0_TFcapture_mapping.proportion.dedup | TFcapture | 3.65 | 96.56 | 26.45 |
| PS1_1_A_FP+25_LY_UCAM-0_TFcapture_mapping.proportion.dedup | TFcapture | 3.21 | 96.43 | 30.04 |
| PS1_1_A_FP+25_LY_UOXC-0_TFcapture_mapping.proportion.dedup | TFcapture | 3.86 | 96.38 | 24.97 |
| PS1_1_A_FP+25_LY_UOXZ-0_TFcapture_mapping.proportion.dedup | TFcapture | 3.84 | 96.66 | 25.17 |
| PS1_1_A_FP+55_LY_ABBV-0_TFcapture_mapping.proportion.dedup | TFcapture | 3.45 | 96.15 | 27.87 |
| PS1_1_A_FP+55_LY_JANS-0_TFcapture_mapping.proportion.dedup | TFcapture | 3.22 | 95.98 | 29.81 |
| PS1_1_A_FP+55_LY_UCAM-0_TFcapture_mapping.proportion.dedup | TFcapture | 2.92 | 95.19 | 32.60 |
| PS1_1_A_FP+55_LY_UOXC-0_TFcapture_mapping.proportion.dedup | TFcapture | 3.59 | 96.13 | 26.78 |
| PS1_1_A_FP+55_LY_UOXZ-0_TFcapture_mapping.proportion.dedup | TFcapture | 3.41 | 96.58 | 28.32 |
| PS1_2_A_FP+25_LY_ABBV-0_TFcapture_mapping.proportion.dedup | TFcapture | 3.64 | 96.39 | 26.48 |
| PS1_2_A_FP+25_LY_JANS-0_TFcapture_mapping.proportion.dedup | TFcapture | 3.28 | 96.06 | 29.29 |
| PS1_2_A_FP+25_LY_UCAM-0_TFcapture_mapping.proportion.dedup | TFcapture | 3.36 | 96.42 | 28.70 |
| PS1_2_A_FP+25_LY_UOXC-0_TFcapture_mapping.proportion.dedup | TFcapture | 3.54 | 96.22 | 27.18 |
| PS1_2_A_FP+25_LY_UOXZ-0_TFcapture_mapping.proportion.dedup | TFcapture | 3.76 | 96.4 | 25.64 |
| PS1_2_A_FP+55_LY_ABBV-0_TFcapture_mapping.proportion.dedup | TFcapture | 3.36 | 96.39 | 28.69 |
| PS1_2_A_FP+55_LY_JANS-0_TFcapture_mapping.proportion.dedup | TFcapture | 3.59 | 96.08 | 26.76 |
| PS1_2_A_FP+55_LY_UCAM-0_TFcapture_mapping.proportion.dedup | TFcapture | 3.21 | 96.1 | 29.94 |
| PS1_2_A_FP+55_LY_UOXC-0_TFcapture_mapping.proportion.dedup | TFcapture | 3.57 | 96.14 | 26.93 |
| PS1_2_A_FP+55_LY_UOXZ-0_TFcapture_mapping.proportion.dedup | TFcapture | 3.46 | 96.33 | 27.84 |

| | | | | |
|---|---|---|---|---|
| PS1_3_A_FP+25_LY_ABBV-0_TFcapture_mapping.proportion.dedup | TFcapture | 3.13 | 96.05 | 30.69 |
| PS1_3_A_FP+25_LY_JANS-0_TFcapture_mapping.proportion.dedup | TFcapture | 3.18 | 96.07 | 30.21 |
| PS1_3_A_FP+25_LY_UCAM-0_TFcapture_mapping.proportion.dedup | TFcapture | 3.36 | 96.35 | 28.68 |
| PS1_3_A_FP+25_LY_UOXC-0_TFcapture_mapping.proportion.dedup | TFcapture | 3.83 | 96.33 | 25.15 |
| PS1_3_A_FP+55_LY_ABBV-0_TFcapture_mapping.proportion.dedup | TFcapture | 3.6 | 96.36 | 26.77 |
| PS1_3_A_FP+55_LY_JANS-0_TFcapture_mapping.proportion.dedup | TFcapture | 3.17 | 96.05 | 30.30 |
| PS1_3_A_FP+55_LY_UCAM-0_TFcapture_mapping.proportion.dedup | TFcapture | 3.04 | 95.83 | 31.52 |
| PS1_3_A_FP+55_LY_UOXC-0_TFcapture_mapping.proportion.dedup | TFcapture | 3.49 | 96.24 | 27.58 |

Mini-bulks

| Capture | Sample ID | Capture Panel | % on-target reads Pre-capture | % on-target reads Post-capture | Fold Enrichment |
|---|---|---|---|---|---|
| NFcapture850 | 227256 | NFcapture | 0.26 | 65.95 | 253.6538462 |
| NFcapture850 | 227280 | NFcapture | 0.27 | 71.8 | 265.9259259 |
| NFcapture850 | 228256 | NFcapture | 0.28 | 68.53 | 244.75 |
| NFcapture850 | 228280 | NFcapture | 0.26 | 71.64 | 275.5384615 |
| NFcapture850 | 255256 | NFcapture | 0.24 | 66.06 | 275.25 |
| NFcapture850 | 255280 | NFcapture | 0.22 | 67.82 | 308.2727273 |
| NFcapture850 | 256256 | NFcapture | 0.23 | 66.68 | 289.9130435 |
| NFcapture850 | 256280 | NFcapture | 0.23 | 67.33 | 292.7391304 |
| TFcapture150 | 227208 | TFcapture | 1.47 | 91.02 | 61.91836735 |
| TFcapture150 | 227232 | TFcapture | 1.4 | 88.89 | 63.49285714 |
| TFcapture150 | 228208 | TFcapture | 1.56 | 91.05 | 58.36538462 |
| TFcapture150 | 228232 | TFcapture | 1.42 | 88.89 | 62.59859155 |
| TFcapture150 | 255208 | TFcapture | 0.69 | 81.49 | 118.1014493 |
| TFcapture150 | 255232 | TFcapture | 1.39 | 87.55 | 62.98561151 |
| TFcapture150 | 256208 | TFcapture | 0.9 | 81.84 | 90.93333333 |
| TFcapture150 | 256232 | TFcapture | 1.51 | 86.75 | 57.45033113 |
| TFcapture850 | 227208 | TFcapture | 1.47 | 89.59 | 60.94557823 |
| TFcapture850 | 227232 | TFcapture | 1.4 | 87.8 | 62.71428571 |
| TFcapture850 | 228208 | TFcapture | 1.56 | 89.15 | 57.1474359 |
| TFcapture850 | 228232 | TFcapture | 1.42 | 87.28 | 61.46478873 |
| TFcapture850 | 255208 | TFcapture | 0.69 | 77.29 | 112.0144928 |
| TFcapture850 | 255232 | TFcapture | 1.39 | 87 | 62.58992806 |
| TFcapture850 | 256208 | TFcapture | 0.9 | 78.42 | 87.13333333 |
| TFcapture850 | 256232 | TFcapture | 1.51 | 85.6 | 56.68874172 |

| Capture | % on-target reads Pre-capture (median) | % on-target reads Post-capture (median) | Fold Enrichment (median) | Max possible fold enrichment | Capture Efficiency (% of possible fold enrichment observed) |
|---|---|---|---|---|---|
| Bulk NG | 0.53 | 86.715 | 164.88 | 188.68 | 87.39 |
| Bulk TF | 3.365 | 96.25 | 28.56 | 29.72 | 96.10 |
| Mini-bulk NG | 0.25 | 67.575 | 275.39 | 400.00 | 68.85 |
| Mini-bulk TF 150ng | 1.41 | 88.22 | 62.79 | 70.92 | 88.54 |
| Mini-bulk TF 850ng | 1.41 | 87.14 | 62.03 | 70.92 | 87.46 |
