# Supplementary material for: Targeted RNA sequencing enhances gene expression profiling of ultra-low input samples
Source: RNA Biol. 2020 Jun 28;17(12):1741–53. doi: 10.1080/15476286.2020.1777768 (PMC7746246; doi:10.1080/15476286.2020.1777768)
Supplement: Supplemental Material [file KRNB_A_1777768_SM6200.zip › TableS8_Gene_capture_enrichments.pdf]

RAW_avePre	The mean of the gene RAW COUNTS in the libraries assayed Pre-capture
RAW_avePost	The mean of the gene RAW COUNTS in the libraries assayed Post-capture
RAW_ratio	RAW_avePost/ RAW_avePre
CPM_avePre	The mean of the gene CPM in the libraries assayed Pre-capture
CPM_avePost	The mean of the gene CPM in the libraries assayed Post-capture
CPM_ratio	This was used as the enrichment factor (EF): CPM_avePost/ CPM_avePre

NG Mini-bulk

Gene	RAW_avePre	RAW_avePost	RAW_ratio	CPM_avePre	CPM_avePost	CPM_ratio
ACMSD	0.625	14.25	22.8	1.31170922	60.7047175	46.2790965
AIF1	0.375	38.5	102.6667	0.79924059	162.150358	202.880535
AKT3	67.75	7927	117.0037	139.631594	32918.0035	235.748963
ALDH1L1	1.125	75.875	67.44444	1.91748634	278.392592	145.186219
ANK3	29	3412.25	117.6638	60.8240069	14065.2831	231.245586
ASPHD1	6.875	462.125	67.21818	14.2639836	1929.97944	135.304379
ATPAF2	27.875	1930.75	69.26457	55.8484946	7904.44241	141.53367
AVP	0	3.5		0	15.1201507	
BAG3	7	675.25	96.46429	13.4740169	2635.64004	195.609079
BCKDK	77.625	5433	69.99034	152.875849	21682.1244	141.828317
BST1	0.25	44.875	179.5	0.54355152	169.411845	311.675781
C4orf27	30.625	3346.125	109.2612	64.1765393	14080.044	219.395501
CACNA1C	4.875	320.375	65.71795	10.6914209	1368.04316	127.957095
CACNA1I	1.125	70.875	63	2.31735172	288.525831	124.506707
CACNB2	3.25	377.5	116.1538	7.01168409	1594.95139	227.470514
CCDC62	0.125	45.75	366	0.2685677	185.589343	691.033754
CLCN3	48.125	5513.125	114.5584	95.2267865	22413.0974	235.36547
CNKSR2	17.75	1938.625	109.2183	36.0941829	8107.83407	224.629938
CNTN4	7.125	915.5	128.4912	15.5115911	3893.97644	251.036558
CRHR1	0	2.125		0	8.04899699	
DAO	0	6		0	24.4258046	
DCUN1D1	18.875	3002.375	159.0662	40.2388341	12724.2203	316.21742
DGKQ	3.5	231.25	66.07143	7.61218654	1005.02503	132.028429
EMX1	8.5	549.75	64.67647	16.191887	2153.50947	132.99929
FAM126A	33.5	3271.5	97.65672	66.8597567	13198.1035	197.399813
FAM47E	0.5	43.625	87.25	1.06457704	168.570411	158.344962
FOXA2	0	0		0	0	
FOXQ1	0	0.125		0	0.55667633	
FXR1	33.25	4090.875	123.0338	68.0580351	16980.8327	249.505185
GAD2	50.75	4307.25	84.87192	113.763814	18969.5686	166.745189
GAK	22.75	1805.5	79.36264	46.8689232	7519.78534	160.442887
GBA	18.5	1620.625	87.60135	38.3472317	6762.01899	176.336562
GCH1	0.125	29.875	239	0.26533645	124.975875	471.009077
GPNMB	7.125	757.5	106.3158	13.2595222	2953.88592	222.774688
GRIA1	99	9777.125	98.75884	206.156064	40836.818	198.086912
GRIN2A	2.875	308.75	107.3913	5.19187334	1162.58823	223.924613
GRM3	30.625	2857.25	93.29796	63.1953055	11957.7455	189.218889
GUSB	24.375	1976.75	81.09744	48.6980164	8125.85699	166.862176
HCN1	1.625	231.375	142.3846	3.33878367	945.01017	283.040252
HIP1R	19.75	1397	70.73418	40.557269	5788.05968	142.71325
HLA-DQA1	0	3.125		0	13.1283701	
HLA-DQA2	0	1.5		0	6.04782621	
HLA-DQB1	1.875	59.375	31.66667	3.65817414	251.307511	68.6975254
HLA-DQB2	0.875	52.375	59.85714	1.91244919	224.006521	117.130705
HSD3B7	7.25	440.75	60.7931	13.4479356	1708.61158	127.05382
IGSF9B	1.875	134.125	71.53333	3.89487542	547.256386	140.506775
KANSL1	15.125	1468.375	97.08264	31.5843836	6179.79254	195.659748
KAT8	23.5	1908.625	81.21809	50.2588946	7954.44169	158.269332
KCNB1	4.25	526.625	123.9118	8.45877315	2060.31103	243.570905
KCNJ6	4	411.375	102.8438	7.66016079	1623.77024	211.976

KCTD13	31.25	2554.25	81.736	64.2378959	10501.4536	163.477547
KLHL7-AS1	3	251.375	83.79167	5.76413531	1019.29306	176.833645
LAMP3	0	0.125		0	0.52333444	
LINC01470	0.125	17.125	137	0.23104549	68.3273611	295.731205
LRRC37A	0.125	17.75	142	0.25611553	76.4991013	298.689823
LRRK2	0	4		0	18.0076542	
MALSU1	18.875	1809.75	95.88079	38.8612571	7516.801	193.426604
MCCC1	24.625	2221.125	90.19797	50.6527165	9301.08093	183.624523
MIOX	0	0		0	0	
MIR4697HG	4.25	46	10.82353	8.46210705	192.03503	22.693524
MKI67	6	569.25	94.875	12.6089704	2385.06492	189.1562
NANOG	0	0		0	0	
NEK1	13.75	1924.25	139.9455	28.8950142	8224.0201	284.617271
NLGN4X	29.75	3387.875	113.8782	62.483666	14197.9965	227.227329
NMUR2	0	0		0	0	
NUPL2	12.625	1254.375	99.35644	25.7963471	5259.22057	203.874624
OTX1	24.5	2000.5	81.65306	48.1802453	8077.01791	167.641693
OXT	0	0		0	0	
PAK6	5	356.375	71.275	9.87089316	1406.03004	142.442028
PITX3	0	0		0	0	
RAB29	10.625	1104.625	103.9647	20.5385667	4391.27682	213.806392
RAI1	13.75	1138.125	82.77273	30.0863877	4782.65611	158.964119
RIMS1	8.125	763.5	93.96923	17.8054776	3248.66341	182.453034
RIT2	2.25	259.5	115.3333	4.83620659	1087.58477	224.883853
RPS19BP1	37.25	2731.875	73.33893	77.7535105	11462.3913	147.419599
SATB2	1.125	176.625	157	2.28483606	746.296142	326.630061
SATB2-AS1	0	0		0	0	
SCARB2	86.875	9281.75	106.8403	180.347403	38686.9389	214.513424
SDCCAG8	24.375	1861.125	76.35385	49.714724	7709.83603	155.081541
SIPA1L2	17.875	1632.875	91.34965	38.2868816	6926.24465	180.90386
SLC41A1	21.375	2100.875	98.28655	41.7376218	8453.70893	202.544097
SMG6	8.5	859.875	101.1618	17.5676767	3508.11843	199.691655
SNAP91	27.625	3071.75	111.1946	56.4028104	12635.7733	224.027371
SNCA	68.625	6044.625	88.08197	135.216996	24282.4087	179.581039
SPPL2C	0	0		0	0	
SREBF1	6.25	406.5	65.04	12.7319462	1692.07035	132.899584
SRR	25.875	2492	96.30918	52.7305006	10273.7771	194.83557
STBD1	1.125	82.375	73.22222	1.96554357	311.732045	158.598389
STH	0.25	24.375	97.5	0.52789129	102.443556	194.061843
STK39	9.375	1048.25	111.8133	20.4839585	4517.0577	220.516835
STX4	10	722.625	72.2625	19.8670285	2983.25213	150.160963
TENM4	13.75	1370.625	99.68182	27.1724188	5602.05831	206.167082
TMEM163	8	854.375	106.7969	16.5494037	3552.186	214.641329
TMEM175	14.5	897.75	61.91379	30.5207344	3695.29886	121.075031
TOM1L2	12.25	1141.25	93.16327	25.8491638	4758.54264	184.088842
TRANK1	0.625	71.125	113.8	1.55538681	322.42326	207.294583
TRIM72	0	1		0	4.45341064	
UBOX5	14.5	1257	86.68966	29.5803211	5199.3089	175.769184
VPS13C	11.875	1489.875	125.4632	24.1889782	6210.45485	256.7473
ZNF646	12.875	948.25	73.65049	27.3652347	4034.94299	147.447776
ZNF804A	5	679.25	135.85	10.8152201	2940.65619	271.899801

NG Bulk

Gene	RAW_avePre	RAW_avePost	RAW_ratio	CPM_avePre	CPM_avePost	CPM_ratio
ACMSD	4.77586207	131.672414	27.5703971	0.28405467	45.4696695	160.073657
AIF1	3.82758621	151.12069	39.481982	0.24054461	62.7553838	260.888754
AKT3	3392.77586	93319.3621	27.505313	217.441411	36202.8214	166.494603
ALDH1L1	73.2586207	2672.81034	36.4845846	4.54960535	991.249069	217.875836
ANK3	2157.27586	90539.5862	41.9694059	138.401467	33301.4992	240.615218
ASPHD1	535.810345	10737.7931	20.040287	32.8393983	3942.27723	120.047182
ATPAF2	343.5	8488	24.7103348	21.6142757	3276.95508	151.610682
AVP	1.37931034	10.4827586	7.6	0.08820316	3.71750488	42.1470703
BAG3	428.706897	13512.5862	31.5194048	26.0783884	5223.67574	200.306693
BCKDK	1058.7931	20393.569	19.2611464	65.7041917	7794.5946	118.631618
BST1	12.4655172	547.017241	43.8824343	0.7954587	211.124951	265.412837
C4orf27	419.448276	12468.8448	29.7267757	27.5765825	5007.08491	181.570175
CACNA1C	771.224138	21078.5517	27.3312915	48.3419879	7535.88434	155.886935
CACNA1I	445.37931	11987.4828	26.9152214	26.6615508	4110.96205	154.190658
CACNB2	133.862069	5629.31034	42.0530654	8.64397885	2088.20493	241.579134
CCDC62	26.1724138	1164.43103	44.4907773	1.56958821	408.961233	260.553202
CLCN3	2084.55172	63484.6207	30.4548071	137.769996	25334.6544	183.890942
CNKSR2	476.931034	18045.4655	37.8366351	30.4512947	6769.86216	222.317712
CNTN4	190.948276	9247.67241	48.4302483	12.243876	3465.82656	283.066126
CRHR1	5.63793103	101.224138	17.9541284	0.34298931	36.2300146	105.630156
DAO	4.4137931	138.948276	31.4804688	0.26366507	47.3955497	179.756651
DCUN1D1	374.465517	15574.0517	41.5900824	24.9406682	6170.22165	247.396004
DGKQ	1493.27586	24024.7069	16.0885925	86.8729119	8315.35165	95.7185786
EMX1	698.810345	7670.82759	10.9769806	38.9489434	2657.22025	68.2231664
FAM126A	631.5	27555.9483	43.6357059	40.9905525	10664.7079	260.174779
FAM47E	15.7413793	648.172414	41.1763417	0.96240099	250.389457	260.171653
FOXA2	0.36206897	12.1551724	33.5714286	0.02051452	4.41485014	215.206127
FOXQ1	6	196.534483	32.7557471	0.35911567	85.4471934	237.937806
FXR1	1975.27586	47664.7241	24.130667	123.073894	18584.2792	151.000985
GAD2	1487.24138	30017.0862	20.1830628	95.001619	11465.8211	120.690797
GAK	2023.7069	37839.4828	18.6981044	122.310932	13798.3982	112.814105
GBA	289.241379	8134.75862	28.1244635	18.4678414	3170.781	171.69202
GCH1	42.0862069	1751.7931	41.6239246	2.70322353	681.982022	252.284731
GPNMB	494.551724	11408.3448	23.0680519	32.8695912	5744.09792	174.754164
GRIA1	4376.77586	98763.8966	22.5654454	291.660544	39038.9098	133.850501
GRIN2A	162.327586	7225.94828	44.5146044	11.7512186	3131.88654	266.515896
GRM3	1148.89655	35734.3621	31.1032025	77.2339639	14087.3942	182.398954
GUSB	723.051724	16754.7414	23.1722584	43.2836102	6197.66766	143.187401
HCN1	98.2068966	3912.51724	39.8395365	6.37264656	1435.22678	225.216755
HIP1R	3938.82759	52383.2414	13.2991963	228.380646	18197.9776	79.6826608
HLA-DQA1	2.22413793	62.362069	28.0387597	0.14753822	27.9693341	189.573483
HLA-DQA2	1.10344828	31.6896552	28.71875	0.07498542	13.5352426	180.504983
HLA-DQB1	17.2931034	367.37931	21.2442672	1.06440152	147.158785	138.254956
HLA-DQB2	13.5862069	206.844828	15.2246193	0.85762893	78.7743931	91.8513705
HSD3B7	197.862069	4543.27586	22.9618334	11.6330814	1643.14145	141.247309
IGSF9B	713.086207	17571.2586	24.6411422	43.468575	6202.83517	142.696998
KANSL1	1734.25862	44391.2241	25.5966576	106.026958	16265.9948	153.413765
KAT8	1295.48276	22550.4828	17.4070111	78.6031919	8420.85018	107.131148
KCNB1	275.62069	10015.4483	36.3377956	16.8391821	3598.48644	213.697222
KCNJ6	49.8103448	2171.55172	43.5964001	3.04488431	774.504114	254.362411

KCTD13	1336.60345	22465.5517	16.8079409	82.5770674	8361.57793	101.25787
KLHL7-AS1	30.7068966	779.344828	25.3801235	1.91770362	295.776977	154.234979
LAMP3	9.0862069	462.793103	50.9335863	0.57656807	178.36269	309.352357
LINC01470	1.18965517	56.6896552	47.6521739	0.07623691	21.1323446	277.193076
LRRC37A	19.6724138	428.655172	21.7896582	1.16967407	151.865321	129.835588
LRRK2	20.6724138	966.051724	46.7314429	1.34861657	359.730205	266.740164
MALSU1	315.672414	7299.12069	23.1224534	19.9095074	2906.18439	145.969679
MCCC1	374.913793	13327.569	35.5483559	23.8650941	5021.14053	210.396846
MIOX	3.74137931	107.844828	28.8248848	0.21379447	38.3221687	179.247706
MIR4697HG	926.844828	4000.74138	4.31651692	58.7189727	1476.31064	25.1419698
MKI67	1584.58621	67780.2069	42.7747046	95.4948022	23778.4029	249.002065
NANOG	0.0862069	4.18965517	48.6	0.00447541	1.54318544	344.814665
NEK1	395.706897	17795.1897	44.9706331	25.3907679	6652.24124	261.994489
NLGN4X	1600.05172	52645.0345	32.9020829	103.87727	20411.0711	196.492179
NMUR2	0.32758621	12.862069	39.2631579	0.01851678	4.50449205	243.265376
NUPL2	259.224138	9731.27586	37.5400067	16.7895846	3787.17111	225.566695
OTX1	542.844828	16507.6897	30.4095919	31.4570978	5990.92654	190.447529
OXT	1.55172414	22.4655172	14.4777778	0.09716316	8.62353347	88.7531214
PAK6	1369.41379	32848.8448	23.987523	87.6306596	12307.8267	140.451148
PITX3	1.51724138	32.1206897	21.1704545	0.10128083	13.7094384	135.360641
RAB29	226.103448	8012.60345	35.4377764	14.749786	3196.83012	216.737391
RAI1	2992.44828	63314.8103	21.1581971	178.6462	22536.4365	126.151222
RIMS1	384.310345	13749.6034	35.7773441	24.3492887	5023.72692	206.319248
RIT2	30.8448276	1435.75862	46.5477921	2.03712365	541.433269	265.783213
RPS19BP1	972.034483	14528.1724	14.9461492	62.5569006	5917.0282	94.586339
SATB2	148.965517	5768	38.7203704	9.58086593	2210.89068	230.76105
SATB2-AS1	1.55172414	72.7413793	46.8777778	0.11105664	30.0255591	270.362575
SCARB2	5694.89655	96818.9138	17.0009961	374.593022	39397.3633	105.173778
SDCCAG8	230.672414	7417	32.1538232	14.458463	2753.51069	190.442836
SIPA1L2	1668.96552	52888.4483	31.6893595	105.675242	20047.9783	189.713106
SLC41A1	1045.94828	33330.3103	31.8661172	64.3549462	12640.0781	196.411914
SMG6	1371.53448	43807.5862	31.9405649	85.1648157	16296.6237	191.353947
SNAP91	1848.46552	48184.069	26.067064	120.154231	18477.259	153.779513
SNCA	1272.89655	30395.7931	23.8792328	81.2929227	11841.5091	145.664699
SPPL2C	11.1206897	330.344828	29.7054264	0.67887545	119.800903	176.469635
SREBF1	1979.62069	26850.3448	13.5633786	116.937957	9827.12244	84.0370628
SRR	245.655172	9845.96552	40.0804323	16.2221296	3855.59712	237.675152
STBD1	37.8103448	780.310345	20.6374829	2.33136439	316.818499	135.894029
STH	2.55172414	71.9655172	28.2027027	0.15583309	25.0697309	160.875528
STK39	1130.36207	32486.7759	28.7401504	72.6927799	12796.4367	176.034493
STX4	562.87931	9933.98276	17.6485129	34.2229992	3732.01793	109.049996
TENM4	2211.43103	68365.7414	30.9147065	138.57474	25238.6959	182.130567
TMEM163	396.224138	11627.3276	29.3453287	25.2437118	4455.90678	176.515514
TMEM175	1196.58621	19230.0345	16.0707472	70.0464081	6667.19011	95.1824696
TOM1L2	1763.58621	42093.9483	23.8683814	106.981716	15253.6474	142.581817
TRANK1	206.706897	9164.05172	44.3335558	12.9567404	3258.41593	251.484234
TRIM72	7.05172414	102.37931	14.5183374	0.45316048	37.1513749	81.9828215
UBOX5	285.5	9061.82759	31.7402017	17.3826423	3313.4267	190.616976
VPS13C	875.931034	40281.7414	45.9873435	54.5766077	14651.8373	268.463686
ZNF646	828.655172	23844.5172	28.7749573	50.5871024	8646.68975	170.926765
ZNF804A	313	12738.4138	40.6978076	21.642673	5004.45901	231.231097

TF mini-bulk 150ng

Gene	RAW_avePre	RAW_avePost	RAW_ratio	CPM_avePre	CPM_avePost	CPM_ratio
AEBP1	1.5	79.125	52.75	4.41820538	172.1384872	38.9611782
AEBP2	8	242.5	30.3125	111.066366	2310.95552	20.8069788
AHR	3.125	156.375	50.04	9.96931878	333.0062588	33.4031107
AIRE	0	0		0	0	
AKNA	3	158.375	52.7916667	7.77354245	285.9211989	36.781326
ALX1	0.125	20.625		0.27984365	40.92096649	146.227964
ALX3	0.125	12.75		0.35939162	23.23763091	64.6582432
ALX4	0.125	0		0.27984365	0	0
AR	0.375	16.5	44	1.31684328	34.01005853	25.8269599
ARHGAP35	34	2066.375	60.7757353	87.1477992	3783.149047	43.4107239
ARID3A	0.375	14.125	37.6666667	1.07248816	23.38353465	21.8030701
ARID3B	1.875	130.25	69.4666667	5.55892141	244.6596802	44.0120775
ARID3C	0	2.75		0	6.001406488	
ARID5A	5.125	250.75	48.9268293	19.8632282	554.3258888	27.90714
ARID5B	12.5	770.5	61.64	37.2060592	1571.127929	42.2277436
ARNT	14.875	787.75	52.9579832	64.7577703	2177.072837	33.6187121
ARNTL	4.75	296.125	62.3421053	13.6350067	587.5517203	43.0914142
ARNTL2	0.75	44	58.6666667	1.94869808	96.96726342	49.760024
ARX	9.75	449.375	46.0897436	24.0035924	808.7724166	33.6938073
ASCL2	0	4.25		0	9.141194703	
ASCL3	0	0		0	0	
ATF1	2.25	174.5	77.5555556	6.33087383	346.4241356	54.7197977
ATF2	27.25	1882	69.0642202	77.523118	3695.541838	47.670191
ATF3	5.25	272.375	51.8809524	16.7673679	578.3526136	34.492749
ATF5	1.5	106.25	70.8333333	4.1124468	224.7605419	54.6537264
ATF6	21.25	1364	64.1882353	61.2449559	2685.395676	43.8468056
ATF6B	13.125	674.875	51.4190476	37.8072036	1291.532169	34.1610076
ATF7	10.75	712.75	66.3023256	31.2770015	1429.011129	45.6888788
ATOH1	0	4.375		0	10.45592618	
ATOH7	1	79.25	79.25	2.92492212	155.8181024	53.2725645
BACH1	11.375	717.5	63.0769231	72.8893364	2497.989331	34.2709847
BACH2	20.875	1389.5	66.5628743	58.5589544	2691.76955	45.9668309
BARHL1	0	0		0	0	
BARHL2	0	6.25		0	12.86654985	
BARX1	0	0		0	0	
BARX2	0	0		0	0	
BATF	0	0		0	0	
BATF3	0.25	8.25	33	0.70240432	15.7612498	22.4389991
BBX	19.75	1116.25	56.5189873	57.1366131	2247.404529	39.3338773
BCL11B	23	1428.5	62.1086957	61.0931438	2598.866413	42.539412
BCL6	7.625	459.25	60.2295082	20.7959921	854.0970005	41.0702696
BCL6B	0	4.25		0	6.999440686	
BHLHA15	0	0		0	0	
BHLHA9	0	0		0	0	
BHLHE22	4	270.625	67.65625	12.8468438	597.5369518	46.5123543
BHLHE23	0	0.125		0	0.275232792	
BHLHE40	11.5	665.75	57.8913043	37.262819	1489.942034	39.9846838
BHLHE41	1.125	47.75	42.4444444	3.85757747	109.9200503	28.4945801
BMPR1A	4.875	578	118.564103	15.9541684	1152.282599	72.2245478
BNC1	0	0		0	0	

BNC2	0.25	7.375	29.5	0.75395904	16.82643362	22.3174374
BRIP1	2.5	158.5	63.4	7.67467375	314.5282812	40.9826256
BSX	0	0		0	0	
BTBD11	2.375	116.625	49.1052632	6.48699554	223.7929833	34.4987108
BTBD3	10.125	661.875	65.3703704	29.3831085	1387.228447	47.2117661
BTG2	19.875	1282	64.5031447	99.5845482	3172.095138	31.8532864
CAMTA1	23.125	1241	53.6648649	62.6542633	2401.759898	38.333543
CARF	9.125	291.75	31.9726027	142.685174	3706.361649	25.9758007
CASZ1	1	51.125	51.125	3.18502503	101.1073897	31.7446138
CC2D1A	1.75	99.625	56.9285714	4.37358662	188.4766356	43.0942958
CC2D1B	2.25	116.375	51.7222222	6.16191176	235.4456266	38.2098342
CDC5L	28.25	1704.25	60.3274336	77.4975978	3240.247239	41.8109377
CDX1	0	0		0	0	
CDX2	0	0		0	0	
CDX4	0	0		0	0	
CEBPA	0.875	31.875	36.4285714	2.49880582	62.91002515	25.1760359
CEBPB	0.125	19		0.27984365	37.35322063	133.478895
CEBPD	0.25	12.25	49	0.73637114	26.24586138	35.6421645
CEBPE	0	0		0	0	
CEBPG	25.875	1666.375	64.4009662	73.2154632	3294.677523	44.9997498
CEBPZ	13.375	779.125	58.2523364	39.9751814	1538.403189	38.4839577
CGGBP1	28	1810.75	64.6696429	79.4725959	3481.863669	43.8121296
CHCHD3	24.75	1468.375	59.3282828	69.0299237	2798.346042	40.5381593
CIC	5	185.25	37.05	44.8327915	1105.968233	24.6687345
CLOCK	1.75	181.25	103.571429	5.17240859	352.9660536	68.2401723
CNOT3	2.25	115.5	51.3333333	6.39223051	210.5310078	32.935453
CREB1	25.375	1592.5	62.7586207	79.1974495	3250.64081	41.0447663
CREB3L1	0.125	5.75		0.37697952	12.09000233	32.0707139
CREB3L2	3	139.625	46.5416667	8.33712879	283.7169927	34.0305398
CREB3L3	0	0		0	0	
CREB3L4	8	349.25	43.65625	23.3662493	685.2123862	29.324877
CREB5	15.75	864.75	54.9047619	85.4494688	2722.72542	31.8635734
CREBBP	6.625	418.375	63.1509434	17.129177	769.6207762	44.9304002
CREBL2	3.125	333.5	106.72	9.02804844	660.8385663	73.1983851
CREBRF	2.375	189.75	79.8947368	6.42261753	353.6271267	55.0596584
CREM	7.625	391.625	51.3606557	24.3060108	810.9828099	33.365525
CRX	0.375	3.25	8.66666667	1.40979564	7.767259448	5.50949318
CSRNP1	4.125	222.125	53.8484848	12.2504018	458.8504248	37.455949
CSRNP2	25	1840	73.6	66.934328	3392.45445	50.6833273
CSRNP3	34.5	1525.25	44.2101449	104.396336	3352.453919	32.1127547
CTBP2	18	629.5	34.9722222	48.573856	1198.237743	24.6683678
CTCF	13.5	877.875	65.0277778	36.3643597	1606.380424	44.1745829
CTCFL	0	0		0	0	
CTNNBL1	34.375	1972.25	57.3745455	91.9326482	3712.43775	40.3821474
CUX1	13.5	717.75	53.1666667	37.8714082	1346.608015	35.557379
CUX2	0.625	55.125	88.2	1.48495622	95.68270136	64.4346954
DACH1	7.125	373.25	52.3859649	21.6490431	776.4368244	35.864718
DACH2	3.75	232.375	61.9666667	10.2152985	443.0178728	43.3680791
DBP	1.625	83.375	51.3076923	5.37171739	194.0507774	36.1245321
DBX1	0	3.375		0	7.431285381	
DBX2	0	0		0	0	
DDIT3	13.5	517.5	38.3333333	39.2958755	1036.17878	26.3686396

DDN	0.5	55.125	110.25	1.43998443	100.904856	70.0735745
DEAF1	15.875	891.25	56.1417323	44.2737	1705.548157	38.5228286
DENND4A	4.875	292.5	60	13.5271479	593.0057612	43.838196
DIDO1	14.375	920.5	64.0347826	40.3788741	1806.81987	44.746663
DLX2	37.75	1729	45.8013245	91.083006	2926.120117	32.1258624
DLX3	0.25	13.25	53	0.75395904	29.15910733	38.6746571
DLX6	29.25	1619.125	55.3547009	68.1108439	2677.956586	39.3176245
DMBX1	1	35.5	35.5	2.87513297	75.30769021	26.1927677
DMRT1	0.25	10.25	41	0.75395904	26.74549113	35.4734007
DMRT2	0	2.375		0	4.164350703	
DMTF1	12.375	761.875	61.5656566	65.5350153	1804.096197	27.5287369
DPF1	14	650.125	46.4375	38.1552351	1176.871559	30.844301
DPF3	0.5	47.75	95.5	1.27847054	103.8413583	81.2231142
DRGX	0	0		0	0	
DUX4	0	0		0	0	
E2F1	6.125	383	62.5306122	18.1446932	699.5746987	38.5553337
E2F2	3.875	243.625	62.8709677	9.59902007	434.1200353	45.2254534
E2F3	3.875	250.25	64.5806452	10.5276836	492.9409592	46.823307
E2F4	3.125	217.625	69.64	8.75243239	428.751126	48.9865111
E2F5	1	34	34	2.92758424	65.64614351	22.423315
E2F6	3.75	178.625	47.6333333	11.9789904	371.3940905	31.003789
E2F7	3.625	138.25	38.137931	9.44629028	248.5104327	26.3077277
E2F8	0.375	29.375	78.3333333	1.09278822	71.9778229	65.8662142
E4F1	15.125	623.125	41.1983471	39.1980054	1116.072585	28.4726881
EAF2	1.125	74.75	66.4444444	3.17314236	143.0641074	45.0859405
EBF1	11.125	682.875	61.3820225	26.4946651	1139.961725	43.0260855
EBF2	0.125	27.125	217	0.30127814	42.66010317	141.597073
EBF3	0.375	34.375	91.6666667	1.29925538	73.59610909	56.6448369
EBF4	0.625	49	78.4	1.73202349	99.32389443	57.3455818
EED	6.125	349.625	57.0816327	15.9992347	631.9917138	39.5013714
EGR1	37.625	1678.25	44.6046512	97.7697591	2997.485754	30.6586186
EGR2	0.125	8.5	68	0.46993188	20.00699769	42.5742509
EGR3	1.25	59.5	47.6	2.88417444	99.17675115	34.38653
EGR4	0	0		0	0	
EHF	0	0.625		0	1.376163959	
ELF1	1.75	133.75	76.4285714	4.88486579	261.9445258	53.6236894
ELF2	11.5	544.5	47.3478261	31.7689325	1037.130975	32.6460757
ELF3	0	0.125		0	0.219176353	
ELF4	0.75	59.75	79.6666667	1.98593042	109.2660128	55.020061
ELF5	0	0		0	0	
ELK1	8.75	653.75	74.7142857	23.3145199	1191.251565	51.0948357
ELK3	0	35.75		0	76.72495457	
ELK4	2.125	117.375	55.2352941	6.44519212	235.7815182	36.582543
EMX1	6.25	306.75	49.08	18.5502657	631.2969646	34.031694
EMX2	3.375	199.75	59.1851852	10.2307669	420.4710055	41.0986789
EN1	0.125	2.75	22	0.27984365	4.48434915	16.0244809
EN2	0	1.375		0	3.027560711	
EOMES	9.25	646.75	69.9189189	28.2754832	1392.790035	49.2578685
EP300	9.125	586.375	64.260274	24.7576917	1103.215542	44.5605169
EPAS1	1.75	85.875	49.0714286	5.64952271	189.8743162	33.6089128
ERF	0.375	24	64	1.12675504	49.3125332	43.7650876
ERG	0	0		0	0	

ESR1	0	0		0	0	
ESR2	0.625	44.125	70.6	1.70729574	85.97546639	50.357688
ESRRA	0.75	31.25	41.6666667	2.17722762	65.42379704	30.0491305
ESRRG	7.125	426.5	59.8596491	17.4582803	714.6008729	40.9319165
ESX1	0	0		0	0	
ETS1	0.125	1.375	11	0.27984365	7.184590964	25.6735898
ETS2	2.5	160.25	64.1	6.89772955	318.5753966	46.1855447
ETV1	13.125	648.375	49.4	35.7764671	1235.253115	34.526973
ETV2	2.375	59.375	25	28.6078251	516.3709341	18.0499892
ETV3	2.125	148.625	69.9411765	5.77181798	278.5513894	48.2605984
ETV4	0.875	97	110.857143	2.51576584	200.2566361	79.600666
ETV5	4.75	433.875	91.3421053	12.9135056	840.6545772	65.0988664
ETV6	1.875	200.5	106.933333	4.97348676	371.3617114	74.6682819
ETV7	0.25	12.25	49	0.62285634	21.80159486	35.0026057
FAM170A	0	0		0	0	
FERD3L	0	0		0	0	
FEV	0	0.25		0	0.597481496	
FEZF1	3.5	284.75	81.3571429	11.2477649	528.0381552	46.9460518
FEZF2	18.375	924.375	50.3061224	55.2148259	1899.921331	34.4096228
FIGLA	0	0		0	0	
FLI1	0	3.625		0	6.356114231	
FOS	8.125	459.625	56.5692308	24.1099082	923.8215214	38.31709
FOSL1	0.125	6.375	51	0.37697952	14.02130378	37.1938078
FOSL2	3.375	149	44.1481481	10.1396087	317.3730921	31.3003293
FOXA1	0	0		0	0	
FOXA2	0	0		0	0	
FOXA3	0	1.625		0	6.673062507	
FOXB1	0.125	8.5	68	0.37697952	19.2800208	51.1434171
FOXB2	0	0		0	0	
FOXC1	0	1.25		0	2.98740748	
FOXC2	0	0		0	0	
FOXD1	0	0.125		0	0.298740748	
FOXD2	0	0		0	0	
FOXD3	0	0		0	0	
FOXD4	0	1.75		0	4.089073491	
FOXD4L1	0	3.25		0	5.621428879	
FOXD4L3	0	0		0	0	
FOXD4L5	0	0		0	0	
FOXD4L6	0	0		0	0	
FOXE1	0	0		0	0	
FOXE3	0	0		0	0	
FOXF1	0	1		0	1.753410822	
FOXF2	0	0		0	0	
FOXG1	38.25	2293.375	59.9575163	116.400848	4628.486038	39.7633361
FOXH1	0	0		0	0	
FOXI1	0	0		0	0	
FOXI2	0	0		0	0	
FOXJ1	0	6.875		0	13.99498009	
FOXJ2	6.5	428.375	65.9038462	17.4911096	836.6552412	47.8331712
FOXJ3	28.125	1384.75	49.2355556	198.365215	5312.248268	26.7801402
FOXK1	4.25	283.125	66.6176471	12.3177302	589.8175204	47.8836207
FOXK2	14.5	807.5	55.6896552	38.8037589	1532.465242	39.4927009

FOXL1	0	0		0	0	
FOXL2	0	0		0	0	
FOXM1	5.125	312.75	61.0243902	13.6761017	599.4350001	43.8308381
FOXN1	0	1		0	2.389925984	
FOXN2	7.125	445.625	62.5438596	21.5966386	925.597925	42.8584255
FOXN3	12	889.75	74.1458333	30.1784908	1634.706284	54.1679269
FOXN4	0.375	25.25	67.3333333	1.17233619	58.833905	50.1851817
FOXO1	1.375	143.75	104.545455	3.30402296	261.9005778	79.2671786
FOXO3	13.375	880.75	65.8504673	36.0920682	1680.754647	46.5685324
FOXO4	0.25	9.125	36.5	0.75395904	18.353427	24.3427375
FOXO6	0.125	7.625	61	0.27984365	14.43003004	51.5646156
FOXP1	29.125	1711.625	58.7682403	74.6802192	3020.671453	40.4480797
FOXP2	6.75	476.625	70.6111111	16.8445527	819.5442231	48.6533681
FOXP3	0	2.125		0	4.622901023	
FOXP4	0.75	54	72	1.94148371	95.99116615	49.4421692
FOXQ1	0	0		0	0	
FOXR1	0	6.25		0	13.23230684	
FOXR2	0	0		0	0	
FOXS1	0.25	23.875	95.5	0.73637114	51.26169998	69.6139448
FUBP1	52	2185.625	42.03125	307.390013	8353.373993	27.175164
FUBP3	7.5	426.5	56.8666667	21.2558375	826.1099216	38.8650845
GABPA	4.5	318.875	70.8611111	12.9241758	635.2573967	49.1526429
GATA1	0	0		0	0	
GATA2	0	0.5		0	0.91719324	
GATA3	0	0.375		0	0.896222244	
GATA4	0	0		0	0	
GATA5	0	0		0	0	
GATA6	0	0		0	0	
GBX1	0	0		0	0	
GBX2	0.25	12.375	49.5	0.8469114	28.84658741	34.0609271
GCFC2	3.625	192.125	53	10.380374	370.6605517	35.7078225
GCM1	0	0		0	0	
GFI1	0.125	0.25	2	2.21012058	4.512553925	2.0417682
GFI1B	0.125	1.25	10	0.35939162	2.596641815	7.22510392
GLI1	0	11		0	17.37859213	
GLI2	0.875	62	70.8571429	2.4344278	113.2726359	46.5294702
GLI3	14.5	828.875	57.1637931	47.2552012	1793.961749	37.9632655
GLIS1	0	0.125		0	0.259664182	
GLIS2	1	63.5	63.5	3.03425341	127.9448691	42.166837
GLIS3	1.75	136.875	78.2142857	6.11428451	320.1843068	52.3666026
GLMP	4.375	241.125	55.1142857	12.651135	469.777773	37.133251
GMEB1	11.75	605.5	51.5319149	32.3074108	1133.987372	35.0999149
GMEB2	2.875	247.25	86	8.03006493	480.7563708	59.8695496
GPER1	0.875	32.25	36.8571429	2.56850505	68.64425169	26.7253716
GRHL1	1	80.75	80.75	2.79560948	158.1778055	56.5807945
GRHL2	0	0.25		0	0.494409145	
GRHL3	0	6.875		0	15.13780355	
GSC	0	4		0	8.807449341	
GSC2	0	0.625		0	1.095881764	
GSX1	1.5	26.625	17.75	3.42242724	43.39653171	12.6800451
GSX2	0.875	53.5	61.1428571	2.0875125	92.72602068	44.4193846
GZF1	6.875	340.625	49.5454545	18.9811271	664.2024487	34.9927824

HAND1	0	0		0	0	
HAND2	0	0.75		0	1.792444488	
HBP1	16.75	1032.5	61.641791	51.6111993	2072.493554	40.1558883
HCFC1	17.125	1084.875	63.350365	46.3556132	2061.120402	44.4632324
HDAC5	22.5	1141.875	50.75	63.2082926	2263.300054	35.8070114
HDGF	18.625	1366.125	73.3489933	52.3213692	2589.019137	49.4830158
HELT	0.75	33.625	44.8333333	1.67906187	54.90138264	32.6976531
HES1	2.125	179	84.2352941	6.2730287	381.3371074	60.789951
HES2	0	1		0	2.155156504	
HES3	0	0		0	0	
HES4	3.25	66	20.3076923	8.73021889	131.6914415	15.0845521
HES5	0.5	25.5	51	1.48999326	49.94817054	33.5224136
HES6	25.875	1174.5	45.3913043	66.7403898	2182.022045	32.6941759
HES7	0	1.25		0	2.277537329	
HESX1	0.25	14.625	58.5	0.70240432	33.01259204	46.9994151
HEY1	40.75	2441.375	59.9110429	117.084221	4790.438227	40.9144647
HEY2	2.625	195	74.2857143	8.92145796	446.7380104	50.074552
HEYL	0	0		0	0	
HHEX	0	0		0	0	
HIC2	1.5	90.125	60.0833333	4.34756283	172.2554076	39.6211428
HIF1A	42	2861.625	68.1339286	118.377776	5571.58323	47.0661254
HIF3A	6	299.5	49.9166667	18.2547753	634.5942968	34.7631941
HINFP	14.125	753.25	53.3274336	40.9295569	1505.920545	36.7929843
HIVEP1	3	172.625	57.5416667	9.18383263	377.7459348	41.1316223
HIVEP2	13	779.75	59.9807692	37.9123735	1550.000399	40.8837605
HIVEP3	2.875	204.75	71.2173913	7.31377175	391.1325844	53.4789159
HLF	1	48.125	48.125	3.38764559	108.6488175	32.0720732
HLTF	14.375	1058.75	73.6521739	41.5576702	2134.618182	51.3652034
HLX	0.5	13.5	27	1.78677516	30.48647449	17.0622892
HMBOX1	9.25	684.875	74.0405405	23.7062312	1238.56631	52.2464453
HMG20A	27.375	1801.5	65.8082192	75.1590684	3437.86032	45.7411247
HMGA1	34.5	1137.25	32.9637681	92.5964606	2155.384546	23.2771807
HMGA2	1.625	99.625	61.3076923	5.03235747	210.5756285	41.8443303
HMX1	0.125	8.5	68	0.46993188	15.1830369	32.3090167
HNF1A	0	0.25		0	0.597481496	
HNF1B	0	1.5		0	3.584888976	
HNF4A	0	0		0	0	
HNF4G	0.125	11.625	93	0.46993188	27.07765088	57.6203746
HNRNPAB	45.75	1910.875	41.7677596	281.721356	7579.236958	26.90331
HOMEZ	4.875	232.5	47.6923077	14.4466919	462.4128197	32.0082149
HOXA1	0	0		0	0	
HOXA10	0	0		0	0	
HOXA11	0	0		0	0	
HOXA13	0	0		0	0	
HOXA2	0	0		0	0	
HOXA3	0	0		0	0	
HOXA4	0	0		0	0	
HOXA5	0	0		0	0	
HOXA6	0	0		0	0	
HOXA7	0	0		0	0	
HOXA9	0	0		0	0	
HOXB1	0	0		0	0	

HOXB13	0	1.25		0	2.98740748	
HOXB2	0.125	4.75	38	0.27984365	7.806863137	27.8972321
HOXB3	0	0		0	0	
HOXB4	0	0		0	0	
HOXB5	0	0		0	0	
HOXB6	0	0		0	0	
HOXB7	0	0		0	0	
HOXB8	0	0		0	0	
HOXB9	0	0		0	0	
HOXC10	0	0		0	0	
HOXC11	0	0		0	0	
HOXC13	0	0		0	0	
HOXC4	0	0		0	0	
HOXC5	0	0		0	0	
HOXC6	0	0		0	0	
HOXC8	0	0		0	0	
HOXD1	0	0		0	0	
HOXD10	0	0		0	0	
HOXD11	0	0		0	0	
HOXD13	0	0		0	0	
HOXD3	0	0		0	0	
HOXD4	0	0		0	0	
HOXD8	0	2.375		0	4.933619449	
HOXD9	0	0		0	0	
HR	0	2.5		0	4.607752812	
HSF1	16.75	596	35.5820896	44.7200875	1141.177607	25.5182329
HSF2	26	1453.625	55.9086538	72.6550048	2792.076218	38.4292345
HSF4	0	1.5		0	3.349018315	
HTATIP2	0.25	54.5	218	0.71999221	115.8863687	160.955031
IER2	11.375	616.25	54.1758242	32.0895483	1194.873625	37.2356012
IFI16	3.25	180.75	55.6153846	10.3123315	387.2329238	37.5504729
IGHMBP2	7.625	425.125	55.7540984	26.2623755	833.8610611	31.7511666
IKZF1	0.25	10.375	41.5	0.93986376	22.71650117	24.1699938
IKZF2	1.625	127.625	78.5384615	4.74004597	245.2107268	51.7317192
IKZF3	0	0		0	0	
IKZF5	8	366.75	45.84375	22.0960968	707.8939706	32.0370597
INSM1	4.125	206.875	50.1515152	10.4601802	392.2058786	37.495136
IRF1	2.75	90.625	32.9545455	7.85490845	178.3028085	22.69954
IRF2	3.125	263.625	84.36	9.52655435	518.8371685	54.4622063
IRF3	8.125	448.875	55.2461538	22.1715575	871.1958981	39.2934009
IRF4	0	2		0	4.40372467	
IRF5	0.875	31.5	36	2.27760073	71.1290097	31.2297975
IRF6	0.25	11	44	0.8469114	24.97798794	29.4930355
IRF7	1.75	74.875	42.7857143	5.53504721	155.4786504	28.0898508
IRF8	0	6.5		0	12.89654099	
IRF9	7.5	321.5	42.8666667	25.5417451	684.4136226	26.7958834
IRX1	0	1.125		0	2.500603083	
IRX2	0	7.375		0	12.10958195	
IRX3	0	2.875		0	6.729989467	
IRX5	0	0.75		0	1.768936532	
IRX6	0	0		0	0	
ISL1	2.125	104.625	49.2352941	5.2017942	173.466439	33.3474244

ISL2	0.375	17.75	47.3333333	0.83953094	31.31884545	37.3051713
JARID2	16.625	736.875	44.3233083	137.974939	3932.387585	28.500738
JDP2	0.375	32.125	85.6666667	1.22389092	72.38365759	59.1422459
JUN	33	1670.375	50.6174242	96.5450113	3369.721736	34.9031161
JUNB	5.25	198	37.7142857	13.8606463	376.3257865	27.1506665
JUND	2	87.25	43.625	5.30752276	163.5133322	30.8078438
KCNH8	0.875	68.75	78.5714286	2.33963534	128.5637092	54.9503194
KCNIP3	1.75	131.625	75.2142857	5.06312735	266.9040478	52.7152547
KHSRP	1.625	127.625	78.5384615	4.37119286	234.4175714	53.6278263
KLF1	0.125	6.875	55	0.46993188	16.33744416	34.7655584
KLF10	33.5	1881.25	56.1567164	138.334822	4443.773908	32.123321
KLF11	9.375	604.5	64.48	28.6984407	1186.903615	41.3577736
KLF12	27.625	1820.125	65.8868778	72.4259162	3329.896262	45.9765846
KLF13	1.375	88.875	64.6363636	3.79486425	173.7789576	45.7931947
KLF14	0	0		0	0	
KLF15	0	4.125		0	8.943104082	
KLF16	0.875	27.5	31.4285714	2.10162254	52.18675284	24.8316487
KLF17	0	0		0	0	
KLF2	0	4.75		0	8.53114055	
KLF3	9	498.25	55.3611111	26.2858045	1046.055828	39.7954656
KLF4	0.25	13	52	0.93986376	27.40694757	29.1605537
KLF6	33.5	1793.75	53.5447761	93.7501648	3521.632157	37.5640103
KLF8	1	75.625	75.625	2.92755975	156.0250274	53.2952495
KLF9	0.25	13.375	53.5	0.93986376	30.62530654	32.5848362
KMT2A	29.875	1865.5	62.4435146	79.4332094	3501.912201	44.0862484
KRBOX4	6.25	363.25	58.12	16.6544903	684.5037217	41.1002505
LBX1	0	0		0	0	
LCORL	10.5	718.25	68.4047619	30.6476658	1400.177937	45.6862831
LEF1	6.75	479.75	71.0740741	19.196831	957.3709457	49.8713015
LHX1	0.75	17.125	22.8333333	2.20137428	33.29776811	15.1259004
LHX2	16.5	973.875	59.0227273	51.1916753	2074.245395	40.5191935
LHX3	0	0		0	0	
LHX4	0	3.25		0	5.111348767	
LHX6	1	91	91	2.32448715	158.5753945	68.2195186
LHX9	0.625	82.5	132	1.78178815	174.9285655	98.1758496
LITAF	27	1646.25	60.9722222	81.8042202	3374.268337	41.2480961
LMO2	1.75	131	74.8571429	4.91915131	267.1514439	54.308442
LMX1A	3.875	228.75	59.0322581	12.346749	482.2300437	39.0572484
LMX1B	0	0		0	0	
LRRFIP1	9.625	418.875	43.5194805	26.4638291	799.4898012	30.2106622
LZTFL1	5	281.375	56.275	14.250815	558.1503275	39.1662039
MACC1	0	0.625		0	1.028123717	
MAF	1.25	92.875	74.3	3.10757389	182.7803211	58.8176912
MAFA	0	0		0	0	
MAFB	6.125	223.375	36.4693878	15.7941462	408.3537062	25.8547503
MAFF	2	94.25	47.125	5.8942877	200.6282382	34.037741
MAFG	6.375	302.875	47.5098039	17.3433449	567.1430821	32.7009054
MAFK	0.375	15.25	40.6666667	0.83953094	27.25455079	32.464022
MAX	14.625	996.375	68.1282051	43.0446815	1892.520792	43.9664257
MAZ	20.875	1154.75	55.3173653	54.2447582	2130.931349	39.2836363
MECOM	0	0.875		0	1.926629543	
MED1	14.75	940.625	63.7711864	39.4090923	1726.200131	43.8020779

MEF2A	6.75	364.25	53.962963	19.336448	724.3566957	37.4606905
MEF2B	0	0.125		0	0.275232792	
MEF2BNB-ME	0	0.625		0	1.33457495	
MEF2C	16.125	885.5	54.9147287	79.4884567	2364.898184	29.7514668
MEF2D	4	232.625	58.15625	11.2611084	450.4134441	39.9972567
MEIS1	17.125	1024	59.7956204	41.2472779	1799.091092	43.6172078
MEIS2	40.125	2494	62.1557632	107.912753	4492.19902	41.6280644
MEOX1	0	0		0	0	
MEOX2	0	0		0	0	
MESP1	2.625	82.5	31.4285714	7.48963837	160.6917144	21.4552034
MESP2	0	5.875		0	12.93594122	
MGA	11.875	837.5	70.5263158	31.7563845	1593.056963	50.164935
MITF	0.5	50	100	1.26209161	95.49390579	75.6632127
MIXL1	0	0		0	0	
MKX	0.5	17.125	34.25	1.6938228	40.39317534	23.8473443
MLX	19.5	1170	60	54.7601339	2295.498643	41.9191569
MLXIP	6.25	383.75	61.4	17.0844877	705.9884523	41.323361
MLXIPL	0	2.25		0	4.970068946	
MN1	17.875	1112	62.2097902	44.6650799	1957.08526	43.8168982
MNT	0.75	42	56	1.85959232	82.41089075	44.3166439
MSC	0	0.125		0	0.205443767	
MSGN1	0	0		0	0	
MSX1	1.375	46.25	33.6363636	4.54360286	104.2744263	22.9497228
MSX2	0.75	35.75	47.6666667	2.43019394	77.35866076	31.8322993
MTF1	9.25	489.625	52.9324324	25.6574174	961.459615	37.4729693
MTF2	32.5	1832.875	56.3961538	87.5128384	3484.538592	39.817456
MXD1	2.75	224.875	81.7727273	7.20829303	412.0484071	57.1631044
MXD3	9.75	401.75	41.2051282	24.752623	710.7090211	28.712473
MXD4	18.25	819.25	44.890411	120.070031	4007.668463	33.3777582
MXI1	12.375	942.625	76.1717172	36.5227931	1797.845619	49.2253048
MYB	0.125	24.25	194	0.30127814	42.55642369	141.252941
MYBL1	0.25	7.5	30	0.55968729	38.91260076	69.5256107
MYBL2	8.25	547.5	66.3636364	23.3330254	1038.374105	44.5023347
MYC	3	174.875	58.2916667	8.5044245	345.6040854	40.6381508
MYCL	5.75	371.125	64.5434783	16.0534855	733.3535559	45.6818898
MYCN	2.625	211.875	80.7142857	7.55924248	433.8783445	57.3970666
MYEF2	15	880.25	58.6833333	41.8089641	1696.546395	40.5785322
MYF5	0	0		0	0	
MYF6	0	1.125		0	2.688666732	
MYOCD	0	0		0	0	
MYOD1	0	0		0	0	
MYOG	0	0		0	0	
MYPOP	0.125	7.5	60	0.37697952	28.54326831	75.7157003
MYRF	0.25	21.375	85.5	0.8469114	47.29492701	55.8440081
MYT1	8.375	553.625	66.1044776	21.2464796	1025.298397	48.257331
MYT1L	19.875	990.5	49.836478	54.0183948	1907.307053	35.3084733
MZF1	3.125	150.75	48.24	9.12968026	299.4659844	32.8013661
NACC1	0.25	9.5	38	0.55968729	17.57573902	31.4027838
NACC2	1.625	110	67.6923077	4.48758621	213.8661895	47.6572882
NANOG	0	0		0	0	
NEUROD1	7.125	445.5	62.5263158	19.6969062	890.2366925	45.1967777
NEUROD2	7.75	348.375	44.9516129	22.4127281	687.5744045	30.6778543

NEUROD4	1.125	83.875	74.5555556	3.6037391	178.1620574	49.438112
NEUROG1	2.125	123.25	58	6.32040401	251.3591138	39.7694694
NEUROG2	7.25	431.625	59.5344828	22.2897149	907.6818896	40.7220054
NEUROG3	0	0.25		0	3.386914279	
NFAT5	8.875	615.375	69.3380282	24.2576861	1216.762396	50.1598705
NFATC1	0.75	51.875	69.1666667	2.27879118	100.665407	44.1749151
NFATC2	0	0.875		0	2.020661368	
NFATC3	10.875	666.125	61.2528736	30.3089023	1247.999859	41.1760165
NFATC4	1.5	123.5	82.3333333	4.763252	279.8005092	58.7414878
NFE2	0	20.25		0	47.70687662	
NFE2L1	6	295	49.1666667	17.3720717	589.1469747	33.9134551
NFE2L2	21.875	1561.625	71.3885714	61.7496265	3095.65615	50.1323866
NFE2L3	0.625	20.5	32.8	1.78299712	41.53625041	23.2957473
NFIC	5.5	328.375	59.7045455	14.6372766	630.5284044	43.0768935
NFIL3	4.875	397.5	81.5384615	17.5697631	745.2749868	42.418044
NFIX	9.625	590.625	61.3636364	29.0509416	1252.154082	43.1020137
NFKB1	19.25	448.875	23.3181818	304.42278	6302.623644	20.7035217
NFKB2	0.5	12.25	24.5	1.75280833	26.83179173	15.3078869
NFKBIZ	0.125	6.25	50	0.37697952	14.53740215	38.5628433
NFX1	15.125	886.625	58.6198347	42.6958646	1704.066389	39.9117434
NFXL1	16.375	967.125	59.0610687	47.5729281	1874.681093	39.4064685
NFYA	20.5	766.75	37.402439	58.9067196	1533.661771	26.03543
NFYC	31.375	1592.25	50.749004	92.00052	3110.712726	33.8119037
NHLH1	4.25	321.5	75.6470588	12.5225286	675.035765	53.9057075
NHLH2	1.625	108.875	67	5.62676473	248.7544114	44.2091368
NKRF	3.625	126.125	34.7931034	10.1965321	270.1079975	26.4901827
NKX1-2	0	0		0	0	
NKX2-1	0.5	12.25	24.5	1.11937458	20.12463571	17.9784641
NKX2-2	0	0		0	0	
NKX2-3	0	0		0	0	
NKX2-5	0.375	8.75	23.3333333	1.02903808	15.9091743	15.4602386
NKX2-6	0	0		0	0	
NKX2-8	0	0		0	0	
NKX3-1	0	12.125		0	23.09940789	
NKX3-2	0	0		0	0	
NKX6-1	0	0.125		0	0.219176353	
NKX6-2	0	0		0	0	
NKX6-3	0	4.875		0	9.318464641	
NOBOX	0	0		0	0	
NPAS1	5.375	259.875	48.3488372	12.7056662	439.0626626	34.5564456
NPAS2	0.875	46.625	53.2857143	2.59949733	101.3440165	38.9860053
NPAS3	4.25	191.625	45.0882353	16.6424825	391.8987203	23.5480926
NPAS4	1.375	72.25	52.5454545	3.0782801	121.0186114	39.31371
NR0B1	0	0		0	0	
NR1D1	4	249.625	62.40625	12.0619828	512.2413025	42.4674209
NR1D2	3.875	260.875	67.3225806	12.0326161	531.6171965	44.1813479
NR1H2	19.5	902.125	46.2628205	58.9609136	1804.711105	30.6086015
NR1H3	2.625	173.25	66	7.94745533	363.7782788	45.7729253
NR1H4	0.125	4.875	39	0.37697952	10.73407888	28.4739046
NR1I2	0	0		0	0	
NR1I3	0	12.25		0	28.04156372	
NR2C1	5.25	268.375	51.1190476	14.8653503	548.4005218	36.891194

NR2E1	13.875	792.375	57.1081081	39.6533776	1603.9967	40.4504432
NR2E3	0	1.625		0	3.170372692	
NR2F1	16.375	831	50.7480916	43.1706621	1553.610061	35.9876357
NR2F2	14.125	836.25	59.2035398	37.786281	1558.863125	41.2547381
NR2F6	6.125	294.25	48.0408163	17.3328811	577.1709057	33.2991903
NR3C1	2.625	192.5	73.3333333	7.43222321	386.7135356	52.0320132
NR3C2	0.125	13.125	105	0.37697952	27.03864743	71.7244466
NR4A1	2	122.75	61.375	6.06139146	252.4414408	41.6474406
NR4A2	12.5	696.75	55.74	37.4042145	1417.605946	37.8996315
NR4A3	7.75	358.75	46.2903226	24.6127411	756.4617994	30.7345613
NR5A1	0	0		0	0	
NR5A2	0	0		0	0	
NR6A1	2	111.625	55.8125	7.63822617	249.9267485	32.7205221
NRF1	4.625	268.875	58.1351351	12.6805908	519.6323874	40.9785629
NRG1	6.125	323.375	52.7959184	16.628876	608.7287116	36.6067262
NRL	1.5	68.25	45.5	3.94500203	124.3140308	31.5117787
OLIG1	0.75	71.875	95.8333333	1.70049637	116.4413605	68.4749244
OLIG2	0.375	15.25	40.6666667	0.94051341	26.05103265	27.698736
OLIG3	0.25	0.875	3.5	0.93986376	2.091185236	2.22498763
ONECUT1	0	0		0	0	
ONECUT2	1.125	103.625	92.1111111	3.0843553	187.3823826	60.7525284
ONECUT3	0	0		0	0	
OSR1	0	1.125		0	2.477095127	
OSR2	0	0		0	0	
OTP	0	1.75		0	3.108956768	
OTX1	15.625	854.75	54.704	46.1410581	1724.224152	37.3685439
OTX2	7.875	492	62.4761905	24.3631893	1046.202508	42.9419356
OVOL1	0	0		0	0	
OVOL2	0	0		0	0	
PATZ1	17.375	1028.75	59.2086331	48.8963437	1991.955288	40.738328
PAX1	0	0		0	0	
PAX2	0	3.5		0	8.364740944	
PAX3	0.25	36.375	145.5	0.75395904	82.54067069	109.476333
PAX4	0	0		0	0	
PAX5	0	0		0	0	
PAX6	36.375	1992	54.7628866	102.678878	3902.813388	38.009895
PAX7	0	0		0	0	
PAX8	0	0		0	0	
PAX9	0	0		0	0	
PBX1	39.625	2645.875	66.7728707	102.59524	4725.980474	46.0643252
PBX2	0.75	58.875	78.5	2.35030905	112.5791759	47.8997331
PBX3	19.25	1178.125	61.2012987	54.6267352	2156.850477	39.4834227
PBX4	2.875	120.75	42	7.59201916	222.0999395	29.2543966
PCGF6	4	198.375	49.59375	11.8001144	398.4548464	33.7670326
PDX1	0	0		0	0	
PEG3	0	0.125		0	0.219176353	
PGR	0.125	0.25	2	2.21012058	4.512553925	2.0417682
PGS1	7.375	509.625	69.1016949	20.9346599	991.1707163	47.3459192
PHB	73.625	1981	26.9066214	211.238972	3912.432225	18.5213561
PHF10	0.375	25.375	67.6666667	1.29925538	54.67905165	42.0849146
PHF20	30.5	1750.25	57.3852459	120.89479	4507.848481	37.2873675
PHF6	41.875	2017.25	48.1731343	299.821991	8579.233569	28.614424

PHF7	1.75	89.25	51	4.58873951	172.3643349	37.5624579
PHOX2A	0	0.25		0	0.597481496	
PHOX2B	0	0		0	0	
PITX1	0.25	4.625	18.5	0.68602539	9.733361441	14.1880484
PITX2	0.375	13.875	37	1.07248816	23.35378359	21.7753299
PITX3	0	0		0	0	
PKNOX1	8.875	729.625	82.2112676	22.9067114	1357.382533	59.2569797
PKNOX2	3.375	229.25	67.9259259	9.03950893	426.5011499	47.1818938
PLAG1	1.125	150.75	134	3.3298949	328.620768	98.6880302
PLAGL1	3.375	232.125	68.7777778	11.6184355	473.7221993	40.7733209
PLAGL2	9.625	600.75	62.4155844	25.268068	1130.62046	44.7450299
PLSCR1	2.5	132.625	53.05	7.83057872	274.5083061	35.0559411
POU1F1	0	0		0	0	
POU2F1	18.125	981.625	54.1586207	49.1893544	1838.951974	37.3851618
POU2F2	0.25	24.25	97	0.70240432	48.42695778	68.9445618
POU2F3	0	0		0	0	
POU3F1	0.25	7.875	31.5	0.55968729	30.78780433	55.0089395
POU3F2	3.625	225.125	62.1034483	9.10087816	397.5538106	43.6830165
POU3F3	3.75	109	29.0666667	10.8011223	218.7502236	20.2525458
POU3F4	0	8.875		0	16.15510589	
POU4F1	0	10.25		0	16.12040765	
POU4F2	0	0		0	0	
POU4F3	0	0		0	0	
POU5F1	0.25	1.625	6.5	0.55968729	2.670768968	4.77189496
POU6F1	3	179.875	59.9583333	8.12327998	339.9060514	41.843449
POU6F2	0.375	11.125	29.6666667	1.31684328	23.14148585	17.5734548
PPARA	2.75	133.125	48.4090909	8.28330996	291.1887566	35.1536714
PPARD	2.625	155.875	59.3809524	8.01149643	303.3829294	37.8684472
PPARG	1.125	73.5	65.3333333	3.66272464	155.6101419	42.484805
PRDM1	0.125	10.25	82	0.27984365	17.00905012	60.7805479
PRDM10	3.125	144.875	46.36	8.84893713	276.2697755	31.2206733
PRDM12	0	1.625		0	3.883629724	
PRDM13	0	4.5		0	7.890348701	
PRDM14	0	0		0	0	
PRDM15	2.125	156.75	73.7647059	5.64163328	296.5625427	52.5667884
PRDM16	2.375	91.25	38.4210526	9.77104673	207.504109	21.2366305
PRDM2	16.125	1201.5	74.5116279	43.6445423	2286.710453	52.3939611
PRDM4	3.25	308	94.7692308	9.33051432	643.6721436	68.9857088
PRDM5	0.625	23	36.8	1.97751304	48.17359972	24.3606989
PREB	38.625	2068.875	53.5631068	107.287166	4024.779316	37.5140799
PROP1	0	0		0	0	
PROX1	2.375	133.375	56.1578947	6.67023405	286.6639617	42.9765971
PRRX1	0	0		0	0	
PRRX2	0	0		0	0	
PTF1A	0	0		0	0	
PTH	0	0		0	0	
PURA	2.875	187.375	65.173913	8.25621053	358.3879316	43.408284
PURB	2	134.875	67.4375	5.19521692	258.2175793	49.7029447
RAD21	35.875	2322.25	64.7317073	103.081263	4455.366753	43.2218875
RAI1	5.375	262.375	48.8139535	13.4701108	481.6771132	35.7589571
RARA	6.25	373.5	59.76	15.7079835	677.2331167	43.1139438
RARB	2.125	96.875	45.5882353	6.11139433	182.0845284	29.7942693

RARG	1.75	127.125	72.6428571	5.40599695	257.7088587	47.6709219
RAX	0	1.5		0	3.075482349	
RAX2	0	0		0	0	
RBPJ	77.75	3868.375	49.7540193	216.128926	7499.809253	34.700627
RBPJL	0	0		0	0	
RCOR1	4.875	242.875	49.8205128	14.8238438	484.4640313	32.6814042
REL	3.25	220.375	67.8076923	9.04175929	421.0167851	46.5635914
RELA	16.25	877.5	54	45.0335903	1693.255006	37.5998226
RELB	0.625	30	48	1.78178815	60.71903341	34.0775829
REST	11.25	507.875	45.1444444	79.0336677	1907.234604	24.1319258
RFX1	1.5	44.125	29.4166667	4.1360401	87.48342035	21.1514923
RFX2	4.25	247	58.1176471	13.0467498	505.7828817	38.7669641
RFX3	29.25	1931.75	66.042735	84.7443618	3889.857734	45.9010801
RFX4	8.625	505.375	58.5942029	27.5766419	1068.993745	38.7644641
RFX5	16.5	1044.375	63.2954545	45.0397051	2007.604536	44.5741049
RFX6	0	0.375		0	0.778992545	
RFX7	20.625	1168.875	56.6727273	98.985818	3267.627182	33.0110641
RHOXF2	0	0		0	0	
RHOXF2B	0	0		0	0	
RNF112	3.125	183.375	58.68	9.3158792	372.4582084	39.9810045
RNF141	25.625	1600.875	62.4731707	83.3790486	3472.13076	41.6427246
RNF2	13.25	894.875	67.5377358	38.0786919	1762.500873	46.2857516
RORA	5.875	438.5	74.6382979	19.2263586	969.7753859	50.4398885
RORB	2.5	165.625	66.25	6.63575796	310.2983756	46.7615573
RORC	0.125	20.375	163	0.46993188	44.44918674	94.586447
RREB1	0.625	78.125	125	1.96113411	154.6362316	78.8504114
RUNX1	0	1		0	2.077313452	
RUNX2	0.125	23.875	191	0.46993188	51.45922707	109.503589
RUNX3	0	0		0	0	
RXRA	3	173.875	57.9583333	8.18204908	326.9912398	39.9644681
RXRB	6.125	354.875	57.9387755	15.6929228	647.7118351	41.2741363
RXRG	14.5	703.75	48.5344828	33.5563835	1149.03944	34.2420523
SALL1	17.875	1039.5	58.1538462	45.6010018	1916.839607	42.0350328
SALL2	24	1308	54.5	64.3669312	2477.716399	38.4936233
SALL3	0.5	24.25	48.5	1.18254363	62.00911273	52.4370612
SALL4	0.25	38	152	0.68602539	72.64507933	105.892698
SARNP	40.75	1442.625	35.4018405	222.735016	4522.721917	20.3053925
SATB1	11.875	437	36.8	139.114197	2655.870838	19.0912998
SATB2	0.625	40.75	65.2	1.76540922	77.50422367	43.9015627
SCML4	0	0.625		0	8.011614989	
SCRT1	0.625	25.5	40.8	1.56842532	45.90097653	29.2656437
SCRT2	6.75	356.75	52.8518519	18.0767377	631.3752225	34.9274981
SCX	0	0.25		0	0.480676559	
SHOX	0	0		0	0	
SHOX2	0	0		0	0	
SIM1	0	0		0	0	
SIM2	0.25	34.125	136.5	0.63923527	65.1938238	101.987214
SIX1	0	0		0	0	
SIX2	0	2.5		0	5.97481496	
SIX3	12	533.625	44.46875	28.2853101	894.791557	31.634497
SIX4	1.25	54	43.2	3.61892758	98.97960809	27.3505357
SIX5	1.125	71.875	63.8888889	3.44640872	157.5352143	45.7099627

SIX6	0	0		0	0	
SKIL	10	472.75	47.275	29.8559474	955.5036604	32.0037964
SMAD1	17.25	966	56	45.9975272	1790.59232	38.9280126
SMAD2	23.5	1616	68.7659574	103.44569	3807.003172	36.8019505
SMAD3	3	159.25	53.0833333	8.8006253	325.0606335	36.9360838
SMAD4	16.25	1069.125	65.7923077	45.1500763	2003.346702	44.3708375
SMAD5	19.375	851.5	43.9483871	155.241061	4419.362342	28.4677411
SNAI1	0.125	12.75	102	0.35939162	26.45815507	73.6192873
SNAI2	2.5	116.25	46.5	6.16161104	209.5467683	34.0084382
SNAI3	0.125	5.125	41	0.37697952	11.58110737	30.7207866
SOHLH1	0	0		0	0	
SOHLH2	0	0		0	0	
SOX1	3.875	281.375	72.6129032	9.39448488	492.8226368	52.4587184
SOX10	0	0.25		0	0.550465584	
SOX12	3.5	206.625	59.0357143	8.41175809	389.245816	46.2740145
SOX13	0.125	20.375	163	0.27984365	56.74098409	202.759594
SOX14	0	0		0	0	
SOX15	1.375	78	56.7272727	3.95572578	154.9393045	39.1683633
SOX17	0	0		0	0	
SOX18	0	0		0	0	
SOX2	25.875	1347.75	52.0869565	81.7622696	2845.313962	34.7998407
SOX21	0.125	16.875	135	0.27984365	33.1965951	118.62551
SOX3	2.875	132.25	46	9.07264272	277.3316378	30.5679003
SOX30	0.125	4	32	0.34301269	9.442164155	27.5271566
SOX5	34.25	1841.25	53.7591241	96.2437838	3542.234912	36.8048177
SOX6	12.5	678.125	54.25	32.2609876	1248.273628	38.6929763
SOX7	0.125	2.125	17	0.37697952	4.398675267	11.6682075
SOX8	1.25	111.875	89.5	3.38597035	196.731562	58.1019742
SOX9	11.75	706.125	60.0957447	34.7514168	1424.652829	40.9955323
SP1	4.125	386.5	93.6969697	10.7309786	745.9144713	69.5103869
SP110	1	59.25	59.25	2.56705221	106.0282549	41.3035055
SP2	4	206.75	51.6875	10.5528586	367.524557	34.8270143
SP3	25.5	1700.75	66.6960784	70.5832367	3318.33382	47.0130582
SP4	8.125	600.875	73.9538462	20.8172916	1116.789161	53.6471883
SP5	0.25	16.625	66.5	0.55968729	28.60373139	51.1066301
SP6	0	1.625		0	3.578026295	
SP7	0	2		0	4.779851968	
SP8	11.5	465.625	40.4891304	28.0288669	831.8991072	29.6800834
SP9	2.75	99.875	36.3181818	6.34947068	164.5962272	25.9228266
SPDEF	0	0		0	0	
SPI1	0.125	3.375	27	0.30127814	5.307939104	17.618069
SPIB	0	0		0	0	
SPIC	0	0		0	0	
SPZ1	0	0		0	0	
SREBF1	3	197.25	65.75	8.2299992	380.1822436	46.1946878
SRF	3.125	185	59.2	8.06563155	326.3762247	40.4650551
SRY	0	0		0	0	
ST18	8.75	670.75	76.6571429	26.3974857	1392.757828	52.7610032
STAG1	9.625	547.875	56.9220779	25.0512167	1041.032345	41.556159
STAG2	17.25	970.25	56.2463768	52.377053	1944.518652	37.1253925
STAT1	14.625	851.375	58.2136752	42.7504006	1766.490529	41.3210287
STAT2	4.875	280.875	57.6153846	13.7526531	570.0949478	41.4534523

STAT3	25.125	1476.25	58.7562189	74.2805159	3041.104527	40.9408105
STAT4	0.875	35.625	40.7142857	2.68826617	66.76062064	24.8340813
STAT5A	0.5	8.5	17	1.50791808	18.59071923	12.3287329
STAT5B	7	347.125	49.5892857	18.430768	643.1617574	34.8960911
STAT6	0.375	27.875	74.3333333	1.29925538	60.19986555	46.3341284
STK16	10.75	587.5	54.6511628	30.9350191	1160.455797	37.5126905
SUB1	59.875	2590.625	43.2672234	206.111857	5801.659024	28.1481091
SUPT20H	28.75	1606	55.8608696	122.877311	3805.017179	30.9659866
SUZ12	9.75	312.5	32.0512821	138.80585	3307.645502	23.8292946
T	0	0		0	0	
TAL1	0	0.125		0	0.196590337	
TBPL1	9.875	776.25	78.6075949	27.8156845	1461.676187	52.5486327
TBR1	28.125	1458.75	51.8666667	82.8959158	2891.346212	34.879236
TBX1	0	3.875		0	6.422977184	
TBX10	0	0.625		0	1.376163959	
TBX15	0	0		0	0	
TBX18	0	0		0	0	
TBX19	0.75	46.625	62.1666667	2.17780871	85.02501972	39.0415463
TBX2	0	0		0	0	
TBX20	0	0		0	0	
TBX21	0	0		0	0	
TBX22	0	0		0	0	
TBX4	0	0		0	0	
TBX5	0	0		0	0	
TBX6	0.125	22.875	183	0.27984365	39.31050765	140.473111
TCF12	45	2616.625	58.1472222	252.388103	8607.63929	34.1047744
TCF15	0	0		0	0	
TCF20	10.25	486.375	47.4512195	80.8696666	1404.965483	17.3732073
TCF21	0	0		0	0	
TCF3	11.75	626.5	53.3191489	32.9131426	1189.484172	36.1400972
TCF7	0.25	10.625	42.5	0.75395904	21.38111574	28.358458
TCF7L1	0.75	55.75	74.3333333	2.1480254	112.6995514	52.4665823
TCF7L2	2.625	167.875	63.952381	7.27789595	330.3620069	45.3925158
TCFL5	1.25	31.25	25	3.41309155	60.63575713	17.7656404
TEAD1	11	698.625	63.5113636	31.1091546	1397.665049	44.9277734
TEAD2	4.125	162.125	39.3030303	10.9530026	305.2898512	27.8727086
TEAD4	2.25	97.25	43.2222222	5.72533734	174.6427027	30.5034782
TEF	2.375	236	99.3684211	6.51950923	464.1081364	71.1875879
TFAM	12.875	732.875	56.9223301	35.6973239	1413.634396	39.6005706
TFAP2A	0.25	17.75	71	0.55968729	32.54925223	58.1561396
TFAP2B	0	0.75		0	1.768936532	
TFAP2C	3.875	219.875	56.7419355	11.7738649	483.4050862	41.0574684
TFAP2D	0	0		0	0	
TFAP2E	0.25	19.125	76.5	0.68602539	33.41138227	48.7028364
TFAP4	0.125	8.5	68	0.35939162	16.40455463	45.6453452
TFCP2	2.75	246.75	89.7272727	8.14182422	478.0254107	58.7123227
TFCP2L1	0	0		0	0	
TFDP1	13	836.875	64.375	36.8272847	1611.31236	43.753222
TFDP2	12.125	736.75	60.7628866	33.2336665	1435.342439	43.1894098
TFDP3	0	0		0	0	
TFE3	3.75	224.625	59.9	10.2189564	451.0107937	44.1347215
TFEB	1	34.625	34.625	2.41543302	65.05009374	26.9310278

TFEC	0	0		0	0	
TGFB1I1	4.125	207.375	50.2727273	11.5945696	414.8768417	35.7819959
TGIF1	15.875	967.625	60.9527559	49.1555585	2083.918876	42.3943688
TGIF2LX	0	0		0	0	
THAP11	20.625	797.875	38.6848485	56.8585605	1542.486374	27.1284809
THRA	28.875	1618.125	56.038961	75.5611989	2996.828865	39.6609491
THRB	3.5	278.125	79.4642857	8.17238402	491.6437918	60.1591642
TLE4	16.125	736.5	45.6744186	166.894357	4447.885587	26.6509046
TLX1	0	0		0	0	
TLX2	0	0		0	0	
TOX2	2	155.625	77.8125	5.62123693	313.9919641	55.8581622
TOX3	10.75	755.625	70.2906977	27.4541201	1369.565037	49.885592
TP53	9.375	501.375	53.48	27.0825091	1046.128661	38.6274645
TP63	0	0		0	0	
TP73	2.375	113.625	47.8421053	7.16852438	237.2568926	33.0970337
TRPS1	3	237.875	79.2916667	8.94487	504.7178009	56.4253926
TSHZ1	4.875	297.125	60.9487179	13.6720447	554.1420854	40.5310322
TSHZ2	3	157	52.3333333	8.31966049	311.4868535	37.4398515
TSHZ3	1.75	160.75	91.8571429	4.79704678	308.9837844	64.4112511
TULP1	0	0.75		0	1.557985089	
TULP2	0	0		0	0	
TULP4	24.75	722.625	29.1969697	92.7007572	1347.91003	14.5404425
TWIST1	0	0		0	0	
TXK	0	0		0	0	
UBP1	2.5	107.875	43.15	7.1006125	212.2801568	29.8960346
USF1	11.875	578.875	48.7473684	32.0987131	1106.667747	34.477013
USF2	10.875	314.625	28.9310345	157.357858	3915.180883	24.8807459
VAX1	1.625	107.5	66.1538462	3.82468785	176.5337456	46.1563799
VAX2	2.625	130.375	49.6666667	6.47747805	231.8847976	35.7986235
VDR	0	0		0	0	
VENTX	0	0		0	0	
VEZF1	45.625	2656	58.2136986	263.26558	8889.725322	33.7671385
VSX2	0.375	11.625	31	1.07817487	24.14876888	22.3978221
WT1	0	0		0	0	
XBP1	30.25	1706.5	56.4132231	88.3694759	3414.000918	38.6332598
YBX3	2.375	138.75	58.4210526	6.9744968	289.2692404	41.4752847
YLPM1	11.25	818.125	72.7222222	32.9439094	1863.161223	56.5555594
YY1	8.5	478.75	56.3235294	22.1393468	898.5702504	40.5870262
YY2	0.625	33.75	54	1.94267416	69.93651892	36.0001282
ZBED1	20.5	1116	54.4390244	57.8040549	2158.644189	37.3441654
ZBTB1	5.75	367.25	63.8695652	16.8896349	747.3026236	44.2462271
ZBTB10	2.875	191	66.4347826	8.3021754	370.5906106	44.6377718
ZBTB14	7.5	434.625	57.95	20.750908	829.6635365	39.9820352
ZBTB16	6.875	370.75	53.9272727	19.1634476	720.7949918	37.6130124
ZBTB17	17.25	890.625	51.6304348	46.2282873	1650.338114	35.699746
ZBTB18	10.625	757.75	71.3176471	31.823593	1544.645994	48.5377624
ZBTB2	2.5	242.125	96.85	6.7421147	485.5679231	72.020122
ZBTB20	34.75	800.75	23.0431655	96.0061427	1615.739756	16.8295456
ZBTB21	5.875	538.875	91.7234043	16.6185364	1059.365043	63.7459894
ZBTB33	14.75	1069.75	72.5254237	41.6351008	2097.434173	50.3765845
ZBTB4	3.75	293.25	78.2	10.4223712	559.986016	53.7292334
ZBTB42	0.875	56	64	2.62630609	109.8958557	41.8442679

ZBTB45	6.875	294.25	42.8	50.4903702	1827.128024	36.1876536
ZBTB5	16.625	948.375	57.0451128	44.6931515	1773.274013	39.6766385
ZBTB7A	0	7.125		0	17.48194515	
ZBTB7B	0	3.625		0	8.030236564	
ZC3H8	2.75	137.75	50.0909091	7.46026681	242.8844749	32.5570762
ZEB1	15	991.5	66.1	43.430141	2048.397673	47.1653471
ZFAND3	4.875	341.5	70.0512821	12.6988605	665.6202184	52.4157439
ZFAT	3.5	175.75	50.2142857	9.30543039	328.4817264	35.3000036
ZFP1	17.125	973.625	56.8540146	47.7016991	1873.043057	39.2657513
ZFP37	6	388.875	64.8125	16.7894461	756.6090873	45.0645652
ZFP42	0	0		0	0	
ZFP69	2.5	133	53.2	7.02704219	261.4430149	37.2052718
ZFP90	14	977.25	69.8035714	38.7948544	1907.275853	49.1631141
ZFX	11.125	688.25	61.8651685	30.4790544	1323.069588	43.4091416
ZFY	0	0		0	0	
ZGPAT	20.375	715.375	35.1104294	224.45641	5341.240149	23.7963361
ZHX2	4.75	298.125	62.7631579	13.5630518	629.2036333	46.3910071
ZHX3	11.5	690.125	60.0108696	31.5765683	1336.664195	42.3308886
ZIC1	2.875	148.375	51.6086957	9.20170709	324.9307718	35.3120099
ZIC2	2.125	196.375	92.4117647	6.643385	437.8002162	65.9001723
ZIC3	4.25	309.375	72.7941176	14.200223	685.2209454	48.2542384
ZIC4	1.5	74.5	49.6666667	4.57379165	164.1220125	35.8831414
ZIC5	0.625	57.75	92.4	1.8673097	128.6894659	68.9170446
ZKSCAN3	16.25	919.875	56.6076923	42.7212512	1707.570494	39.9700488
ZKSCAN5	8.625	560	64.9275362	24.9222001	1041.38257	41.7853386
ZMYND8	16.375	847	51.7251908	69.0876584	2310.645669	33.4451293
ZNF10	7.625	527.875	69.2295082	20.9726573	1000.686955	47.7138849
ZNF117	2	89.5	44.75	5.78638098	194.8693858	33.6772477
ZNF124	4.875	289.75	59.4358974	13.4516621	542.288476	40.3138639
ZNF131	12	722.5	60.2083333	35.2743513	1389.289746	39.3852671
ZNF132	2.875	183.125	63.6956522	7.89959012	352.9336019	44.6774575
ZNF133	10.5	607.125	57.8214286	29.7195954	1194.764221	40.2012277
ZNF134	22.25	1381.125	62.0730337	61.4019135	2607.601571	42.4677575
ZNF135	7.25	337.625	46.5689655	20.469614	666.7984845	32.5750395
ZNF136	10.375	534.625	51.5301205	28.8781364	1022.978182	35.4239681
ZNF138	7.875	302.125	38.3650794	24.9986162	613.3371409	24.5348437
ZNF140	15.25	814.75	53.4262295	42.544268	1607.149136	37.7759264
ZNF143	12.375	791.875	63.989899	35.2656855	1540.762821	43.6901424
ZNF148	28.25	1901.25	67.300885	80.7238959	3646.918393	45.1776807
ZNF154	1.875	103	54.9333333	5.26521308	206.8061929	39.2778392
ZNF155	2	133.75	66.875	5.64717364	269.0765611	47.6480056
ZNF157	1	107.125	107.125	2.55067328	192.4651632	75.4566117
ZNF165	1.25	52.25	41.8	3.59243751	107.0896737	29.8097527
ZNF174	8.875	551.5	62.1408451	25.1172356	1084.500994	43.1775618
ZNF175	0.625	32.5	52	1.96113411	79.14657305	40.3575526
ZNF177	0	0		0	0	
ZNF18	8.625	495	57.3913043	23.8573717	973.097517	40.7881275
ZNF189	5.875	354.625	60.3617021	15.9576763	660.7887972	41.4088357
ZNF19	4	269.25	67.3125	11.8002808	535.7852887	45.4044526
ZNF202	2.875	239.875	83.4347826	8.46240579	483.309897	57.1125882
ZNF205	4.25	234.375	55.1470588	11.3713521	435.1028987	38.2630752
ZNF214	1.625	165.5	101.846154	4.2961471	331.3712696	77.1321982

ZNF215	0.125	16.5	132	0.34301269	33.64874225	98.0976588
ZNF217	3.5	294.125	84.0357143	10.8774113	600.538381	55.2096788
ZNF219	5.125	203.875	39.7804878	14.0452735	391.8613949	27.8998764
ZNF22	30.625	449.875	14.6897959	82.343713	862.9443501	10.4797843
ZNF224	0.75	39.25	52.3333333	2.25735669	76.45648498	33.8699176
ZNF23	12.875	746.5	57.9805825	35.4297451	1436.939985	40.5574463
ZNF230	5.375	353.625	65.7906977	14.7575908	686.8361032	46.5412081
ZNF239	8.25	552	66.9090909	23.7576108	1067.060177	44.9144564
ZNF24	33.75	2200.625	65.2037037	94.5056975	4222.087741	44.6754836
ZNF250	17.125	1061.625	61.9927007	46.8127893	2066.380333	44.1413632
ZNF251	4.625	228.75	49.4594595	12.8770991	450.3456024	34.9725974
ZNF252P	5.375	329.875	61.372093	14.6073224	651.0365028	44.5691882
ZNF256	4.125	265.125	64.2727273	11.0762139	485.1763927	43.8034511
ZNF263	11	638.375	58.0340909	32.3743213	1316.093787	40.6523978
ZNF267	2.25	116.25	51.6666667	5.97888474	220.1413216	36.8197968
ZNF268	12.25	922.625	75.3163265	35.3053177	1784.611476	50.5479512
ZNF274	18.875	1069	56.6357616	53.0972274	2074.492465	39.0696947
ZNF281	22.125	1899.875	85.8700565	57.7586567	3471.28662	60.0998503
ZNF284	1.875	76.5	40.8	5.45026396	159.2007031	29.2097235
ZNF300	13.625	790.125	57.9908257	38.469477	1529.097544	39.748332
ZNF302	23	1013.125	44.048913	89.8163336	1951.577107	21.7285323
ZNF304	11.875	688.125	57.9473684	32.1361822	1305.357241	40.6195494
ZNF317	14.25	969.25	68.0175439	36.7682657	1786.04868	48.5758207
ZNF322	4.625	312.5	67.5675676	13.224199	604.1782679	45.6873243
ZNF326	12.125	622.125	51.3092784	32.5987154	1179.550609	36.1839598
ZNF333	7.125	332	46.5964912	21.8420394	685.020619	31.3624844
ZNF34	8	524.625	65.578125	22.2178899	1003.629027	45.1721127
ZNF35	7.25	429.5	59.2413793	27.9953106	896.8937402	32.0372849
ZNF350	7.375	377.75	51.220339	21.5526763	724.9945364	33.6382604
ZNF354A	4.5	306.375	68.0833333	12.0246473	623.3134597	51.8363194
ZNF354C	0.875	90.75	103.714286	2.77320663	177.2539649	63.9166093
ZNF367	1	63.125	63.125	2.80184954	120.5375611	43.0207117
ZNF37A	7.375	362.875	49.2033898	19.6691433	663.7486392	33.7456812
ZNF382	4	273.875	68.46875	11.9580299	548.9287308	45.9046126
ZNF384	10.75	687.5	63.9534884	28.4702807	1302.651372	45.754778
ZNF395	2.875	235	81.7391304	7.74250391	475.6835844	61.4379521
ZNF41	3.125	170.875	54.68	8.89607368	335.6979677	37.7355201
ZNF423	5.125	276.75	54	15.2896618	579.5888551	37.9072384
ZNF429	9.125	213.625	23.4109589	60.2696396	968.1848366	16.0642214
ZNF43	7.625	236.25	30.9836066	22.0194104	475.641429	21.6010066
ZNF431	3	55.125	18.375	8.3161549	106.8347029	12.8466466
ZNF433	7.875	442.25	56.1587302	22.4556145	863.4863914	38.4530288
ZNF45	5.625	382.5	68	15.7553483	750.4297603	47.63016
ZNF467	2.125	76.75	36.1176471	6.14846028	156.0685139	25.3833491
ZNF468	7.75	272.75	35.1935484	21.0575504	516.0607891	24.5071615
ZNF470	2.75	201.125	73.1363636	7.59649961	397.5831638	52.3376798
ZNF492	0.125	0	0	0.34301269	0	0
ZNF496	8.625	511.5	59.3043478	23.6710841	994.5609028	42.0158578
ZNF512B	2.625	158.375	60.3333333	6.76627987	288.1014355	42.5790007
ZNF513	2.375	111	46.7368421	6.37147425	204.9889694	32.172926
ZNF517	1	29.375	29.375	3.3348819	65.71180796	19.7043883
ZNF536	19.25	1070.375	55.6038961	50.2937467	1972.510729	39.2198008

ZNF580	8.25	456.5	55.3333333	22.9677391	906.9408404	39.4875976
ZNF589	4	301.125	75.28125	11.287577	605.7131674	53.6619299
ZNF592	5.375	320.125	59.5581395	14.9839647	662.6827518	44.2261286
ZNF639	3.875	229.875	59.3225806	11.4301787	464.654167	40.6515226
ZNF652	20.75	726.75	35.0240964	58.0705363	1338.964299	23.0575501
ZNF668	9.875	506.25	51.2658228	27.8564748	967.5878909	34.7347572
ZNF674	0.5	61.25	122.5	1.35922748	125.5318688	92.3553052
ZNF687	13	859.875	66.1442308	38.3809363	1754.935825	45.7241536
ZNF69	0.5	20.75	41.5	1.45217984	41.33299136	28.4627222
ZNF692	5.125	327.875	63.9756098	13.9696649	664.9088969	47.5966247
ZNF7	11.375	508	44.6593407	35.200362	1087.796225	30.902984
ZNF705D	0	0		0	0	
ZNF711	9.75	642.625	65.9102564	26.8088086	1299.037361	48.4556169
ZNF720	1.875	82.75	44.1333333	5.75293077	161.37128	28.0502732
ZNF746	3	199.375	66.4583333	8.10866663	379.1280317	46.7559032
ZNF750	0	0		0	0	
ZNF76	6.75	434.125	64.3148148	18.9500959	869.431116	45.8800378
ZNF8	5.375	401.125	74.627907	14.9359657	796.3945645	53.3205942
ZNF80	0	0		0	0	
ZNF804A	3.125	189	60.48	8.15119989	359.9810303	44.1629496
ZNF821	26.5	1597	60.2641509	71.1765331	2996.014586	42.0927299
ZNF84	24.125	1503.5	62.3212435	67.1425658	2867.856863	42.7129471
ZNF85	6.25	150.5	24.08	16.2365359	284.911026	17.5475254
ZNF92	14.625	305	20.8547009	190.84619	2967.28548	15.5480468
ZNF93	10.75	408.375	37.9883721	30.270347	775.1137551	25.6063717
ZNF98	0	0.125		0	0.298740748	
ZNRD1	11.75	719.75	61.2553191	79.3361465	3049.955872	38.4434587
ZSCAN10	0	1		0	2.248878247	
ZSCAN12	4.875	265.25	54.4102564	13.5923971	486.4612776	35.789219
ZSCAN21	5.5	372	67.6363636	15.0230875	703.6370863	46.8370489
ZSCAN26	19.625	1123.5	57.2484076	52.2931432	2166.803139	41.4357028
ZSCAN31	3.125	226.75	72.56	10.6234573	438.5672555	41.2829122
ZSCAN4	0	0		0	0	
ZSCAN9	8.875	643.625	72.5211268	23.542262	1243.431233	52.8169823

TF mini-bulk 850ng

Gene	RAW_avePre	RAW_avePost	RAW_ratio	CPM_avePre	CPM_avePost	CPM_ratio
AEBP1	1.5	91.375	60.9166667	4.41820538	175.542675	39.7316693
AEBP2	8	276.5	34.5625	111.066366	2352.41476	21.1802624
AHR	3.125	193.5	61.92	9.96931878	351.874449	35.2957366
AIRE	0	0		0	0	
AKNA	3	186.875	62.2916667	7.77354245	294.739499	37.9157251
ALX1	0.125	24.125	193	0.27984365	41.3297888	147.688859
ALX3	0.125	14.875	119	0.35939162	23.006258	64.0144529
ALX4	0.125	0	0	0.27984365	0	0
AR	0.375	20.625	55	1.31684328	37.3746609	28.3820114
ARHGAP35	34	2398.75	70.5514706	87.1477992	3775.91136	43.3276732
ARID3A	0.375	15.5	41.3333333	1.07248816	22.0792379	20.5869292
ARID3B	1.875	157.25	83.8666667	5.55892141	253.435283	45.5907296
ARID3C	0	2.875		0	5.40355707	
ARID5A	5.125	301.25	58.7804878	19.8632282	584.66248	29.4344139
ARID5B	12.5	888.875	71.11	37.2060592	1556.90449	41.8454554
ARNT	14.875	914.875	61.5042017	64.7577703	2207.44542	34.0877304
ARNTL	4.75	351.5	74	13.6350067	604.017666	44.2990371
ARNTL2	0.75	52	69.3333333	1.94869808	105.689313	54.235858
ARX	9.75	541.125	55.5	24.0035924	840.547797	35.0175834
ASCL2	0	4.75		0	8.97074961	
ASCL3	0	0		0	0	
ATF1	2.25	210.25	93.4444444	6.33087383	351.496795	55.5210551
ATF2	27.25	2164	79.412844	77.523118	3680.20282	47.4723272
ATF3	5.25	303.25	57.7619048	16.7673679	562.178604	33.5281367
ATF5	1.5	122.375	81.5833333	4.1124468	217.862876	52.9764606
ATF6	21.25	1577.125	74.2176471	61.2449559	2697.78345	44.0490716
ATF6B	13.125	780.125	59.4380952	37.8072036	1288.29168	34.0752966
ATF7	10.75	834.625	77.6395349	31.2770015	1489.22433	47.6140376
ATOH1	0	4.75		0	9.97584795	
ATOH7	1	96.625	96.625	2.92492212	163.945293	56.0511651
BACH1	11.375	827.875	72.7802198	72.8893364	2528.02908	34.6831129
BACH2	20.875	1607.125	76.988024	58.5589544	2691.70865	45.9657909
BARHL1	0	0		0	0	
BARHL2	0	7.625		0	13.7487185	
BARX1	0	0		0	0	
BARX2	0	0		0	0	
BATF	0	0		0	0	
BATF3	0.25	9.125	36.5	0.70240432	14.8133319	21.0894659
BBX	19.75	1301	65.8734177	57.1366131	2290.61424	40.0901298
BCL11B	23	1700.75	73.9456522	61.0931438	2660.91688	43.5550819
BCL6	7.625	524.25	68.7540984	20.7959921	852.084377	40.9734902
BCL6B	0	4.375		0	6.26291523	
BHLHA15	0	0		0	0	
BHLHA9	0	0		0	0	
BHLHE22	4	330.25	82.5625	12.8468438	634.431279	49.3842136
BHLHE23	0	0.25		0	0.48483062	
BHLHE40	11.5	760.875	66.1630435	37.262819	1486.19977	39.8842549
BHLHE41	1.125	51.25	45.5555556	3.85757747	103.414423	26.8081259
BMPR1A	4.875	686.75	140.871795	15.9541684	1182.9644	74.1476693
BNC1	0	0		0	0	

BNC2	0.25	9.125	36.5	0.75395904	18.3421647	24.3278
BRIP1	2.5	184	73.6	7.67467375	320.145249	41.7145092
BSX	0	0		0	0	
BTBD11	2.375	132.625	55.8421053	6.48699554	218.430351	33.6720365
BTBD3	10.125	767.125	75.7654321	29.3831085	1384.94933	47.1342006
BTG2	19.875	1505.25	75.7358491	99.5845482	3241.49167	32.5501469
CAMTA1	23.125	1429.25	61.8054054	62.6542633	2407.30799	38.4220939
CARF	9.125	315	34.5205479	142.685174	3656.59125	25.626988
CASZ1	1	60	60	3.18502503	101.648561	31.914525
CC2D1A	1.75	118.875	67.9285714	4.37358662	198.697576	45.4312657
CC2D1B	2.25	133.25	59.2222222	6.16191176	238.385556	38.6869473
CDC5L	28.25	1946.25	68.8938053	77.4975978	3179.28991	41.0243672
CDX1	0	0		0	0	
CDX2	0	0		0	0	
CDX4	0	0		0	0	
CEBPA	0.875	41.625	47.5714286	2.49880582	70.7776752	28.3245999
CEBPB	0.125	21.875	175	0.27984365	36.9360482	131.988161
CEBPD	0.25	14.5	58	0.73637114	31.4064023	42.6502351
CEBPE	0	0		0	0	
CEBPG	25.875	1923.125	74.3236715	73.2154632	3289.83001	44.9335409
CEBPZ	13.375	918.5	68.6728972	39.9751814	1578.4023	39.4845563
CGGBP1	28	2098.25	74.9375	79.4725959	3513.5104	44.210339
CHCHD3	24.75	1670.25	67.4848485	69.0299237	2765.48554	40.0621266
CIC	5	222.125	44.425	44.8327915	1186.92637	26.4745141
CLOCK	1.75	220	125.714286	5.17240859	370.867018	71.701029
CNOT3	2.25	135.875	60.3888889	6.39223051	219.477053	34.3349715
CREB1	25.375	1851.25	72.955665	79.1974495	3298.60152	41.6503503
CREB3L1	0.125	7.375	59	0.37697952	13.2549623	35.1609614
CREB3L2	3	161.25	53.75	8.33712879	290.591728	34.8551325
CREB3L3	0	0		0	0	
CREB3L4	8	415.25	51.90625	23.3662493	711.639668	30.4558793
CREB5	15.75	999.125	63.4365079	85.4494688	2719.52263	31.8260917
CREBBP	6.625	493.125	74.4339623	17.129177	798.660032	46.6257096
CREBL2	3.125	394.25	126.16	9.02804844	691.012486	76.5406268
CREBRF	2.375	225.125	94.7894737	6.42261753	367.57798	57.2318028
CREM	7.625	454.375	59.5901639	24.3060108	812.166988	33.4142445
CRX	0.375	4	10.6666667	1.40979564	8.40071406	5.9588169
CSRNP1	4.125	274.5	66.5454545	12.2504018	491.203973	40.0969683
CSRNP2	25	2153.125	86.125	66.934328	3429.54539	51.2374665
CSRNP3	34.5	1791.25	51.9202899	104.396336	3422.24214	32.7812477
CTBP2	18	757.25	42.0694444	48.573856	1257.31557	25.8846153
CTCF	13.5	1025	75.9259259	36.3643597	1624.36988	44.6692832
CTCFL	0	0		0	0	
CTNNBL1	34.375	2226.875	64.7818182	91.9326482	3668.1882	39.9008217
CUX1	13.5	860.375	63.7314815	37.8714082	1386.64974	36.6146867
CUX2	0.625	65.75	105.2	1.48495622	96.6226662	65.067687
DACH1	7.125	439	61.6140351	21.6490431	809.721728	37.4021949
DACH2	3.75	278.5	74.2666667	10.2152985	456.251151	44.6635163
DBP	1.625	99.125	61	5.37171739	200.918358	37.4030024
DBX1	0	3.25		0	6.30279805	
DBX2	0	0		0	0	
DDIT3	13.5	546.125	40.4537037	39.2958755	950.016712	24.1759905

DDN	0.5	68.625	137.25	1.43998443	109.138002	75.7910985
DEAF1	15.875	1023.75	64.488189	44.2737	1669.27975	37.7036423
DENND4A	4.875	346.375	71.0512821	13.5271479	604.243055	44.6689176
DIDO1	14.375	1103.625	76.773913	40.3788741	1923.58833	47.6384837
DLX2	37.75	2035.125	53.910596	91.083006	2971.20679	32.6208688
DLX3	0.25	15.125	60.5	0.75395904	29.3145684	38.8808501
DLX6	29.25	1877.875	64.2008547	68.1108439	2634.19741	38.6751544
DMBX1	1	43.625	43.625	2.87513297	80.5474975	28.0152251
DMRT1	0.25	11	44	0.75395904	22.5590745	29.9208225
DMRT2	0	3		0	4.52515989	
DMTF1	12.375	897.5	72.5252525	65.5350153	1894.59459	28.9096535
DPF1	14	737.625	52.6875	38.1552351	1161.60482	30.4441794
DPF3	0.5	61	122	1.27847054	110.105551	86.1228692
DRGX	0	0		0	0	
DUX4	0	0		0	0	
E2F1	6.125	461	75.2653061	18.1446932	722.871704	39.8392906
E2F2	3.875	287.875	74.2903226	9.59902007	442.517452	46.1002736
E2F3	3.875	296.625	76.5483871	10.5276836	512.931631	48.7221741
E2F4	3.125	250.75	80.24	8.75243239	428.445244	48.9515628
E2F5	1	35.25	35.25	2.92758424	61.625359	21.0499012
E2F6	3.75	218	58.1333333	11.9789904	397.017182	33.1427916
E2F7	3.625	173.75	47.9310345	9.44629028	275.458893	29.1605365
E2F8	0.375	35	93.3333333	1.09278822	70.4271986	64.4472528
E4F1	15.125	738.625	48.8347107	39.1980054	1150.45212	29.3497618
EAF2	1.125	93.125	82.7777778	3.17314236	154.716055	48.7579938
EBF1	11.125	807.625	72.5955056	26.4946651	1162.14641	43.8634118
EBF2	0.125	31.125	249	0.30127814	41.5115125	137.78468
EBF3	0.375	37	98.6666667	1.29925538	69.6664787	53.6203119
EBF4	0.625	61.375	98.2	1.73202349	112.429253	64.9120831
EED	6.125	415	67.755102	15.9992347	649.067077	40.5686326
EGR1	37.625	1935.875	51.4518272	97.7697591	2975.56463	30.4344069
EGR2	0.125	10.5	84	0.46993188	21.6904722	46.1566307
EGR3	1.25	72.125	57.7	2.88417444	101.619648	35.2335304
EGR4	0	0		0	0	
EHF	0	0.875		0	1.69690717	
ELF1	1.75	158.75	90.7142857	4.88486579	269.640938	55.199252
ELF2	11.5	638.625	55.5326087	31.7689325	1046.42773	32.9387123
ELF3	0	0.125		0	0.18854833	
ELF4	0.75	71.25	95	1.98593042	114.396038	57.6032458
ELF5	0	0		0	0	
ELK1	8.75	777.25	88.8285714	23.3145199	1235.90036	53.0098998
ELK3	0	45.5		0	88.3354317	
ELK4	2.125	143.375	67.4705882	6.44519212	246.327562	38.2188082
EMX1	6.25	376.5	60.24	18.5502657	673.448126	36.3039612
EMX2	3.375	236.25	70	10.2307669	435.502455	42.5679186
EN1	0.125	3.375	27	0.27984365	4.65563132	16.6365447
EN2	0	1		0	1.93932248	
EOMES	9.25	737.25	79.7027027	28.2754832	1394.25428	49.3096536
EP300	9.125	691.75	75.8082192	24.7576917	1165.16083	47.0625791
EPAS1	1.75	100.75	57.5714286	5.64952271	194.196755	34.3740108
ERF	0.375	30	80	1.12675504	53.697156	47.6564594
ERG	0	0		0	0	

ESR1	0	0		0	0	
ESR2	0.625	52.125	83.4	1.70729574	85.1304116	49.8627211
ESRRA	0.75	34.375	45.8333333	2.17722762	64.5865354	29.6645765
ESRRG	7.125	522	73.2631579	17.4582803	774.60782	44.369079
ESX1	0	0		0	0	
ETS1	0.125	2.125	17	0.27984365	9.84411923	35.1772119
ETS2	2.5	188.5	75.4	6.89772955	330.94587	47.978957
ETV1	13.125	756.125	57.6095238	35.7764671	1246.17661	34.8322992
ETV2	2.375	70.5	29.6842105	28.6078251	539.152876	18.8463427
ETV3	2.125	162.75	76.5882353	5.77181798	262.354493	45.4543947
ETV4	0.875	114.875	131.285714	2.51576584	209.633043	83.3277248
ETV5	4.75	519.25	109.315789	12.9135056	878.455489	68.026105
ETV6	1.875	231.125	123.266667	4.97348676	369.433484	74.2805806
ETV7	0.25	13.875	55.5	0.62285634	20.9072441	33.5667196
FAM170A	0	0		0	0	
FERD3L	0	0		0	0	
FEV	0	0.875		0	1.8376562	
FEZF1	3.5	329.625	94.1785714	11.2477649	536.110074	47.6636983
FEZF2	18.375	1068.75	58.1632653	55.2148259	1910.60204	34.6030619
FIGLA	0	0		0	0	
FLI1	0	4.5		0	6.78773983	
FOS	8.125	536.125	65.9846154	24.1099082	956.413907	39.6689154
FOSL1	0.125	8.125	65	0.37697952	15.7031282	41.6551227
FOSL2	3.375	173.75	51.4814815	10.1396087	317.034609	31.266947
FOXA1	0	0		0	0	
FOXA2	0	0		0	0	
FOXA3	0	1.875		0	3.93783472	
FOXB1	0.125	9.75	78	0.37697952	19.4713903	51.6510561
FOXB2	0	0.125		0	0.22473122	
FOXC1	0	1.75		0	3.6753124	
FOXC2	0	0		0	0	
FOXD1	0	0.125		0	0.26252231	
FOXD2	0	0		0	0	
FOXD3	0	0		0	0	
FOXD4	0	1.75		0	3.6753124	
FOXD4L1	0	3.375		0	4.86128496	
FOXD4L3	0	0		0	0	
FOXD4L5	0	0		0	0	
FOXD4L6	0	0		0	0	
FOXE1	0	0		0	0	
FOXE3	0	0		0	0	
FOXF1	0	2.25		0	3.39386992	
FOXF2	0	0		0	0	
FOXG1	38.25	2716.875	71.0294118	116.400848	4742.81865	40.7455679
FOXH1	0	0.125		0	0.22473122	
FOXI1	0	0		0	0	
FOXI2	0	0		0	0	
FOXJ1	0	11.125		0	19.5530885	
FOXJ2	6.5	506	77.8461538	17.4911096	860.937456	49.2214316
FOXJ3	28.125	1622.75	57.6977778	198.365215	5536.63967	27.9113435
FOXK1	4.25	332	78.1176471	12.3177302	606.117503	49.2069151
FOXK2	14.5	952.75	65.7068966	38.8037589	1578.16177	40.6703323

FOXL1	0	0		0	0	
FOXL2	0	0		0	0	
FOXM1	5.125	360.75	70.3902439	13.6761017	579.470602	42.3710363
FOXN1	0	1.625		0	3.41279009	
FOXN2	7.125	536.125	75.245614	21.5966386	969.33703	44.8836992
FOXN3	12	1046.5	87.2083333	30.1784908	1656.72341	54.8974906
FOXN4	0.375	30.25	80.6666667	1.17233619	60.1173499	51.2799572
FOXO1	1.375	167.875	122.090909	3.30402296	270.285605	81.8050021
FOXO3	13.375	1030.625	77.0560748	36.0920682	1695.90365	46.9882645
FOXO4	0.25	12.25	49	0.75395904	20.5640228	27.2747215
FOXO6	0.125	11	88	0.27984365	17.8355945	63.7341413
FOXP1	29.125	2036.25	69.9141631	74.6802192	3079.62305	41.2374666
FOXP2	6.75	572	84.7407407	16.8445527	839.277374	49.8248537
FOXP3	0	2.125		0	4.12106027	
FOXP4	0.75	62.25	83	1.94148371	92.3639767	47.5739128
FOXQ1	0	0		0	0	
FOXR1	0	6.875		0	12.6608467	
FOXR2	0	0		0	0	
FOXS1	0.25	25.375	101.5	0.73637114	49.6495489	67.4246262
FUBP1	52	2446.5	47.0480769	307.390013	8424.97963	27.4081112
FUBP3	7.5	521.875	69.5833333	21.2558375	876.282236	41.2254861
GABPA	4.5	379.25	84.2777778	12.9241758	650.637426	50.3426631
GATA1	0	0		0	0	
GATA2	0	0.625		0	0.9492885	
GATA3	0	0.25		0	0.52504463	
GATA4	0	0		0	0	
GATA5	0	0		0	0	
GATA6	0	0		0	0	
GBX1	0	0		0	0	
GBX2	0.25	14.125	56.5	0.8469114	28.9612764	34.1963474
GCFC2	3.625	224.375	61.8965517	10.380374	391.803775	37.7446685
GCM1	0	0		0	0	
GFI1	0.125	0.25	2	2.21012058	4.40497586	1.993093
GFI1B	0.125	1.125	9	0.35939162	2.02258099	5.62779116
GLI1	0	12.375		0	16.6559821	
GLI2	0.875	75.5	86.2857143	2.4344278	117.46854	48.253039
GLI3	14.5	975.625	67.2844828	47.2552012	1824.89288	38.6178206
GLIS1	0	0		0	0	
GLIS2	1	83	83	3.03425341	148.077345	48.8019045
GLIS3	1.75	168.375	96.2142857	6.11428451	343.613229	56.1984364
GLMP	4.375	284	64.9142857	12.651135	485.734583	38.3945457
GMEB1	11.75	712.75	60.6595745	32.3074108	1152.05951	35.6592954
GMEB2	2.875	292	101.565217	8.03006493	484.85695	60.380203
GPER1	0.875	36.5	41.7142857	2.56850505	67.8690951	26.4235787
GRHL1	1	97.625	97.625	2.79560948	165.899906	59.3430188
GRHL2	0	0.5		0	0.96966124	
GRHL3	0	7		0	13.5752573	
GSC	0	3.75		0	7.27245929	
GSC2	0	0.875		0	1.3198383	
GSX1	1.5	29.625	19.75	3.42242724	43.6493843	12.7539262
GSX2	0.875	64.25	73.4285714	2.0875125	92.1222761	44.1301674
GZF1	6.875	397.75	57.8545455	18.9811271	663.427101	34.951934

HAND1	0	0		0	0	
HAND2	0	0.625		0	1.31261157	
HBP1	16.75	1189.75	71.0298507	51.6111993	2077.70738	40.2569096
HCFC1	17.125	1241.625	72.5036496	46.3556132	2014.82904	43.4646184
HDAC5	22.5	1339.625	59.5388889	63.2082926	2299.93372	36.386582
HDGF	18.625	1580.25	84.8456376	52.3213692	2583.46434	49.3768488
HELT	0.75	40.5	54	1.67906187	56.0027856	33.3536163
HES1	2.125	219	103.058824	6.2730287	408.748214	65.1596276
HES2	0	0.875		0	1.66153899	
HES3	0	0		0	0	
HES4	3.25	84.25	25.9230769	8.73021889	142.251684	16.2941715
HES5	0.5	28.75	57.5	1.48999326	55.3922861	37.176199
HES6	25.875	1377.75	53.2463768	66.7403898	2190.49865	32.8211846
HES7	0	1.375		0	2.25159234	
HESX1	0.25	19.75	79	0.70240432	38.7839096	55.2159329
HEY1	40.75	2777.75	68.1656442	117.084221	4733.79703	40.4307002
HEY2	2.625	228.125	86.9047619	8.92145796	457.648386	51.2974884
HEYL	0	0		0	0	
HHEX	0	0		0	0	
HIC2	1.5	100.875	67.25	4.34756283	168.623454	38.785743
HIF1A	42	3264	77.7142857	118.377776	5486.39092	46.3464607
HIF3A	6	350.625	58.4375	18.2547753	640.603416	35.0923747
HINFP	14.125	879.375	62.2566372	40.9295569	1567.99304	38.3095532
HIVEP1	3	202.375	67.4583333	9.18383263	388.068054	42.2555669
HIVEP2	13	919.75	70.75	37.9123735	1585.05909	41.8084901
HIVEP3	2.875	246.375	85.6956522	7.31377175	447.687384	61.2115608
HLF	1	57.25	57.25	3.38764559	113.802767	33.5934689
HLTF	14.375	1213.875	84.4434783	41.5576702	2149.19185	51.7158888
HLX	0.5	16	32	1.78677516	33.5750649	18.7908729
HMBOX1	9.25	808.125	87.3648649	23.7062312	1274.57523	53.7654099
HMG20A	27.375	2110.75	77.1050228	75.1590684	3520.17365	46.836313
HMGA1	34.5	1356	39.3043478	92.5964606	2208.40914	23.8498224
HMGA2	1.625	112.875	69.4615385	5.03235747	207.630889	41.2591693
HMX1	0.125	11.875	95	0.46993188	17.9250515	38.1439359
HNF1A	0	0.125		0	0.26252231	
HNF1B	0	1.125		0	2.36270083	
HNF4A	0	0		0	0	
HNF4G	0.125	14.125	113	0.46993188	28.9411694	61.5858823
HNRNPAB	45.75	2170.5	47.442623	281.721356	7648.09785	27.1477391
HOMEZ	4.875	262.375	53.8205128	14.4466919	452.404505	31.3154394
HOXA1	0	0		0	0	
HOXA10	0	0		0	0	
HOXA11	0	0		0	0	
HOXA13	0	0		0	0	
HOXA2	0	0		0	0	
HOXA3	0	0		0	0	
HOXA4	0	0		0	0	
HOXA5	0	0		0	0	
HOXA6	0	0		0	0	
HOXA7	0	0		0	0	
HOXA9	0	0		0	0	
HOXB1	0	0		0	0	

HOXB13	0	2		0	4.20035703	
HOXB2	0.125	6.5	52	0.27984365	9.03397609	32.282227
HOXB3	0	0		0	0	
HOXB4	0	0		0	0	
HOXB5	0	0		0	0	
HOXB6	0	0		0	0	
HOXB7	0	0		0	0	
HOXB8	0	0		0	0	
HOXB9	0	0		0	0	
HOXC10	0	0		0	0	
HOXC11	0	0		0	0	
HOXC13	0	0		0	0	
HOXC4	0	0		0	0	
HOXC5	0	0		0	0	
HOXC6	0	0		0	0	
HOXC8	0	0		0	0	
HOXD1	0	0		0	0	
HOXD10	0	0		0	0	
HOXD11	0	0		0	0	
HOXD13	0	0		0	0	
HOXD3	0	0		0	0	
HOXD4	0	0		0	0	
HOXD8	0	3.625		0	6.51720542	
HOXD9	0	0		0	0	
HR	0	2.875		0	4.76754741	
HSF1	16.75	700.625	41.8283582	44.7200875	1144.27059	25.5873961
HSF2	26	1666.5	64.0961538	72.6550048	2770.48934	38.1321197
HSF4	0	2.25		0	7.25006923	
HTATIP2	0.25	63.875	255.5	0.71999221	117.269865	162.876574
IER2	11.375	733.375	64.4725275	32.0895483	1217.70371	37.9470504
IFI16	3.25	219.875	67.6538462	10.3123315	406.330639	39.4024029
IGHMBP2	7.625	503.125	65.9836066	26.2623755	865.706408	32.963751
IKZF1	0.25	12.125	48.5	0.93986376	23.0688863	24.5449259
IKZF2	1.625	149.625	92.0769231	4.74004597	248.490145	52.4235728
IKZF3	0	0		0	0	
IKZF5	8	439.125	54.890625	22.0960968	737.516932	33.3777019
INSM1	4.125	250.125	60.6363636	10.4601802	401.909245	38.4227841
IRF1	2.75	110.25	40.0909091	7.85490845	188.714508	24.0250423
IRF2	3.125	309.625	99.08	9.52655435	519.491315	54.5308719
IRF3	8.125	529.75	65.2	22.1715575	883.652873	39.8552457
IRF4	0	2.125		0	4.12106027	
IRF5	0.875	39.75	45.4285714	2.27760073	77.7699093	34.145541
IRF6	0.25	12.875	51.5	0.8469114	25.7820039	30.4423862
IRF7	1.75	98.75	56.4285714	5.53504721	179.661803	32.4589469
IRF8	0	7.875		0	13.2281995	
IRF9	7.5	382.75	51.0333333	25.5417451	721.18896	28.2356964
IRX1	0	1.375		0	2.68667541	
IRX2	0	9.5		0	13.3408464	
IRX3	0	2.625		0	5.35211257	
IRX5	0	1		0	2.10017852	
IRX6	0	0		0	0	
ISL1	2.125	121	56.9411765	5.2017942	169.077415	32.5036724

ISL2	0.375	24.375	65	0.83953094	36.8075465	43.84299
JARID2	16.625	836	50.2857143	137.974939	3913.36266	28.3628511
JDP2	0.375	36.5	97.3333333	1.22389092	72.1167597	58.9241726
JUN	33	1935.375	58.6477273	96.5450113	3389.24511	35.1053365
JUNB	5.25	234.875	44.7380952	13.8606463	376.474876	27.1614228
JUND	2	110.5	55.25	5.30752276	181.51217	34.1990375
KCNH8	0.875	86.25	98.5714286	2.33963534	151.906226	64.9273089
KCNIP3	1.75	156.25	89.2857143	5.06312735	282.414846	55.7787364
KHSRP	1.625	149.875	92.2307692	4.37119286	238.177057	54.4878857
KLF1	0.125	8.125	65	0.46993188	16.9751584	36.1225939
KLF10	33.5	2151.25	64.2164179	138.334822	4486.22155	32.4301682
KLF11	9.375	704.375	75.1333333	28.6984407	1188.41794	41.4105406
KLF12	27.625	2158.875	78.1493213	72.4259162	3400.46635	46.9509608
KLF13	1.375	107.125	77.9090909	3.79486425	189.516319	49.9402105
KLF14	0	0		0	0	
KLF15	0	4.375		0	8.41585084	
KLF16	0.875	34.5	39.4285714	2.10162254	55.9430574	26.6189842
KLF17	0	0		0	0	
KLF2	0	4.375		0	6.74392308	
KLF3	9	579.125	64.3472222	26.2858045	1025.96457	39.031127
KLF4	0.25	14.625	58.5	0.93986376	26.5217795	28.2187491
KLF6	33.5	2063	61.5820896	93.7501648	3481.57484	37.136733
KLF8	1	87	87	2.92755975	156.721691	53.5332167
KLF9	0.25	17.625	70.5	0.93986376	38.2992491	40.7497883
KMT2A	29.875	2167.125	72.539749	79.4332094	3511.73102	44.2098593
KRBOX4	6.25	410.625	65.7	16.6544903	666.819866	40.0384434
LBX1	0	0		0	0	
LCORL	10.5	844	80.3809524	30.6476658	1435.44888	46.8371355
LEF1	6.75	554.625	82.1666667	19.196831	950.388062	49.5075496
LHX1	0.75	20.125	26.8333333	2.20137428	34.4152009	15.6335073
LHX2	16.5	1113.25	67.469697	51.1916753	2104.7467	41.115019
LHX3	0	0		0	0	
LHX4	0	3.875		0	5.16809995	
LHX6	1	100.25	100.25	2.32448715	145.663092	62.6646146
LHX9	0.625	96.5	154.4	1.78178815	178.9898	100.455153
LITAF	27	1875	69.4444444	81.8042202	3347.12214	40.9162527
LMO2	1.75	148	84.5714286	4.91915131	258.97399	52.6460712
LMX1A	3.875	274.75	70.9032258	12.346749	500.939474	40.572581
LMX1B	0	0		0	0	
LRRFIP1	9.625	495.875	51.5194805	26.4638291	819.635114	30.9719017
LZTFL1	5	338.375	67.675	14.250815	577.483718	40.5228555
MACC1	0	1		0	1.51524613	
MAF	1.25	119.25	95.4	3.10757389	212.423567	68.3567229
MAFA	0	0		0	0	
MAFB	6.125	267.625	43.6938776	15.7941462	417.628874	26.4420038
MAFF	2	110	55	5.8942877	203.788462	34.5738911
MAFG	6.375	358.875	56.2941176	17.3433449	575.379829	33.1758281
MAFK	0.375	18.375	49	0.83953094	28.5360386	33.9904551
MAX	14.625	1172.5	80.1709402	43.0446815	1921.36256	44.6364682
MAZ	20.875	1332.25	63.8203593	54.2447582	2090.58568	38.5398654
MECOM	0	0.875		0	1.69690717	
MED1	14.75	1096	74.3050847	39.4090923	1734.91169	44.0231325

MEF2A	6.75	436.125	64.6111111	19.336448	749.278886	38.7495617
MEF2B	0	0.125		0	0.26252231	
MEF2BNB-ME	0	1.25		0	2.38319418	
MEF2C	16.125	1056.375	65.5116279	79.4884567	2418.48809	30.4256516
MEF2D	4	277.375	69.34375	11.2611084	459.186447	40.7763099
MEIS1	17.125	1206.625	70.459854	41.2472779	1832.6508	44.4308303
MEIS2	40.125	2884.5	71.8878505	107.912753	4480.55436	41.5201563
MEOX1	0	0		0	0	
MEOX2	0	0		0	0	
MESP1	2.625	100.125	38.1428571	7.48963837	164.110936	21.9117303
MESP2	0	7.625		0	14.7873339	
MGA	11.875	984.75	82.9263158	31.7563845	1656.20412	52.1534219
MITF	0.5	56.625	113.25	1.26209161	96.479084	76.4438044
MIXL1	0	0		0	0	
MKX	0.5	19.875	39.75	1.6938228	41.187745	24.3164427
MLX	19.5	1331.125	68.2628205	54.7601339	2276.62163	41.574435
MLXIP	6.25	457.625	73.22	17.0844877	735.930537	43.07595
MLXIPL	0	3.5		0	6.81500443	
MN1	17.875	1326.25	74.1958042	44.6650799	2039.02376	45.6514073
MNT	0.75	54.125	72.1666667	1.85959232	90.7960585	48.8257869
MSC	0	0.125		0	0.17373031	
MSGN1	0	0		0	0	
MSX1	1.375	54.375	39.5454545	4.54360286	111.314666	24.4992067
MSX2	0.75	41.625	55.5	2.43019394	79.0394718	32.5239359
MTF1	9.25	556.375	60.1486486	25.6574174	947.6861	36.9361454
MTF2	32.5	2128.25	65.4846154	87.5128384	3503.78253	40.0373545
MXD1	2.75	283.75	103.181818	7.20829303	455.39104	63.1759888
MXD3	9.75	498.25	51.1025641	24.752623	770.317403	31.1206373
MXD4	18.25	972.625	53.2945205	120.070031	4178.22462	34.7982305
MXI1	12.375	1094.5	88.4444444	36.5227931	1793.67539	49.1111233
MYB	0.125	29.75	238	0.30127814	46.2743812	153.593556
MYBL1	0.25	8.375	33.5	0.55968729	23.027056	41.1427173
MYBL2	8.25	634.875	76.9545455	23.3330254	1029.03406	44.1020416
MYC	3	201.75	67.25	8.5044245	351.472053	41.3281408
MYCL	5.75	435.625	75.7608696	16.0534855	754.313142	46.9874995
MYCN	2.625	254.5	96.952381	7.55924248	452.643917	59.8795339
MYEF2	15	1046	69.7333333	41.8089641	1788.33057	42.7738551
MYF5	0	0		0	0	
MYF6	0	1		0	2.10017852	
MYOCD	0	0		0	0	
MYOD1	0	0		0	0	
MYOG	0	0		0	0	
MYPOP	0.125	9.25	74	0.37697952	39.0447071	103.572489
MYRF	0.25	26.625	106.5	0.8469114	53.8534324	63.588036
MYT1	8.375	652.5	77.9104478	21.2464796	1044.65186	49.1682332
MYT1L	19.875	1193.625	60.0566038	54.0183948	1987.11488	36.7858927
MZF1	3.125	178.75	57.2	9.12968026	314.830686	34.4843058
NACC1	0.25	12.875	51.5	0.55968729	20.3261341	36.3169478
NACC2	1.625	130.5	80.3076923	4.48758621	220.86437	49.2167414
NANOG	0	0		0	0	
NEUROD1	7.125	517.625	72.6491228	19.6969062	894.483379	45.4123794
NEUROD2	7.75	415.625	53.6290323	22.4127281	711.247637	31.7340948

NEUROD4	1.125	96	85.3333333	3.6037391	177.956853	49.3811699
NEUROG1	2.125	144	67.7647059	6.32040401	257.448029	40.7328438
NEUROG2	7.25	508.375	70.1206897	22.2897149	927.780545	41.6237063
NEUROG3	0	0.25		0	3.27645876	
NFAT5	8.875	728.125	82.0422535	24.2576861	1239.95017	51.1157643
NFATC1	0.75	65.625	87.5	2.27879118	109.108328	47.8799151
NFATC2	0	1.375		0	2.82742444	
NFATC3	10.875	792.75	72.8965517	30.3089023	1279.51599	42.2158472
NFATC4	1.5	142.5	95	4.763252	284.261403	59.6780105
NFE2	0	20.625		0	40.1465439	
NFE2L1	6	347.25	57.875	17.3720717	594.698921	34.2330455
NFE2L2	21.875	1768.875	80.8628571	61.7496265	3031.89479	49.0998078
NFE2L3	0.625	23.875	38.2	1.78299712	41.4090391	23.2244004
NFIC	5.5	383.75	69.7727273	14.6372766	640.933217	43.7877369
NFIL3	4.875	459.375	94.2307692	17.5697631	775.209106	44.1217733
NFIX	9.625	683.875	71.0519481	29.0509416	1275.89749	43.9193163
NFKB1	19.25	480	24.9350649	304.42278	6393.98573	21.0036376
NFKB2	0.5	15.25	30.5	1.75280833	29.3187357	16.7267209
NFKBIZ	0.125	6.25	50	0.37697952	12.7641896	33.8591063
NFX1	15.125	1021.25	67.5206612	42.6958646	1700.71592	39.8332704
NFXL1	16.375	1090.75	66.610687	47.5729281	1834.25397	38.5566758
NFYA	20.5	890.625	43.445122	58.9067196	1563.82399	26.5474636
NFYC	31.375	1809.625	57.6772908	92.00052	3028.62264	32.9196252
NHLH1	4.25	382.125	89.9117647	12.5225286	694.022713	55.4219307
NHLH2	1.625	131.125	80.6923077	5.62676473	263.444015	46.8198027
NKRF	3.625	155.125	42.7931034	10.1965321	296.648287	29.0930568
NKX1-2	0	0		0	0	
NKX2-1	0.5	16	32	1.11937458	29.9396321	26.74675
NKX2-2	0	0		0	0	
NKX2-3	0	0		0	0	
NKX2-5	0.375	9.375	25	1.02903808	14.6476852	14.234347
NKX2-6	0	0		0	0	
NKX2-8	0	0		0	0	
NKX3-1	0	15.5		0	28.0139166	
NKX3-2	0	0		0	0	
NKX6-1	0	0.125		0	0.18854833	
NKX6-2	0	0		0	0	
NKX6-3	0	4.625		0	6.96077348	
NOBOX	0	0		0	0	
NPAS1	5.375	321.875	59.8837209	12.7056662	459.024639	36.1275538
NPAS2	0.875	55.25	63.1428571	2.59949733	103.496034	39.8138644
NPAS3	4.25	233.25	54.8823529	16.6424825	408.534292	24.5476774
NPAS4	1.375	85	61.8181818	3.0782801	118.501133	38.4958902
NR0B1	0	0		0	0	
NR1D1	4	292.375	73.09375	12.0619828	542.508777	44.9767492
NR1D2	3.875	303.875	78.4193548	12.0326161	533.341486	44.3246492
NR1H2	19.5	1095.625	56.1858974	58.9609136	1896.51401	32.1656143
NR1H3	2.625	200.75	76.4761905	7.94745533	357.314527	44.9596144
NR1H4	0.125	5.625	45	0.37697952	10.9086889	28.9370864
NR1I2	0	0		0	0	
NR1I3	0	14		0	30.3241218	
NR2C1	5.25	331.875	63.2142857	14.8653503	596.740805	40.1430705

NR2E1	13.875	916.25	66.036036	39.6533776	1610.10007	40.6043614
NR2E3	0	1.625		0	2.89336399	
NR2F1	16.375	972.25	59.3740458	43.1706621	1592.4605	36.8875626
NR2F2	14.125	990.125	70.0973451	37.786281	1609.51224	42.5951483
NR2F6	6.125	366.375	59.8163265	17.3328811	614.675072	35.4629487
NR3C1	2.625	221.375	84.3333333	7.43222321	390.611215	52.556443
NR3C2	0.125	13.75	110	0.37697952	24.9686661	66.2334816
NR4A1	2	141.375	70.6875	6.06139146	254.395115	41.969755
NR4A2	12.5	802.375	64.19	37.4042145	1416.91766	37.8812302
NR4A3	7.75	438.625	56.5967742	24.6127411	807.154166	32.7941598
NR5A1	0	0		0	0	
NR5A2	0	0		0	0	
NR6A1	2	133.875	66.9375	7.63822617	262.733439	34.3971799
NRF1	4.625	306.5	66.2702703	12.6805908	512.259257	40.3971128
NRG1	6.125	368.375	60.1428571	16.628876	587.720486	35.3433681
NRL	1.5	84	56	3.94500203	133.048422	33.7258183
OLIG1	0.75	84.75	113	1.70049637	116.319252	68.403117
OLIG2	0.375	18	48	0.94051341	26.2053221	27.8627842
OLIG3	0.25	1.75	7	0.93986376	3.6753124	3.91047359
ONECUT1	0	0		0	0	
ONECUT2	1.125	127.625	113.444444	3.0843553	201.500346	65.3298102
ONECUT3	0	0		0	0	
OSR1	0	1.25		0	2.4241531	
OSR2	0	0		0	0	
OTP	0	1.875		0	2.86440782	
OTX1	15.625	1014.5	64.928	46.1410581	1784.92612	38.6841177
OTX2	7.875	560.5	71.1746032	24.3631893	1034.67853	42.4689277
OVOL1	0	0		0	0	
OVOL2	0	0		0	0	
PATZ1	17.375	1209.5	69.6115108	48.8963437	2035.47243	41.6283157
PAX1	0	0		0	0	
PAX2	0	2.375		0	4.98792397	
PAX3	0.25	45.25	181	0.75395904	89.7888	119.089759
PAX4	0	0		0	0	
PAX5	0	0		0	0	
PAX6	36.375	2231.75	61.3539519	102.678878	3777.78518	36.7922326
PAX7	0	0		0	0	
PAX8	0	0		0	0	
PAX9	0	0		0	0	
PBX1	39.625	3122.625	78.8044164	102.59524	4801.61268	46.8015153
PBX2	0.75	73.875	98.5	2.35030905	123.556117	52.5701576
PBX3	19.25	1414.875	73.5	54.6267352	2220.13315	40.6418788
PBX4	2.875	139.125	48.3913043	7.59201916	227.624939	29.9821344
PCGF6	4	242.875	60.71875	11.8001144	419.160373	35.5217213
PDX1	0	0		0	0	
PEG3	0	0.625		0	1.01671563	
PGR	0.125	0.25	2	2.21012058	4.40497586	1.993093
PGS1	7.375	602.25	81.6610169	20.9346599	1012.31592	48.3559764
PHB	73.625	2224.125	30.2088285	211.238972	3836.12447	18.1601171
PHF10	0.375	29.125	77.6666667	1.29925538	58.0829185	44.7047743
PHF20	30.5	2030	66.557377	120.89479	4553.34063	37.6636629
PHF6	41.875	2316.25	55.3134328	299.821991	8675.0335	28.9339466

PHF7	1.75	110.125	62.9285714	4.58873951	183.739592	40.0414082
PHOX2A	0	0.375		0	0.78756694	
PHOX2B	0	0		0	0	
PITX1	0.25	6.125	24.5	0.68602539	10.8461888	15.8101857
PITX2	0.375	16.625	44.3333333	1.07248816	23.3225289	21.7461877
PITX3	0	0		0	0	
PKNOX1	8.875	843	94.9859155	22.9067114	1342.4974	58.6071644
PKNOX2	3.375	273	80.8888889	9.03950893	436.665862	48.3063699
PLAG1	1.125	174.75	155.333333	3.3298949	335.98279	100.898917
PLAGL1	3.375	265.625	78.7037037	11.6184355	472.343254	40.6546349
PLAGL2	9.625	711.75	73.9480519	25.268068	1157.20307	45.7970536
PLSCR1	2.5	161.375	64.55	7.83057872	286.374256	36.571276
POU1F1	0	0		0	0	
POU2F1	18.125	1124.75	62.0551724	49.1893544	1898.19011	38.5894495
POU2F2	0.25	30.5	122	0.70240432	67.3149964	95.8351122
POU2F3	0	0		0	0	
POU3F1	0.25	9.5	38	0.55968729	36.6058372	65.4040886
POU3F2	3.625	274	75.5862069	9.10087816	414.958429	45.5954273
POU3F3	3.75	136.125	36.3	10.8011223	236.828735	21.9263081
POU3F4	0	9.625		0	14.9305806	
POU4F1	0	12		0	16.0044386	
POU4F2	0	0		0	0	
POU4F3	0	0		0	0	
POU5F1	0.25	1.375	5.5	0.55968729	1.9110334	3.41446631
POU6F1	3	217	72.3333333	8.12327998	350.92144	43.1994762
POU6F2	0.375	12.875	34.3333333	1.31684328	23.5301856	17.8686302
PPARA	2.75	157.75	57.3636364	8.28330996	293.230786	35.4001948
PPARD	2.625	183.5	69.9047619	8.01149643	305.738738	38.1625007
PPARG	1.125	81.25	72.2222222	3.66272464	145.403783	39.6982568
PRDM1	0.125	10.875	87	0.27984365	15.2095213	54.3500686
PRDM10	3.125	170.625	54.6	8.84893713	284.074749	32.1026971
PRDM12	0	1.875		0	3.93783472	
PRDM13	0	4.25		0	6.41064318	
PRDM14	0	0		0	0	
PRDM15	2.125	173.875	81.8235294	5.64163328	284.823341	50.4859722
PRDM16	2.375	118.75	50	9.77104673	239.410179	24.5019992
PRDM2	16.125	1446.125	89.6821705	43.6445423	2380.34172	54.5392755
PRDM4	3.25	360.375	110.884615	9.33051432	668.913731	71.6909817
PRDM5	0.625	26.75	42.8	1.97751304	49.3130447	24.9368999
PREB	38.625	2394.875	62.0032362	107.287166	4016.81486	37.439845
PROP1	0	0		0	0	
PROX1	2.375	169.125	71.2105263	6.67023405	327.12134	49.0419583
PRRX1	0	0.125		0	0.24241531	
PRRX2	0	0		0	0	
PTF1A	0	0		0	0	
PTH	0	0		0	0	
PURA	2.875	224.5	78.0869565	8.25621053	377.653544	45.7417532
PURB	2	160	80	5.19521692	260.011417	50.0482311
RAD21	35.875	2698.875	75.2299652	103.081263	4491.39345	43.5713855
RAI1	5.375	326.625	60.7674419	13.4701108	521.220675	38.6946094
RARA	6.25	447.875	71.66	15.7079835	689.273036	43.8804279
RARB	2.125	107.625	50.6470588	6.11139433	178.140124	29.1488512

RARG	1.75	146.875	83.9285714	5.40599695	259.998003	48.0943673
RAX	0	1.5		0	2.63875634	
RAX2	0	0		0	0	
RBPJ	77.75	4433.625	57.0241158	216.128926	7438.8153	34.418416
RBPJL	0	0		0	0	
RCOR1	4.875	284.625	58.3846154	14.8238438	494.767576	33.3764699
REL	3.25	266.25	81.9230769	9.04175929	436.30563	48.2545062
RELA	16.25	1021	62.8307692	45.0335903	1714.6706	38.0753697
RELB	0.625	37.625	60.2	1.78178815	66.1251553	37.111682
REST	11.25	584.125	51.9222222	79.0336677	1968.47786	24.9068267
RFX1	1.5	55.625	37.0833333	4.1360401	95.2143084	23.0206444
RFX2	4.25	281	66.1176471	13.0467498	504.993361	38.7064493
RFX3	29.25	2197.125	75.1153846	84.7443618	3844.05674	45.3606194
RFX4	8.625	571	66.2028986	27.5766419	1035.87453	37.5634763
RFX5	16.5	1221.25	74.0151515	45.0397051	2025.22025	44.96522
RFX6	0	1.375		0	2.47204344	
RFX7	20.625	1342	65.0666667	98.985818	3279.12485	33.1272188
RHOXF2	0	0		0	0	
RHOXF2B	0	0		0	0	
RNF112	3.125	214.875	68.76	9.3158792	384.690145	41.2940246
RNF141	25.625	1852.75	72.302439	83.3790486	3483.75227	41.7821062
RNF2	13.25	1029.5	77.6981132	38.0786919	1767.95835	46.4290727
RORA	5.875	512.125	87.1702128	19.2263586	998.550224	51.9365234
RORB	2.5	194.375	77.75	6.63575796	312.142638	47.0394851
RORC	0.125	23.125	185	0.46993188	46.3922441	98.7212108
RREB1	0.625	94.625	151.4	1.96113411	170.910251	87.1486811
RUNX1	0	0.625		0	1.12365611	
RUNX2	0.125	27.25	218	0.46993188	50.8727594	108.255604
RUNX3	0	0		0	0	
RXRA	3	203.625	67.875	8.18204908	336.965447	41.1835036
RXRB	6.125	438.375	71.5714286	15.6929228	707.492614	45.0835465
RXRG	14.5	829.875	57.2327586	33.5563835	1150.0521	34.27223
SALL1	17.875	1210.25	67.7062937	45.6010018	1910.79975	41.9025827
SALL2	24	1513.75	63.0729167	64.3669312	2475.02841	38.4518629
SALL3	0.5	26.25	52.5	1.18254363	46.7712341	39.5513814
SALL4	0.25	41	164	0.68602539	67.5595034	98.4795964
SARNP	40.75	1432.5	35.1533742	222.735016	4194.75091	18.8329208
SATB1	11.875	501.375	42.2210526	139.114197	2749.10439	19.7614942
SATB2	0.625	47.875	76.6	1.76540922	78.9543262	44.72296
SCML4	0	2.25		0	33.1453592	
SCRT1	0.625	31.375	50.2	1.56842532	50.5419536	32.2246479
SCRT2	6.75	440.125	65.2037037	18.0767377	668.840217	37.0000509
SCX	0	0		0	0	
SHOX	0	0		0	0	
SHOX2	0	0		0	0	
SIM1	0	0		0	0	
SIM2	0.25	42.375	169.5	0.63923527	67.1275961	105.012348
SIX1	0	0		0	0	
SIX2	0	2.625		0	5.5129686	
SIX3	12	627	52.25	28.2853101	899.027059	31.7842391
SIX4	1.25	62.75	50.2	3.61892758	97.5674584	26.9603235
SIX5	1.125	86.125	76.5555556	3.44640872	166.000676	48.1662767

SIX6	0	0		0	0	
SKIL	10	581.875	58.1875	29.8559474	1014.28705	33.9726969
SMAD1	17.25	1105.375	64.0797101	45.9975272	1779.78613	38.6930829
SMAD2	23.5	1875.875	79.8244681	103.44569	3842.93938	37.1493424
SMAD3	3	185.25	61.75	8.8006253	333.937937	37.9447966
SMAD4	16.25	1257.25	77.3692308	45.1500763	2051.51091	45.4375955
SMAD5	19.375	982	50.683871	155.241061	4561.63116	29.3841792
SNAI1	0.125	15.75	126	0.35939162	28.5412289	79.4153985
SNAI2	2.5	130.375	52.15	6.16161104	201.746846	32.7425482
SNAI3	0.125	7	56	0.37697952	14.0377185	37.2373504
SOHLH1	0	0		0	0	
SOHLH2	0	0		0	0	
SOX1	3.875	338.875	87.4516129	9.39448488	507.268695	53.9964353
SOX10	0	0		0	0	
SOX12	3.5	247.875	70.8214286	8.41175809	386.43283	45.9396033
SOX13	0.125	25.25	202	0.27984365	55.3097526	197.645197
SOX14	0	0		0	0	
SOX15	1.375	91.875	66.8181818	3.95572578	156.575807	39.582068
SOX17	0	0		0	0	
SOX18	0	0.125		0	0.18854833	
SOX2	25.875	1538.75	59.468599	81.7622696	2805.24805	34.3098114
SOX21	0.125	18.5	148	0.27984365	37.0081757	132.245903
SOX3	2.875	154.375	53.6956522	9.07264272	279.472555	30.8038753
SOX30	0.125	5	40	0.34301269	10.2121956	29.7720631
SOX5	34.25	2136.375	62.3759124	96.2437838	3551.39217	36.8999642
SOX6	12.5	799.375	63.95	32.2609876	1283.2352	39.776687
SOX7	0.125	3.25	26	0.37697952	5.97959617	15.8618595
SOX8	1.25	134.75	107.8	3.38597035	215.313798	63.5899832
SOX9	11.75	854.5	72.7234043	34.7514168	1519.19505	43.716061
SP1	4.125	456.125	110.575758	10.7309786	803.579055	74.8840426
SP110	1	75.5	75.5	2.56705221	122.360711	47.6658445
SP2	4	259.125	64.78125	10.5528586	407.555952	38.6204314
SP3	25.5	1973.125	77.377451	70.5832367	3330.32329	47.1829211
SP4	8.125	713.375	87.8	20.8172916	1158.52022	55.6518228
SP5	0.25	20.375	81.5	0.55968729	29.6230554	52.9278686
SP6	0	1.25		0	2.4442601	
SP7	0	3.125		0	6.56305786	
SP8	11.5	567.25	49.326087	28.0288669	900.831235	32.1394097
SP9	2.75	129	46.9090909	6.34947068	185.777528	29.2587426
SPDEF	0	0		0	0	
SPI1	0.125	3.75	30	0.30127814	5.00138705	16.6005639
SPIB	0	0		0	0	
SPIC	0	0		0	0	
SPZ1	0	0		0	0	
SREBF1	3	234.875	78.2916667	8.2299992	396.625177	48.1926142
SRF	3.125	210.375	67.32	8.06563155	317.370221	39.348465
SRY	0	0		0	0	
ST18	8.75	781.25	89.2857143	26.3974857	1406.96756	53.299302
STAG1	9.625	650.25	67.5584416	25.0512167	1060.94711	42.351121
STAG2	17.25	1147.75	66.5362319	52.377053	2014.66116	38.4645764
STAT1	14.625	1002.5	68.5470085	42.7504006	1795.34358	41.9959475
STAT2	4.875	325.5	66.7692308	13.7526531	577.62353	42.0008798

STAT3	25.125	1727.375	68.7512438	74.2805159	3090.45321	41.6051662
STAT4	0.875	41.125	47	2.68826617	71.3179805	26.5293598
STAT5A	0.5	7.75	15.5	1.50791808	14.8166768	9.82591629
STAT5B	7	403.875	57.6964286	18.430768	652.407699	35.3977491
STAT6	0.375	35.875	95.6666667	1.29925538	68.6947339	52.8723875
STK16	10.75	683.5	63.5813953	30.9350191	1175.06447	37.9849277
SUB1	59.875	2891.875	48.2985386	206.111857	5635.63029	27.3425817
SUPT20H	28.75	1860.625	64.7173913	122.877311	3881.5638	31.5889382
SUZ12	9.75	350.125	35.9102564	138.80585	3341.21438	24.0711351
T	0	0		0	0	
TAL1	0	0.125		0	0.1667129	
TBPL1	9.875	934.75	94.6582278	27.8156845	1546.52172	55.5989093
TBR1	28.125	1699.75	60.4355556	82.8959158	2911.7433	35.1252926
TBX1	0	4.25		0	5.90683052	
TBX10	0	0.375		0	0.72724593	
TBX15	0	0		0	0	
TBX18	0	0		0	0	
TBX19	0.75	53.75	71.6666667	2.17780871	86.9931784	39.9452798
TBX2	0	0		0	0	
TBX20	0	0		0	0	
TBX21	0	0		0	0	
TBX22	0	0		0	0	
TBX4	0	0		0	0	
TBX5	0	0		0	0	
TBX6	0.125	26.75	214	0.27984365	41.690514	148.977883
TCF12	45	2979.375	66.2083333	252.388103	8547.86707	33.8679478
TCF15	0	0		0	0	
TCF20	10.25	561	54.7317073	80.8696666	1486.69772	18.3838735
TCF21	0	0		0	0	
TCF3	11.75	743.875	63.3085106	32.9131426	1223.80479	37.1828604
TCF7	0.25	12.125	48.5	0.75395904	23.3459505	30.9644813
TCF7L1	0.75	65.125	86.8333333	2.1480254	116.854528	54.4009059
TCF7L2	2.625	209.375	79.7619048	7.27789595	360.609373	49.5485749
TCFL5	1.25	39.5	31.6	3.41309155	66.4747601	19.476407
TEAD1	11	812	73.8181818	31.1091546	1411.77844	45.3814467
TEAD2	4.125	189	45.8181818	10.9530026	308.408259	28.1574167
TEAD4	2.25	119.625	53.1666667	5.72533734	180.752939	31.5707055
TEF	2.375	276.75	116.526316	6.51950923	480.271707	73.6668498
TFAM	12.875	874.625	67.9320388	35.6973239	1449.6805	40.6103411
TFAP2A	0.25	19.125	76.5	0.55968729	32.4095727	57.9065724
TFAP2B	0	0.75		0	1.51481287	
TFAP2C	3.875	253.875	65.516129	11.7738649	485.427049	41.2292015
TFAP2D	0	0		0	0	
TFAP2E	0.25	20.375	81.5	0.68602539	30.3451747	44.2333114
TFAP4	0.125	9.125	73	0.35939162	15.1099377	42.0430994
TFCP2	2.75	298.75	108.636364	8.14182422	504.381286	61.9494197
TFCP2L1	0	0		0	0	
TFDP1	13	986.125	75.8557692	36.8272847	1637.95123	44.476568
TFDP2	12.125	861.75	71.0721649	33.2336665	1457.55336	43.8577357
TFDP3	0	0		0	0	
TFE3	3.75	265.75	70.8666667	10.2189564	473.364582	46.322204
TFEB	1	44.875	44.875	2.41543302	71.7139756	29.6899045

TFEC	0	0		0	0	
TGFB1I1	4.125	254	61.5757576	11.5945696	430.862797	37.1607409
TGIF1	15.875	1108.625	69.8346457	49.1555585	2098.00639	42.6809592
TGIF2LX	0	0		0	0	
THAP11	20.625	979	47.4666667	56.8585605	1641.98978	28.8784971
THRA	28.875	1843.875	63.8571429	75.5611989	2964.8373	39.2375629
THRB	3.5	335.125	95.75	8.17238402	524.159233	64.1378613
TLE4	16.125	852.875	52.8914729	166.894357	4532.61574	27.1585919
TLX1	0	0		0	0	
TLX2	0	0		0	0	
TOX2	2	186.75	93.375	5.62123693	321.86485	57.2587233
TOX3	10.75	897.75	83.5116279	27.4541201	1392.70822	50.7285687
TP53	9.375	588.25	62.7466667	27.0825091	1077.65284	39.7914695
TP63	0	0		0	0	
TP73	2.375	137.125	57.7368421	7.16852438	246.512244	34.3881433
TRPS1	3	285.25	95.0833333	8.94487	533.968456	59.6954966
TSHZ1	4.875	348.5	71.4871795	13.6720447	548.659026	40.1299905
TSHZ2	3	185.875	61.9583333	8.31966049	324.963749	39.0597368
TSHZ3	1.75	177	101.142857	4.79704678	294.477473	61.3872424
TULP1	0	1.25		0	2.24731221	
TULP2	0	0		0	0	
TULP4	24.75	866.75	35.020202	92.7007572	1389.38126	14.9878091
TWIST1	0	0.125		0	0.22473122	
TXK	0	0		0	0	
UBP1	2.5	128.25	51.3	7.1006125	219.59819	30.9266546
USF1	11.875	679.5	57.2210526	32.0987131	1125.19661	35.0542593
USF2	10.875	361.375	33.2298851	157.357858	4020.06952	25.547307
VAX1	1.625	129.875	79.9230769	3.82468785	181.054802	47.3384518
VAX2	2.625	161.125	61.3809524	6.47747805	249.987899	38.5933997
VDR	0	0		0	0	
VENTX	0	0		0	0	
VEZF1	45.625	3026.75	66.339726	263.26558	8923.95656	33.897164
VSX2	0.375	12.375	33	1.07817487	22.2483909	20.6352343
WT1	0	0		0	0	
XBP1	30.25	1908.375	63.0867769	88.3694759	3298.90185	37.3307844
YBX3	2.375	158.875	66.8947368	6.9744968	288.723683	41.3970629
YLPM1	11.25	963.125	85.6111111	32.9439094	1933.14897	58.6800111
YY1	8.5	557.125	65.5441176	22.1393468	905.189156	40.885992
YY2	0.625	41.875	67	1.94267416	75.7340966	38.9844566
ZBED1	20.5	1323.375	64.554878	57.8040549	2217.44979	38.361492
ZBTB1	5.75	426.625	74.1956522	16.8896349	721.419593	42.7137469
ZBTB10	2.875	226	78.6086957	8.3021754	382.865959	46.1163419
ZBTB14	7.5	507.25	67.6333333	20.750908	835.474691	40.2620786
ZBTB16	6.875	426.375	62.0181818	19.1634476	721.933568	37.6724264
ZBTB17	17.25	1048.875	60.8043478	46.2282873	1665.91868	36.0367813
ZBTB18	10.625	888.875	83.6588235	31.823593	1580.63994	49.6688084
ZBTB2	2.5	279	111.6	6.7421147	483.340983	71.6898191
ZBTB20	34.75	935.625	26.9244604	96.0061427	1645.55043	17.1400535
ZBTB21	5.875	626.625	106.659574	16.6185364	1070.81874	64.4352015
ZBTB33	14.75	1287.875	87.3135593	41.6351008	2177.42542	52.29783
ZBTB4	3.75	347.625	92.7	10.4223712	587.304241	56.3503476
ZBTB42	0.875	71.5	81.7142857	2.62630609	122.952599	46.8157916

ZBTB45	6.875	352.125	51.2181818	50.4903702	2001.42379	39.6397131
ZBTB5	16.625	1103.875	66.3984962	44.6931515	1792.16558	40.0993333
ZBTB7A	0	11		0	19.9541797	
ZBTB7B	0	2.5		0	4.96894822	
ZC3H8	2.75	166	60.3636364	7.46026681	255.036049	34.1859152
ZEB1	15	1148.375	76.5583333	43.430141	2077.18402	47.8281666
ZFAND3	4.875	399	81.8461538	12.6988605	659.531158	51.9362473
ZFAT	3.5	196.125	56.0357143	9.30543039	319.690639	34.355277
ZFP1	17.125	1151.125	67.2189781	47.7016991	1918.71787	40.2232604
ZFP37	6	450.75	75.125	16.7894461	766.955566	45.6808142
ZFP42	0	0		0	0	
ZFP69	2.5	152.75	61.1	7.02704219	269.966245	38.4181904
ZFP90	14	1137	81.2142857	38.7948544	1925.54868	49.6341257
ZFX	11.125	808.875	72.7078652	30.4790544	1340.87741	43.9934057
ZFY	0	0		0	0	
ZGPAT	20.375	824.5	40.4662577	224.45641	5492.14766	24.4686604
ZHX2	4.75	343.75	72.3684211	13.5630518	638.993291	47.1127959
ZHX3	11.5	821.25	71.4130435	31.5765683	1346.21175	42.6332508
ZIC1	2.875	180.375	62.7391304	9.20170709	342.250718	37.1942634
ZIC2	2.125	241.25	113.529412	6.643385	462.977387	69.689983
ZIC3	4.25	355.75	83.7058824	14.200223	695.395712	48.97076
ZIC4	1.5	83.25	55.5	4.57379165	159.637965	34.9027629
ZIC5	0.625	69.75	111.6	1.8673097	139.025885	74.4525054
ZKSCAN3	16.25	1047.125	64.4384615	42.7212512	1674.48263	39.1955429
ZKSCAN5	8.625	653.625	75.7826087	24.9222001	1045.83593	41.9640292
ZMYND8	16.375	984.5	60.1221374	69.0876584	2401.0267	34.7533375
ZNF10	7.625	615	80.6557377	20.9726573	1028.02943	49.0176051
ZNF117	2	109.625	54.8125	5.78638098	204.081023	35.2691991
ZNF124	4.875	340.375	69.8205128	13.4516621	558.74172	41.5370023
ZNF131	12	861.5	71.7916667	35.2743513	1436.06258	40.7112399
ZNF132	2.875	202.375	70.3913043	7.89959012	342.184938	43.3167966
ZNF133	10.5	706.875	67.3214286	29.7195954	1219.70109	41.0402993
ZNF134	22.25	1570.625	70.5898876	61.4019135	2578.81523	41.9989392
ZNF135	7.25	395.75	54.5862069	20.469614	692.014199	33.8069003
ZNF136	10.375	621.5	59.9036145	28.8781364	1027.12129	35.5674366
ZNF138	7.875	353.125	44.8412698	24.9986162	626.738899	25.0709437
ZNF140	15.25	937.375	61.4672131	42.544268	1594.17706	37.4710187
ZNF143	12.375	917.125	74.1111111	35.2656855	1539.74683	43.6613327
ZNF148	28.25	2228.125	78.8716814	80.7238959	3711.51945	45.9779525
ZNF154	1.875	119.75	63.8666667	5.26521308	205.958225	39.1167882
ZNF155	2	152.875	76.4375	5.64717364	279.148055	49.431463
ZNF157	1	124.375	124.375	2.55067328	193.569226	75.8894633
ZNF165	1.25	59.75	47.8	3.59243751	99.8142507	27.7845475
ZNF174	8.875	642.125	72.3521127	25.1172356	1122.73835	44.699917
ZNF175	0.625	39.75	63.6	1.96113411	82.6185166	42.1279279
ZNF177	0	0		0	0	
ZNF18	8.625	570.375	66.1304348	23.8573717	989.470584	41.4744171
ZNF189	5.875	413.875	70.4468085	15.9576763	669.067464	41.9276246
ZNF19	4	308.5	77.125	11.8002808	543.31328	46.0424028
ZNF202	2.875	280	97.3913043	8.46240579	480.90612	56.8285345
ZNF205	4.25	281.625	66.2647059	11.3713521	458.353829	40.3077687
ZNF214	1.625	191.625	117.923077	4.2961471	331.664711	77.2005017

ZNF215	0.125	16.5	132	0.34301269	29.430494	85.800014
ZNF217	3.5	344.75	98.5	10.8774113	615.836899	56.6161272
ZNF219	5.125	247.75	48.3414634	14.0452735	411.459594	29.2952354
ZNF22	30.625	583.5	19.0530612	82.343713	969.723774	11.7765369
ZNF224	0.75	43.375	57.8333333	2.25735669	74.306602	32.917528
ZNF23	12.875	859.75	66.776699	35.4297451	1436.91373	40.5567053
ZNF230	5.375	408.625	76.0232558	14.7575908	684.188832	46.3618245
ZNF239	8.25	630.5	76.4242424	23.7576108	1060.18649	44.6251306
ZNF24	33.75	2528.375	74.9148148	94.5056975	4200.93595	44.4516687
ZNF250	17.125	1217.625	71.1021898	46.8127893	2080.50784	44.4431505
ZNF251	4.625	258.75	55.9459459	12.8770991	454.387903	35.2865114
ZNF252P	5.375	389.625	72.4883721	14.6073224	670.44268	45.8977121
ZNF256	4.125	304	73.6969697	11.0762139	468.757988	42.3211391
ZNF263	11	747.375	67.9431818	32.3743213	1346.82002	41.6014906
ZNF267	2.25	146.875	65.2777778	5.97888474	248.80972	41.6147376
ZNF268	12.25	1084.5	88.5306122	35.3053177	1836.15308	52.0078335
ZNF274	18.875	1250.625	66.2582781	53.0972274	2096.18998	39.4783322
ZNF281	22.125	2205.375	99.6779661	57.7586567	3478.13175	60.218363
ZNF284	1.875	92.875	49.5333333	5.45026396	168.253261	30.8706628
ZNF300	13.625	935.5	68.6605505	38.469477	1550.30487	40.2996086
ZNF302	23	1203.125	52.3097826	89.8163336	1999.63782	22.2636323
ZNF304	11.875	806.5	67.9157895	32.1361822	1311.94085	40.8244153
ZNF317	14.25	1124.625	78.9210526	36.7682657	1793.12049	48.7681553
ZNF322	4.625	361.75	78.2162162	13.224199	605.289322	45.771341
ZNF326	12.125	735.5	60.6597938	32.5987154	1215.22985	37.2784582
ZNF333	7.125	406.25	57.0175439	21.8420394	721.956247	33.0535182
ZNF34	8	599.375	74.921875	22.2178899	992.67573	44.6791182
ZNF35	7.25	489.25	67.4827586	27.9953106	898.048184	32.0785219
ZNF350	7.375	424.5	57.559322	21.5526763	714.057379	33.1307987
ZNF354A	4.5	343.125	76.25	12.0246473	595.360593	49.5116885
ZNF354C	0.875	114.375	130.714286	2.77320663	197.679009	71.2817455
ZNF367	1	77.75	77.75	2.80184954	135.629277	48.4070523
ZNF37A	7.375	443	60.0677966	19.6691433	710.969589	36.1464441
ZNF382	4	321.625	80.40625	11.9580299	559.008495	46.7475411
ZNF384	10.75	810.875	75.4302326	28.4702807	1318.323	46.3052335
ZNF395	2.875	275.125	95.6956522	7.74250391	467.917255	60.4348749
ZNF41	3.125	199.375	63.8	8.89607368	338.235508	38.0207628
ZNF423	5.125	318.5	62.1463415	15.2896618	592.16369	38.7296788
ZNF429	9.125	251.75	27.5890411	60.2696396	1026.83522	17.0373546
ZNF43	7.625	283.125	37.1311475	22.0194104	488.637242	22.1912047
ZNF431	3	63.5	21.1666667	8.3161549	108.542176	13.0519666
ZNF433	7.875	496.125	63	22.4556145	832.847844	37.0886241
ZNF45	5.625	443.75	78.8888889	15.7553483	762.578332	48.401236
ZNF467	2.125	88	41.4117647	6.14846028	161.178022	26.2143716
ZNF468	7.75	310	40	21.0575504	505.405472	24.0011522
ZNF470	2.75	232.125	84.4090909	7.59649961	404.05036	53.1890187
ZNF492	0.125	0	0	0.34301269	0	0
ZNF496	8.625	599.125	69.4637681	23.6710841	1001.42572	42.3058665
ZNF512B	2.625	194.625	74.1428571	6.76627987	307.075302	45.3831806
ZNF513	2.375	138.875	58.4736842	6.37147425	221.472388	34.7599911
ZNF517	1	36.375	36.375	3.3348819	68.9116182	20.6638856
ZNF536	19.25	1265.75	65.7532468	50.2937467	2052.01477	40.8005945

ZNF580	8.25	532	64.4848485	22.9677391	929.380403	40.4646011
ZNF589	4	352	88	11.287577	619.077822	54.8459444
ZNF592	5.375	382.125	71.0930233	14.9839647	695.289941	46.4022676
ZNF639	3.875	270.375	69.7741935	11.4301787	473.024968	41.3838647
ZNF652	20.75	820.375	39.5361446	58.0705363	1302.28539	22.4259232
ZNF668	9.875	605.75	61.3417722	27.8564748	1015.98765	36.4722263
ZNF674	0.5	71.25	142.5	1.35922748	123.782314	91.0681369
ZNF687	13	1004.375	77.2596154	38.3809363	1815.33914	47.2979379
ZNF69	0.5	23.625	47.25	1.45217984	40.0419403	27.5736787
ZNF692	5.125	388.125	75.7317073	13.9696649	681.87603	48.8111945
ZNF7	11.375	592.625	52.0989011	35.200362	1109.48997	31.5192774
ZNF705D	0	0		0	0	
ZNF711	9.75	770.125	78.9871795	26.8088086	1395.76359	52.0636188
ZNF720	1.875	95.5	50.9333333	5.75293077	159.674489	27.7553295
ZNF746	3	243.125	81.0416667	8.10866663	384.996077	47.4795789
ZNF750	0	0		0	0	
ZNF76	6.75	509.375	75.462963	18.9500959	877.575061	46.3097953
ZNF8	5.375	461	85.7674419	14.9359657	803.002441	53.763008
ZNF80	0	0		0	0	
ZNF804A	3.125	235.125	75.24	8.15119989	396.798963	48.6798224
ZNF821	26.5	1840.875	69.4669811	71.1765331	2994.63653	42.0733689
ZNF84	24.125	1754.125	72.7098446	67.1425658	2913.67928	43.3954115
ZNF85	6.25	178.25	28.52	16.2365359	285.080919	17.557989
ZNF92	14.625	361.625	24.7264957	190.84619	3193.69111	16.7343719
ZNF93	10.75	489.625	45.5465116	30.270347	808.809478	26.7195311
ZNF98	0	0		0	0	
ZNRD1	11.75	801.5	68.212766	79.3361465	3041.32185	38.3346304
ZSCAN10	0	1.25		0	2.48447411	
ZSCAN12	4.875	309.375	63.4615385	13.5923971	486.32305	35.7790495
ZSCAN21	5.5	417.125	75.8409091	15.0230875	685.050189	45.5998268
ZSCAN26	19.625	1304.75	66.4840764	52.2931432	2186.85925	41.8192351
ZSCAN31	3.125	265.25	84.88	10.6234573	441.379384	41.5476215
ZSCAN4	0	0		0	0	
ZSCAN9	8.875	734.5	82.7605634	23.542262	1224.59294	52.0167917

TF Bulk

Gene	RAW_avePre	RAW_avePost	RAW_ratio	CPM_avePre	CPM_avePost	CPM_ratio
AEBP1	1441.08621	12067.8276	8.37411914	85.2500337	2594.06775	30.4289352
AEBP2	375	2837.46552	7.56657471	22.9427269	631.169435	27.5106546
AHR	137.206897	1173.51724	8.55290274	8.87385731	273.041562	30.769208
AIRE	0.5862069	2.98275862	5.08823529	0.03496384	0.58895431	16.8446669
AKNA	767.948276	7356.2069	9.57903954	45.9426722	1574.24876	34.2655027
ALX1	9.55172414	111.62069	11.6859206	0.6086044	25.4627984	41.8380125
ALX3	100.741379	799.362069	7.93479377	5.95722619	170.445195	28.6115029
ALX4	6.81034483	78.2413793	11.4886076	0.40160861	16.2946334	40.573417
AR	116.137931	1343.7069	11.5699228	7.53986432	317.832081	42.1535544
ARHGAP35	4557.36207	37745.0517	8.28221483	291.844099	8781.49934	30.089693
ARID3A	192.431034	1331.10345	6.91730132	11.1584787	275.998024	24.7343774
ARID3B	521.586207	4346.96552	8.33412667	30.3452298	902.808427	29.751247
ARID3C	45.0517241	290.793103	6.45464983	2.78431684	64.3299018	23.1043755
ARID5A	243.568966	2071.81034	8.50605224	14.5312607	450.082862	30.9734214
ARID5B	368.034483	4116.10345	11.1840157	23.2195495	946.516876	40.7637916
ARNT	786.517241	6566.13793	8.34837126	47.4356733	1436.55081	30.2841872
ARNTL	218.413793	1945.12069	8.90566782	14.0407273	454.007354	32.3350312
ARNTL2	158.12069	1407.51724	8.90153746	10.4861275	340.715556	32.4920288
ARX	3074.36207	13161.0172	4.28089371	190.415254	2995.22243	15.72995
ASCL2	16.4137931	95.3275862	5.80777311	1.01588405	20.9497399	20.6221761
ASCL3	0.31034483	2.68965517	8.66666667	0.02147348	0.57158462	26.6181584
ATF1	165.034483	1377.41379	8.34621814	10.7188069	327.307175	30.5357842
ATF2	1412	10508.7586	7.44246361	90.9142007	2475.62676	27.2303638
ATF3	325.465517	2771.12069	8.51432961	20.8553833	665.993167	31.9338733
ATF5	837.396552	6784.41379	8.10179332	52.6516977	1554.74087	29.5287889
ATF6	792.672414	7574.75862	9.55597607	51.4395333	1791.80384	34.8332056
ATF6B	1811.55172	12547.9483	6.92662987	111.194378	2802.59266	25.2044458
ATF7	408.551724	4423.2069	10.826553	24.973078	981.998127	39.3222705
ATOH1	1.56896552	15.1896552	9.68131868	0.08405521	2.92323483	34.7775556
ATOH7	30.2241379	319.189655	10.560753	1.87361393	71.2734922	38.0406502
BACH1	587.362069	5742.5	9.77676344	37.406154	1326.45578	35.4608971
BACH2	1814.72414	17557.6379	9.67510023	117.537148	4152.36576	35.328114
BARHL1	28.2241379	311.62069	11.0409285	1.58170646	58.3857027	36.9131087
BARHL2	78.6896552	762.637931	9.69171779	4.9143733	169.323503	34.4547499
BARX1	9.43103448	80.1034483	8.49360146	0.60475433	19.5690279	32.3586404
BARX2	0.53448276	6.03448276	11.2903226	0.03307397	1.24034818	37.5022463
BATF	0	0.06896552		0	0.01157973	
BATF3	20.0172414	187.844828	9.38415159	1.274878	43.6503171	34.238819
BBX	944.896552	8990.48276	9.51477994	59.5342769	2057.32857	34.557043
BCL11B	2003.72414	14362.3621	7.16783403	128.204609	3367.71713	26.2683
BCL6	413.810345	3933.15517	9.50472897	25.2261568	877.93452	34.8025476
BCL6B	3.32758621	42.5689655	12.7927461	0.194942	9.0671353	46.5119636
BHLHA15	16.7068966	130.965517	7.83900929	1.11729025	30.6600313	27.4414202
BHLHA9	0.01724138	0.27586207	16	0.00118619	0.05627469	47.4413857
BHLHE22	1063.03448	4909.24138	4.61813935	61.5600323	1027.63981	16.6932955
BHLHE23	14.6896552	60.4827586	4.11737089	0.92882634	13.8865535	14.9506457
BHLHE40	751.241379	6948.41379	9.24924263	47.355306	1657.21303	34.9952977
BHLHE41	128.293103	917.241379	7.14957667	8.32473005	224.907397	27.0167796
BMPR1A	555.431034	4619.84483	8.31758498	35.1982462	1068.8294	30.3659844
BNC1	0.86206897	8.87931034	10.3	0.05444644	2.03900336	37.4497113

BNC2	18.637931	217.913793	11.6919519	1.32897268	56.8623518	42.7866972
BRIP1	137.637931	1405.55172	10.2119504	9.13522215	336.574321	36.8435836
BSX	0.12068966	1.39655172	11.5714286	0.01156568	0.41457342	35.8451548
BTBD11	281.258621	2550.25862	9.06730828	17.9579323	602.116724	33.5292901
BTBD3	1000.98276	9473.31034	9.46400951	64.708263	2232.50927	34.5011466
BTG2	912.534483	7994.25862	8.76050031	55.6273801	1786.46439	32.1148397
CAMTA1	1561.94828	13176.2241	8.43576214	99.8187591	3055.99231	30.6154108
CARF	240.724138	2177.65517	9.04626844	15.089471	492.38502	32.6310325
CASZ1	238.517241	2193.84483	9.19784589	13.5689509	445.958152	32.8660747
CC2D1A	1794.34483	10781.8448	6.00879199	104.707092	2260.7411	21.5910982
CC2D1B	660.948276	4671.86207	7.06842311	39.6981393	1008.22819	25.3973663
CDC5L	980.655172	7974.63793	8.13194908	64.6311318	1920.55272	29.7155978
CDX1	0.27586207	4.36206897	15.8125	0.01854383	0.90620333	48.8681956
CDX2	0.05172414	1.06896552	20.6666667	0.00281728	0.21708039	77.0532675
CDX4	0	0.10344828		0	0.01958246	
CEBPA	117.206897	1130.51724	9.64548397	7.30339071	256.561167	35.1290486
CEBPB	163.224138	1035.51724	6.34414281	10.2840861	241.741988	23.5064143
CEBPD	108.775862	662.275862	6.08844508	7.211678	168.313948	23.3390825
CEBPE	0.12068966	1.74137931	14.4285714	0.00880897	0.42275986	47.9920005
CEBPG	1192.34483	10340.2069	8.67216149	75.71548	2410.68104	31.8386813
CEBPZ	642.258621	5344.44828	8.32133366	41.3034333	1252.27929	30.3190119
CGGBP1	2003.5	15517.6034	7.74524754	131.938862	3766.56896	28.5478358
CHCHD3	612.551724	4414.43103	7.20662576	39.8504548	1058.48932	26.5615368
CIC	5516.12069	32664.4828	5.92164033	323.888021	6907.53093	21.3269108
CLOCK	386.586207	4087.15517	10.5724289	25.0496919	959.858578	38.318179
CNOT3	1494.91379	10254.3103	6.85946601	88.3280595	2179.19178	24.6715686
CREB1	961.5	8057.37931	8.38000968	63.0739426	1935.83805	30.6915657
CREB3L1	114.775862	1234.08621	10.7521406	6.89242166	268.992645	39.0273054
CREB3L2	396.862069	4017	10.1219046	24.4955169	915.811105	37.3868864
CREB3L3	0.22413793	2.4137931	10.7692308	0.01237999	0.47503904	38.3715049
CREB3L4	206.5	1617.7069	7.8339317	12.3476117	345.692994	27.9967497
CREB5	862.206897	8260.81034	9.58100704	51.5202838	1780.52401	34.5596701
CREBBP	2853	22291.7241	7.81343293	176.175606	4969.19763	28.2059347
CREBL2	550.741379	5103.01724	9.26572332	36.2368767	1240.74489	34.2398408
CREBRF	314.103448	3318.56897	10.5652102	19.3329313	746.01022	38.5875378
CREM	272.793103	2348.84483	8.61035267	17.5634558	556.155325	31.6654837
CRX	7.39655172	75.2758621	10.1771562	0.48026196	20.3056399	42.2803422
CSRNP1	273.017241	2282.94828	8.3619198	17.4846649	552.775389	31.6148689
CSRNP2	2129.15517	18371.2759	8.62843446	140.288088	4437.20793	31.6292566
CSRNP3	1636.62069	4797.63793	2.93142935	110.573404	1151.6276	10.4150506
CTBP2	2314.24138	5968.39655	2.57898619	145.450362	1374.69419	9.45129438
CTCF	1650.56897	12663.569	7.67224468	105.154435	2940.31396	27.9618634
CTCFL	0.4137931	5.22413793	12.625	0.02484391	1.15047083	46.3079553
CTNNBL1	779.603448	5335.25862	6.84355442	49.1699778	1224.64124	24.90628
CUX1	2549.27586	19185.1379	7.52572062	156.923159	4294.23124	27.3651848
CUX2	704.655172	6292.06897	8.92928799	42.3151076	1362.18756	32.1915181
DACH1	927.62069	7256.10345	7.82227426	58.3609071	1660.95279	28.4600235
DACH2	244.741379	1794.10345	7.33060937	15.6280756	416.087555	26.6243628
DBP	533.12069	3182.15517	5.9689208	31.713692	696.773486	21.9707465
DBX1	74.9310345	614.844828	8.2054763	4.34787124	130.038448	29.9085324
DBX2	1.22413793	11.9137931	9.73239437	0.08448106	3.08632474	36.5327426
DDIT3	496.034483	1566.36207	3.15776851	31.8737549	375.486083	11.7804157

DDN	687.87931	6781.46552	9.85851066	45.2342842	1610.59519	35.60563
DEAF1	3055.86207	14808.2241	4.8458418	188.679706	3335.695	17.6791404
DENND4A	327.62069	3111.87931	9.49842122	21.0733365	720.239618	34.1777686
DIDO1	2231.74138	21601.6897	9.6792979	135.796528	4741.19333	34.9139509
DLX2	2059.24138	12254.5345	5.95099467	128.845779	2821.28003	21.8965653
DLX3	2.89655172	34.1551724	11.7916667	0.16906181	6.86719396	40.6194281
DLX6	904.931034	5973.65517	6.60122699	56.356522	1363.53945	24.1948829
DMBX1	45.4655172	416.844828	9.16837315	2.41486819	77.7684521	32.2040153
DMRT1	5.87931034	62.4655172	10.6246334	0.36037239	14.3754735	39.8906071
DMRT2	8.51724138	66.3793103	7.79352227	0.50399105	14.2179548	28.2107285
DMTF1	1435.7069	11488	8.00163322	86.9921413	2512.37232	28.8804515
DPF1	2265.93103	11774.5862	5.19635683	143.685194	2721.22304	18.9387854
DPF3	84.8965517	817.931034	9.63444354	5.32120533	188.053971	35.3404839
DRGX	0.20689655	2.96551724	14.3333333	0.01196268	0.69239553	57.8796168
DUX4	0.05172414	0	0	0.00304411	0	0
E2F1	654.051724	4722.7931	7.2208251	41.019522	1085.88713	26.4724472
E2F2	262.37931	2532.13793	9.65067683	16.3041586	572.250276	35.0984244
E2F3	450.206897	4409.53448	9.79446232	28.7922019	1027.58992	35.6898693
E2F4	1144.77586	8848.81034	7.72973176	68.5732705	1925.49121	28.0793259
E2F5	304.551724	1812.31034	5.95074728	19.388235	420.890632	21.7085584
E2F6	349.396552	2411.81034	6.90278806	22.4307037	566.50739	25.2558903
E2F7	216.982759	2064.48276	9.51450139	13.5342657	467.555711	34.5460717
E2F8	83.7413793	957.362069	11.4323657	5.09294035	212.012273	41.6286582
E4F1	1379.25862	6911.32759	5.01090041	81.3444568	1470.26936	18.0746104
EAF2	19.5344828	181.396552	9.28596646	1.23017504	42.7700422	34.7674443
EBF1	505.793103	4236.44828	8.3758522	31.7580544	985.197613	31.0219763
EBF2	60.3275862	659.344828	10.9294084	3.65607928	142.503218	38.977059
EBF3	254.672414	2323.81034	9.12470381	16.1272665	522.656441	32.4082472
EBF4	1528.77586	9608.17241	6.28487972	87.9197398	1996.9448	22.7132701
EED	307.672414	2587.2931	8.40924629	19.3679365	592.185319	30.5755505
EGR1	1215.17241	9132.94828	7.51576334	62.0469542	1677.8169	27.041084
EGR2	105.344828	1038.44828	9.85761047	5.10733295	184.481131	36.1208351
EGR3	543.62069	4089.13793	7.5220425	28.4315538	790.501443	27.8036666
EGR4	187.086207	1469.48276	7.85457562	8.93855921	260.130631	29.1020762
EHF	2.67241379	17.637931	6.6	0.17478201	4.31447079	24.684868
ELF1	117	1187.37931	10.1485411	7.57995585	279.980692	36.9369819
ELF2	723.155172	4148	5.73597501	45.5271006	943.328454	20.7201522
ELF3	10.0517241	109.913793	10.9348199	0.60945561	25.3270039	41.5567656
ELF4	149.103448	1625.2069	10.8998612	8.74688843	350.692331	40.093381
ELF5	0.03448276	0.18965517	5.5	0.00217689	0.04353511	19.998755
ELK1	918.155172	7614.7931	8.2935797	58.9659902	1793.60993	30.4177022
ELK3	50.5862069	588.051724	11.6247444	3.20084668	135.87607	42.4500402
ELK4	447.431034	4411.13793	9.85881084	26.3540671	939.176491	35.6368711
EMX1	698.810345	3390.67241	4.85206385	38.9489434	673.97256	17.3040011
EMX2	1259.25862	7062.91379	5.60878733	72.192904	1472.14121	20.3917716
EN1	32.9310345	322.827586	9.80314136	1.64289498	57.1190176	34.7672969
EN2	58.7413793	495.724138	8.43909598	2.99481608	84.451061	28.199081
EOMES	416.586207	3969.32759	9.52822614	27.0751485	927.441034	34.2543286
EP300	2058.31034	18158.9483	8.82225964	128.227859	4061.03297	31.6704419
EPAS1	188.655172	1782.18966	9.44681046	12.0987509	442.54536	36.5777727
ERF	1382.13793	8870.25862	6.41778105	81.2082852	1909.61646	23.5150447
ERG	0.27586207	2.94827586	10.6875	0.01900741	0.80598588	42.4037661

ESR1	1.53448276	12.4310345	8.1011236	0.09411638	2.75981744	29.3234552
ESR2	30.5689655	212.241379	6.9430344	1.79705452	44.7937652	24.9262138
ESRRA	470.431034	2761.36207	5.86985523	29.7493853	631.762394	21.2361495
ESRRG	410.758621	4013.31034	9.77048355	26.9921043	952.991353	35.3063008
ESX1	0.15517241	2.79310345	18	0.0085833	0.57065195	66.4839646
ETS1	28.2758621	332.948276	11.775	1.93776048	83.2201125	42.9465424
ETS2	314.051724	2834.43103	9.02536371	19.5266987	646.686837	33.1180834
ETV1	710.103448	6307.05172	8.88187734	51.1543744	1692.93001	33.0945305
ETV2	49.7068966	462.258621	9.29968783	3.05850286	102.305479	33.4495286
ETV3	161.913793	1700.36207	10.5016505	9.66264611	365.462542	37.8222008
ETV4	134.051724	1274.01724	9.50392283	8.41114829	296.934151	35.3024511
ETV5	529.637931	4673.17241	8.82333409	34.0700648	1112.18546	32.6440665
ETV6	174.5	1935.93103	11.0941607	10.8488439	432.556891	39.871243
ETV7	2.4137931	25.3103448	10.4857143	0.17058461	6.57738857	38.5579239
FAM170A	0.25862069	1.96551724	7.6	0.01529014	0.42338002	27.6897351
FERD3L	0.20689655	2.63793103	12.75	0.01550246	0.60617162	39.1016298
FEV	1.34482759	11.8448276	8.80769231	0.08537856	2.66322222	31.1931041
FEZF1	370.793103	3234.60345	8.72347252	22.9180978	734.644121	32.0551962
FEZF2	1776.94828	8453.63793	4.75739111	102.396762	1756.58982	17.1547399
FIGLA	0.12068966	0.72413793	6	0.00697843	0.1597655	22.8941912
FLI1	2.77586207	35.2241379	12.689441	0.16342978	7.08439291	43.3482366
FOS	1587.68966	7631.41379	4.80661556	81.2424372	1447.24819	17.8139436
FOSL1	24.7413793	250.12069	10.1094077	1.58315722	62.0252341	39.1781898
FOSL2	974.293103	7896.03448	8.10437275	59.619953	1838.88155	30.8433915
FOXA1	22.8275862	226.155172	9.9070997	1.34985748	47.3544408	35.0810672
FOXA2	0.36206897	4.5862069	12.6666667	0.02051452	1.01433753	49.4448609
FOXA3	1.12068966	14.4827586	12.9230769	0.06890573	3.34603365	48.5595815
FOXB1	37.6034483	273.741379	7.27968822	2.21926161	56.0927367	25.2754053
FOXB2	0.20689655	1.17241379	5.66666667	0.01111066	0.26963454	24.2680953
FOXC1	25.362069	222.62069	8.77770224	1.59982378	51.6391164	32.2780027
FOXC2	3.43103448	32.4310345	9.45226131	0.21349436	7.24299013	33.9259089
FOXD1	53.5689655	462.362069	8.63115546	2.9161302	88.8136648	30.4560012
FOXD2	6.34482759	50.637931	7.98097826	0.37401589	10.5805228	28.2889663
FOXD3	0.87931034	9.31034483	10.5882353	0.05360271	2.28063013	42.5469157
FOXD4	23.137931	466.37931	20.1564829	1.5167054	109.66305	72.303461
FOXD4L1	13.5517241	289.586207	21.3689567	0.82879403	65.0119228	78.4415916
FOXD4L3	1.48275862	26.2413793	17.6976744	0.09424615	5.80872713	61.6335719
FOXD4L5	1.22413793	25.9310345	21.1830986	0.07579079	5.75244769	75.8990303
FOXD4L6	2	54.9137931	27.4568966	0.12525428	12.4638817	99.508632
FOXE1	8.43103448	70.2068966	8.32719836	0.48793339	14.6120286	29.9467689
FOXE3	4.13793103	39.5344828	9.55416667	0.24371735	8.443521	34.6447265
FOXF1	2.17241379	18.8103448	8.65873016	0.14019905	4.36377402	31.1255615
FOXF2	6.51724138	55.137931	8.46031746	0.37730264	11.5854169	30.7058992
FOXG1	6943.01724	25156.5862	3.62329306	415.300402	5524.82556	13.303203
FOXH1	3.5	30.862069	8.81773399	0.1990318	6.33136809	31.8108371
FOXI1	0.03448276	0.86206897	25	0.00175921	0.17436964	99.1183594
FOXI2	0.5862069	4.62068966	7.88235294	0.0362314	0.95177332	26.2692977
FOXJ1	329.758621	3160.55172	9.58444003	21.8875375	772.452552	35.2918893
FOXJ2	975.948276	9857.37931	10.1003092	61.0436145	2226.46854	36.473406
FOXJ3	1563.34483	12287.1207	7.85950769	99.2258269	2845.03134	28.6722865
FOXK1	1557.13793	15528.9828	9.97277277	93.9898769	3384.40498	36.0081861
FOXK2	2944.74138	19656.4138	6.67509002	183.92833	4471.96215	24.3136125

FOXL1	2.70689655	28.9655172	10.7006369	0.1848226	7.37926317	39.9261951
FOXL2	0.5	5.86206897	11.7241379	0.03309039	1.50708324	45.544432
FOXM1	749.034483	7052.91379	9.41600681	46.1308129	1582.93293	34.3140047
FOXN1	0.10344828	1.46551724	14.1666667	0.00596904	0.3539138	59.2915474
FOXN2	239.310345	2303.13793	9.6240634	14.906961	521.444841	34.979956
FOXN3	1942.37931	16602.0345	8.54726695	124.13727	3867.46931	31.1547798
FOXN4	234.758621	2130.32759	9.07454465	13.0647318	424.011199	32.4546424
FOXO1	262.431034	2632.5	10.0312069	17.2066002	638.562407	37.1114804
FOXO3	1489.62069	11724.4138	7.87073775	94.2572684	2701.5364	28.6613059
FOXO4	248.758621	2693.2069	10.8265872	14.569151	568.743368	39.0375092
FOXO6	1098.08621	6587.91379	5.99945045	66.9895513	1452.43466	21.6815105
FOXP1	735.396552	6264.34483	8.51832228	48.9084917	1528.90453	31.2605127
FOXP2	501.172414	4771.89655	9.52146691	36.6672667	1271.53164	34.6775682
FOXP3	17.7413793	155.051724	8.73955296	1.04958815	33.0115998	31.4519557
FOXP4	2616.22414	18675.7241	7.13842666	161.788633	4199.98644	25.9597128
FOXQ1	6	55.5344828	9.25574713	0.35911567	12.8183541	35.6942214
FOXR1	2.32758621	22.862069	9.82222222	0.14385585	4.94586538	34.3807038
FOXR2	0.01724138	0.20689655	12	0.00117868	0.04086155	34.6671739
FOXS1	12.1551724	140.965517	11.5971631	0.77351931	33.9031167	43.8296968
FUBP1	2775.7069	12543.1034	4.51888615	170.773442	2799.62865	16.3938176
FUBP3	1187.91379	7675.86207	6.46163224	73.4572425	1732.25343	23.5817922
GABPA	457.413793	4015.2069	8.77806257	28.9364454	924.631078	31.9538583
GATA1	2.03448276	16.3103448	8.01694915	0.1212567	3.39200282	27.9737362
GATA2	8.75862069	92.6034483	10.5728346	0.60632182	24.437628	40.3047145
GATA3	20.7241379	241.034483	11.6306156	1.65569579	71.9023312	43.4272598
GATA4	0.46551724	4.48275862	9.62962963	0.02828202	0.97238021	34.3815673
GATA5	1.86206897	19.1206897	10.2685185	0.11898123	4.44550022	37.3630389
GATA6	3.4137931	25.5862069	7.49494949	0.22721358	6.45240396	28.3979676
GBX1	18.2241379	136.810345	7.50709555	1.16772337	31.9376137	27.3503251
GBX2	110.603448	1065.24138	9.63117693	7.54035229	260.650555	34.5674241
GCFC2	218.603448	1606.91379	7.35081631	14.1573579	381.683774	26.9600992
GCM1	1.22413793	12.2586207	10.0140845	0.07420258	2.75251205	37.094561
GFI1	2.43103448	27.0172414	11.1134752	0.14597919	5.74203248	39.334597
GFI1B	0.27586207	1.74137931	6.3125	0.0163121	0.41432433	25.399823
GLI1	97.8793103	984.758621	10.0609477	5.56320717	203.66591	36.609442
GLI2	853.086207	7124.87931	8.35188666	50.0522752	1493.888	29.8465553
GLI3	1497.01724	13436.2241	8.97533025	92.0490547	2971.74342	32.2843448
GLIS1	24.0862069	210.87931	8.75518969	1.42809914	45.9831026	32.1988167
GLIS2	1134.55172	10162.9138	8.95764695	67.4739213	2191.98097	32.4863432
GLIS3	430.327586	4379	10.1759686	27.6712109	1034.79326	37.3960236
GLMP	161.017241	1179.89655	7.32776529	10.8818447	298.317718	27.4142599
GMEB1	280.5	2495.67241	8.89722786	17.6567562	565.340496	32.0183667
GMEB2	810.931034	7453.68966	9.19152103	49.0041353	1632.16754	33.3067307
GPER1	23.0172414	254.431034	11.0539326	1.45974387	58.3023676	39.9401353
GRHL1	105.637931	1041.25862	9.85686307	6.33695829	220.871536	34.8545036
GRHL2	0.53448276	6.53448276	12.2258065	0.03493736	1.60008368	45.7986367
GRHL3	12.0517241	115.5	9.58369099	0.78996281	27.3412049	34.6107494
GSC	2.65517241	22.0517241	8.30519481	0.16216352	4.88476081	30.1224392
GSC2	5.67241379	47.9827586	8.45896657	0.37170192	11.1812302	30.0811739
GSX1	39.5172414	222.241379	5.62390925	2.01696284	40.7387299	20.1980567
GSX2	289.568966	2384.75862	8.23554629	18.6598906	569.817378	30.5370159
GZF1	462.982759	4329.46552	9.35124567	29.128853	997.810939	34.2550714

HAND1	1.05172414	9.05172414	8.60655738	0.061737	2.09959823	34.0087494
HAND2	1.44827586	17.0862069	11.797619	0.07477702	3.13330113	41.9019236
HBP1	562.603448	4174.36207	7.41972358	35.4184018	956.416878	27.0033889
HCFC1	3837.36207	28405.1207	7.40225191	227.883689	6064.11852	26.6105861
HDAC5	4020.2069	19518.2931	4.85504692	245.526446	4320.88781	17.5984619
HDGF	4703.84483	22215.5517	4.72284961	297.408683	5195.44229	17.4690337
HELT	19.7241379	151.396552	7.6756993	1.34184853	38.2508083	28.5060552
HES1	817	5902.67241	7.22481324	48.4962812	1286.86691	26.5353731
HES2	7.84482759	69.0862069	8.80659341	0.50356953	16.1216377	32.0147207
HES3	2.05172414	12.8965517	6.28571429	0.12823565	2.88874324	22.5268347
HES4	3254.63793	5512.41379	1.6937103	190.836231	1166.42678	6.11218728
HES5	946.586207	4115.32759	4.34754654	54.0227177	861.528469	15.9475218
HES6	4274.67241	12638.5517	2.95661293	263.780316	2876.42585	10.9046266
HES7	193.637931	1089.08621	5.62434334	11.3218192	228.63742	20.1944065
HESX1	16.6551724	152.396552	9.15010352	1.029554	34.9065109	33.9044972
HEY1	736.275862	5838.37931	7.92960847	44.4793895	1276.72032	28.7036386
HEY2	123.775862	1109.2931	8.96211171	6.68717614	211.675096	31.6538837
HEYL	15.0344828	144.982759	9.64334862	0.88769035	31.0003766	34.9225116
HHEX	0.55172414	4.62068966	8.375	0.03439296	1.13010867	32.8587196
HIC2	839.465517	7688.98276	9.15937892	49.8851208	1644.88457	32.9734506
HIF1A	1533.77586	11756.1897	7.66486809	100.619635	2822.11042	28.0473132
HIF3A	1064.77586	7482.74138	7.02752724	59.4408967	1497.73278	25.1970085
HINFP	574.931034	4993.36207	8.68514964	35.8939474	1128.22484	31.432175
HIVEP1	342.62069	3932.89655	11.4788647	21.4831107	878.64954	40.899549
HIVEP2	941	10243.5	10.8857598	61.5766676	2426.56432	39.4072042
HIVEP3	349.586207	4033.91379	11.5391103	21.8147088	906.121099	41.5371624
HLF	70.4310345	792.586207	11.253366	4.19483485	171.146271	40.799287
HLTF	943.051724	8248.84483	8.74696967	60.5965907	1919.17498	31.6713359
HLX	4.34482759	43.1551724	9.93253968	0.25616807	8.86363065	34.6008411
HMBOX1	127.775862	1279.44828	10.0132236	7.8750497	284.004597	36.0638482
HMG20A	1263.17241	10278.3448	8.13692946	81.8763721	2436.16618	29.7542027
HMGA1	5250.77586	13403.1897	2.55261127	331.540889	3117.75371	9.40382864
HMGA2	948.189655	7309.10345	7.70848259	54.3297928	1524.40923	28.05844
HMX1	158.189655	949.775862	6.0040327	8.98693617	193.678466	21.5511118
HNF1A	42.637931	15.362069	0.36029114	2.70553628	3.15853132	1.16743262
HNF1B	5.32758621	53.3103448	10.0064725	0.3157019	11.4192044	36.170845
HNF4A	0.22413793	2.60344828	11.6153846	0.01464461	0.63078016	43.0725175
HNF4G	10.1896552	106.396552	10.4416244	0.689825	25.086634	36.3666639
HNRNPAB	3562.81034	13466.569	3.77976026	227.158312	3147.73046	13.85699
HOMEZ	148.258621	1638.2069	11.0496569	9.46421766	379.593731	40.1083053
HOXA1	0.67241379	6.89655172	10.2564103	0.05391354	2.17880641	40.4129753
HOXA10	0.06896552	0.55172414	8	0.00386801	0.13398161	34.6383795
HOXA11	0.03448276	0.86206897	25	0.00213077	0.20523042	96.317301
HOXA13	0.0862069	1.34482759	15.6	0.00626265	0.28457532	45.44006
HOXA2	0.0862069	0.4137931	4.8	0.00594878	0.0942128	15.8373439
HOXA3	0.31034483	4.34482759	14	0.01771198	0.85736169	48.4057419
HOXA4	1.86206897	21.5517241	11.5740741	0.10740431	4.52032594	42.0870079
HOXA5	2.4137931	25.0172414	10.3642857	0.14787299	5.51172249	37.2733555
HOXA6	0.81034483	10.4137931	12.8510638	0.04676672	2.20554646	47.1605933
HOXA7	0.22413793	2.77586207	12.3846154	0.01083497	0.55066839	50.8232386
HOXA9	0	0.13793103		0	0.03081868	
HOXB1	0	0.01724138		0	0.00459697	

HOXB13	3.87931034	47.3448276	12.2044444	0.23620606	10.2461055	43.3778273
HOXB2	0.93103448	10.0517241	10.7962963	0.05521345	2.19845338	39.8173515
HOXB3	0.5	5.56896552	11.137931	0.0282497	1.04684621	37.0569013
HOXB4	0.13793103	1.5862069	11.5	0.00656266	0.31519301	48.0282263
HOXB5	0.25862069	1.4137931	5.46666667	0.01480779	0.26923901	18.1822596
HOXB6	0.05172414	0.27586207	5.33333333	0.00326591	0.05005224	15.3256432
HOXB7	0.12068966	1.37931034	11.4285714	0.00725499	0.29593589	40.790674
HOXB8	0.0862069	1.12068966	13	0.00511709	0.25781894	50.3839367
HOXB9	0.89655172	8.82758621	9.84615385	0.05952766	2.08847109	35.0840444
HOXC10	5.20689655	72.7068966	13.9635762	0.31231218	15.6025467	49.9581753
HOXC11	0.01724138	0.13793103	8	0.00091522	0.03510645	38.3584098
HOXC13	3.4137931	42.8793103	12.5606061	0.20614269	8.97612187	43.5432458
HOXC4	0.31034483	2.81034483	9.05555556	0.01867771	0.59908288	32.0747581
HOXC5	0.01724138	0.36206897	21	0.00076563	0.06853269	89.5118403
HOXC6	0.13793103	1.86206897	13.5	0.00874873	0.40025839	45.75043
HOXC8	0.75862069	9.18965517	12.1136364	0.0448594	1.86987055	41.6829148
HOXD1	0.12068966	1.43103448	11.8571429	0.00769699	0.35131952	45.6437746
HOXD10	0.0862069	0.55172414	6.4	0.00442006	0.11373933	25.7325292
HOXD11	0.06896552	0.70689655	10.25	0.00350064	0.14885369	42.5218806
HOXD13	1.94827586	16.6551724	8.54867257	0.11122824	3.47302771	31.2243347
HOXD3	0.32758621	4.20689655	12.8421053	0.02369443	0.99506892	41.9958962
HOXD4	0.98275862	5.5862069	5.68421053	0.05624351	1.20798142	21.4777051
HOXD8	4.0862069	29.9482759	7.32911392	0.27788984	8.02244446	28.8691537
HOXD9	7.60344828	80.1206897	10.537415	0.42630265	16.1057794	37.7801526
HR	26.1724138	267.327586	10.2140975	1.66770299	60.6334534	36.3574653
HSF1	1938.08621	8790.51724	4.53566885	116.562841	1919.77571	16.4698775
HSF2	944.775862	6682.5	7.07310619	60.1446625	1557.16936	25.8903999
HSF4	1103.91379	5075.87931	4.59807581	63.2298732	1051.91279	16.6363262
HTATIP2	20.1551724	208.137931	10.326775	1.2896047	50.5426993	39.1923969
IER2	1233.82759	8194.82759	6.64179313	70.8558906	1727.41536	24.3792767
IFI16	89.2241379	960.034483	10.7598068	5.58284921	214.579855	38.4355455
IGHMBP2	594.758621	4957.74138	8.33572008	36.3728961	1093.61273	30.0666939
IKZF1	60.3275862	631.793103	10.4727065	4.05324822	156.595364	38.6345361
IKZF2	132.37931	1411.96552	10.6660589	8.1633699	313.87727	38.4494729
IKZF3	1.10344828	10.1724138	9.21875	0.07093553	2.48440001	35.0233503
IKZF5	288.103448	2809.46552	9.75158588	18.0494497	641.990509	35.5684257
INSM1	1318.44828	7748.15517	5.8767229	82.5055595	1791.1142	21.7090122
IRF1	63.6206897	382.482759	6.01192412	4.13950704	94.0286002	22.714927
IRF2	164.224138	1647.2931	10.0307612	10.3847608	377.984619	36.3980091
IRF3	631.034483	5475.86207	8.67759563	38.0679082	1202.54131	31.5893718
IRF4	3.79310345	41.8793103	11.0409091	0.23457561	9.30922064	39.685374
IRF5	48.0862069	492.172414	10.2352098	3.09719954	114.436004	36.948218
IRF6	22.2068966	230.586207	10.3835404	1.57753681	59.98209	38.0226247
IRF7	191.344828	1308.7931	6.83997117	11.2330377	276.394751	24.6055216
IRF8	3.93103448	35.6034483	9.05701754	0.25052507	8.48236324	33.8583409
IRF9	240.448276	1486.31034	6.18141403	14.8180584	330.138886	22.2794969
IRX1	177.103448	1171.56897	6.61516745	10.3453738	248.716986	24.0413725
IRX2	135.689655	1119.34483	8.24930114	7.91333734	238.434171	30.1306719
IRX3	72.2241379	478.913793	6.63093817	3.93927974	90.4757162	22.9675784
IRX5	76.4827586	579.517241	7.57709648	4.43737937	119.897343	27.019854
IRX6	7.20689655	76.8275862	10.6602871	0.44978099	18.1399232	40.3305686
ISL1	262.568966	2428.72414	9.24985226	15.8804526	527.797141	33.2356486

ISL2	1.84482759	13.7586207	7.45794393	0.10410068	2.70567597	25.9909539
JARID2	1288.13793	10406.6897	8.07886283	77.0635373	2245.30963	29.1358237
JDP2	160.87931	1360.63793	8.45750723	10.0249521	305.126777	30.4367315
JUN	4297.81034	21153.2759	4.92187281	252.247343	4560.4338	18.0792144
JUNB	1686.01724	6134.39655	3.63839491	87.7993107	1207.15975	13.7490801
JUND	6899.46552	10316.431	1.49525076	415.993747	2262.18128	5.4380175
KCNH8	551.206897	5410.60345	9.81592118	31.4127608	1101.5795	35.0678981
KCNIP3	202.137931	1886.36207	9.33205391	11.8898026	395.23378	33.2414079
KHSRP	4967.77586	16507.5862	3.32293297	303.750624	3677.66965	12.1075295
KLF1	5.70689655	61.9137931	10.8489426	0.34150429	13.6074195	39.8455297
KLF10	603.586207	5727.56897	9.48923103	37.5081759	1292.92237	34.4704143
KLF11	613.706897	6000.08621	9.77679449	38.6077909	1376.79103	35.6609638
KLF12	1507.44828	15313.1379	10.1583173	98.237328	3625.98981	36.9105094
KLF13	2194.24138	18985.6897	8.65250735	137.31916	4325.98641	31.5031523
KLF14	4.60344828	47.7241379	10.3670412	0.25794836	9.65213794	37.4188765
KLF15	148.655172	1501.74138	10.1021805	9.31913252	340.167505	36.5020568
KLF16	591.810345	4651.12069	7.85914057	36.0595329	1031.32382	28.6005874
KLF17	0.5	5.48275862	10.9655172	0.03399713	1.37098846	40.3265937
KLF2	51.0689655	364.931034	7.1458474	3.1900305	83.7477947	26.2529762
KLF3	633.5	6355.5	10.0323599	40.5111539	1476.54556	36.4478771
KLF4	87.7586207	829.172414	9.44833006	5.34726603	189.807387	35.4961556
KLF6	1300.48276	11067.1897	8.51006258	81.1199395	2526.66326	31.1472527
KLF8	183.362069	1847.55172	10.0759756	12.3625648	449.203321	36.3357709
KLF9	57.5172414	629.155172	10.9385492	3.55458384	143.918653	40.4881864
KMT2A	2486.24138	25595.931	10.2950306	156.15197	5776.62319	36.9935979
KRBOX4	232.793103	2273.27586	9.76521997	14.9246133	527.123175	35.3190508
LBX1	0.22413793	3.48275862	15.5384615	0.01289083	0.6171376	47.8741508
LCORL	341.810345	3098.12069	9.06385876	21.5090959	706.977551	32.8687711
LEF1	706.482759	5593.34483	7.91717103	43.2125307	1224.02721	28.325747
LHX1	154.5	1461.91379	9.4622252	9.76078132	332.086348	34.022517
LHX2	2815.17241	13252.6552	4.70758207	162.541449	2769.17755	17.036747
LHX3	1.87931034	16.2413793	8.64220183	0.11944988	3.58542999	30.0161882
LHX4	22.2758621	75.8103448	3.40325077	1.31441485	16.2993772	12.4004816
LHX6	636.603448	5294.12069	8.31619858	36.3301888	1121.43436	30.8678373
LHX9	179.844828	1712.75862	9.52353561	11.3193834	378.867729	33.4707038
LITAF	840.655172	6888.7931	8.19455269	54.4823184	1647.69547	30.2427561
LMO2	315.172414	2487.55172	7.89266958	20.5398357	602.807354	29.3482071
LMX1A	295.586207	2782.44828	9.41332245	18.4698503	639.553998	34.6269183
LMX1B	10.4482759	123	11.7722772	0.59426831	24.1259778	40.5977865
LRRFIP1	739.551724	4924.03448	6.658134	46.3794619	1127.43619	24.3089537
LZTFL1	369.137931	3307.15517	8.95913125	23.8118606	783.390585	32.8991756
MACC1	4.03448276	46.2931034	11.474359	0.25267749	10.5507697	41.7558752
MAF	945.413793	5478.67241	5.79499945	56.8616284	1220.54086	21.4651056
MAFA	12.7413793	106.086207	8.32611637	0.85108807	25.9925093	30.5403287
MAFB	283.896552	2278.15517	8.02459614	18.9764068	557.767608	29.3926882
MAFF	353.655172	2731.32759	7.72313768	21.8102628	635.403857	29.1332508
MAFG	1198.2069	11105.2241	9.26820249	75.3445142	2550.07089	33.8454753
MAFK	358.810345	2822.43103	7.86608044	21.4677443	612.541858	28.5331263
MAX	933.12069	6889.77586	7.38358493	60.1401588	1614.08152	26.8386641
MAZ	9330.01724	31018.2931	3.32456975	582.998049	7126.19555	12.2233609
MECOM	25.4827586	275.896552	10.826793	1.77454263	73.2863549	41.2987287
MED1	1196.62069	10034.1724	8.38542447	78.1791181	2370.73622	30.3244176

MEF2A	738.62069	5866.31034	7.94225023	48.4354501	1404.88821	29.0053713
MEF2B	0.39655172	3.67241379	9.26086957	0.02238383	0.77205817	34.4917888
MEF2BNB-ME	2.31034483	32.0172414	13.858209	0.14174234	7.11455566	50.1935801
MEF2C	913.310345	7947.03448	8.70135166	64.4236259	2054.86364	31.896119
MEF2D	1729.63793	13917.7759	8.04664121	106.260175	3101.86697	29.1912465
MEIS1	738.465517	5834.34483	7.90063272	46.1416368	1327.25137	28.7647223
MEIS2	4722.2069	23192.0172	4.91126665	319.226956	5761.5049	18.0483033
MEOX1	5.60344828	57.9137931	10.3353846	0.3210292	13.0229153	40.5661393
MEOX2	0.29310345	2.87931034	9.82352941	0.01827418	0.6589823	36.060844
MESP1	277.275862	1521.43103	5.48706629	17.2370827	345.618522	20.050871
MESP2	8.13793103	81.4482759	10.0084746	0.50379898	18.5544192	36.8290132
MGA	688	7246.58621	10.5328288	44.8846334	1708.63571	38.0672757
MITF	40.6034483	470.224138	11.5808917	2.48283515	105.261682	42.3957597
MIXL1	2.39655172	23.6896552	9.88489209	0.17140897	5.92164583	34.5468836
MKX	21.1551724	221.396552	10.4653627	1.24119526	47.5328066	38.2959942
MLX	568.896552	4380.03448	7.69917566	36.699555	1039.91437	28.3358851
MLXIP	1519.62069	13867.3448	9.12553042	91.1177658	2988.64758	32.7998339
MLXIPL	137.931034	1136.01724	8.236125	8.14392309	243.588865	29.9105066
MN1	4092.63793	27322.7759	6.67607942	249.023037	6053.49233	24.3089651
MNT	1483.72414	10753.1897	7.2474319	87.4177089	2291.08888	26.2085212
MSC	3.55172414	48.1034483	13.5436893	0.25292846	13.4492725	53.1742161
MSGN1	0.13793103	1.62068966	11.75	0.00691647	0.32282075	46.674231
MSX1	252.568966	1618.2931	6.40733156	15.9151349	379.71115	23.858494
MSX2	36.9827586	393.603448	10.6428904	2.36333601	93.0224652	39.3606599
MTF1	327.310345	3581.65517	10.9426886	20.8190883	823.931145	39.5757553
MTF2	774.689655	6617.7931	8.54250868	48.229639	1488.53577	30.8635063
MXD1	522.965517	5101.51724	9.75497824	32.2547758	1158.90818	35.9298165
MXD3	804.189655	3535.82759	4.39675836	46.3123782	737.596609	15.9265544
MXD4	2645.24138	16950.5862	6.40795443	162.870483	3796.15249	23.307799
MXI1	1174.75862	8123.18966	6.91477339	75.5716891	1918.39533	25.3851059
MYB	58.3103448	530.189655	9.09254879	3.58603255	116.97645	32.620019
MYBL1	56.1551724	518.741379	9.237642	3.4973633	117.534228	33.6065252
MYBL2	995.344828	7849.06897	7.88577862	63.6052875	1834.40152	28.8403935
MYC	278.87931	2736.74138	9.81335394	18.1027835	662.983827	36.6233086
MYCL	704.551724	6821.65517	9.68226312	43.678481	1519.82032	34.7956312
MYCN	1366.84483	10368.8103	7.58594548	84.8086331	2326.11875	27.4278533
MYEF2	1749.65517	12033.8621	6.87784785	107.769831	2689.51604	24.9561127
MYF5	0	0.03448276		0	0.00858788	
MYF6	0.5	5.01724138	10.0344828	0.03393646	1.35294242	39.8669327
MYOCD	1.01724138	10.3965517	10.220339	0.06488548	2.57114285	39.6258577
MYOD1	0.79310345	9.03448276	11.3913043	0.04693372	1.93506744	41.2297915
MYOG	3.55172414	31.8448276	8.96601942	0.20846861	6.71937746	32.2320831
MYPOP	276.224138	1893.48276	6.85487797	16.7250012	415.627081	24.8506458
MYRF	169.275862	1645.58621	9.72132817	9.39570979	322.767801	34.3526788
MYT1	1756.2069	13935.1724	7.93481249	105.652726	3012.59466	28.5141215
MYT1L	1616.03448	13768	8.51962019	103.345432	3176.30124	30.7348007
MZF1	962.413793	6333.63793	6.58099248	55.9944662	1330.85926	23.7676925
NACC1	1691.46552	13535.1207	8.00200805	106.591778	3107.26035	29.1510321
NACC2	729.206897	6100.91379	8.36650589	46.6500533	1427.44011	30.5988956
NANOG	0.0862069	0.96551724	11.2	0.00447541	0.23048671	51.5007443
NEUROD1	113.275862	1228.48276	10.8450533	6.801301	258.371972	37.9886101
NEUROD2	2497.87931	8426.31034	3.3733857	161.85255	1956.13067	12.085881

NEUROD4	66.1896552	698.844828	10.5582183	4.71234469	181.390342	38.4925879
NEUROG1	92.5517241	837.482759	9.04880775	5.64624439	181.73881	32.1875563
NEUROG2	1202.41379	8231.12069	6.84549756	72.8599878	1794.93703	24.6354286
NEUROG3	3.20689655	25.4482759	7.93548387	0.1802624	5.00169725	27.7467586
NFAT5	604.517241	5836.06897	9.65409845	37.6757353	1315.59736	34.9189565
NFATC1	110.844828	1189.55172	10.7316846	6.62245112	257.148628	38.8298264
NFATC2	7.67241379	85.6206897	11.1595506	0.51700259	20.2725383	39.2116764
NFATC3	518.224138	5192.01724	10.0188642	33.4068833	1218.69345	36.4803096
NFATC4	1384.58621	12887.3966	9.30776032	78.7329465	2657.98652	33.7595205
NFE2	7.56896552	90.362069	11.9384966	0.49191443	21.5295046	43.7667682
NFE2L1	5489.31034	35707.8966	6.50498775	339.383356	8113.36929	23.9062086
NFE2L2	794.551724	6873.32759	8.65057287	51.1080879	1632.842	31.9487985
NFE2L3	114.362069	979.672414	8.56641037	7.14100267	223.27451	31.266549
NFIC	667.689655	7167.2931	10.7344678	40.5968337	1564.68457	38.5420345
NFIL3	754.189655	6310.98276	8.36789886	48.1375568	1470.59137	30.5497717
NFIX	1474.68966	13253.9655	8.98763036	91.3868669	2945.76325	32.2339889
NFKB1	227.258621	1949.31034	8.57749791	14.4499444	448.527781	31.0401044
NFKB2	317.034483	2398.77586	7.56629323	18.8618329	525.074973	27.8379612
NFKBIZ	30.6034483	250.603448	8.18873239	1.83188158	54.07893	29.5209749
NFX1	885.051724	7303.60345	8.25217696	55.3100791	1645.72019	29.7544357
NFXL1	284.637931	2390.65517	8.39893391	17.6024358	535.902022	30.4447651
NFYA	1285.41379	7093.84483	5.51872468	82.1672057	1646.15101	20.0341608
NFYC	1202.39655	7269.34483	6.0457133	76.0127832	1680.98918	22.114559
NHLH1	654.793103	5469.72414	8.35336247	37.3647163	1117.36633	29.9043172
NHLH2	58.2068966	553.724138	9.51303318	3.18389858	106.375694	33.4105159
NKRF	415.5	2685.5	6.46329723	27.0829531	633.549049	23.3929087
NKX1-2	2.84482759	17.6724138	6.21212121	0.1927616	4.58163993	23.7684263
NKX2-1	340.965517	2285.87931	6.70413633	18.1522925	443.200337	24.4156675
NKX2-2	68.3275862	604.155172	8.84203886	3.96601204	124.316304	31.3454176
NKX2-3	3.67241379	34.7931034	9.4741784	0.23869498	8.04603	33.7084167
NKX2-5	1.48275862	10.9482759	7.38372093	0.09586512	2.94148111	30.6835385
NKX2-6	0.22413793	1.12068966	5	0.01445168	0.22863098	15.8203712
NKX2-8	3.32758621	21.9827586	6.60621762	0.19353368	4.64150768	23.9829452
NKX3-1	2	23.137931	11.5689655	0.1424641	6.21709465	43.639728
NKX3-2	4.67241379	39.1206897	8.37269373	0.28238481	8.8836849	31.4595004
NKX6-1	7.44827586	57.5344828	7.72453704	0.44530709	12.3678677	27.7737946
NKX6-2	20.3965517	167.655172	8.21978022	1.05253102	31.3288085	29.7652117
NKX6-3	11.4310345	114.87931	10.0497738	0.67329921	23.9582331	35.5833376
NOBOX	0.03448276	0.65517241	19	0.00215172	0.13990018	65.0177023
NPAS1	320.672414	2309.87931	7.20323673	18.0373122	467.690776	25.9290725
NPAS2	489.137931	4062.94828	8.30634473	32.719308	982.024268	30.0136014
NPAS3	1154.81034	9907.37931	8.57922632	73.3378747	2288.01923	31.1983301
NPAS4	2671.98276	12896.2414	4.82646879	129.663128	2290.19472	17.662652
NR0B1	2.96551724	29.2758621	9.87209302	0.19596166	7.07197076	36.0885432
NR1D1	321.448276	3079.86207	9.58120575	19.7648304	704.408537	35.639493
NR1D2	477.948276	4280.01724	8.95497998	30.6811893	1011.00962	32.9521002
NR1H2	861.275862	5489.74138	6.37396405	53.7326908	1253.30695	23.3248499
NR1H3	141.034483	1204.43103	8.53997555	8.8897411	276.80503	31.1375806
NR1H4	0.27586207	3.12068966	11.3125	0.01943945	0.75640227	38.9106785
NR1I2	5.29310345	47.5517241	8.98371336	0.29581647	9.50592252	32.134528
NR1I3	38.7241379	274.448276	7.08726625	2.45880211	62.3747427	25.3679394
NR2C1	435.37931	3272.98276	7.51754316	26.2939774	718.59957	27.329436

NR2E1	1171.62069	9463.96552	8.07767019	69.7951683	2051.76959	29.3970146
NR2E3	11.3965517	88.6724138	7.7806354	0.62624521	17.3471727	27.7002881
NR2F1	10885.3448	31172.2931	2.86369367	636.114739	6590.66466	10.3608111
NR2F2	4892.98276	22715.4483	4.64245418	296.712422	4993.79891	16.8304342
NR2F6	892.655172	4707.32759	5.27339978	55.7285016	1082.3808	19.422392
NR3C1	190.431034	2121.89655	11.1425985	12.8622161	524.511073	40.7792149
NR3C2	72.6206897	810.793103	11.1647673	4.75697559	191.434429	40.2428866
NR4A1	1866.18966	12619.2414	6.76203587	96.4569229	2466.2388	25.5682923
NR4A2	396.775862	3787.75862	9.54634337	24.8992295	863.933262	34.6971887
NR4A3	568.310345	4731.94828	8.32634549	31.9899845	992.075606	31.012069
NR5A1	12.0344828	124.844828	10.3739255	0.67966595	25.4213603	37.4027276
NR5A2	1.32758621	13.0862069	9.85714286	0.10770393	4.28891134	39.8213093
NR6A1	89.1896552	980.155172	10.9895612	5.31930417	208.830059	39.2589053
NRF1	566.62069	4265.96552	7.5287853	35.4391732	967.638363	27.3042026
NRG1	332.568966	2740.68966	8.24096635	21.0432946	631.29043	29.9996005
NRL	54.4482759	507.448276	9.31982267	3.56180289	122.006833	34.2542351
OLIG1	422.62069	3211.05172	7.59795202	24.0152989	664.268383	27.6602172
OLIG2	160.241379	1255.77586	7.83677641	9.92251111	283.074854	28.52855
OLIG3	71.7931034	557.603448	7.76681076	4.04938442	112.165924	27.6995002
ONECUT1	15.637931	148.086207	9.46968026	0.83043068	27.3903807	32.9833439
ONECUT2	139.37931	1612.17241	11.5667986	9.18547538	388.252042	42.2680401
ONECUT3	3.84482759	26.1896552	6.81165919	0.2259824	5.62812342	24.9051407
OSR1	4.17241379	41.7931034	10.0165289	0.25100994	8.63911785	34.4174332
OSR2	0.72413793	8.62068966	11.9047619	0.05039635	1.99713134	39.6284891
OTP	35.8275862	325.689655	9.09047161	2.04190705	66.3173727	32.4781545
OTX1	542.844828	5254.93103	9.68035572	31.4570978	1093.27293	34.7544118
OTX2	836.465517	7108.81034	8.49862929	52.7433235	1683.05799	31.9103514
OVOL1	9.4137931	102.844828	10.9249084	0.55554657	21.6369915	38.9472144
OVOL2	0.25862069	2.86206897	11.0666667	0.01649979	0.65387648	39.6293935
PATZ1	2246.15517	16778.2759	7.46977594	142.332038	3890.47936	27.3338274
PAX1	11.5172414	126.913793	11.0194611	0.64846501	25.5513522	39.402823
PAX2	8.44827586	85.8965517	10.1673469	0.48125905	16.2715166	33.8103078
PAX3	81.5689655	851.362069	10.4373283	4.11241473	146.948498	35.7328984
PAX4	0.12068966	0.94827586	7.85714286	0.00677111	0.21840866	32.2559781
PAX5	18.1551724	188.5	10.382716	1.06944622	39.3500561	36.7947967
PAX6	2217.03448	11623.5862	5.24285314	138.276014	2590.60126	18.735001
PAX7	22.0862069	237.810345	10.7673692	1.06408616	37.7506471	35.4770586
PAX8	3.93103448	46.6551724	11.8684211	0.34338902	15.1705006	44.1787581
PAX9	0.9137931	8.79310345	9.62264151	0.05836751	2.07172034	35.4944072
PBX1	4127.91379	28448.5172	6.89174209	282.324642	7194.77267	25.4840407
PBX2	2665.2931	13922.2586	5.22353755	158.654116	3013.51133	18.994221
PBX3	2705.44828	15402.1207	5.69300135	184.139222	3902.17128	21.1914183
PBX4	199.293103	1475.82759	7.40531188	11.5135489	306.170134	26.59216
PCGF6	139.103448	1111	7.98686168	8.9508181	262.162048	29.2891717
PDX1	0.9137931	10.7413793	11.754717	0.0494636	2.15060137	43.4784609
PEG3	6.84482759	83.8448276	12.2493703	0.41963359	18.2610988	43.516771
PGR	0.87931034	9.12068966	10.372549	0.05943664	2.41423922	40.6187038
PGS1	622.155172	4723.67241	7.59243453	38.0244038	1044.08348	27.4582472
PHB	1551.24138	3918.37931	2.52596363	101.957395	956.55333	9.38189256
PHF10	423.396552	3197.67241	7.55242904	26.1516171	715.813459	27.3716709
PHF20	1214.84483	10514.5172	8.65502902	78.5615126	2463.07935	31.3522394
PHF6	1481.53448	12008.8103	8.105657	95.5412244	2843.7002	29.7641172

PHF7	164.189655	1175.39655	7.1587735	9.60039916	246.881579	25.7157619
PHOX2A	1.10344828	9.01724138	8.171875	0.06250353	1.98073316	31.68994
PHOX2B	0.10344828	2.4137931	23.3333333	0.00674838	0.48122056	71.3090251
PITX1	4.37931034	43.2241379	9.87007874	0.23399262	8.55304677	36.5526346
PITX2	6.34482759	68.2586207	10.7581522	0.44107537	17.1933269	38.9804735
PITX3	1.51724138	13.5517241	8.93181818	0.10128083	3.15990437	31.1994311
PKNOX1	586.793103	4797.2931	8.1754422	38.3917488	1149.46735	29.9404789
PKNOX2	683.793103	6821.22414	9.97556732	41.8190154	1497.65396	35.8127503
PLAG1	193.37931	2138.7069	11.0596469	11.2021679	445.398617	39.7600376
PLAGL1	551.827586	5211.89655	9.4447916	32.3520064	1092.99078	33.7843275
PLAGL2	861.172414	8837.91379	10.2626532	52.9420505	1959.08028	37.0042388
PLSCR1	42.1724138	452.896552	10.739166	2.78249149	111.010143	39.8959507
POU1F1	0.43103448	3.36206897	7.8	0.02889792	0.87527657	30.2885623
POU2F1	767.931034	7729.68966	10.065604	48.7183622	1777.357	36.4822814
POU2F2	789.224138	6948.60345	8.80434735	45.2552485	1416.3827	31.297645
POU2F3	1.89655172	15.9482759	8.40909091	0.12167109	3.82589202	31.4445456
POU3F1	424.172414	2055.01724	4.84476872	24.5001079	430.21492	17.5597153
POU3F2	1573.32759	9833.60345	6.25019451	98.5416557	2288.36238	23.2222847
POU3F3	5542.13793	16676.0172	3.00895024	329.781059	3623.1456	10.9865182
POU3F4	210.931034	1896.05172	8.98896518	13.1188128	427.69751	32.6018456
POU4F1	34.362069	322.793103	9.39387858	2.32532437	79.5968344	34.230422
POU4F2	3.70689655	41.862069	11.2930233	0.21855067	8.16722425	37.3699344
POU4F3	2.31034483	19.2758621	8.34328358	0.13949041	4.01183065	28.760619
POU5F1	1.89655172	11.5517241	6.09090909	0.11404212	2.53074792	22.1913444
POU6F1	1084.41379	9233.27586	8.51453193	66.0192629	2029.52491	30.7414052
POU6F2	55.2758621	556.086207	10.0601996	3.3267673	118.835017	35.7208684
PPARA	198.189655	2107.53448	10.6339278	12.7391152	495.862054	38.9243717
PPARD	773.034483	6982.37931	9.0324293	46.7159949	1522.13603	32.5827597
PPARG	14.7413793	147	9.97192982	0.85762135	30.3067536	35.338152
PRDM1	4.0862069	47.2586207	11.5654008	0.28102635	11.8943852	42.3248047
PRDM10	512.327586	5019.27586	9.79700488	31.950038	1129.66583	35.3572608
PRDM12	42.362069	387.931034	9.15750916	2.56114612	85.4803329	33.3758125
PRDM13	6.29310345	53.362069	8.47945205	0.31669582	9.07104529	28.6427691
PRDM14	0.03448276	0.72413793	21	0.00208456	0.155508	74.5997537
PRDM15	345.034483	3322.87931	9.63057166	20.6529232	712.316805	34.4898782
PRDM16	843.465517	8566.89655	10.1567834	50.0050463	1836.70828	36.7304585
PRDM2	1575.32759	14895.2241	9.45531854	99.6496293	3386.91074	33.988192
PRDM4	921.706897	9096.89655	9.86961971	58.406208	2095.48604	35.8777964
PRDM5	91.362069	808.982759	8.85468956	5.68137179	182.81295	32.1776073
PREB	1033.31034	7500.65517	7.25886004	65.5415113	1733.36747	26.4468646
PROP1	0.0862069	1.05172414	12.2	0.0037989	0.24360225	64.1244109
PROX1	156.241379	1664.22414	10.6516222	9.91434819	375.730417	37.8976418
PRRX1	33.0689655	364	11.0072993	2.30208609	92.2608972	40.0770838
PRRX2	2.98275862	19.3275862	6.47976879	0.18497669	4.32392595	23.3755183
PTF1A	4.39655172	43.6896552	9.9372549	0.23865318	8.15710412	34.1797418
PTH	0.01724138	1.15517241	67	0.00162747	0.28688728	176.278115
PURA	230.603448	2091.87931	9.0713271	14.6743261	490.025392	33.393383
PURB	999.086207	9322.03448	9.33056068	62.8458406	2131.03264	33.9088891
RAD21	4810.37931	24701.9483	5.13513523	313.078315	5921.35312	18.9133288
RAI1	2992.44828	24619.7414	8.22729054	178.6462	5290.42997	29.6140079
RARA	1036.58621	9424.36207	9.09173015	62.5028483	2058.84203	32.9399714
RARB	106.189655	1122.01724	10.5661633	6.77970428	261.763809	38.6099154

RARG	214.327586	2169.05172	10.1202639	12.7687955	463.72392	36.3169667
RAX	63.0172414	607.103448	9.63392613	3.97099252	144.044675	36.2742247
RAX2	0.37931034	4.05172414	10.6818182	0.02148007	0.92140355	42.8957471
RBPJ	2282.34483	16604.3621	7.27513295	145.982334	3915.2166	26.8197973
RBPJL	0.12068966	1.17241379	9.71428571	0.00649236	0.27405225	42.2115264
RCOR1	998.810345	9318.37931	9.32947817	61.4249677	2079.14192	33.848482
REL	177.741379	1875.05172	10.5493258	11.0634222	417.514838	37.7383084
RELA	1423.55172	9159.58621	6.43431921	86.8630242	2050.49796	23.6061084
RELB	114.551724	870.568966	7.59978928	7.16064795	201.993719	28.2088604
REST	311.551724	3153.18966	10.1209186	19.3490706	710.227372	36.70602
RFX1	679.413793	4860.96552	7.1546465	40.4913286	1038.2054	25.6401911
RFX2	430.775862	4065.27586	9.43710226	25.4517398	876.344014	34.4315957
RFX3	1033.22414	9573.89655	9.26604035	65.955718	2228.81292	33.7925655
RFX4	556.310345	5039.74138	9.05922643	33.3257102	1080.57292	32.4246029
RFX5	991.741379	8925.77586	9.00010431	59.9935335	1961.80423	32.7002614
RFX6	1.94827586	16.2413793	8.33628319	0.11722666	3.52207217	30.0449748
RFX7	1228.53448	11840.2931	9.6377377	77.9438714	2723.17013	34.9375785
RHOXF2	0	0		0	0	
RHOXF2B	0	0		0	0	
RNF112	567.155172	4641.10345	8.18312813	32.9650993	959.720055	29.113216
RNF141	1095.87931	8563.67241	7.81443023	71.7953331	2062.11956	28.722195
RNF2	525.327586	4065.84483	7.73963701	34.6181884	986.371441	28.4928671
RORA	224.741379	2278.58621	10.1387035	13.6853086	504.062045	36.8323478
RORB	194.275862	1754.55172	9.03123891	12.0977602	396.767931	32.7968092
RORC	10.9655172	115.517241	10.5345912	0.68501945	27.8939397	40.7199236
RREB1	157.775862	1508.01724	9.55797181	9.31887893	320.23513	34.364126
RUNX1	14.8965517	147.724138	9.91666667	1.06047265	40.6349395	38.3177629
RUNX2	25.6551724	277.568966	10.8192204	1.46421606	56.8114703	38.7999231
RUNX3	0.82758621	11.0689655	13.375	0.04883338	2.34528559	48.0262771
RXRA	1501.67241	13991.4138	9.31722103	91.7140031	3090.74493	33.6998149
RXRB	1681.24138	9926.03448	5.9039913	103.389344	2239.9958	21.665635
RXRG	886.224138	5790.82759	6.53426976	57.7877885	1403.76999	24.2918102
SALL1	1579.44828	14116.3448	8.93751637	96.5396893	3132.7027	32.4498941
SALL2	3097.93103	25025.1552	8.07802204	191.964199	5641.032	29.3858544
SALL3	663.155172	5289.34483	7.97602891	40.3351325	1176.53914	29.1690907
SALL4	76.5862069	809.793103	10.5736155	4.25325928	160.034176	37.6262451
SARNP	896.155172	2557.08621	2.85339669	58.2824233	615.82978	10.5663036
SATB1	825.310345	7109.39655	8.61420991	49.5082389	1534.29348	30.9906697
SATB2	148.965517	1513.81034	10.1621528	9.58086593	346.466425	36.162329
SCML4	51.8965517	394.189655	7.59568106	3.16912333	87.4468445	27.5933863
SCRT1	1745.44828	11635.8966	6.66642301	107.107199	2575.57226	24.0466773
SCRT2	1486.65517	9832.41379	6.61378239	95.1951112	2263.61739	23.7787147
SCX	83.2413793	462.689655	5.55840928	4.79246751	96.0700077	20.0460426
SHOX	7.67241379	75.5344828	9.84494382	0.45375329	14.5445754	32.053928
SHOX2	112.655172	948.655172	8.42087542	7.45323749	225.938283	30.3141129
SIM1	5.01724138	56.3965517	11.2405498	0.33130655	13.4690261	40.65427
SIM2	40.7931034	409.982759	10.0502959	2.74155189	101.091212	36.8737182
SIX1	1.68965517	14.6724138	8.68367347	0.11834538	3.79902625	32.1011784
SIX2	7.94827586	81.9655172	10.3123644	0.47720498	17.0554789	35.7403625
SIX3	2158.34483	11232.7759	5.2043472	137.742461	2596.49264	18.850343
SIX4	56.5689655	552.948276	9.77476379	3.37645477	119.846684	35.4948288
SIX5	685.5	5272.91379	7.69206972	39.9320032	1111.40404	27.8324139

SIX6	0.12068966	1.51724138	12.5714286	0.00627949	0.34252133	54.54608
SKIL	371.87931	3520.62069	9.46710557	23.5606045	812.942446	34.504312
SMAD1	563.103448	4153.17241	7.37550521	37.1178405	994.703908	26.7985393
SMAD2	995.189655	8734.27586	8.77649382	65.3988154	2109.39526	32.2543343
SMAD3	755.517241	7085.03448	9.37772707	46.1986041	1576.92463	34.1335991
SMAD4	1612.24138	12605.1897	7.81842584	103.702931	2971.88214	28.6576484
SMAD5	853.965517	7804.37931	9.13898647	54.088473	1810.93826	33.4810387
SNAI1	37.2241379	452.689655	12.1611857	2.38150543	107.298083	45.0547296
SNAI2	47.6551724	485.034483	10.1780029	2.96205113	113.190101	38.2134191
SNAI3	55.4482759	523.931034	9.44900498	3.411742	114.301891	33.5025013
SOHLH1	0.51724138	4.34482759	8.4	0.03038806	0.89905886	29.585925
SOHLH2	0.79310345	6.93103448	8.73913043	0.04708573	1.5288115	32.4686828
SOX1	2374	11775.5172	4.96020103	145.488375	2726.52025	18.7404681
SOX10	7.17241379	65.2586207	9.09855769	0.45642751	15.0732144	33.024334
SOX12	5158.05172	26395.6034	5.1173592	314.354473	5824.7428	18.5292188
SOX13	474.948276	4134.10345	8.70432352	27.1223979	841.476505	31.0251515
SOX14	9.25862069	128.206897	13.8472998	0.60298679	28.6713319	47.5488562
SOX15	76.2413793	609.137931	7.98959747	4.44849964	127.727572	28.7125058
SOX17	0.27586207	2.51724138	9.125	0.01930071	0.60362756	31.2748924
SOX18	5.36206897	34.1551724	6.36977492	0.32934441	7.65936584	23.2564018
SOX2	6121.65517	22309.2414	3.64431526	374.130278	5019.63489	13.4168101
SOX21	1198.82759	7527.2931	6.27887879	73.2851655	1698.81404	23.180872
SOX3	1310.53448	7340.08621	5.60083409	73.7165661	1483.30133	20.1216823
SOX30	2.5862069	17.8965517	6.92	0.16509673	4.14333022	25.0963802
SOX5	781.844828	6229.22414	7.96734073	50.2017287	1439.6514	28.6773272
SOX6	662.637931	6496.87931	9.80456899	42.2295548	1513.79274	35.8467606
SOX7	14.362069	135.37931	9.42617047	0.87738319	29.447469	33.562837
SOX8	1027.87931	7236.94828	7.04065954	60.9563928	1564.3424	25.6633034
SOX9	2910	17058.6379	5.86207489	173.314366	3696.33335	21.3273339
SP1	1015.60345	10222.8448	10.0657839	61.7786279	2253.89404	36.4833943
SP110	15.8103448	154.172414	9.75136314	1.00124056	35.8010459	35.7566875
SP2	435.586207	4535.2931	10.41193	26.243633	978.793208	37.2964067
SP3	1538.13793	13261.069	8.62150832	98.1777897	3101.97612	31.5954977
SP4	538.603448	5130.2931	9.52517686	35.5312122	1225.24798	34.4837089
SP5	67.9827586	512.155172	7.53360385	4.8872328	138.528144	28.3449038
SP6	21.4827586	242.258621	11.276886	1.36339264	54.8167935	40.2061678
SP7	9.06896552	97.2931034	10.7281369	0.58614381	23.0162415	39.2672261
SP8	863.327586	5414.75862	6.27196293	60.5085165	1381.63145	22.8336693
SP9	1098.81034	5339.25862	4.85912664	70.6115695	1251.28428	17.7206694
SPDEF	23.1034483	192.965517	8.35223881	1.30604937	39.363909	30.1396791
SPI1	1.13793103	7.9137931	6.95454545	0.0749719	1.8481687	24.6514868
SPIB	2.43103448	19.4137931	7.9858156	0.14565007	4.24696277	29.1586736
SPIC	0	0		0	0	
SPZ1	0.0862069	0.48275862	5.6	0.00472395	0.1147943	24.3005018
SREBF1	1979.62069	11458.8276	5.78839555	116.937957	2453.55874	20.9817137
SRF	1493.18966	10720.9828	7.17992033	90.2396551	2388.10199	26.4639973
SRY	0	0		0	0	
ST18	445.948276	3994.84483	8.95809008	27.4396972	877.058763	31.9631357
STAG1	729.603448	5111.25862	7.00552969	48.3147699	1234.68792	25.5550823
STAG2	1578.87931	12377.5345	7.83944308	101.784166	2925.25564	28.7397908
STAT1	757.551724	6055.84483	7.99396877	49.6525214	1456.84912	29.3408889
STAT2	1458.01724	10323.4138	7.08044699	88.3383973	2241.29902	25.3717419

STAT3	1809.75862	12850.8793	7.10088029	114.995289	2990.72325	26.0073545
STAT4	41.1896552	361.965517	8.78777731	2.67106485	85.2798279	31.9272772
STAT5A	31.6551724	316.086207	9.98529412	2.00967889	72.691326	36.1706173
STAT5B	1562.74138	13728.8793	8.78512561	93.3366356	2964.55578	31.7619738
STAT6	64.362069	601.87931	9.35145995	4.31460683	146.868979	34.0399449
STK16	641.327586	5243.2069	8.17555179	39.7221245	1183.98459	29.8066784
SUB1	2069.18966	10354.7759	5.0042662	140.151703	2592.44103	18.4973924
SUPT20H	1188.94828	7139.22414	6.00465494	73.234522	1588.33565	21.688346
SUZ12	729.758621	5609.53448	7.68683551	47.0563062	1316.89181	27.985448
T	0.29310345	1.4137931	4.82352941	0.01782907	0.30077357	16.8698409
TAL1	8.39655172	82.3275862	9.80492813	0.67686117	24.525613	36.2343332
TBPL1	397.258621	3103.06897	7.81120611	26.2051683	756.465282	28.8670263
TBR1	1568.03448	11078.6379	7.06530249	97.6363352	2483.66818	25.4379496
TBX1	11.2241379	79.0689655	7.04454685	0.69184997	17.5214547	25.3255118
TBX10	13.4655172	87.2241379	6.47759283	0.79029829	18.0029475	22.7799399
TBX15	3.46551724	41.9137931	12.0945274	0.22406526	9.73349021	43.4404261
TBX18	5.36206897	52.7931034	9.84565916	0.27200411	8.79632834	32.3389535
TBX19	41	472.827586	11.5323802	2.41553053	98.2144415	40.6595736
TBX2	3.93103448	29.8275862	7.5877193	0.22649631	6.23927158	27.5469016
TBX20	0.10344828	1.06896552	10.3333333	0.00556261	0.2387624	42.9227414
TBX21	1.39655172	18.3965517	13.1728395	0.08650159	4.42146358	51.1142487
TBX22	0.22413793	1.77586207	7.92307692	0.01371579	0.35641284	25.9855776
TBX4	1.27586207	12.2413793	9.59459459	0.0719427	2.63903425	36.6824488
TBX5	5.55172414	50.362069	9.07142857	0.32562691	10.8978321	33.4672344
TBX6	37.4310345	359.103448	9.59373561	2.24293687	76.5359134	34.1230796
TCF12	1902	15579.0862	8.19089706	124.262771	3722.77502	29.9588926
TCF15	12.5517241	101.827586	8.11263736	0.78191045	22.6183955	28.9270919
TCF20	2232.81034	20686.0862	9.26459619	139.500327	4656.76383	33.3817413
TCF21	0.05172414	0.82758621	16	0.00403552	0.18809307	46.6093557
TCF3	4997.15517	26371.7759	5.27735781	296.088113	5700.10215	19.2513712
TCF7	63.8965517	629.293103	9.84862385	4.03942396	146.949265	36.3787674
TCF7L1	560.068966	5460.41379	9.74953823	31.6459504	1113.70768	35.1927391
TCF7L2	958	7719.81034	8.05825714	60.4481641	1720.99148	28.4705335
TCFL5	501.413793	2335.25862	4.65734819	31.1837938	529.959501	16.994709
TEAD1	1232.44828	12342.8966	10.0149408	78.5074832	2873.27869	36.5987874
TEAD2	952.12069	5232.41379	5.49553628	59.5206224	1197.44631	20.1181752
TEAD4	69.4137931	554.741379	7.99180328	4.25465173	124.953806	29.3687507
TEF	571.206897	5967.96552	10.4479928	35.7529768	1362.1257	38.0982459
TFAM	601.5	4710.41379	7.83111188	40.1112089	1153.81009	28.7652784
TFAP2A	32.6034483	302.775862	9.28662084	1.765238	55.9218211	31.6794796
TFAP2B	38.5517241	445.931034	11.5670841	2.44056678	99.4719596	40.7577291
TFAP2C	200.12069	1755.2931	8.77117257	12.3976735	389.719449	31.4348857
TFAP2D	3.24137931	36.0862069	11.1329787	0.26247161	10.8501223	41.3382694
TFAP2E	134.655172	1033.51724	7.67528809	7.59586217	210.00168	27.6468524
TFAP4	339.017241	2638.2069	7.78192544	20.4292374	574.092462	28.1015122
TFCP2	762.534483	6091.75862	7.98883035	48.6033698	1407.49526	28.9587999
TFCP2L1	5.13793103	55.6034483	10.8221477	0.35508725	14.3610757	40.4437944
TFDP1	1517.13793	9598.75862	6.32688592	98.5870312	2289.92039	23.2273999
TFDP2	1308.98276	11461.2241	8.75582513	79.4787954	2502.52734	31.4867296
TFDP3	0.24137931	1.72413793	7.14285714	0.01573552	0.40284213	25.6008186
TFE3	1698.06897	12748.7931	7.50781821	104.477575	2896.95043	27.727964
TFEB	95.6551724	756.206897	7.90555155	5.84142089	169.229801	28.970657

TFEC	0.32758621	3.68965517	11.2631579	0.02234434	0.75002791	33.5667932
TGFB1I1	185.568966	1572.75862	8.47533216	11.9286473	372.691924	31.2434357
TGIF1	476.103448	4634.24138	9.73368581	30.6884305	1094.20278	35.6552211
TGIF2LX	0	0		0	0	
THAP11	975.241379	4906.53448	5.03109752	62.1032099	1146.9316	18.4681532
THRA	6708.98276	28044.4138	4.18012906	423.609744	6481.9144	15.3016178
THRB	98.9310345	1122.32759	11.3445451	6.97451574	278.580069	39.9425679
TLE4	1584.56897	11018.0345	6.95333225	101.629225	2572.52751	25.3128715
TLX1	1.93103448	19.4482759	10.0714286	0.1089312	3.94846494	36.2473281
TLX2	3.39655172	23.4137931	6.89340102	0.19042248	4.59471178	24.1290403
TOX2	634.793103	5725.41379	9.01933837	37.6022275	1215.67893	32.3299711
TOX3	932.068966	6860.93103	7.36096929	56.3953176	1508.73495	26.7528408
TP53	907.12069	6979.22414	7.69382092	54.6578633	1532.02653	28.0293893
TP63	1.22413793	14.3275862	11.7042254	0.0888155	3.68426289	41.4822072
TP73	96.2413793	938.775862	9.75438911	5.47846309	189.308057	34.5549571
TRPS1	285.655172	3163.43103	11.0742999	18.0495043	718.47827	39.8059835
TSHZ1	1296.03448	11997.7759	9.25729679	84.1596201	2809.42446	33.3820953
TSHZ2	410.189655	4483.55172	10.9304359	24.5136386	957.121761	39.0444592
TSHZ3	294.224138	3348.98276	11.3824202	17.9661673	730.396185	40.6539787
TULP1	2.10344828	17.9655172	8.54098361	0.13141845	4.16690549	31.7071585
TULP2	1.05172414	9.18965517	8.73770492	0.06385098	2.13975949	33.5117709
TULP4	1110.08621	8485.75862	7.64423391	71.2174006	1946.43987	27.3309592
TWIST1	5.9137931	44.2241379	7.47813411	0.34626106	9.18812212	26.5352449
TXK	0.48275862	3.94827586	8.17857143	0.03049763	0.95335272	31.2598952
UBP1	1525.12069	10427.3448	6.83706208	94.0391399	2340.81066	24.8918765
USF1	1755.39655	10173.5345	5.7955762	106.245449	2231.31028	21.001467
USF2	3476.93103	14125.1552	4.06253533	212.645352	3161.82986	14.8690288
VAX1	329.844828	2499.18966	7.57686477	19.9905427	561.355068	28.0810319
VAX2	151.948276	1296.51724	8.53262226	8.93618064	275.773821	30.8603678
VDR	7.5	85.137931	11.3517241	0.46284361	19.4094239	41.935167
VENTX	0.82758621	8.24137931	9.95833333	0.05135399	1.76826399	34.4328461
VEZF1	1458.96552	12187.7069	8.35366344	94.009446	2871.60926	30.5459651
VSX2	5.03448276	62.7068966	12.4554795	0.31751737	14.5239017	45.7420696
WT1	2.60344828	24.4137931	9.37748344	0.16067831	5.63743953	35.0852559
XBP1	881.655172	6097.84483	6.91636029	56.9345807	1469.46752	25.809754
YBX3	735.948276	4494.58621	6.10720394	45.4737001	1044.48003	22.9688816
YLPM1	2415.77586	20973.9828	8.68208971	151.277226	4725.32727	31.2362104
YY1	1853.65517	10013	5.4017598	116.000777	2306.42193	19.8828145
YY2	28.7758621	317.689655	11.0401438	1.78872483	72.3364871	40.4402544
ZBED1	1659.27586	12751	7.68467757	106.851584	3010.31326	28.1728464
ZBTB1	461.051724	4031.10345	8.74327811	29.7723984	951.123236	31.9464768
ZBTB10	819.103448	7188.7069	8.77631136	51.522071	1659.09326	32.2016026
ZBTB14	435.534483	4393.86207	10.0884367	28.2745681	1037.83396	36.7055637
ZBTB16	161.327586	1389.93103	8.61558192	9.46935243	288.939275	30.5130976
ZBTB17	1470.43103	10827.2931	7.36334643	88.0312698	2327.04982	26.4343548
ZBTB18	2758.7069	15554.1034	5.63818631	169.438763	3460.48283	20.4232064
ZBTB2	283.706897	2978.44828	10.4983288	18.1161706	700.252925	38.6534738
ZBTB20	509.189655	608.137931	1.19432499	33.4128766	136.036543	4.07138076
ZBTB21	446.5	4949.13793	11.0842955	28.9478521	1171.15655	40.4574595
ZBTB33	769.5	7134.15517	9.27115682	51.7175615	1756.06997	33.9550032
ZBTB4	1694.12069	15012	8.86123409	106.244397	3426.81755	32.2541014
ZBTB42	65.7758621	725.913793	11.036173	4.1368646	169.691948	41.019459

ZBTB45	662.62069	5075.81034	7.66020504	41.5411178	1157.86674	27.8727873
ZBTB5	1564.7931	13113.5345	8.38036316	99.0271137	3042.07073	30.7195739
ZBTB7A	404.241379	3322.91379	8.22012284	24.6250714	728.85402	29.598047
ZBTB7B	222.913793	1990.7931	8.93077578	13.3117412	434.849702	32.6666283
ZC3H8	149.741379	1127.03448	7.52654001	9.47130766	256.371139	27.0681883
ZEB1	1233.81034	11064.2931	8.96758011	76.8132494	2495.70881	32.4906032
ZFAND3	2487.03448	15843.8103	6.3705632	159.178179	3735.71716	23.4687768
ZFAT	159.758621	1495.37931	9.36024174	10.0220956	340.609764	33.9858825
ZFP1	665.896552	6011.58621	9.02780799	43.8913912	1446.62679	32.9592376
ZFP37	272.775862	2442.81034	8.95537577	17.6035512	572.415279	32.5170343
ZFP42	0.03448276	0.22413793	6.5	0.00186858	0.0699887	37.4555044
ZFP69	125.896552	1166.41379	9.26485894	8.26745854	277.49742	33.5650211
ZFP90	909.965517	7722.81034	8.48692637	58.4118879	1795.62531	30.7407512
ZFX	570.965517	5644.68966	9.88621814	36.8410118	1326.22367	35.998568
ZFY	0.01724138	0.13793103	8	0.00129183	0.03295039	25.506798
ZGPAT	710.603448	4888.17241	6.87890331	41.6899656	1037.61618	24.8888712
ZHX2	427.431034	4991.24138	11.6773022	26.3567949	1106.17698	41.9693285
ZHX3	986.172414	9709.84483	9.84599112	62.4221905	2222.4996	35.6043193
ZIC1	1847.03448	11640.0172	6.30200321	116.0413	2753.29327	23.7268393
ZIC2	1766.62069	10178.7931	5.76173095	104.920136	2226.844	21.2241815
ZIC3	748.551724	6099.17241	8.14796388	44.7332552	1334.38788	29.8298856
ZIC4	365.396552	3700.36207	10.1269759	22.5486585	835.086091	37.0348458
ZIC5	343.637931	2821.12069	8.20957303	20.5393953	609.29195	29.6645514
ZKSCAN3	237.62069	1996.2069	8.40081265	14.8478541	450.224626	30.3225383
ZKSCAN5	485.862069	5028.75862	10.3501774	30.7985697	1154.78166	37.4946521
ZMYND8	1531.98276	13221.1034	8.63006021	94.5442344	2926.64971	30.9553483
ZNF10	301.862069	3014	9.98469271	19.8513	722.470321	36.3941062
ZNF117	749.189655	2938.82759	3.92267507	44.3221989	629.516806	14.2031944
ZNF124	132.310345	913.948276	6.90761011	8.68107452	219.054459	25.2335651
ZNF131	510.155172	4431.12069	8.68582919	33.0317173	1043.51474	31.5912953
ZNF132	89.8793103	951.068966	10.5816229	5.7860374	221.255638	38.2395796
ZNF133	473.206897	4265.46552	9.01395467	28.9985666	938.046981	32.3480465
ZNF134	456.672414	4080.7931	8.93593083	28.8703662	939.771103	32.5514092
ZNF135	292.051724	2551.12069	8.73516737	18.5324844	584.165719	31.5211769
ZNF136	228.224138	1671.12069	7.32227846	14.9342303	397.305552	26.6036845
ZNF138	329.258621	1773.37931	5.38597686	20.8456505	404.94419	19.4258361
ZNF140	331.827586	2314.60345	6.97531955	22.0002653	558.484889	25.3853706
ZNF143	388.275862	3287.62069	8.46722913	24.4909402	751.319256	30.6774362
ZNF148	896.724138	8728.7069	9.73399346	59.532873	2110.72714	35.4548175
ZNF154	495.413793	3516.56897	7.09824598	31.9284374	822.43712	25.7587651
ZNF155	93.7586207	779.62069	8.31518941	5.99122149	179.769695	30.0055164
ZNF157	45.7413793	390.568966	8.53863551	2.83645212	87.1901909	30.7391725
ZNF165	30.0517241	212.344828	7.0659782	1.89385238	49.4278866	26.0991233
ZNF174	312.568966	3398.22414	10.8719179	19.7673362	776.336578	39.2737075
ZNF175	216.448276	2121.36207	9.80078063	13.8835871	490.775216	35.3493095
ZNF177	0.4137931	1.48275862	3.58333333	0.02492369	0.32184388	12.9131735
ZNF18	220.465517	2272.60345	10.3082036	13.8962609	518.223871	37.2923245
ZNF189	355.724138	3832.7069	10.7743796	23.650786	926.804634	39.1870542
ZNF19	95.5	1001.03448	10.4820365	5.96993979	228.502636	38.2755345
ZNF202	388.12069	4097.43103	10.5571054	24.6062549	933.734741	37.9470481
ZNF205	572.655172	3819.53448	6.66986813	34.9752817	840.060386	24.0186882
ZNF214	67.637931	734.913793	10.8654091	4.32647115	169.41479	39.1577302

ZNF215	30.7068966	342.758621	11.1622684	1.97665154	79.7940521	40.3682948
ZNF217	453.155172	5008.32759	11.0521249	28.4526916	1142.81127	40.1653132
ZNF219	2703.7069	12520.9138	4.63101744	159.610856	2679.9734	16.7906712
ZNF22	697.655172	2597	3.72246936	46.5297076	635.885134	13.6662181
ZNF224	56.8275862	480.724138	8.45934466	3.35707829	100.885645	30.0516213
ZNF23	512.293103	4302.82759	8.39915189	31.734907	958.486295	30.2029023
ZNF230	104.862069	921.758621	8.79020059	6.93199005	223.350304	32.2202286
ZNF239	98.0344828	1091	11.1287372	6.6460549	266.958883	40.1680226
ZNF24	1801.39655	15999.8793	8.88193069	116.39751	3791.10946	32.5703657
ZNF250	579.12069	5139.75862	8.87510792	36.9337502	1182.58902	32.0191969
ZNF251	677	5928.2931	8.75671064	40.6490807	1284.32981	31.5955439
ZNF252P	223.241379	2018.58621	9.04216867	15.2602157	505.307379	33.1127285
ZNF256	164.362069	1612.98276	9.81359488	10.0036857	352.023756	35.1894057
ZNF263	948.155172	8836.93103	9.32013165	58.9734735	2003.65817	33.9755834
ZNF267	81.7758621	606.258621	7.41366224	5.27341914	143.763283	27.2618731
ZNF268	359.051724	3512.5	9.78271309	23.590023	839.638969	35.5929696
ZNF274	734.534483	6524	8.88181583	45.6968998	1467.33495	32.1101641
ZNF281	648.034483	6665.56897	10.2858245	42.091149	1570.43308	37.3102925
ZNF284	65.3275862	566.448276	8.67088942	4.11110552	127.394579	30.9879127
ZNF300	944.793103	6442.65517	6.81911749	58.8419978	1452.24151	24.6803569
ZNF302	787.62069	5032.87931	6.38997855	50.6853915	1178.28112	23.246957
ZNF304	477.413793	4733.36207	9.9145901	30.9276962	1112.59344	35.974016
ZNF317	867.672414	8767.34483	10.1044411	55.2480286	2023.10817	36.618649
ZNF322	316.12069	2438.65517	7.71431688	20.5906909	575.299773	27.9397994
ZNF326	486.362069	3219.24138	6.6190223	29.4804498	707.667797	24.0046472
ZNF333	761.37931	5928.98276	7.78716033	44.9353418	1260.0658	28.0417541
ZNF34	257.862069	2331.15517	9.04031827	15.9584457	519.487454	32.5525094
ZNF35	153.672414	1511.77586	9.83765287	9.98569001	356.103019	35.6613332
ZNF350	138.965517	1201.17241	8.64367246	8.83504255	276.399945	31.2845064
ZNF354A	320.034483	2689.32759	8.40324319	20.4628645	627.83458	30.6816565
ZNF354C	267.810345	2317.75862	8.65447756	17.4128113	545.819201	31.3458402
ZNF367	190.551724	1646.39655	8.64015563	11.9218226	377.557766	31.6694669
ZNF37A	350.758621	2497.93103	7.12151003	22.2891641	571.226931	25.6280105
ZNF382	274.982759	2740.24138	9.96513888	16.6185065	587.584973	35.3572671
ZNF384	1417.91379	11275.3621	7.9520787	85.2680191	2460.69907	28.8584055
ZNF395	1014.31034	8998.03448	8.87108618	62.6739732	2027.36495	32.3477967
ZNF41	157.672414	1691.48276	10.7278294	10.5568603	411.21914	38.9527879
ZNF423	1142.77586	10258.8276	8.9771126	68.2447174	2198.51273	32.2151342
ZNF429	141.086207	551.327586	3.90773555	9.20532769	130.22901	14.1471347
ZNF43	340.551724	1216.12069	3.57103078	21.5601593	279.789568	12.9771568
ZNF431	185.103448	514.103448	2.7773845	11.4512058	114.571749	10.0052127
ZNF433	183.310345	1267.53448	6.9146915	11.4527625	287.67895	25.1187389
ZNF45	352.637931	3172.56897	8.9966753	22.8447456	742.486971	32.5014331
ZNF467	590	4750.67241	8.05198714	35.1289618	1021.66136	29.083164
ZNF468	167.706897	739.224138	4.40783386	11.2259658	182.908284	16.2933228
ZNF470	271.741379	2424.84483	8.92335512	17.6764157	572.141616	32.367513
ZNF492	0.79310345	3.94827586	4.97826087	0.04923058	0.79275656	16.1029292
ZNF496	943.87931	7690.34483	8.14759339	56.7276478	1667.01454	29.38628
ZNF512B	3238.12069	22870.0172	7.06274393	192.271549	4890.18161	25.4337246
ZNF513	732.896552	5296.77586	7.22718077	44.822045	1166.57441	26.0268001
ZNF517	541.155172	3152.58621	5.82566031	32.9427694	690.574198	20.9628459
ZNF536	652.965517	6318.36207	9.67641001	39.9937749	1388.40366	34.7154941

ZNF580	1151.63793	6630.67241	5.75760162	69.9767262	1469.45784	20.9992368
ZNF589	578.448276	6091.01724	10.5299255	34.9533536	1322.88243	37.8470819
ZNF592	1231.98276	12616.4655	10.2407809	76.3947806	2820.62831	36.921741
ZNF639	625.103448	3762.32759	6.01872793	40.729818	914.262676	22.4470111
ZNF652	511.258621	4374.25862	8.55586281	32.4050119	1008.46265	31.1205765
ZNF668	535.206897	4458.72414	8.33084208	32.6578205	986.238621	30.1991562
ZNF674	68.0517241	696.931034	10.2411958	4.36935287	161.00522	36.8487564
ZNF687	1015.62069	9384.25862	9.23992463	61.2192817	2047.24484	33.4411771
ZNF69	50.7758621	257.362069	5.06859083	3.19131487	58.0796407	18.1992824
ZNF692	1472.36207	10514.931	7.14153893	87.210461	2224.85686	25.511353
ZNF7	364.948276	2431.46552	6.6624935	22.7339232	549.182083	24.1569429
ZNF705D	0.01724138	0.03448276	2	0.001099	0.00553919	5.04019161
ZNF711	1427.13793	10804.2759	7.5705898	86.5385809	2375.90917	27.4549126
ZNF720	111.758621	796.068966	7.12311015	6.90574222	178.545937	25.8547063
ZNF746	777.137931	6397.65517	8.23232906	48.3352415	1437.63764	29.7430527
ZNF750	1.68965517	14.4482759	8.55102041	0.09581688	3.19444569	33.3390702
ZNF76	886.172414	6691.32759	7.5508191	52.2946885	1421.79176	27.1880722
ZNF8	353.603448	3169.44828	8.96328441	21.4299206	689.862398	32.1915517
ZNF80	1.24137931	10.3965517	8.375	0.07061062	2.21178195	31.3236458
ZNF804A	313	2968.93103	9.48540267	21.642673	749.24659	34.6189489
ZNF821	1145.65517	7978.62069	6.96424272	71.5727204	1812.05388	25.3176612
ZNF84	1578.62069	12325.8966	7.8080166	101.272206	2875.10909	28.3899126
ZNF85	130.810345	488.672414	3.73573217	8.67764037	119.748234	13.7996309
ZNF92	324.775862	1538.51724	4.73716622	21.7308513	378.007708	17.3949793
ZNF93	164.62069	792.206897	4.81231672	10.9265767	191.958632	17.5680487
ZNF98	0.03448276	0.06896552	2	0.00163149	0.01417974	8.69130503
ZNRD1	333.482759	2977.46552	8.92839417	20.9020219	686.108353	32.8249753
ZSCAN10	9.10344828	68.137931	7.48484848	0.50943058	13.7793768	27.0485859
ZSCAN12	344.5	3079.39655	8.9387418	21.8319524	705.171998	32.299997
ZSCAN21	171.87931	1387.32759	8.07152172	10.7343535	314.595893	29.3073908
ZSCAN26	498.706897	4602.2069	9.22828003	32.014175	1070.20027	33.4289506
ZSCAN31	79.8965517	827.482759	10.3569271	5.26552626	197.0283	37.418539
ZSCAN4	0.70689655	7.96551724	11.2682927	0.04598189	1.83859821	39.9852681
ZSCAN9	364.172414	3249.7069	8.92353944	22.8449683	737.834422	32.2974588

Enrichment and variability

Sample	CV (enrichment factor (EF), all targetted genes)	CV EF (above expression threshold)	CV EF (below expression threshold)	Mean enrichment (above expression threshold)	Std Dev enrichment (above expression threshold)	Mean enrichment (below expression threshold)	Std Dev enrichment (below expression threshold)
NG Mini-bulk	41.98	28.9	49.72	185.3	53.56	352.2	175.1
NG Bulk	34.95	33.2	53.02	179.4	59.56	198.8	105.4
TF mini 150	44.37	30.71	77.63	40.33	12.39	55.56	43.13
TF mini 850	44.43	30.99	76.07	41.3	12.8	57.68	43.87
TF bulk	33.95	28.19	63.92	30.77	8.673	48.25	30.84
