# Supplementary material for: Targeted RNA sequencing enhances gene expression profiling of ultra-low input samples
Source: RNA Biol. 2020 Jun 28;17(12):1741–53. doi: 10.1080/15476286.2020.1777768 (PMC7746246; doi:10.1080/15476286.2020.1777768)
Supplement: Supplemental Material [file KRNB_A_1777768_SM6200.zip › TableS9_ERCC_expression_summary.pdf]

NG_Capture

ERCC	concentration_in_Mix_1_(attomoles/ul)	Pre-cap CPKM	227256	227280	228256	228280	255256	255280	256256	256280	Post-cap CPKM	227256	227280	228256	228280	255256	255280	256256	256280
ERCC-00048	0.014305	Pre-cap	0	0	0	0	0	0	0	0	Post-cap	0	0	0	0	0	0	0	0
ERCC-00057	0.014305	Pre-cap	0	0	0	0	0	0	0	0	Post-cap	0	0	0	0	0	0	0	0
ERCC-00083	0.02861	Pre-cap	0	0	0	0	0	0	0	0	Post-cap	0	0	0	0	0	0	0	0
ERCC-00098	0.05722	Pre-cap	0	0	0	0	0	0	0	0	Post-cap	0	0	0	0	0	0	0	0
ERCC-00117	0.05722	Pre-cap	0	0	0	0	0	0	0	0	Post-cap	0	0	0	0	0	0	0	0
ERCC-00012	0.114441	Pre-cap	0	0	0	0	0	0	0	0	Post-cap	0	0	0	0	0	0	0	0
ERCC-00086	0.114441	Pre-cap	0	0	0	0	0	0	0	0	Post-cap	0	0	0	0	0	0	0	0
ERCC-00138	0.114441	Pre-cap	0	0	0	0	0	0	0	0	Post-cap	0	0	0	0	0	0	0	4.3964
ERCC-00024	0.228882	Pre-cap	0	0	0	0	0	0	0	0	Post-cap	0	0	0	0	0	0	0	0
ERCC-00041	0.228882	Pre-cap	0	0	0	0	0	0	0	0	Post-cap	0	0	0	0	0	0	0	0
ERCC-00104	0.228882	Pre-cap	0	0	0	0	0	0	0	0	Post-cap	0	0	0	0	0	0	0	0
ERCC-00123	0.228882	Pre-cap	0	0	0	0	0	0	0	0	Post-cap	0	0	0	0	0	0	0	0
ERCC-00156	0.457764	Pre-cap	0	0	0	0	0	0	0	0	Post-cap	0	0	0	0	0	0	0	0
ERCC-00158	0.457764	Pre-cap	0	0	0	0	0	0	0	0	Post-cap	0	0	0	0	0	0	0	0
ERCC-00164	0.457764	Pre-cap	0	0	0	0	0	0	0	0	Post-cap	0	0	0	0	0	0	0	0
ERCC-00168	0.457764	Pre-cap	0	0	0	0	0	0	0	0	Post-cap	0	0	0	0	0	0	0	0
ERCC-00109	0.915527	Pre-cap	0	0	0	0	0	0	0	0	Post-cap	0	0	0	0	0	0	0	0
ERCC-00120	0.915527	Pre-cap	0	0	0	0	0	0	0	0	Post-cap	30.216	0	6.1733	0	16.617	0	0	0
ERCC-00137	0.915527	Pre-cap	0	0	0	0	0	0	0	0	Post-cap	0	0	0	0	0	0	0	0
ERCC-00147	0.915527	Pre-cap	0	0	0	0	0	0	0	0	Post-cap	0	0	0	0	0	0	0	0
ERCC-00031	1.831055	Pre-cap	0	0	0	0	0	0	0	0	Post-cap	0	0	0	0	0	0	0	0
ERCC-00058	1.831055	Pre-cap	0	0	0	0	0	0	0	0	Post-cap	0	0	2.9128	0	3.9203	0	3.68545	0
ERCC-00069	1.831055	Pre-cap	0	0	0	0	0	0	0	0	Post-cap	0	0	0	0	0	0	0	0
ERCC-00134	1.831055	Pre-cap	0	0	0	0	0	0	0	0	Post-cap	0	0	0	0	0	0	0	0
ERCC-00014	3.662109	Pre-cap	0	0	0	0	0	0	0	0	Post-cap	0	0	0	0	0	0	0	0
ERCC-00028	3.662109	Pre-cap	0	0	0	0	0	0	0	0	Post-cap	0	0	0	0	0	0	0	0
ERCC-00143	3.662109	Pre-cap	0	0	0	0	0	0	0	0	Post-cap	0	0	0	0	0	0	0	5.7422
ERCC-00150	3.662109	Pre-cap	0	0	0	0	0	0	0	0	Post-cap	0	0	0	0	0	0	0	0
ERCC-00034	7.324219	Pre-cap	0	0	0	0	0	0	0	0	Post-cap	7.9469	0	0	0	0	0	4.10861	4.4179
ERCC-00085	7.324219	Pre-cap	0	0	0	0	0	0	0	0	Post-cap	0	0	7.841	14.576	0	0	14.8815	5.334
ERCC-00157	7.324219	Pre-cap	0	0	0	0	0	0	0	0	Post-cap	0	0	0	0	0	0	0	0
ERCC-00160	7.324219	Pre-cap	0	0	0	0	0	10.392	0	0	Post-cap	10.899	0	4.4534	0	17.981	815.97	11.2697	0
ERCC-00059	14.64844	Pre-cap	0	0	0	4.1572	0	0	0	0	Post-cap	0	0	0	203.08	0	9.3885	0	8.575
ERCC-00099	14.64844	Pre-cap	0	0	0	0	0	0	0	0	Post-cap	0	2.9866	0	0	0	0	6.20248	3.3347
ERCC-00148	14.64844	Pre-cap	0	0	0	0	0	0	0	0	Post-cap	0	0	0	0	0	0	0	0
ERCC-00170	14.64844	Pre-cap	0	0	0	0	0	0	0	0	Post-cap	87.074	0	0	0	0	0	0	0
ERCC-00019	29.29688	Pre-cap	0	0	0	0	0	0	0	0	Post-cap	50.298	12.521	25.69	25.47	41.491	0	19.5031	0
ERCC-00078	29.29688	Pre-cap	0	0	0	0	0	0	0	0	Post-cap	12.233	24.362	0	8.2593	8.9696	9.9274	8.43238	9.0672
ERCC-00084	29.29688	Pre-cap	0	0	0	0	0	0	0	0	Post-cap	4.0734	16.225	19.973	37.13	4.4803	24.794	50.5434	31.703
ERCC-00144	29.29688	Pre-cap	0	0	0	0	0	0	0	0	Post-cap	0	7.4942	18.451	0	0	9.1617	7.78192	0
ERCC-00051	58.59375	Pre-cap	0	0	0	0	0	0	0	0	Post-cap	14.777	14.715	0	29.932	65.013	611.62	0	16.43
ERCC-00071	58.59375	Pre-cap	0	0	0	0	0	4.0088	0	0	Post-cap	6.3068	56.522	0	25.55	0	46.065	6.5213	56.098
ERCC-00165	58.59375	Pre-cap	0	0	0	0	0	0	0	0	Post-cap	23.217	13.871	22.768	9.4054	25.536	45.22	9.60247	5.1627
ERCC-00035	117.1875	Pre-cap	1.6357	0	0	0	0	0	0	0	Post-cap	318.9	71.361	20.498	105.24	11.823	82.877	14.8201	59.759
ERCC-00044	117.1875	Pre-cap	0	0	0	0	0	0	0	0	Post-cap	84.061	48.829	22.899	17.737	335.16	8.5276	97.7857	66.204
ERCC-00131	117.1875	Pre-cap	2.3974	0	8.4693	0	0	0	2.7867	0	Post-cap	236.32	26.147	386.25	340.4	57.761	31.965	32.5811	40.873
ERCC-00022	234.375	Pre-cap	0	0	4.3474	8.7185	0	0	0	0	Post-cap	26.957	85.899	352.48	890.04	302.43	85.322	44.5984	203.81
ERCC-00092	234.375	Pre-cap	0	0	2.9047	1.9418	0	0	0	0	Post-cap	93.659	71.742	394.48	47.429	43.583	96.475	33.5232	108.14
ERCC-00042	468.75	Pre-cap	0	0	1.5958	8.5338	0	0	0	14.02	Post-cap	91.032	130.06	300.81	304.65	104.48	115.64	454.273	2129.9

ERCC-00043	468.75	Pre-cap	0	0	0	4.2669	0	0	4.2005	2.0029	Post-cap	19.79	35.471	29.111	188.4	52.239	679.36	290.571	268.44
ERCC-00111	468.75	Pre-cap	0	0	1.6423	0	0	0	0	0	Post-cap	150.72	328.55	109.85	165.02	125.45	138.84	197.962	158.52
ERCC-00108	937.5	Pre-cap	7.2343	10.637	3.1947	0	4.154	12.591	23.125	24.058	Post-cap	241.67	299.83	184.55	144.45	296.31	607.68	925.821	955.88
ERCC-00145	937.5	Pre-cap	0	2.0866	4.7	4.1891	0	14.819	2.0619	0	Post-cap	182.63	286.33	336.61	610	89.752	1669.8	96.4301	397.48
ERCC-00046	3750	Pre-cap	0	24.991	15.637	8.3622	12.199	34.512	20.58	51.027	Post-cap	721.37	957.77	963.51	832.72	1689.2	2275.6	1291.29	2647.7
ERCC-00171	3750	Pre-cap	14.641	17.221	12.93	43.218	33.627	71.348	8.5091	77.088	Post-cap	689.53	1477	1094.2	1526.6	1199.3	2684.1	986.563	4082.9
ERCC-00004	7500	Pre-cap	21.205	12.472	12.485	16.692	16.235	118.1	28.757	23.506	Post-cap	789.66	848.01	733.91	799.76	1898.9	4787.6	1488.95	1919.5

TF_Capture_150

ERCC	concentration_in_Mix_1_(attomoles/ul)	Pre-cap CPKM	227208	227232	228208	228232	255232	256232	Post-cap CPKM	227208	227232	228208	228232	255232	256232
ERCC-00048	0.014305	Pre-cap	0	0	0	0	0	0	Post-cap	0	0	0	0	0	0
ERCC-00057	0.014305	Pre-cap	0	0	0	0	0	0	Post-cap	0	0	0	0	0	0
ERCC-00083	0.02861	Pre-cap	0	0	0	0	0	0	Post-cap	0	0	0	0	0	0
ERCC-00098	0.05722	Pre-cap	0	0	0	0	0	0	Post-cap	0	4.6021	0	5.45227	8.3637	7.70555
ERCC-00117	0.05722	Pre-cap	0	0	0	0	0	0	Post-cap	0	0	0	0	0	0
ERCC-00012	0.114441	Pre-cap	0	0	0	0	0	0	Post-cap	0	0	0	0	0	0
ERCC-00086	0.114441	Pre-cap	0	0	0	0	0	0	Post-cap	0	0	0	0	0	0
ERCC-00138	0.114441	Pre-cap	0	0	0	0	0	0	Post-cap	0	0	0	0	0	0
ERCC-00024	0.228882	Pre-cap	0	0	0	0	0	0	Post-cap	0	0	0	0	0	0
ERCC-00041	0.228882	Pre-cap	0	0	0	0	0	0	Post-cap	0	0	0	0	0	0
ERCC-00104	0.228882	Pre-cap	0	0	0	0	0	0	Post-cap	0	0	0	0	0	0
ERCC-00123	0.228882	Pre-cap	0	0	0	0	0	0	Post-cap	0	0	0	0	0	0
ERCC-00156	0.457764	Pre-cap	0	0	0	0	0	0	Post-cap	0	0	0	0	0	0
ERCC-00158	0.457764	Pre-cap	0	0	0	0	0	0	Post-cap	0	0	0	0	0	0
ERCC-00164	0.457764	Pre-cap	0	0	0	0	0	0	Post-cap	0	0	0	0	0	0
ERCC-00168	0.457764	Pre-cap	0	0	0	0	0	0	Post-cap	0	0	0	0	0	0
ERCC-00109	0.915527	Pre-cap	0	0	0	0	0	0	Post-cap	0	0	0	0	0	0
ERCC-00120	0.915527	Pre-cap	0	0	0	0	0	0	Post-cap	0	0	0	0	0	0
ERCC-00137	0.915527	Pre-cap	0	0	0	0	0	0	Post-cap	0	0	0	0	0	0
ERCC-00147	0.915527	Pre-cap	0	0	0	0	0	0	Post-cap	0	0	0	0	0	0
ERCC-00031	1.831055	Pre-cap	0	0	0	0	0	0	Post-cap	0	0	0	0	0	0
ERCC-00058	1.831055	Pre-cap	0	0	0	0	0	0	Post-cap	0	0	0	0	0	0
ERCC-00069	1.831055	Pre-cap	0	0	0	0	0	0	Post-cap	0	0	0	0	0	0
ERCC-00134	1.831055	Pre-cap	0	0	0	0	0	0	Post-cap	0	0	0	0	0	0
ERCC-00014	3.662109	Pre-cap	0	0	0	0	0	0	Post-cap	0	0	0	1.06148	0	0
ERCC-00028	3.662109	Pre-cap	0	0	0	0	0	0	Post-cap	0	0	0	0	0	0
ERCC-00143	3.662109	Pre-cap	0	0	0	0	0	0	Post-cap	0	0	0	0	0	0
ERCC-00150	3.662109	Pre-cap	3.24391	0	0	0	0	0	Post-cap	0	7.0797	0	0	0	0
ERCC-00034	7.324219	Pre-cap	0	0	0	0	0	0	Post-cap	0	12.045	0	8.15432	9.38146	12.9648
ERCC-00085	7.324219	Pre-cap	0	0	0	0	0	0	Post-cap	0	0	1.94733	0	8.495	0
ERCC-00157	7.324219	Pre-cap	0	0	0	0	0	0	Post-cap	0	0	0	0	0	0
ERCC-00160	7.324219	Pre-cap	0	0	0	0	0	0	Post-cap	4.23344	2.3599	8.84818	5.59169	0	8.89043
ERCC-00059	14.64844	Pre-cap	0	0	0	0	0	0	Post-cap	2.99566	3.3398	12.5223	23.7407	9.10448	12.5821

ERCC-00099	14.64844	Pre-cap	0	0	0	0	0	0	Post-cap	0	0	0	0	0	0
ERCC-00148	14.64844	Pre-cap	0	0	0	0	0	0	Post-cap	0	0	3.32702	4.20509	0	4.45721
ERCC-00170	14.64844	Pre-cap	0	0	0	0	0	0	Post-cap	1.53736	3.428	0	0	7.00858	4.30472
ERCC-00019	29.29688	Pre-cap	0	0	0	0	0	0	Post-cap	0	8.1681	0	3.22564	25.9775	3.41904
ERCC-00078	29.29688	Pre-cap	0	0	2.25453	0	0	0	Post-cap	6.33524	5.2973	302.89	12.5517	4.81355	13.3043
ERCC-00084	29.29688	Pre-cap	0	0	0	0	0	0	Post-cap	4.74665	15.876	4.96041	6.26956	9.61741	6.64546
ERCC-00144	29.29688	Pre-cap	0	0	0	0	0	0	Post-cap	2.92328	6.5183	3.05493	223.948	8.88448	8.18536
ERCC-00051	58.59375	Pre-cap	0	0	0	0	0	0	Post-cap	17.2196	0	0	7.58144	8.72236	0
ERCC-00071	58.59375	Pre-cap	0	0	0	0	0	0	Post-cap	7.34917	13.656	7.68014	35.5926	22.3358	54.8751
ERCC-00165	58.59375	Pre-cap	16.5841	0	0	0	0	0	Post-cap	625.843	4.0216	13.1936	2.38224	8.22222	20.2006
ERCC-00035	117.1875	Pre-cap	0	0	0	12.7218	0	0	Post-cap	8.35074	21.724	0	536.793	25.3797	21.4341
ERCC-00044	117.1875	Pre-cap	0	0	1.93663	0	0	0	Post-cap	12.2444	19.718	55.4485	30.5487	41.3482	24.7614
ERCC-00131	117.1875	Pre-cap	3.1261	0	0	0	0	0	Post-cap	57.1157	25.016	27.7123	32.3317	58.8957	37.1261
ERCC-00022	234.375	Pre-cap	0	3.65393	0	0	0	0	Post-cap	23.0359	312.86	15.3194	58.0873	50.9172	76.2296
ERCC-00092	234.375	Pre-cap	0	0	15.9342	0	0	0	Post-cap	194.492	60.839	305.607	57.2925	63.7881	117.537
ERCC-00042	468.75	Pre-cap	4.71207	0	0	0	0	0	Post-cap	221.38	54.848	205.645	292.408	161.197	187.255
ERCC-00043	468.75	Pre-cap	0	0	0	0	3.67493	0	Post-cap	72.2561	51.42	41.7716	32.4898	67.7496	129.141
ERCC-00111	468.75	Pre-cap	16.9734	0	4.50453	0	3.78215	3.03404	Post-cap	716.744	89.964	195.11	100.313	259.67	159.491
ERCC-00108	937.5	Pre-cap	18.8667	18.7952	32.8584	25.3192	3.67853	11.8037	Post-cap	450.888	423.77	808.91	811.006	465.357	312.397
ERCC-00145	937.5	Pre-cap	2.31308	0	6.44554	0	0	0	Post-cap	46.7893	79.089	194.008	105.66	91.7438	63.3933
ERCC-00046	3750	Pre-cap	27.7037	5.2569	4.28879	5.50792	21.6061	23.1099	Post-cap	1027.39	571.03	755.655	449.687	883.632	839.407
ERCC-00171	3750	Pre-cap	42.9545	21.7355	48.7648	39.8533	29.7779	0	Post-cap	1202.12	958.3	1685.86	867.947	1022.23	754.301
ERCC-00004	7500	Pre-cap	55.3015	15.7405	25.6835	32.9843	28.753	0	Post-cap	1290.05	750.98	848.487	599.76	987.044	842.012

TF_Capture_850

ERCC	concentration_in_Mix_1_(attomoles/ul)	Pre-cap CPKM	227208	227232	228208	228232	255232	256232	Post-cap CPKM	227208	227232	228208	228232	255232	256232
ERCC-00048	0.014305	Pre-cap	0	0	0	0	0	0	Post-cap	0	0	0	0	0	0
ERCC-00057	0.014305	Pre-cap	0	0	0	0	0	0	Post-cap	0	0	0	0	0	0
ERCC-00083	0.02861	Pre-cap	0	0	0	0	0	0	Post-cap	0	0	0	0	0	0
ERCC-00098	0.05722	Pre-cap	0	0	0	0	0	0	Post-cap	0	9.23771	0	9.43753	5.51228	10.18
ERCC-00117	0.05722	Pre-cap	0	0	0	0	0	0	Post-cap	0	0	0	0	0	0
ERCC-00012	0.114441	Pre-cap	0	0	0	0	0	0	Post-cap	0	0	0	0	0	0
ERCC-00086	0.114441	Pre-cap	0	0	0	0	0	0	Post-cap	0	0	0	0	0	0
ERCC-00138	0.114441	Pre-cap	0	0	0	0	0	0	Post-cap	0	0	0	0	0	0
ERCC-00024	0.228882	Pre-cap	0	0	0	0	0	0	Post-cap	0	0	0	0	0	0
ERCC-00041	0.228882	Pre-cap	0	0	0	0	0	0	Post-cap	0	0	0	0	0	0
ERCC-00104	0.228882	Pre-cap	0	0	0	0	0	0	Post-cap	0	0	0	0	0	0
ERCC-00123	0.228882	Pre-cap	0	0	0	0	0	0	Post-cap	0	0	0	0	0	0
ERCC-00156	0.457764	Pre-cap	0	0	0	0	0	0	Post-cap	0	0	0	0	0	0
ERCC-00158	0.457764	Pre-cap	0	0	0	0	0	0	Post-cap	0	0	0	0	0	0
ERCC-00164	0.457764	Pre-cap	0	0	0	0	0	0	Post-cap	0	0	0	0	0	0
ERCC-00168	0.457764	Pre-cap	0	0	0	0	0	0	Post-cap	0	0	0	0	0	0
ERCC-00109	0.915527	Pre-cap	0	0	0	0	0	0	Post-cap	0	0	0	0	0	0
ERCC-00120	0.915527	Pre-cap	0	0	0	0	0	0	Post-cap	0	0	0	0	0	0
ERCC-00137	0.915527	Pre-cap	0	0	0	0	0	0	Post-cap	0	0	0	0	0	0
ERCC-00147	0.915527	Pre-cap	0	0	0	0	0	0	Post-cap	0	0	0	0	0	0
ERCC-00031	1.831055	Pre-cap	0	0	0	0	0	0	Post-cap	0	0	0	0	0	0
ERCC-00058	1.831055	Pre-cap	0	0	0	0	0	0	Post-cap	0	0	0	0	0	0
ERCC-00069	1.831055	Pre-cap	0	0	0	0	0	0	Post-cap	0	0	0	0	0	0
ERCC-00134	1.831055	Pre-cap	0	0	0	0	0	0	Post-cap	0	0	0	0	0	0
ERCC-00014	3.662109	Pre-cap	0	0	0	0	0	0	Post-cap	0	0	0	0	0	0
ERCC-00028	3.662109	Pre-cap	0	0	0	0	0	0	Post-cap	0	0	0	0	0	0
ERCC-00143	3.662109	Pre-cap	0	0	0	0	0	0	Post-cap	0	0	0	0	0	0
ERCC-00150	3.662109	Pre-cap	3.24391	0	0	0	0	0	Post-cap	0	8.12052	0	0	0	0
ERCC-00034	7.324219	Pre-cap	0	0	0	0	0	0	Post-cap	0	4.44078	0	12.3503	22.6712	3.8063
ERCC-00085	7.324219	Pre-cap	0	0	0	0	0	0	Post-cap	0	5.36156	0	2.13015	2.48836	2.2978
ERCC-00157	7.324219	Pre-cap	0	0	0	0	0	0	Post-cap	0	0	0	0	0	1.9032
ERCC-00160	7.324219	Pre-cap	0	0	0	0	0	0	Post-cap	5.38507	8.12052	9.3529	19.3577	0	5.2202
ERCC-00059	14.64844	Pre-cap	0	0	0	0	0	0	Post-cap	0	2.87312	0	13.6979	16.0014	7.3879

ERCC-00099	14.64844	Pre-cap	0	0	0	0	0	0	Post-cap	0	0	0	0	0	0
ERCC-00148	14.64844	Pre-cap	0	0	0	0	0	0	Post-cap	0	0	2.8134	7.27874	8.50275	0
ERCC-00170	14.64844	Pre-cap	0	0	0	0	0	0	Post-cap	0	0	0	1.75743	8.21184	1.8957
ERCC-00019	29.29688	Pre-cap	0	0	0	0	0	0	Post-cap	0	4.68443	0	0	9.78344	18.068
ERCC-00078	29.29688	Pre-cap	0	0	2.2545	0	0	0	Post-cap	2.68621	3.03804	305.12	10.8631	23.2648	15.624
ERCC-00084	29.29688	Pre-cap	0	0	0	0	0	0	Post-cap	8.05052	10.6224	4.1947	3.6174	6.33857	5.8531
ERCC-00144	29.29688	Pre-cap	0	0	0	0	0	0	Post-cap	12.395	14.0185	5.1667	230.579	19.5184	18.023
ERCC-00051	58.59375	Pre-cap	0	0	0	0	0	0	Post-cap	0	11.0101	0	0	22.9947	21.233
ERCC-00071	58.59375	Pre-cap	0	0	0	0	0	0	Post-cap	6.23226	30.5437	10.824	39.2054	42.527	39.27
ERCC-00165	58.59375	Pre-cap	16.5841	0	0	0	0	0	Post-cap	596.496	1.7298	11.157	4.12351	2.40846	8.896
ERCC-00035	117.1875	Pre-cap	0	0	0	12.7218	0	0	Post-cap	12.983	21.3577	0	547.31	20.4442	17.162
ERCC-00044	117.1875	Pre-cap	0	0	1.9366	0	0	0	Post-cap	9.22978	52.1933	54.103	46.657	59.9532	50.328
ERCC-00131	117.1875	Pre-cap	3.1261	0	0	0	0	0	Post-cap	44.9757	27.3896	28.842	30.3139	65.3752	50.307
ERCC-00022	234.375	Pre-cap	0	3.65393	0	0	0	0	Post-cap	17.759	315.335	27.76	47.8788	53.1337	105.88
ERCC-00092	234.375	Pre-cap	0	0	15.934	0	0	0	Post-cap	185.105	48.3113	269.56	46.3858	85.9504	110.42
ERCC-00042	468.75	Pre-cap	4.71207	0	0	0	0	0	Post-cap	195.558	56.03	203.79	286.461	131.389	181.99
ERCC-00043	468.75	Pre-cap	0	0	0	0	3.6749	0	Post-cap	65.1859	75.1982	58.42	54.4803	69.8007	104.26
ERCC-00111	468.75	Pre-cap	16.9734	0	4.5045	0	3.7821	3.03404	Post-cap	585.005	89.532	198.55	75.9655	245.091	134.62
ERCC-00108	937.5	Pre-cap	18.8667	18.7952	32.858	25.3192	3.6785	11.8037	Post-cap	266.219	212.532	511.33	489.043	314.41	185.96
ERCC-00145	937.5	Pre-cap	2.31308	0	6.4455	0	0	0	Post-cap	33.2786	49.218	177.4	112.15	96.7453	55.835
ERCC-00046	3750	Pre-cap	27.7037	5.2569	4.2888	5.50792	21.606	23.1099	Post-cap	613.197	355.424	375.42	268.644	462.683	416.1
ERCC-00171	3750	Pre-cap	42.9545	21.7355	48.765	39.8533	29.778	0	Post-cap	604.788	507.774	943.99	423.652	503.211	376.34
ERCC-00004	7500	Pre-cap	55.3015	15.7405	25.684	32.9843	28.753	0	Post-cap	691.078	415.311	526.17	426.259	441.72	493.17
